# Iridium/Silver-Catalyzed H/D Exchange for Perdeuteration of Indoles and Site-Selective Deuteration of Carbazoles: Application in Late-Stage Functionalization

**DOI:** 10.1021/acs.joc.5c00702

**Published:** 2025-07-10

**Authors:** Prakriti Dhillon, Subban Kathiravan, Jesper G. Wiklander, Ian A. Nicholls

**Affiliations:** Bioorganic & Biophysical Chemistry Laboratory, Linnaeus University Centre for Biomaterials Chemistry, Department of Chemistry & Biomedical Sciences, 4180 Linnaeus University, Kalmar SE-391 82, Sweden

## Abstract

A novel iridium/silver-based method for catalyzing C–H deuterium labeling of indoles and carbazoles using D_2_O is presented. The method leverages a carbonyl-based directing group to achieve isotopic incorporation. This method demonstrates broad substrate scope and excellent functional group tolerance, enabling diverse and precise labeling of biologically important heterocycles. Notably, the developed protocol is successfully applied to the late-stage functionalization of carvedilol, showcasing its potential for modifying complex molecules. The operational simplicity, mild conditions, commercially available [Cp*IrCl_2_]_2_ as catalyst, D_2_O as the easily available cheap deuterium source, and high isotopic enrichment make this approach a valuable tool for the synthesis of deuterium-labeled compounds in pharmaceutical and mechanistic studies.

## Graphical abstract

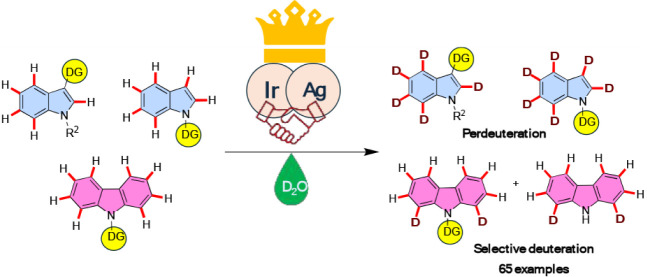

## Introduction

The incorporation of the hydrogen isotopes deuterium (^2^H) and tritium (^3^H) into organic molecules plays a crucial role in advancing our understanding of chemical processes at the molecular level and is a vital technique for probing reaction mechanisms, particularly through the analysis of kinetic isotope effects.^1^ Additionally, hydrogen isotope-labeled compounds are indispensable in mass spectrometry, where they serve as reference materials for accurate quantification and structural identification.^2^ In the pharmaceutical industry, isotope labeling, especially deuterium incorporation, has proven invaluable in the study of drug metabolism.^3^ By altering the hydrogen-to-deuterium ratio in drug molecules, researchers can gain deeper insights into the absorption, distribution, metabolism, and excretion properties of pharmaceutical compounds.^4^ The ability to track these compounds in vivo allows for the evaluation of metabolic pathways and the identification of potential toxicological risks. Such detailed metabolic profiling is critical for optimizing drug efficacy and safety, making it an essential aspect of drug discovery and medicinal chemistry.

The increasing importance of deuterium-labeled compounds is reflected in the first deuterated drug, Austedo, which was approved by the U.S. Food and Drug Administration in 2017, marking a milestone in the application of isotopic labeling in therapeutic agents.^5^ Since then, the development of deuterated drugs has gained significant momentum, with numerous candidates showing promising improvements in pharmacokinetic and pharmacodynamic properties.^1b^ These developments have captured the attention of synthetic organic chemists and medicinal chemists alike, who have directed attention to the methods for deuteration and the potential of deuterated compounds for improving drug performance.

The selective functionalization of complex organic molecules remains a central challenge in synthetic chemistry, particularly when it comes to controlling regioselectivity and maintaining a high functional group compatibility. Over the past decade, C–H activation has evolved as a versatile method to introduce various functional groups into organic molecules under mild reaction conditions with high efficiency, which increases the overall atom and step economy.^6^ C–H activation has proven especially powerful for the functionalization of aromatic compounds, offering an efficient and direct method for forming new bonds without the need for prefunctionalized starting materials.^7^ However, the selective incorporation of isotopes, such as deuterium, into specific positions of a molecule continues to be a highly sought-after transformation, providing valuable insights into reaction mechanisms and enabling the development of isotopically labeled compounds for various applications.^8^

Traditionally, deuterium labeling of aromatic compounds often involves H-D exchange facilitated by acids or bases.^9^ Both homogeneous and heterogeneous transition metal catalysts, such as platinum, rhodium, iridium, and palladium, among others, have also been used extensively.^10a,10b,4,8a^ Generally, due to their robust and harsh reaction conditions, heterogeneous transition metal catalysts can provide either selective or perdeuteration.^11^ Homogeneous catalysts have also been employed for deuteration, often offering greater selectivity and milder reaction conditions compared to their heterogeneous counterparts.^12^ Advances in ligand design and catalyst tuning have further enhanced their efficiency, making homogeneous catalysts valuable tools for both selective and nonselective isotopic labeling in complex molecular settings.^13^

Indoles and carbazoles, two important classes of heterocyclic compounds, are widely used in pharmaceutical and material sciences due to their bioactive properties and versatile chemistries.^14^ The ability to selectively incorporate deuterium into these heterocycles offers a tool for enabling understanding of biological pathways, enhancing the stability of drug candidates, and providing a means of probing reaction mechanisms.^15^

Significant studies have been reported on the deuteration of indole; for example, under acidic/basic conditions, C3 could be selectively deuterated due to its inherent reactivity.^16^ For the deuteration of C2 and C4–C7, various methods have been developed, including a directing group installed at the C3 carbon.^17^ When a directing group is introduced at C3 or at the indole nitrogen, the formation of five- or six-membered metallacycle intermediates becomes possible (Figure 1a,b).^18^ An analogous opportunity arises when introducing a directing group at the carbazole nitrogen, where a six-membered metallacycle intermediate may be formed (Figure 1c).^19^ These metallacycles play a crucial role in determining the selectivity of C–H activation reactions.^20^ Various transition metal-catalyzed methods have been developed for the selective deuteration of indoles, with or without a directing group (e.g., pyridine, pyrimidine, and acetyl derivatives) at nitrogen, to facilitate the deuteration via the formation of either five- or six-membered metallacycle intermediates.^21,25–27^

**Figure 1:**
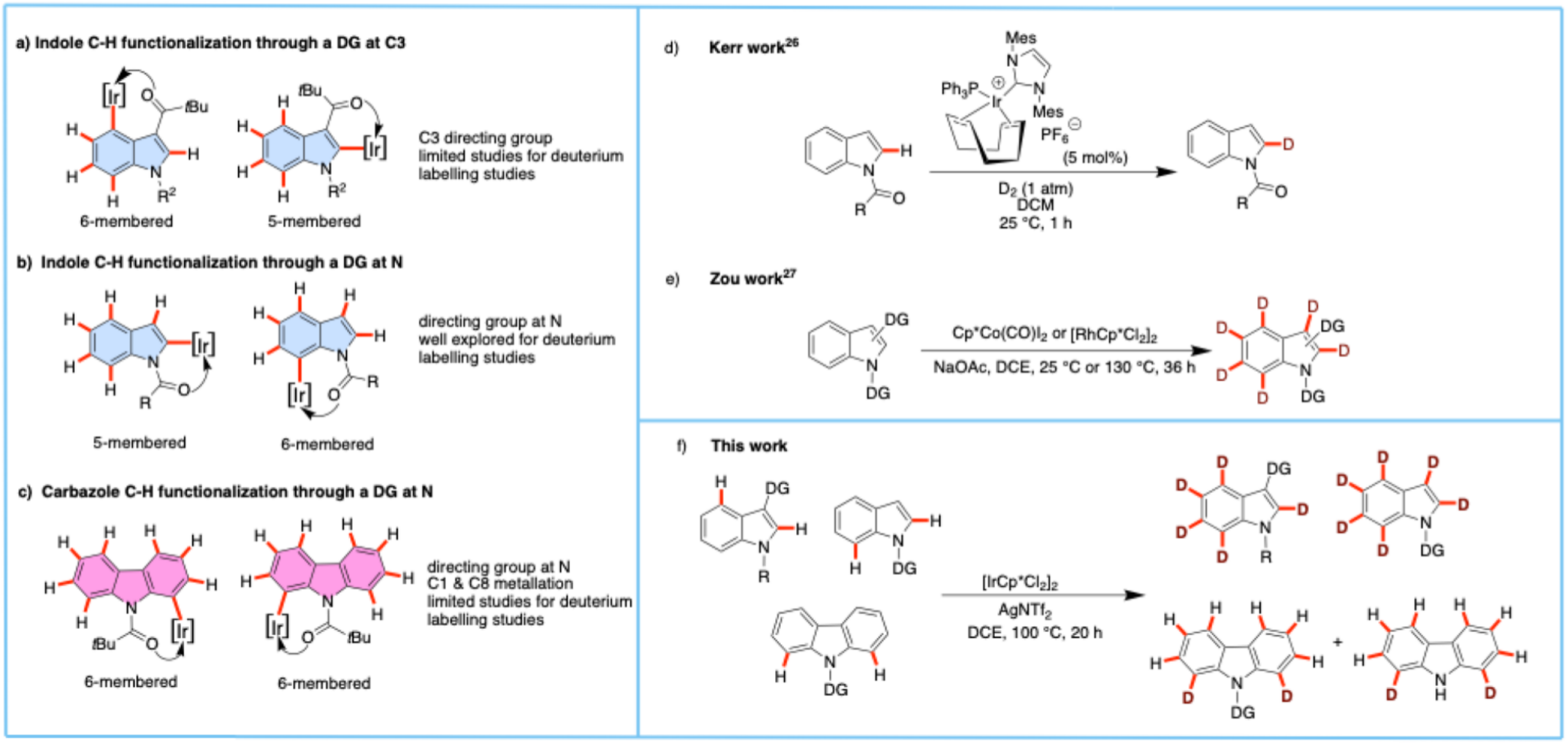
Overview of indole and carbazole C–H functionalization and deuterium labeling studies.

Iridium-catalyzed C–H deuteration was pioneered by Crabtree, who developed an efficient iridium­(I) catalyst, which has been a widely used method in the pharmaceutical industry to produce deuterated compounds.^22^ While this catalyst can achieve highly selective deuterium incorporation, its limitations, such as sensitivity to air and moisture, dependency on solvents like toluene or dichloromethane, functional group compatibility, and phosphine ligand instability, underscore the need for the development of new and improved catalytic systems.^22a^ Kerr later developed a more efficient catalyst, which could achieve deuteration under milder reaction conditions than the Crabtree catalyst.^23^ In both these catalytic reactions, the ligand plays a crucial role.^24^ Rhodium-catalyzed deuteration was also achieved on C2 by having a directing group at nitrogen; however, it required an additional step for removal after the deuteration, thus limiting the overall efficiency.^25^ Later, Kerr et al. reported a site-selective deuteration of *N*-heterocycles, indoles, and pyrroles via a Crabtree-type iridium-catalyzed hydrogen isotope exchange reaction (Figure 1d).^26^ In this work, the authors used common *N*-protecting groups to facilitate the selective deuteration of C2. Recently, Zou and coworkers reported a versatile regioselective deuteration of indoles using cobalt or rhodium, or both catalysts in combination (Figure 1e).^27^ In this work, the authors showed highly effective deuteration of indole in D_2_O with C2, C2/C7, C2/C3/C7, and C4 selectivity. The choice of directing group facilitated the success of deuterium exchange at the various sites of indoles. Although methoxy amide is a versatile removable directing group for this reaction, the authors did not observe deuteration when using commercially available [Cp*IrCl_2_]_2_ as catalyst.

We have previously worked with iridium catalysts for C–H halogenation,^28^ regioselective C2/C4 arylsulfenylation^29^ and C2-methylation^30^ of indoles through C–H activation reactions. During these studies, we found that a selective C–H deuteration (up to 95%) of indoles could be achieved under oxidative C–H activation reaction conditions.^30^ Based on these observations, we envisioned that the simple, commercially available iridium catalyst [Cp*IrCl_2_]_2_ could be a complement to existing methods for deuterium labeling studies, thus expanding its synthetic applicability beyond C–H activation reactions for C–C, C–N, and C–X bond formation. Moreover, the deuteration of carbazole, a structural analogue of indole, has not previously been studied using [Cp*IrCl_2_]_2_ as a catalyst. Herein, we present an iridium/silver-catalyzed ligand-free method for C–H deuteration of indoles and carbazoles, utilizing a directing group strategy (Figure 1e). By employing a carbonyl-based directing group, we demonstrate the ability to achieve both perdeuteration of indoles and selective deuteration of carbazoles under mild catalytic conditions. The utility of this strategy is further highlighted through its application in late-stage functionalization, expanding the scope of synthetic methodologies available for the modification of bioactive molecules and complex heterocycles.^31^

## Results and Discussion

We began our investigation into the targeted deuteration of 3-pivaloyl-*N*-methylindole (**1a**), employing [IrCp*Cl_2_]_2_ as the catalyst and D_2_O as the deuterium source, with AgNTf_2_ as an additive in 1,2-DCE at 100 °C, following established literature protocols (Table 1).^29^ Under the standard reaction conditions, we observed perdeuteration of 3-pivaloyl-*N*-methylindole (**1a**) in 86% isolated yield with nearly complete perdeuteration across the arene positions (C2–C7), most notably at C4 (88%), C5 (86%), and moderate levels at C2 (60%), C6 (59%), and C7 (57%) (Table 1, entry 1). Next, various solvents were screened to evaluate their impact on the reaction efficiency. The yield increased slightly in THF and decreased in 1,4-dioxane, but only minimal labeling was retained at C4 in both solvents, with 17% and 14% labeling, while the other positions remained virtually undeuterated (Table 1, entries 2 and 3). In contrast, toluene afforded a significantly reduced yield (64%) but showed an unusual deuteration pattern, particularly enhanced incorporation at C5 (74%) and moderate levels at C4 (45%), C6 (39%), and C7 (48%) (Table 1, entry 4). The pronounced selectivity for C5 in toluene may indicate a solvent-dependent influence on the catalyst-substrate interaction. 2-methyltetrahydrofuran (2-MeTHF) offered a favorable compromise, affording a relatively high yield of 79% with deuteration levels at C4 (61%) and C5 (56%), albeit with diminished labeling at C2 (10%), C6 (20%), and C7 (17%) (Table 1, entry 5). Acetonitrile completely suppressed the reaction, likely due to coordination with the metal catalyst, resulting in no detectable deuterium incorporation (Table 1, entry 6). We then tested the catalyst dependence by excluding [IrCp*Cl_2_]_2_. No deuteration of indole formation was observed, confirming the necessity of this catalyst (Table 1, entry 7). Substitution of [IrCp*Cl_2_]_2_ with other catalysts, such as [RhCp*Cl_2_]_2_ or Pd­(OAc)_2_ failed to initiate the reaction, highlighting the unique efficacy of iridium for this transformation (Table 1, entries 8 and 9). Finally, the role of AgNTf_2_ as an additive was shown to be essential for the reaction, as in its absence no product formation was detected (Table 1, entry 10). This silver additive likely facilitates the generation of an active catalytic species, enabling efficient H/D exchange.

**Table 1:**
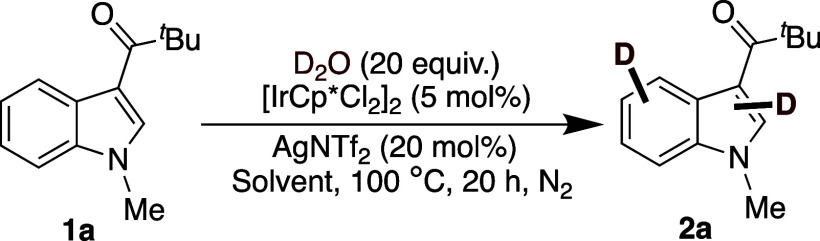
Optimization of Reaction Conditions^a^

			D contents (%)^b^				
No.	Deviation from reaction conditions	Yield (%)^c^	C2	C4	C5	C6	C7
1.	None	86	60	88	86	59	57
2.	D_2_O (20 equiv) in THF	89	0	17	0	0	0
3.	D_2_O (20 equiv) in 1,4-dioxane	74	0	14	0	0	0
4.	D_2_O (20 equiv) in toluene	64	11	45	74	39	48
5.	D_2_O (20 equiv) in 2-MeTHF	79	10	61	56	20	17
6.	D_2_O (20 equiv) in CH_3_ CN	NR	0	0	0	0	0
7.	No [IrCp*Cl_2_]_2_	NR	0	0	0	0	0
8.	[RhCp*Cl_2_]_2_ as catalyst	NR	0	0	0	0	0
9.	Pd(OAc)_2_ as catalyst	NR	0	0	0	0	0
10.	No AgNTf_2_	NR	0	0	0	0	0

a (0.23 mmol), D_2_O (20 equiv), [IrCp*Cl_2_]_2_ (5 mol %), AgNTf_2_ (20 mol %), 1,2-DCE (0.917 mL), 100 °C, under N_2_.

b Deuteration % determined by ^1^H NMR.

c Isolated yield.

With the optimized reaction conditions established, we sought to investigate the factors influencing the deuteration outcomes on various substituted indoles (Scheme 1). Using **1a** with a pivaloyl-directing group at C3 as the reference substrate, we observed that the nature and position of substituents significantly affected the H/D exchange pattern, as determined by NMR. The C–H bond at C4 consistently exhibited the highest reactivity, undergoing preferential deuterium incorporation when the pivaloyl directing group was positioned at C3 (**2a–2bo**). This preference suggests that the deuteration process is facilitated by the formation of a more favorable six-membered iridacycle intermediate, compared to the five-membered iridacycle intermediate. Consequently, C2 functionalization emerged as the second most favored site for deuteration, although its extent was notably influenced by the electronic and steric effects of the substituents. In the case of **2a**, higher deuterium incorporation at C5 compared to C2 was observed, which can be explained by the coordination and activation effects of the directing group at the C3 position. Specifically, the pivaloyl group at the C3 position plays a crucial role in directing C–H activation at C4 forming a six membered metallacycle that may facilitate activation at the C5 position. The C2 position, on the other hand, is less activated for C–H activation, presumably due to the formation of a less stable 5 membered metallacycle. For the electron rich indoles (entries **2b**–**2d**), in addition to high levels of deuteration on C4/C2, a significant H/D exchange was achieved for sites ortho to the electron-donating ester and methoxy substituents. However, negligible isotope exchange was seen for compound **2c** at C7 (5%), possibly due to steric hindrance arising from the proximity of the ester substituent. In some cases, such as compound **2b** and **2c**, the isolated yield was relatively low (14% and 13%) despite exhibiting high levels of deuterium incorporation as confirmed by NMR spectroscopy. This discrepancy is primarily attributed to challenges associated with product isolation and purification, rather than low conversion or catalytic inefficiency. Preparative TLC analysis of the crude reaction mixture for **2b** & **2c** revealed multiple spots, suggesting the formation of minor side-products or partial decomposition products under the reaction conditions. Although these additional components were present in small amounts and were not fully characterized, they likely complicated purification and contributed to product loss. Furthermore, control experiments conducted in the absence of the directing group also showed signs of substrate decomposition, indicating that the reaction conditions may promote background degradation to a minor extent. Collectively, these factors, decomposition, side-product formation, and purification challenges, account for the reduced isolated mass despite the successful isotopic incorporation. For the halogen-substituted indoles (**2e**–**2h**), a clear trend in deuterium incorporation at C6 was observed with a gradual decline in the reactivity of the C–H bond, attributable to a combination of steric-electronic factors from the halogen atom, especially noticeable for **2g**, wherein a corresponding decrease in H/D exchange was observed at C2. Additionally, for compound **2h** with chloro-substitution on C6, the reactivity of the C–H bond at C2 was drastically improved in comparison to C5 chloro-substituted indole, achieving a deuterium incorporation of 91%. Meanwhile, alkyl substituents were found to have a limited influence on the reactivity of the C2 C–H bond (**2i, 2j, 2l**) which could be improved upon by protecting the nitrogen with either ethyl or hexyl groups (**2k, 2m**). Interestingly, substituting the indole with a bulky benzyl group (**2n**) leads to site-selective deuteration at C2/C4. On the contrary, this site-specificity for deuteration was lost when the nitrogen atom was substituted with a phenyl ring (**2ao–2bo**). Furthermore, the depivaloylated side product (**2bo**) displayed improved deuteration on all sites, which could be a result of changed electronic properties on the indole with the cleavage of the pivaloyl group.

**Scheme 1:**
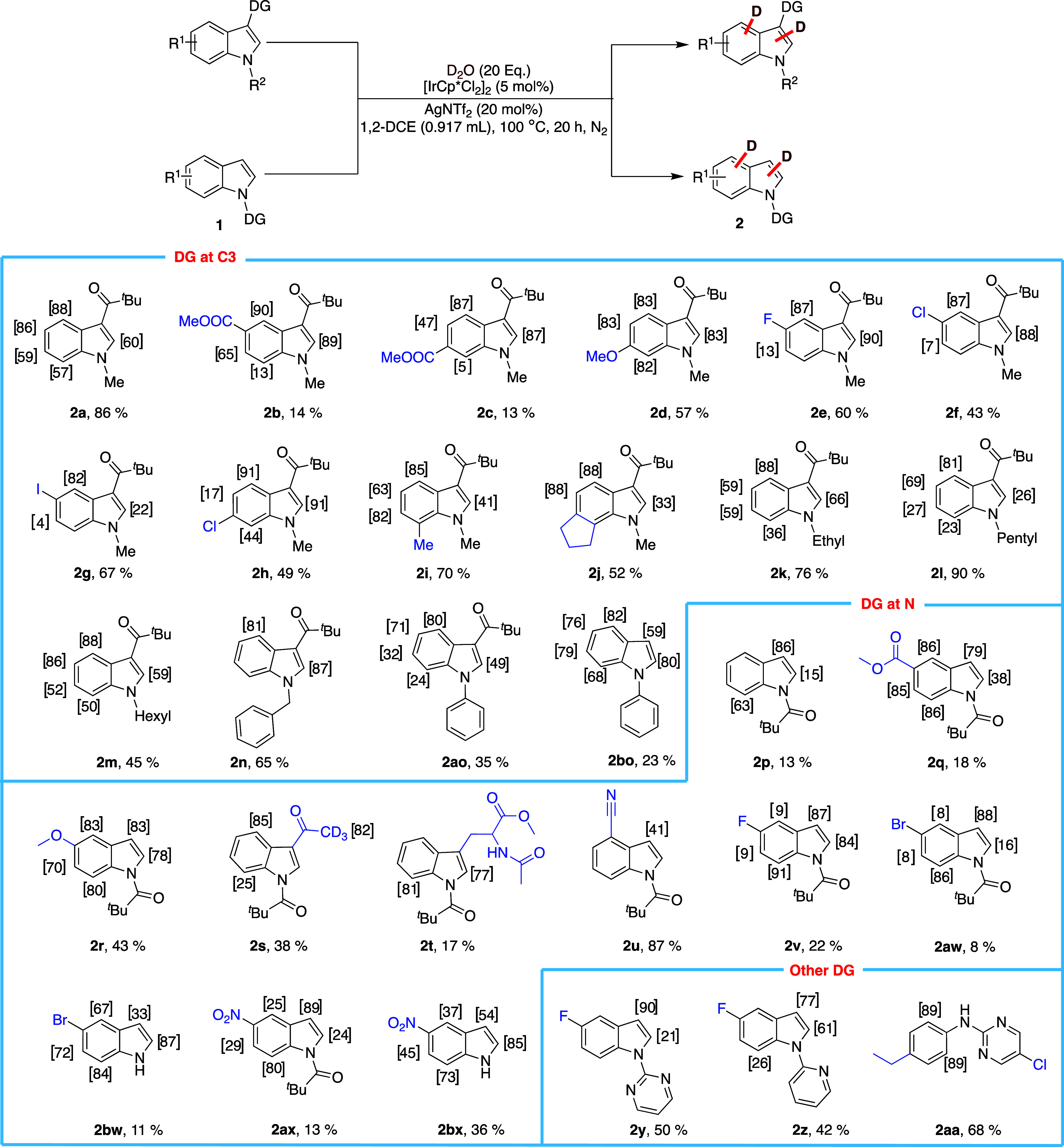
Scope of Indoles

With the pivaloyl group on the indole nitrogen (**1p**), more site-selective deuteration was achieved (**2p**). Moreover, we observed that when the directing group is at the C3 position, deuterium incorporation occurs at C5 and C6. In contrast, when the directing group is at the N1 position, as in substrates **2p**, **2s**, and **2t**, no deuterium incorporation is observed at these positions. This suggests that selective deuteration at C5 and C6 in indoles is strongly influenced by the position of the directing group and its coordination with the metal catalyst during the C–H activation process. When the pivaloyl group is located at C3, it facilitates C–H activation at C5 and C6 through strong coordination with the metal center, activating these adjacent positions for deuteration. However, when the pivaloyl group is positioned at N1, the resulting electronic and steric environment differs significantly. Although the pivaloyl group remains a directing group, its location at the nitrogen leads to less favorable coordination, particularly with the C7–H bond, and does not effectively promote activation at C5 or C6. Consequently, no deuterium incorporation is observed at these positions in such cases. Additionally, the C–H bond at C7, now accessible for directed activation through a 6-membered intermediate, appears to be a preferred site for H/D exchange as well. Electron-donating substituents (**2q**–**2r**) had a positive impact on the degree of deuteration on most sites. Regioselective deuteration was observed for **2s** and **2t** owing to a catalytically directed C–H activation at C4/C7 and C2/C7, respectively. Noteworthy was the regioselective H/D exchange (41%) found at the most reactive carbon for indole **2u**, substituted with a strongly electron-withdrawing cyano group. However, no deuterium incorporation was seen at the ortho positions of the directing group at C2 and C7. The iridium catalyst facilitates the activation of the C–H bond at C3, which is more reactive due to the electron density distribution and steric accessibility of the position in the indole structure or perhaps due to the coordination of the metal catalyst with the cyano group, which brings the metal catalyst closer to C3 rather than to the C2 and C7 positions. The 5-fluoroindole (**2v**) exhibited higher deuteration at the C7, C2, and C3 positions, with minor exchange also observed at the C4 and C6 positions. In contrast, the 5-bromo-substituted indole (**1w**) produced two distinct products (**2aw** and **2bw**). In the depivaloylated product (**2bw**), similar levels of deuterium exchange were observed at the C7 and C2 positions. For product **2aw**, the deuterium exchange was predominantly at the C7 and C2 positions. A similar pattern of isotope exchange is also noticed for indole **1x,** giving deuterated products **2ax** and **2bx**. For **2y**, instead of the pivaloyl-DG, pyrimidine-substituted indole (**1y**) resulted in selective H/D exchange at C3 (90%), while a moderate amount of deuteration was observed at C2 (21%), and surprisingly, no deuterium incorporation was found at C7. With pyridine-protected indole (**1z**), the deuteration reaction yielded **2z**, which showed a significant reduction in the deuteration level at C3 (77%) but drastically improved the reactivity of C2 (61%), along with a minimal amount of deuteration at C7 (26%) compared to **2y**. Although the nitrogen of such heterocycles can offer a strong coordination to the catalyst to bring about directed C–H activation,^32^ the isotope exchange on **2y** and **2z** suggests the pyrimidine and pyridine to be acting as substituents rather than DGs, exerting electronic effects on the indole substrate for the observed deuteration. Nonetheless, if these heterocycles were to direct the H/D exchange, the deuteration is indicative of a preference for a 5-membered intermediate over a 6-membered one. Lastly, in the case of **2aa**, excellent levels of *ortho*-directed deuteration were observed with 89% deuterium incorporation, consistent with the anticipated directing effects of its substituent.^33^

To further assess the influence of directing groups on the reaction outcome, we conducted a control experiment using an unprotected/directing-group-free indole (**1′**) under the standard conditions (Scheme 2). Interestingly, the reaction resulted exclusively in the formation of a 2,3-linked dimerized product (**1″**), and we found that deuterium incorporation occurred, albeit to a lesser extent, at the C2, C3, C5, and C6 positions, with the highest incorporation observed at C2 (71%). This unexpected outcome suggests a fundamentally different reactivity pathway when the indole lacks a directing group. The absence of a strong coordinating functionality likely shifts the reaction mechanism, promoting homo dimerization over site-selective deuteration. These findings highlight the significant role of directing groups in controlling the selectivity and suggest that further studies are necessary to expand our understanding of the mechanistic underpinnings governing this transformation.

**Scheme 2:**
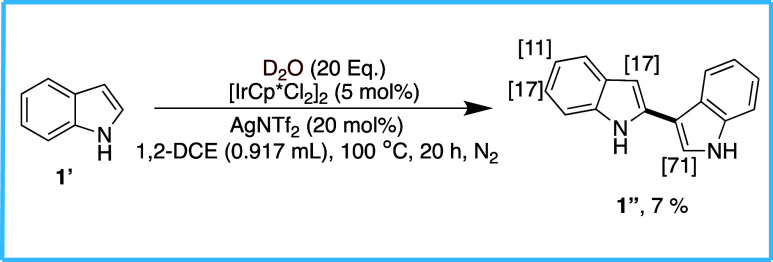
Control Study without Directing Group^a^

a TLC of the crude mixture showed few low-intensity and diffuse spots. These could not be isolated in sufficient quantities for characterization, and partial decomposition was also observed.

Next, we started with control experiments on carbazole using **3a** and **3e**, where they were subjected to the optimized reaction conditions (Scheme 3). The reaction of **3a** yielded the depivaloylated side product **4ba** in a 76% isolated yield, while delivering excellent site-selective deuteration with 89% incorporation exclusively on C1 and C8 (Scheme 3a). In contrast, the deuteration on **3e** gave two products, **4ae** and **4be**. For **4ae**, isotope exchange was concentrated on C1/C8, with no significant difference in the degree of deuteration between the two sites, despite the presence of the methoxy substituent at C2 (Scheme 3b). However, for **4be**, a significant difference in the deuteration levels was observed between C1 and C8, with a higher amount of deuterium incorporation at C1. This indicates that the loss of the pivaloyl directing group during the reaction leads to a differentiation of the reactivity of C1 and C8. Additionally, C3 was also found to be deuterated to a small degree, anticipated to arise from ortho-directed C–H activation of C3 by a possible weak coordination of the iridium catalyst with the methoxy functionality. Importantly, when **3a′** and **3e′** were treated under the same conditions, no deuteration was found to occur on **3a′**, while small amounts of deuterium incorporation were observed in **4be′** at C1 (20%) and C2 (21%). This result underscored the crucial role played by the pivaloyl group in efficiently directing the isotope exchange on carbazole to the C1/C8 positions (Scheme 3c,d).

**Scheme 3:**
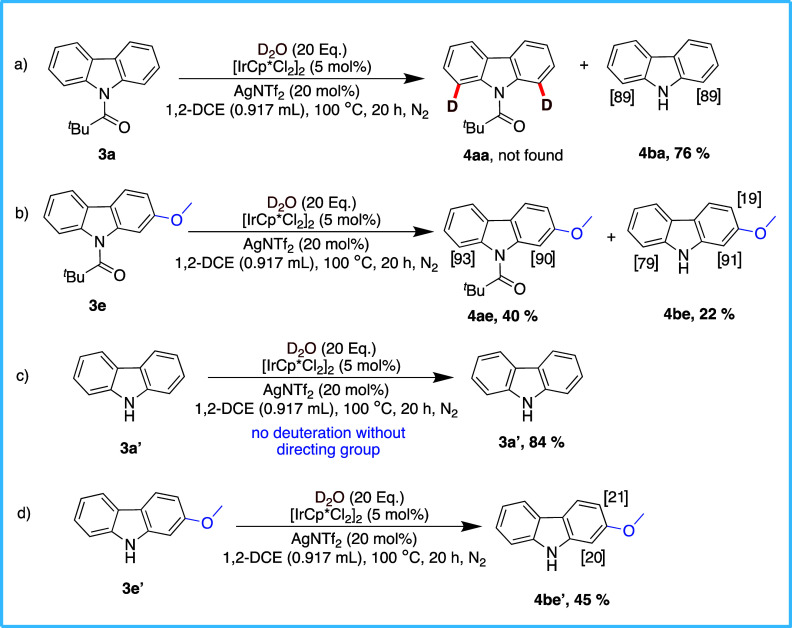
Control Studies on Carbazole

With the insights gained from the indole and control studies on carbazole, we proceeded to explore and investigate the scope of deuterium exchange on *N*-pivaloyl carbazoles (**3a**–**o**) (Scheme 4). The isotope exchange reaction on carbazoles was found to selectively activate the C–H bonds at C1/C8. The deuteration on carbazoles demonstrated good resilience to substituent effects, which depended solely on the directing ability of the pivaloyl group through a 6-membered metallacycle intermediate with excellent levels of deuterium incorporation. While the deuteration of several carbazoles resulted in two products, some gave only a single product, as shown in Scheme 3. For some substrates, the directing group was completely removed during the deuteration of carbazole, such as **4ab**, **4bi**, and **4bk**, while for others, it was only partially removed. The ability of the directing group to be completely removed during the deuteration of carbazole substrates (e.g., **4ab**, **4bi**, **4bk**) versus partial removal in other substrates [3,6-dimethyl (**4ab**), 4-tert-butyl (**4ac**), 4-phenyl (**4af**)] can be explained by electronic effects, which influence the coordination and reactivity of the directing group during the deuteration process.

**Scheme 4:**
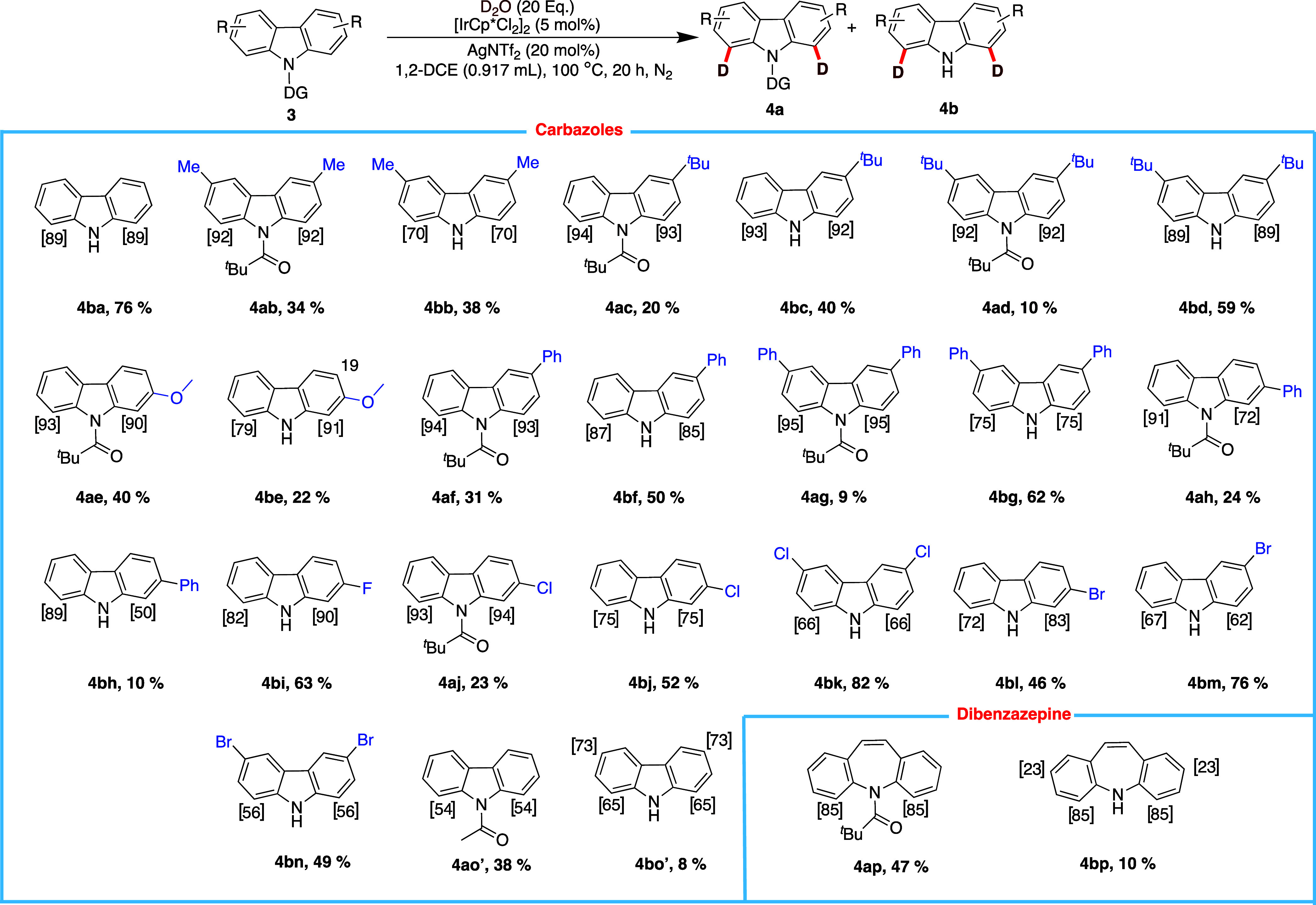
Scope of Carbazoles

For substrates such as **4ba** (unsubstituted carbazole), **4bi** (2-F carbazole), and **4bk** (3,6-dichlorocarbazole), the directing group at the N1 position can be more easily removed because these substrates have more favorable electronic properties that allow for more efficient removal of the pivaloyl group. For example, **4ba** is an unsubstituted carbazole, making it more flexible and allowing the directing group to be removed completely with minimal electronic interference. Similarly, **4bi** and **4bk** have electron-withdrawing substituents (fluorine and chlorine) that can also enhance the reactivity of the carbazole ring and facilitate the removal of the directing group. On the other hand, for substrates like 3,6-dimethyl (**4ab** & **4bb**), 4-tert-butyl (**4ac** & **4bc**), and 4-phenyl carbazole (**4af** &**4bf**), steric hindrance and electronic influence play a more significant role in preventing complete removal of the *N*-pivaloyl group. Substituents such as methyl, tert-butyl, and phenyl groups create additional bulk, making it harder for the directing group to be displaced. This steric bulk impedes the ability of the metal catalyst to fully engage with the directing group, resulting in only partial removal of the pivaloyl group. Additionally, the electron-donating effects of these substituents can further stabilize the directing group, thereby making it more resistant to complete removal. To our delight, the depivaloylated product in most cases was isolated as the major compound without compromising the regioselectivity of deuteration and is an important step toward a traceless C–H activation strategy. However, the depivaloylated products (**4bb**, **4be**, **4bg**, **4bh**, **4bj**, **4bk**, **4bl**, **4bm**, and **4bn**) displayed a lower degree of incorporation of deuterium, indicating the importance of the directing group for facilitating the reaction. When the directing group was changed to acetyl (**4ao′**), a considerable decrease in the deuteration levels was observed (54%), although the site for isotope exchange remained unchanged (C1/C8). Interestingly, the depivaloylated product **4bo′** showed not only a slightly higher degree of H/D exchange at C1/C8 but also a good amount of distal deuterium incorporation on the electron-rich sites C3/C6. Furthermore, the deuteration reaction on *N*-acetyl carbazole (**3o′**) favored the formation of the *N*-protected product (**4ao′**, 38%) over the deacetylated product (**4bo′**, 8%). Interestingly, the H/D exchange on **3p** showed a similar pattern of deuteration and reactivity to **3o′**. The deuteration reaction predominantly led to the formation of **4ap** (47%) with 85% deuterium exchange at C4/C6, while the depivaloylated product **4bp** was obtained in 10% isolated yield with an H/D exchange of 85% at C4/C6 and a moderate amount of 23% at C2/C8.

Finally, to expand the applicability of our reaction system, we extended our study to the late-stage deuteration of a drug using the commercially available carvedilol (**3q′**), useful for the treatment of high blood pressure and chronic heart failure (Scheme 5).^34^ A unique result was obtained for the deuteration on **3q**′, where the reaction exclusively produced **4bq′** in a 19% isolated yield. This product featured a complete removal of the side chain at C4, leaving a trideuterated 4-hydroxycarbazole. C1 and C3 exhibited isotope exchanges of 64% and 48%, respectively, owing to the activating effect of the hydroxy group, while C2 showed the lowest incorporation of 41%. Next, we performed the deuteration on **3r**, which yielded the product **4br** in a 35% yield with negligible deuteration at C1, while a moderate (21%) H/D exchange at C3. This suggests a weak ortho-para-activating effect of the aliphatic side chain on the nucleophilicity of the C–H bonds at these positions. Lastly, the deuteration on **3s** yielded the two expected products, **4as** and **4bs**, with yields of 67% and 5%, respectively. Interestingly, the reaction favored the formation of **4as** over **4bs**, which contrasts with the reactivity trends observed for most other carbazoles presented in Scheme 4. Notably, in **4as**, the C–H bond at C1 demonstrated higher reactivity toward activation than that at C8, incorporating almost double the amount of deuterium (92% at C1 versus 50% at C8). For **4bs**, the deuteration pattern at C1 and C8 (74% and 19%, respectively) was similar to that of **4as** but with significantly lower degrees of deuteration. Additionally, the reactivity of the C–H bond at C3 showed a marked improvement, allowing a 64% deuterium incorporation.

**Scheme 5:**
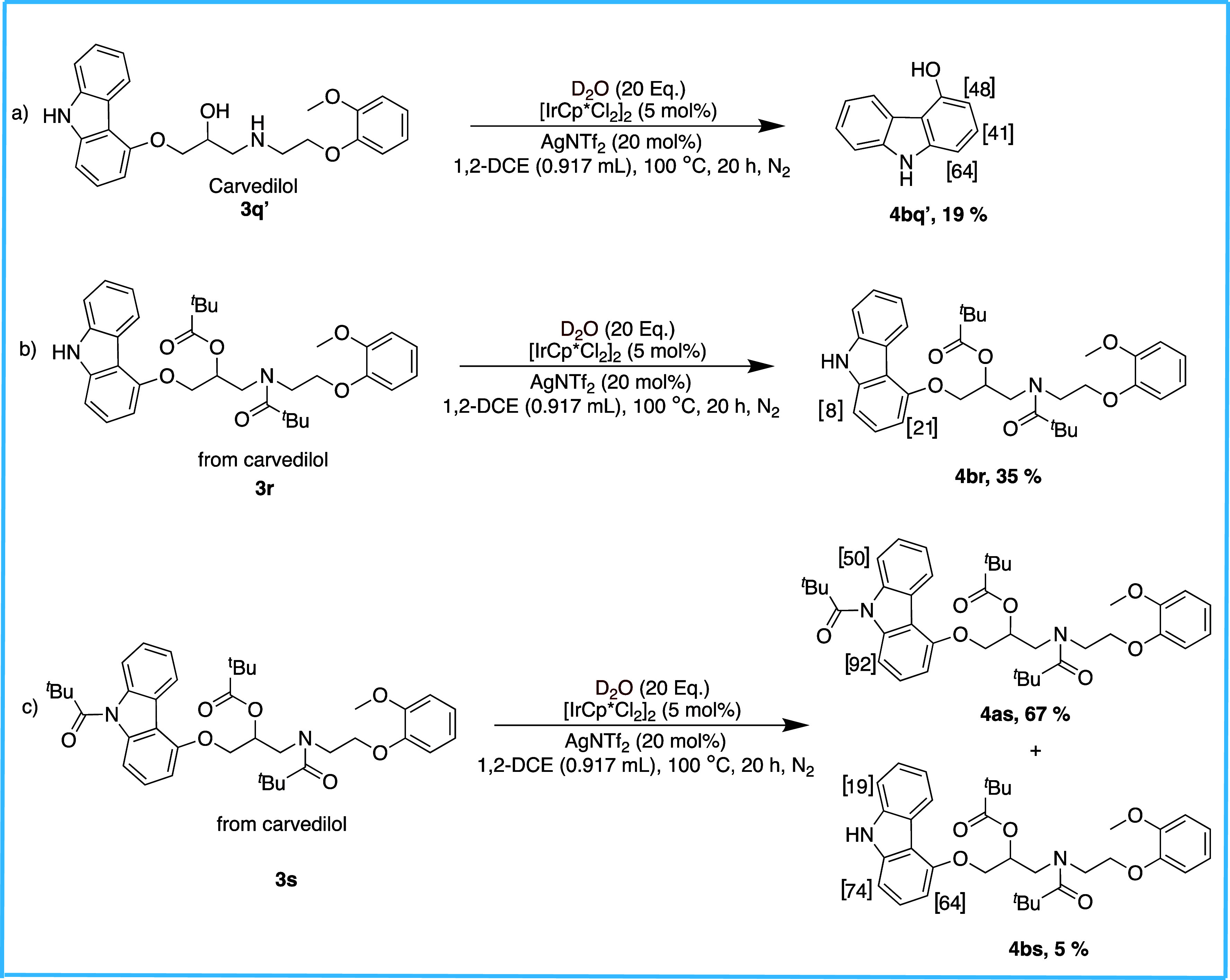
Late-Stage Functionalization of Carvedilol

Based on the observed reactivity and literature precedence,^23,26,27,29,30^ we propose that the deuteration of *N*-methyl-3-pivaloyl indole (**1**) and *N*-pivaloyl carbazole (**3**) using [Cp*IrCl_2_]_2_ as a catalyst, AgNTf_2_ as an additive, and D_2_O as the deuterium source in 1,2-DCE at 100 °C under N_2_, follows an Ir­(III)-catalyzed electrophilic metalation and H/D exchange pathway (Figure 2). The reaction is initiated by catalyst activation, where AgNTf_2_ abstracts chloride from [Cp*IrCl_2_]_2_, generating a cationic Ir­(III) species that enhances the electrophilicity of the activated iridium complex. There is also the possibility that the silver additive could act as a pH modulator. To investigate this, we attempted to estimate the acidity of the reaction mixture by directly applying pH indicator paper to the reaction mixture. Although the presence of organic components made the measurement somewhat challenging (as the mixture partially obscured the paper), we observed a color change corresponding to a pH of approximately 3. This suggests the reaction environment is mildly acidic, which may be attributed to the presence of AgNTf_2_ or its interaction with other components. We acknowledge the limitations of this approach, but the result supports the possibility that the silver salt contributes to modulating the acidity of the reaction medium. Thus, the activated Ir­(III) complex then coordinates to the substrate, with the pivaloyl group directing the metal to a specific C–H bond via carbonyl oxygen coordination (**A** & **C**). The C–H activation occurs through an electrophilic metalation mechanism, where Ir­(III) directly interacts with the electron-rich arene, leading to metalation without requiring an external base (**B** & **D**). Subsequent H/D exchange with D_2_O allows the incorporation of deuterium, and multiple exchange cycles (for **1** & **3**) can occur before product release.

**Figure 2:**
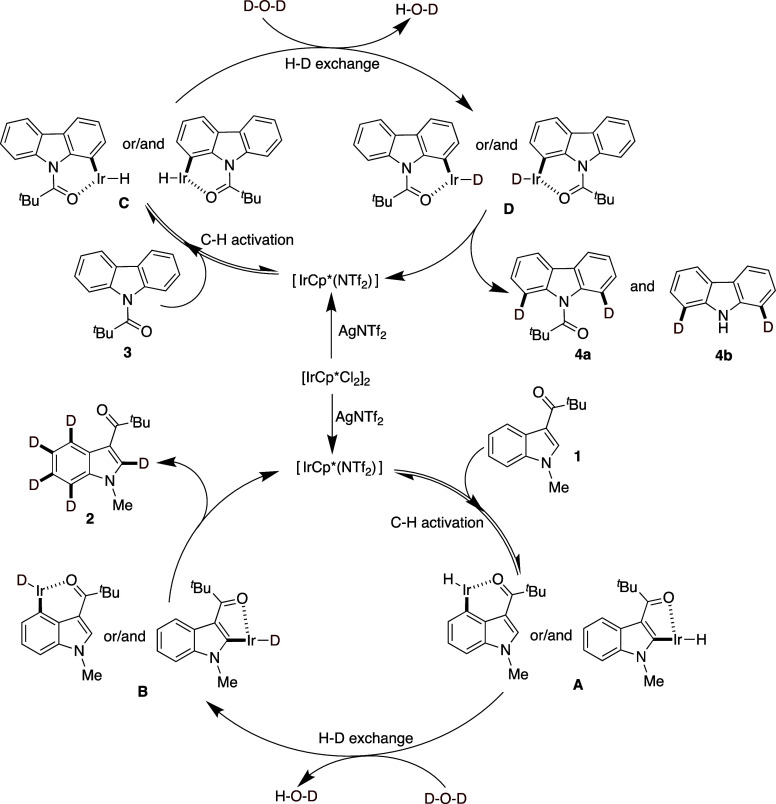
Proposed Catalytic Cycle.

A key observation in this study is the contrasting deuteration behavior between indole (**1**) and carbazole (**3**). In the case of indole, near-perdeuteration was observed across the C2–C7 positions. We attribute this extensive isotopic incorporation to strong π-interactions between the electron-rich indole ring and the Ir­(III) center, which may facilitate successive, nondirected C–H activation events beyond the initial directing group. While direct η^6^-arene coordination is well-established for certain group 8 and 9 metal complexes, such as [Ru­(*p*-cymene)_2_Cl_2_]_2_ systems,^35^ analogous transient π-complexation modes may also contribute to extended reactivity in iridium catalysis, particularly with electron-rich substrates like indoles.

In contrast, *N*-pivaloyl carbazole (**3**) exhibits more selective deuteration, often leading to a mixture of isolated products, including both the intact pivaloyl-substituted compound and a deprotected derivative in which the directing group has been cleaved. This reduced extent of deuteration likely stems from the more rigid, less electron-rich aromatic system of carbazole, which may be less effective in stabilizing extended π-interactions with the Ir­(III) center. Additionally, the fused benzene ring could impose steric constraints, limiting the metal’s access to certain C–H bonds and restricting activation to positions near the directing group.

These results suggest that arene electronics, coordination flexibility, and steric accessibility influence the extent of deuterium incorporation. Further mechanistic and computational studies are underway to validate this π-coordination-based hypothesis.

## Conclusions

In conclusion, we have developed an efficient iridium/silver-catalyzed methodology for C–H deuteration, which, in many cases, offers potential for the deuteration of indoles and carbazoles utilizing a directing group strategy. This approach demonstrates a broad substrate scope and, in the case of the carbazoles, excellent site selectivity. The reaction conditions were compatible with a range of functional groups, which allows for its use in the late-stage deuteration of complex molecules. The selective deuteration of carvedilol highlights the practical applicability of this method for the synthesis of isotopically labeled compounds. The results presented here represent a significant development in deuterium-labeling techniques for indoles and carbazoles and offer a valuable tool for application in drug development, mechanistic studies, and materials science.

## Methods

### General Method for the Deuteration of Indoles and Carbazoles

To a reaction tube was added the indole (**1**) or carbazole (**3**) substrate (0.23 mmol), [Cp*IrCl_2_]_2_ (5 mol %), and AgNTf_2_ (20 mol %). The reaction tube was then sealed with a Teflon-lined screw cap, evacuated, and purged with N_2_ (3 cycles). Then, under N_2_, 1,2-DCE (917 μL) and D_2_O (83 μL, 20 equiv) were added using a syringe. The reaction tube was sealed with parafilm and allowed to stir at 100 °C for 20 h. The TLC for the reaction mixture was checked in petroleum ether/acetone (9:1 or 4:1) mostly, and for certain cases, in petroleum ether/ethyl acetate (9:1 or 4:1). The reaction mixture was then filtered through a pad of Celite in DCM (5 mL), methanol (5 mL), and acetone (5 mL). The crude material was concentrated under reduced pressure and was purified by preparative TLC. The preparative TLC was run in petroleum ether/acetone or petroleum ether/ethyl acetate solvent combinations (9:1 or 4:1). The collected material was then sonicated in 5 mL each of DCM and methanol, followed by filtration through a sintered Buchner glass funnel. Finally, the residue was concentrated under reduced pressure and dried under high vacuum. The site and degree of deuterium incorporation were determined by comparing the integrals of the characteristic NMR peaks with those of the starting substrates. Structural assignments were made with additional information from gCOSY, gHSQC, and gHMBC experiments.

### Safety Statement

**Caution!** 1,2-dichloroethane (1,2-DCE) is a volatile, toxic, and suspected carcinogen. It is harmful if inhaled, ingested, or absorbed into the skin. Vapors may form explosive mixtures with air. Use only in a well-ventilated fume hood, and avoid contact with heat, open flames, and strong bases.

**Caution!** Silver bis­(trifluoromethanesulfonyl)­imide (AgNTf_2_) is moisture-sensitive and highly reactive, especially in the presence of light or reducing agents. It should be handled under an inert atmosphere, and contact with water, amines, and strong nucleophiles should be avoided. It can cause eye and skin irritation.

## Supplementary Material





## Data Availability

The data underlying this study are available in the published article and its Supporting Information.

## Notes

General information, detailed experimental details, characterization data for all compounds (PDF)
All NMR (^1^H, ^13^C, COSY, NOESY, HSQC) spectra (PDF)
