# Supplementary material for: Iridium/Silver-Catalyzed H/D Exchange for Perdeuteration of Indoles and Site-Selective Deuteration of Carbazoles: Application in Late-Stage Functionalization
Source: J Org Chem. 2025 Jul 10;90(33):11791–801. doi: 10.1021/acs.joc.5c00702 (PMC12381928; doi:10.1021/acs.joc.5c00702)
Supplement: Supplementary file 1 [file jo5c00702_si_001.pdf]

Iridium/Silver-Catalyzed H/D Exchange for Perdeuteration of Indoles and Site-Selective Deuteration Carbazoles: Application in Late-Stage Functionalization

Prakriti Dhillon, Subban Kathiravan, Jesper G. Wiklander, and Ian A. Nicholls*

Bioorganic & Biophysical Chemistry Laboratory, Linnaeus University Centre for Biomaterials Chemistry, Department of Chemistry & Biomedical Sciences, Linnaeus University, Kalmar SE-39182, Sweden.

Email: suppan.kathiravan@lnu.se

Table of Contents

1. General Information: S3
2. Analytical information S3
3. Experimental section S4
   3.1. Synthesis of substrates S4
      3.1.1. Synthesis of *N*-alkyl-indoles S4
      3.1.2. Synthesis of *N*-benzyl-indoles S4
      3.1.3. Synthesis of 3-pivaloyl indoles[3] S4
      3.1.4. Synthesis of *N*-pivaloyl indoles S4
      3.1.5. Synthesis of *N*-pivaloyl carbazoles[1] S5
      3.1.6. 1-((9*H*-carbazol-4-yl)oxy)-3-(*N*-(2-(2-methoxyphenoxy)ethyl)pivalamido)-propan-2-yl pivalate (3r) S5
      3.1.7. Synthesis of 1-(*N*-(2-(2-methoxyphenoxy)ethyl)pivalamido)-3-((9-pivaloyl-9*H*-carbazol-4-yl)oxy)propan-2-yl pivalate (3s) S6
   3.2. General method for the deuteration of indoles and carbazoles S7
4. Characterization data S8
   4.1. NMR data of indole substrates S8
   4.2. NMR data of carbazole substrates S16
   4.3. Characterization of deuterated products S28
      4.3.1. Characterization of deuterated indoles S28
      4.3.2. Characterization of deuterated carbazole products and control compounds S43
5. Scaleup experiment (1 mmol) S57
6. Control experiment S58
7. References S59

1. General Information:

All the solvents, starting indoles and carbazoles were purchased commercially (Aldrich, Chemtronica, Angene) and used without further purification. 1,2-DCE was dried over molecular sieves before use. All the glassware was dried at 120°C before use. The compounds were purified through column chromatography using silica gel (Carlo Erba, 60 Å). Preparative thin layer chromatography was performed on glass plates precoated with silica gel (UV254, 20 x 20 cm, 500 µ) purchased from Aldrich. The compounds were visualized under UV using silica gel preloaded on an aluminum foil containing fluorescence indicator for 254 nm.

Safety Statement:

Caution! 1,2-Dichloroethane (1,2-DCE) is a volatile, toxic, and suspected carcinogen. It is harmful if inhaled, ingested, or absorbed through the skin. Vapors may form explosive mixtures with air. Use only in a well-ventilated fume hood and avoid contact with heat, open flames, and strong bases.

Caution! Silver bis(trifluoromethanesulfonyl)imide ($AgNTf_2$) is moisture sensitive and highly reactive, especially in the presence of light or reducing agents. It should be handled under an inert atmosphere and contact with water, amines, and strong nucleophiles should be avoided. Can cause eye and skin irritation.

Caution! Cp^*IrCl_2 dimer is a moisture- and air-sensitive organometallic compound. It may decompose upon prolonged exposure to air or light, releasing hazardous decomposition products. Avoid contact with skin, eyes, and clothing.

Caution! Many indole and carbazole derivatives are toxic and may cause skin, eye, or respiratory irritation. Proper risk assessment must be conducted before use. Handle all such compounds with appropriate personal protective equipment (PPE) and use only in a well-ventilated fume hood. Avoid skin contact and inhalation.

2. Analytical information

All NMR spectra were recorded on a Bruker 400'54 Ascend spectrometer. $CDCl_3$ and MeOD-d_4 were used as the NMR solvents. The chemical shifts (δ) are reported in ppm with the spectra referenced to non-deuterated solvent signals or TMS. 2D-NMR spectra are presented in the order COSY, NOESY, HSQC, HMBC. HRMS data was obtained from Stockholm University and recorded on a Waters I-Class UPLC and XEVO G2-XS QToF instrument using the MS^E, 50 – 1200 m/z, positive or negative ESI mode. IR spectra were recorded using an Agilent Cary 630 FTIR spectrometer equipped with an

ATR accessory. Melting points (uncorrected) were recorded using a Stuart Scientific Melting Point SMP1.

3. Experimental section

3.1. Synthesis of substrates

3.1.1. Synthesis of *N*-alkyl-indoles

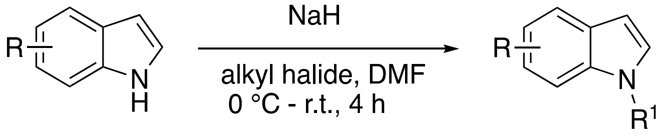

N-alkyl indoles were synthesized using a reported procedure.[1]

3.1.2. Synthesis of *N*-benzyl-indoles

N-benzyl-indoles were synthesized using a reported procedure.[2]

3.1.3. Synthesis of 3-pivaloyl indoles[3]

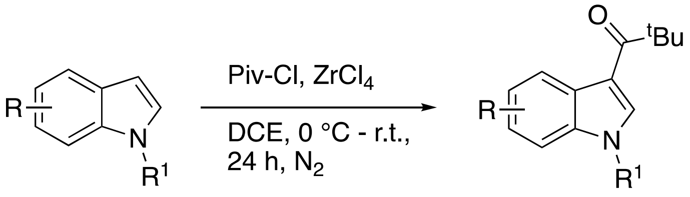

To a round bottom flask (RBF) was added the indole (1.1g) which was then dissolved in dry 1,2-DCE (25 mL). After cooling the solution to 0 °C, under N_2 was added $ZrCl_4$ (1.99 g, 8.53 mmol, 1.5 equiv.) was added followed by dropwise addition of pivaloyl chloride (1.1 mL, 8.53 mmol, 1.5 equiv.). The reaction mixture was allowed to stir at room temperature under inert conditions for 24 h. The reaction mixture was quenched with water (50 mL) and extracted with DCM. The product was purified by column chromatography at 1% acetone in petroleum ether.

3.1.4. Synthesis of *N*-pivaloyl indoles

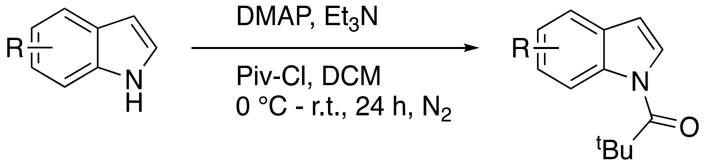

N-pivaloyl-indoles were synthesized using a reported procedure[4&5]

3.1.5. Synthesis of *N*-pivaloyl carbazoles[1]

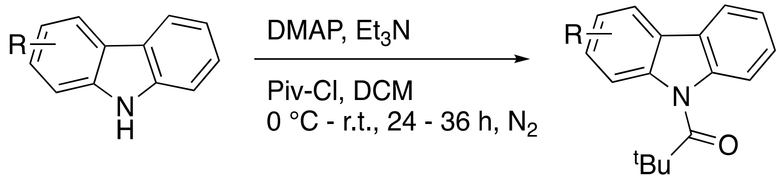

To an RBF was added the carbazole (3.0 g, 17.9 mmol) and DMAP (218 mg, 1.79 mmol, 10 mol%). Dry DCM (50 mL) was added under the flow of nitrogen, followed by triethylamine (5 mL, 35.83 mmol, 2 equiv.). The solution mixture was then cooled to 0 °C in an ice-bath. Pivaloyl chloride (3.31 mL, 26.87 mmol, 1.5 equiv.) was added dropwise to the solution using a syringe and the reaction mixture was left to stir at 0 °C for about 5-10 mins after which it was allowed to stir at room temperature under nitrogen for 24–36 h. Upon completion of the reaction monitored by TLC, work-up was carried out using brine and DCM. The organic layer was dried over $MgSO_4$ and the solvent removed under reduced pressure. TLC for the title compounds was checked in petroleum ether/acetone (9:1) or petroleum ether/ethyl acetate (4:1) solvent combination. Purification was performed by column chromatography using silica gel and the compounds were eluted at 5-10 % acetone or ethyl acetate in petroleum ether. The *N*-pivaloyl carbazoles were obtained in 85–90 % isolated yields.

3.1.6. 1-((9*H*-carbazol-4-yl)oxy)-3-(*N*-(2-(2-methoxyphenoxy)ethyl)pivalamido)-propan-2-yl pivalate (3r)

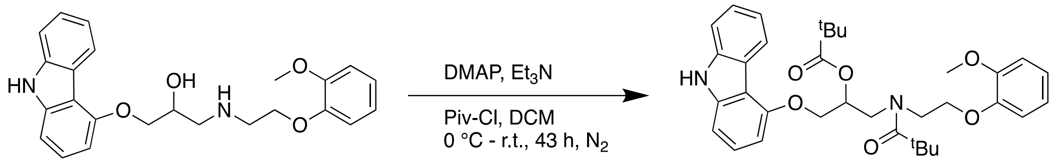

To an RBF was added Carvedilol (500 mg, 1.23 mmol) and DMAP (15.0 mg, 0.123 mmol, 10 mol%). The RBF was then purged with nitrogen gas and dry DCM (16 mL) was added followed by triethylamine (0.35 mL, 2.46 mmol, 2 equiv.). The solution mixture was then cooled to 0 °C in an ice-bath. Pivaloyl chloride (0.23 mL, 1.84 mmol, 1.5 equiv.) was added dropwise to the solution using a syringe stirred at 0 °C for about 5-10 mins after which it was allowed to stir at room temperature under inert conditions for 43 h. Upon completion of the reaction monitored by TLC, work-up was carried out using brine and DCM. The organic layer was dried over $MgSO_4$ and the solvent was

removed under reduced pressure. TLC for the title compounds was checked in petroleum ether/acetone (3:2). Purification of the title compound was performed by column chromatography using silica gel which was eluted at 25 % acetone in petroleum ether as an oily liquid in an 87 % isolated yield.

3.1.7. Synthesis of 1-(*N*-(2-(2-methoxyphenoxy)ethyl)pivalamido)-3-((9-pivaloyl-9*H*-carbazol-4-yl)oxy)propan-2-yl pivalate (3s)

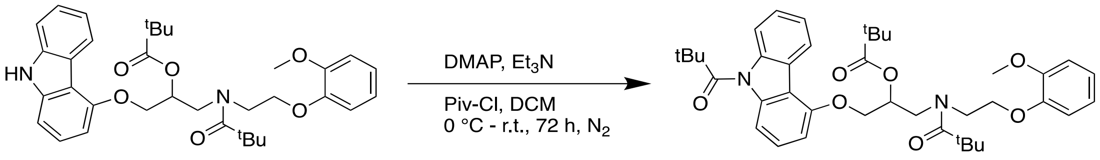

To an RBF was added *O*-protected Carvedilol (337 mg, 0.587 mmol.) and DMAP (7.2 mg, 0.0587 mmol, 10 mol%). The RBF was then purged with nitrogen gas and dry DCM (17 mL) was added followed by triethylamine (0.2 mL, 1.17 mmol, 2 equiv.). The solution mixture was then cooled to 0 °C in an ice-bath. Pivaloyl chloride (0.11 mL, 0.88 mmol, 0.11mL, 1.5 equiv.) was added dropwise to the solution using a syringe and stirred at 0 °C for about 5-10 mins after which it was allowed to stir at room temperature under inert conditions for 72 h. Upon completion of the reaction monitored by TLC, work-up was carried out using brine and DCM. The organic layer was dried over $MgSO_4$ and the solvent was removed under reduced pressure. Petroleum ether/acetone (4:1) solvent combination was used to check the TLC. The title compound was purified by silica gel chromatography which was eluted at 15 % acetone in petroleum ether as a semi-solid in a 57 % isolated yield.

3.2. General method for the deuteration of indoles and carbazoles

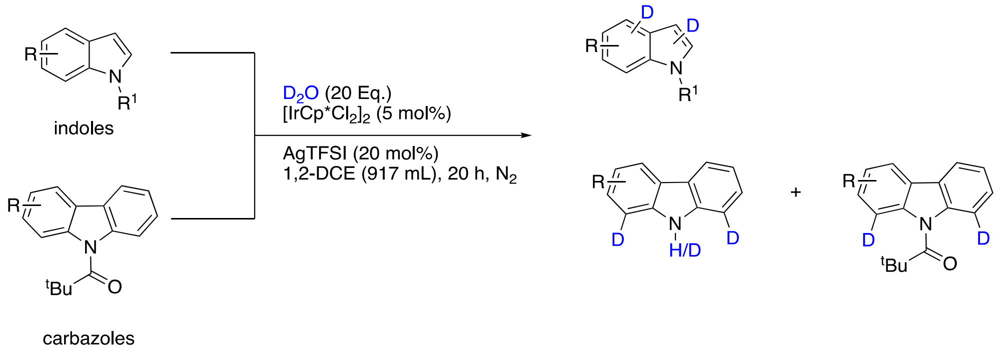

To a reaction tube was added the indole or carbazole substrate (0.23 mmol), $[Cp^*IrCl_2]_2$ (5 mol%), and $AgNTf_2$ (20 mol%). The reaction tube was then sealed with a Teflon lined screw cap and evacuated and purged with N_2 (3 cycles). Then under N_2 was added 1,2-DCE (917 µL) and D_2O (83 µL, 20 equiv.) using a syringe. The reaction tube was sealed with parafilm and allowed to stir at 100 °C for 20 h. The TLC for the reaction mixture was checked in petroleum ether/acetone (9:1 or 4:1) mostly and for certain cases in petroleum ether/ethyl acetate (9:1 or 4:1). The reaction mixture was then filtered through a pad of celite in DCM (5 mL), methanol (5 mL) and acetone (5 mL). The crude material was concentrated under reduced pressure and was purified by preparative TLC. The preparative TLC were run in petroleum ether/acetone or petroleum ether/ethyl acetate solvent combination (9:1 or 4:1). The collected material was then sonicated in 5mL each of DCM and methanol, followed by filtration through a sintered Buchner glass funnel. Finally, the residue was concentrated under reduced pressure and dried under high vacuum. The site and degree of deuterium incorporation was determined by comparing the integrals of the characteristic ^1H NMR peaks with those of the starting substrates.

4. Characterization data

4.1. NMR data of indole substrates

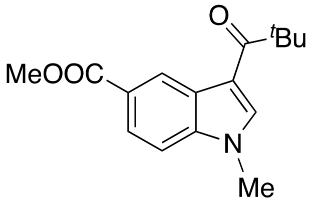

Methyl 1-methyl-3-pivaloyl-1*H*-indole-5-carboxylate (1b)[6]

Yellow solid

^{1}H NMR (400 MHz, $CDCl_3$) δ 9.23 (d, *J* = 1.6 Hz, 1H), 8.02 (dd, *J* = 8.6, 1.7 Hz, 1H), 7.83 (s, 1H), 7.34 (dd, *J* = 8.6, 0.7 Hz, 1H), 3.93 (s, 3H), 3.88 (s, 3H), 1.42 (s, 9H).

^{13}C{^{1}H} NMR (101 MHz, $CDCl_3$) δ 201.8, 168.0, 138.9, 135.3, 127.8, 126.1, 124.8, 124.5, 113.8, 109.1, 51.9, 44.2, 33.7, 28.8.

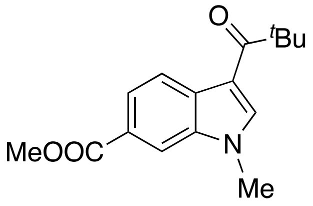

Methyl 1-methyl-3-pivaloyl-1*H*-indole-6-carboxylate (1c)[6]

Yellow solid

^{1}H NMR (400 MHz, $CDCl_3$) δ 8.54 (dd, *J* = 8.5, 0.6 Hz, 1H), 8.09 (d, *J* = 1.4 Hz, 1H), 7.97 (dd, *J* = 8.5, 1.5 Hz, 1H), 7.91 (s, 1H), 3.96 (s, 3H), 3.92 (s, 3H), 1.42 (s, 9H).

^{13}C{^{1}H} NMR (101 MHz, $CDCl_3$) δ 201.8, 167.8, 136.6, 136.0, 132.0, 125.0, 123.4, 123.1, 113.0, 111.6, 52.1, 44.1, 33.7, 28.8.

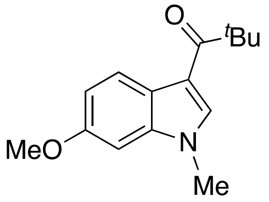

1-(6-methoxy-1-methyl-1*H*-indol-3-yl)-2,2-dimethylpropan-1-one (1d) [6]

Colorless solid

^{1}H NMR (400 MHz, $CDCl_3$) δ 8.38 (d, *J* = 8.8 Hz, 1H), 7.69 (s, 1H), 6.94 (dd, *J* = 8.8, 2.3 Hz, 1H), 6.76 (d, *J* = 2.3 Hz, 1H), 3.88 (s, 3H), 3.79 (s, 3H), 1.41 (s, 9H).

^{13}C{^{1}H} NMR (101 MHz, $CDCl_3$) δ 202.0, 157.2, 137.3, 133.5, 124.2, 122.3, 112.8, 111.6, 93.0, 55.7, 44.0, 33.4, 29.0.

1-(5-fluoro-1-methyl-1*H*-indol-3-yl)-2,2-dimethylpropan-1-one (1e) [6] (Yellow solid)

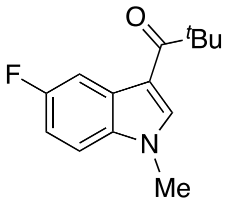

^{1}H NMR (400 MHz, $CDCl_3$) δ 8.20 (dd, *J* = 10.2, 2.6 Hz, 1H), 7.81 (s, 1H), 7.23 (dd, *J* = 8.9, 4.3 Hz, 1H), 7.03 (td, *J* = 8.9, 2.6 Hz, 1H), 3.85 (s, 3H), 1.41 (s, 9H).

^{13}C{^{1}H} NMR (101 MHz, $CDCl_3$) δ 201.7, 159.8 (d, *J* = 237.3 Hz), 135.3, 133.0, 129.0 (d, *J* = 11.5 Hz), 112.7 (d, *J* = 5.0 Hz), 111.6 (d, *J* = 26.8 Hz), 109.9 (d, *J* = 10.1 Hz), 108.7 (d, *J* = 25.0 Hz), 44.0, 33.7, 28.8.

1-(5-chloro-1-methyl-1*H*-indol-3-yl)-2,2-dimethylpropan-1-one (1f) [6] (Colorless solid)

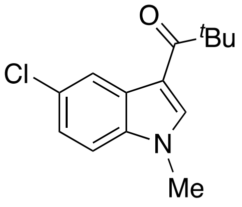

^{1}H NMR (400 MHz, MeOD) δ 8.33 (d, *J* = 2.1 Hz, 1H), 8.26 (s, 1H), 7.41 (d, *J* = 8.9 Hz, 1H), 7.23 (dd, *J* = 8.7, 2.1 Hz, 1H), 3.88 (s, 3H), 1.40 (s, 9H).

^{13}C{^{1}H} NMR (101 MHz, MeOD) δ 204.3, 138.5, 136.6, 130.6, 129.3, 124.3, 123.2, 113.0, 112.1, 45.0, 33.7, 29.2.

1-(5-iodo-1-methyl-1*H*-indol-3-yl)-2,2-dimethylpropan-1-one (1g) [6] (Yellow solid)

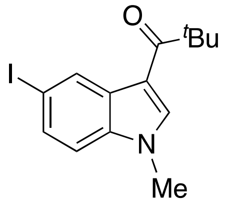

^{1}H NMR (400 MHz, $CDCl_3$) δ 8.91 (d, *J* = 1.7 Hz, 1H), 7.71 (s, 1H), 7.55 (dd, *J* = 8.6, 1.7 Hz, 1H), 7.07 (d, *J* = 8.6 Hz, 1H), 3.82 (s, 3H), 1.40 (s, 9H).

^{13}C{^{1}H} NMR (101 MHz, $CDCl_3$) δ 201.8, 135.6, 134.5, 132.2, 131.7, 130.4, 112.1, 111.1, 86.9, 44.1, 33.6, 28.8.

1-(6-chloro-1-methyl-1*H*-indol-3-yl)-2,2-dimethylpropan-1-one (1h) [6] (Yellow solid)

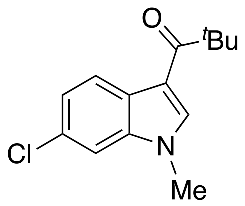

^{1}H NMR (400 MHz, $CDCl_3$) δ 8.42 (dd, *J* = 8.5, 0.5 Hz, 1H), 7.76 (s, 1H), 7.31 (d, *J* = 1.8 Hz, 1H), 7.24 (m, 1H), 3.82 (s, 3H), 1.41 (s, 9H).

^{13}C{^{1}H} NMR (101 MHz, $CDCl_3$) δ 201.9, 136.9, 134.6, 129.3, 126.8, 124.4, 123.0, 112.9, 109.3, 44.1, 33.6, 28.9.

1-(1,7-dimethyl-1*H*-indol-3-yl)-2,2-dimethylpropan-1-one (1i) [6]

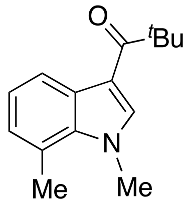

Yellow solid

^{1}H NMR (400 MHz, $CDCl_3$) δ 8.39 (d, *J* = 8.1 Hz, 1H), 7.68 (s, 1H), 7.13 (dd, *J* = 8.1, 7.2 Hz, 1H), 6.98 (dt, *J* = 7.2, 1.1 Hz, 1H), 4.10 (s, 3H), 2.75 (s, 3H), 1.40 (s, 9H).

^{13}C{^{1}H} NMR (101 MHz, $CDCl_3$) δ 201.9, 136.0, 135.2, 129.4, 126.0, 122.6, 121.4, 121.0, 112.2, 44.1, 37.7, 29.0, 19.7.

2,2-dimethyl-1-(1-methyl-1,6,7,8-tetrahydrocyclopenta[*g*]indol-3-yl)propan-1-one (1j) [6] (Colorless solid)

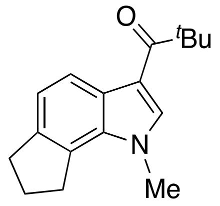

^{1}H NMR (400 MHz, $CDCl_3$) δ 8.32 (dd, *J* = 8.1, 1.0 Hz, 1H), 7.66 (s, 1H), 7.17 (d, *J* = 8.2 Hz, 1H), 4.00 (s, 3H), 3.36 (t, J = 7.4 Hz, 2H), 3.01 (t, *J* = 7.3 Hz, 2H), 2.18 (p, *J* = 7.5 Hz, 2H), 1.40 (s, 9H).

^{13}C{^{1}H} NMR (101 MHz, $CDCl_3$) δ 201.9, 140.5, 134.5, 133.8, 127.4, 125.1, 121.5, 119.5, 112.9, 44.1, 35.9, 32.6, 31.3, 29.0, 25.4.

1-(1-ethyl-1*H*-indol-3-yl)-2,2-dimethylpropan-1-one (1k) [6]

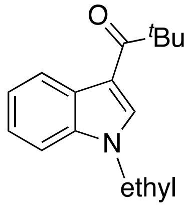

Colorless solid; ^{1}H NMR (400 MHz, $CDCl_3$) δ 8.52 (m, 1H), 7.84 (s, 1H), 7.35 (ddd, *J* = 7.9, 3.1, 1.6 Hz, 1H), 7.28 (m, 2H), 4.23 (q, *J* = 7.3 Hz, 2H), 1.53 (t, *J* = 7.3 Hz, 3H), 1.42 (s, 9H).

^{13}C{^{1}H} NMR (101 MHz, $CDCl_3$) δ 202.0, 135.5, 132.6, 128.5, 123.5, 123.1, 122.4, 112.9, 109.3, 44.1, 41.7, 29.0, 15.3.

2,2-dimethyl-1-(1-pentyl-1*H*-indol-3-yl)propan-1-one (1l) [6]

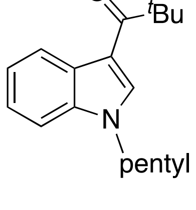

Colorless gum; ^{1}H NMR (400 MHz, MeOD) δ 8.37 (m, 1H), 8.16 (s, 1H), 7.35 (m, 1H), 7.21 (m, 1H), 7.16 (m, 1H), 4.14 (t, *J* = 7.1 Hz, 2H), 1.77 (p, *J* = 7.3 Hz, 2H), 1.38 (s, 9H), 1.24 (m, 4H), 0.83 (t, J = 7.1 Hz, 3H).

^{13}C{^{1}H} NMR (101 MHz, MeOD) δ 204.5, 137.2, 136.5, 129.6, 124.0, 123.9, 123.1, 113.3, 110.9, 47.6, 45.0, 30.7, 29.9, 29.4, 23.2, 14.3.

1-(1-hexyl-1*H*-indol-3-yl)-2,2-dimethylpropan-1-one (1m) [6]

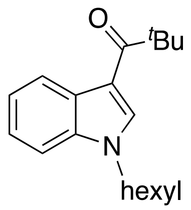

Colorless gum; ^{1}H NMR (400 MHz, MeOD) δ 8.36 (m, 1H), 8.19 (s, 1H), 7.39 (m, 1H), 7.23 (m, 1H), 7.18 (m, 1H), 4.20 (t, *J* = 7.0 Hz, 2H), 1.82 (p, *J* = 7.2 Hz, 2H), 1.39 (s, 9H), 1.27 (m, 6H), 0.84 (t, *J* = 7.1 Hz, 3H).

^{13}C{^{1}H} NMR (101 MHz, MeOD) δ 204.6, 137.2, 136.6, 129.6, 124.0, 124.0, 123.2, 113.3, 111.0, 47.7, 45.0, 32.4, 30.9, 29.4, 27.4, 23.6, 14.3.

1-(1-benzyl-1*H*-indol-3-yl)-2,2-dimethylpropan-1-one (1n) [6]

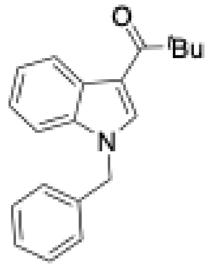

Colorless solid; ^{1}H NMR (400 MHz, $CDCl_3$) δ 8.53 (dt, *J* = 7.4, 1.4 Hz, 1H), 7.85 (s, 1H), 7.29 (m, 6H), 7.13 (m, 2H), 5.37 (s, 2H), 1.40 (s, 9H).

^{13}C{^{1}H} NMR (101 MHz, $CDCl_3$) δ 202.1, 136.0, 136.0, 133.6, 129.0, 128.4, 128.1, 126.7, 123.4, 123.3, 122.6, 113.2, 109.7, 50.6, 44.1, 28.9.

2,2-dimethyl-1-(1-phenyl-1*H*-indol-3-yl)propan-1-one (1o)[1]

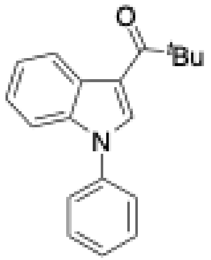

Colorless solid; ^{1}H NMR (400 MHz, $CDCl_3$) δ 8.58 (dt, *J* = 8.0, 1.0 Hz, 1H), 8.01 (s, 1H), 7.58 (m, 2H), 7.52 (m, 2H), 7.47 (m, 2H), 7.33 (ddd, *J* = 8.0, 7.0, 1.3 Hz, 1H), 7.28 (ddd, *J* = 8.4, 7.1, 1.5 Hz, 1H), 1.45 (s, 9H).

^{13}C{^{1}H} NMR (101 MHz, $CDCl_3$) δ 202.3, 138.5, 136.0, 133.1, 129.8, 128.5, 127.9, 125.1, 123.8, 123.5, 123.0, 114.5, 110.4, 44.3, 28.8.

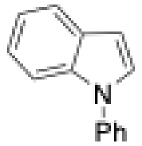

1-phenyl-1*H*-indole (1o') [1]

Colorless liquid; ^{1}H NMR (400 MHz, $CDCl_3$) δ 7.68 (m, 1H), 7.56 (m, 1H), 7.49 (m, 4H), 7.33 (m, 2H), 7.21 (ddd, *J* = 8.3, 7.0, 1.4 Hz, 1H), 7.16 (ddd, *J* = 8.3, 7.1, 1.2 Hz, 1H), 6.67 (dd, *J* = 3.3, 0.9 Hz, 1H).

^{13}C{^{1}H} NMR (101 MHz, $CDCl_3$) δ 139.8, 135.8, 129.6, 129.3, 127.9, 126.4, 124.3, 122.3, 121.1, 120.3, 110.5, 103.5.

1-(1*H*-indol-1-yl)-2,2-dimethylpropan-1-one (1p)[7]

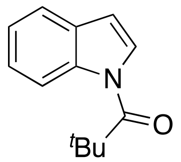

(Yellow solid)^{1}H NMR (400 MHz, $CDCl_3$) δ 8.52 (dq, *J* = 8.4, 0.9 Hz, 1H), 7.74 (d, *J* = 3.9 Hz, 1H), 7.56 (ddd, *J* = 7.6, 1.4, 0.7 Hz, 1H), 7.35 (ddd, *J* = 8.5, 7.2, 1.4 Hz, 1H), 7.26 (td, *J* = 7.5, 1.1 Hz, 1H), 6.62 (dd, *J* = 3.9, 0.8 Hz, 1H), 1.52 (s, 9H).

^{13}C{^{1}H} NMR (101 MHz, $CDCl_3$) δ 177.1, 136.8, 129.4, 125.7, 125.1, 123.6, 120.5, 117.3, 108.2, 41.3, 28.7.

Methyl 1-pivaloyl-1*H*-indole-5-carboxylate (1q)[8]

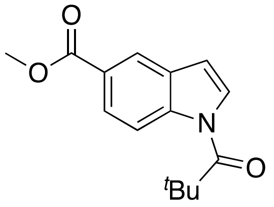

(Yellow solid) ^{1}H NMR (400 MHz, CDCl3) δ 8.54 (dd, *J* = 8.8, 0.8 Hz, 1H), 8.29 (m, 1H), 8.04 (dd, *J* = 8.8, 1.8 Hz, 1H), 7.80 (d, *J* = 3.9 Hz, 1H), 6.69 (dd, *J* = 3.9, 0.7 Hz, 1H), 3.94 (s, 3H), 1.53 (s, 9H).

^{13}C{^{1}H} NMR (101 MHz, $CDCl_3$) δ 177.2, 167.4, 139.4, 129.2, 126.9, 126.4, 125.4, 122.7, 117.0, 108.6, 52.1, 41.4, 28.6.

1-(5-methoxy-1*H*-indol-1-yl)-2,2-dimethylpropan-1-one (1r)[9]

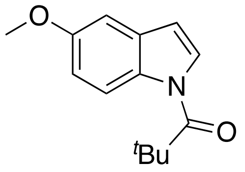

(Yellow solid) ^{1}H NMR (400 MHz, $CDCl_3$) δ 8.41 (d, *J* = 9.1 Hz, 1H), 7.71 (d, *J* = 3.8 Hz, 1H), 7.02 (d, *J* = 2.6 Hz, 1H), 6.95 (dd, *J* = 9.1, 2.6 Hz, 1H), 6.55 (dd, *J* = 3.8, 0.7 Hz, 1H), 3.86 (s, 3H), 1.51 (s, 9H).

^{13}C{^{1}H} NMR (101 MHz, $CDCl_3$) δ 176.7, 156.4, 131.5, 130.3, 126.3, 118.1, 113.4, 108.1, 103.3, 55.7, 41.1, 28.8.

1-(3-acetyl-1*H*-indol-1-yl)-2,2-dimethylpropan-1-one (1s)[10]

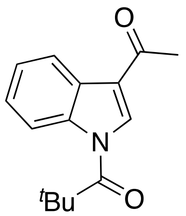

(colorless solid) ^{1}H NMR (400 MHz, MeOD) δ 8.57 (s, 1H), 8.37 (m, 1H), 8.27 (m, 1H), 7.35 (m, 2H), 2.61 (s, 3H), 1.57 (s, 9H).

^{13}C{^{1}H} NMR (101 MHz, MeOD) δ 196.7, 179.0, 138.7, 134.2, 127.5, 127.0, 125.8, 123.0, 121.7, 117.8, 42.8, 28.9, 27.8.

Methyl N^{α}-acetyl-1-pivaloyltryptophanate (1t)[11]

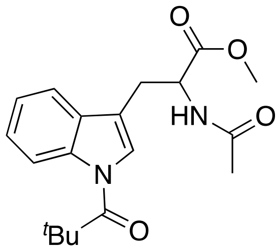

Yellow solid; ^{1}H NMR (400 MHz, $CDCl_3$) δ 8.50 (dt, *J* = 8.4, 1.0 Hz, 1H), 7.53 (d, *J* = 1.1 Hz, 1H), 7.46 (dt, *J* = 7.8, 1.0 Hz, 1H), 7.36 (ddd, *J* = 8.4, 7.2, 1.4 Hz, 1H), 7.29 (dd, J = 7.5, 1.2 Hz, 1H), 6.03 (d, *J* = 7.9 Hz, 1H), 5.03 (dt, *J* = 7.9, 5.5 Hz, 1H), 3.72 (s, 3H), 3.34 (ddd, *J* = 15.1, 5.7, 0.9 Hz, 1H), 3.23 (ddd, *J* = 15.0, 5.4, 1.0 Hz, 1H), 1.98 (s, 3H), 1.50 (s, 9H).

^{13}C{^{1}H} NMR (101 MHz, $CDCl_3$) δ 176.8, 172.2, 169.7, 137.0, 129.5, 125.6, 123.8, 123.6, 118.2, 117.5, 115.8, 52.6, 52.3, 41.2, 28.6, 27.5, 23.3.

1-pivaloyl-1*H*-indole-4-carbonitrile (1u)[12]

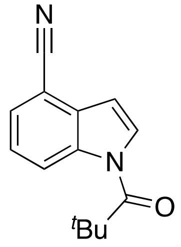

Yellow solid; ^{1}H NMR (400 MHz, $CDCl_3$) δ 8.74 (dd, *J* = 8.6, 1.0 Hz, 1H), 7.92 (d, *J* = 3.9 Hz, 1H), 7.60 (dd, *J* = 7.5, 0.9 Hz, 1H), 7.40 (dd, *J* = 8.5, 7.5 Hz, 1H), 6.86 (dd, *J* = 3.9, 0.7 Hz, 1H), 1.54 (s, 9H).

^{13}C{^{1}H} NMR (101 MHz, $CDCl_3$) δ 177.2, 136.6, 131.3, 128.1, 128.0, 125.0, 122.0, 117.8, 106.3, 103.5, 41.5, 28.6.

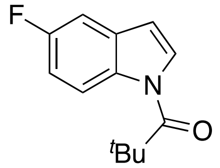

1-(5-fluoro-1*H*-indol-1-yl)-2,2-dimethylpropan-1-one (1v)[8]

Yellow solid; ^{1}H NMR (400 MHz, $CDCl_3$) δ 8.47 (dd, *J* = 9.1, 4.8 Hz, 1H), 7.78 (d, *J* = 3.8 Hz, 1H), 7.20 (dd, *J* = 8.7, 2.6 Hz, 1H), 7.06 (td, *J* = 9.1, 2.6 Hz, 1H), 6.58 (d, *J* = 3.8 Hz, 1H), 1.52 (s, 9H).

^{13}C{^{1}H} NMR (101 MHz, $CDCl_3$) δ 176.8, 159.6 (d, *J* = 239.9 Hz), 133.1, 130.3 (d, *J* = 10.1 Hz), 127.0, 118.3 (d, *J* = 8.8 Hz), 112.7 (d, *J* = 24.4 Hz), 107.8 (d, *J* = 4.0 Hz), 105.9 (d, *J* = 23.7 Hz), 41.2, 28.7.

1-(5-bromo-1*H*-indol-1-yl)-2,2-dimethylpropan-1-one (1w) [8]

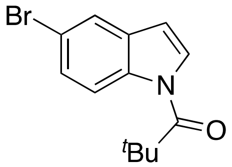

Yellow solid; ^{1}H NMR (400 MHz, $CDCl_3$) δ 8.38 (dt, *J* = 8.9, 0.6 Hz, 1H), 7.74 (d, *J* = 3.9 Hz, 1H), 7.68 (d, *J* = 2.0 Hz, 1H), 7.43 (dd, *J* = 8.9, 2.0 Hz, 1H), 6.56 (dd, *J* = 3.9, 0.7 Hz, 1H), 1.52 (s, 9H).

^{13}C{^{1}H} NMR (101 MHz, $CDCl_3$) δ 177.0, 135.5, 131.1, 127.9, 126.7, 123.1, 118.7, 116.8, 107.4, 41.3, 28.6.

5-bromo-1*H*-indole (1w')

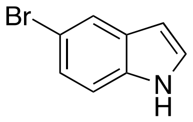

Colorless solid; ^{1}H NMR (400 MHz, MeOD) δ 7.67 (d, *J* = 1.9 Hz, 1H), 7.29 (dt, *J* = 8.6, 0.7 Hz, 1H), 7.25 (d, *J* = 3.2 Hz, 1H), 7.17 (dd, *J* = 8.6, 1.9 Hz, 1H), 6.40 (dd, *J* = 3.2, 0.9 Hz, 1H).

^{13}C{^{1}H} NMR (101 MHz, MeOD) δ 136.3, 131.2, 127.0, 124.9, 123.6, 113.7, 113.1, 102.0.

2,2-dimethyl-1-(5-nitro-1*H*-indol-1-yl)propan-1-one (1x) [8]

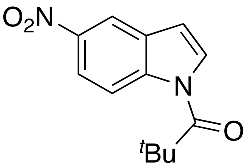

Yellow solid; ^{1}H NMR (400 MHz, $CDCl_3$) δ 8.61 (d, *J* = 9.2 Hz, 1H), 8.48 (d, *J* = 2.3 Hz, 1H), 8.23 (dd, J = 9.2, 2.4 Hz, 1H), 7.91 (d, *J* = 3.8 Hz, 1H), 6.77 (d, *J* = 3.8 Hz, 1H), 1.54 (s, 9H).

^{13}C{^{1}H} NMR (101 MHz, $CDCl_3$) δ 177.2, 144.2, 139.8, 129.3, 128.5, 120.3, 117.4, 116.7, 108.6, 41.5, 28.5.

5-nitro-1*H*-indole (1x')

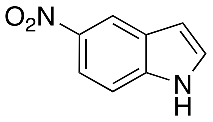

Yellow solid; ^{1}H NMR (400 MHz, $CDCl_3$) δ 8.62 (d, *J* = 2.2 Hz, 1H), 8.12 (dd, *J* = 8.9, 2.3 Hz, 1H), 7.44 (d, *J* = 9.0 Hz, 1H), 7.38 (dd, *J* = 3.2, 1.8 Hz, 1H), 6.74 (d, *J* = 3.5 Hz, 1H).

^{13}C{^{1}H} NMR (101 MHz, $CDCl_3$) δ 142.0, 138.7, 127.3, 127.2, 118.0, 117.7, 111.0, 105.1.

5-fluoro-1-(pyrimidin-2-yl)-1*H*-indole (1y)[13]

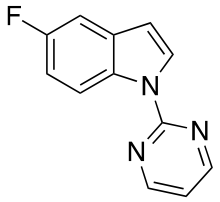

Brown solid; ^{1}H NMR (400 MHz, $CDCl_3$) δ 8.77 (ddt, *J* = 9.1, 4.8, 0.6 Hz, 1H), 8.70 (d, *J* = 4.8 Hz, 2H), 8.31 (d, *J* = 3.7 Hz, 1H), 7.26 (dd, *J* = 9.0, 2.6 Hz, 1H), 7.06 (m, 2H), 6.66 (dd, *J* = 3.6, 0.7 Hz, 1H).

^{13}C{^{1}H} NMR (101 MHz, $CDCl_3$) δ 158.9 (d, *J* = 238.0 Hz), 158.1, 157.5, 132.0 (d, *J* = 10.1 Hz), 131.8, 127.3, 117.2 (d, *J* = 9.0 Hz), 116.3, 111.3 (d, *J* = 24.9 Hz), 106.6 (d, *J* = 4.1 Hz), 106.0 (d, *J* = 23.5 Hz).

5-fluoro-1-(pyridin-2-yl)-1*H*-indole (1z)[14]

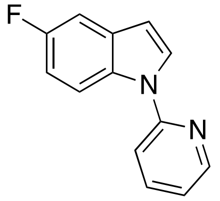

Brown solid; ^{1}H NMR (400 MHz, $CDCl_3$) δ 8.56 (ddd, *J* = 4.8, 1.9, 0.9 Hz, 1H), 8.23 (ddt, *J* = 9.1, 4.5, 0.7 Hz, 1H), 7.83 (ddd, *J* = 8.2, 7.4, 2.0 Hz, 1H), 7.73 (d, *J* = 3.5 Hz, 1H), 7.45 (dt, *J* = 8.2, 0.9 Hz, 1H), 7.29 (dd, *J* = 9.1, 2.6 Hz, 1H), 7.18 (ddd, *J* = 7.4, 4.9, 0.9 Hz, 1H), 7.03 (td, *J* = 9.1, 2.6 Hz, 1H), 6.67 (dd, *J* = 3.5, 0.8 Hz, 1H).

^{13}C{^{1}H} NMR (101 MHz, $CDCl_3$) δ 158.5 (d, *J* = 236.9 Hz), 152.4, 148.9, 138.5, 131.7, 130.9 (d, *J* = 10.3 Hz), 127.2, 120.1, 114.2 (d, *J* = 9.1 Hz), 114.1, 111.2 (d, *J* = 25.4 Hz), 105.9 (d, *J* = 23.3 Hz), 105.4 (d, *J* = 4.3 Hz).

5-chloro-*N*-(4-ethylphenyl)pyrimidin-2-amine (1aa)[15]

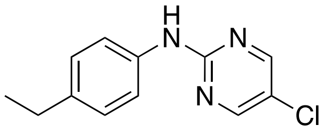

Brown solid; ^{1}H NMR (400 MHz, CDCl3) δ 8.32 (s, 2H), 7.45 (m, 2H), 7.22 (s, 1H), 7.18 (m, 2H), 2.63 (q, *J* = 7.6 Hz, 2H), 1.23 (t, *J* = 7.6 Hz, 3H).

^{13}C{^{1}H} NMR (101 MHz, $CDCl_3$) δ 158.4, 156.2, 139.4, 136.4, 128.3, 120.5, 120.0, 28.2, 15.7.

4.2. NMR data of carbazole substrates

1-(9*H*-carbazol-9-yl)-2,2-dimethylpropan-1-one (3a)

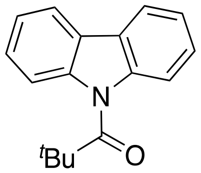

Colorless solid; ^{1}H NMR (400 MHz, $CDCl_3$) δ 8.01 (ddd, *J* = 7.8, 1.4, 0.7 Hz, 2H), 7.66 (dt, *J* = 8.4, 0.8 Hz, 2H), 7.43 (ddd, *J* = 8.4, 7.2, 1.3 Hz, 2H), 7.30 (ddd, *J* = 8.1, 7.3, 1.0 Hz, 2H), 1.51 (s, 9H).

$^{13}C\{^{1}H\}$ NMR (101 MHz, $CDCl_3$) δ 184.1, 139.2, 126.3, 124.7, 121.8, 120.0, 113.7, 43.7, 28.4.

HRMS: (ESI) m/z calculated for $C_{17}H_{17}NO$ $[M+H]^+$ 252.1389, found 252.1387

9*H*-carbazole (3a')

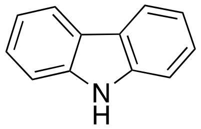

^{1}H NMR (400 MHz, MeOD) δ 8.03 (dt, *J* = 7.8, 1.0 Hz, 2H), 7.43 (dt, *J* = 8.2, 1.0 Hz, 2H), 7.35 (ddd, *J* = 8.2, 7.1, 1.2 Hz, 2H), 7.14 (ddd, J = 8.0, 7.0, 1.1 Hz, 2H).

$^{13}C\{^{1}H\}$ NMR (101 MHz, MeOD) δ 141.4, 126.4, 124.3, 120.8, 119.6, 111.6.

1-(3,6-dimethyl-9*H*-carbazol-9-yl)-2,2-dimethylpropan-1-one (3b)

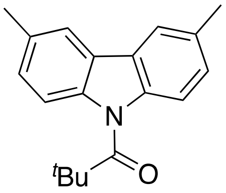

Colorless solid; ^{1}H NMR (400 MHz, $CDCl_3$) δ 7.77 (dt, *J* = 1.7, 0.8 Hz, 2H), 7.63 (d, *J* = 8.4 Hz, 2H), 7.23 (m, 2H), 2.50 (s, 6H), 1.52 (s, 9H).

$^{13}C\{^{1}H\}$ NMR (101 MHz, $CDCl_3$) δ 183.0, 137.6, 131.5, 127.5, 125.1, 119.9, 114.2, 43.2, 28.4, 21.2.

HRMS: (ESI) m/z calculated for $C_{19}H_{21}NO$ $[M+H]^+$ 280.1696, found 280.1709

3,6-dimethyl-9*H*-carbazole (3b')

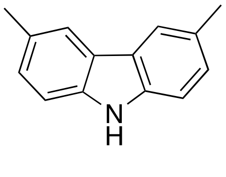

^{1}H NMR (400 MHz, $CDCl_3$) δ 7.83 (m, 3H), 7.28 (d, *J* = 8.2 Hz, 2H), 7.21 (dd, *J* = 8.3, 1.7 Hz, 2H), 2.52 (s, 6H).

$^{13}C\{^{1}H\}$ NMR (101 MHz, $CDCl_3$) δ 138.1, 128.5, 127.0, 123.4, 120.2, 110.2, 21.4.

1-(3-(*tert*-butyl)-9*H*-carbazol-9-yl)-2,2-dimethylpropan-1-one (3c)

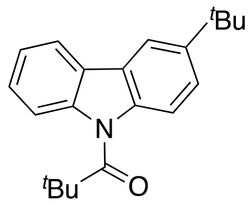

Colorless solid; ^{1}H NMR (400 MHz, MeOD) δ 8.07 (ddd, *J* = 7.7, 1.6, 0.7 Hz, 2H), 7.67 (dt, *J* = 8.4, 0.9 Hz, 1H), 7.60 (dd, *J* = 8.8, 0.7 Hz, 1H), 7.52 (dd, *J* = 8.8, 2.0 Hz, 1H), 7.41 (ddd, *J* = 8.4, 7.2, 1.3 Hz, 1H), 7.29 (m, 1H), 1.48 (s, 9H), 1.43 (s, 9H).

^{13}C{^{1}H} NMR (101 MHz, MeOD) δ 185.3, 146.4, 140.9, 138.5, 127.3, 126.3, 125.8, 125.3, 123.0, 120.9, 117.2, 114.9, 114.5, 44.6, 35.6, 32.2, 28.6.

HRMS: (ESI) m/z calculated for $C_{21}H_{25}NO$ $[M+H]^+$ 308.2009, found 308.2007

3-(*tert*-butyl)-9*H*-carbazole (3c')

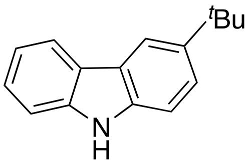

^{1}H NMR (400 MHz, MeOD) δ 8.05 (dd, *J* = 1.9, 0.7 Hz, 1H), 8.03 (dt, *J* = 7.8, 1.0 Hz, 1H), 7.46 (dd, *J* = 8.6, 1.9 Hz, 1H), 7.40 (dt, *J* = 8.2, 1.0 Hz, 1H), 7.36 (dd, *J* = 8.6, 0.7 Hz, 1H), 7.32 (ddd, *J* = 8.2, 7.1, 1.2 Hz, 1H), 7.12 (ddd, *J* = 8.0, 7.0, 1.0 Hz, 1H), 1.43 (s, 9H).

^{13}C{^{1}H} NMR (101 MHz, MeOD) δ 142.6, 141.9, 139.6, 126.2, 124.6, 124.5, 124.0, 120.7, 119.4, 116.8, 111.6, 111.2, 35.5, 32.5.

1-(3,6-di-*tert*-butyl-9*H*-carbazol-9-yl)-2,2-dimethylpropan-1-one (3d)

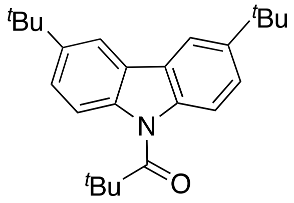

Colorless solid; ^{1}H NMR (400 MHz, $CDCl_3$) δ 7.99 (dd, *J* = 2.0, 0.6 Hz, 2H), 7.72 (dd, *J* = 8.8, 0.6 Hz, 2H), 7.47 (dd, *J* = 8.8, 2.0 Hz, 2H), 1.55 (s, 9H), 1.44 (s, 18H).

^{13}C{^{1}H} NMR (101 MHz, $CDCl_3$) δ 182.7, 145.2, 137.6, 125.2, 124.0, 115.9, 114.4, 42.9, 34.7, 31.8, 28.4.

HRMS: (ESI) m/z calculated for $C_{25}H_{33}NO$ $[M+H]^+$ 364.2635, found 364.2650

3,6-di-*tert*-butyl-9*H*-carbazole (3d')

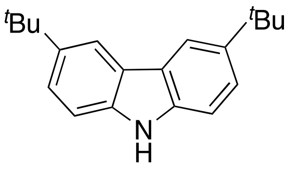

^{1}H NMR (400 MHz, $CDCl_3$) δ 8.07 (d, *J* = 1.9 Hz, 2H), 7.74 (s, 1H), 7.45 (dd, *J* = 8.5, 1.9 Hz, 2H), 7.28 (d, *J* = 8.5 Hz, 2H), 1.45 (s, 18H).

^{13}C{^{1}H} NMR (101 MHz, $CDCl_3$) δ 142.2, 138.0, 123.5, 123.2, 116.1, 110.0, 34.6, 32.0.

1-(2-methoxy-9*H*-carbazol-9-yl)-2,2-dimethylpropan-1-one (3e)

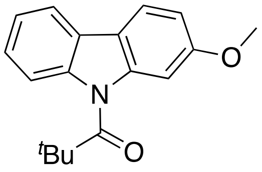

Colorless solid; ^{1}H NMR (400 MHz, $CDCl_3$) δ 7.91 (ddd, *J* = 7.7, 1.4, 0.7 Hz, 1H), 7.88 (d, *J* = 8.6 Hz, 1H), 7.60 (dt, *J* = 8.3, 0.9 Hz, 1H), 7.35 (ddd, *J* = 8.3, 7.2, 1.4 Hz, 1H), 7.27 (td, *J* = 7.5, 1.0 Hz, 1H), 7.20 (d, *J* = 2.2 Hz, 1H), 6.92 (dd, *J* = 8.6, 2.2 Hz, 1H), 3.90 (s, 3H), 1.52 (s, 9H).

^{13}C{^{1}H} NMR (101 MHz, $CDCl_3$) δ 184.1, 159.2, 140.6, 139.1, 125.0, 124.9, 121.9, 120.6, 119.3, 118.4, 113.7, 110.0, 98.5, 55.7, 43.7, 28.4.

HRMS: (ESI) m/z calculated for $C_{18}H_{19}NO_2$ $[M+H]^+$ 282.1489, found 282.1500

2-methoxy-9*H*-carbazole (3e')

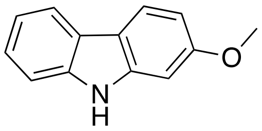

^{1}H NMR (400 MHz, $CDCl_3$) δ 8.04 – 7.89 (m, 3H), 7.38 (dt, *J* = 8.0, 1.1 Hz, 1H), 7.34 (ddd, *J* = 8.1, 6.9, 1.2 Hz, 1H), 7.20 (ddd, *J* = 8.1, 6.9, 1.3 Hz, 1H), 6.91 (d, *J* = 2.2 Hz, 1H), 6.86 (dd, *J* = 8.6, 2.3 Hz, 1H), 3.90 (s, 3H).

^{13}C{^{1}H} NMR (101 MHz, $CDCl_3$) δ 159.1, 140.8, 139.5, 124.6, 123.5, 121.1, 119.6, 119.5, 117.3, 110.3, 108.2, 94.7, 55.6.

2-methoxy-9*H*-carbazole (3e')

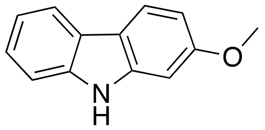

^{1}H NMR (400 MHz, MeOD) δ 7.92 (dt, *J* = 7.8, 1.0 Hz, 1H), 7.88 (d, *J* = 8.6 Hz, 1H), 7.37 (dt, *J* = 8.1, 0.9 Hz, 1H), 7.26 (ddd, *J* = 8.2, 7.1, 1.2 Hz, 1H), 7.10 (ddd, *J* = 8.0, 7.1, 1.1 Hz, 1H), 6.96 (d, *J* = 2.3 Hz, 1H), 6.77 (dd, *J* = 8.6, 2.3 Hz, 1H), 3.87 (s, 3H).

^{13}C{^{1}H} NMR (101 MHz, MeOD) δ 160.4, 142.8, 141.5, 125.2, 124.6, 121.6, 120.0, 119.7, 118.2, 111.3, 108.8, 95.4, 55.9.

2,2-dimethyl-1-(3-phenyl-9*H*-carbazol-9-yl)propan-1-one (3f)

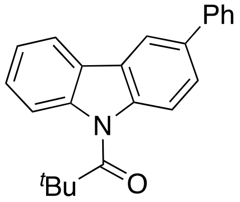

Colorless solid; ^{1}H NMR (400 MHz, $CDCl_3$) δ 8.22 (dd, *J* = 1.8, 0.7 Hz, 1H), 8.07 (m, 1H), 7.75 (dd, *J* = 8.6, 0.7 Hz, 1H), 7.70 (m, 4H), 7.47 (m, 3H), 7.36 (m, 2H), 1.55 (s, 9H).

^{13}C{^{1}H} NMR (101 MHz, $CDCl_3$) δ 183.8, 141.3, 139.6, 138.6, 135.3, 128.8, 127.3, 126.9, 126.5, 125.8, 125.3, 124.8, 122.0, 120.1, 118.4, 114.1, 114.0, 43.6, 28.4.

HRMS: (ESI) m/z calculated for $C_{23}H_{21}NO$ $[M+H]^+$ 328.1696, found 328.1694

3-phenyl-9*H*-carbazole (3f')

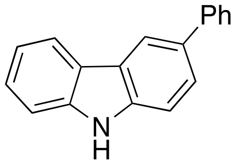

^{1}H NMR (400 MHz, MeOD) δ 8.29 (dd, *J* = 1.8, 0.7 Hz, 1H), 8.12 (dt, *J* = 7.8, 1.0 Hz, 1H), 7.71 (m, 2H), 7.65 (dd, *J* = 8.4, 1.8 Hz, 1H), 7.51 (dd, *J* = 8.5, 0.7 Hz, 1H), 7.44 (m, 3H), 7.38 (ddd, *J* = 8.2, 7.0, 1.2 Hz, 1H), 7.29 (m, 1H), 7.17 (ddd, *J* = 7.9, 7.1, 1.1 Hz, 1H).

^{13}C{^{1}H} NMR (101 MHz, $CDCl_3$) δ 143.7, 142.0, 141.0, 133.4, 129.7, 128.0, 127.2, 126.7, 125.9, 124.9, 124.4, 121.0, 119.8, 119.2, 111.9, 111.8.

1-(3,6-diphenyl-9*H*-carbazol-9-yl)-2,2-dimethylpropan-1-one (3g)

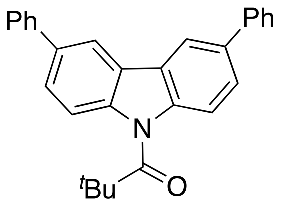

Colorless solid; ^{1}H NMR (400 MHz, $CDCl_3$) δ 8.28 (dd, *J* = 1.9, 0.7 Hz, 2H), 7.79 (dd, *J* = 8.7, 0.6 Hz, 2H), 7.71 (ddd, *J* = 8.6, 5.8, 1.6 Hz, 6H), 7.49 (m, 4H), 7.37 (m, 2H), 1.58 (s, 9H).

^{13}C{^{1}H} NMR (101 MHz, $CDCl_3$) δ 183.5, 141.2, 139.1, 135.5, 128.9, 127.3, 127.0, 126.0, 125.5, 118.5, 114.4, 43.5, 28.4.

HRMS: (ESI) m/z calculated for $C_{29}H_{25}NO$ $[M+H]^+$ 404.2009, found 404.2006

3,6-diphenyl-9*H*-carbazole (3g')

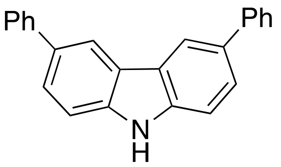

^{1}H NMR (400 MHz, MeOD) δ 8.36 (dd, *J* = 1.9, 0.7 Hz, 2H), 7.72 (m, 4H), 7.67 (dd, *J* = 8.4, 1.8 Hz, 2H), 7.52 (dd, *J* = 8.4, 0.7 Hz, 2H), 7.44 (dd, *J* = 8.4, 7.0 Hz, 4H), 7.29 (m, 2H).

^{13}C{^{1}H} NMR (101 MHz, MeOD) δ 143.6, 141.5, 133.6, 129.7, 128.1, 127.3, 126.2, 125.0, 119.4, 112.1.

2,2-dimethyl-1-(2-phenyl-9*H*-carbazol-9-yl)propan-1-one (3h)

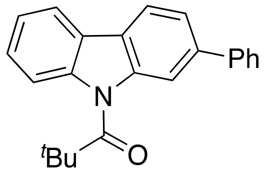

Colorless solid; ^{1}H NMR (400 MHz, $CDCl_3$) δ 8.06 (dd, *J* = 8.1, 0.6 Hz, 1H), 8.03 (ddd, *J* = 7.7, 1.3, 0.7 Hz, 1H), 7.87 (dd, *J* = 1.5, 0.6 Hz, 1H), 7.67 (m, 3H), 7.56 (dd, *J* = 8.1, 1.5 Hz, 1H), 7.46 (m, 3H), 7.37 (m, 1H), 7.32 (m, 1H), 1.54 (s, 9H).

^{13}C{^{1}H} NMR (101 MHz, $CDCl_3$) δ 184.1, 141.6, 139.9, 139.8, 139.6, 128.9, 127.5, 127.3, 126.4, 124.5, 123.9, 122.0, 121.5, 120.3, 120.1, 113.8, 112.3, 43.8, 28.5.

HRMS: (ESI) m/z calculated for $C_{23}H_{21}NO$ $[M+H]^+$ 328.1696, found 328.1696

2-phenyl-9*H*-carbazole (3h')

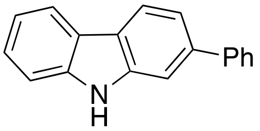

^{1}H NMR (400 MHz, MeOD) δ 8.10 (d, *J* = 8.1 Hz, 1H), 8.06 (dt, *J* = 7.8, 1.0 Hz, 1H), 7.70 (m, 2H), 7.67 (dd, *J* = 1.6, 0.6 Hz, 1H), 7.45 (m, 4H), 7.37 (m, 1H), 7.33 (m, 1H), 7.16 (ddd, *J* = 8.0, 7.1, 1.0 Hz, 1H).

^{13}C{^{1}H} NMR (101 MHz, MeOD) δ 143.6, 142.1, 142.0, 140.2, 129.8, 128.3, 127.9, 126.6, 124.1, 123.7, 121.2, 121.0, 119.9, 119.3, 111.7, 110.0.

1-(2-fluoro-9*H*-carbazol-9-yl)-2,2-dimethylpropan-1-one (3i)

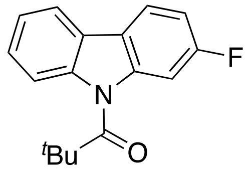

Colorless solid; ^{1}H NMR (400 MHz, $CDCl_3$) δ 7.98 (m, 1H), 7.94 (dd, *J* = 8.7, 5.6 Hz, 1H), 7.63 (dt, *J* = 8.3, 0.9 Hz, 1H), 7.41 (m, 2H), 7.32 (td, *J* = 7.5, 1.0 Hz, 1H), 7.06 (td, *J* = 8.8, 2.3 Hz, 1H), 1.52 (s, 9H).

^{13}C{^{1}H} NMR (101 MHz, $CDCl_3$) δ 183.7, 162.0 (d, J = 242.5 Hz), 139.8 (d, *J* = 11.9 Hz), 125.8, 122.1, 120.9 (d, *J* = 1.5 Hz), 120.8 (d, *J* = 10.2 Hz), 119.7, 113.8, 109.9 (d, *J* = 24.0 Hz), 101.1 (d, *J* = 28.3 Hz), 43.6, 28.3.

HRMS: (ESI) m/z calculated for $C_{17}H_{16}FNO$ $[M+H]^+$ 270.1289, found 270.1296

2-fluoro-9*H*-carbazole (3i')

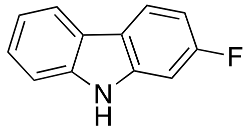

^{1}H NMR (400 MHz, $CDCl_3$) δ 8.05 (s, 1H), 8.01 (dq, *J* = 7.8, 0.9 Hz, 1H), 7.98 (dd, *J* = 8.6, 5.4 Hz, 1H), 7.40 (m, 2H), 7.24 (ddd, *J* = 8.1, 5.8, 2.4 Hz, 1H), 7.09 (dd, *J* = 9.6, 2.3 Hz, 1H), 6.97 (ddd, *J* = 9.5, 8.6, 2.3 Hz, 1H).

^{13}C{^{1}H} NMR (101 MHz, $CDCl_3$) δ 162.0 (d, *J* = 241.9 Hz), 140.0 (d, *J* = 12.4 Hz), 139.9, 125.5, 123.0, 121.2 (d, *J* = 10.8 Hz), 119.9, 119.9, 119.8 (d, *J* = 1.0 Hz), 110.6, 107.7 (d, *J* = 24.1 Hz), 97.4 (d, *J* = 26.3 Hz).

1-(2-chloro-9*H*-carbazol-9-yl)-2,2-dimethylpropan-1-one (3j)

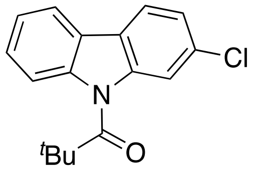

Colorless solid; ^{1}H NMR (400 MHz, $CDCl_3$) δ 7.99 (ddd, *J* = 7.8, 1.3, 0.7 Hz, 1H), 7.93 (d, *J* = 8.3 Hz, 1H), 7.67 (d, *J* = 1.8 Hz, 1H), 7.62 (dt, *J* = 8.4, 0.9 Hz, 1H), 7.45 (ddd, *J* = 8.4, 7.2, 1.3 Hz, 1H), 7.33 (m, 1H), 7.29 (dd, *J* = 8.3, 1.8 Hz, 1H), 1.52 (s, 9H).

^{13}C{^{1}H} NMR (101 MHz, $CDCl_3$) δ 183.8, 139.8, 139.2, 132.2, 126.5, 124.0, 123.1, 122.3, 122.1, 120.7, 120.1, 113.8, 113.8, 43.7, 28.3.

HRMS: (ESI) m/z calculated for $C_{17}H_{16}ClNO$ $[M+H]^+$ 286.0993, found 286.0997

2-chloro-9*H*-carbazole (3j')

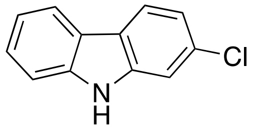

^{1}H NMR (400 MHz, MeOD) δ 8.03 (dt, *J* = 7.9, 1.0 Hz, 1H), 8.00 (dd, J = 8.4, 0.5 Hz, 1H), 7.45 (m, 2H), 7.39 (ddd, *J* = 8.2, 7.0, 1.2 Hz, 1H), 7.18 (ddd, *J* = 8.0, 7.0, 1.1 Hz, 1H), 7.14 (dd, *J* = 8.3, 1.9 Hz, 1H).

^{13}C{^{1}H} NMR (101 MHz, MeOD) δ 142.0, 141.8, 132.0, 126.9, 123.7, 123.0, 121.9, 120.9, 120.2, 119.9, 111.8, 111.5.

1-(3,6-dichloro-9*H*-carbazol-9-yl)-2,2-dimethylpropan-1-one (3k)

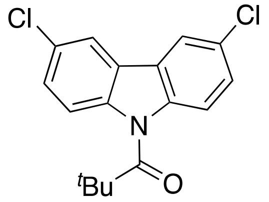

Colorless solid; ^{1}H NMR (400 MHz, $CDCl_3$) δ 7.94 (d, *J* = 2.1 Hz, 2H), 7.59 (d, *J* = 8.8 Hz, 2H), 7.42 (dd, *J* = 8.8, 2.1 Hz, 2H), 1.50 (s, 9H).

^{13}C{^{1}H} NMR (101 MHz, $CDCl_3$) δ 183.3, 138.0, 127.8, 127.2, 125.0, 120.0, 115.0, 43.7, 28.3.

HRMS: (ESI) m/z calculated for $C_{17}H_{15}Cl_2NO$ $[M+H]^+$ 320.0603, found 320.0616

3,6-dichloro-9*H*-carbazole (3k')

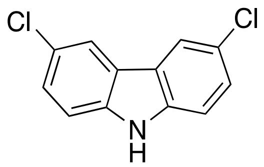

^{1}H NMR (400 MHz, $CDCl_3$) δ 8.07 (s, 1H), 7.97 (d, *J* = 1.9 Hz, 2H), 7.39 (dd, *J* = 8.6, 2.0 Hz, 2H), 7.34 (d, *J* = 8.6 Hz, 2H).

^{13}C{^{1}H} NMR (101 MHz, $CDCl_3$) δ 138.2, 126.7, 125.3, 123.6, 120.2, 111.8.

1-(2-bromo-9*H*-carbazol-9-yl)-2,2-dimethylpropan-1-one (3l)

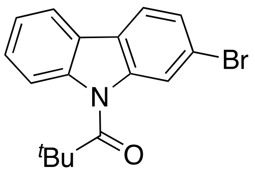

Colorless solid; ^{1}H NMR (400 MHz, $CDCl_3$) δ 8.00 (dt, *J* = 7.7, 1.0 Hz, 1H), 7.87 (d, *J* = 8.3 Hz, 1H), 7.83 (d, *J* = 1.6 Hz, 1H), 7.62 (dt, *J* = 8.4, 0.9 Hz, 1H), 7.45 (m, 2H), 7.33 (td, *J* = 7.5, 1.0 Hz, 1H), 1.52 (s, 9H).

^{13}C{^{1}H} NMR (101 MHz, $CDCl_3$) δ 183.8, 140.0, 139.0, 126.6, 125.1, 124.0, 123.5, 122.1, 121.1, 120.1, 120.0, 116.7, 113.8, 43.7, 28.3.

HRMS: (ESI) m/z calculated for $C_{17}H_{16}BrNO$ $[M+H]^+$ 330.0488, found 330.0497

2-bromo-9*H*-carbazole (3l')

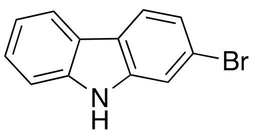

^{1}H NMR (400 MHz, $CDCl_3$) δ 8.04 (dq, *J* = 7.7, 0.9 Hz, 1H), 7.92 (d, *J* = 8.2 Hz, 1H), 7.59 (d, *J* = 1.7 Hz, 1H), 7.44 (m, 2H), 7.34 (dd, *J* = 8.3, 1.7 Hz, 1H), 7.25 (m, 1H).

^{13}C{^{1}H} NMR (101 MHz, $CDCl_3$) δ 140.2, 139.5, 126.3, 122.7, 122.7, 122.3, 121.4, 120.3, 119.9, 119.2, 113.6, 110.7.

1-(3-bromo-9*H*-carbazol-9-yl)-2,2-dimethylpropan-1-one (3m)

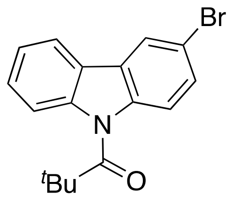

Colorless solid; ^{1}H NMR (400 MHz, $CDCl_3$) δ 8.14 (dd, *J* = 1.8, 0.7 Hz, 1H), 7.98 (ddd, *J* = 7.8, 1.3, 0.7 Hz, 1H), 7.65 (dt, *J* = 8.4, 0.9 Hz, 1H), 7.55 (dd, *J* = 8.9, 0.7 Hz, 1H), 7.52 (dd, *J* = 8.8, 1.8 Hz, 1H), 7.47 (ddd, *J* = 8.4, 7.2, 1.3 Hz, 1H), 7.33 (ddd, *J* = 8.0, 7.3, 1.0 Hz, 1H), 1.51 (s, 9H).

$^{13}C\{^{1}H\}$ NMR (101 MHz, $CDCl_3$) δ 183.7, 139.4, 137.9, 129.1, 127.1, 126.5, 123.6, 122.8, 122.1, 120.3, 115.1, 114.8, 113.8, 43.7, 28.3.

HRMS: (ESI) m/z calculated for $C_{17}H_{16}BrNO$ $[M+H]^+$ 330.0488, found 330.0491

3-bromo-9*H*-carbazole (3m')

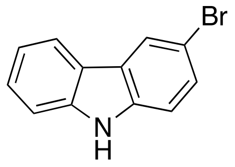

^{1}H NMR (400 MHz, $CDCl_3$) δ 8.19 (m, 1H), 8.05 (s, 1H), 8.02 (dq, *J* = 7.9, 0.9 Hz, 1H), 7.49 (dd, *J* = 8.6, 2.0 Hz, 1H), 7.43 (m, 2H), 7.30 (dd, *J* = 8.6, 0.5 Hz, 1H), 7.25 (m, 1H).

$^{13}C\{^{1}H\}$ NMR (101 MHz, $CDCl_3$) δ 139.8, 138.0, 128.5, 126.6, 125.2, 123.1, 122.4, 120.5, 119.9, 112.2, 112.0, 110.8.

1-(3,6-dibromo-9*H*-carbazol-9-yl)-2,2-dimethylpropan-1-one (3n)

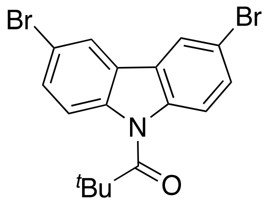

Colorless solid; ^{1}H NMR (400 MHz, $CDCl_3$) δ 8.10 (dd, *J* = 1.9, 0.7 Hz, 2H), 7.55 (dd, *J* = 8.8, 1.9 Hz, 2H), 7.52 (dd, *J* = 8.8, 0.7 Hz, 2H), 1.49 (s, 9H).

$^{13}C\{^{1}H\}$ NMR (101 MHz, $CDCl_3$) δ 183.3, 138.2, 129.9, 125.3, 123.1, 115.3, 115.1, 43.8, 28.3.

HRMS: (ESI) m/z calculated for $C_{17}H_{15}Br_2NO$ $[M+H]^+$ 407.9593, found 407.9589

3,6-dibromo-9*H*-carbazole (3n')

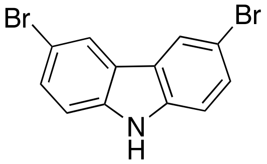

^{1}H NMR (400 MHz, $CDCl_3$) δ 8.12 (d, *J* = 1.9 Hz, 2H), 8.09 (s, 1H), 7.52 (dd, *J* = 8.6, 1.9 Hz, 2H), 7.30 (d, *J* = 8.5 Hz, 2H).

$^{13}C\{^1H\}$ NMR (101 MHz, $CDCl_3$) δ 138.4, 129.3, 124.1, 123.3, 112.7, 112.2.

1-(9*H*-carbazol-9-yl)ethan-1-one (commercial) 3o'

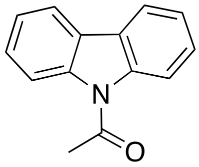

Colorless solid; ^{1}H NMR (400 MHz, $CDCl_3$) δ 8.22 (d, *J* = 8.4 Hz, 2H), 8.00 (m, 2H), 7.48 (ddd, *J* = 8.5, 7.2, 1.4 Hz, 2H), 7.39 (td, *J* = 7.5, 1.0 Hz, 2H), 2.89 (s, 3H).

$^{13}C\{^1H\}$ NMR (101 MHz, $CDCl_3$) δ 170.1, 138.6, 127.4, 126.4, 123.7, 119.8, 116.3, 27.8.

1-(5*H*-dibenzo[*b,f*]azepin-5-yl)-2,2-dimethylpropan-1-one (3p)

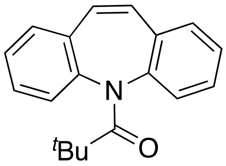

Colorless solid; ^{1}H NMR (400 MHz, $CDCl_3$) δ 7.46 (dd, *J* = 7.9, 1.5 Hz, 2H), 7.41 (td, *J* = 7.4, 1.7 Hz, 2H), 7.37 (dd, *J* = 7.8, 1.7 Hz, 2H), 7.30 (ddd, *J* = 7.9, 6.9, 1.5 Hz, 2H), 7.00 (s, 2H), 0.99 (s, 9H).

$^{13}C\{^1H\}$ NMR (101 MHz, $CDCl_3$) δ 178.0, 141.1, 134.7, 130.3, 128.9, 128.8, 128.7, 127.3, 40.6, 28.9.

HRMS: (ESI) m/z calculated for $C_{19}H_{19}NO$ $[M+H]^+$ 278.1539, found 278.1546

5*H*-dibenzo[*b,f*]azepine (3p')

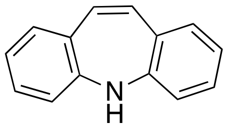

^{1}H NMR (400 MHz, $CDCl_3$) δ 7.02 (td, *J* = 7.5, 2.0 Hz, 2H), 6.86 (dd, *J* = 7.5, 2.0 Hz, 2H), 6.82 (td, *J* = 7.3, 1.1 Hz, 2H), 6.49 (dd, *J* = 7.9, 1.1 Hz, 2H), 6.31 (s, 2H), 4.93 (s, 1H).

$^{13}C\{^1H\}$ NMR (101 MHz, $CDCl_3$) δ 148.4, 132.1, 130.5, 129.7, 129.5, 123.0, 119.3.

1-(9H-Carbazol-4-yloxy)-3-[[2-(2-methoxyphenoxy)ethyl]amino]-2-propanol (3q')

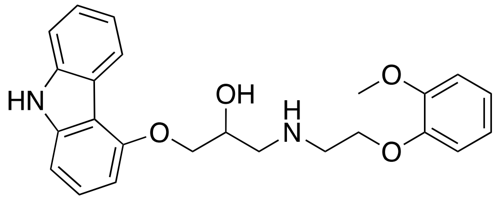

Colorless solid; ^{1}H NMR (400 MHz, $CDCl_3$) δ 8.26 (dd, *J* = 7.8, 1.1 Hz, 1H), 8.13 (s, 1H), 7.38 (m, 2H), 7.31 (t, *J* = 8.0 Hz, 1H), 7.21 (ddd, *J* = 8.1, 6.5, 1.7 Hz, 1H), 7.05 (d, *J* = 8.1 Hz, 1H), 6.91 (m, 4H), 6.67 (d, *J* = 8.0 Hz, 1H), 4.26 (dtd, *J* = 19.4, 7.6, 3.7 Hz, 3H), 4.15 (t, J = 5.2 Hz, 2H), 3.83 (s, 3H), 3.44 (s, 1H), 3.11 (m, 3H), 2.99 (dd, *J* = 12.3, 7.0 Hz, 1H), 1.77 (s, 1H).

^{13}C{^{1}H} NMR (101 MHz, $CDCl_3$) δ 155.2, 149.7, 148.2, 140.9, 138.7, 126.7, 125.0, 123.0, 122.5, 121.6, 120.9, 119.7, 114.1, 112.7, 111.9, 110.0, 103.8, 101.3, 70.3, 68.9, 68.5, 55.8, 51.9, 48.7.

1-((9*H*-carbazol-4-yl)oxy)-3-(*N*-(2-(2-methoxyphenoxy)ethyl)pivalamido)propan-2-yl pivalate (3r)

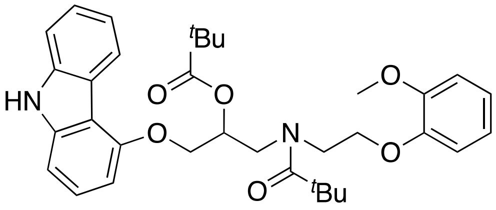

Colorless solid; ^{1}H NMR (400 MHz, $CDCl_3$) δ 8.29 (d, *J* = 7.8 Hz, 1H), 8.17 (s, 1H), 7.38 (m, 1H), 7.34 (m, 1H), 7.30 (t, *J* = 8.0 Hz, 1H), 7.15 (ddd, *J* = 8.0, 6.8, 1.4 Hz, 1H), 7.05 (d, *J* = 8.1 Hz, 1H), 6.92 (ddd, *J* = 7.8, 5.4, 3.8 Hz, 1H), 6.84 (m, 3H), 6.61 (d, *J* = 8.0 Hz, 1H), 5.78 (qd, *J* = 5.9, 3.0 Hz, 1H), 4.39 (dd, *J* = 10.5, 3.2 Hz, 1H), 4.33 (dd, *J* = 10.5, 5.1 Hz, 1H), 4.18 (td, *J* = 5.9, 1.6 Hz, 2H), 4.04 (m, 4H), 3.75 (s, 3H), 1.33 (s, 9H), 1.18 (s, 9H).

$^{13}C\{^1H\}$ NMR (101 MHz, $CDCl_3$) δ 178.4, 178.1, 155.0, 149.7, 148.0, 141.0, 138.7, 126.6, 125.0, 123.3, 122.5, 121.9, 120.9, 119.6, 114.2, 112.7, 112.0, 109.9, 104.0, 100.9, 70.3, 67.8, 67.5, 55.7, 48.8, 48.8, 39.3, 38.9, 28.6, 27.2.

HRMS: (ESI) m/z calculated for $C_{34}H_{42}N_2O_6$ $[M+H]^+$ 575.3116, found 575.3119

1-(*N*-(2-(2-methoxyphenoxy)ethyl)pivalamido)-3-((9-pivaloyl-9*H*-carbazol-4-yl)oxy)propan-2-yl pivalate (3s)

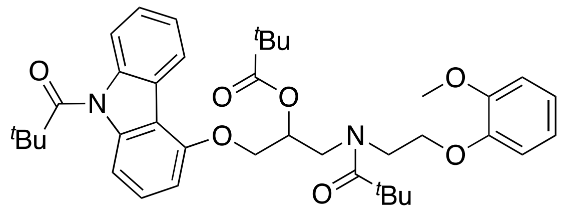

Colorless solid; ^1H NMR (400 MHz, MeOD) δ 8.27 (d, J = 7.7 Hz, 1H), 7.52 (dt, J = 8.3, 0.9 Hz, 1H), 7.37 (m, 2H), 7.19 (m, 2H), 6.86 (m, 5H), 5.86 (m, 1H), 4.38 (t, J = 3.8 Hz, 2H), 4.20 (m, 2H), 4.00 (m, 4H), 3.68 (s, 3H), 1.45 (s, 9H), 1.32 (s, 9H), 1.15 (s, 9H).

$^{13}C\{^1H\}$ NMR (101 MHz, MeOD) δ 186.38, 180.6, 179.5, 156.1, 151.2, 149.3, 141.7, 139.7, 128.3, 126.6, 124.6, 124.5, 123.1, 122.7, 121.9, 115.5, 114.6, 113.3, 113.2, 107.1, 104.4, 71.4, 69.1, 56.1, 49.5, 45.1, 40.3, 39.8, 29.1, 28.6, 27.6.

HRMS: (ESI) m/z calculated for $C_{39}H_{50}N_2O_7$ $[M+H]^+$ 659.3691, found 659.3710

4.3. Characterization of deuterated products

4.3.1. Characterization of deuterated indoles

2,2-dimethyl-1-(1-methyl-1*H*-indol-3-yl-2,4,5,6,7-*d*5)propan-1-one (2a)

Isolated yield: 43 mg, 86 %; Physical appearance: Brown solid; m.p: 129–131 °C.

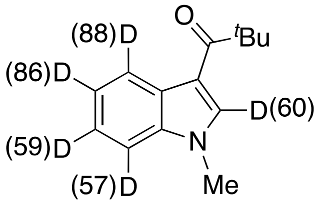

^{1}H NMR (400 MHz, MeOD) δ **8.33 (m, 0.12H), 8.20 (s, 0.40H), 7.42 (m, 0.43H), 7.27 (m, 0.41H), 7.21 (m, 0.14H)**, 3.87 (s, 3H), 1.41 (s, 9H).

^{13}C{^{1}H} NMR (101 MHz, MeOD) δ 204.7, 138.2, 137.4, 129.4, 124.0, 123.7, 123.1, 113.4, 110.7, 45.0, 33.4, 29.4.

FTIR (cm^{-1}): 2967, 2927, 1616, 1494, 1435, 1362, 1331, 1077, 1012, 933, 891, 798

HRMS: (ESI) m/z calculated for $C_{14}H_{12}D_{5}NO$ [M+H]$^{+}$ 220.1624, found 220.1631.

Methyl 1-methyl-3-pivaloyl-1*H*-indole-5-carboxylate-2,4,6,7-*d*4 (2b)

Isolated yield: 11 mg, 14 %; Physical appearance: Colorless solid; m.p: 150–152 °C

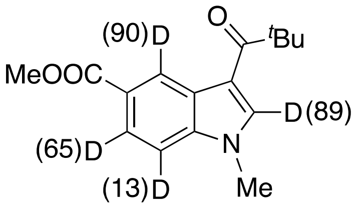

^{1}H NMR (400 MHz, $CDCl_3$) δ **9.23 (s, 0.10H), 8.03 (d, *J* = 8.6 Hz, 0.35H), 7.83 (s, 0.11H), 7.35 (t, *J* = 4.3 Hz, 0.87H)**, 3.93 (s, 3H), 3.88 (s, 3H), 1.42 (s, 9H).

^{13}C{^{1}H} NMR (101 MHz, $CDCl_3$) δ 201.8, 168.0, 138.9, 135.2, 127.7, 126.1, 124.8, 124.4, 113.6, 109.1, 51.9, 44.2, 33.6, 28.8.

FTIR (cm^{-1}): 2975, 2947, 2332, 1715, 1623, 1497, 1430, 1340, 1267, 1121, 1083, 1050, 906, 879, 739, 696

HRMS: (ESI) m/z calculated for $C_{16}H_{15}D_{4}NO_{3}$ [M+Na]$^{+}$ 300.1509, found 300.1496.

Methyl 1-methyl-3-pivaloyl-1*H*-indole-6-carboxylate-2,4,5,7-d_4 (2c)

Isolated yield: 10 mg, 13 %; Physical appearance: Yellow solid; m.p: 239–241 °C

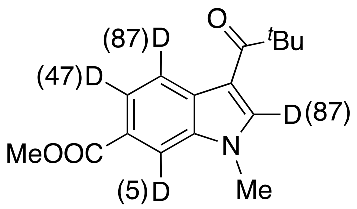

^{1}H NMR (400 MHz, $CDCl_3$) δ **8.54 (m, 0.13H), 8.10 (d, *J* = 1.3 Hz, 0.95H), 7.96 (m, 0.53H), 7.91 (s, 0.13H)**, 3.96 (s, 3H), 3.92 (s, 3H), 1.42 (s, 9H).

$^{13}C\{^1H\}$ NMR (101 MHz, $CDCl_3$) δ 201.8, 167.8, 136.6, 136.0, 131.9, 125.0, 123.3, 123.1, 112.9, 111.6, 52.1, 44.1, 33.7, 28.8.

FTIR (cm^{-1}): 2949, 2865, 1698, 1474, 1432, 1344, 1256, 1156, 1079, 989, 878, 774

Note: HRMS: (ESI) m/z not found for labelled compound.

1-(6-methoxy-1-methyl-1*H*-indol-3-yl-2,4,5,7-d_4)-2,2-dimethylpropan-1-one (2d)

Isolated yield: 30 mg, 57 %; Physical appearance: Brown solid; m.p: 114–116 °C

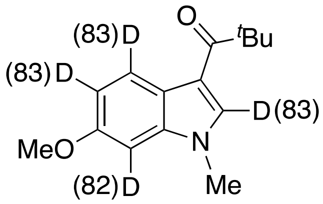

^{1}H NMR (400 MHz, $CDCl_3$) **δ 8.38 (m, 0.17H), 7.69 (s, 0.17H), 6.94 (m, 0.17H), 6.76 (s, 0.18H)**, 3.87 (s, 3H), 3.78 (s, 3H), 1.41 (s, 9H).

$^{13}C\{^1H\}$ NMR (101 MHz, $CDCl_3$) δ 202.0, 157.0, 137.2, 133.5, 124.0, 122.2, 112.6, 111.5, 93.0, 55.6, 44.0, 33.4, 29.0.

FTIR (cm^{-1}): 2982, 2942, 2878, 1631, 1605, 1490, 1435, 1340, 1211, 1074, 1041, 957, 878, 800, 667

HRMS: (ESI) m/z calculated for $C_{15}H_{15}D_4NO_2$ [M+Na]$^+$ 272.1559, found 272.1562.

1-(5-fluoro-1-methyl-1*H*-indol-3-yl-2,4,6-*d*$_3$)-2,2-dimethylpropan-1-one (2e)

Isolated yield: 33 mg, 60 %; Physical appearance: Brown solid; m.p: 91–93 °C

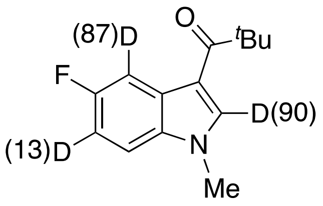

^{1}H NMR (400 MHz, $CDCl_3$) δ **8.21 (dd, *J* = 10.2, 2.5 Hz, 0.13H), 7.81 (s, 0.10H)**, 7.23 (dd, J = 8.9, 4.3 Hz, 1H), **7.03 (t, *J* = 8.9 Hz, 0.90H)**, 3.84 (s, 3H), 1.41 (s, 9H).

$^{13}C\{^1H\}$ NMR (101 MHz, $CDCl_3$) δ 201.73, 159.8 (d, *J* = 237.0 Hz), 135.4, 133.0, 128.9 (d, *J* = 11.3 Hz), 112.6 (d, *J* = 5.1 Hz), 111.6 (d, *J* = 26.3 Hz), 109.9 (d, *J* = 9.6 Hz), 108.7 (d, *J* = 25.3 Hz), 44.0, 33.7, 28.9.

FTIR (cm^{-1}): 2969, 2931, 2872, 1618, 1472, 1413, 1355, 1207, 1119, 1085, 1046, 990, 873, 784

HRMS: (ESI) m/z calculated for $C_{14}H_{13}D_3FNO$ $[M+Na]^+$ 259.1297, found 259.1285.

1-(5-chloro-1-methyl-1*H*-indol-3-yl-2,4,6-*d*$_3$)-2,2-dimethylpropan-1-one (2f)

Isolated yield: 25 mg, 43 %; Physical appearance: Brown solid; m.p: 136 – 138 °C

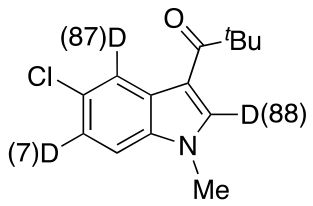

^{1}H NMR (400 MHz, MeOD) δ **8.33 (dd, *J* = 2.1, 0.6 Hz, 0.13H), 8.25 (s, 0.12H)**, 7.39 (d, J = 8.7 Hz, 1H), **7.22 (d, *J* = 8.7 Hz, 0.93H)**, 3.87 (s, 3H), 1.39 (s, 9H).

$^{13}C\{^1H\}$ NMR (101 MHz, MeOD) δ 204.3, 138.5, 136.6, 130.5, 129.2, 124.2, 123.2, 112.8, 112.1, 45.0, 33.6, 29.2.

FTIR (cm^{-1}): 2958, 2931, 2872, 1627, 1486, 1441, 1408, 1353, 1127, 1083, 1046, 1015, 948, 906, 780, 765

Note: HRMS: (ESI) m/z not found for labelled compound.

1-(5-iodo-1-methyl-1*H*-indol-3-yl-2,4,6-*d*$_3$)-2,2-dimethylpropan-1-one (2g)

Isolated yield: 53 mg, 67 %; Physical appearance: Brown colored solid; m.p: > 250 °C

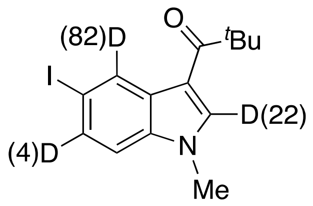

^{1}H NMR (400 MHz, CDCl$_3$) δ **8.94 (d, *J* = 1.6 Hz, 0.18H), 7.74 (s, 0.78H), 7.56 (d, *J* = 8.6 Hz, 0.96H)**, 7.09 (d, *J* = 8.6 Hz, 1H), 3.84 (s, 3H), 1.43 (s, 9H).

^{13}C{^{1}H} NMR (101 MHz, CDCl$_3$) δ 201.8, 135.6, 134.6, 132.2, 131.7, 130.4, 112.1, 111.2, 86.8, 44.1, 33.6, 28.8.

FTIR (cm^{-1}): 1627, 1516, 1444, 1362, 1077, 895, 776.

HRMS: (ESI) m/z calculated for $C_{14}H_{13}D_3INO$ [M+Na]$^+$ 367.0358, found 367.0338.

1-(6-chloro-1-methyl-1*H*-indol-3-yl-2,4,5,7-*d*$_4$)-2,2-dimethylpropan-1-one (2h)

Isolated yield: 28 mg, 49 %; Physical appearance: Brown solid; m.p: 70–72 °C

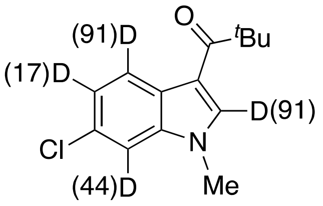

^{1}H NMR (400 MHz, CDCl$_3$) δ **8.42 (m, 0.09H), 7.76 (s, 0.09H), 7.31 (d, *J* = 1.4 Hz, 0.83H), 7.24 (m, 0.56H)**, 3.81 (s, 3H), 1.41 (s, 9H).

^{13}C{^{1}H} NMR (101 MHz, CDCl$_3$) δ 201.9, 136.9, 134.7, 129.3, 126.7, 124.4, 123.0, 112.7, 109.4, 44.1, 33.5, 28.9.

FTIR (cm^{-1}): 2949, 2909, 1638, 1598, 1483, 1450, 1415, 1335, 1077, 933, 908, 895, 838

Note: HRMS: (ESI) m/z not found for labelled compound.

1-(1,7-dimethyl-1*H*-indol-3-yl-2,4,5,6-*d*$_4$)-2,2-dimethylpropan-1-one (2i)

Isolated yield: 37 mg, 70 %; Physical appearance: Yellow solid; m.p: 141-143 °C

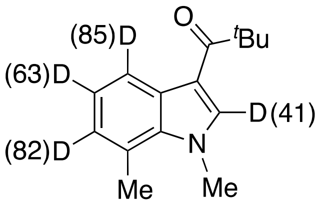

^{1}H NMR (400 MHz, $CDCl_3$) δ **8.40 (m, 0.15H), 7.68 (s, 0.59H), 7.13 (m, 0.37H), 6.98 (m, 0.18H)**, 4.09 (s, 3H), 2.74 (s, 3H), 1.41 (s, 9H).

$^{13}C\{^1H\}$ NMR (101 MHz, $CDCl_3$) δ 201.8, 136.0, 135.2, 129.3, 125.8, 122.3, 121.2, 120.9, 112.2, 44.1, 37.7, 29.0, 19.6.

FTIR (cm^{-1}): 2964, 2927, 1623, 1528, 1501, 1452, 1397, 1351, 1163, 1094, 994, 927, 875, 836, 795

HRMS: (ESI) m/z calculated for $C_{14}H_{16}D_4NO$ $[M+H]^+$ 234.1791, found 234.1783.

2,2-dimethyl-1-(1-methyl-1,6,7,8-tetrahydrocyclopenta[*g*]indol-3-yl-2,4,5-*d*$_3$)propan-1-one (2j)

Isolated yield: 30 mg, 52 %; Physical appearance: Yellow solid; m.p: 129–131 °C

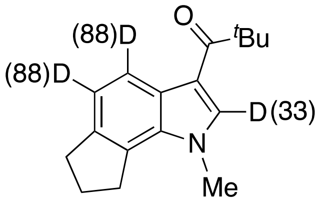

^{1}H NMR (400 MHz, $CDCl_3$) δ **8.36 (s, 0.12H), 7.69 (s, 0.67H), 7.20 (s, 0.12H)**, 4.03 (s, 3H), 3.39 (t, J = 7.4 Hz, 2H), 3.04 (t, J = 7.5 Hz, 2H), 2.21 (p, J = 7.5 Hz, 2H), 1.44 (s, 9H).

$^{13}C\{^1H\}$ NMR (101 MHz, $CDCl_3$) δ 201.9, 140.3, 134.5, 133.8, 127.3, 125.1, 121.3, 119.4, 112.8, 44.1, 35.9, 32.6, 31.3, 29.0, 25.4.

FTIR (cm^{-1}): 2936, 1612, 1521, 1435, 1397, 1335, 1127, 1074, 968, 849

HRMS: (ESI) m/z calculated for $C_{17}H_{18}D_3NO$ $[M+H]^+$ 259.1885, found 259.1880.

1-(1-ethyl-1*H*-indol-3-yl-2,4,5,6,7-*d*5)-2,2-dimethylpropan-1-one (2k)

Isolated yield: 40 mg, 76 %; Physical appearance: Brown solid; m.p: 81–83 °C

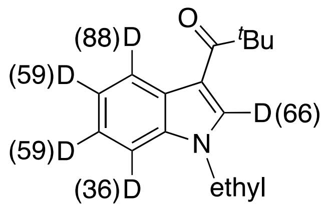

^{1}H NMR (400 MHz, $CDCl_3$) δ **8.56 (s, 0.12H), 7.88 (s, 0.34H), 7.38 (m, 0.64H), 7.33 (m, 0.83H)**, 4.25 (q, *J* = 7.3 Hz, 2H), 1.56 (t, *J* = 7.3 Hz, 3H), 1.46 (s, 9H).

$^{13}C\{^{1}H\}$ NMR (101 MHz, $CDCl_3$) δ 202.0, 135.5, 132.7, 128.4, 123.4, 123.0, 122.3, 112.9, 109.3, 44.1, 41.7, 29.0, 15.3.

FTIR (cm^{-1}): 2971, 2865, 1623, 1516, 1486, 1384, 1192, 1081, 1048, 924, 886, 798

HRMS: (ESI) m/z calculated for $C_{15}H_{14}D_5NO$ $[M+Na]^+$ 257.1673, found 257.1665.

2,2-dimethyl-1-(1-pentyl-1*H*-indol-3-yl-2,4,5,6,7-*d*5)propan-1-one (2l)

Isolated yield: 56 mg, 90 %; Physical appearance: Yellow semi-solid

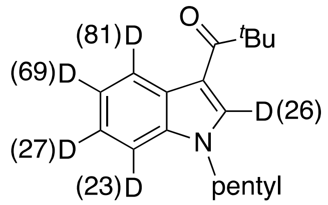

^{1}H NMR (400 MHz, MeOD) δ **8.34 (m, 0.19H), 8.21 (s, 0.74H), 7.42 (m, 0.77H), 7.24 (m, 0.73H), 7.19 (m, 0.31H)**, 4.23 (t, *J* = 7.1 Hz, 2H), 1.84 (p, *J* = 7.2 Hz, 2H), 1.40 (s, 9H), 1.30 (m, 4H), 0.87 (t, *J* = 7.1 Hz, 3H).

$^{13}C\{^{1}H\}$ NMR (101 MHz, MeOD) δ 204.7, 137.3, 136.6, 129.6, 124.0, 123.9, 123.1, 113.3, 111.0, 47.7, 45.0, 30.7, 30.0, 29.4, 23.3, 14.3.

FTIR (cm^{-1}): 2952, 2927, 2865, 1616, 1523, 1439, 1386, 1357, 1194, 1103, 1054, 1010, 893, 813

HRMS: (ESI) m/z calculated for $C_{18}H_{20}D_5NO$ $[M+H]^+$ 277.2323, found 277.2306.

1-(1-hexyl-1*H*-indol-3-yl-2,4,5,6,7-*d*5)-2,2-dimethylpropan-1-one (2m)

Isolated yield: 30 mg, 45 %; Physical appearance: Yellow semi-solid

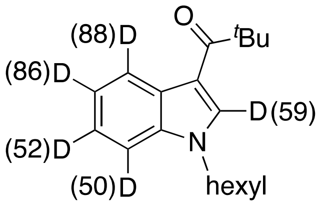

^{1}H NMR (400 MHz, MeOD) δ **8.35 (s, 0.12H), 8.21 (s, 0.41H), 7.42 (m, 0.50H), 7.23 (m, 0.48H), 7.19 (t, *J* = 3.6 Hz, 0.14H)**, 4.23 (t, *J* = 7.1 Hz, 2H), 1.84 (p, J = 7.0 Hz, 1H), 1.40 (s, 9H), 1.28 (m, 7H), 0.85 (m, 3H).

^{13}C{^{1}H} NMR (101 MHz, MeOD) δ 204.7, 137.2, 136.6, 129.6, 123.9, 123.8, 123.1, 113.3, 111.0, 47.7, 45.0, 32.4, 30.9, 29.4, 27.5, 23.6, 14.3.

FTIR (cm^{-1}): 2931, 2858, 1618, 1519, 1483, 1439, 1384, 1189, 1101, 1054, 891, 798

HRMS: (ESI) m/z calculated for $C_{19}H_{22}D_5NO$ [M+H]$^+$ 290.2406, found 290.2412.

1-(1-benzyl-1*H*-indol-3-yl-2,4-*d*2)-2,2-dimethylpropan-1-one (2n)

Isolated yield: 44 mg, 65 %; Physical appearance: Beige solid; m.p: 105–107 °C.

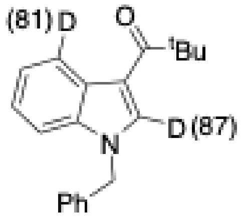

^{1}H NMR (400 MHz, $CDCl_3$) δ **8.53 (t, *J* = 3.9 Hz, 0.19H)**, **7.85 (s, 0.13H)**, 7.30 (m, 6H), 7.14 (m, 2H), 5.38 (s, 2H), 1.40 (s, 9H).

^{13}C{^{1}H} NMR (101 MHz, $CDCl_3$) δ 202.2, 136.1, 136.0, 133.9, 129.0, 128.4, 128.1, 126.8, 123.4, 123.3, 122.5, 111.3, 109.8, 50.7, 44.2, 28.9.

FTIR (cm^{-1}): 2964, 2927, 2323, 1618, 1488, 1446, 1379, 1353, 1132, 1048, 895, 717.

HRMS: (ESI) m/z calculated for $C_{20}H_{19}D_2NO$ [M+H]$^+$ 294.1821, found 294.1839

2,2-dimethyl-1-(1-phenyl-1*H*-indol-3-yl-2,4,5,6,7-*d*$_5$)propan-1-one (2ao)

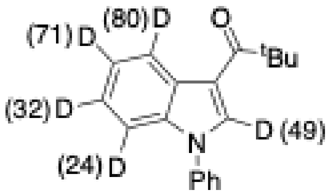

Isolated yield: 22 mg, 35 %; Physical appearance: Brown solid; m.p: 115–117 °C

^{1}H NMR (400 MHz, $CDCl_3$) δ **8.57 (d, *J* = 3.6 Hz, 0.20H), 8.01 (s, 0.51H), 7.58 (m, 2H), 7.52 (m, 2H), 7.47 (m, 1.76H), 7.34 (m, 0.29H), 7.29 (m, 0.68H), 1.45 (s, 9H).**

$^{13}C\{^1H\}$ NMR (101 MHz, $CDCl_3$) δ 202.39, 138.59, 133.14, 129.91, 127.95, 123.83, 123.73, 110.43, 110.32, 44.31, 28.91.

FTIR (cm^{-1}): 3112, 2969, 2931, 1629, 1585, 1521, 1490, 1428, 1331, 1251, 1134, 1096, 948, 922, 895, 764, 689.

HRMS: (ESI) m/z calculated for $C_{19}H_{14}D_5NO$ $[M+H]^+$ 283.1853, found 283.1851

1-phenyl-1*H*-indole-2,3,4,5,6,7-*d*$_6$ (2bo)

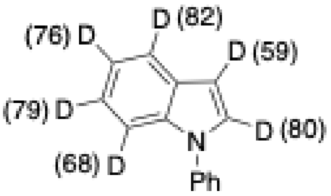

Isolated yield: 10 mg, 23 %; Physical appearance: Brown compound

^{1}H NMR (400 MHz, $CDCl_3$) δ **7.69 (d, *J* = 4.3 Hz, 0.18H), 7.57 (m, 0.32H), 7.51 (m, 4H), 7.35 (m, 1.20H), 7.22 (t, *J* = 4.2 Hz, 0.21H), 7.16 (m, 0.24H), 6.68 (s, 0.41H).**

$^{13}C\{^1H\}$ NMR (101 MHz, $CDCl_3$) δ 139.8, 135.7, 129.6, 129.2, 127.9, 126.4, 124.3, 122.1, 120.9, 120.1, 110.4, 103.3.

FTIR (cm^{-1}): 3046, 2920, 1656, 1589, 1501, 1395, 1315, 1198, 758, 696.

HRMS: (ESI) m/z calculated for $C_{14}H_5D_6N$ $[M+H]^+$ 200.1341, found 200.1348

1-(1*H*-indol-1-yl-2,3,7-d_3)-2,2-dimethylpropan-1-one (2p)

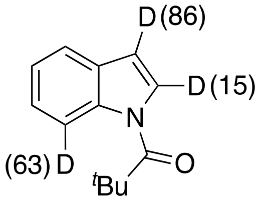

Isolated yield: 6 mg, 13 %; Physical appearance: Orange solid; m.p: 54–56 °C

^{1}H NMR (400 MHz, $CDCl_3$) δ **8.52 (dt, *J* = 8.4, 0.9 Hz, 0.37H)**, **7.74 (s, 0.85H)**, 7.56 (dd, J = 7.7, 1.3 Hz, 1H), 7.35 (dtd, J = 7.1, 4.2, 1.4 Hz, 1H), 7.26 (m, 1H), **6.62 (d, *J* = 3.7 Hz, 0.14H)**, 1.52 (s, 9H).

^{13}C{^{1}H} NMR (101 MHz, $CDCl_3$) δ 177.0, 136.6, 129.2, 125.5, 124.9, 123.5, 120.4, 117.3, 108.2, 41.2, 28.6.

FTIR (cm^{-1}): 2980, 2922, 2842, 1676, 1415, 1298, 1178, 924, 760.

HRMS: ESI m/z calculated for $C_{14}H_{15}D_6N$ $[M+H]^+$ 205.1415, found 205.1451

Methyl 1-pivaloyl-1*H*-indole-5-carboxylate-2,3,4,6,7-d_5 (2q)

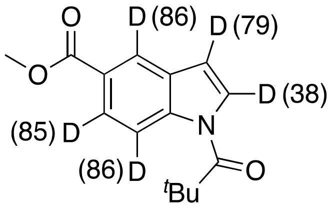

Isolated yield:11 mg, 18 %; Physical appearance: Brown solid; m.p: 94–96 °C

^{1}H NMR (400 MHz, $CDCl_3$) δ 8.54 (m, 0.14H), 8.29 (s, 0.14H), 8.04 (d, J = 4.8 Hz, 0.15H), 7.80 (d, *J* = 2.0 Hz, 0.62H), 6.69 (d, *J* = 3.6 Hz, 0.21H), 3.94 (s, 3H), 1.53 (s, 9H).

^{13}C{^{1}H} NMR (101 MHz, $CDCl_3$) δ 177.1, 167.4, 139.3, 129.0, 126.7, 126.2, 125.2, 122.7, 116.8, 108.5, 52.0, 41.3, 28.5.

FTIR (cm^{-1}): 2975, 2947, 1707, 1682, 1355, 1289, 1262, 1165, 937, 902, 798, 742, 685.

HRMS: (ESI) m/z calculated for $C_{15}H_{12}D_5NO_3$ $[M+H]^+$ 265.1595, found 265.1570

1-(5-methoxy-1*H*-indol-1-yl-2,3,4,6,7-*d*$_5$)-2,2-dimethylpropan-1-one (2r)

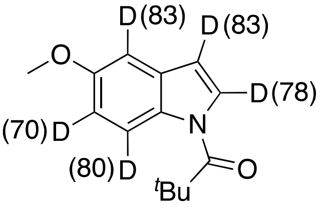

Isolated yield: 23 mg, 43 %; Physical appearance: Brown solid; m.p: 84–86 °C

^{1}H NMR (400 MHz, $CDCl_3$) δ **8.41 (t, *J* = 4.6 Hz, 0.20H)**, **7.71 (s, 0.22H)**, **7.02 (s, 0.17H)**, **6.95 (m, 0.30H)**, **6.55 (d, *J* = 3.3 Hz, 0.17H)**, 3.86 (s, 3H), 1.51 (s, 9H).

$^{13}C\{^1H\}$ NMR (101 MHz, $CDCl_3$) δ 176.6, 156.2, 131.3, 130.2, 126.1, 117.9, 113.2, 107.8, 103.2, 55.6, 41.0, 28.7.

FTIR (cm^{-1}): 2975, 2931, 1671, 1607, 1452, 1402, 1373, 1346, 1306, 1145, 1085, 1012, 898, 748.

HRMS: (ESI) m/z calculated for $C_{14}H_{12}D_5NO_2$ $[M+H]^+$ 237.1646, found 237.1638

1-(3-(acetyl-d$_3$)-1H-indol-1-yl-4,7-d$_2$)-2,2-dimethylpropan-1-one (2s)

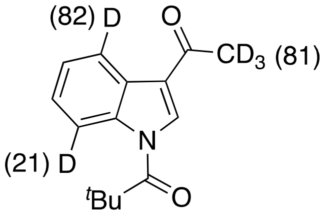

Isolated yield: 21 mg, 38 %; Physical appearance: Colorless solid; m. p: 111-113 °C

^{1}H NMR (400 MHz, MeOD) δ 8.58 (s, 1H), **8.37 (dd, *J* = 7.9, 1.6 Hz, 0.79H)**, **8.27 (m, 0.18H)**, 7.35 (m, 2H), **2.59 (m, 0.56H)**, 1.57 (s, 9H).

$^{13}C\{^1H\}$ NMR (101 MHz, MeOD) δ 196.8, 179.0, 138.7, 134.2, 127.4, 126.9, 125.7, 122.9, 121.7, 117.7, 42.7, 28.8, 27.5.

FTIR (cm^{-1}): 3163, 2986, 2924, 1704, 1649, 1541, 1421, 1320, 1258, 1176, 1138, 1049, 940, 898, 802, 759, 679

HRMS: (ESI) m/z calculated for $C_{15}H_{12}D_5NO_2$ $[M+H]^+$ 249.1646, found 249.1648

Methyl 2-acetamido-3-(1-pivaloyl-1*H*-indol-3-yl-2,7-*d*$_2$)propanoate (2t)

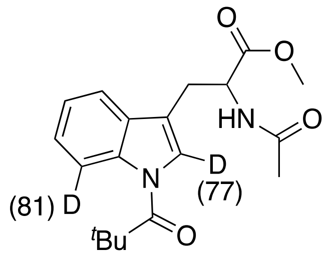

Isolated yield: 14 mg, 17 %; Physical appearance: Beige solid; m.p: 158–160 °C

^{1}H NMR (400 MHz, CDCl$_3$) δ **8.49 (dt, *J* = 8.3, 0.9 Hz, 0.19H)**, **7.54 (s, 0.23H)**, 7.46 (dd, *J* = 7.7, 1.4 Hz, 1H), 7.35 (m, 1H), 7.28 (t, *J* = 7.6 Hz, 1H), 6.05 (d, *J* = 7.9 Hz, 1H), 5.02 (dt, *J* = 7.9, 5.5 Hz, 1H), 3.72 (s, 3H), 3.33 (dd, *J* = 15.0, 5.7 Hz, 1H), 3.23 (dd, J = 15.0, 5.4 Hz, 1H), 1.98 (s, 3H), 1.50 (s, 9H).

^{13}C{^{1}H} NMR (101 MHz, CDCl$_3$) δ 176.8, 172.2, 169.7, 136.9, 129.6, 125.4, 123.8, 123.6, 118.2, 117.5, 115.7, 52.6, 52.3, 41.2, 28.6, 27.5, 23.3.

FTIR (cm^{-1}): 3329, 2975, 1751, 1689, 1649, 1539, 1300, 1172, 898, 814, 760.

HRMS: (ESI) m/z calculated for $C_{19}H_{22}D_2N_2O_4$ [M+H]$^+$ 347.1835, found 347.1808

1-pivaloyl-1*H*-indole-4-carbonitrile-3-*d* (2u)

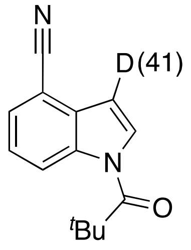

Isolated yield: 45 mg, 87 %; Physical appearance: Brown solid; m.p: 80–82 °C

^{1}H NMR (400 MHz, CDCl$_3$) δ 8.74 (dd, *J* = 8.5, 0.9 Hz, 1H), 7.92 (m, 1H), 7.60 (dd, *J* = 7.5, 0.9 Hz, 1H), 7.40 (dd, *J* = 8.5, 7.5 Hz, 1H), **6.86 (dd, *J* = 3.9, 0.8 Hz, 0.59H)**, 1.54 (s, 9H).

^{13}C{^{1}H} NMR (101 MHz, CDCl$_3$) δ 177.1, 136.5, 131.2, 128.1, 127.9, 125.0, 121.9, 117.8, 106.3, 103.4, 41.4, 28.6.

FTIR (cm^{-1}): 3170, 2960, 2916, 2224, 1687, 1474, 1417, 1399, 1300, 1196, 1156, 1079, 898, 794, 753.

HRMS: (ESI) m/z calculated for $C_{14}H_{13}DN_2O$ [M-H]$^+$ 226.1096, found 226.1064

1-(5-fluoro-1*H*-indol-1-yl-2,3,4,6,7-*d*$_5$)-2,2-dimethylpropan-1-one (2v)

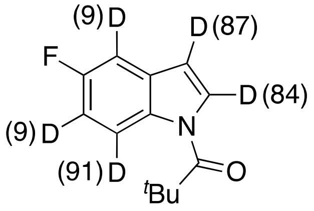

Isolated yield: 11 mg, 22 %; Physical appearance: Brown solid; m.p: 72–74 °C

^{1}H NMR (400 MHz, $CDCl_3$) δ **8.47 (dd, *J* = 9.1, 4.8 Hz, 0.09H)**, **7.78 (s, 0.16H)**, **7.20 (dd, *J* = 8.7, 2.6 Hz, 0.91H)**, **7.06 (dd, *J* = 9.3, 2.5 Hz, 0.91H)**, **6.58 (s, 0.13H)**, 1.52 (s, 9H).

$^{13}C\{^1H\}$ NMR (101 MHz, $CDCl_3$) δ 176.8, 160.7, 158.4, 133.0, 130.3, 129.3, 127.0, 118.4, 118.3 (d, *J*=9.0 Hz), 112.6 (d, *J*=24.0 Hz), 112.4, 107.7 (d, *J* = 4.0 Hz), 107.6, 106.0 (d, *J* = 24.0 Hz), 105.8, 41.1, 28.7.

FTIR (cm^{-1}): 2978, 2936, 1682, 1609, 1439, 1399, 1351, 1306, 1176, 1119, 956, 902, 859.

HRMS: (ESI) m/z calculated for $C_{13}H_9D_5FNO$ $[M+H]^+$ 225.1446, found 225.1417

1-(5-bromo-1*H*-indol-1-yl-2,3,4,6,7-*d*$_5$)-2,2-dimethylpropan-1-one (2aw)

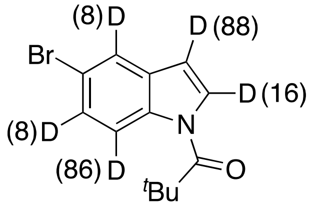

Isolated yield: 3 mg, 8 %; Physical appearance: Beige solid; m.p: 107–109 °C

^{1}H NMR **(400 MHz, $CDCl_3$) δ 8.38 (d, *J* = 8.9 Hz, 0.14H), 7.74 (s, 0.84H), 7.68 (d, *J* = 2.0 Hz, 0.92H), 7.43 (m, 0.92H), 6.56 (d, *J* = 3.8 Hz, 0.12H),** 1.51 (s, 9H).

$^{13}C\{^1H\}$ NMR (101 MHz, $CDCl_3$) δ 177.0, 135.4, 131.1, 127.8, 126.6, 123.1, 118.7, 116.8, 107.4, 41.3, 28.6.

FTIR (cm^{-1}): 3168, 2964, 1682, 1398, 1342, 1295, 1259, 1178, 1014, 905, 796, 676.

HRMS: (ESI) m/z calculated for $C_{13}H_9D_5BrNO$ $[M+H]^+$ 285.0646, found 285.0483

5-bromo-1*H*-indole-2,3,4,6,7-d_5 (2bw)

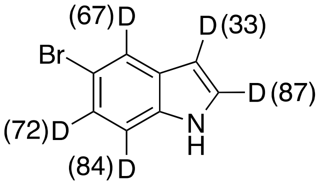

Isolated yield: 7 mg, 11 %; Physical appearance: Brown coloured solid; m.p: 77–79 °C.

^{1}H NMR **(400 MHz, MeOD) δ 7.67 (d, *J* = 1.5 Hz, 0.33H), 7.29 (m, 0.16H), 7.25 (m, 0.13H), 7.17 (m, 0.28H), 6.40 (s, 0.67H).**

^{13}C{^{1}H} NMR (101 MHz, MeOD) δ 136.1, 131.1, 127.0, 124.7, 123.5, 113.5, 112.8, 101.7.

FTIR (cm^{-1}): 3411, 2920, 1428, 1315, 1258, 1009, 872, 795, 669.

HRMS: (ESI) m/z calculated for $[M+H]^+$ not found

2,2-dimethyl-1-(5-nitro-1*H*-indol-1-yl-2,3,4,6,7-d_5)propan-1-one (2ax)

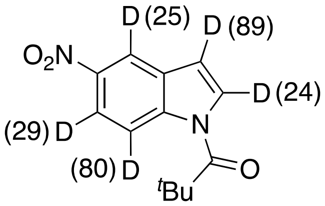

Isolated yield: 7 mg, 13 %; Physical appearance: Yellow crystalline solid; m.p: 132–134 °C.

^{1}H NMR (400 MHz, $CDCl_3$) δ **8.61 (d, *J* = 9.4 Hz, 0.20H)**, **8.48 (d, *J* = 2.3 Hz, 0.75H)**, **8.23 (q, *J* = 2.3 Hz, 0.71H)**, **7.91 (s, 0.76H)**, **6.77 (d, J = 3.8 Hz, 0.11H)**, 1.54 (s, 9H).

^{13}C{^{1}H} NMR (101 MHz, $CDCl_3$) δ 177.1, 144.1, 139.7, 129.2, 128.3, 120.1, 117.4, 116.6, 108.6, 41.4, 28.5.

FTIR (cm^{-1}): 3163, 2982, 2920, 2847, 1684, 1512, 1335, 1285, 1175, 1127, 901, 748, 673.

HRMS: ESI (positive/negative) not found.

5-nitro-1*H*-indole-2,3,4,6,7-d_5 (2bx)

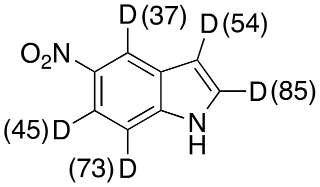

Isolated yield: 14 mg, 36 %; Physical appearance: Yellow solid; m.p: 140–142 °C.

^{1}H NMR (400 MHz, $CDCl_3$) δ **8.62 (m, 0.63H)**, 8.53 (s, 1H), **8.13 (ddd, *J* = 4.8, 3.8, 2.2 Hz, 0.55H)**, **7.44 (m, 0.27H)**, **7.38 (q, *J* = 1.9 Hz, 0.15H)**, **6.75 (d, *J* = 2.1 Hz, 0.46H)**.

^{13}C{^{1}H} NMR (101 MHz, $CDCl_3$) δ 142.0, 138.6, 127.2, 127.1, 118.0, 117.6, 110.9, 105.0.

FTIR (cm^{-1}): 3320, 1607, 1563, 1494, 1457, 1295, 1066, 892, 810, 741.

HRMS: (ESI) m/z calculated for $C_8HD_5N_2O_2$ [M-H]$^+$: 166.0670, found 166.0632

5-fluoro-1-(pyrimidin-2-yl)-1*H*-indole-2,3-d_2 (2y)

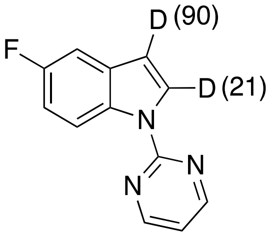

Isolated yield: 25 mg, 50 %; Physical appearance: Yellow solid; m.p: 112–114 °C.

^{1}H NMR (400 MHz, $CDCl_3$) δ 8.76 (dd, J = 9.1, 4.8 Hz, 1H), 8.69 (d, J = 4.8 Hz, 2H), **8.31 (s, 0.79H)**, 7.26 (dd, J = 9.1, 2.5 Hz, 1H), 7.06 (m, 2H), **6.65 (d, *J* = 3.7 Hz, 0.10H)**.

^{13}C{^{1}H} NMR (101 MHz, $CDCl_3$) δ 159.0 (d, *J* = 237.9 Hz), 158.1, 157.6, 132.0 (d, *J* = 10.2 Hz), 131.8, 127.2, 117.2 (d, *J* = 9.0 Hz), 116.3, 111.4 (d, *J* = 24.7 Hz), 106.6 (d, *J* = 3.8 Hz), 106.0 (d, *J* = 23.6 Hz).

FTIR (cm^{-1}): 2920, 2849, 2102, 1583, 1561, 1435, 951, 843, 793.

HRMS: (ESI) m/z calculated for $C_{12}H_6D_2FN_3$ [M+H]$^+$ 216.0901, found 216.0900

5-fluoro-1-(pyridin-2-yl)-1*H*-indole-2,3,7-*d*$_3$ (2z)

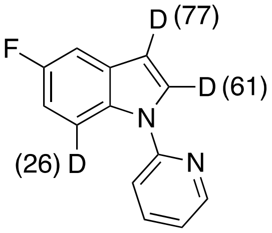

Isolated yield: 21 mg, 42 %; Physical appearance: Yellow solid; m.p: 50–52 °C.

^{1}H NMR (400 MHz, $CDCl_3$) δ 8.56 (m, 1H), **8.23 (ddt, *J* = 9.1, 4.6, 0.7 Hz, 0.74H)**, 7.82 (ddd, J = 8.2, 7.4, 2.0 Hz, 1H), **7.73 (s, 0.39H)**, 7.45 (dd, J = 8.3, 0.9 Hz, 1H), 7.29 (dd, J = 9.2, 2.6 Hz, 1H), 7.18 (ddd, J = 7.4, 4.9, 0.9 Hz, 1H), 7.03 (td, J = 9.1, 2.6 Hz, 1H), **6.67 (d, *J* = 3.3 Hz, 0.23H)**.

$^{13}C\{^1H\}$ NMR (101 MHz, $CDCl_3$) δ 158.5 (d, J = 236.9 Hz), 152.4, 148.9, 138.5, 131.7, 130.9 (d, J = 10.3 Hz), 127.1, 120.2, 114.3 (d, J = 89.7 Hz), 114.1, 111.2 (d, J = 24.9 Hz), 105.9 (d, J = 23.5 Hz), 105.4 (d, J = 4.1 Hz).

FTIR (cm^{-1}): 2098, 1687, 1587, 1441, 1134, 852, 772.

HRMS: (ESI) m/z calculated for $C_{13}H_6D_3FN_2$ [M+H]$^+$: 216.1011, found 216.1020

5-chloro-*N*-(4-ethylphenyl-2,6-*d*$_2$)pyrimidin-2-amine (2aa)

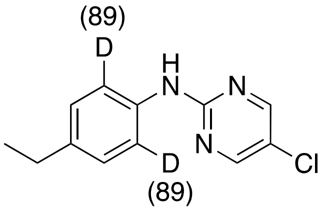

Isolated yield: 37 mg, 68 %; Physical appearance: Colorless solid; m.p: 113–115 °C.

^{1}H NMR (400 MHz, $CDCl_3$) δ 8.33 (s, 2H), **7.44 (d, *J* = 8.9 Hz, 0.22H**), 7.18 (s, 2H), 7.07 (s, 1H), 2.63 (q, *J* = 7.6 Hz, 2H), 1.23 (t, *J* = 7.6 Hz, 3H).

$^{13}C\{^1H\}$ NMR (101 MHz, $CDCl_3$) δ 158.4, 156.2, 139.4, 136.2, 128.2, 120.6, 119.9, 28.2, 15.7.

FTIR (cm^{-1}): 3263, 2962, 1600, 1576, 1516, 1428, 1130, 936, 895, 781.

HRMS: (ESI) m/z calculated for $C_{12}H_{10}D_2ClN_3$ [M+H]$^+$ 236.0918, found 236.0943

4.3.2. Characterization of deuterated carbazole products and control compounds

9*H*-carbazole-1,8-*d*$_2$ (4ba)

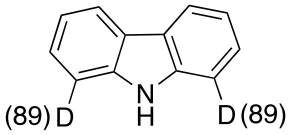

Physical appearance: Brown solid. 30 mg, 76%

^{1}H NMR (400 MHz, MeOD) δ 8.03 (dd, *J* = 7.9, 1.2 Hz, 2H), **7.43 (dt, *J* = 8.1, 1.0 Hz, 0.22H)**, 7.35 (m, 2H), 7.14 (dd, *J* = 7.8, 7.2 Hz, 2H).

^{13}C{^{1}H} NMR (101 MHz, MeOD) δ 141.42, 126.35, 124.32, 120.85, 119.60, 111.65.

FTIR (cm^{-1}): 3415, 3046, 1589, 1486, 1450, 1424, 1302, 789, 741.

HRMS: (ESI) m/z calculated for $C_{12}H_7D_2N$ [M+H]$^+$: 170.0933, found 170.0958

1-(3,6-dimethyl-9*H*-carbazol-9-yl-1,8-*d*$_2$)-2,2-dimethylpropan-1-one (4ab)

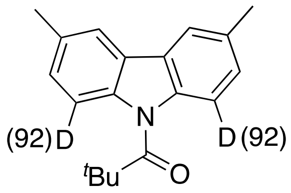

Isolated yield: 22 mg, 34 %; Physical appearance: Colorless semi-solid; m.p: 75–77 °C.

^{1}H NMR (400 MHz, $CDCl_3$) δ 7.77 (dd, *J* = 1.8, 0.9 Hz, 2H), **7.64 (d, *J* = 8.4 Hz, 0.15H)**, 7.23 (m, 2H), 2.51 (s, 6H), 1.53 (s, 9H).

^{13}C{^{1}H} NMR (101 MHz, $CDCl_3$) δ 182.9, 137.5, 131.5, 127.3, 125.2, 119.8, 114.2, 43.0, 28.4, 21.2.

FTIR (cm^{-1}): 2964, 2914, 2858, 1689, 1477, 1417, 1284, 1262, 1161, 1088, 990, 853, 799, 748.

HRMS: (ESI) m/z calculated for $C_{19}H_{19}D_2NO$ [M-H]$^+$: 280.1676, found 280.1718

3,6-dimethyl-9*H*-carbazole-1,8-*d*$_2$ (4bb)

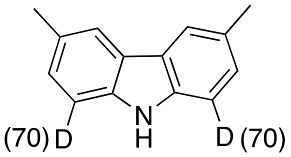

Isolated yield: 17 mg, 38 %; Physical appearance: Colorless solid; m.p: 223–225 °C

^{1}H NMR (400 MHz, $CDCl_3$) δ 7.83 (dd, *J* = 1.7, 0.9 Hz, 2H), 7.30 (d, *J* = 8.5 Hz, 0.60H), 7.21 (m, 2H), 2.52 (t, *J* = 0.7 Hz, 6H).

^{13}C{^{1}H} NMR (101 MHz, $CDCl_3$) δ 137.9, 128.4, 126.8, 123.4, 120.1, 110.2, 21.4.

FTIR (cm^{-1}): 3391, 2905, 2854, 1605, 1439, 1293, 1238, 869, 800, 757.

HRMS: (ESI) m/z calculated for $C_{14}H_{11}D_2N$ [M-H]$^+$: 196.1101, found 196.1071.

1-(3-(*tert*-butyl)-9*H*-carbazol-9-yl-1,8-d_2)-2,2-dimethylpropan-1-one (4ac)

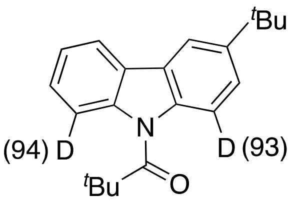

Isolated yield: 14 mg, 20%; Physical appearance: Colorless liquid

^{1}H NMR (400 MHz, MeOD) δ 8.08 (m, 2H), **7.69 (dt, *J* = 8.4, 0.9 Hz, 0.06H), 7.62 (dd, *J* = 8.8, 0.7 Hz, 0.07H)**, 7.54 (d, *J* = 2.0 Hz, 1H), 7.42 (dd, *J* = 7.2, 1.3 Hz, 1H), 7.30 (t, *J* = 7.5 Hz, 1H), 1.50 (s, 10H), 1.44 (s, 9H).

^{13}C{^{1}H} NMR (101 MHz, MeOD) δ 185.2, 146.4, 140.8, 138.3, 127.1, 126.3, 125.8, 125.2, 123.0, 120.8, 117.2, 44.5, 35.5, 32.1, 28.6.

FTIR (cm^{-1}): 2958, 2869, 1684, 1474, 1413, 1362, 1280, 1189, 1141, 1123, 1079, 1028, 878, 800, 744.

HRMS: ESI m/z calculated for $C_{14}H_5D_6N$ [M+H]$^+$ 310.2135, found 310.2125.

3-(*tert*-butyl)-9*H*-carbazole-1,8-d_2 (4bc)

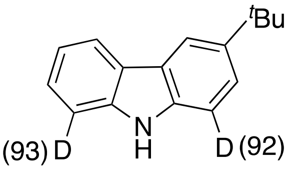

Isolated yield: 21 mg, 40 %; Physical appearance: Beige solid; m.p: 149–151 °C.

^{1}H NMR (400 MHz, MeOD) δ 8.05 (d, *J* = 2.0 Hz, 1H), 8.03 (dd, *J* = 7.8, 1.2 Hz, 1H), 7.46 (d, *J* = 2.0 Hz, 1H), **7.40 (m, 0.07H), 7.37 (d, *J* = 0.7 Hz, 0.08H)**, 7.33 (m, 1H), 7.12 (dd, *J* = 7.8, 7.2 Hz, 1H), 1.43 (s, 9H).

^{13}C{^{1}H} NMR (101 MHz, MeOD) δ 142.6, 141.8, 139.5, 126.0, 124.6, 124.0, 120.6, 119.4, 116.8, 35.4, 32.5.

FTIR (cm^{-1}): 3413, 3057, 2953, 2858, 1596, 1463, 1430, 1362, 1320, 1284, 1236, 1028, 917, 882, 805, 747.

HRMS: (ESI) m/z calculated for $C_{16}H_{15}D_2N$ [M+H]$^+$: 226.1559, found 226.1560

1-(3,6-di-*tert*-butyl-9*H*-carbazol-9-yl-1,8-*d*$_2$)-2,2-dimethylpropan-1-one (4ad)

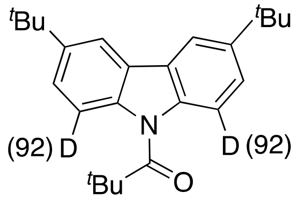

Isolated yield: 8 mg, 10 %; Physical appearance: Colorless solid; m.p: 140–142 °C.

^{1}H NMR (400 MHz, $CDCl_3$) δ 8.00 (d, *J* = 2.0 Hz, 2H), **7.73 (d, *J* = 8.8 Hz, 0.16H)**, 7.47 (m, 2H), 1.55 (s, 9H), 1.44 (s, 18H).

$^{13}C\{^1H\}$ NMR (101 MHz, $CDCl_3$) δ 182.5, 145.2, 137.5, 125.2, 123.9, 115.8, 114.4, 42.8, 34.7, 31.8, 28.3.

FTIR (cm^{-1}): 2953, 1682, 1477, 1424, 1362, 1284, 1134, 1079, 968, 872, 750.

HRMS: (ESI) m/z calculated for $C_{25}H_{31}D_2NO$ [M+H]$^+$: 366.2760, found 366.2772

3,6-di-*tert*-butyl-9*H*-carbazole-1,8-*d*$_2$ (4bd)

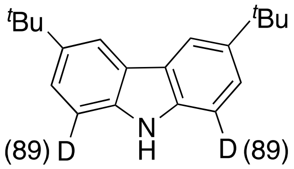

Isolated yield: 38 mg, 59 %; Physical appearance: Brown solid; m.p: 242 – 244 °C.

^{1}H NMR (400 MHz, $CDCl_3$) δ 8.07 (d, *J* = 2.0 Hz, 2H), 7.85 (s, 1H), 7.46 (d, *J* = 1.9 Hz, 2H), **7.34 (d, *J*= 8.4 Hz, 0.21H)**, 1.45 (s, 18H).

$^{13}C\{^1H\}$ NMR (101 MHz, $CDCl_3$) δ 142.2, 137.9, 123.4, 120.6, 116.1, 103.6, 34.7, 32.0.

FTIR (cm^{-1}): 3409, 2953, 2893, 2860, 1490, 1461, 1439, 1362, 1291, 878, 820, 753.

HRMS: (ESI) m/z calculated for $C_{20}H_{23}D_2N$ [M+H]$^+$: 282.2185, found 282.2204

1-(2-methoxy-9*H*-carbazol-9-yl-1,8-*d*$_2$)-2,2-dimethylpropan-1-one (4ae)

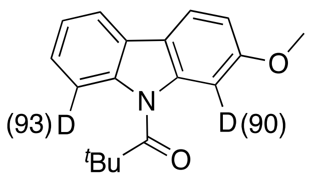

Isolated yield: 21 mg, 40 %; Physical appearance: Colorless solid; m.p: 48–50 °C

^{1}H NMR (400 MHz, CDCl$_3$) δ 7.92 (dd, J = 7.6, 1.4 Hz, 1H), 7.88 (d, J = 8.6 Hz, 1H), **7.61 (d, *J* = 8.2 Hz, 0.07H)**, 7.35 (dd, *J* = 7.3, 1.4 Hz, 1H), 7.28 (t, J = 7.4 Hz, 1H), **7.21 (q, *J* = 2.7 Hz, 0.10H)**, 6.93 (d, J = 8.6 Hz, 1H), 3.90 (s, 3H), 1.52 (s, 8H).

^{13}C{^{1}H} NMR (101 MHz, CDCl$_3$) δ 184.0, 159.2, 140.5, 139.0, 124.9, 124.8, 121.9, 120.5, 119.2, 118.4, 113.7, 110.1, 98.6, 55.7, 43.6, 28.4.

FTIR (cm^{-1}): 3000, 2964, 2918, 1687, 1616, 1594, 1572, 1479, 1424, 1397, 1309, 1280, 1247, 1143, 1088, 924, 794, 743.

HRMS: ESI m/z calculated for $C_{14}H_5D_6N$ [M+H]$^+$ 284.1541, found 284.1598

2-methoxy-9*H*-carbazole-1,3,8-*d*$_3$ (4be)

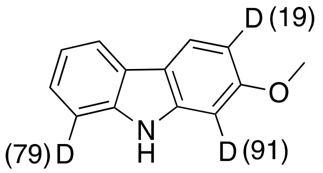

Isolated yield: 10 mg, 22 %; Physical appearance: Colorless solid; m.p: 235–237 °C

^{1}H NMR (400 MHz, CDCl$_3$) δ 7.95 (m, 3H), **7.41 – 7.36 (m, 0.21H)**, 7.34 (dt, *J* = 7.3, 2.2 Hz, 1H), 7.20 (t, *J* = 7.5 Hz, 1H), **6.91 (d, J = 2.1 Hz, 0.09H), 6.86 (d, *J* = 8.6 Hz, 0.81H)**, 3.90 (s, 3H).

^{13}C{^{1}H} NMR (101 MHz, CDCl$_3$) δ 159.0, 140.7, 139.4, 124.4, 123.5, 121.0, 119.5, 119.5, 117.3, 110.3, 108.2, 94.7, 55.6.

FTIR (cm^{-1}): 3384, 3057, 2964, 1596, 1565, 1461, 1421, 1291, 1264, 1207, 1057, 970, 816, 796, 746.

HRMS: (ESI) m/z calculated for $C_{13}H_8D_3NO$ [M+H]$^+$: 201.1099, found 201.1001

2,2-dimethyl-1-(3-phenyl-9*H*-carbazol-9-yl-1,8-*d*$_2$)propan-1-one (4af)

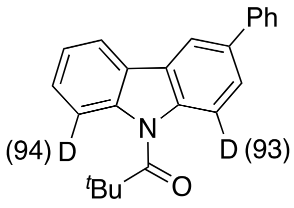

Isolated yield: 23 mg, 31 %; Physical appearance: Colorless solid; m.p: 79–81 °C.

^{1}H NMR (400 MHz, $CDCl_3$) δ 8.22 (d, *J* = 1.9 Hz, 1H), 8.07 (dd, J = 7.7, 1.3 Hz, 1H), **7.75 (d, *J* = 0.6 Hz, 0.07H)**, **7.70 (m, 3.06H)**, 7.47 (m, 3H), 7.35 (m, 2H), 1.55 (s, 8H).

^{13}C{^{1}H} NMR (101 MHz, $CDCl_3$) δ 183.6, 141.3, 139.6, 138.5, 135.4, 128.8, 127.3, 127.0, 126.4, 125.7, 125.4, 124.9, 122.0, 120.1, 118.4, 114.1, 114.0, 43.5, 28.4.

FTIR (cm^{-1}): 2962, 2869, 1687, 1596, 1461, 1408, 1276, 1189, 1136, 1101, 878, 853, 801, 749, 694, 662.

HRMS: (ESI) m/z calculated for $C_{23}H_{19}D_2NO$ $[M+H]^+$: 330.1822, found 330.1826

3-phenyl-9*H*-carbazole-1,8-*d*$_2$ (4bf)

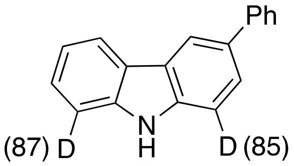

Isolated yield: 28 mg, 50 %; Physical appearance: Brown crystalline solid; m.p: 232–234 °C.

^{1}H NMR (400 MHz, MeOD) δ 8.29 (d, *J* = 1.8 Hz, 1H), 8.11 (dd, *J* = 7.8, 1.2 Hz, 1H), 7.70 (m, 2H), 7.64 (dd, J = 3.7, 1.8 Hz, 1H), **7.50 (d, *J* = 8.5 Hz, 0.15H), 7.44 (m, 2.13H),** 7.37 (m, 1H), 7.29 (m, 1H), 7.16 (dd, *J* = 7.8, 7.2 Hz, 1H).

^{13}C{^{1}H} NMR (101 MHz, MeOD) δ 143.7, 141.9, 140.9, 133.4, 129.7, 128.0, 127.2, 126.6, 125.8, 124.9, 124.4, 121.0, 119.8, 119.2, 111.9, 111.8.

FTIR (cm^{-1}): 3407, 3055, 1598, 1461, 1428, 1231, 1057, 885, 800, 753, 694.

HRMS: (ESI) m/z calculated for $C_{18}H_{11}D_2N$ $[M-H]^+$: 244.1101, found 244.1106

1-(3,6-diphenyl-9*H*-carbazol-9-yl-1,8-*d*2)-2,2-dimethylpropan-1-one (4ag)

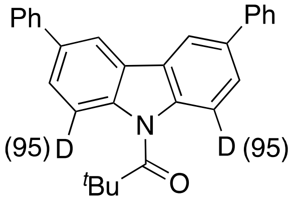

Isolated yield: 14 mg, 9 %; Physical appearance: Beige solid; m.p: 112–114 °C.

^{1}H NMR (400 MHz, $CDCl_3$) δ 8.28 (d, *J* = 1.9 Hz, 2H), **7.80 (d, *J* = 8.6 Hz, 0.10H)**, 7.70 (m, 6H), 7.49 (t, *J* = 7.7 Hz, 4H), 7.37 (m, 2H), 1.58 (s, 9.51H).

^{13}C{^{1}H} NMR (101 MHz, $CDCl_3$) δ 183.3, 141.2, 139.0, 135.5, 128.8, 127.2, 127.0, 125.8, 125.5, 118.4, 114.5, 43.4, 28.4.

FTIR (cm^{-1}): 2969, 2918, 2847, 1687, 1596, 1468, 1421, 1262, 1192, 1105, 955, 864, 757, 688.

HRMS: (ESI) m/z calculated for $C_{29}H_{23}D_2NO$ $[M+H]^+$ 406.2134, found 406.2143

3,6-diphenyl-9*H*-carbazole-1,8-*d*2 (4bg)

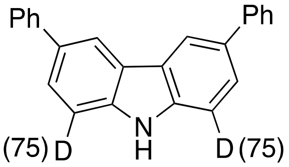

Isolated yield: 46 mg, 62 %; Physical appearance: Brown crystalline solid; m.p: 177–179 °C.

^{1}H NMR (400 MHz, MeOD) δ 8.36 (d, *J* = 1.8 Hz, 2H), 7.72 (m, 4H), 7.66 (m, 2H), **7.52 (dd, *J* = 8.5, 0.7 Hz, 0.51H)**, 7.44 (m, 4H), 7.29 (m, 2H).

^{13}C{^{1}H} NMR (101 MHz, MeOD) δ 143.6, 141.4, 133.6, 129.7, 128.1, 127.3, 126.1, 125.0, 119.4, 112.1.

FTIR (cm^{-1}): 3407, 3031, 1594, 1472, 1432, 1234, 1262, 880, 818, 754, 691.

HRMS: ESI m/z calculated for $C_{14}H_5D_6N$ $[M+H]^+$ 322.1560, found 322.1554

2,2-dimethyl-1-(2-phenyl-9*H*-carbazol-9-yl-1,8-*d*2)propan-1-one (4ah)

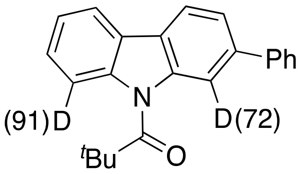

Isolated yield: 18 mg, 24 %; Physical appearance: Colorless liquid

^{1}H NMR (400 MHz, $CDCl_3$) δ 8.07 (d, *J* = 8.0 Hz, 1H), 8.04 (dd, *J* = 7.7, 1.3 Hz, 1H), **7.88 (d, *J* = 1.5 Hz, 0.28H), 7.68 (m, 2.09H)**, 7.56 (dd, *J* = 8.1, 1.2 Hz, 1H), 7.47 (m, 3H), 7.38 (m, 1H), 7.33 (t, *J* = 7.5 Hz, 1H), 1.55 (s, 9H).

^{13}C{^{1}H} NMR (101 MHz, $CDCl_3$) δ 184.0, 141.5, 139.8, 139.7, 139.5, 128.8, 127.5, 127.3, 126.2, 124.5, 123.9, 122.0, 121.5, 120.2, 120.1, 112.4, 43.7, 28.4.

FTIR (cm^{-1}): 2960, 2925, 2854, 1687, 1594, 1393, 1315, 1278, 1138, 1085, 801, 747, 695.

HRMS: (ESI) m/z calculated for $C_{23}H_{19}D_2NO$ $[M-H]^+$: 328.1676, found 328.1671

2-phenyl-9*H*-carbazole-1,8-*d*2 (4bh)

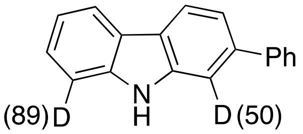

Isolated yield: 6 mg, 10 %; Physical appearance: Yellow solid; m.p: > 250 °C

^{1}H NMR (400 MHz, MeOD) δ 8.10 (d, *J* = 8.1 Hz, 1H), 8.06 (dd, *J* = 7.9, 1.2 Hz, 1H), 7.70 (m, 2H), **7.67 (dd, *J* = 1.6, 0.7 Hz, 0.50H)**, **7.44 (m, 3.11H)**, 7.34 (m, 2H), 7.16 (dd, J = 7.8, 7.2 Hz, 1H).

^{13}C{^{1}H} NMR (101 MHz, MeOD) δ 143.5, 142.1, 142.0, 140.1, 129.8, 128.3, 127.9, 126.4, 124.1, 123.6, 121.2, 120.9, 119.8, 119.2, 111.7, 109.9.

FTIR (cm^{-1}): 3407, 1421, 821, 803, 748, 695

HRMS: (ESI) m/z calculated for $C_{18}H_{11}D_2N$ $[M-H]^+$: 244.1101, found 244.1106

2-fluoro-9*H*-carbazole-1,8-d_2 (4bi)

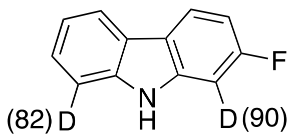

Isolated yield: 27 mg, 63 %; Physical appearance: Colorless solid; m.p: 228–230 °C.

^{1}H NMR (400 MHz, $CDCl_3$) δ 8.01 (dd, *J* = 7.8, 1.1 Hz, 1H), 7.98 (dd, *J* = 8.6, 5.4 Hz, 1H), **7.39 (m, 1.18H)**, 7.24 (t, *J* = 7.5 Hz, 1H), **7.09 (dd, *J* = 9.5, 2.3 Hz, 0.10H)**, 6.97 (dd, J = 9.6, 8.5 Hz, 1H).

$^{13}C\{^1H\}$ NMR (101 MHz, $CDCl_3$) δ 162.0 (d, *J* = 241.3 Hz), 140.0 (d, *J* = 12.4 Hz), 139.8, 125.4, 123.0, 121.2 (d, *J* = 10.3 Hz), 120.0, 119.9, 119.8, 110.6, 107.7 (d, *J* = 24.3 Hz), 97.4 (d, *J* = 26.9 Hz).

FTIR (cm^{-1}): 3409, 1620, 1598, 1472, 1421, 1333, 1295, 1262, 1198, 1012, 794, 743.

HRMS: (ESI) m/z calculated for $C_{12}H_6D_2FN$ $[M-H]^+$: 186.0693, found 186.0701

1-(2-chloro-9*H*-carbazol-9-yl-1,8-d_2)-2,2-dimethylpropan-1-one (4aj)

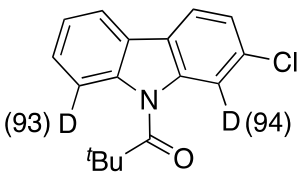

Isolated yield: 15 mg, 23%; Physical appearance: Colorless solid; m.p: 65–67 °C.

^{1}H NMR (400 MHz, $CDCl_3$) δ 7.99 (dd, *J* = 7.8, 1.3 Hz, 1H), 7.92 (d, *J* = 8.3 Hz, 1H), **7.68 (d, *J* = 1.8 Hz, 0.06H)**, **7.62 (d, J = 8.4 Hz, 0.07H)**, 7.45 (dd, *J* = 7.3, 1.3 Hz, 1H), 7.33 (t, *J* = 7.5 Hz, 1H), 7.29 (d, *J* = 8.3 Hz, 1H), 1.52 (s, 9H).

$^{13}C\{^1H\}$ NMR (101 MHz, $CDCl_3$) δ 183.7, 139.7, 139.1, 132.1, 126.4, 124.1, 123.2, 122.4, 122.1, 120.7, 120.1, 113.9, 43.7, 28.3.

FTIR (cm^{-1}): 2971, 2927, 1689, 1421, 1388, 1304, 1273, 1132, 1083, 913, 796, 742.

HRMS: (ESI) m/z calculated for $C_{17}H_{14}D_2ClNO$ $[M-H]^+$: 286.0973, found 286.0953

2-chloro-9*H*-carbazole-1,8-*d*2 (4bj)

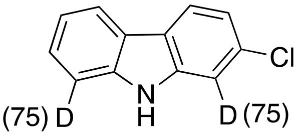

Isolated yield: 24 mg, 52 %; Physical appearance: Colorless solid; m.p: 246–248 °C.

^{1}H NMR (400 MHz, MeOD) δ 8.02 (dd, *J* = 7.8, 1.2 Hz, 1H), 7.98 (d, *J* = 8.3 Hz, 1H), **7.43 (m, 0.50H)**, 7.37 (dtd, *J* = 7.0, 4.0, 1.2 Hz, 1H), 7.16 (t, *J* = 7.5 Hz, 1H), 7.12 (d, J = 8.3 Hz, 1H).

^{13}C{^{1}H} NMR (101 MHz, MeOD) δ 141.9, 141.7, 131.9, 126.8, 123.7, 123.0, 121.9, 120.9, 120.2, 119.9, 111.8, 111.5.

FTIR (cm^{-1}): 3389, 1594, 1421, 1328, 1143, 1065, 924, 796, 746.

HRMS: (ESI) m/z calculated for $C_{12}H_6D_2ClN$ [M-H]$^+$: 202.0397, found 202.0387

3,6-dichloro-9*H*-carbazole-1,8-*d*2 (4bk)

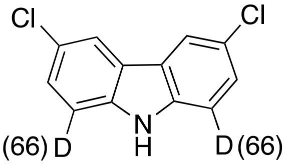

Isolated yield: 45 mg, 82 %; Physical appearance: Yellow solid; m.p: 205–207 °C.

^{1}H NMR (400 MHz, $CDCl_3$) δ 8.08 (s, 1H), 7.98 (dd, *J* = 2.1, 0.7 Hz, 2H), 7.40 (m, 2H), 7.35 (dd, *J* = 8.6, 0.7 Hz, 0.68H).

^{13}C{^{1}H} NMR (101 MHz, $CDCl_3$) δ 138.1, 126.6, 125.3, 123.6, 120.2, 111.8.

HRMS: (ESI) m/z calculated for $C_{12}H_5D_2Cl_2N$ [M-H]$^+$: 236.0008, found 236.0012

FTIR (cm^{-1}): 3400, 1466, 1419, 1278, 1068, 931, 865, 806, 751.

2-bromo-9*H*-carbazole-1,8-*d*2 (4bl)

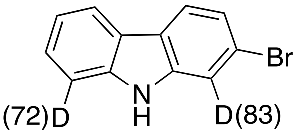

Isolated yield: 27 mg, 46 %; Physical appearance: Beige solid; m.p: > 250 °C.

^{1}H NMR (400 MHz, $CDCl_3$) δ 8.05 (s, 1H), 8.04 (dt, *J* = 8.0, 0.9 Hz, 1H), 7.92 (dd, *J* = 8.4, 0.7 Hz, 1H), **7.59 (d, *J* = 1.7 Hz, 0.17H)**, **7.44 (m, 1.28H)**, 7.34 (d, J = 8.3 Hz, 1H), 7.26 (m, 1H).

^{13}C{^{1}H} NMR (101 MHz, $CDCl_3$) δ 140.1, 139.5, 126.2, 122.8, 122.7, 122.3, 121.4, 120.3, 120.0, 119.1, 113.6, 110.7.

FTIR (cm^{-1}): 3384, 2918, 2845, 1596, 1419, 1322, 1222, 1141, 1050, 906, 798, 751.

HRMS: (ESI) m/z calculated for $C_{12}H_6D_2BrN$ [M-H]$^+$: 245.9893, found 245.9859

3-bromo-9*H*-carbazole-1,8-*d*2 (4bm)

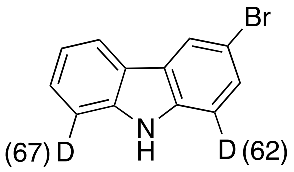

Isolated yield: 44 mg, 76 %; Physical appearance: Brown solid; m.p: 193–195 °C.

^{1}H NMR (400 MHz, $CDCl_3$) δ 8.19 (d, J = 1.9 Hz, 1H), 8.05 (s, 1H), 8.02 (dt, J = 7.9, 0.9 Hz, 1H), 7.49 (m, 1H), **7.44 (m, 1.33H)**, **7.30 (d, *J* = 8.5 Hz, 0.38H)**, 7.25 (m, 1H).

^{13}C{^{1}H} NMR (101 MHz, $CDCl_3$) δ 139.7, 137.9, 128.4, 126.4, 125.1, 123.1, 122.3, 120.5, 119.8, 112.2, 112.0, 110.7.

FTIR (cm^{-1}): 3400, 3048, 2920, 2847, 1596, 1420, 1268, 1053, 1003, 874, 805, 746.

HRMS: (ESI) m/z calculated for $C_{12}H_6D_2BrN$ [M-H]$^+$: 245.9893, found 245.9859

3,6-dibromo-9*H*-carbazole-1,8-*d*2 (4bn)

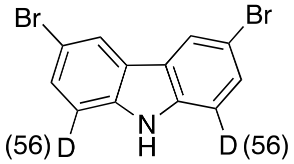

Isolated yield: 37 mg, 49 %; Physical appearance: Yellow solid; m.p: 207–209 °C.

^{1}H NMR (400 MHz, $CDCl_3$) δ 8.14 (dd, *J* = 2.0, 0.7 Hz, 2H), 8.09 (s, 1H), 7.52 (td, *J* = 4.3, 1.9 Hz, 2H), **7.31 (d, *J* = 8.6 Hz, 0.87H)**.

^{13}C{^{1}H} NMR (101 MHz, $CDCl_3$) δ 138.3, 129.2, 124.1, 123.3, 112.6, 112.2.

FTIR (cm^{-1}): 3402, 1461, 1417, 1278, 904, 864, 803, 748.

HRMS: (ESI) m/z calculated for $C_{12}H_5D_2Br_2N$ [M+H]$^+$: 323.8998, found 323.8927

1-(9*H*-carbazol-9-yl-1,8-*d*2)ethan-1-one (4ao')

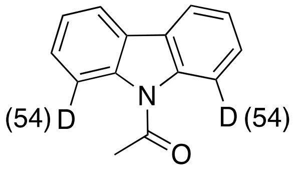

Isolated yield: 18 mg, 38 %; Physical appearance: Colorless solid; m.p: 70–72 °C.

^{1}H NMR (400 MHz, $CDCl_3$) δ **8.22 (d, *J* = 8.4 Hz, 0.92H)**, 8.00 (dd, *J* = 7.7, 1.4 Hz, 2H), 7.48 (dtd, *J* = 7.2, 4.3, 1.4 Hz, 2H), 7.39 (t, *J* = 7.5 Hz, 2H), 2.88 (s, 3H).

^{13}C{^{1}H} NMR (101 MHz, $CDCl_3$) δ 170.1, 138.6, 138.5, 127.3, 127.2, 126.4, 123.7, 119.8, 116.2, 116.0, 115.7, 29.7, 27.7.

FTIR (cm^{-1}): 2920, 2854, 1676, 1410, 1362, 1324, 1295, 1011, 737.

HRMS: (ESI) m/z calculated for $C_{14}H_9D_2NO$ [M+H]$^+$: 212.1039, found 212.0947

9*H*-carbazole-1,3,6,8-*d*4 (4bo')

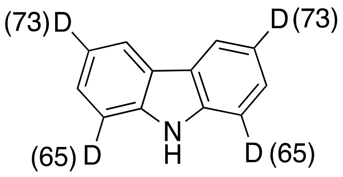

Isolated yield: 3 mg, 8 %; Physical appearance: Colorless solid; m.p: 220-222 °C.

^{1}H NMR (400 MHz, MeOD) δ 8.03 (m, 2H), **7.43 (dd, *J* = 8.2, 0.8 Hz, 0.70H)**, 7.35 (td, *J* = 3.4, 1.8 Hz, 2H), **7.14 (m, 0.54H)**.

^{13}C{^{1}H} NMR (101 MHz, MeOD) δ 141.4, 126.4, 124.3, 120.8, 119.6, 111.6.

FTIR (cm^{-1}): 3418, 2925, 2541, 1729, 1587, 1416, 1269, 901, 824, 749.

HRMS: (ESI) m/z calculated for $C_{12}H_5D_4N$ [M+H]$^+$: 172.1059, found 172.1059

1-(5*H*-dibenzo[*b,f*]azepin-5-yl-4,6-*d*2)-2,2-dimethylpropan-1-one (4ap)

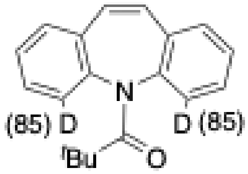

Isolated yield: 30 mg, 47 %; Physical appearance: Yellow solid; m.p: 92 – 94 °C.

^{1}H NMR (400 MHz, $CDCl_3$) δ **7.46 (dd, *J* = 7.9, 1.5 Hz, 0.31H)**, 7.42 (ddd, *J* = 7.2, 3.5, 1.7 Hz, 2H), 7.37 (dd, *J* = 7.8, 1.8 Hz, 2H), 7.30 (m, 2H), 7.00 (s, 2H), 0.99 (s, 9H).

^{13}C{^{1}H} NMR (101 MHz, $CDCl_3$) δ 177.9, 141.0, 134.7, 130.3, 128.8, 128.7, 128.7, 127.3, 40.5, 28.8.

FTIR (cm^{-1}): 2964, 2931, 2869, 1642, 1474, 1432, 1395, 1304, 1218, 1154, 966, 885, 828, 765, 726.

HRMS: (ESI) m/z calculated for $C_{19}H_{17}D_2NO$ [M+H]$^+$: 280.1665, found 280.1616

5*H*-dibenzo[*b,f*]azepine-2,4,6,8-*d*4 (4bp)

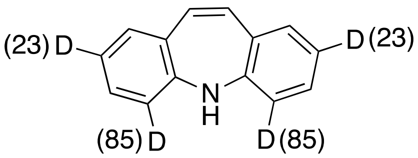

Isolated yield: 5 mg, 10%; Physical appearance: Yellow solid; m.p: 194–196 °C.

^{1}H NMR (400 MHz, $CDCl_3$) δ 7.02 (dp, *J* = 5.5, 2.1 Hz, 2H), 6.86 (m, 2H), **6.82 (m, 1.54H)**, **6.49 (dd, *J* = 7.8, 1.0 Hz, 0.29H)**, 6.31 (s, 2H), 4.93 (s, 0.95H).

^{13}C{^{1}H} NMR (101 MHz, $CDCl_3$) δ 148.3, 132.1, 130.5, 129.7, 129.4, 123.0, 119.3.

FTIR (cm^{-1}): 3358, 3046, 1572, 1426, 1069, 912, 821, 747, 710, 675.

HRMS: (ESI) m/z calculated for $C_{14}H_7D_4N$ [M+H]$^+$: 198.1215, found 198.1221

9*H*-carbazol-1,2,3-*d*$_3$-4-ol (4bq')

Isolated yield: 3 mg, 19 %; Physical appearance: Brown solid; m.p: 175–177 °C.

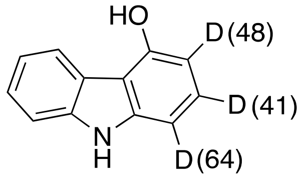

^{1}H NMR (400 MHz, $CDCl_3$) δ 8.27 (dd, *J* = 7.8, 1.0 Hz, 1H), 8.05 (s, 1H), 7.40 (m, 2H), **7.25 (m, 1.59H)**, **7.02 (d, *J* = 8.1 Hz, 0.36H)**, **6.58 (d, *J* = 7.8 Hz, 0.52H)**, 5.32 (s, 0.76H).

$^{13}C\{^1H\}$ NMR (101 MHz, $CDCl_3$) δ 151.9, 141.3, 138.9, 126.5, 125.2, 122.8, 122.3, 119.7, 111.8, 110.0, 105.2, 103.4.

FTIR (cm^{-1}): 3395, 3183, 1629, 1605, 1576, 1483, 1441, 1329, 1295, 1256, 1189, 1037, 926, 805, 784, 750.

HRMS: (ESI) m/z calculated for $C_{12}H_6D_3NO$ $[M+H]^+$: 187.0945, found 187.0823

1-((9*H*-carbazol-4-yl-1,3-*d*$_2$)oxy)-3-(*N*-(2-(2-methoxyphenoxy)ethyl)pivalamido)propan-2-yl pivalate (4br)

Isolated yield: 17 mg, 35 %; Physical appearance: Colorless solid.

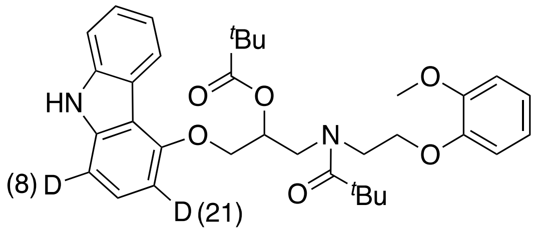

^{1}H NMR (400 MHz, $CDCl_3$) δ 8.29 (d, *J* = 7.7 Hz, 1H), 8.17 (s, 1H), 7.38 (dt, *J* = 8.1, 1.1 Hz, 1H), 7.34 (ddd, *J* = 8.0, 6.8, 1.2 Hz, 1H), 7.30 (m, 1H), 7.15 (ddd, *J* = 8.0, 6.8, 1.4 Hz, 1H), **7.05 (d, *J* = 8.0 Hz, 0.92H)**, 6.92 (ddd, *J* = 7.8, 5.4, 3.8 Hz, 1H), 6.84 (m, 3H), **6.61 (d, *J* = 8.0 Hz, 0.79H)**, 5.78 (qd, *J* = 5.8, 3.1 Hz, 1H), 4.36 (m, 2H), 4.18 (td, *J* = 5.9, 1.6 Hz, 2H), 4.04 (m, 5H), 3.75 (s, 3H), 1.33 (s, 9H), 1.18 (s, 9H).

$^{13}C\{^1H\}$ NMR (101 MHz, $CDCl_3$) δ 178.4, 178.2, 155.0, 149.8, 148.0, 141.0, 138.7, 126.6, 125.0, 123.3, 122.5, 121.9, 120.9, 119.6, 114.2, 112.7, 112.0, 109.9, 104.0, 100.9, 70.2, 67.8, 67.5, 55.7, 48.7, 39.2, 38.8, 28.6, 27.1.

FTIR (cm^{-1}): 2971, 2927, 2876, 1722, 1605, 1505, 1455, 1329, 1251, 1214, 1147, 1119, 1096, 1019, 904, 780, 722.

HRMS: (ESI) m/z calculated for $C_{34}H_{40}D_2N_2O_6$ $[M+H]^+$: 577.3241, found 577.3214

1-(*N*-(2-(2-methoxyphenoxy)ethyl)pivalamido)-3-((9-pivaloyl-9*H*-carbazol-4-yl-1,8-*d*$_2$)oxy)propan-2-yl pivalate (4as)

Isolated yield: 67 mg, 67 %; Physical appearance: Yellow solid; m.p: 58-60 °C.

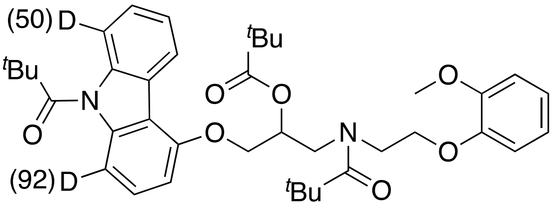

^{1}H NMR (400 MHz, MeOD) δ 8.27 (dd, *J* = 7.8, 1.3 Hz, 1H), **7.53 (dt, J = 8.4, 0.9 Hz, 0.50H)**, 7.37 (m, 2H), **7.20 (m, 0.08H)**, 6.86 (m, 5H), 5.86 (qd, *J* = 5.5, 3.6 Hz, 1H), 4.38 (t, *J* = 3.8 Hz, 2H), 4.20 (p, *J* = 5.1 Hz, 2H), 4.01 (dd, *J* = 13.1, 7.1 Hz, 3H), 3.68 (s, 3H), 1.45 (s, 6H), 1.32 (s, 10H), 1.15 (s, 9H).

^{13}C{^{1}H} NMR (101 MHz, MeOD) δ 186.4, 180.7, 179.6, 156.2, 151.3, 149.4, 141.7, 139.8, 128.3, 126.6, 124.6, 124.6, 123.1, 122.8, 122.0, 115.6, 114.7, 113.4, 113.3, 107.2, 104.5, 71.4, 69.1, 56.2, 49.5, 45.2, 40.4, 39.9, 29.1, 28.6, 27.6.

FTIR (cm^{-1}): 2971, 2931, 2876, 1722, 1629, 1578, 1501, 1450, 1388, 1251, 1143, 1105, 1021, 903, 806, 743.

HRMS: (ESI) m/z calculated for $C_{39}H_{48}D_2N_2O_7$: 660.3743, found 660.3737

1-((9*H*-carbazol-4-yl-1,3,8-*d*$_3$)oxy)-3-(*N*-(2-(2-methoxyphenoxy)ethyl)pivalamido)propan-2-yl pivalate (4bs)

Isolated yield: 5 mg, 5 %; Physical appearance: Brown solid; m.p: 65–67 °C.

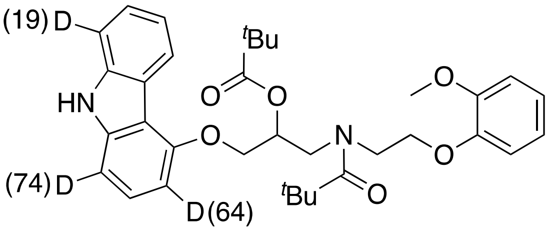

^{1}H NMR (400 MHz, MeOD) δ 8.22 (dd, *J* = 7.9, 1.2 Hz, 1H), **7.39 (dt, *J* = 8.2, 0.9 Hz, 0.81H)**, 7.28 (m, 2H), **7.05 (m, 0.26H)**, 6.90 (m, 3H), 6.83 (ddd, *J* = 7.4, 5.9, 3.3 Hz, 1H), **6.64 (d, *J* = 8.0 Hz, 0.36H)**, 5.86 (ddd, *J* = 12.9, 5.8, 3.6 Hz, 1H), 4.35 (m, 2H), 4.19 (m, 2H), 4.01 (m, 4H), 3.70 (s, 3H), 1.32 (s, 9H), 1.16 (s, 9H).
^{13}C{^{1}H} NMR (101 MHz, MeOD) δ 180.6, 179.6, 156.2, 151.2, 149.4, 142.8, 140.7, 127.2, 125.6, 124.1, 123.3, 123.1, 122.0, 119.7, 115.5, 113.4, 113.3, 111.0, 105.1, 101.1, 71.5, 69.0, 68.7, 56.2, 49.5, 40.4, 39.9, 29.1, 27.6.
FTIR (cm^{-1}): 2925, 1722, 1605, 1501, 1452, 1340, 1251, 1218, 1147, 1121, 1027, 740.
HRMS: (ESI) m/z calculated for $C_{34}H_{39}D_3N_2O_6$ [M+H]$^+$: 578.3304, found 578.3285

2-methoxy-9*H*-carbazole-1,3-*d*₂ (4be')

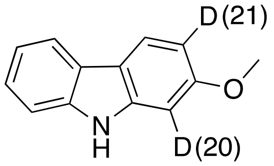

Isolated yield: 20 mg, 45 %; Physical appearance: Colorless solid; m.p: 223–225 °C.

^{1}H NMR (400 MHz, MeOD) δ 7.91 (dt, J = 7.8, 1.0 Hz, 1H), 7.88 (m, 1H), 7.37 (dt, J = 8.0, 0.9 Hz, 1H), 7.26 (ddd, J = 8.2, 7.1, 1.2 Hz, 1H), 7.10 (ddd, J = 8.1, 7.1, 1.1 Hz, 1H), **6.96 (d, J = 2.1 Hz, 0.80H), 6.77 (dd, J = 8.5, 2.2 Hz, 0.79H)**, 3.87 (s, 3H).

$^{13}C\{^1H\}$ NMR (101 MHz, MeOD) δ 160.4, 142.8, 141.5, 125.2, 124.6, 121.6, 120.0, 119.7, 118.2, 111.3, 108.8, 95.4, 55.9.

HRMS: (ESI) m/z calculated for $C_{13}H_9D_2NO$ $[M+H]^+$: 200.1039, found 200.1032

FTIR (cm^{-1}): 3382, 3059, 2920, 2843, 1624, 1599, 1457, 1327, 1298, 1218, 1187, 1155, 1028, 812, 742, 724.

5. Scaleup experiment (1 mmol)

To a reaction tube was added the indole (**1a**) (0.25 g, 1.16 mmol), $[Cp^*IrCl_2]_2$ (5 mol%, 46 mg), and $AgNTf_2$ (20 mol%, 90 mg). The reaction tube was then sealed with a Teflon lined screw cap and evacuated and purged with N_2 (3 cycles). Then under N_2 was added 1,2-DCE (5 mL) and D_2O (418 µL, 20 equiv.) using a syringe. The reaction tube was sealed with parafilm and allowed to stir at 100 °C for 20 h. The TLC for the reaction mixture was checked in petroleum ether/acetone (9:1 or 4:1). The reaction mixture was then filtered through a pad of celite in DCM (5 mL), methanol (5 mL) and acetone (5 mL). The crude material was concentrated under reduced pressure and was purified by column chromatography using petroleum ether/acetone solvent combination (9:1) to get pure product **2a** (0.248 g, 97%). The product was characterized by ^{1}H NMR and found to be identical to that obtained in the small-scale experiment. The site and degree of deuterium incorporation were determined by comparing the integrals of characteristic $^{13}C\{^1H\}$ NMR peaks with those of the starting materials.

6. Control experiment

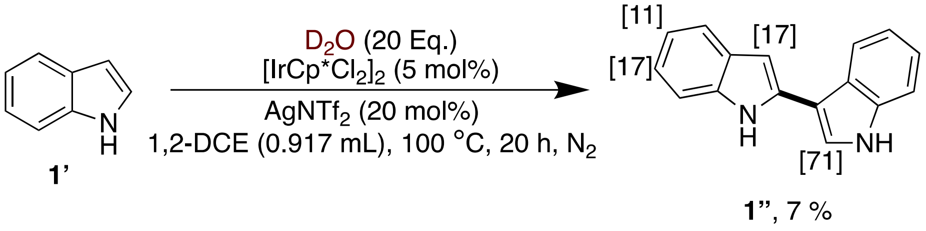

To a reaction tube was added the indole (**1'**) (0.23 mmol), $[Cp^*IrCl_2]_2$ (5 mol%, 9.1 mg), and $AgNTf_2$ (20 mol%, 18 mg). The reaction tube was then sealed with a Teflon lined screw cap and evacuated and purged with N_2 (3 cycles). Then under N_2 was added 1,2-DCE (917 µL) and D_2O (83 µL, 20 equiv.) using a syringe. The reaction tube was sealed with parafilm and allowed to stir at 100 °C for 20 h. The TLC for the reaction mixture was checked in petroleum ether/acetone (9:1 or 4:1) mostly and for certain cases in petroleum ether/ethyl acetate (9:1 or 4:1). The reaction mixture was then filtered through a pad of celite in DCM (5 mL), methanol (5 mL) and acetone (5 mL). The crude material was concentrated under reduced pressure and was purified by preparative TLC. The preparative TLC were run in petroleum ether/acetone or petroleum ether/ethyl acetate solvent combination (9:1 or 4:1). The collected material was then sonicated in 5mL each of DCM and methanol, followed by filtration through a sintered Buchner glass funnel. Finally, the residue was concentrated under reduced pressure and dried under high vacuum. The site and degree of deuterium incorporation was determined by comparing the integrals of the characteristic $^{13}C\{^1H\}$ NMR peaks with those of the starting substrates. The product is a known compound (**1''**). The spectroscopic data are consistent with those reported in the literature.[16]

^1H NMR (400 MHz, MeOD) δ 8.00 (m, 1H), **7.66 (s, 0.29H)**, 7.51 (m, 1H), 7.43 (m, 1H), 7.35 (m, 1H), 7.19 (m, 1H), 7.15 (m, 1H), **7.04 (m, 0.91H)**, **6.97 (m, 0.89H)**, **6.72 (d, *J* = 0.9 Hz, 0.83H)**.

$^{13}C\{^1H\}$ NMR (101 MHz, MeOD) δ 138.5, 137.9, 135.6, 131.1, 126.6, 123.2, 123.0, 121.5, 120.8, 120.8, 120.3, 120.0, 112.6, 111.3, 110.5, 98.4.

7. References

(1) Kathiravan, S.; Anaspure, P.; Zhang, T.; Nicholls, I. A. Tandem Iridium-Catalyzed Decarbonylative C-H Activation of Indole: Sacrificial Electron-Rich Ketone-Assisted Bis-arylsulfenylation. *Org. Lett.* **2021**, *23*, 3331-3336.

(2) Kuznetcova, I.; Bacher, F.; Vegh, D.; Chuang, H. Y.; Arion, V. B. Ready access to 7,8-dihydroindolo[2,3-*d*][1]benzazepine-6[5*H*]-one scaffold and analogues via early stage Fischer ring-closure reaction. *Beilstein J. Org. Chem.* **2022**, *18*, 143-151.

(3) Chen, X.; Zheng, G.; Li, Y.; Song, G.; Li, X. Rhodium-Catalyzed Site-Selective Coupling of Indoles with Diazo Esters: C4-Alkylation versus C2-Annulation. *Org. Lett.,* **2017**, *19*, 6184-6187.

(4) Stuart, D. R.; Villemure, E.; Fagnou, K. Elements of regiocontrol in palladium-catalyzed oxidative arene cross-coupling. *J. Am. Chem. Soc.* **2007**, *129*, 12072-12073.

(5) Kathiravan, S.; Nicholls, I. A. Rhodium(III) catalyzed, redox neutral C(sp^2)-H alkenylation using pivalimide as a directing group with internal alkynes. *Tetrahedron Lett.* **2017**, *58*, 1-4.

(6) Kathiravan, S.; Zhang, T.; Nicholls, I. A. Iridium catalyzed C2 site-selective methylation of indoles using pivaloyl directing group through weak chelation-assistance. *RSC Adv.* **2023**, *13*, 11291-11295.

(7) Lv, J.; Chen, X.; Xue, X.-S.; Zhao, B.; Liang, Y.; Wang, M.; Jin, L.; Yuan, Y.; Han, Y.; Zhao, Y.; Lu, Y.; Zhao, J.; Sun, W.-Y.; Houk, K. N.; Shi, Z. Metal-free directed sp2 C-H borylation. *Nature* **2019**, *575*, 336-340.

(8) Sheng, Y.; Zhou, J.; Gao, Y.; Duan, B.; Wang, Y.; Samorodov, A.; Liang, G.; Zhao, Q.; Song, Z. Ruthenium(II)-catalyzed direct C7-selective amidation of indoles with dioxazolones at room temperature. *J. Org. Chem.* **2021**, *86*, 2827-2839.

(9) Watanabe, K.; Moriyama, K. Copper-catalyzed indole-selective C-N coupling reaction of indolyl(2-alkoxy-phenyl)iodonium imides: Effect of substituent on iodoarene as dummy ligand. *J. Org. Chem.* **2018**, *83*, 14827-14833.

(10) Kim, Y.; Park, J.; Chang, S. A direct access to 7-aminoindoles via iridium-catalyzed mild C-H amidation of *N*-pivaloylindoles with organic azides. *Org. Lett.* **2016**, *18*, 1892-1895.

(11) Zhang, Y.; Hu, S.; Li, Y.; Wang, Y.; Yu, T.; Chen, Q.; Wang, J.; Liu, H. Macrocyclization of maleimide-decorated peptides via late-stage Rh(III)-catalyzed Trp(C7) alkenylation. *Org. Lett.* **2023**, *25*, 2546-2460.

(12) Xu, L.; Zhagn, C.; He, Y.; Tan, L.; Ma, D. Rhodium-catalyzed regioselective C7 functionalization of N-pivaloylindoles. *Angew. Chem. Int. Ed.* **2016**, *55*, 321-325.
(13) Wang, X.; Xun, X.; Song, H.; Liu, Y.; Wang, Q. Palladium metallaphotoredox-catalyzed 2-arylation of indole derivatives. *Org. Lett.* **2022**, *24*, 4580-4585.
(14) Zhang, J.; Zou, M.; Tian, Q.; Sun, Z.; Chu, W. N-cyano-2,2'-biphenyldicarboimide as a cyanation reagent for Co(III)-catalyzed C-H cyanation of indoles in ionic liquids. *Org. Lett.* **2023**, *25*, 1436-1440.
(15) Wei, Y.; He, X.; Long, Z.; Le, Y.; Liu, L.; Yan, L. Discovery of noncovalent diaminopyrimidine-based inhibitors for glioblastoma via a dual FAK/DNA targeting strategy. *Eur. J. Med. Chem.* **2025**, *286*, 117288.
(16) Huang, X.; Yang, Y.; He, Q.; Liu, C.; Yuan, M.; Jin, Y. Copper-catalyzed aerobic cross-dehydrogenation coupling of indoles for synthesis of 2,3'bisindoles. *Chem. Asian J.* **2024**, *19*, e-2024010015.
