# Supplementary material for: Iridium/Silver-Catalyzed H/D Exchange for Perdeuteration of Indoles and Site-Selective Deuteration of Carbazoles: Application in Late-Stage Functionalization
Source: J Org Chem. 2025 Jul 10;90(33):11791–801. doi: 10.1021/acs.joc.5c00702 (PMC12381928; doi:10.1021/acs.joc.5c00702)
Supplement: Supplementary file 2 [file jo5c00702_si_002.pdf]

Iridium/Silver-Catalyzed H/D Exchange for Perdeuteration of Indoles and Site-Selective Deuteration Carbazoles: Application in Late-Stage Functionalization

Prakriti Dhillon, Subban Kathiravan, Jesper G. Wiklander, and Ian A. Nicholls*

Bioorganic & Biophysical Chemistry Laboratory, Linnaeus University Centre for Biomaterials Chemistry, Department of Chemistry & Biomedical Sciences, Linnaeus University, Kalmar SE-39182, Sweden.

Email: suppan.kathiravan@lnu.se

Table of contents

1a ... S2
2a ... S8
1b ... S10
2b ... S16
1c ... S18
2c ... S24
1d ... S26
2d ... S32
1e ... S34
2e ... S40
1f ... S42
2f ... S48
1g ... S50
2g ... S56
1h ... S58
2h ... S64
1i ... S66
2i ... S72
1j ... S74
2j ... S80
1k ... S82
2k ... S88
1l ... S90
2l ... S96
1m ... S98
2m ... S104
1n ... S106
2n ... S112
1o ... S114
2ao ... S120
1o' ... S122
2bo ... S128
1p ... S130
2p ... S136
1q ... S138
2q ... S144
1r ... S146
2r ... S152
1s ... S154
2s ... S160
1t ... S162
2t ... S167
1u ... S169
2u ... S175
1v ... S177
2v ... S183
1w ... S185
2aw ... S191
1w' ... S193
2bw ... S199
1x ... S201
2ax ... S207
1x' ... S209
2bx ... S215
1y ... S217
2y ... S222
1z ... S224
2z ... S229
1aa ... S231
2aa ... S237
1'' ... S239
3a ... S246
4ba ... S252
3a' ... S254
4ba' ... S260
3b ... S262
4ab ... S268
3b' ... S270
4bb ... S276
3c ... S278
4ac ... S284
3c' ... S286
4bc ... S292
3d ... S294
4ad ... S300
3d' ... S302
4bd ... S308
3e ... S310
4ae ... S316
3e' ... S318
4be ... S324
3e' ... S326
4be' ... S332
3f ... S334
4af ... S340
3f' ... S342
4bf ... S348
3g ... S350
4ag ... S356
3g' ... S358
4bg ... S364
3h ... S366
4ah ... S372
3h' ... S374
4bh ... S380
3i ... S382
3i' ... S388
4bi ... S394
3j ... S396
4aj ... S402
3j' ... S404
4bj ... S410
3k ... S412
3k' ... S418
4bk ... S424
3l ... S426
3l' ... S432
4bl ... S438
3m ... S440
3m' ... S446
4bm ... S452
3n ... S454
3n' ... S460
4bn ... S466
3o' ... S468
4ao' ... S474
Carbazole = **3a'** ... S476
4bo' ... S482
3p ... S484
4ap ... S490
3p' ... S492
4bp ... S498
3q' ... S500
4bq' ... S506
3r ... S512
4ar ... S518
3s ... S520
4as ... S526
4bs ... S528

1a

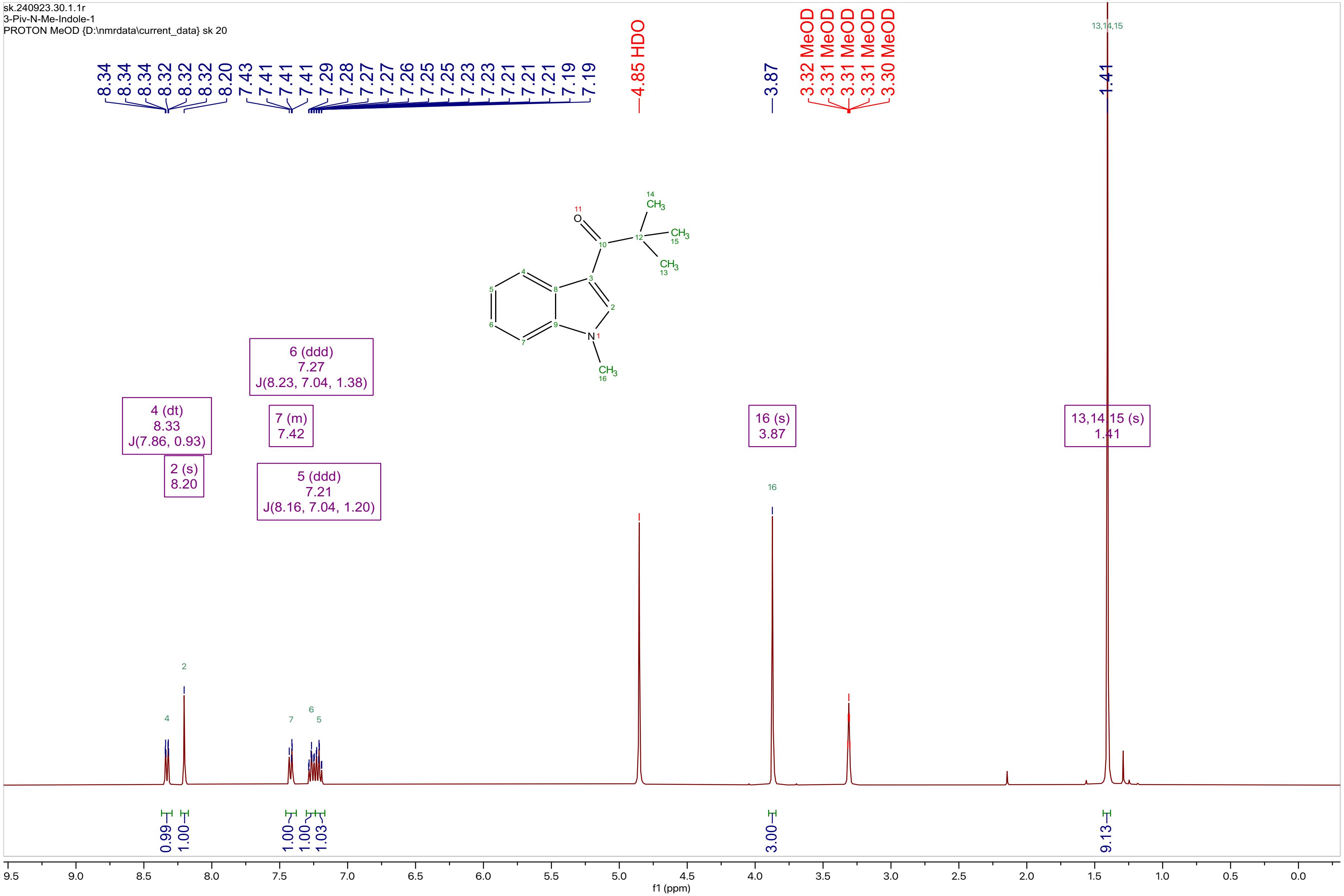

^{1}H NMR (400 MHz, MeOD) of 1a

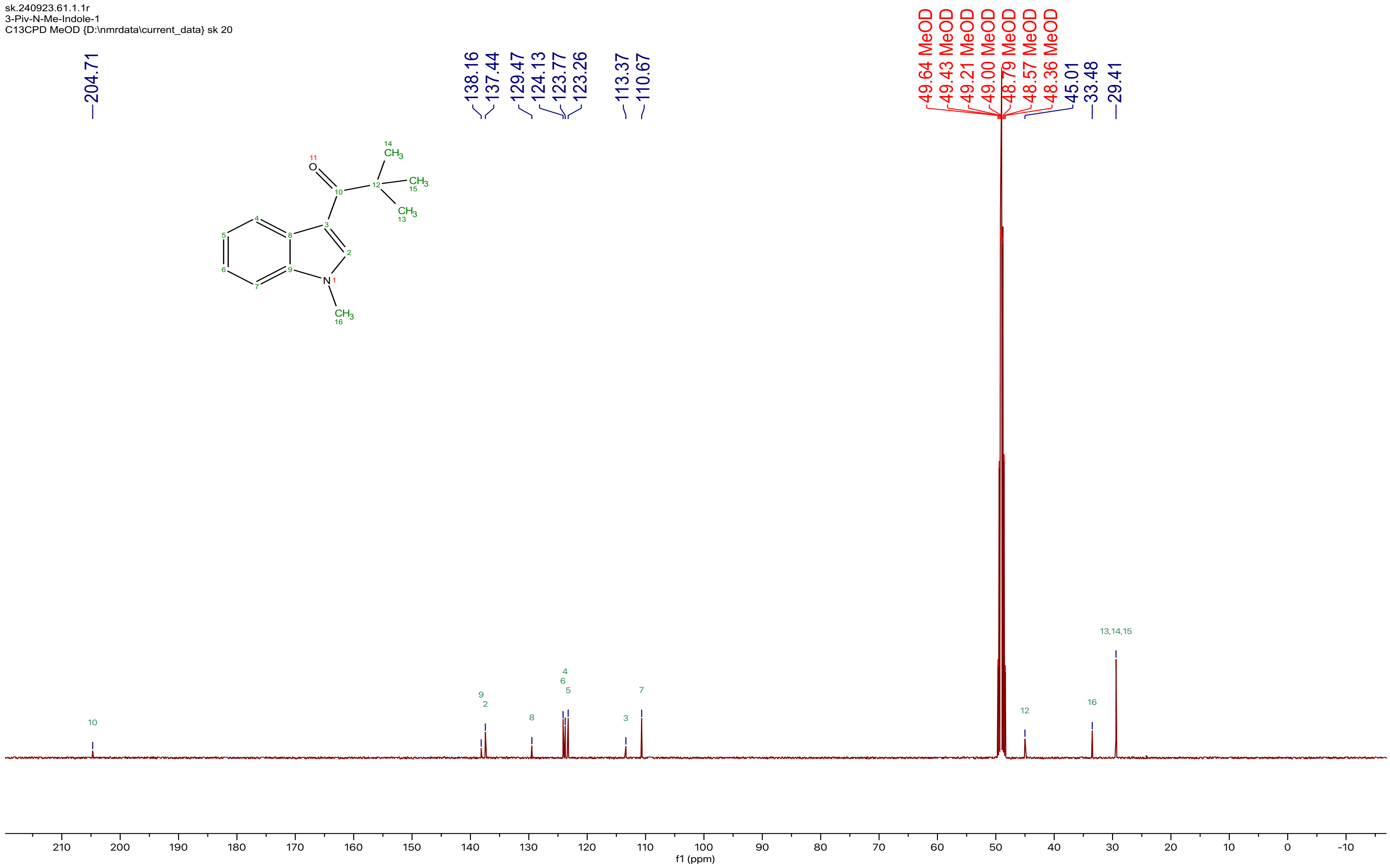

$^{13}C\{^1H\}$ NMR (101 MHz, MeOD) of 1a

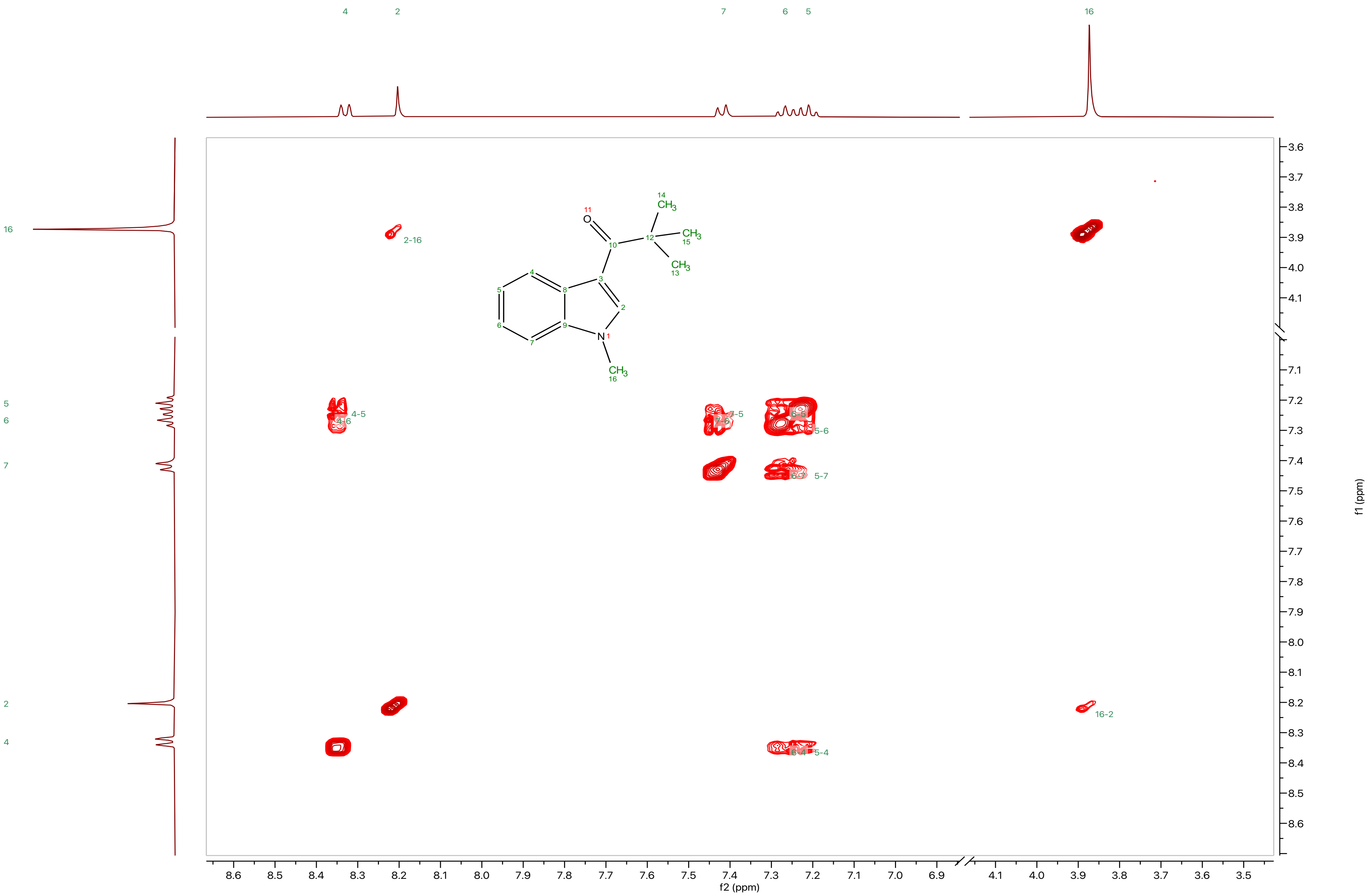

^{1}H-^{1}H COSY (400 MHz, MeOD) of 1a

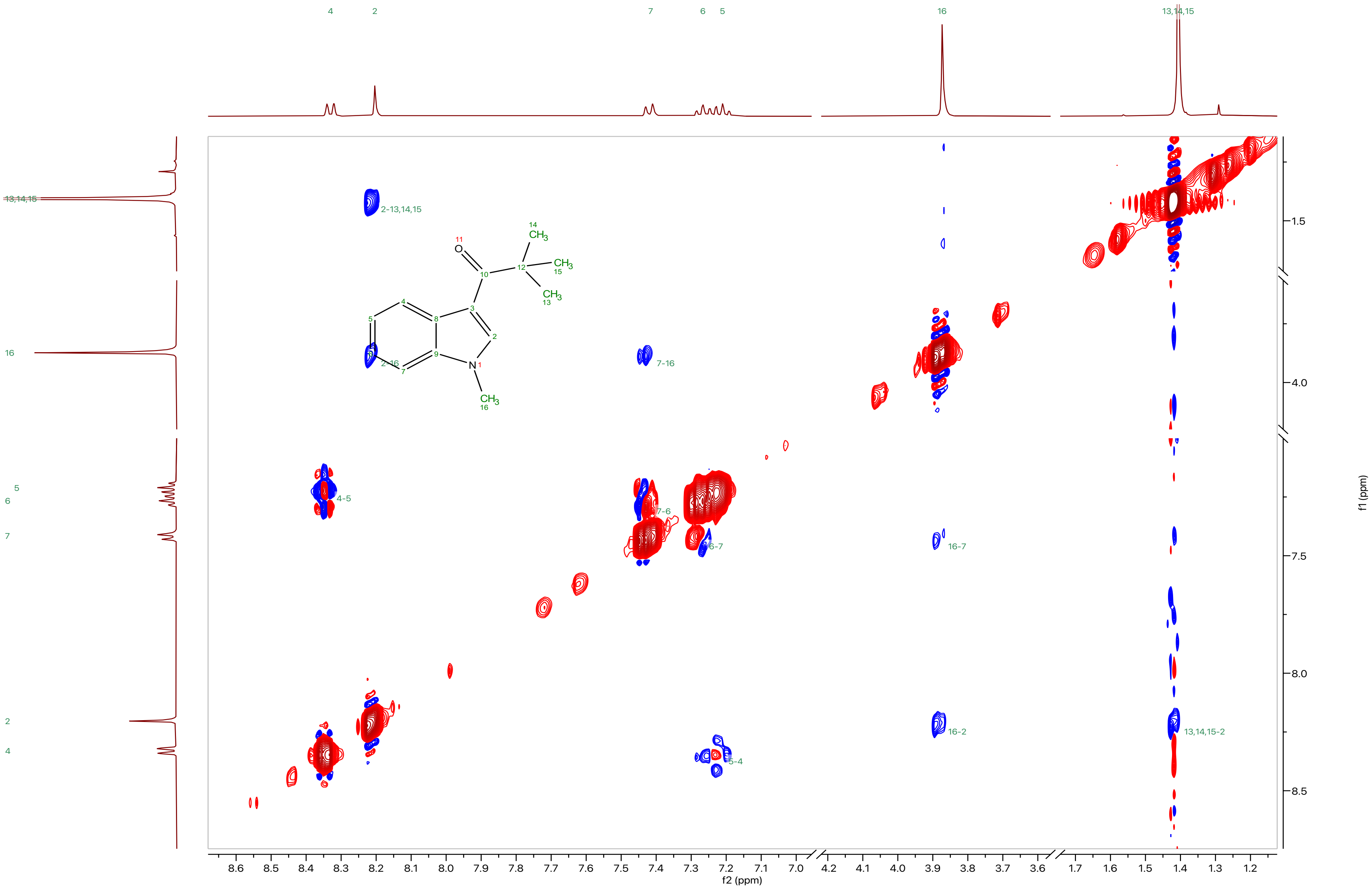

^1H-^1H NOESY (400 MHz, MeOD) of 1a

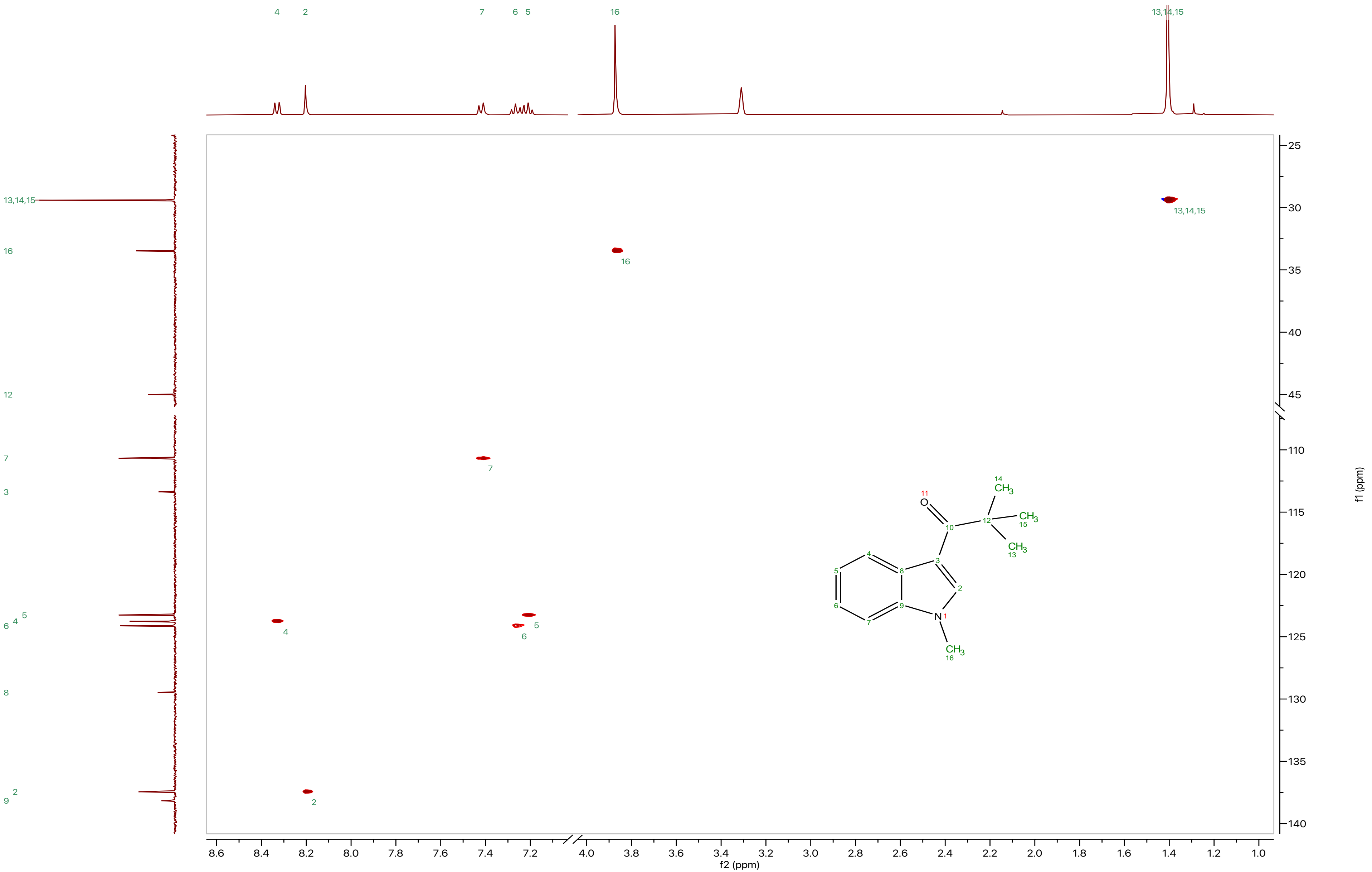

^{1}H-$^{13}C\{^{1}H\}$ HSQC NMR (400/101 MHz, MeOD) of 1a

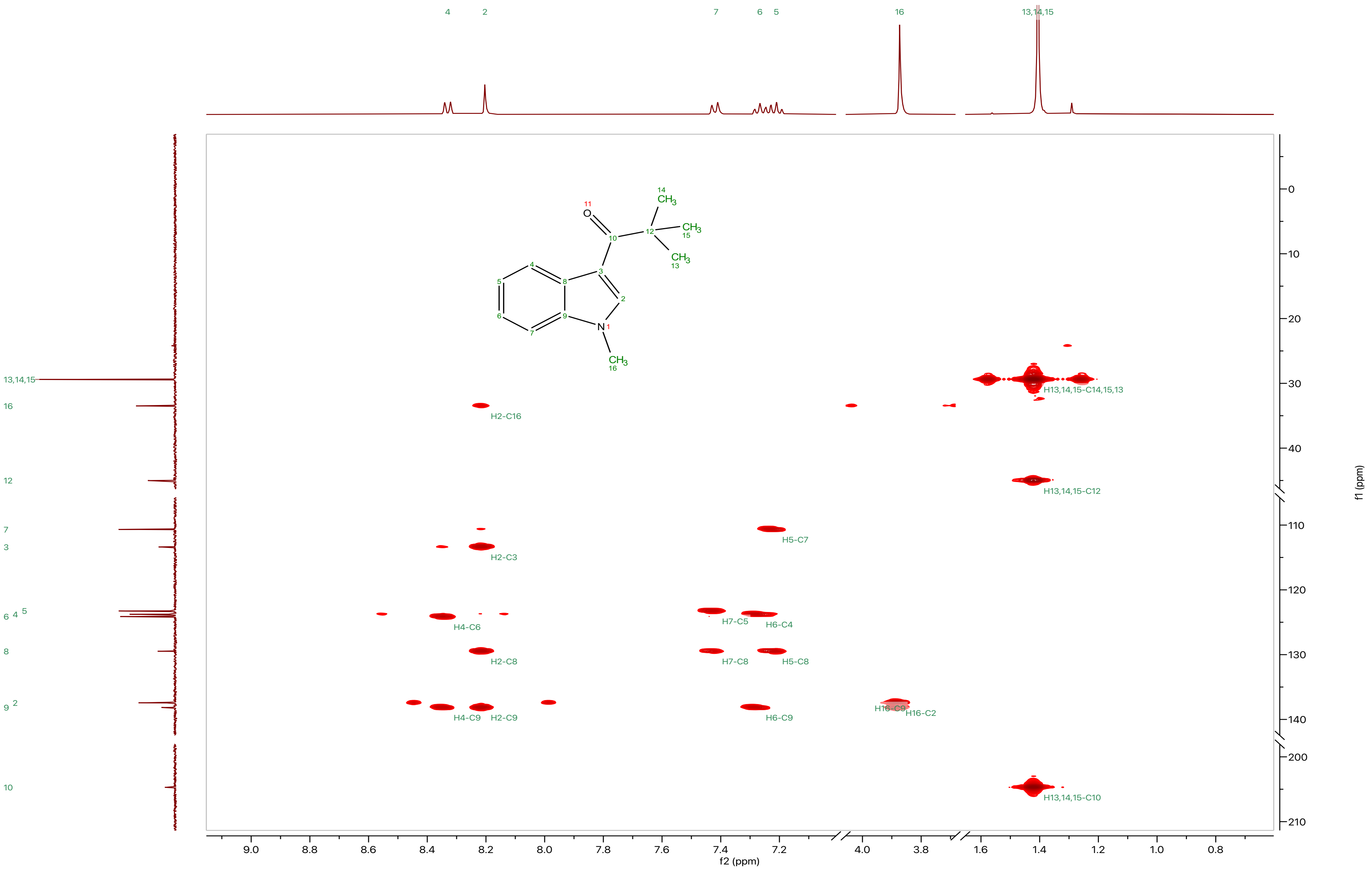

^{1}H-^{13}C{^{1}H} HMBC NMR (400/101 MHz, MeOD) of 1a

2a

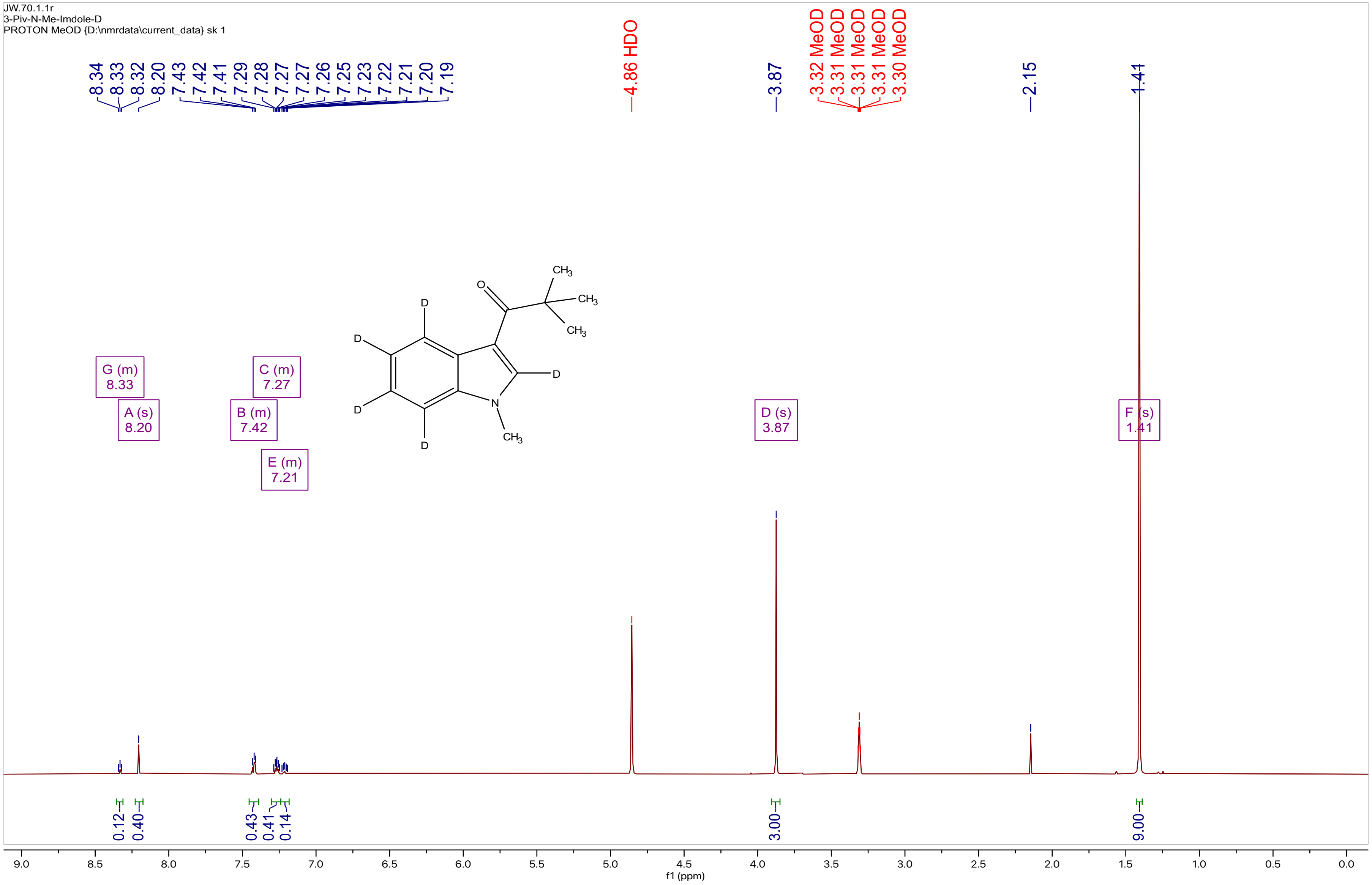

^{1}H NMR (400 MHz, MeOD) of 2a

JW.71.1.1r
3-Piv-N-Me-Imdole-D
C13CPD MeOD {D:\nmrdata\current_data} sk 1

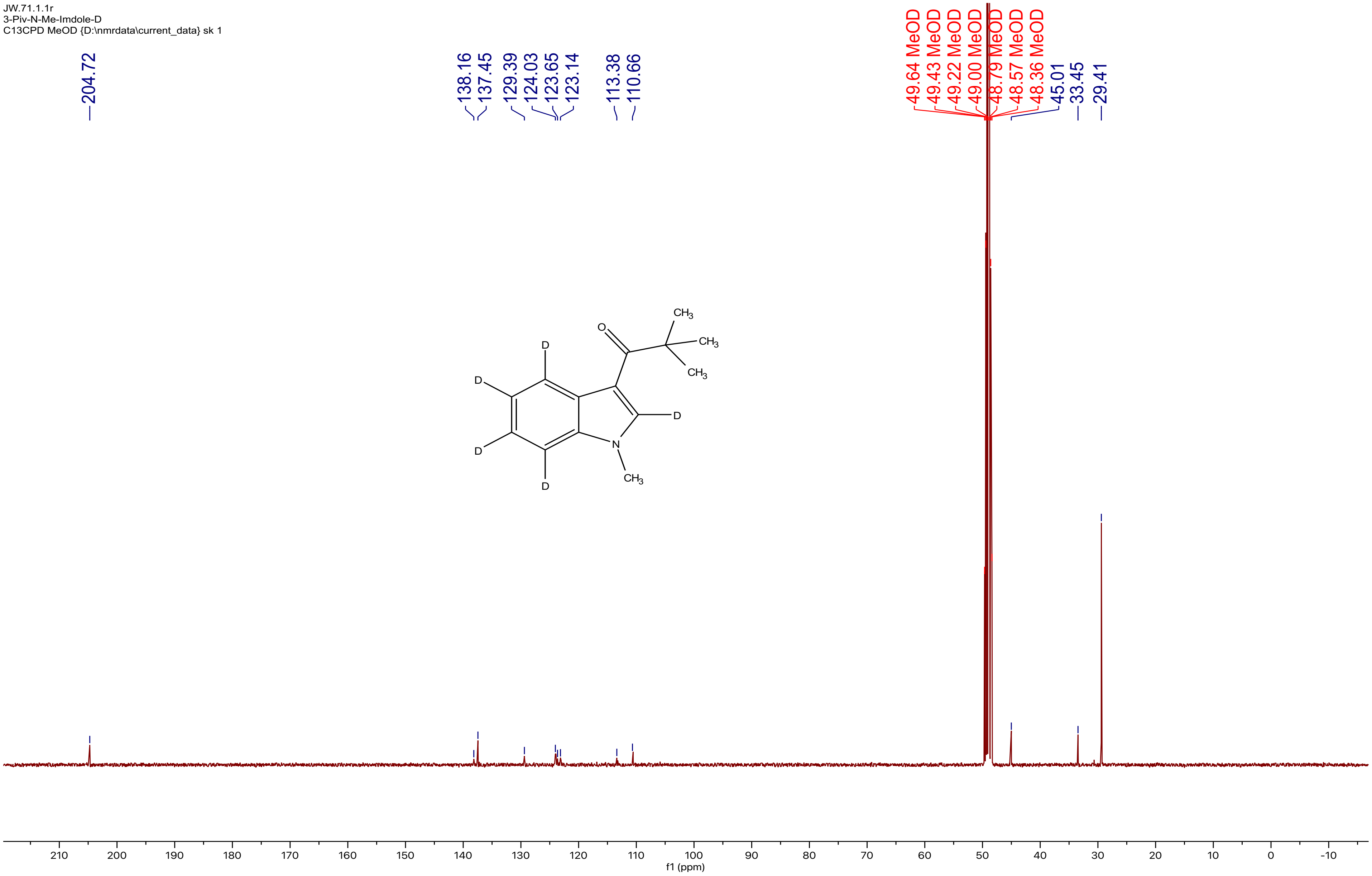

$^{13}C\{^1H\}$ NMR (101 MHz, MeOD) of 2a

1b

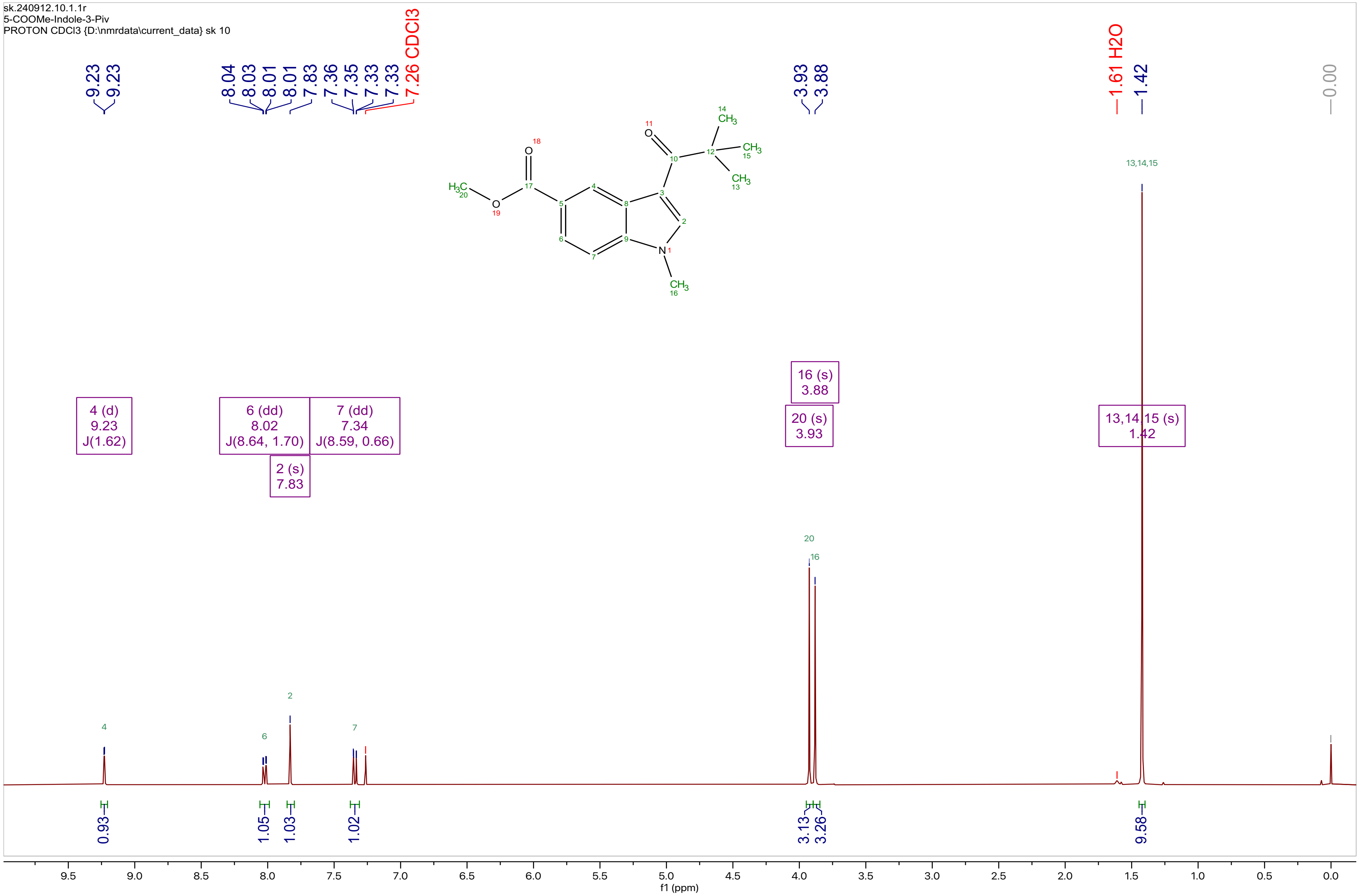

^{1}H NMR (400 MHz, $CDCl_3$) of 1b

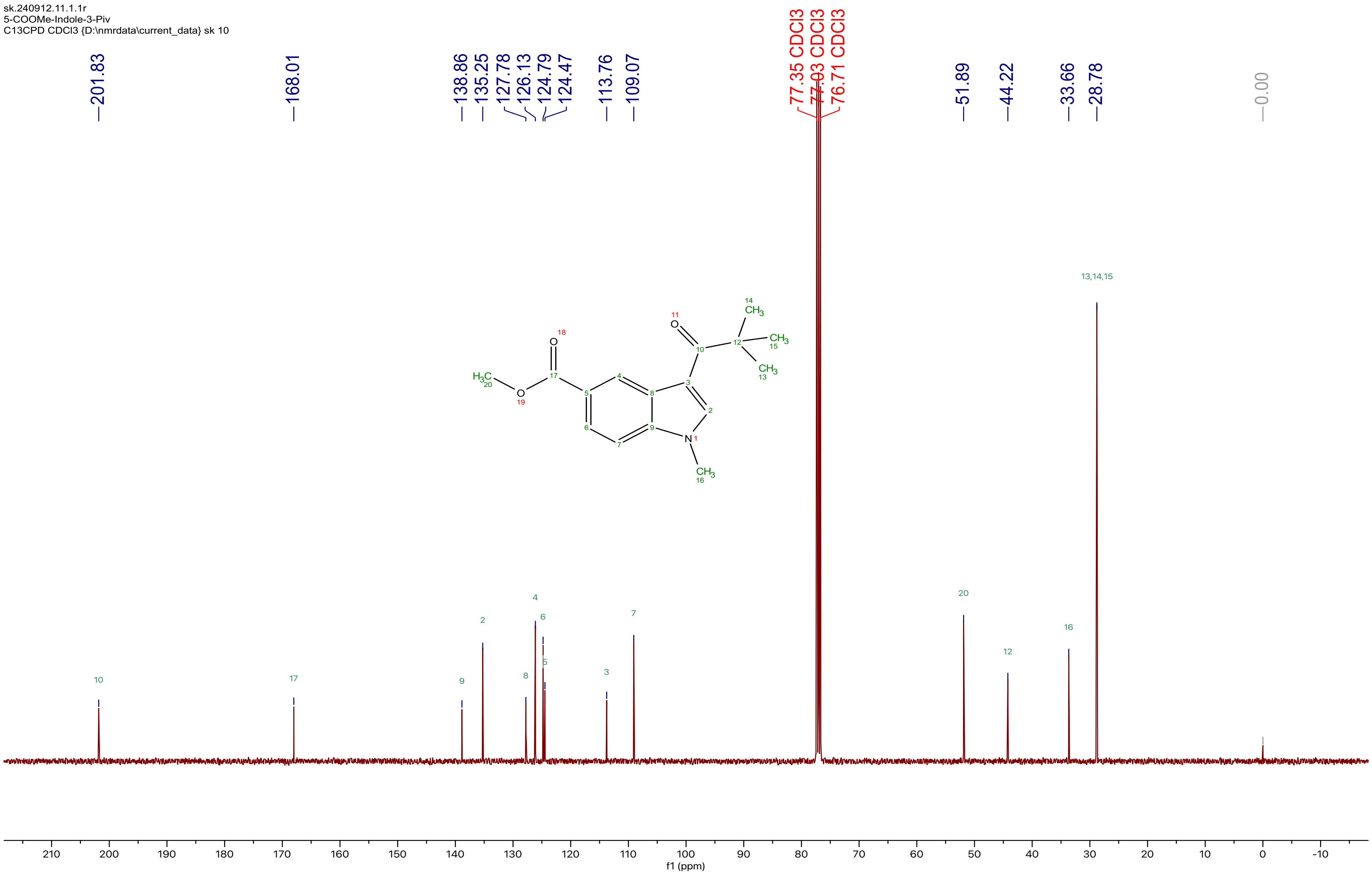

$^{13}C\{^1H\}$ NMR (101 MHz, $CDCl_3$) of 1b

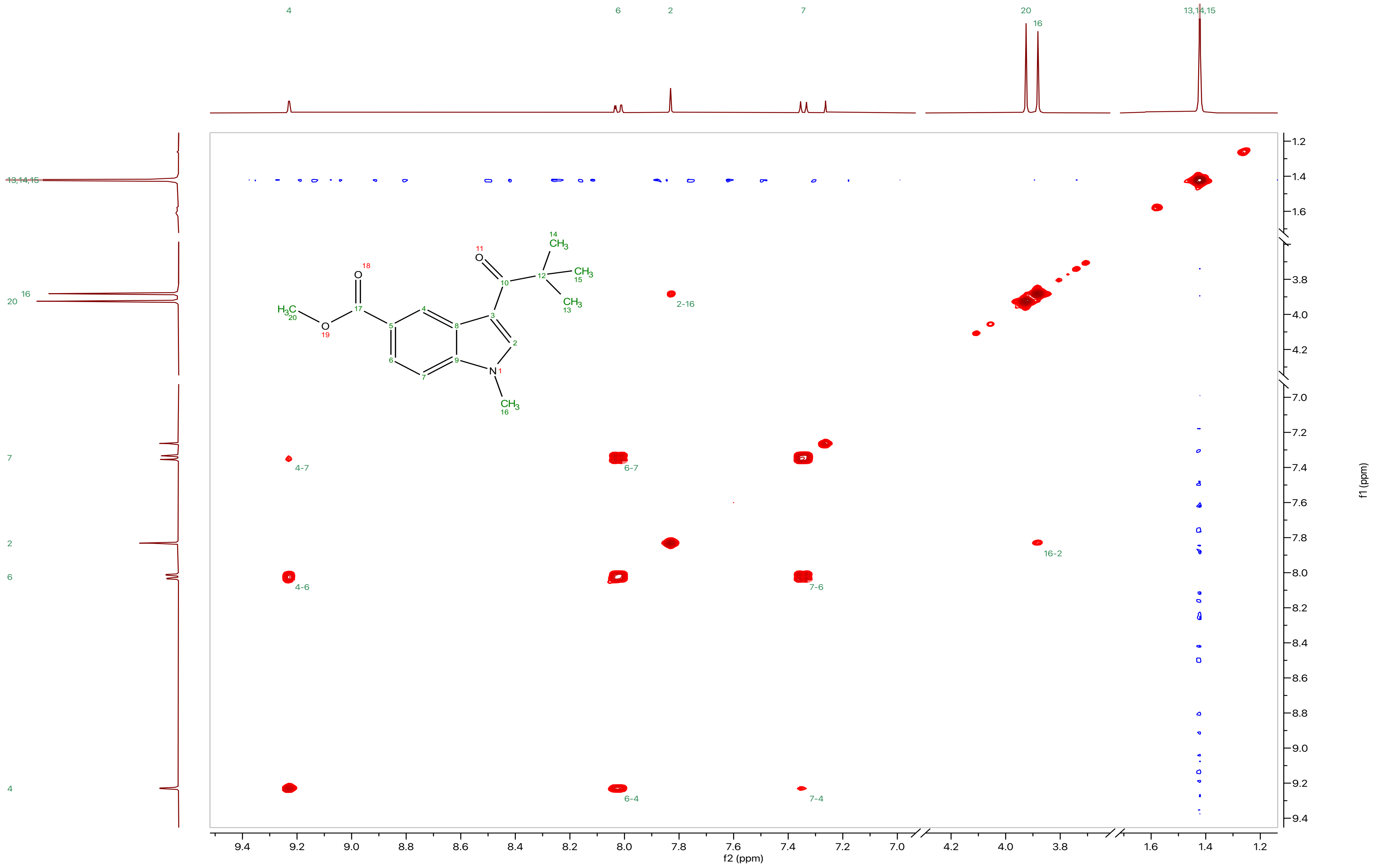

^{1}H-^{1}H COSY (400 MHz, $CDCl_3$) of 1b

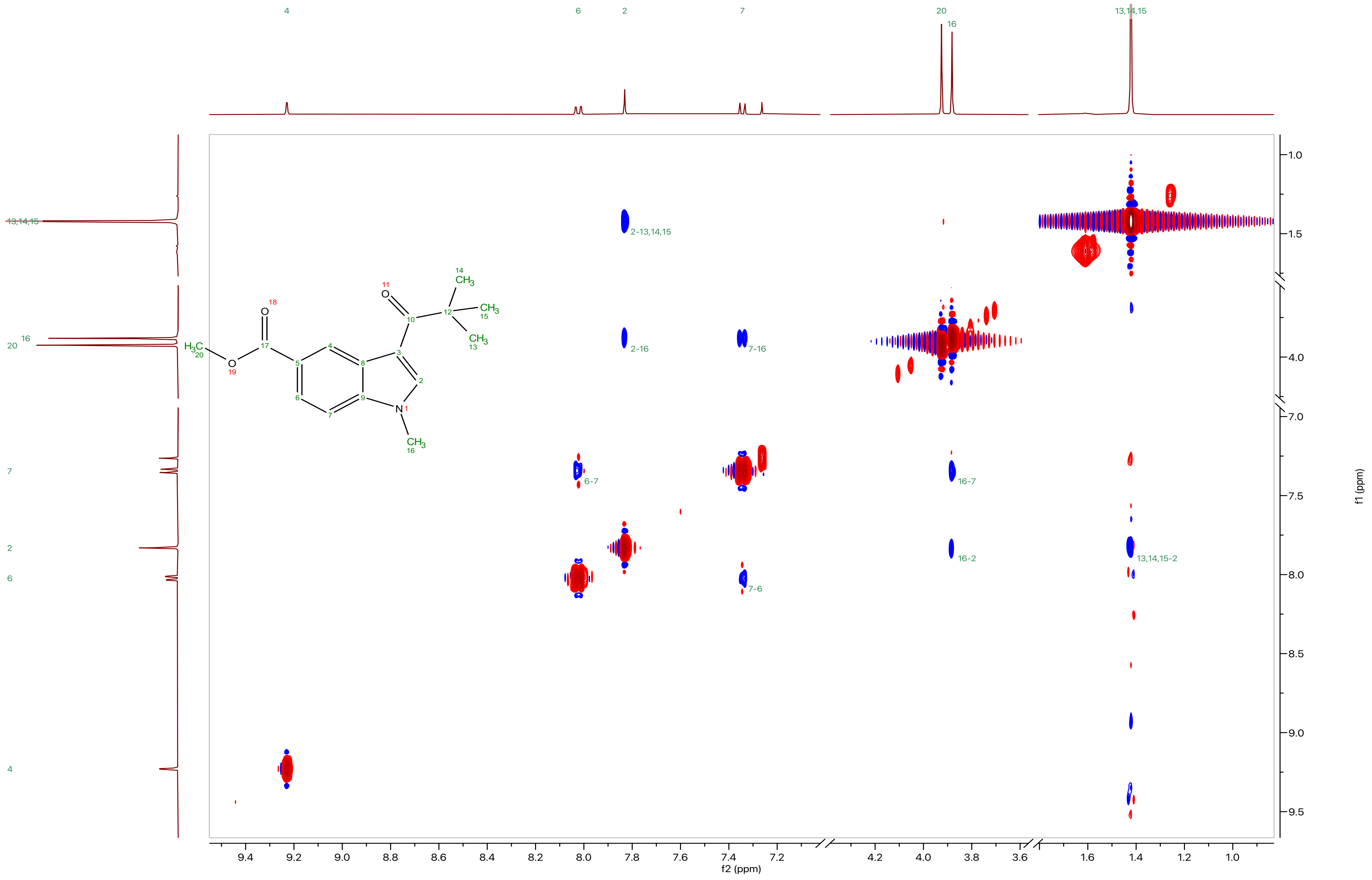

^{1}H-^{1}H NOESY (400 MHz, $CDCl_3$) of 1b

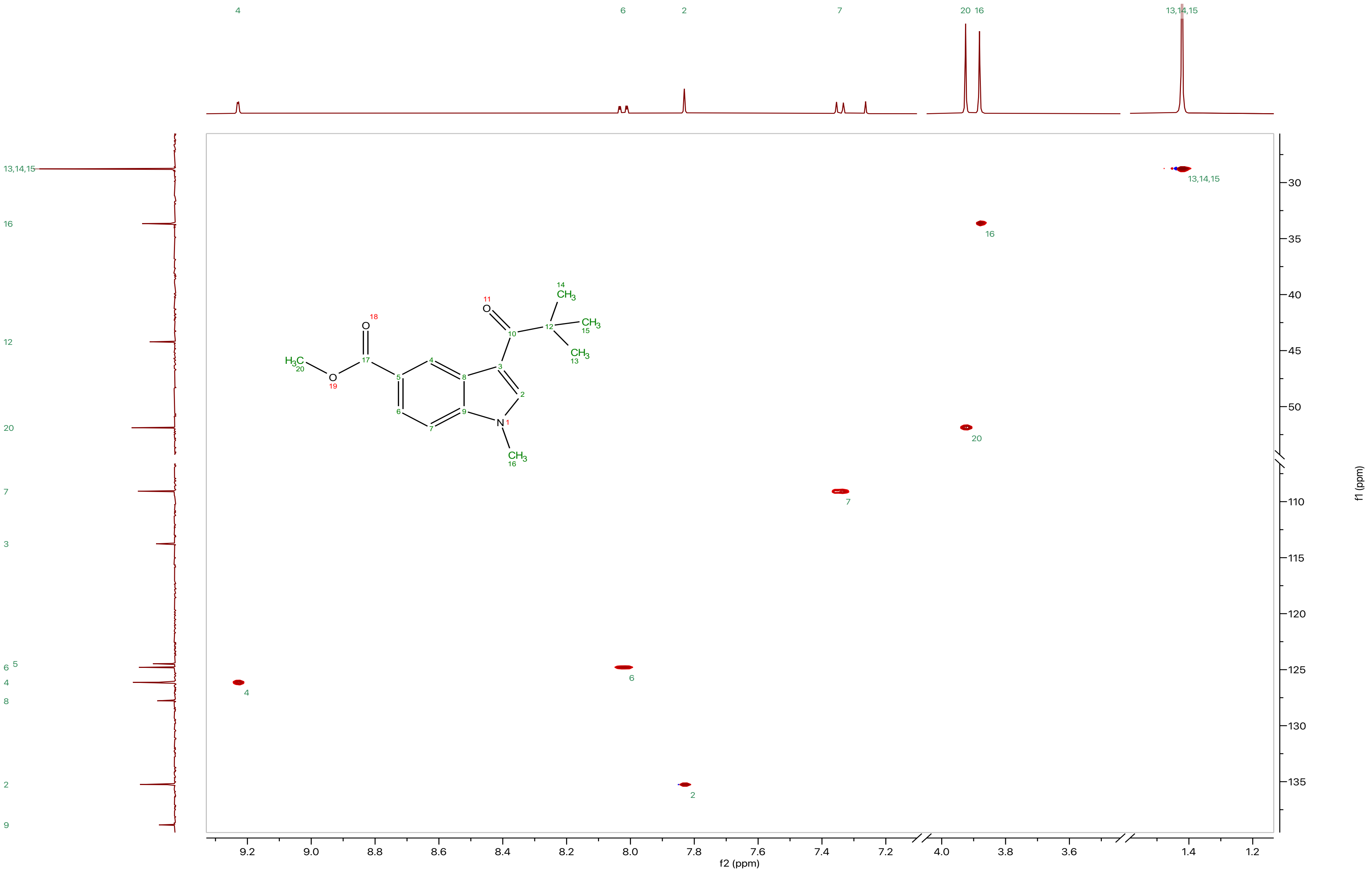

^{1}H-^{13}C{^{1}H} HSQC NMR (400/101 MHz, $CDCl_3$) of 1b

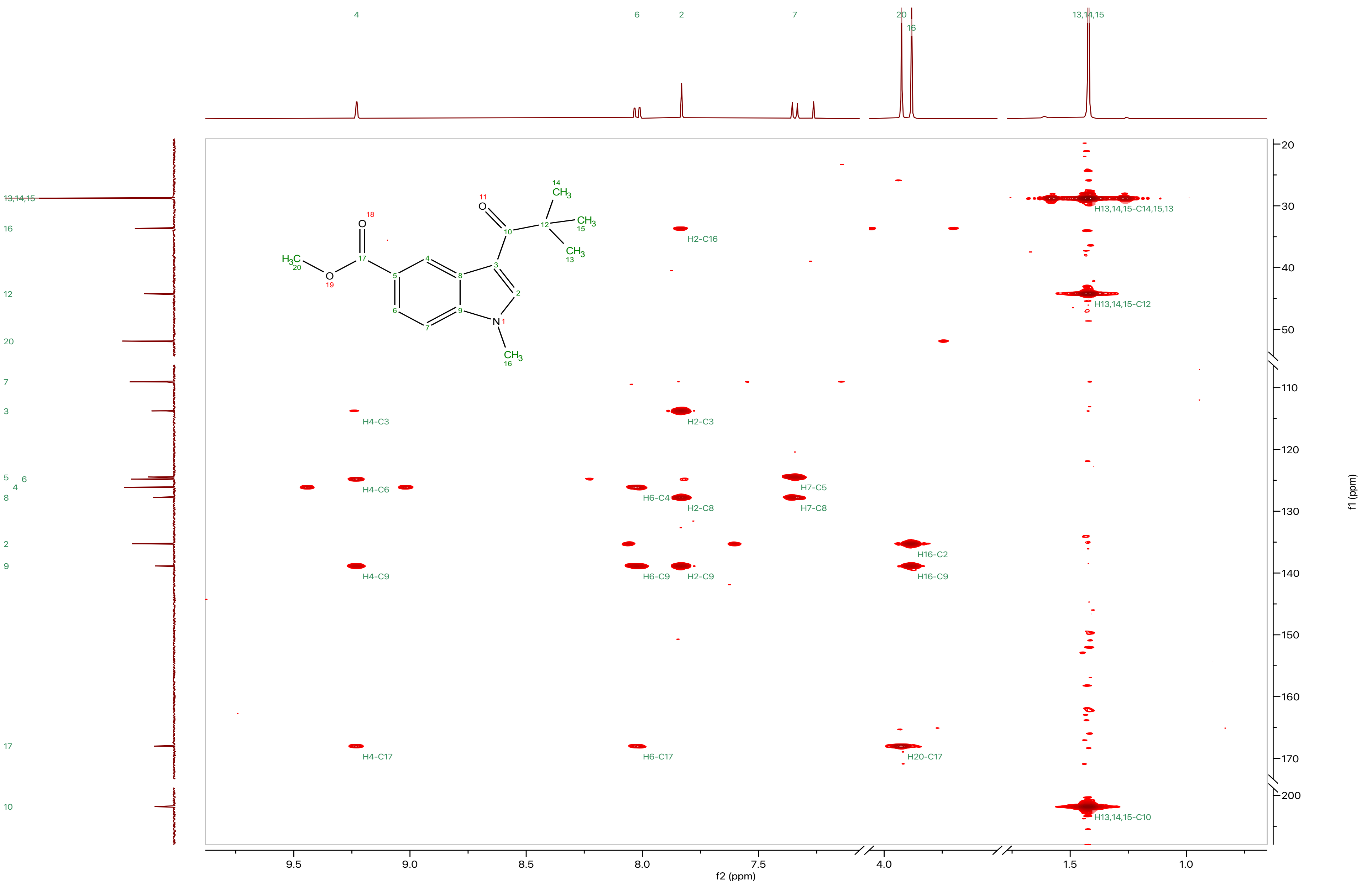

^{1}H-$^{13}C\{^{1}H\}$ HMBC NMR (400/101 MHz, $CDCl_3$) of 1b

2b

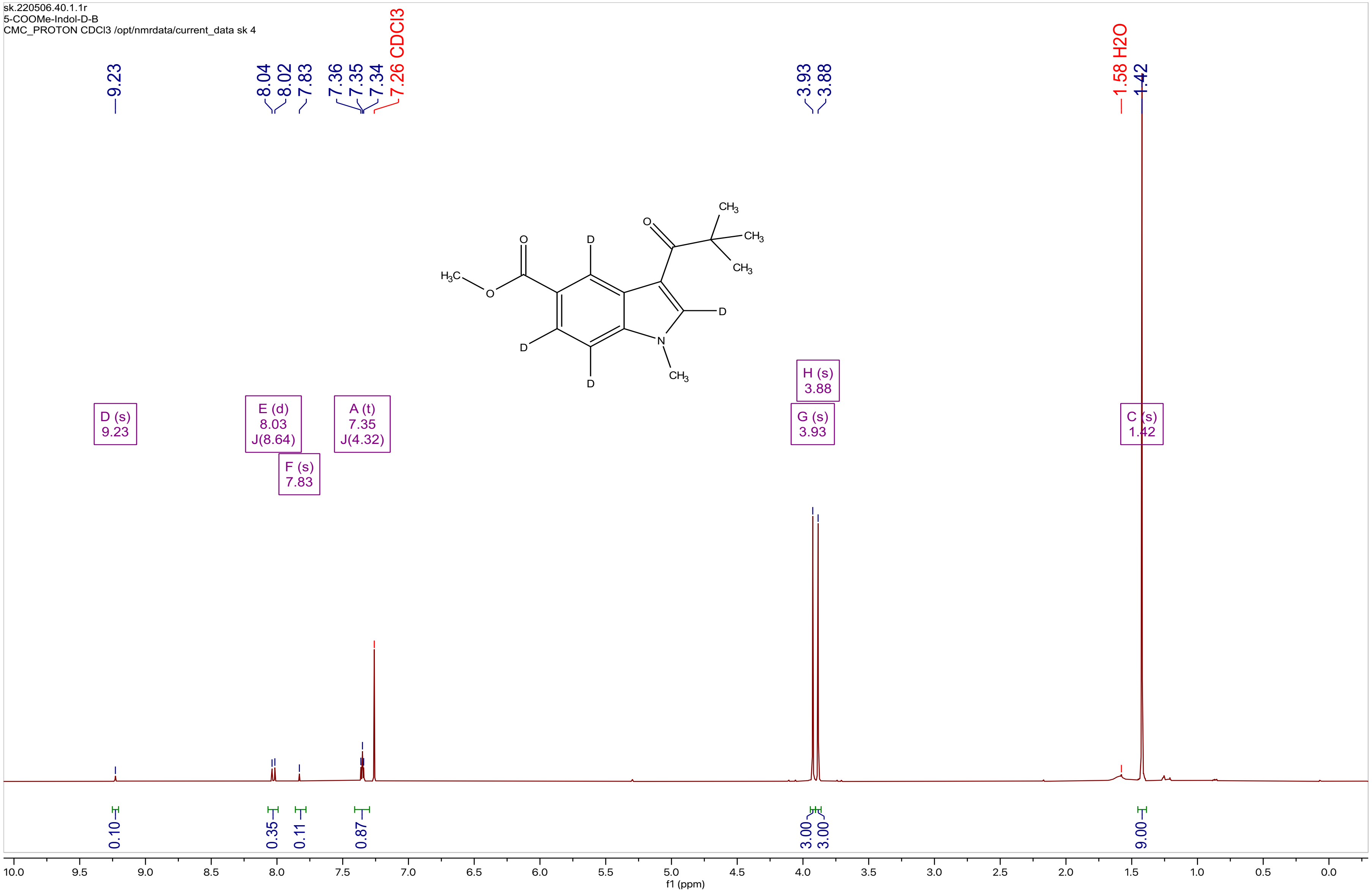

^{1}H NMR (400 MHz, $CDCl_3$) of 2b

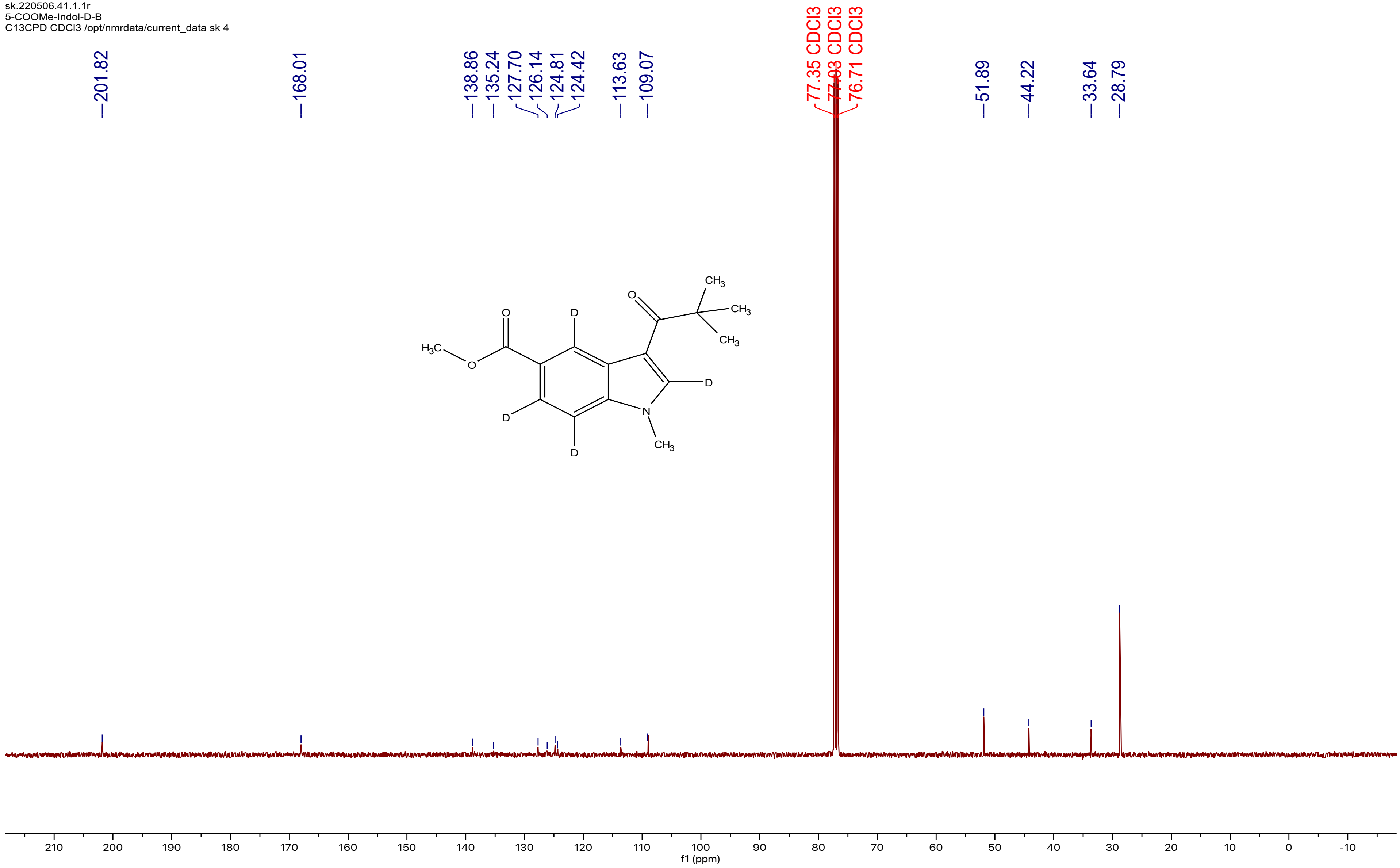

$^{13}C\{^1H\}$ NMR (101 MHz, $CDCl_3$) of 2b

1c

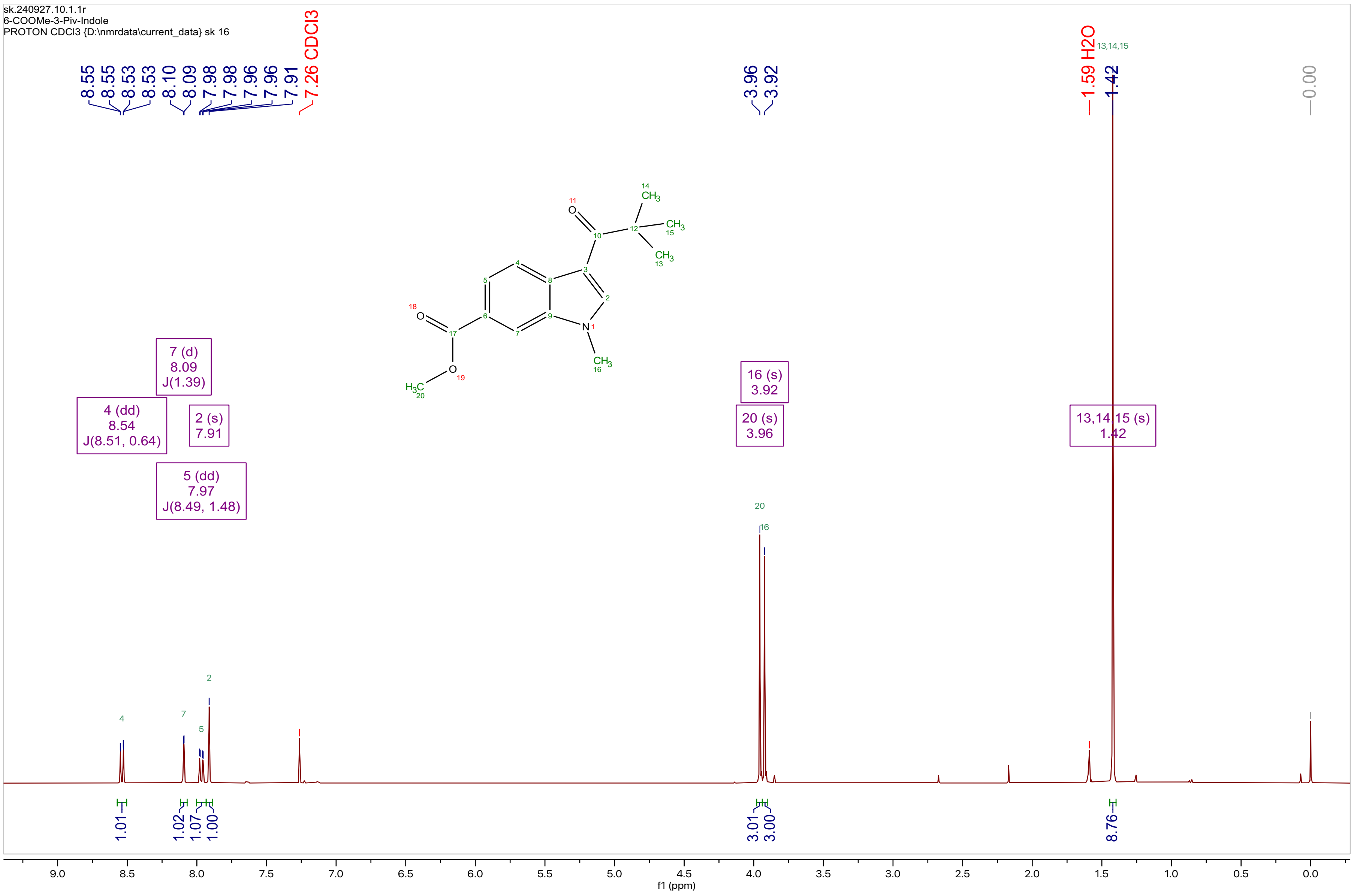

^{1}H NMR (400 MHz, $CDCl_3$) of 1c

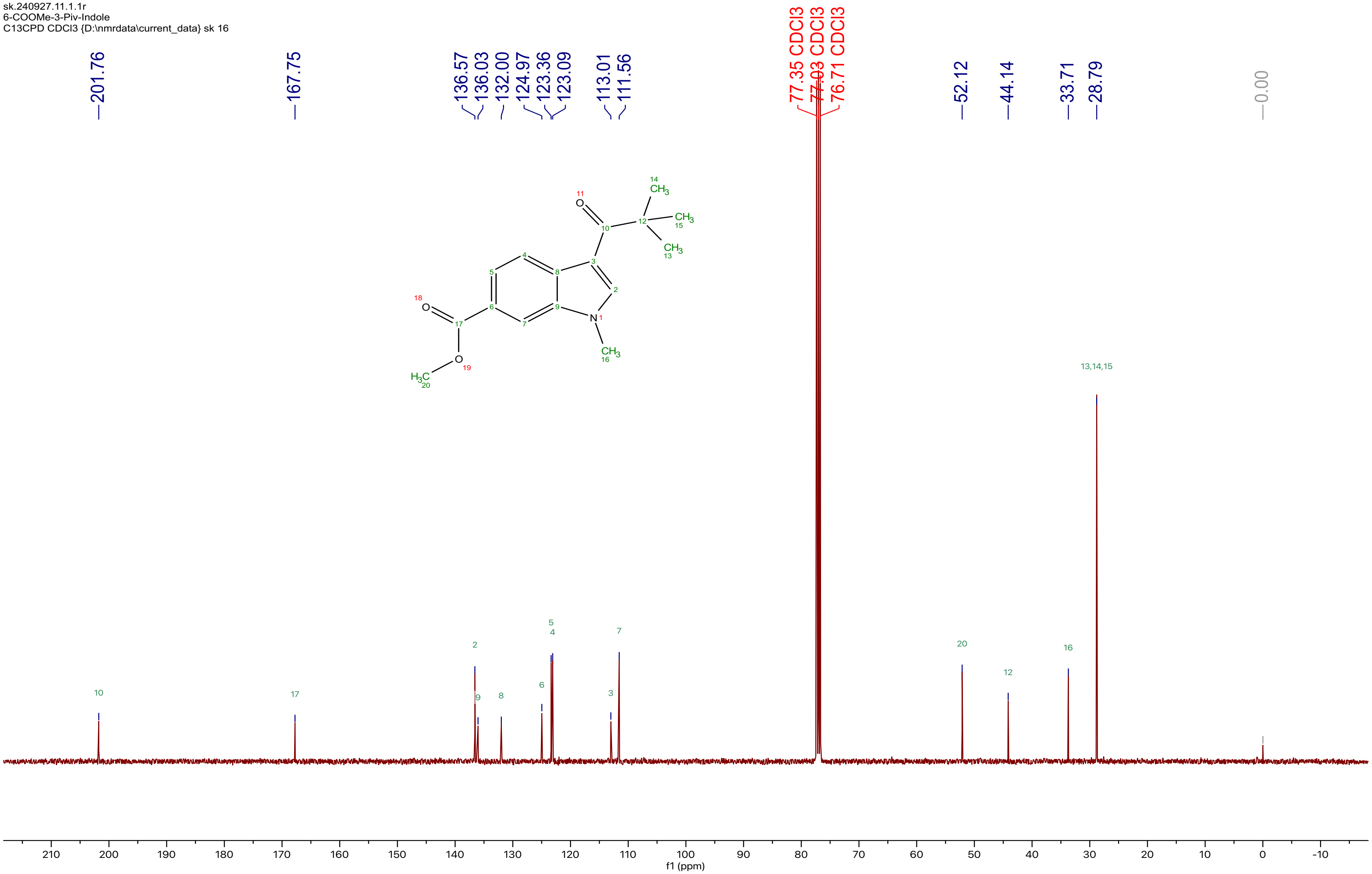

$^{13}C\{^{1}H\}$ NMR (101 MHz, $CDCl_3$) of 1c

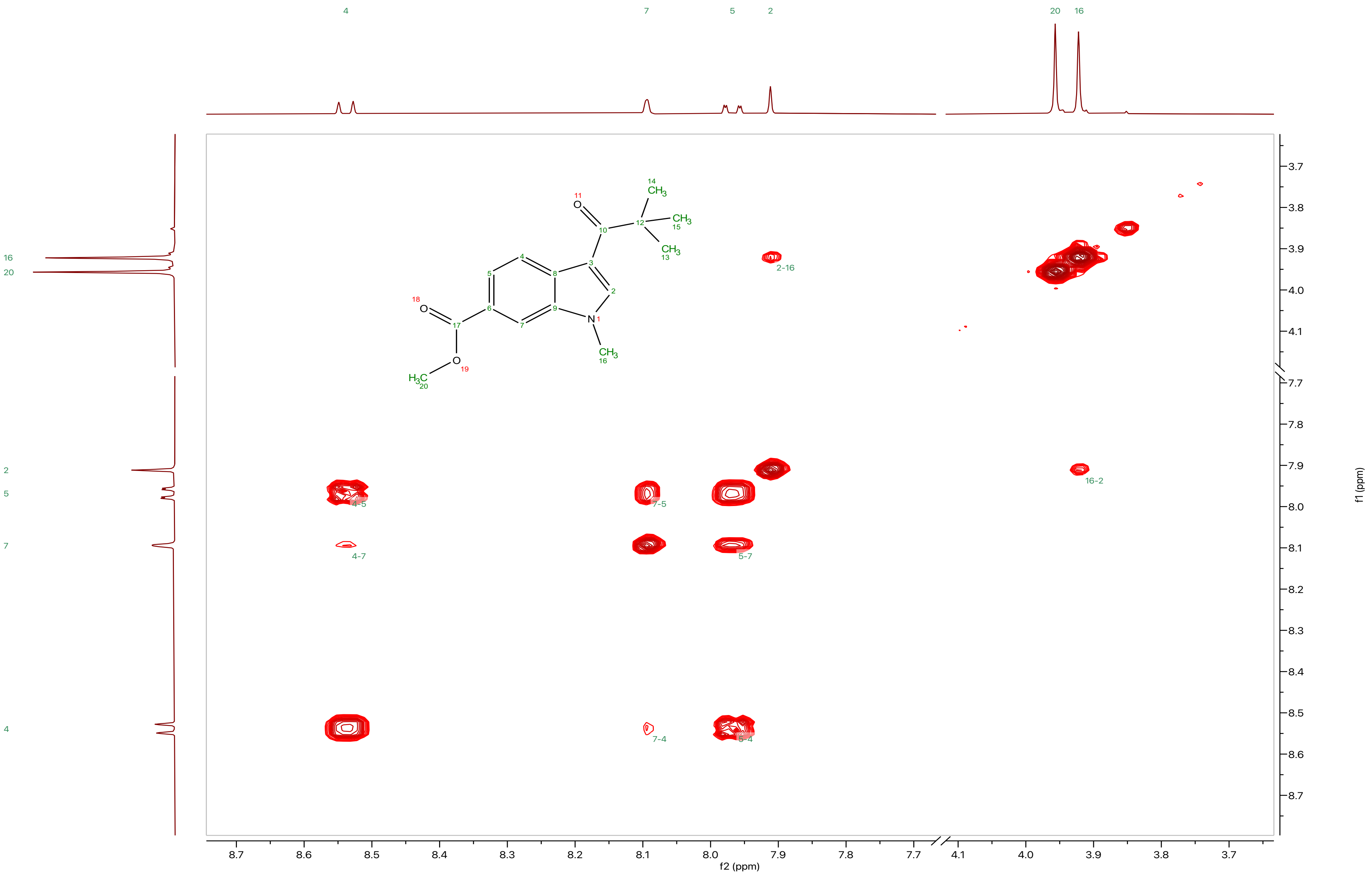

^{1}H-^{1}H COSY (400 MHz, $CDCl_3$) of 1c

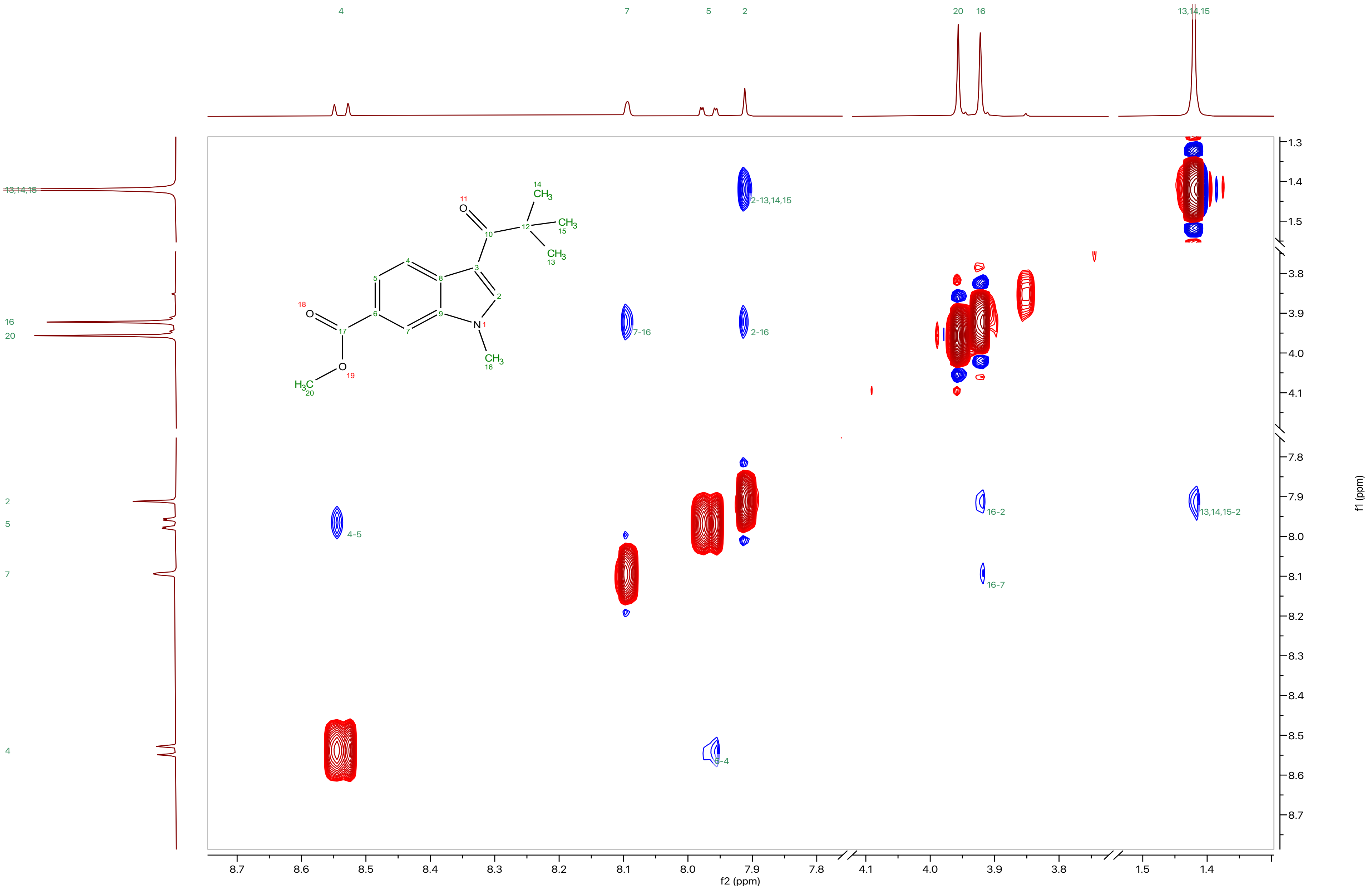

^{1}H-^{1}H NOESY (400 MHz, $CDCl_3$) of 1c

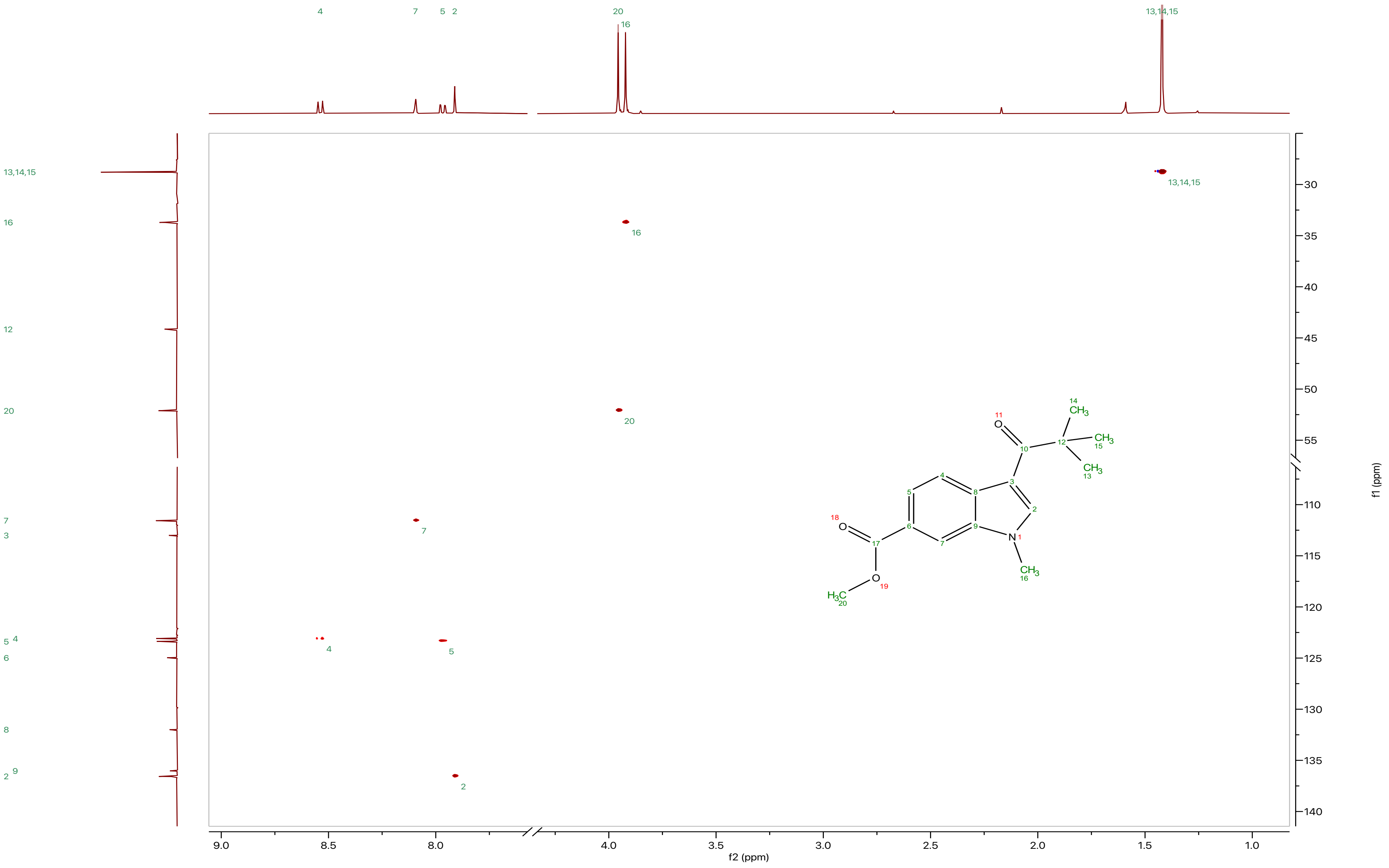

^{1}H-$^{13}C\{^{1}H\}$ HSQC NMR (400/101 MHz, $CDCl_3$) of 1c

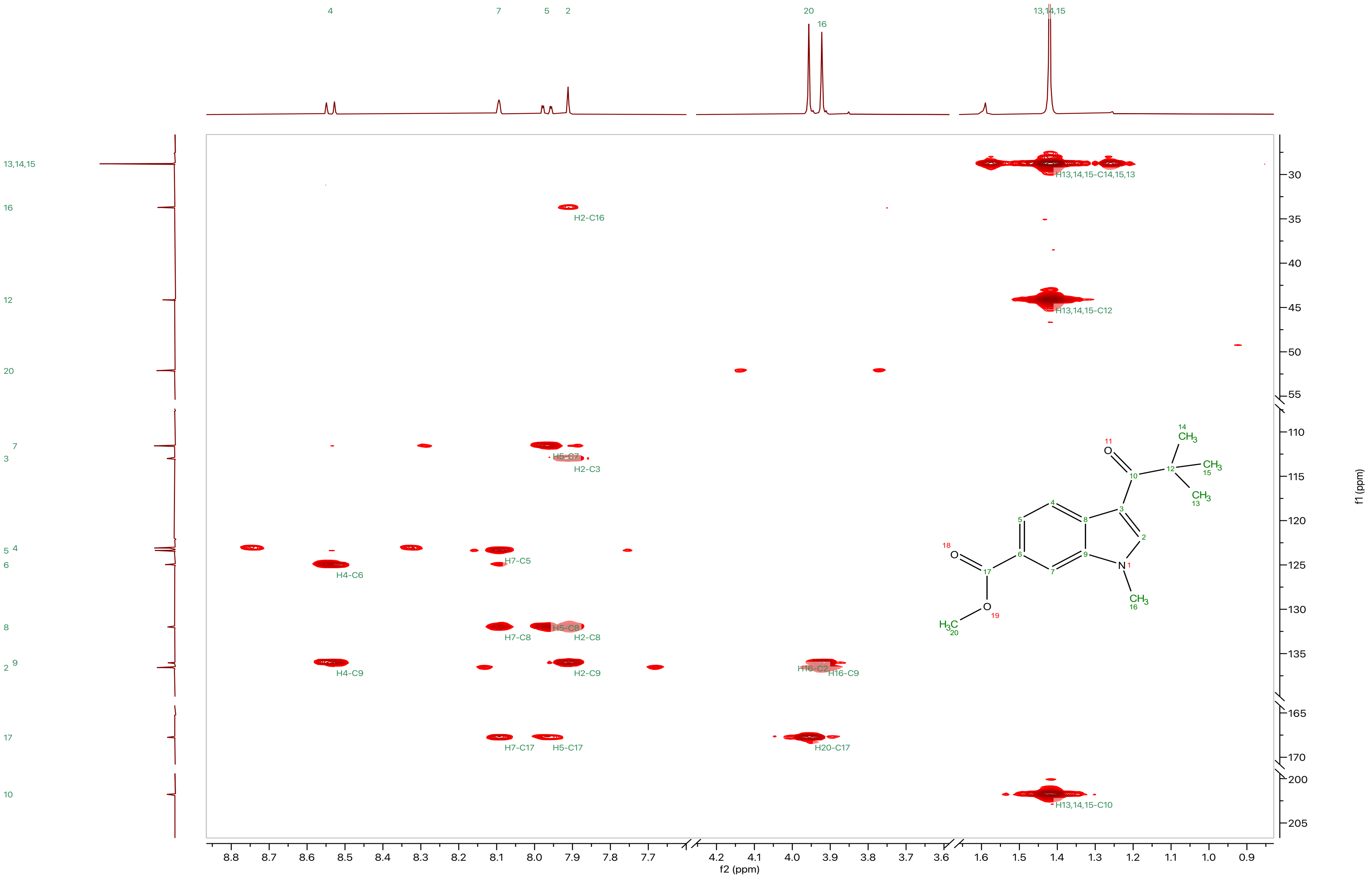

^{1}H-$^{13}C\{^{1}H\}$ HMBC NMR (400/101 MHz, $CDCl_3$) of 1c

2c

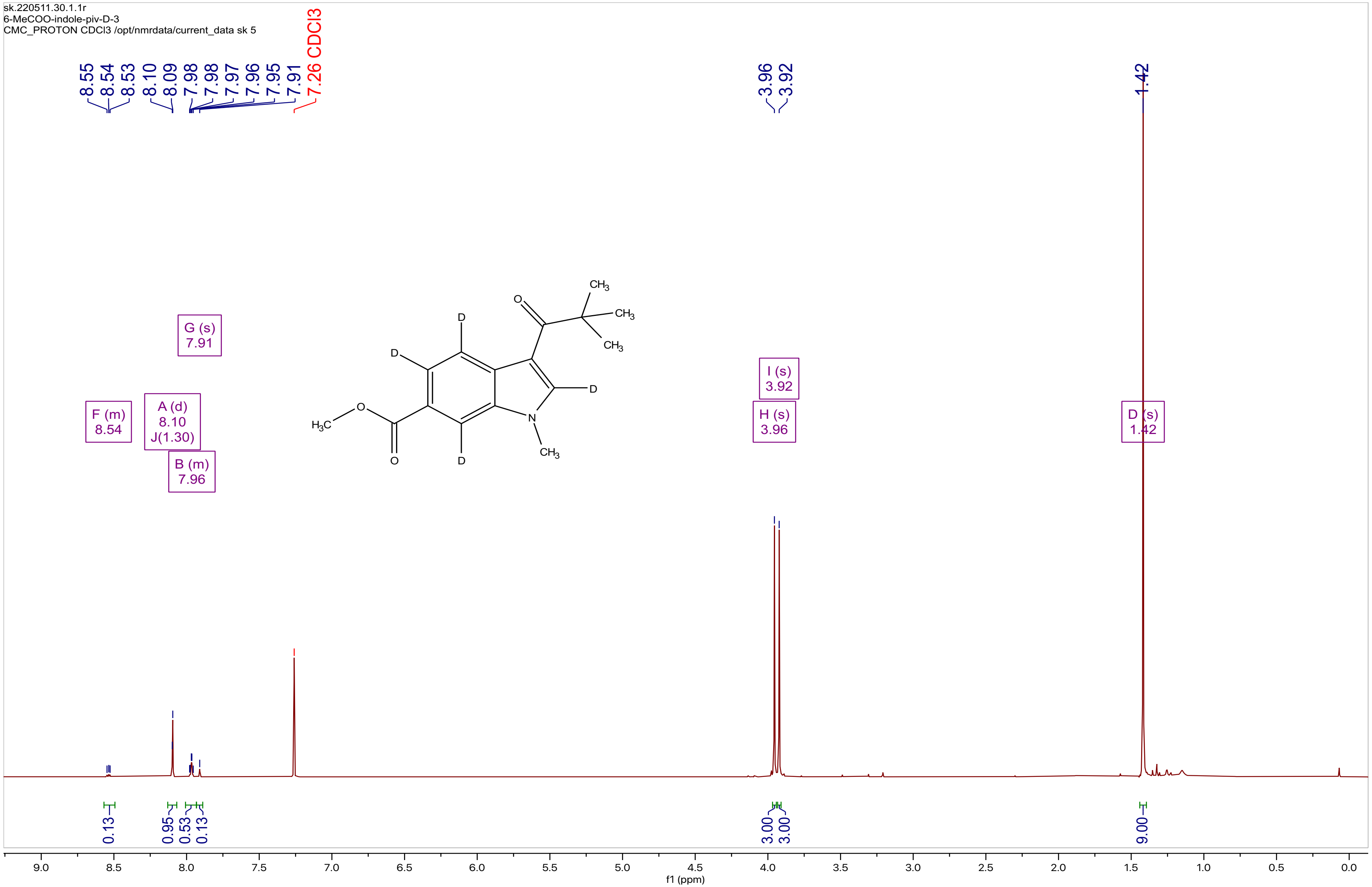

^{1}H NMR (400 MHz, $CDCl_3$) of 2c

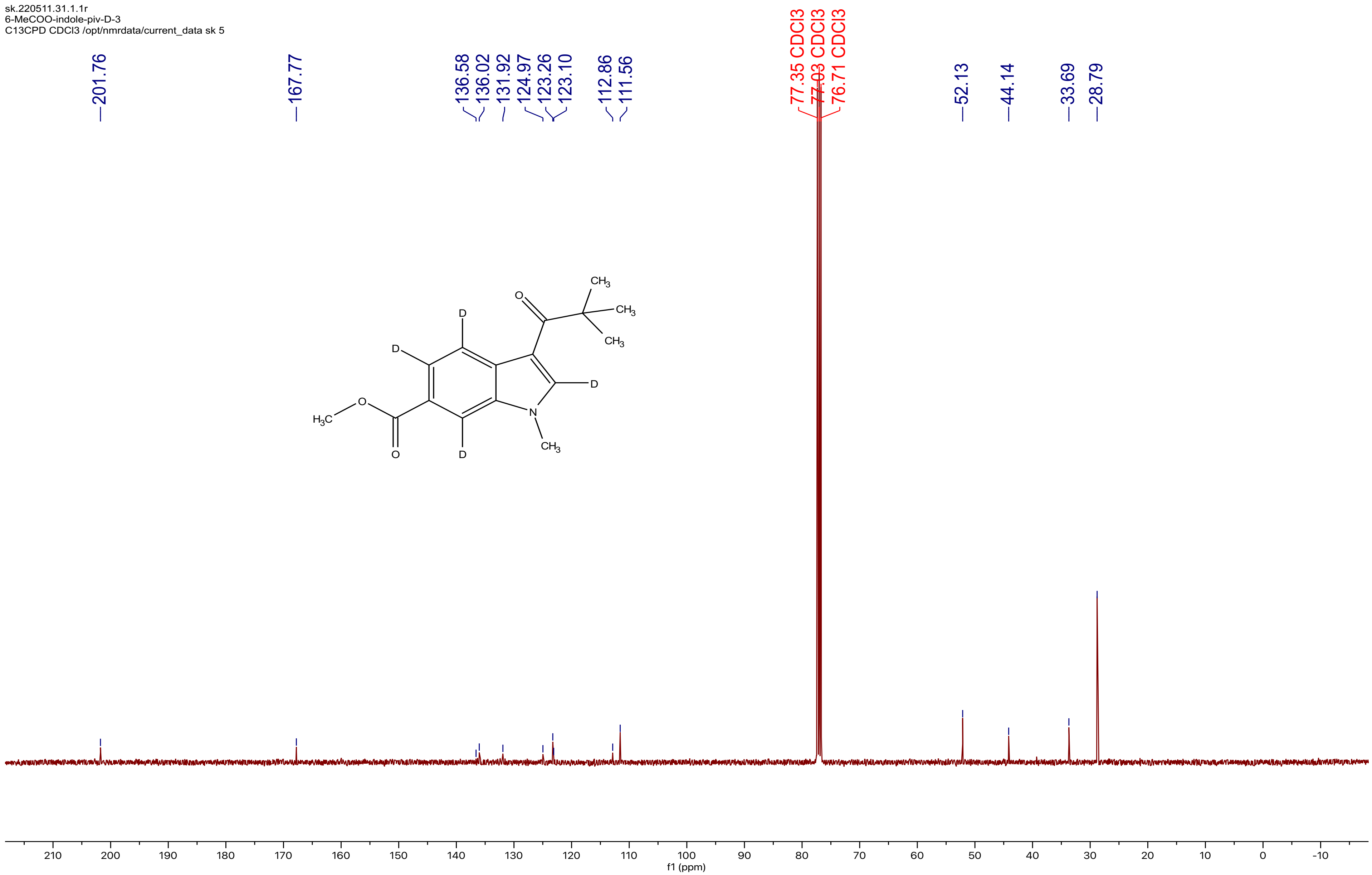

$^{13}C\{^{1}H\}$ NMR (101 MHz, $CDCl_3$) of 2c

1d

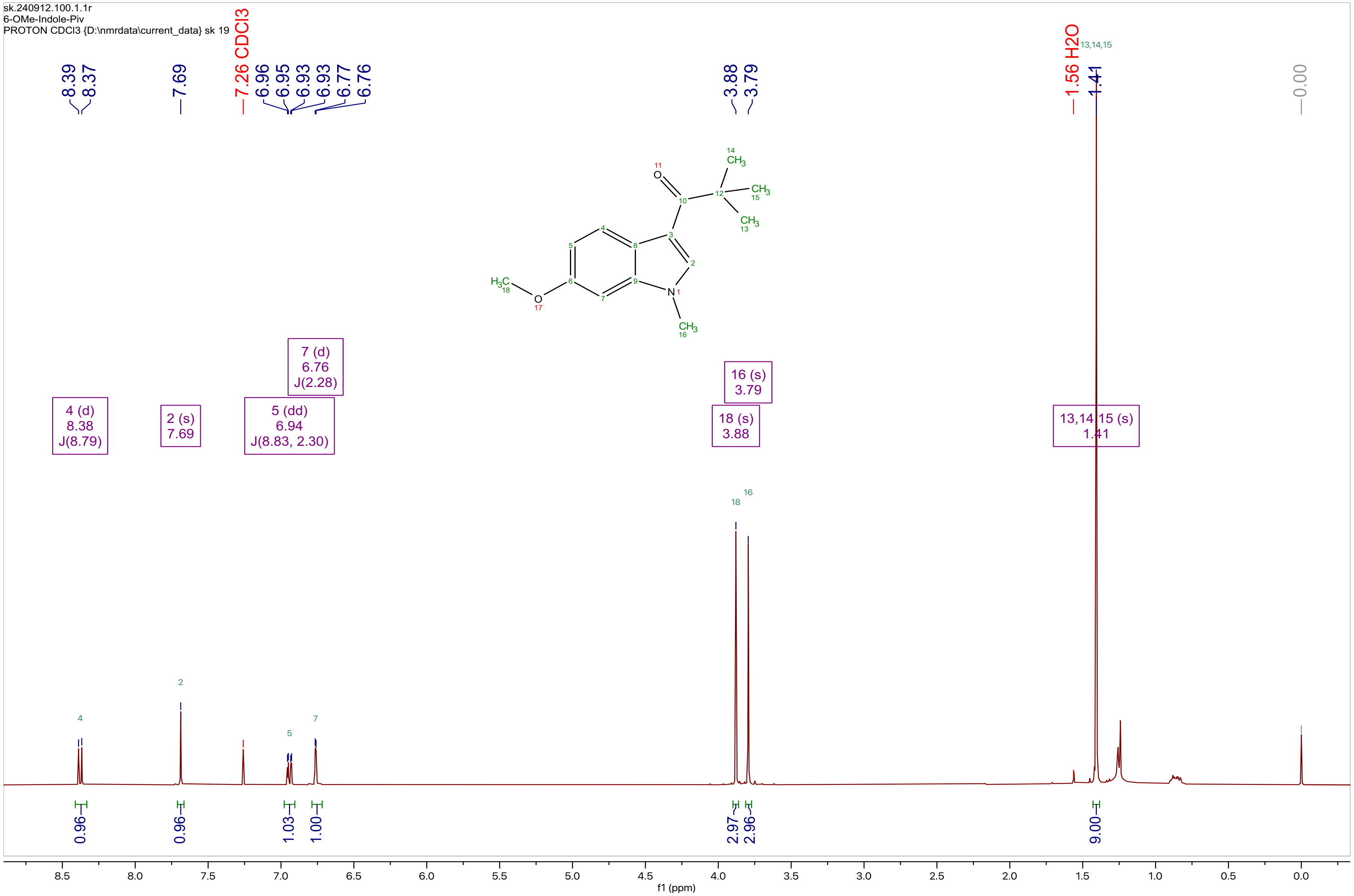

^{1}H NMR (400 MHz, $CDCl_3$) of 1d

sk.240912.101.1.1r
6-OMe-Indole-Piv
C13CPD CDCl3 {D:\nmrdata\current_data} sk 19

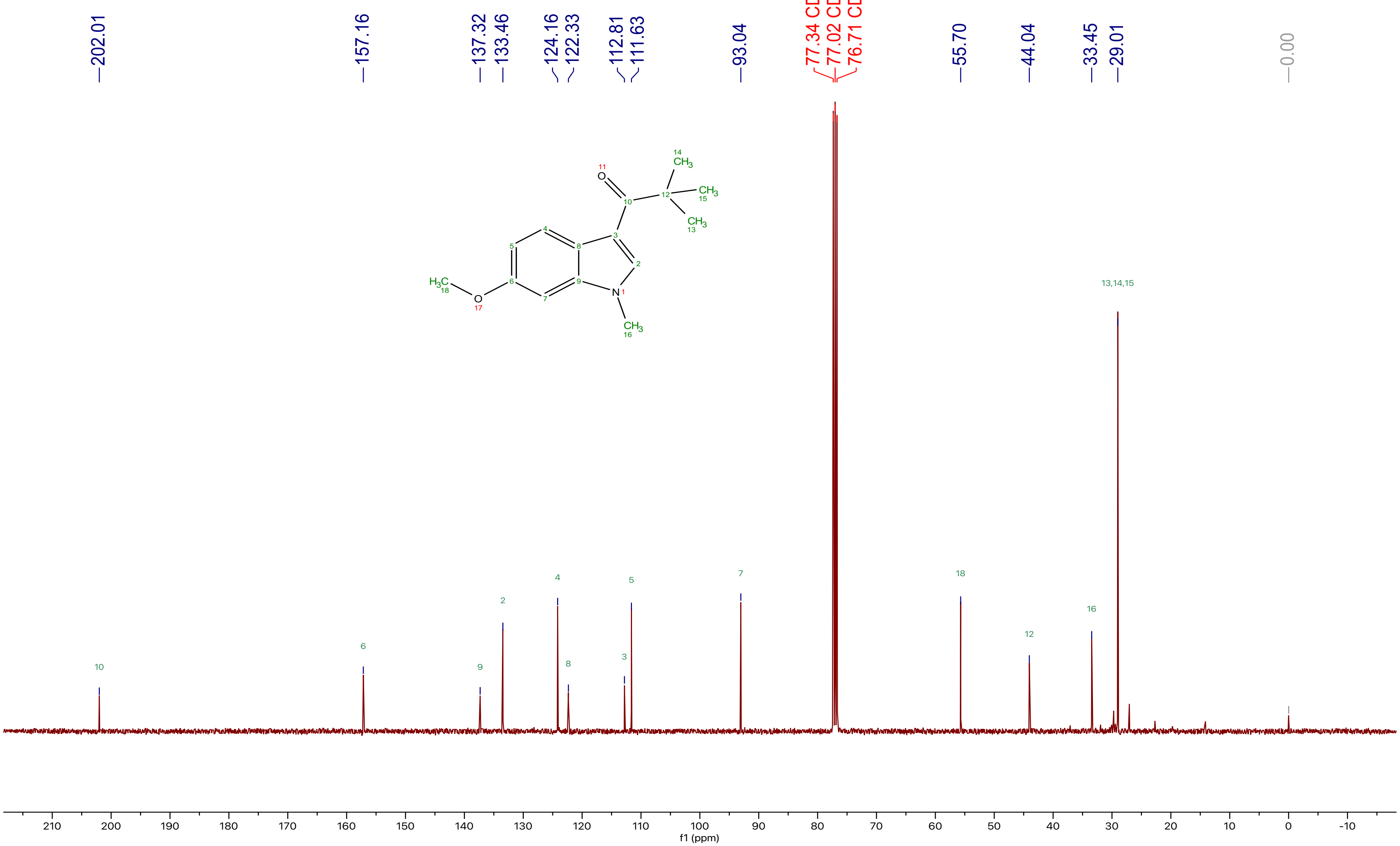

$^{13}C\{^1H\}$ NMR (101 MHz, $CDCl_3$) of 1d

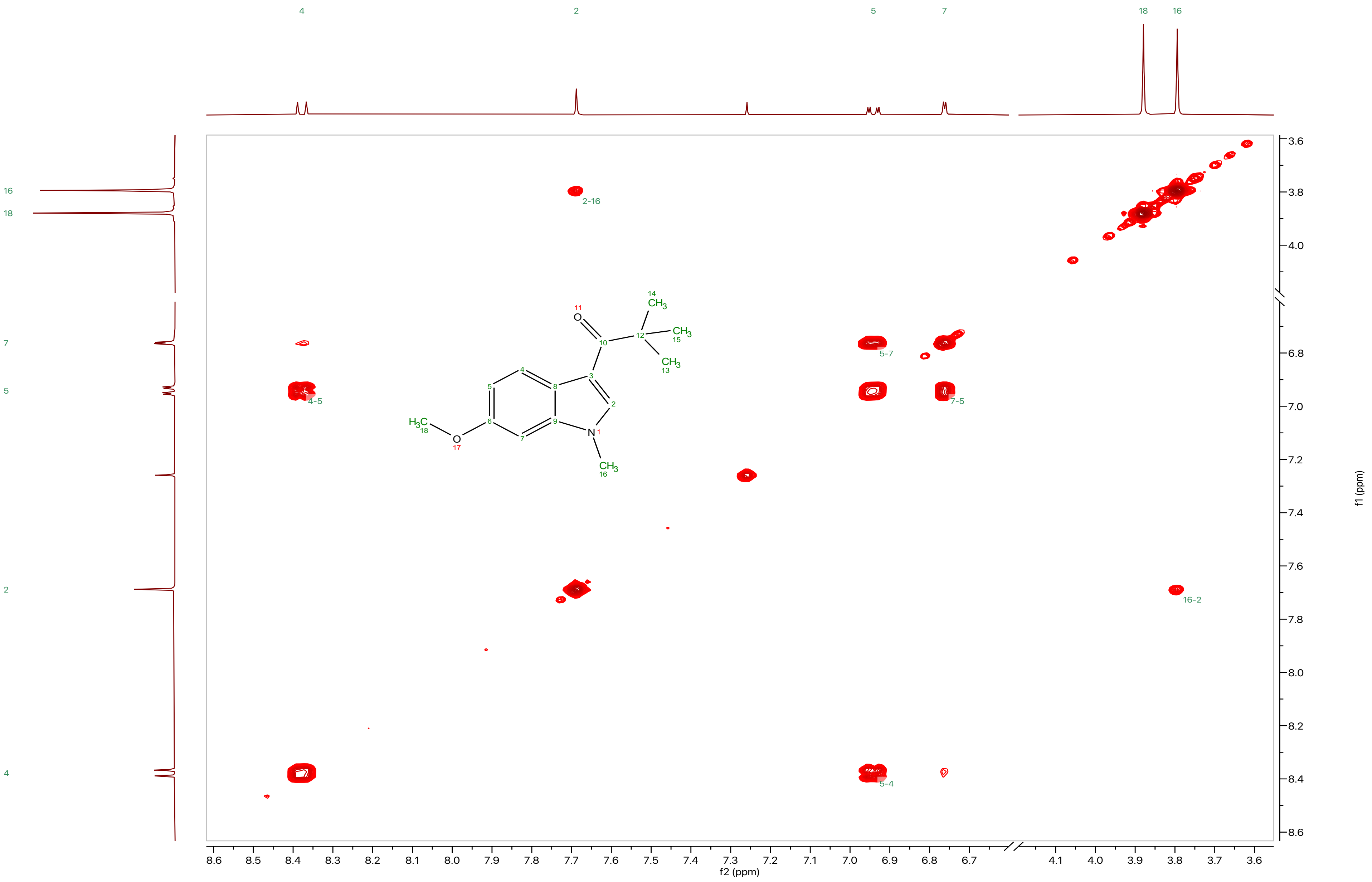

^{1}H-^{1}H COSY (400 MHz, $CDCl_3$) of 1d

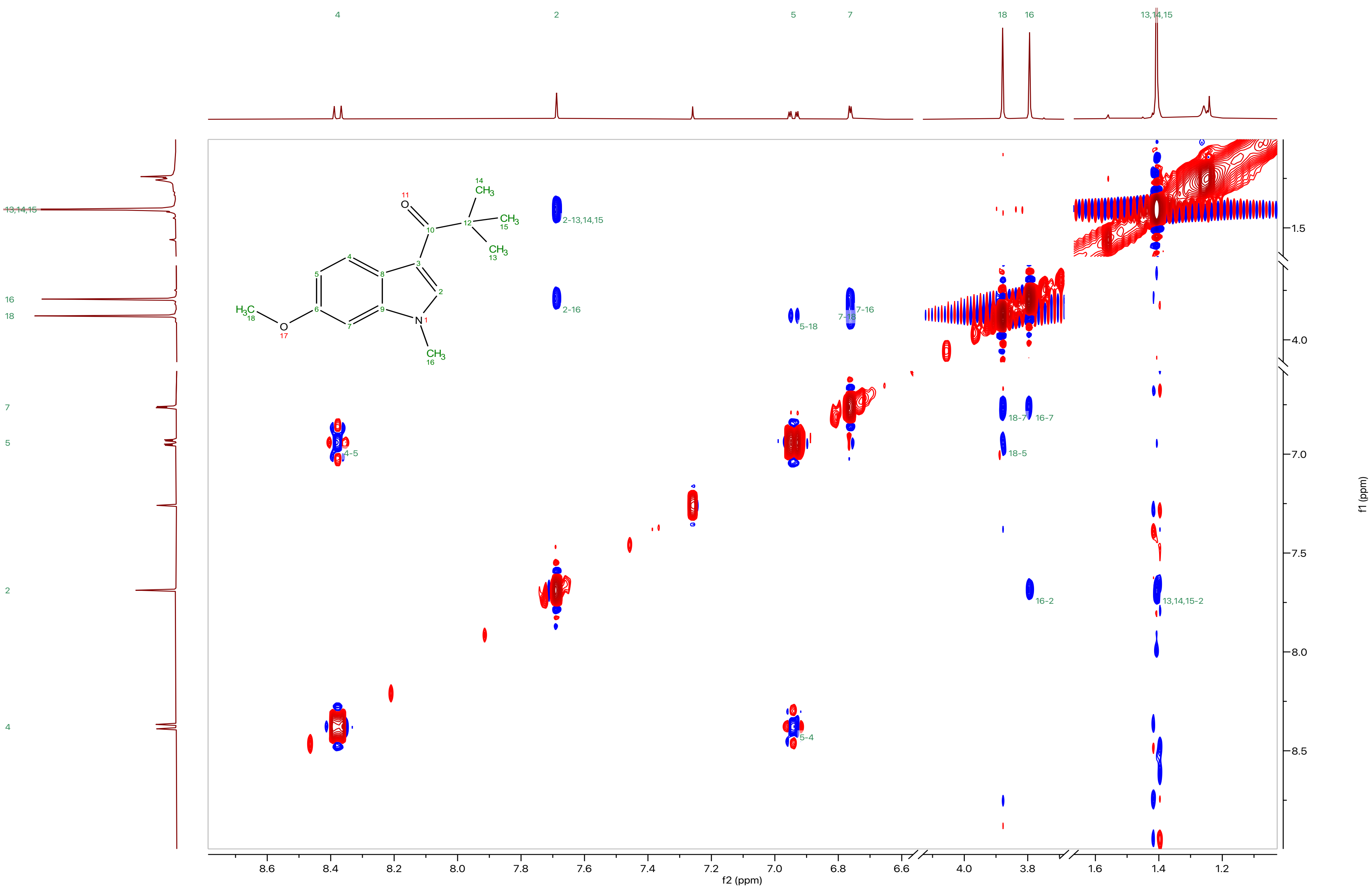

^1H-^1H NOESY (400 MHz, $CDCl_3$) of 1d

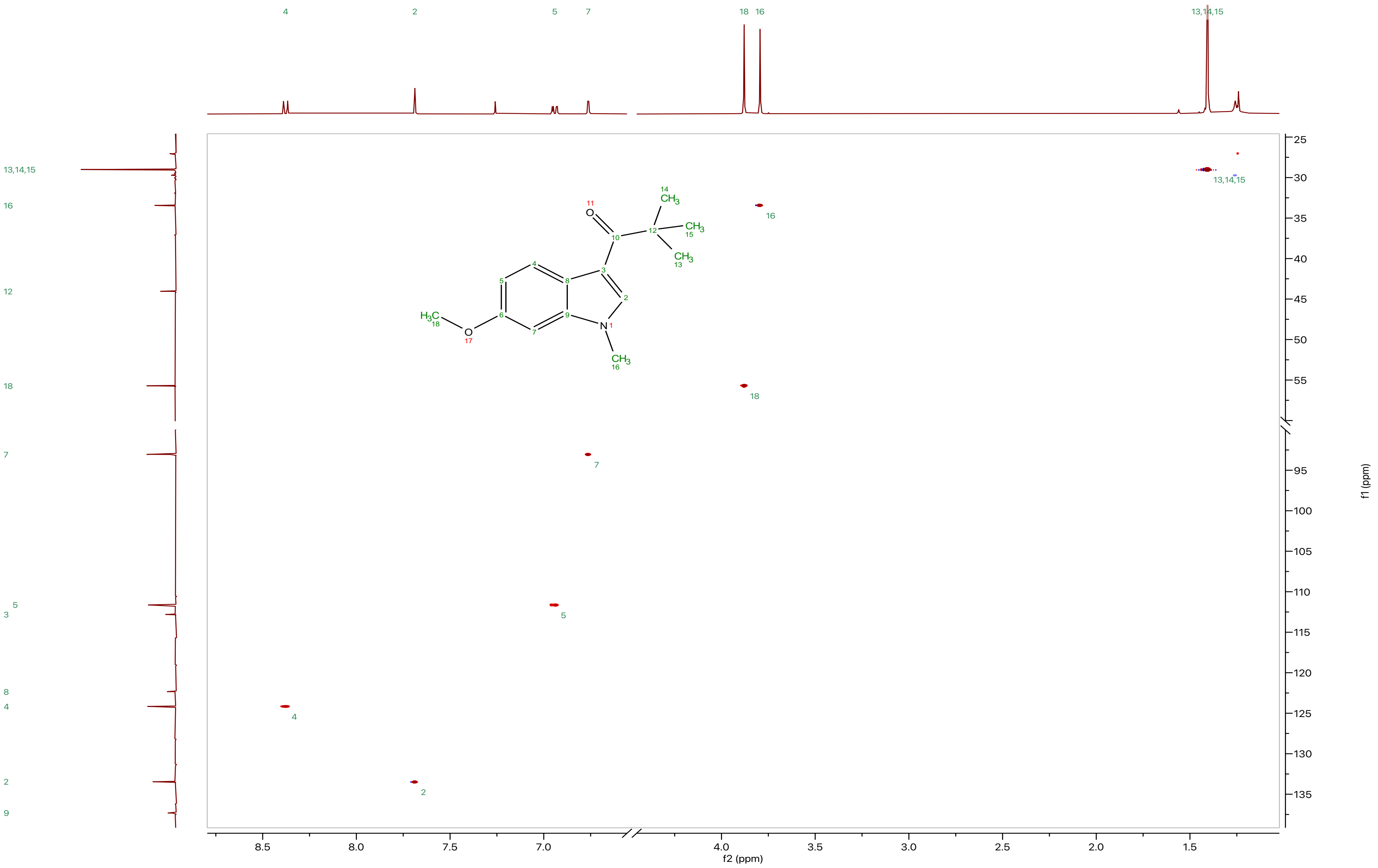

^1H-$^{13}C\{^1H\}$ HSQC NMR (400/101 MHz, $CDCl_3$) of 1d

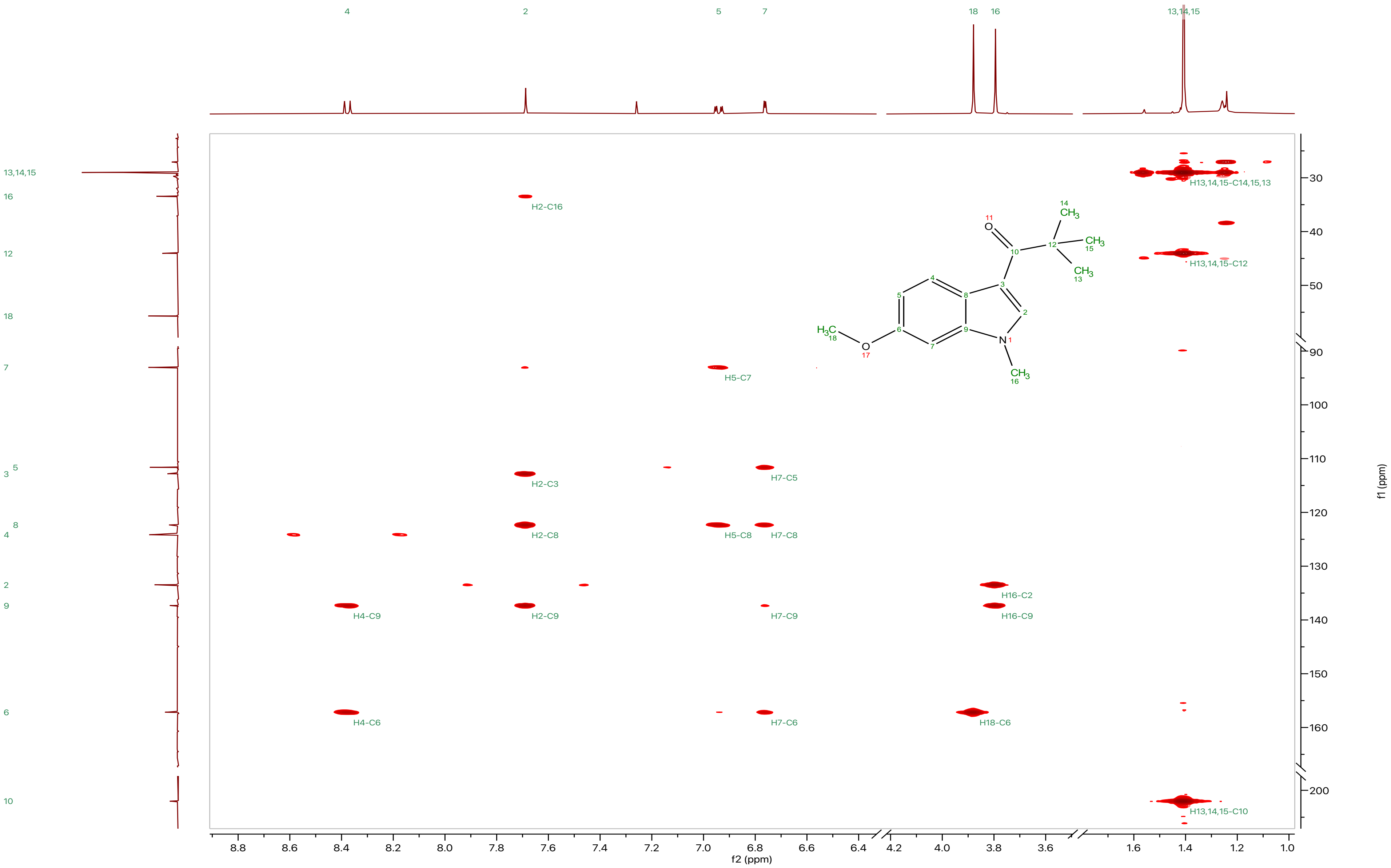

^{1}H-$^{13}C\{^{1}H\}$ HMBC NMR (400/101 MHz, $CDCl_3$) of 1d

2d

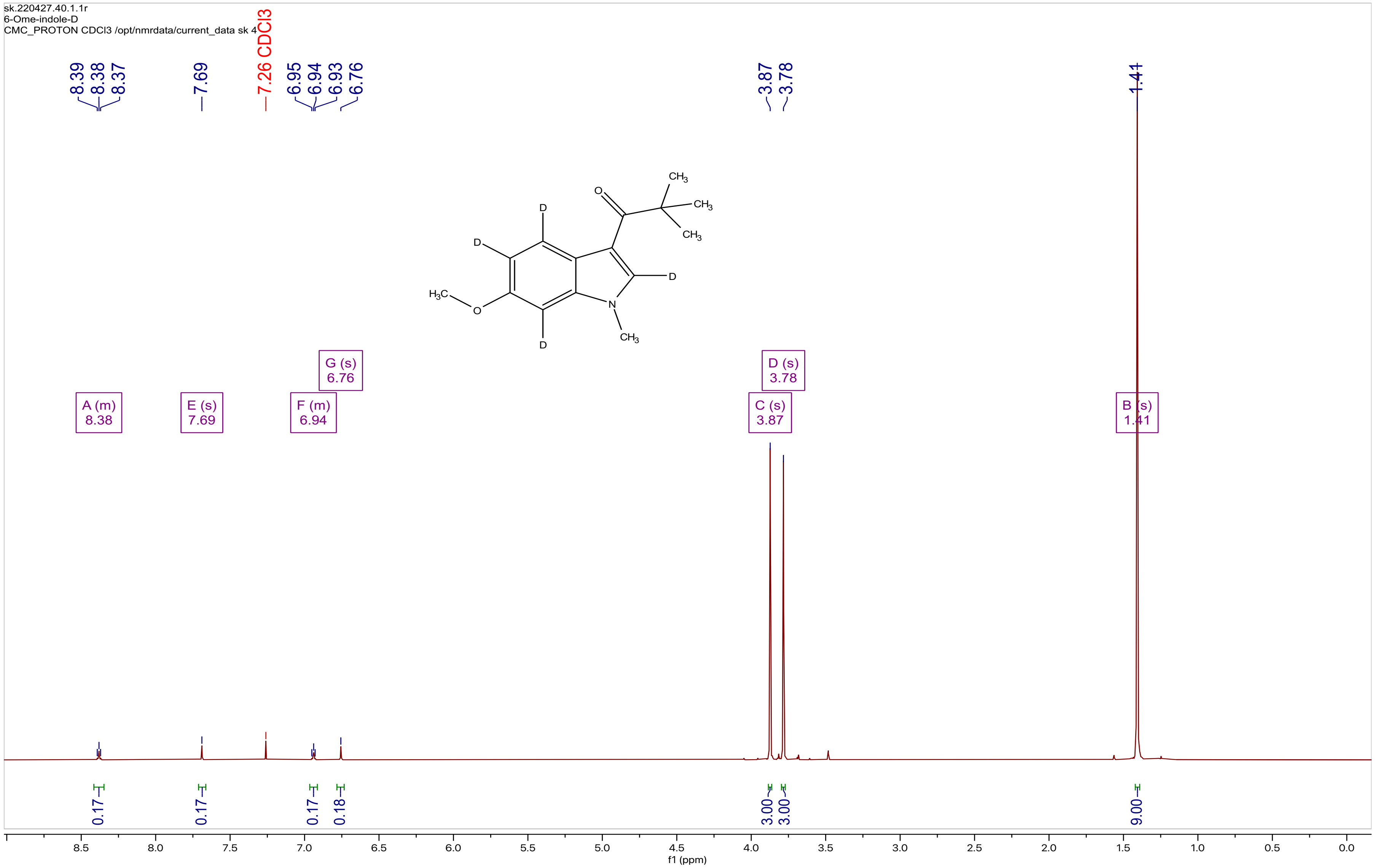

^{1}H NMR (400 MHz, $CDCl_3$) of 2d

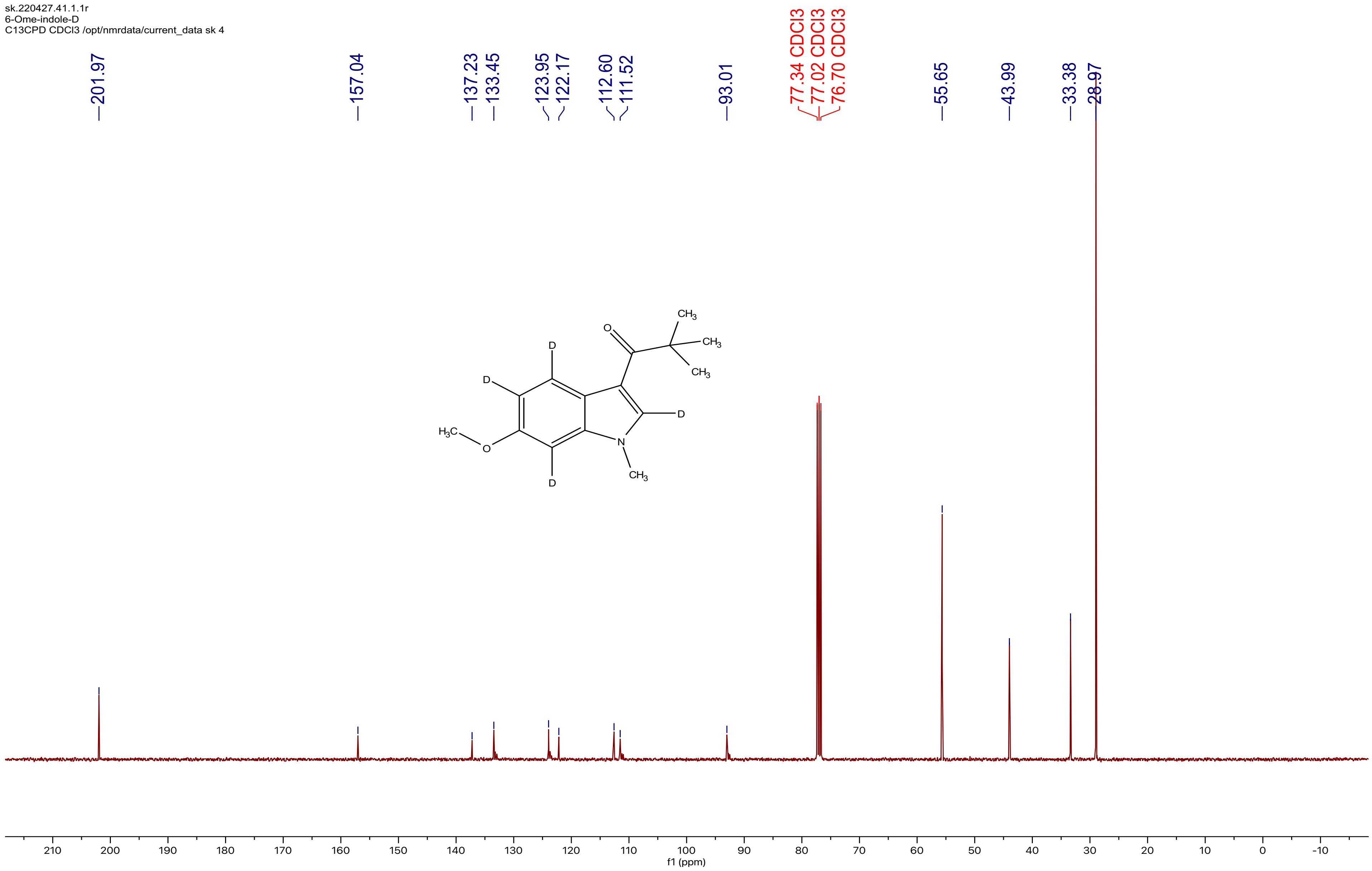

$^{13}C\{^{1}H\}$ NMR (101 MHz, $CDCl_3$) of 2d

1e

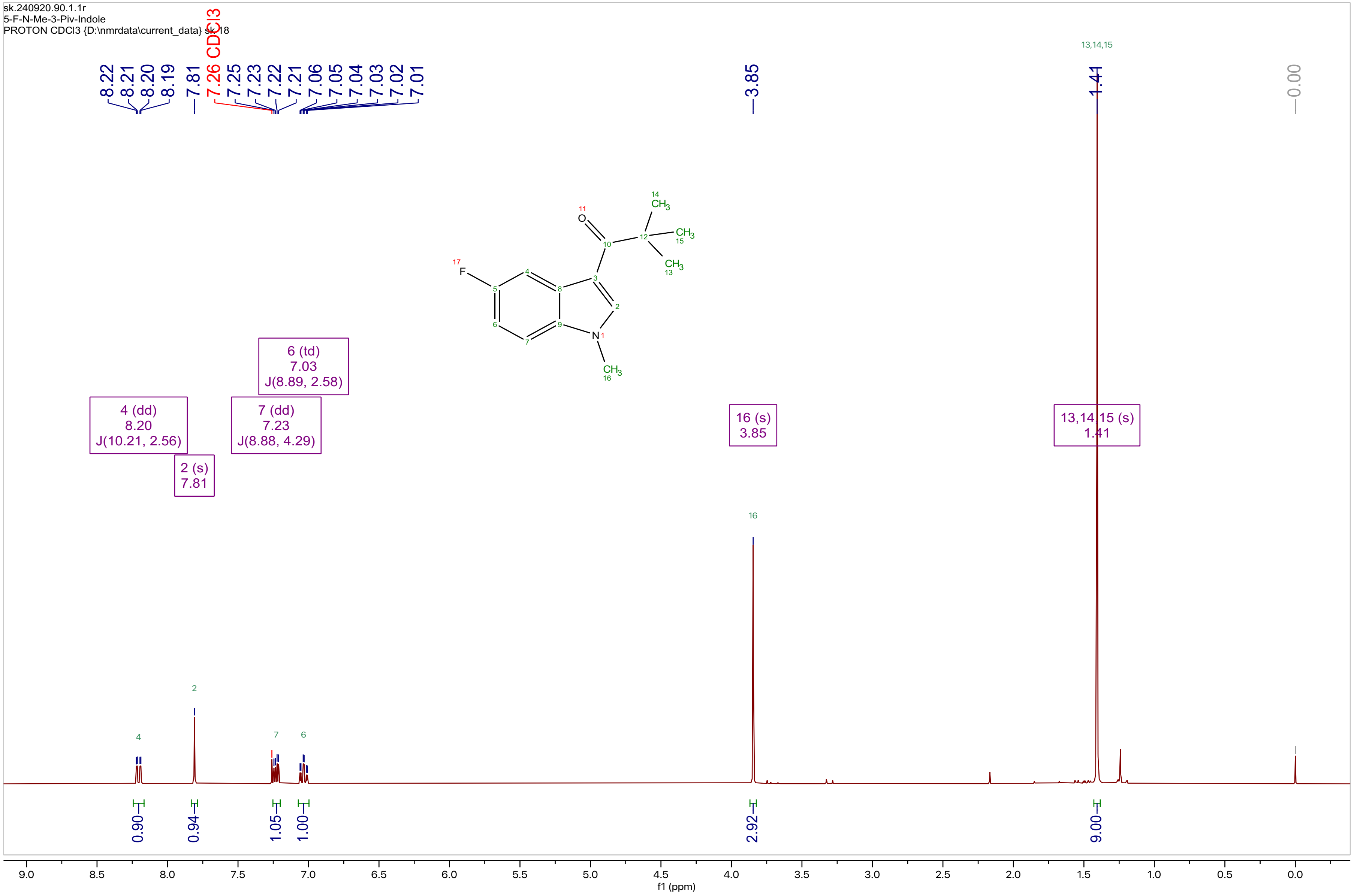

^{1}H NMR (400 MHz, $CDCl_3$) of 1e

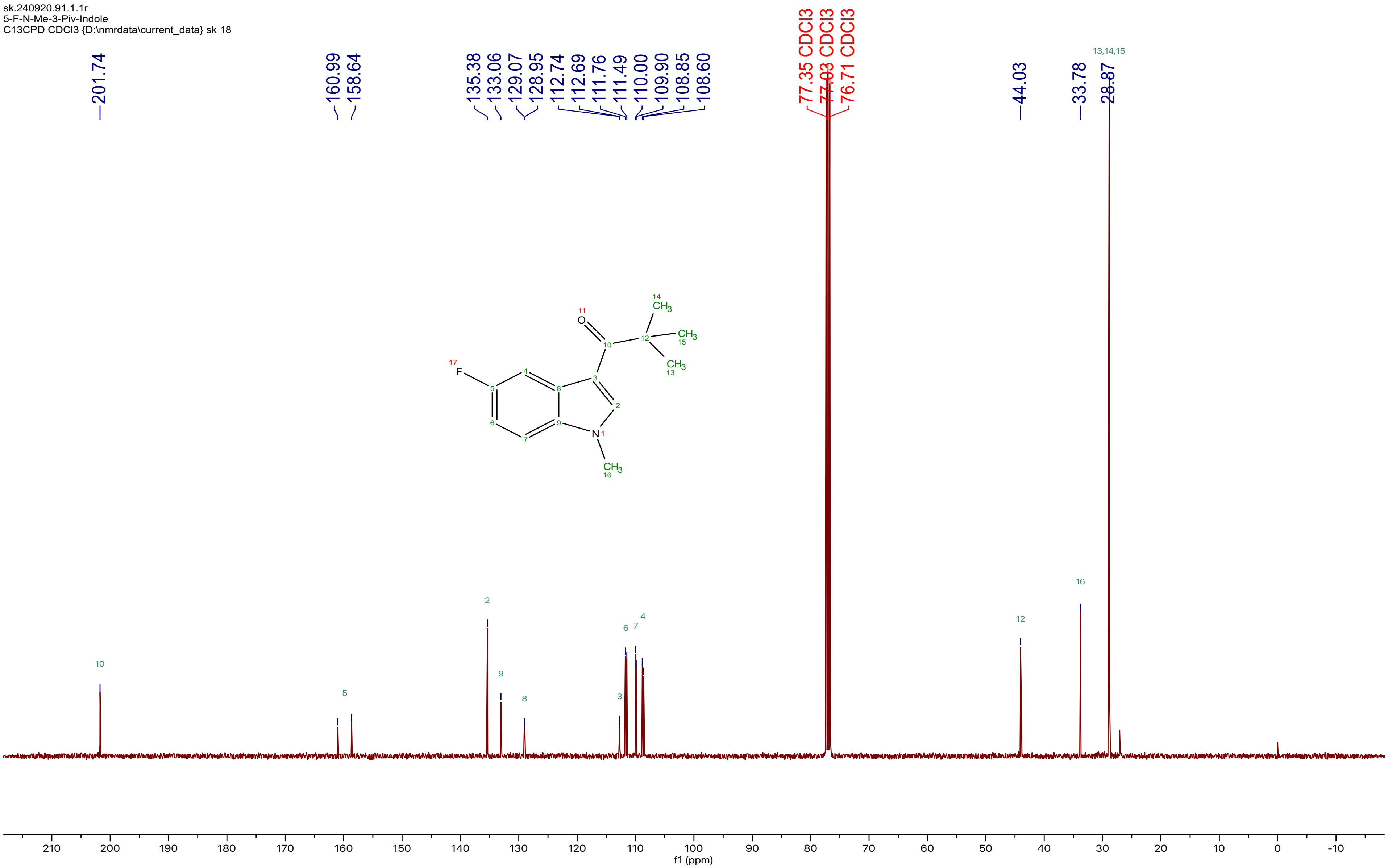

$^{13}C\{^{1}H\}$ NMR (101 MHz, $CDCl_3$) of 1e

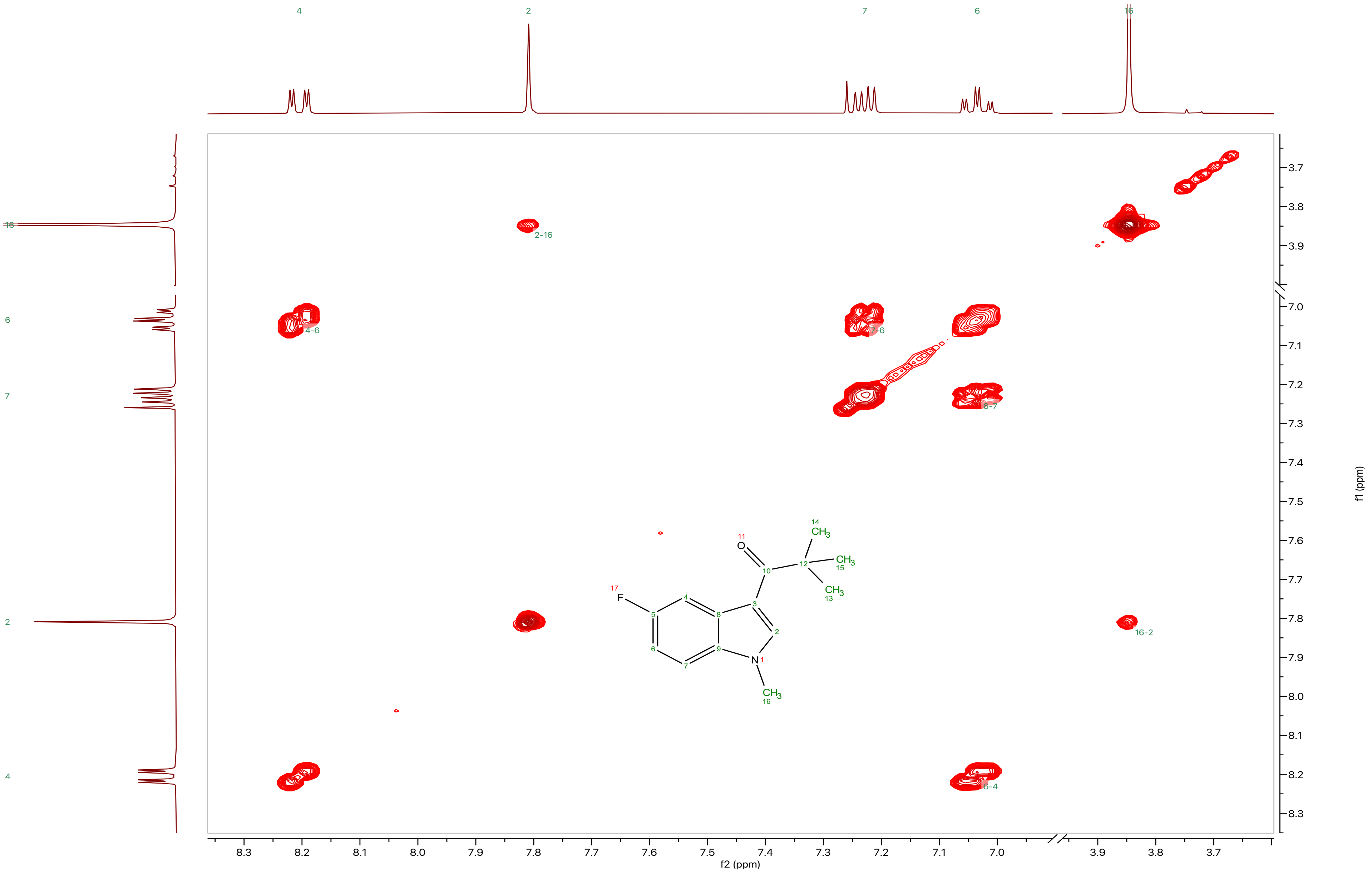

^{1}H-^{1}H COSY (400 MHz, $CDCl_3$) of 1e

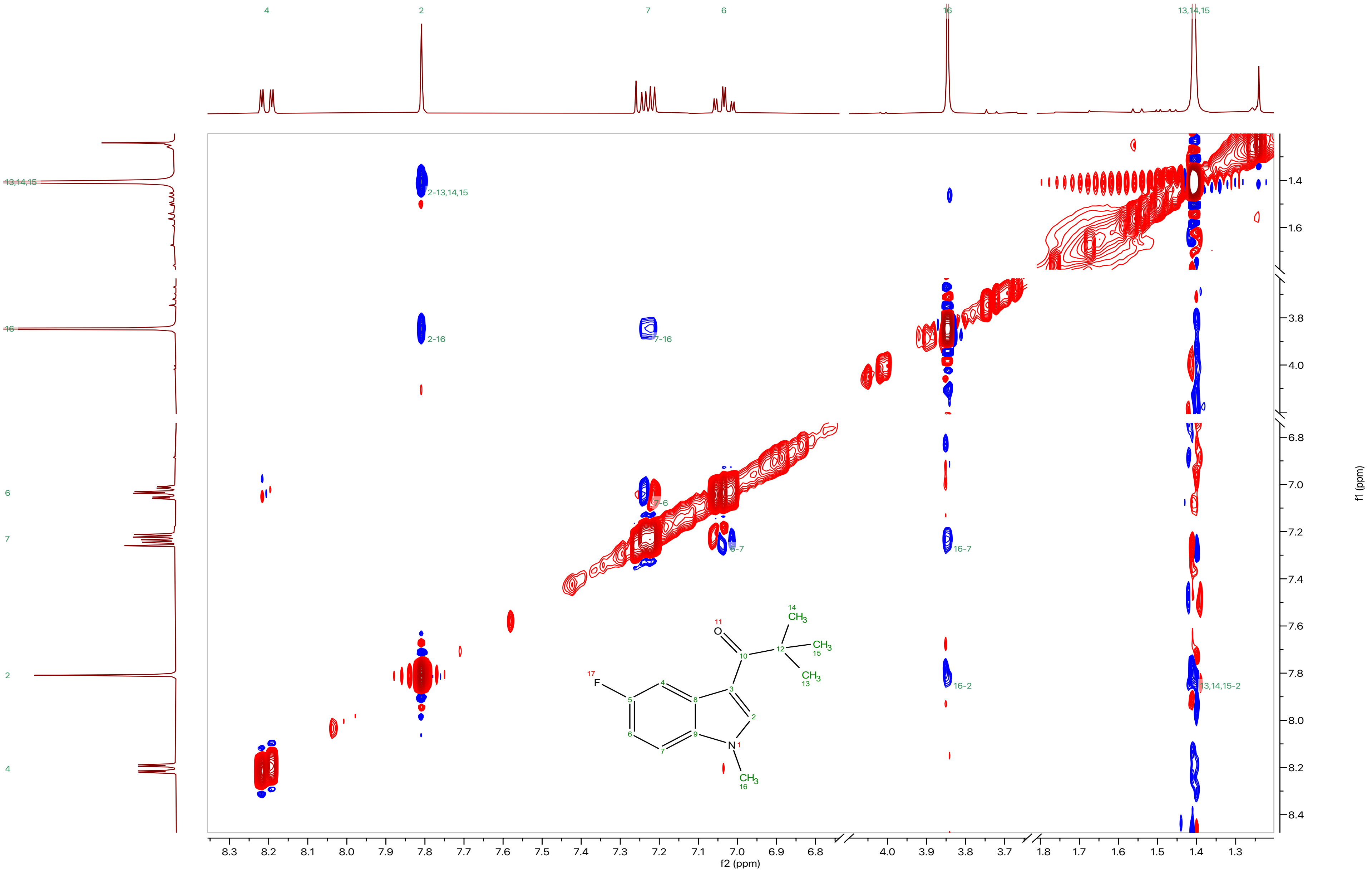

^1H-^1H NOESY (400 MHz, $CDCl_3$) of 1e

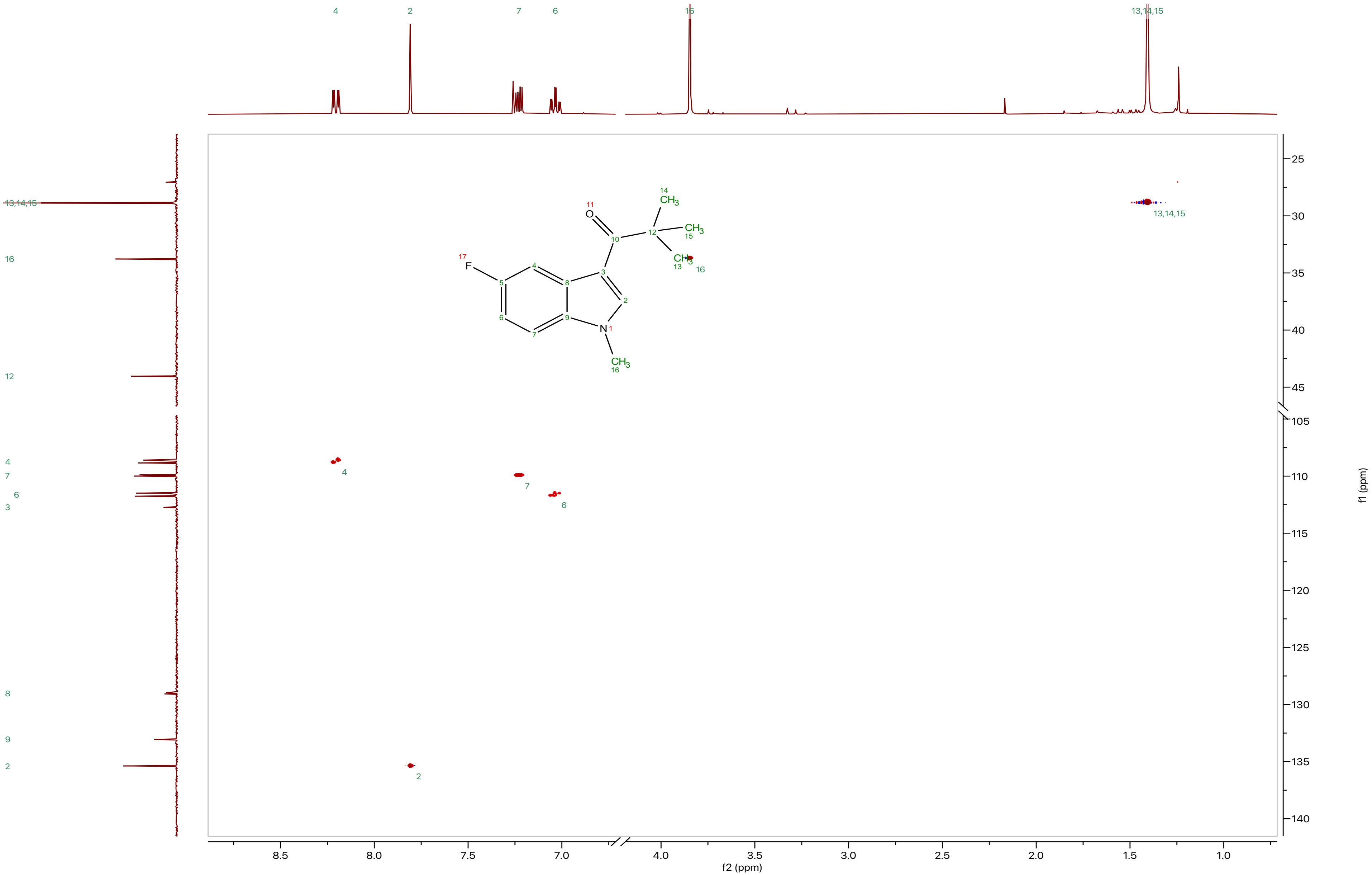

^1H-$^{13}C\{^1H\}$ HSQC NMR (400/101 MHz, $CDCl_3$) of 1e

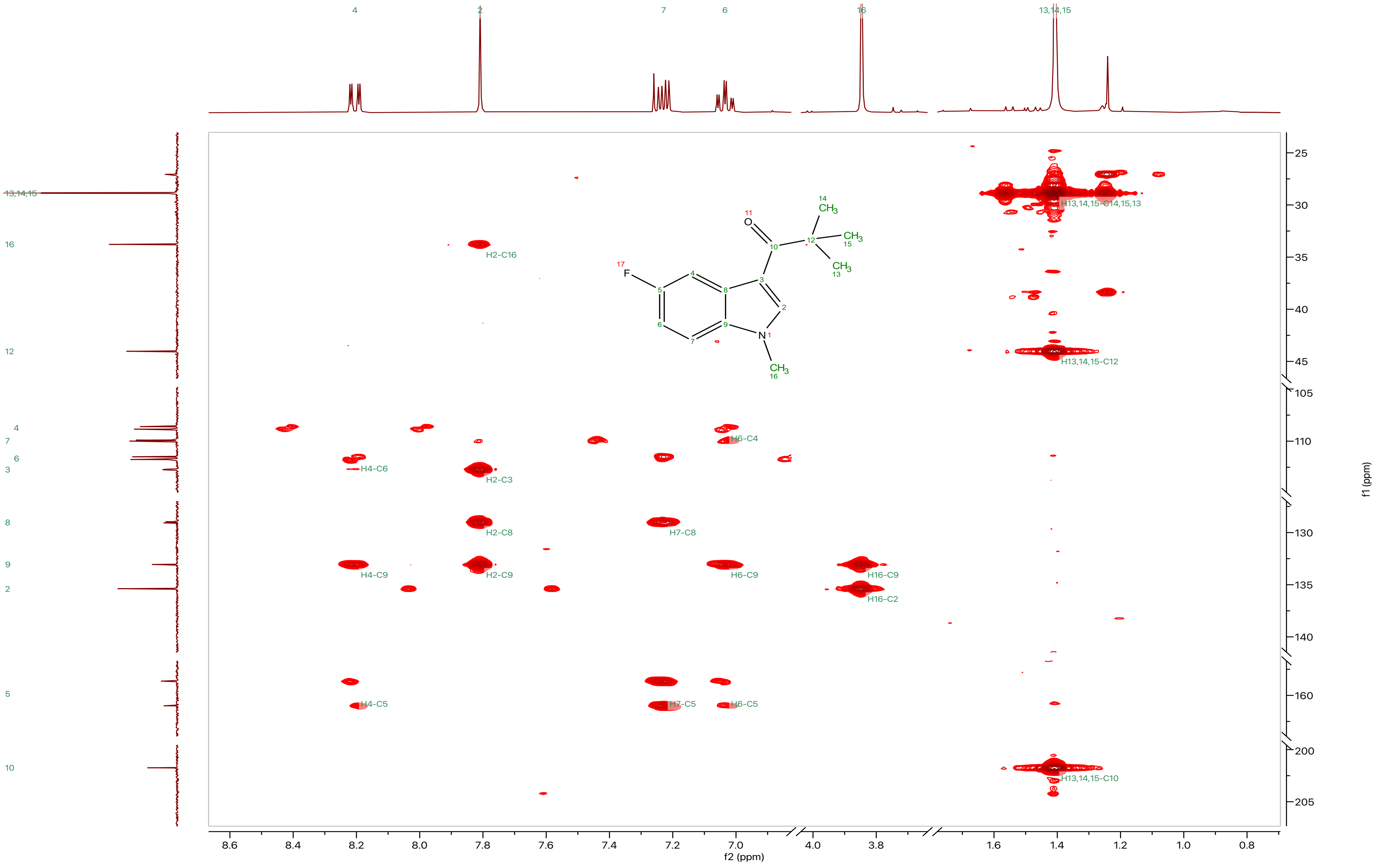

^{1}H-$^{13}C\{^{1}H\}$ HMBC NMR (400/101 MHz, $CDCl_3$) of 1e

2e

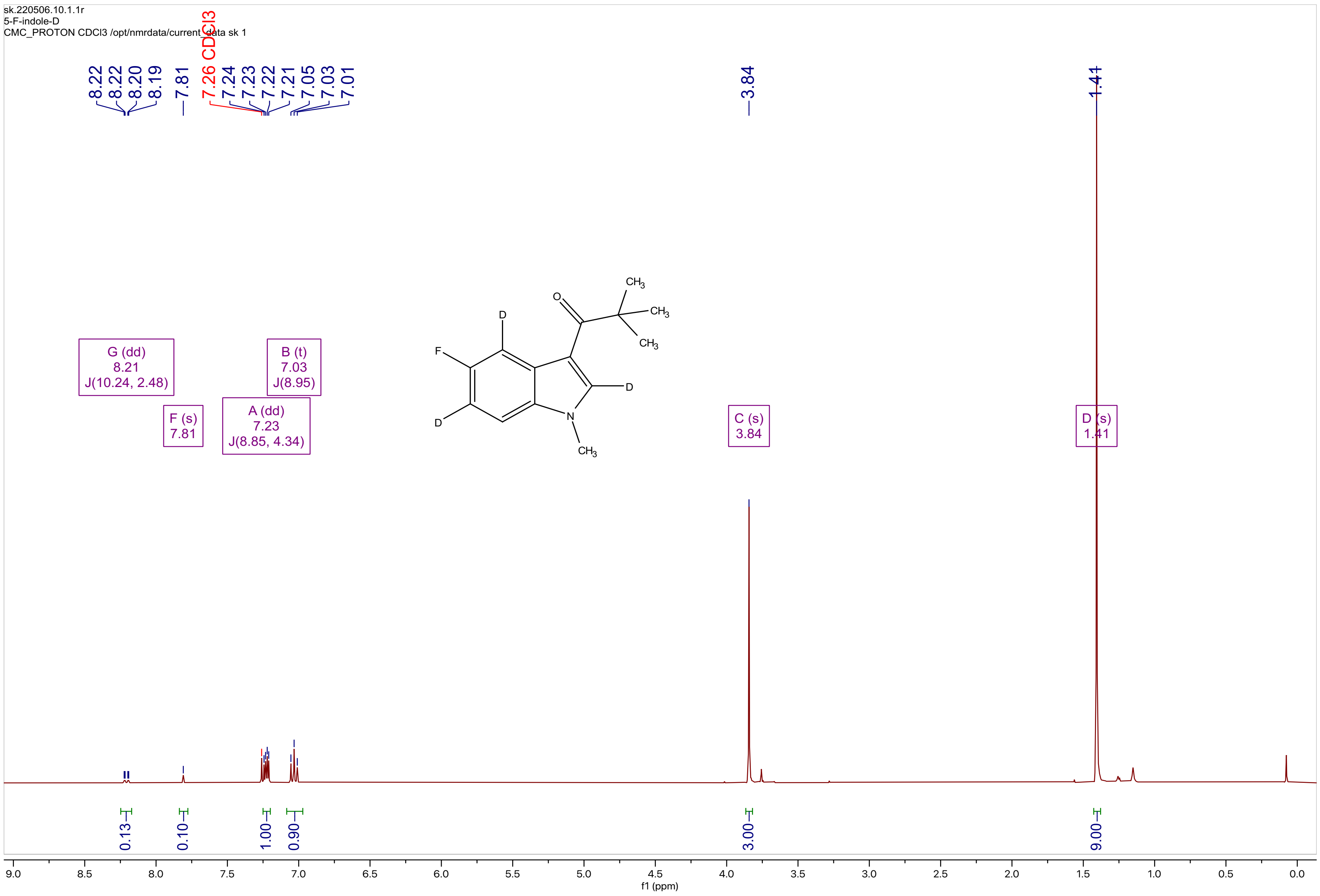

^{1}H NMR (400 MHz, CDCl$_3$) of 2e

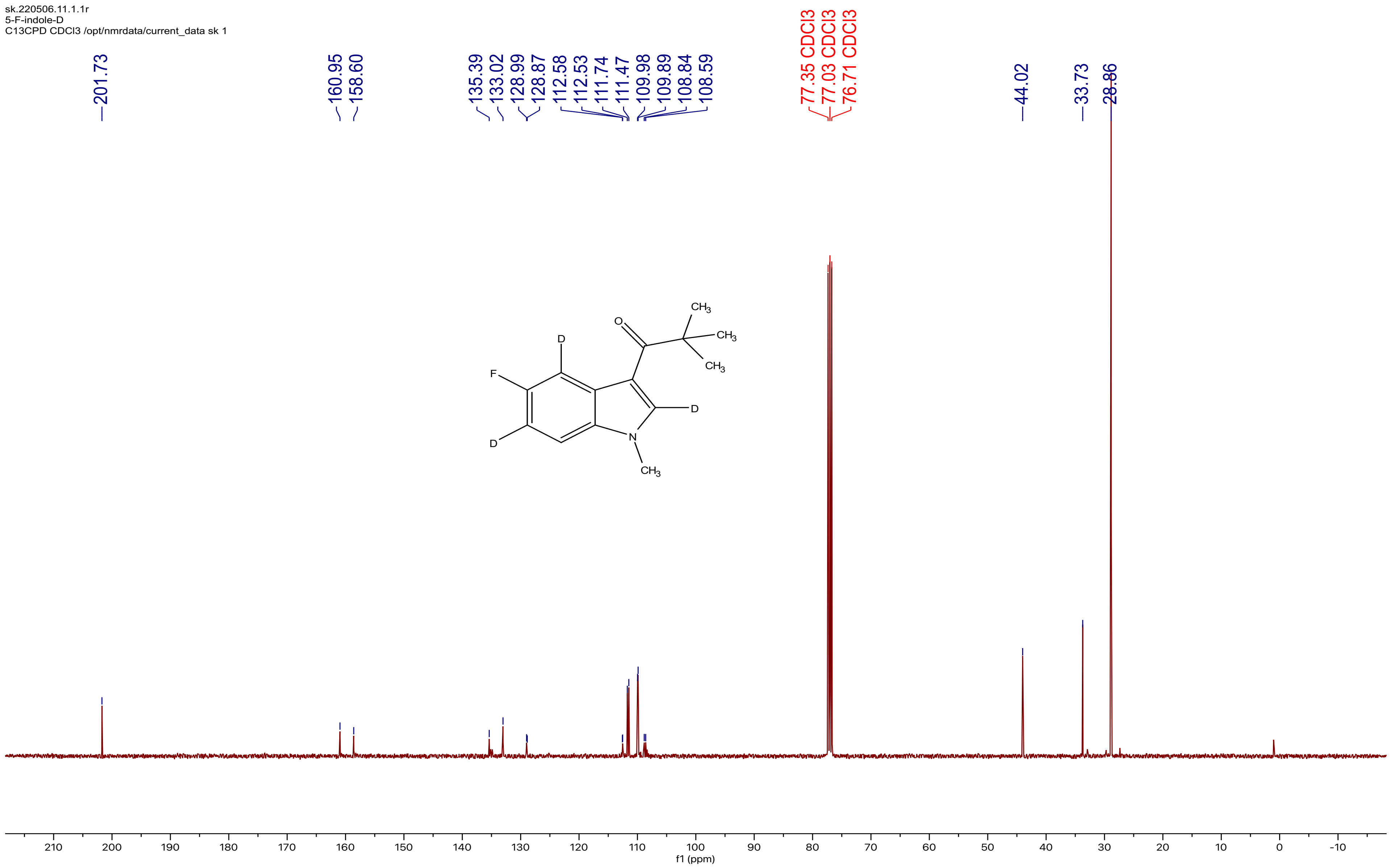

$^{13}C\{^{1}H\}$ NMR (101 MHz, $CDCl_3$) of 2e

1f

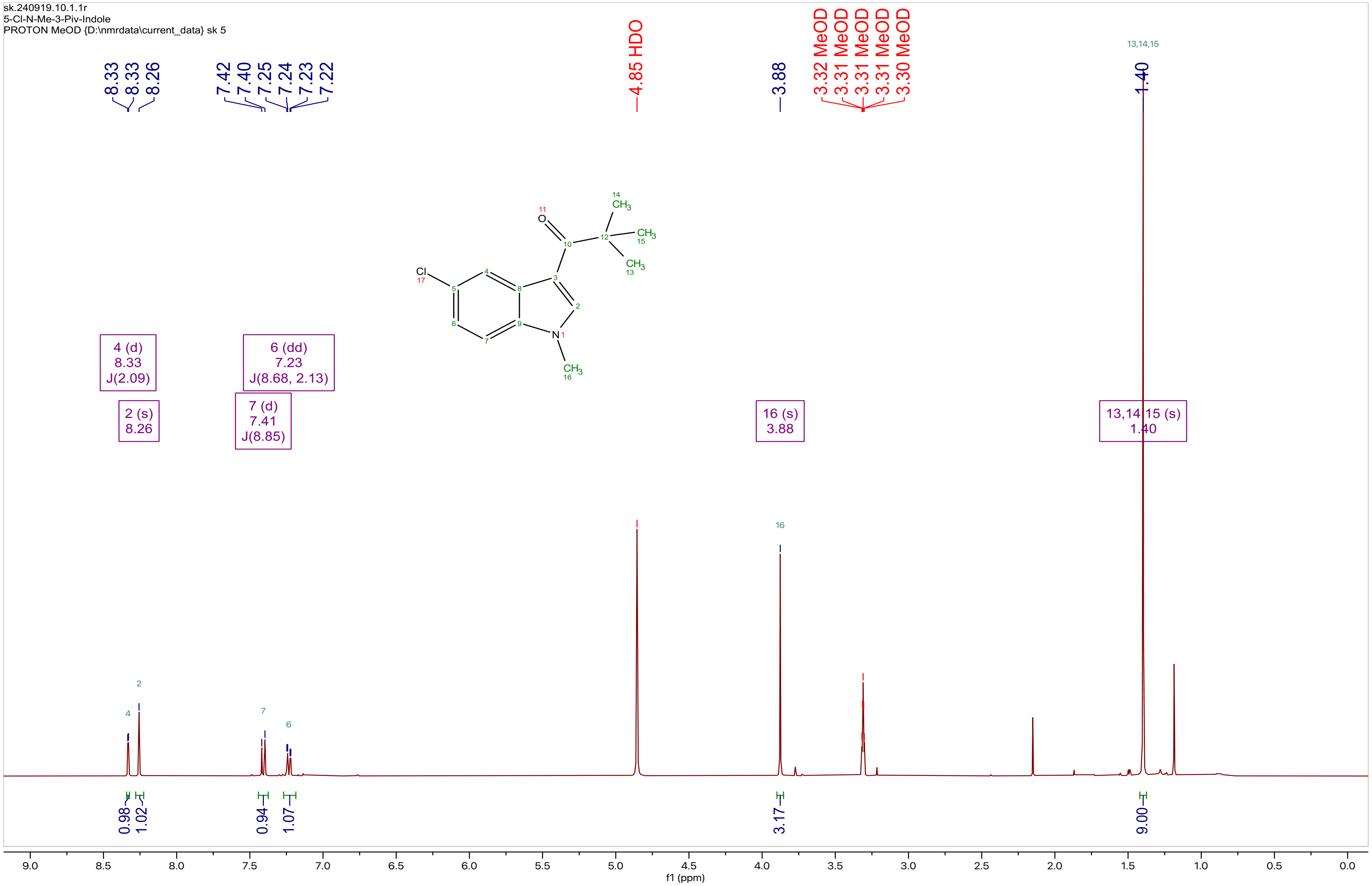

^{1}H NMR (400 MHz, MeOD) of 1f

sk.240919.11.1.1r
5-Cl-N-Me-3-Piv-Indole
C13CPD MeOD {D:\nmrdata\current_data} sk 5

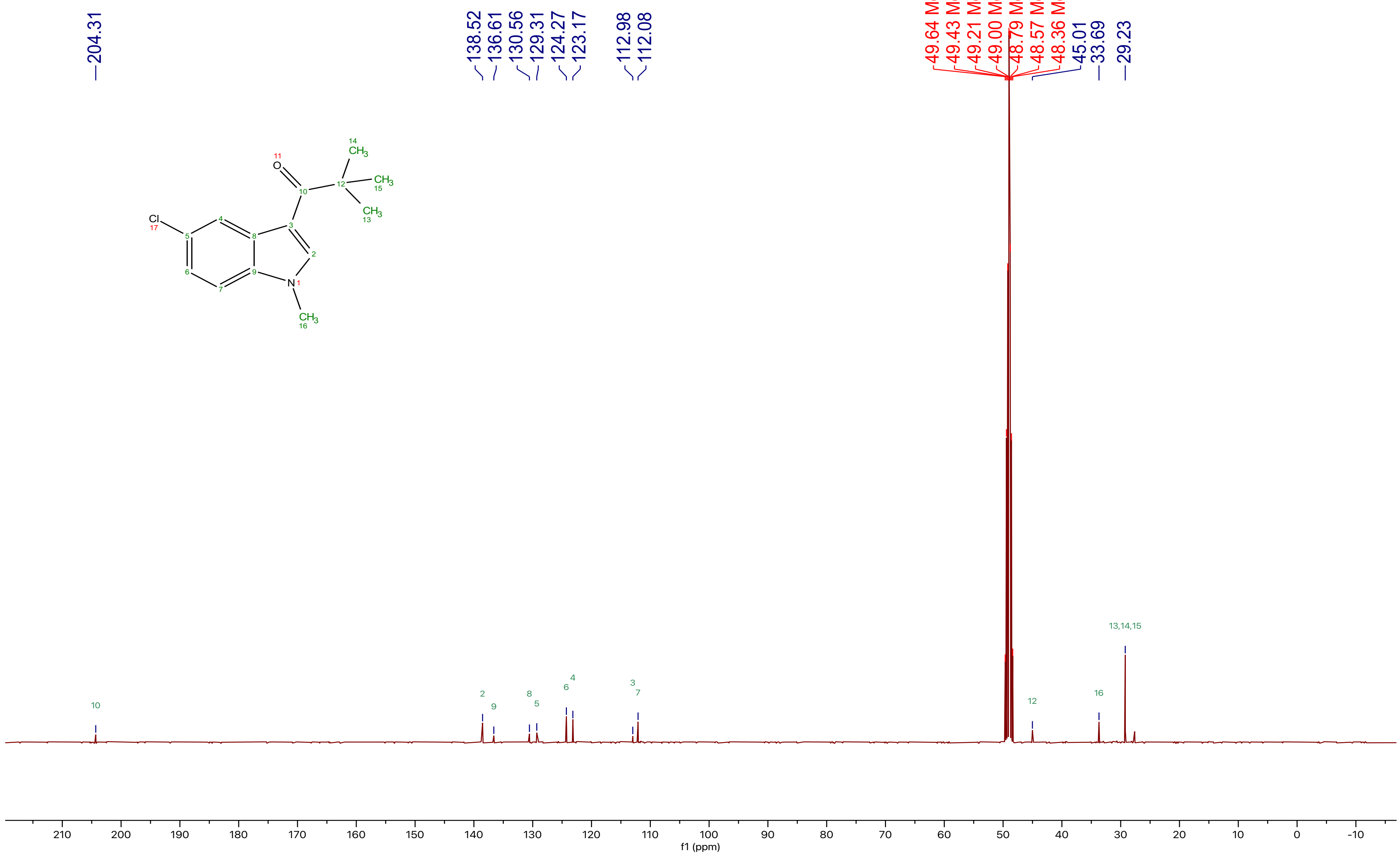

$^{13}C\{^{1}H\}$ NMR (101 MHz, MeOD) of 1f

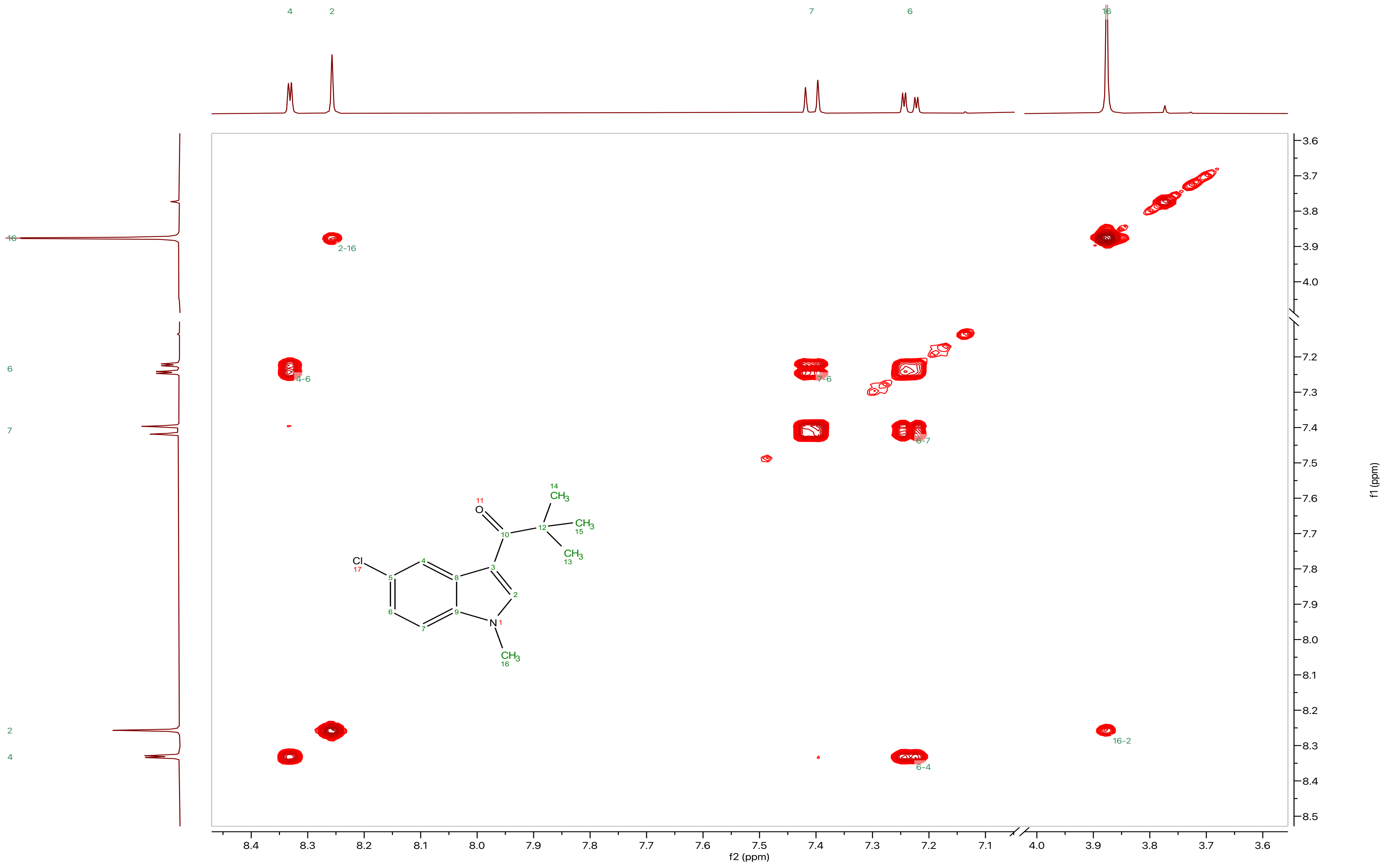

^{1}H-^{1}H COSY (400 MHz, MeOD) of 1f

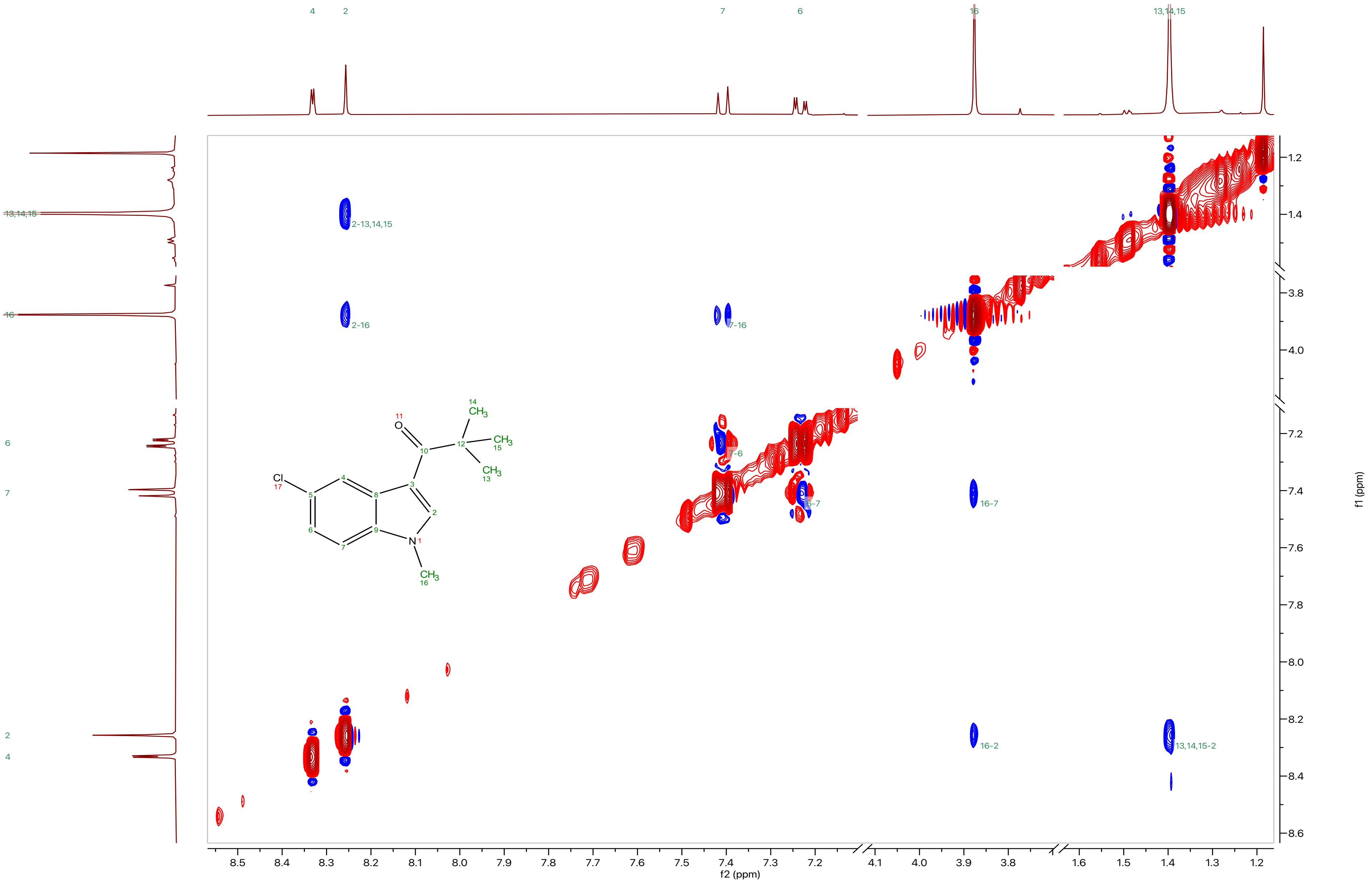

^{1}H-^{1}H NOESY (400 MHz, MeOD) of 1f

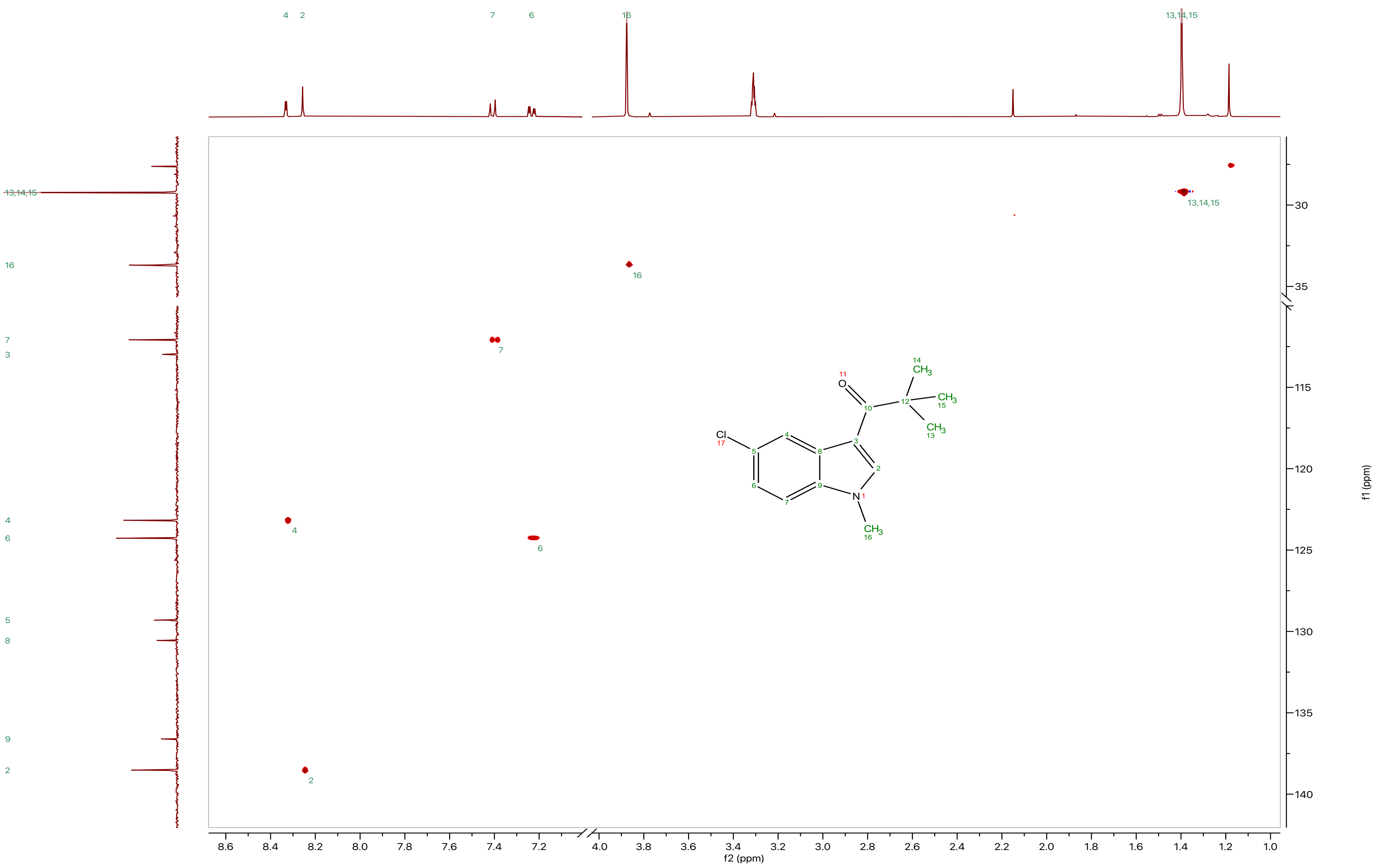

^{1}H-$^{13}C\{^{1}H\}$ HSQC NMR (400/101 MHz, MeOD) of 1f

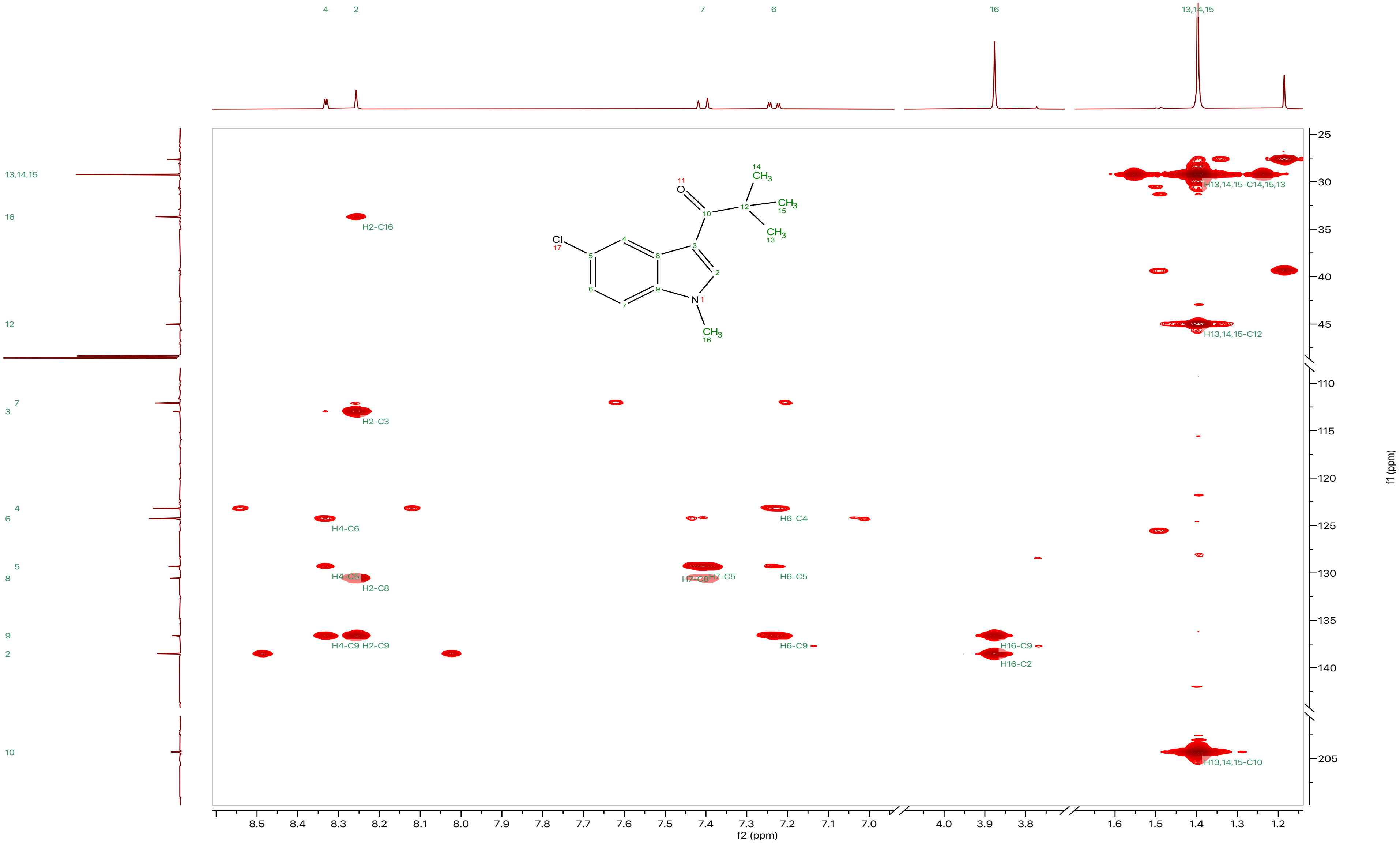

^{1}H-$^{13}C\{^{1}H\}$ HMBC NMR (400/101 MHz, MeOD) of 1f

2f

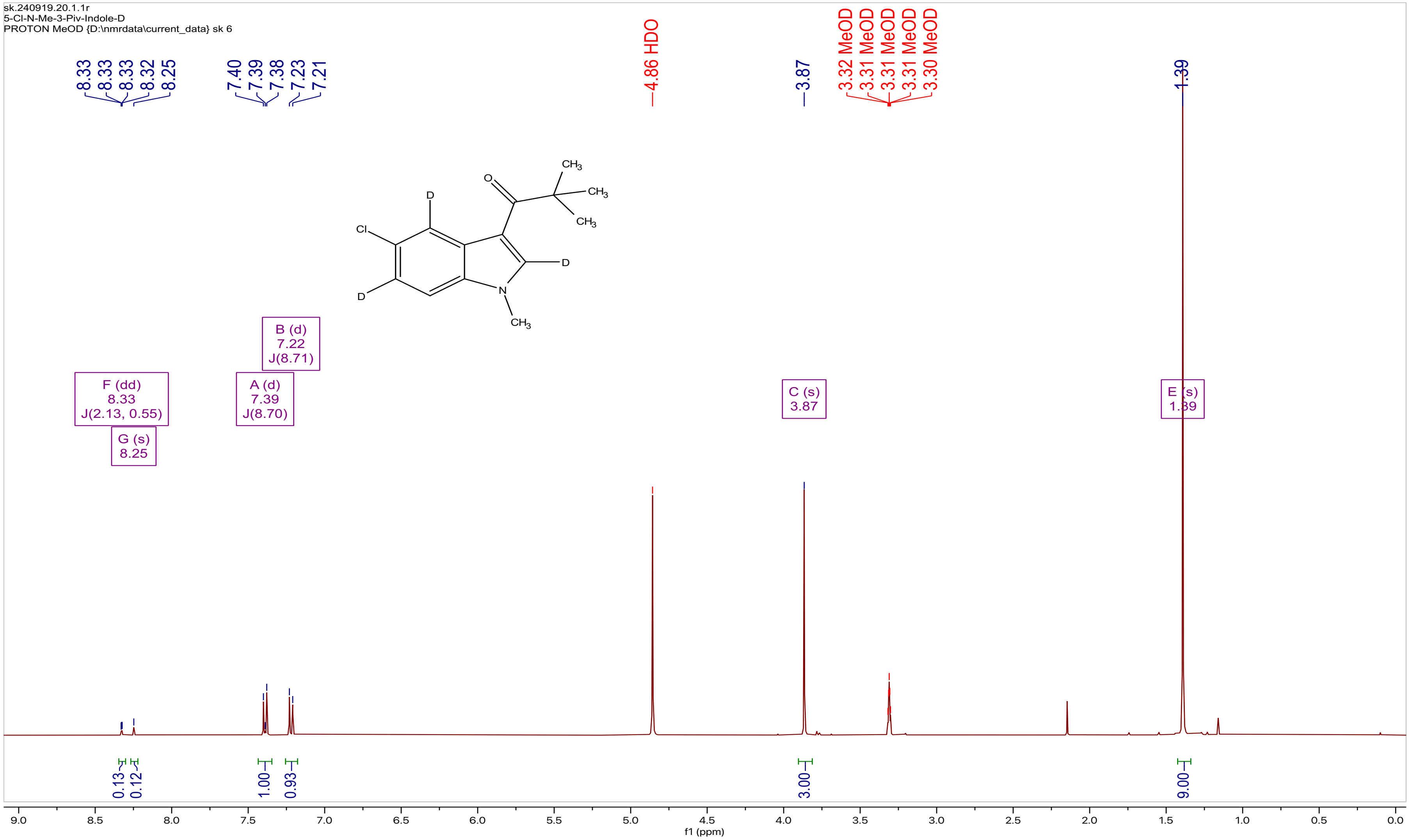

^{1}H NMR (400 MHz, MeOD) of 2f

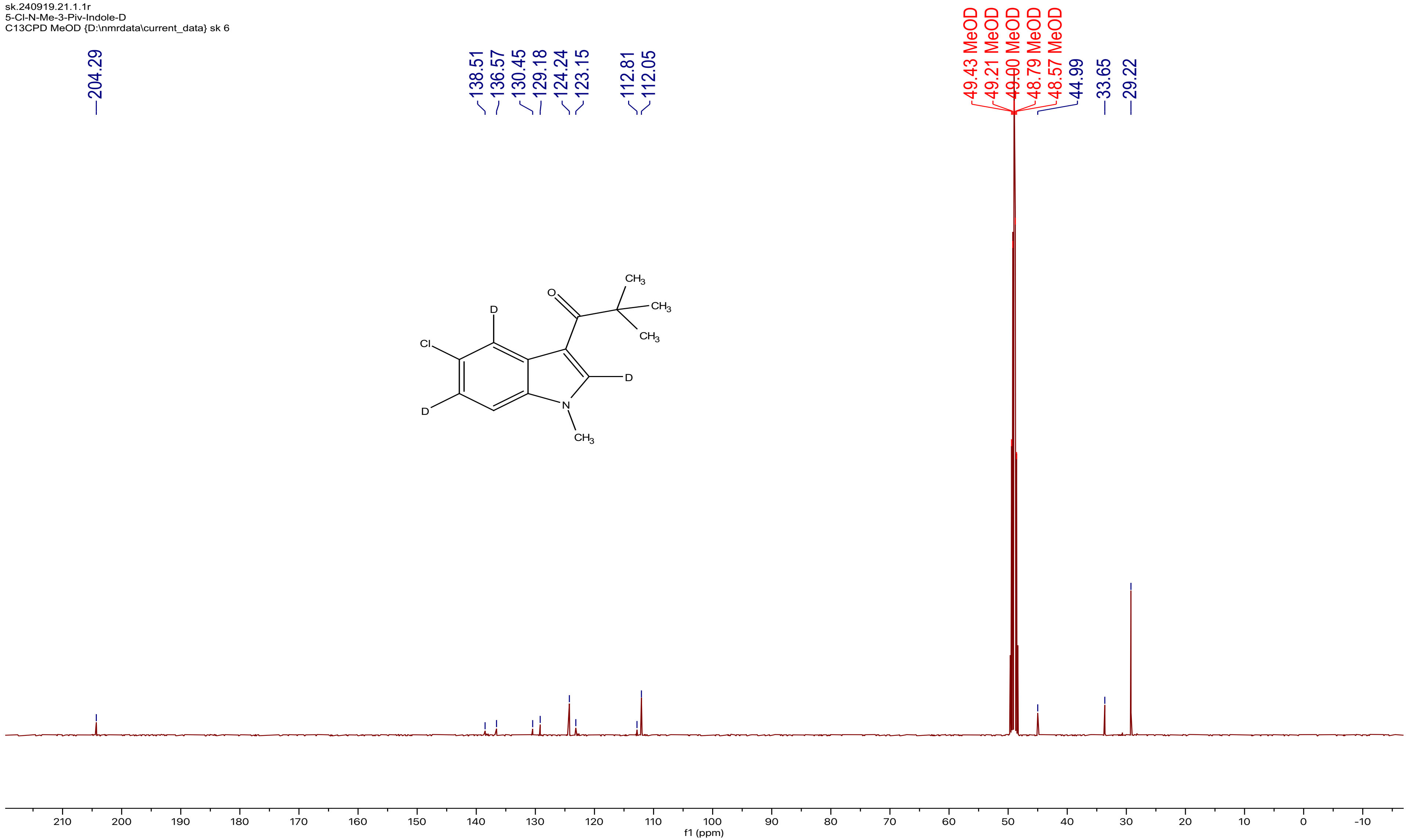

$^{13}C\{^{1}H\}$ NMR (101 MHz, MeOD) of 2f

1g

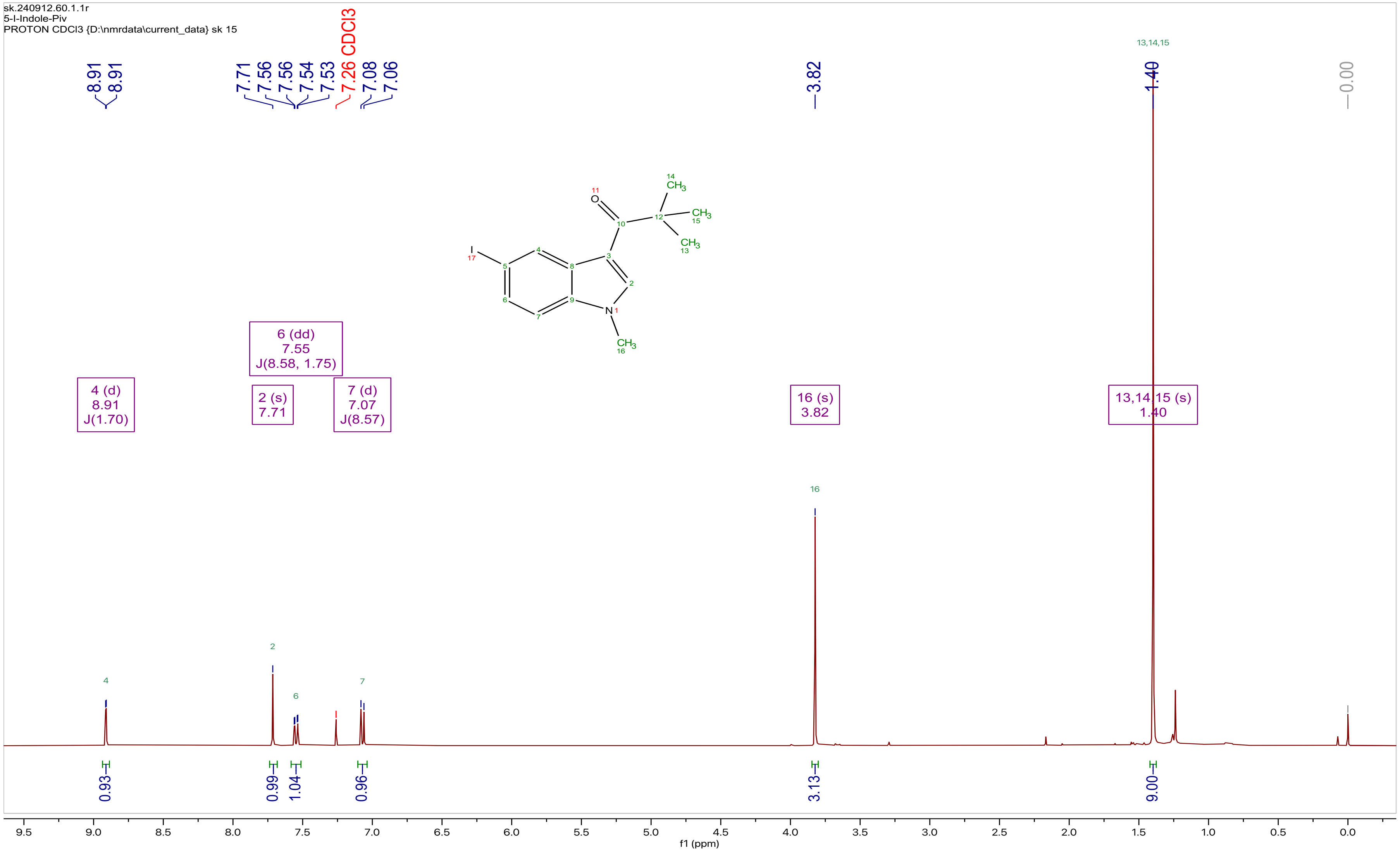

¹H NMR (400 MHz, MeOD) of 1g

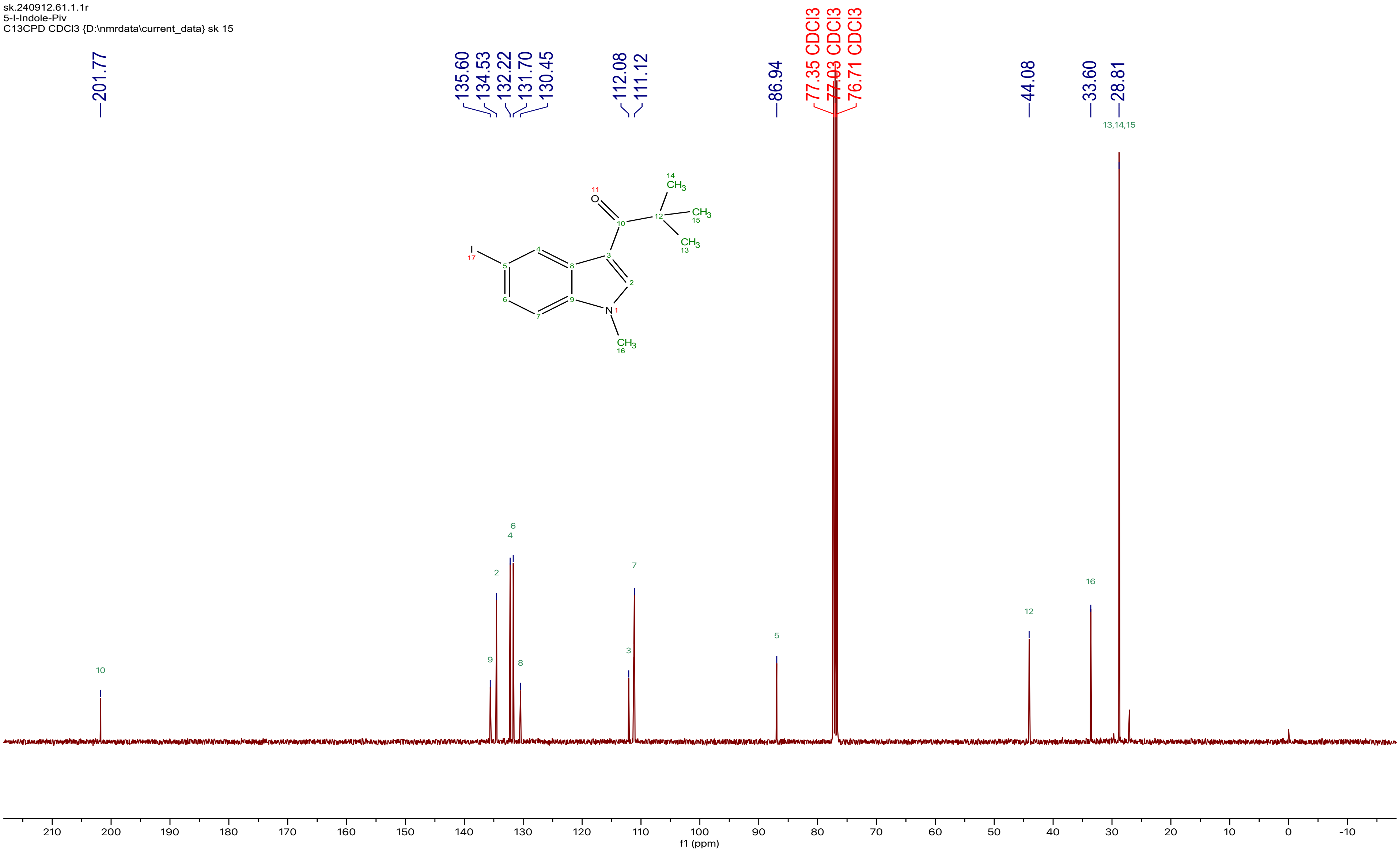

$^{13}C\{^{1}H\}$ NMR (101 MHz, MeOD) of 1g

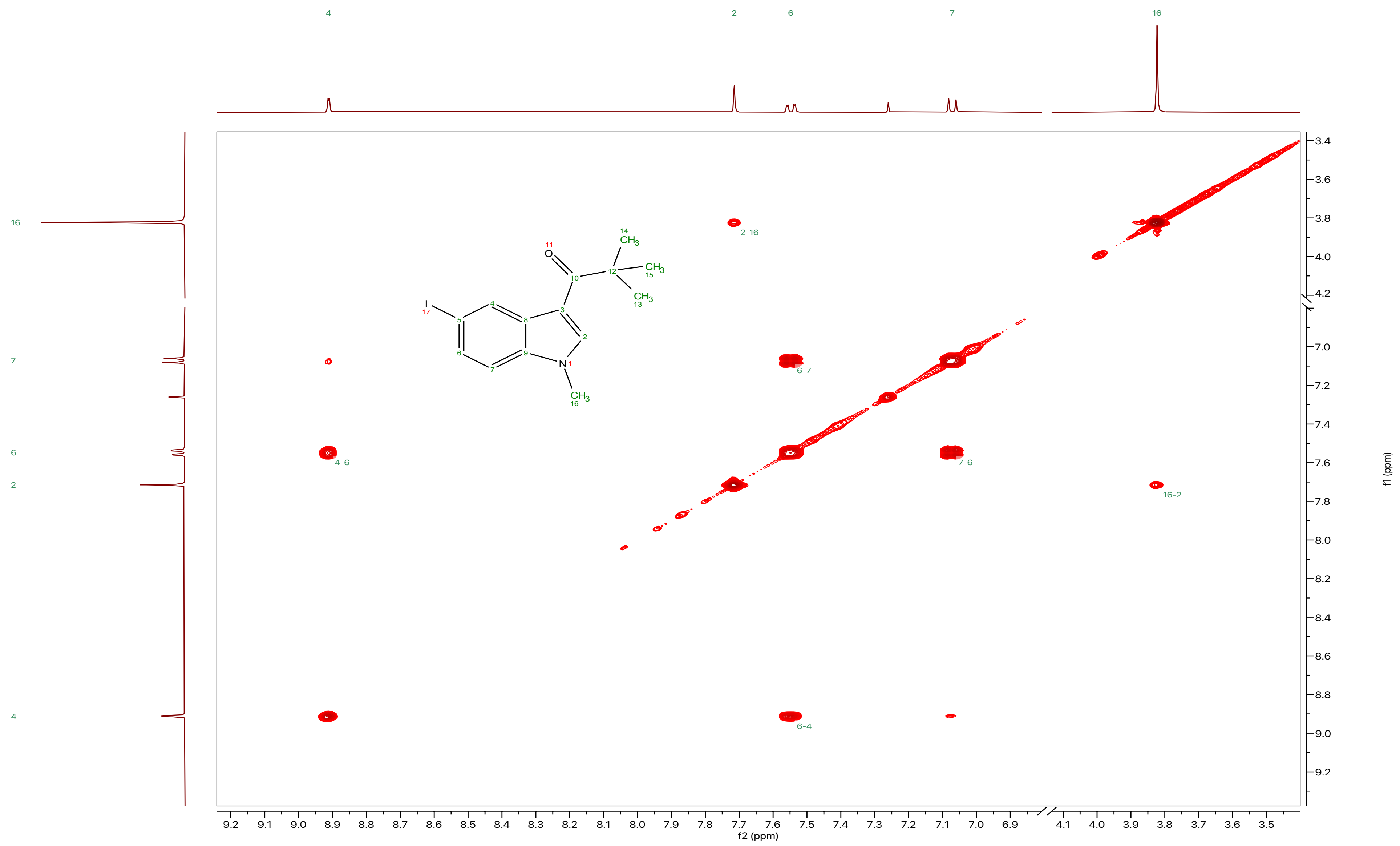

^{1}H-^{1}H COSY (400 MHz, MeOD) of 1g

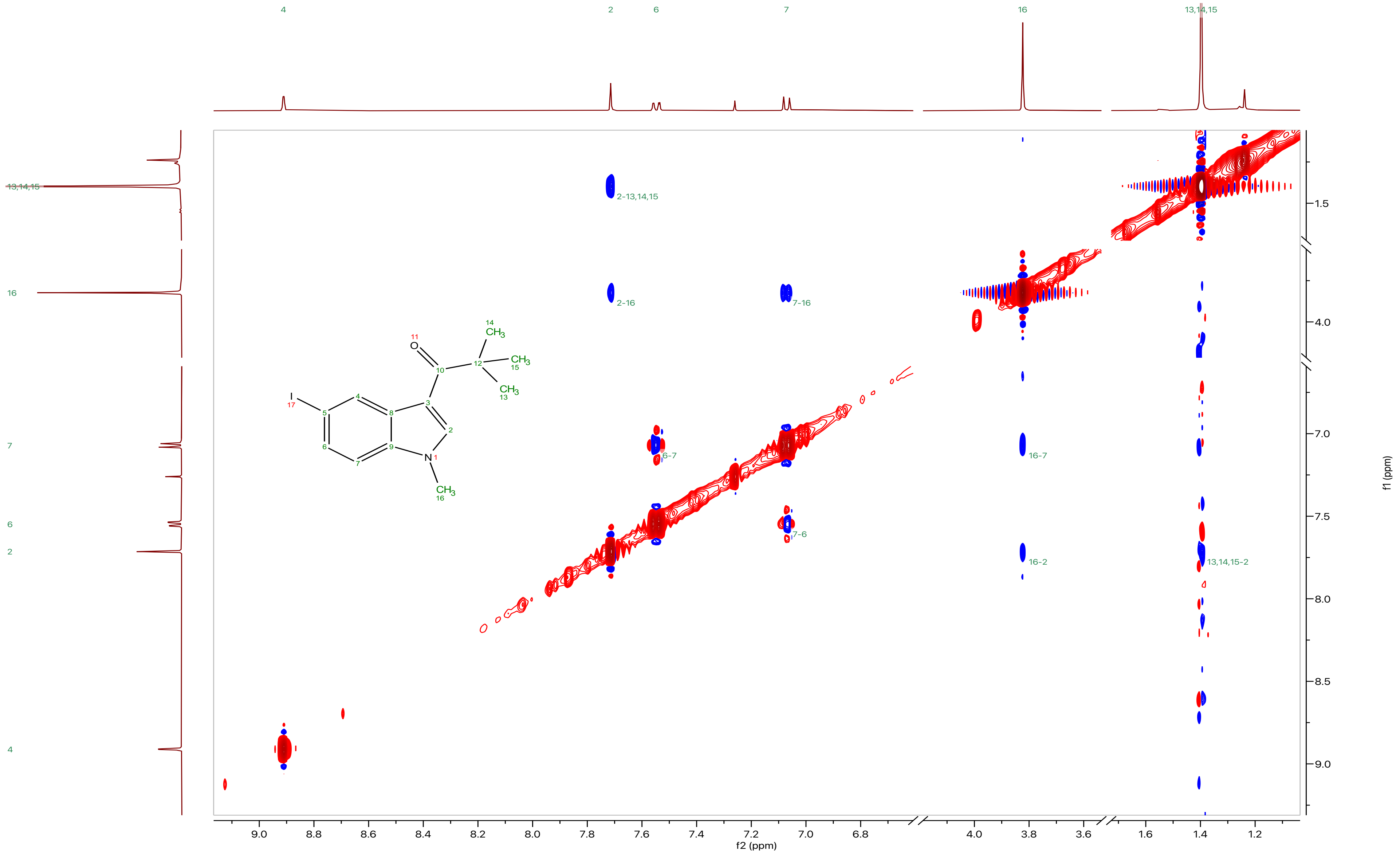

^{1}H-^{1}H NOESY (400 MHz, MeOD) of 1g

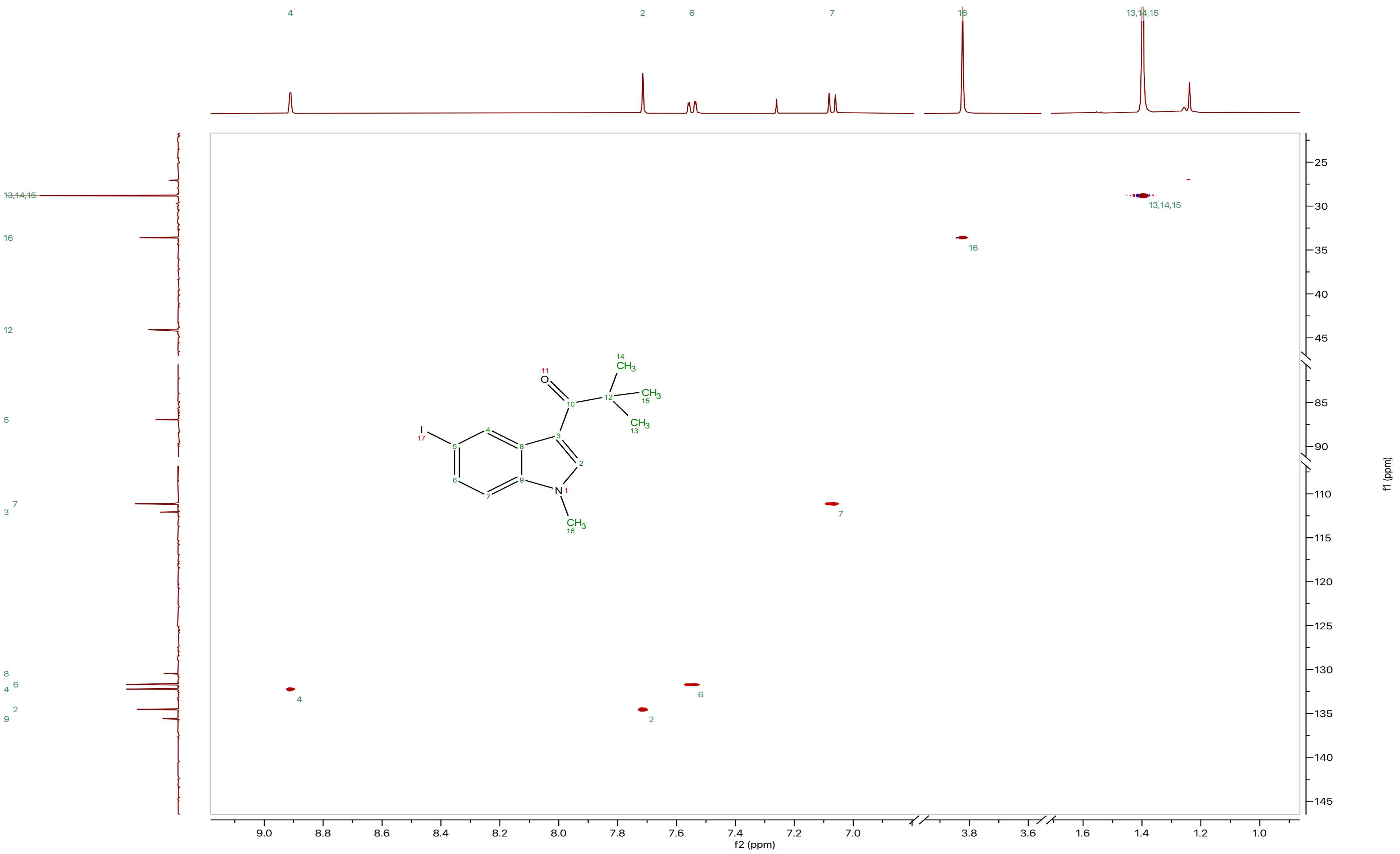

^{1}H-^{13}C{^{1}H} HSQC NMR (400/101 MHz, MeOD) of 1g

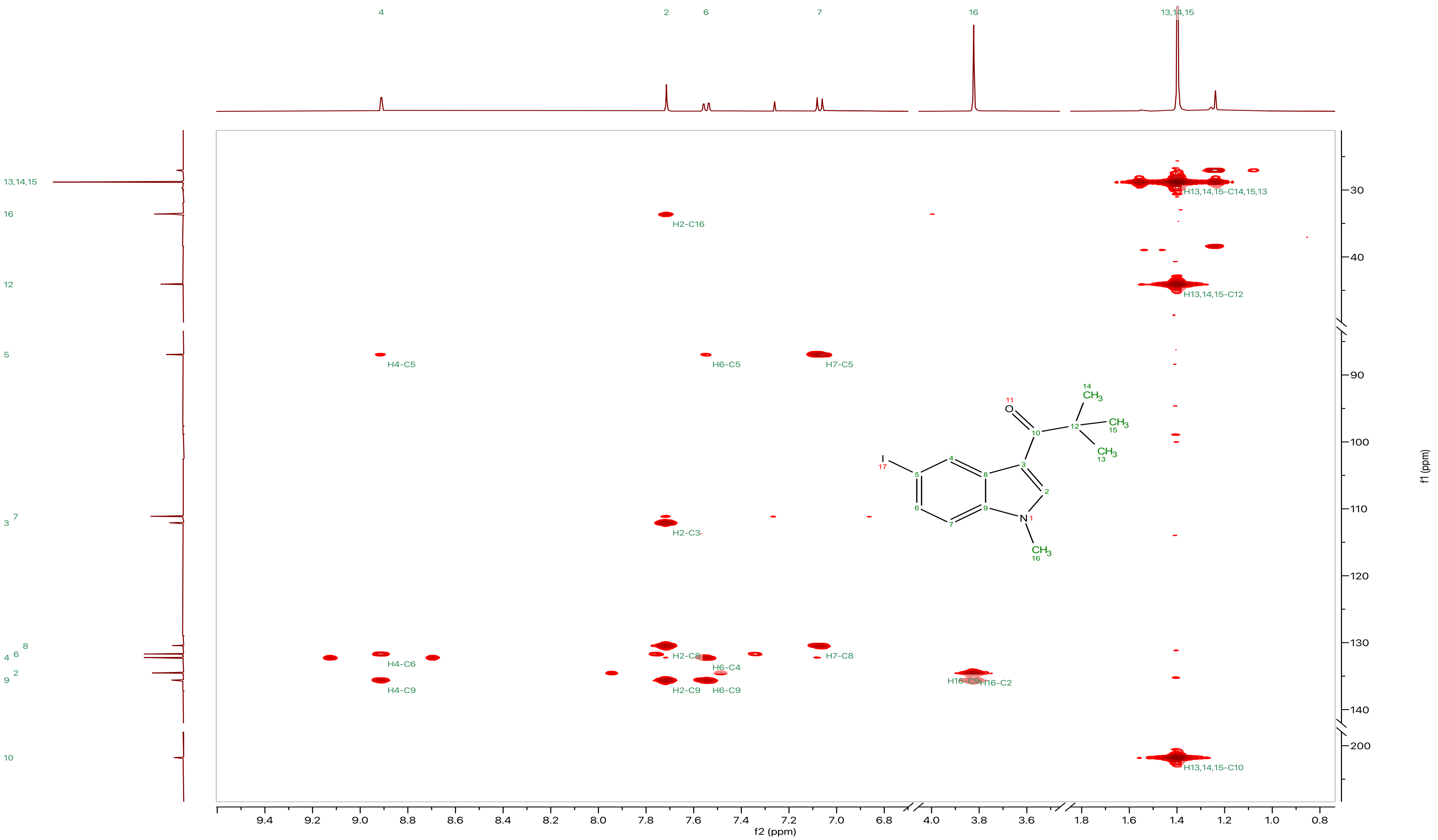

^{1}H-$^{13}C\{^{1}H\}$ HMBC NMR (400/101 MHz, MeOD) of 1g

2g

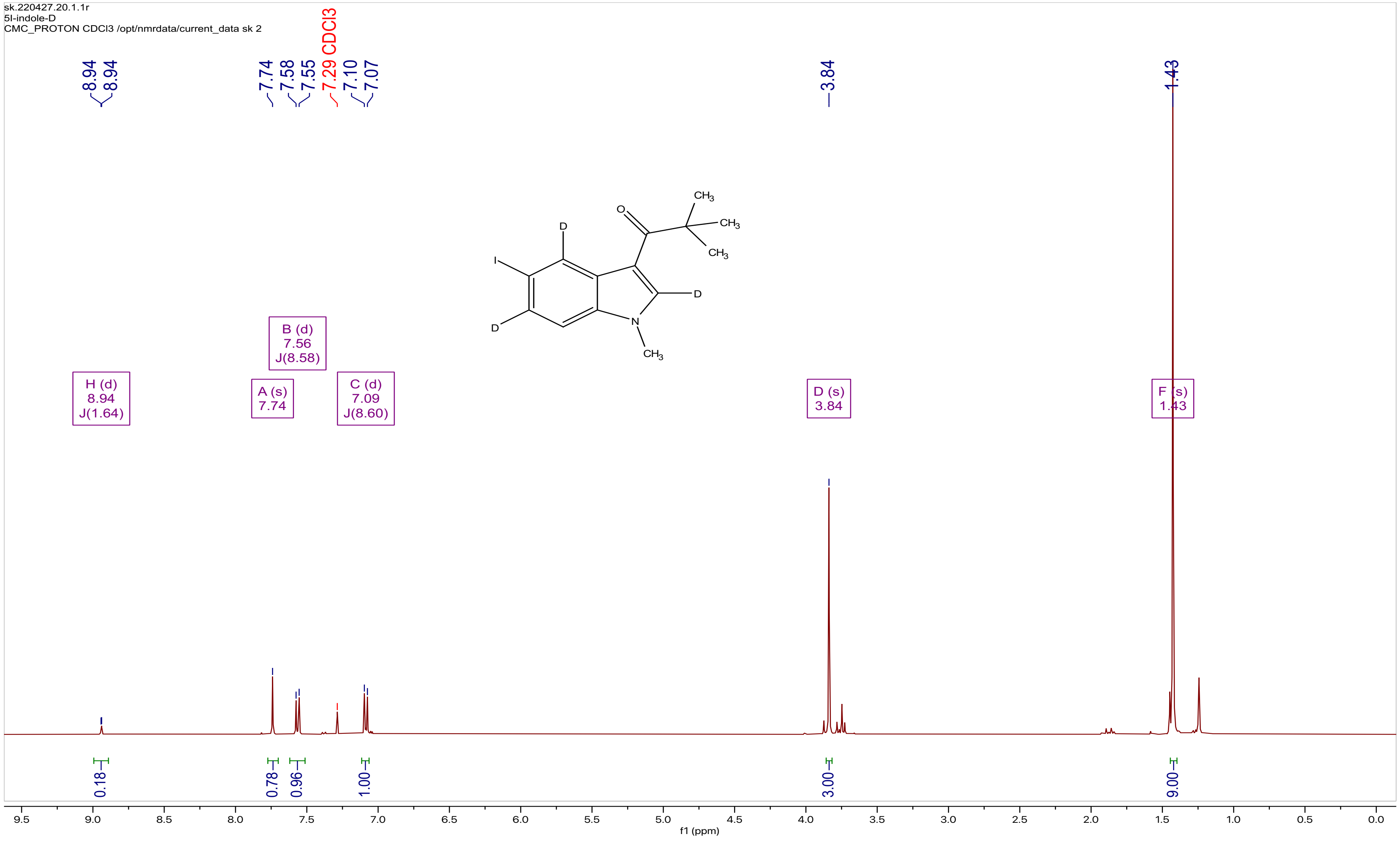

^{1}H NMR (400 MHz, $CDCl_3$) of 2g

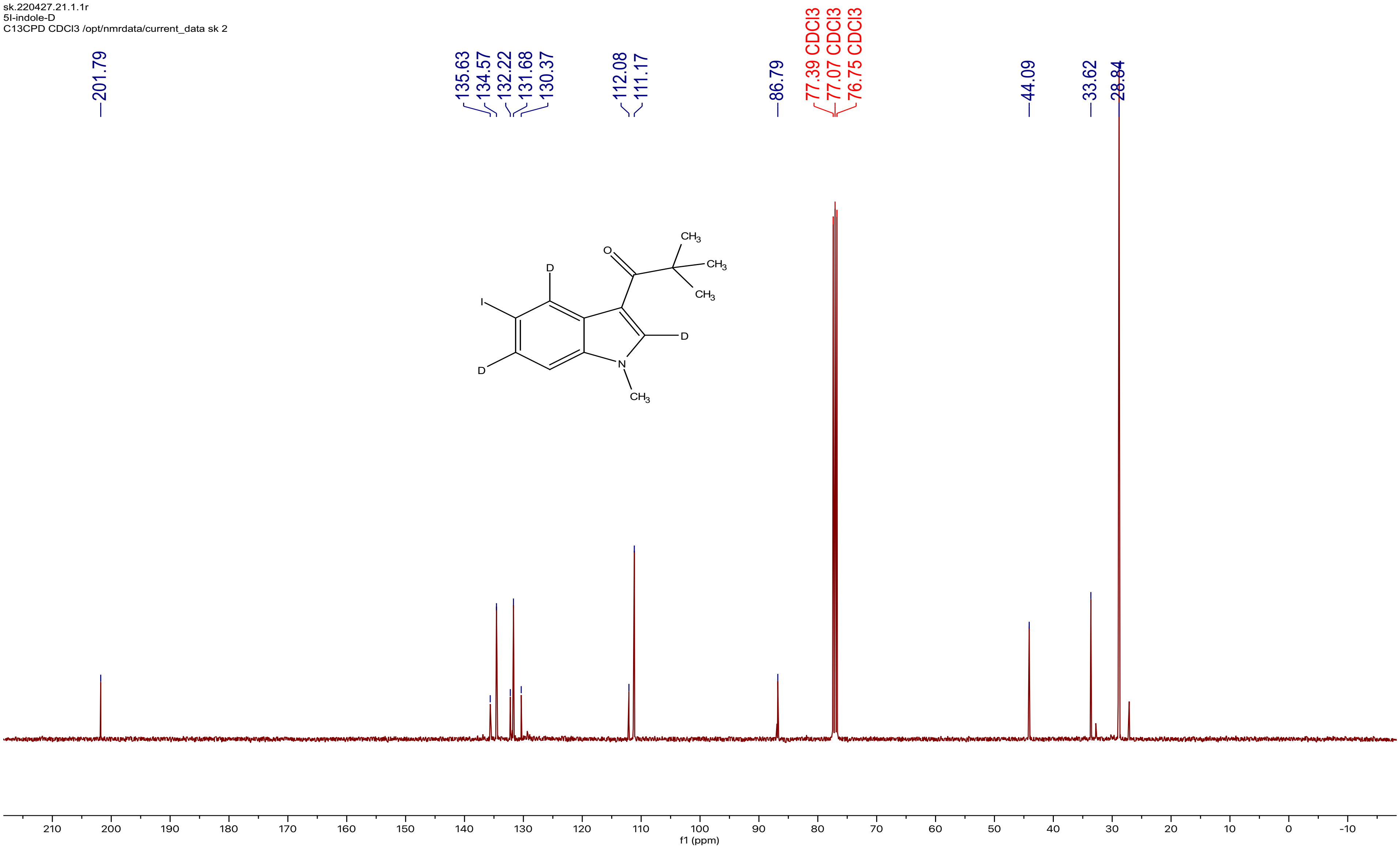

$^{13}C\{^{1}H\}$ NMR (101 MHz, $CDCl_3$) of 2g

1h

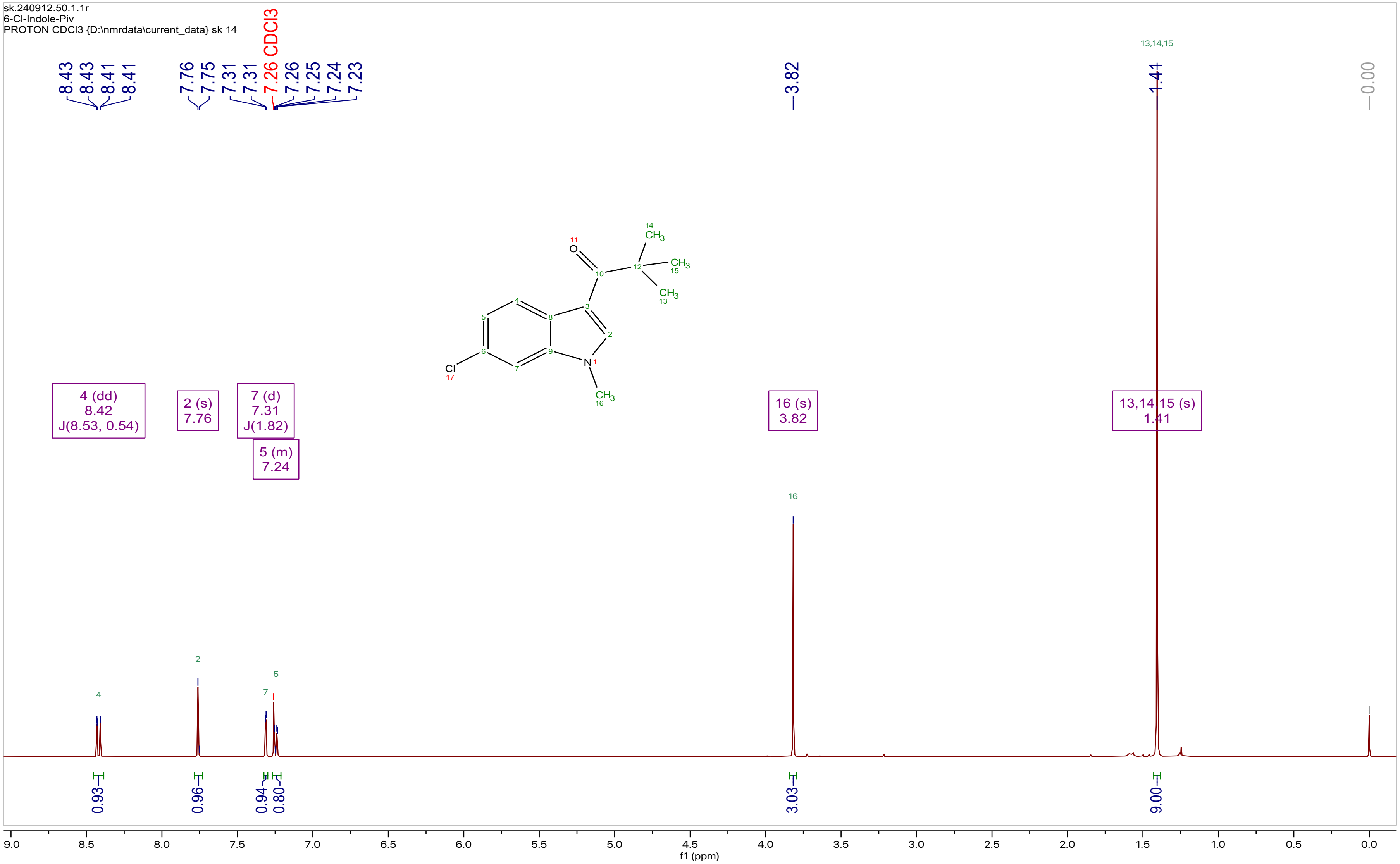

^{1}H NMR (400 MHz, $CDCl_3$) of 1h

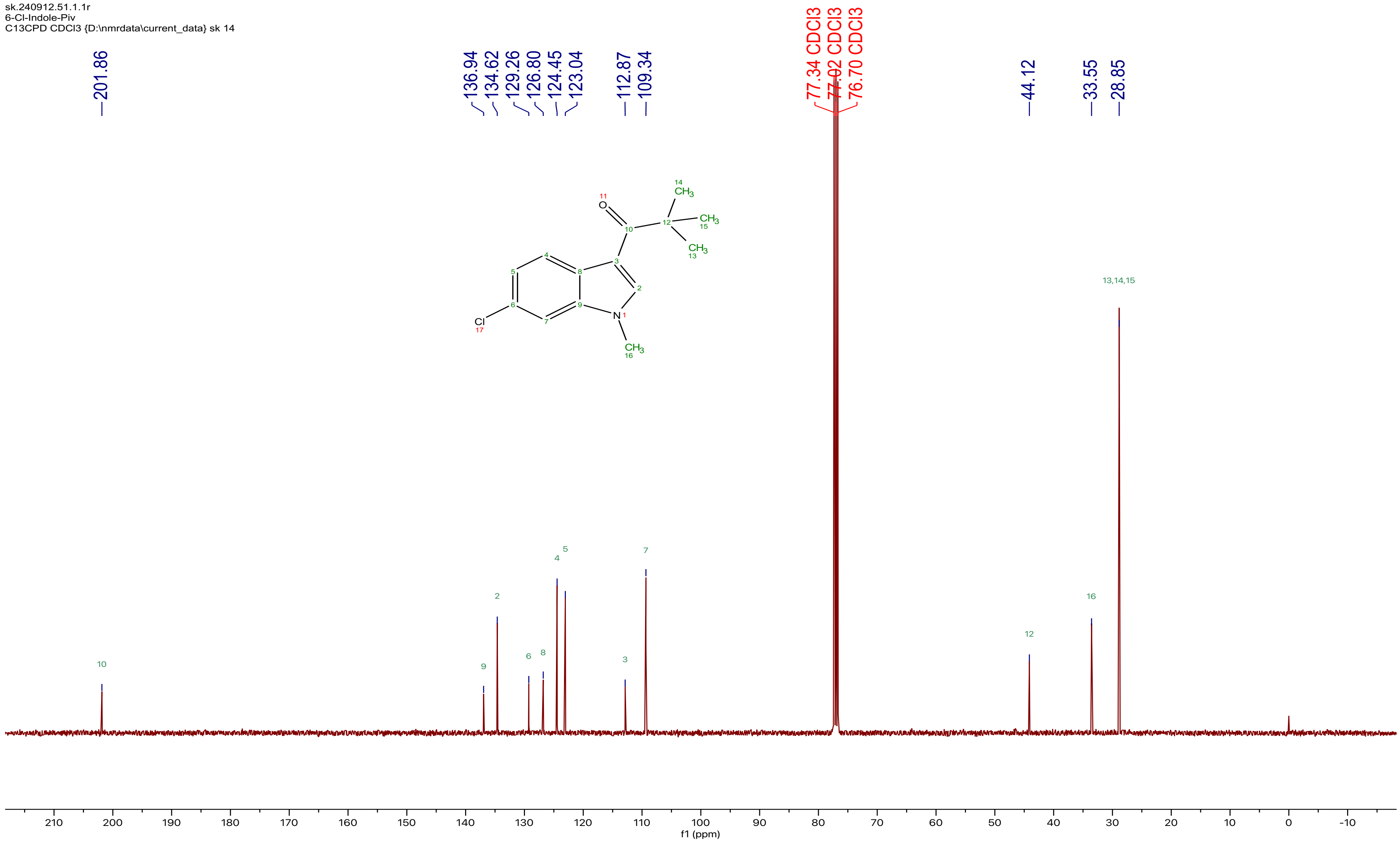

$^{13}C\{^1H\}$ NMR (101 MHz, $CDCl_3$) of 1h

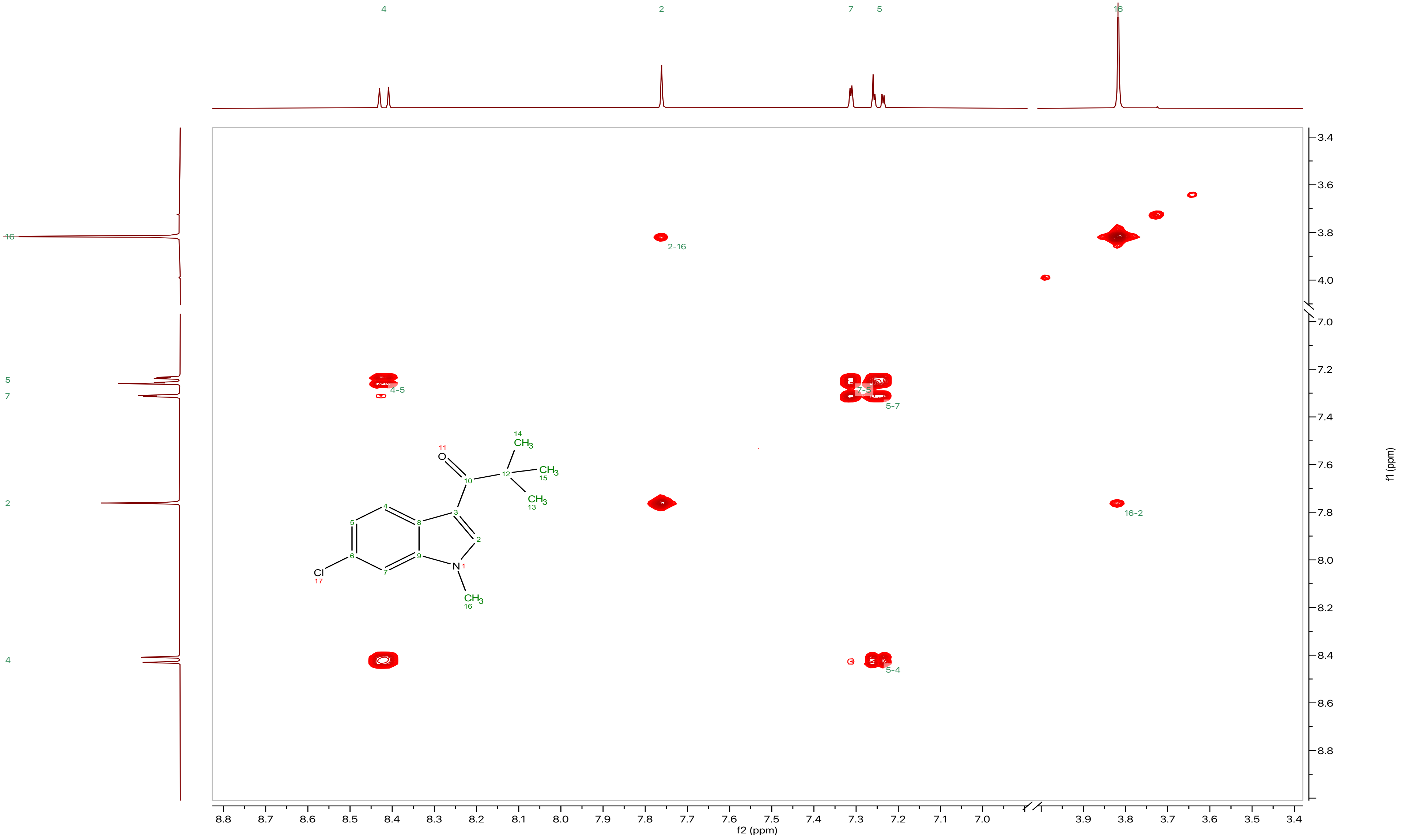

^{1}H-^{1}H COSY (400 MHz, $CDCl_3$) of 1h

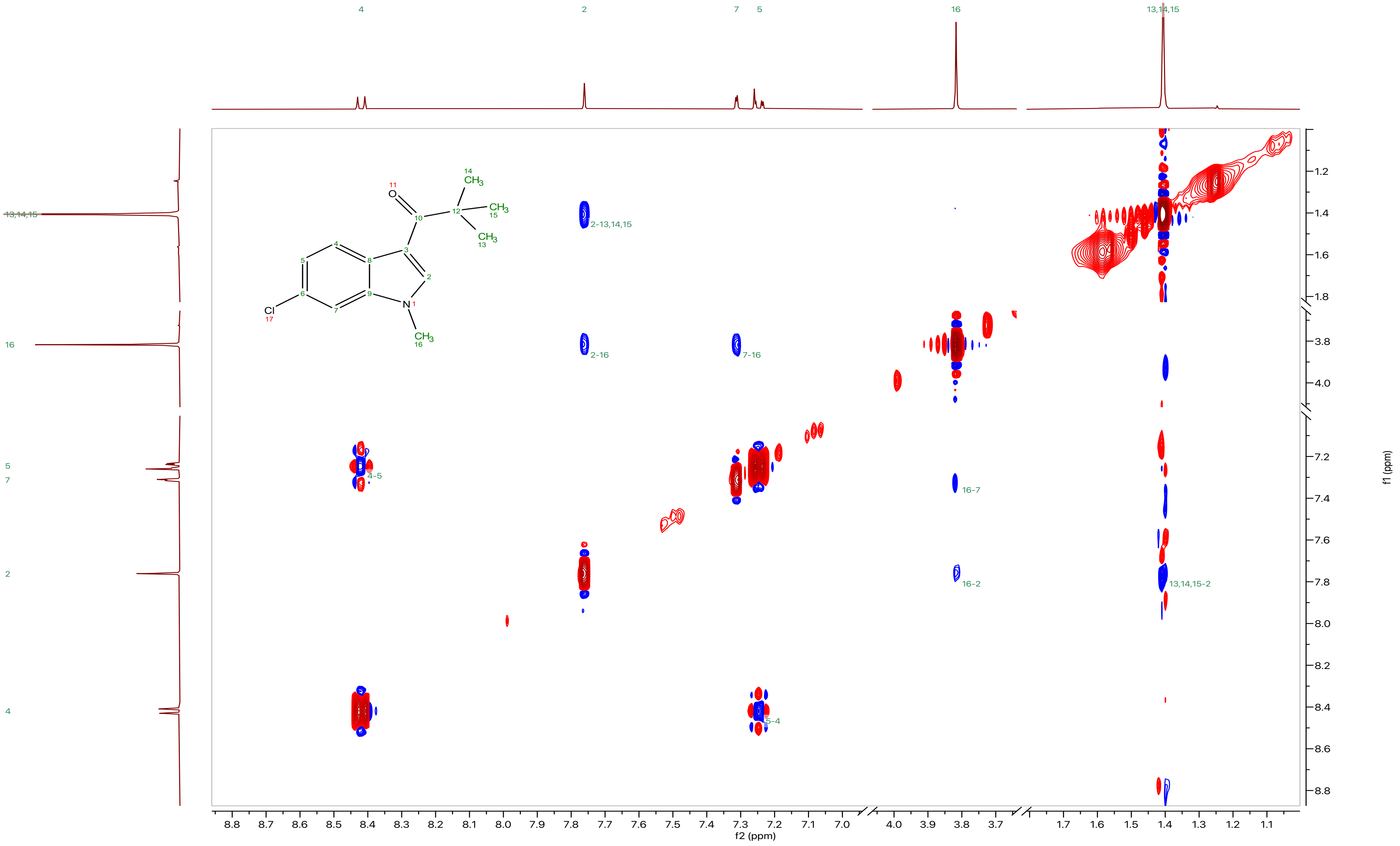

^1H-^1H NOESY (400 MHz, $CDCl_3$) of 1h

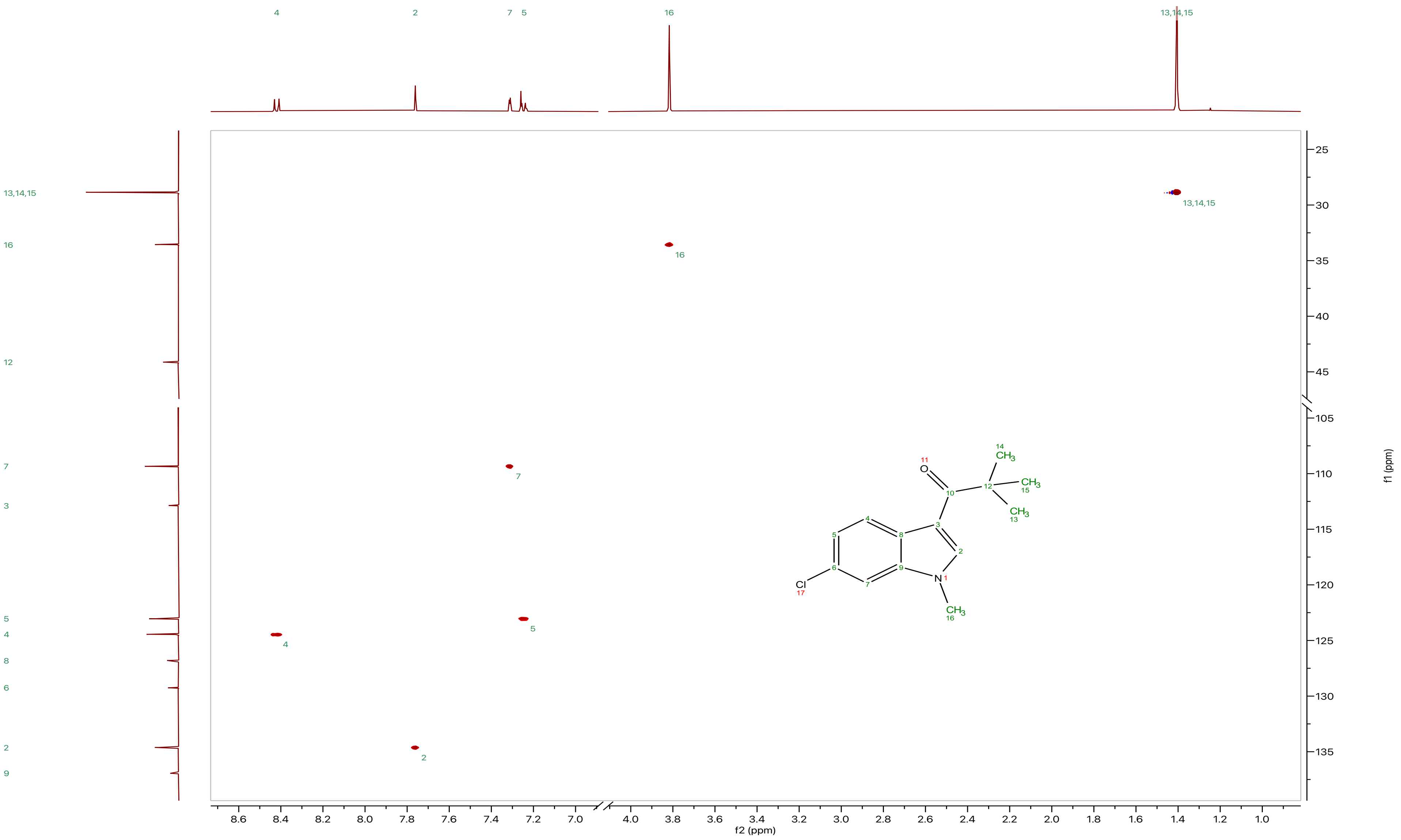

^{1}H-^{13}C{^{1}H} HSQC NMR (400/101 MHz, $CDCl_3$) of 1h

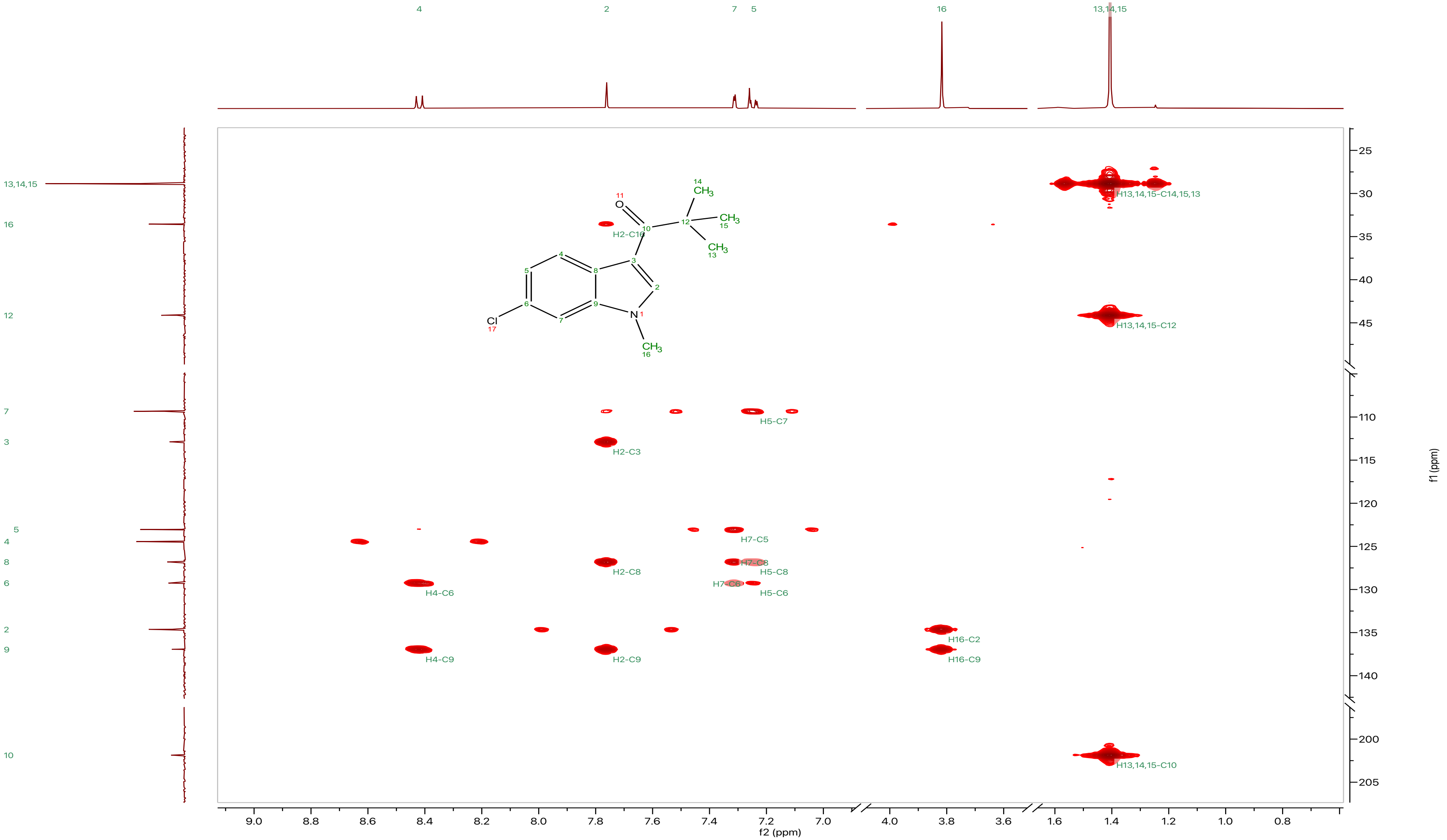

^{1}H-$^{13}C\{^{1}H\}$ HMBC NMR (400/101 MHz, $CDCl_3$) of 1h

2h

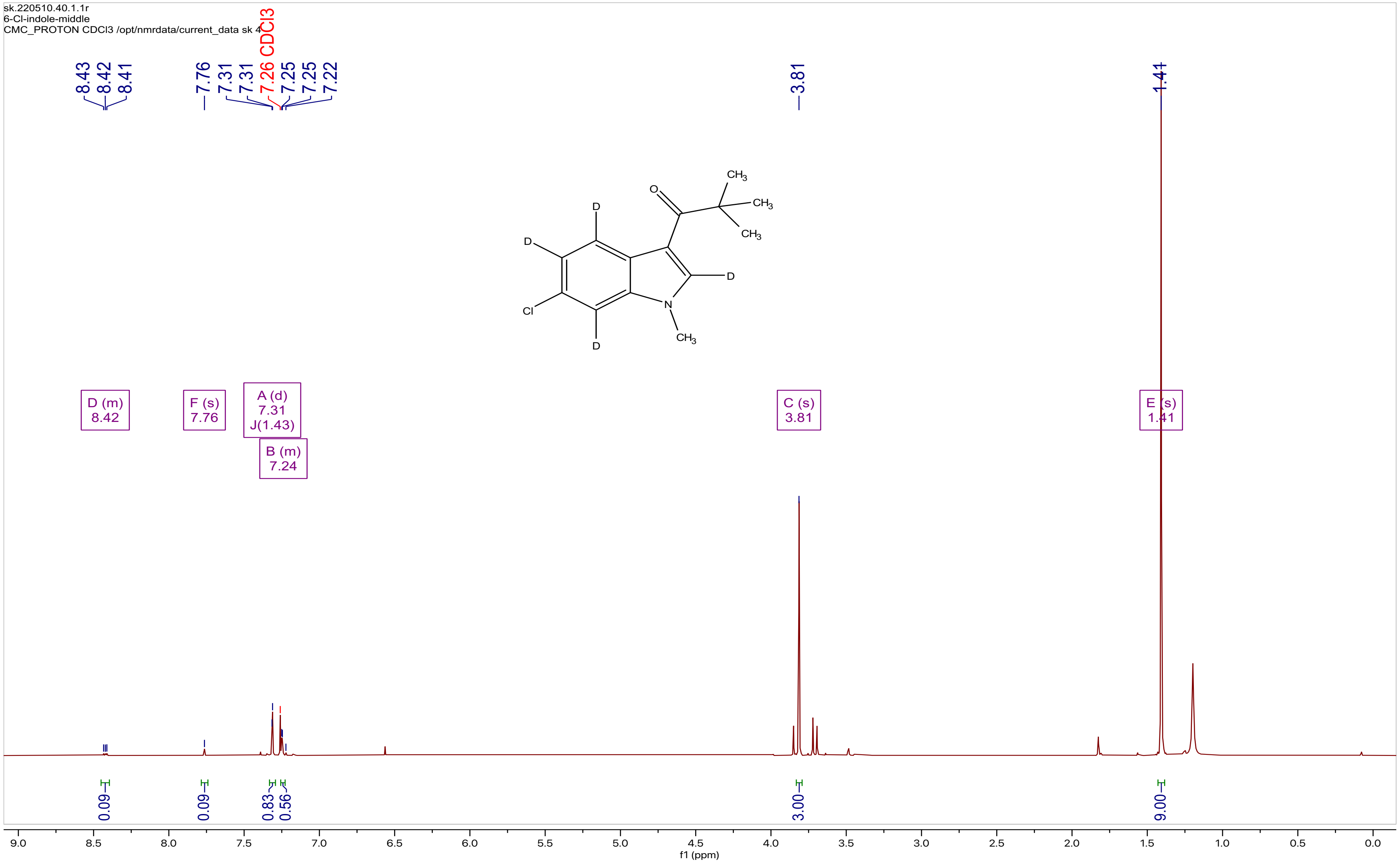

^{1}H NMR (400 MHz, $CDCl_3$) of 2h

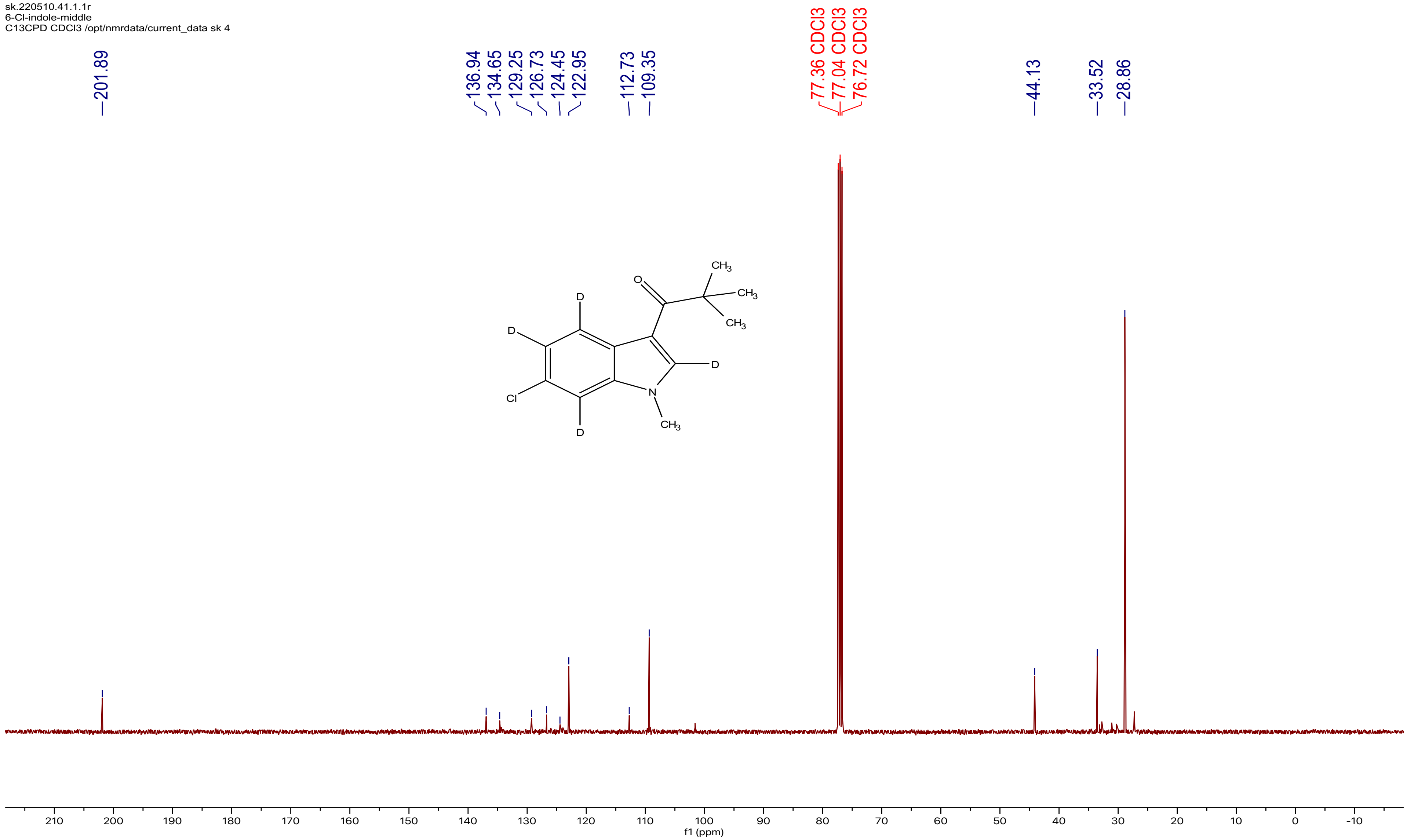

$^{13}C\{^{1}H\}$ NMR (101 MHz, $CDCl_3$) of 2h

1i

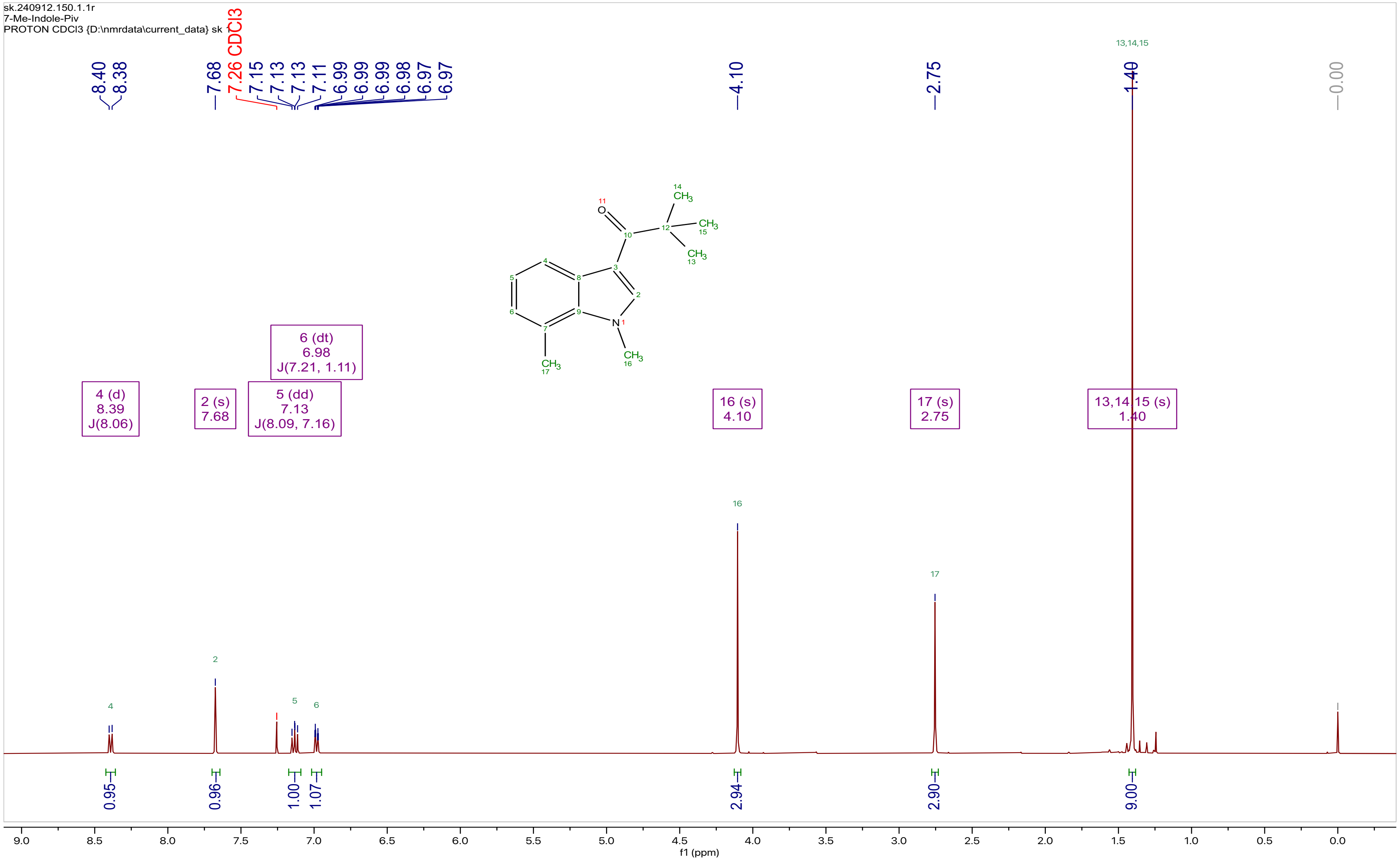

^{1}H NMR (400 MHz, $CDCl_3$) of 1i

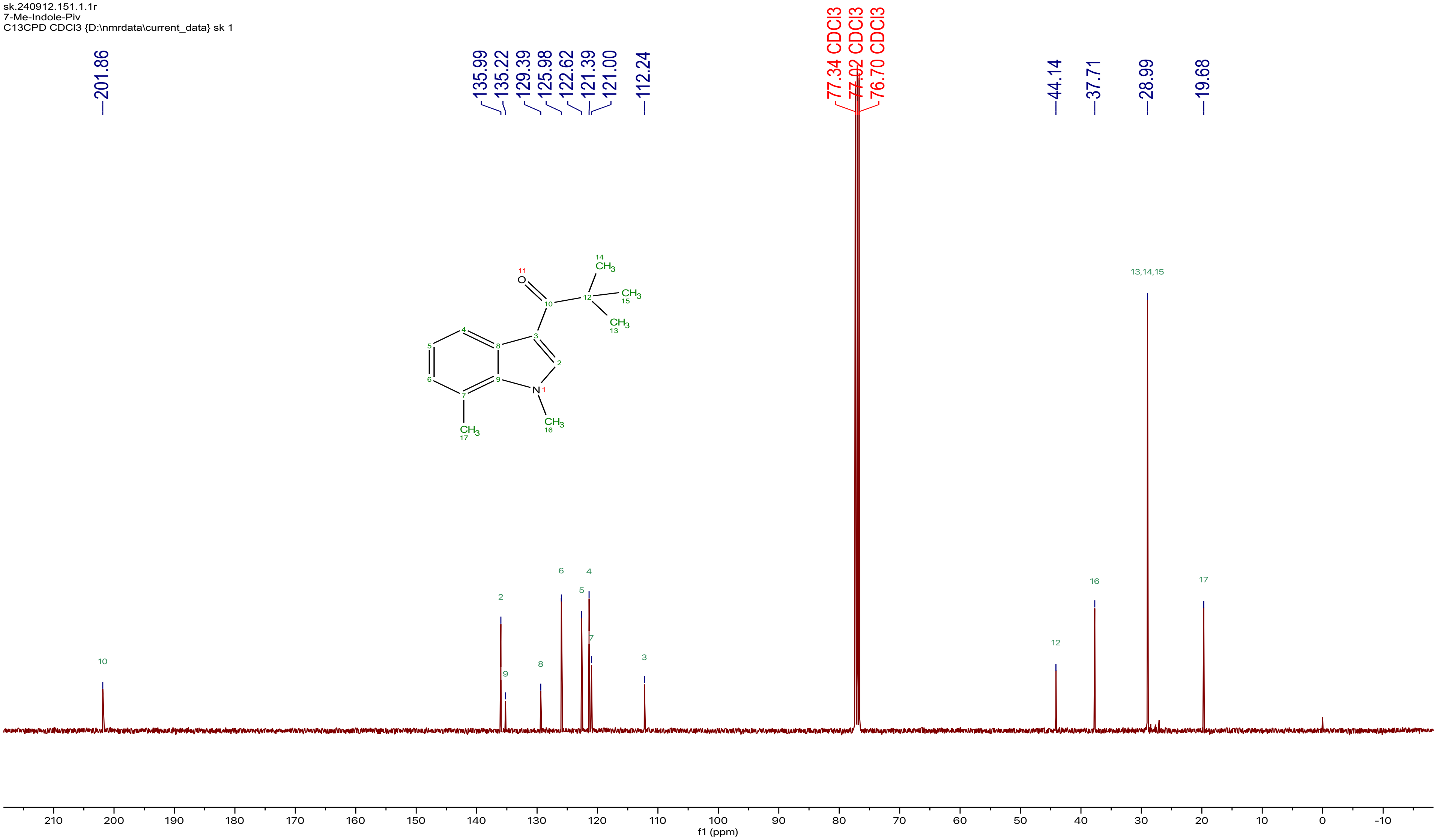

$^{13}C\{^{1}H\}$ NMR (101 MHz, $CDCl_3$) of 1i

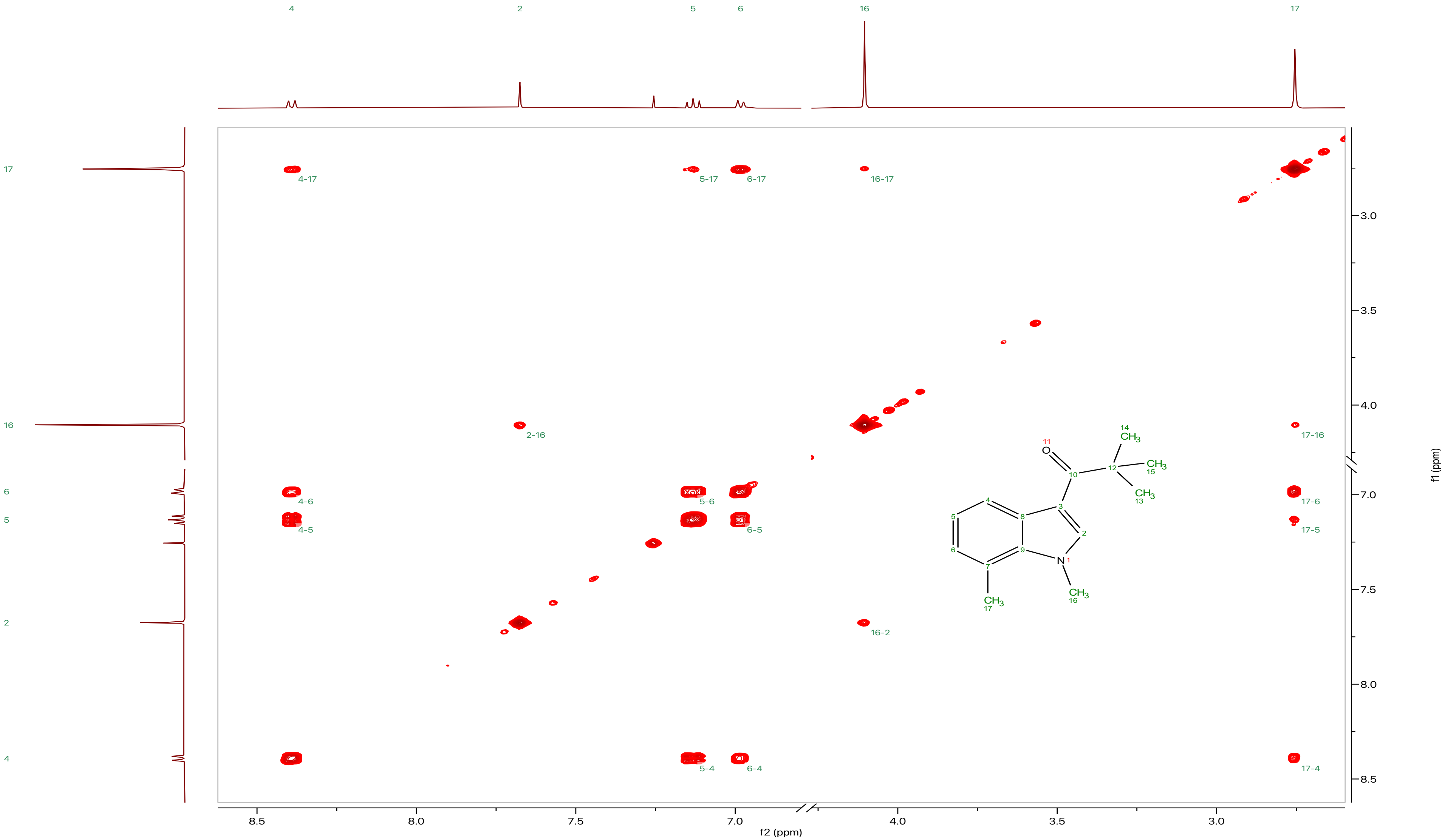

^1H-^1H COSY (400 MHz, $CDCl_3$) of 1i

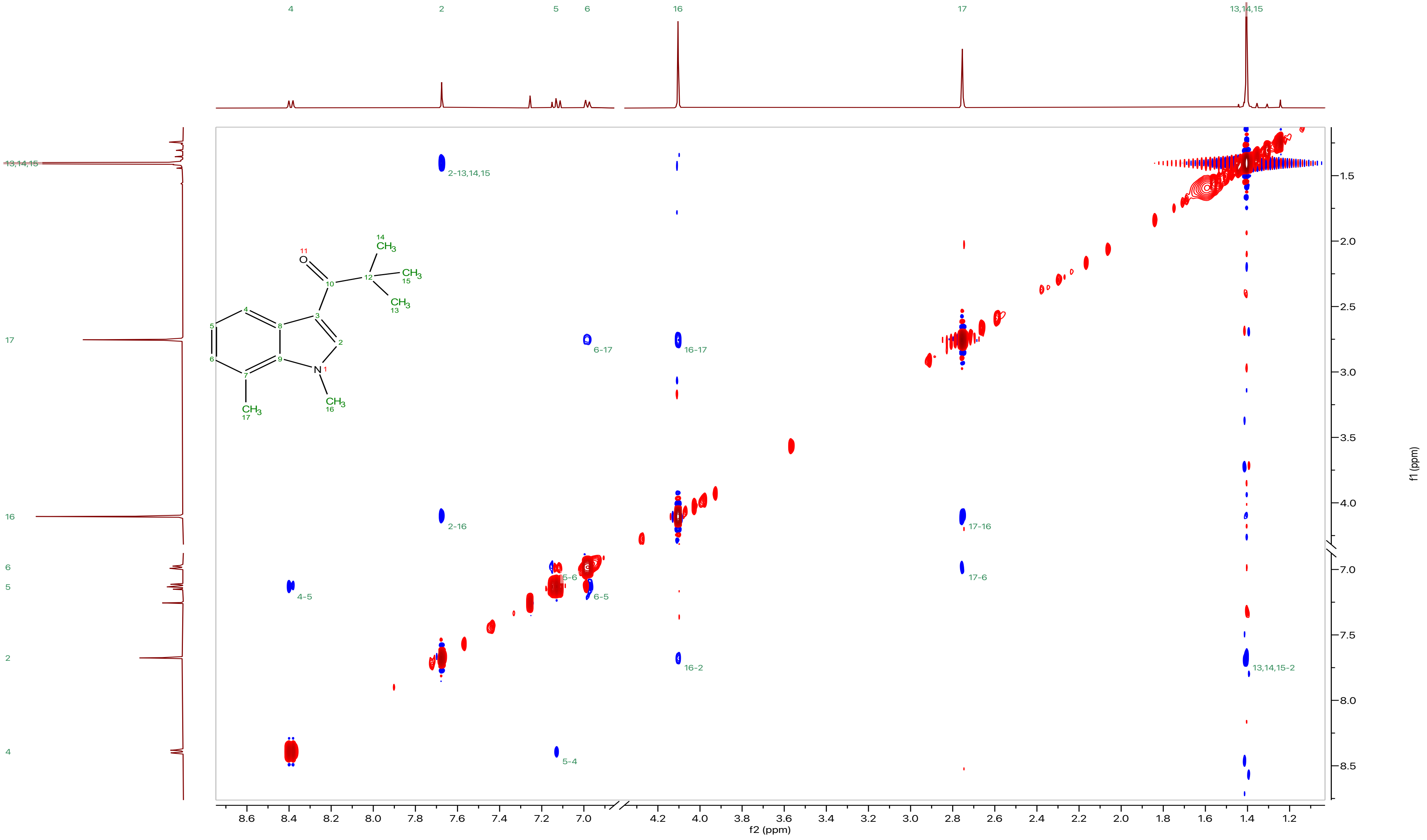

^{1}H-^{1}H NOESY (400 MHz, $CDCl_3$) of 1i

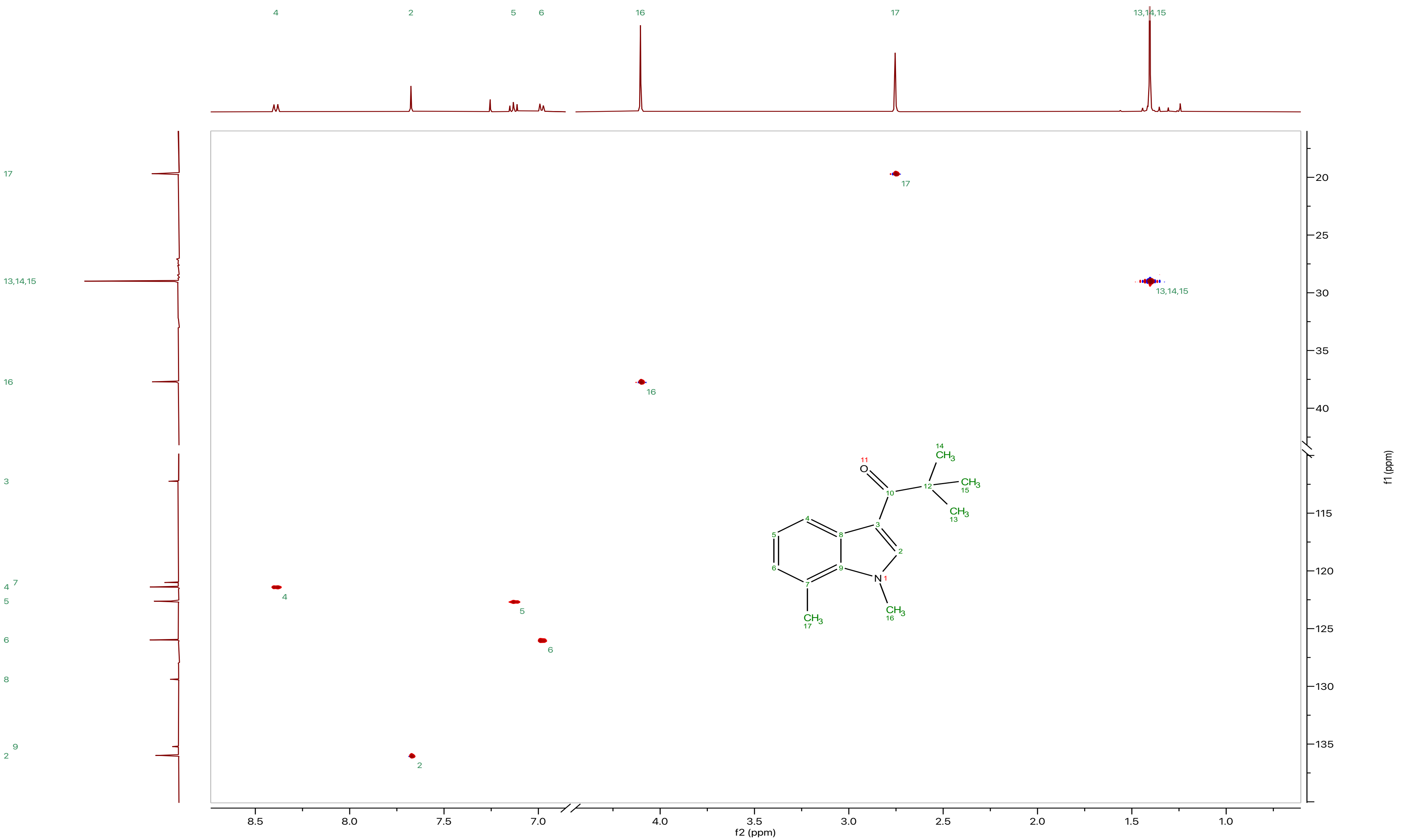

^{1}H-$^{13}C\{^{1}H\}$ HSQC NMR (400/101 MHz, $CDCl_3$) of 1i

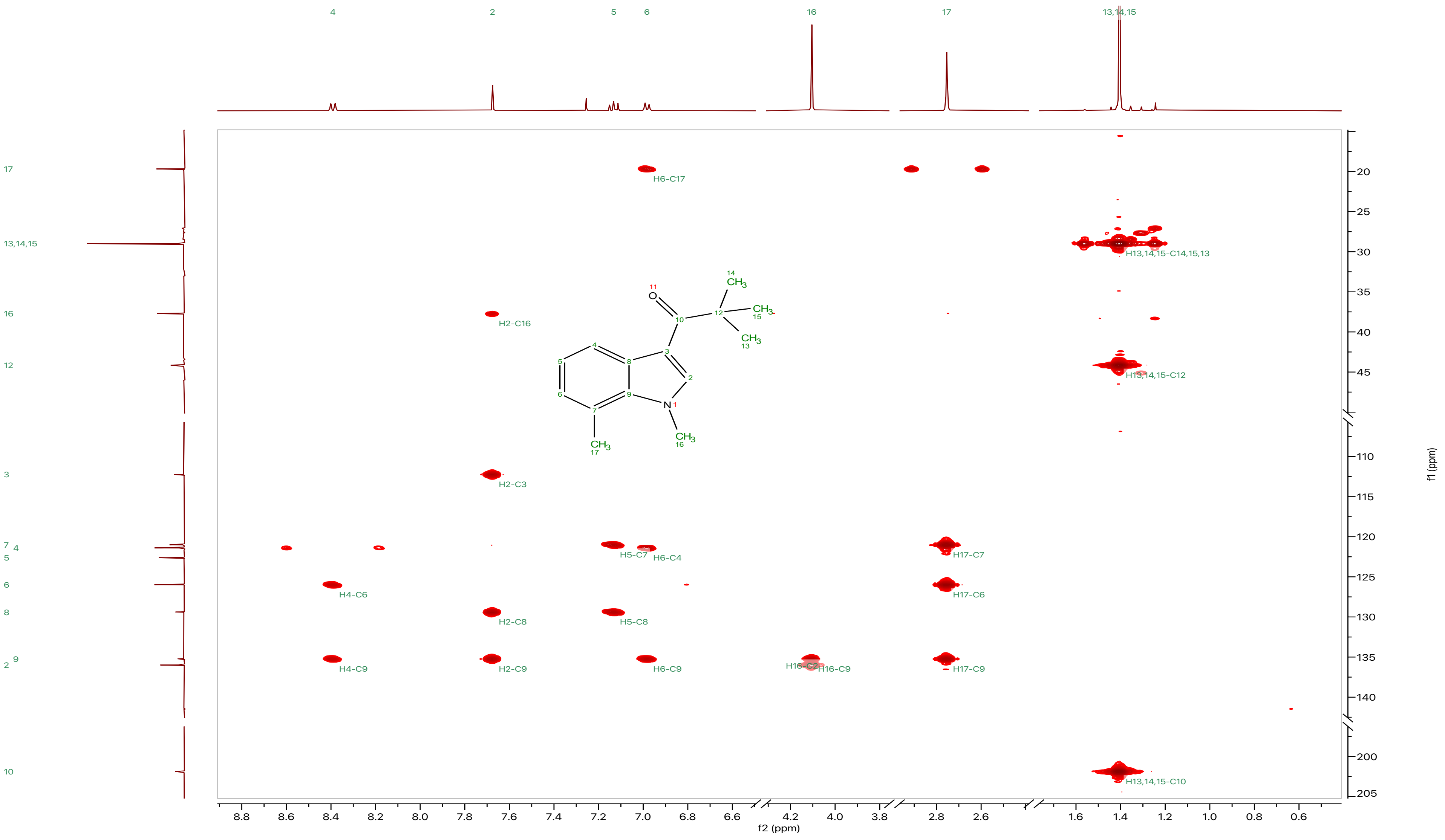

^{1}H-$^{13}C\{^{1}H\}$ HMBC NMR (400/101 MHz, $CDCl_3$) of 1i

2i

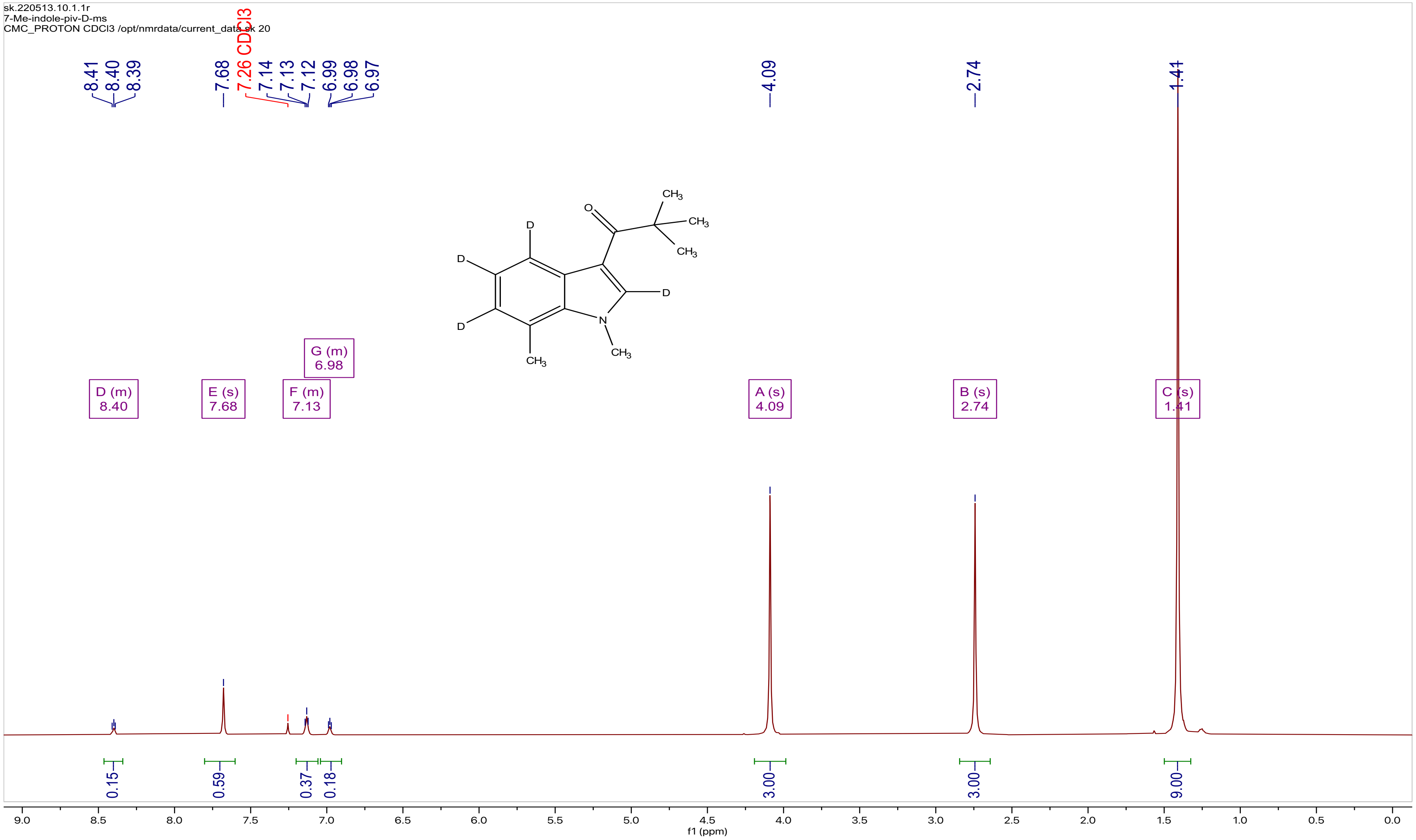

^{1}H NMR (400 MHz, $CDCl_3$) of 2i

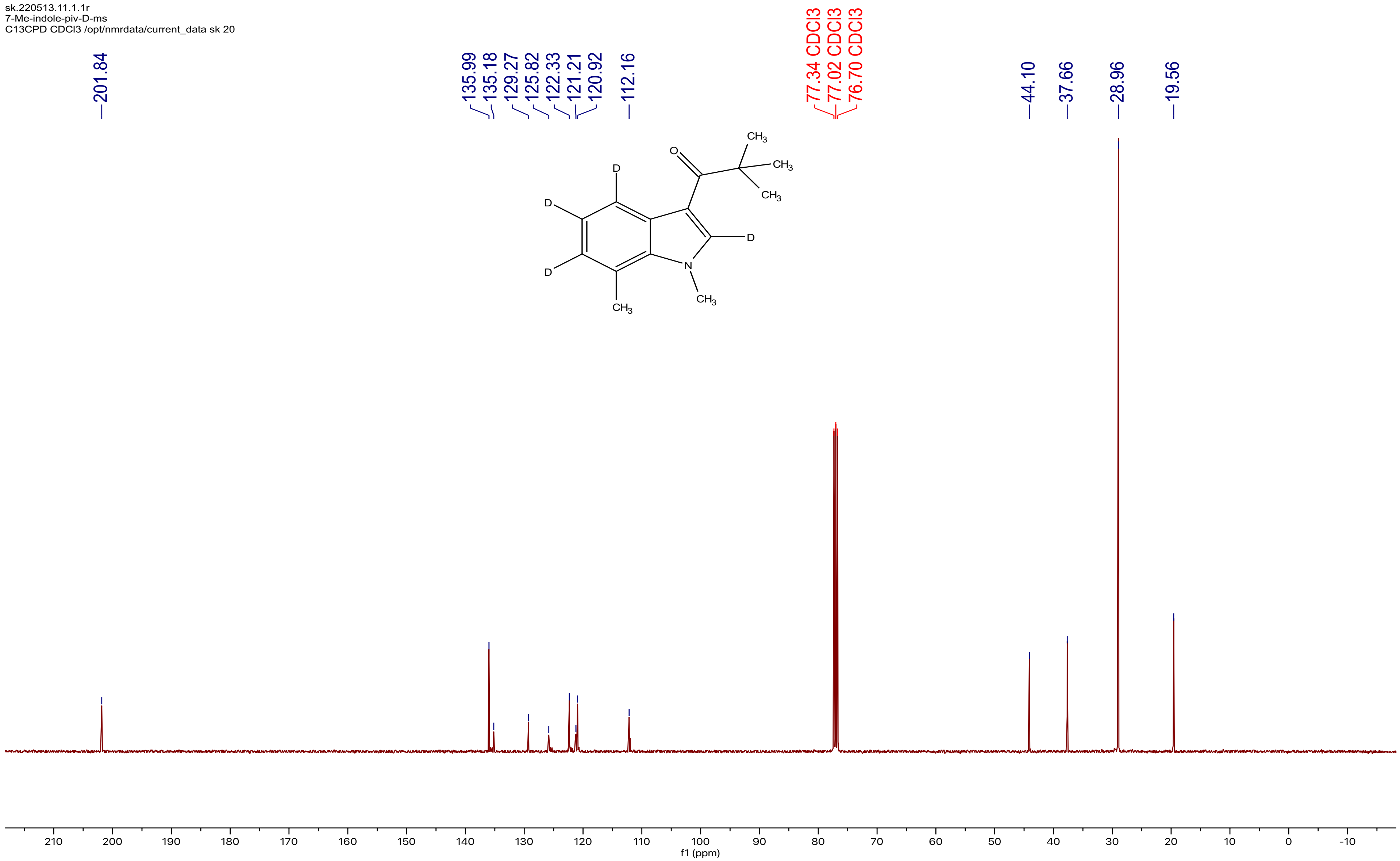

$^{13}C\{^{1}H\}$ **NMR (101 MHz, $CDCl_3$) of 2i**

1j

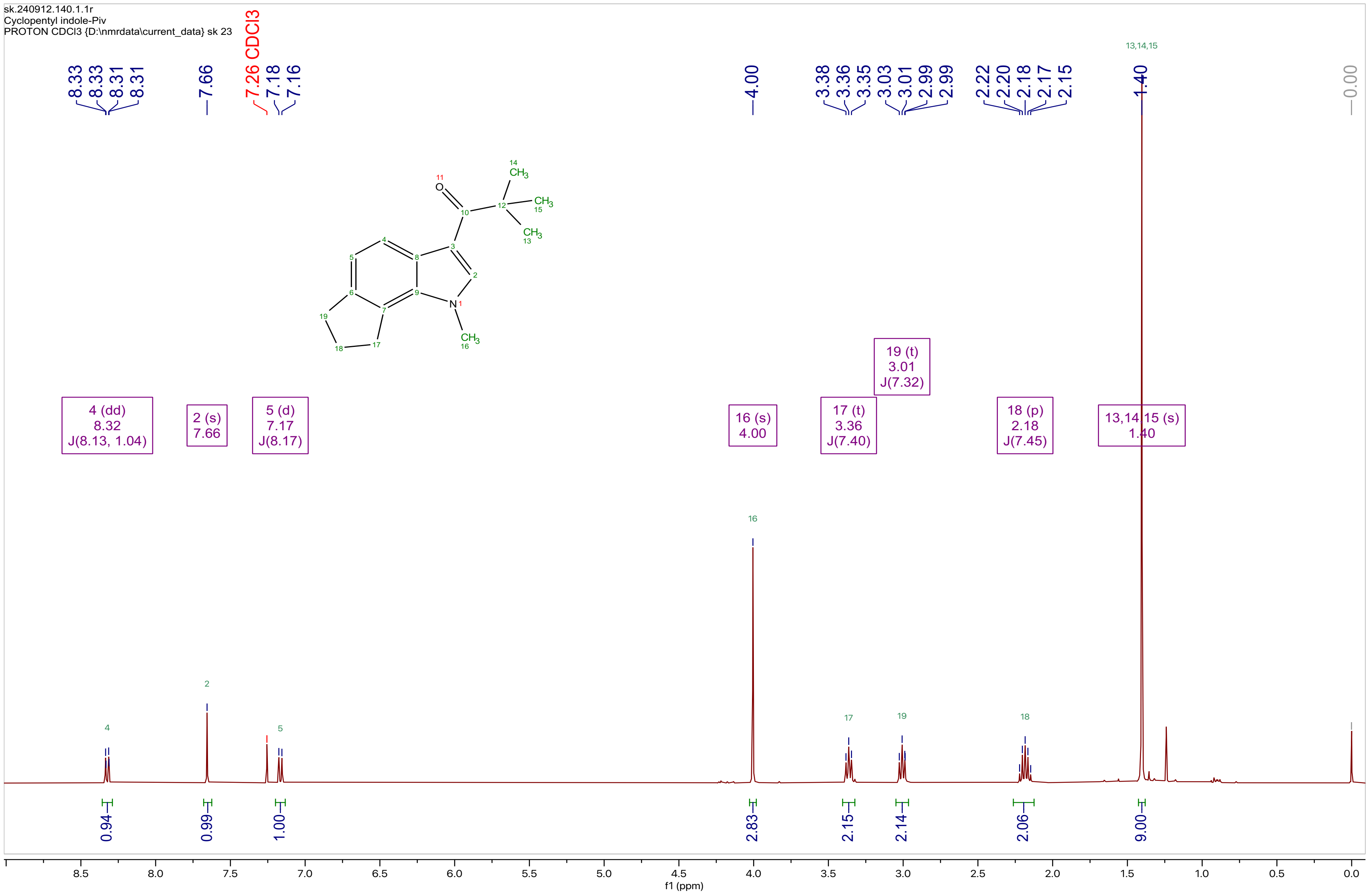

^{1}H NMR (400 MHz, $CDCl_3$) of 1j

sk.240912.141.1.1r
Cyclopentyl indole-Piv
C13CPD CDCl3 {D:\nmrdata\current_data} sk 23

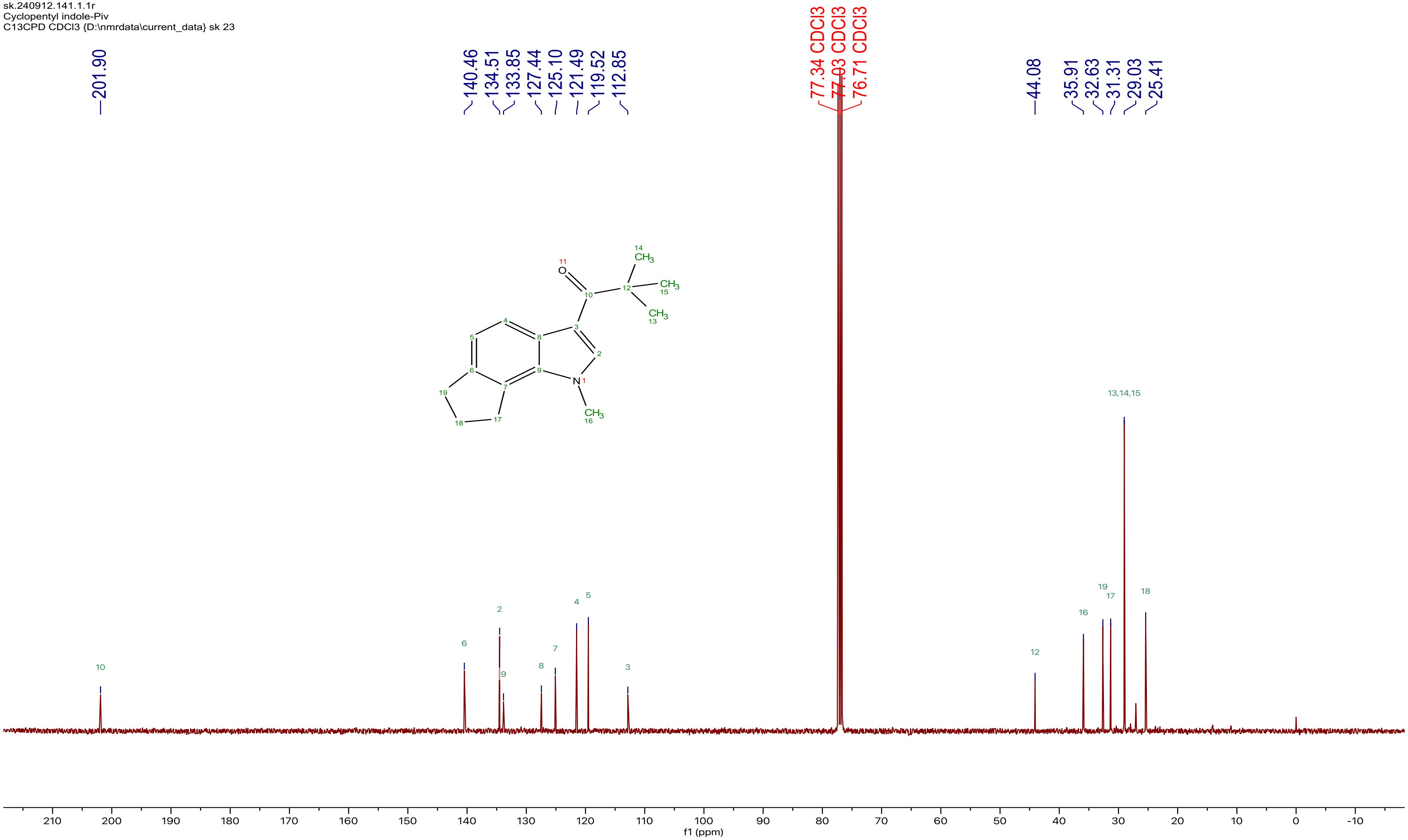

$^{13}C\{^{1}H\}$ NMR (101 MHz, $CDCl_3$) of 1j

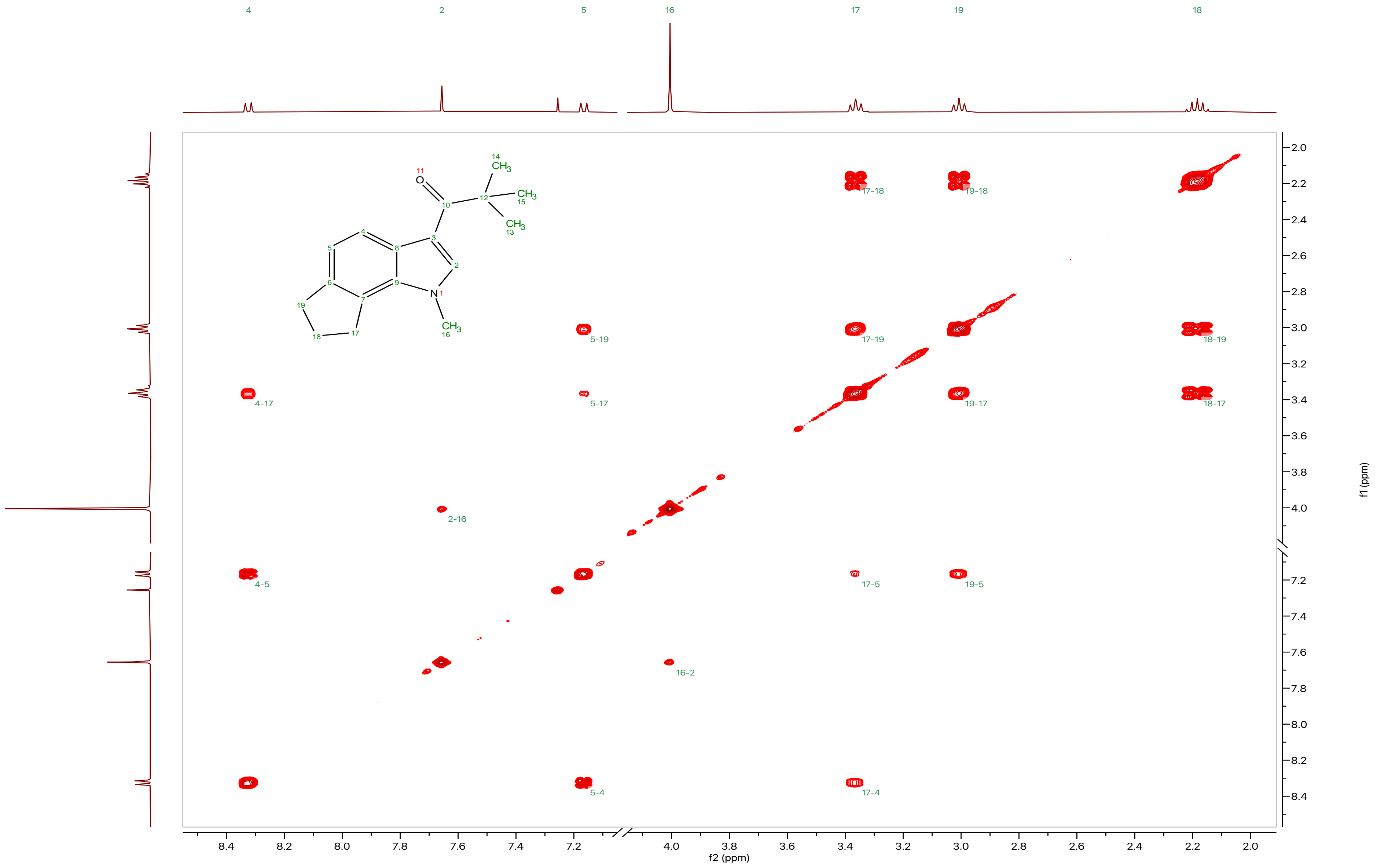

^1H-^1H COSY (400 MHz, $CDCl_3$) of 1j

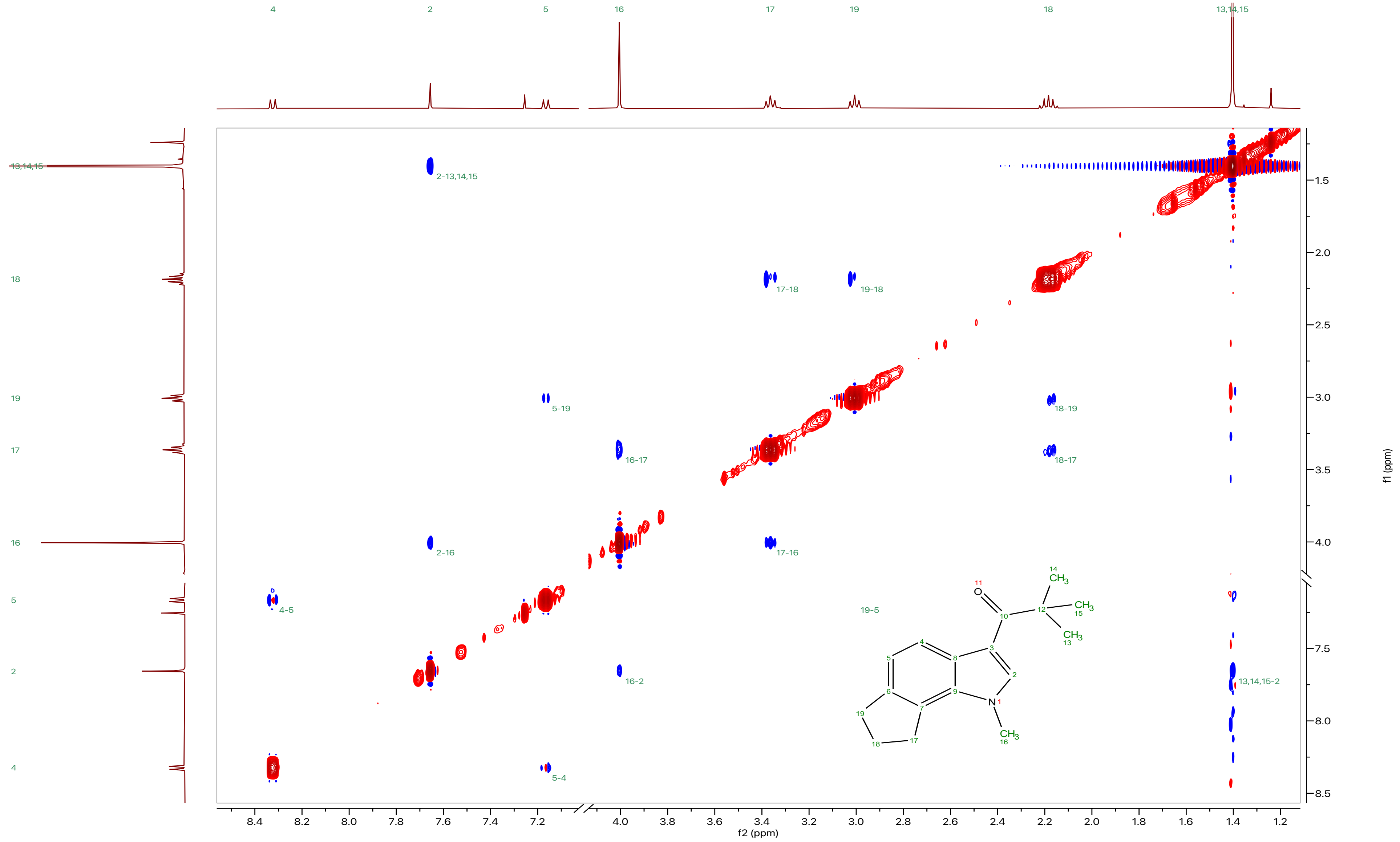

^1H-^1H NOESY (400 MHz, $CDCl_3$) of 1j

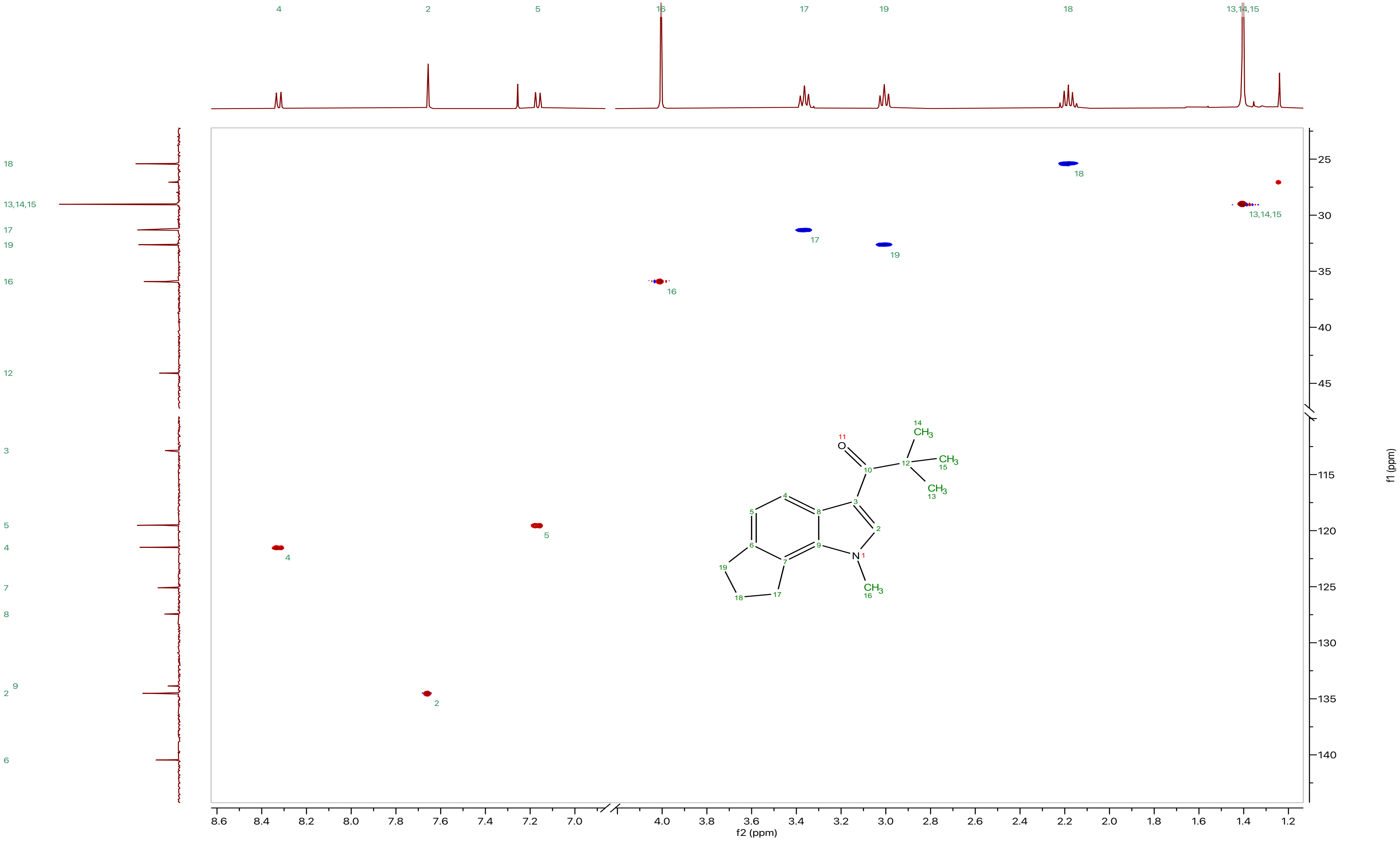

^{1}H-$^{13}C\{^{1}H\}$ HSQC NMR (400/101 MHz, $CDCl_3$) of 1j

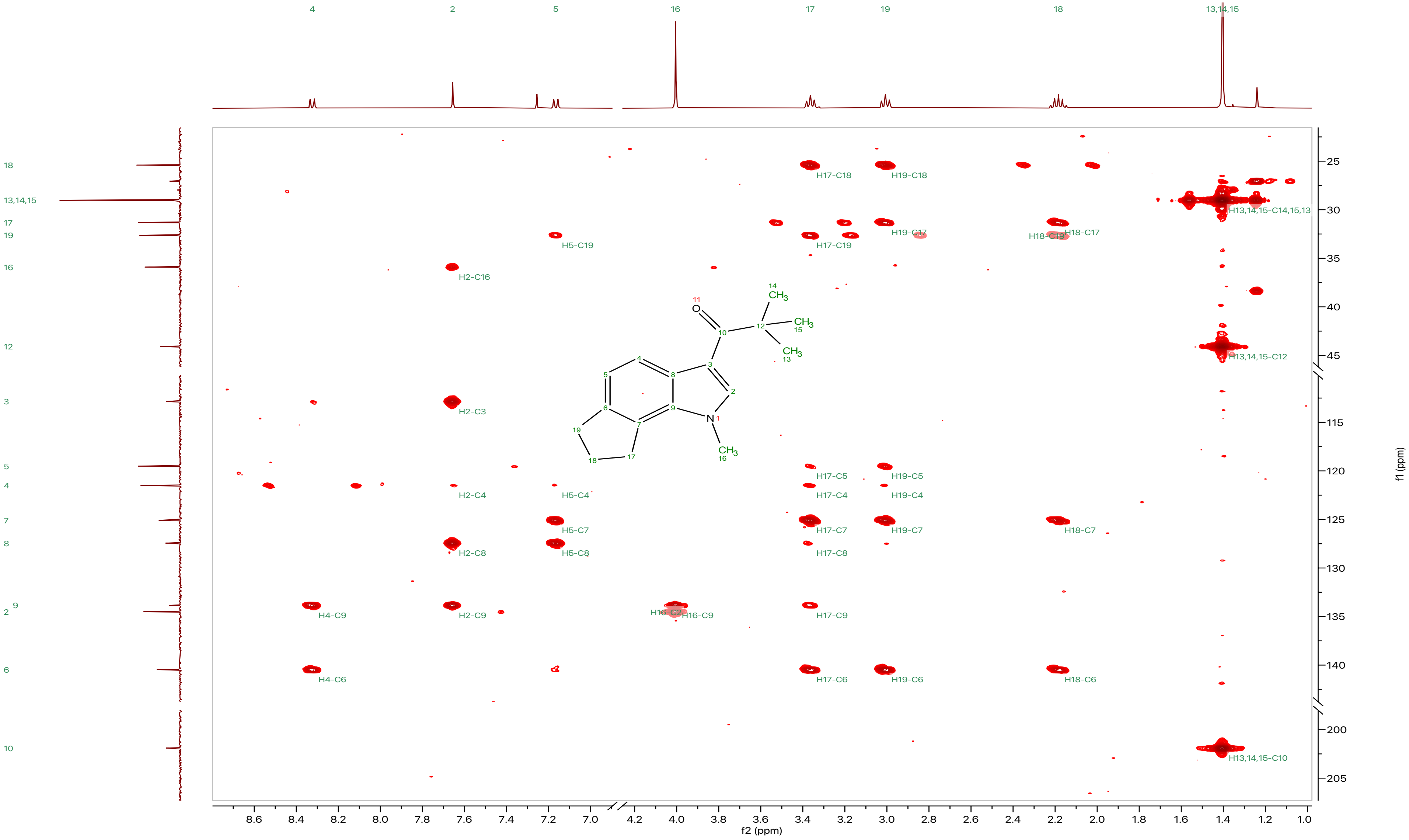

^{1}H-$^{13}C\{^{1}H\}$ HMBC NMR (400/101 MHz, $CDCl_3$) of 1j

2j

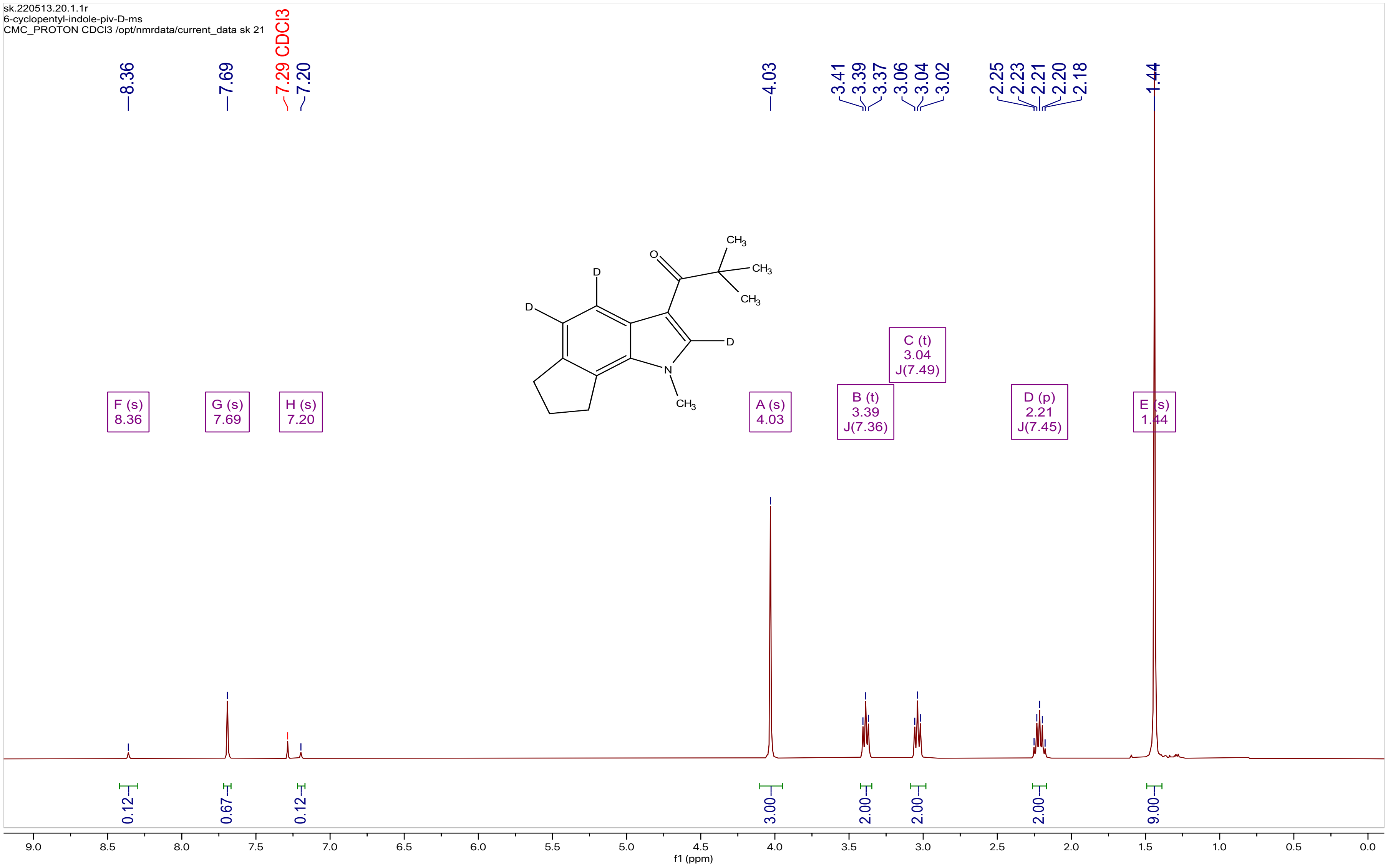

^{1}H NMR (400 MHz, $CDCl_3$) of 2j

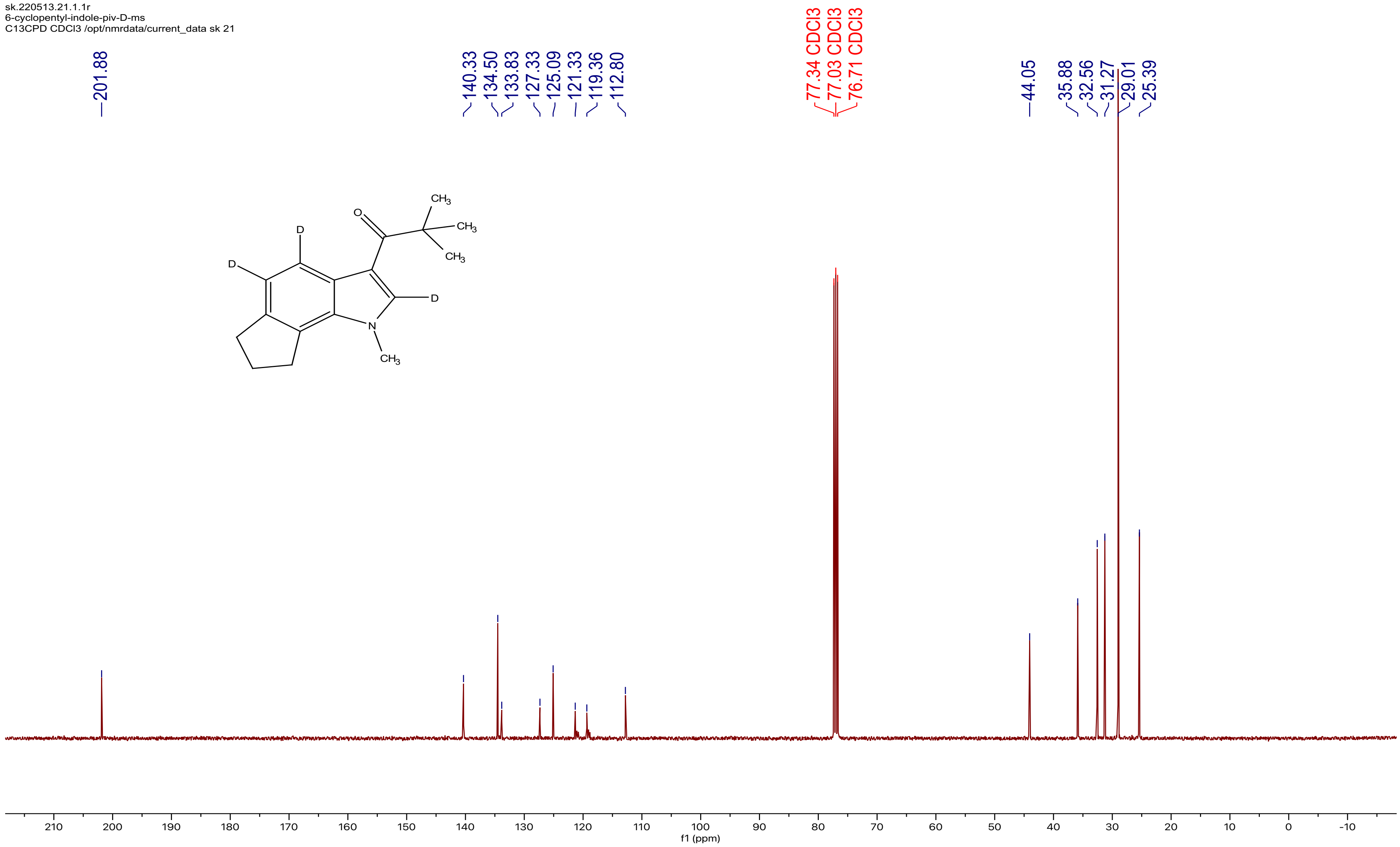

$^{13}C\{^{1}H\}$ **NMR (101 MHz, $CDCl_3$) of 2j**

1k

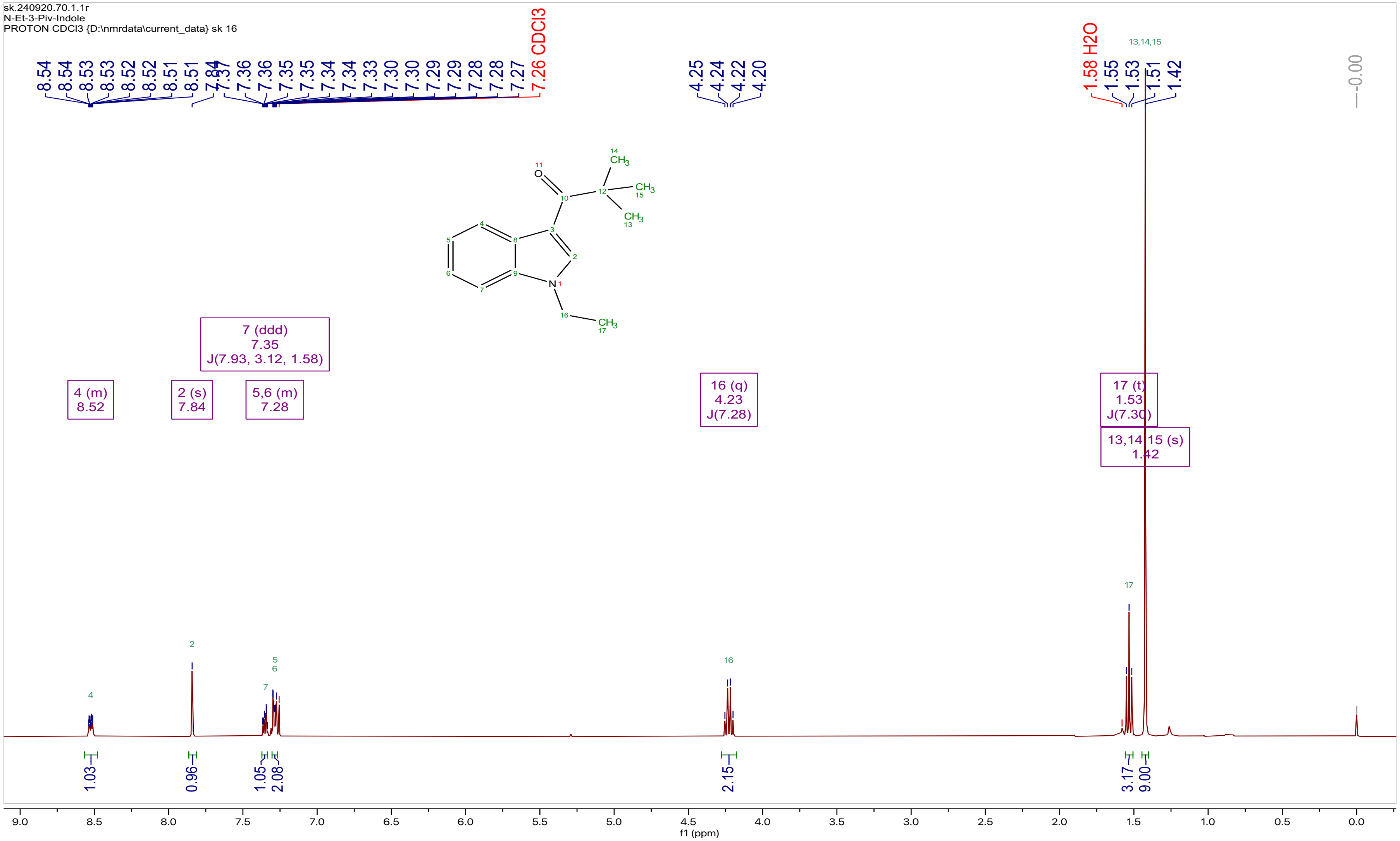

^{1}H NMR (400 MHz, $CDCl_3$) of 1k

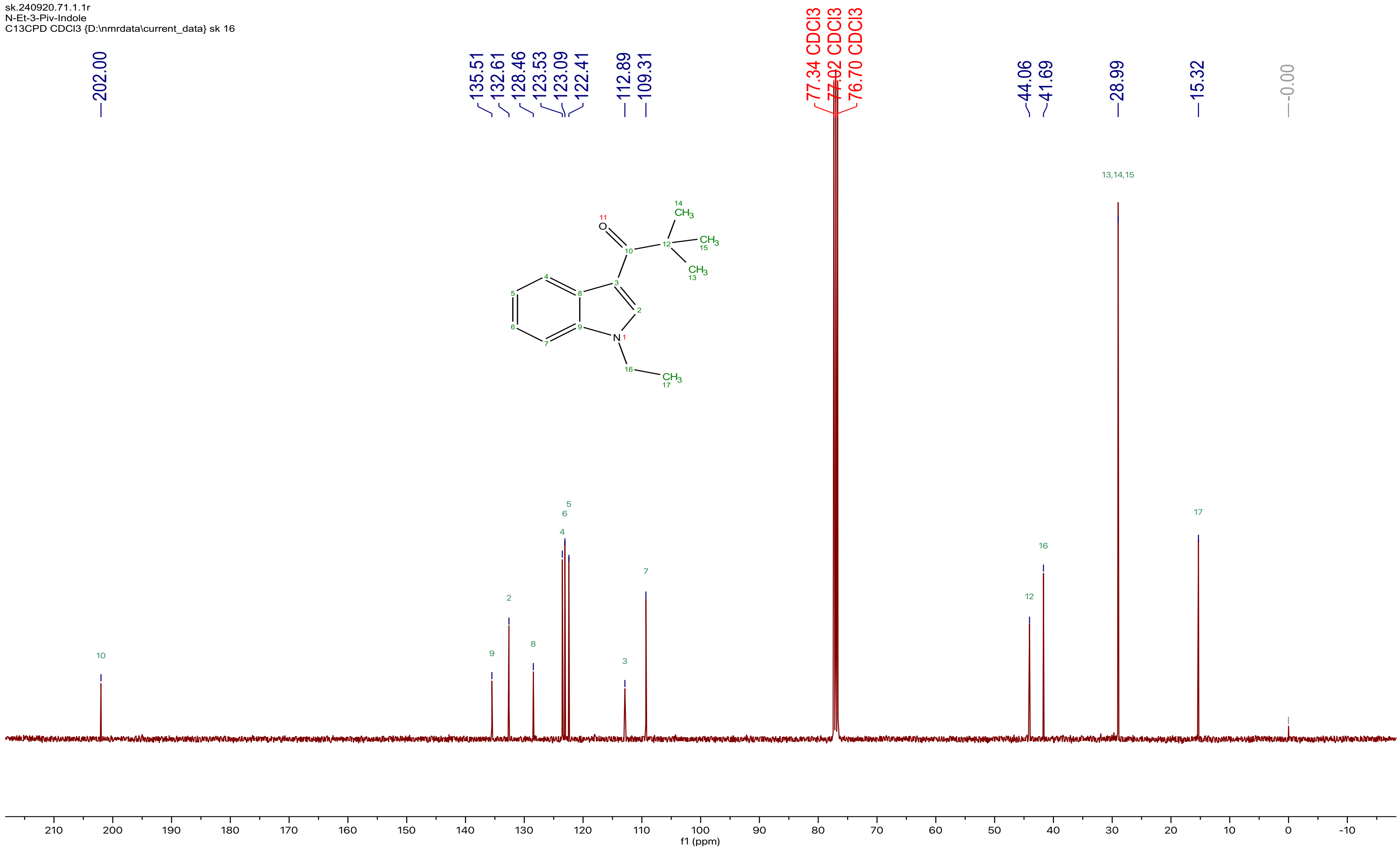

$^{13}C\{^1H\}$ NMR (101 MHz, $CDCl_3$) of 1k

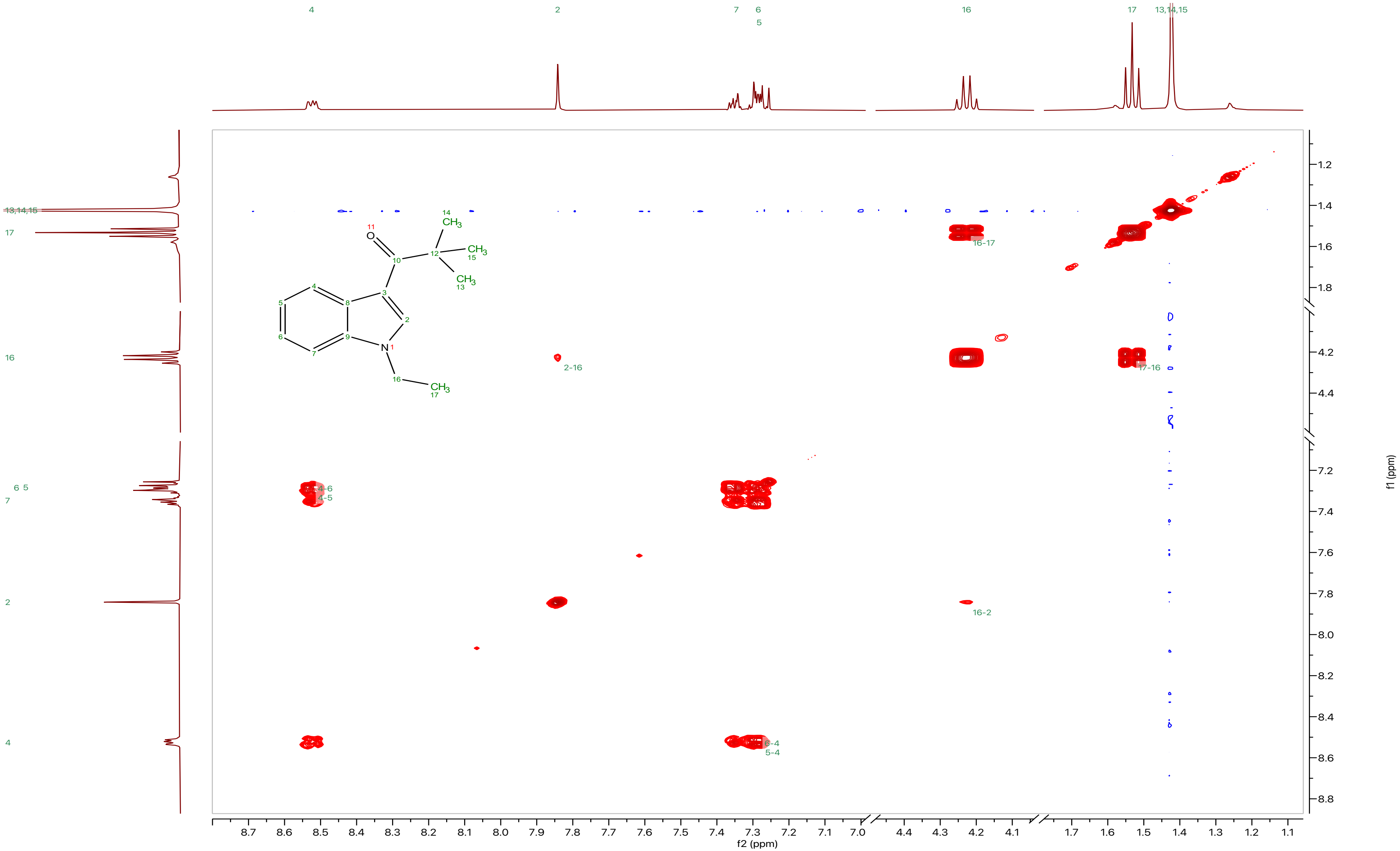

^{1}H-^{1}H COSY (400 MHz, $CDCl_3$) of 1k

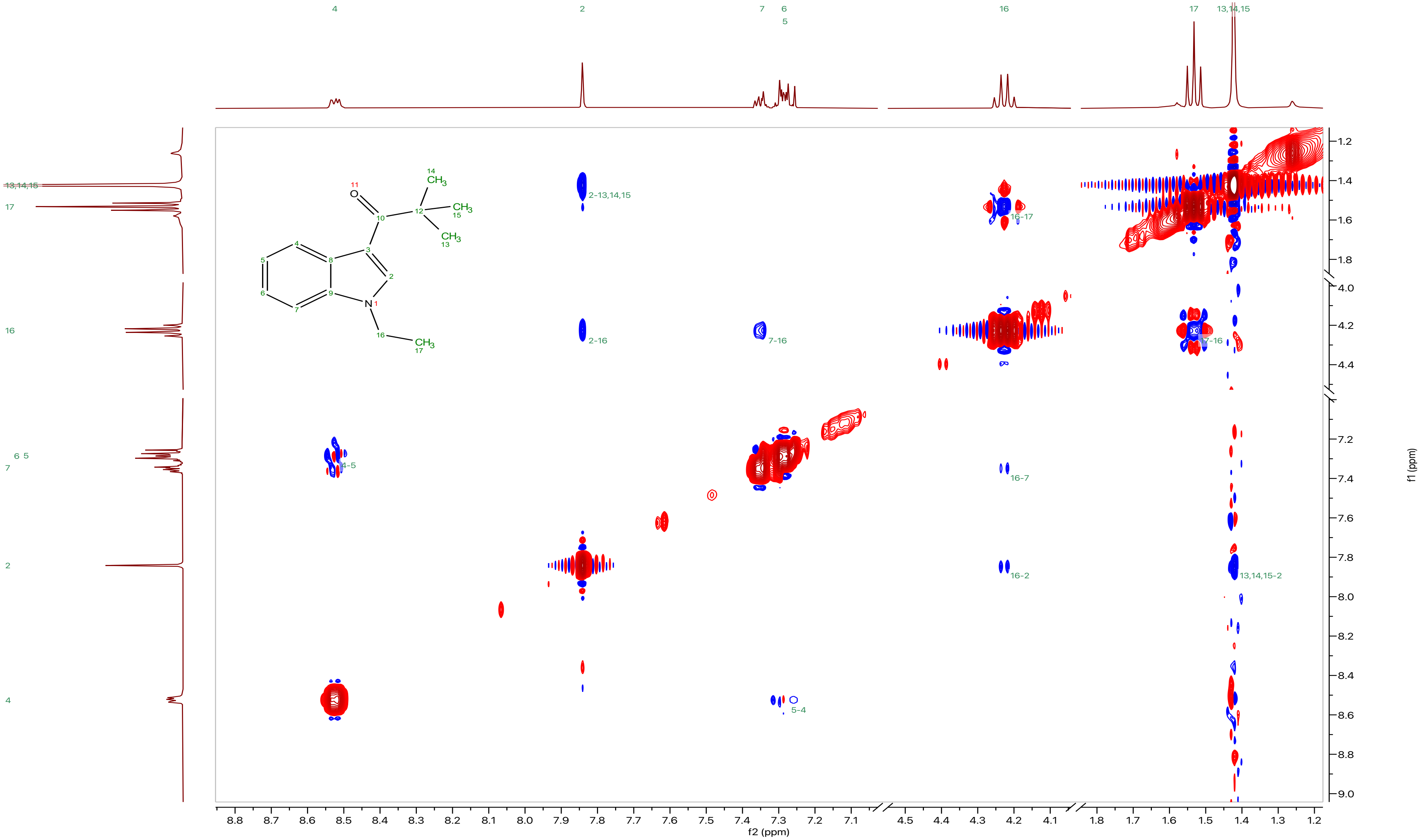

^{1}H-^{1}H NOESY (400 MHz, $CDCl_3$) of 1k

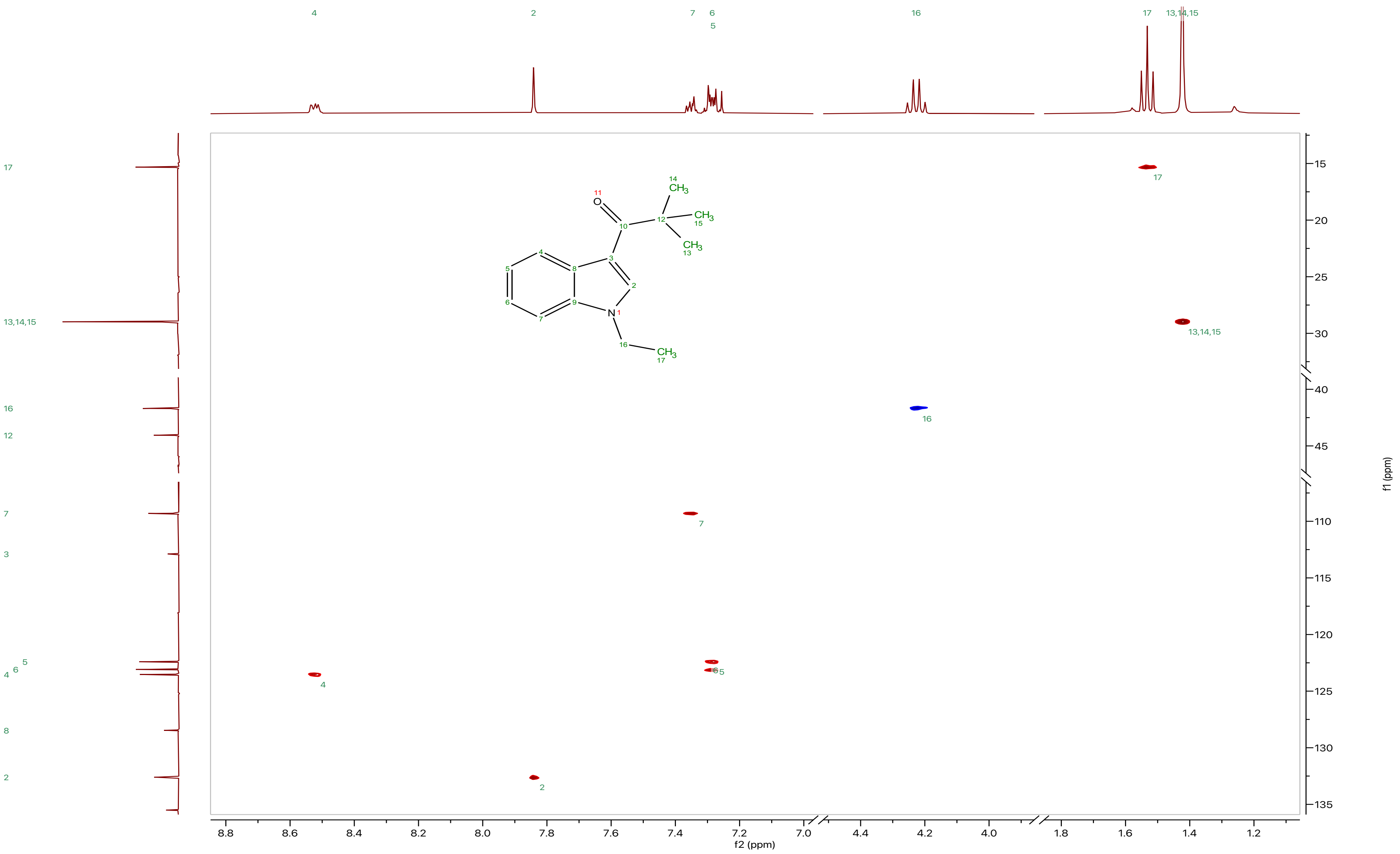

^{1}H-^{13}C{^{1}H} HSQC NMR (400/101 MHz, $CDCl_3$) of 1k

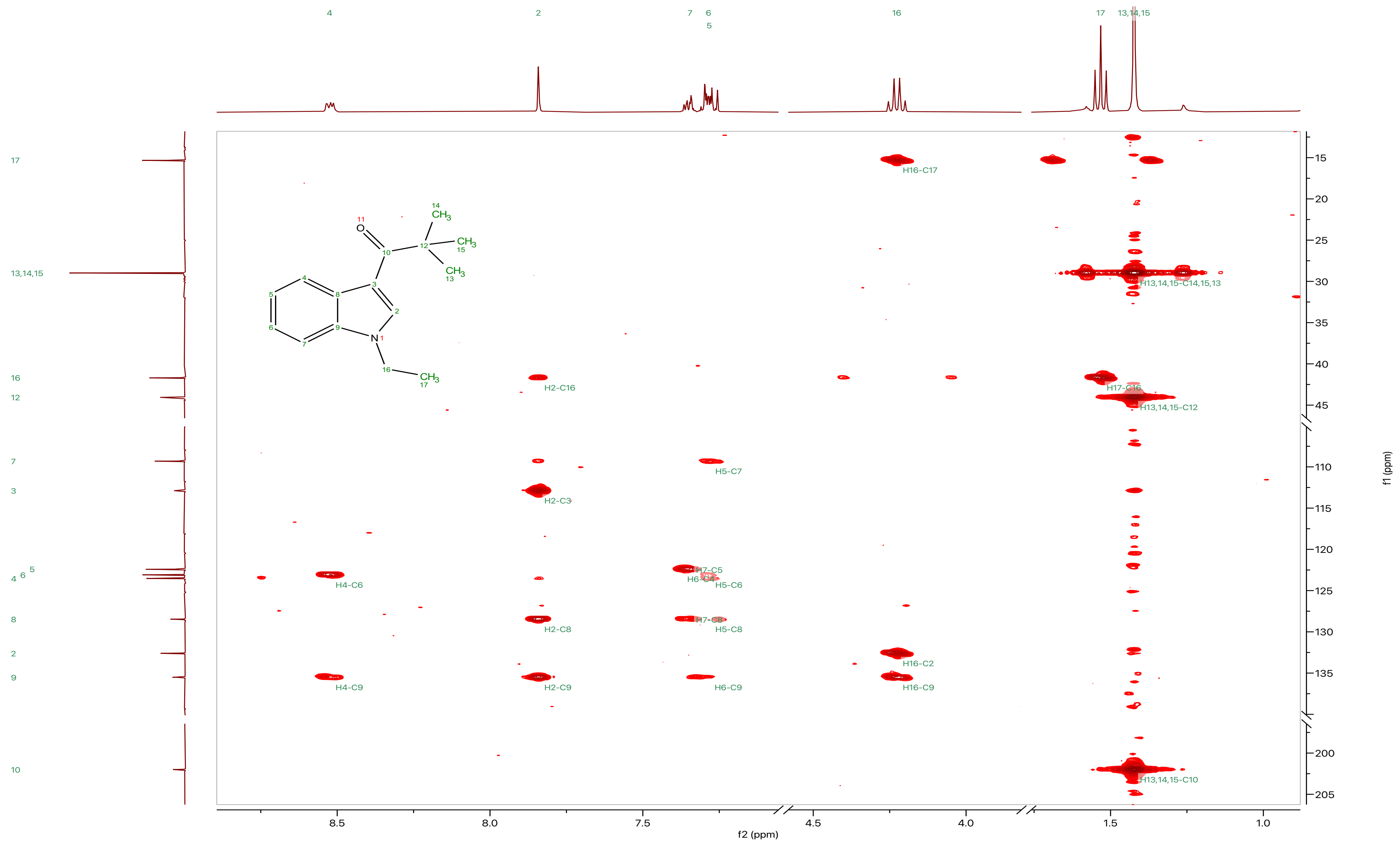

^{1}H-$^{13}C\{^{1}H\}$ HMBC NMR (400/101 MHz, $CDCl_3$) of 1k

2k

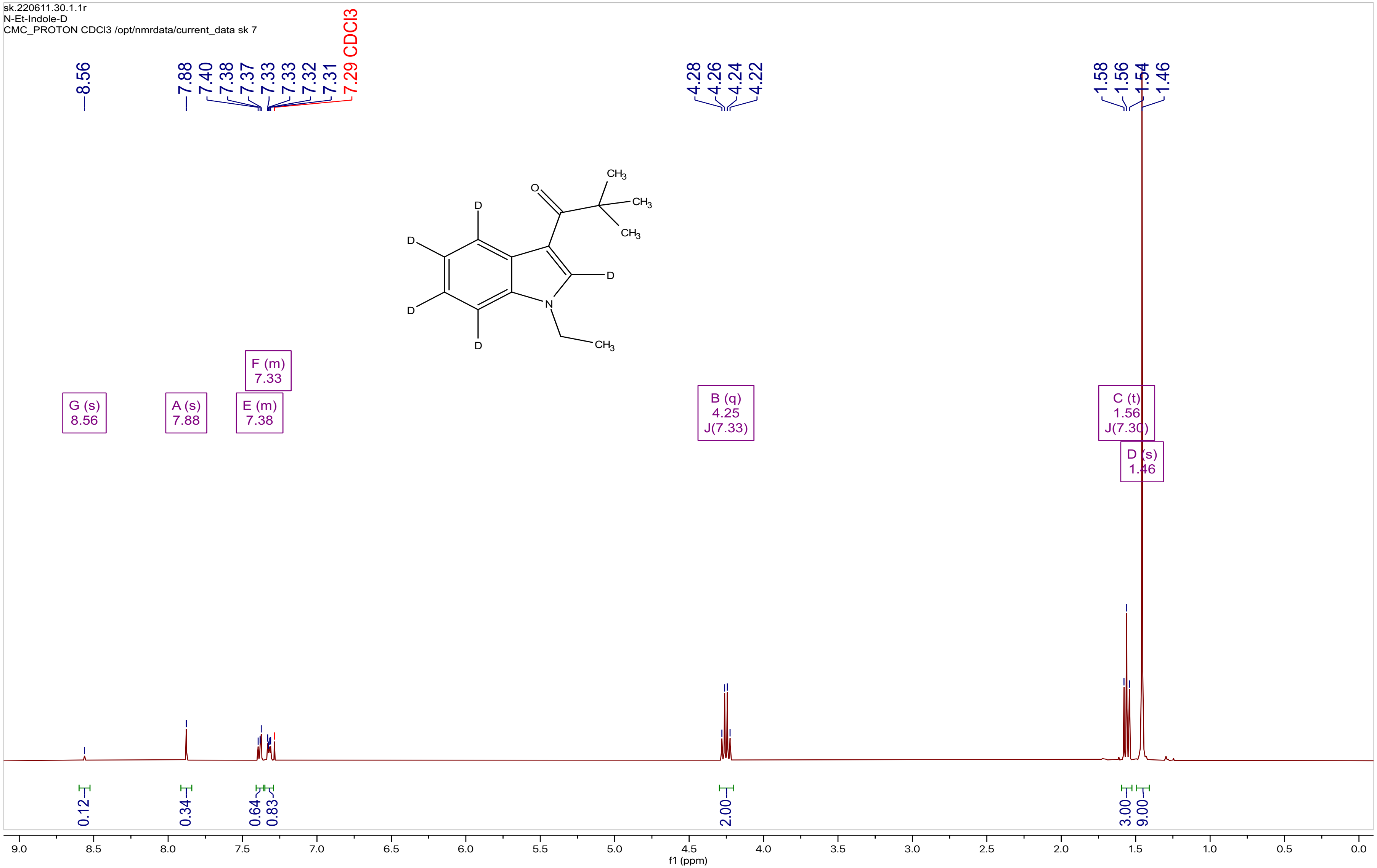

^1H NMR (400 MHz, $CDCl_3$) of 2k

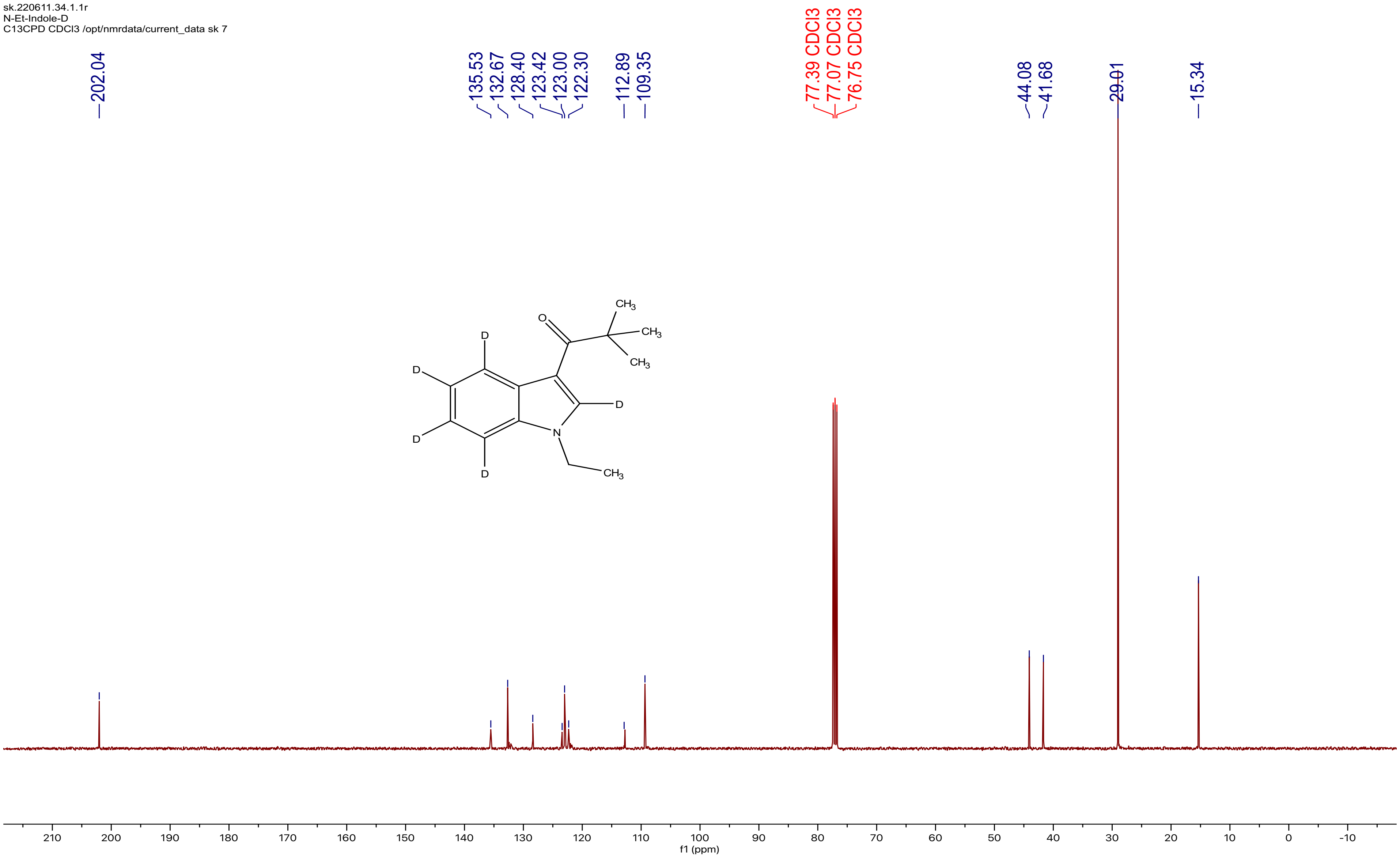

$^{13}C\{^1H\}$ NMR (101 MHz, $CDCl_3$) of 2k

1l

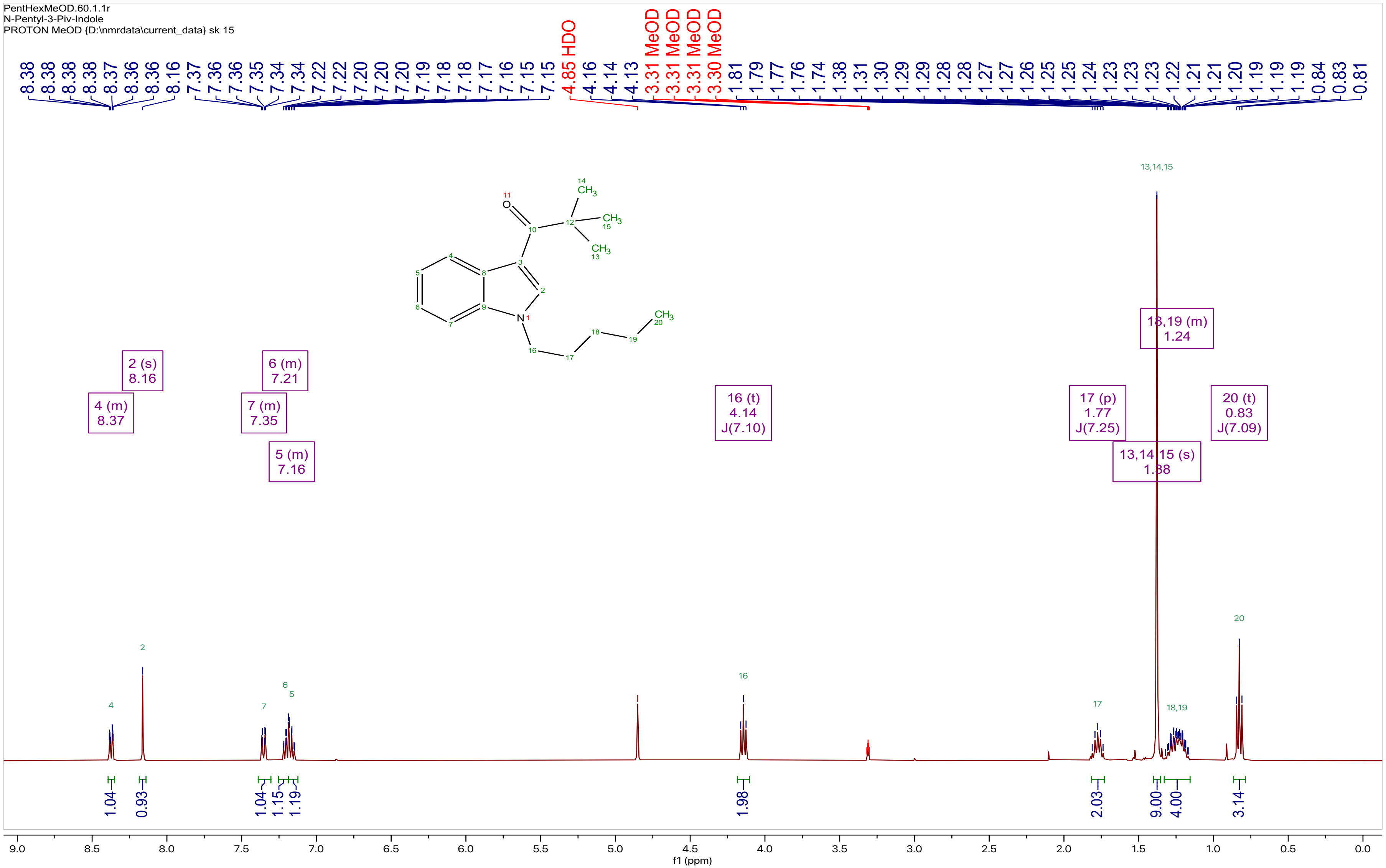

^{1}H NMR (400 MHz, MeOD) of 1l

PentHexMeOD.61.1.1r
N-Pentyl-3-Piv-Indole
C13CPD MeOD {D:\nmrdata\current_data} sk 15

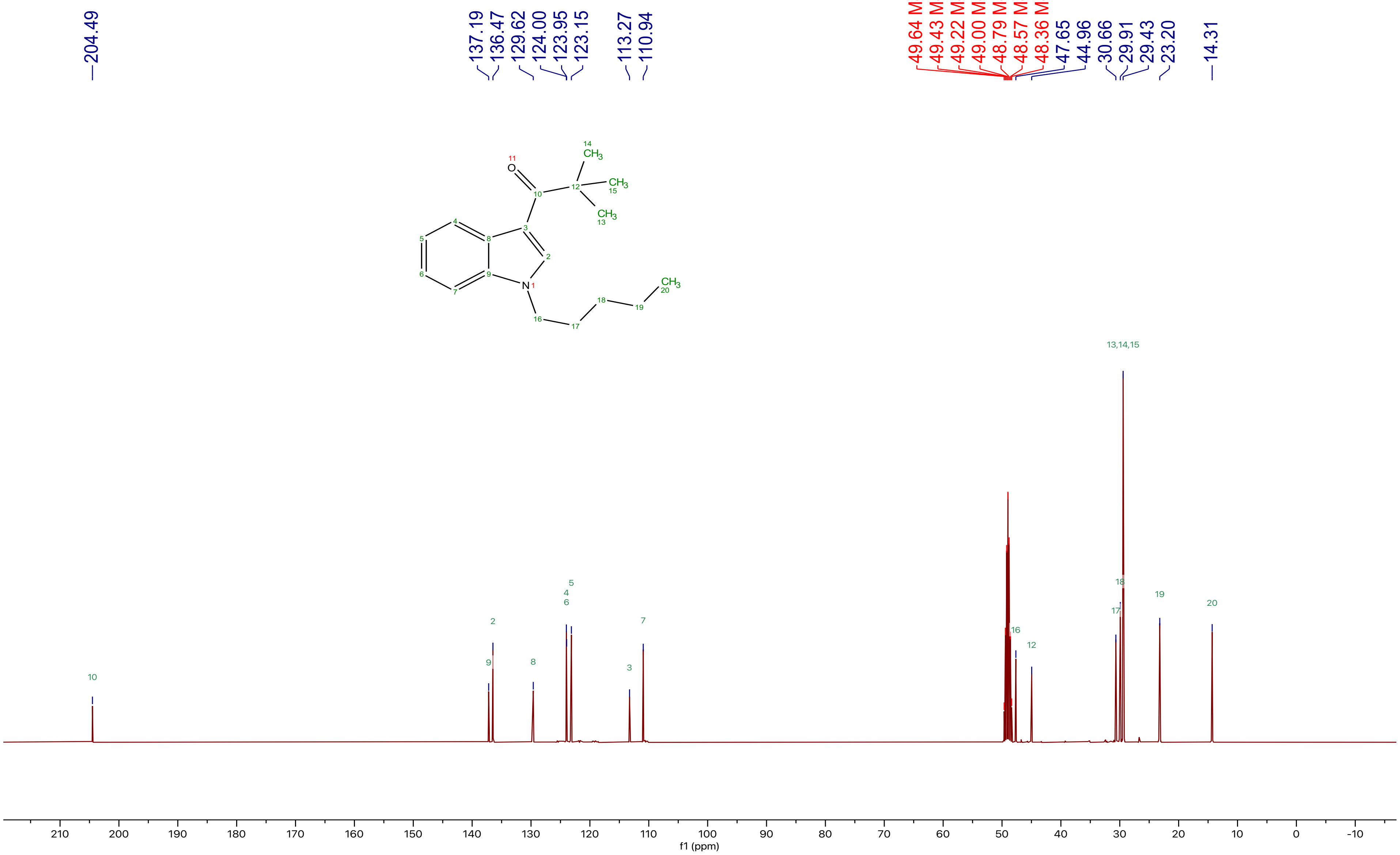

$^{13}C\{^{1}H\}$ NMR (101 MHz, MeOD) of 1l

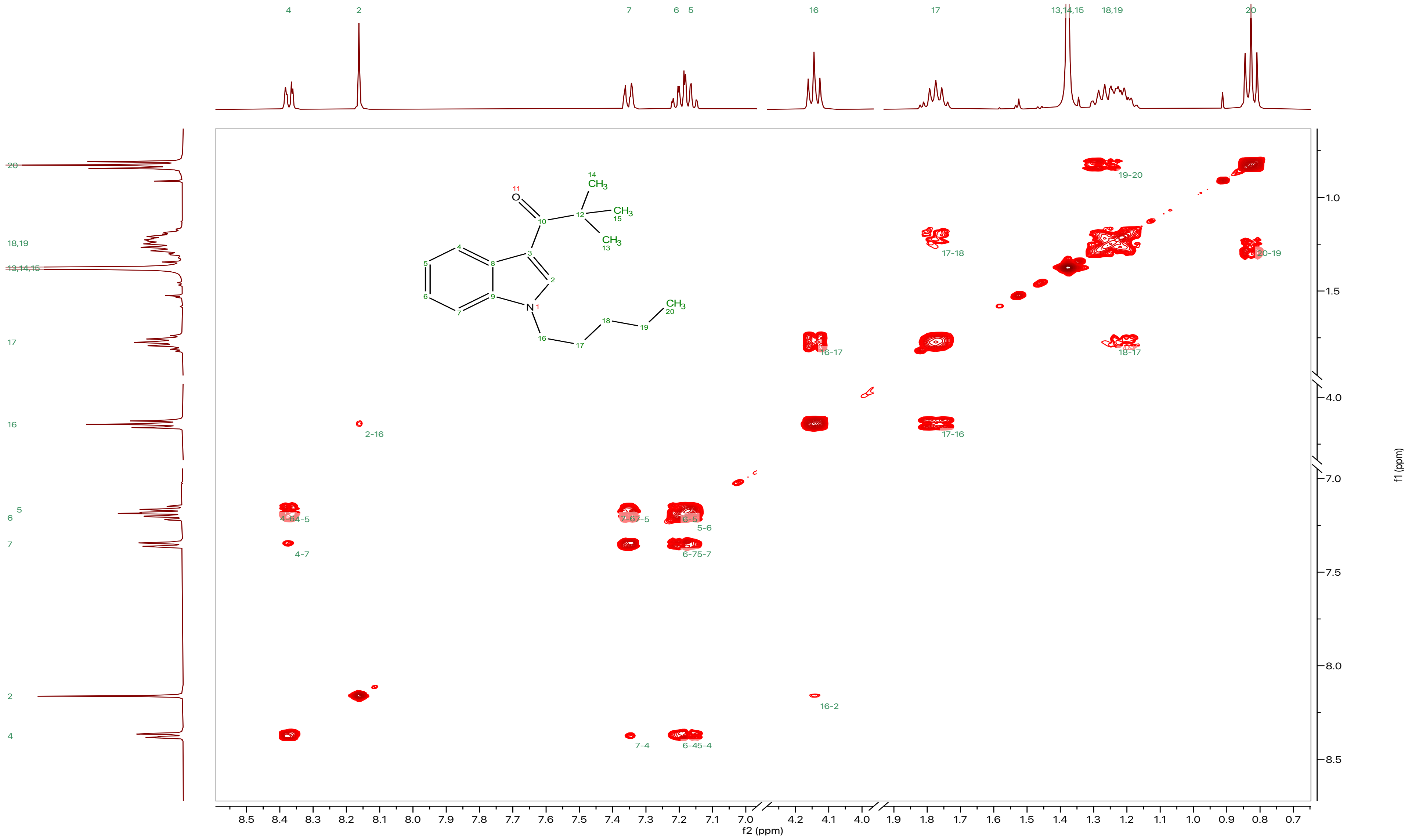

¹H-¹H COSY (400 MHz, MeOD) of 1l

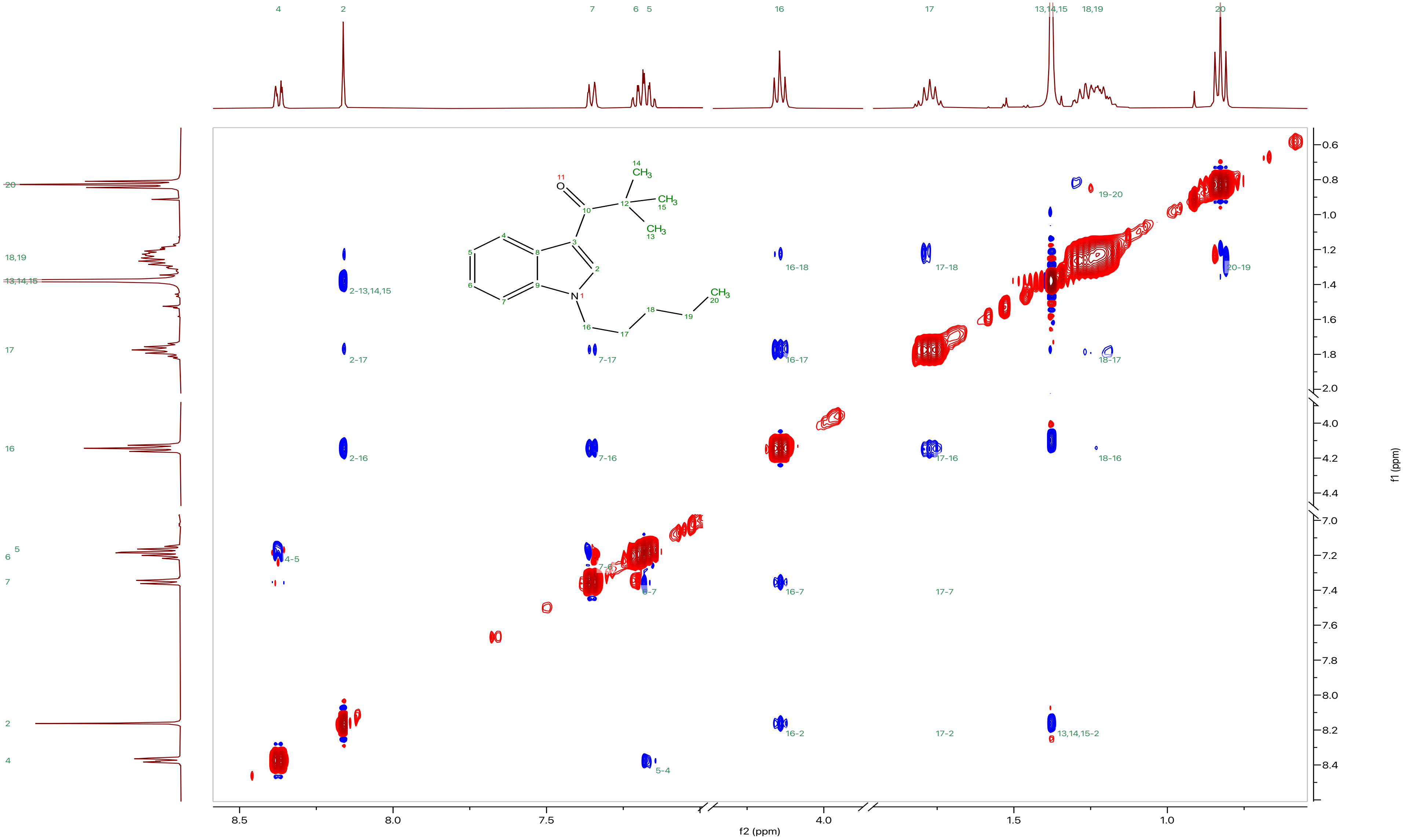

^{1}H-^{1}H NOESY (400 MHz, MeOD) of 1l

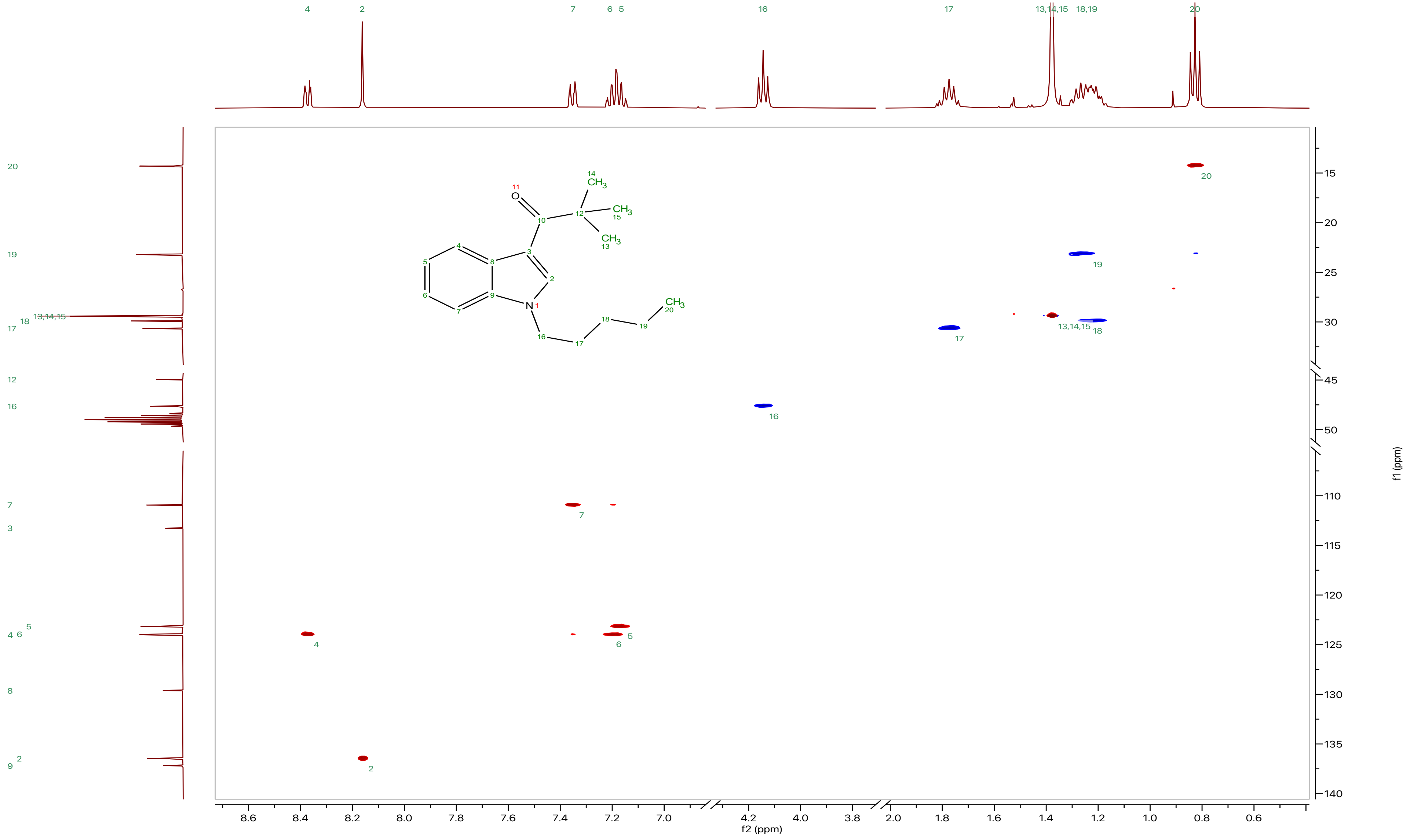

^{1}H-$^{13}C\{^{1}H\}$ HSQC NMR (400/101 MHz, MeOD) of 1l

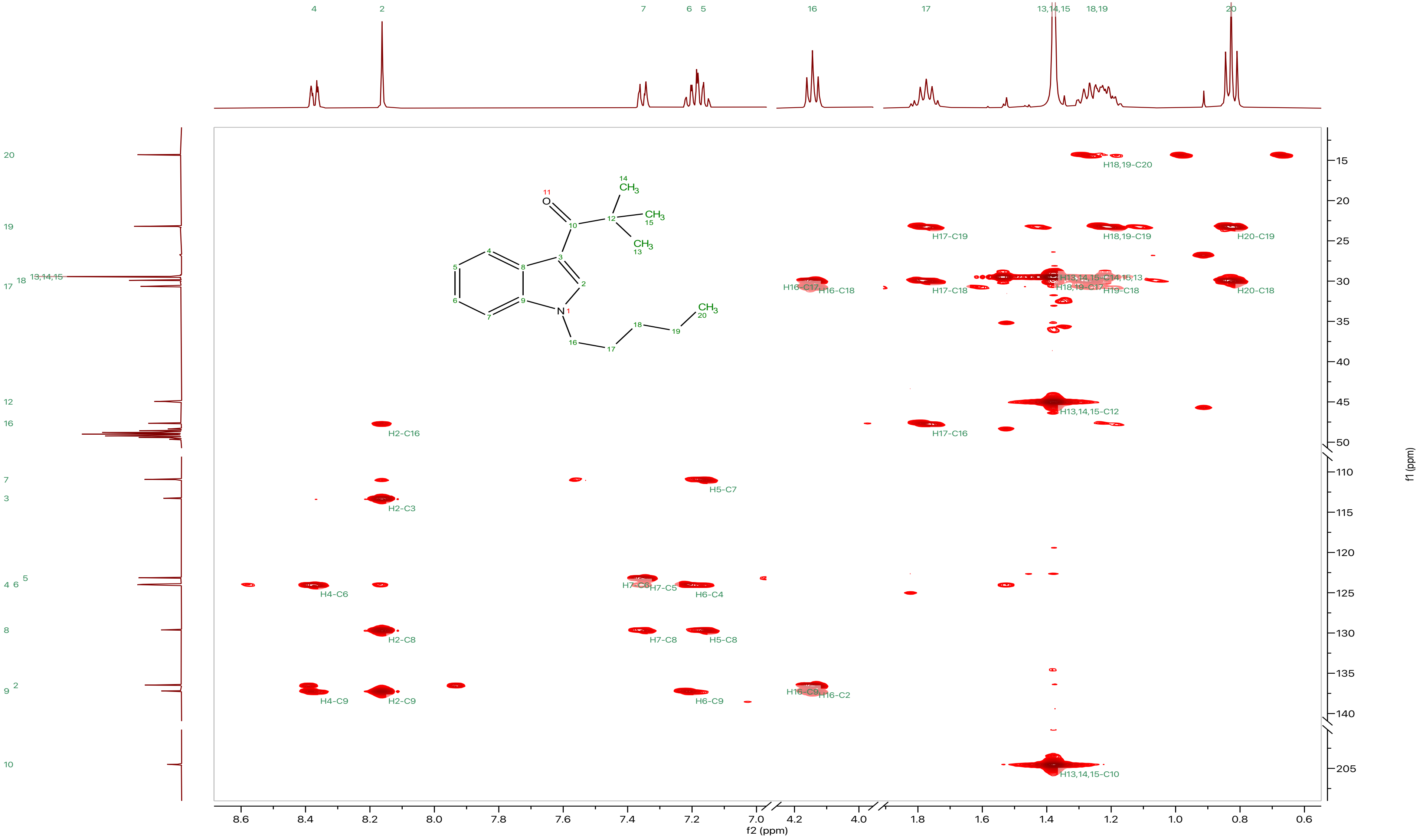

$^{1}H-^{13}C\{^{1}H\}$ HMBC NMR (400/101 MHz, MeOD) of 1l

2l

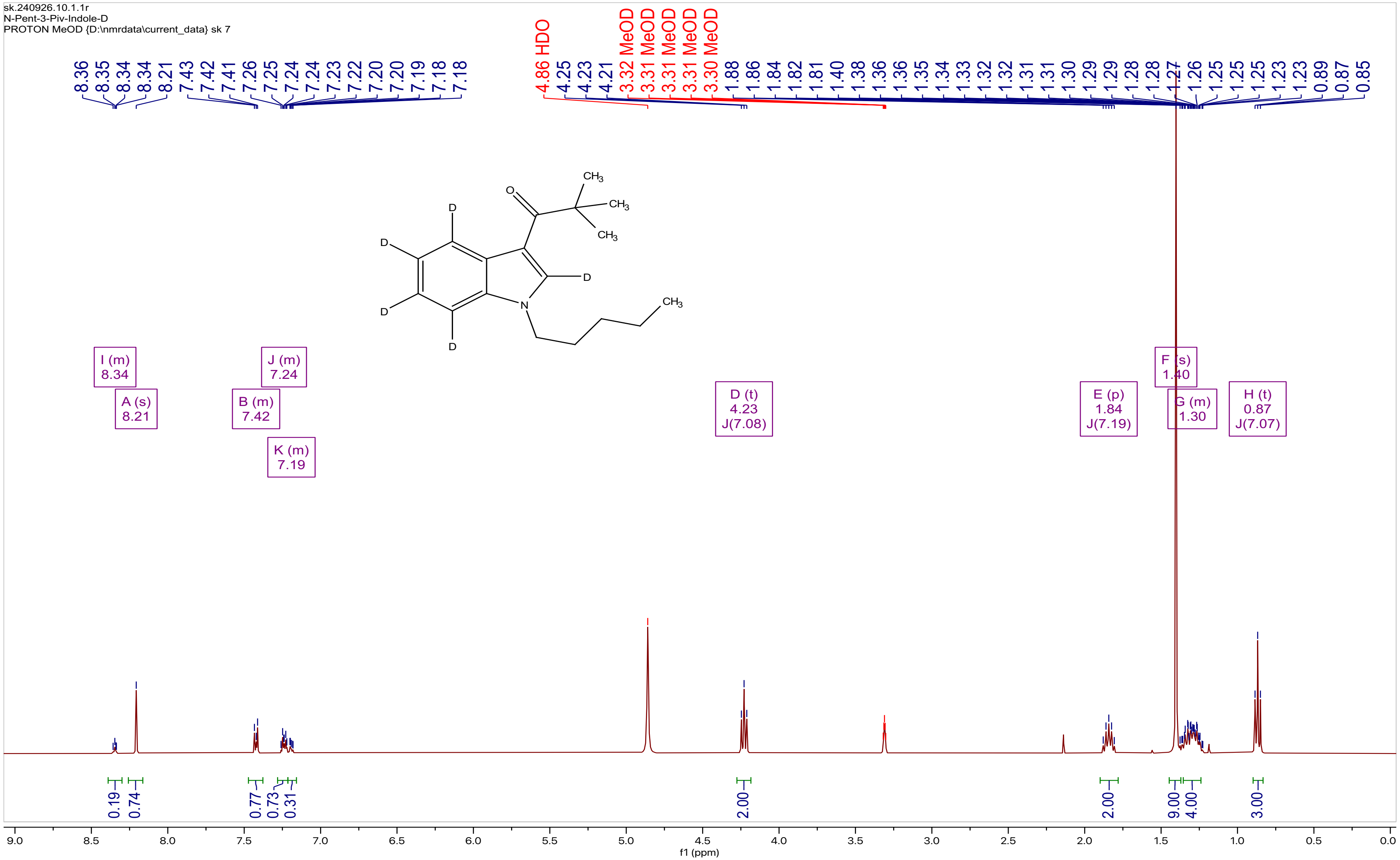

^{1}H NMR (400 MHz, MeOD) of 2l

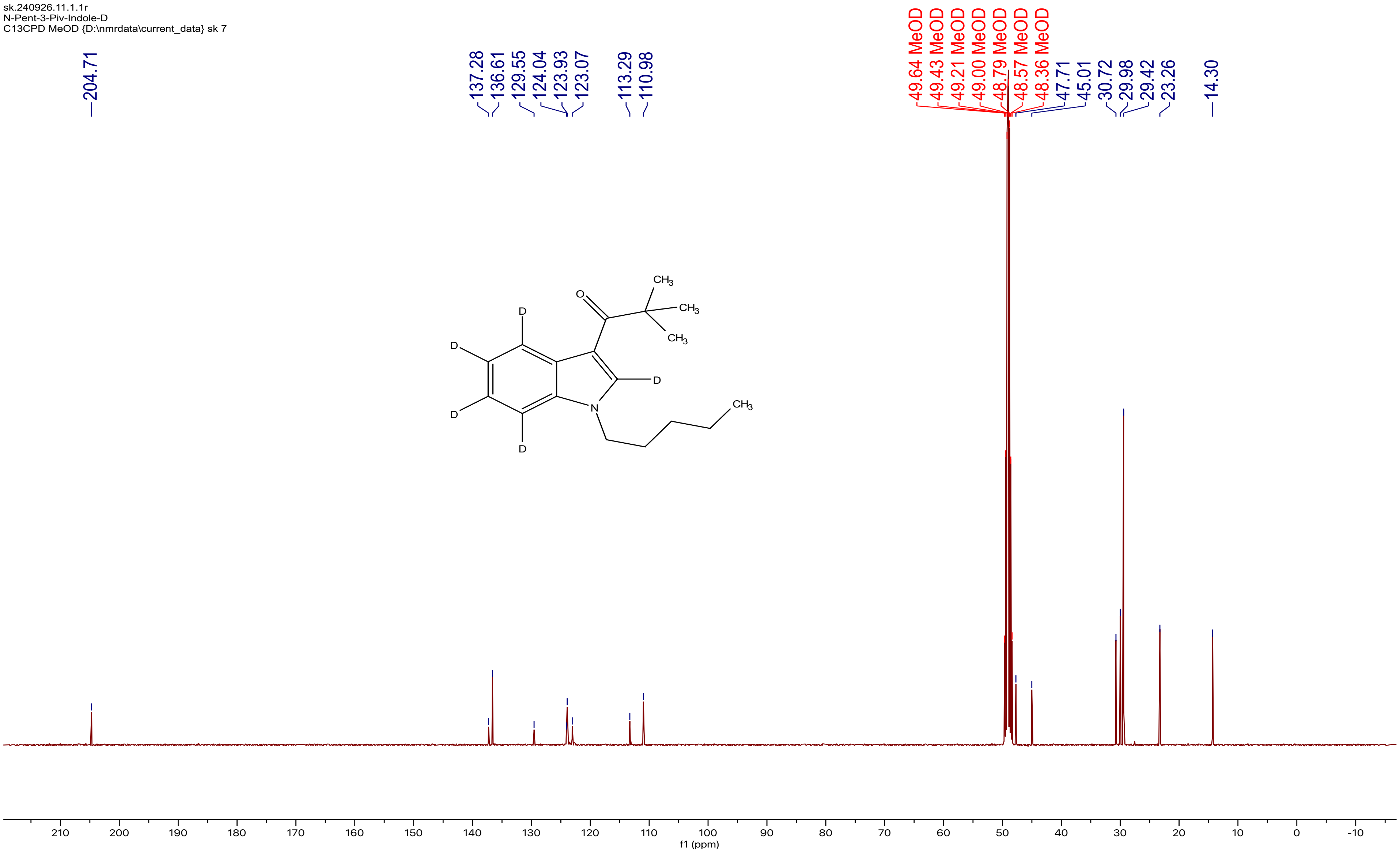

$^{13}C\{^{1}H\}$ NMR (101 MHz, MeOD) of 2l

1m

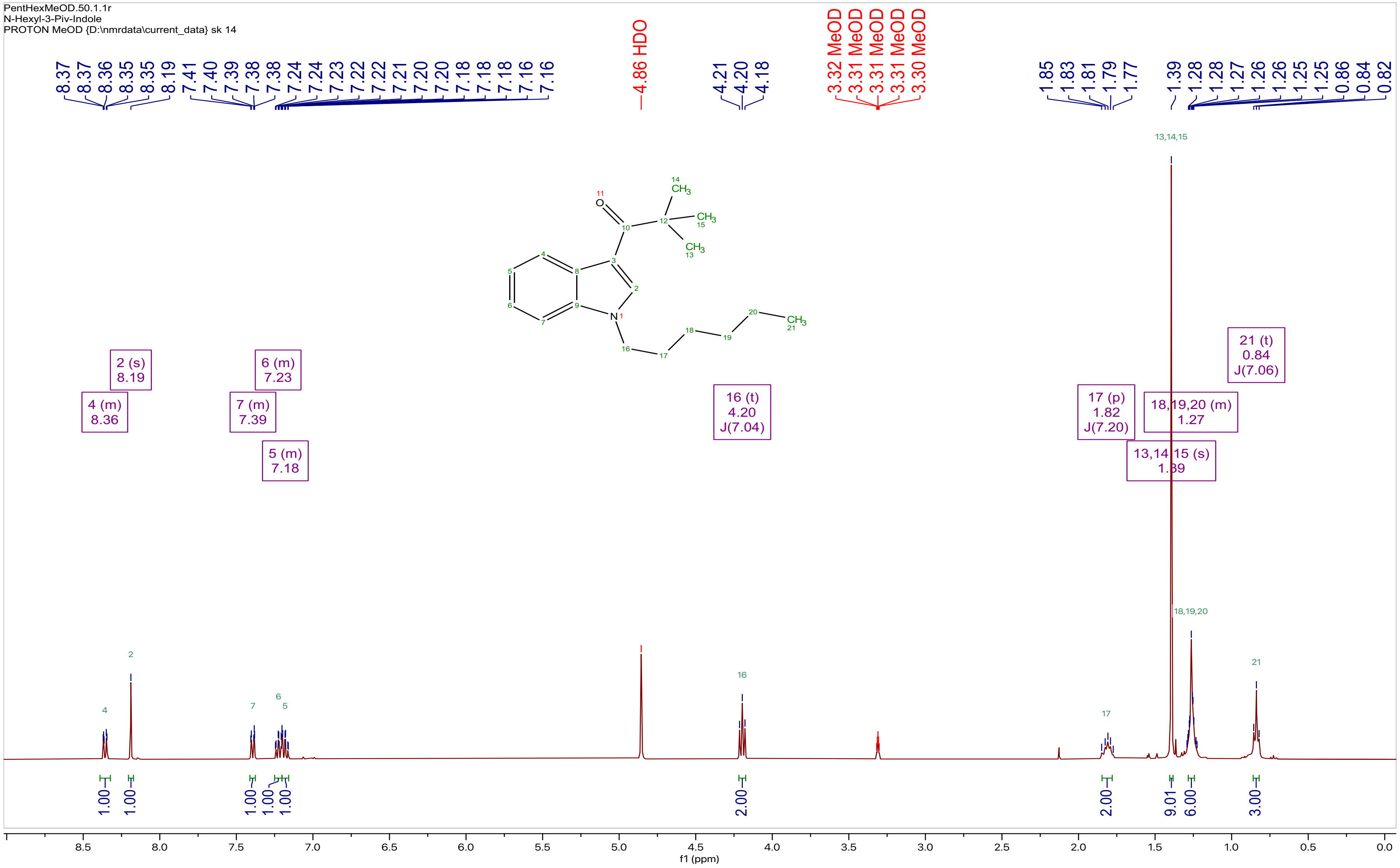

¹H NMR (400 MHz, MeOD) of 1m

PentHexMeOD.51.1.1r
N-Hexyl-3-Piv-Indole
C13CPD MeOD {D:\nmrdata\current_data} sk 14

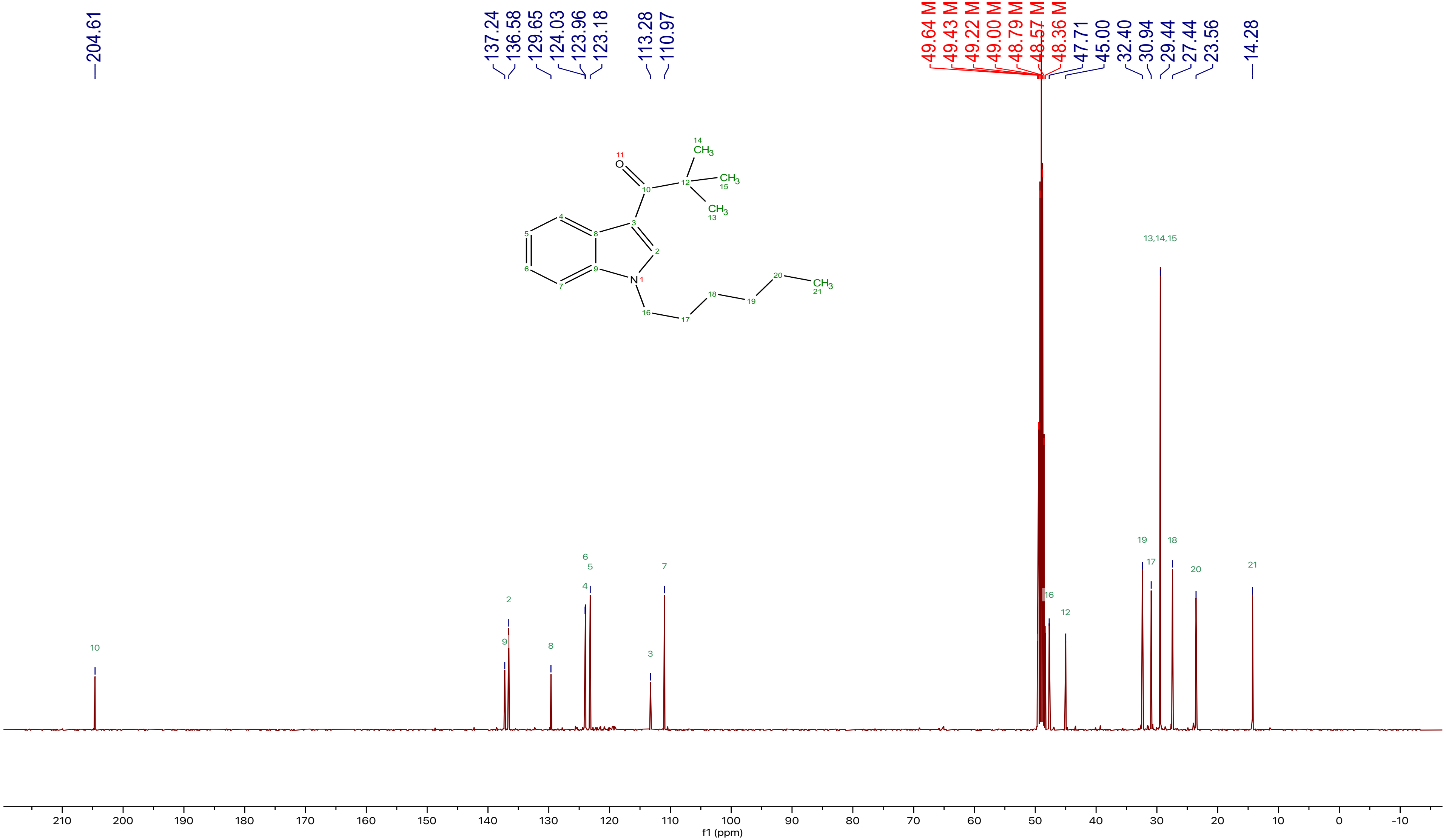

$^{13}C\{^{1}H\}$ NMR (101 MHz, MeOD) of 1m

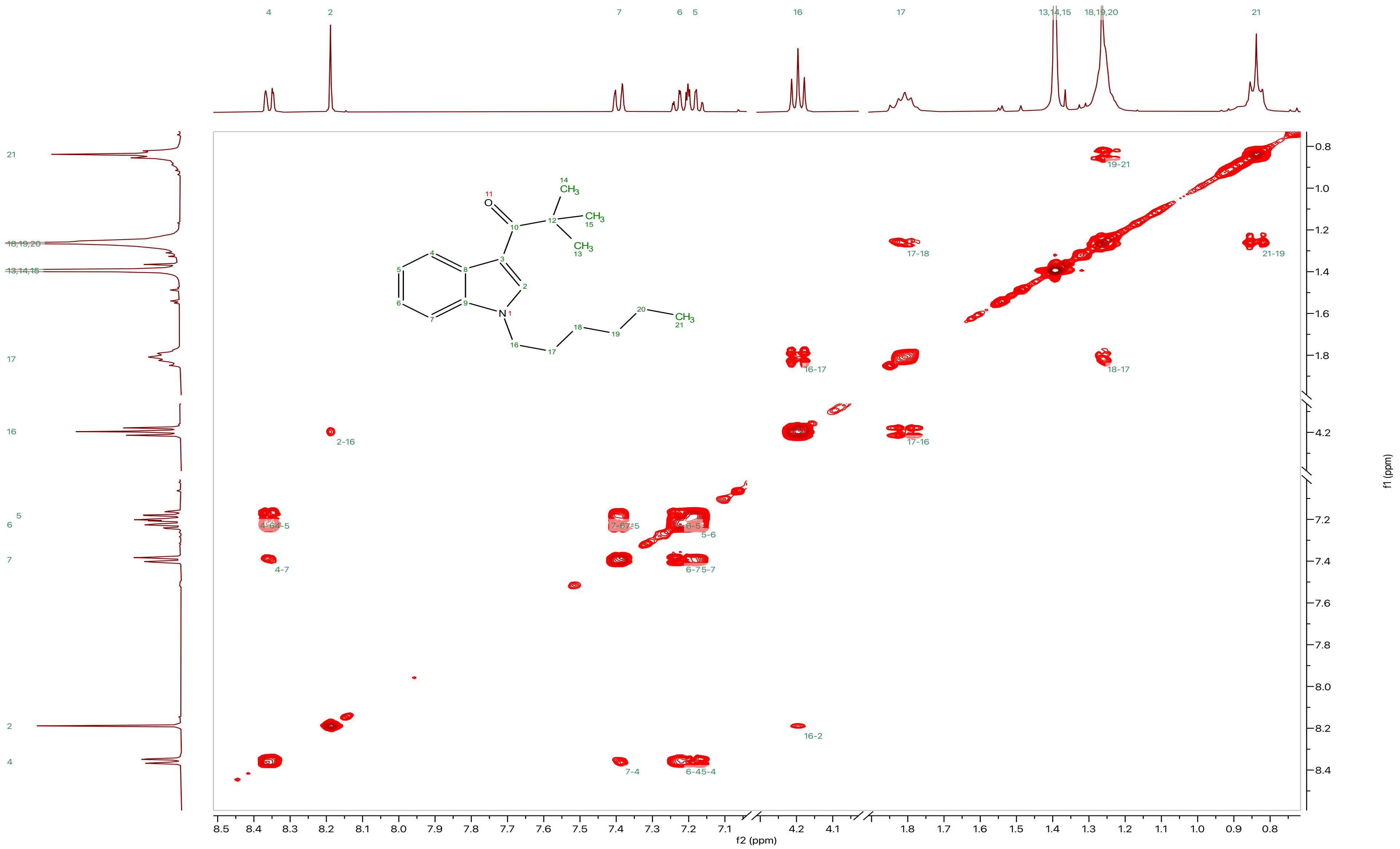

^{1}H-^{1}H COSY (400 MHz, MeOD) of 1m

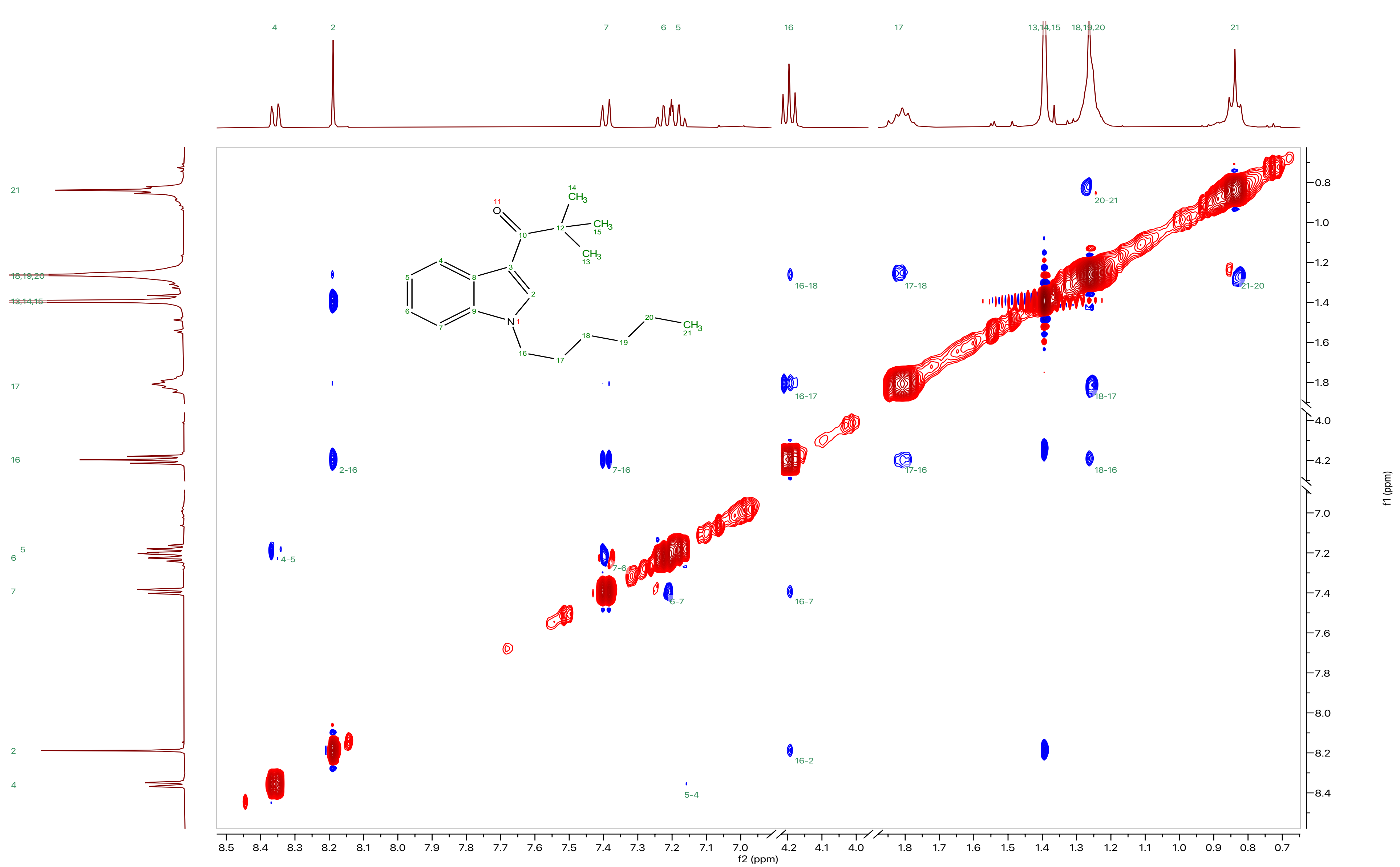

^1H-^1H NOESY (400 MHz, MeOD) of 1m

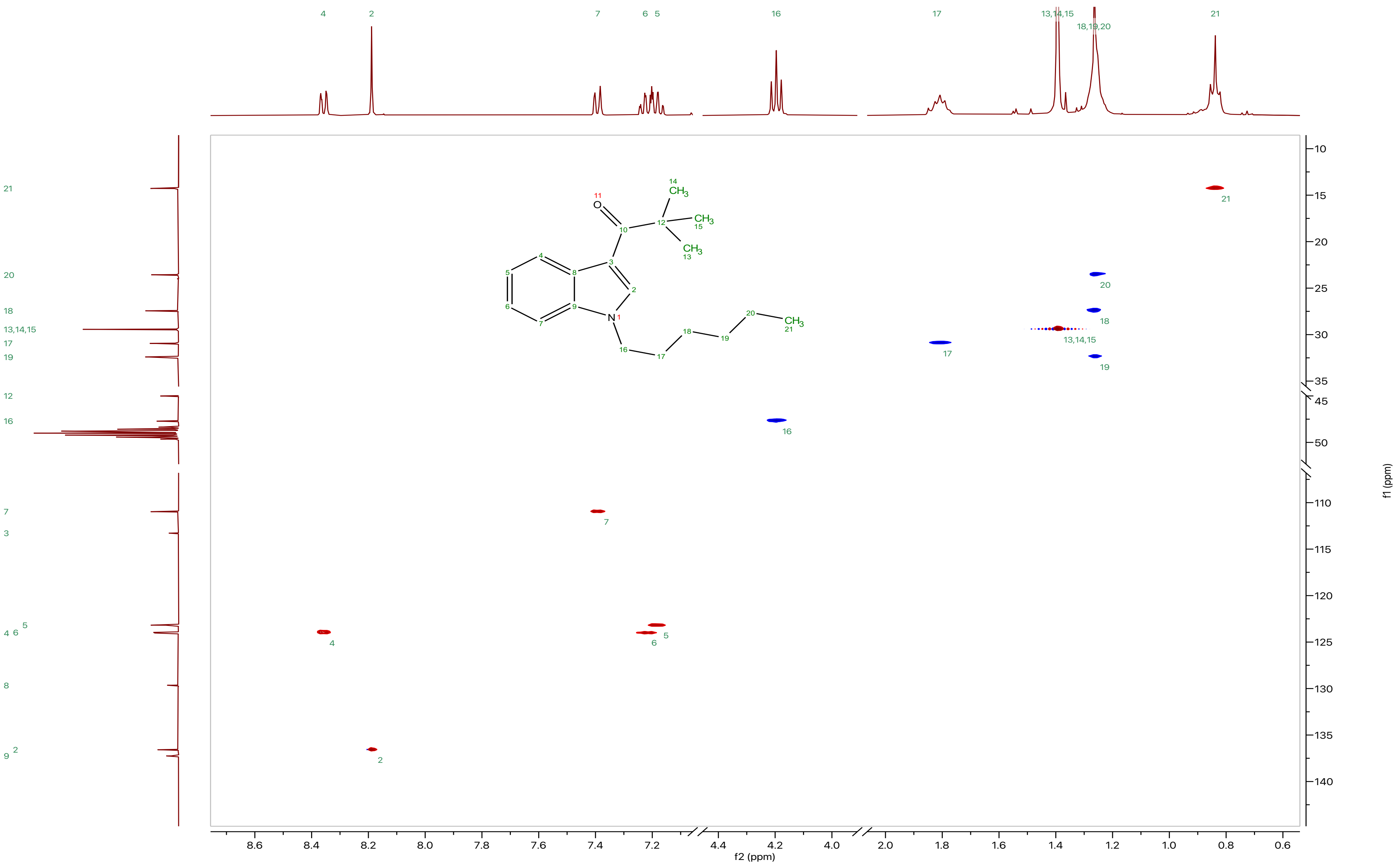

^{1}H-$^{13}C\{^{1}H\}$ HSQC NMR (400/101 MHz, MeOD) of 1m

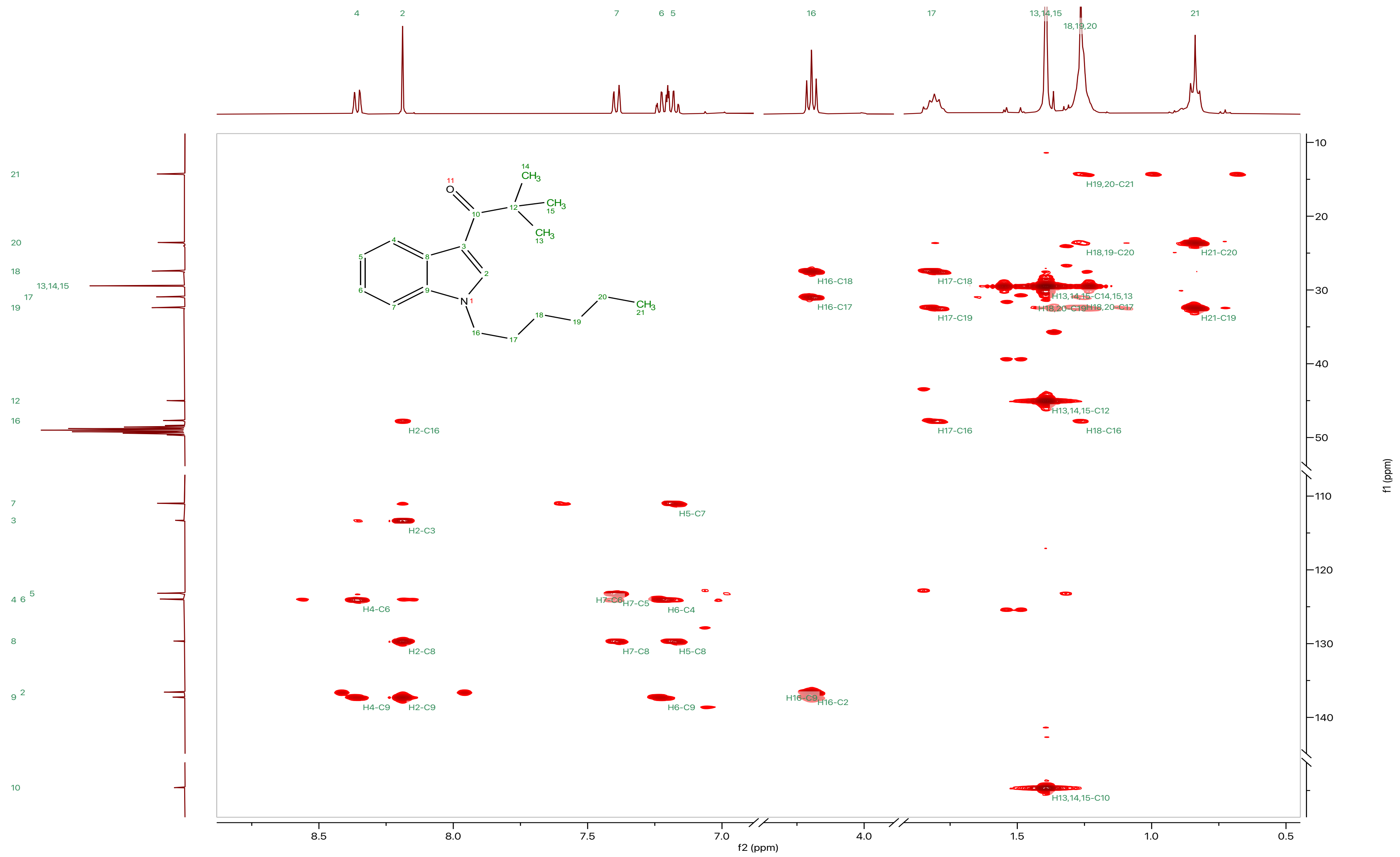

^{1}H-$^{13}C\{^{1}H\}$ HMBC NMR (400/101 MHz, MeOD) of 1m

2m

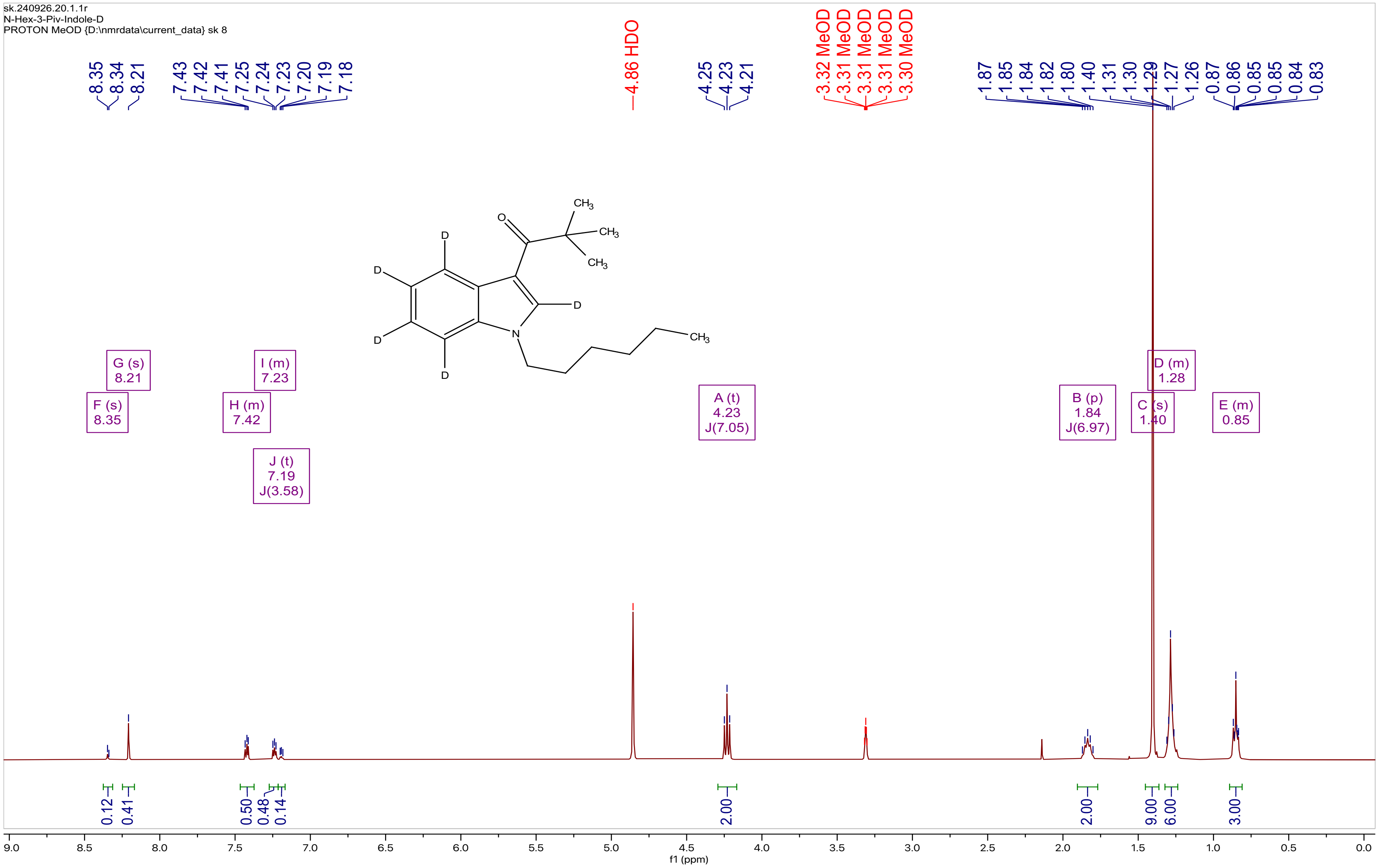

^{1}H NMR (400 MHz, MeOD) of 2m

sk.240926.21.1.1r
N-Hex-3-Piv-Indole-D
C13CPD MeOD {D:\nmrdata\current_data} sk 8

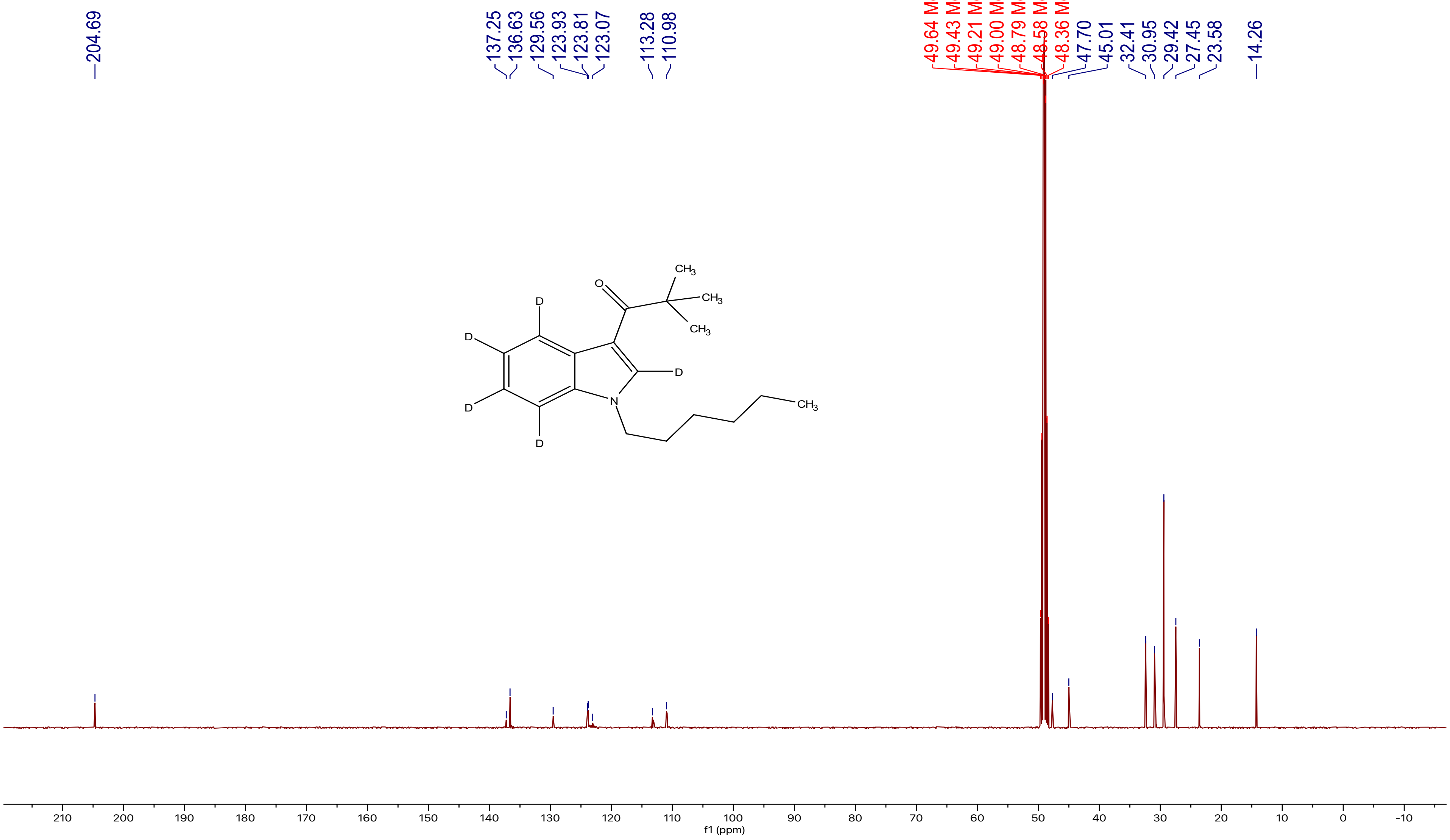

$^{13}C\{^1H\}$ NMR (101 MHz, MeOD) of 2m

1n

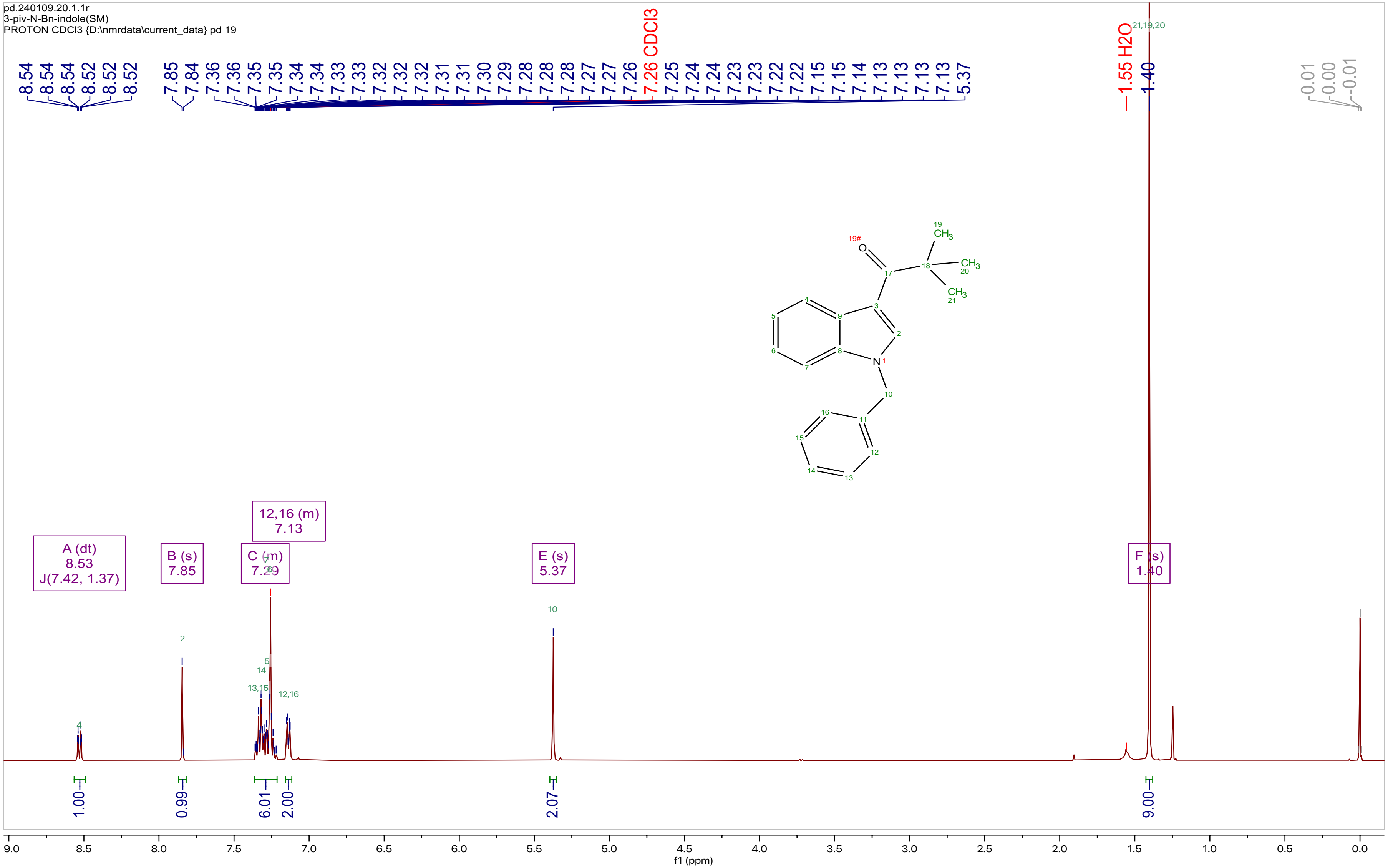

^{1}H NMR (400 MHz, $CDCl_3$) of 1n

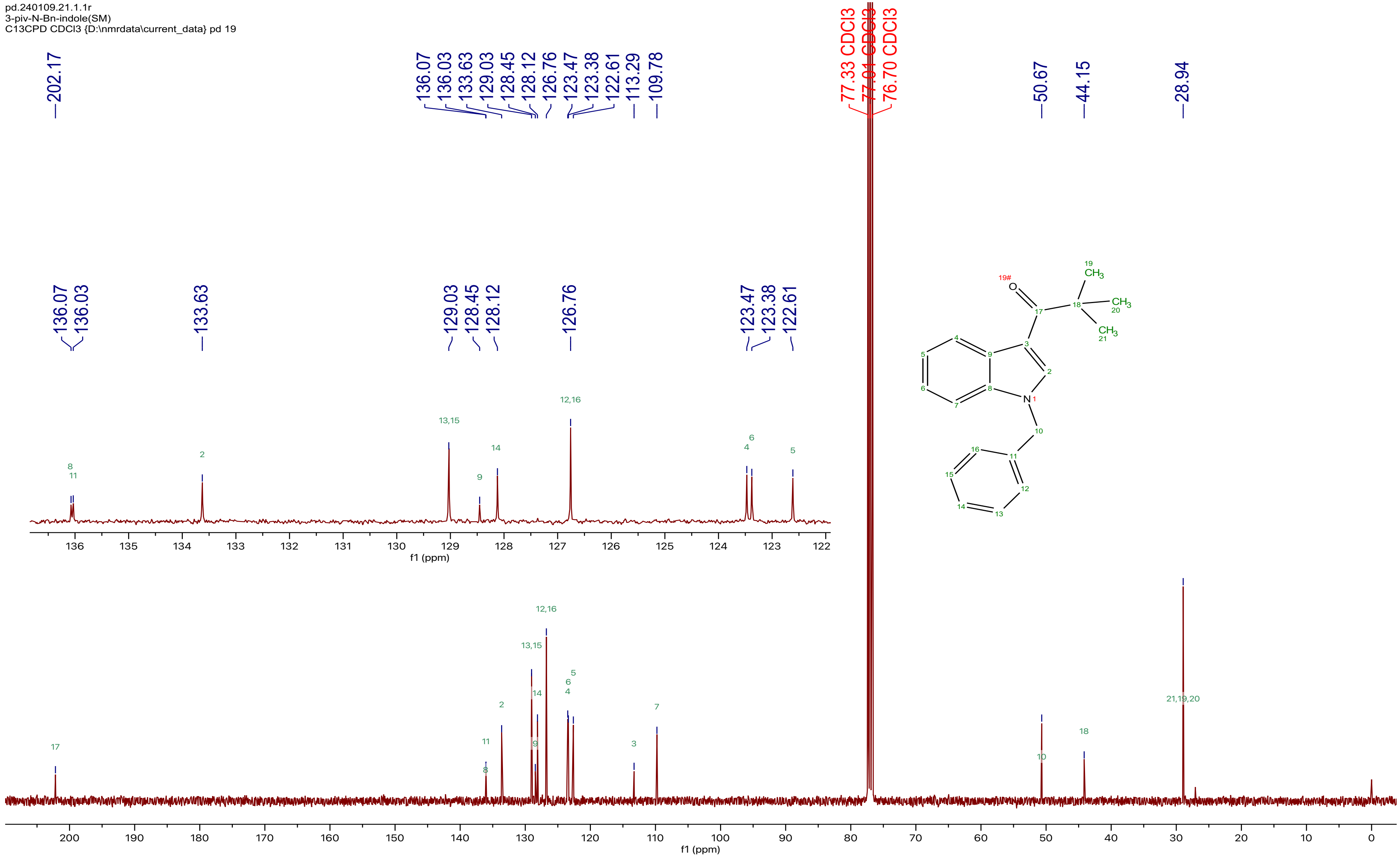

$^{13}C\{^{1}H\}$ NMR (101 MHz, $CDCl_3$) of 1n

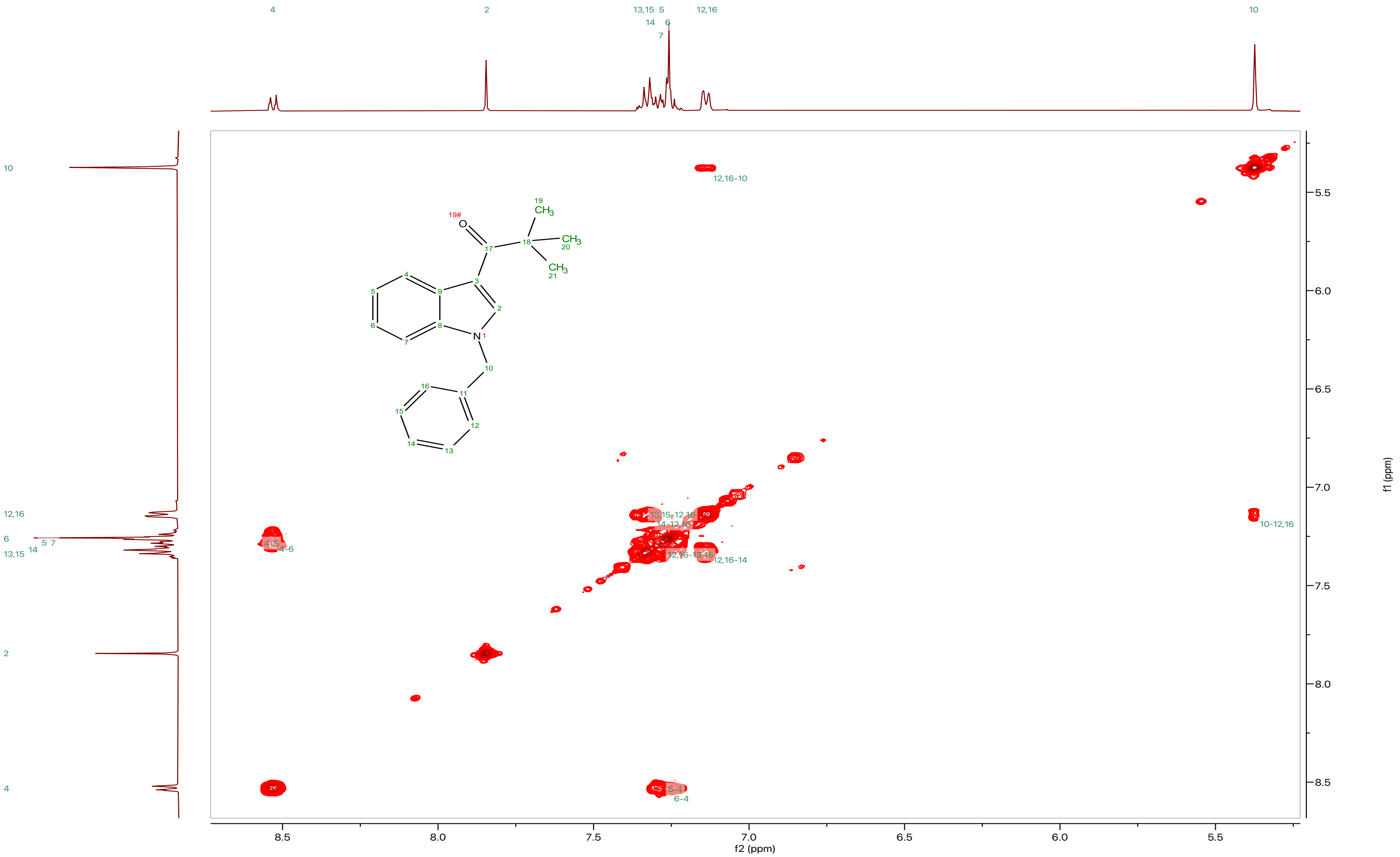

^1H-^1H COSY (400 MHz, $CDCl_3$) of 1n

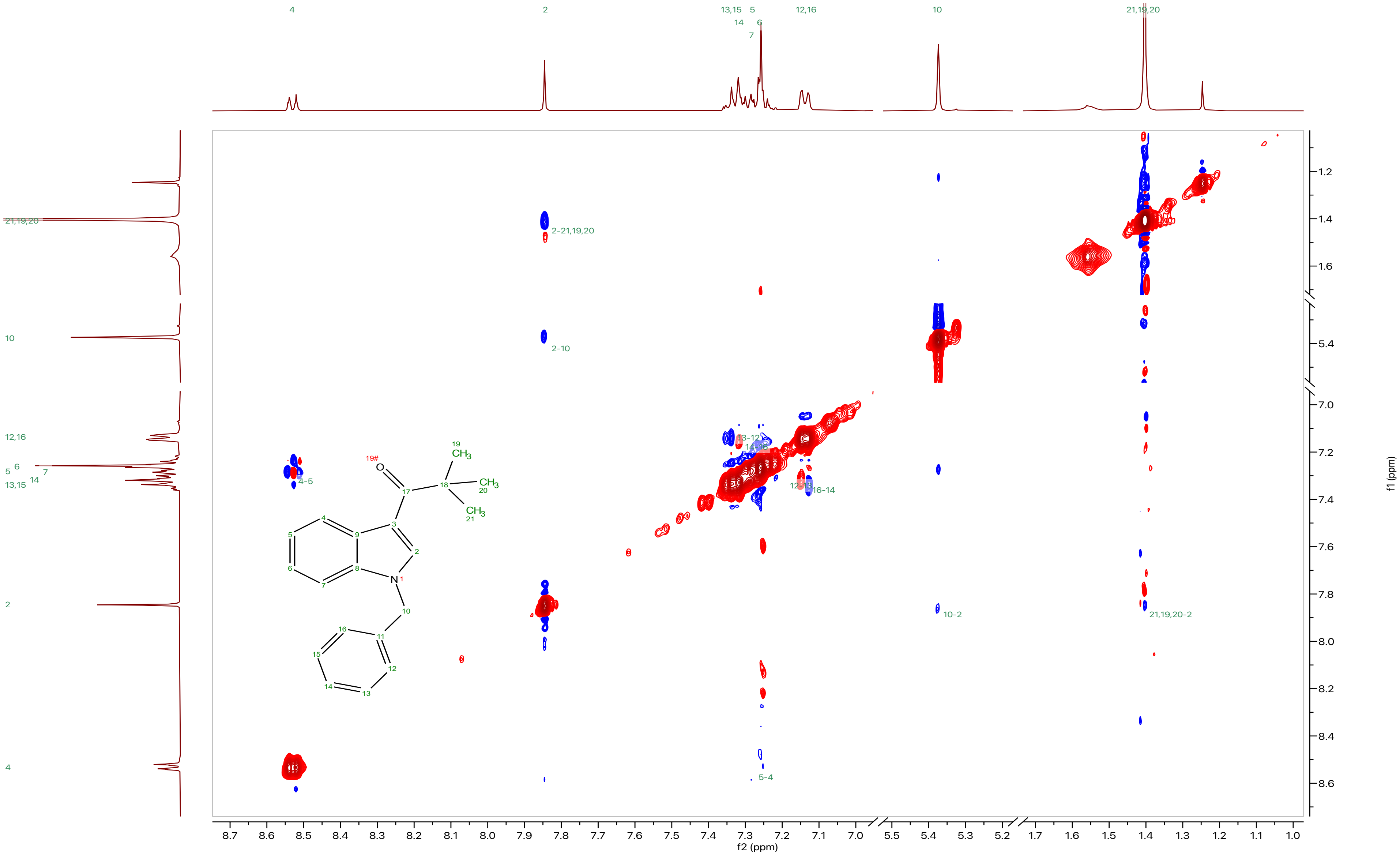

^{1}H-^{1}H NOESY (400 MHz, $CDCl_3$) of 1n

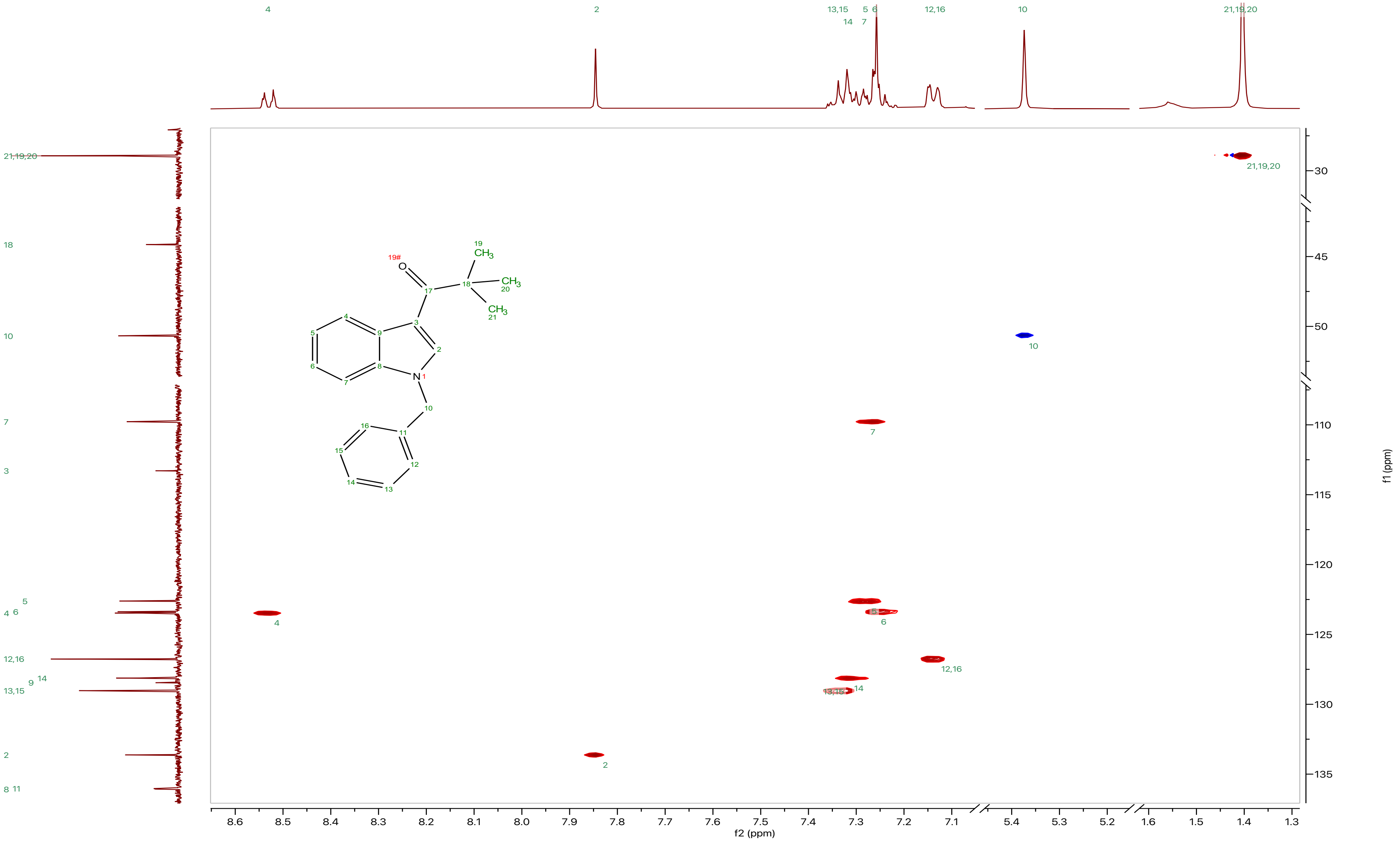

^{1}H-$^{13}C\{^{1}H\}$ HSQC NMR (400/101 MHz, $CDCl_3$) of 1n

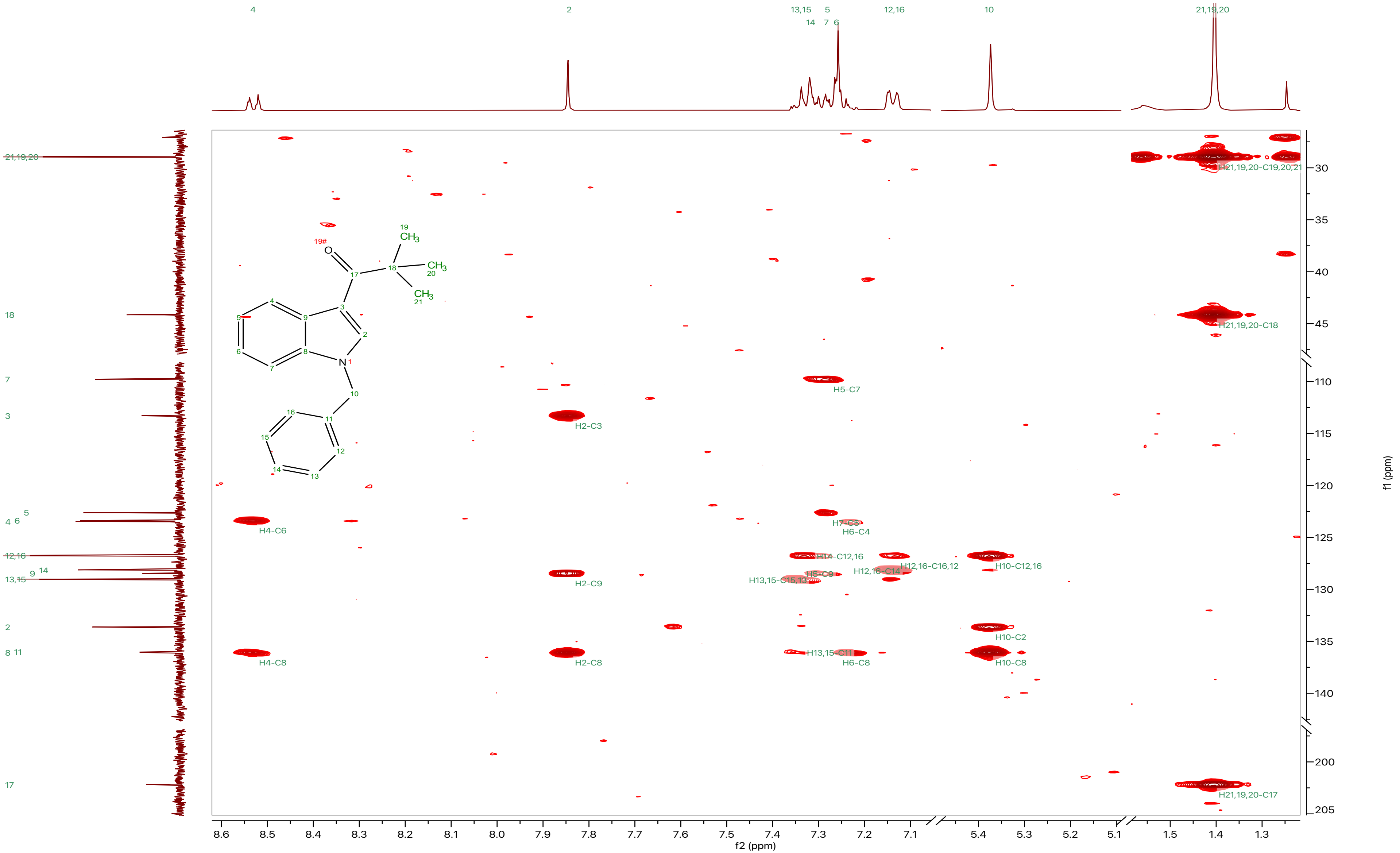

^{1}H-^{13}C{^{1}H} HMBC NMR (400/101 MHz, $CDCl_3$) of 1n

2n

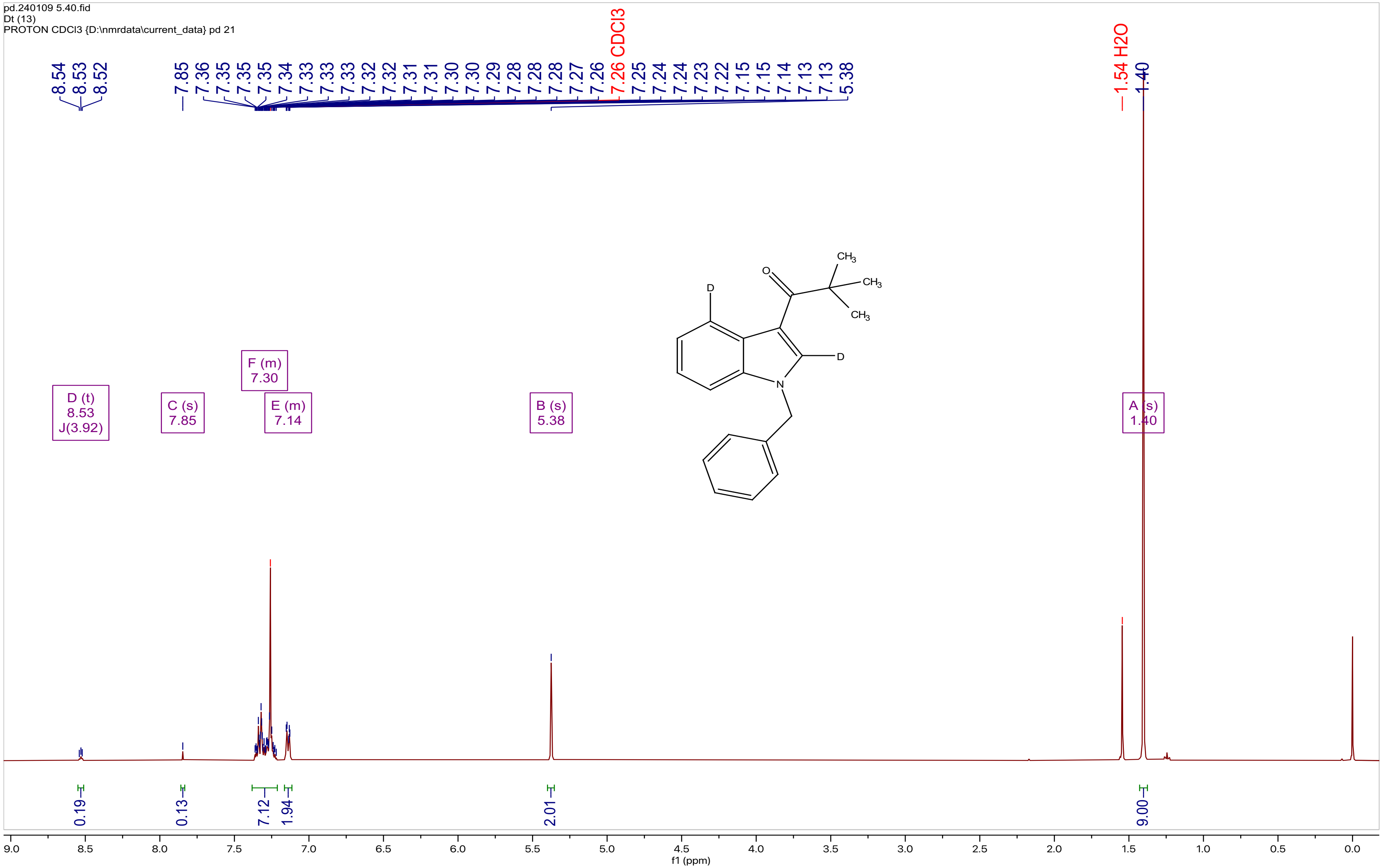

^{1}H NMR (400 MHz, $CDCl_3$) of 2n

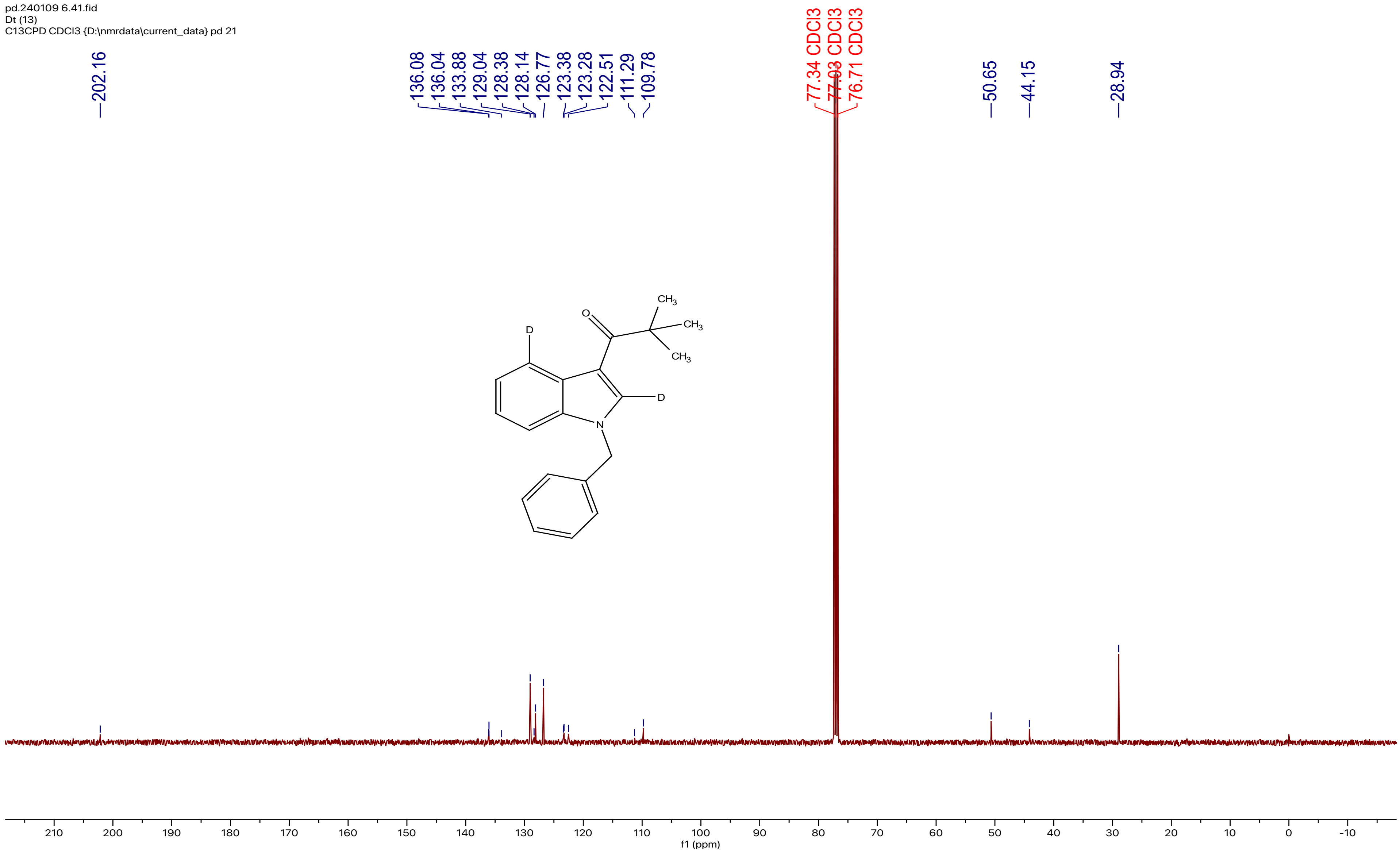

$^{13}C\{^1H\}$ NMR (101 MHz, $CDCl_3$) of 2n

1o

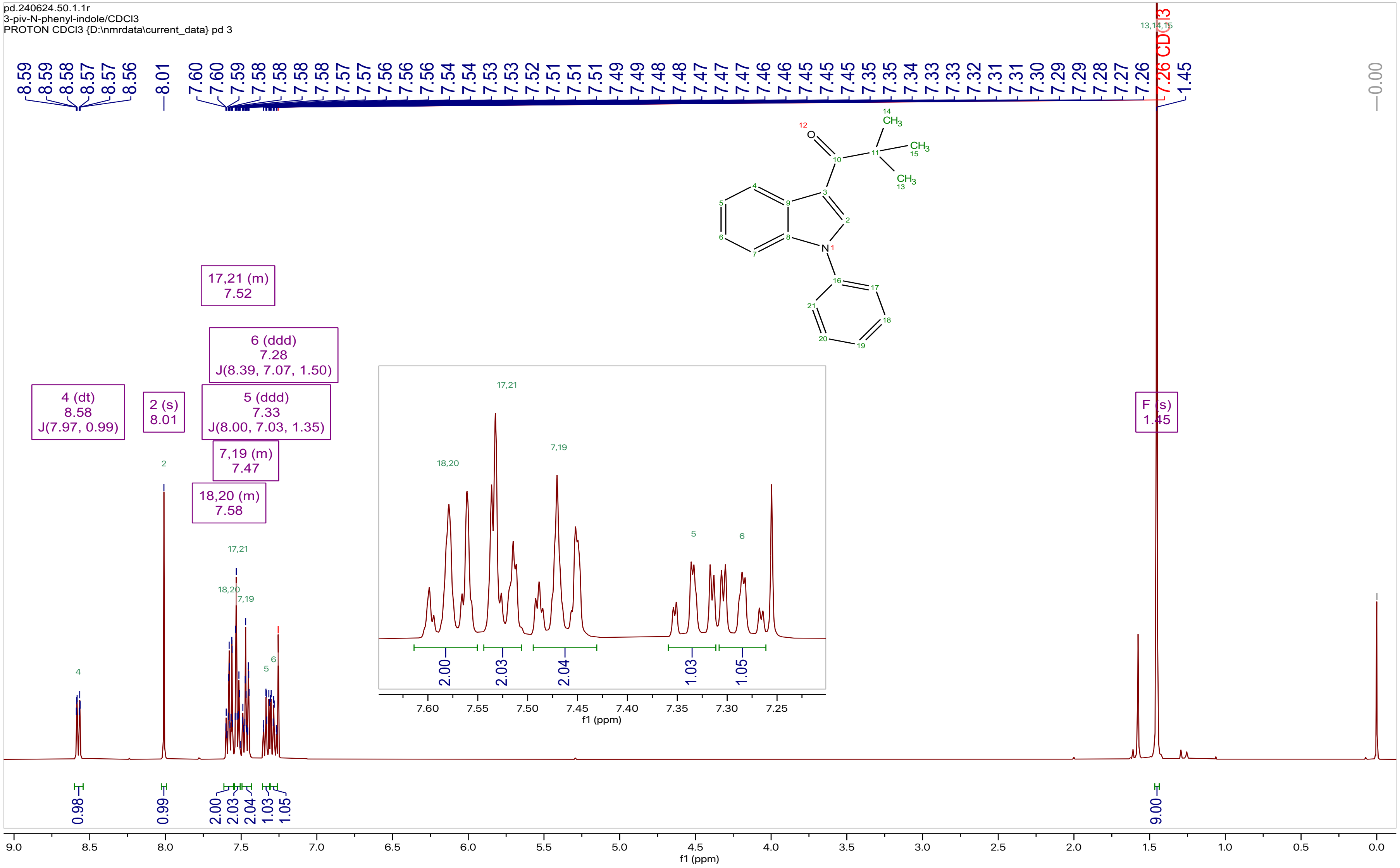

^1H NMR (400 MHz, $CDCl_3$) of 1o

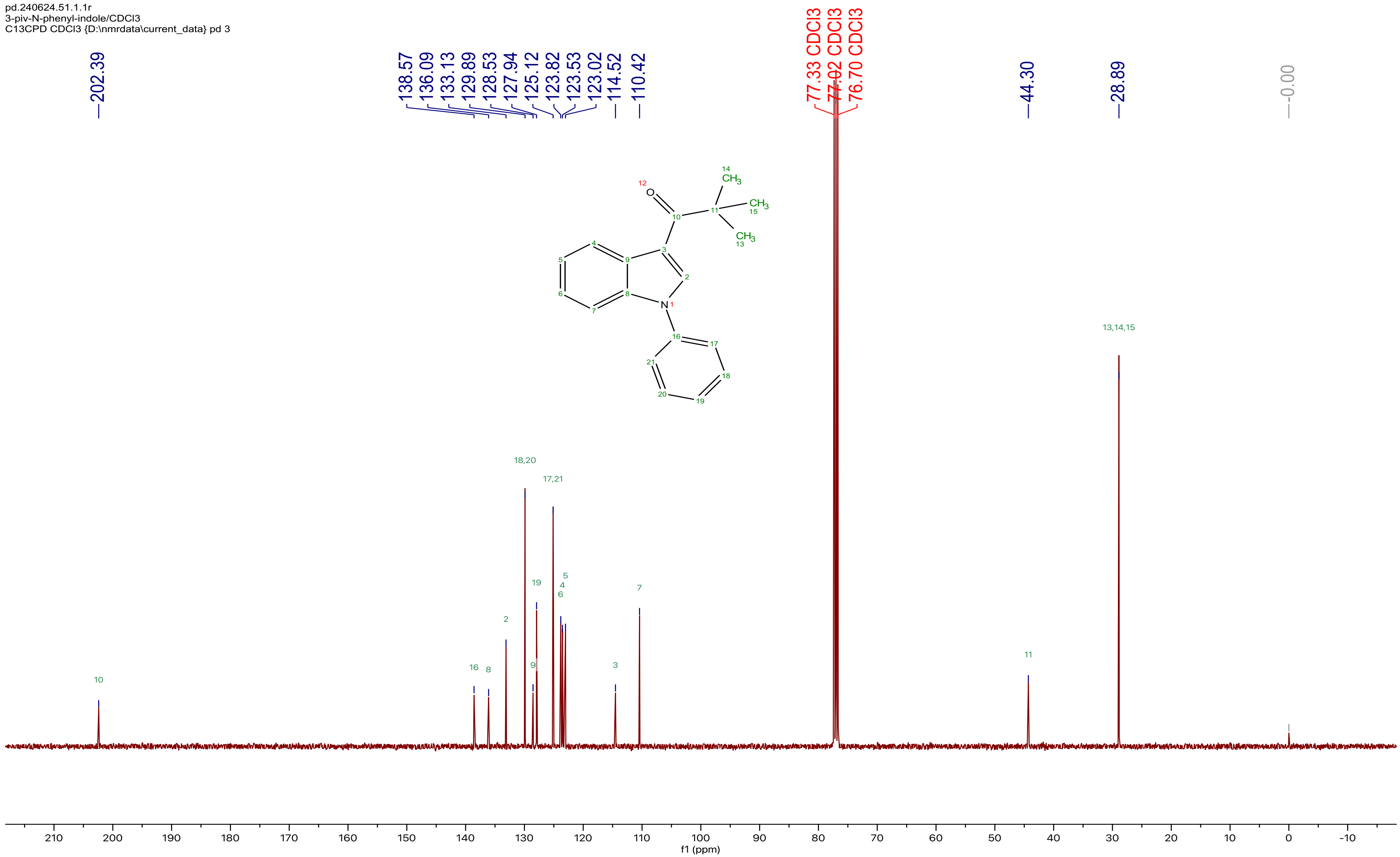

$^{13}C\{^{1}H\}$ NMR (101 MHz, $CDCl_3$) of 1o

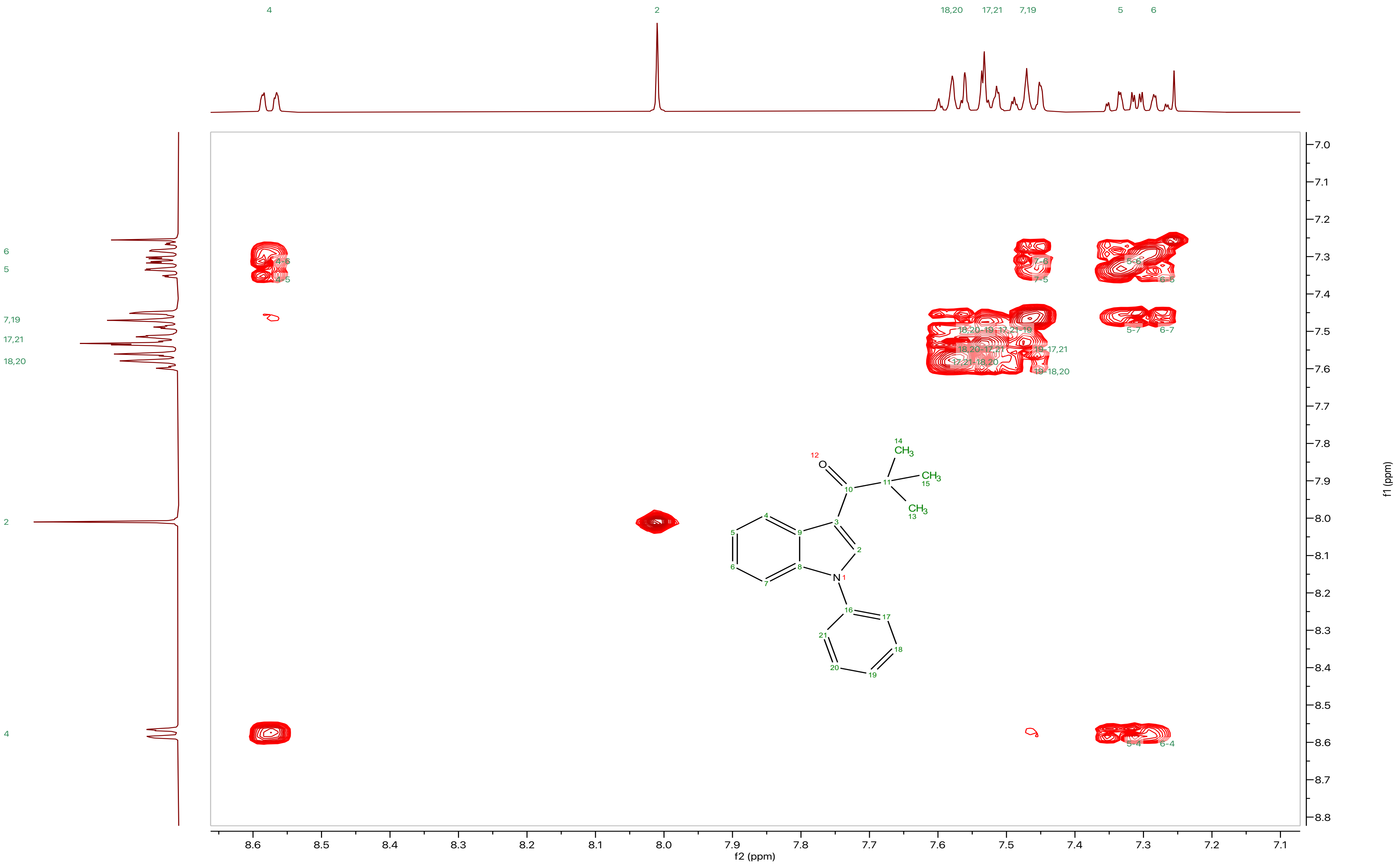

^{1}H-^{1}H COSY (400 MHz, CDCl$_3$) of 1o

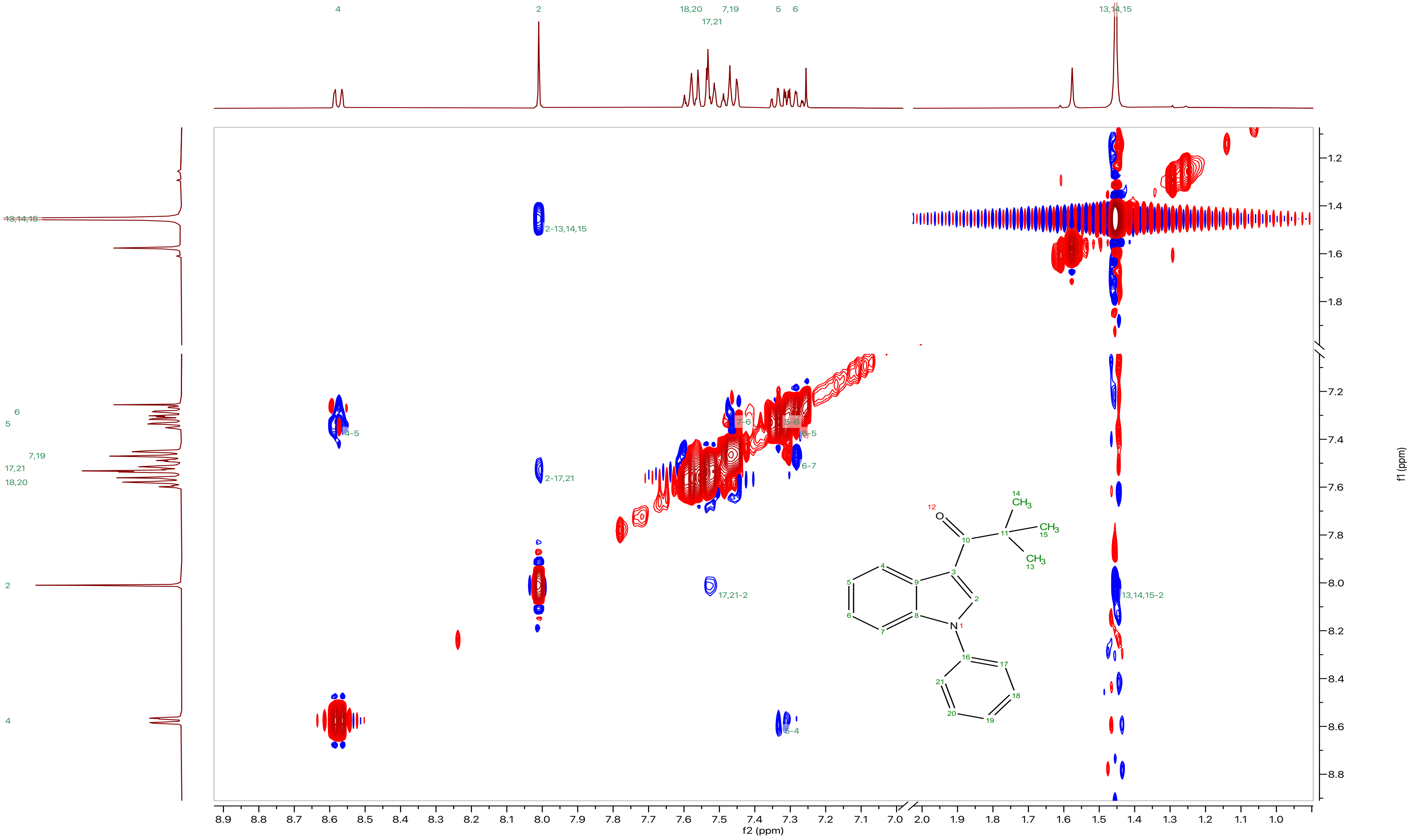

^1H-^1H NOESY (400 MHz, $CDCl_3$) of 1o

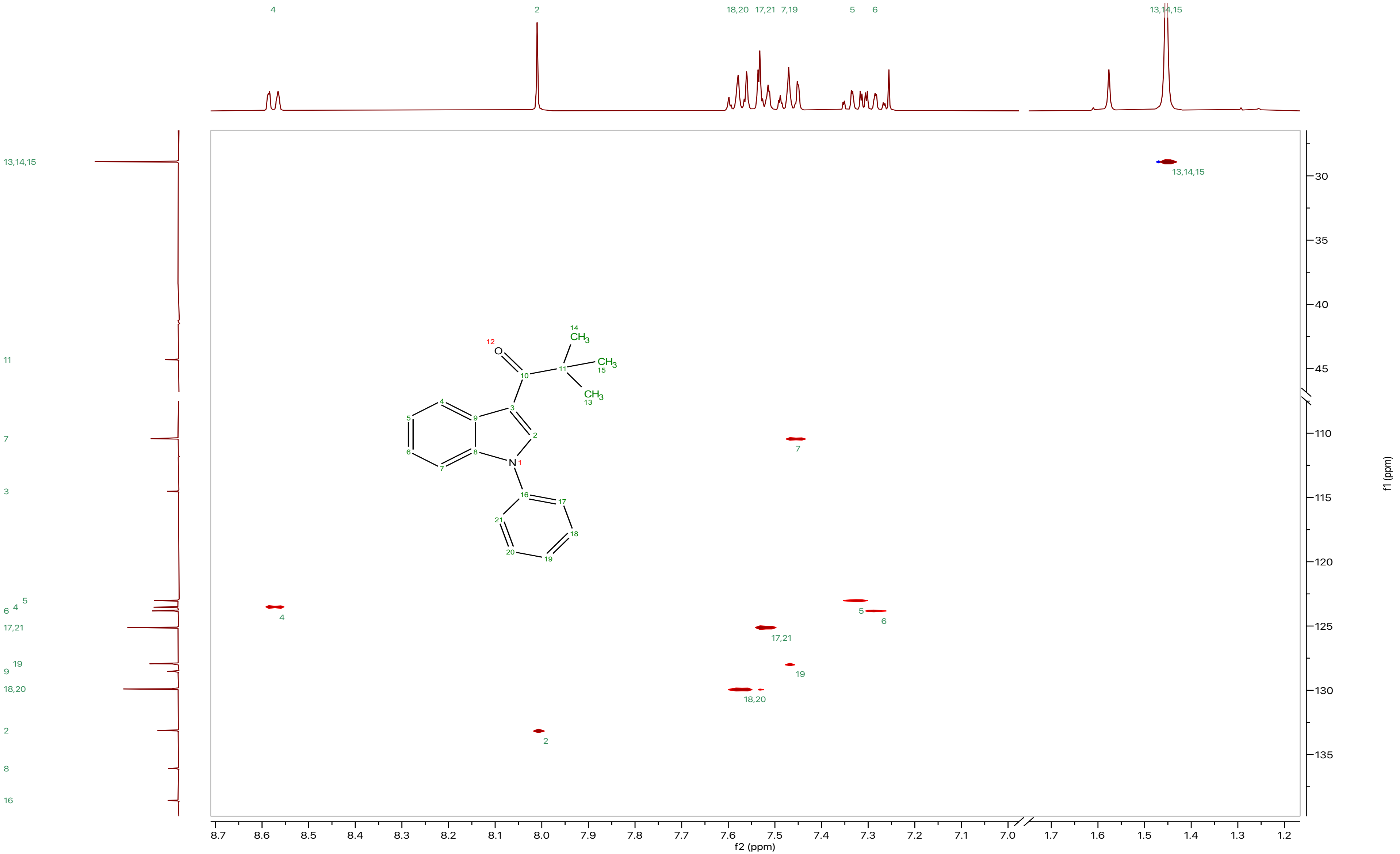

^{1}H-$^{13}C\{^{1}H\}$ HSQC NMR (400/101 MHz, $CDCl_3$) of 1o

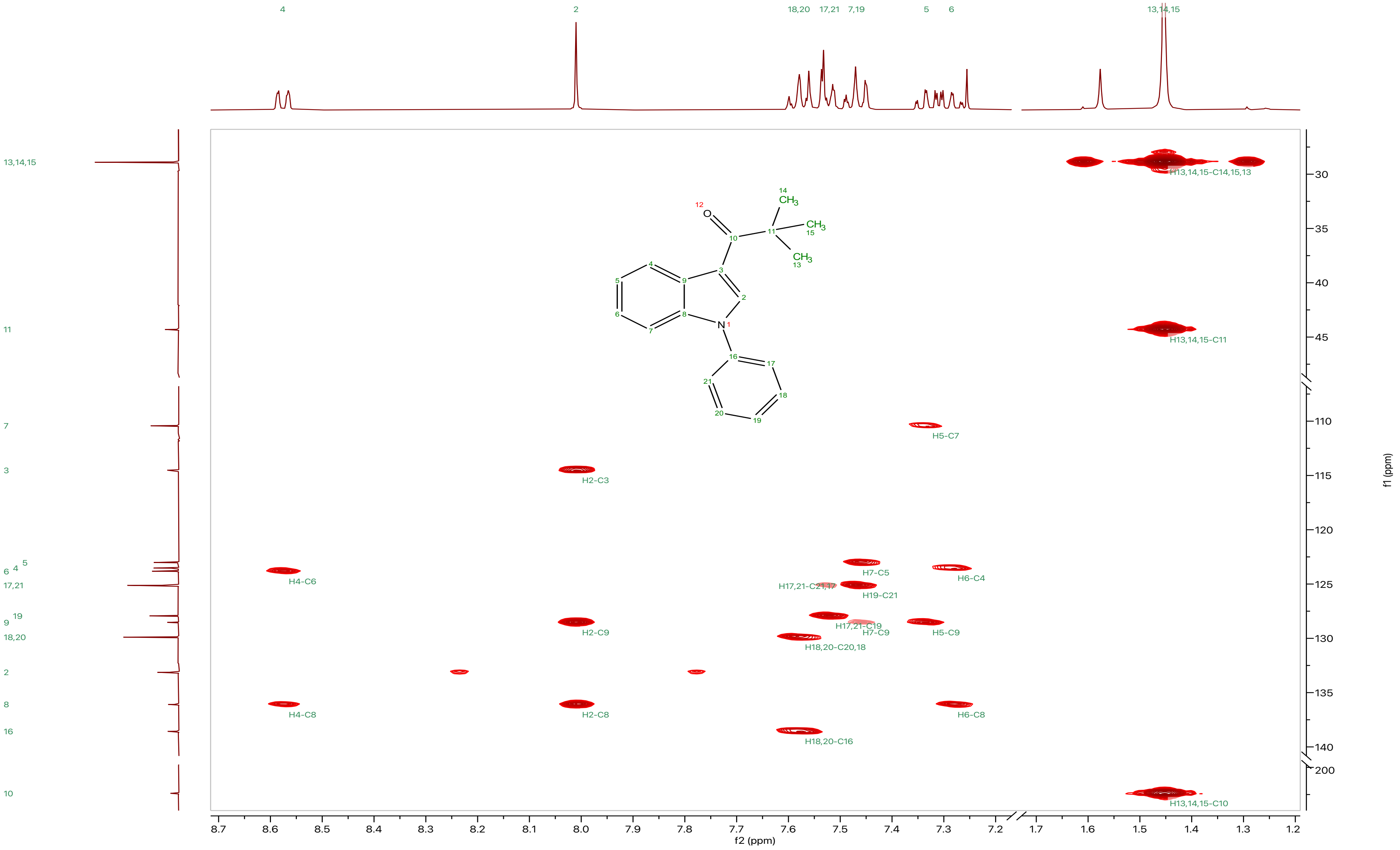

^{1}H-^{13}C{^{1}H} HMBC NMR (400/101 MHz, $CDCl_3$) of 1o

2ao

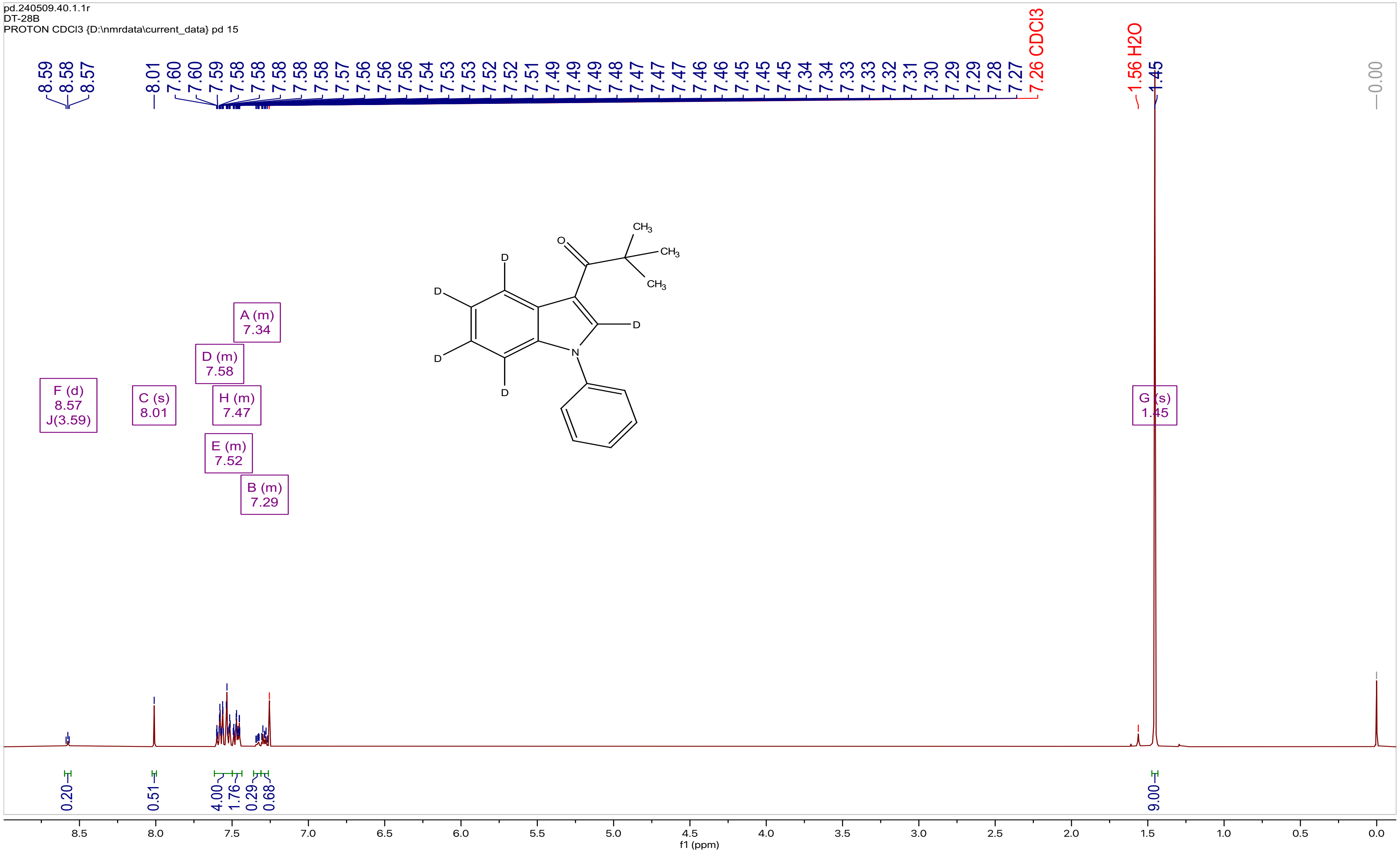

^{1}H NMR (400 MHz, CDCl$_3$) of 2ao

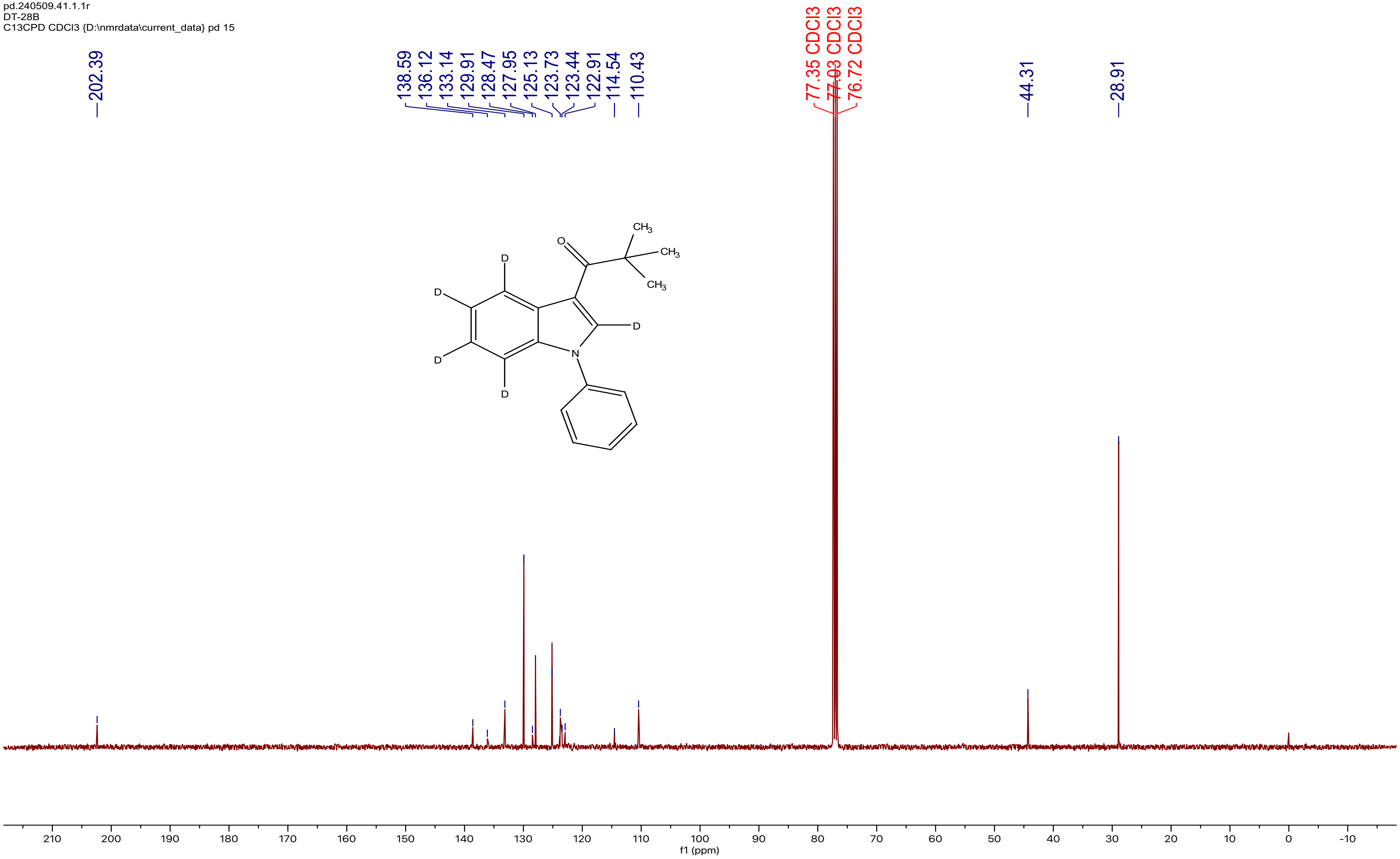

$^{13}C\{^1H\}$ NMR (101 MHz, $CDCl_3$) of 2ao

1o’

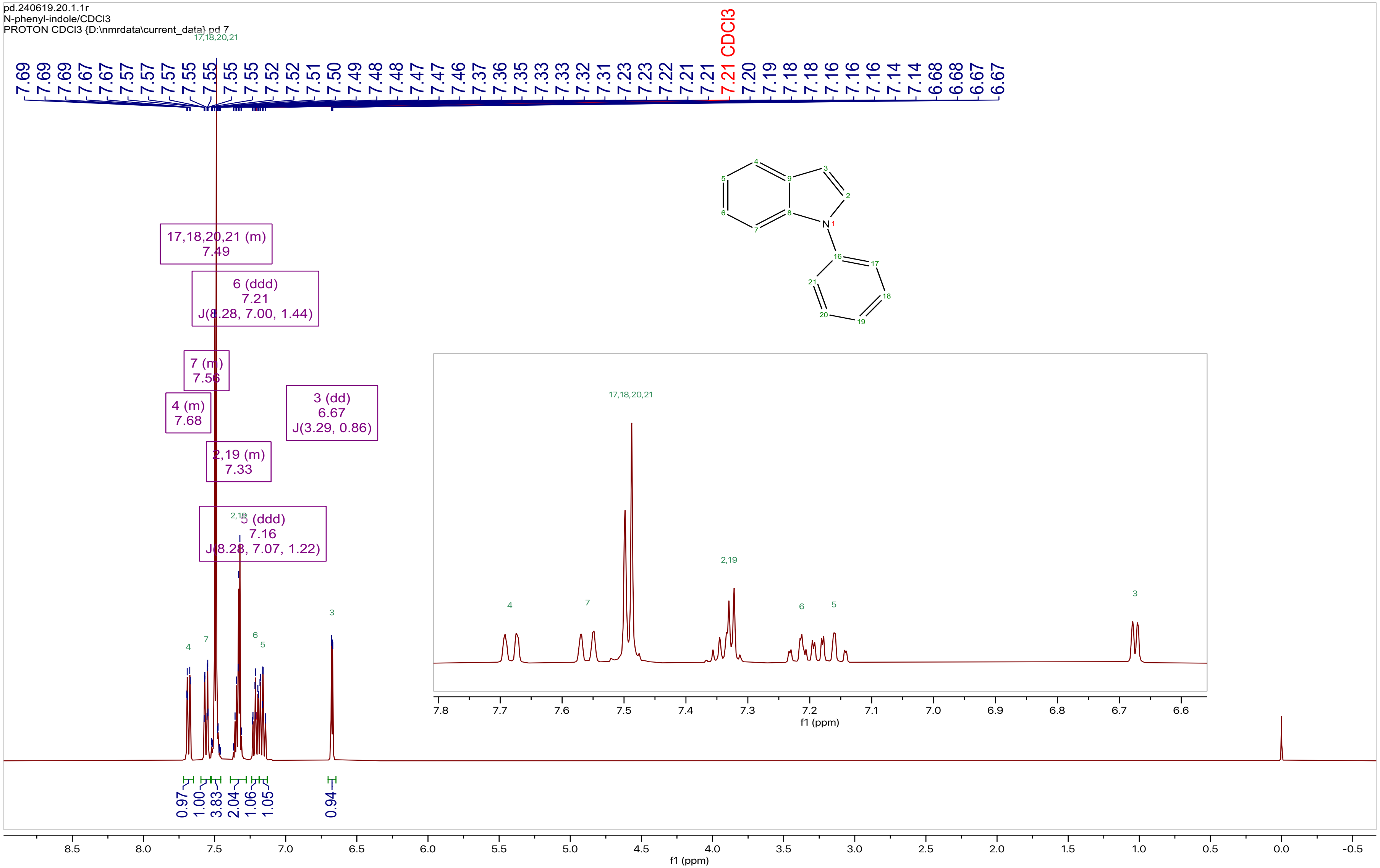

^{1}H NMR (400 MHz, $CDCl_3$) of 1o’

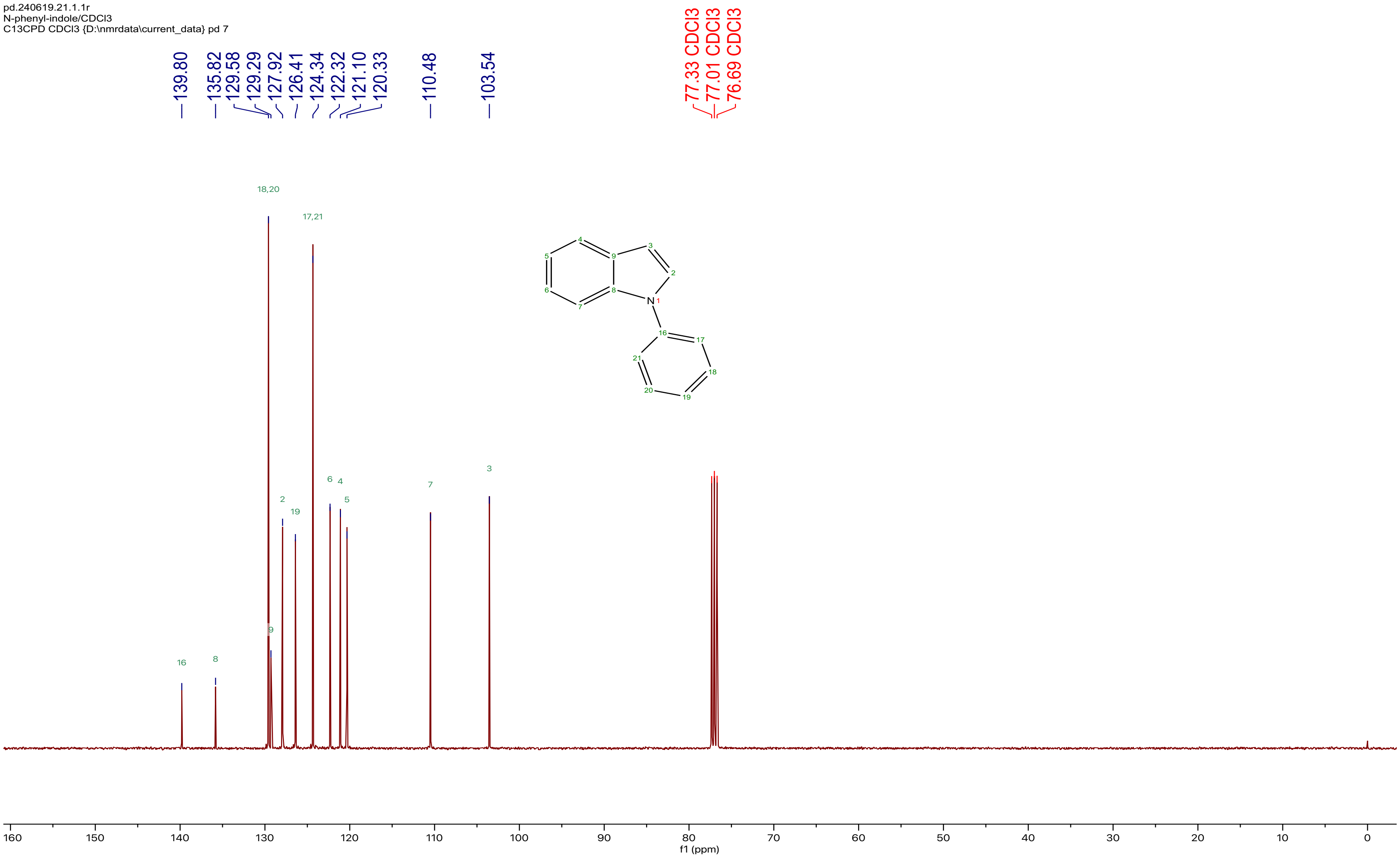

$^{13}C\{^{1}H\}$ NMR (101 MHz, $CDCl_3$) of 1o’

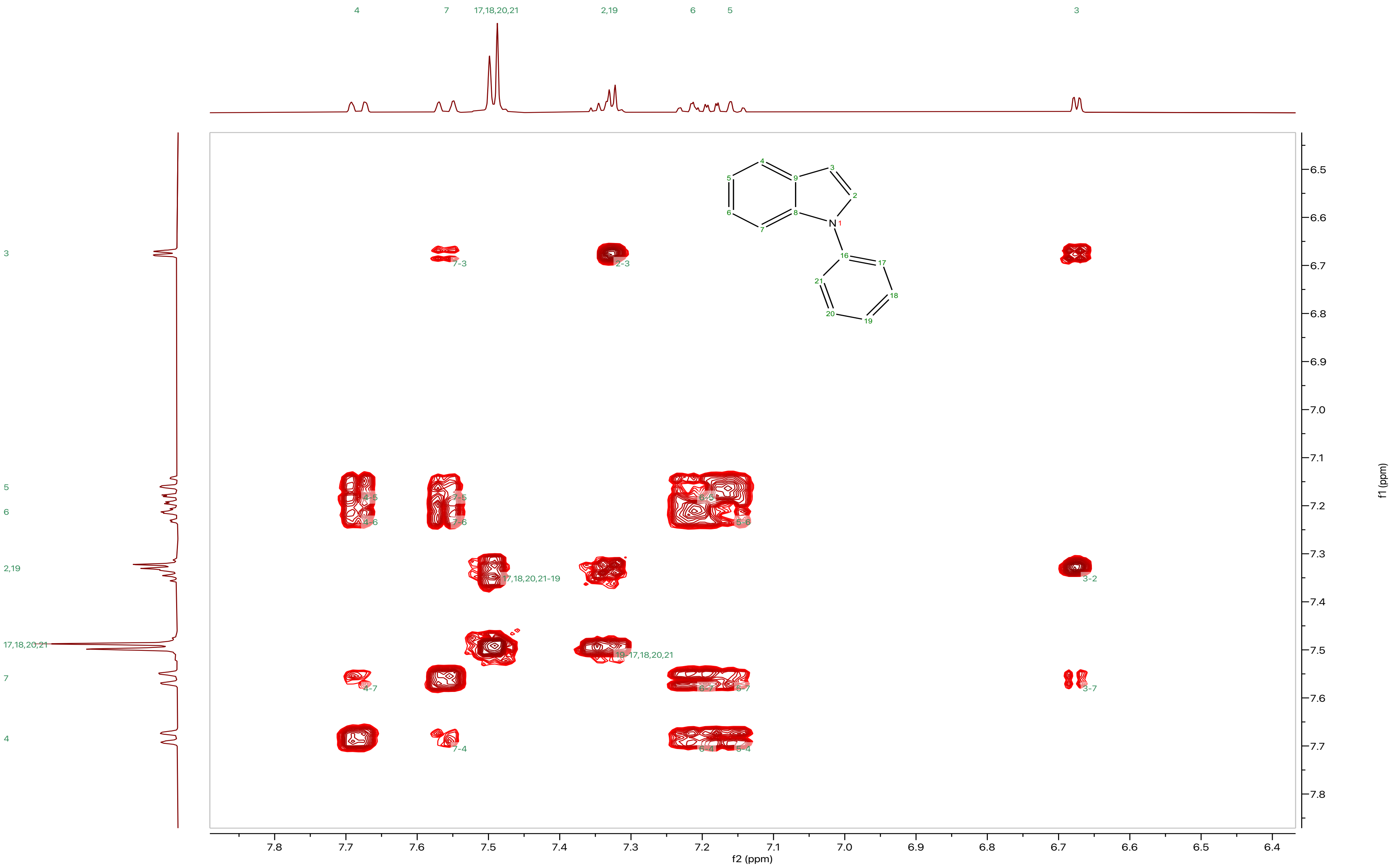

^1H-^1H COSY (400 MHz, $CDCl_3$) of 1o'

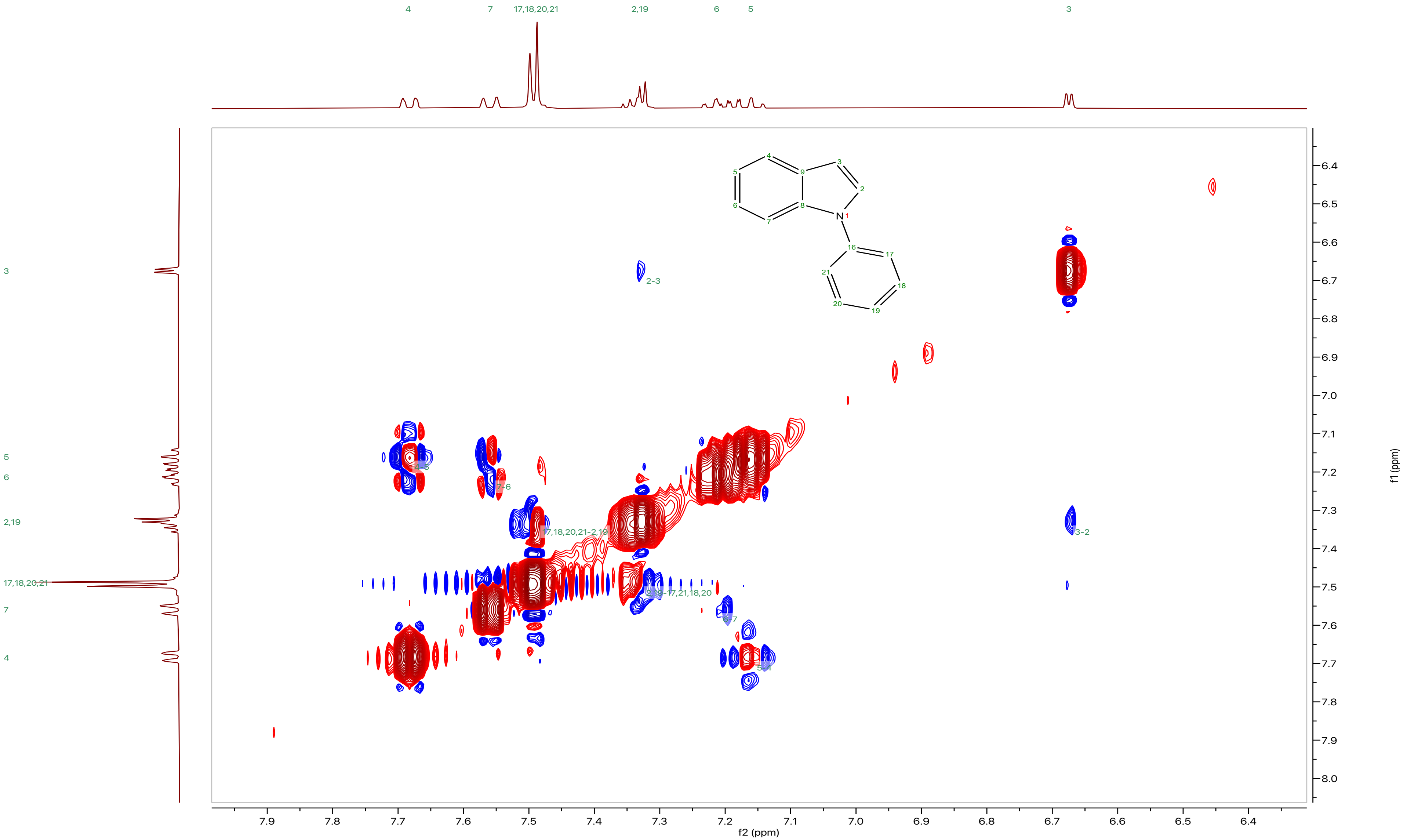

^1H-^1H NOESY (400 MHz, $CDCl_3$) of 1o'

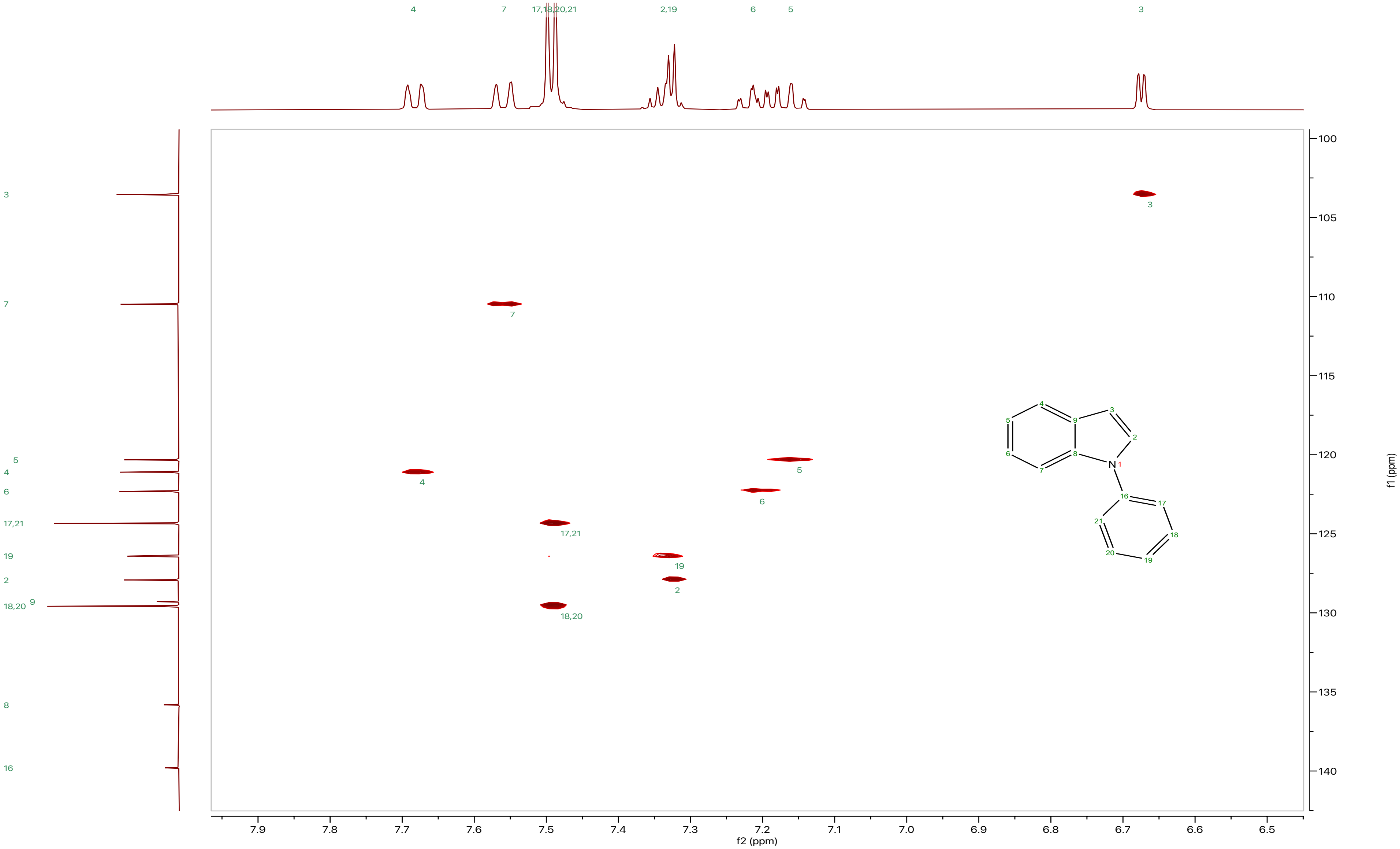

^{1}H-^{13}C{^{1}H} HSQC NMR (400/101 MHz, $CDCl_3$) of 1o'

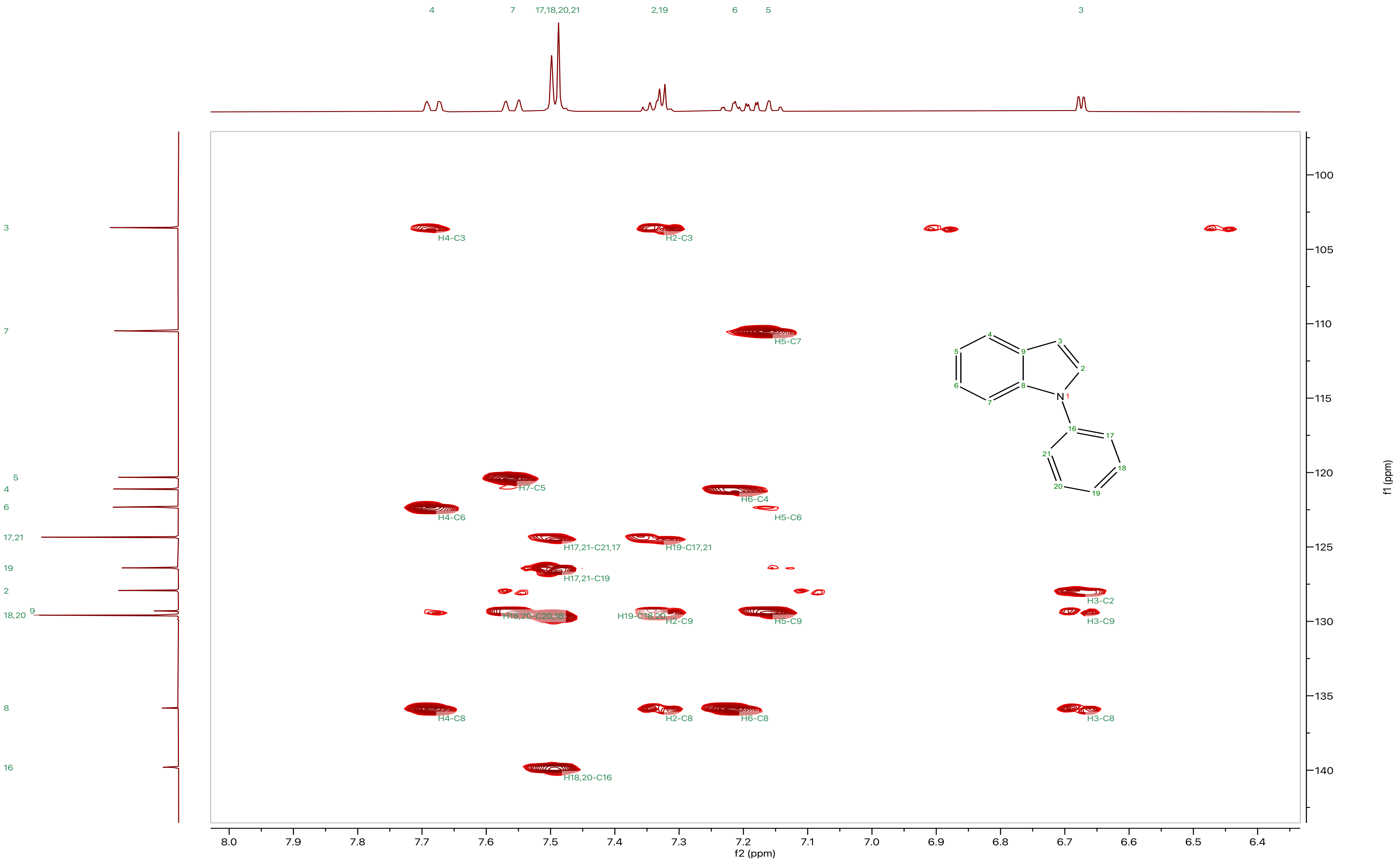

^{1}H-$^{13}C\{^{1}H\}$ HMBC NMR (400/101 MHz, $CDCl_3$) of 1o'

2bo

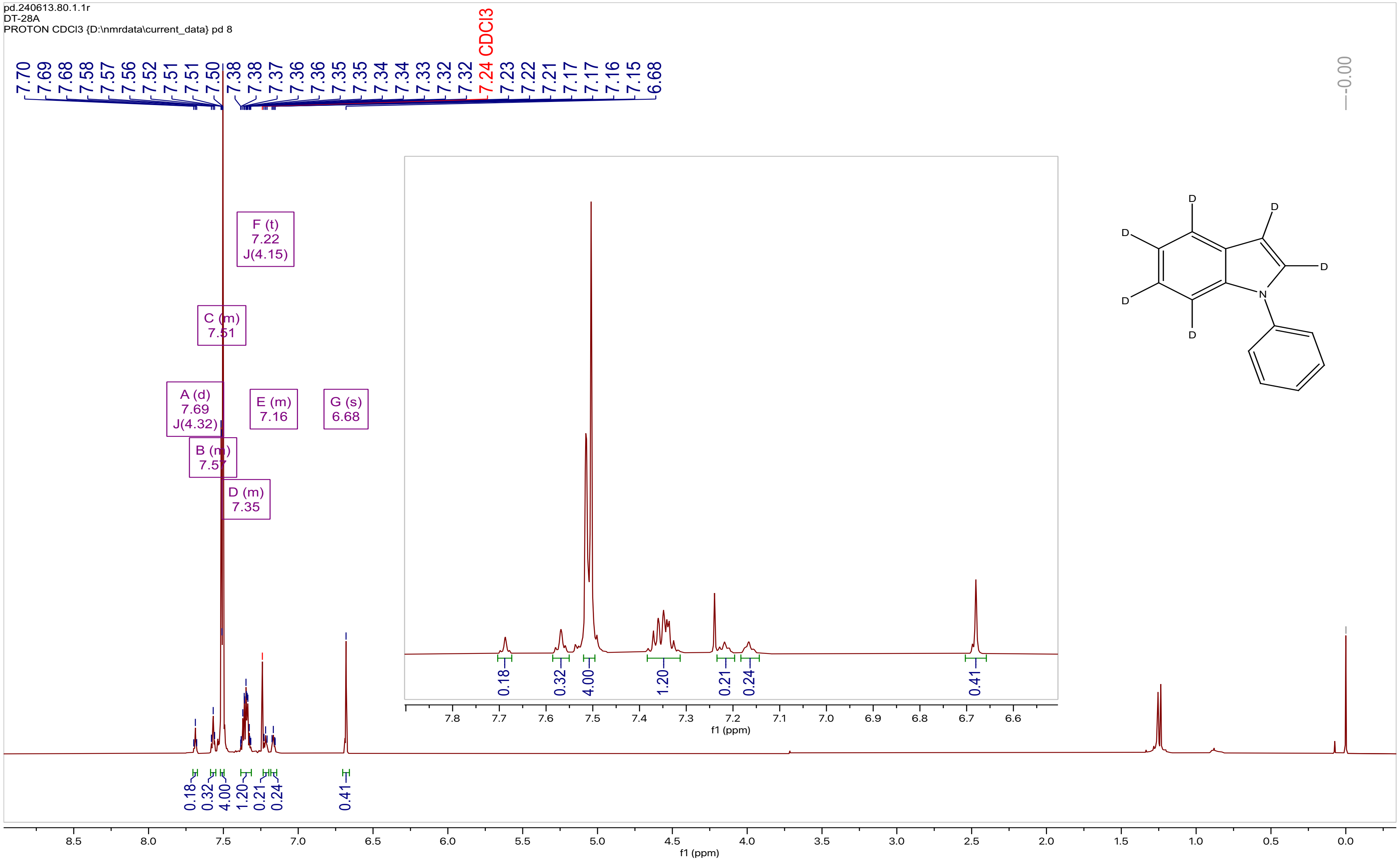

^1H NMR (400 MHz, $CDCl_3$) of 2bo

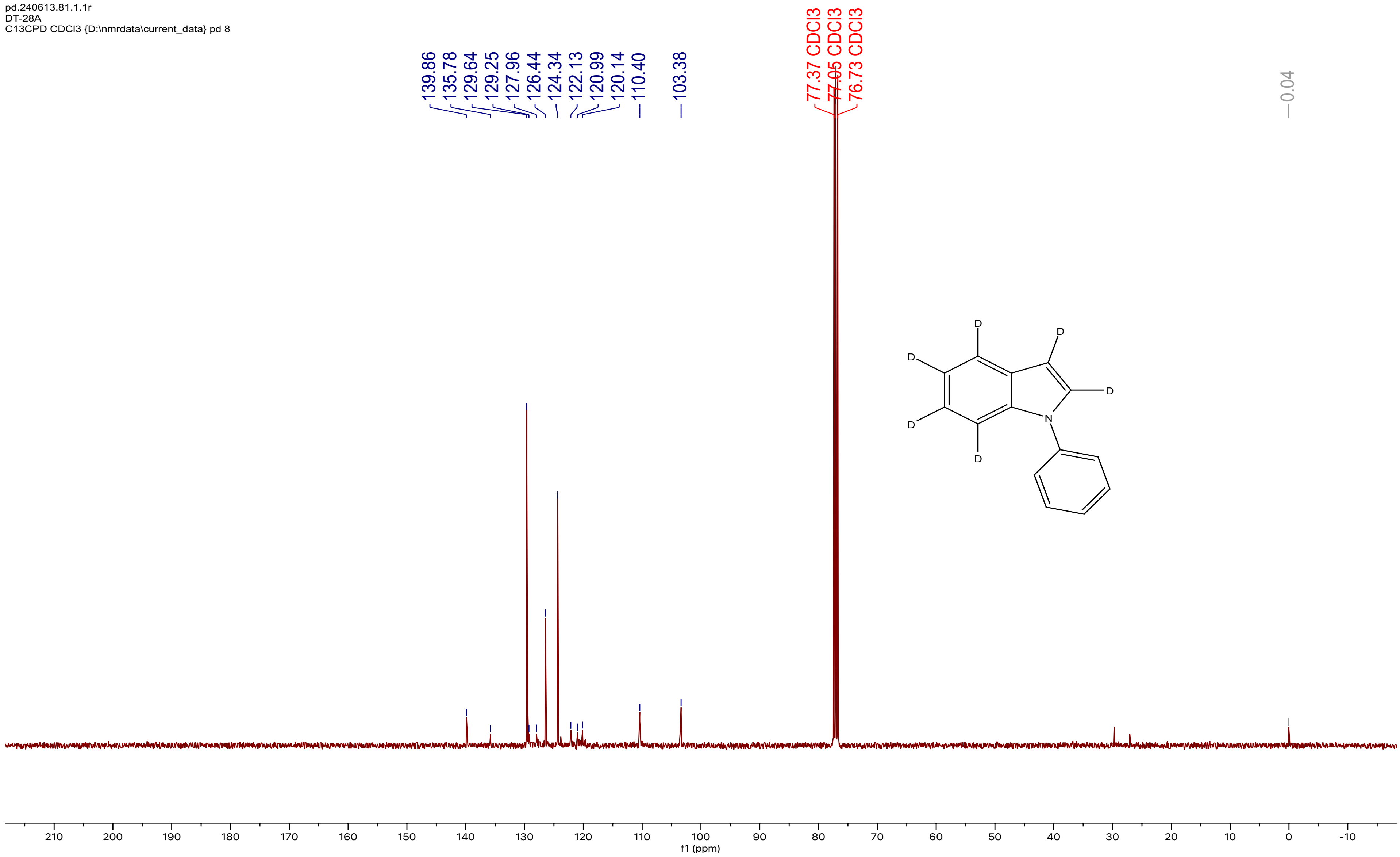

$^{13}C\{^{1}H\}$ NMR (101 MHz, $CDCl_3$) of 2bo

1p

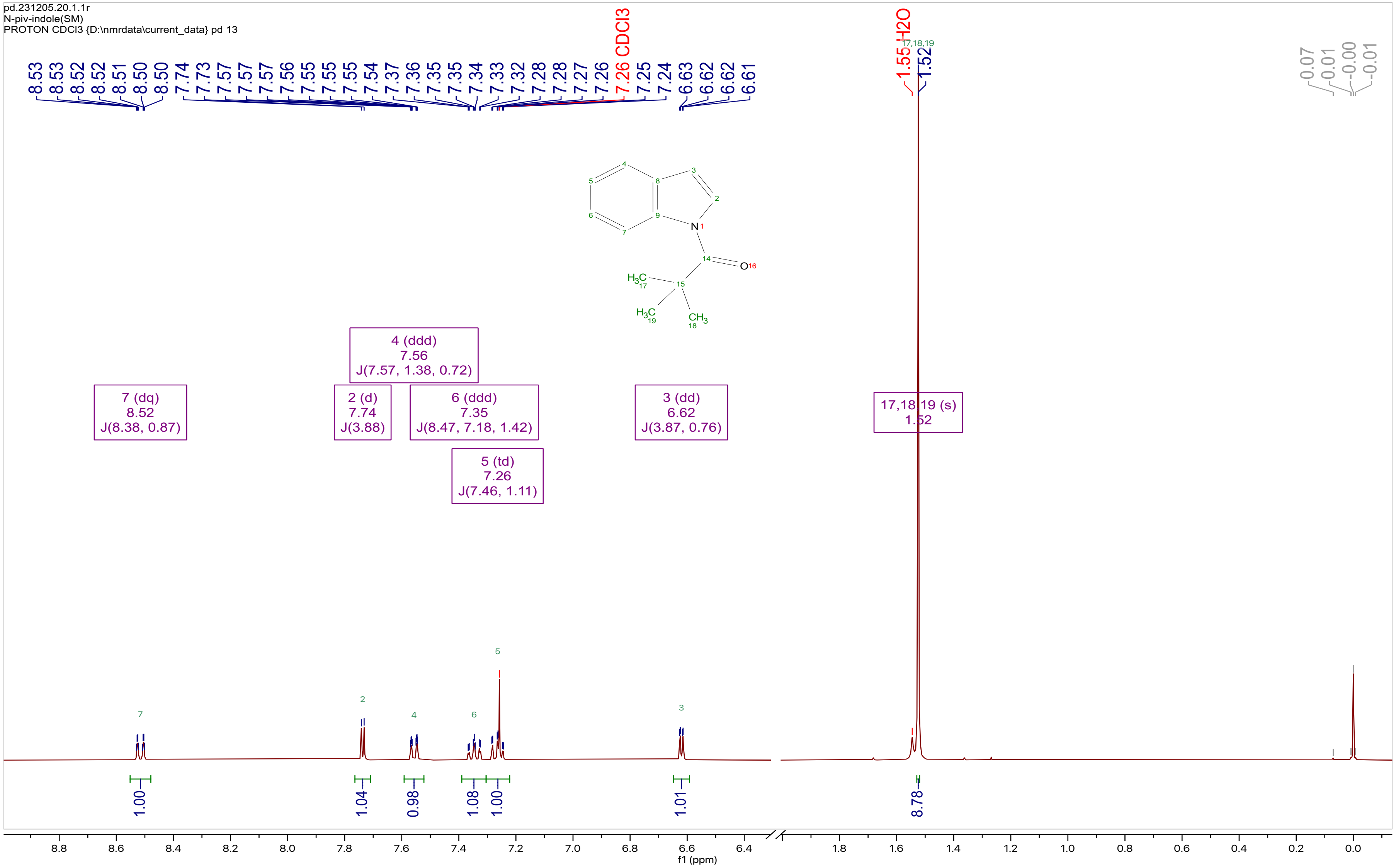

^{1}H NMR (400 MHz, $CDCl_3$) of 1p

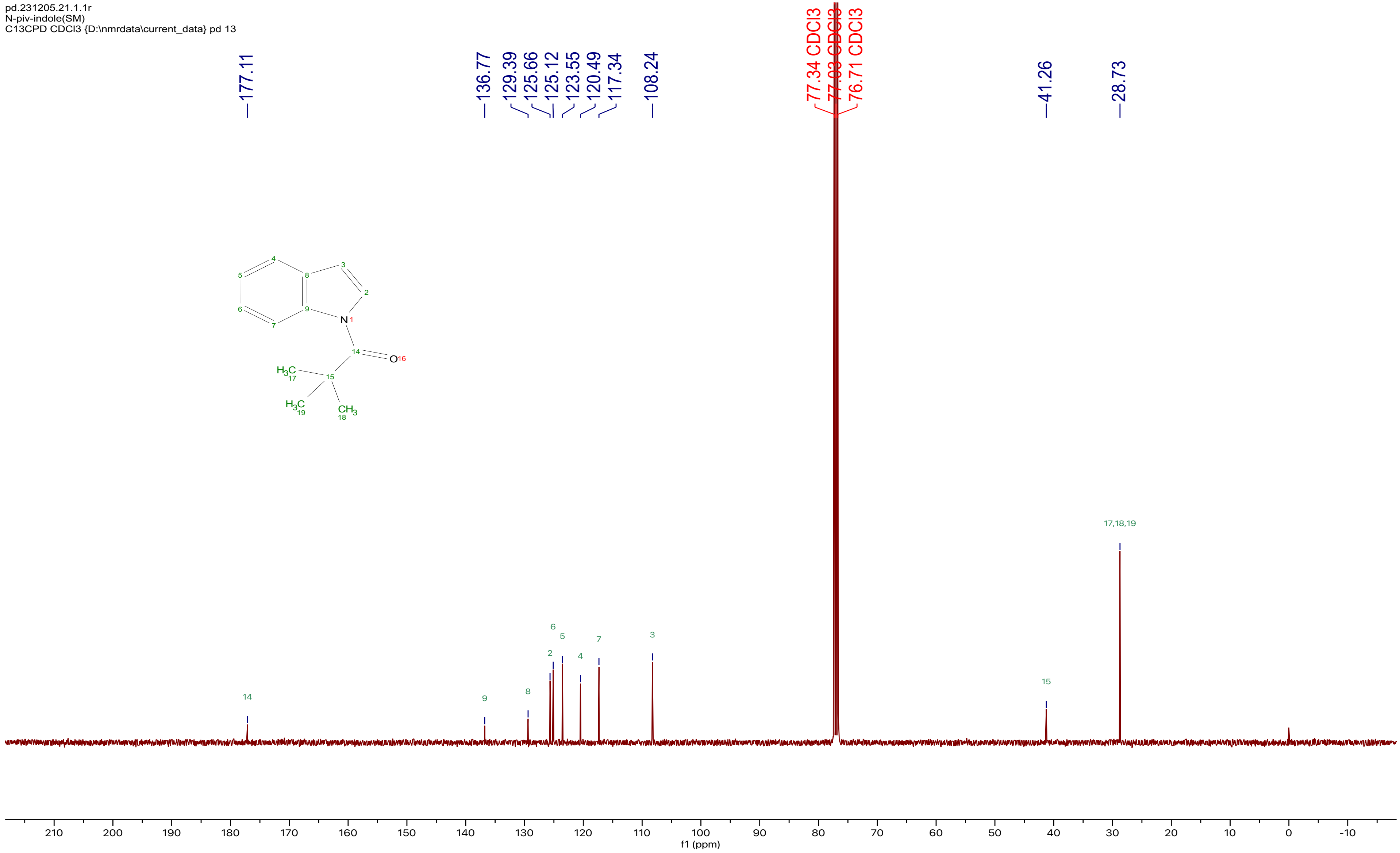

$^{13}C\{^{1}H\}$ NMR (101 MHz, $CDCl_3$) of 1p

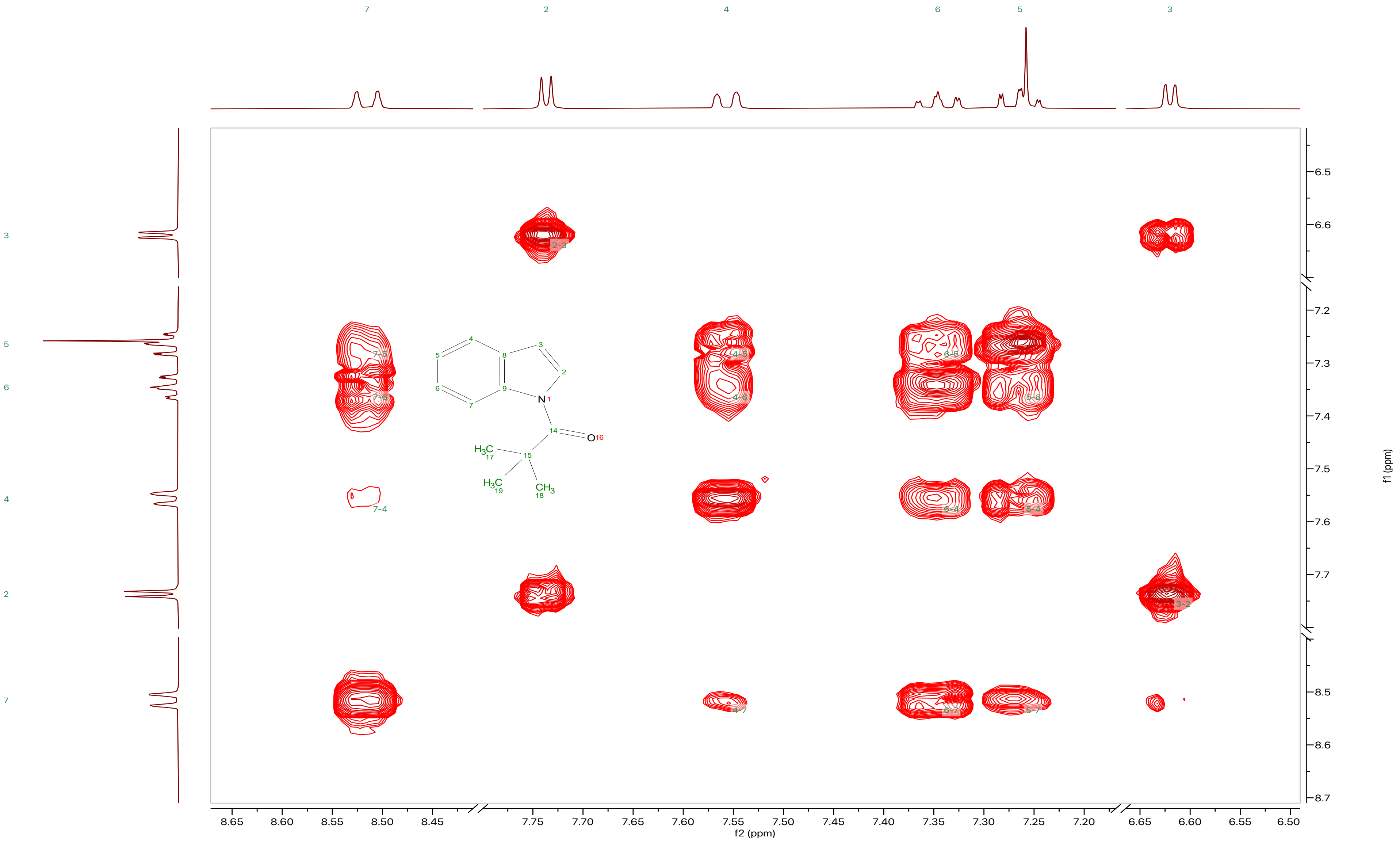

^1H-^1H COSY (400 MHz, $CDCl_3$) of 1p

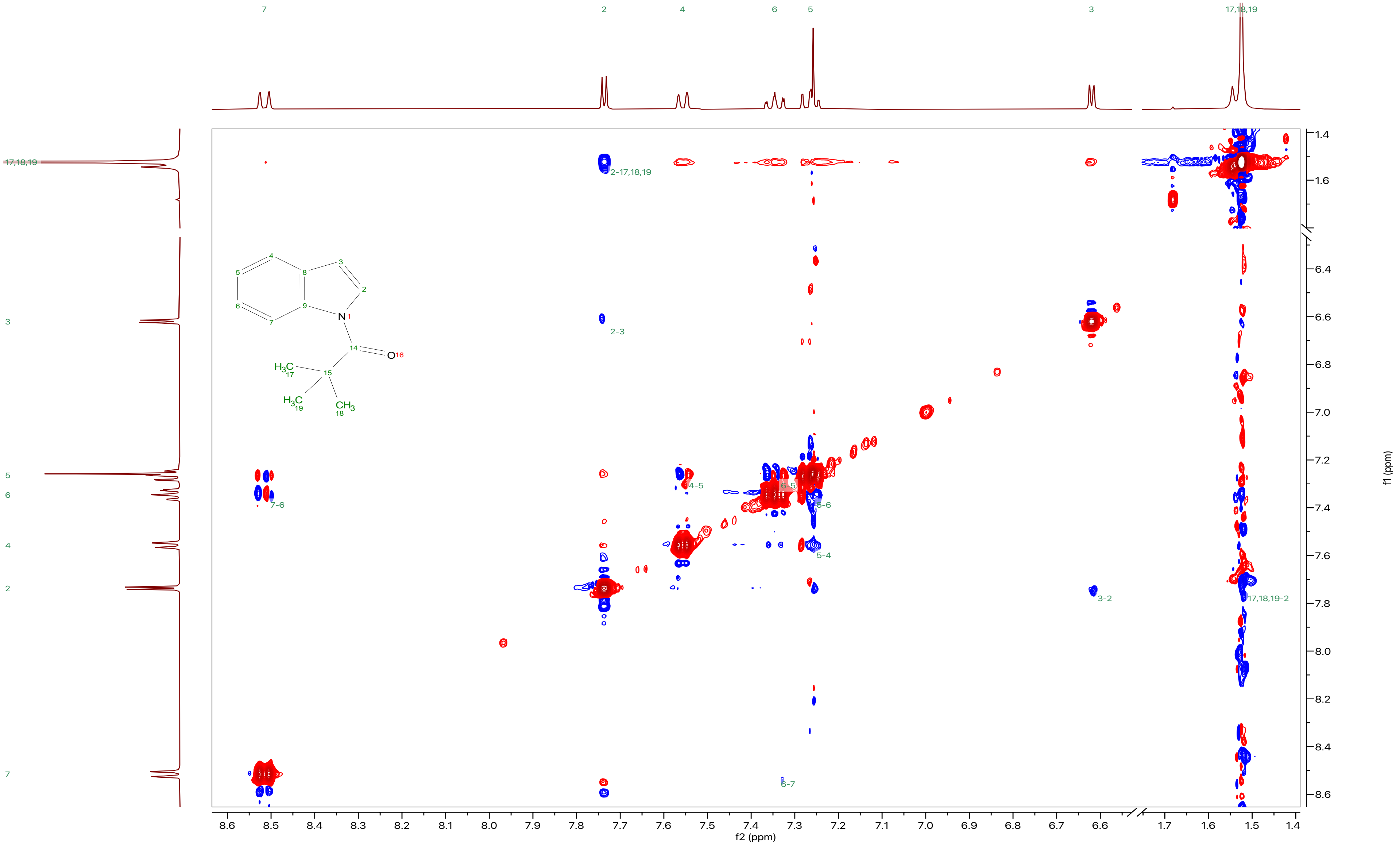

^{1}H-^{1}H NOESY (400 MHz, $CDCl_3$) of 1p

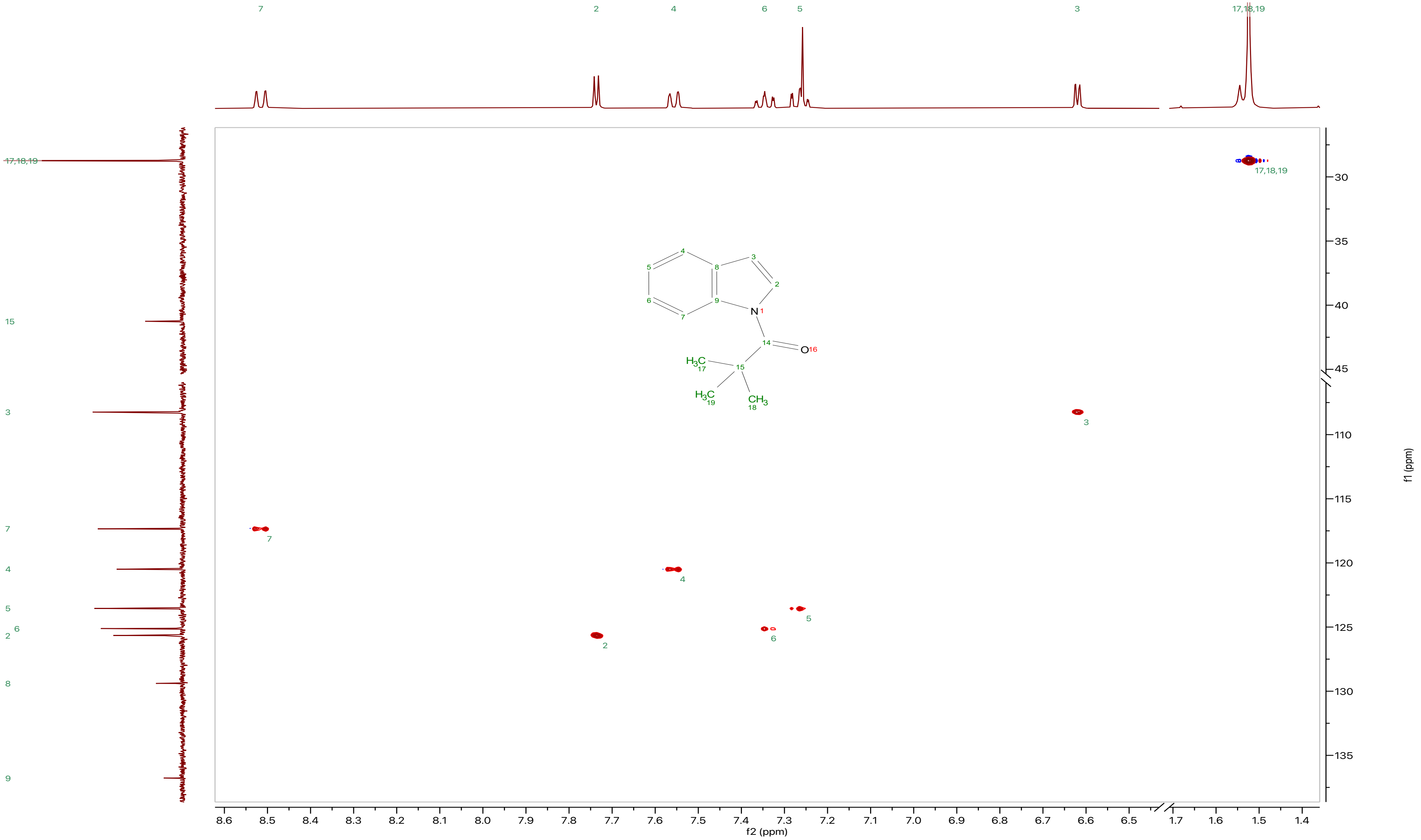

^{1}H-$^{13}C\{^{1}H\}$ HSQC NMR (400/101 MHz, $CDCl_3$) of 1p

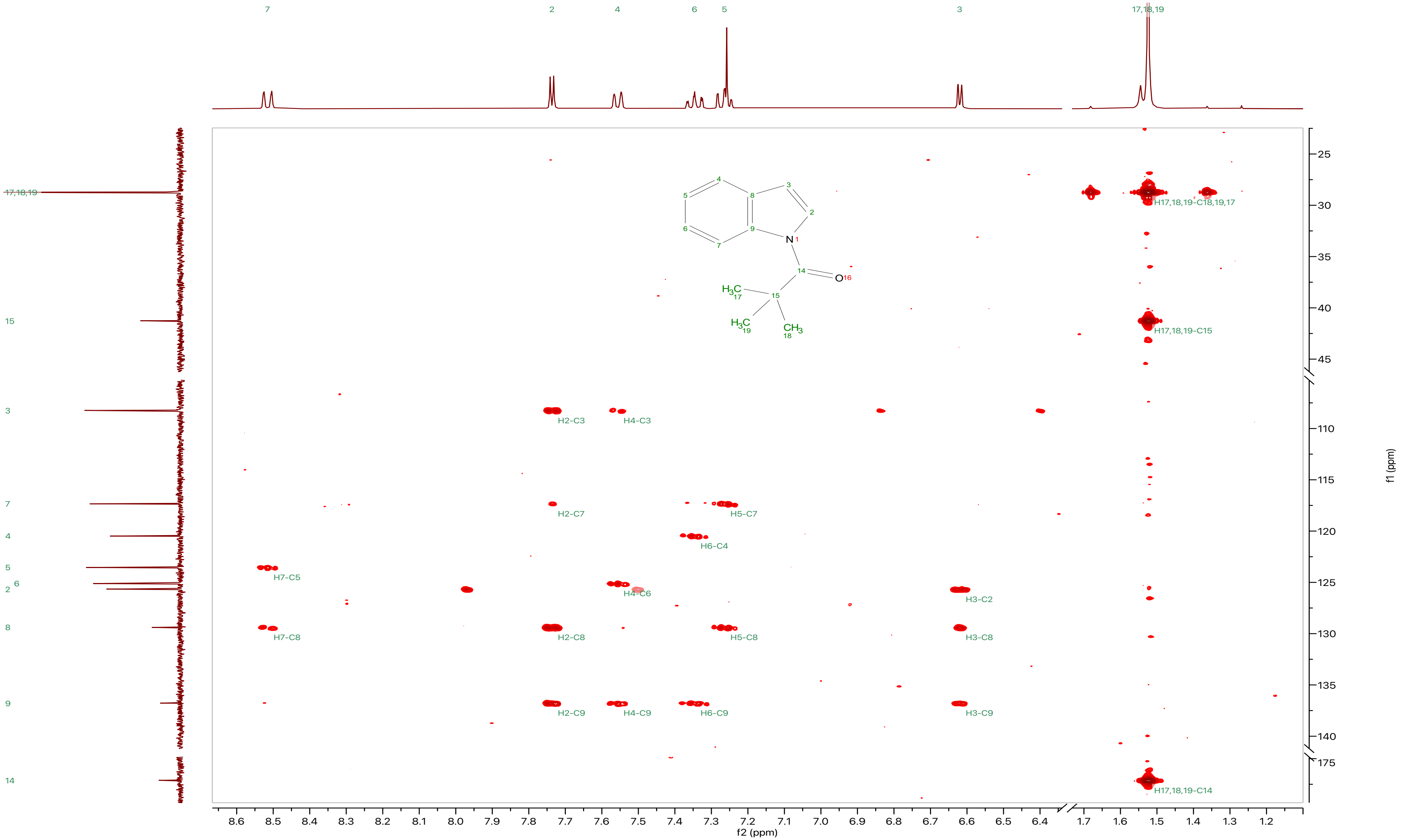

^{1}H-^{13}C{^{1}H} HMBC NMR (400/101 MHz, CDCl$_3$) of 1p

2p

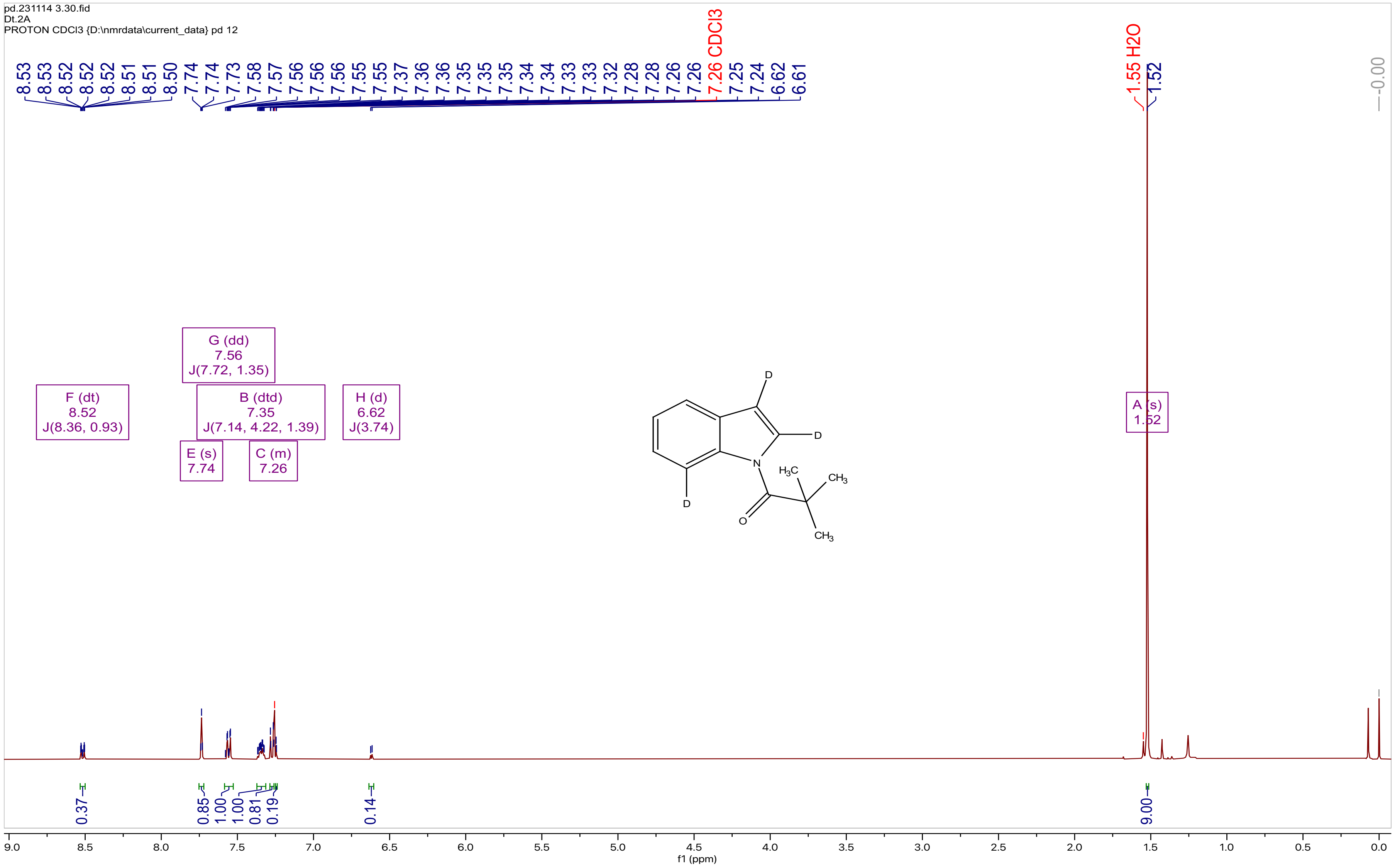

^{1}H NMR (400 MHz, $CDCl_3$) of 2p

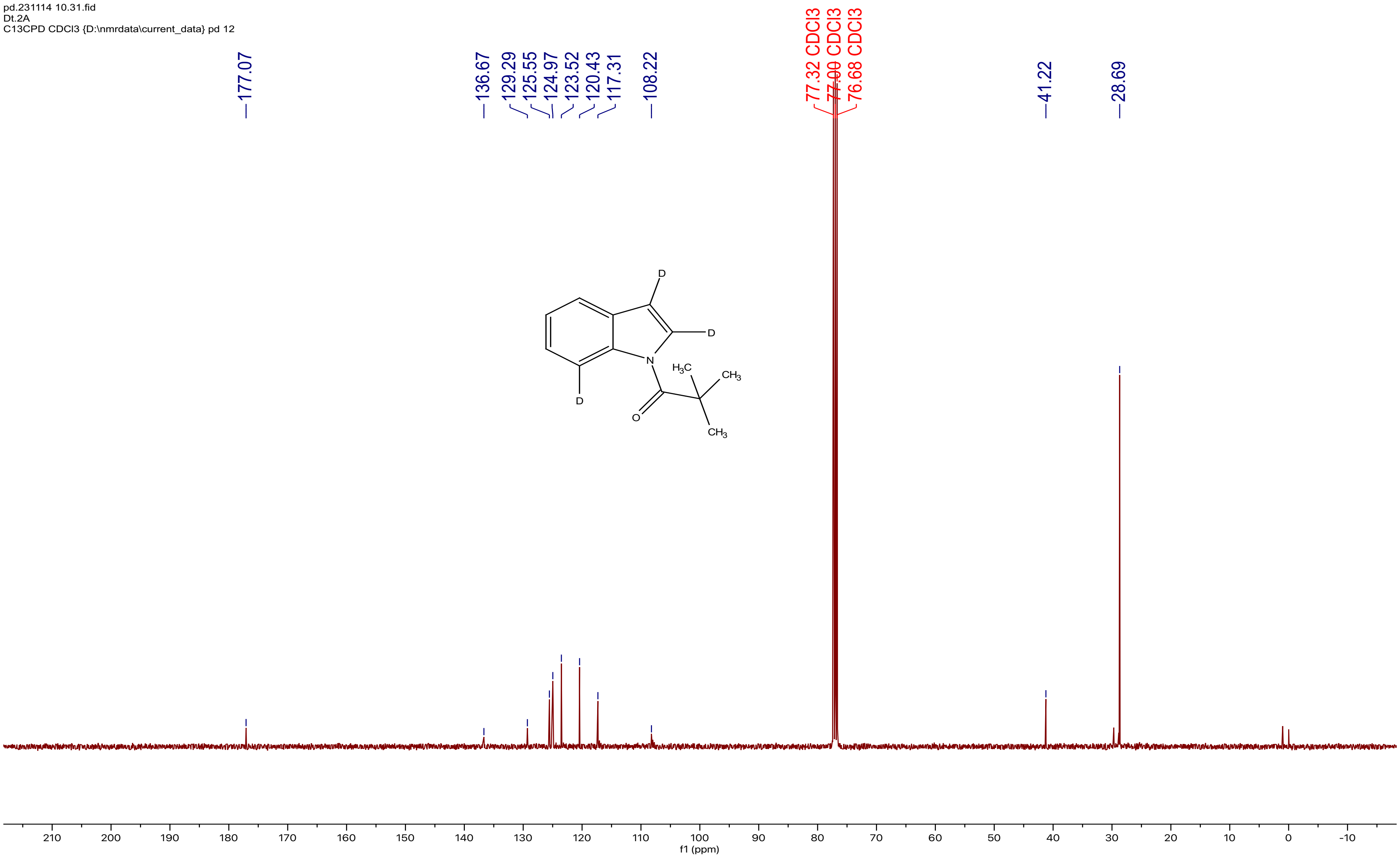

$^{13}C\{^1H\}$ NMR (101 MHz, $CDCl_3$) of 2p

1q

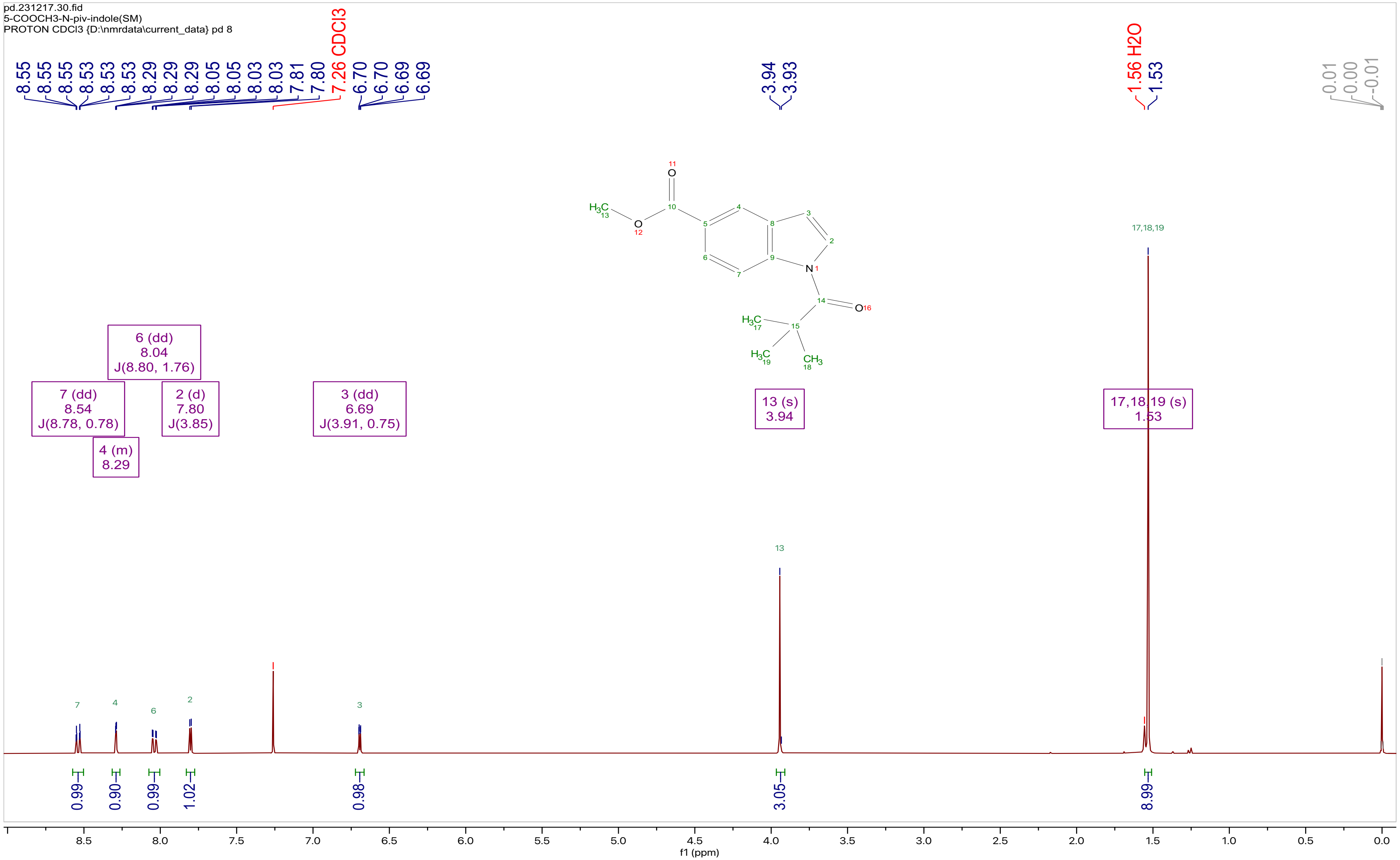

^{1}H NMR (400 MHz, $CDCl_3$) of 1q

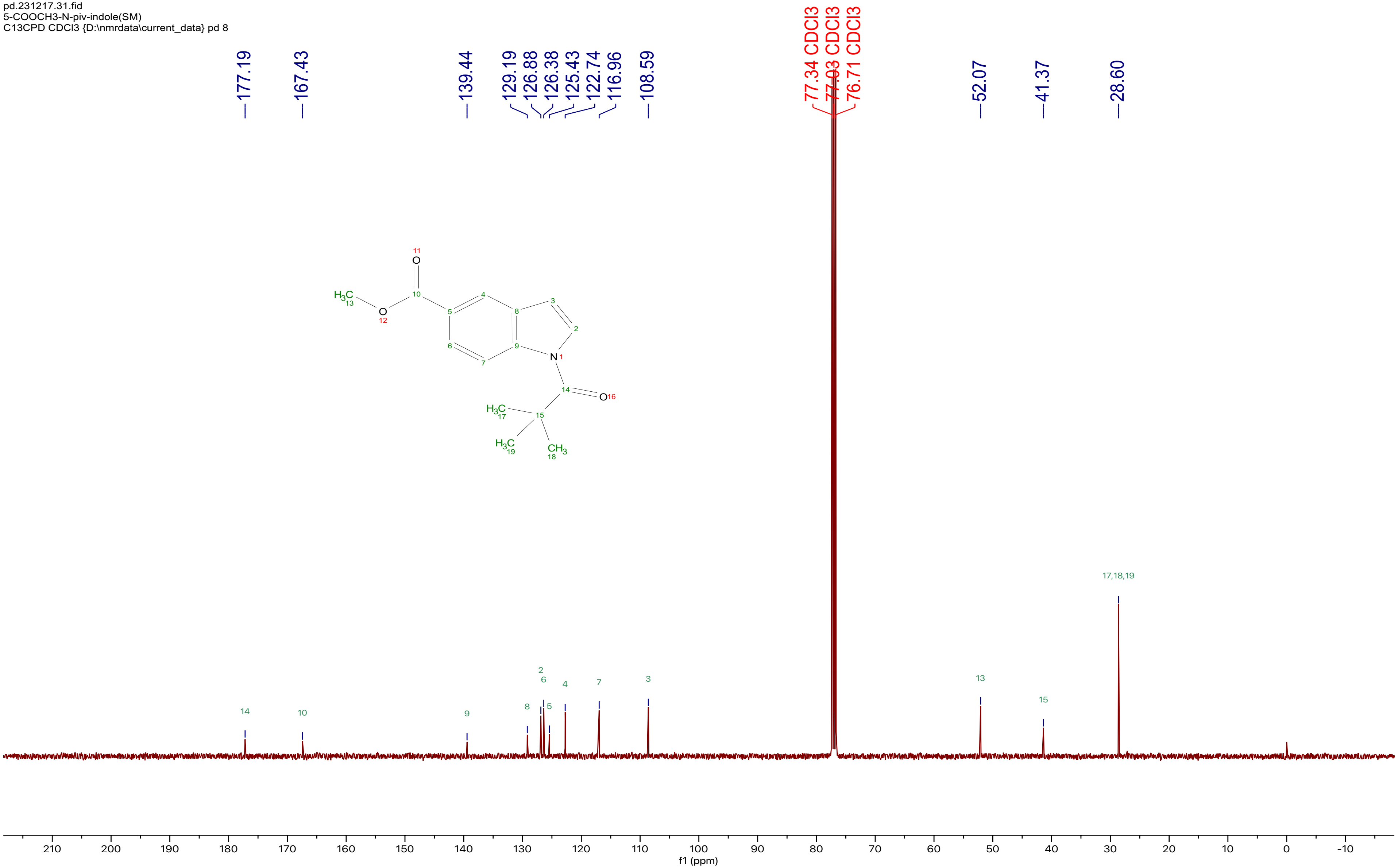

$^{13}C\{^{1}H\}$ NMR (101 MHz, $CDCl_3$) of 1q

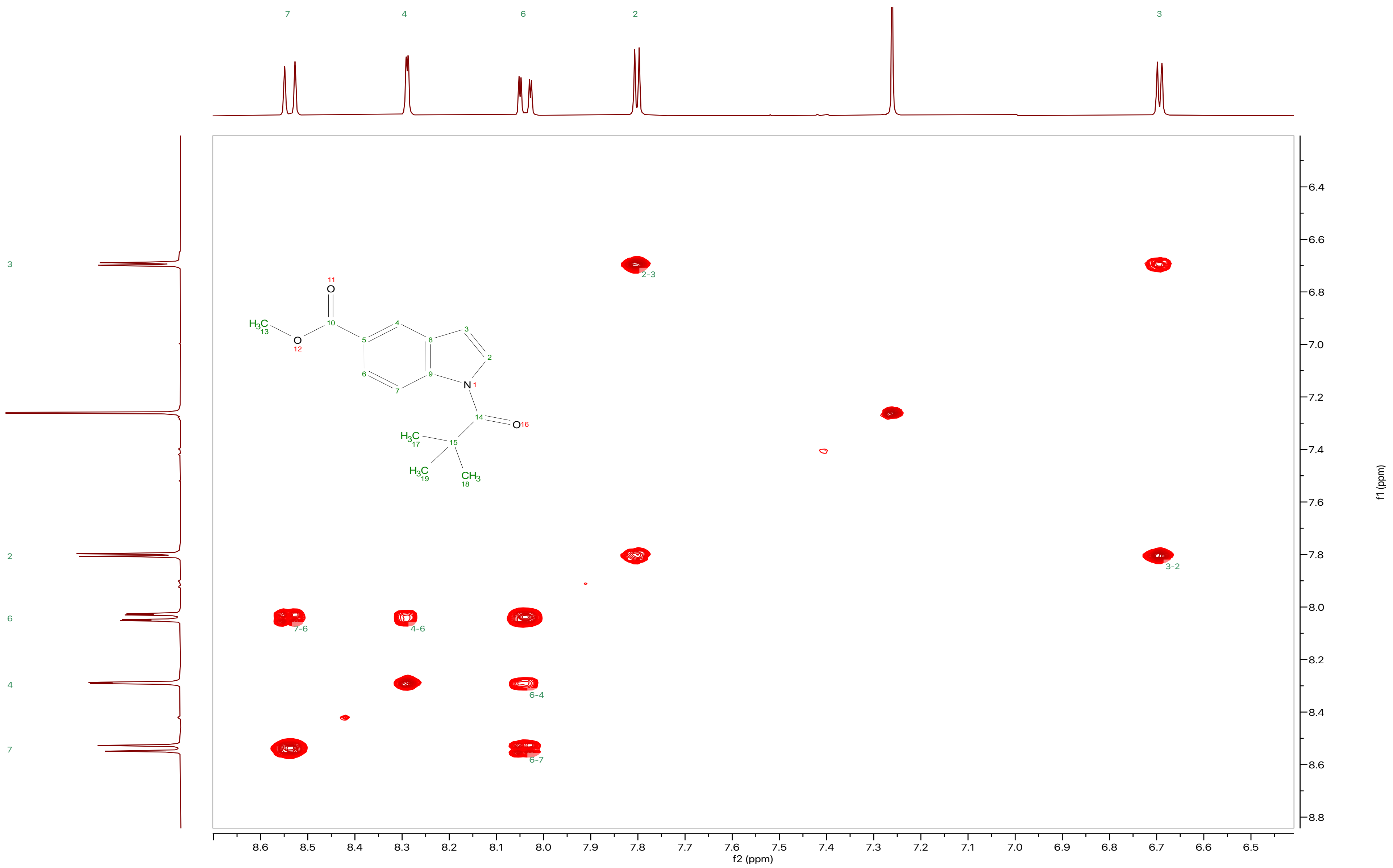

^{1}H-^{1}H COSY (400 MHz, $CDCl_3$) of 1q

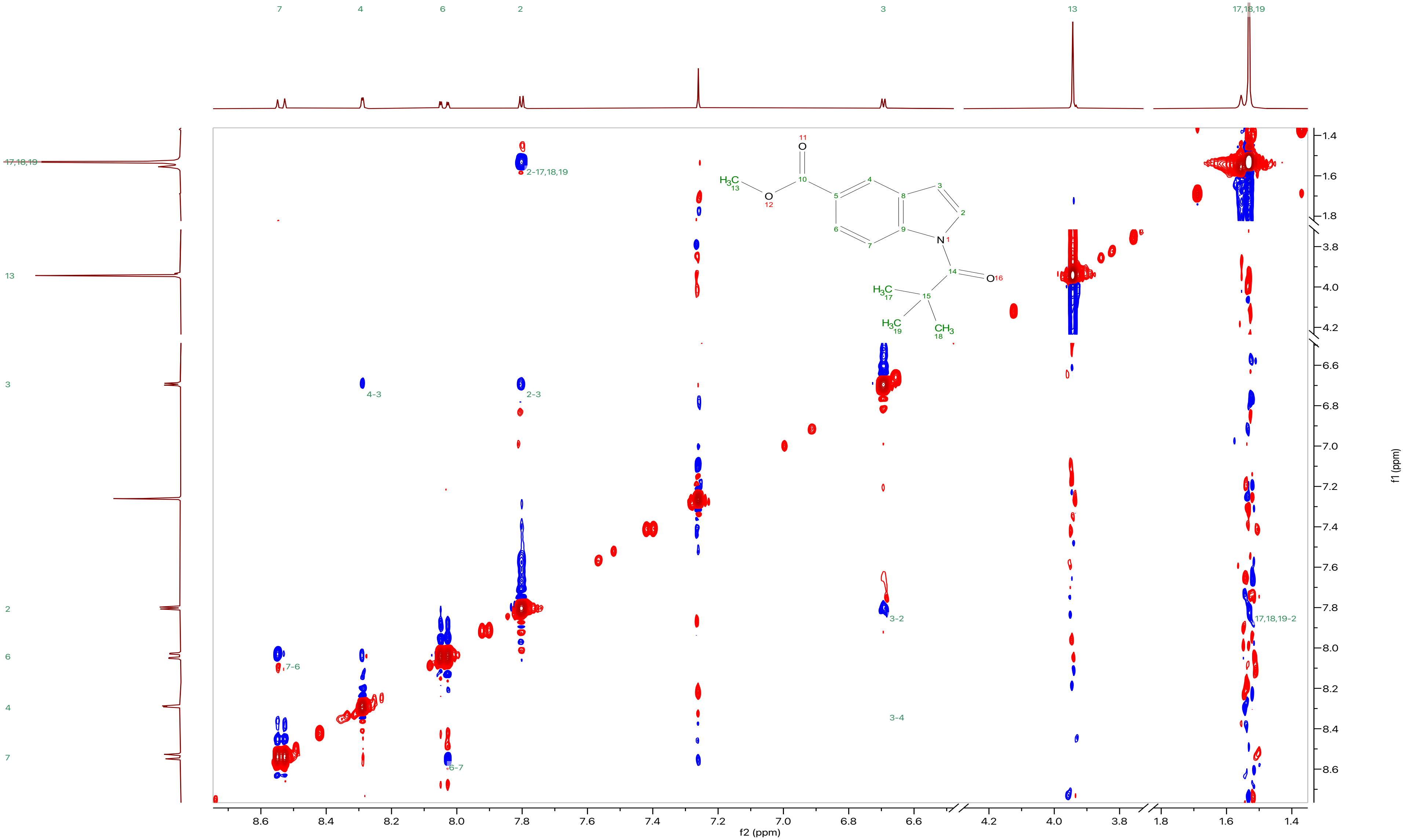

^{1}H-^{1}H NOESY (400 MHz, CDCl$_3$) of 1q

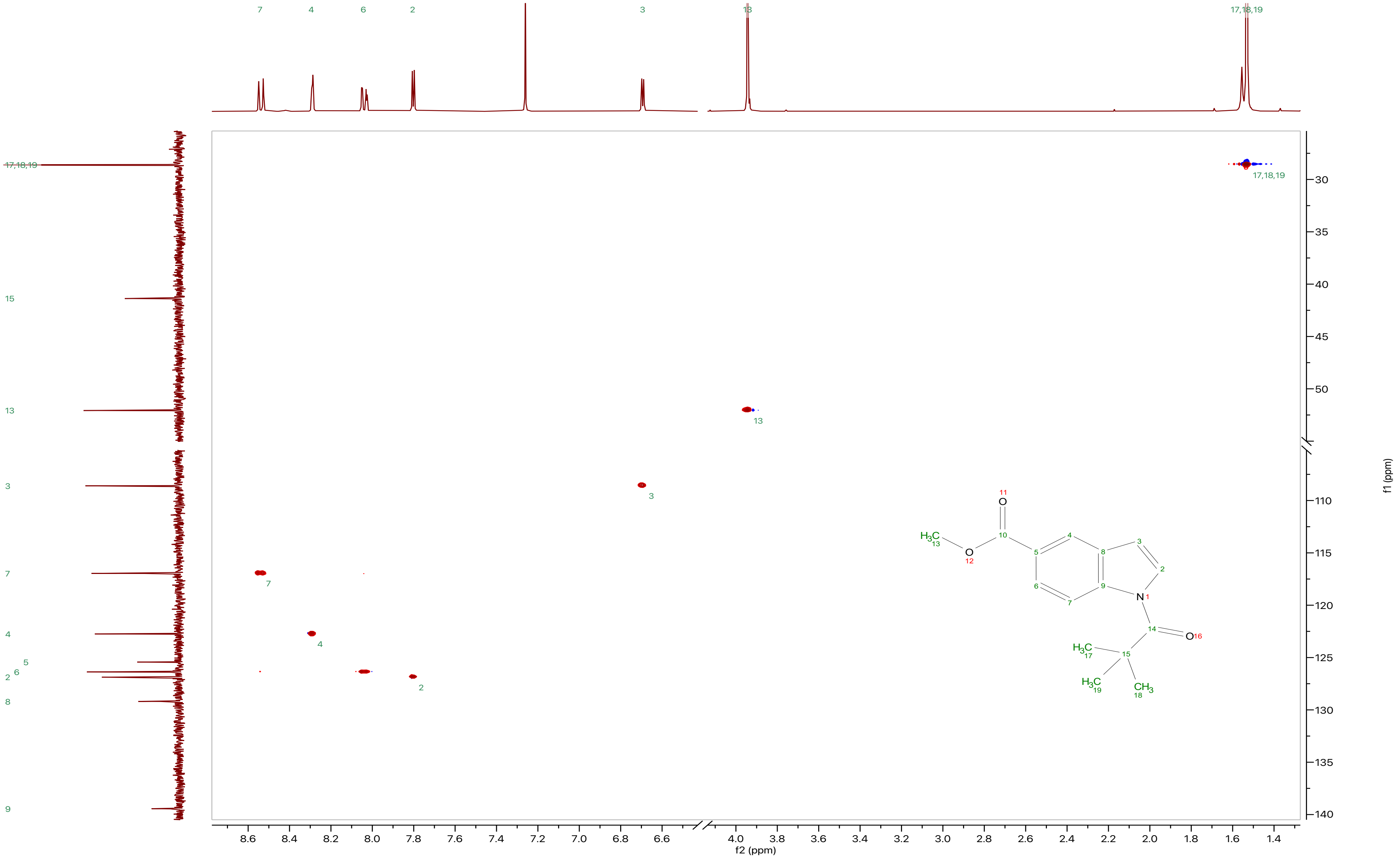

^{1}H-$^{13}C\{^{1}H\}$ HSQC NMR (400/101 MHz, $CDCl_3$) of 1q

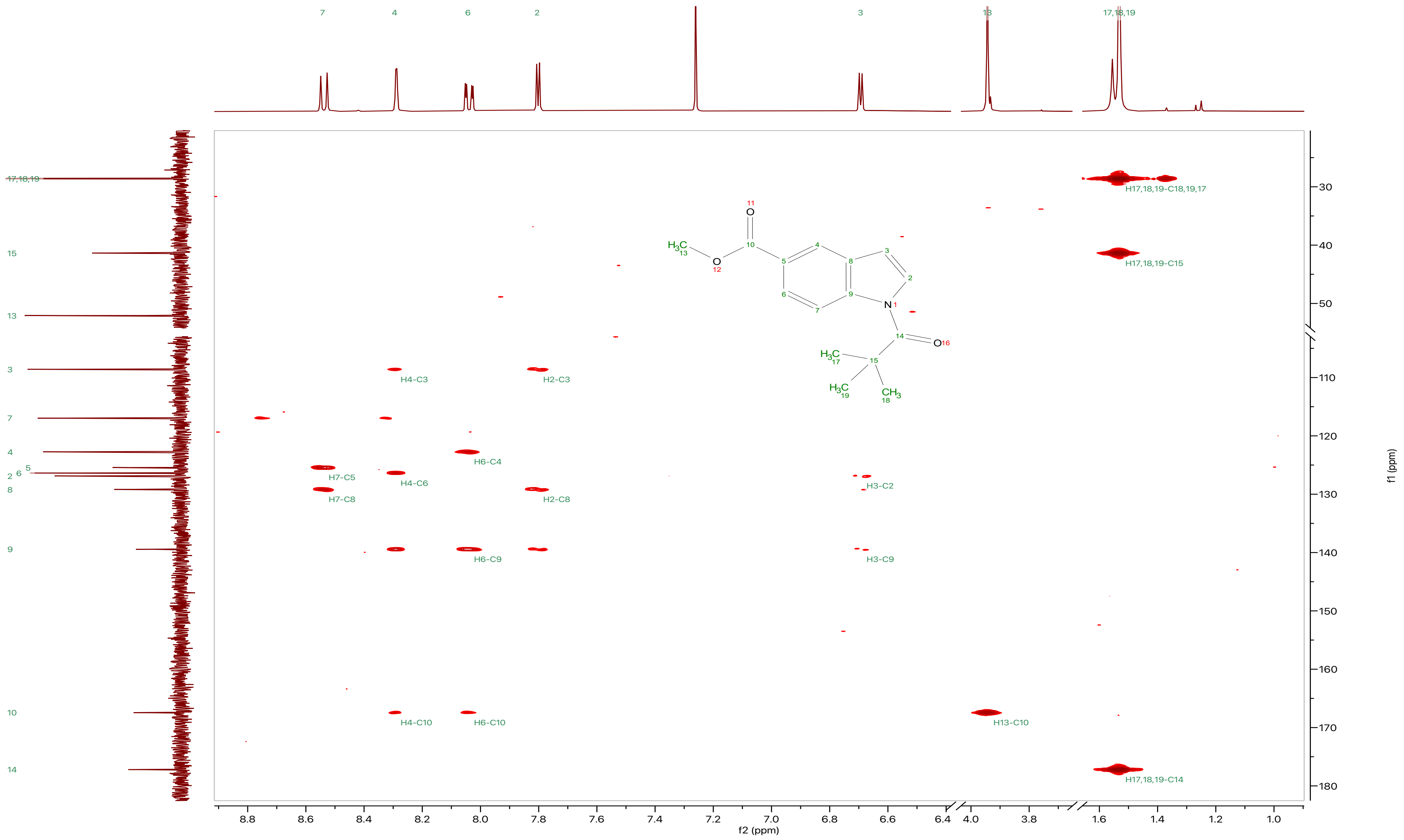

^{1}H-$^{13}C\{^{1}H\}$ HMBC NMR (400/101 MHz, $CDCl_3$) of 1q

2q

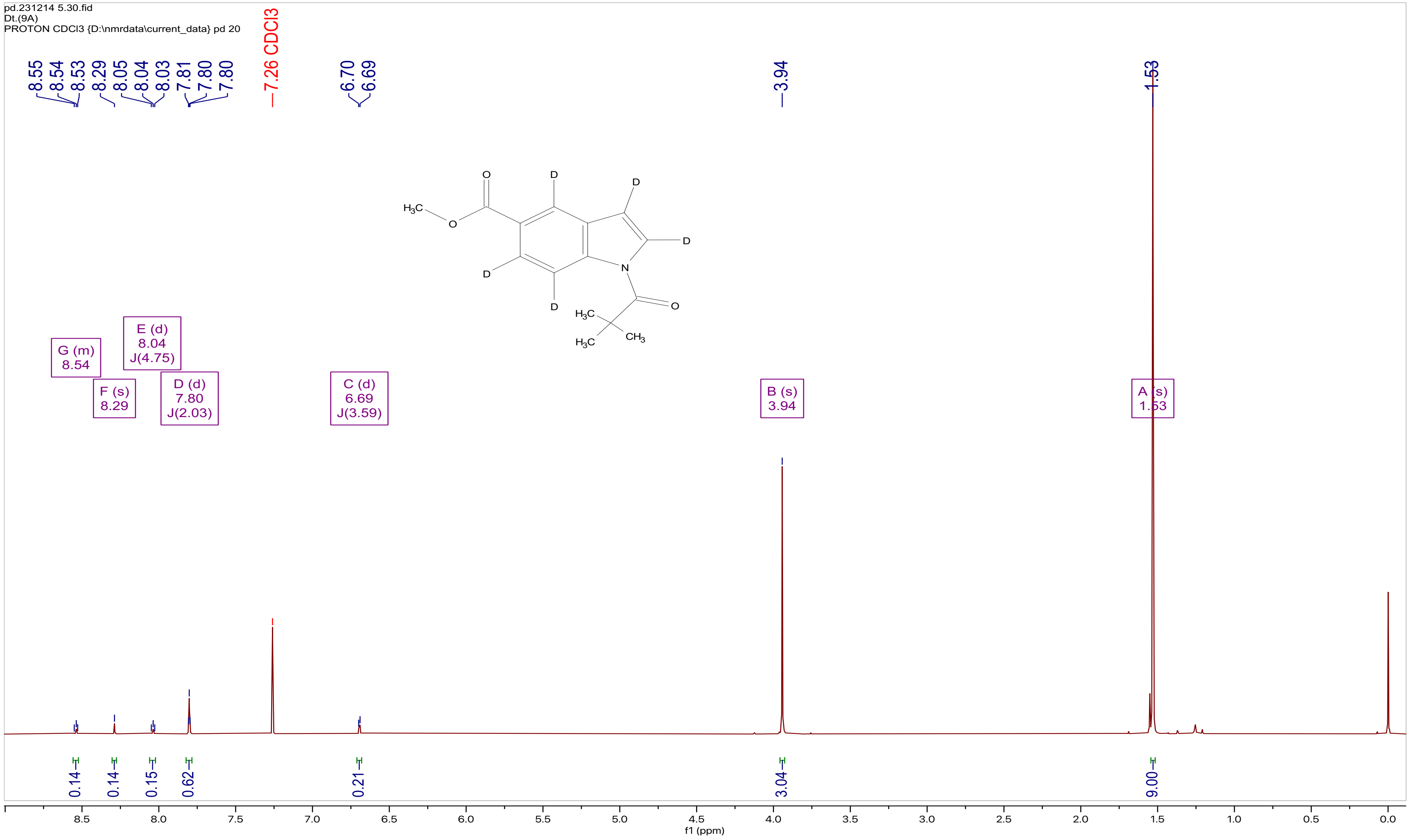

^{1}H NMR (400 MHz, $CDCl_3$) of 2q

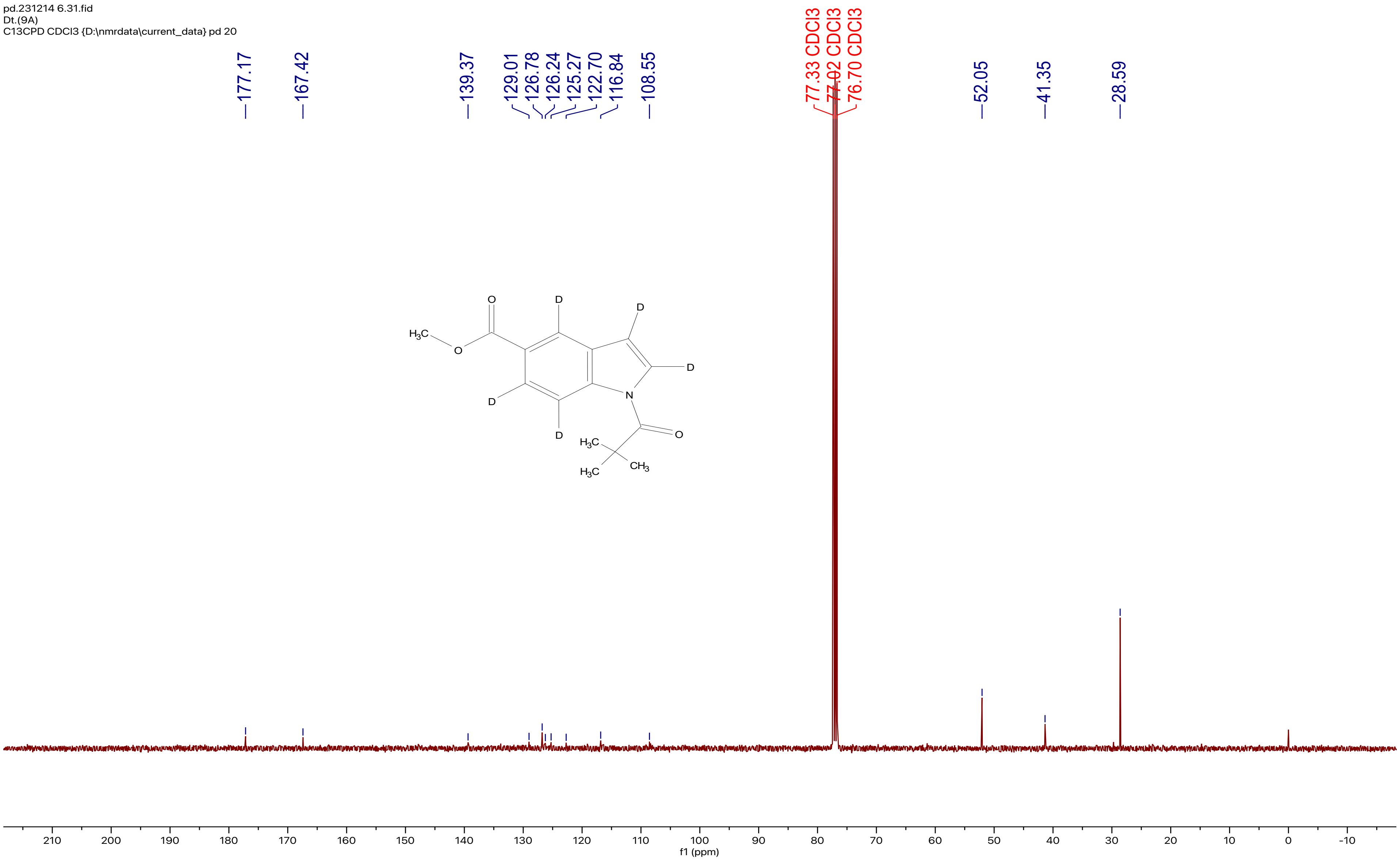

$^{13}C\{^1H\}$ NMR (101 MHz, $CDCl_3$) of 2q

1r

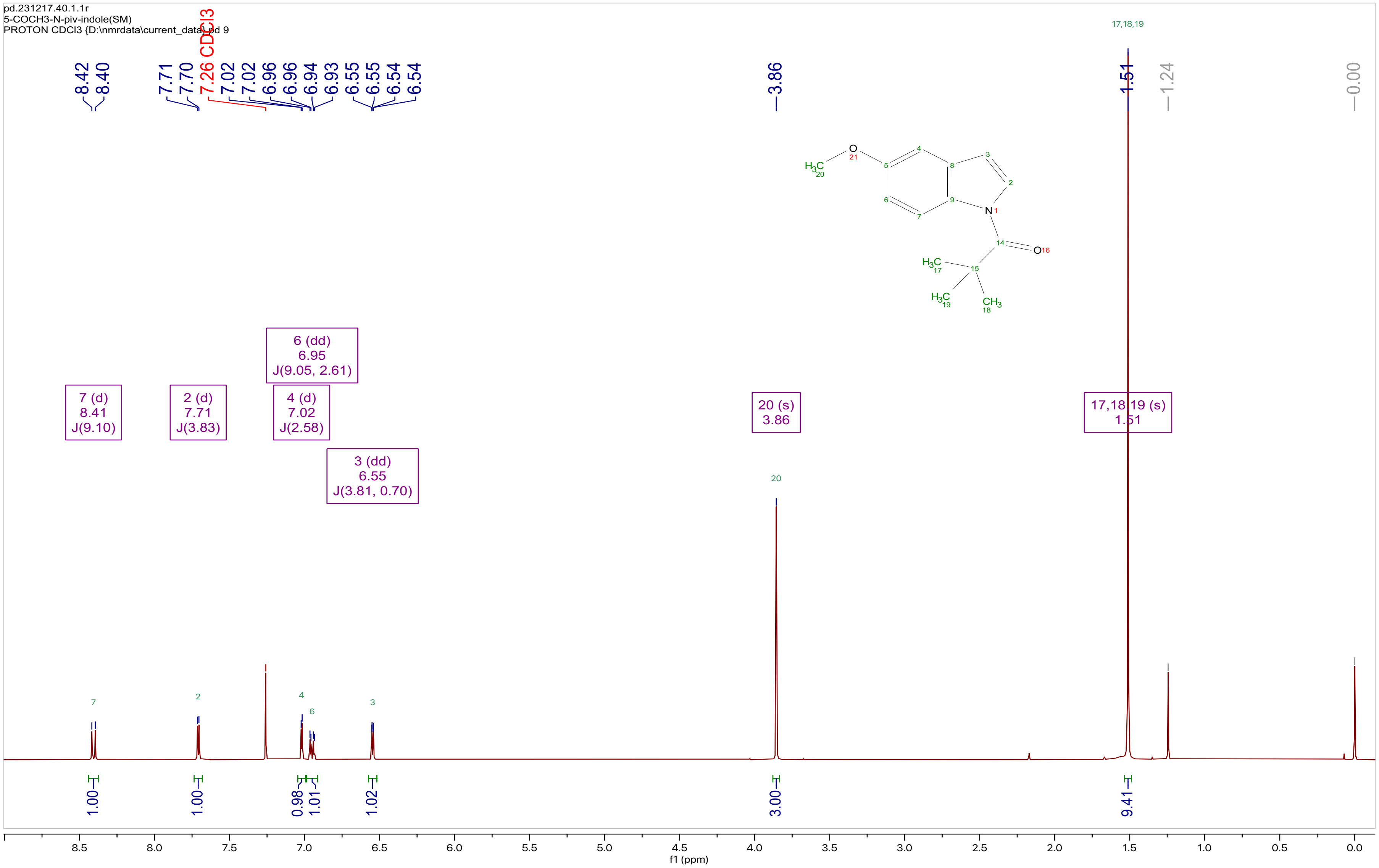

^{1}H NMR (400 MHz, $CDCl_3$) of 1r

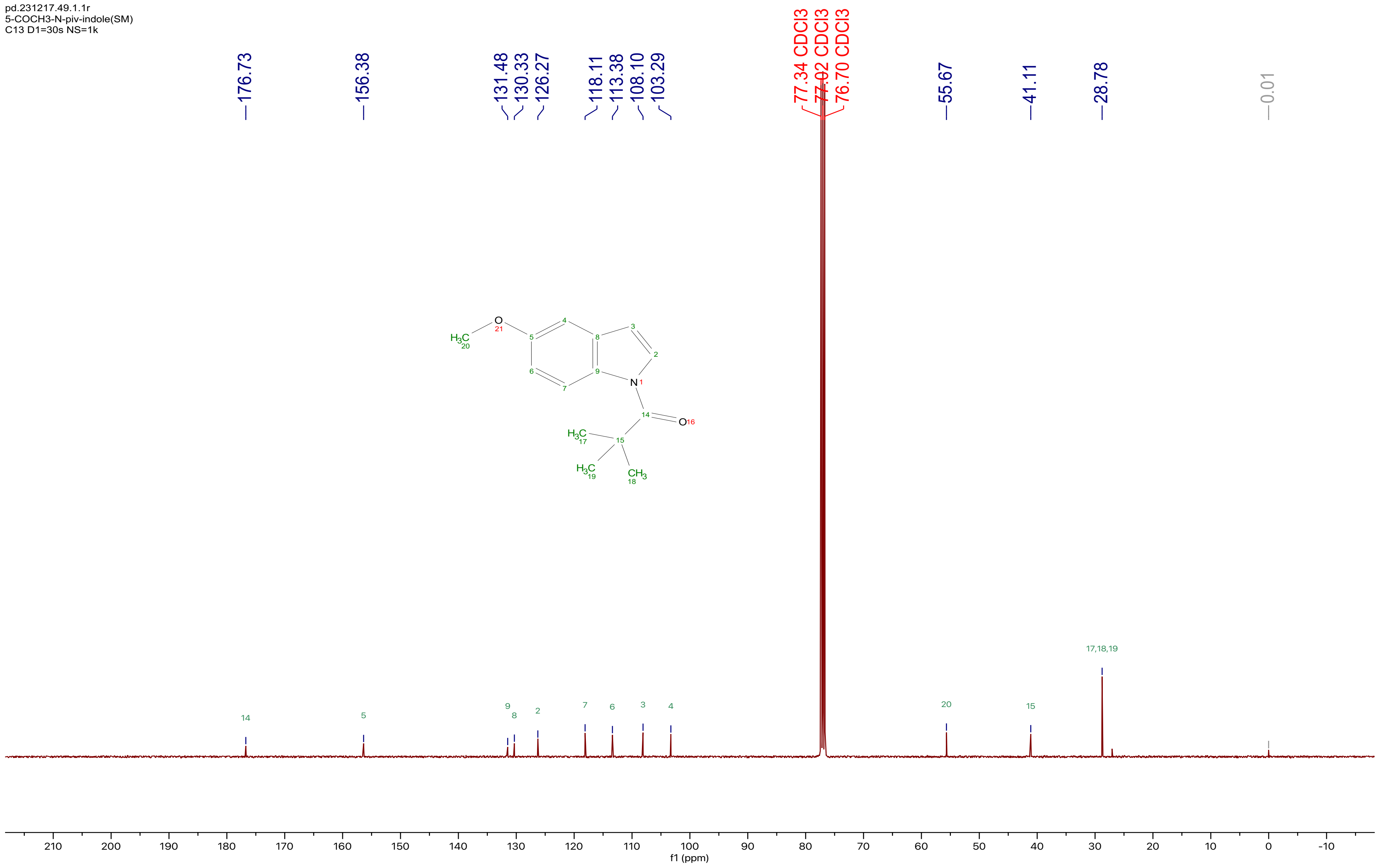

$^{13}C\{^{1}H\}$ NMR (101 MHz, $CDCl_3$) of 1r

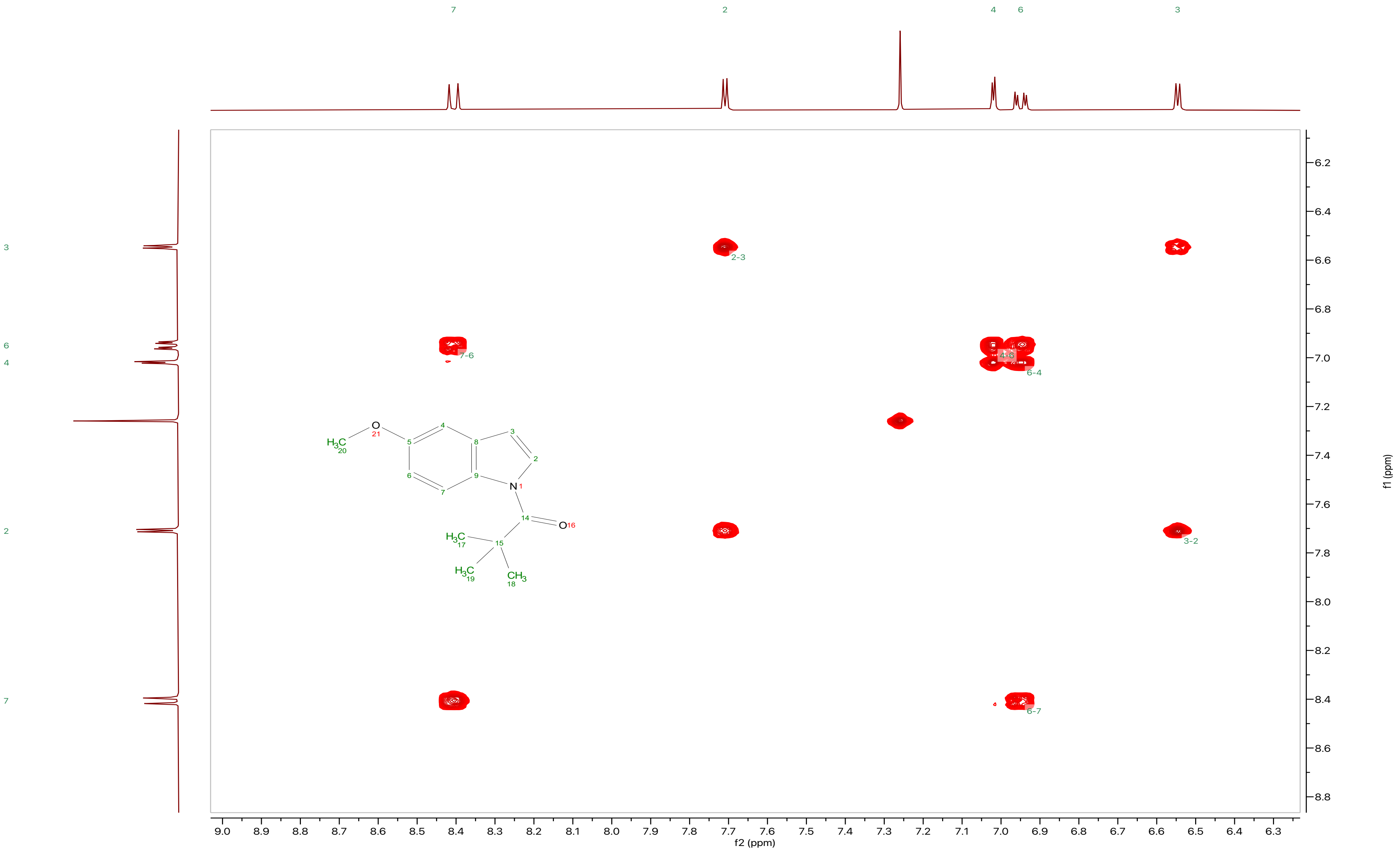

^{1}H-^{1}H COSY (400 MHz, $CDCl_3$) of 1r

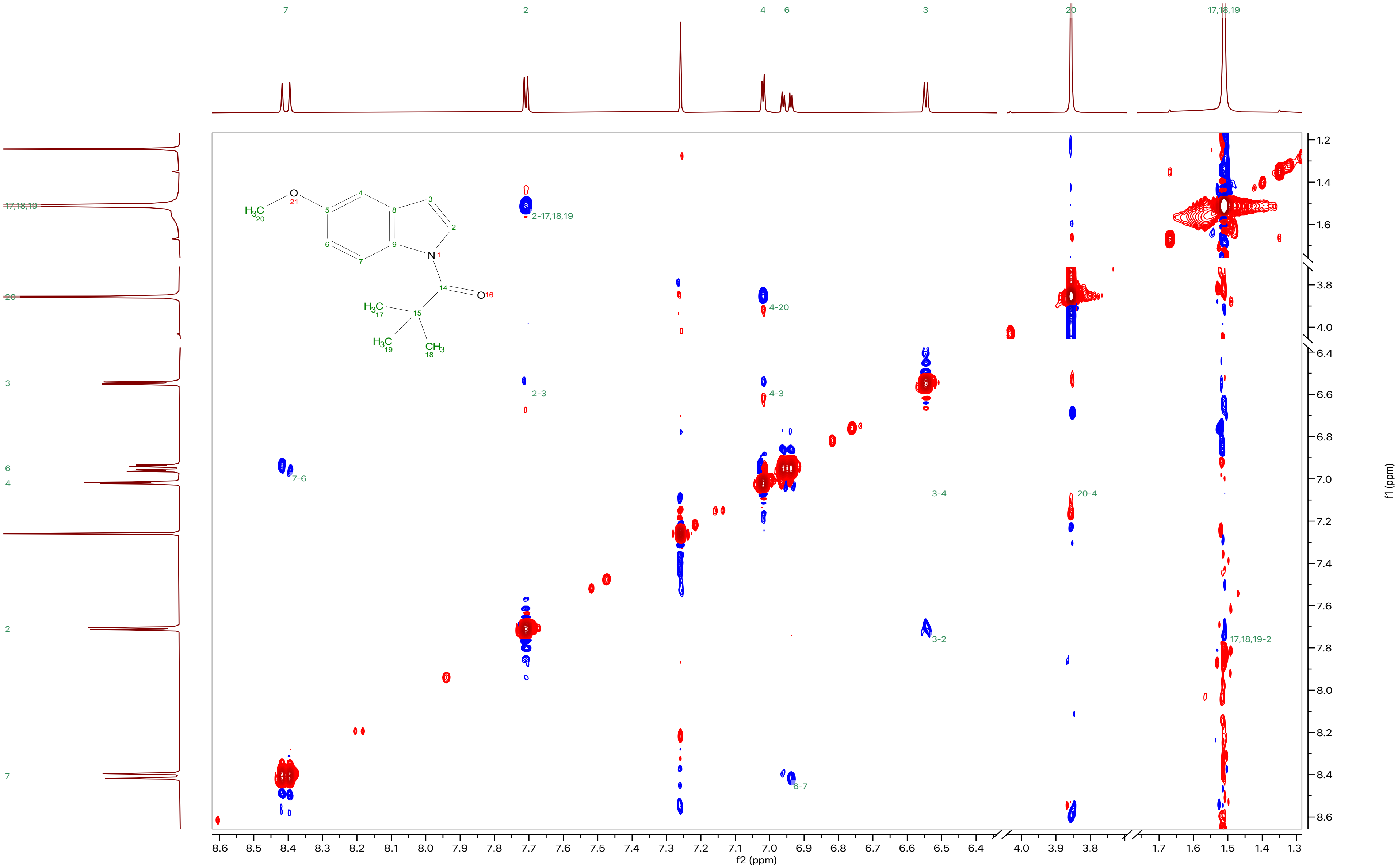

^1H-^1H NOESY (400 MHz, $CDCl_3$) of 1r

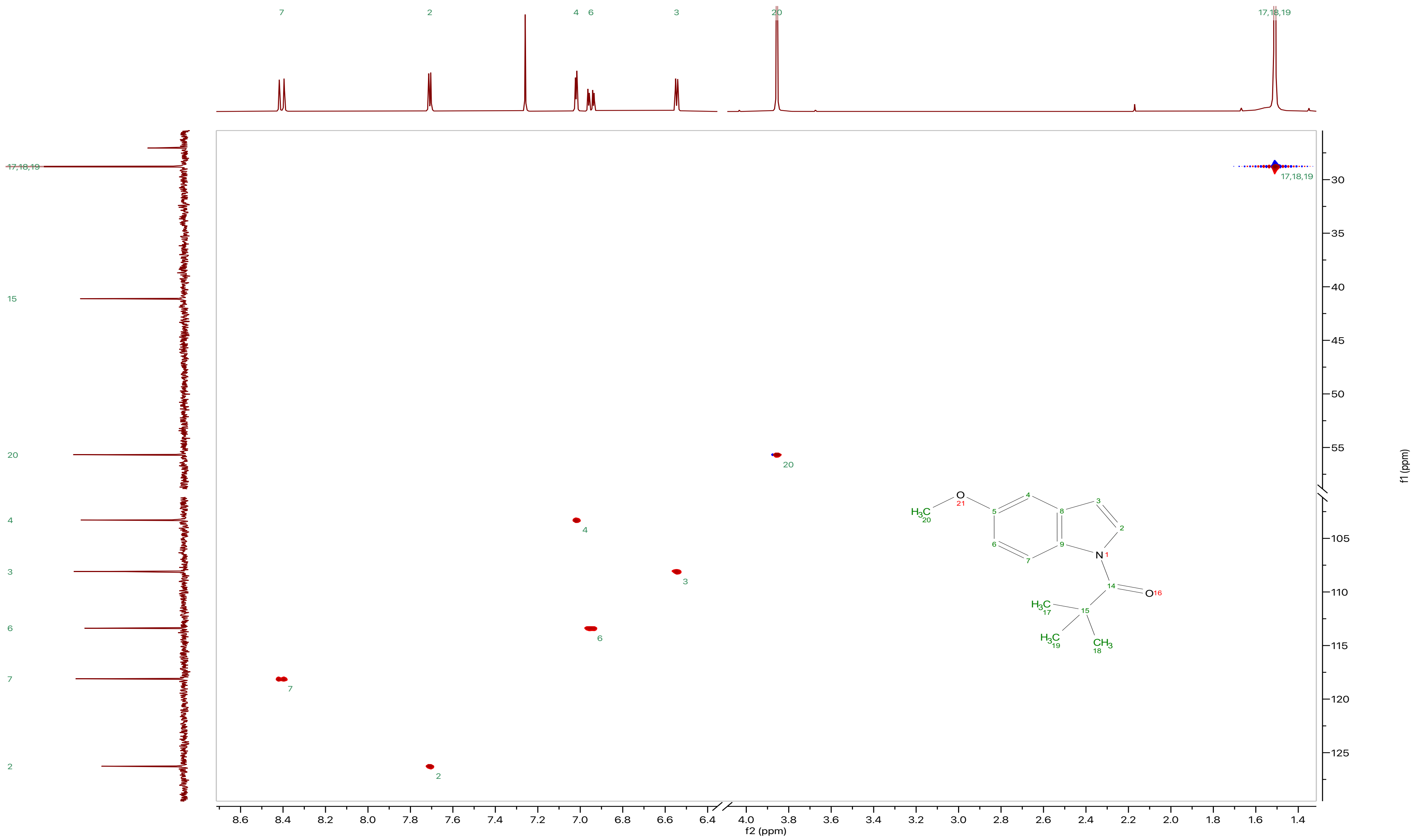

^{1}H-$^{13}C\{^{1}H\}$ HSQC NMR (400/101 MHz, $CDCl_3$) of 1r

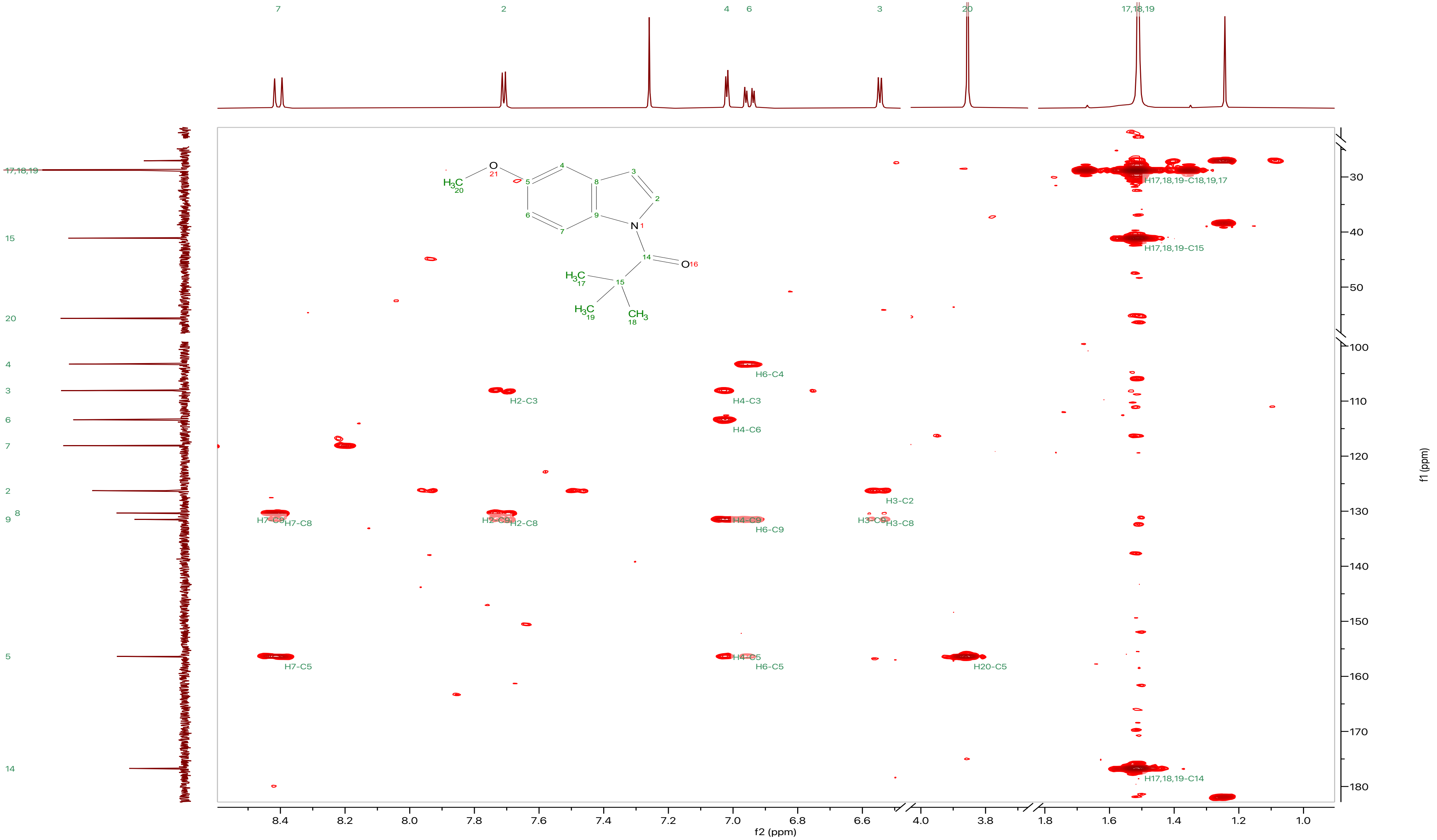

^{1}H-$^{13}C\{^{1}H\}$ HMBC NMR (400/101 MHz, $CDCl_3$) of 1r

2r

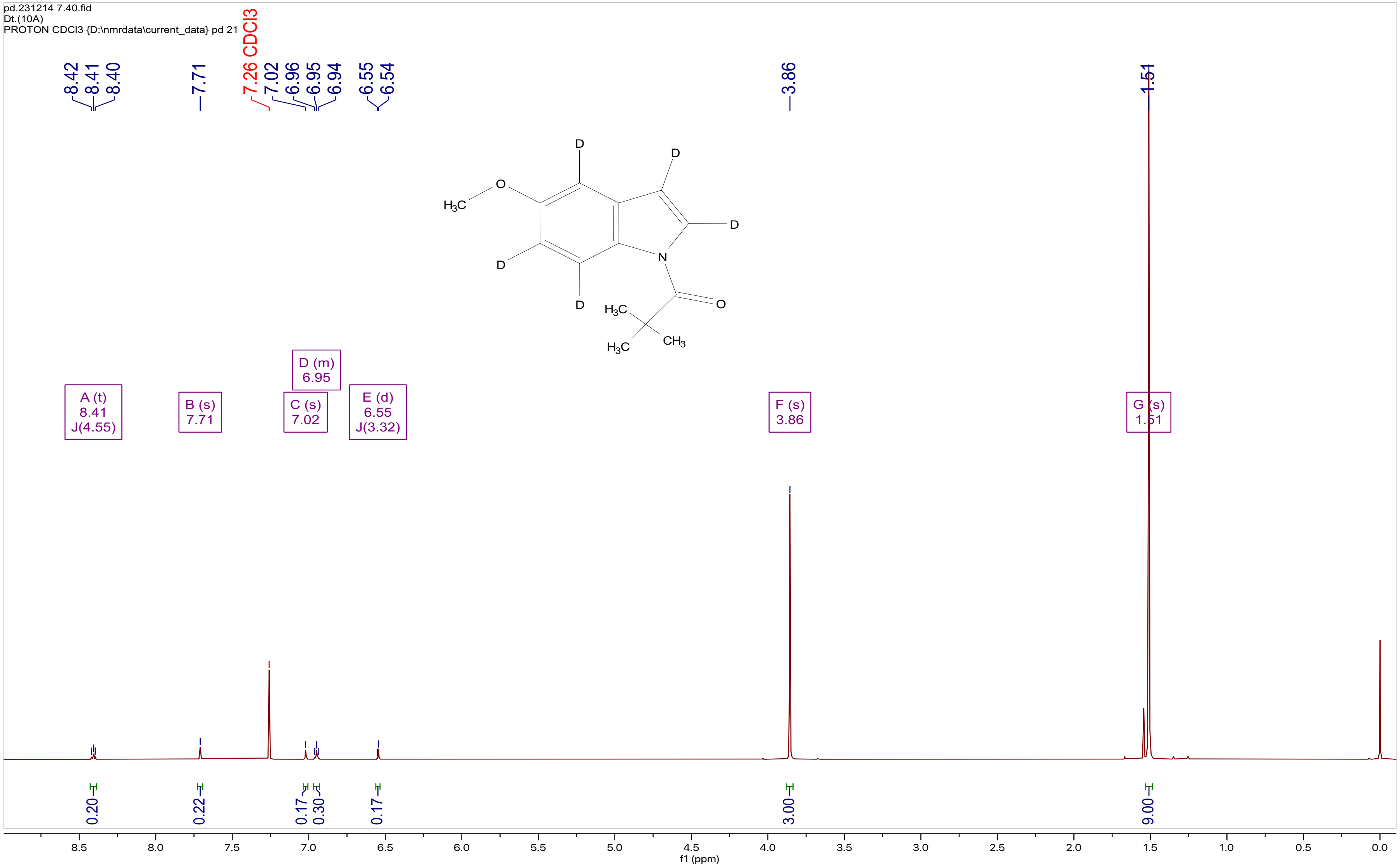

^{1}H NMR (400 MHz, $CDCl_3$) of 2r

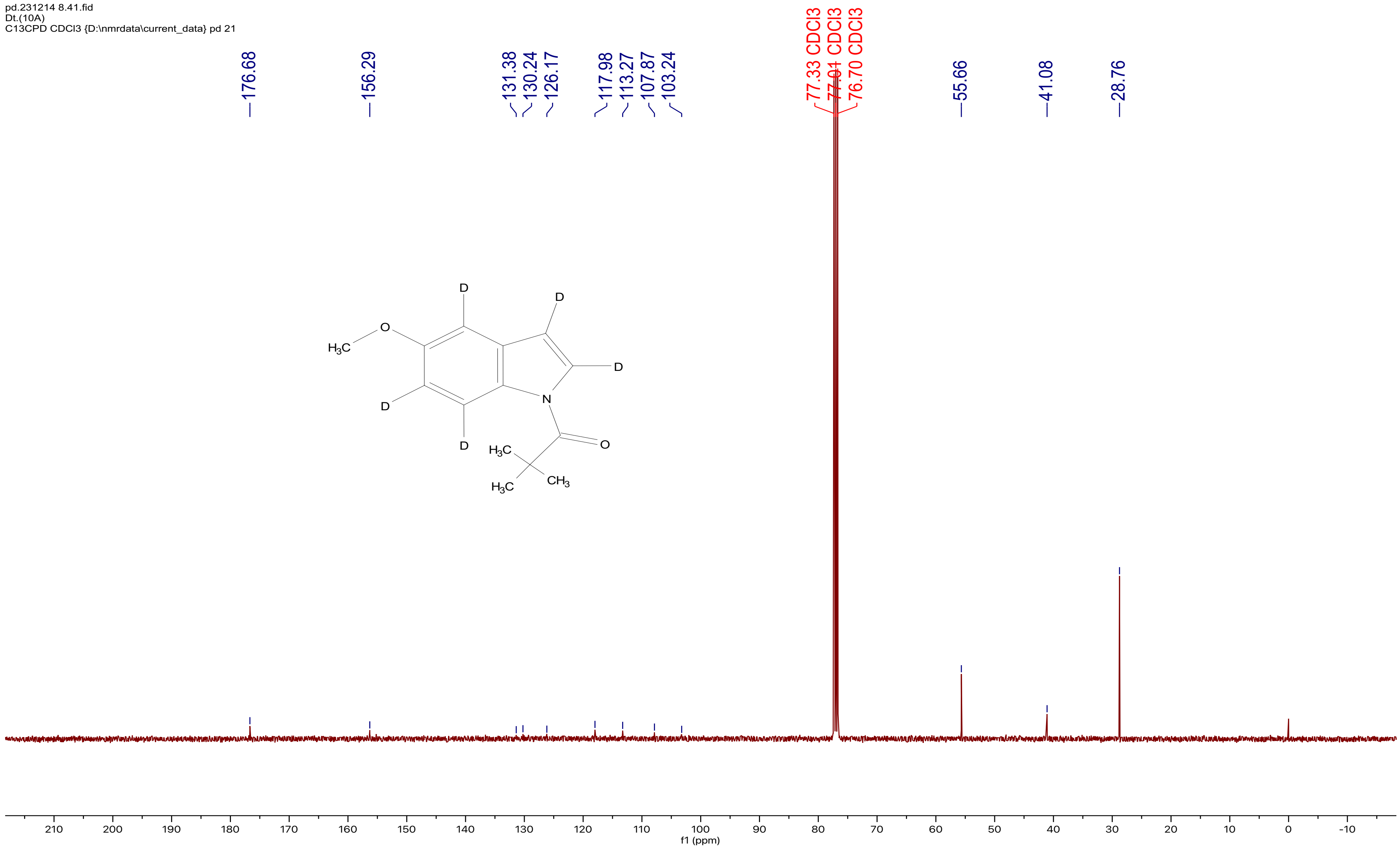

$^{13}C\{^{1}H\}$ NMR (101 MHz, $CDCl_3$) of 2r

1s

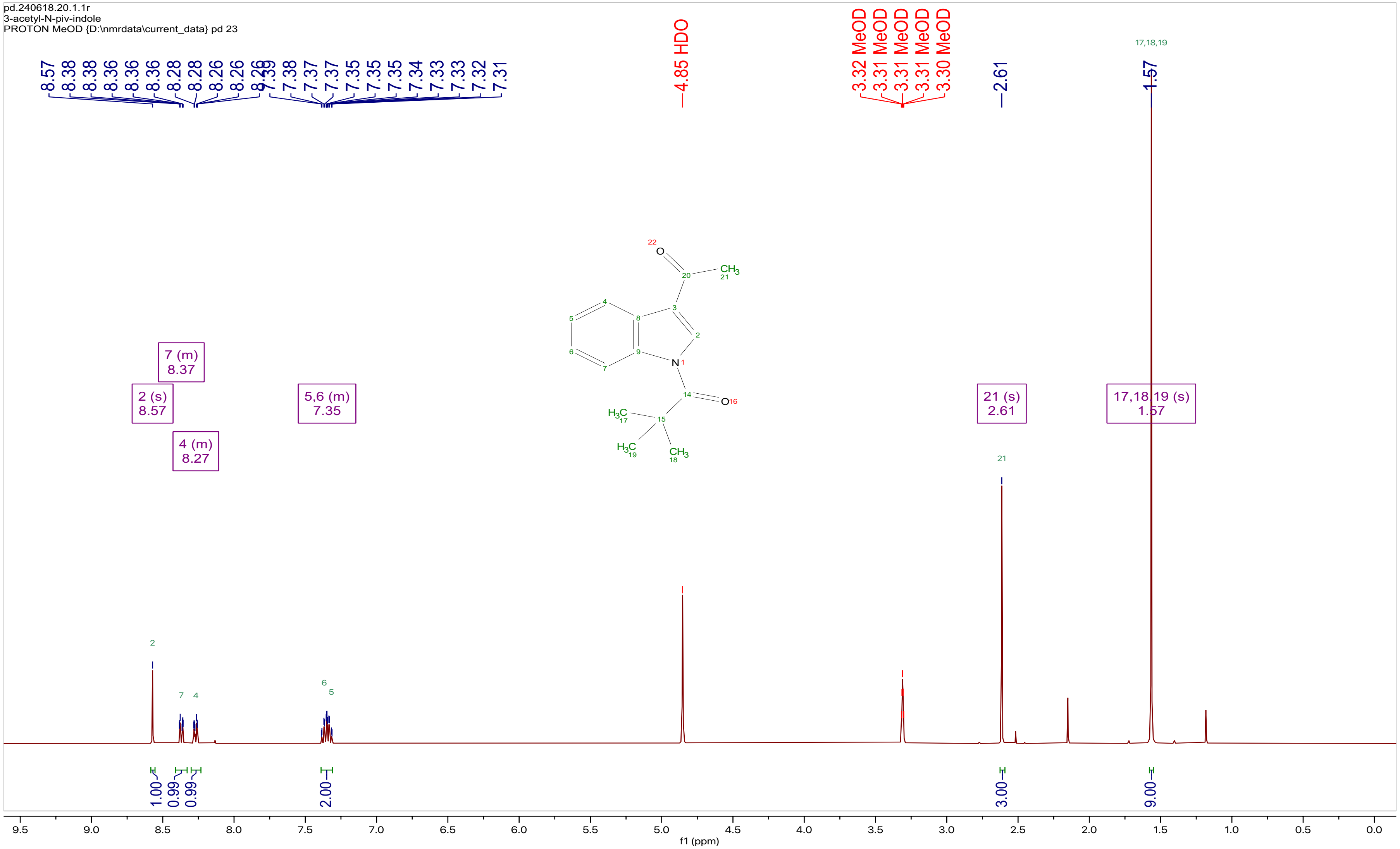

^{1}H NMR (400 MHz, MeOD) of 1s

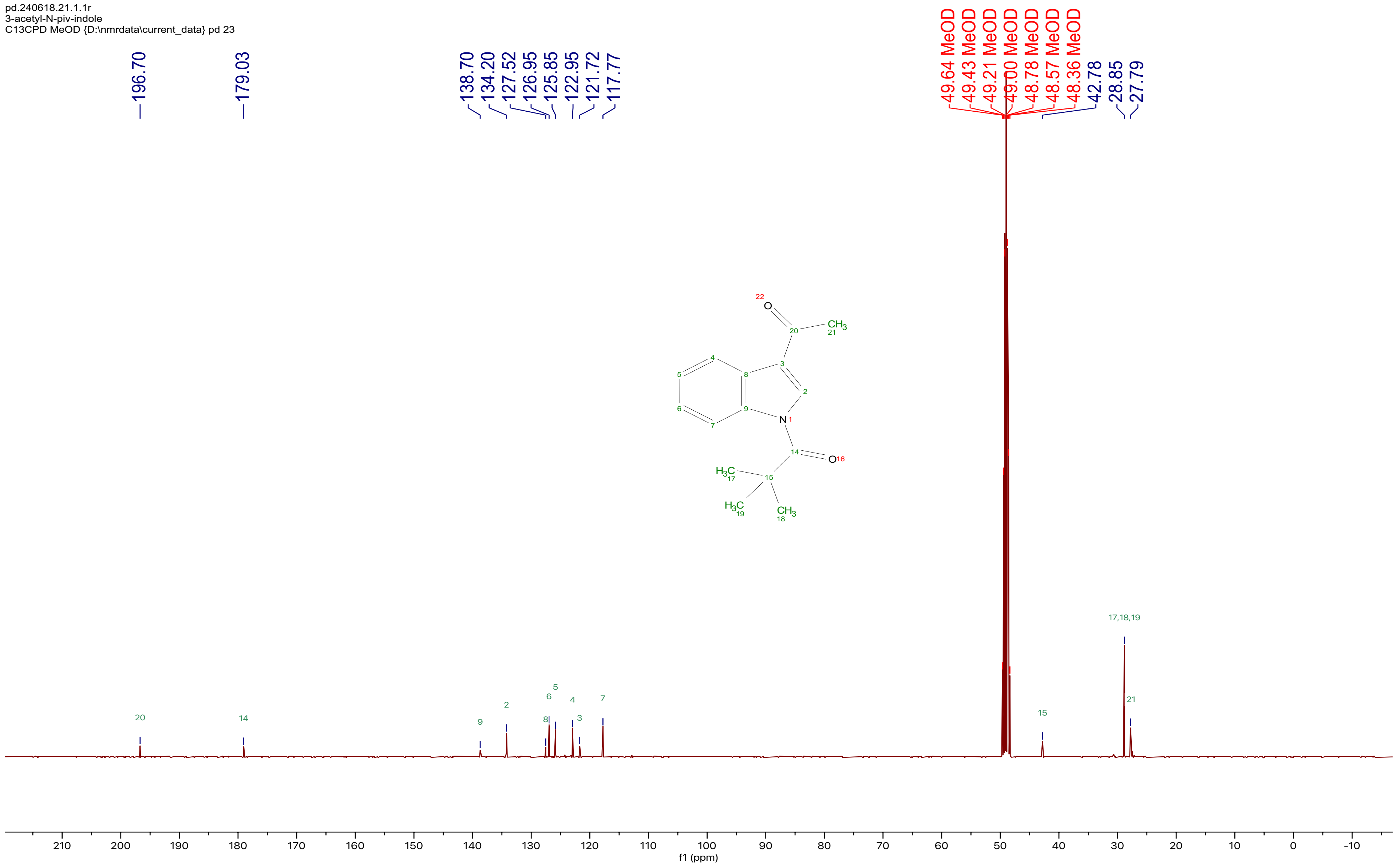

$^{13}C\{^{1}H\}$ NMR (101 MHz, MeOD) of 1s

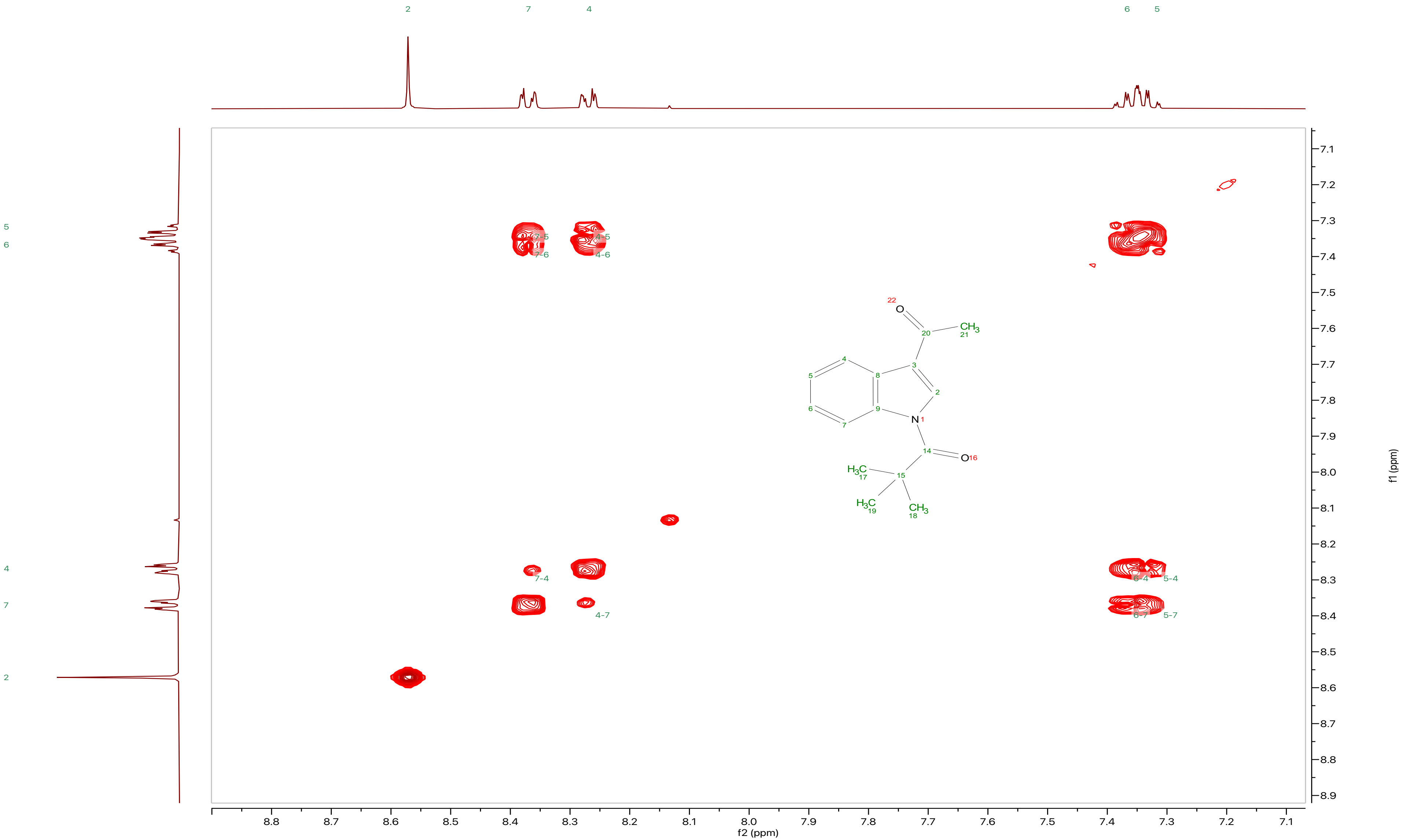

^{1}H-^{1}H COSY (400 MHz, MeOD) of 1s

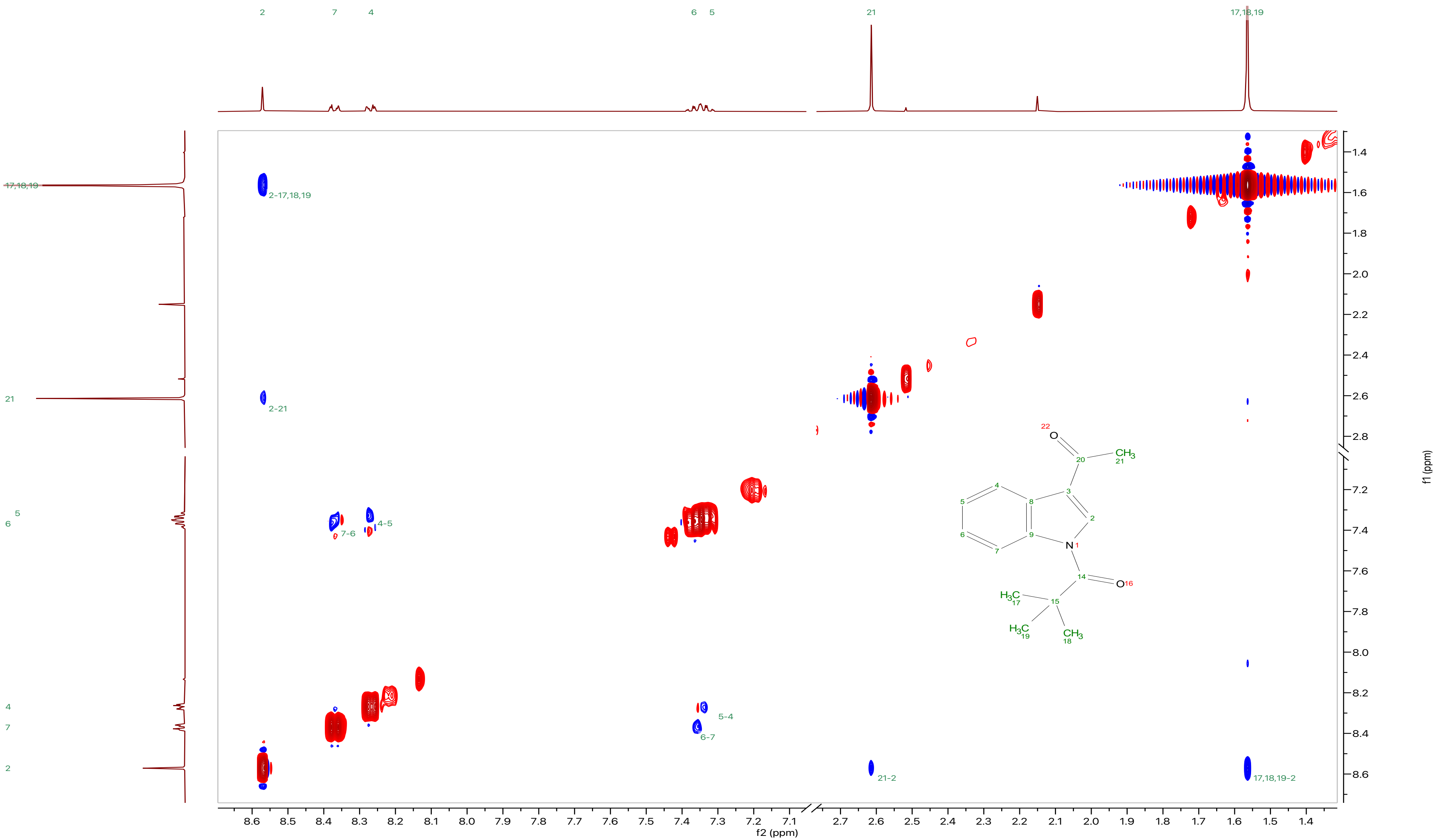

^1H-^1H NOESY (400 MHz, MeOD) of 1s

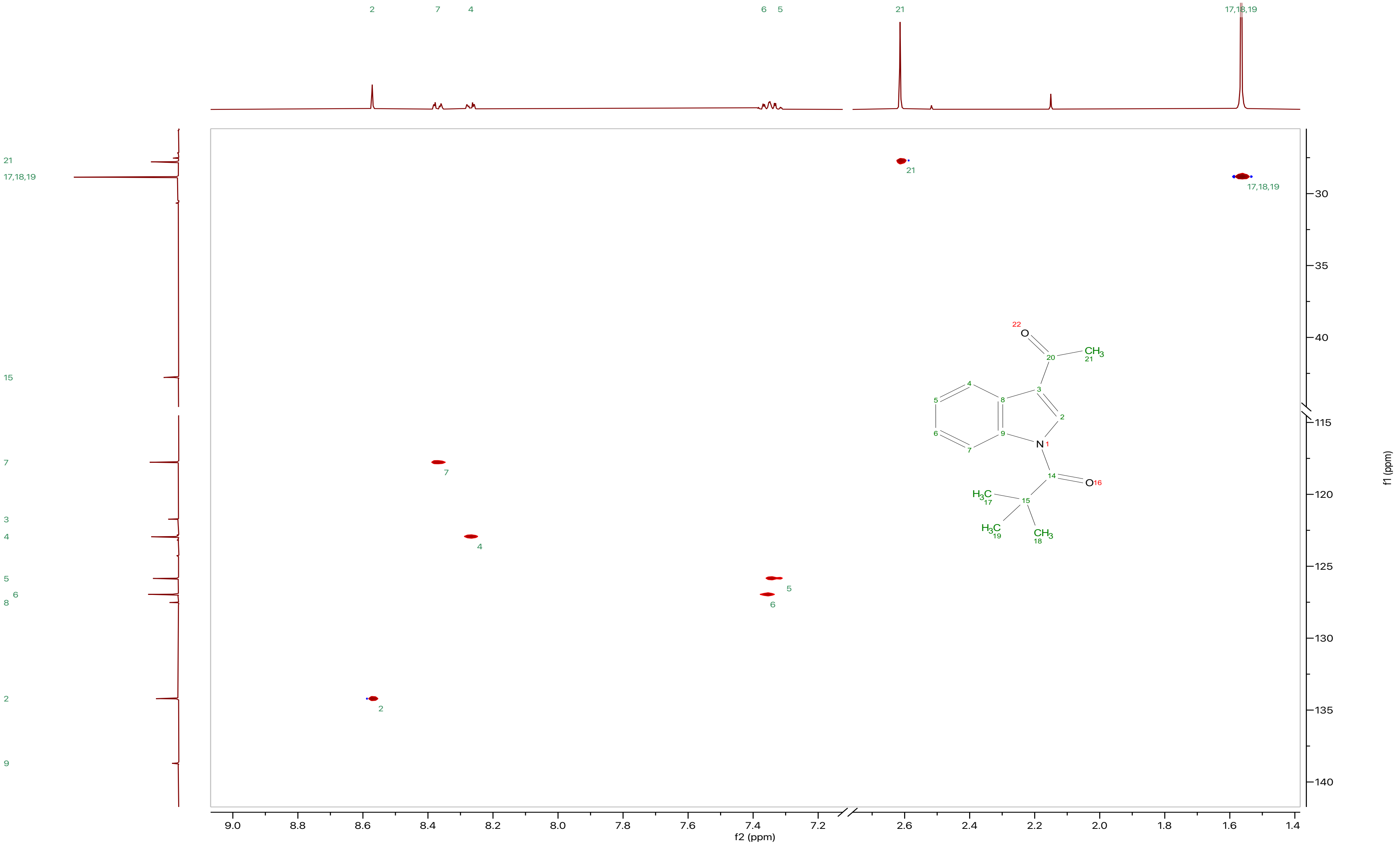

^{1}H-$^{13}C\{^{1}H\}$ HSQC NMR (400/101 MHz, MeOD) of 1s

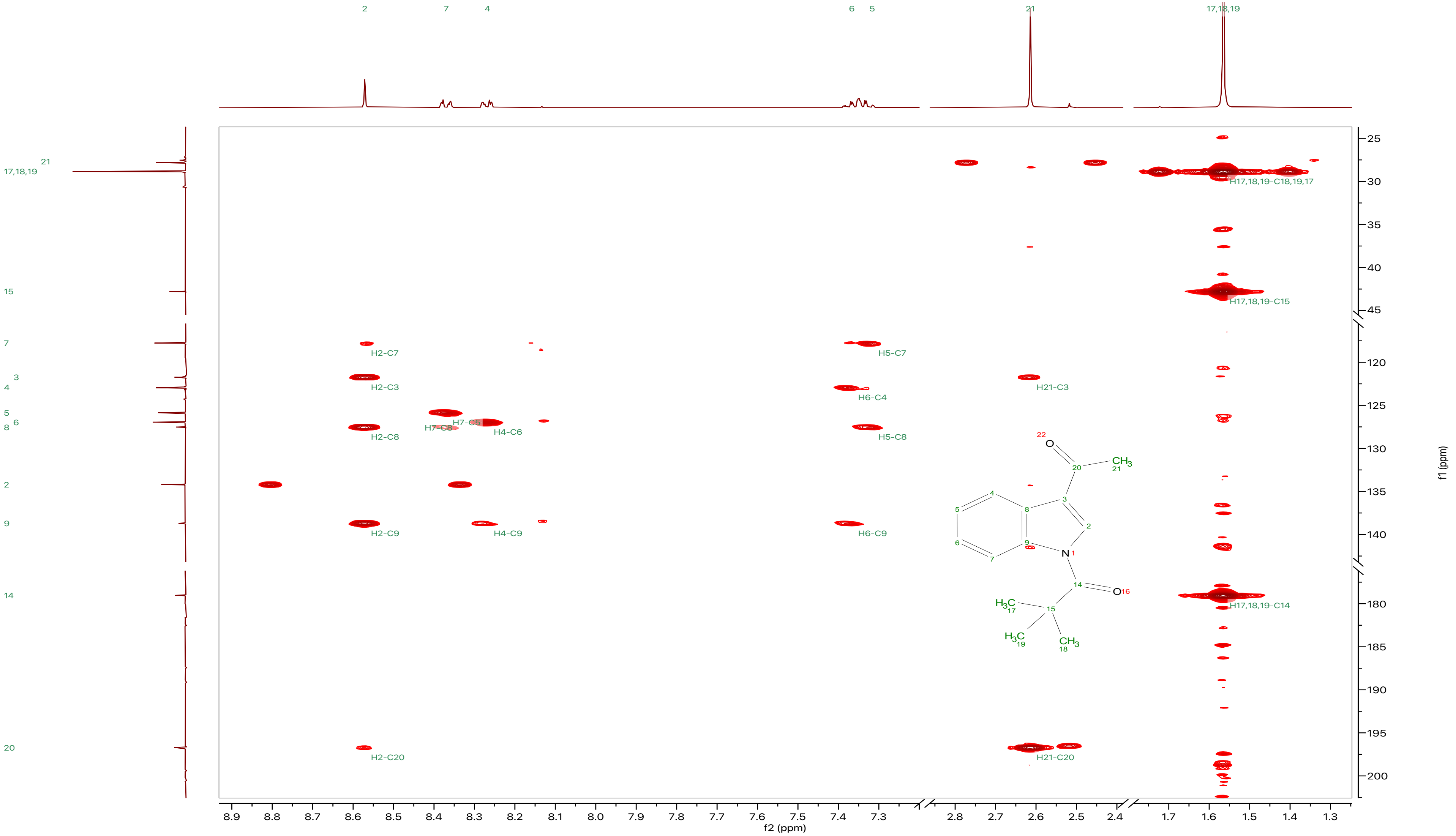

^1H-$^{13}C\{^1H\}$ HMBC NMR (400/101 MHz, MeOD) of 1s

2s

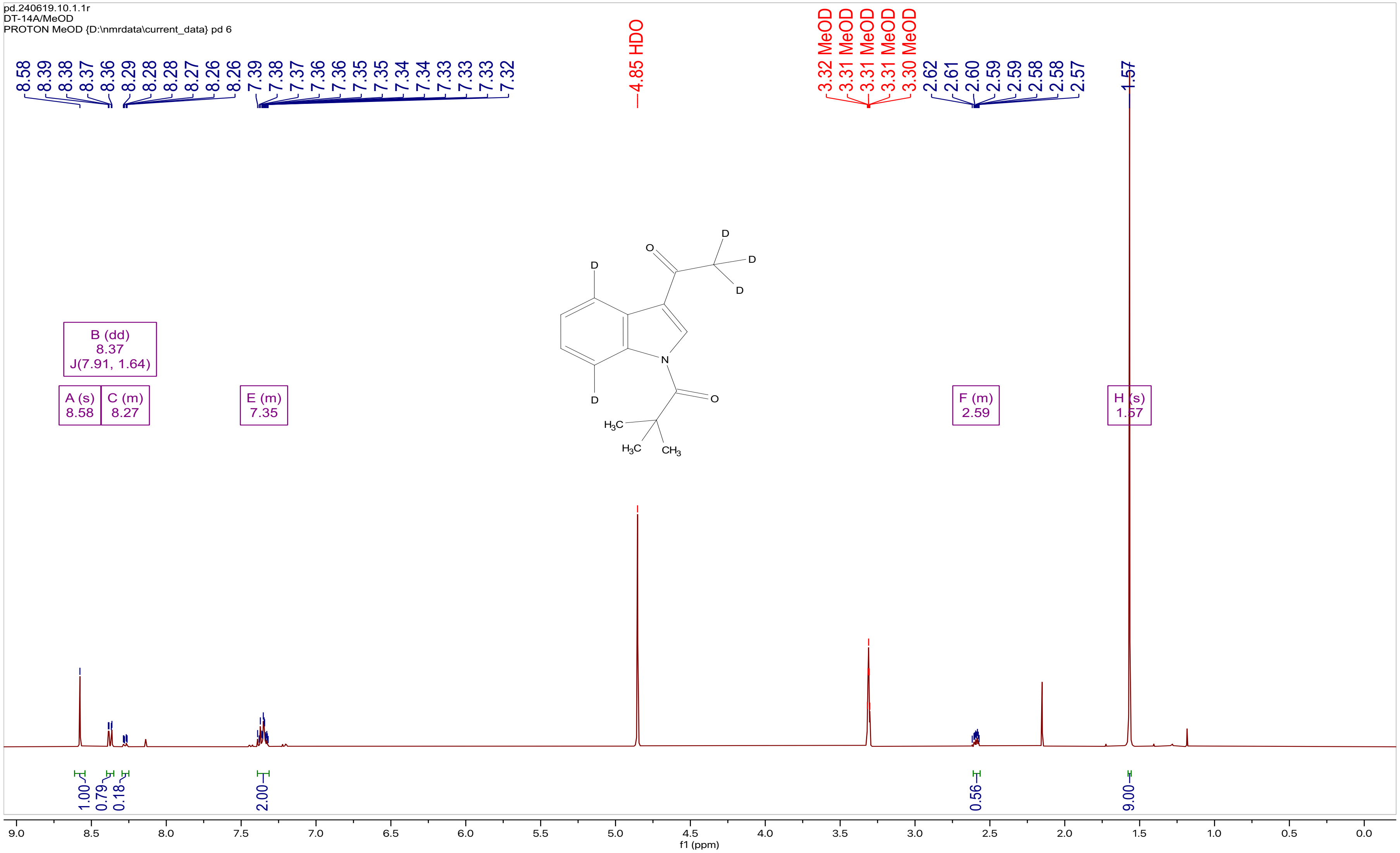

^{1}H NMR (400 MHz, MeOD) of 2s

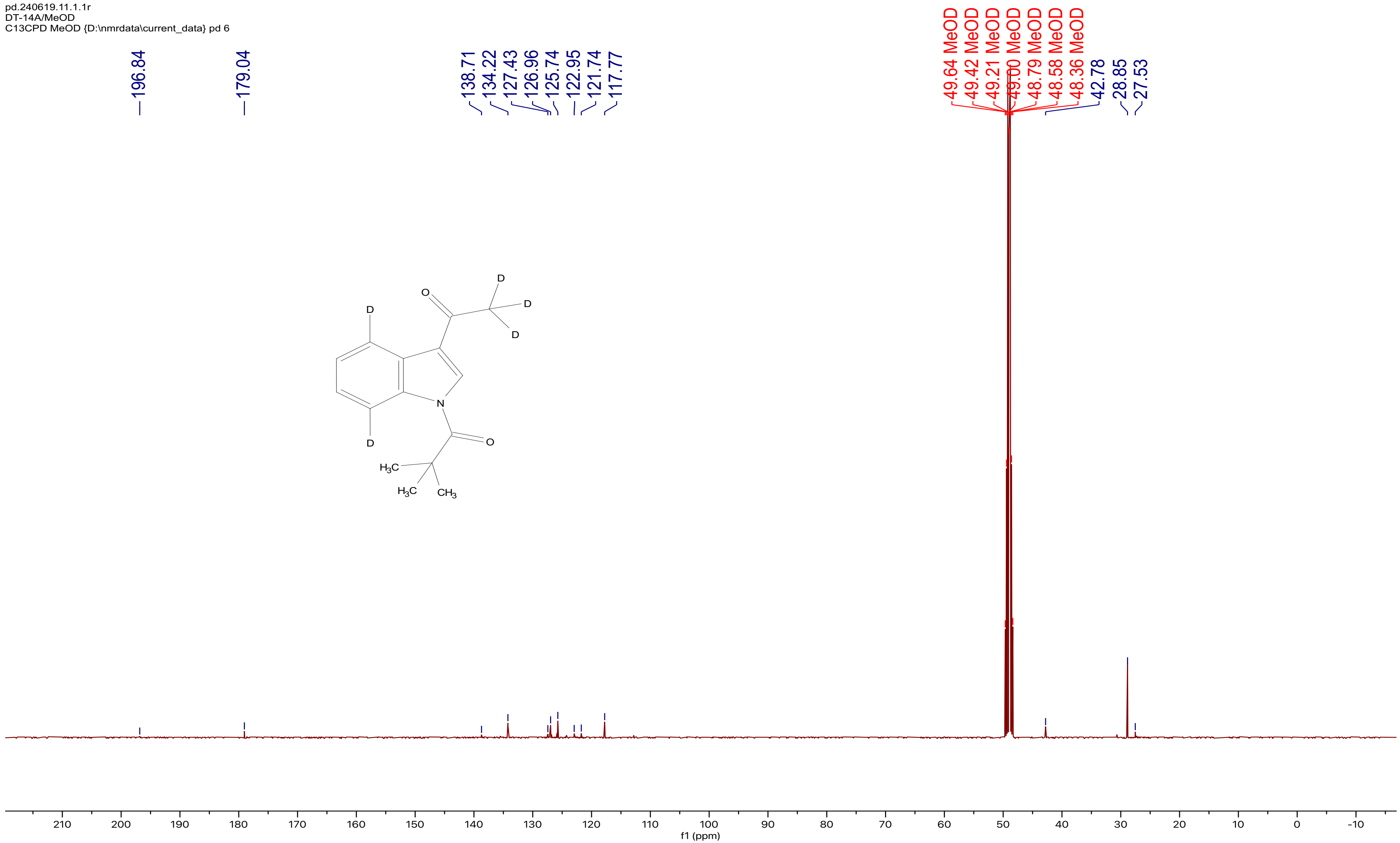

$^{13}C\{^1H\}$ NMR (101 MHz, MeOD) of 2s

1t

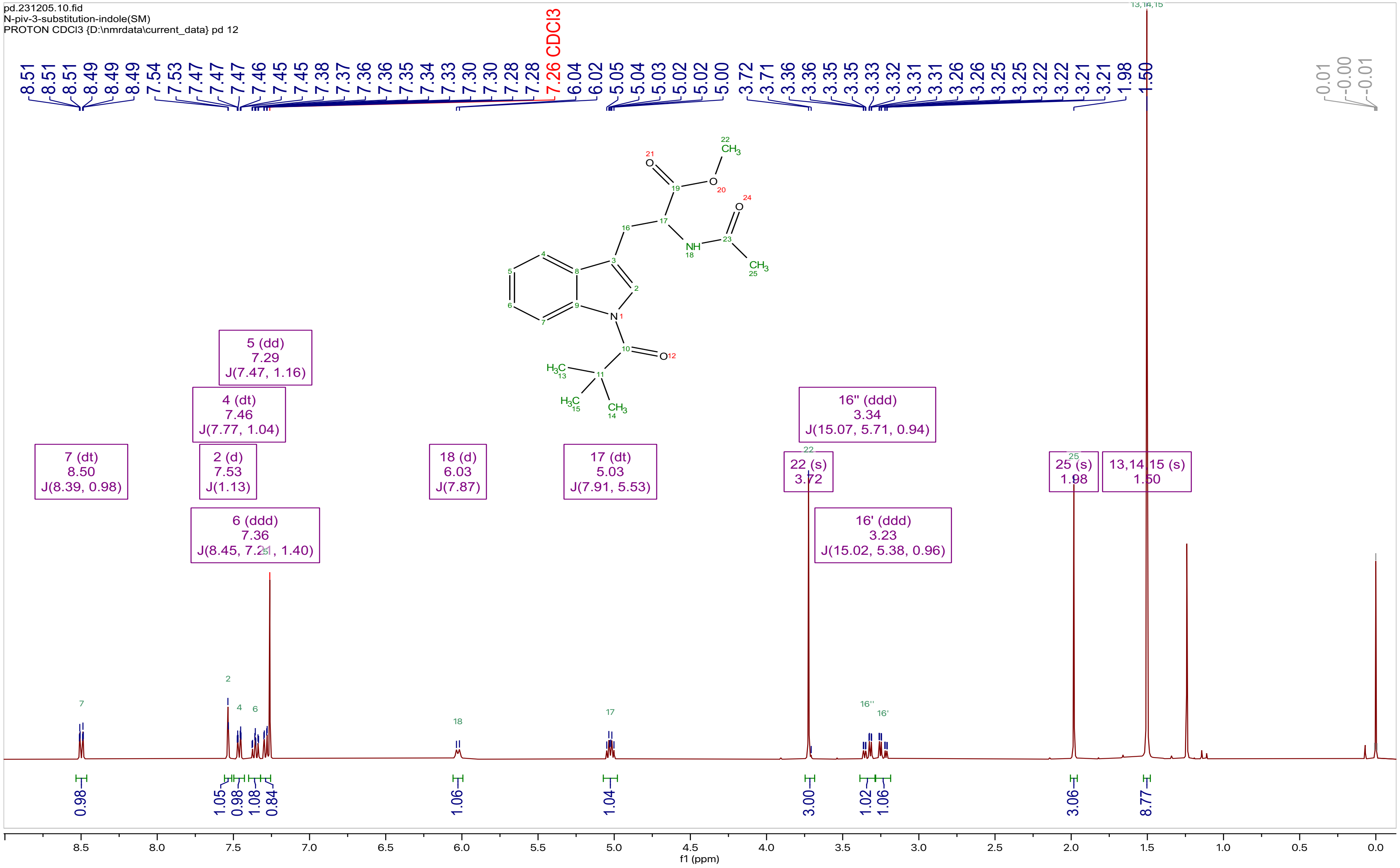

^{1}H NMR (400 MHz, $CDCl_3$) of 1t

pd.231205.11.fid
N-piv-3-substitution-indole(SM)
C13CPD CDCl3 {D:\nmrdata\current_data} pd 12

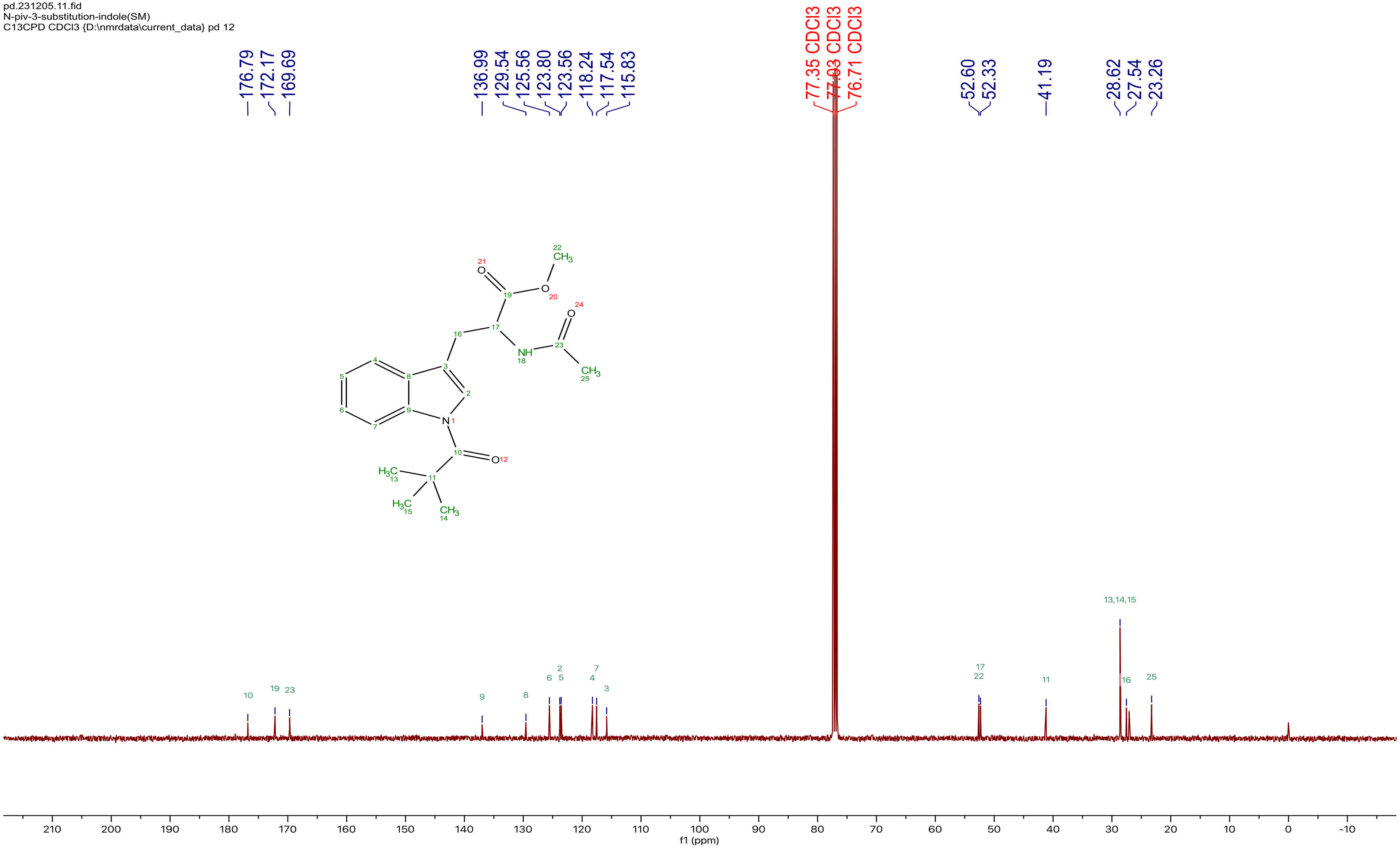

$^{13}C\{^{1}H\}$ NMR (101 MHz, $CDCl_3$) of 1t

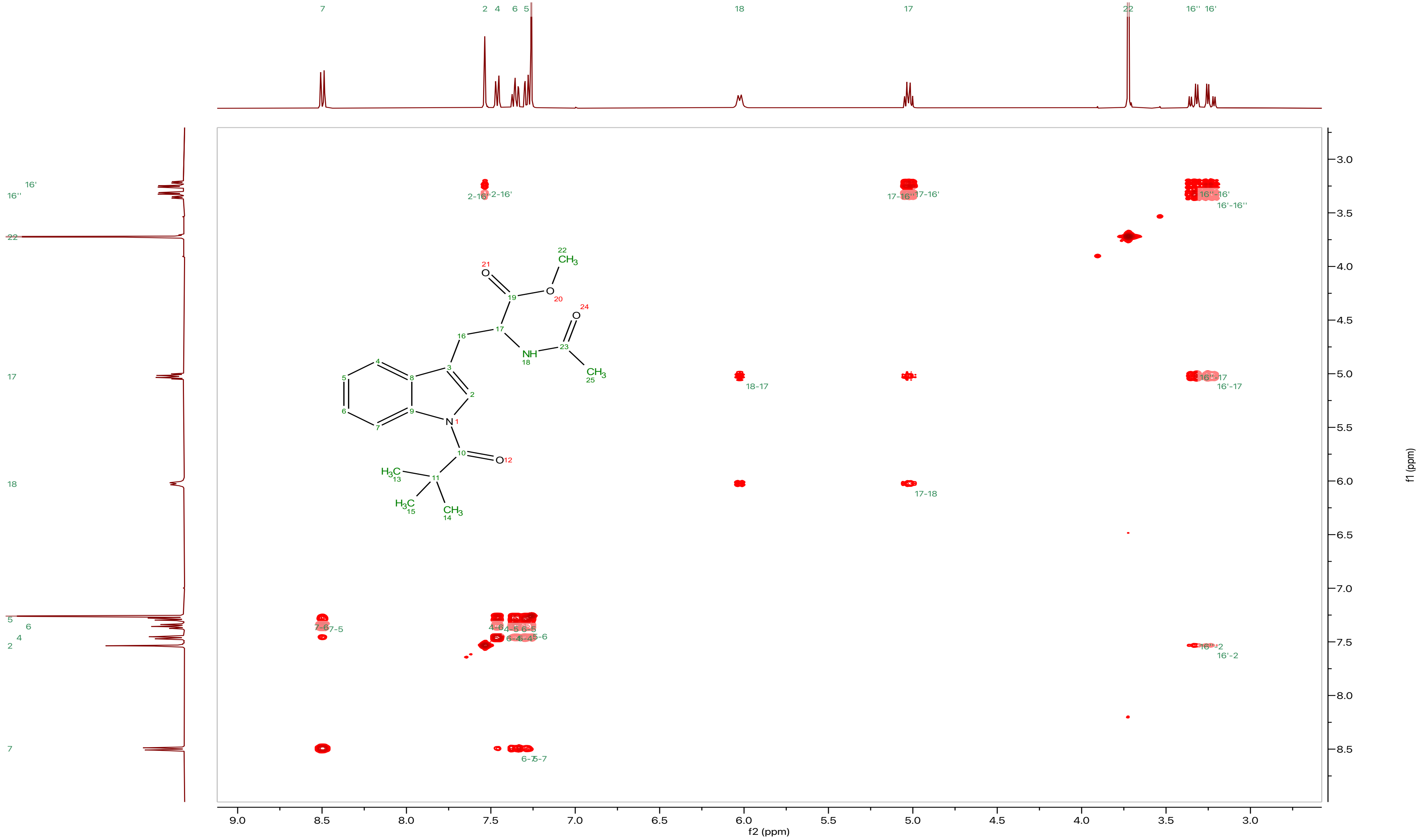

^{1}H-^{1}H COSY (400 MHz, $CDCl_3$) of 1t

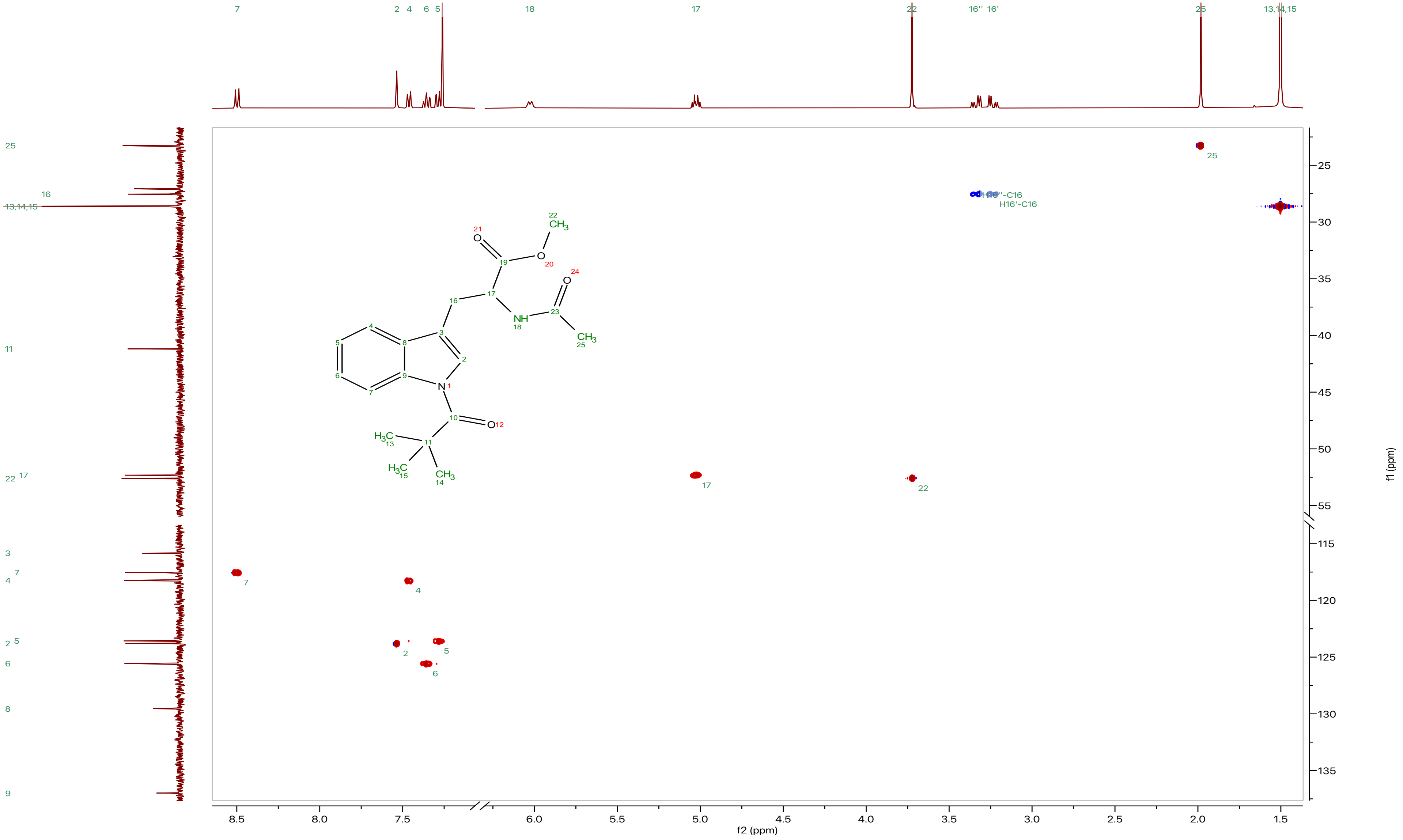

^{1}H-$^{13}C\{^{1}H\}$ HSQC NMR (400/101 MHz, $CDCl_3$) of 1t

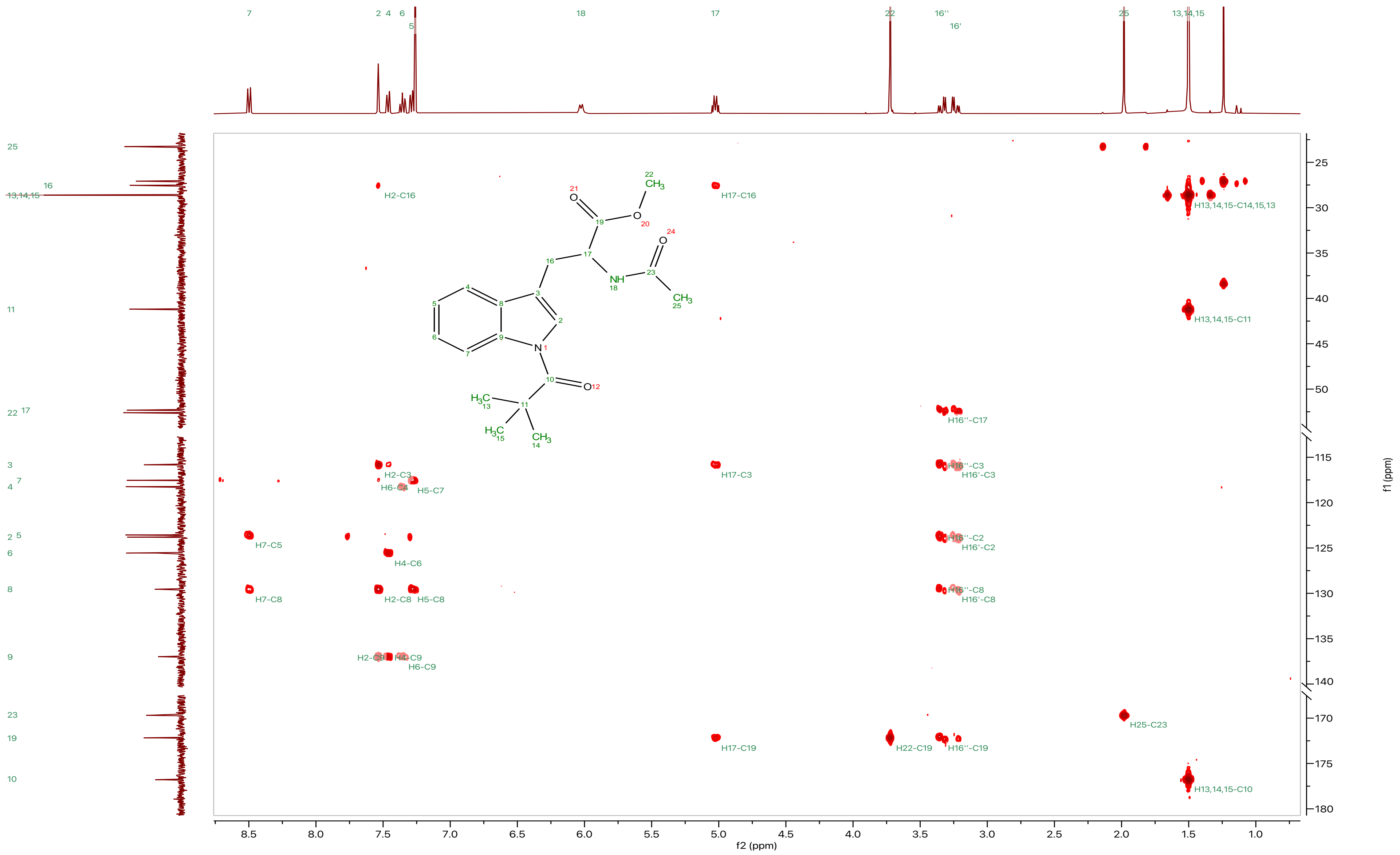

^{1}H-^{13}C{^{1}H} HMBC NMR (400/101 MHz, $CDCl_3$) of 1t

2t

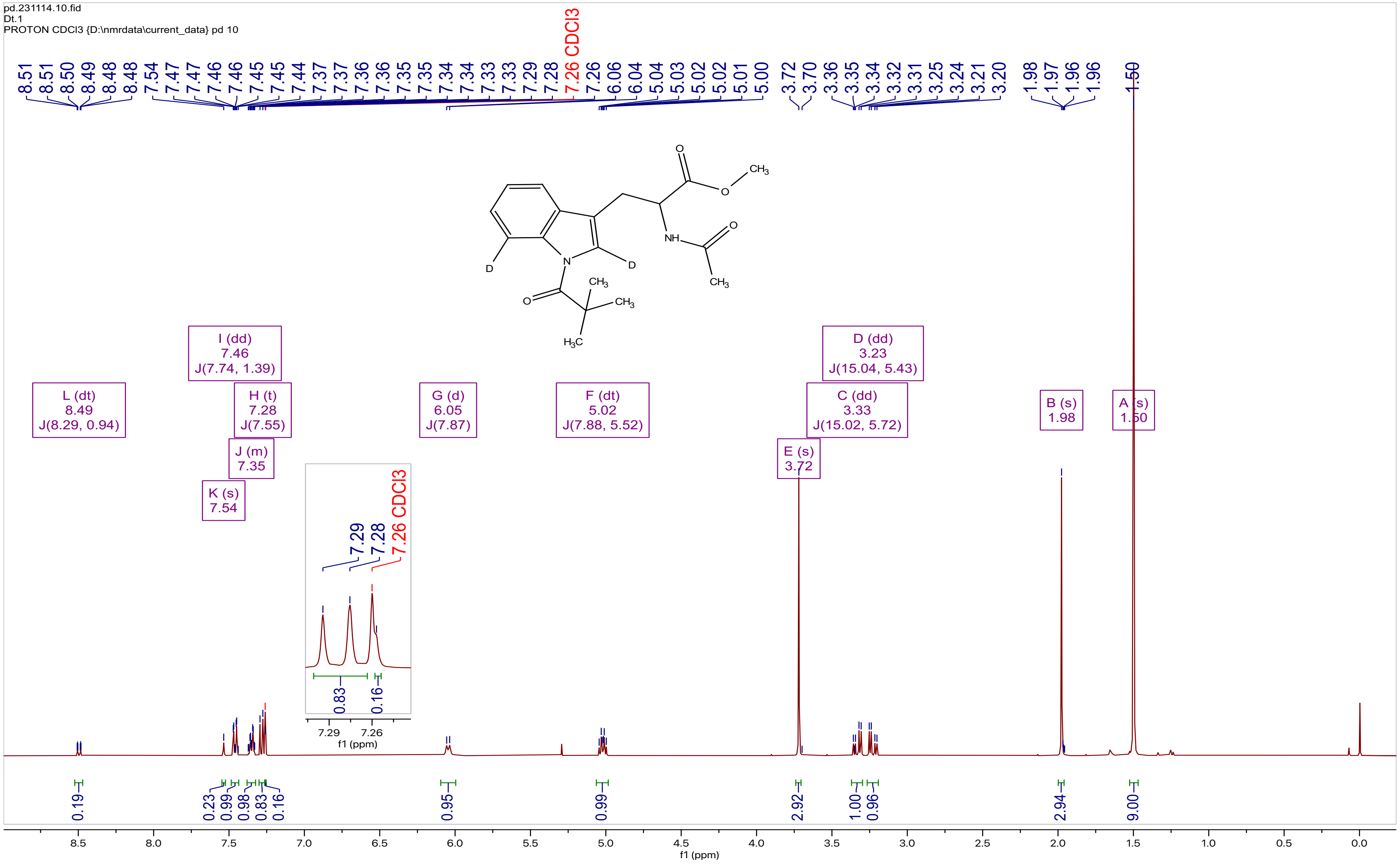

^{1}H NMR (400 MHz, $CDCl_3$) of 2t

pd.231114 8.11.fid
Dt.1
C13CPD CDCl3 {D:\nmrdata\current_data} pd 10

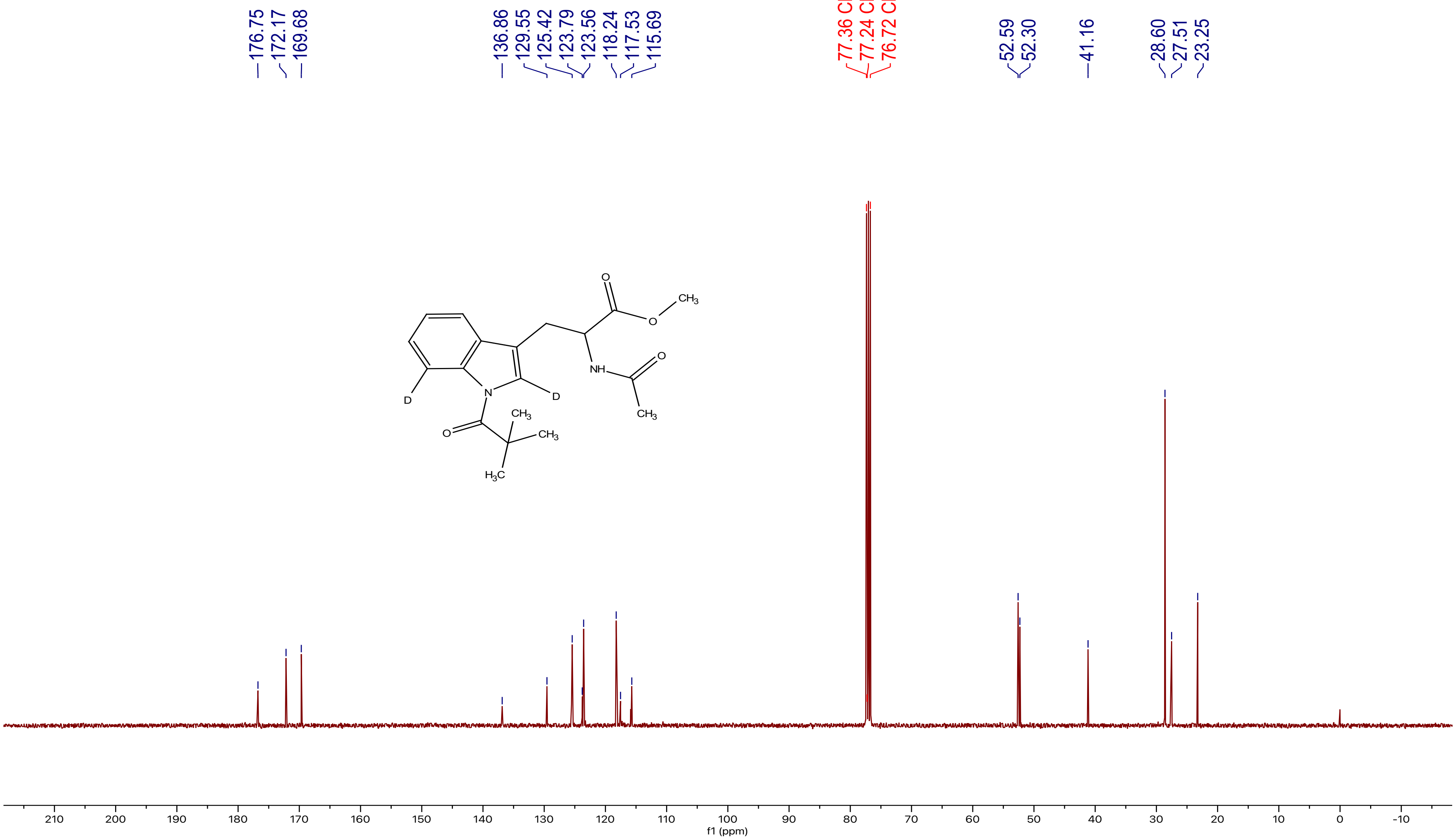

$^{13}C\{^{1}H\}$ NMR (101 MHz, $CDCl_3$) of 2t

1u

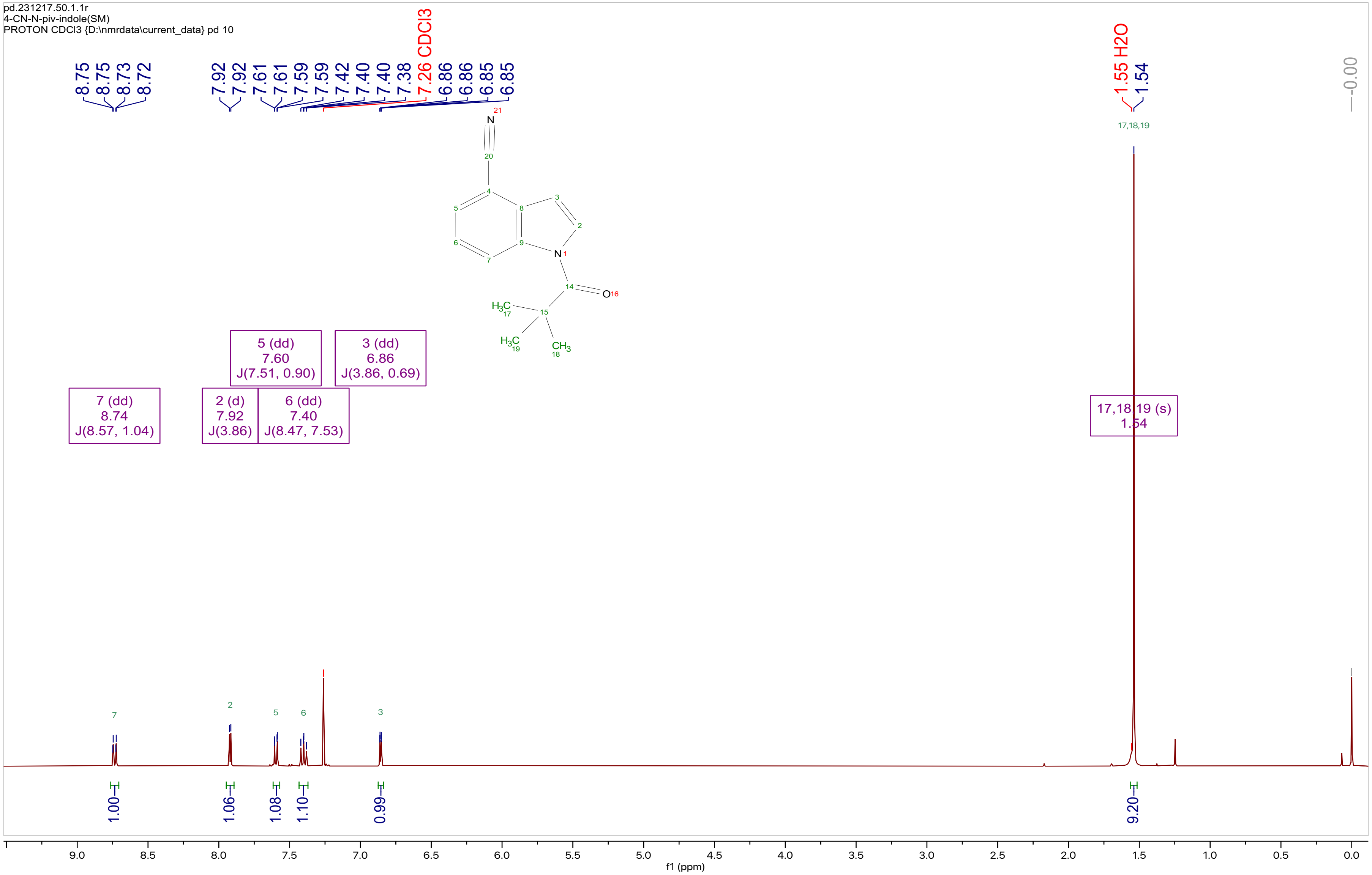

^1H NMR (400 MHz, $CDCl_3$) of 1u

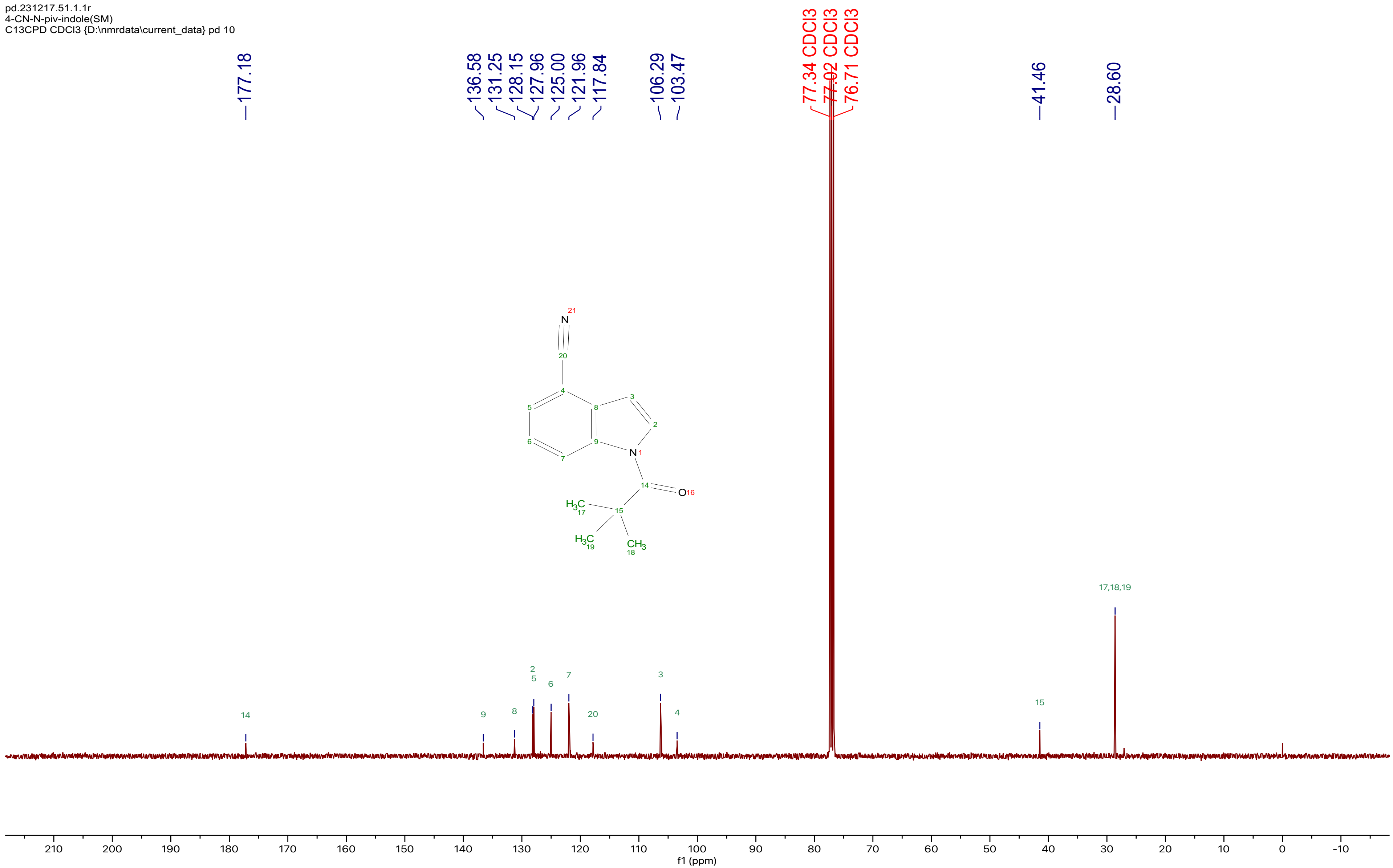

$^{13}C\{^{1}H\}$ NMR (101 MHz, $CDCl_3$) of 1u

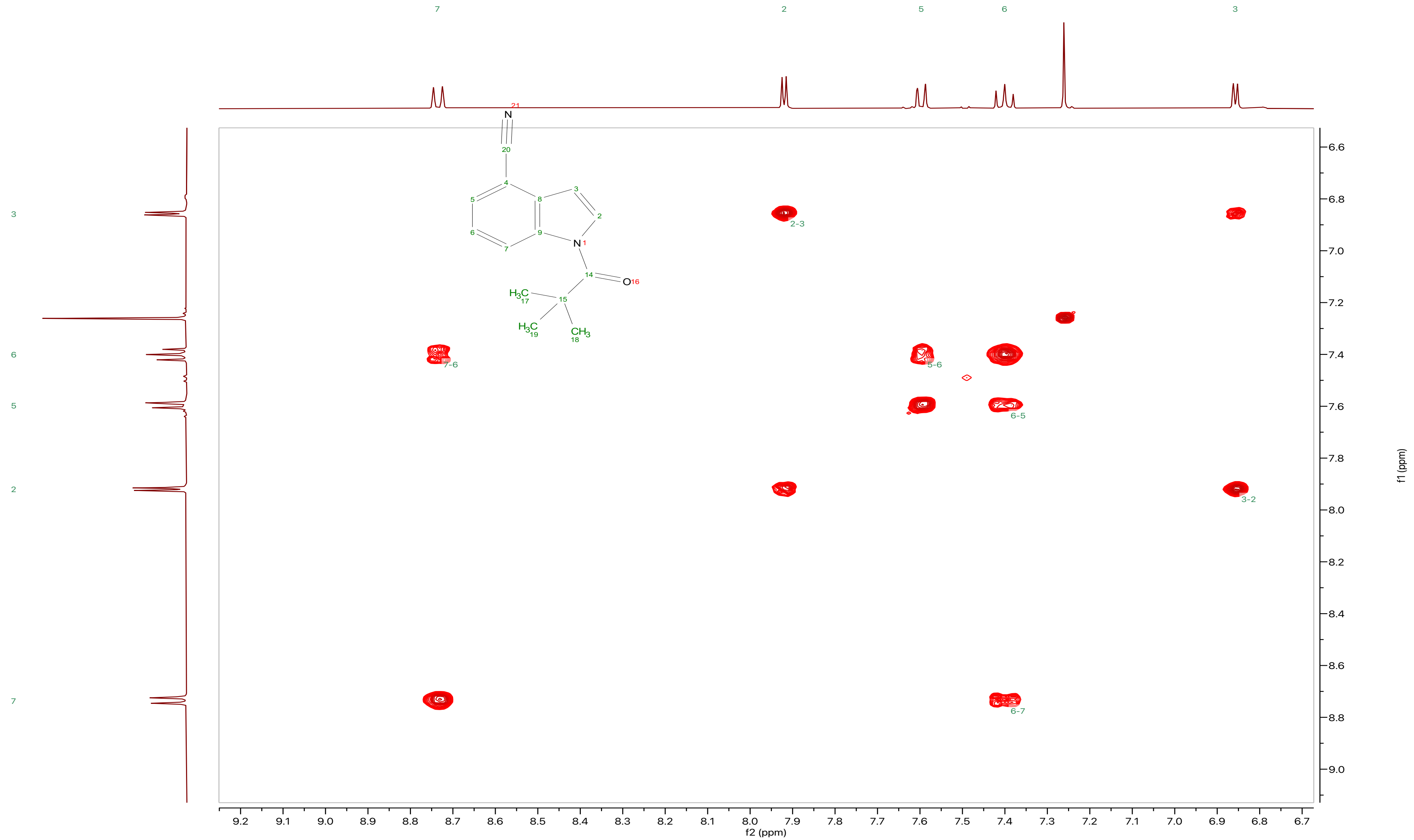

^1H-^1H COSY (400 MHz, $CDCl_3$) of 1u

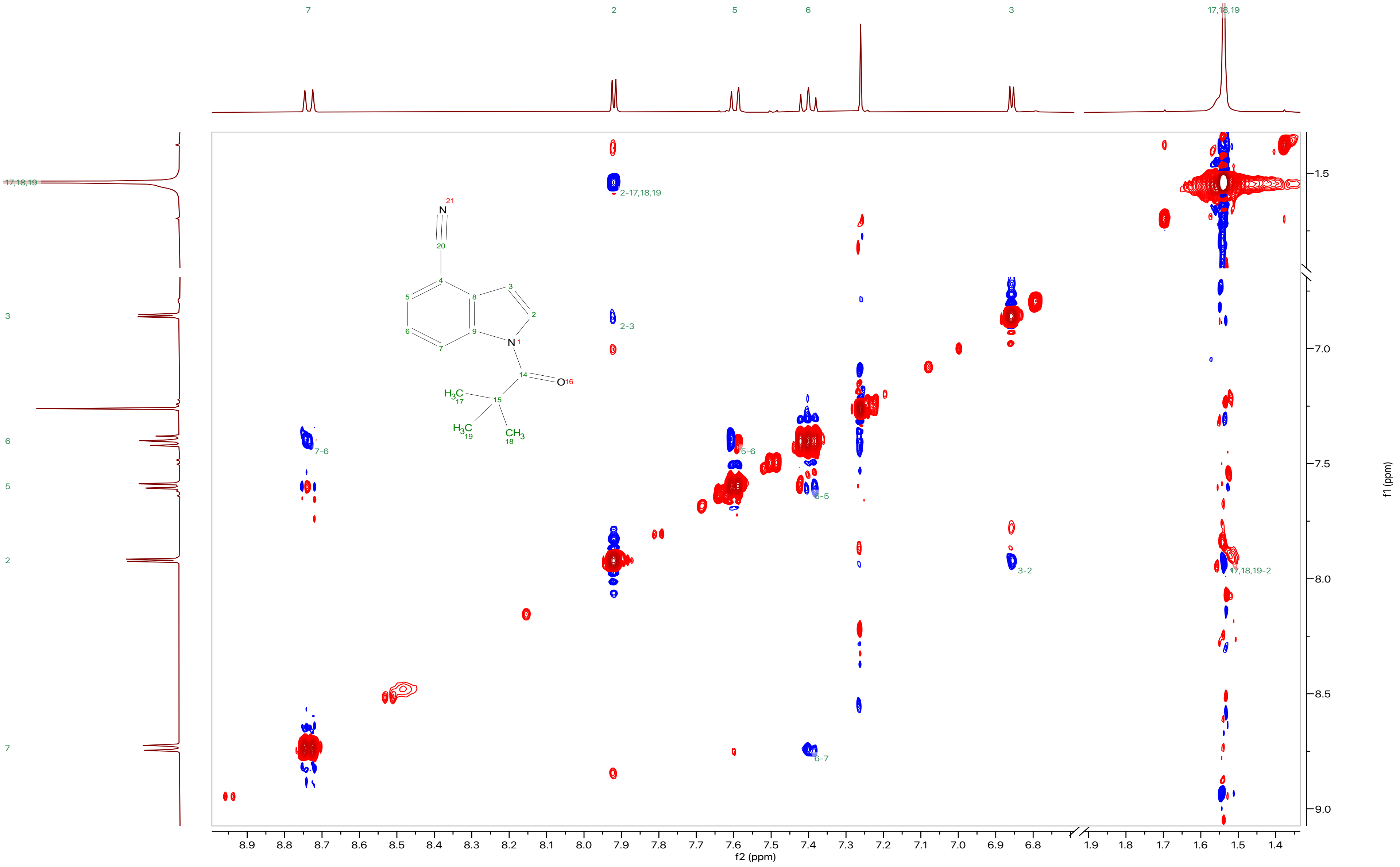

^{1}H-^{1}H NOESY (400 MHz, $CDCl_3$) of 1u

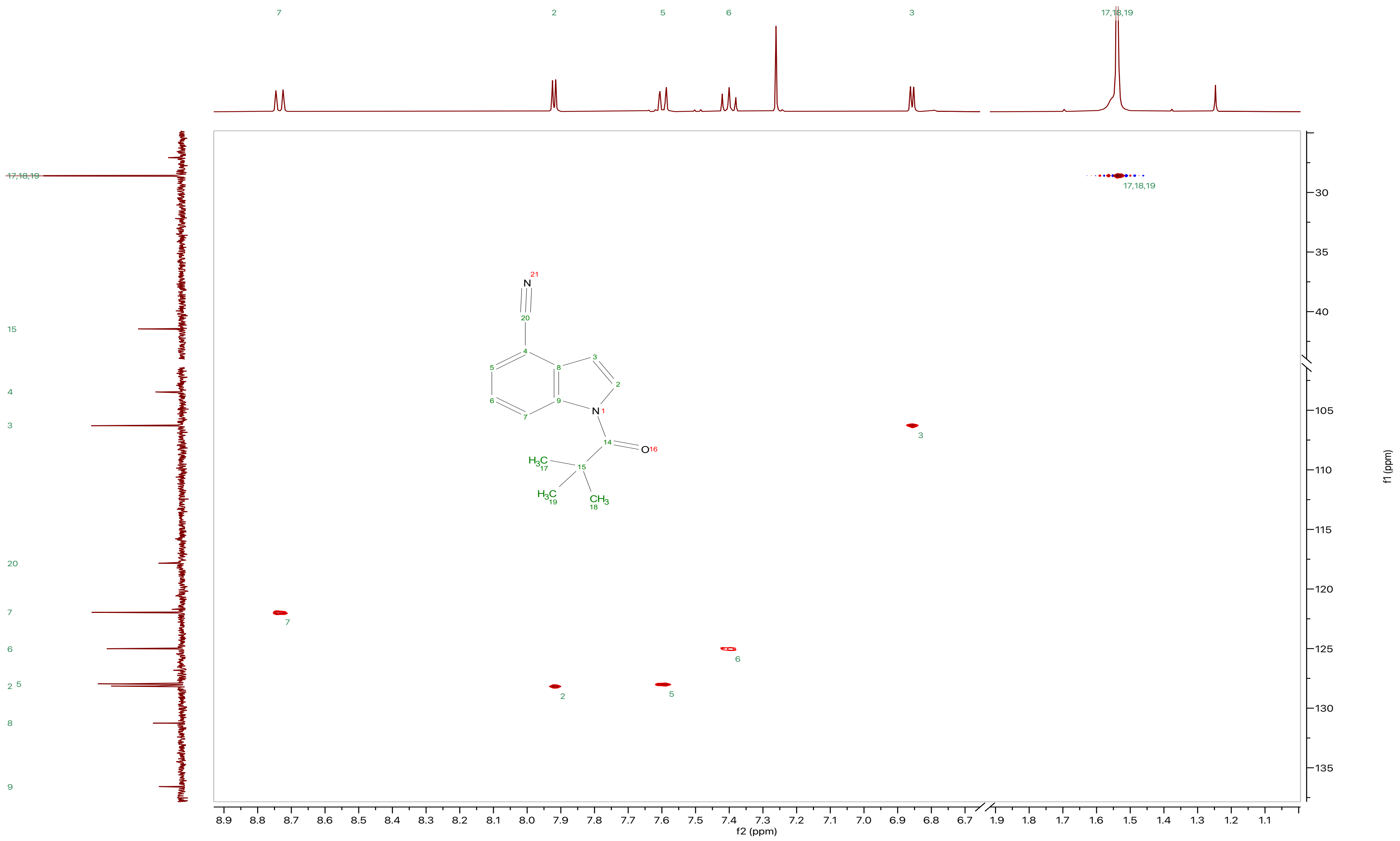

^{1}H-$^{13}C\{^{1}H\}$ HSQC NMR (400/101 MHz, $CDCl_3$) of 1u

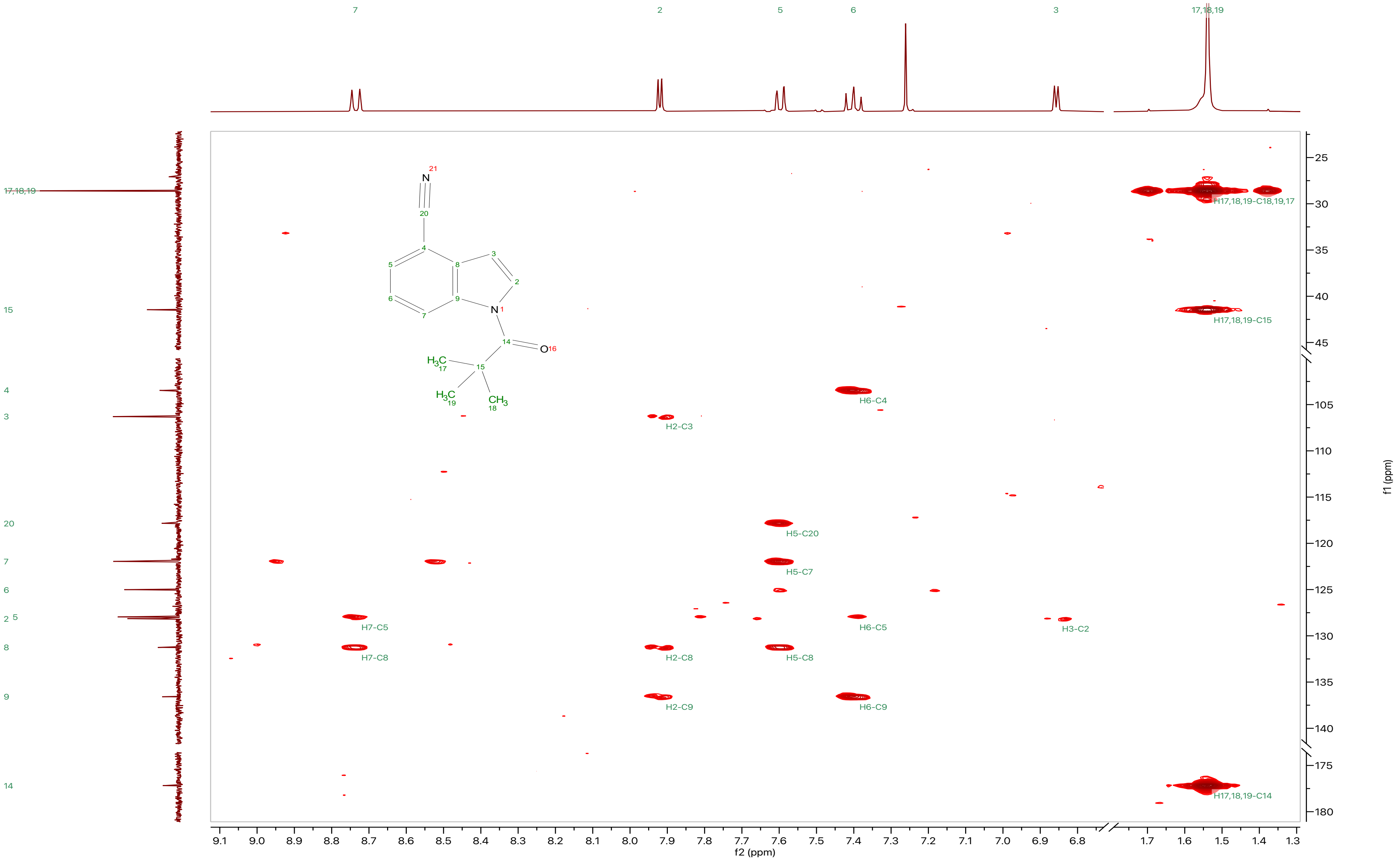

^{1}H-$^{13}C\{^{1}H\}$ HMBC NMR (400/101 MHz, $CDCl_3$) of 1u

2u

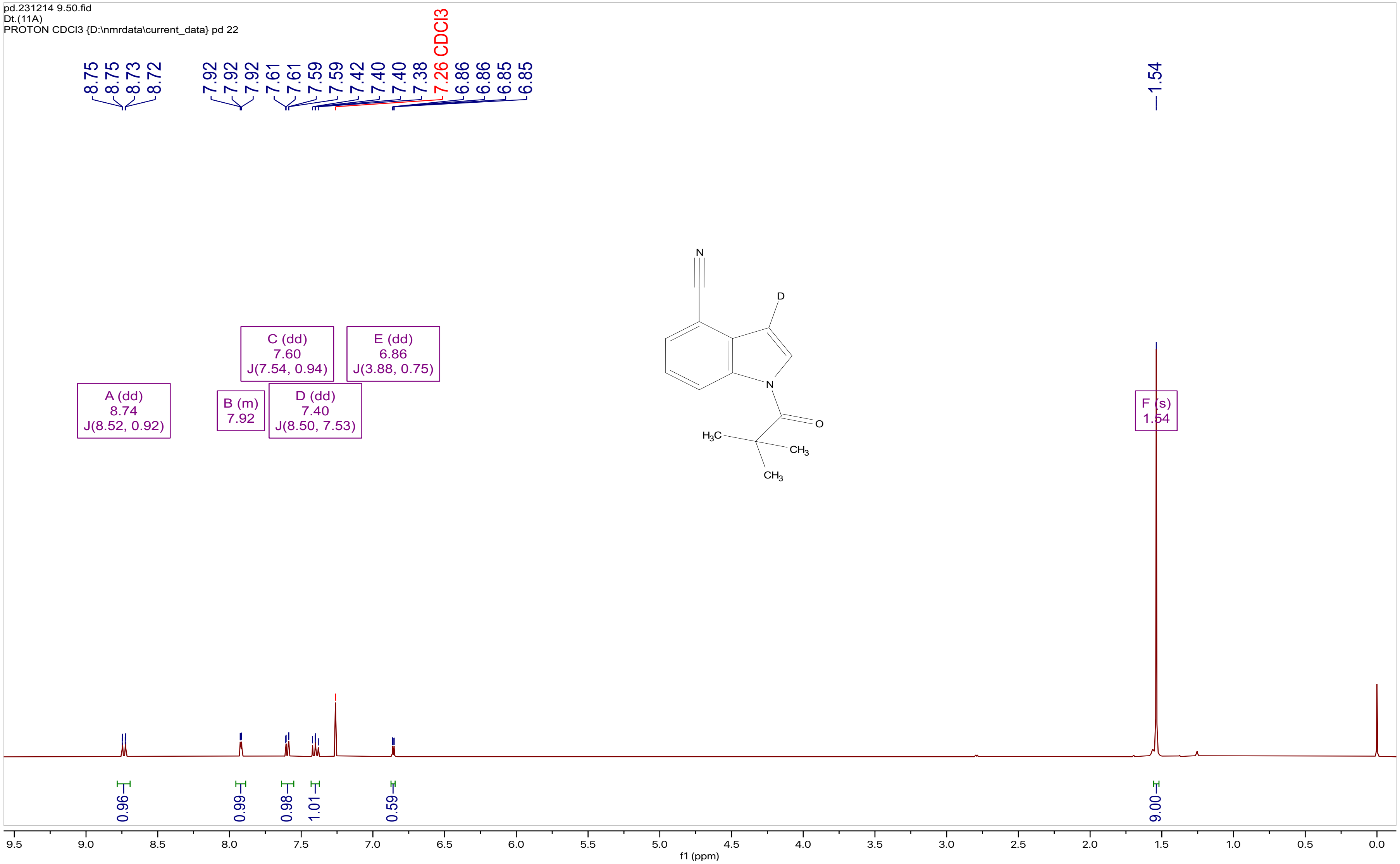

^{1}H NMR (400 MHz, $CDCl_3$) of 2u

pd.231214 10.51.fid
Dt.(11A)
C13CPD CDCl3 {D:\nmrdata\current_data} pd 22

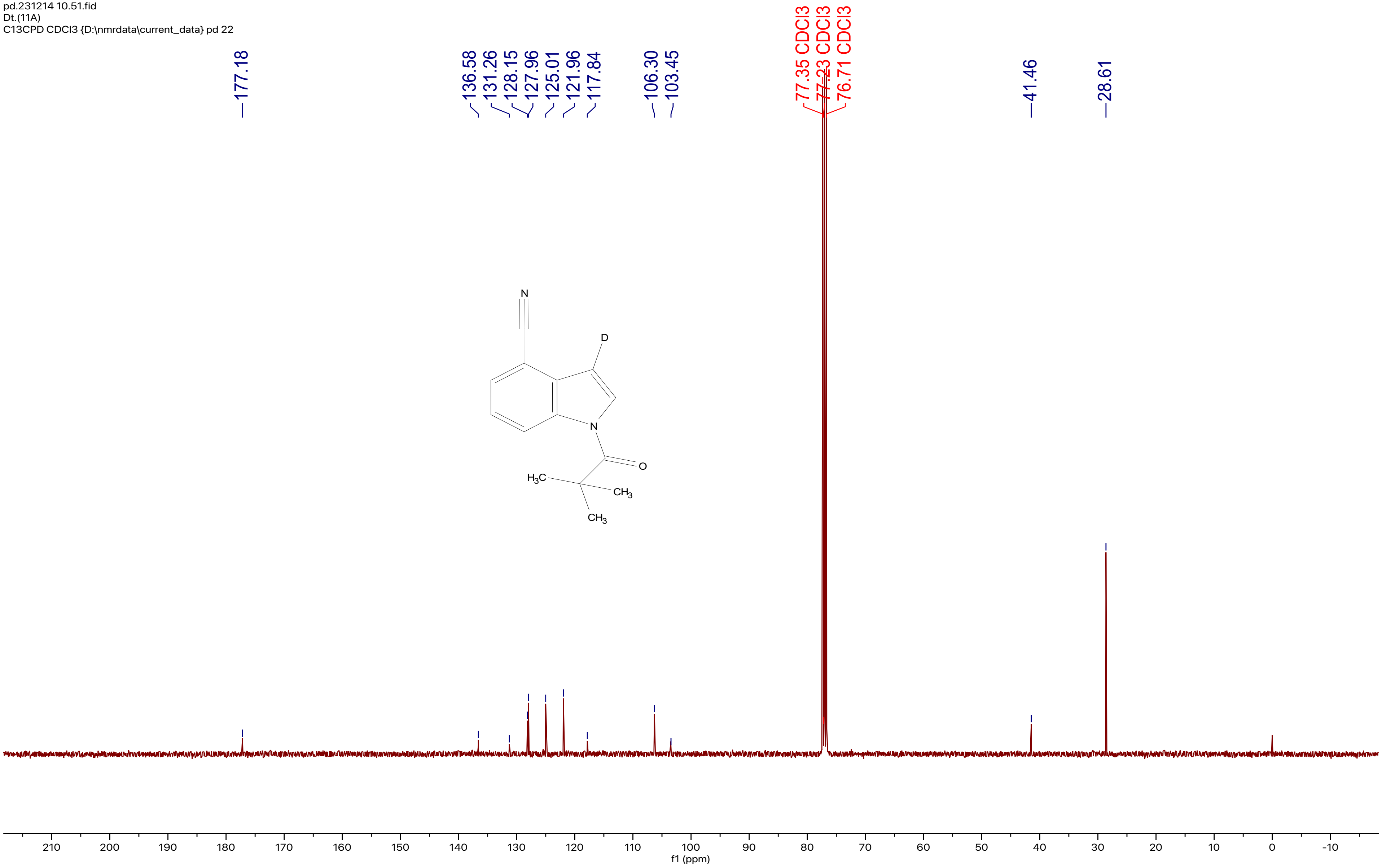

$^{13}C\{^1H\}$ NMR (101 MHz, $CDCl_3$) of 2u

1v

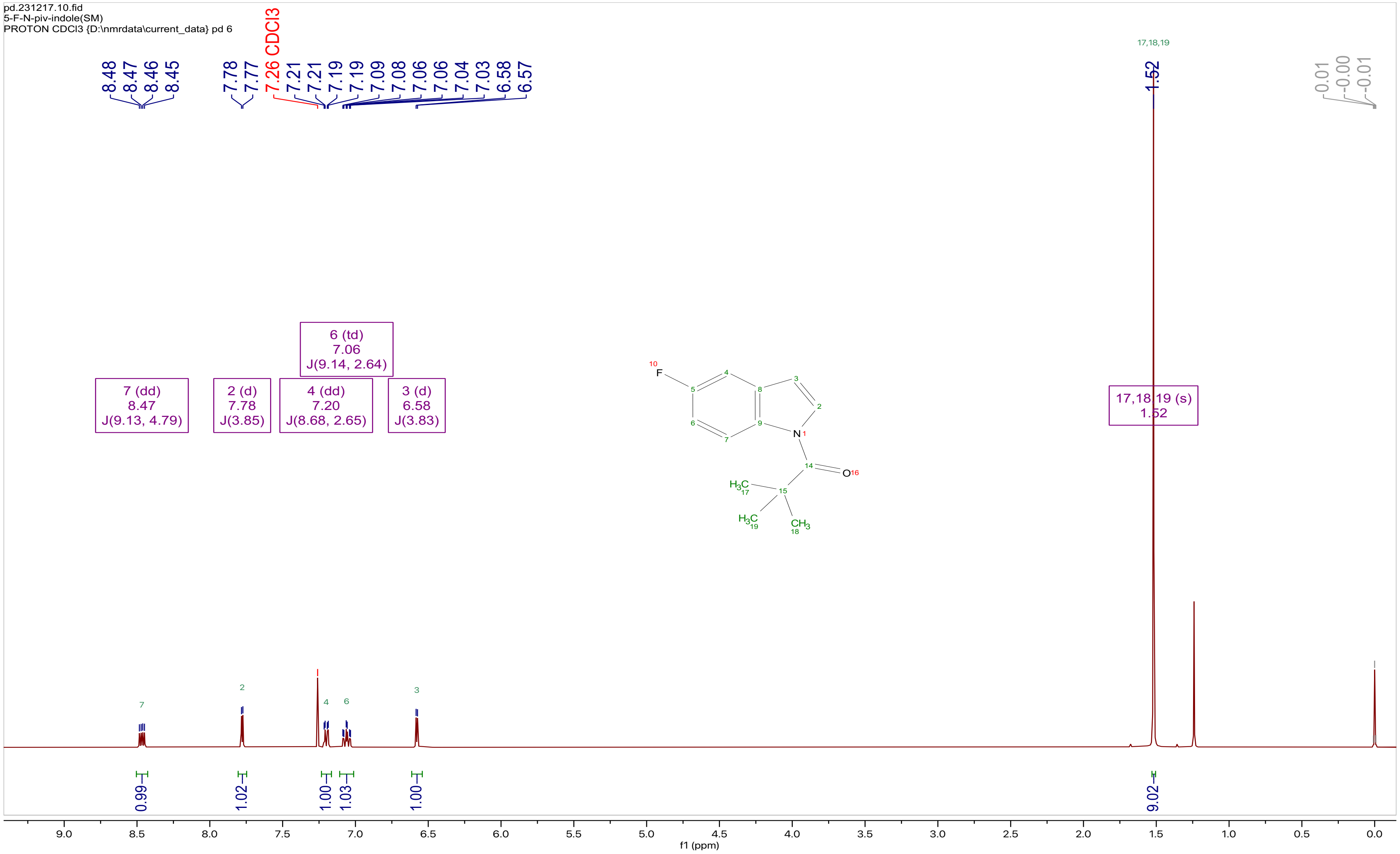

^{1}H NMR (400 MHz, $CDCl_3$) of 1v

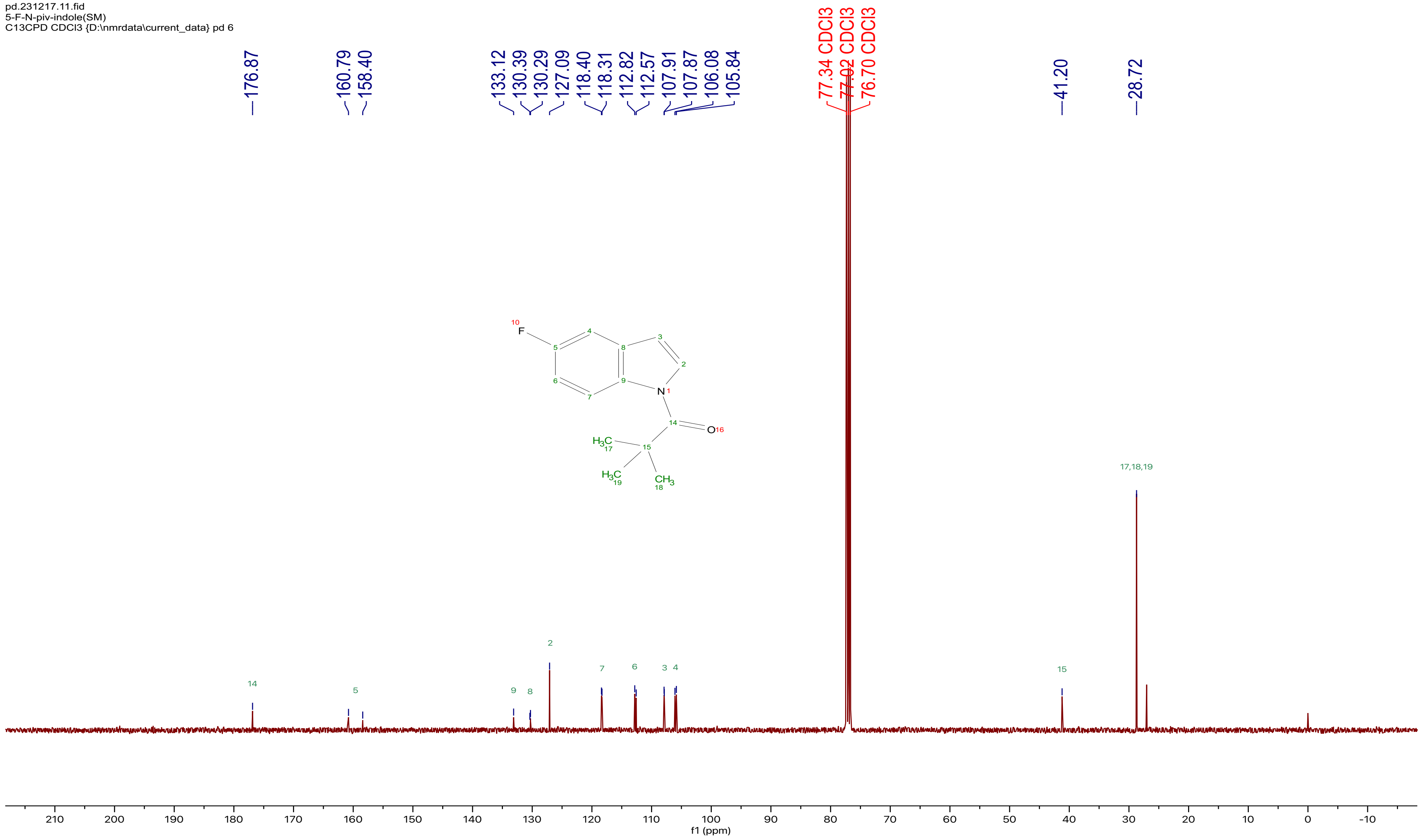

$^{13}C\{^1H\}$ NMR (101 MHz, $CDCl_3$) of 1v

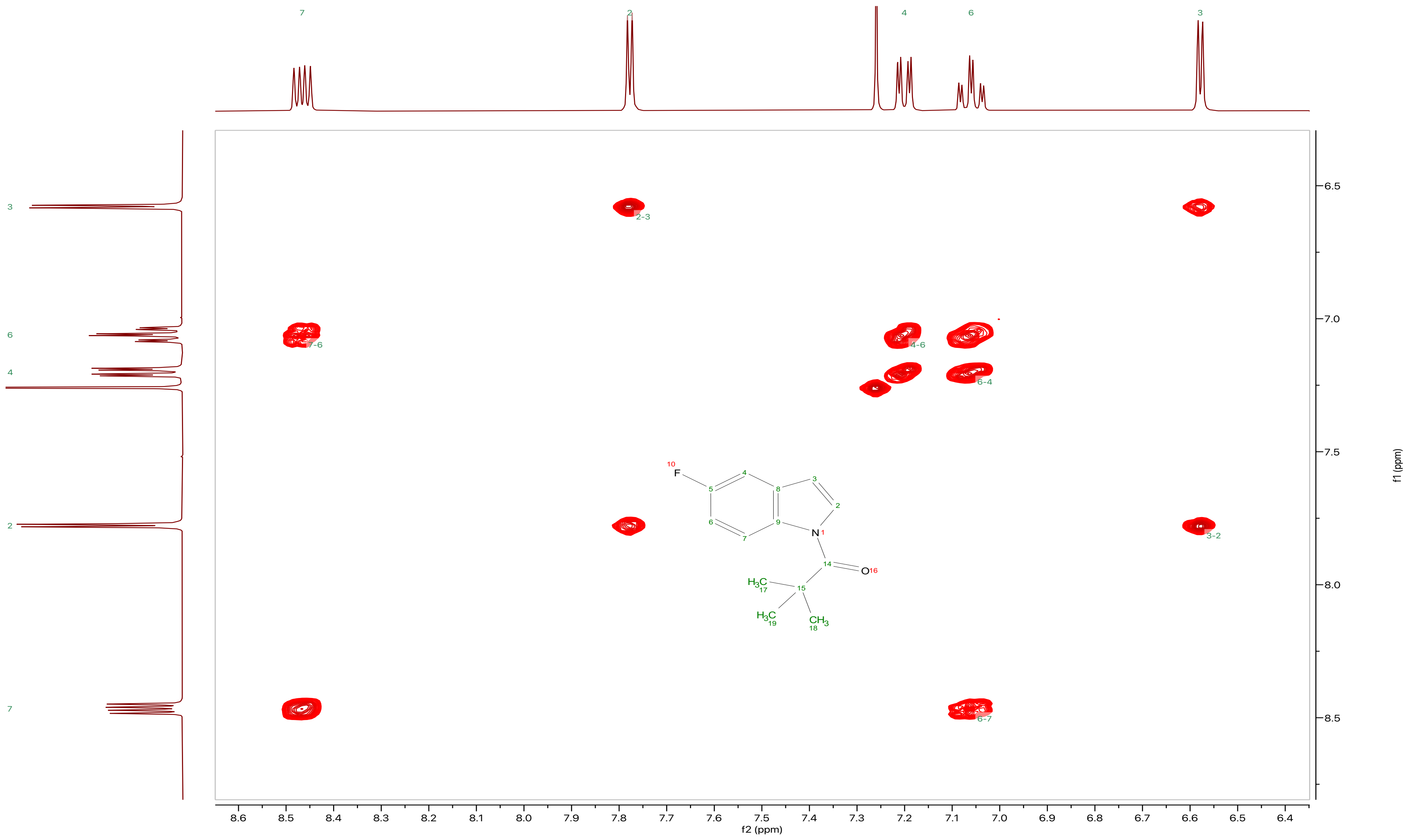

^{1}H-^{1}H COSY (400 MHz, $CDCl_3$) of 1v

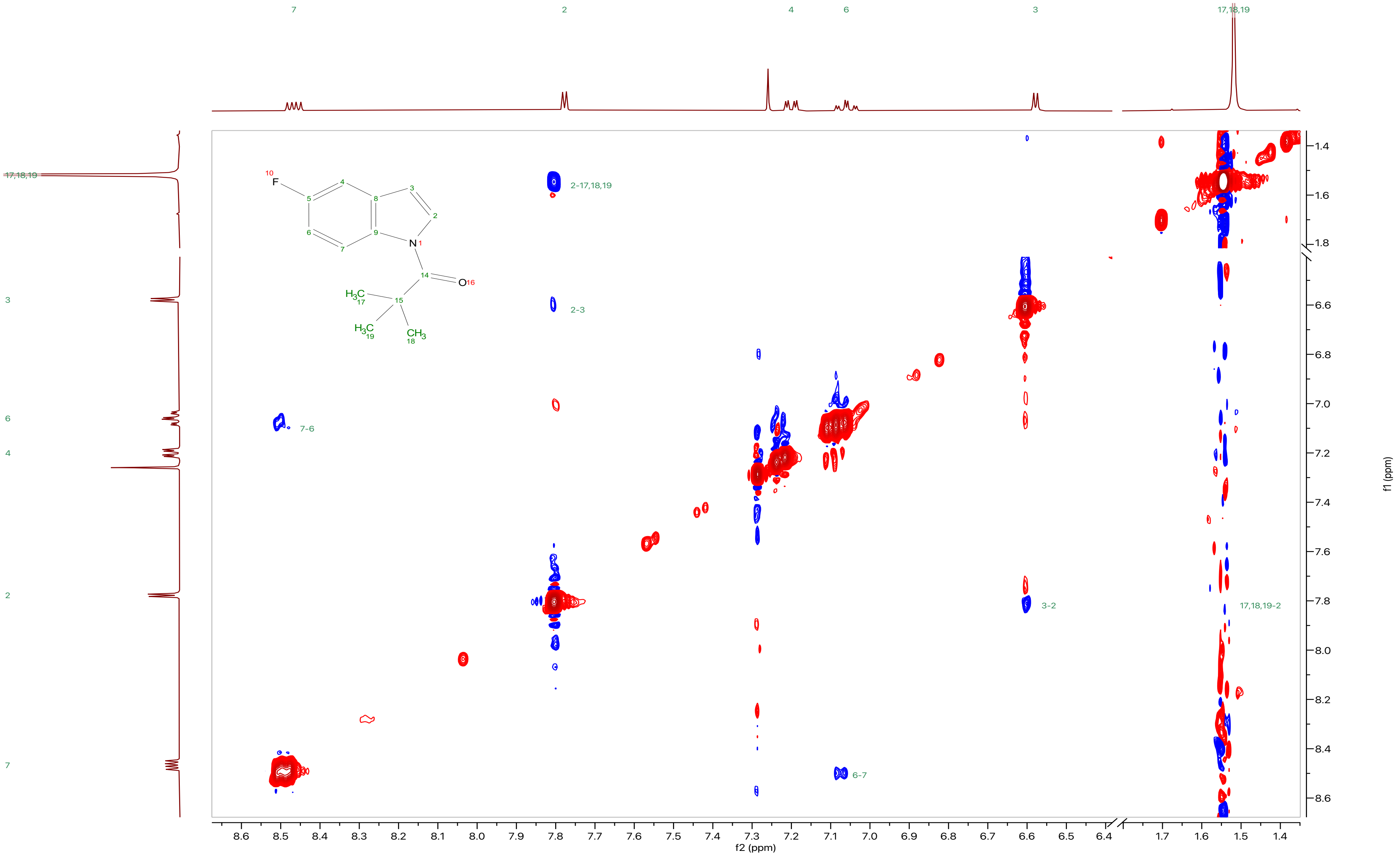

^{1}H-^{1}H NOESY (400 MHz, $CDCl_3$) of 1v

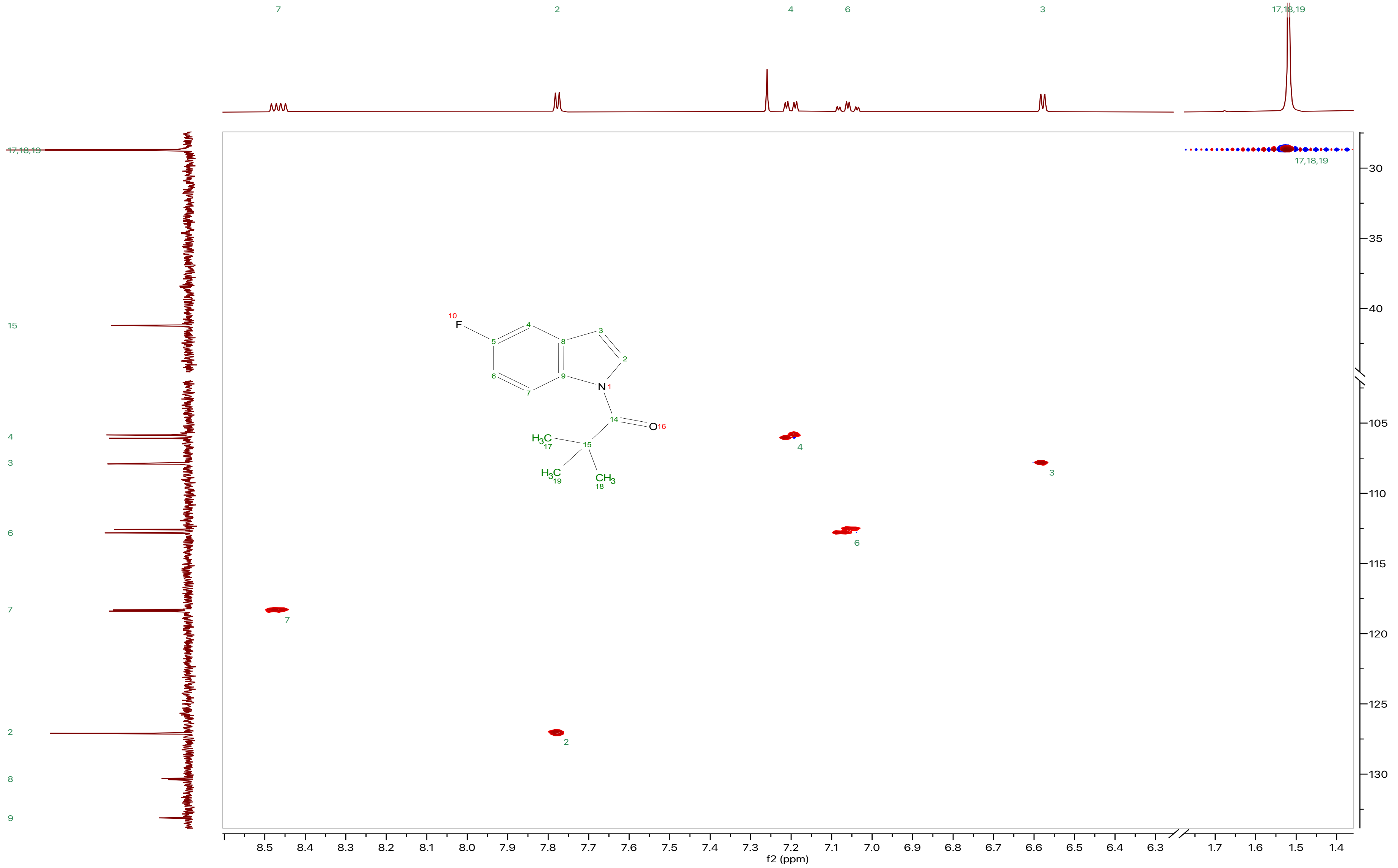

^{1}H-^{13}C{^{1}H} HSQC NMR (400/101 MHz, $CDCl_3$) of 1v

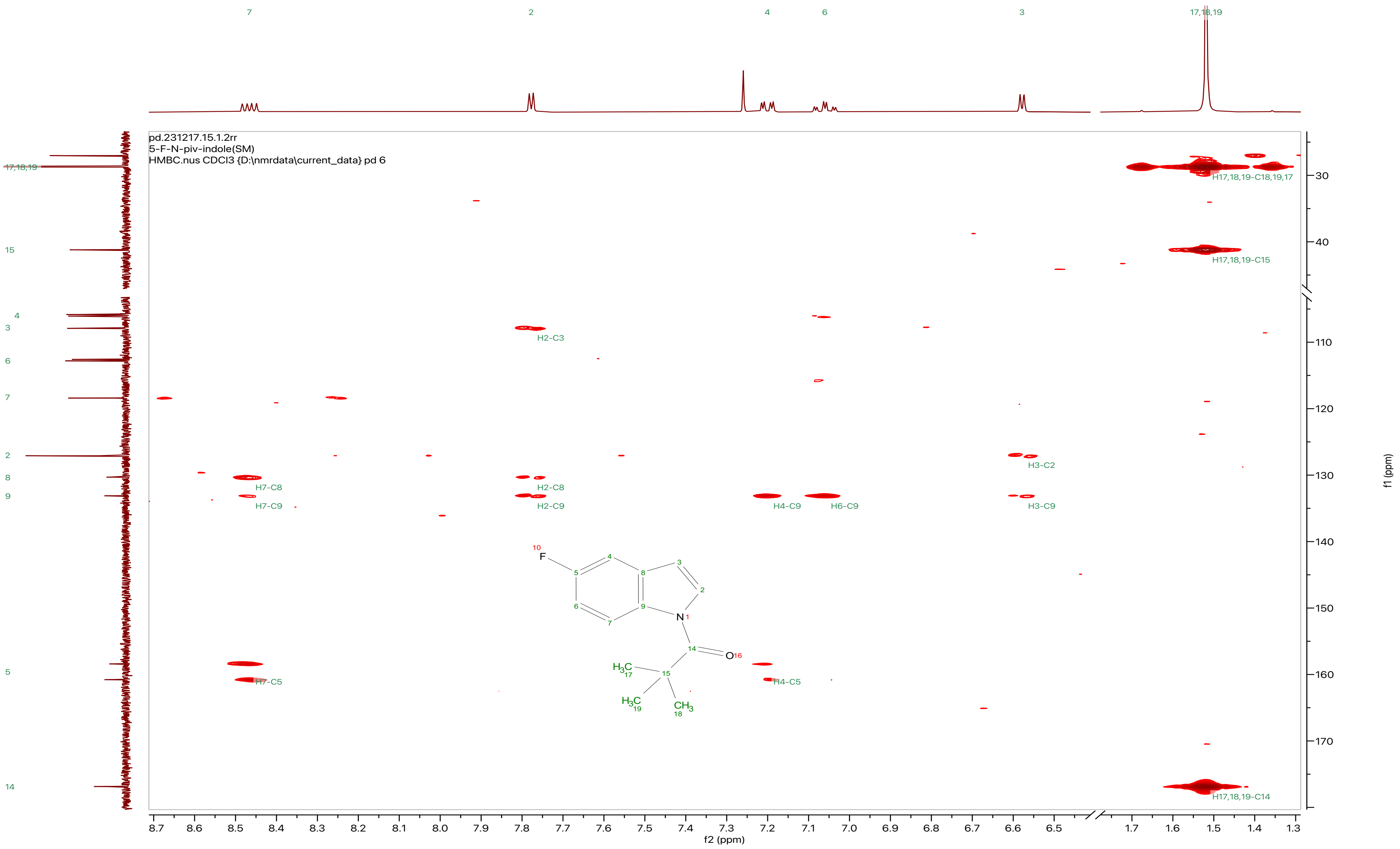

^{1}H-$^{13}C\{^{1}H\}$ HMBC NMR (400/101 MHz, $CDCl_3$) of 1v

2v

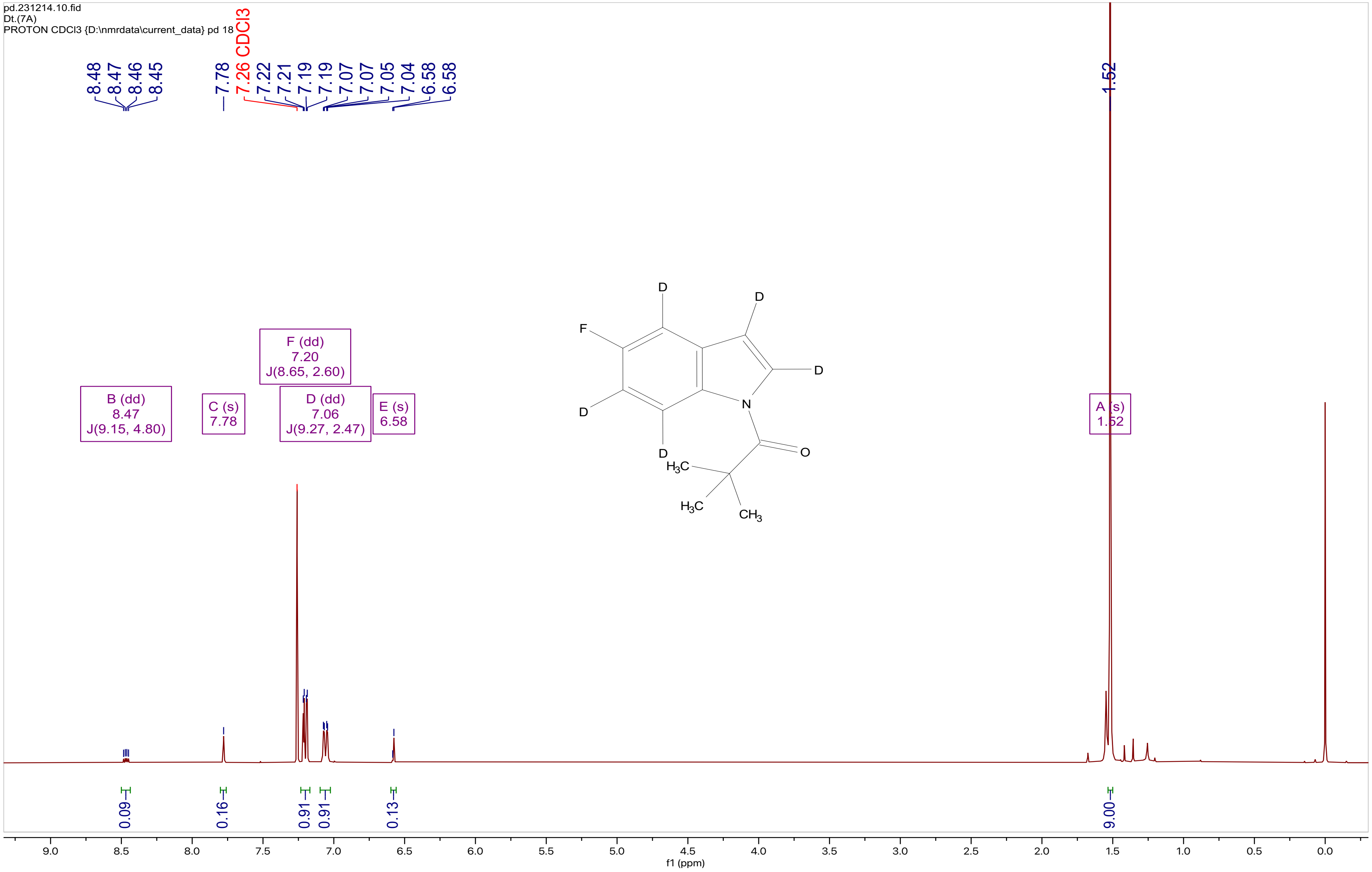

^1H NMR (400 MHz, $CDCl_3$) of 2v

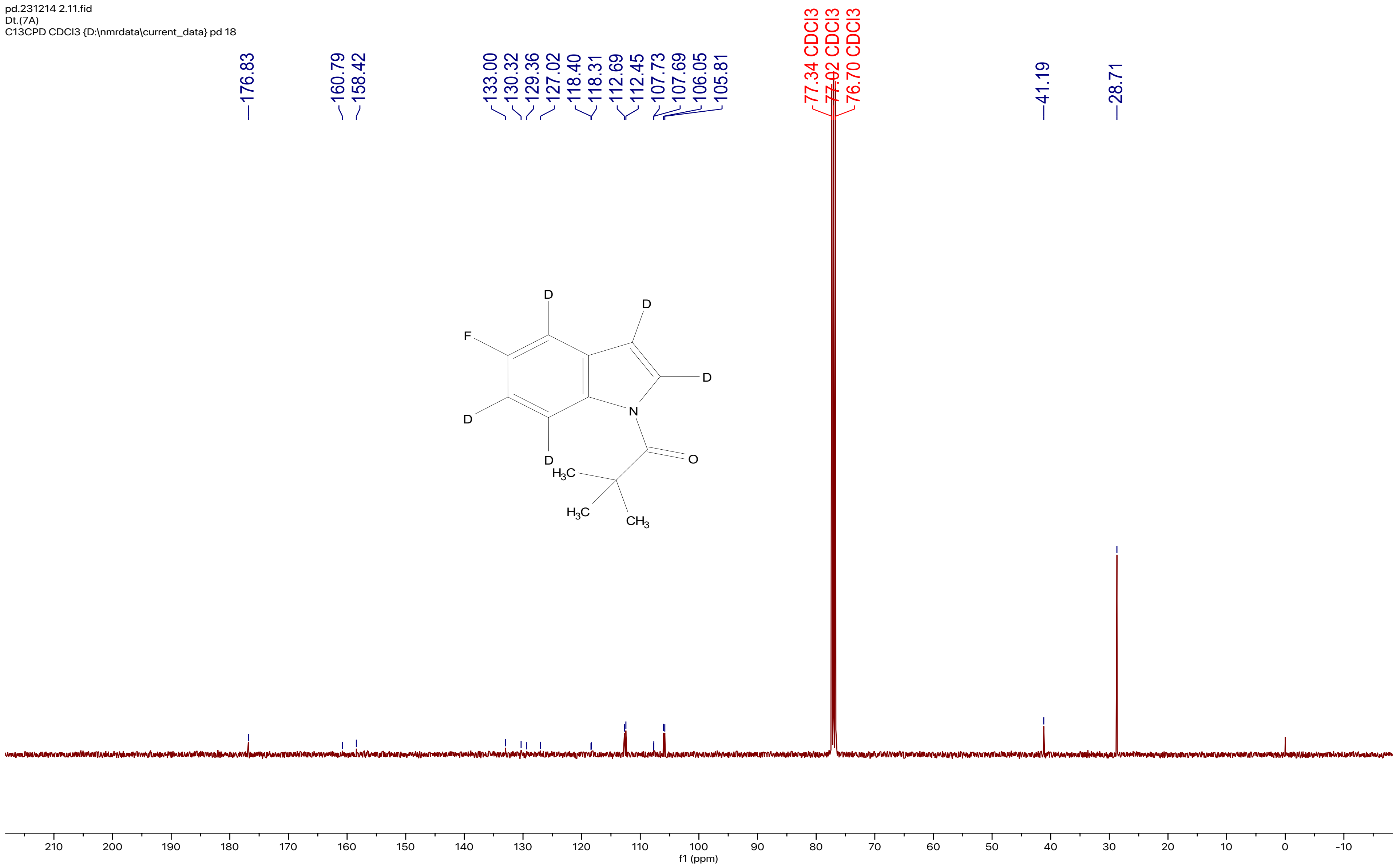

$^{13}C\{^{1}H\}$ NMR (101 MHz, $CDCl_3$) of 2v

1w

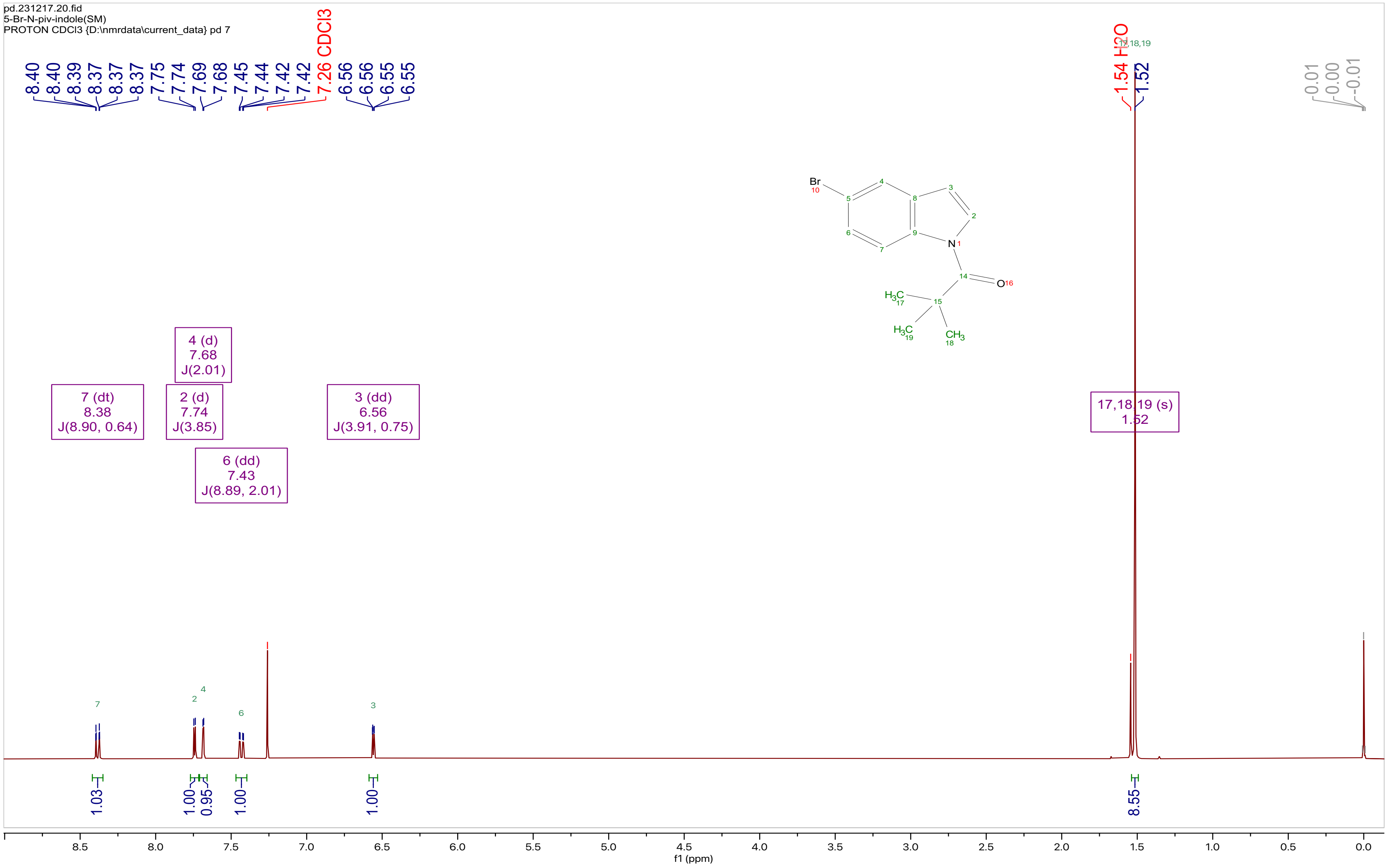

^{1}H NMR (400 MHz, $CDCl_3$) of 1w

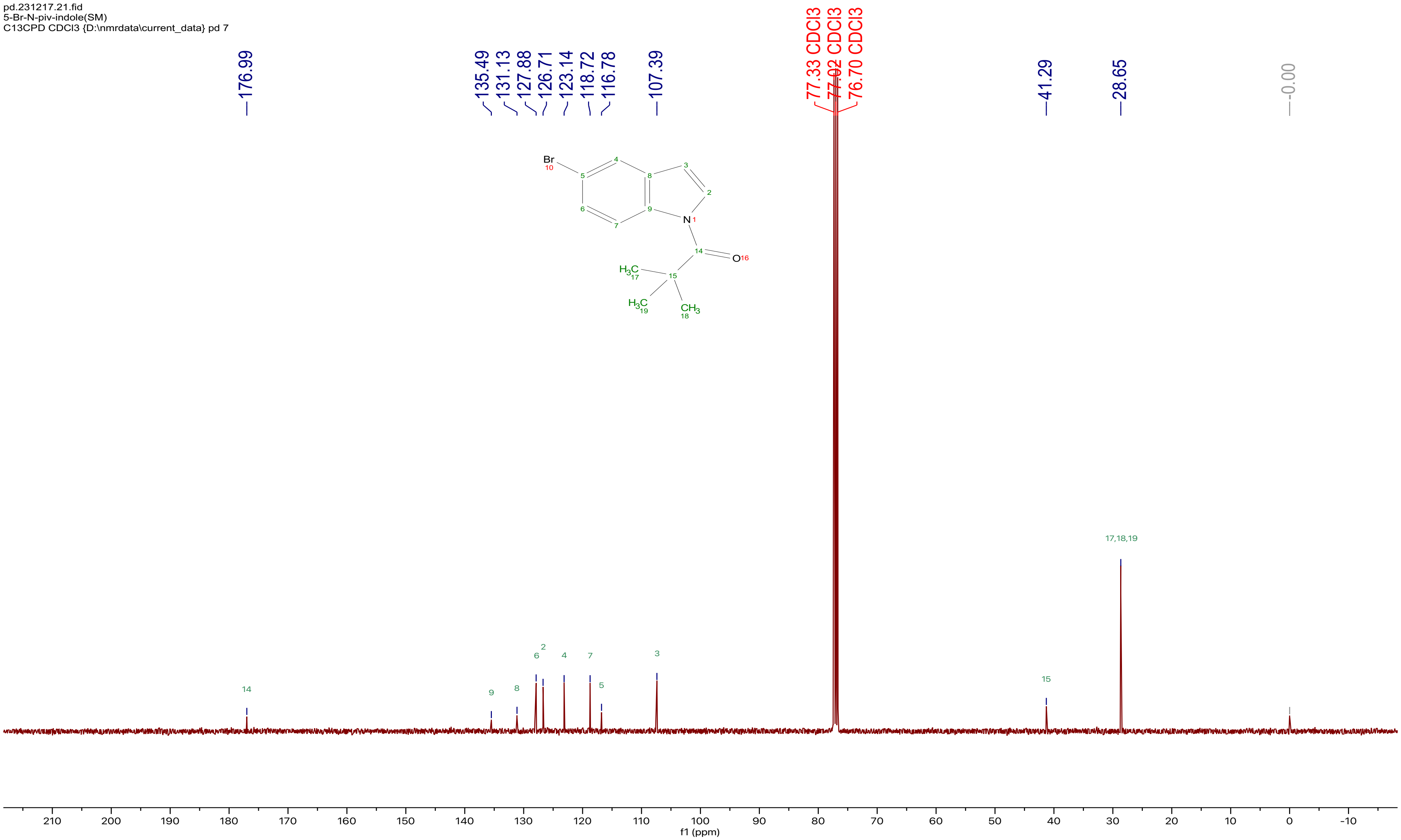

$^{13}C\{^1H\}$ NMR (101 MHz, $CDCl_3$) of 1w

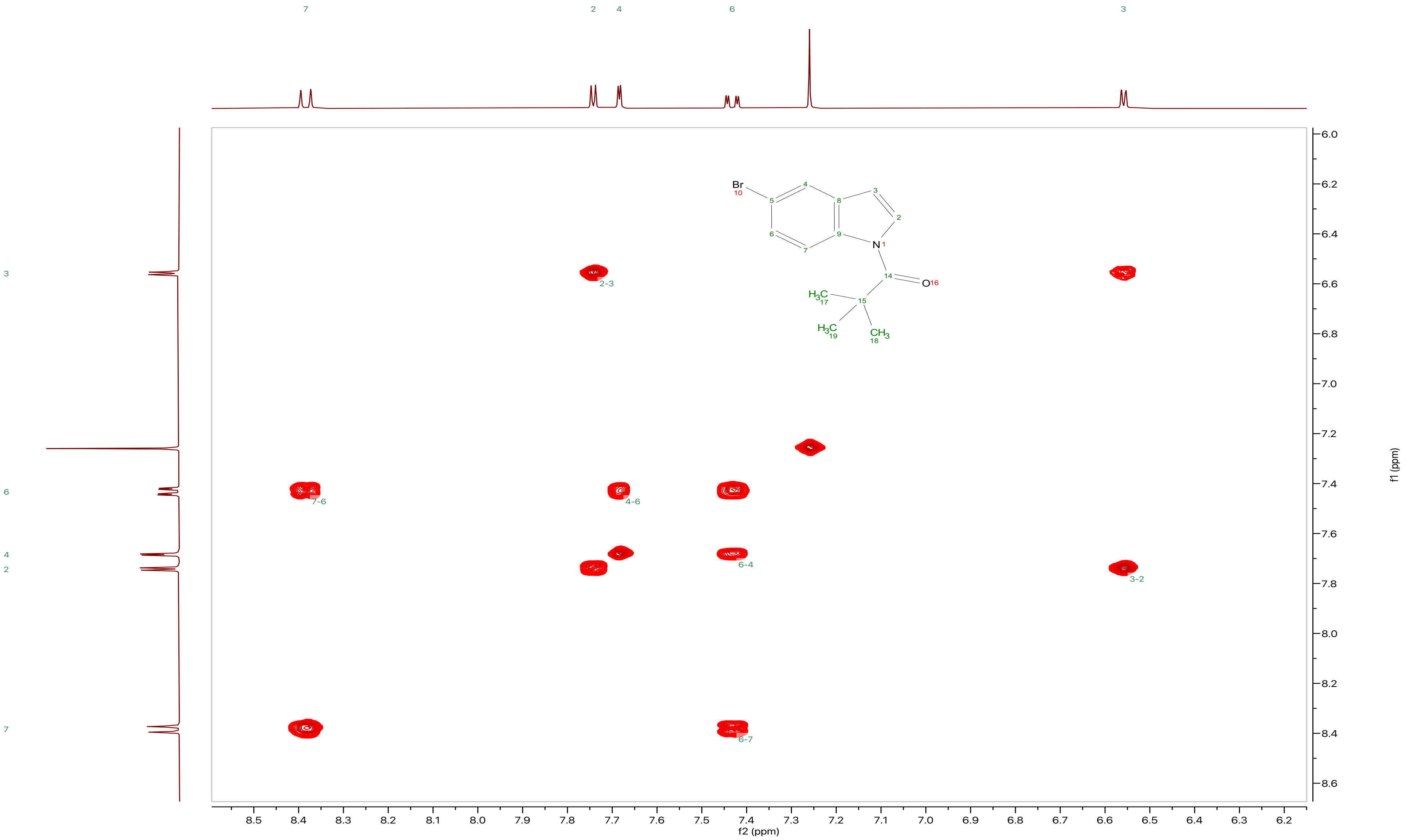

^1H-^1H COSY (400 MHz, $CDCl_3$) of 1w

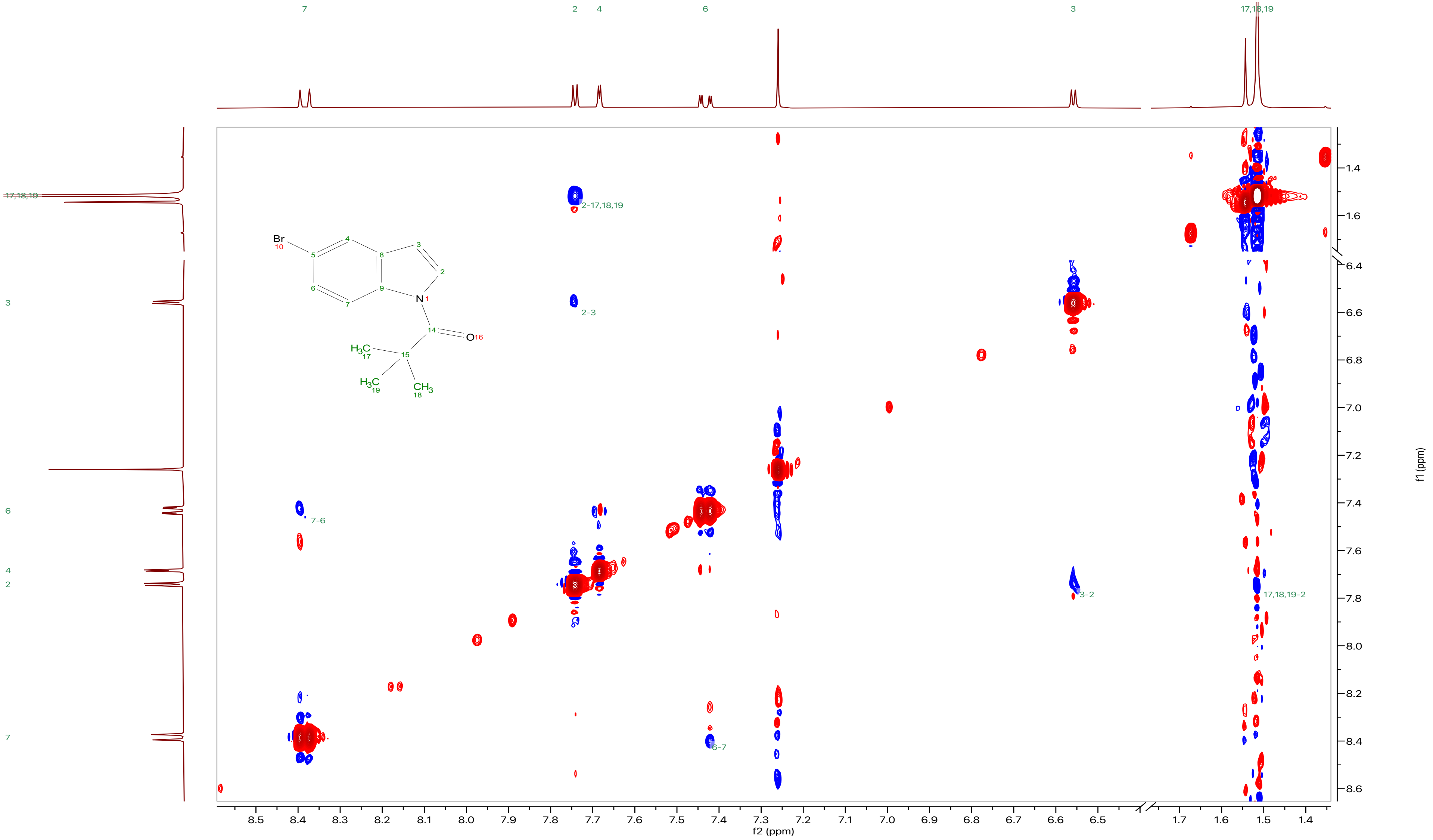

^{1}H-^{1}H NOESY (400 MHz, $CDCl_3$) of 1w

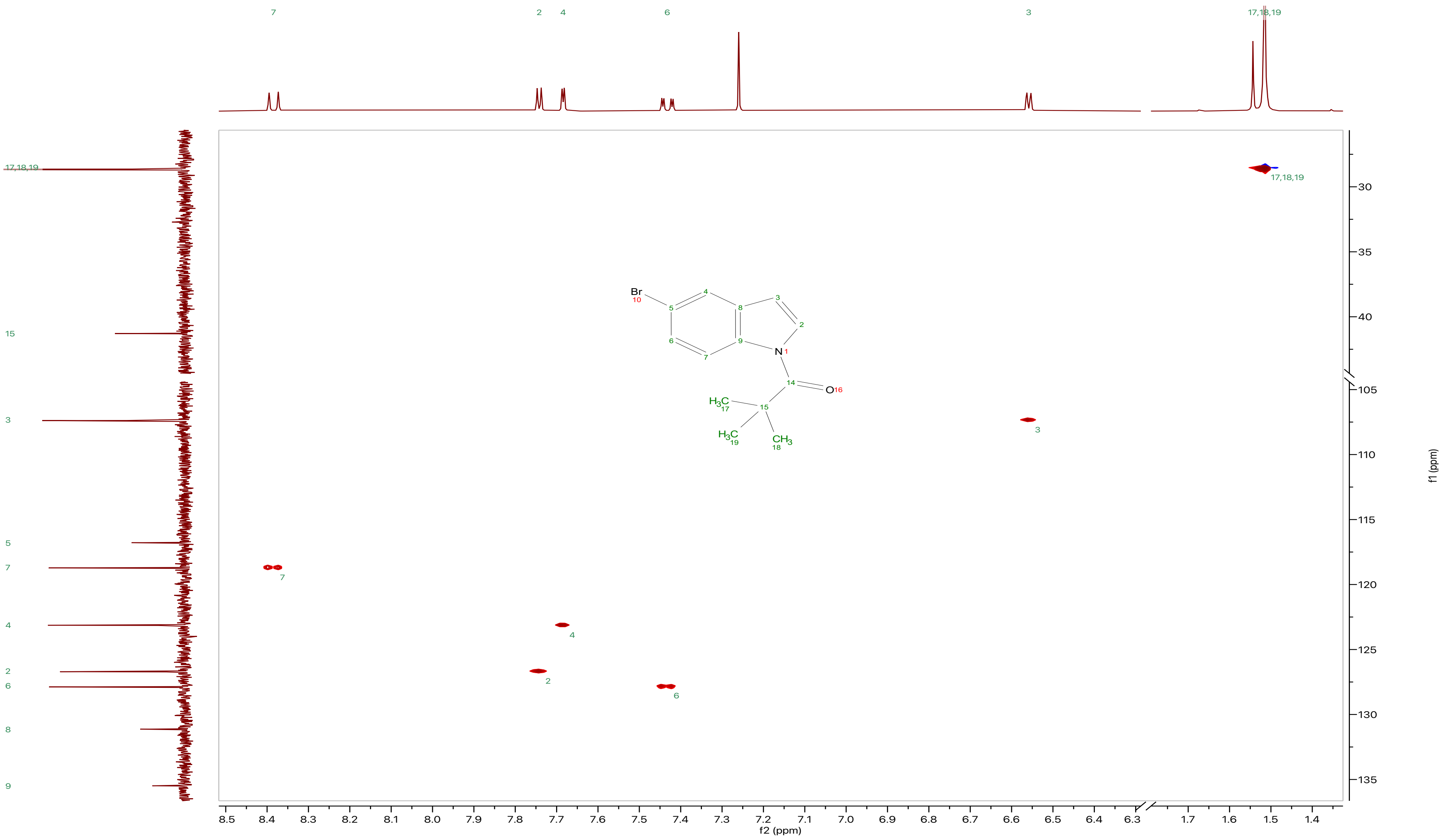

^1H-$^{13}C\{^1H\}$ HSQC NMR (400/101 MHz, $CDCl_3$) of 1w

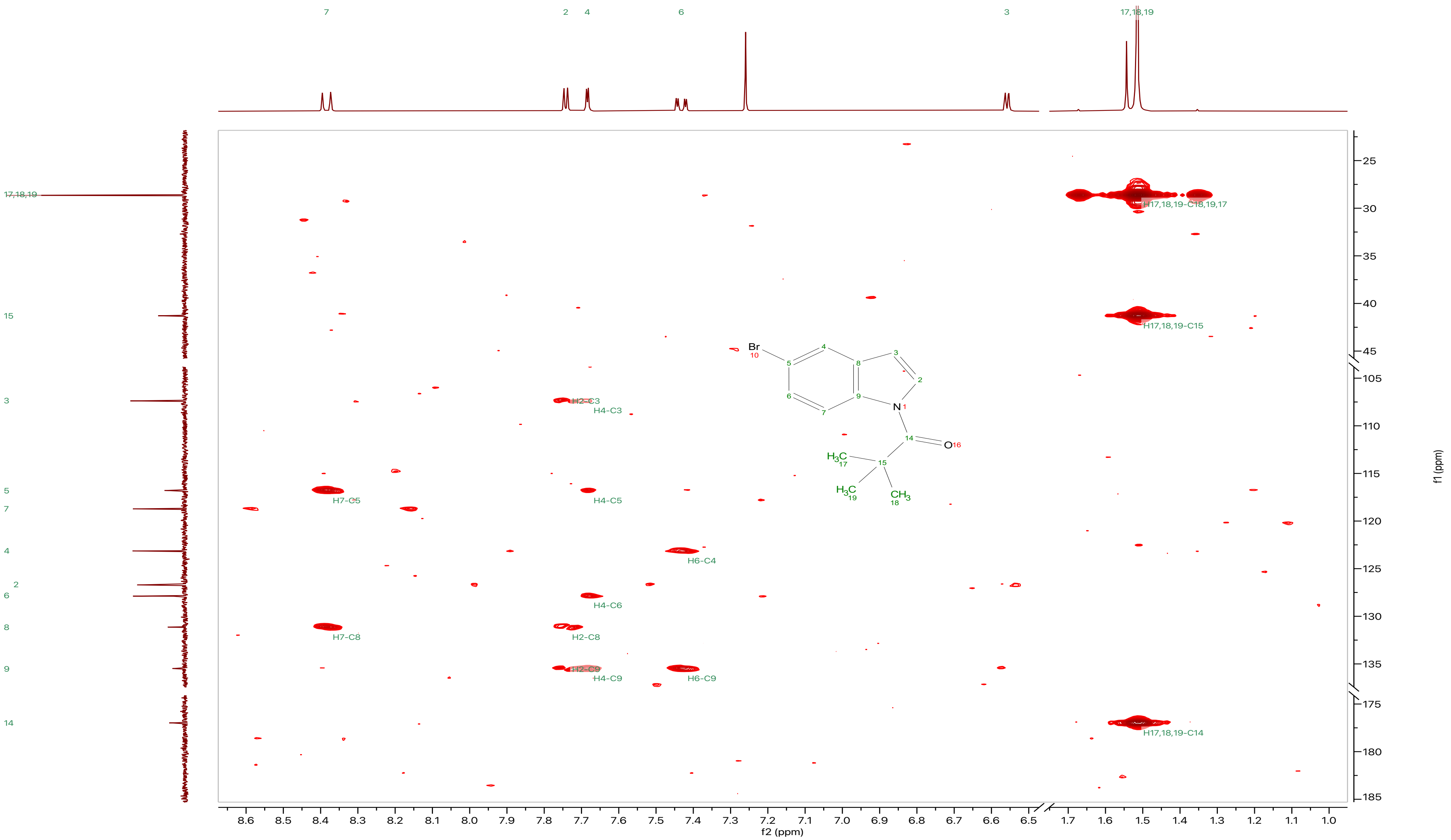

^{1}H-^{13}C{^{1}H} HMBC NMR (400/101 MHz, $CDCl_3$) of 1w

2aw

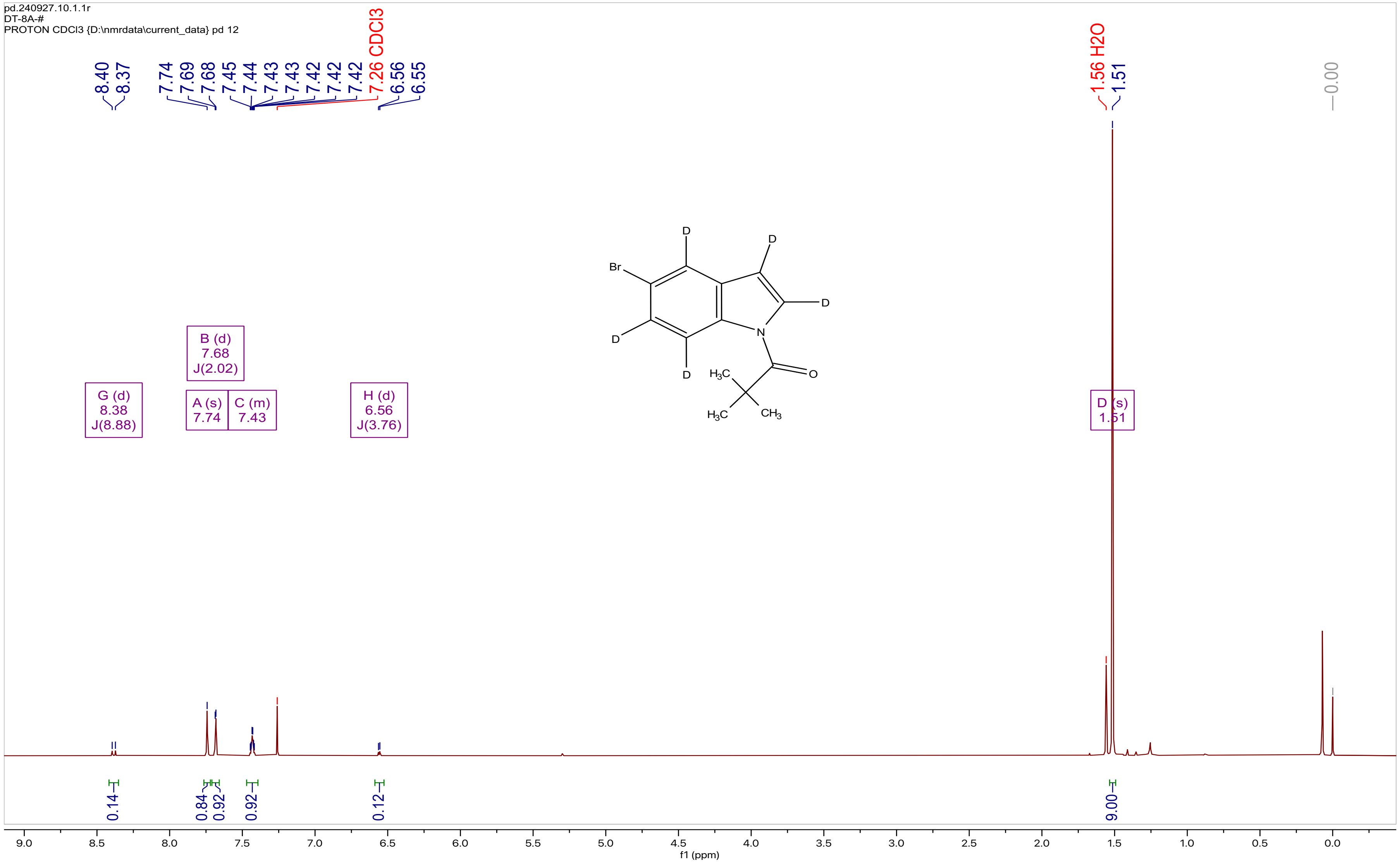

^{1}H NMR (400 MHz, $CDCl_3$) of 2aw

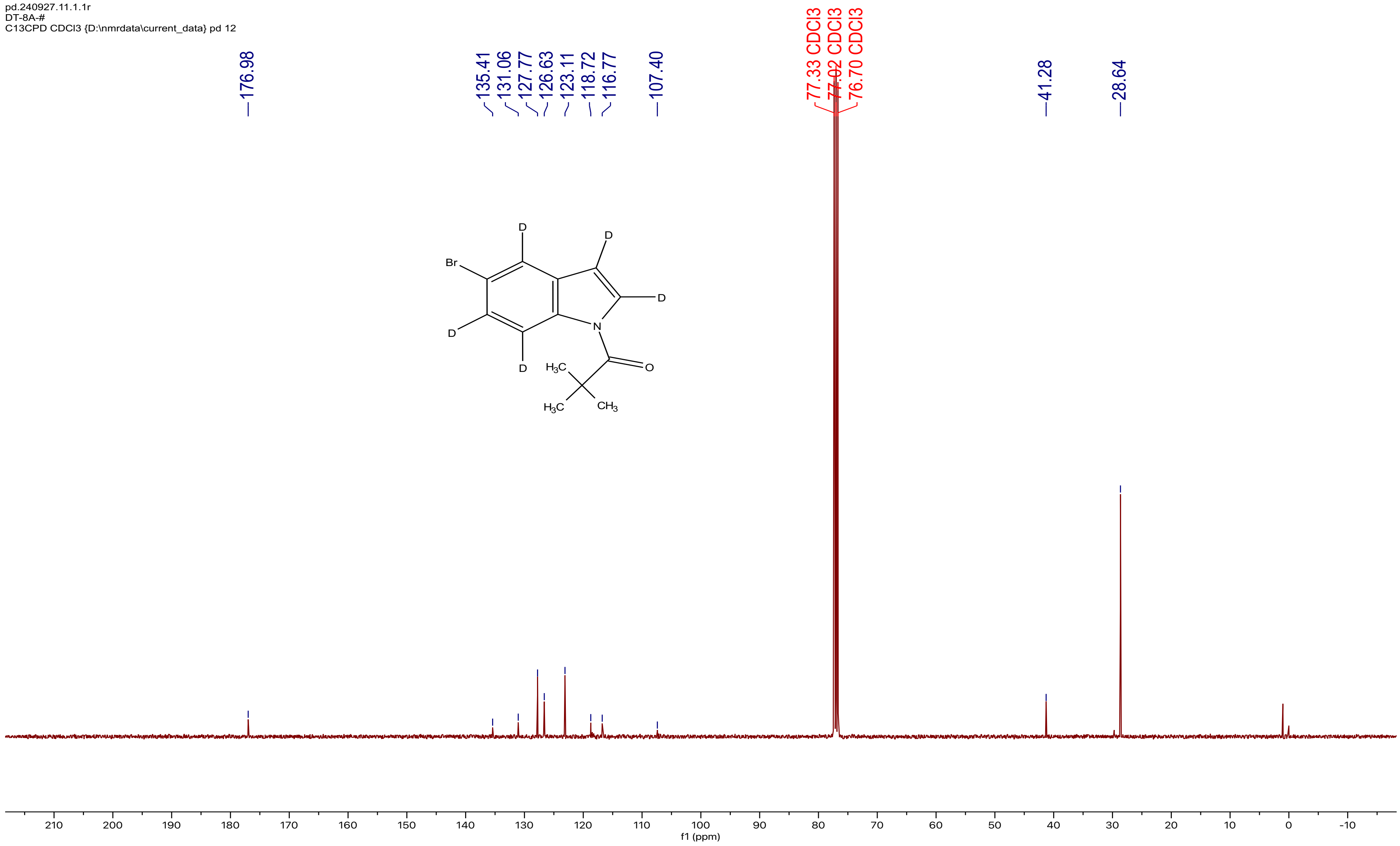

$^{13}C\{^1H\}$ NMR (101 MHz, $CDCl_3$) of 2aw

1w'

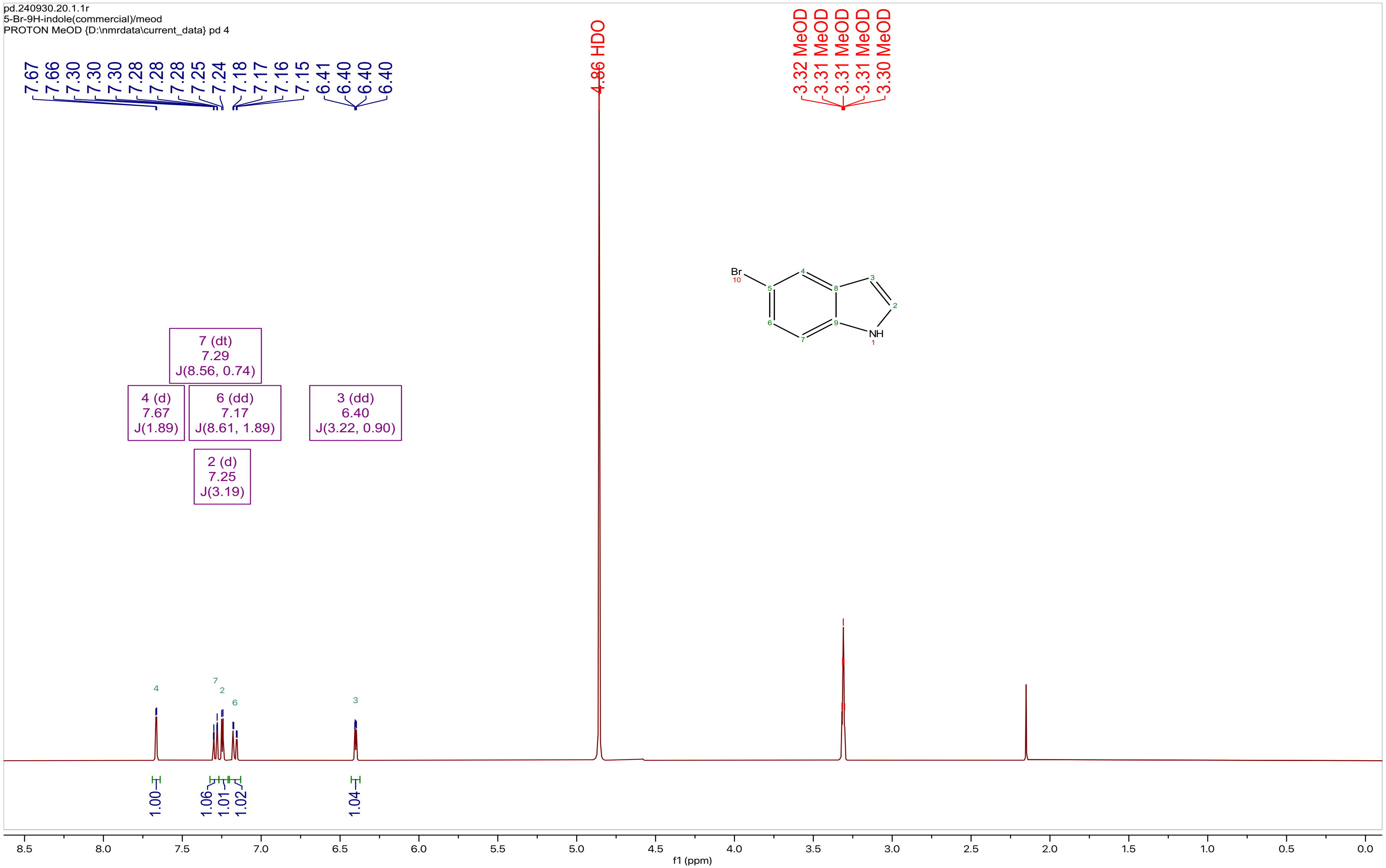

¹H NMR (400 MHz, MeOD) of 1w'

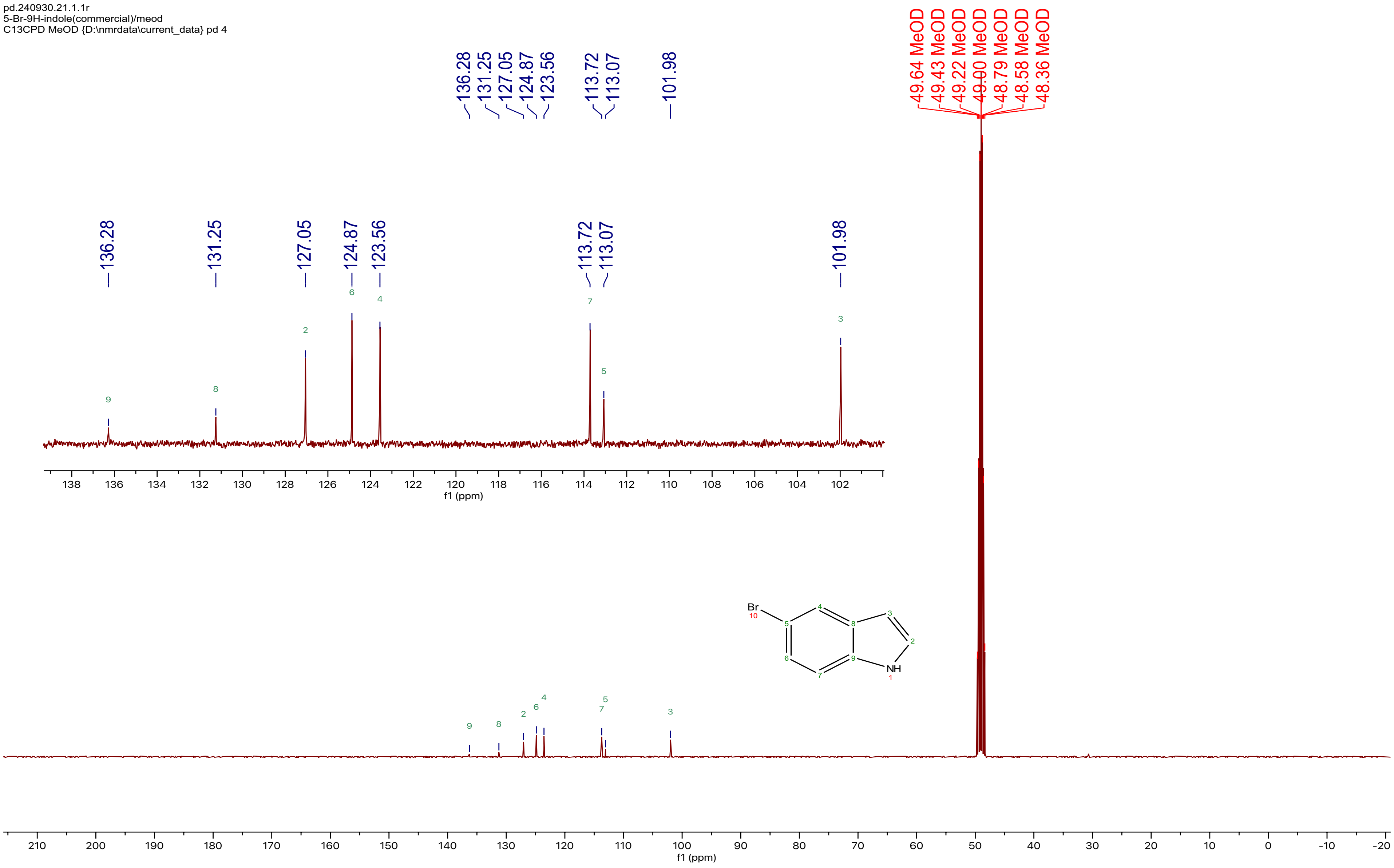

$^{13}C\{^{1}H\}$ NMR (101 MHz, MeOD) of 1w'

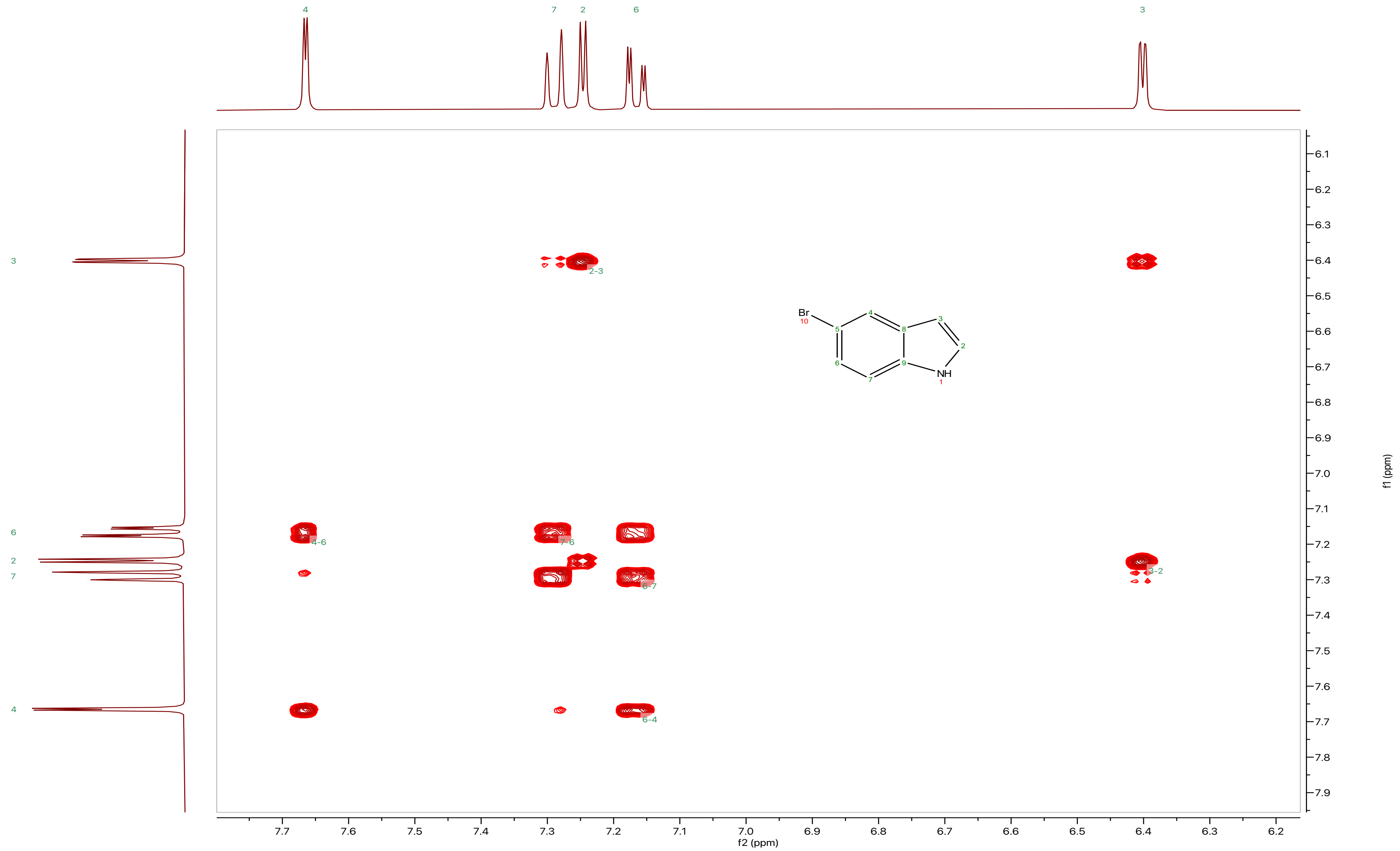

^1H-^1H COSY (400 MHz, MeOD) of 1w'

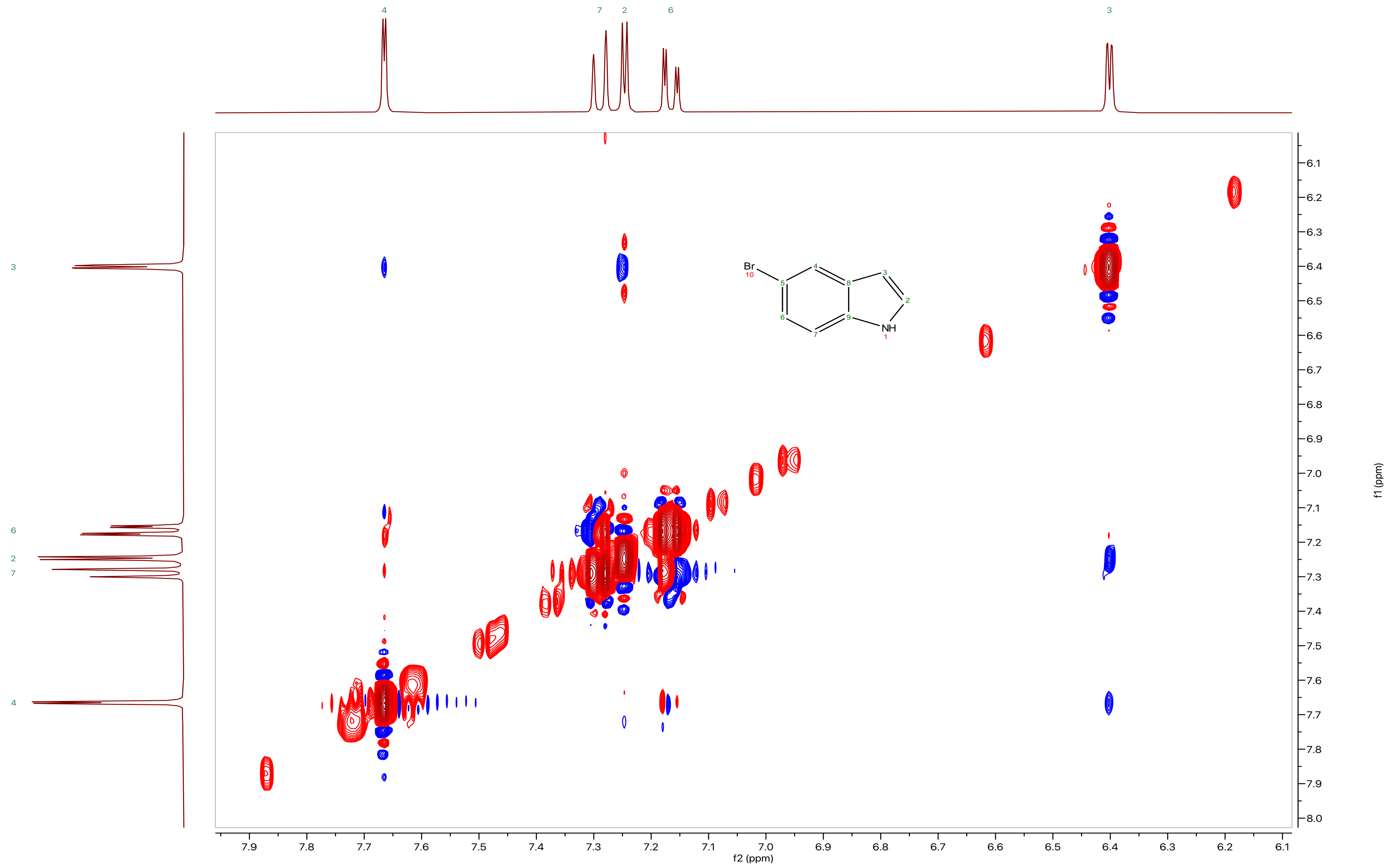

^{1}H-^{1}H NOESY (400 MHz, MeOD) of 1w'

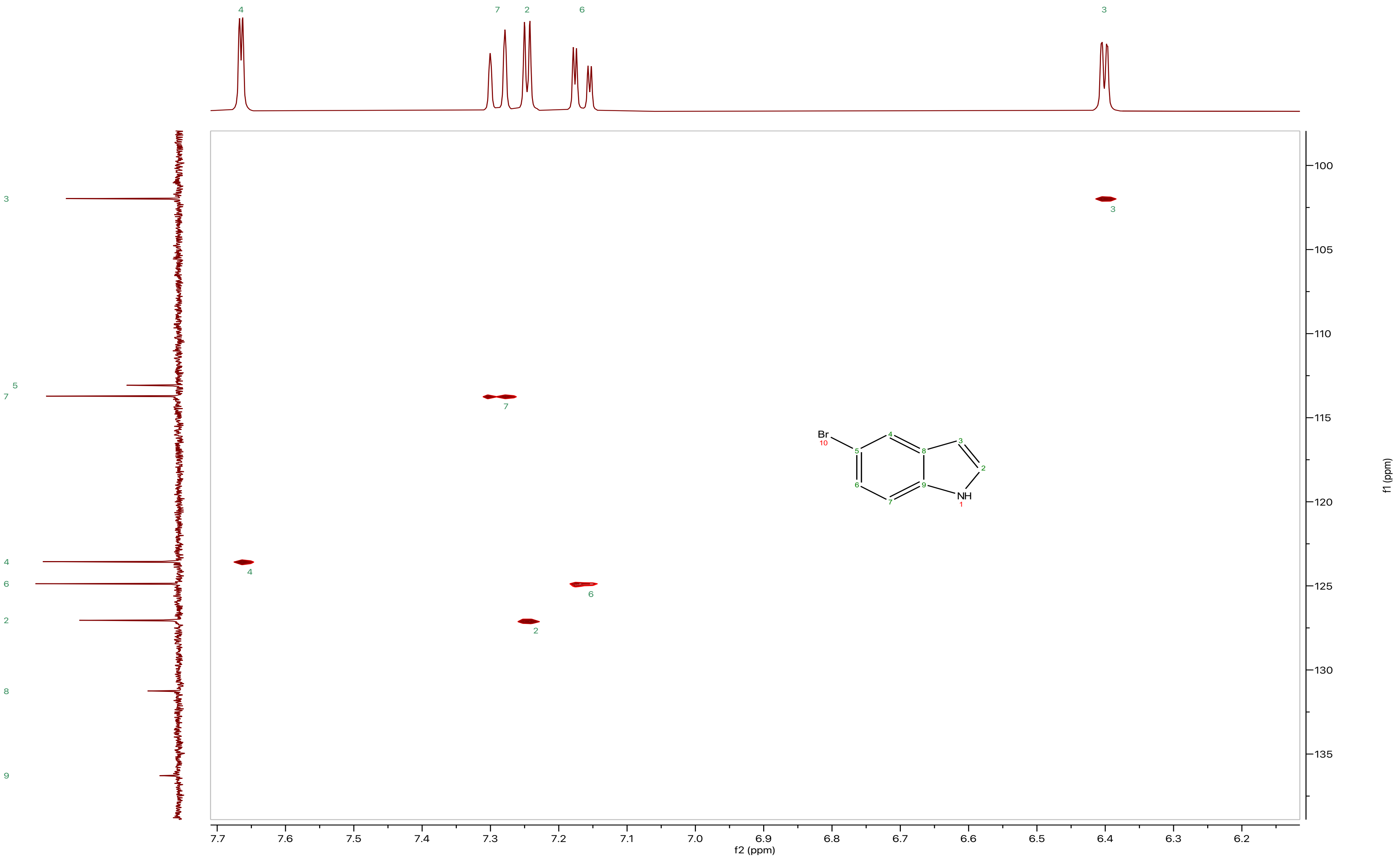

^1H-$^{13}C\{^1H\}$ HSQC NMR (400/101 MHz, MeOD) of 1w'

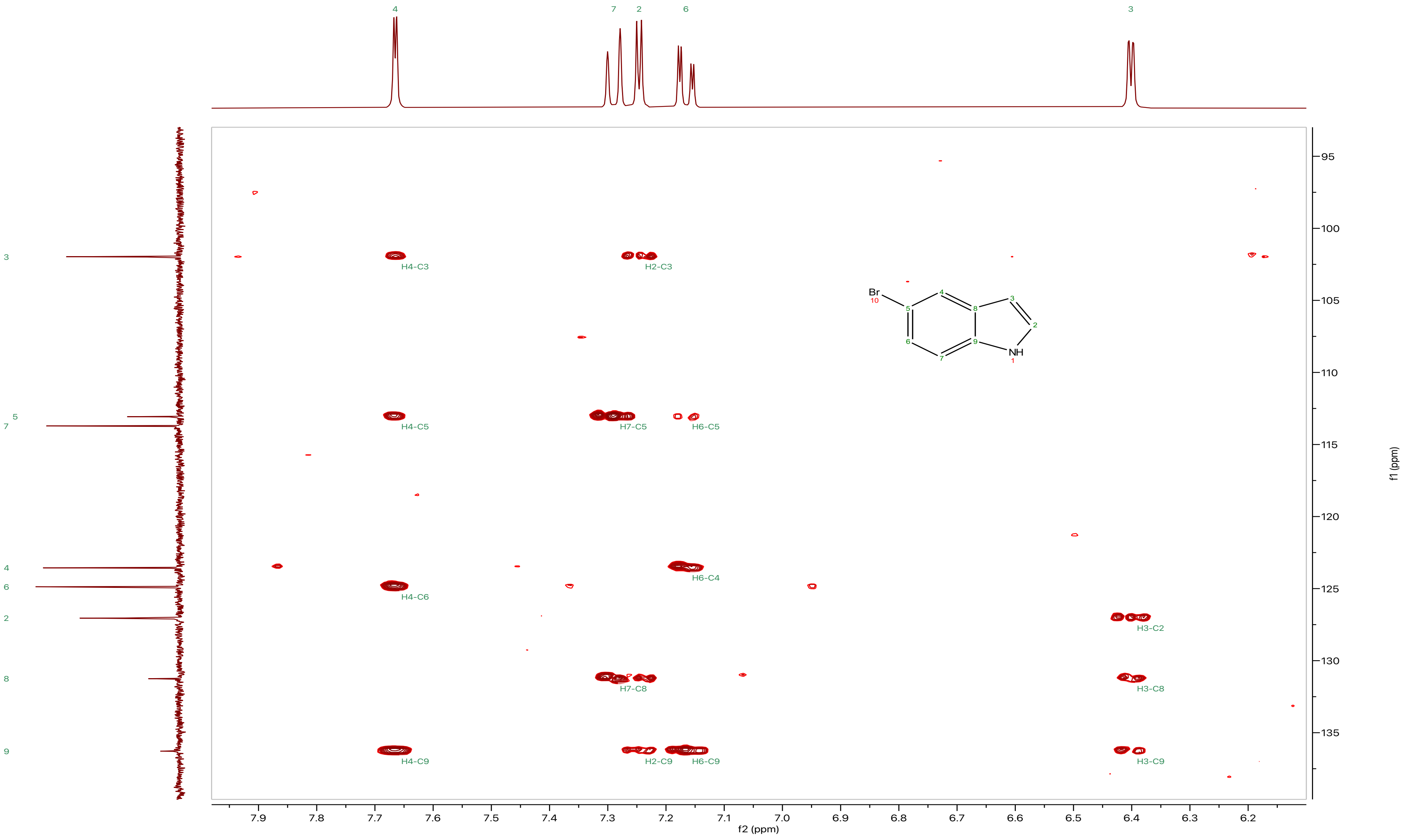

^{1}H-$^{13}C\{^{1}H\}$ HMBC NMR (400/101 MHz, MeOD) of 1w'

2bw

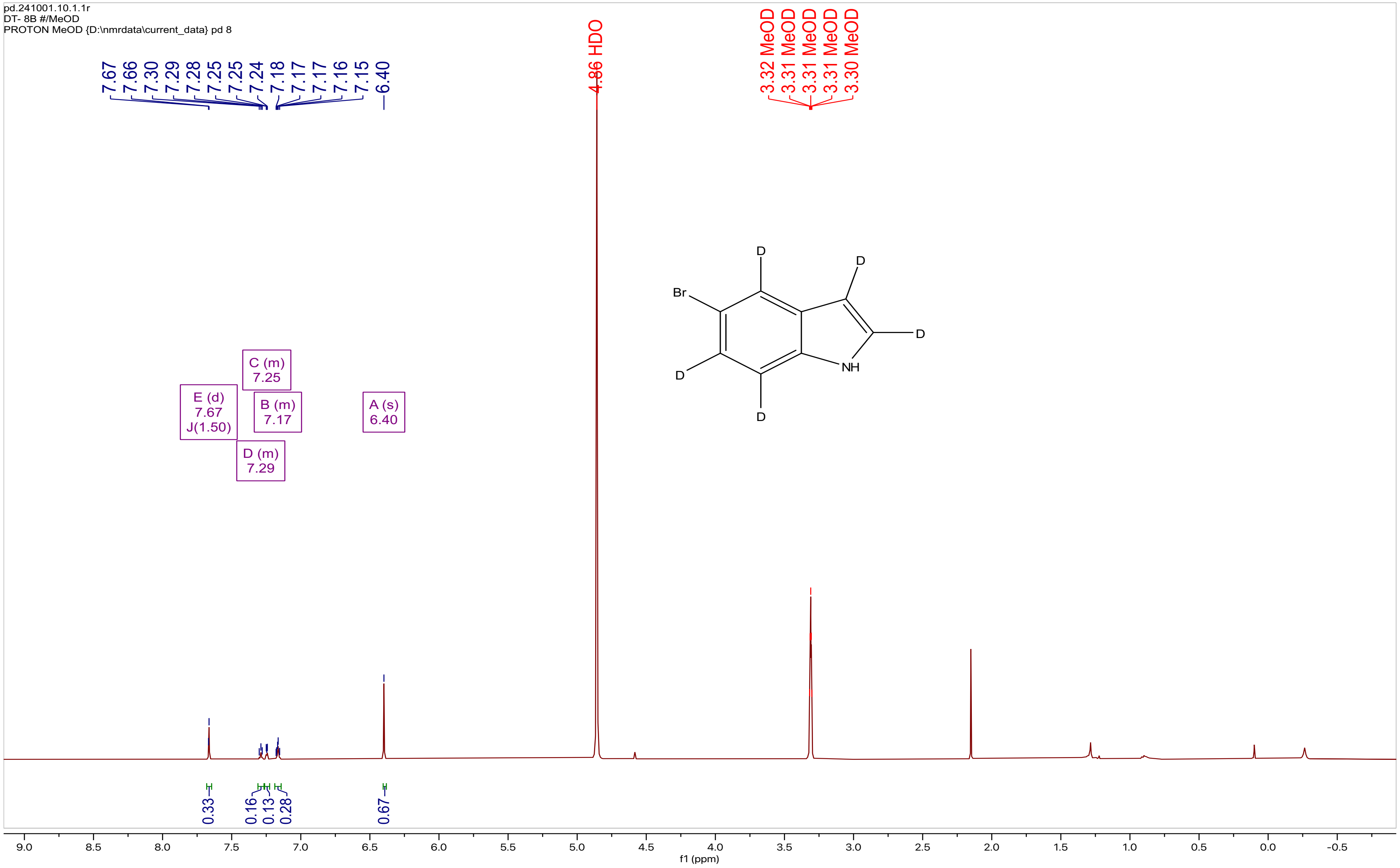

^{1}H NMR (400 MHz, MeOD) of 2bw

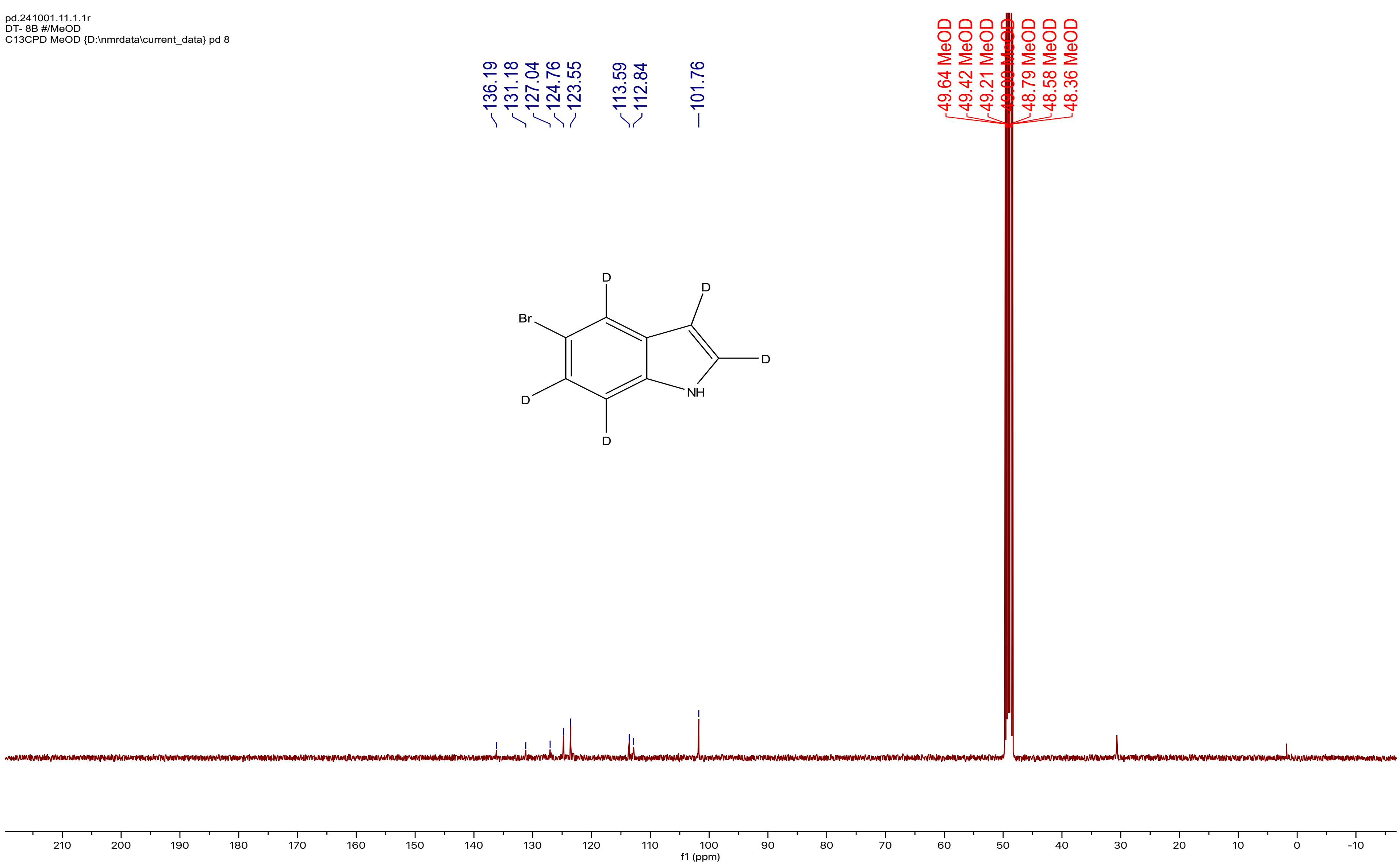

$^{13}C\{^1H\}$ NMR (101 MHz, MeOD) of 2bw

1x

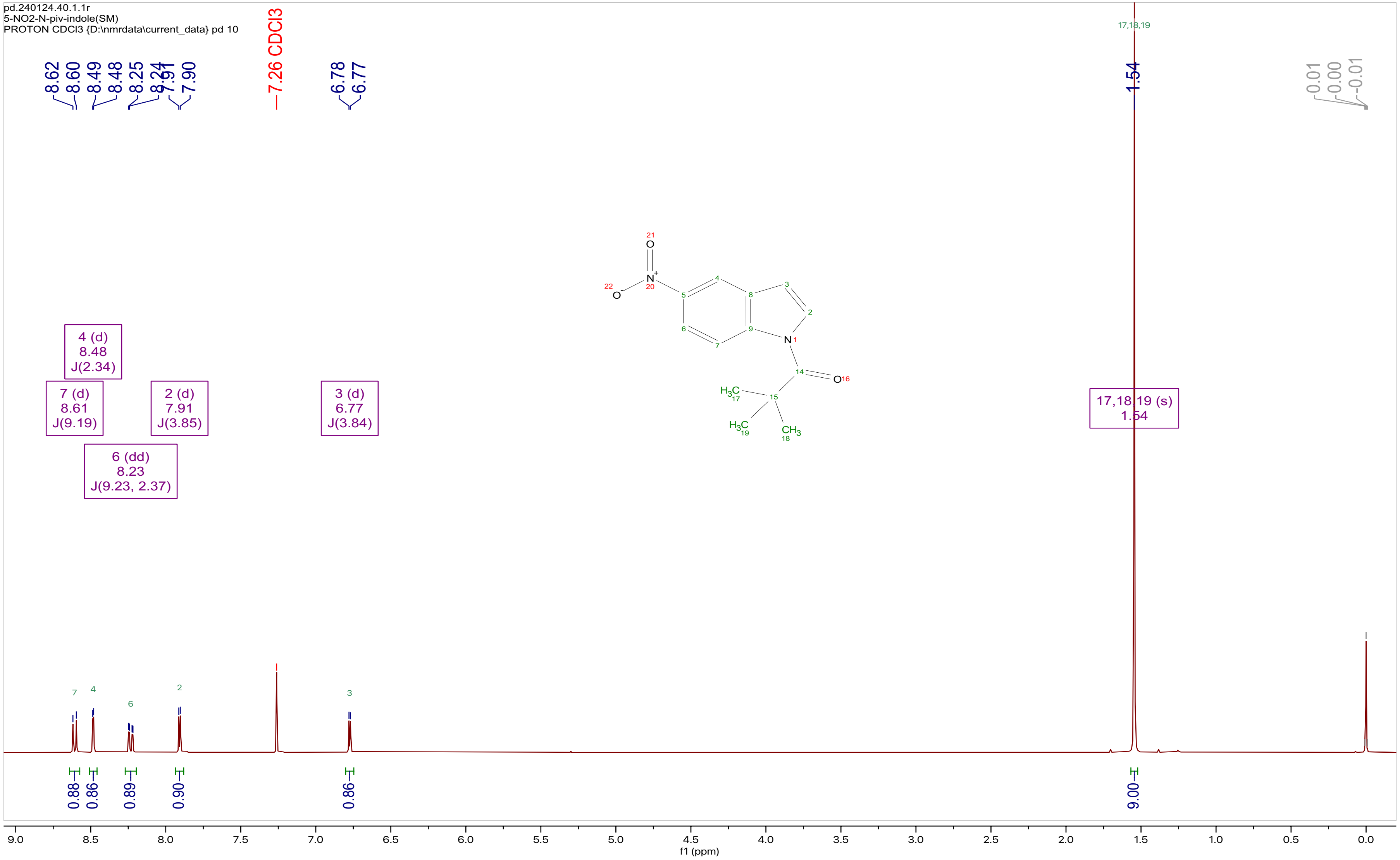

^1H NMR (400 MHz, $CDCl_3$) of 1x

pd.240124.41.1.1r
5-NO2-N-piv-indole(SM)
C13CPD CDCl3 {D:\nmrdata\current_data} pd 10

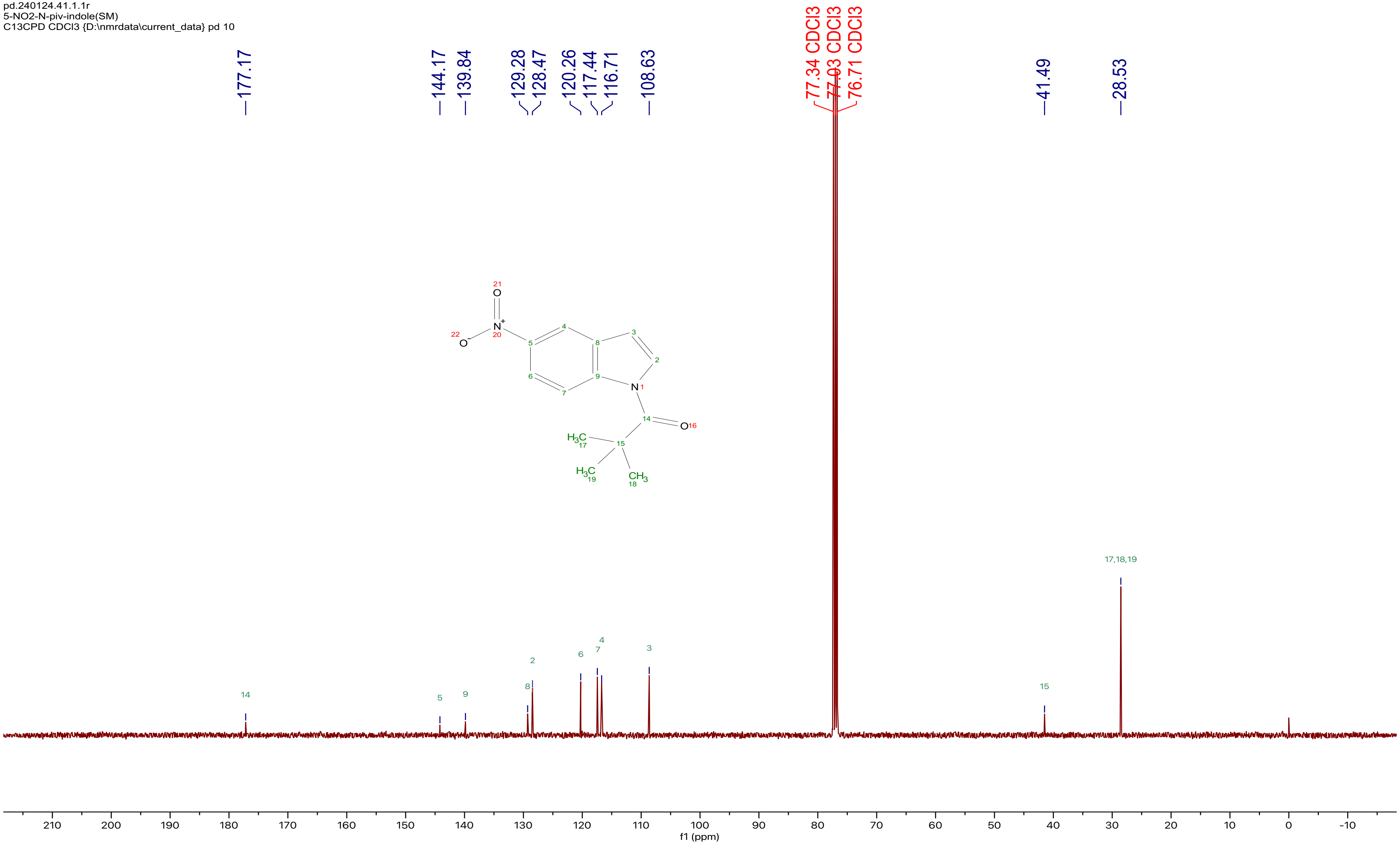

$^{13}C\{^{1}H\}$ NMR (101 MHz, $CDCl_3$) of 1x

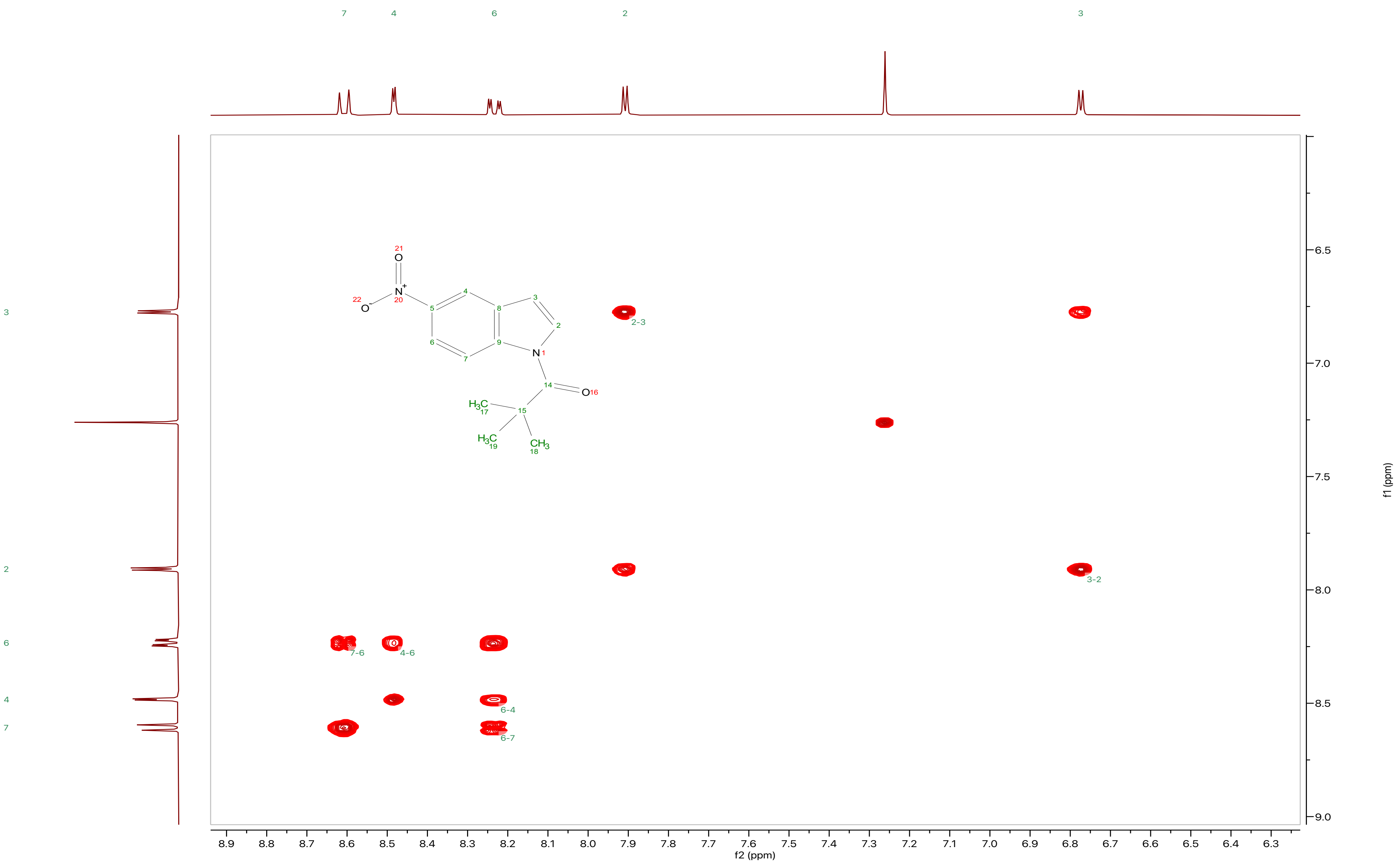

^1H-^1H COSY (400 MHz, $CDCl_3$) of 1x

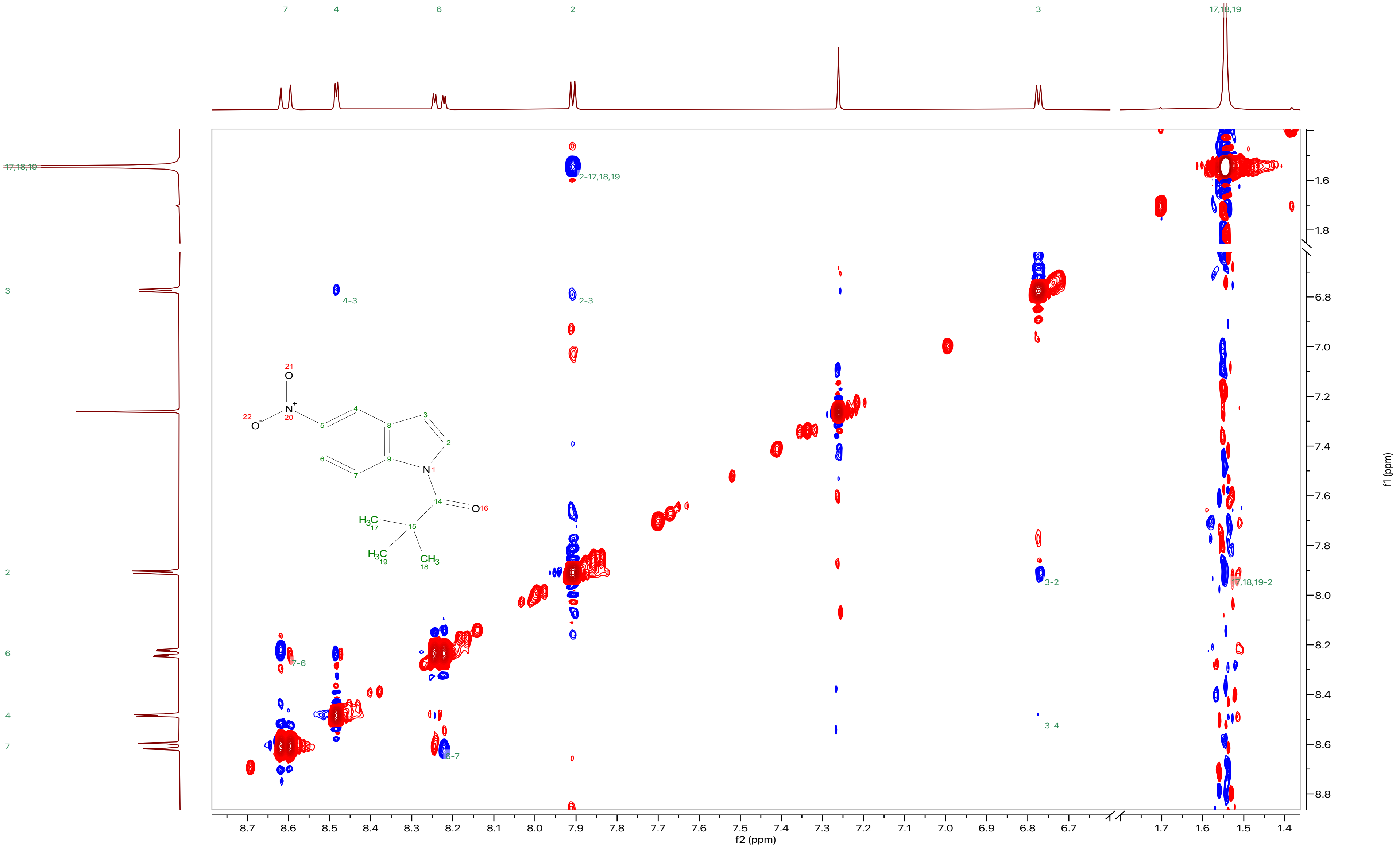

^{1}H-^{1}H NOESY (400 MHz, $CDCl_3$) of 1x

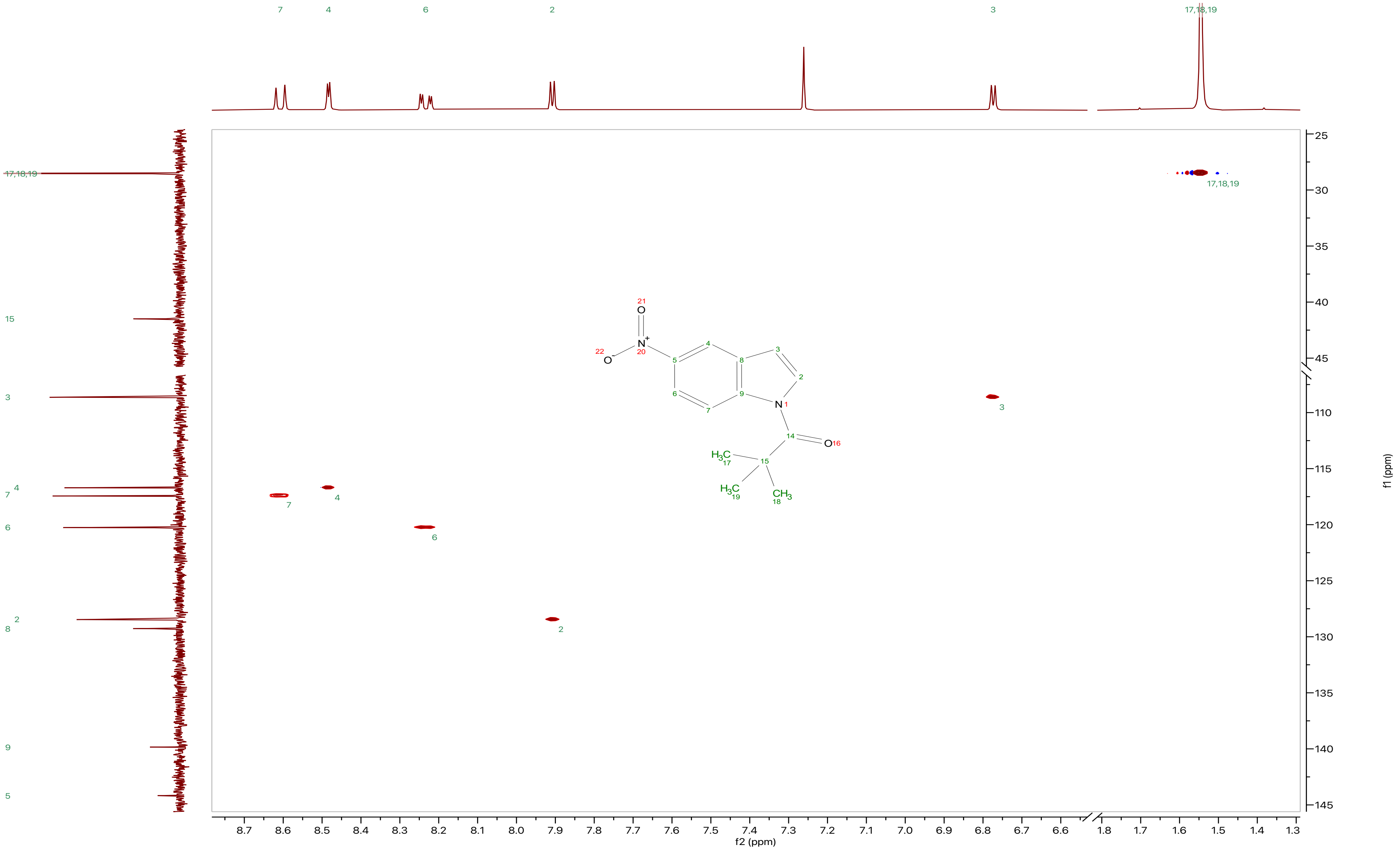

^{1}H-$^{13}C\{^{1}H\}$ HSQC NMR (400/101 MHz, $CDCl_3$) of 1x

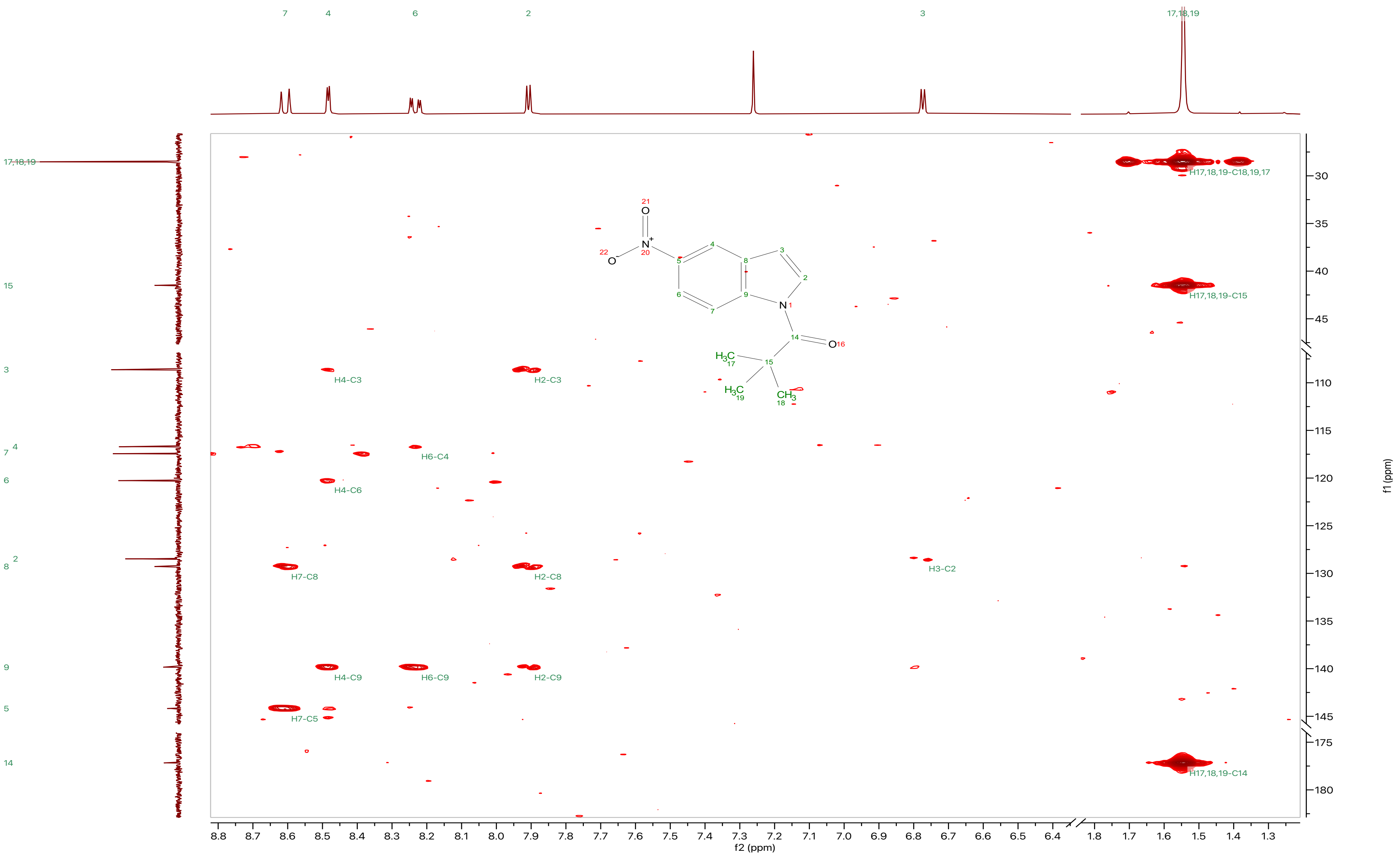

^{1}H-$^{13}C\{^{1}H\}$ HMBC NMR (400/101 MHz, $CDCl_3$) of 1x

2ax

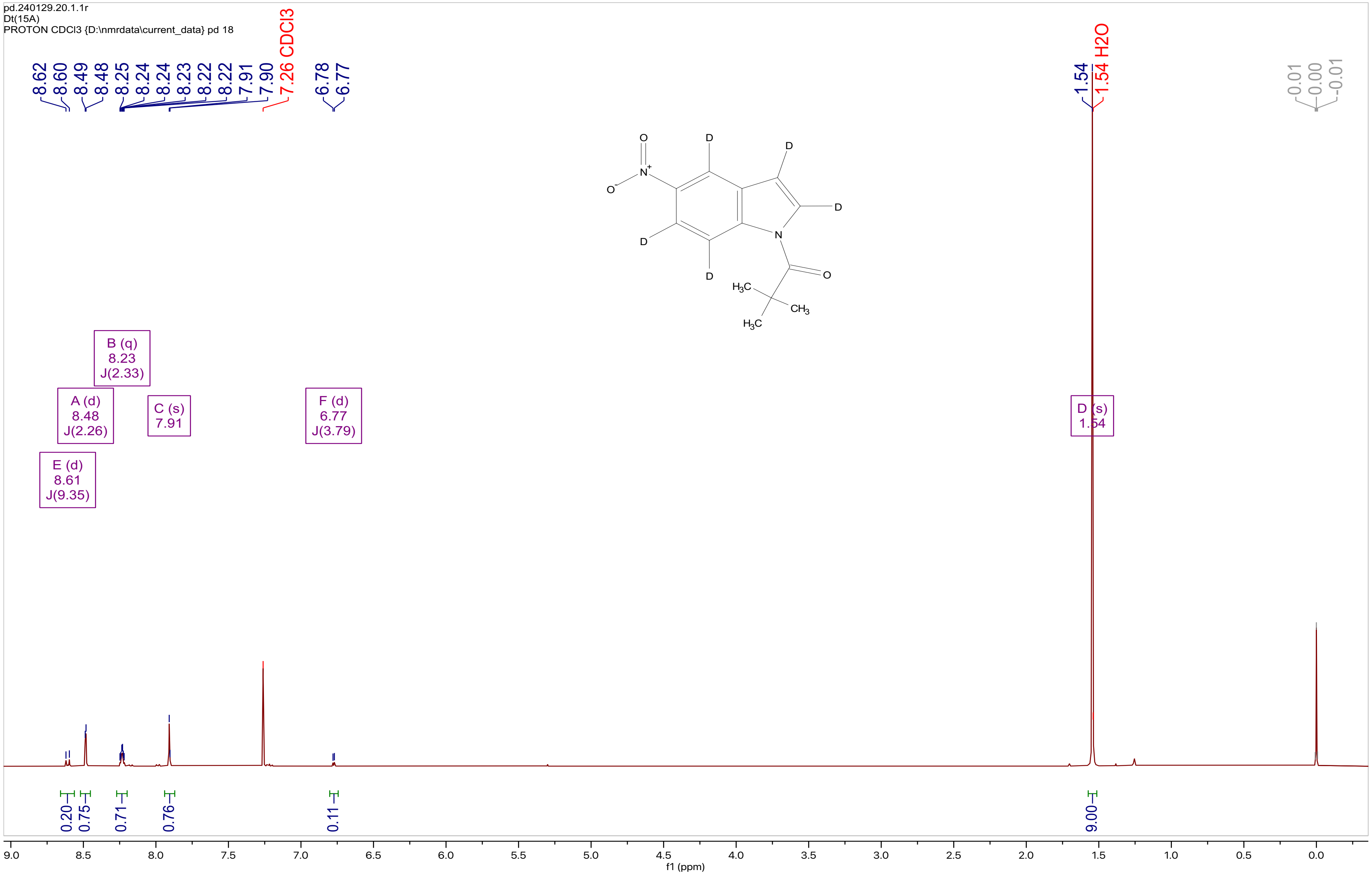

^1H NMR (400 MHz, $CDCl_3$) of 2ax

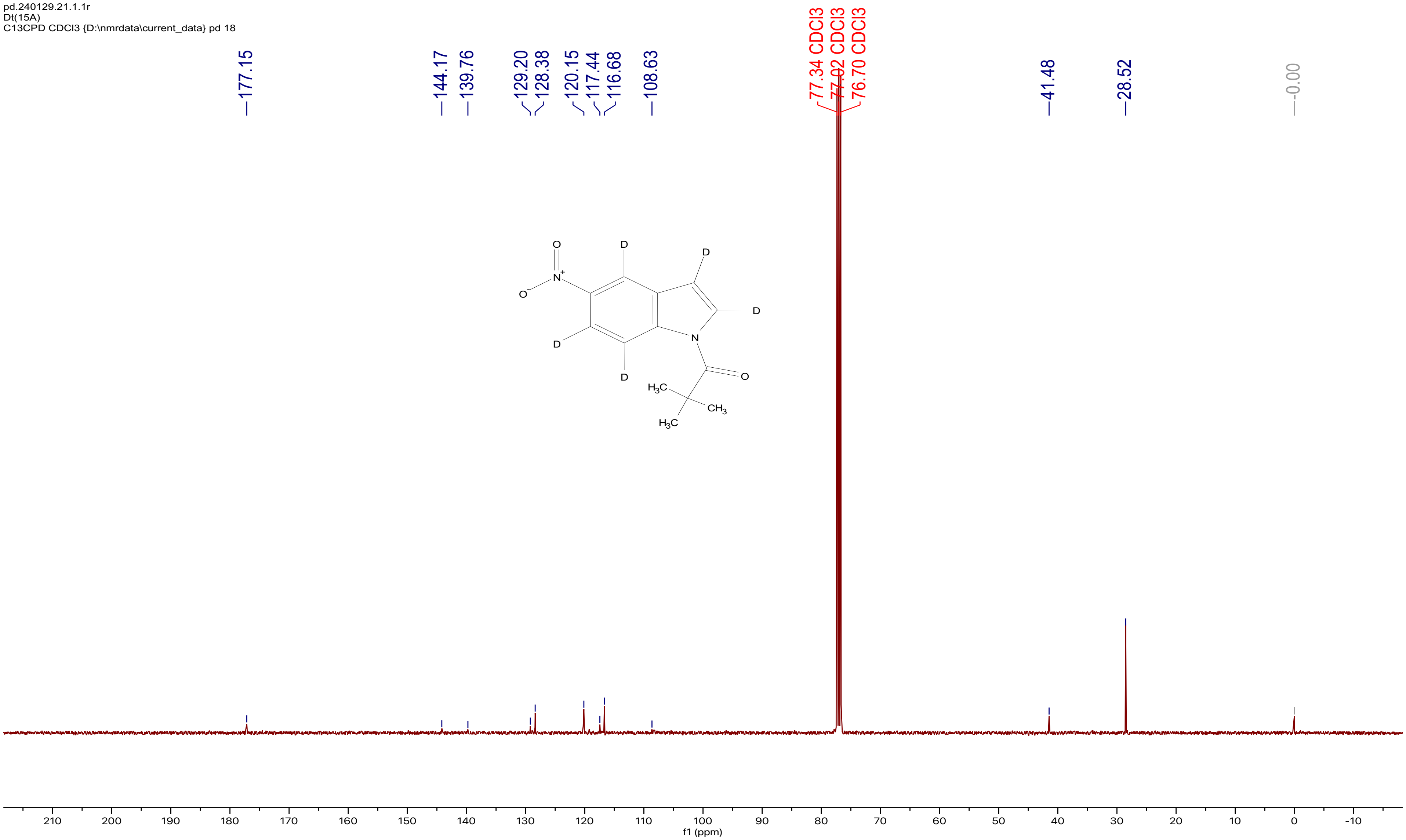

$^{13}C\{^{1}H\}$ NMR (101 MHz, $CDCl_3$) of 2ax

1x’

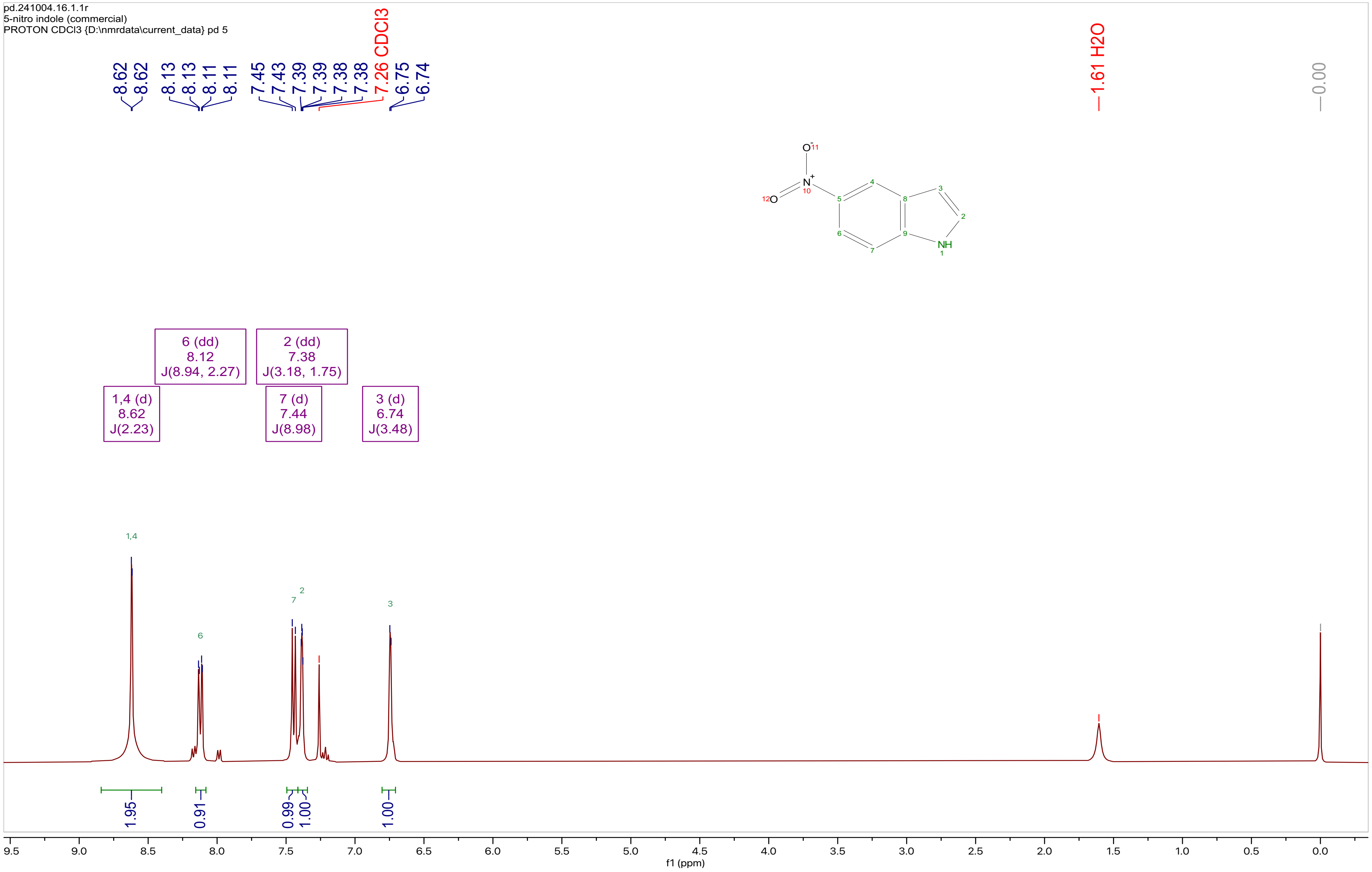

^1H NMR (400 MHz, $CDCl_3$) of 1x’

pd.241004.17.1.1r
5-nitro indole (commercial)
C13CPD CDCl3 {D:\nmrdata\current_data} pd 5

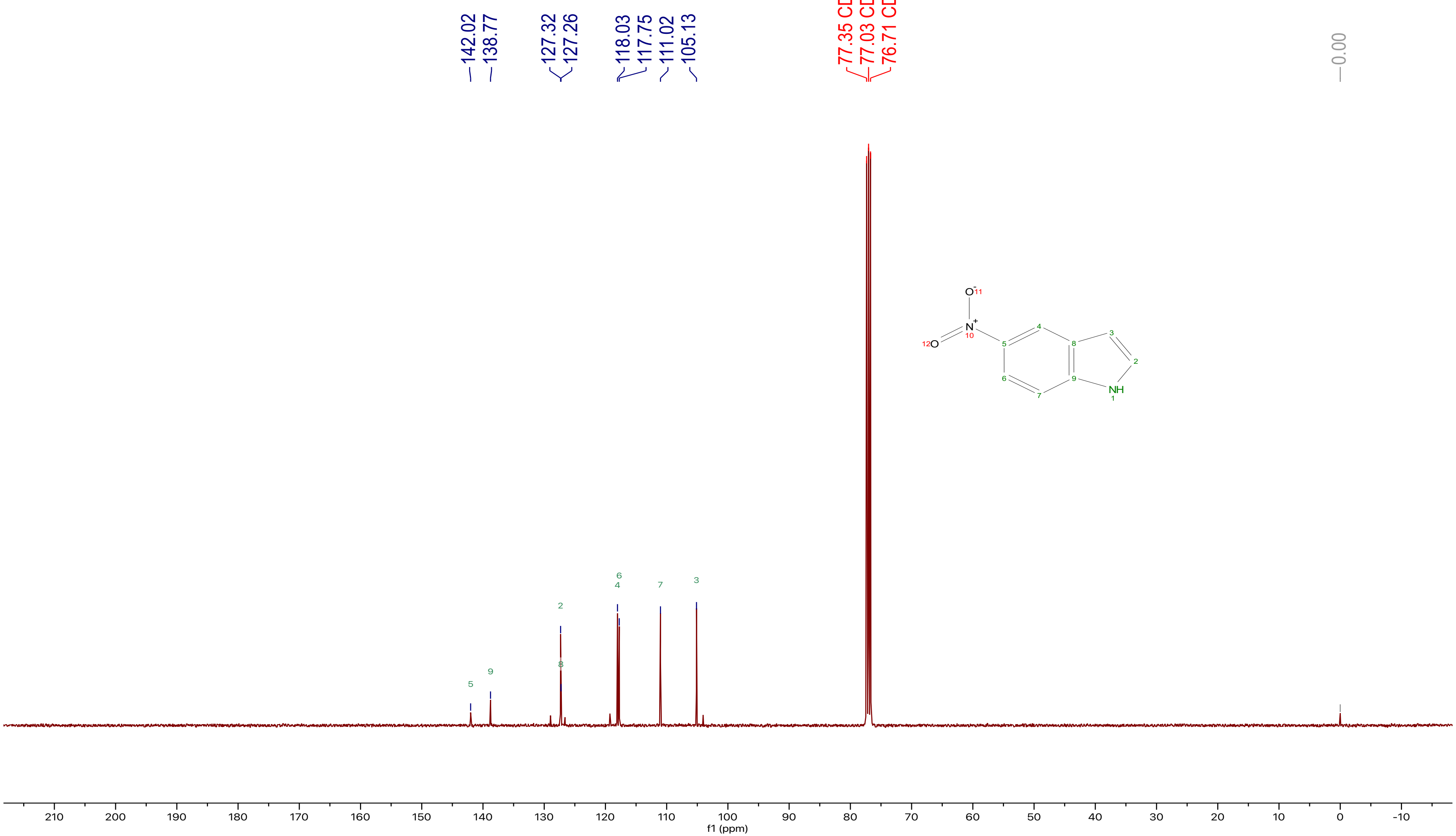

$^{13}C\{^1H\}$ NMR (101 MHz, $CDCl_3$) of 1x’

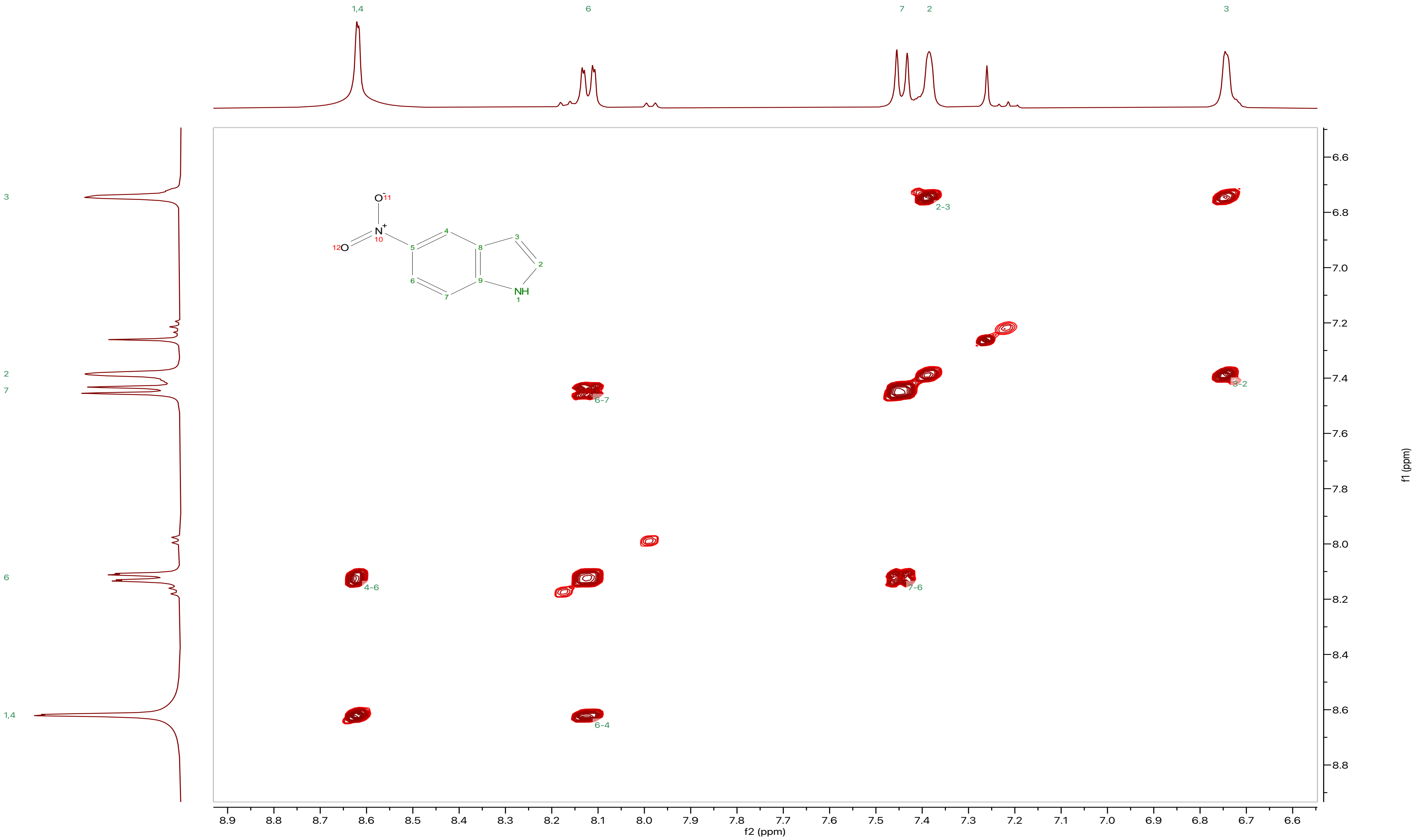

^{1}H-^{1}H COSY (400 MHz, $CDCl_3$) of 1x'

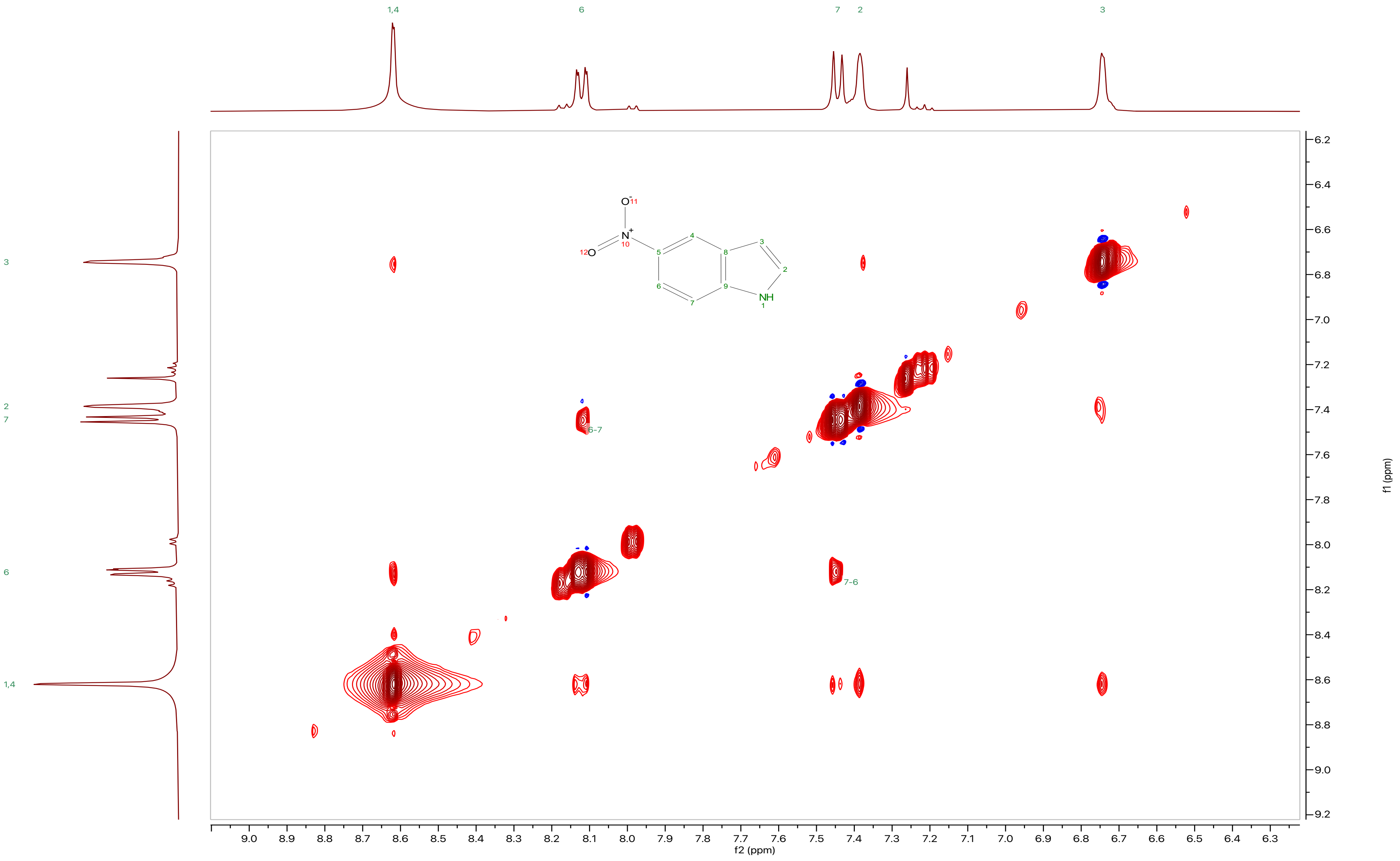

^{1}H-^{1}H NOESY (400 MHz, $CDCl_3$) of 1x'

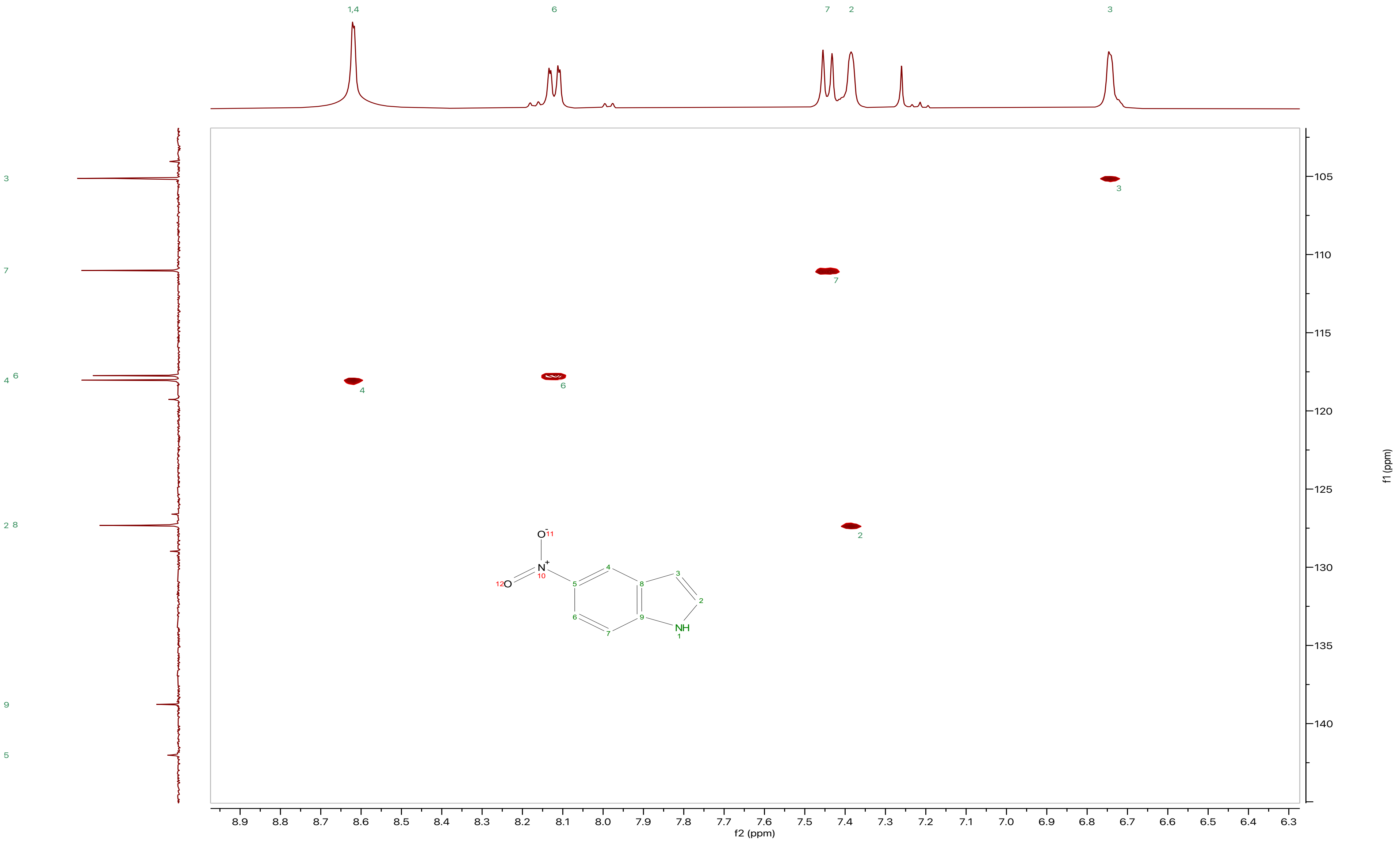

^{1}H-^{13}C{^{1}H} HSQC NMR (400/101 MHz, $CDCl_3$) of 1x'

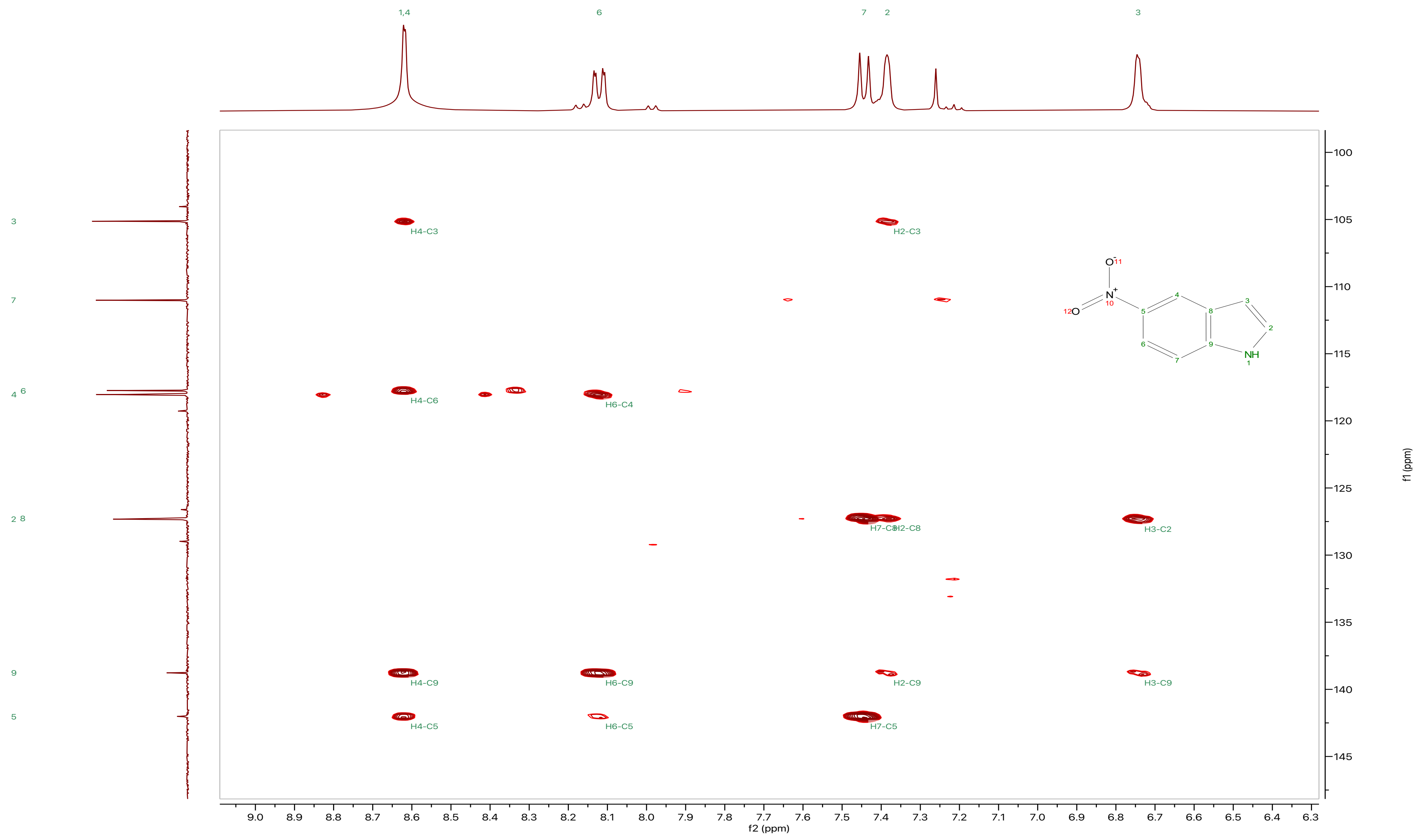

^{1}H-^{13}C{^{1}H} HMBC NMR (400/101 MHz, $CDCl_3$) of 1x’

2bx

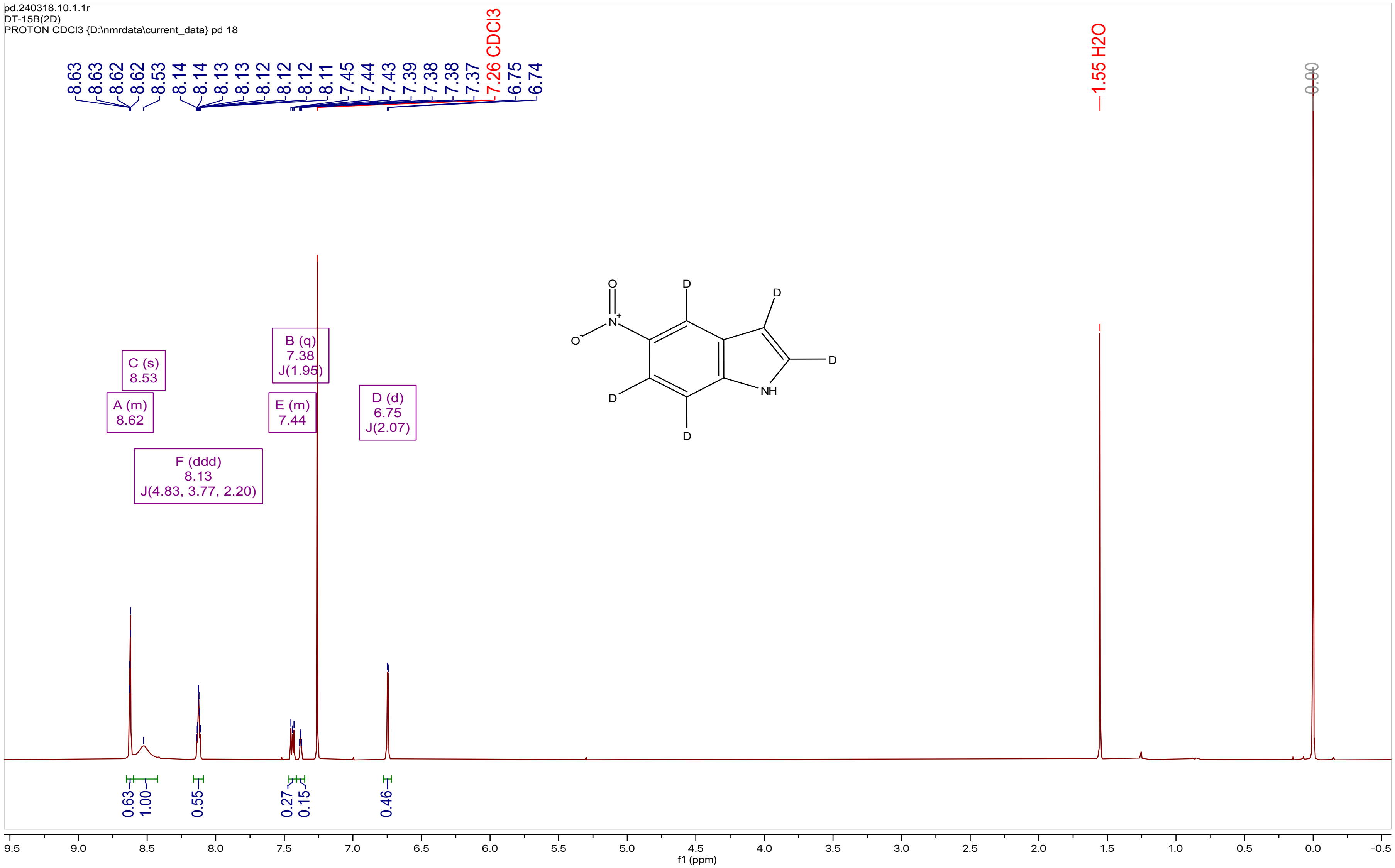

^{1}H NMR (400 MHz, $CDCl_3$) of 2bx

pd.240318.11.1.1r
DT-15B(2D)
C13CPD CDCl3 {D:\nmrdata\current_data} pd 18

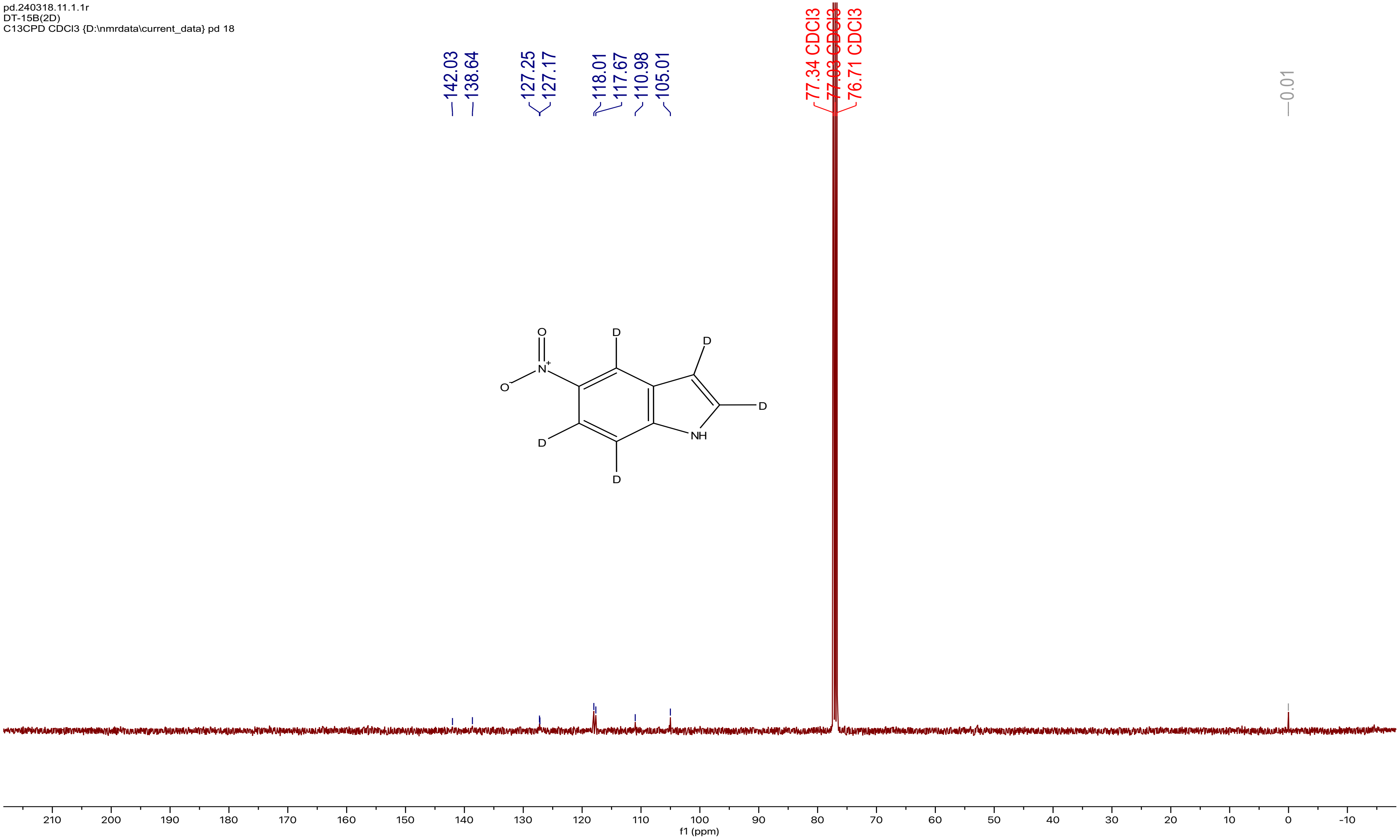

$^{13}C\{^1H\}$ NMR (101 MHz, $CDCl_3$) of 2bx

1y

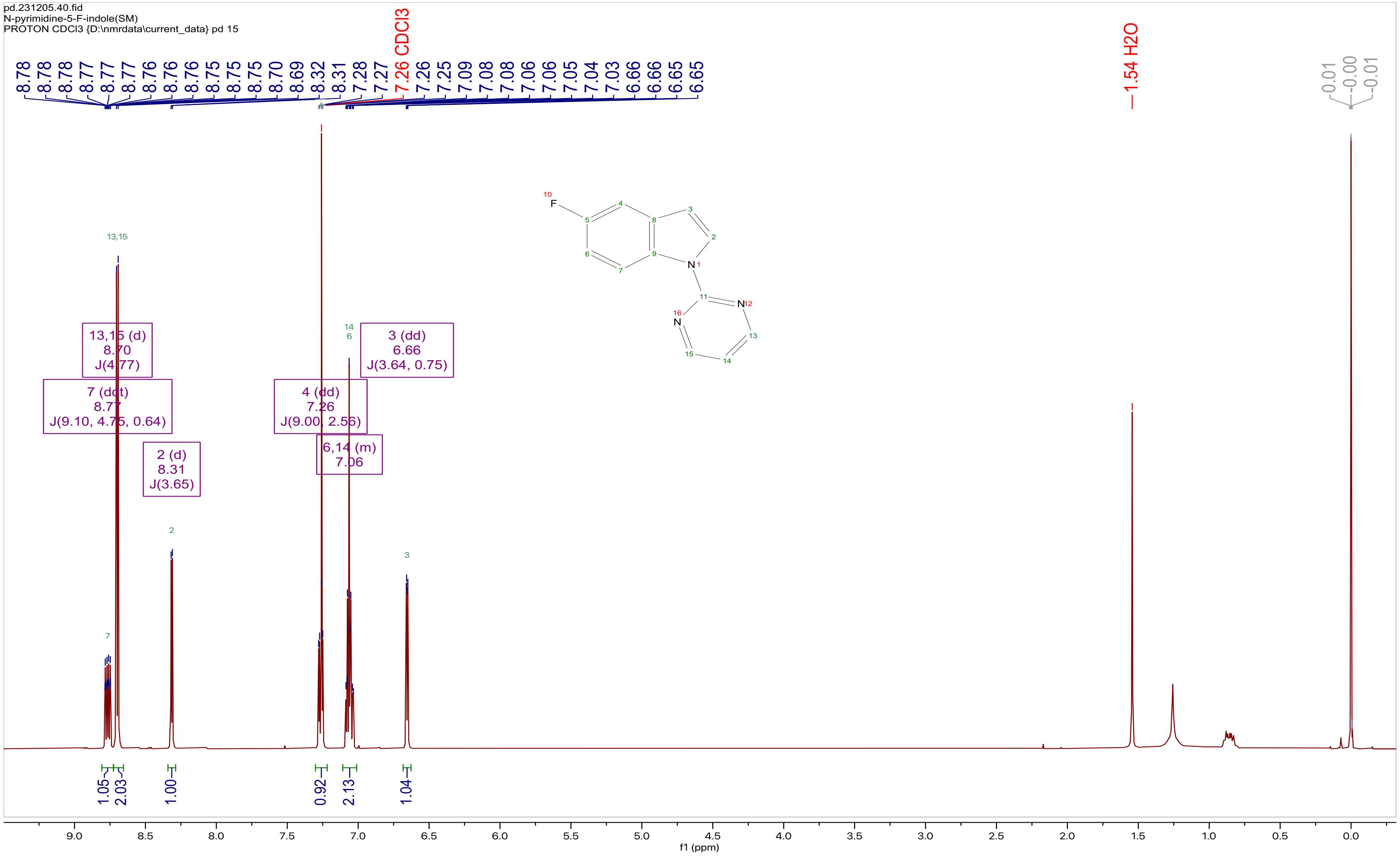

^{1}H NMR (400 MHz, $CDCl_3$) of 1y

pd.231205.41.fid
N-pyrimidine-5-F-indole(SM)
C13CPD CDCl3 {D:\nmrdata\current_data} pd 15

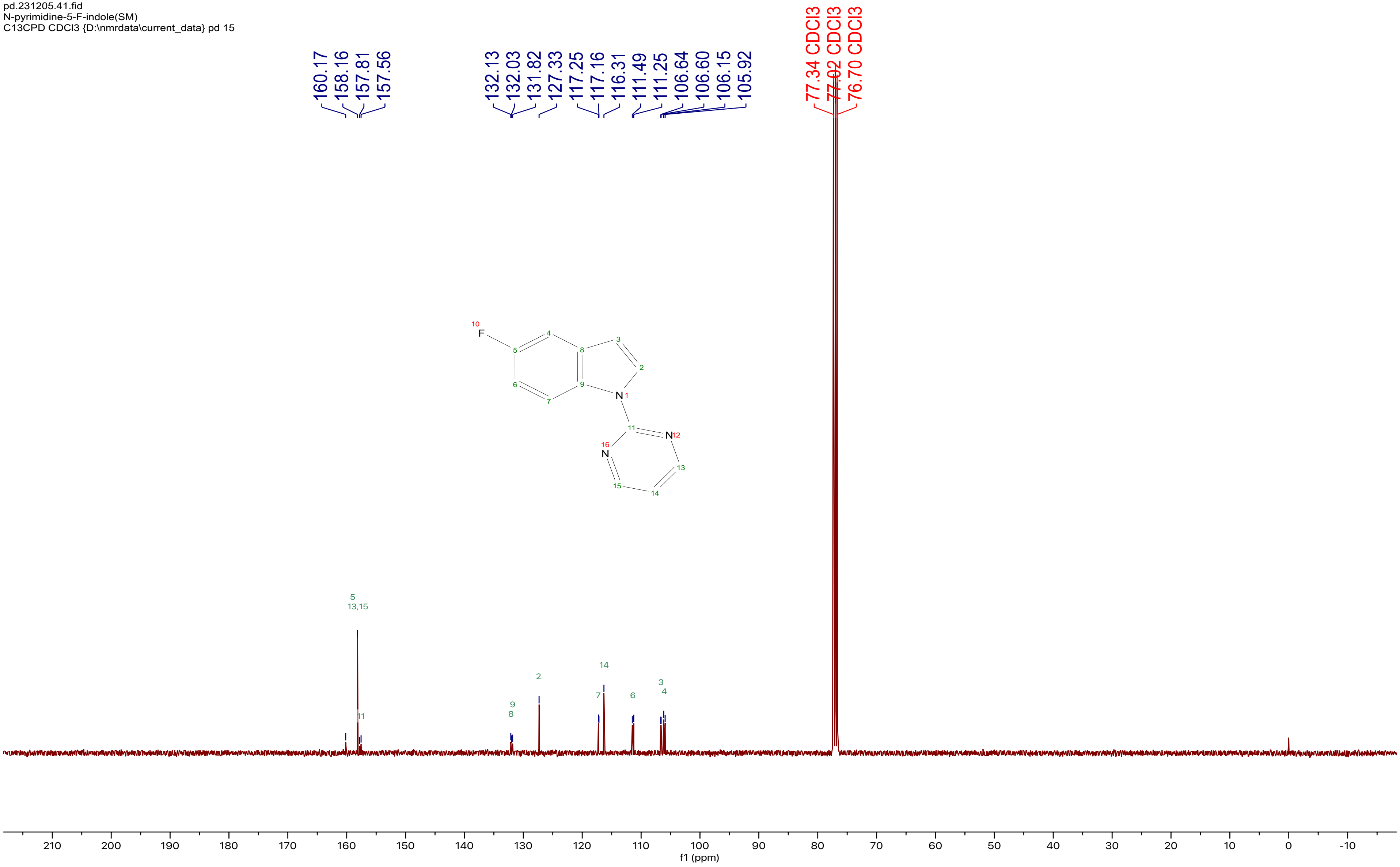

$^{13}C\{^{1}H\}$ NMR (101 MHz, $CDCl_3$) of 1y

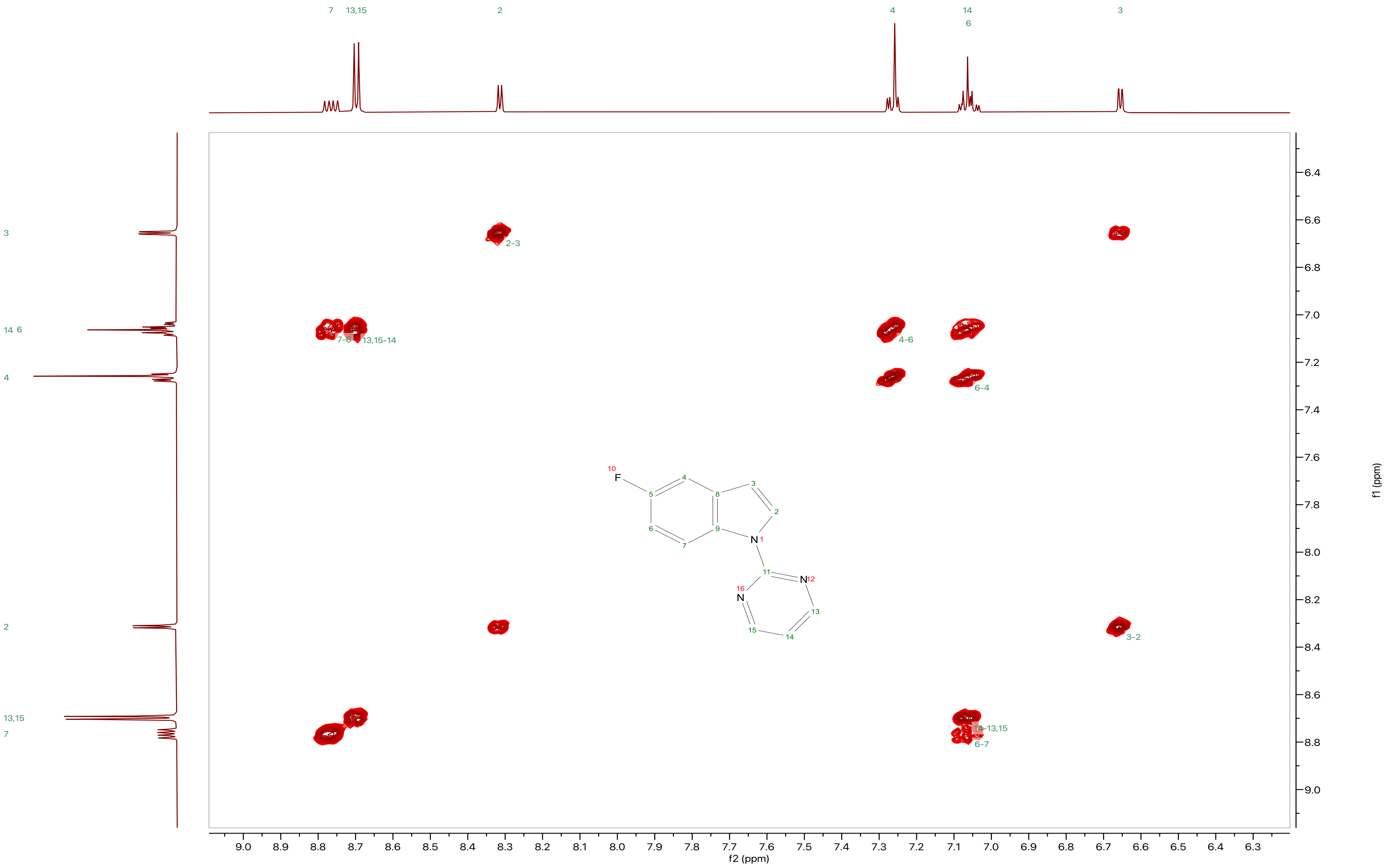

^{1}H-^{1}H COSY (400 MHz, $CDCl_3$) of 1y

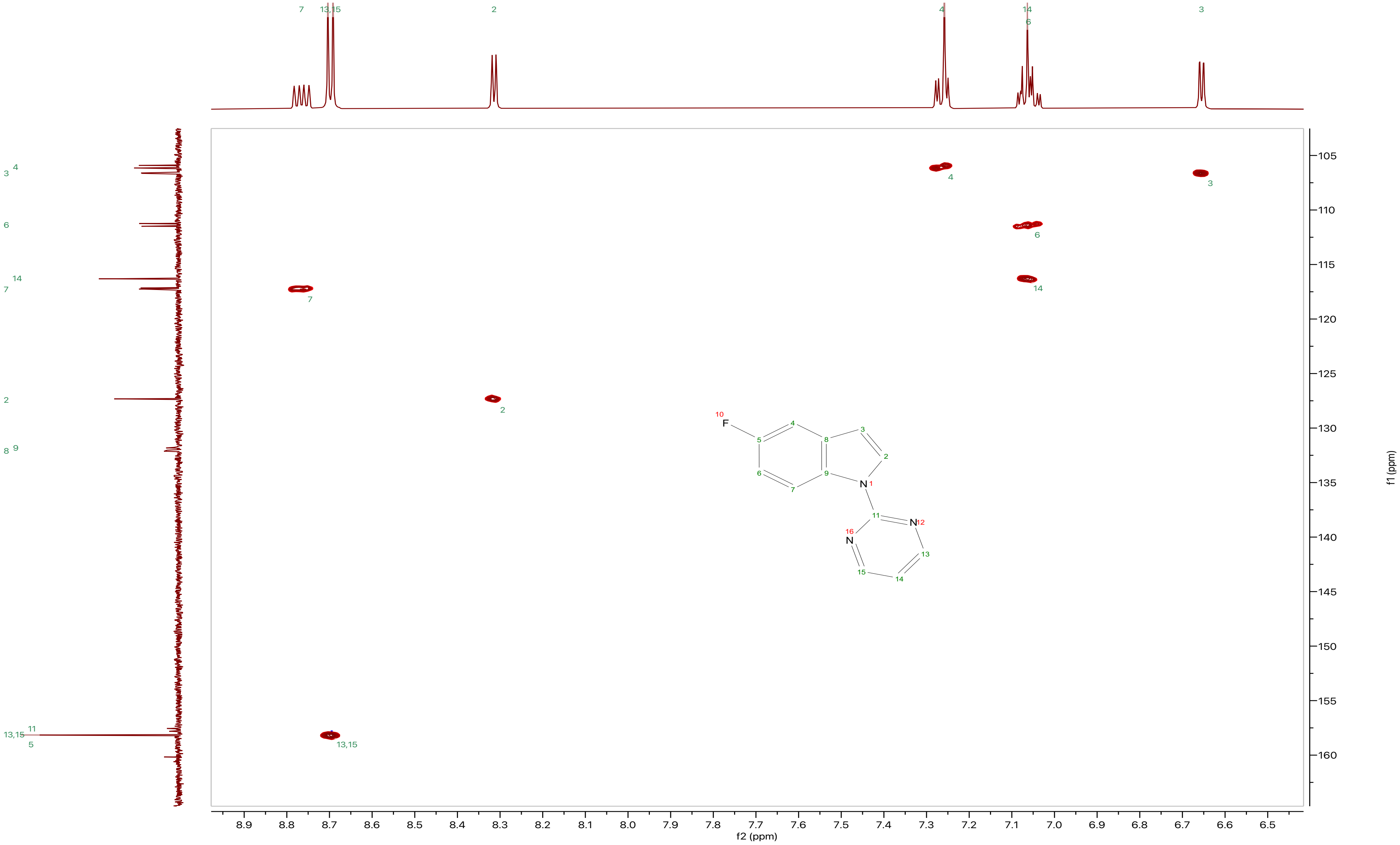

^{1}H-^{1}H NOESY (400 MHz, $CDCl_3$) of 1y

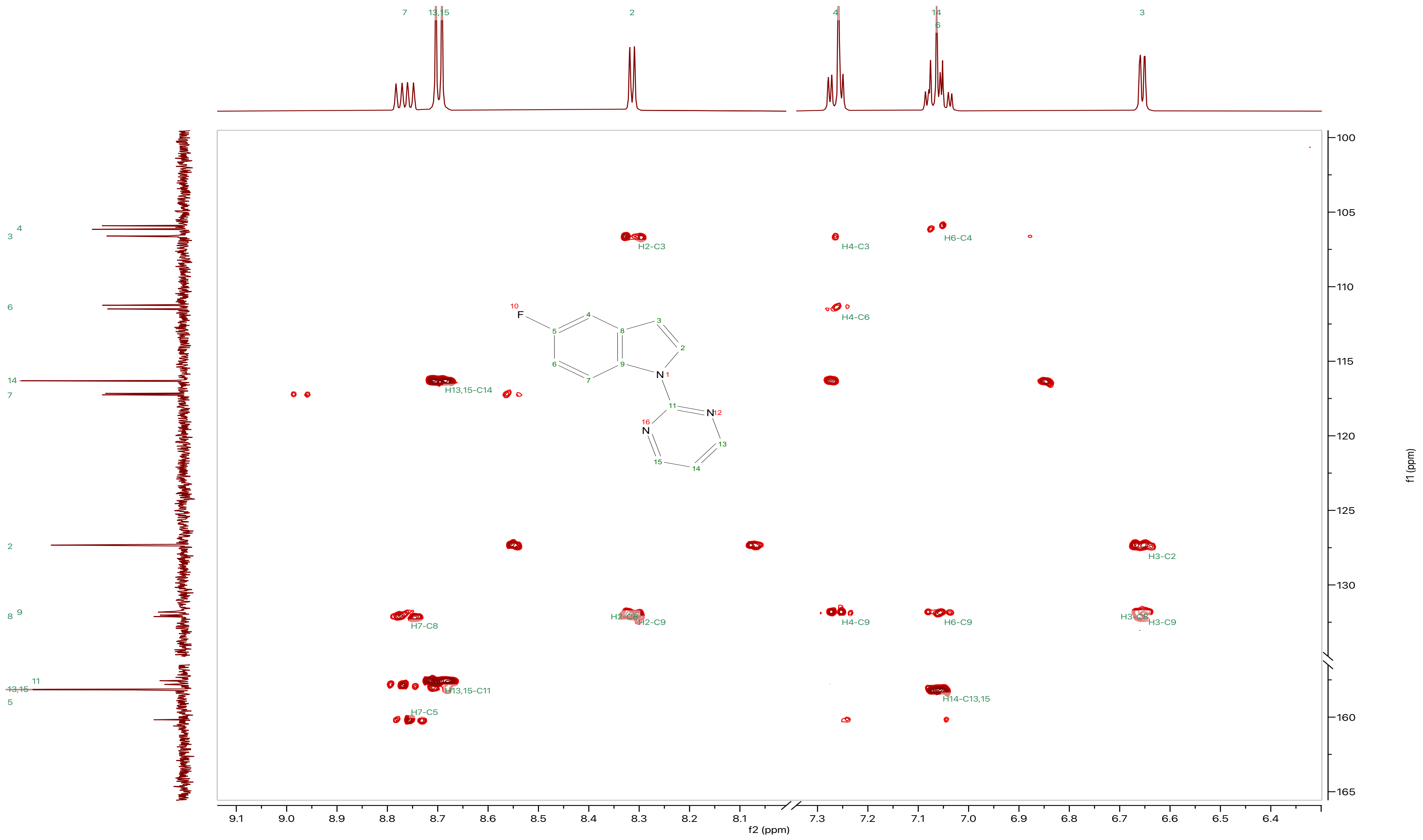

^{1}H-$^{13}C\{^{1}H\}$ HSQC NMR (400/101 MHz, $CDCl_3$) of 1y

2y

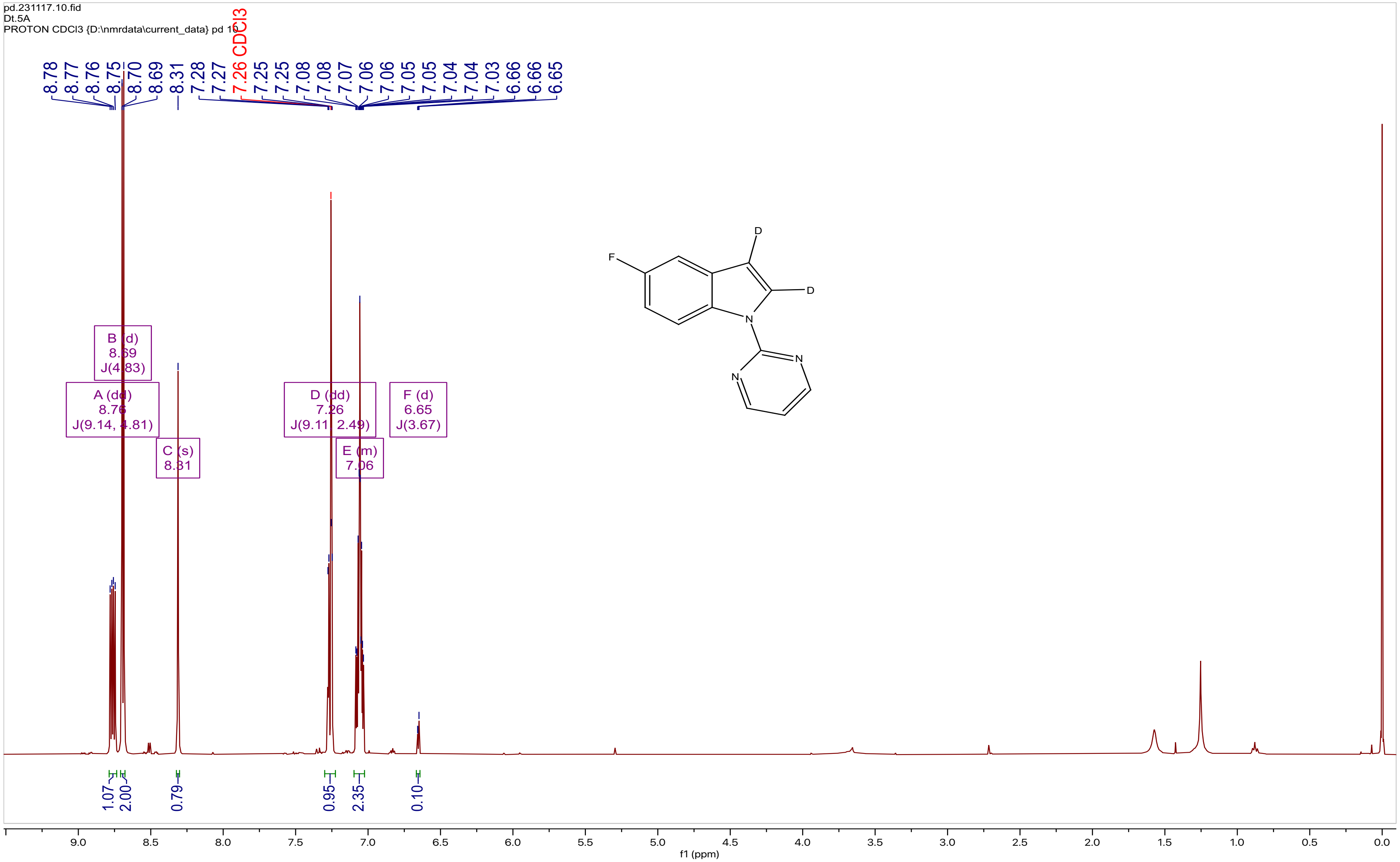

^{1}H NMR (400 MHz, $CDCl_3$) of 2y

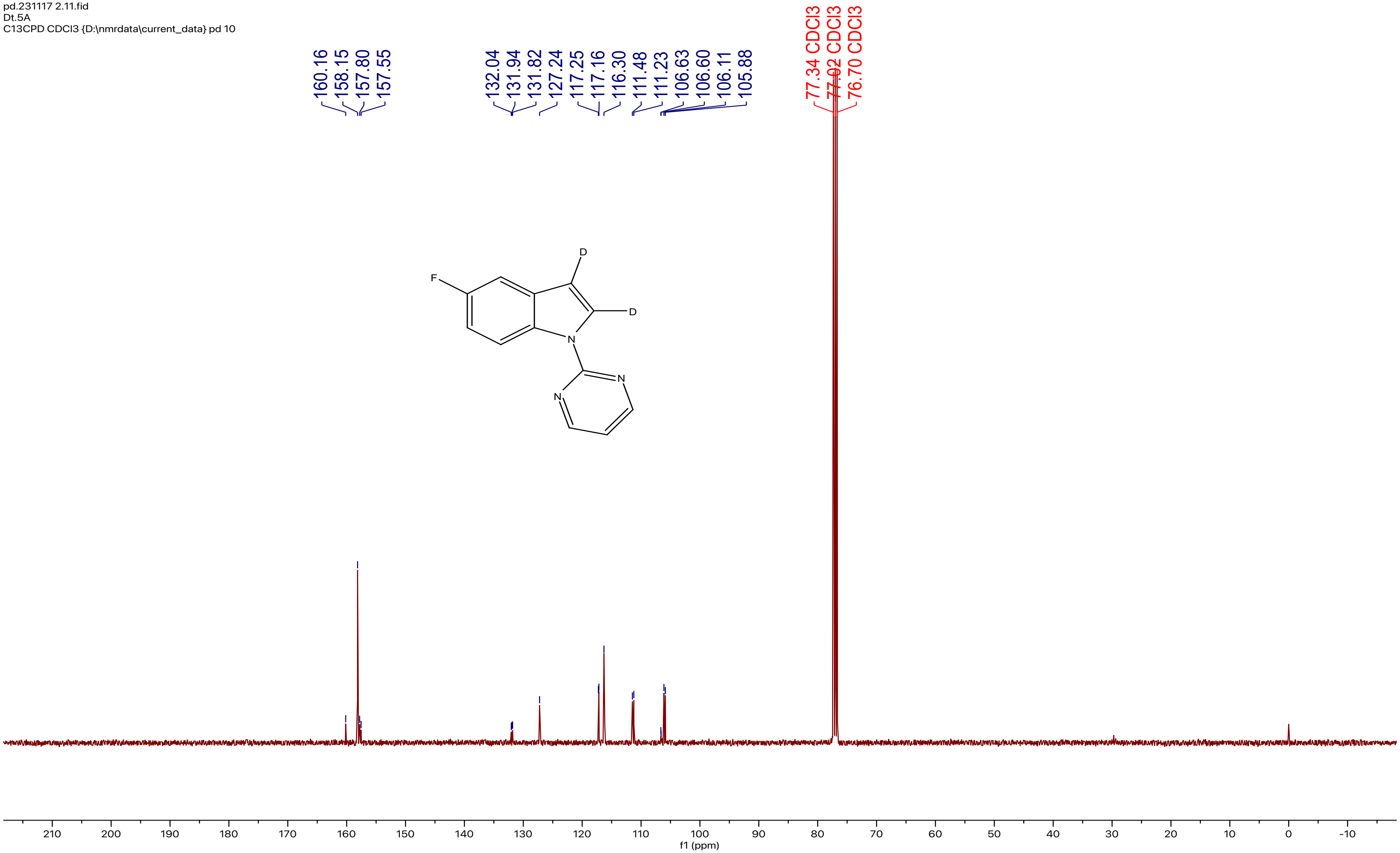

$^{13}C\{^1H\}$ NMR (101 MHz, $CDCl_3$) of 2y

1z

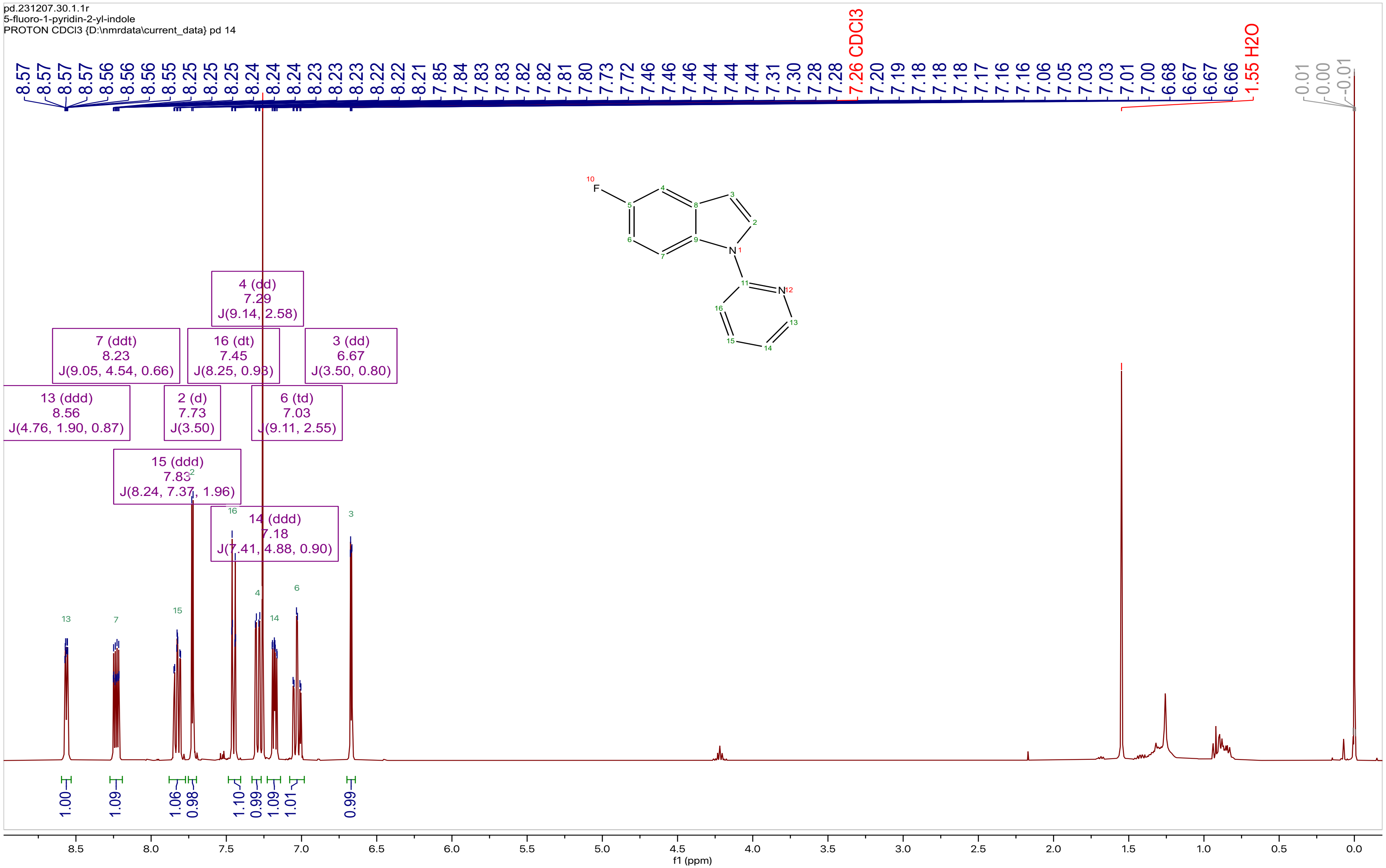

^{1}H NMR (400 MHz, $CDCl_3$) of 1z

pd.231207.31.1.1r
5-fluoro-1-pyridin-2-yl-indole
C13CPD CDCl3 {D:\nmrdata\current_data} pd 14

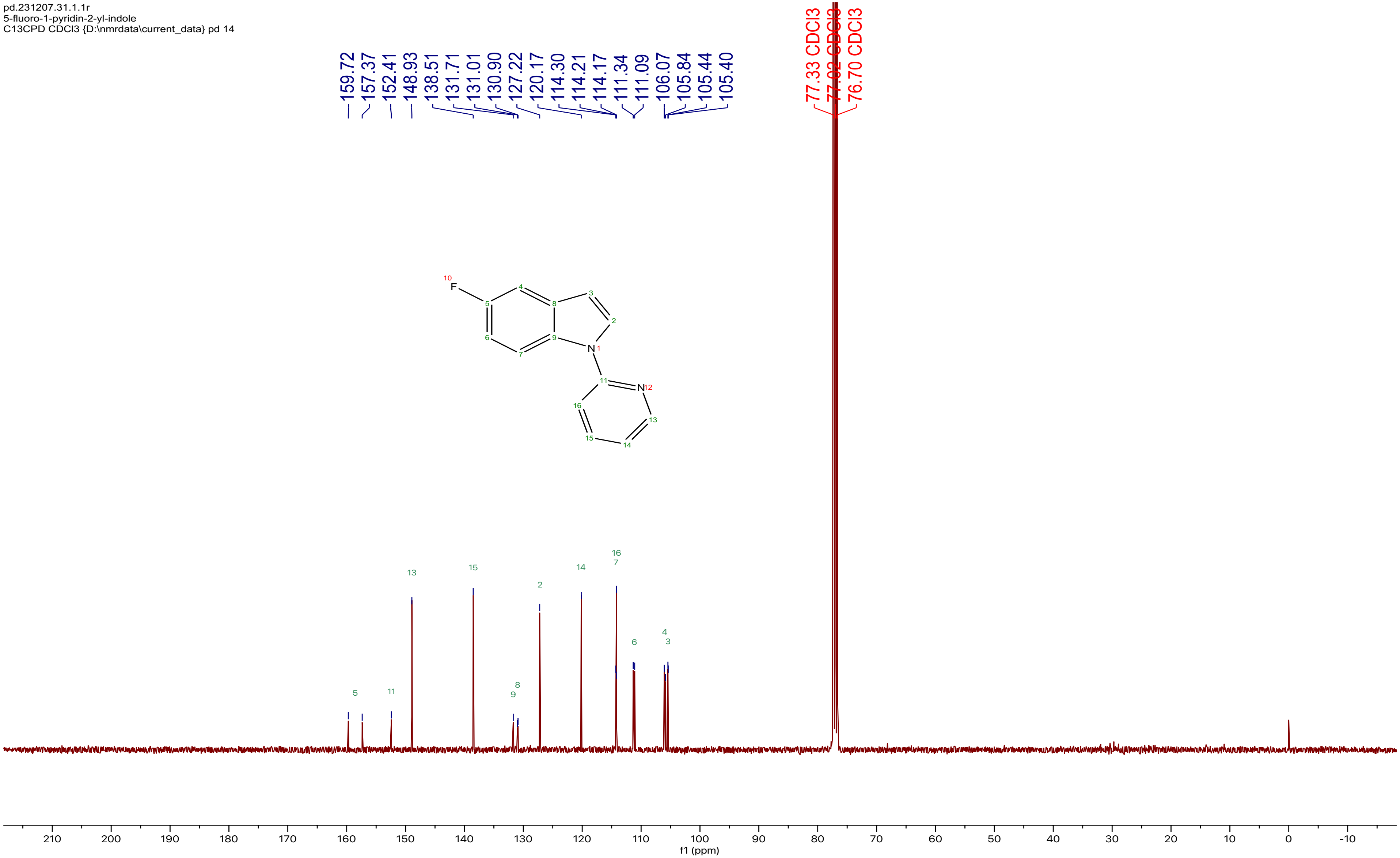

$^{13}C\{^{1}H\}$ NMR (101 MHz, $CDCl_3$) of 1z

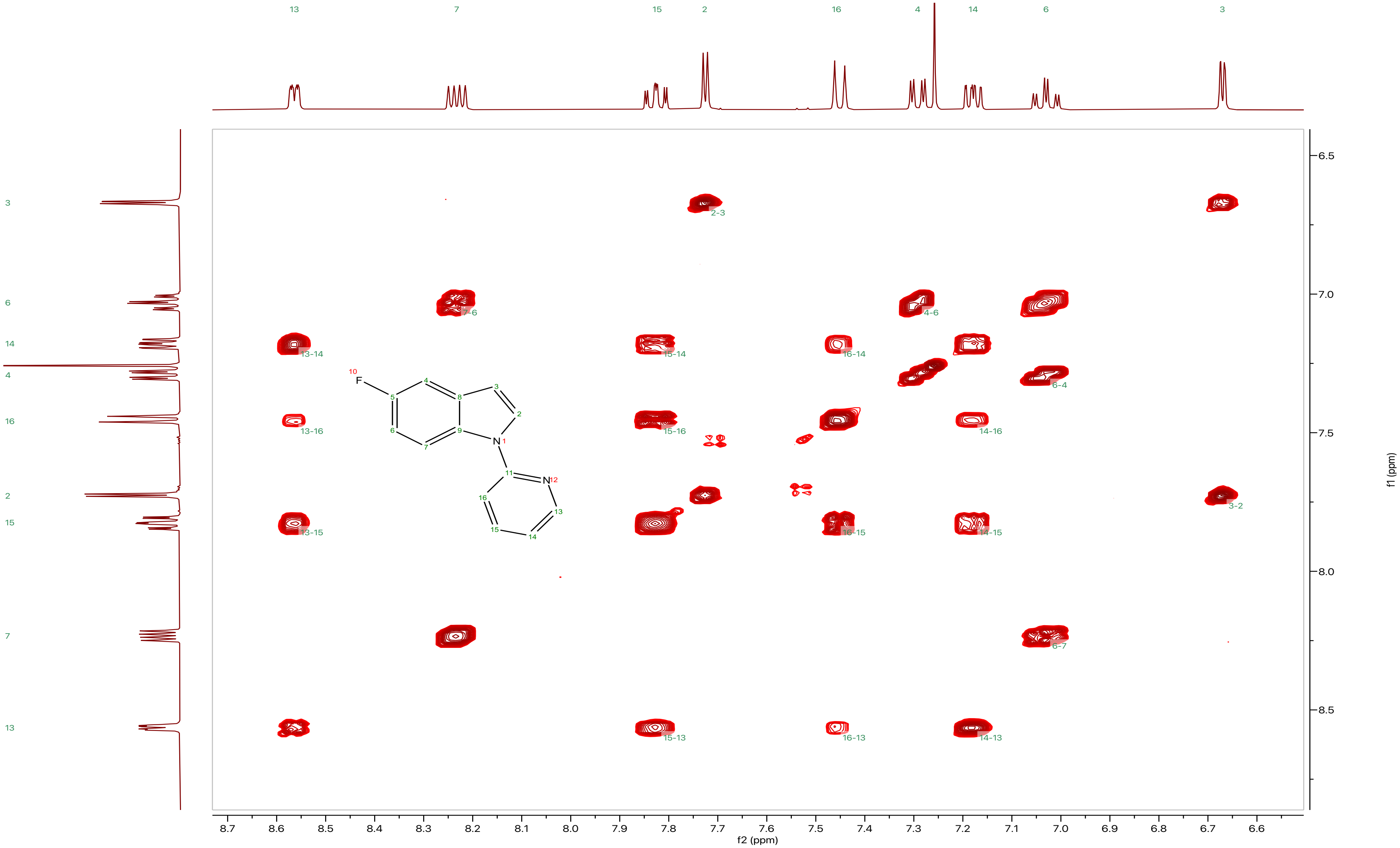

^{1}H-^{1}H COSY (400 MHz, $CDCl_3$) of 1z

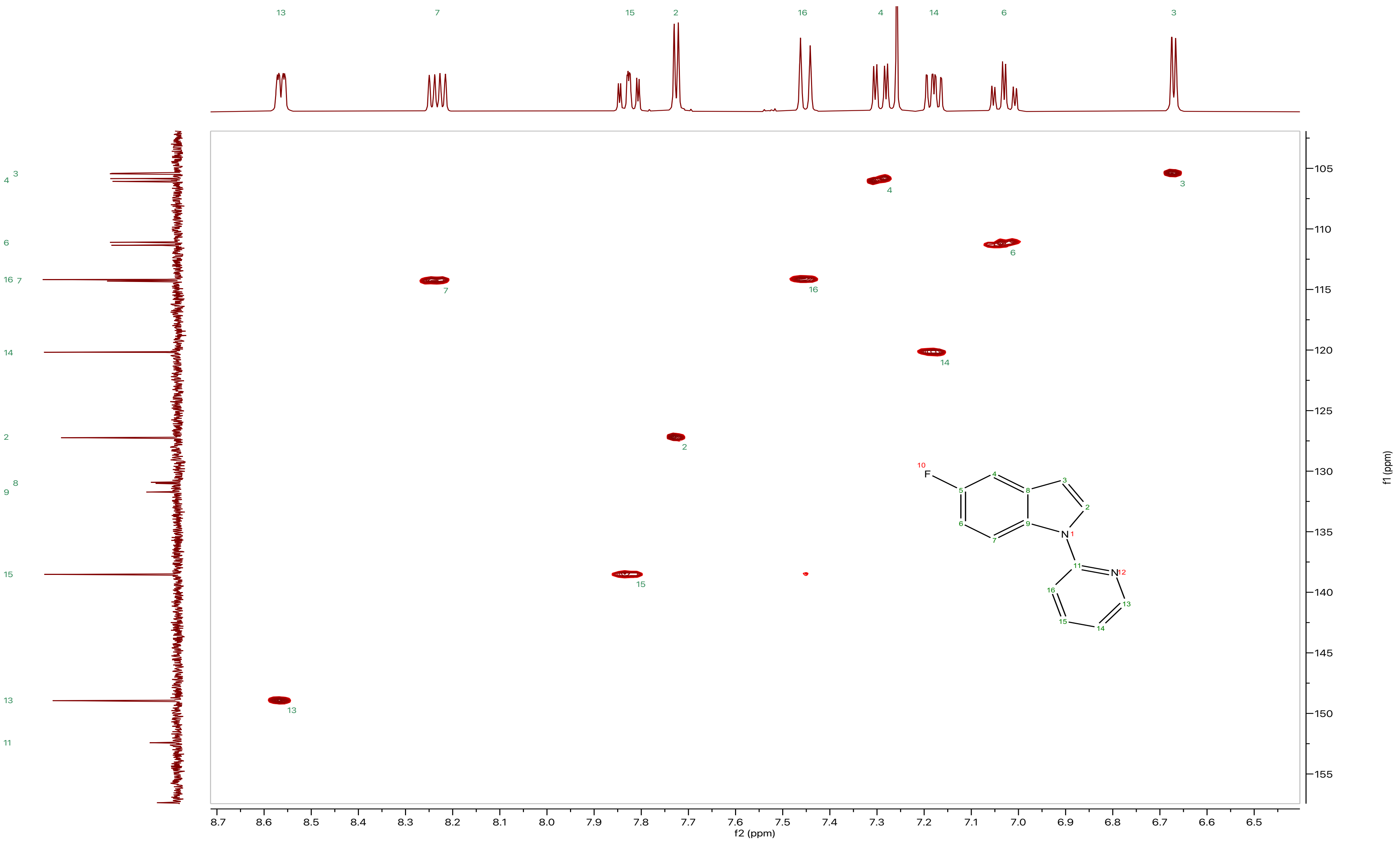

^{1}H-$^{13}C\{^{1}H\}$ HSQC NMR (400/101 MHz, $CDCl_3$) of 1z

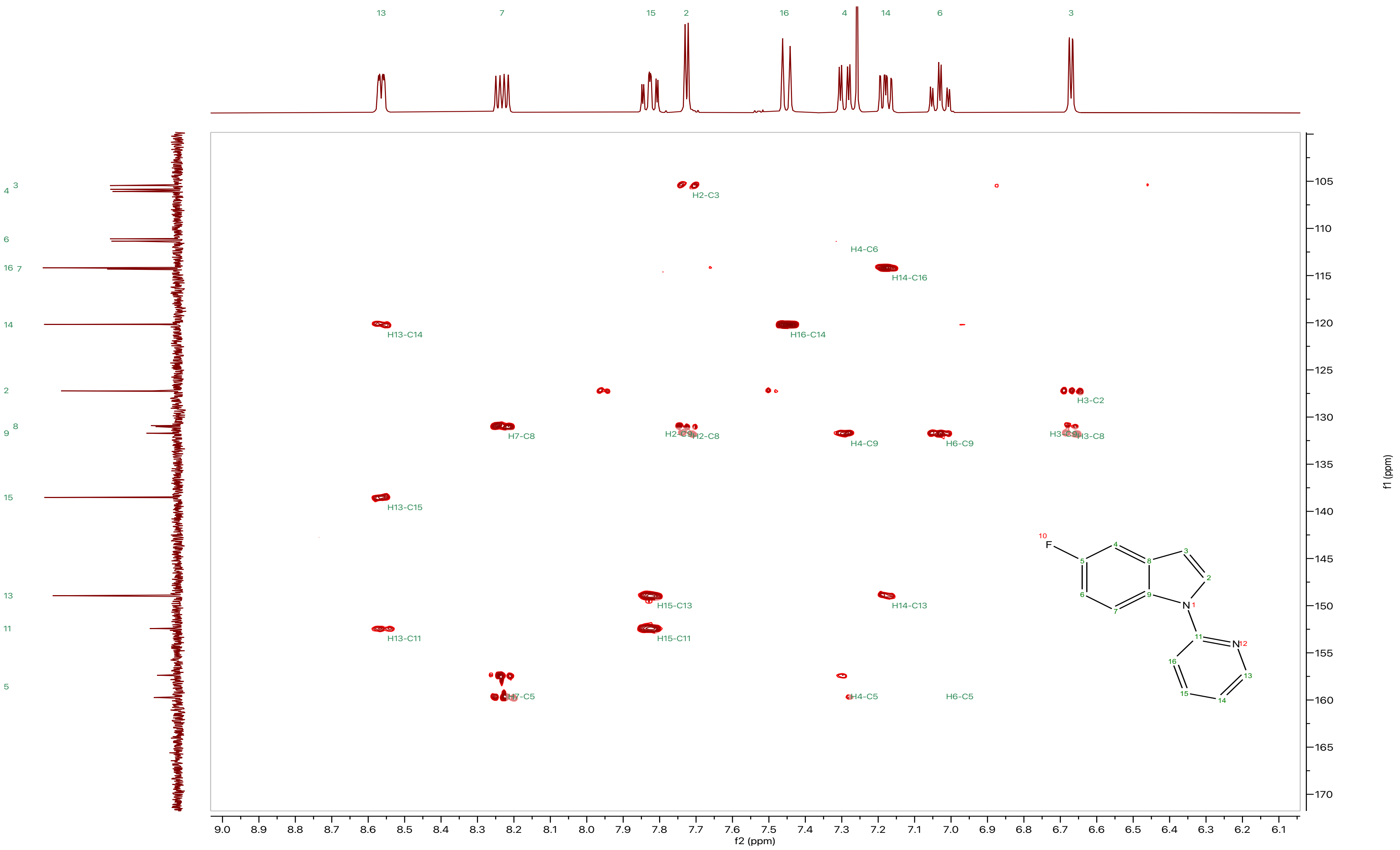

^{1}H-$^{13}C\{^{1}H\}$ HMBC NMR (400/101 MHz, $CDCl_3$) of 1z

2z

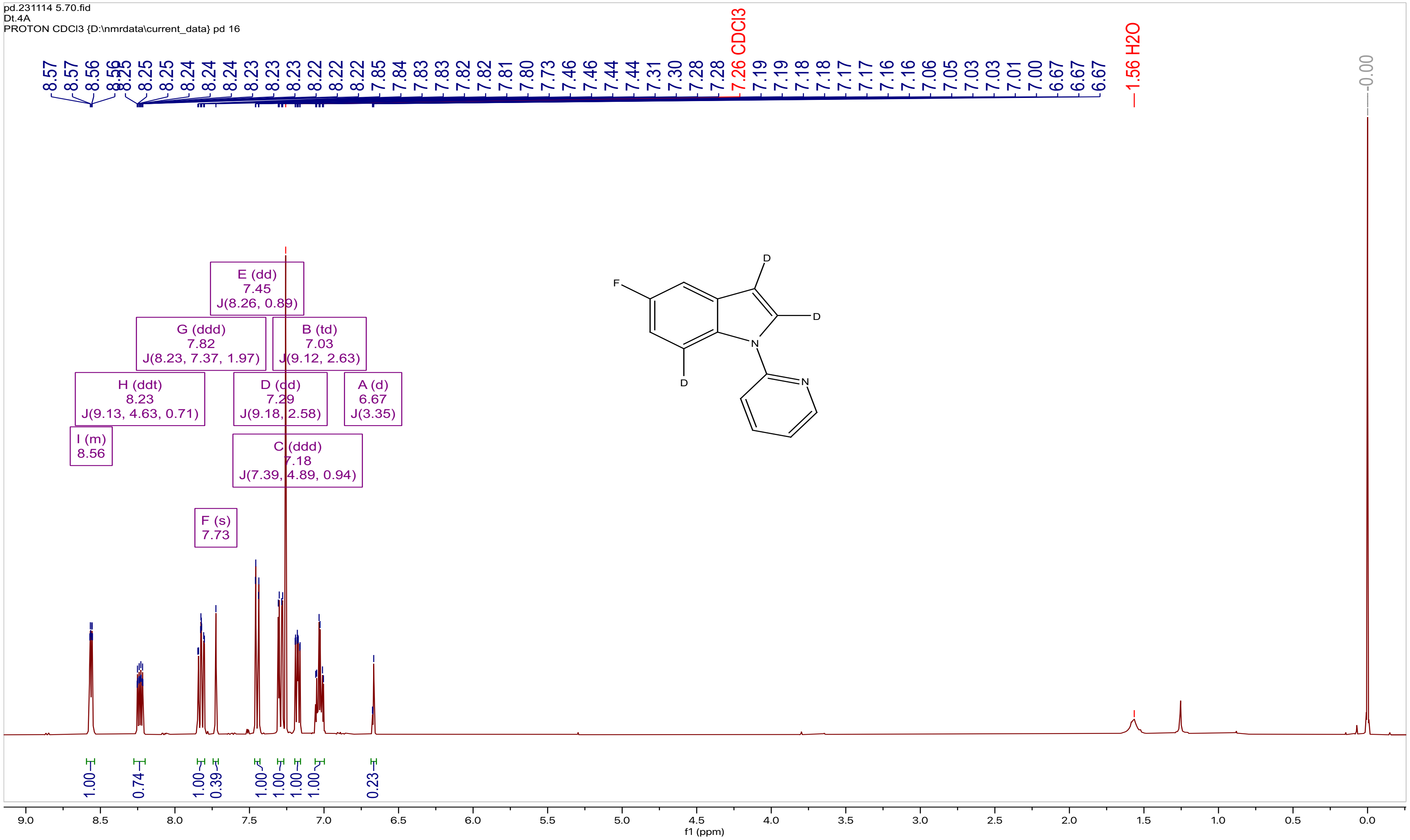

^{1}H NMR (400 MHz, $CDCl_3$) of 2z

pd.231114 11.71.fid
Dt.4A
C13CPD CDCl3 {D:\nmrdata\current_data} pd 16

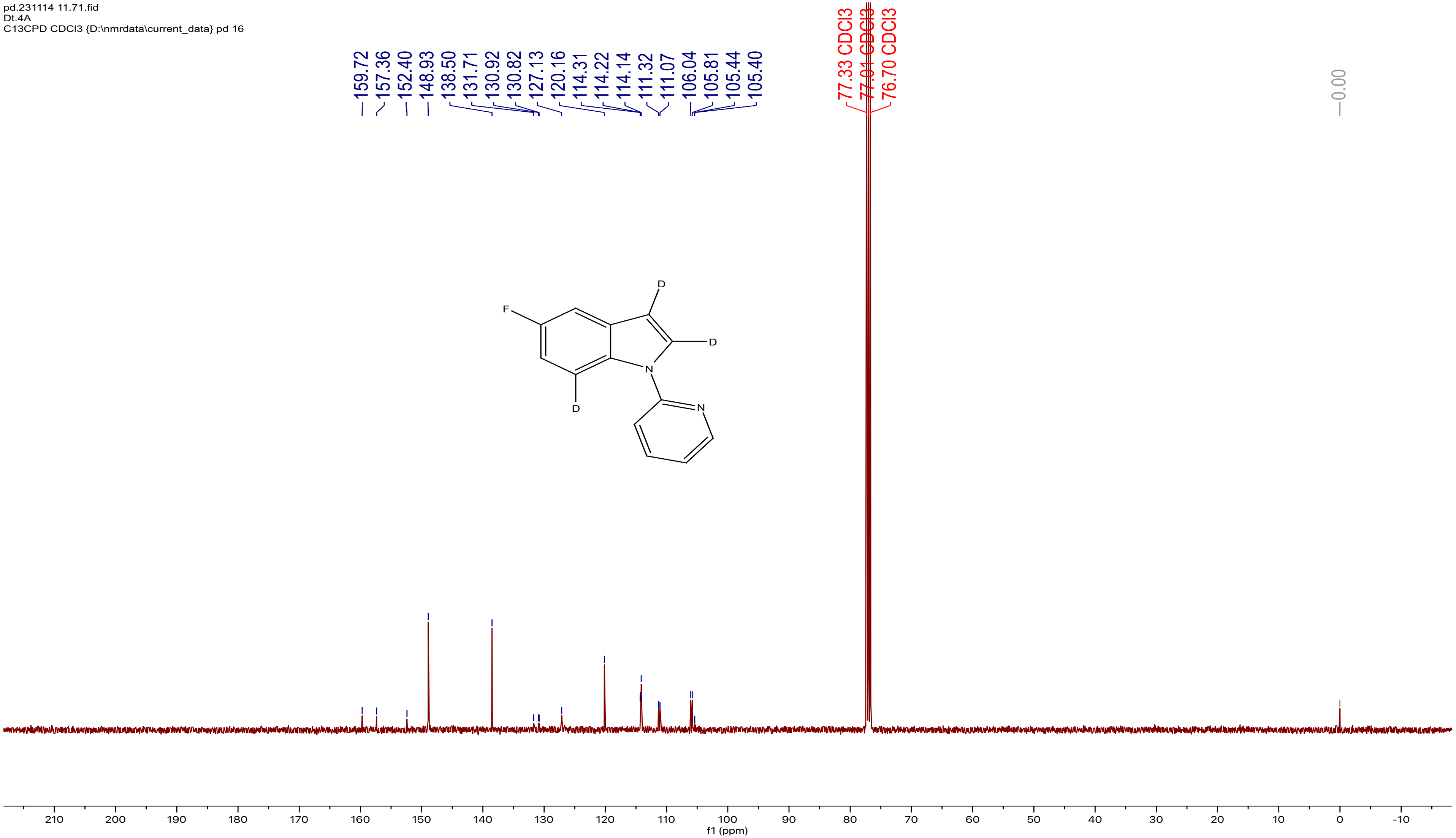

$^{13}C\{^1H\}$ NMR (101 MHz, $CDCl_3$) of 2z

1aa

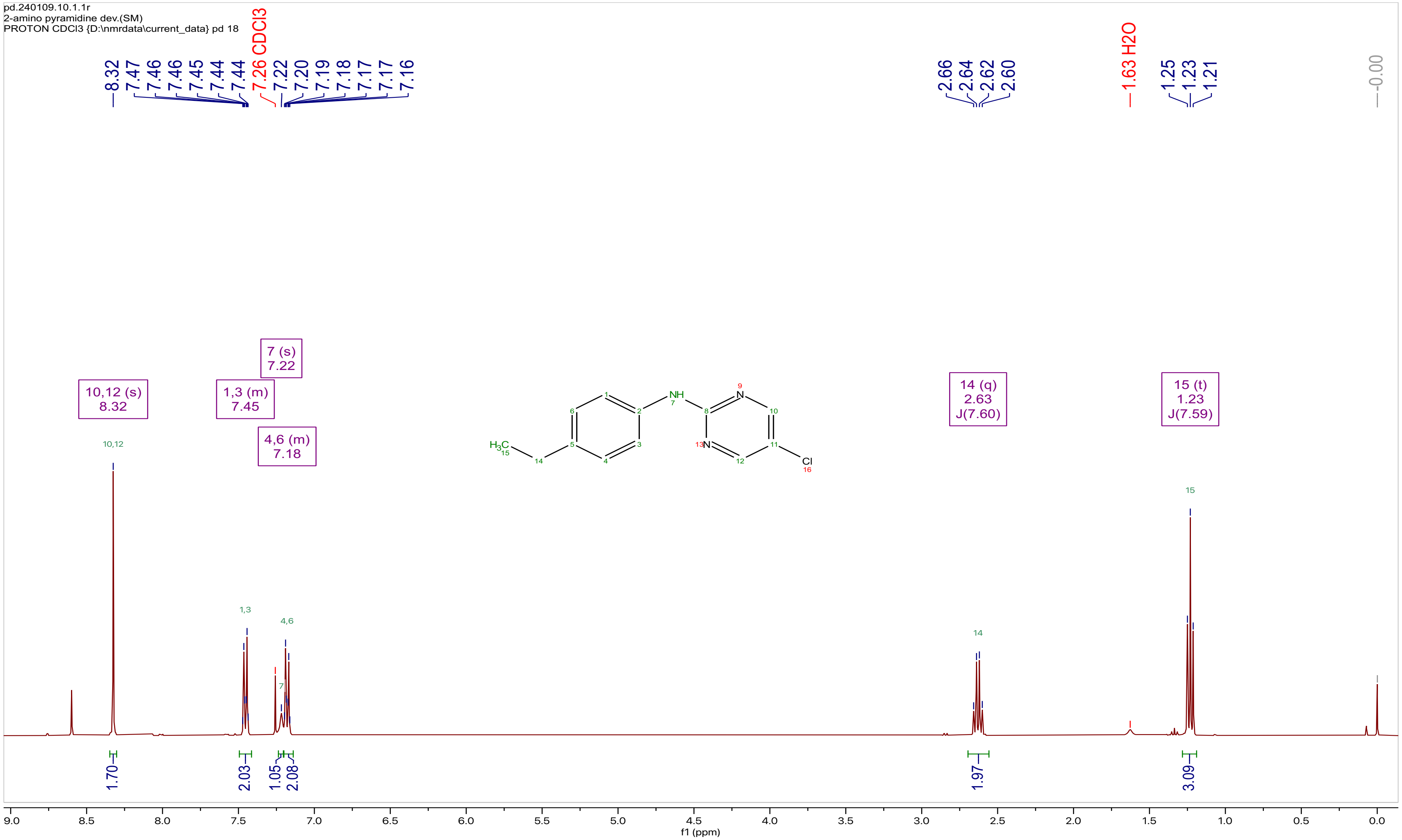

^{1}H NMR (400 MHz, $CDCl_3$) of 1aa

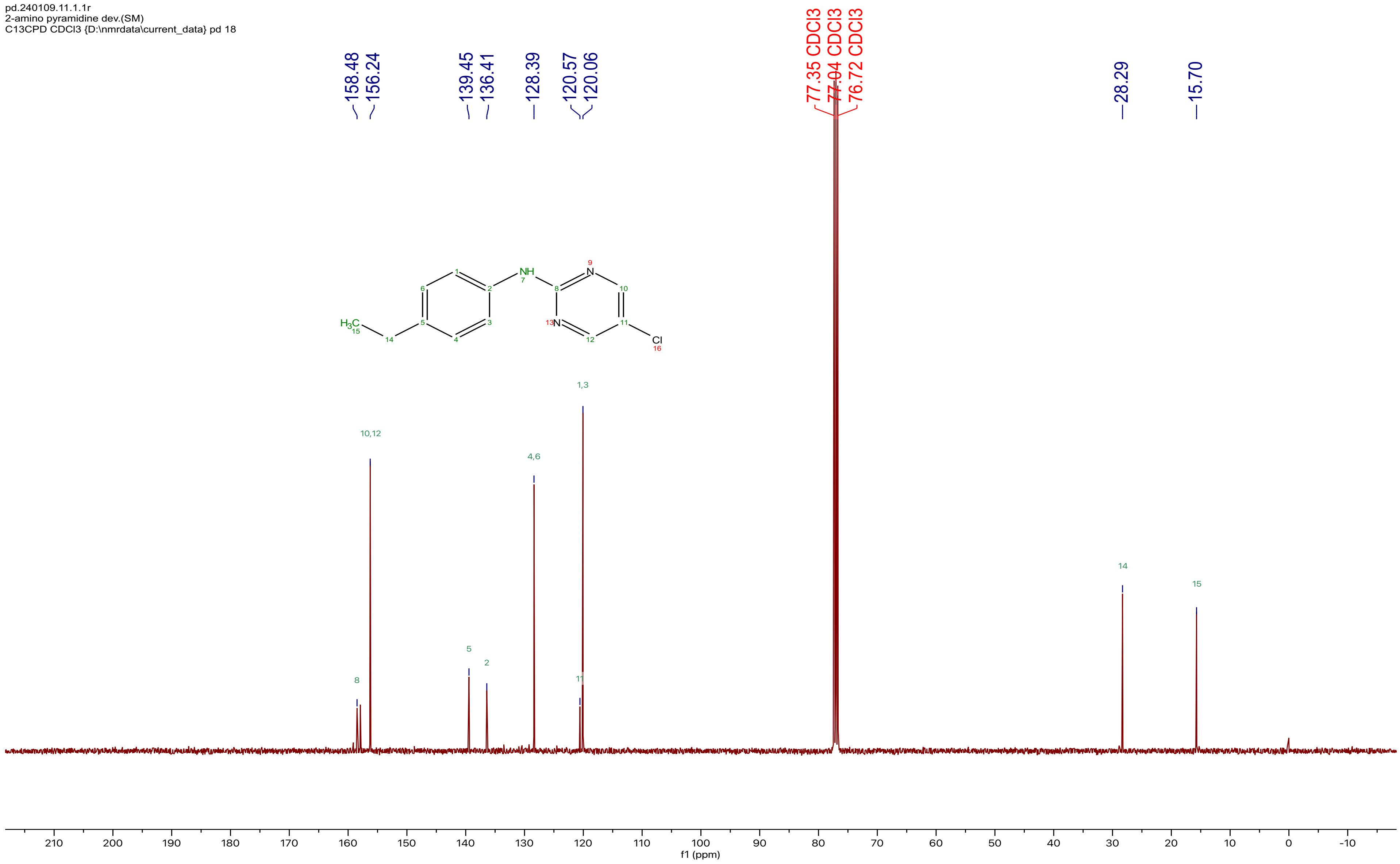

$^{13}C\{^1H\}$ NMR (101 MHz, $CDCl_3$) of 1aa

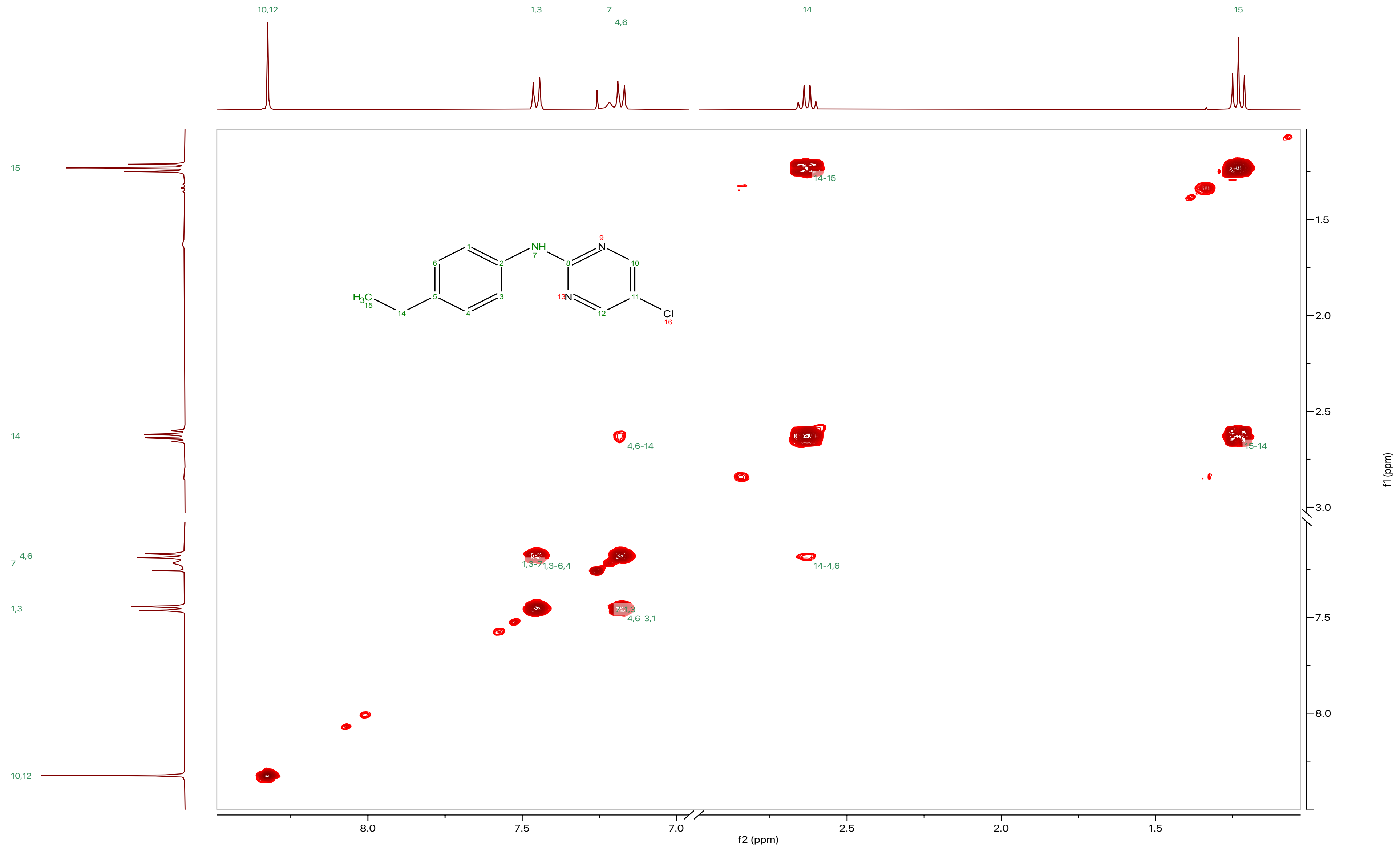

^1H-^1H COSY (400 MHz, $CDCl_3$) of 1aa

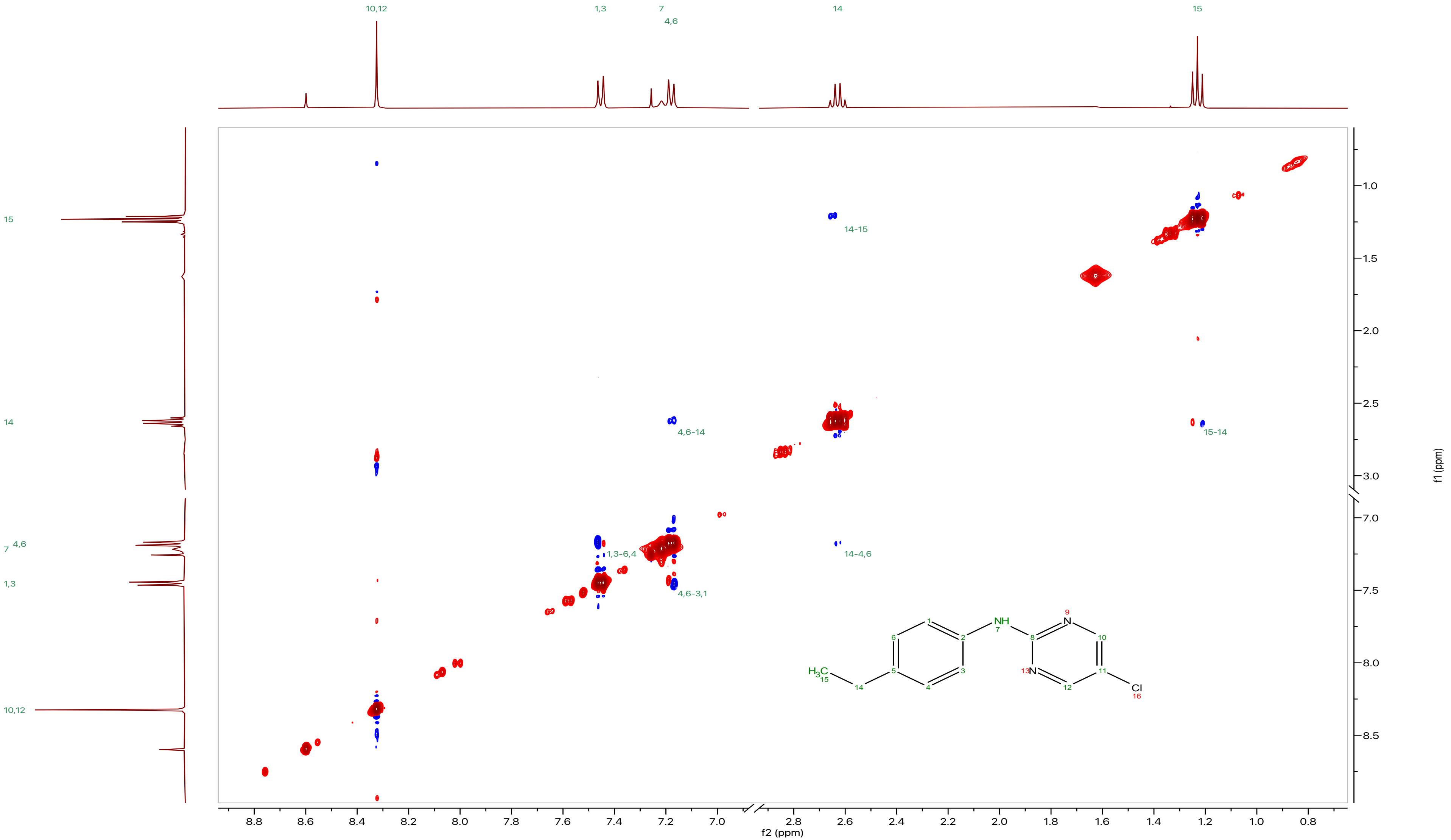

^1H-^1H NOESY (400 MHz, $CDCl_3$) of 1aa

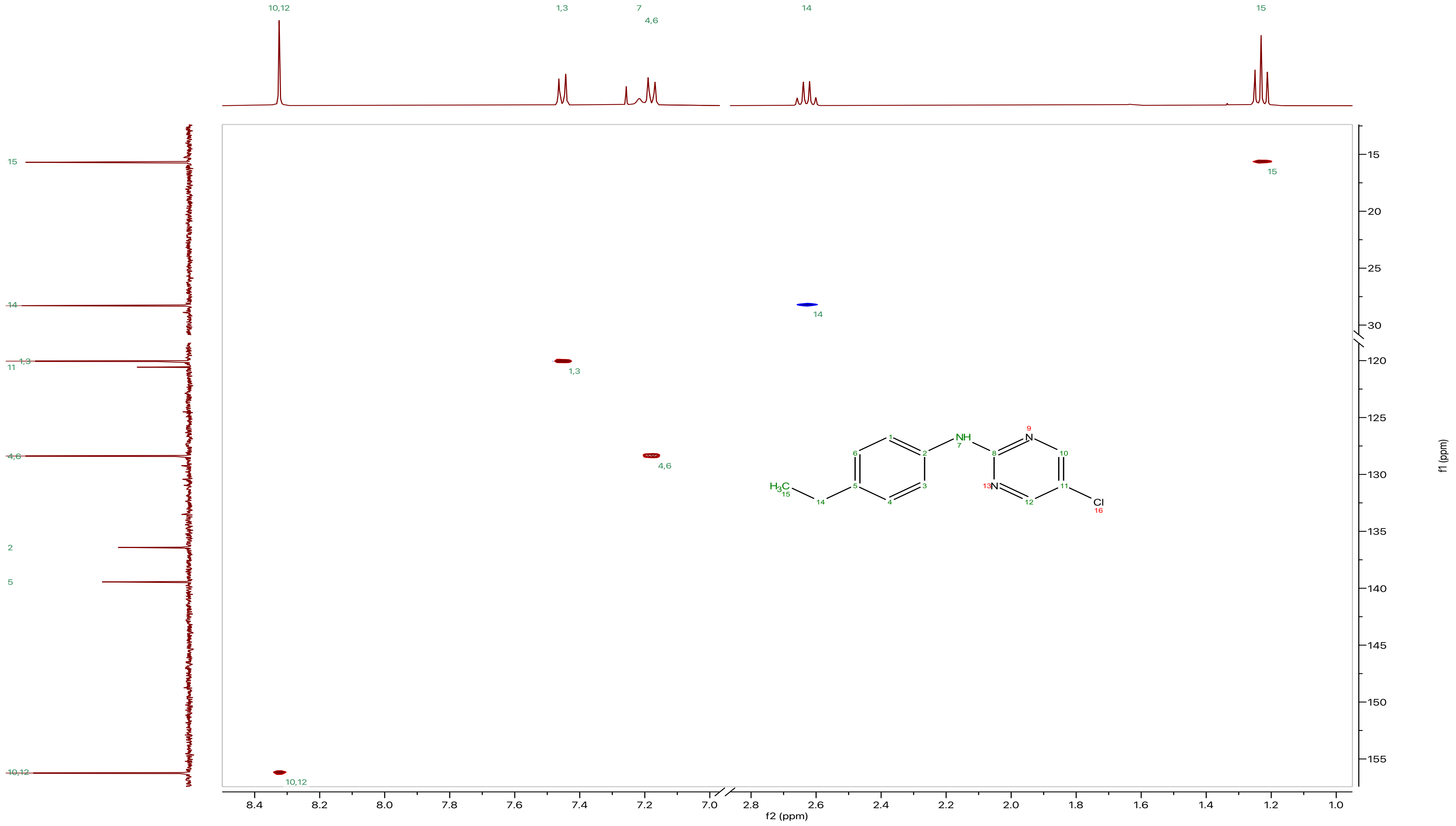

^{1}H-^{13}C{^{1}H} HSQC NMR (400/101 MHz, $CDCl_3$) of 1aa

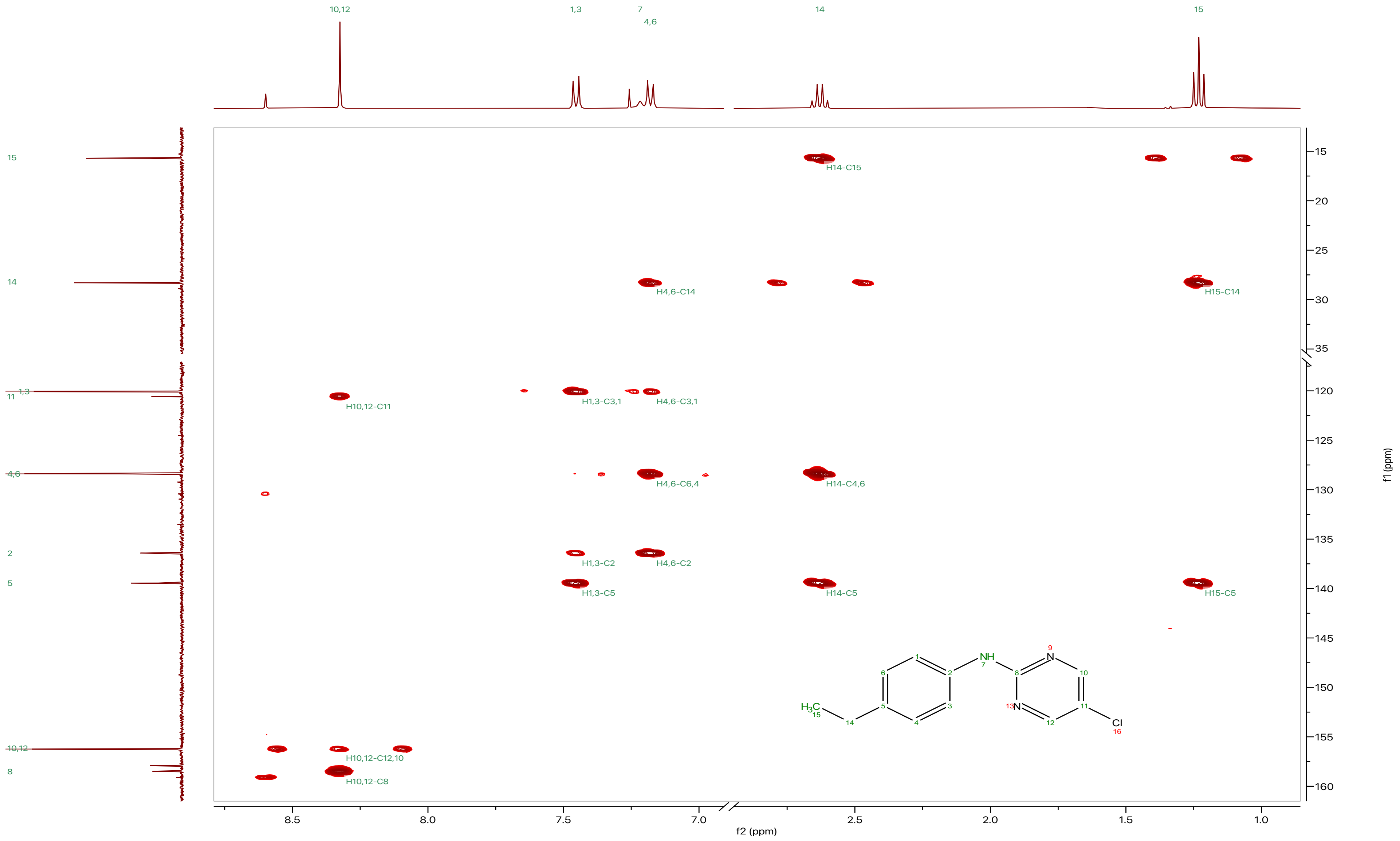

^{1}H-$^{13}C\{^{1}H\}$ HMBC NMR (400/101 MHz, $CDCl_3$) of 1aa

2aa

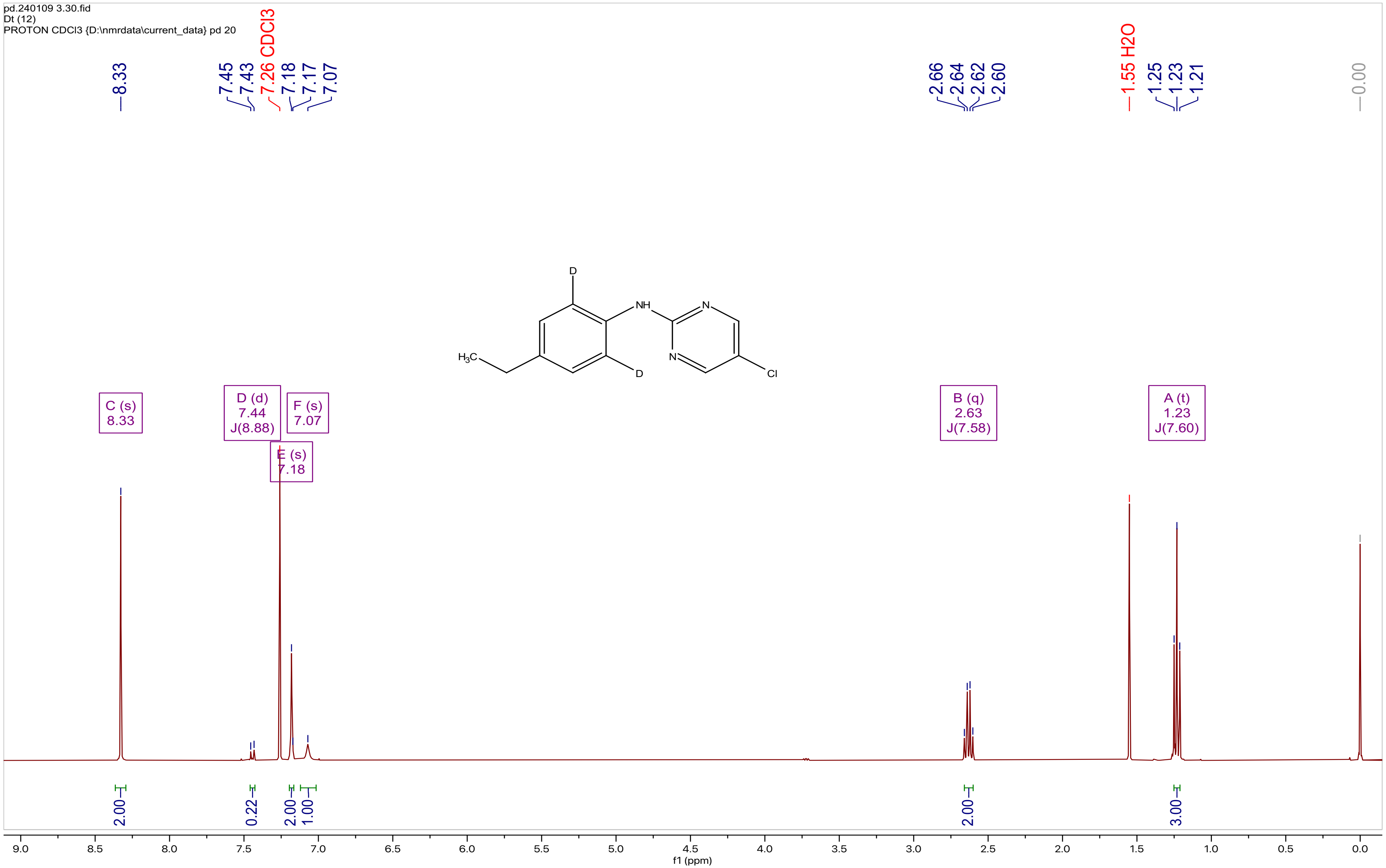

^{1}H NMR (400 MHz, $CDCl_3$) of 2aa

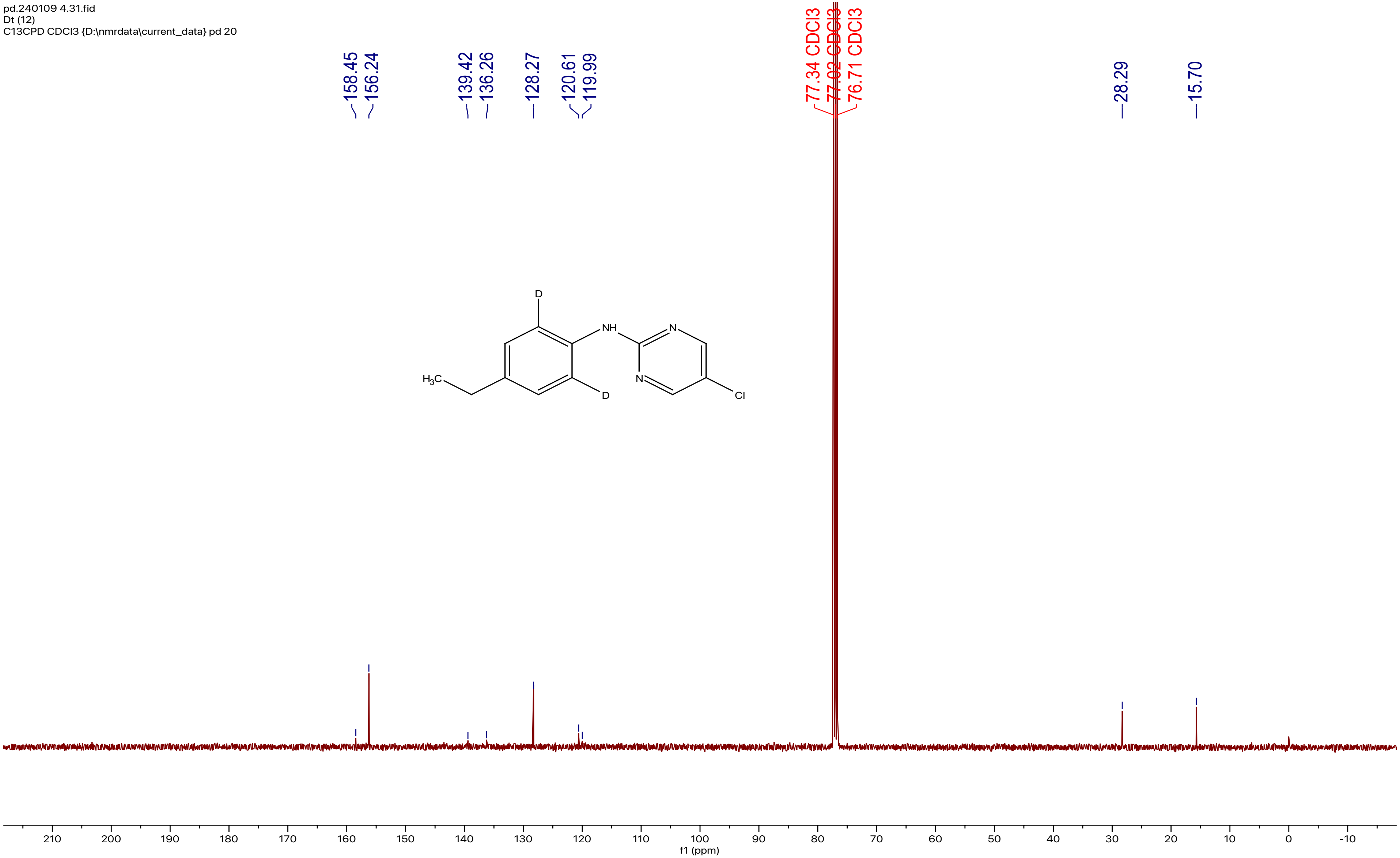

$^{13}C\{^{1}H\}$ **NMR (101 MHz, $CDCl_3$) of 2aa**

1’’

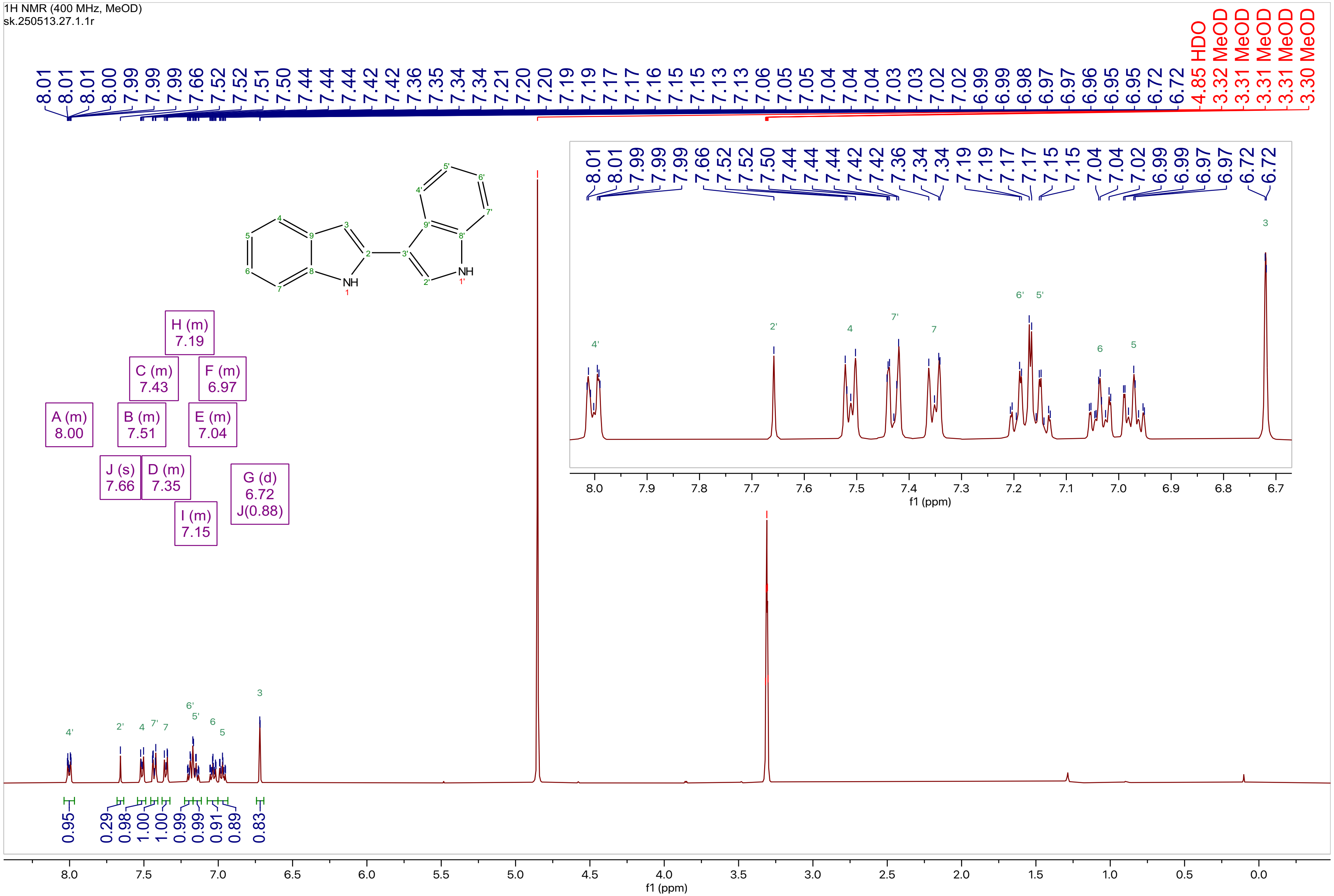

^{1}H NMR (400 MHz, MeOD) of 1’’

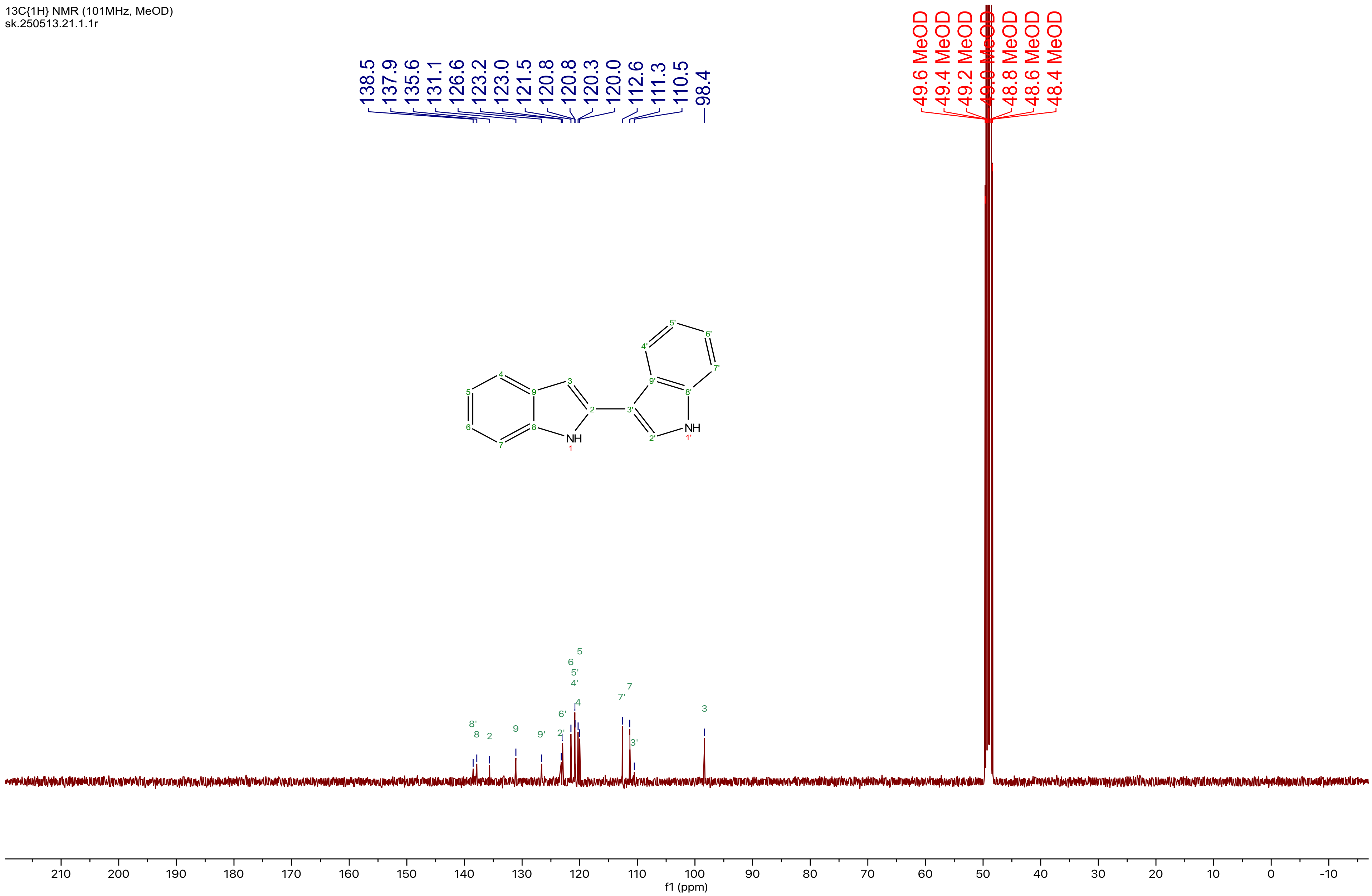
13C{1H} NMR (101MHz, MeOD)
sk.250513.21.1.1r
138.5
137.9
135.6
131.1
126.6
123.2
123.0
121.5
120.8
120.8
120.3
120.0
112.6
111.3
110.5
98.4
49.6 MeOD
49.4 MeOD
49.2 MeOD
49.0 MeOD
48.8 MeOD
48.6 MeOD
48.4 MeOD
NH
NH
f1 (ppm)

$^{13}C\{^{1}H\}$ NMR (101 MHz, MeOD) of 1''

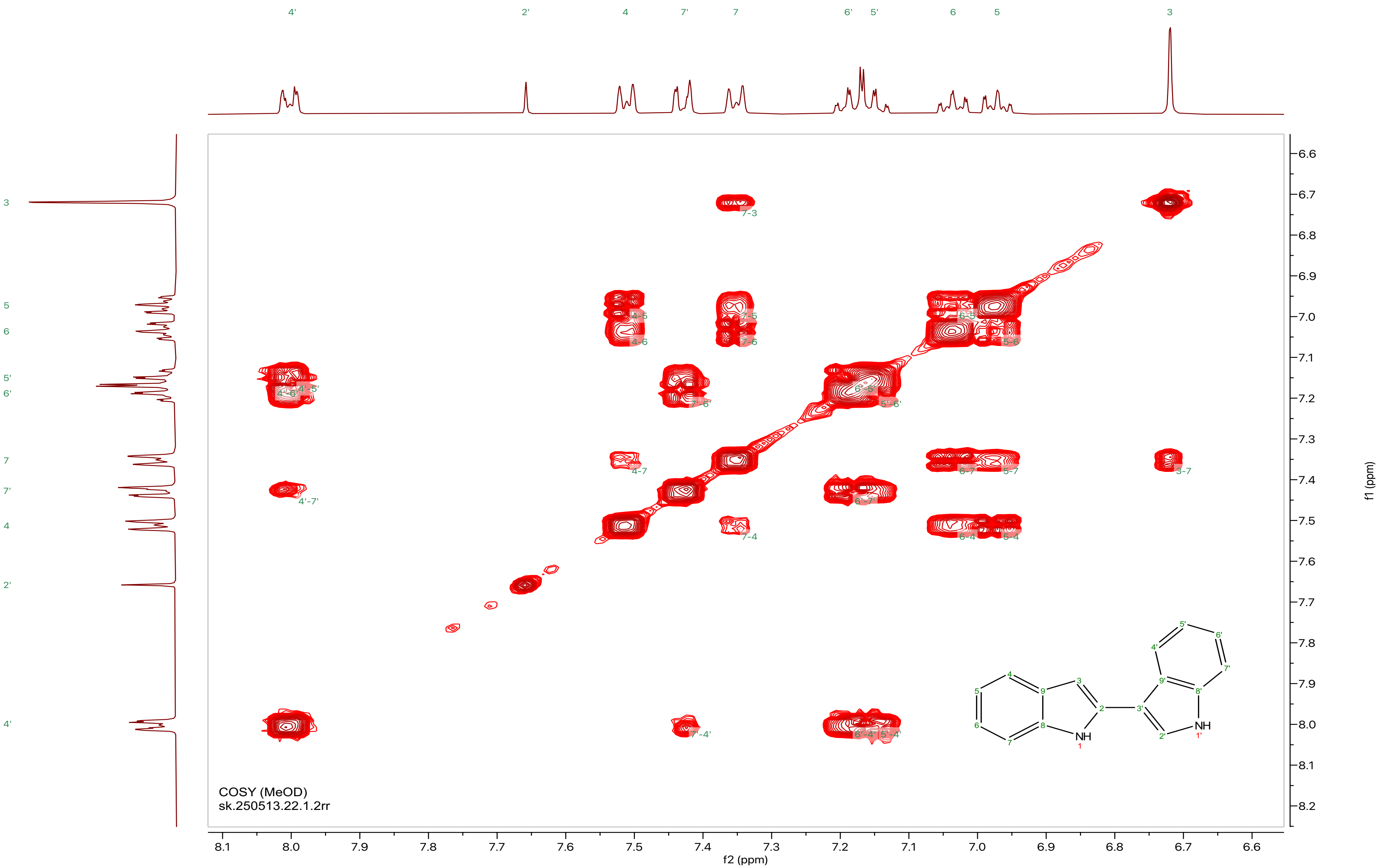

^{1}H-^{1}H COSY (400 MHz, MeOD) of 1''

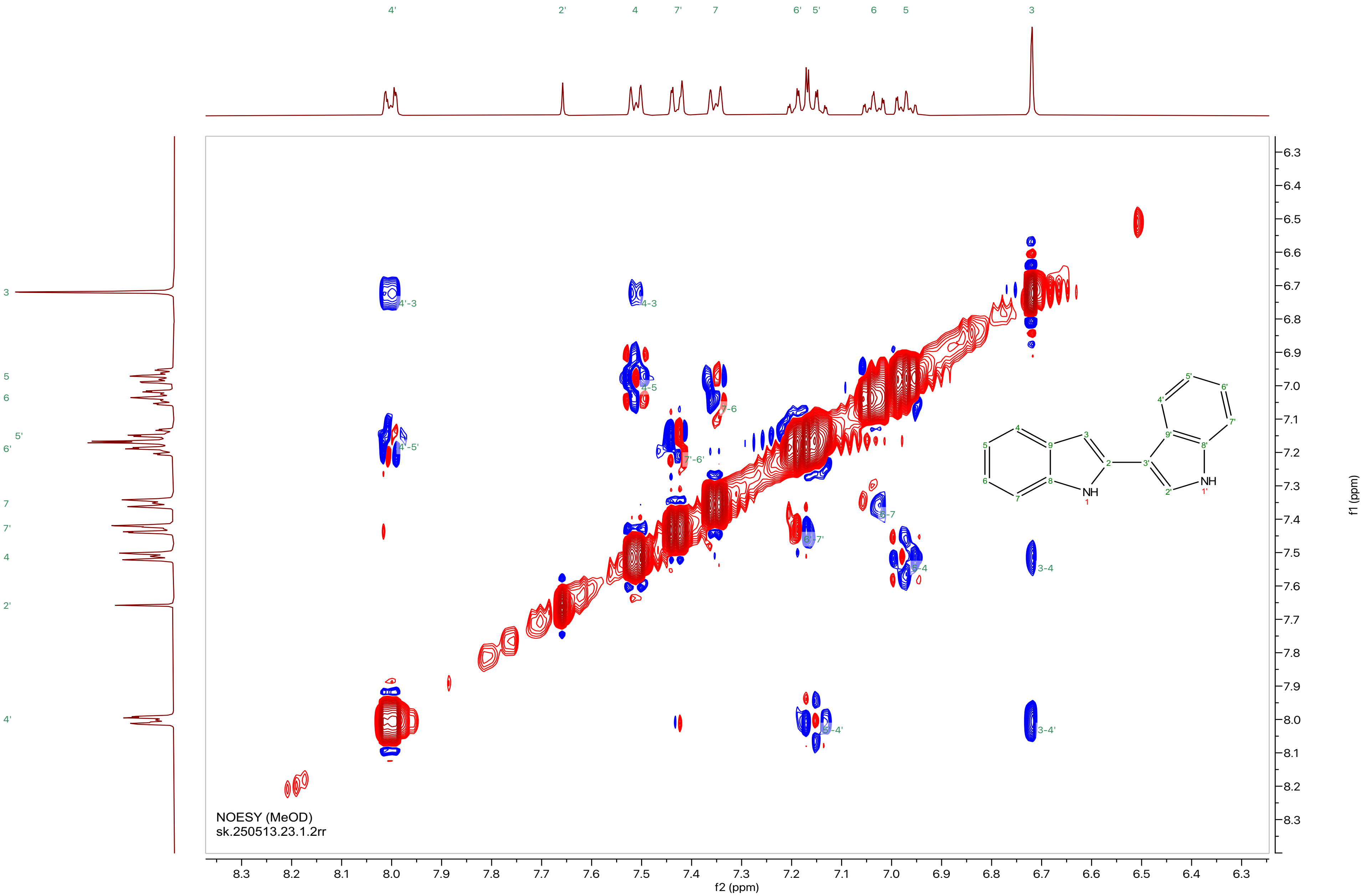
NOESY (MeOD)
sk.250513.23.1.2rr
f2 (ppm)
f1 (ppm)
4'-3
4-3
4-5
7-6
4'-5'
7'-6'
6-7
6'-7'
5-4
3-4
5'-4'
3-4'
NH
NH

^{1}H-^{1}H NOESY (400 MHz, MeOD) of 1''

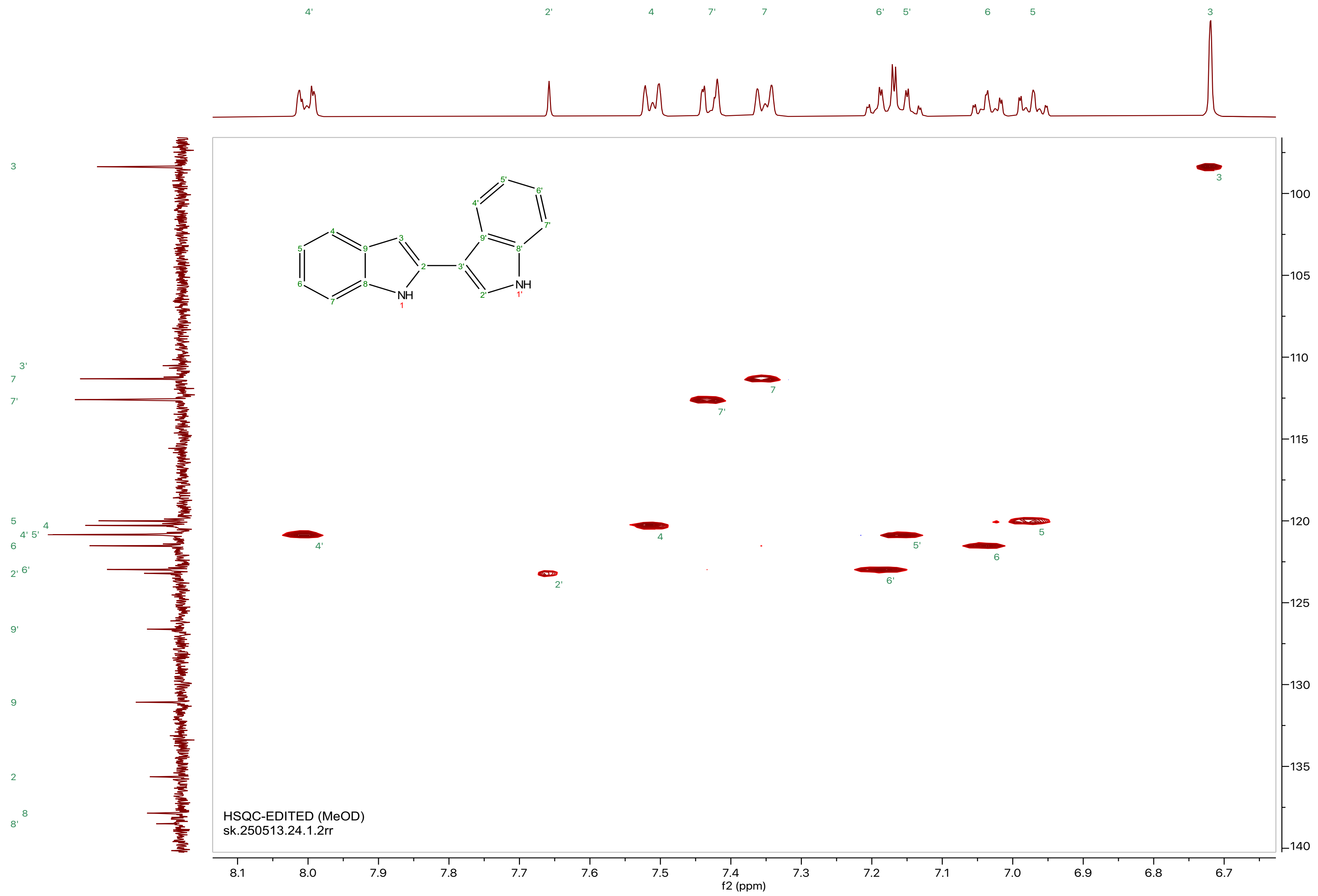

^{1}H-$^{13}C\{^{1}H\}$ HSQC NMR (400/101 MHz, MeOD) of 1''

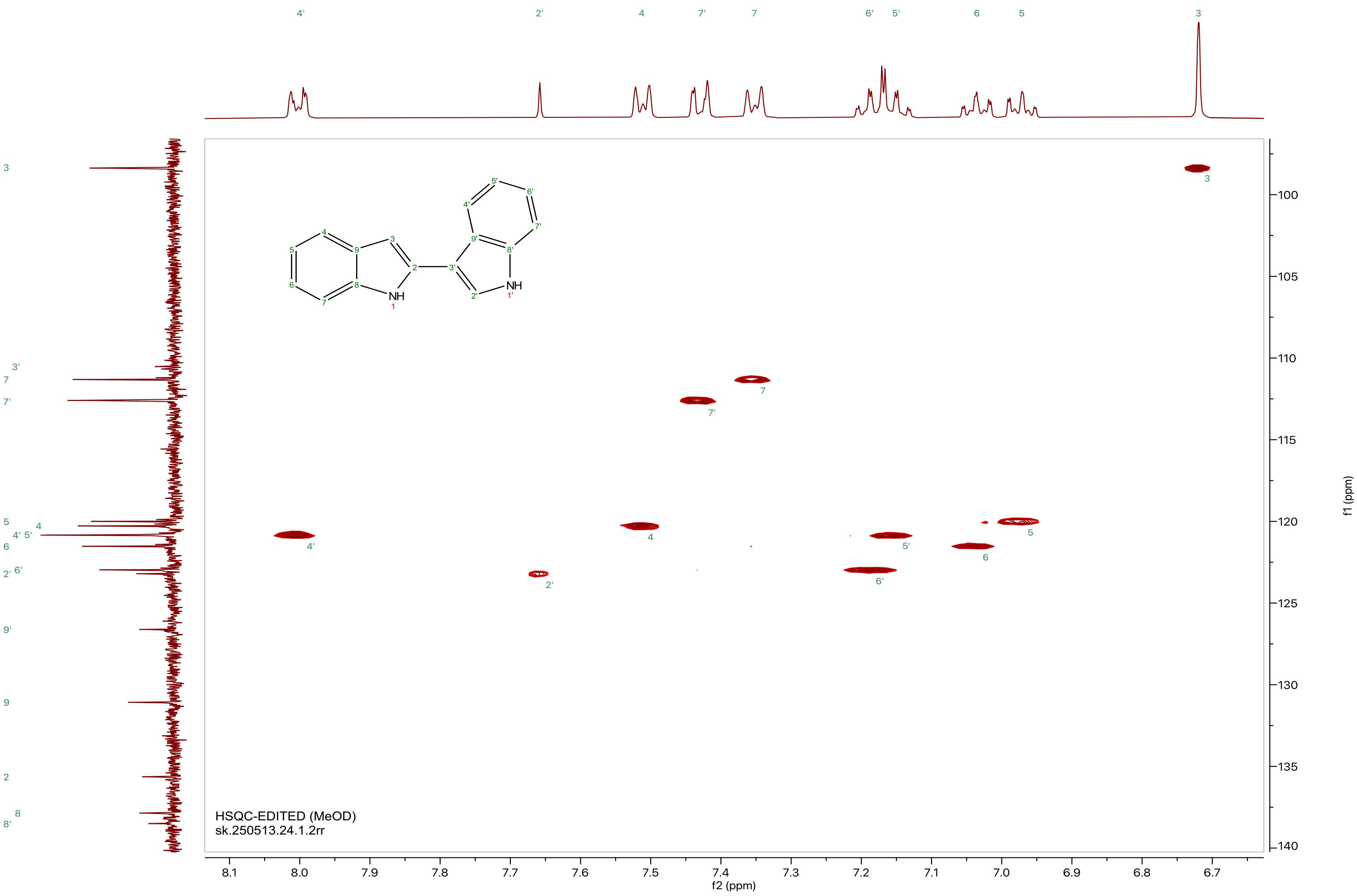

^{1}H-$^{13}C\{^{1}H\}$ HMBC NMR (400/101 MHz, MeOD) of 1’’

3a

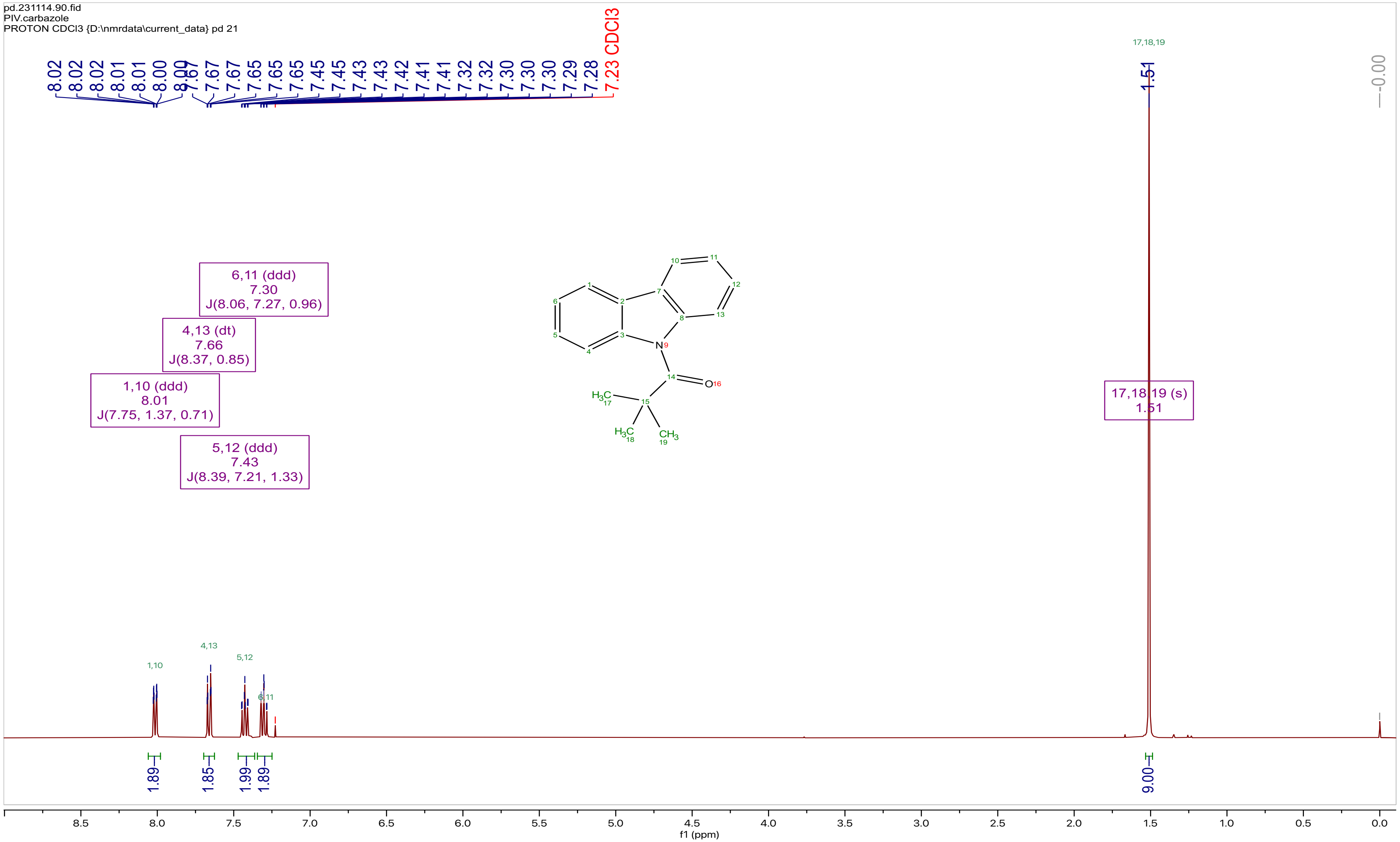

^{1}H NMR (400 MHz, $CDCl_3$) of 3a

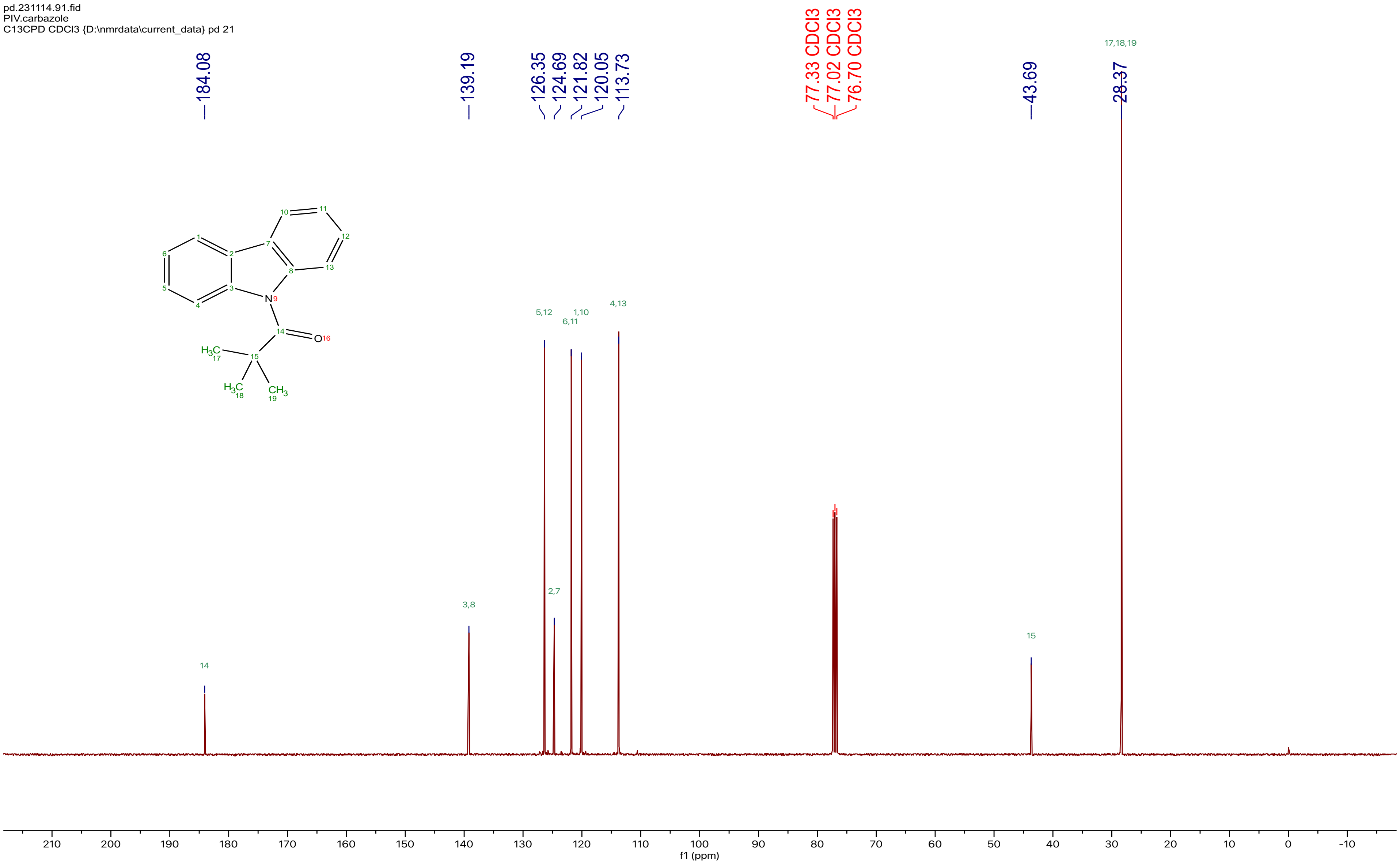

$^{13}C\{^1H\}$ NMR (101 MHz, $CDCl_3$) of 3a

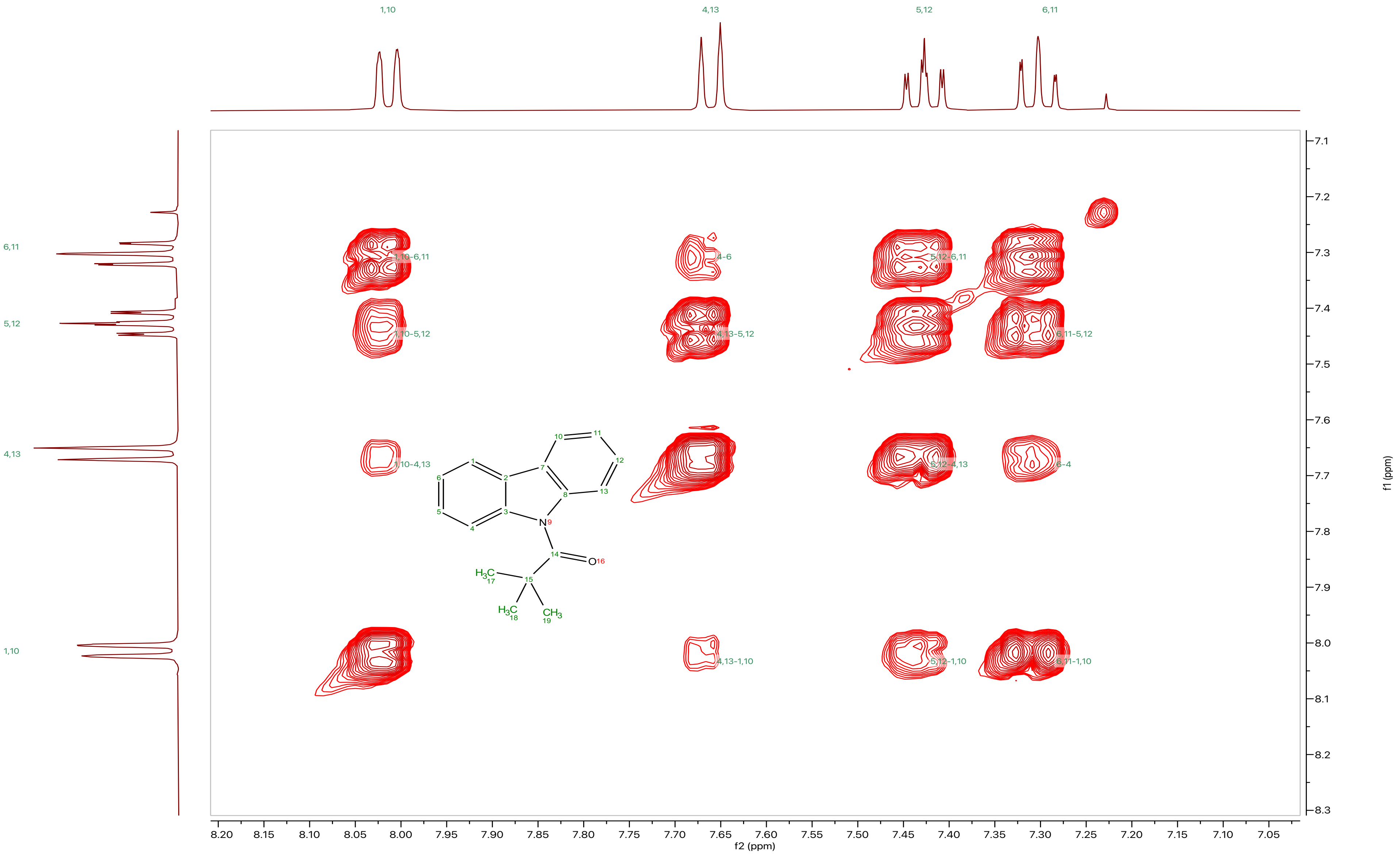

^{1}H-^{1}H COSY (400 MHz, $CDCl_3$) of 3a

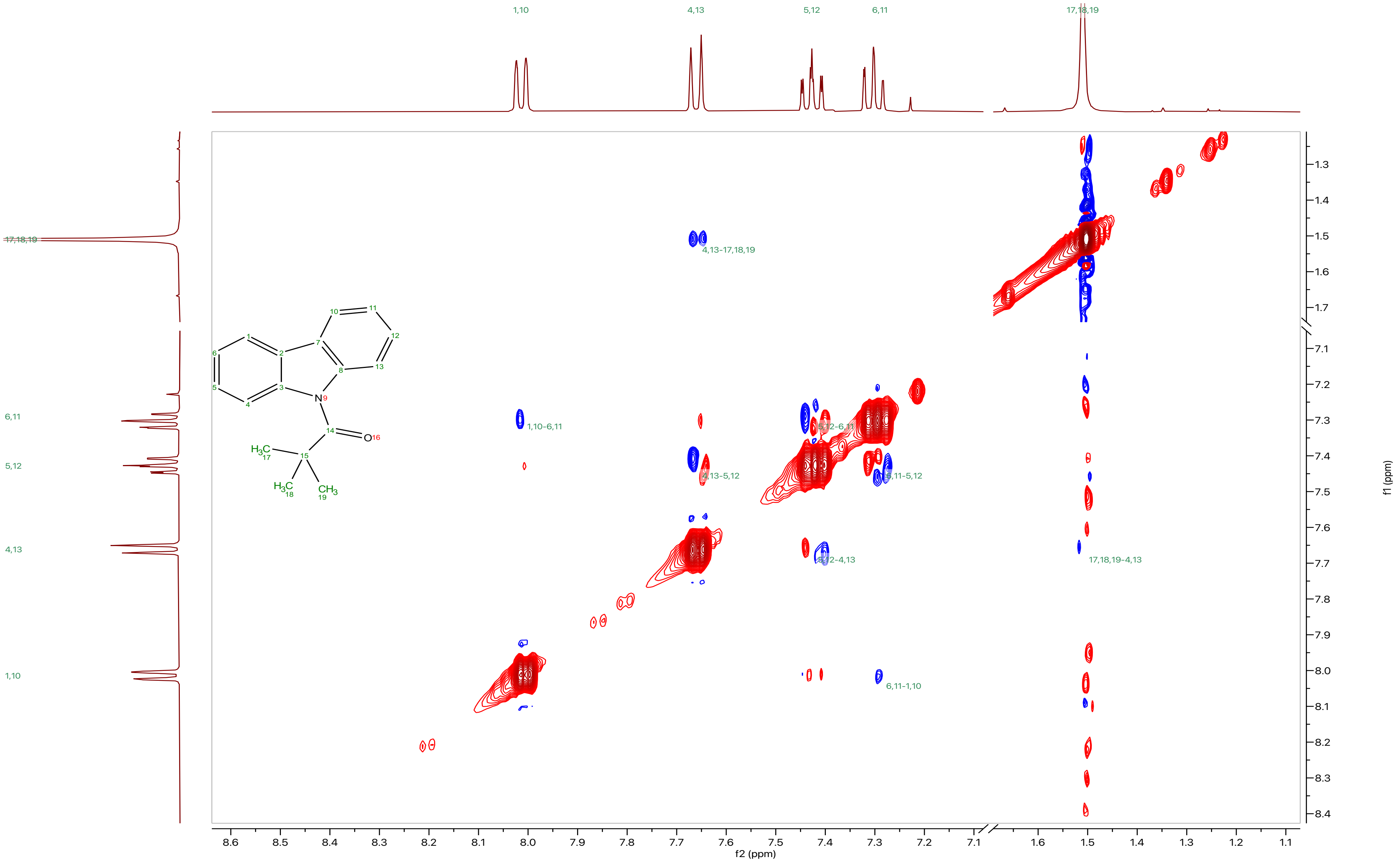

^1H-^1H NOESY (400 MHz, $CDCl_3$) of 3a

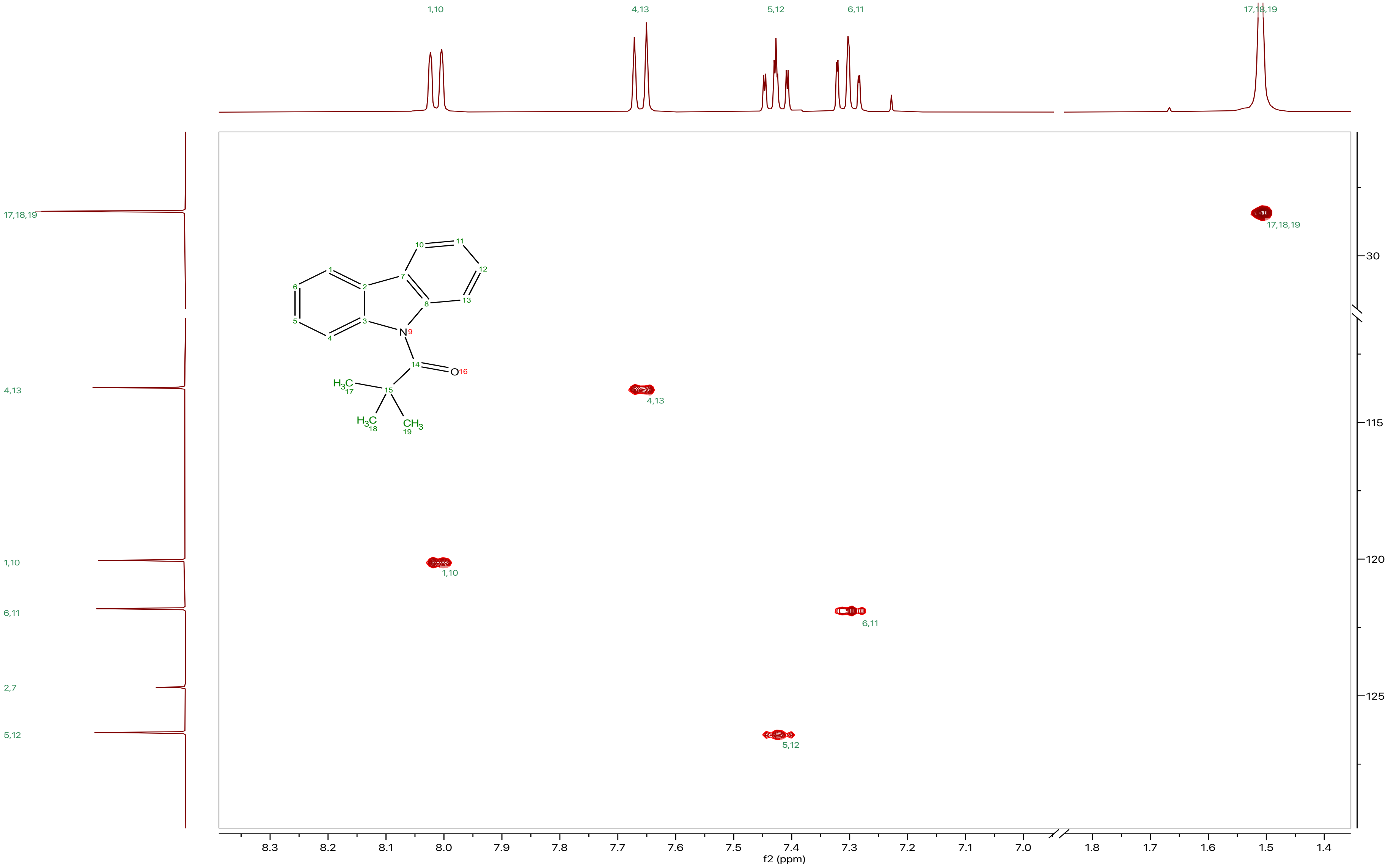

^{1}H-$^{13}C\{^{1}H\}$ HSQC NMR (400/101 MHz, $CDCl_3$) of 3a

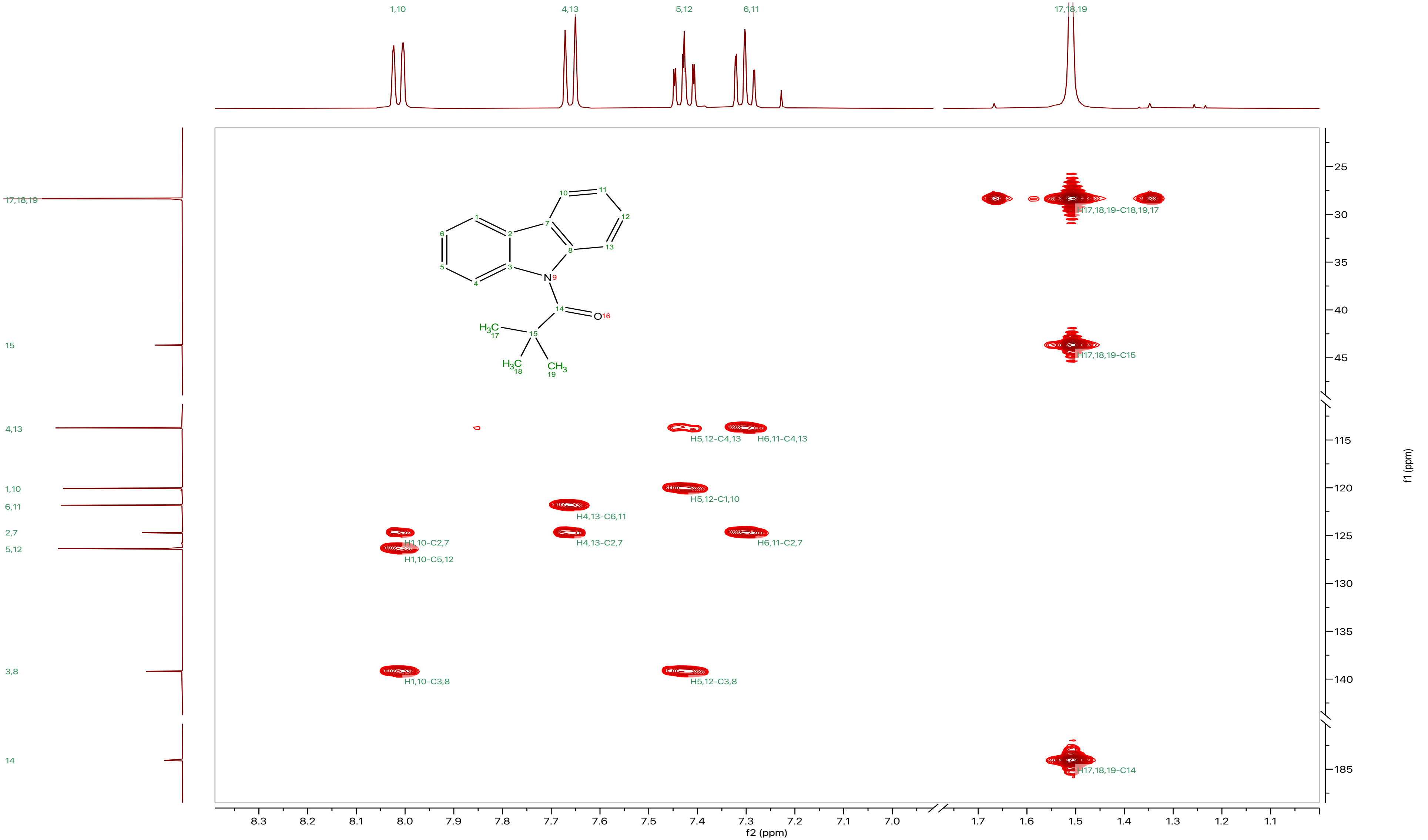

^{1}H-^{13}C{^{1}H} HMBC NMR (400/101 MHz, $CDCl_3$) of 3a

4ba

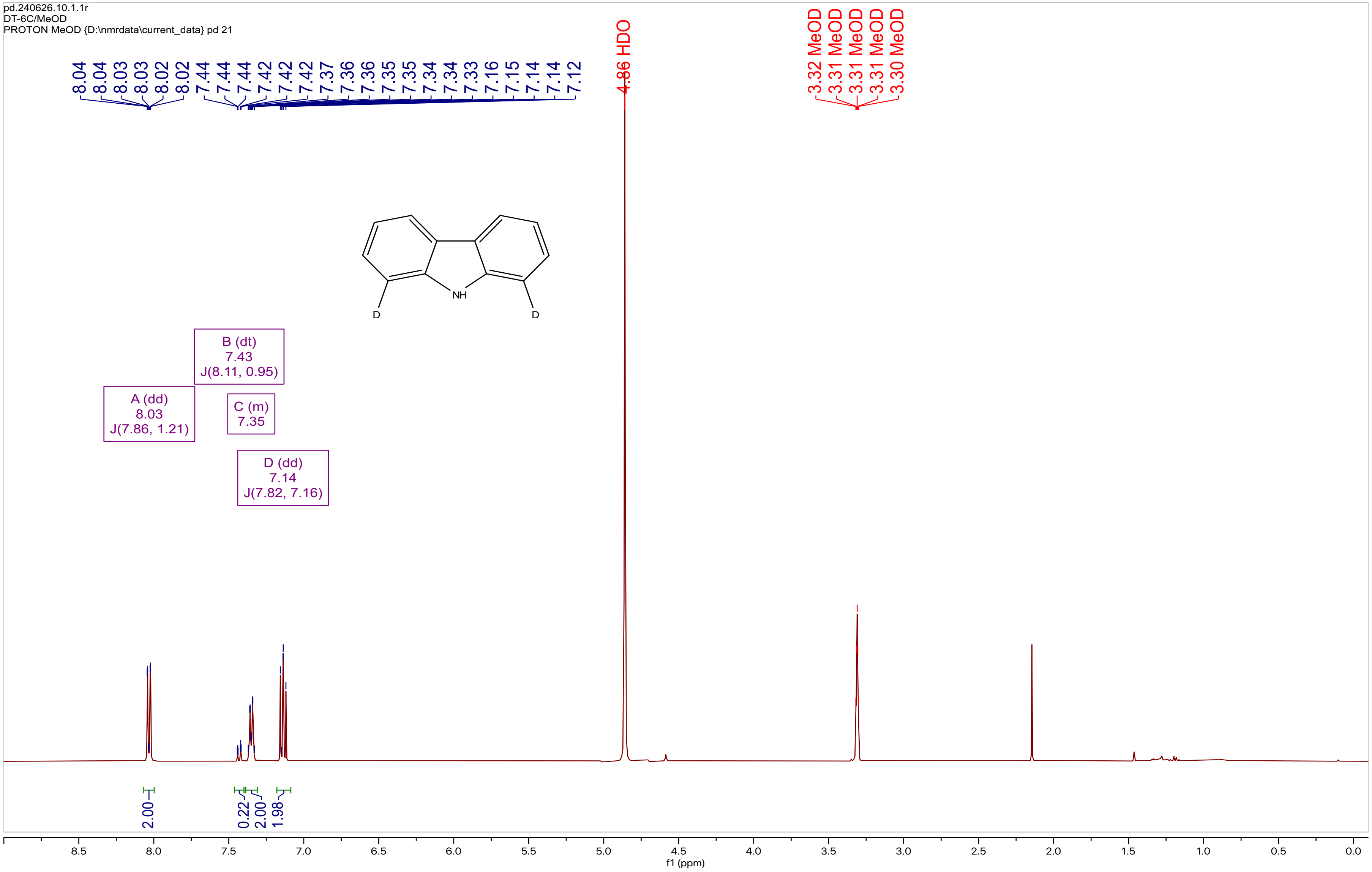

^{1}H NMR (400 MHz, MeOD) of 4ba

pd.240626.11.1.1r
DT-6C/MeOD
C13CPD MeOD {D:\nmrdata\current_data} pd 21

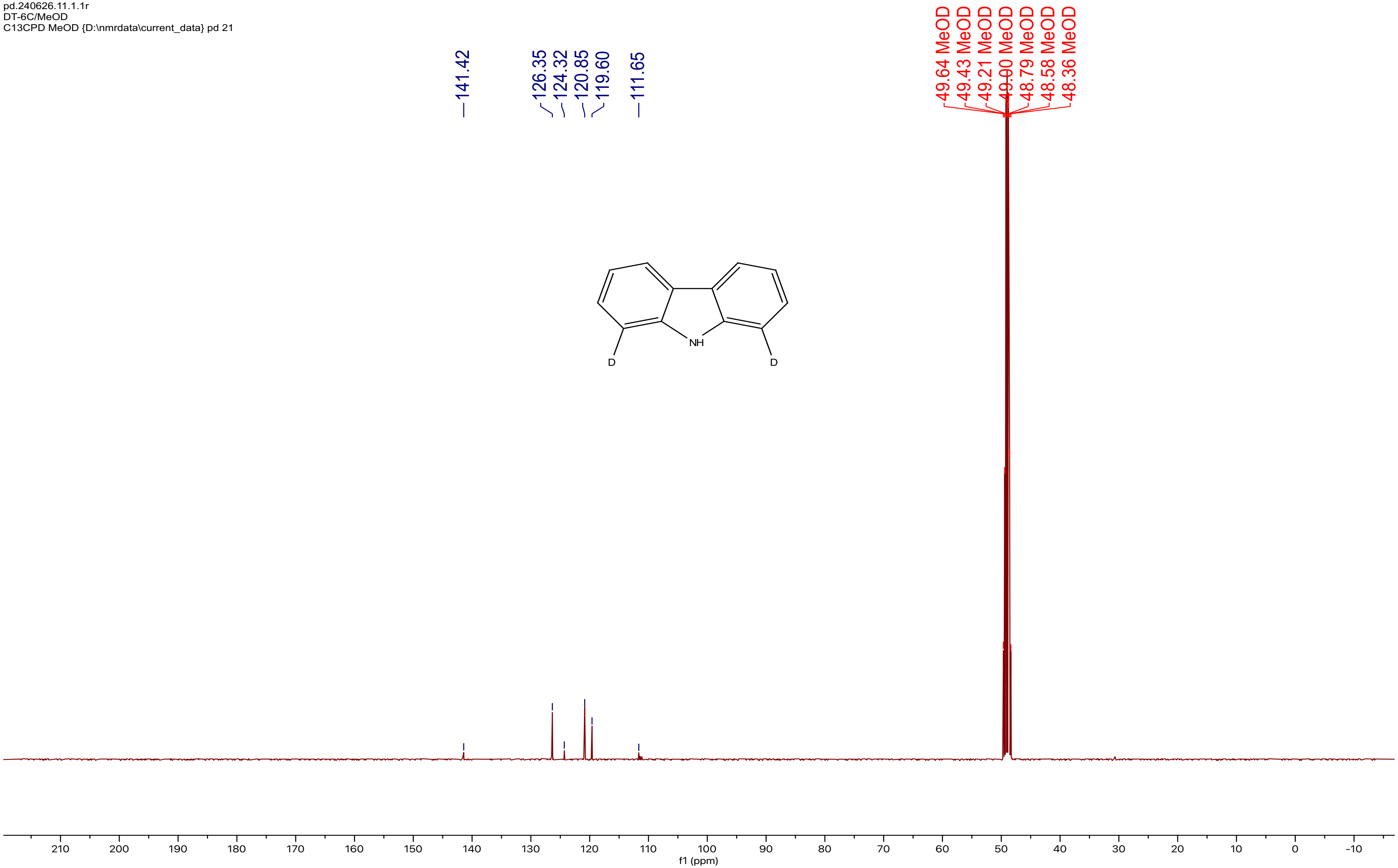

$^{13}C\{^{1}H\}$ NMR (101 MHz, MeOD) of 4ba

3a’

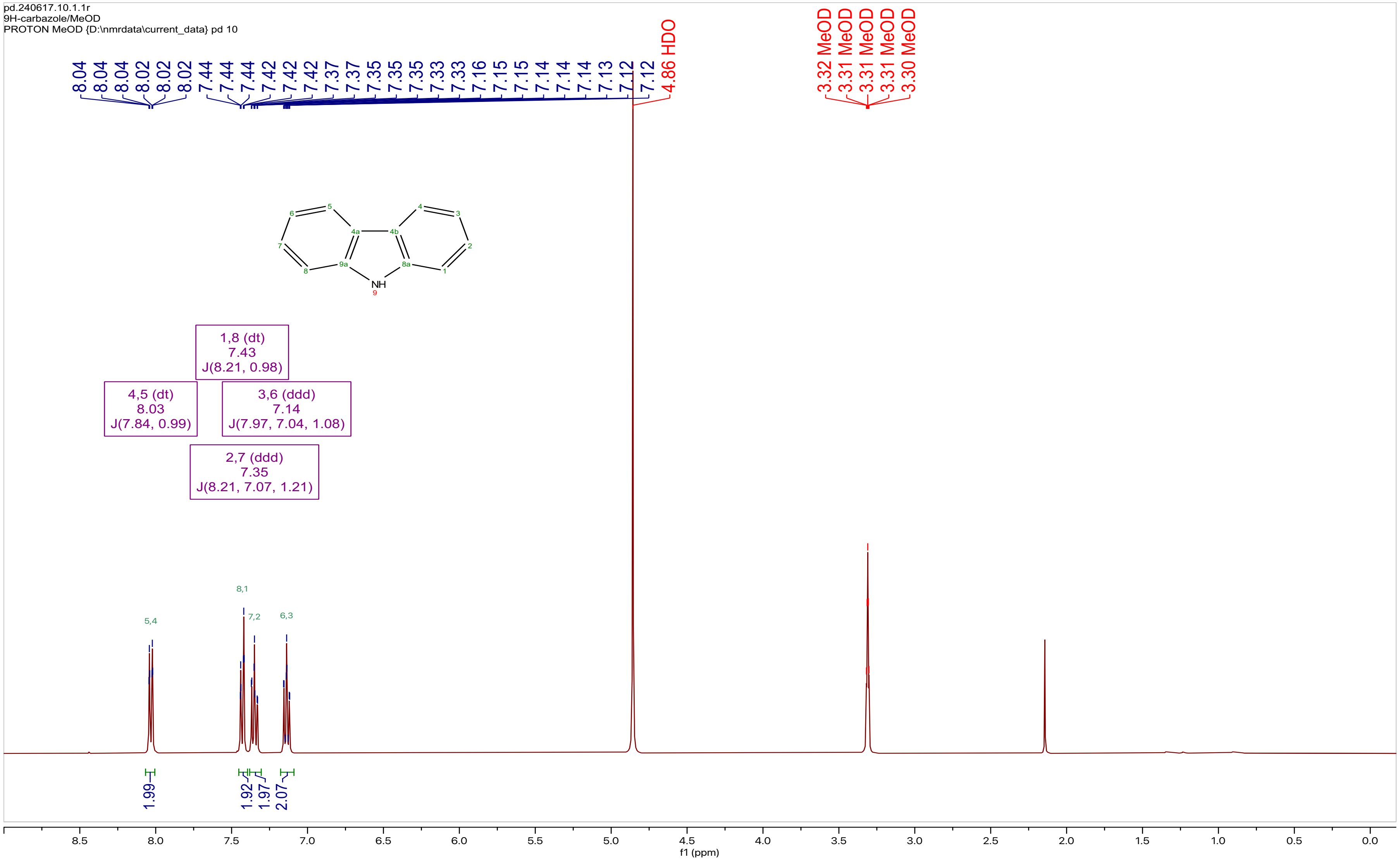

^{1}H NMR (400 MHz, MeOD) of 3a’

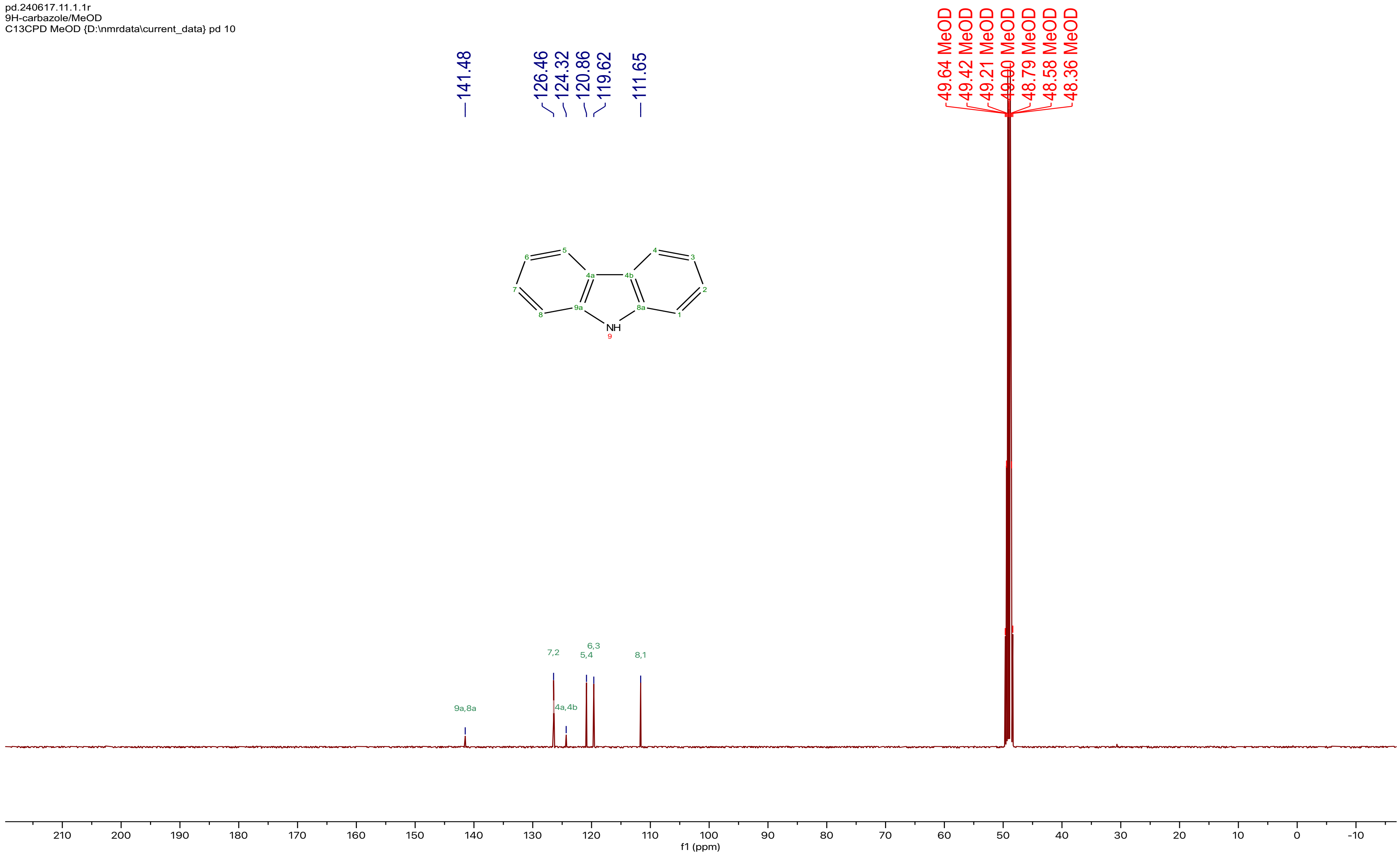

$^{13}C\{^{1}H\}$ NMR (101 MHz, MeOD) of 3a’

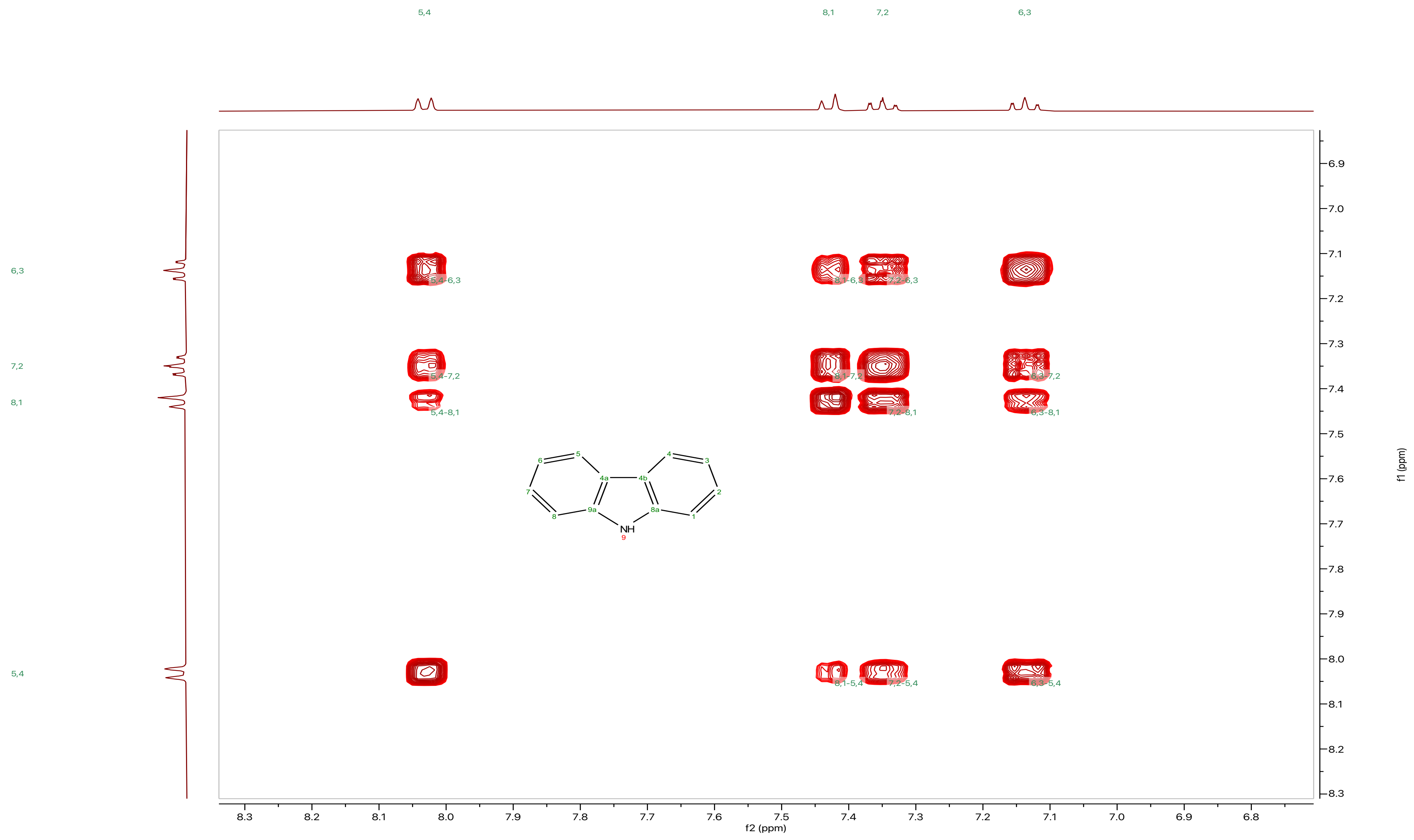

^{1}H-^{1}H COSY (400 MHz, MeOD) of 3a'

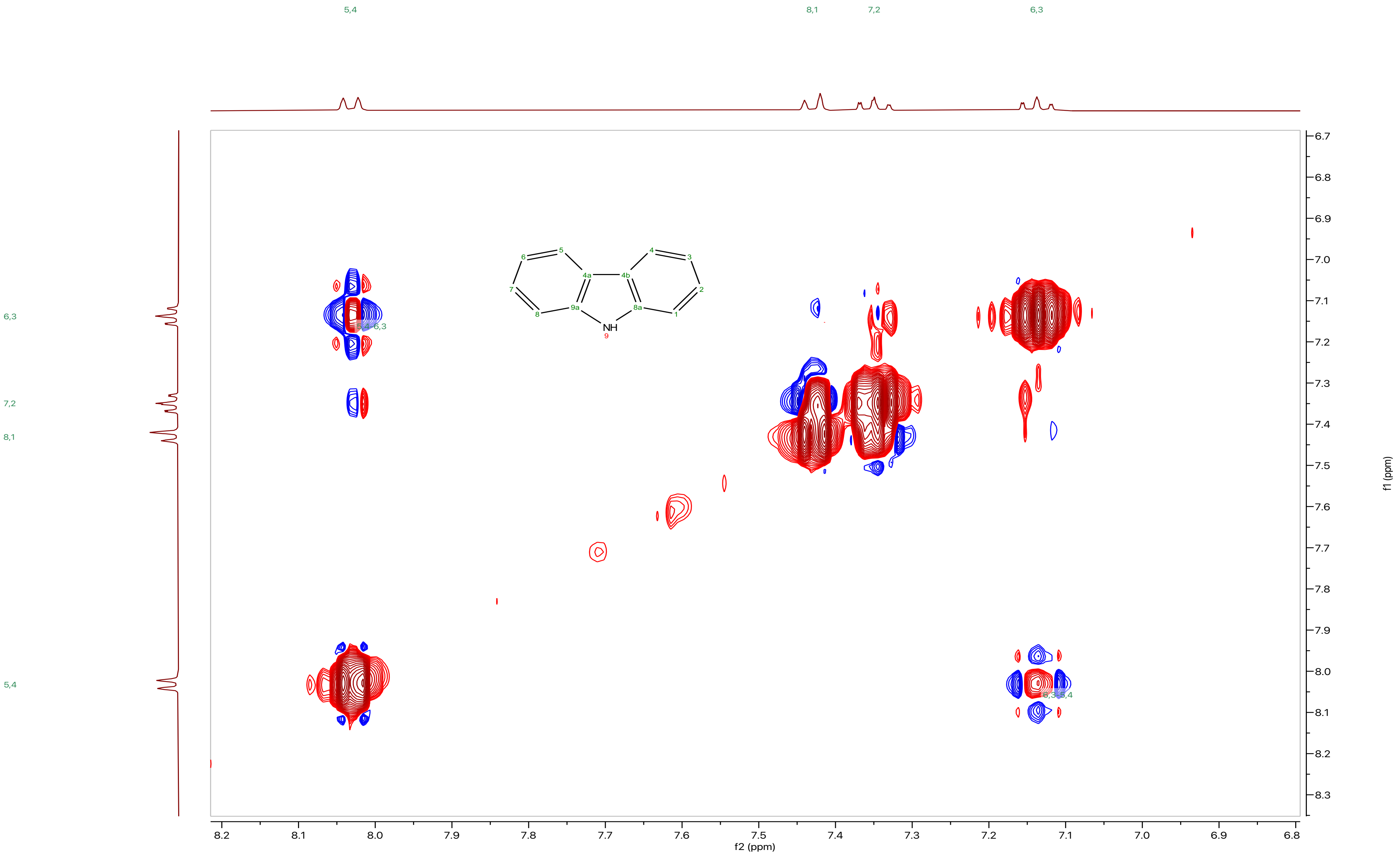

^1H-^1H NOESY (400 MHz, MeOD) of 3a’

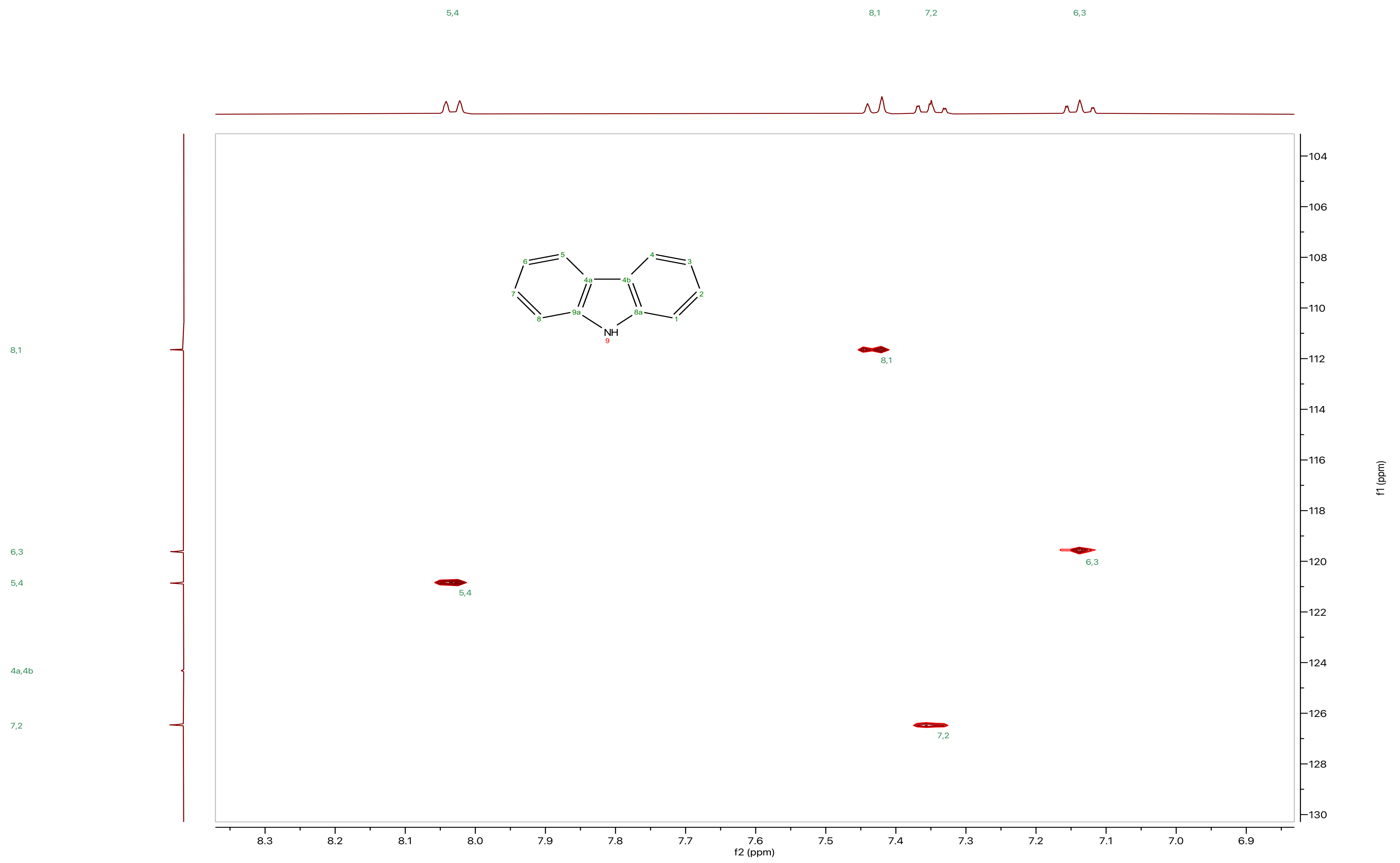

^{1}H-$^{13}C\{^{1}H\}$ HSQC NMR (400/101 MHz, MeOD) of 3a'

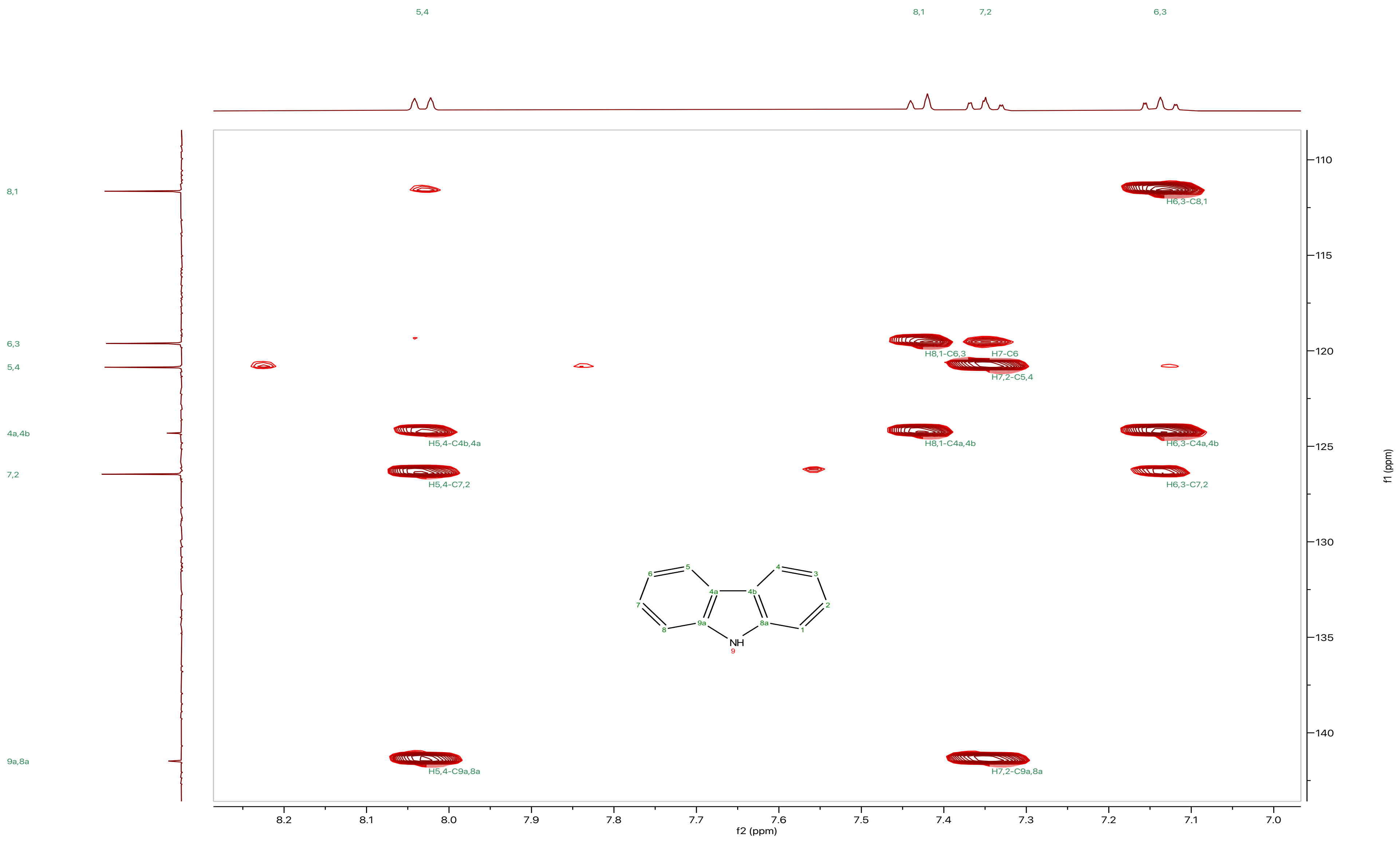

^{1}H-$^{13}C\{^{1}H\}$ HMBC NMR (400/101 MHz, MeOD) of 3a'

4ba’

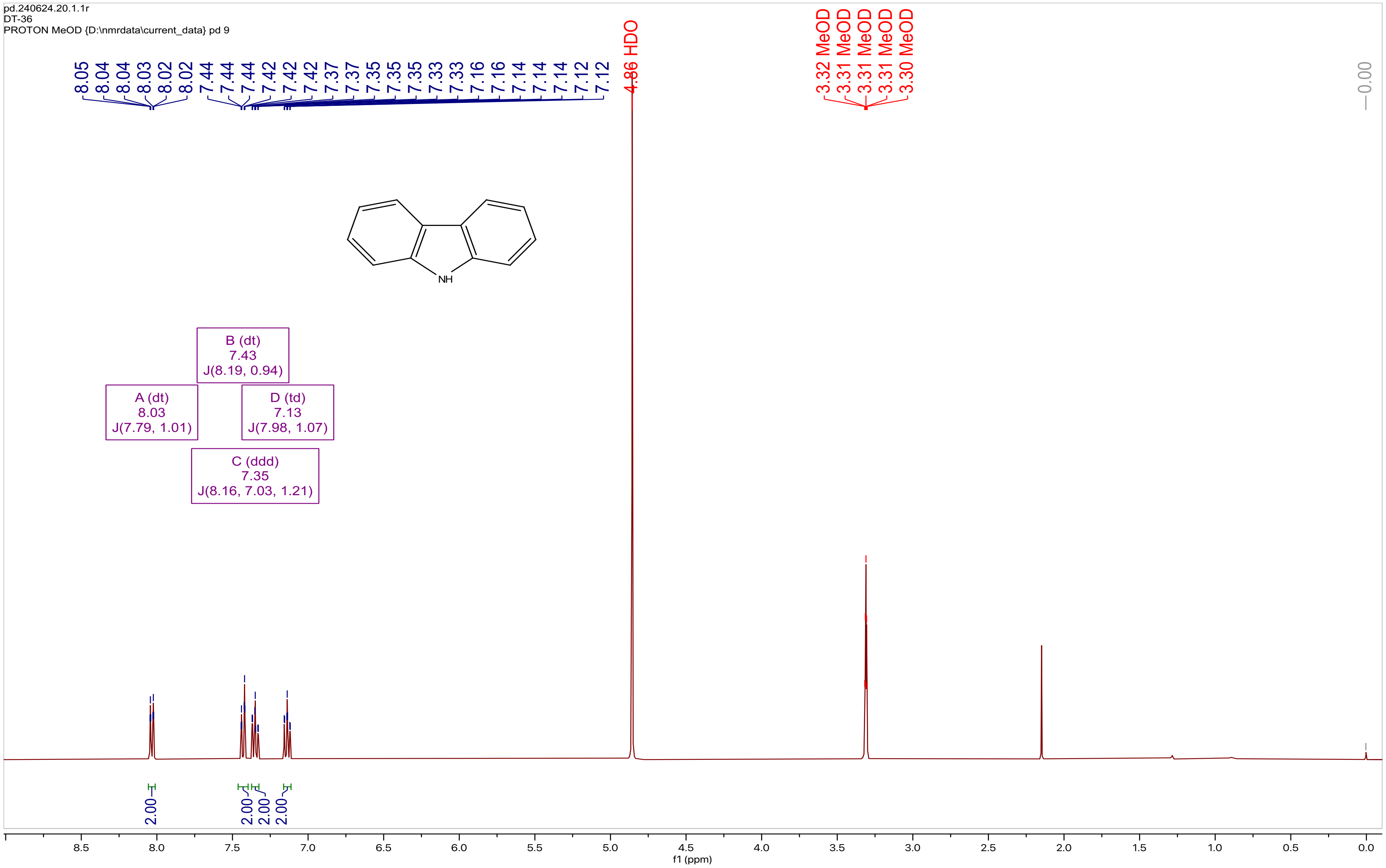

^{1}H NMR (400 MHz, MeOD) of 4ba’

pd.240624.21.1.1r
DT-36
C13CPD MeOD {D:\nmrdata\current_data} pd 9

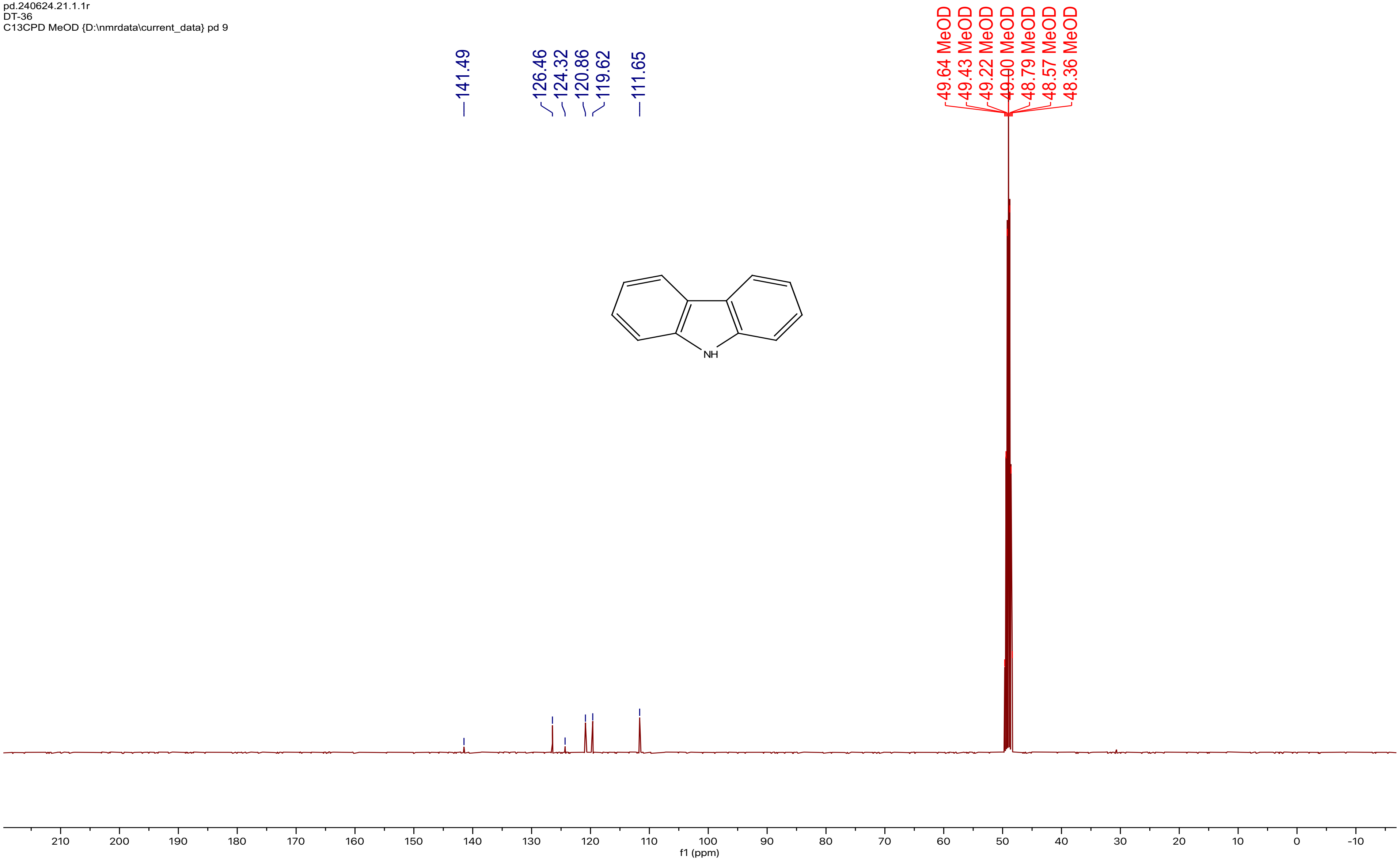

$^{13}C\{^1H\}$ NMR (101 MHz, MeOD) of 4ba'

3b

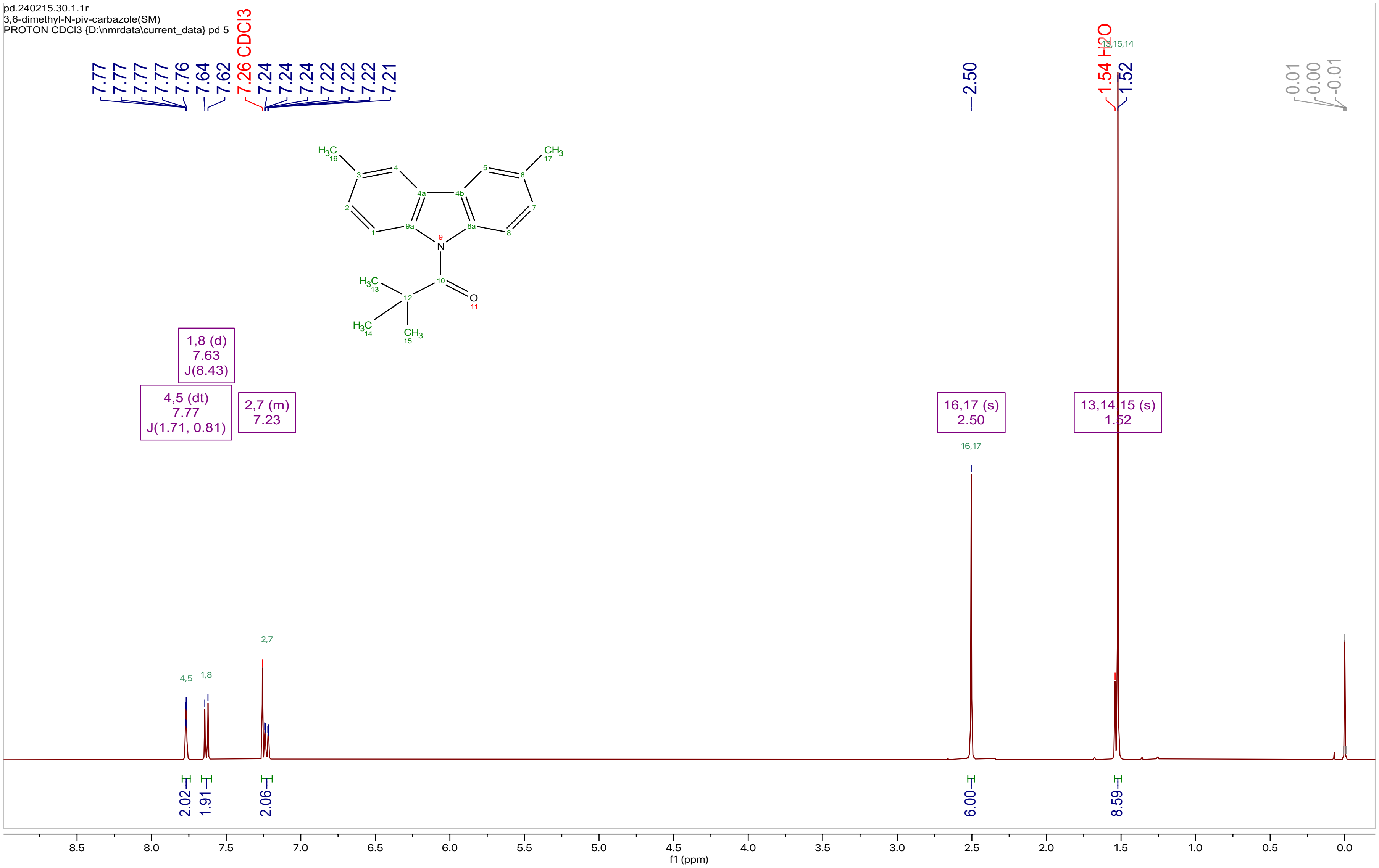

^1H NMR (400 MHz, $CDCl_3$) of 3b

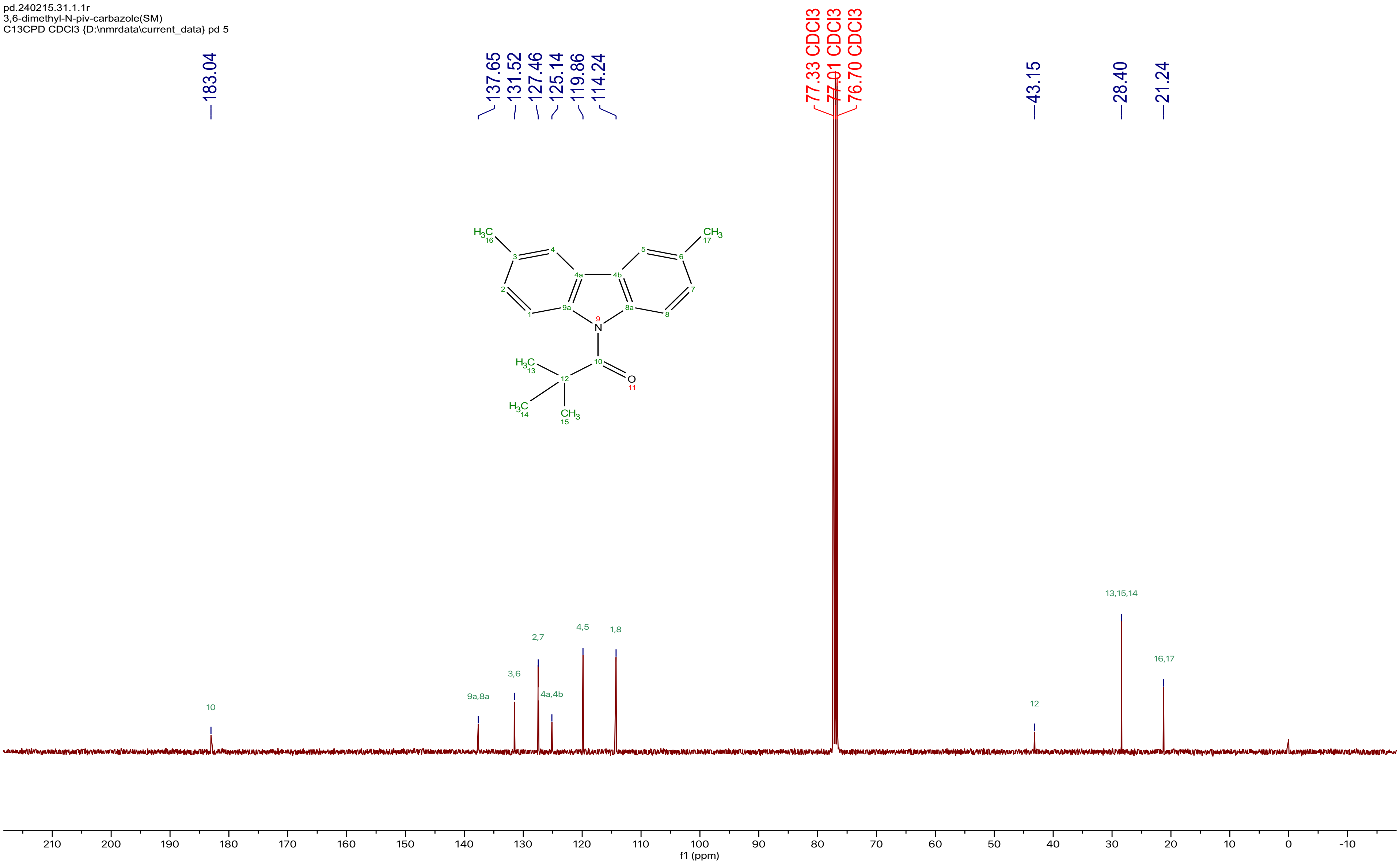

$^{13}C\{^1H\}$ NMR (101 MHz, $CDCl_3$) of 3b

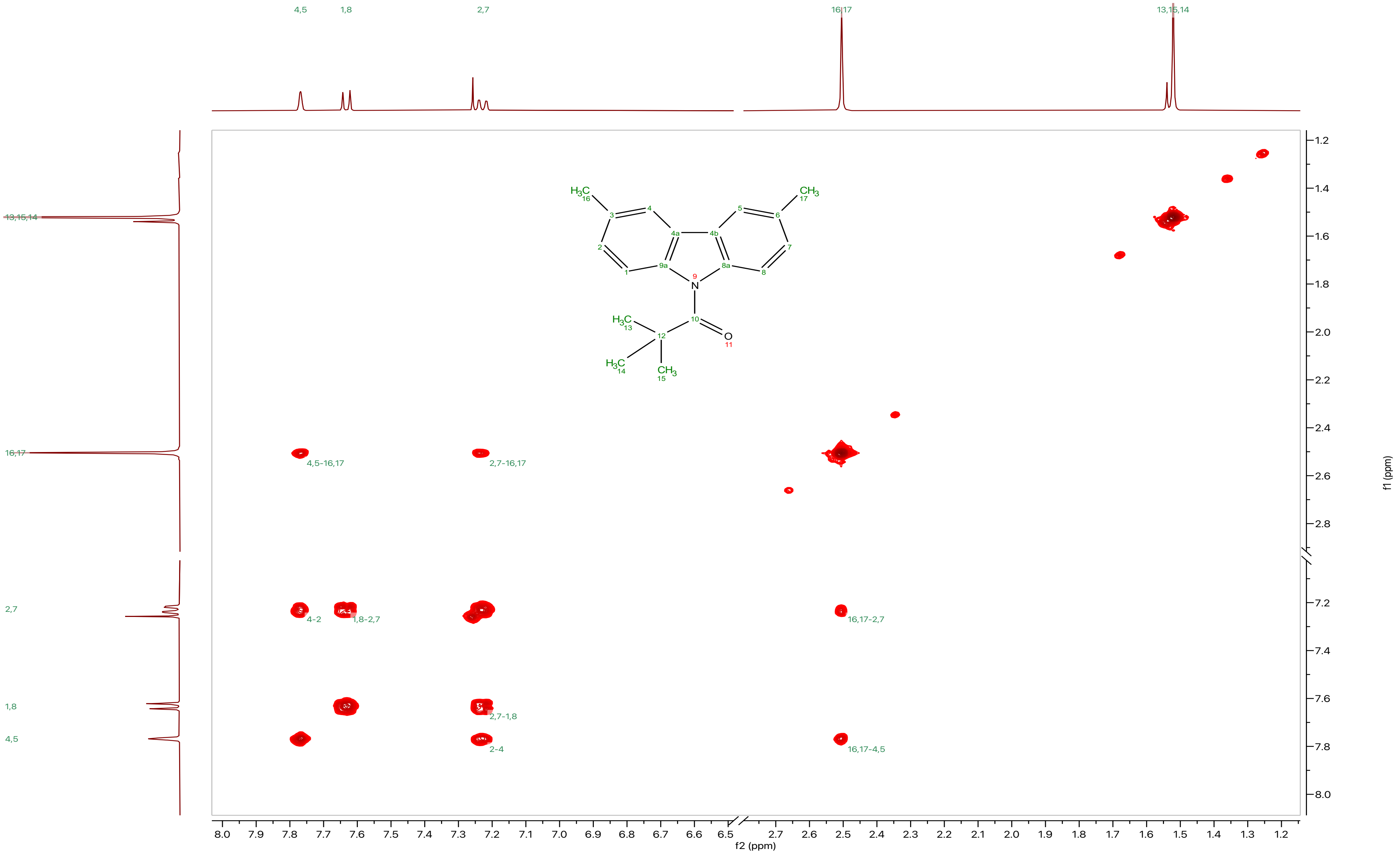

^{1}H-^{1}H COSY (400 MHz, $CDCl_3$) of 3b

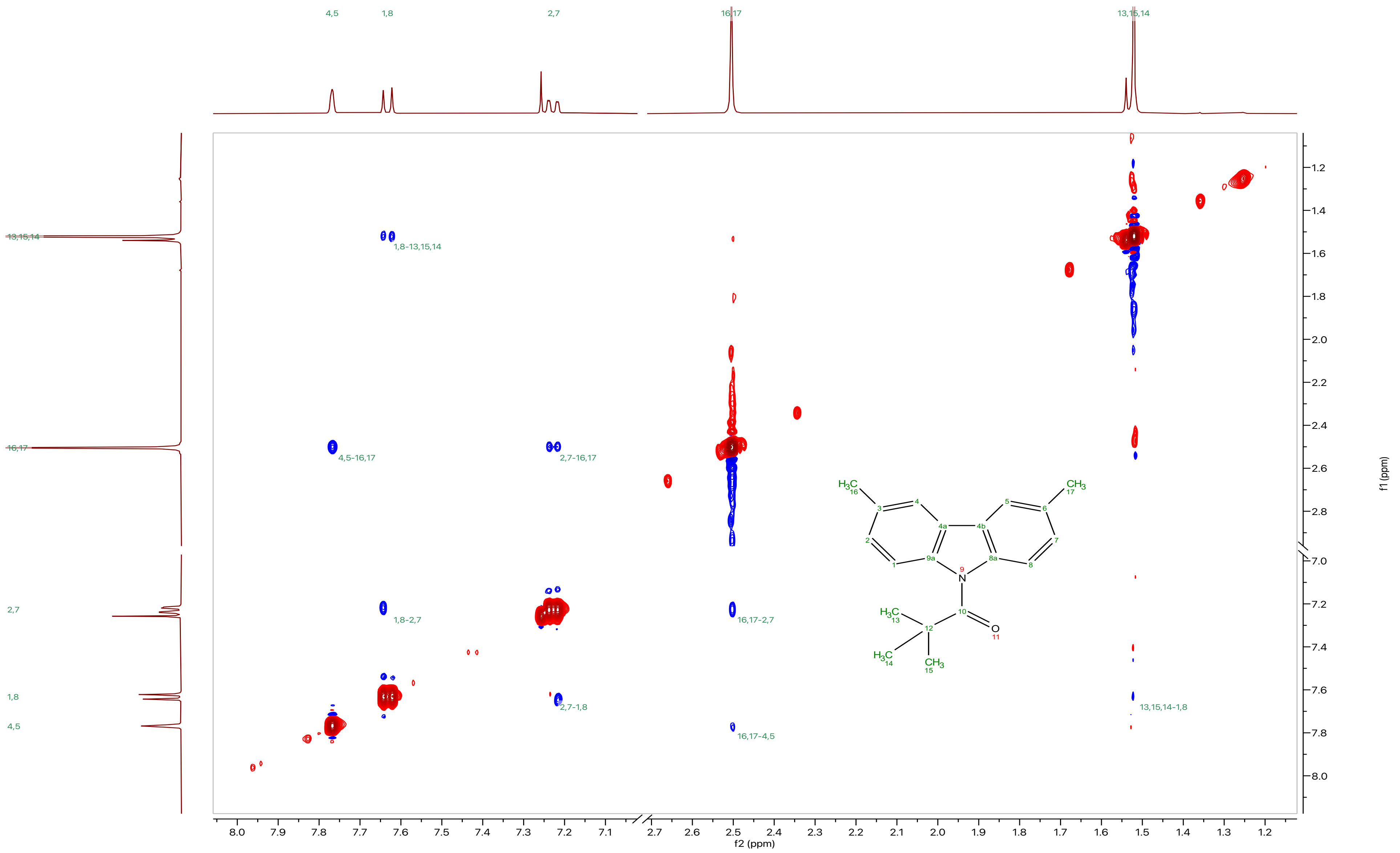

^1H-^1H NOESY (400 MHz, $CDCl_3$) of 3b

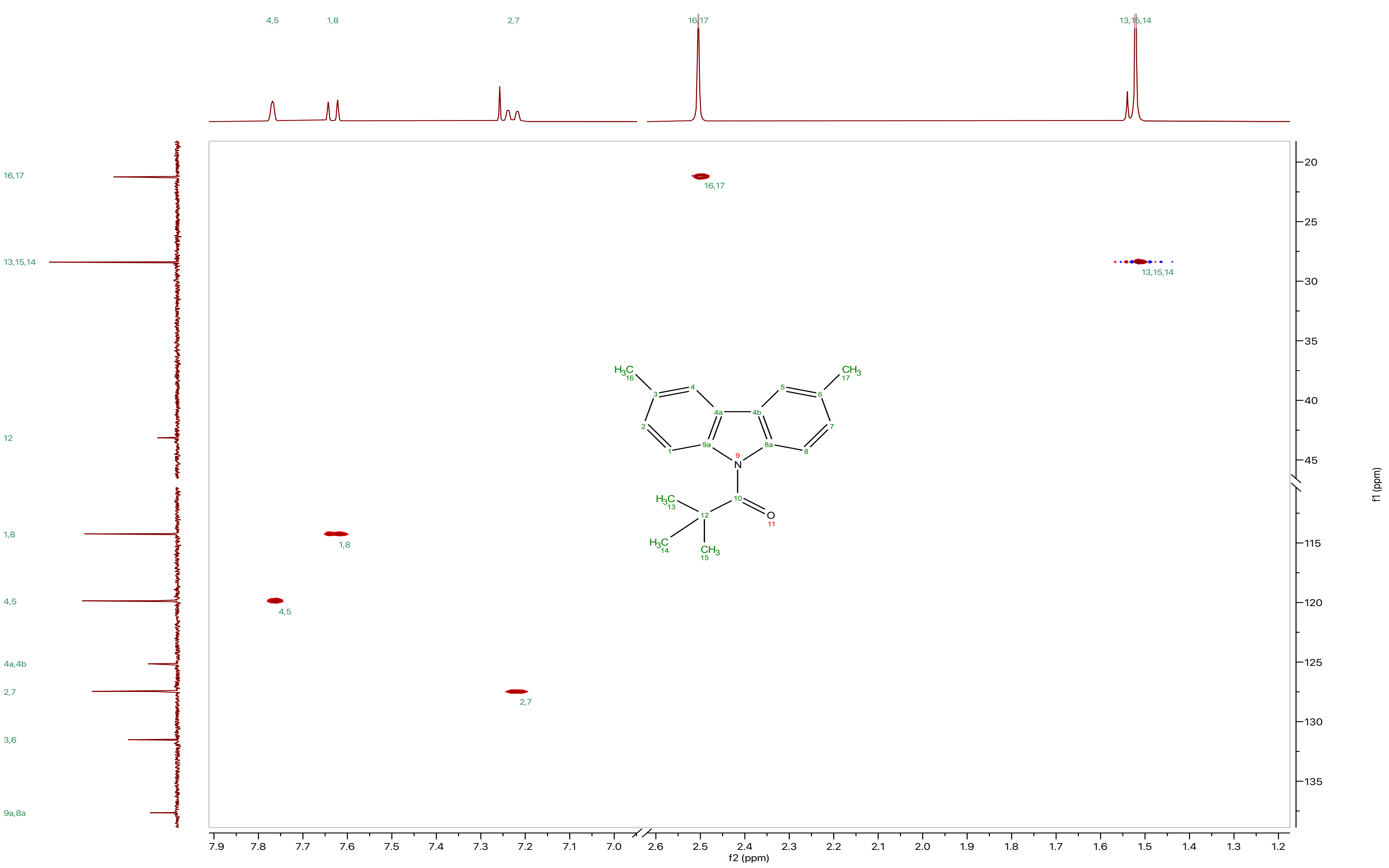

^{1}H-$^{13}C\{^{1}H\}$ HSQC NMR (400/101 MHz, $CDCl_3$) of 3b

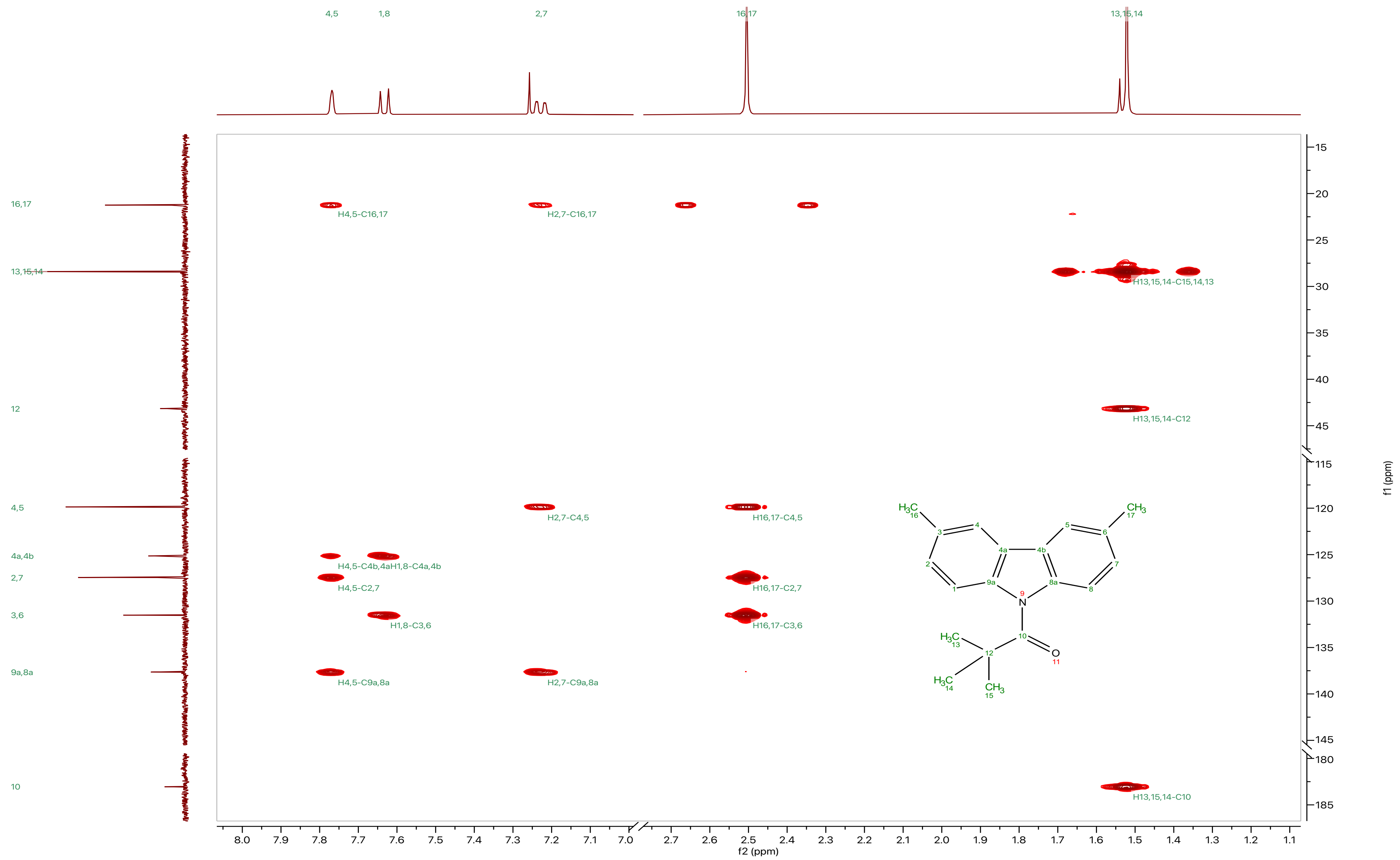

^{1}H-^{13}C{^{1}H} HMBC NMR (400/101 MHz, CDCl$_3$) of 3b

4ab

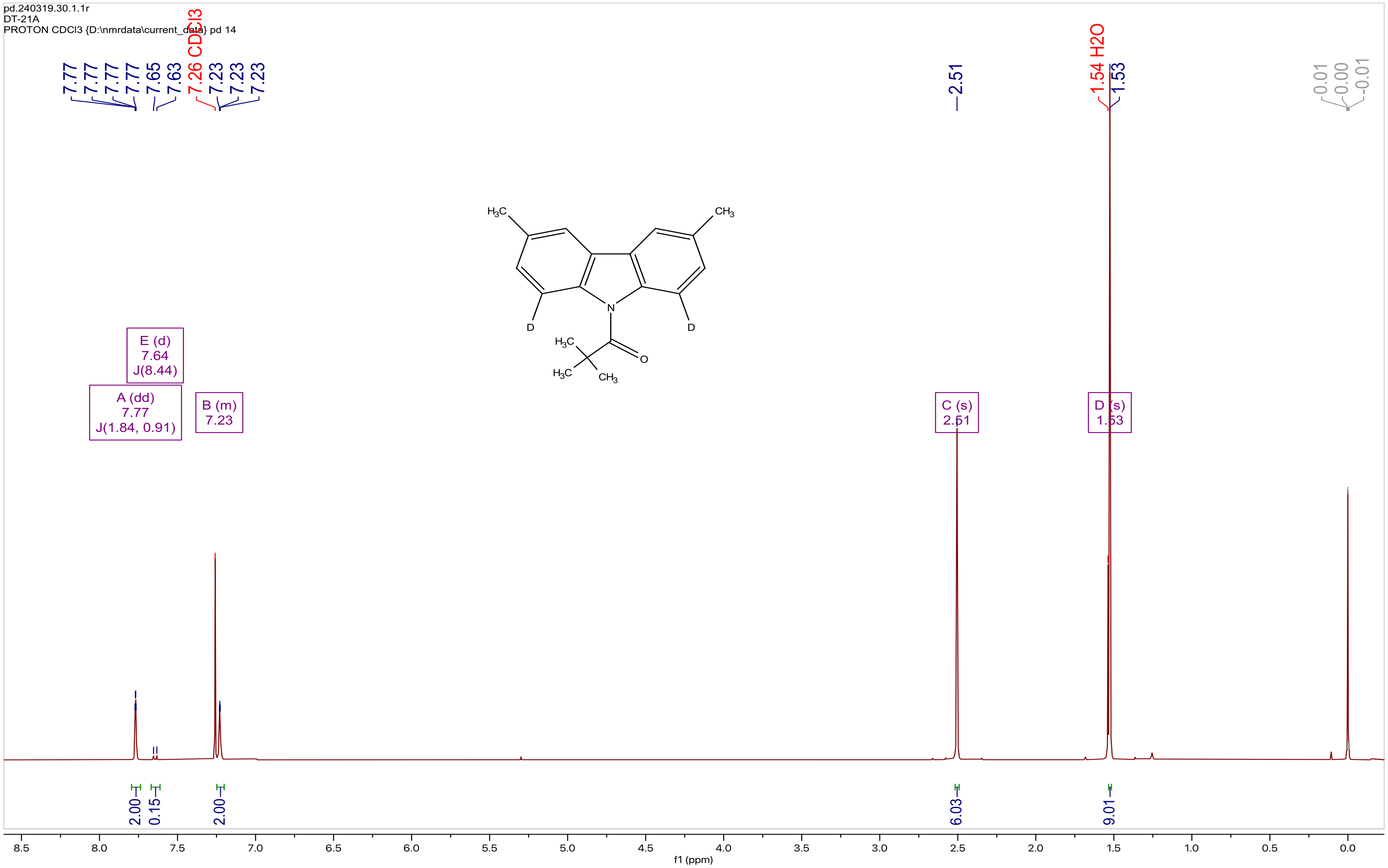

^{1}H NMR (400 MHz, $CDCl_3$) of 4ab

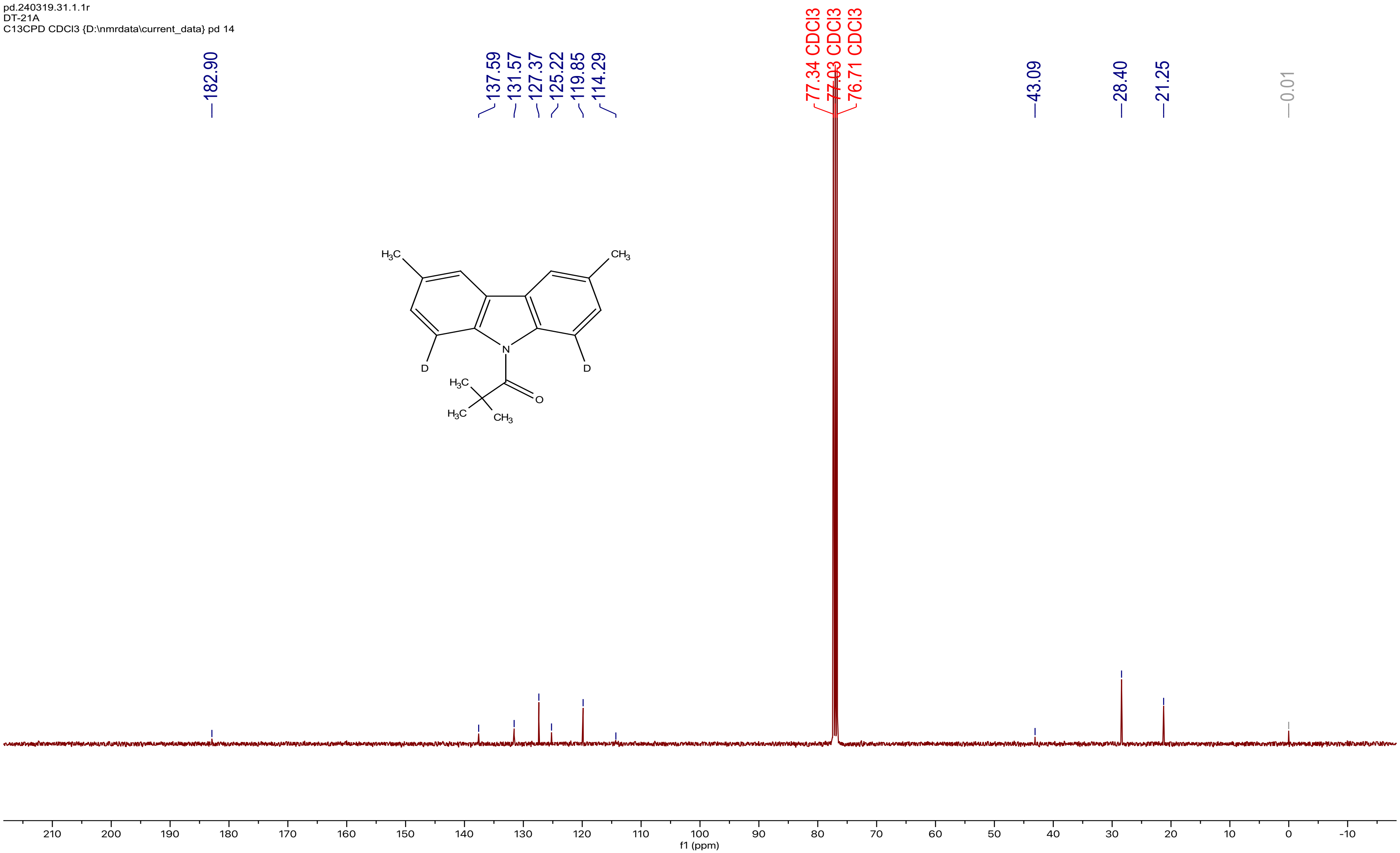

$^{13}C\{^1H\}$ NMR (101 MHz, $CDCl_3$) of 4ab

3b’

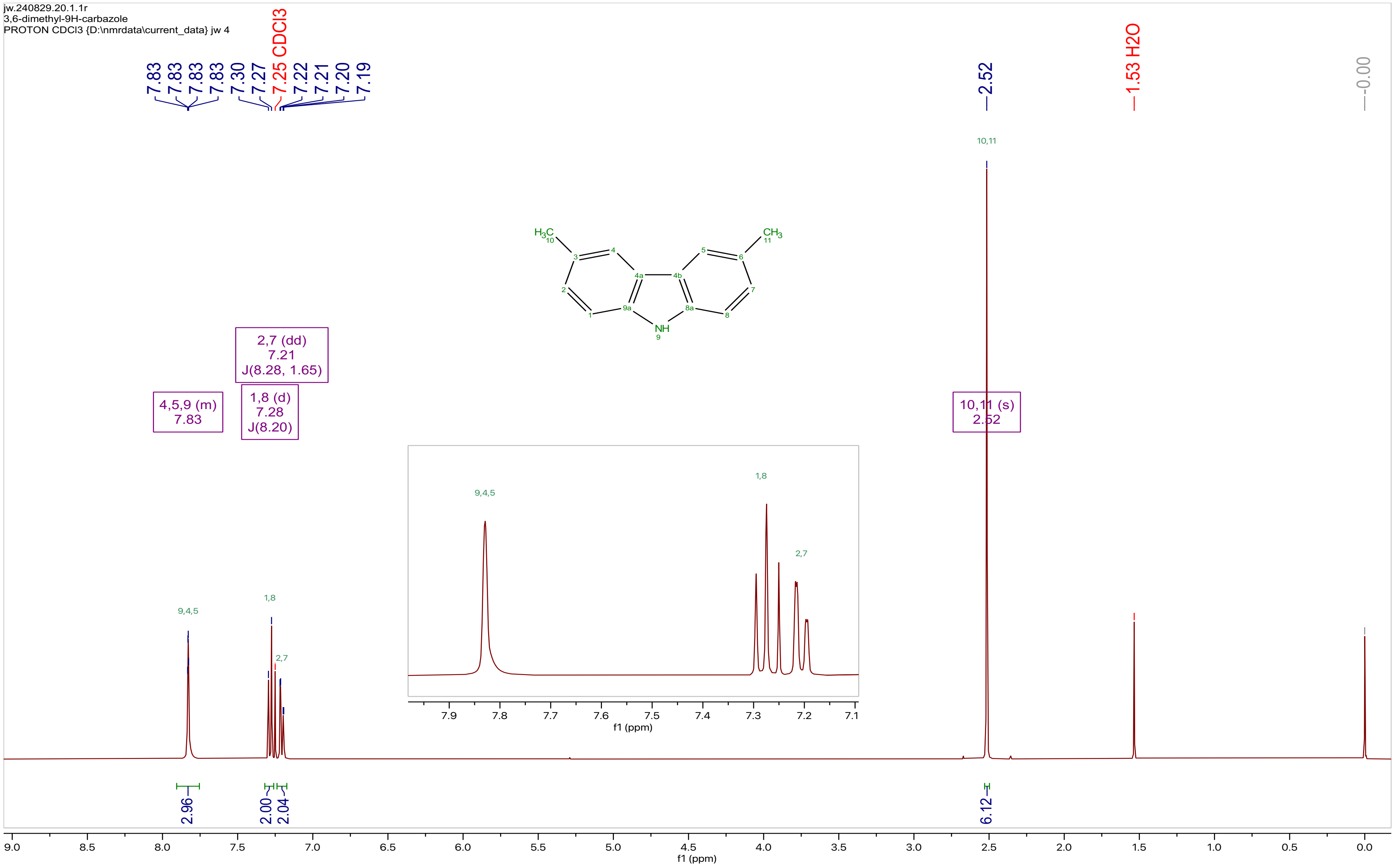

^{1}H NMR (400 MHz, $CDCl_3$) of 3b’

jw.240829.21.1.1r
3,6-dimethyl-9H-carbazole
C13CPD CDCl3 {D:\nmrdata\current_data} jw 4

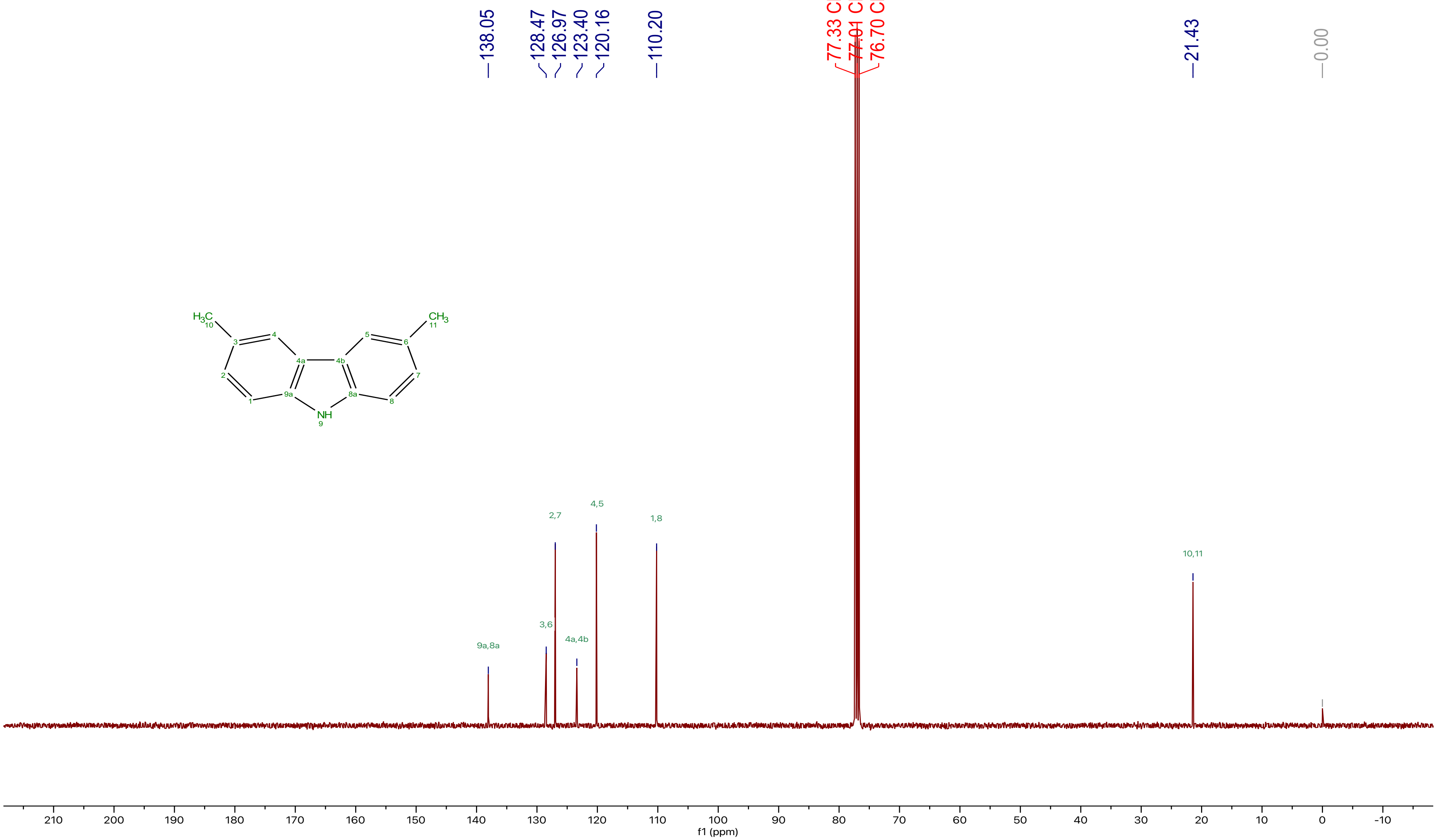

$^{13}C\{^1H\}$ NMR (101 MHz, $CDCl_3$) of 3b'

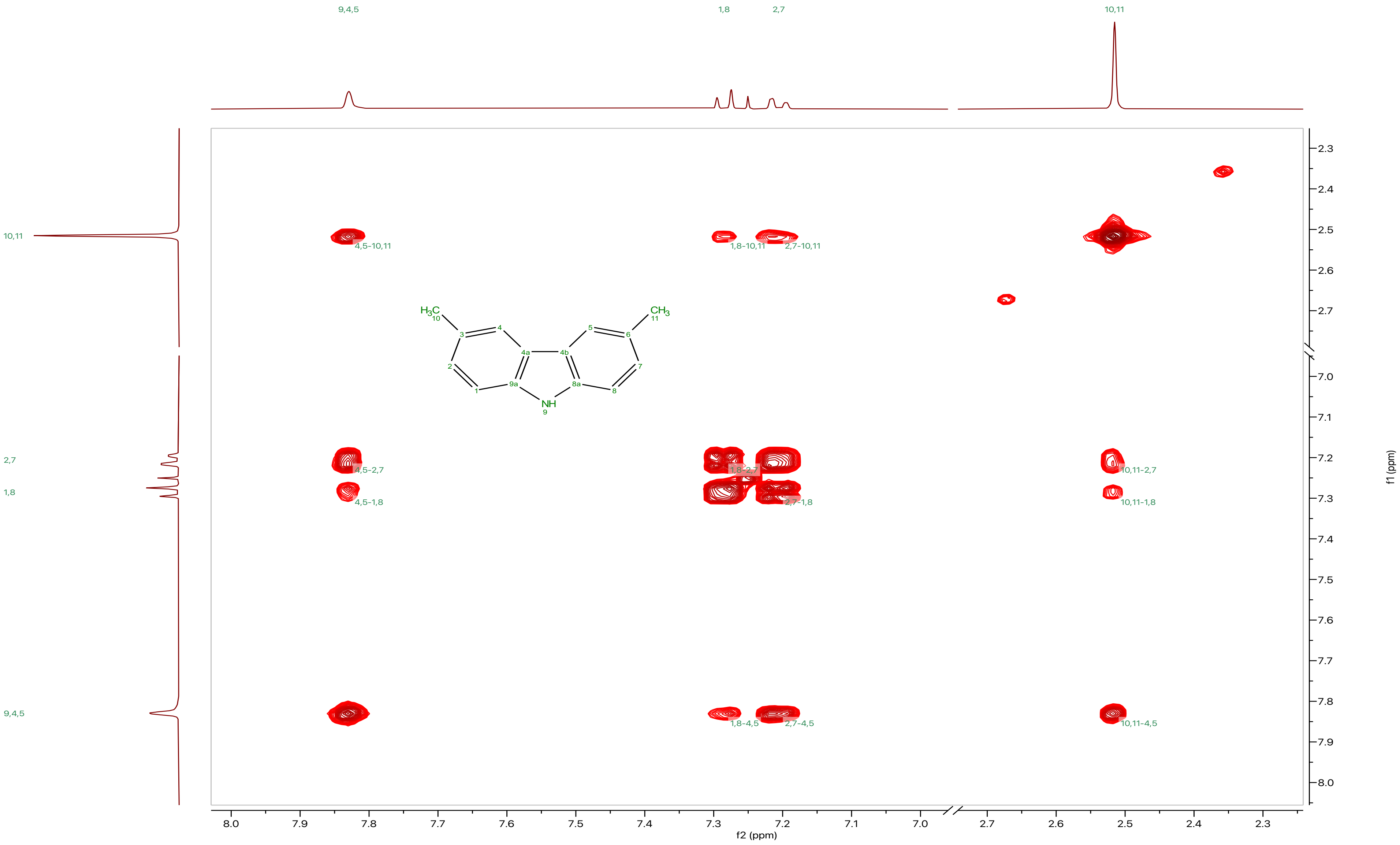

^{1}H-^{1}H COSY (400 MHz, $CDCl_3$) of 3b'

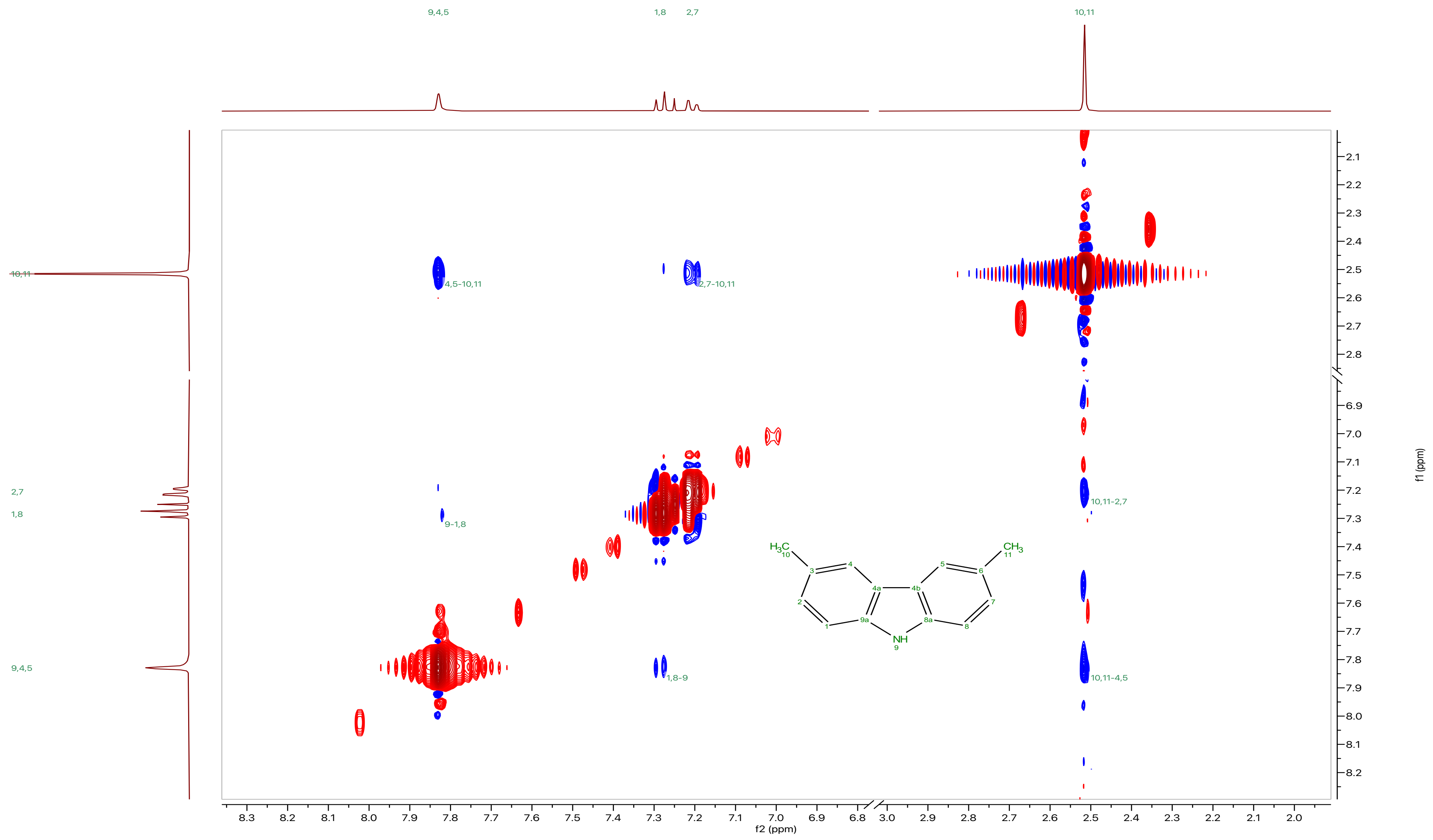

¹H-¹H NOESY (400 MHz, CDCl₃) of 3b’

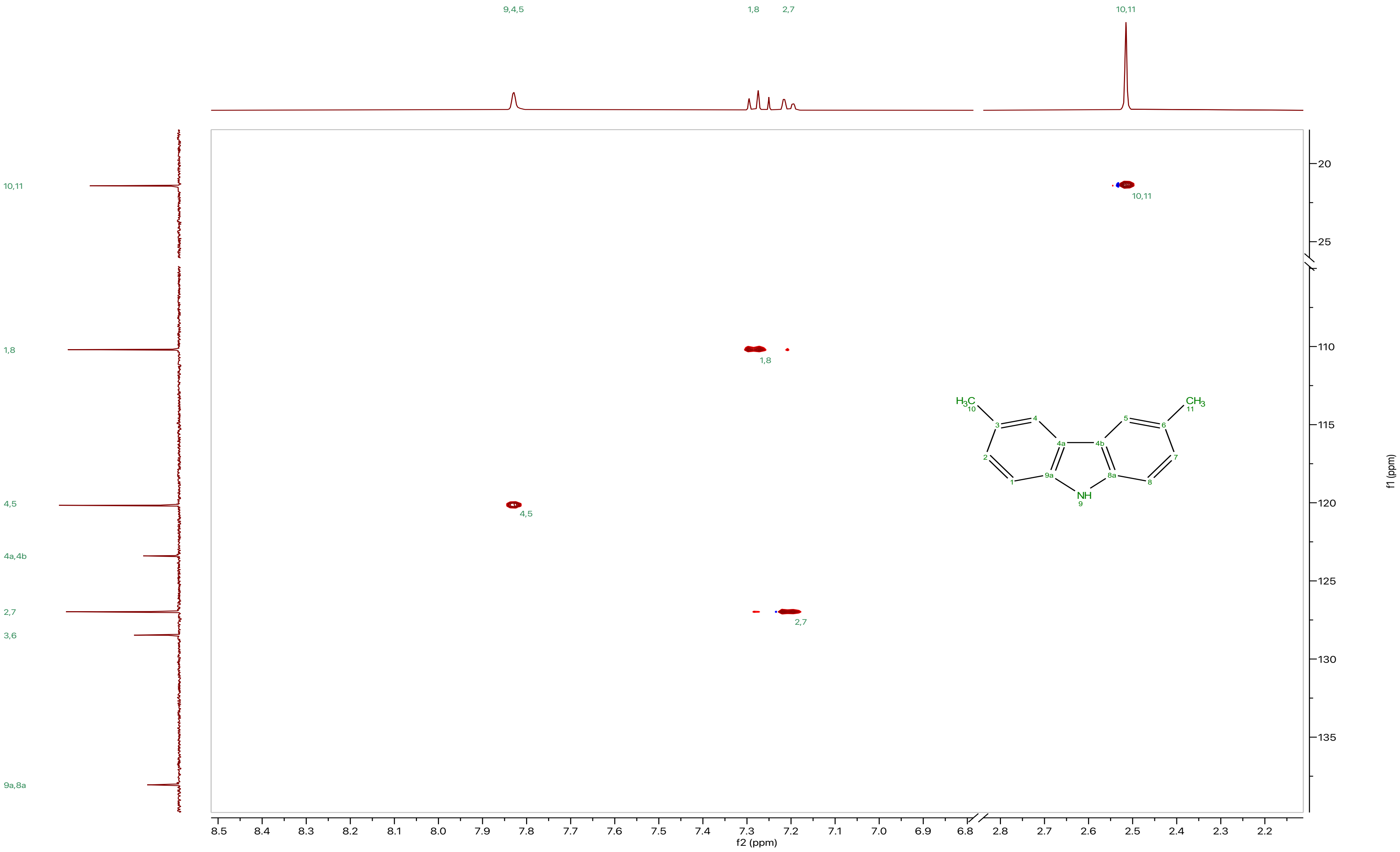

^{1}H-$^{13}C\{^{1}H\}$ HSQC NMR (400/101 MHz, $CDCl_3$) of 3b’

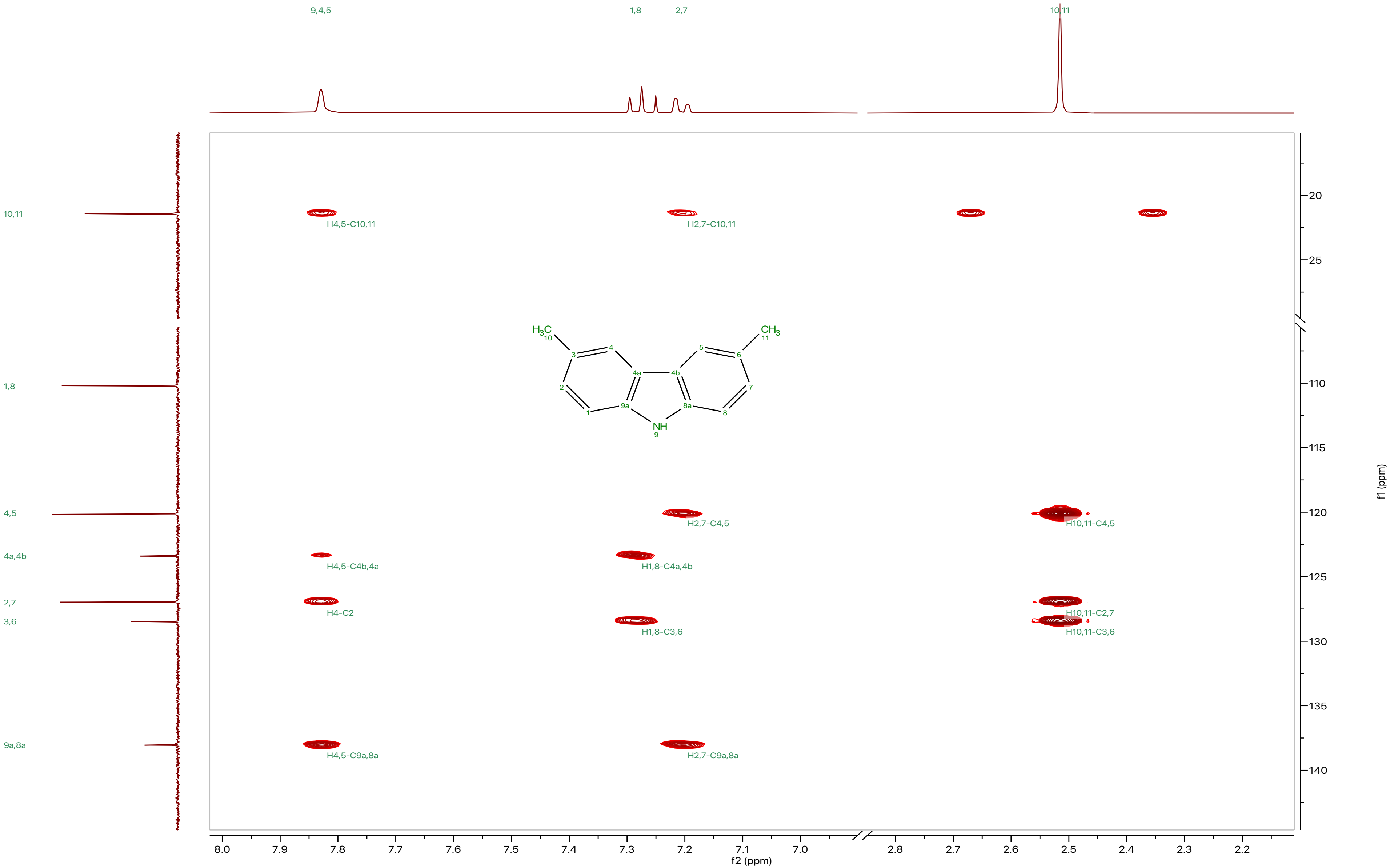

^{1}H-^{13}C{^{1}H} HMBC NMR (400/101 MHz, $CDCl_3$) of 3b'

4bb

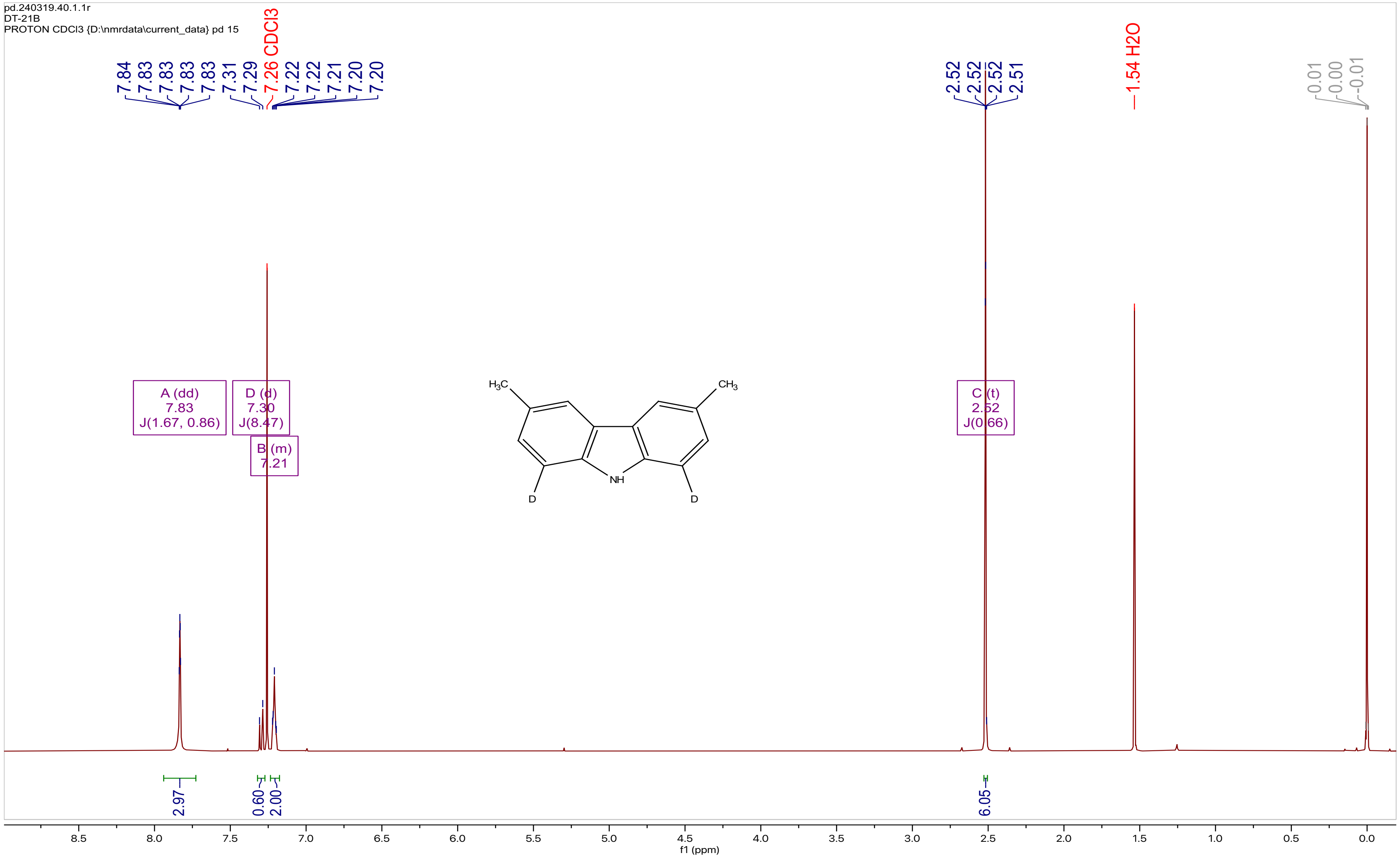

^{1}H NMR (400 MHz, $CDCl_3$) of 4bb

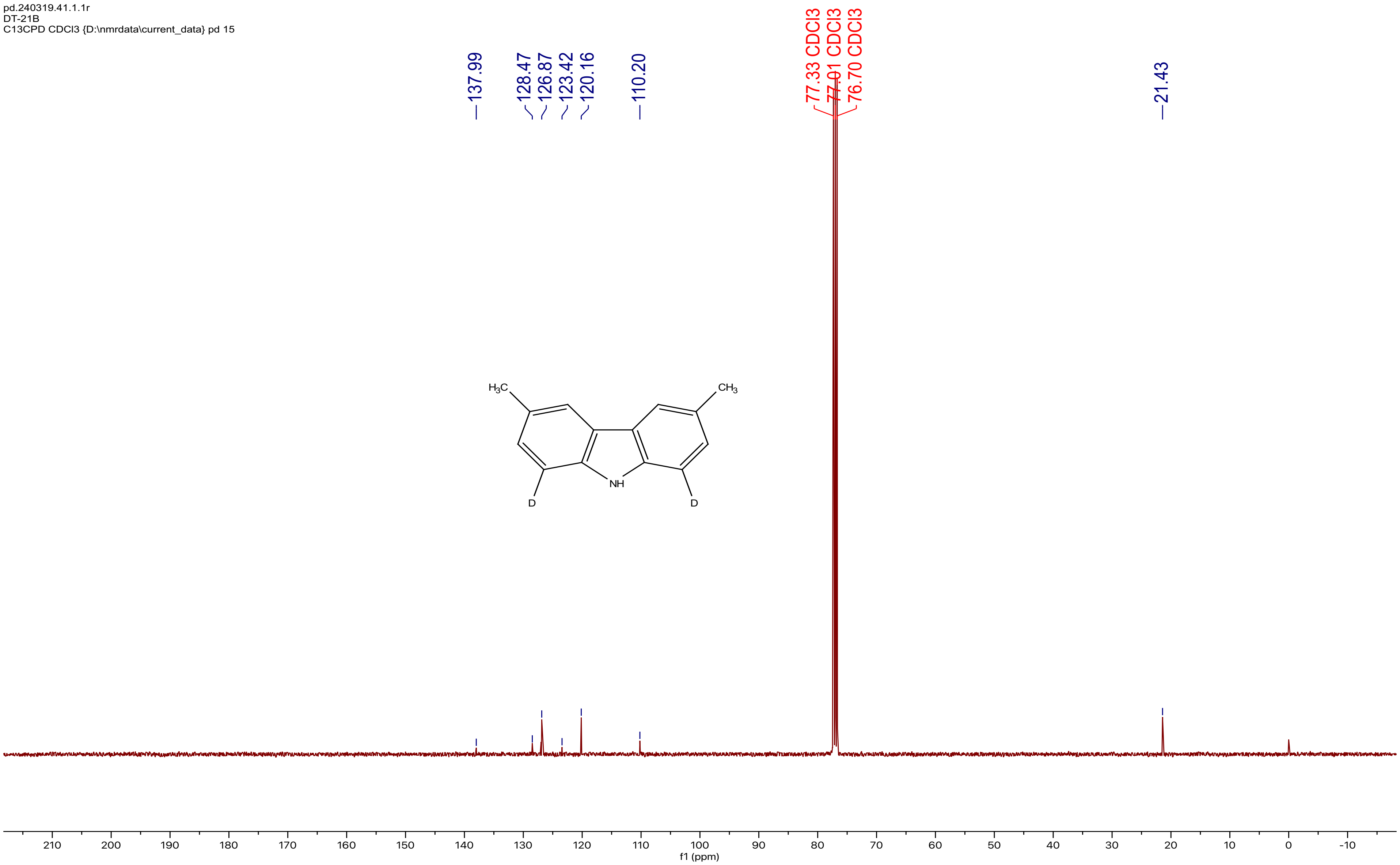

$^{13}C\{^{1}H\}$ NMR (101 MHz, $CDCl_3$) of 4bb

3c

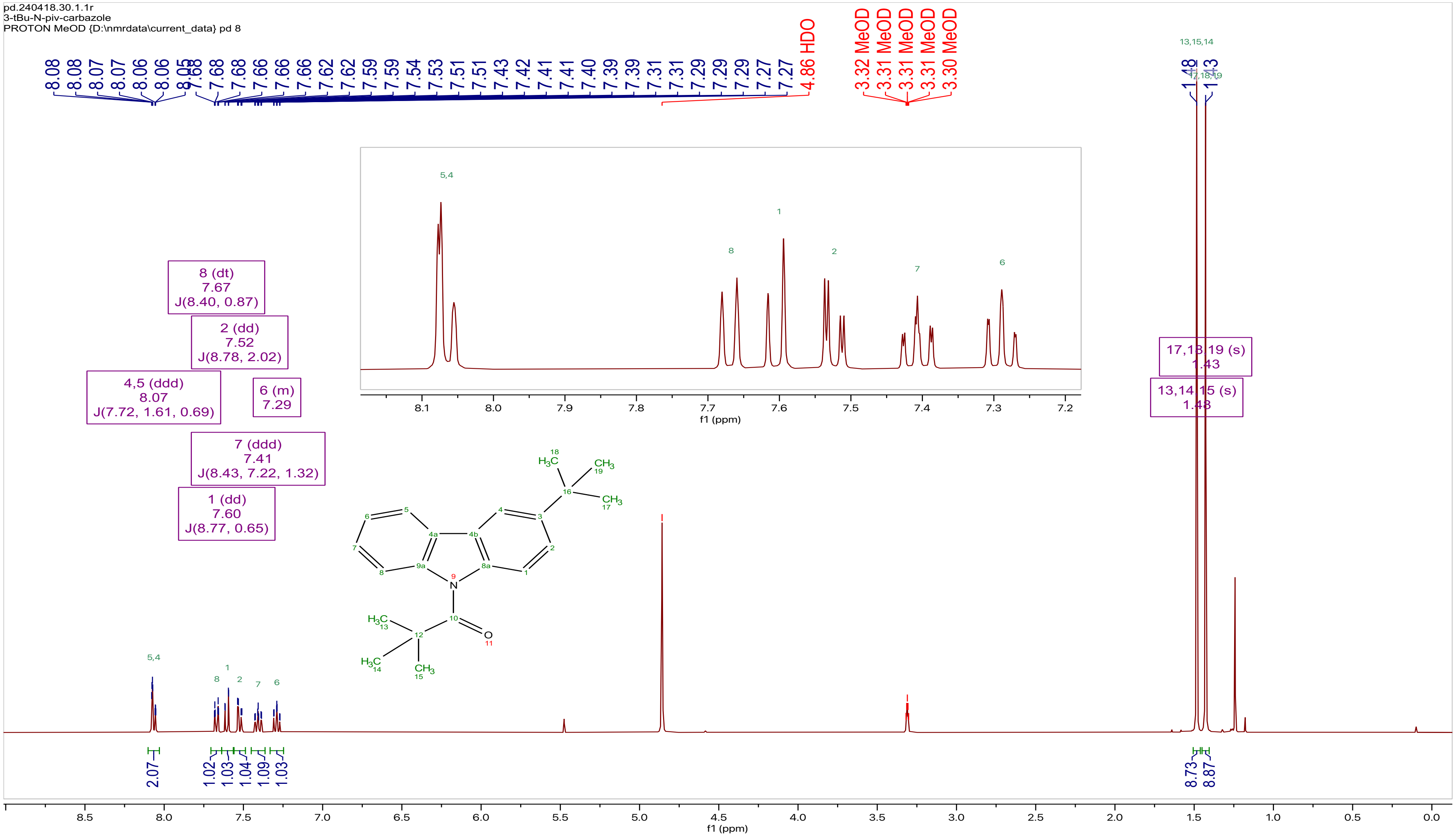

^{1}H NMR (400 MHz, MeOD) of 3c

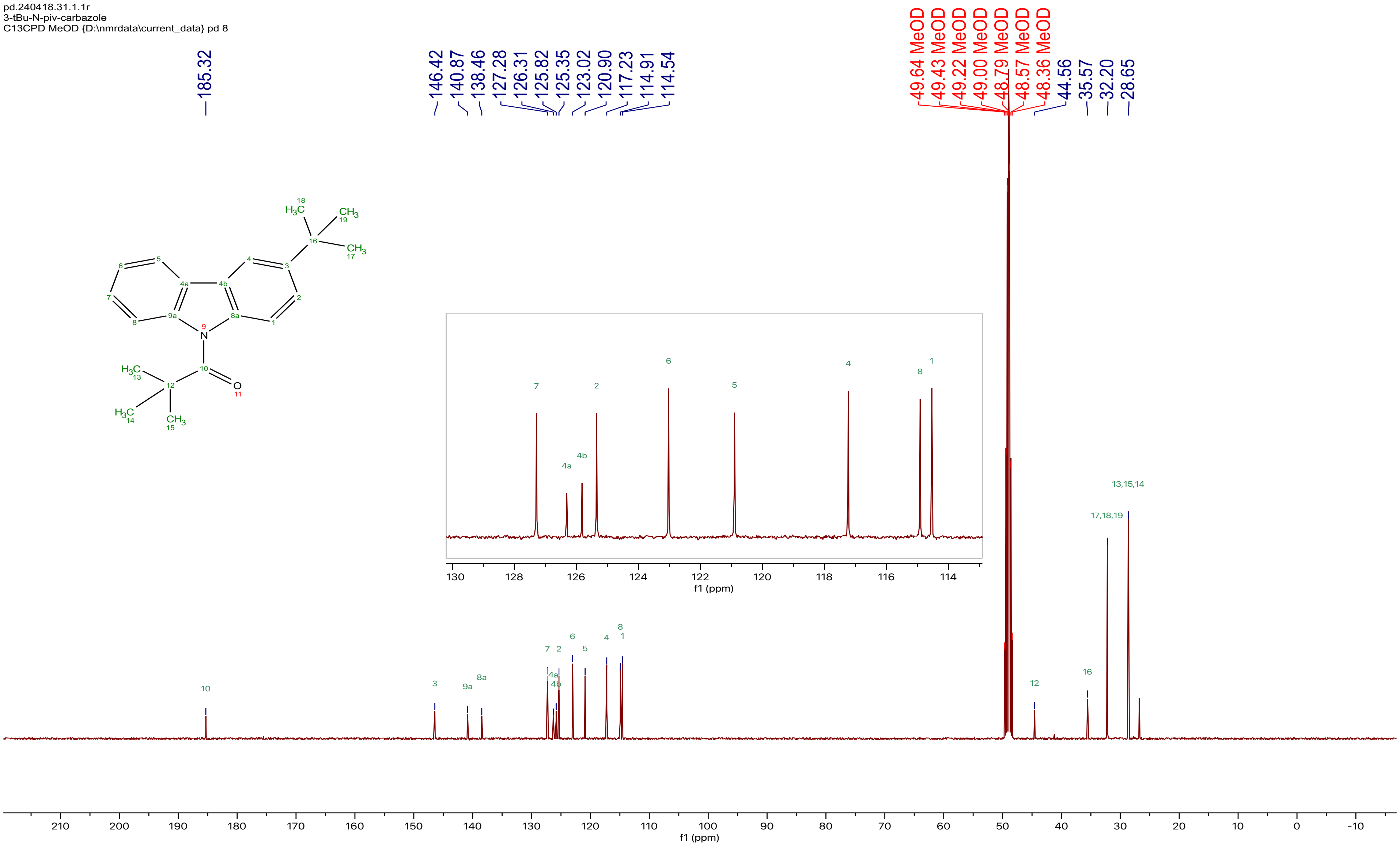

$^{13}C\{^1H\}$ NMR (101 MHz, MeOD) of 3c

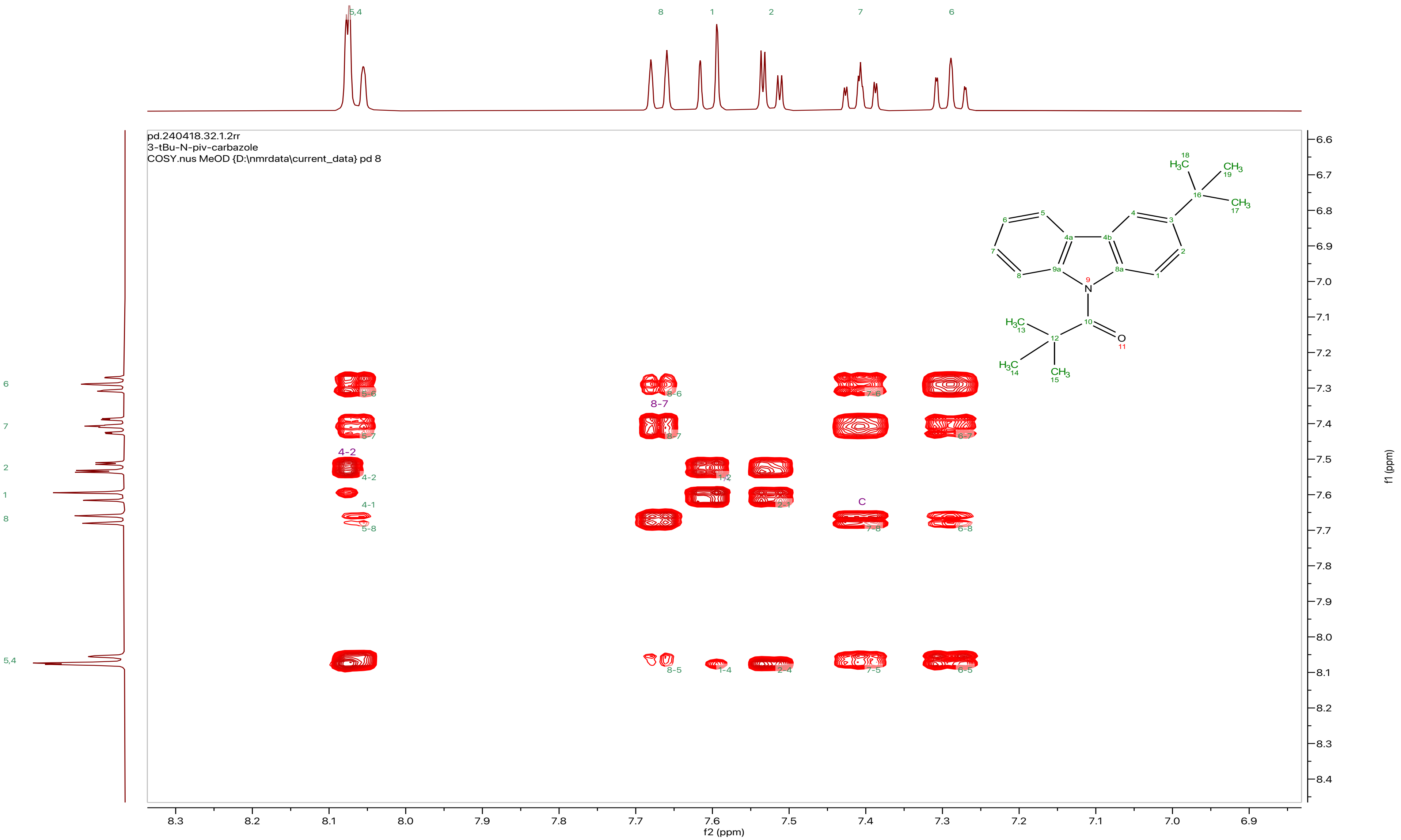

¹H-¹H COSY (400 MHz, MeOD) of 3c

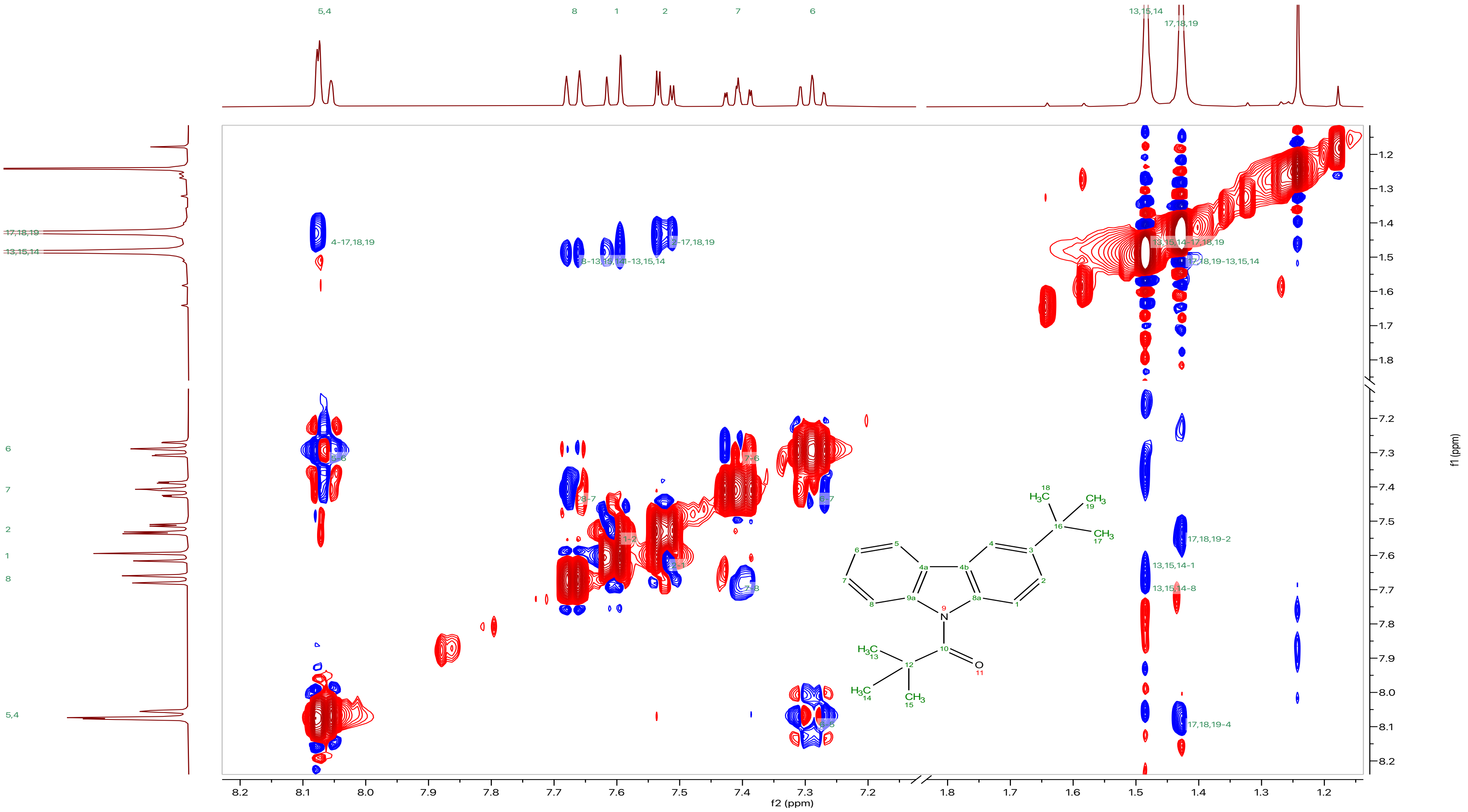

^{1}H-^{1}H NOESY (400 MHz, MeOD) of 3c

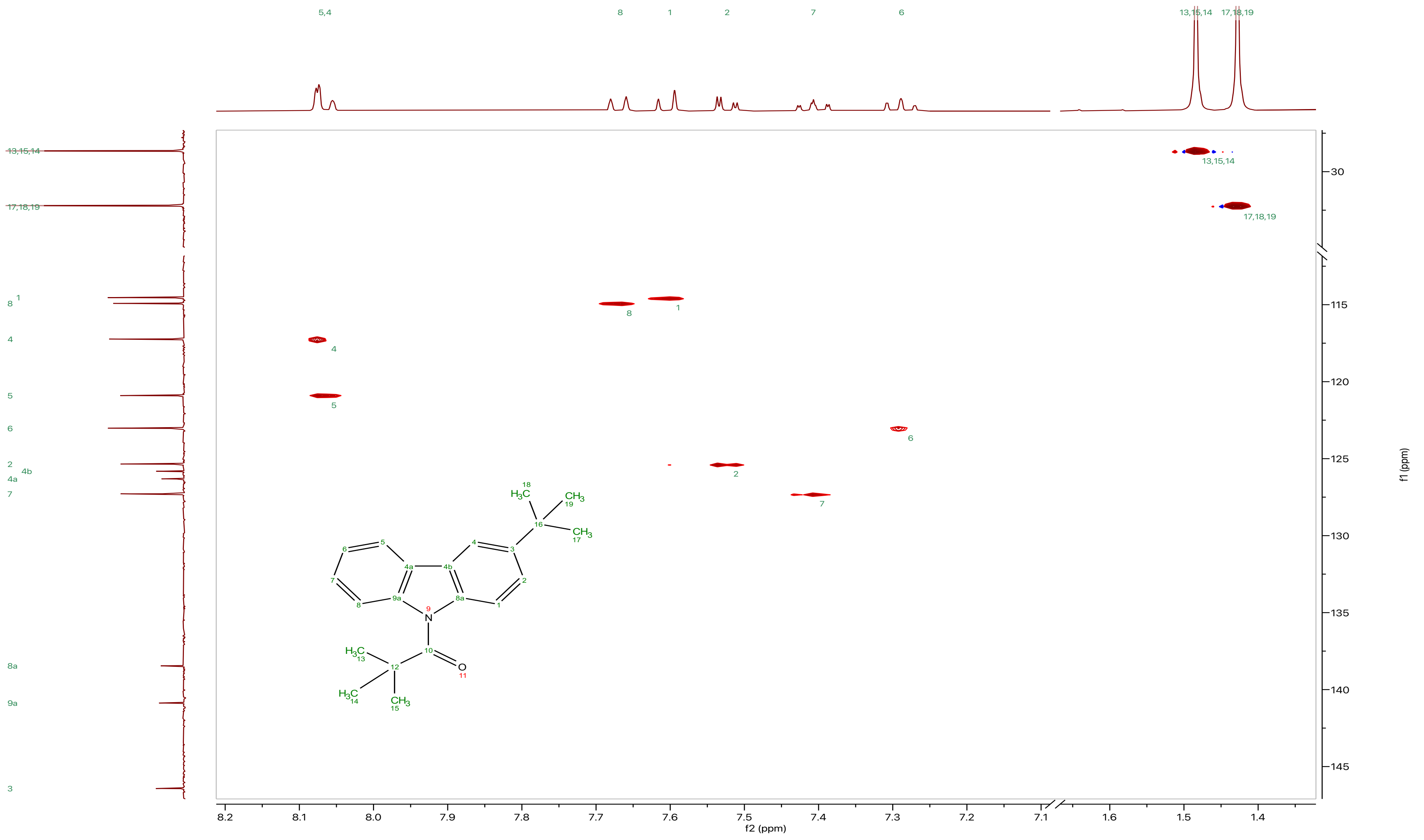

^{1}H-^{13}C{^{1}H} HSQC NMR (400/101 MHz, MeOD) of 3c

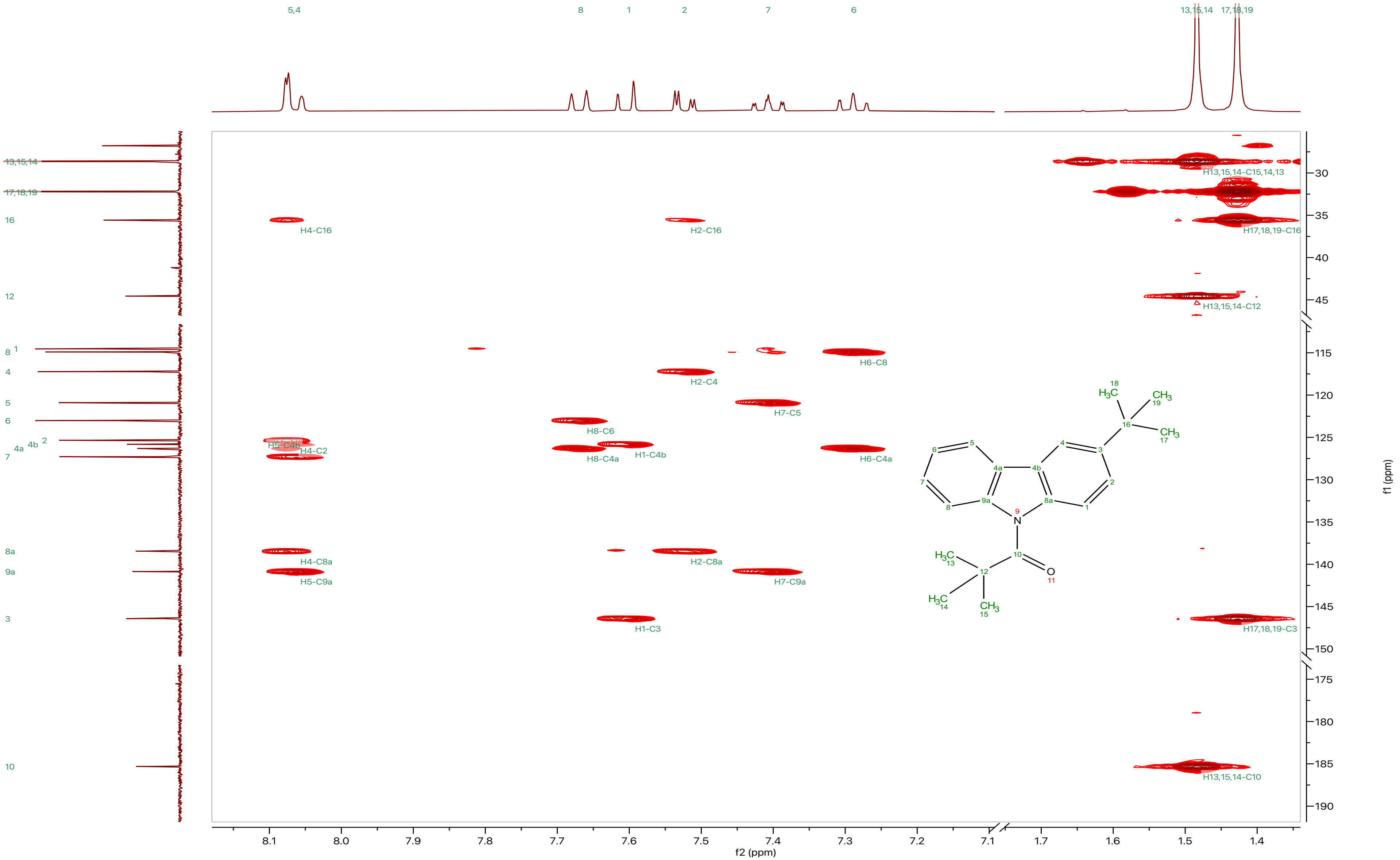

^{1}H-^{13}C{^{1}H} HMBC NMR (400/101 MHz, MeOD) of 3c

4ac

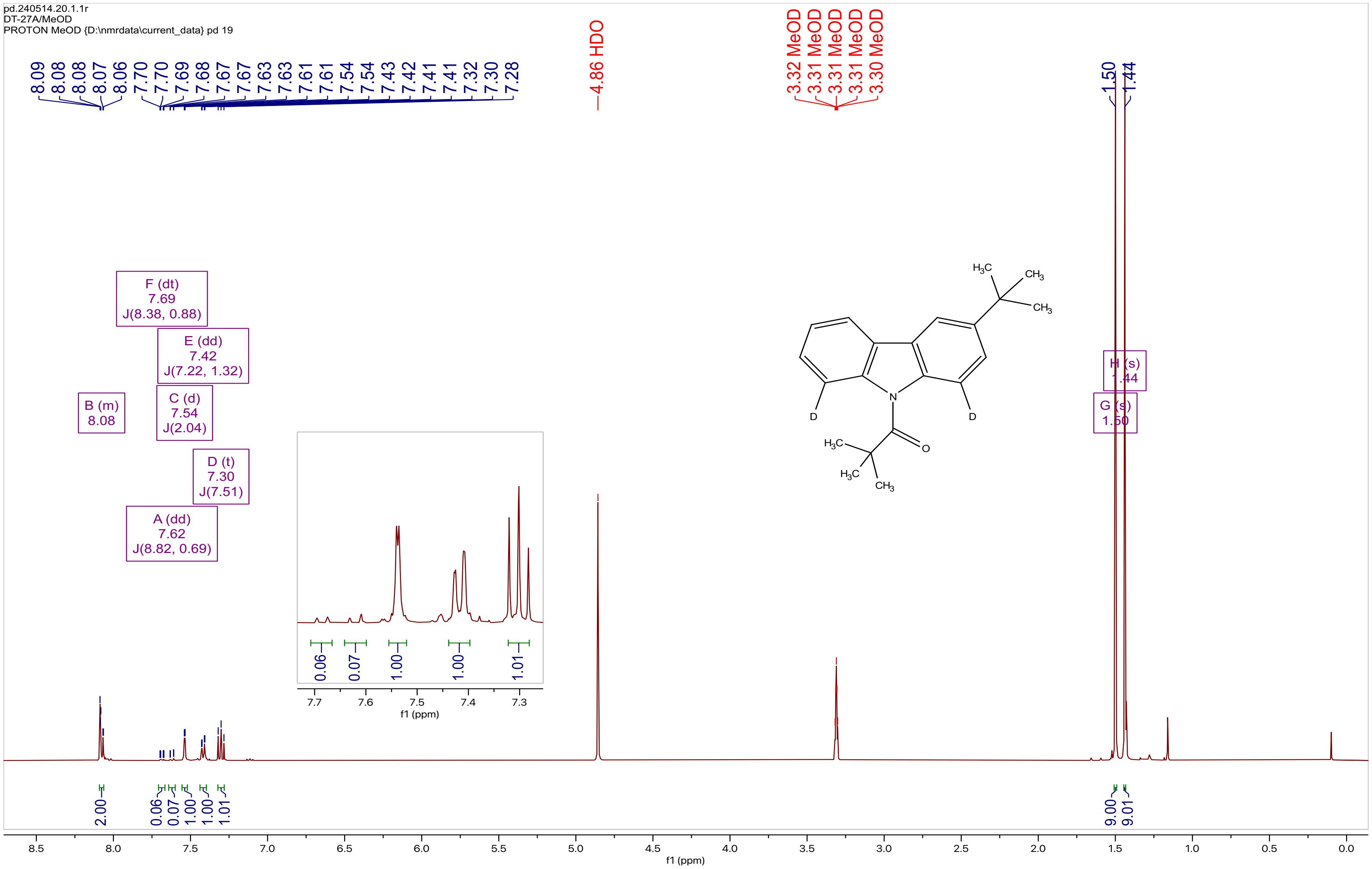

^{1}H NMR (400 MHz, MeOD) of 4ac

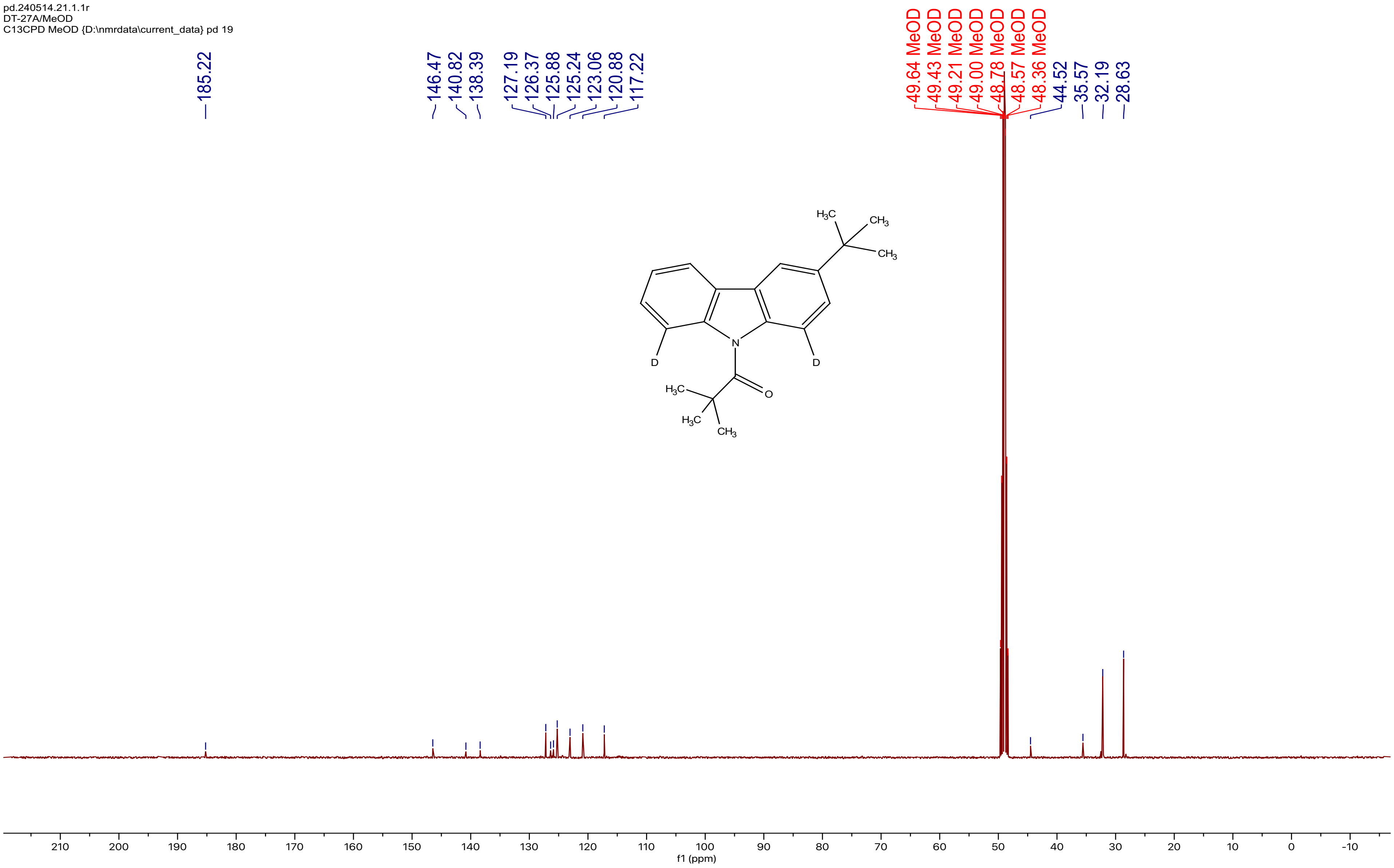

$^{13}C\{^{1}H\}$ NMR (101 MHz, MeOD) of 4ac

3c’

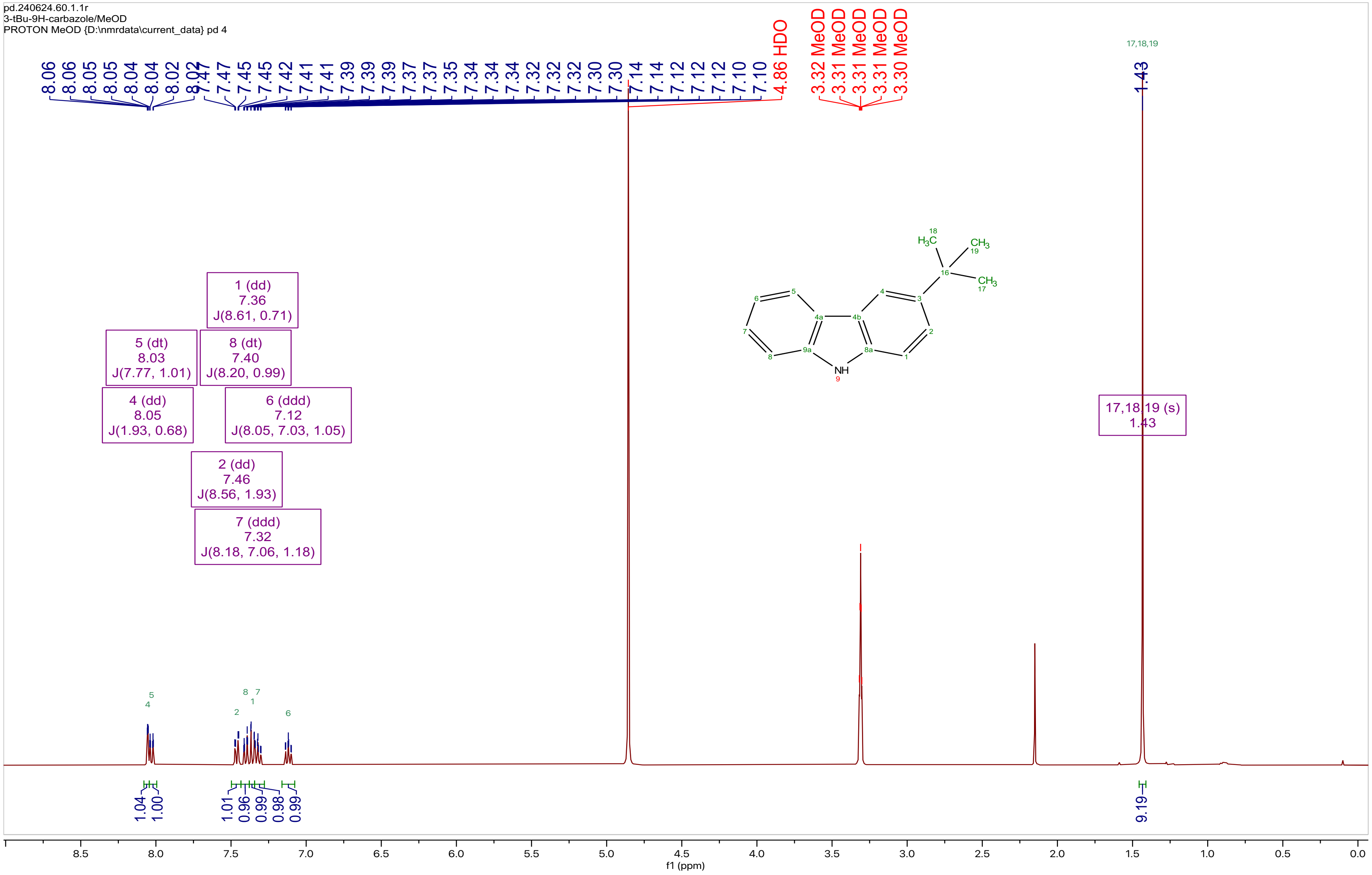

^{1}H NMR (400 MHz, MeOD) of 3c’

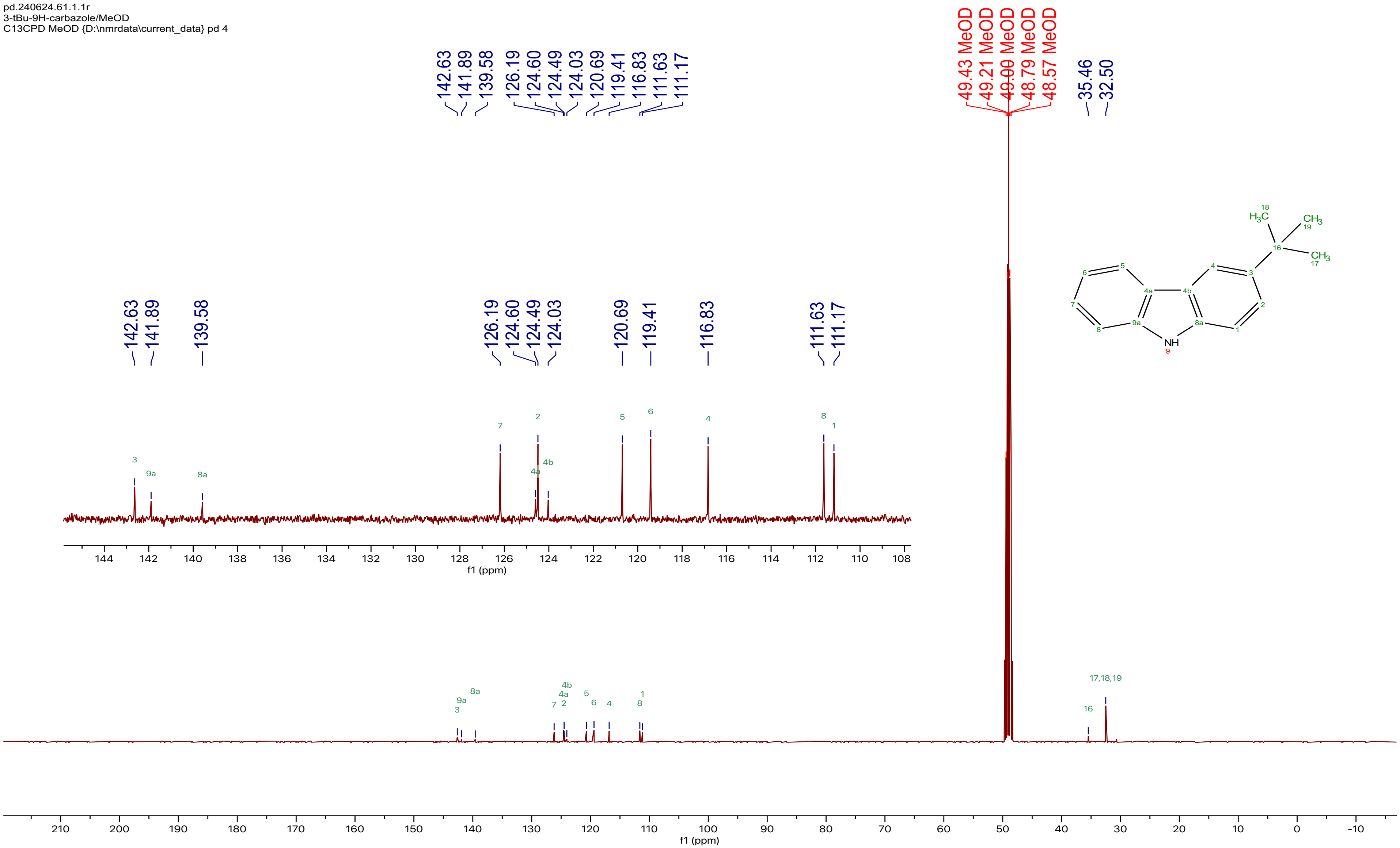

$^{13}C\{^{1}H\}$ NMR (101 MHz, MeOD) of 3c'

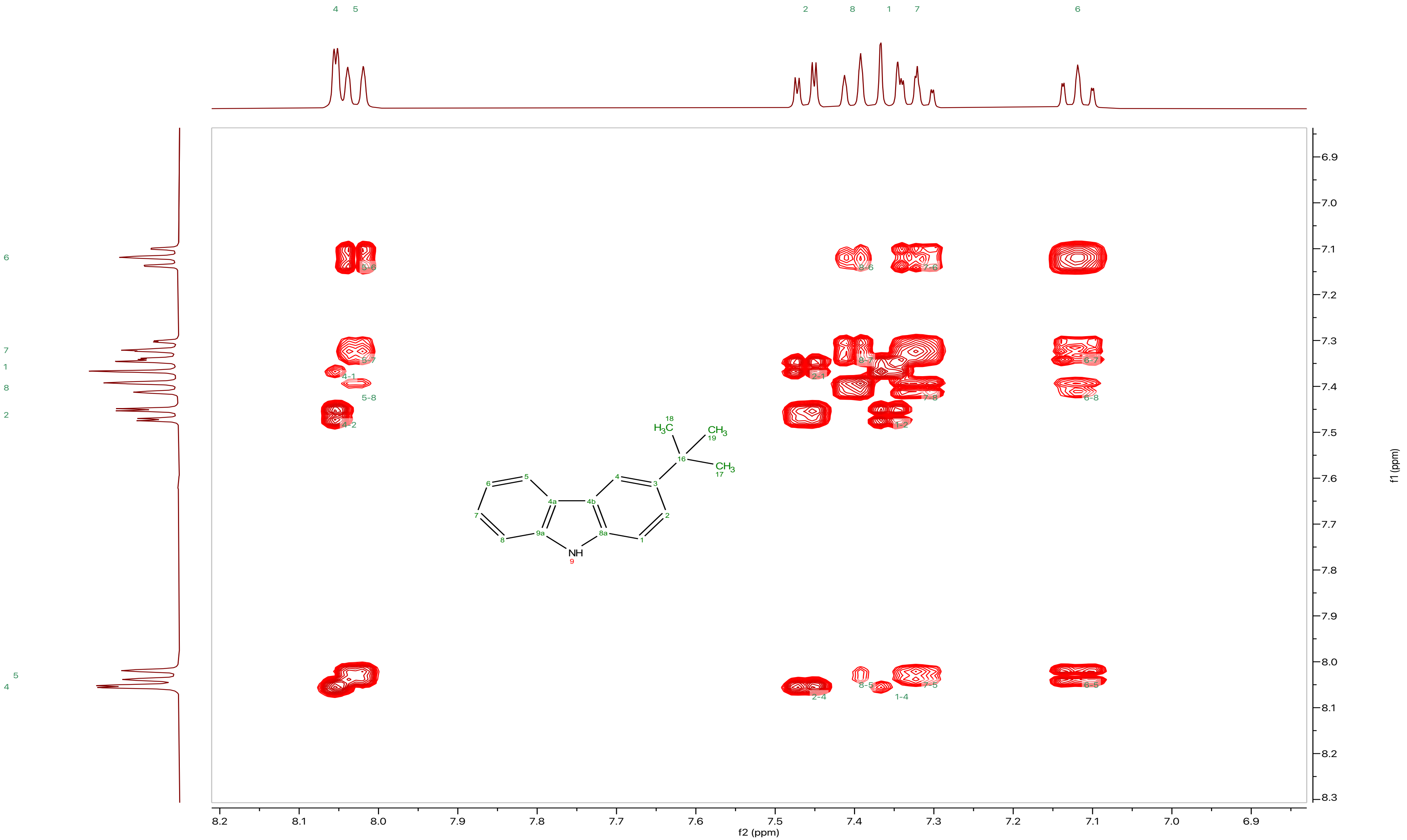

^1H-^1H COSY (400 MHz, MeOD) of 3c'

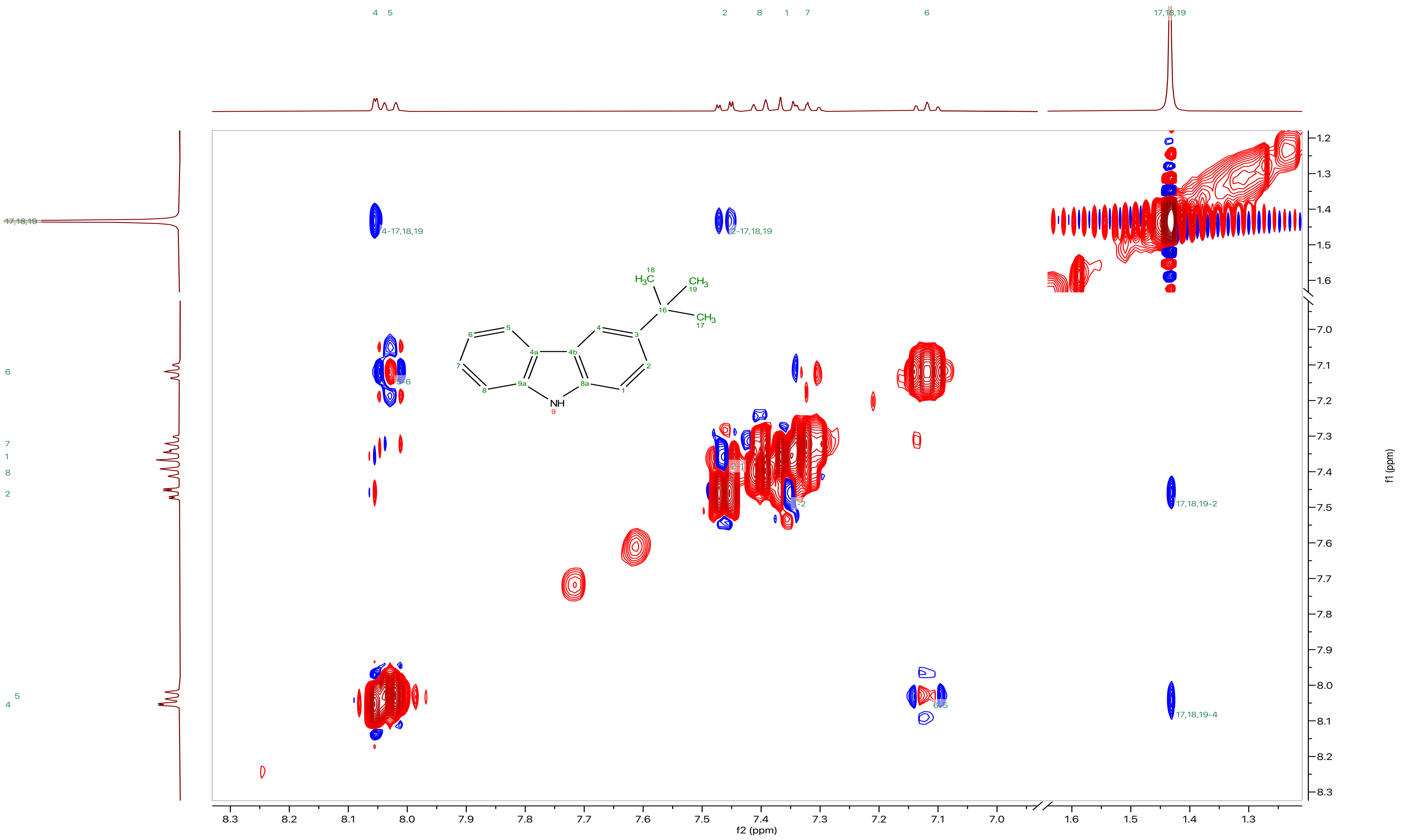

^1H-^1H NOESY (400 MHz, MeOD) of 3c'

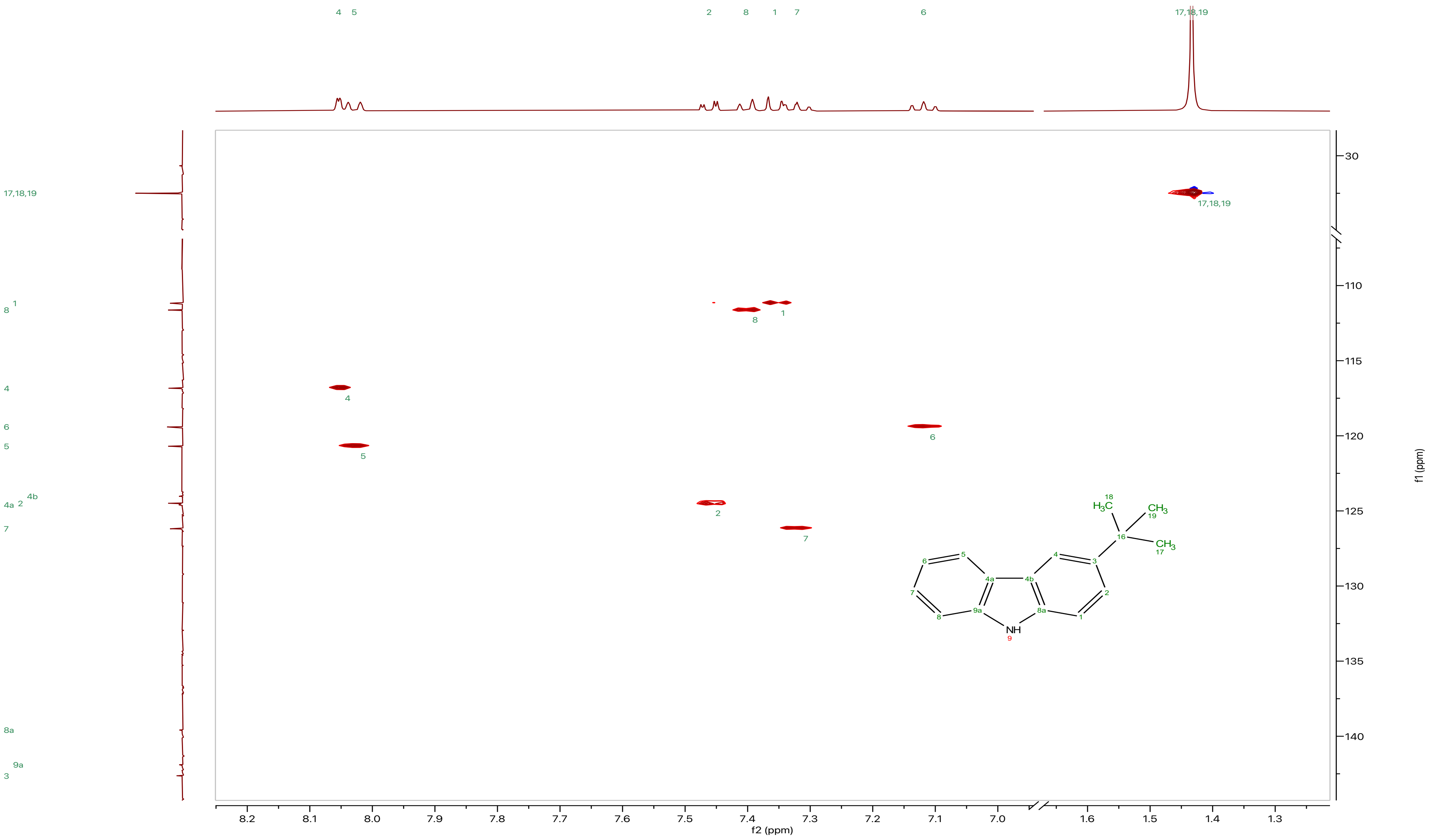

^{1}H-$^{13}C\{^{1}H\}$ HSQC NMR (400/101 MHz, MeOD) of 3c'

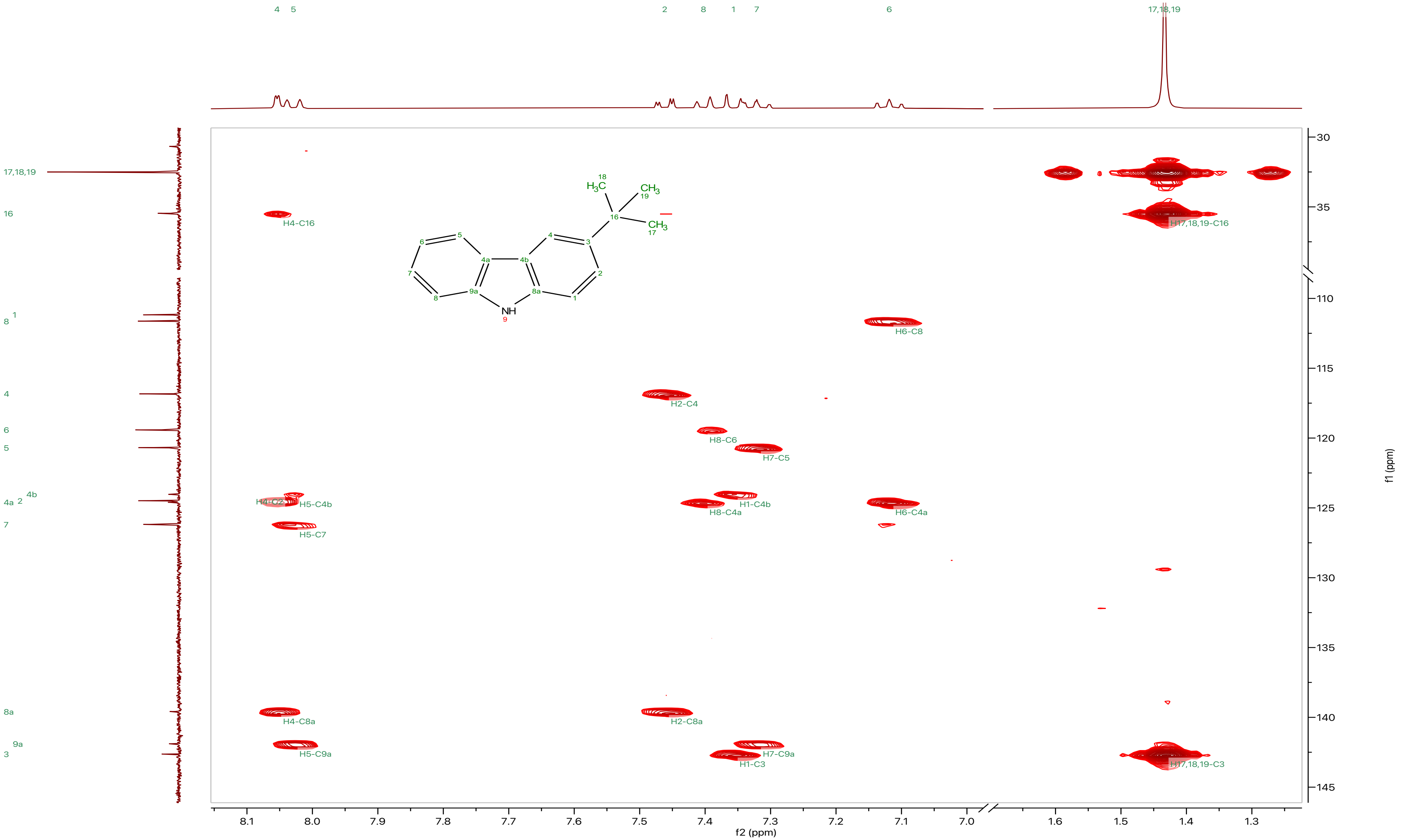

^1H-$^{13}C\{^1H\}$ HMBC NMR (400/101 MHz, MeOD) of 3c'

4bc

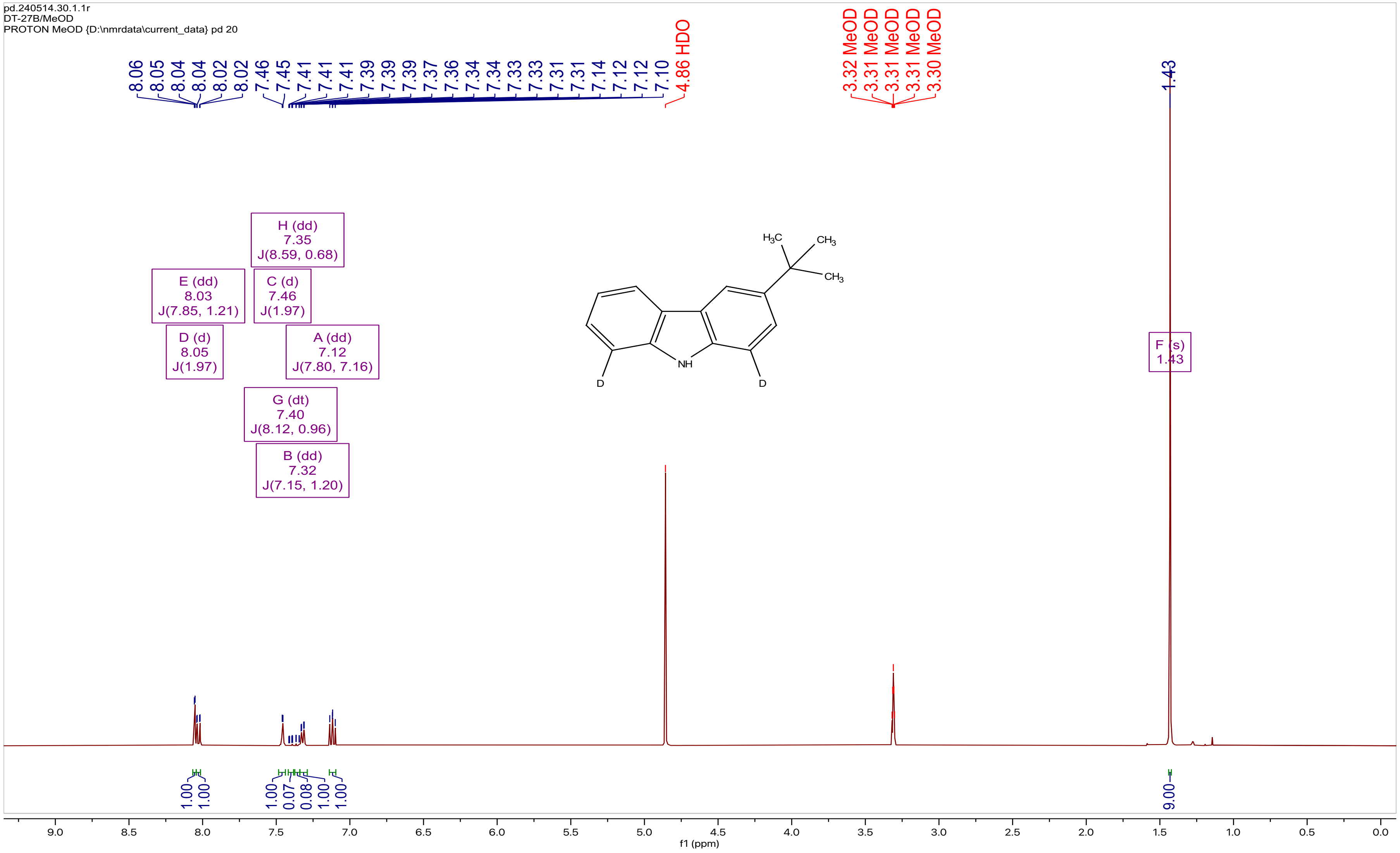

^{1}H NMR (400 MHz, MeOD) of 4bc

pd.240514.31.1.1r
DT-27B/MeOD
C13CPD MeOD {D:\nmrdata\current_data} pd 20

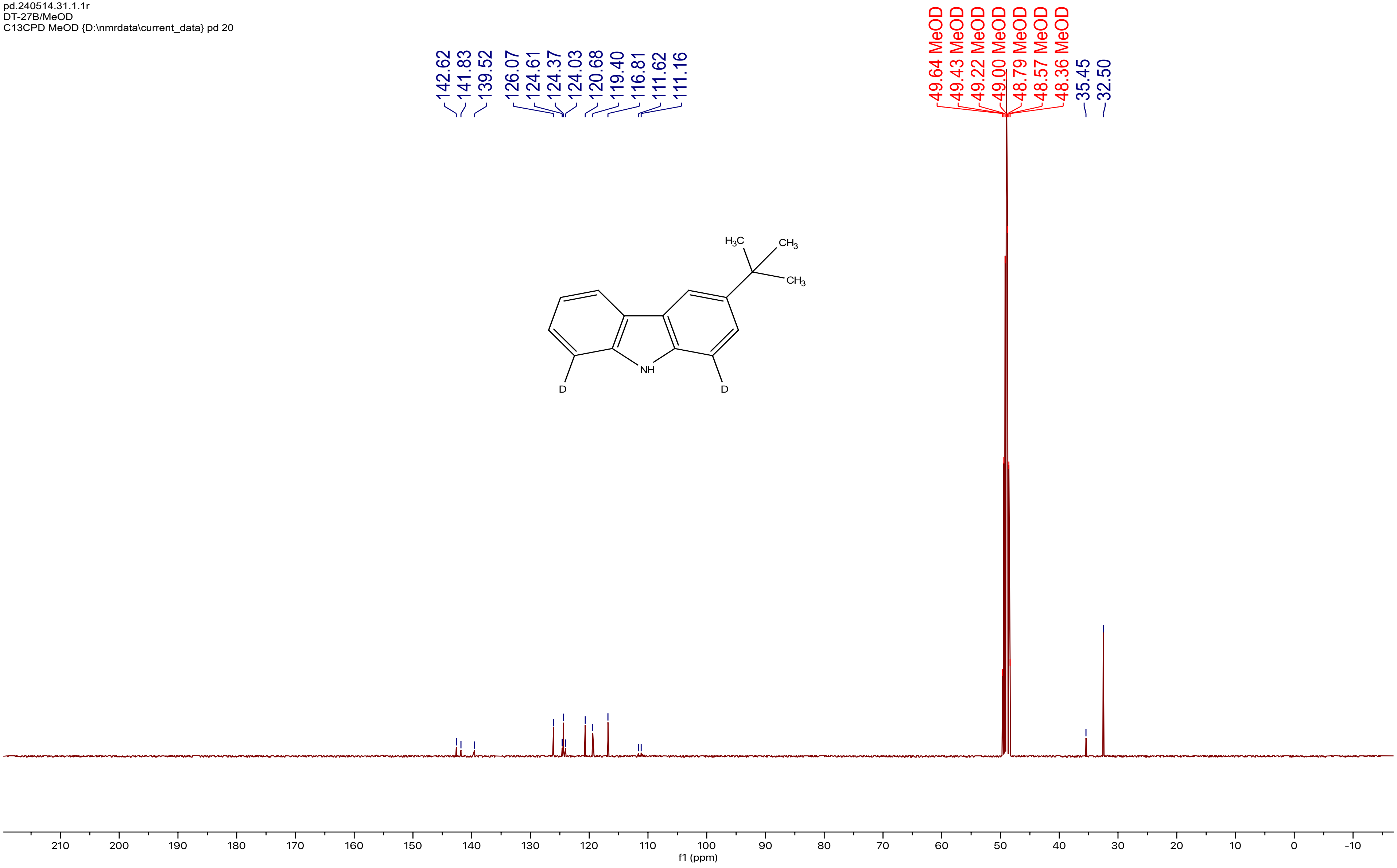

$^{13}C\{^{1}H\}$ NMR (101 MHz, MeOD) of 4bc

3d

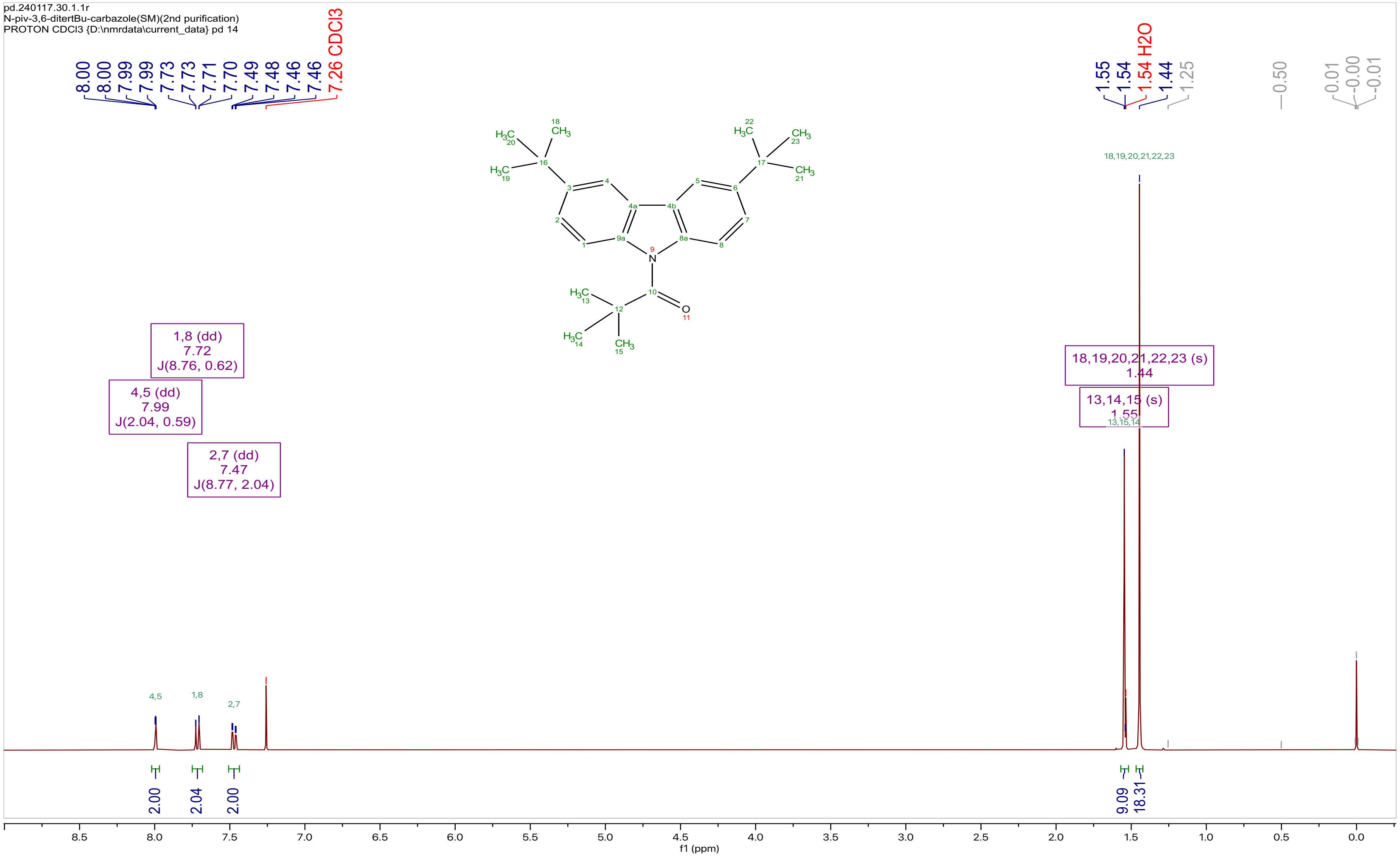

^{1}H NMR (400 MHz, $CDCl_3$) of 3d

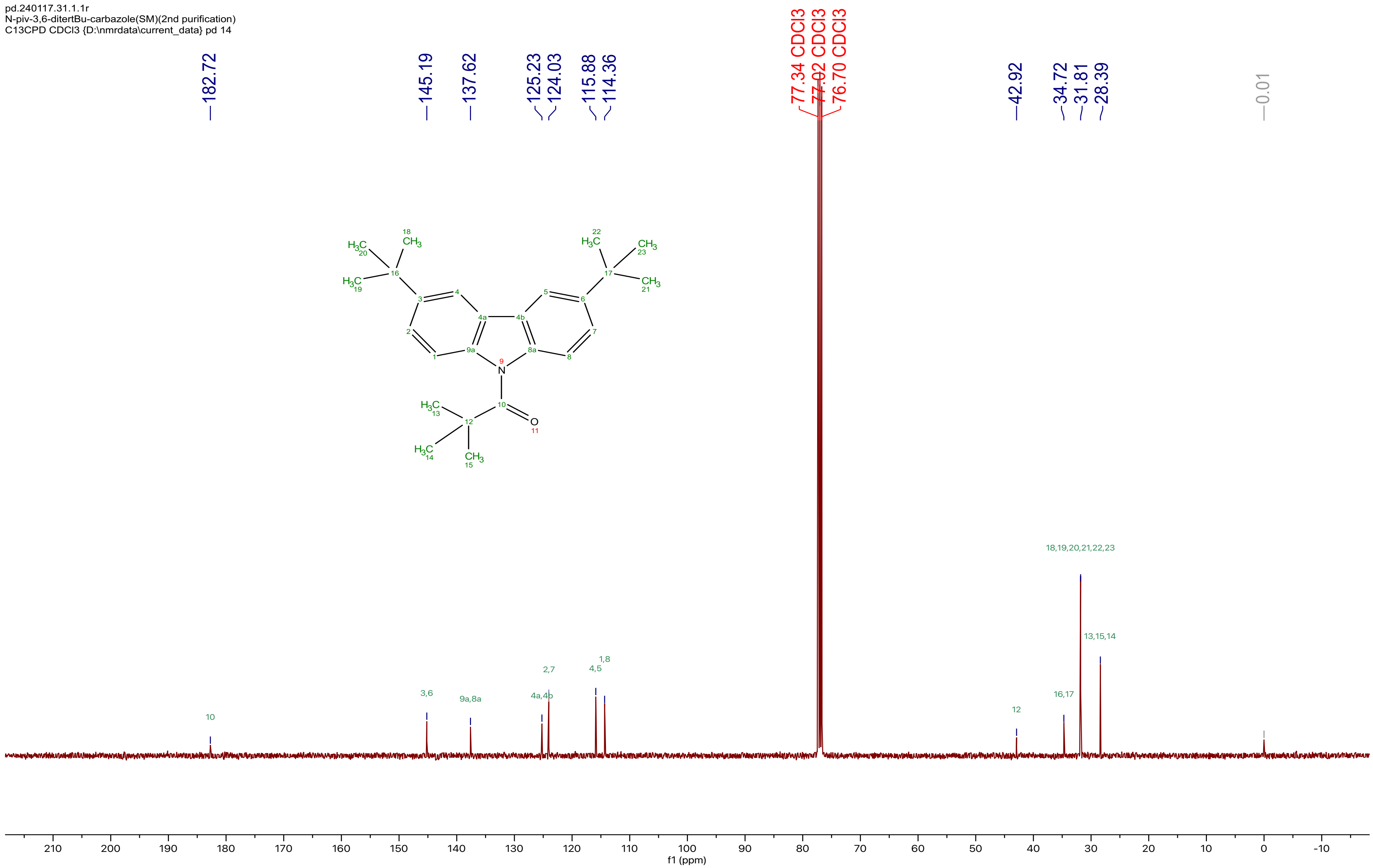

$^{13}C\{^{1}H\}$ NMR (101 MHz, $CDCl_3$) of 3d

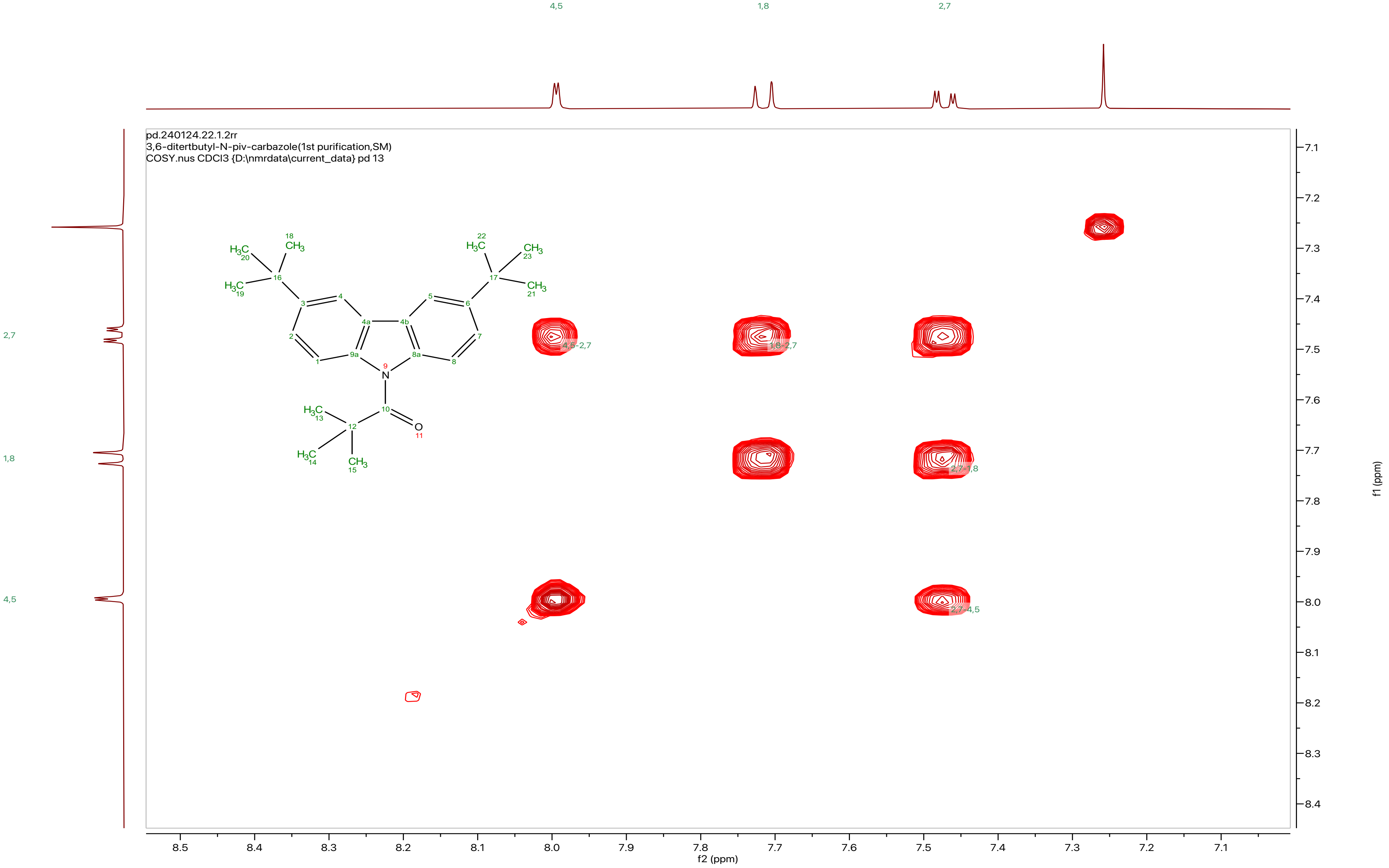

^{1}H-^{1}H COSY (400 MHz, $CDCl_3$) of 3d

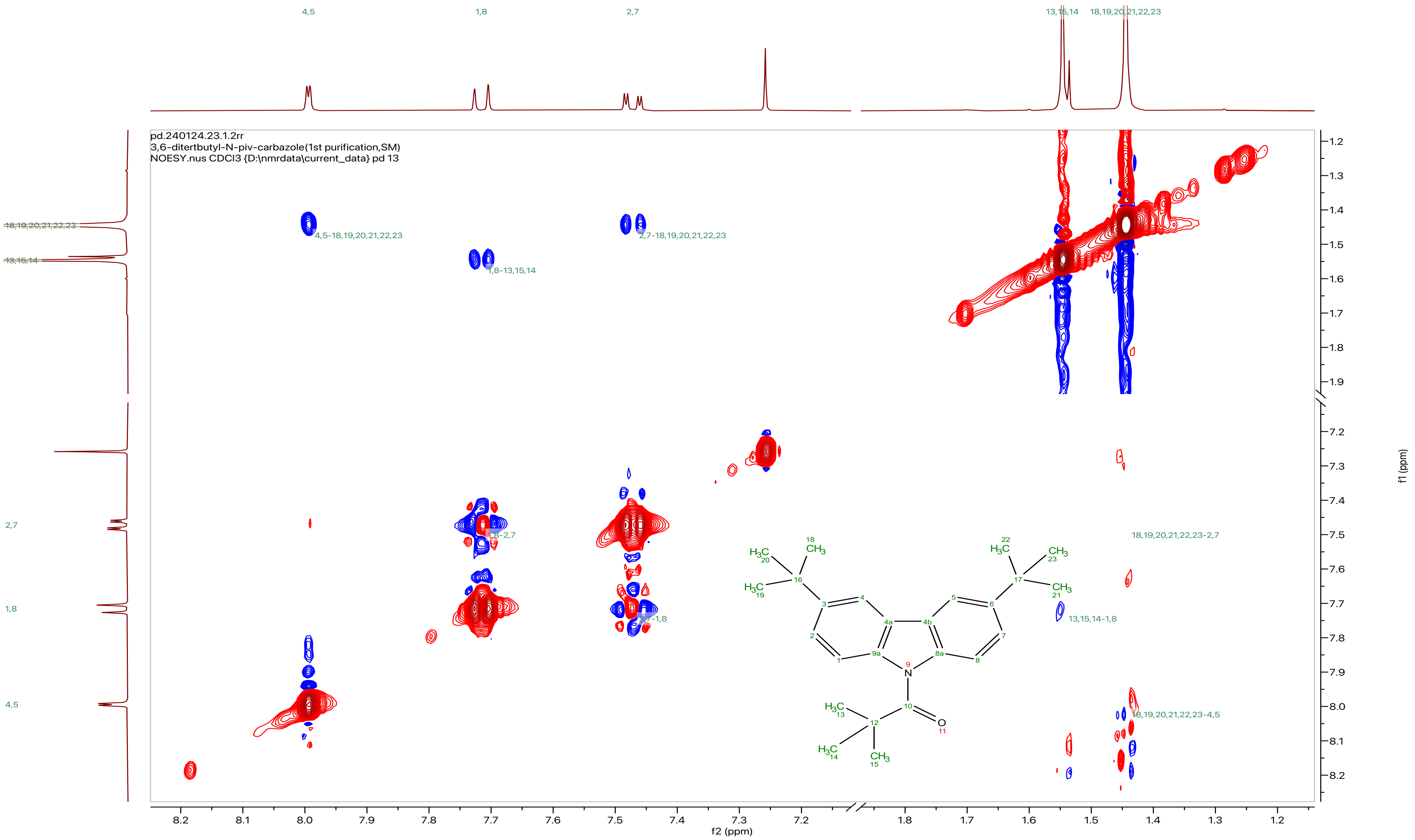

^1H-^1H NOESY (400 MHz, $CDCl_3$) of 3d

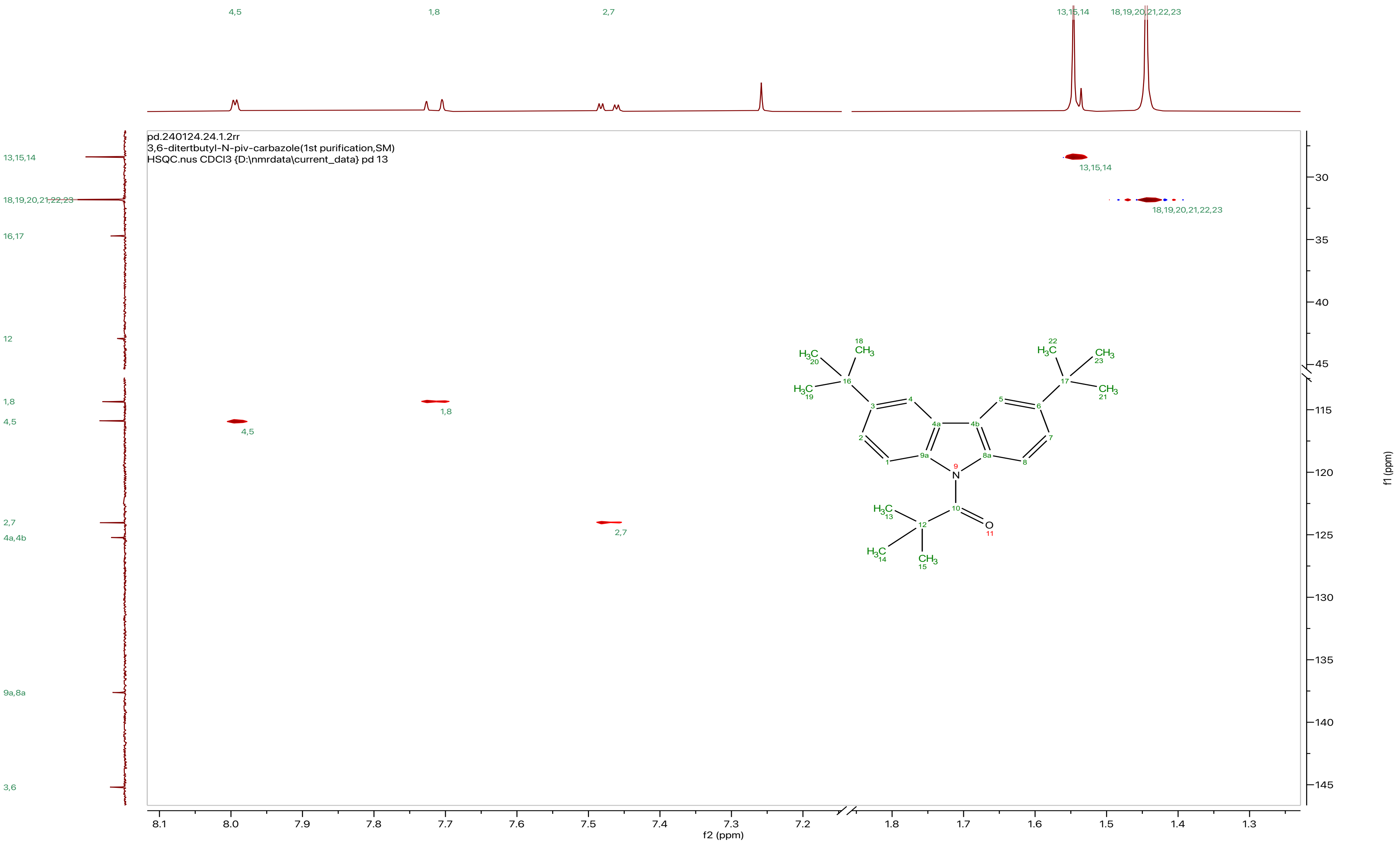

^{1}H-^{13}C{^{1}H} HSQC NMR (400/101 MHz, $CDCl_3$) of 3d

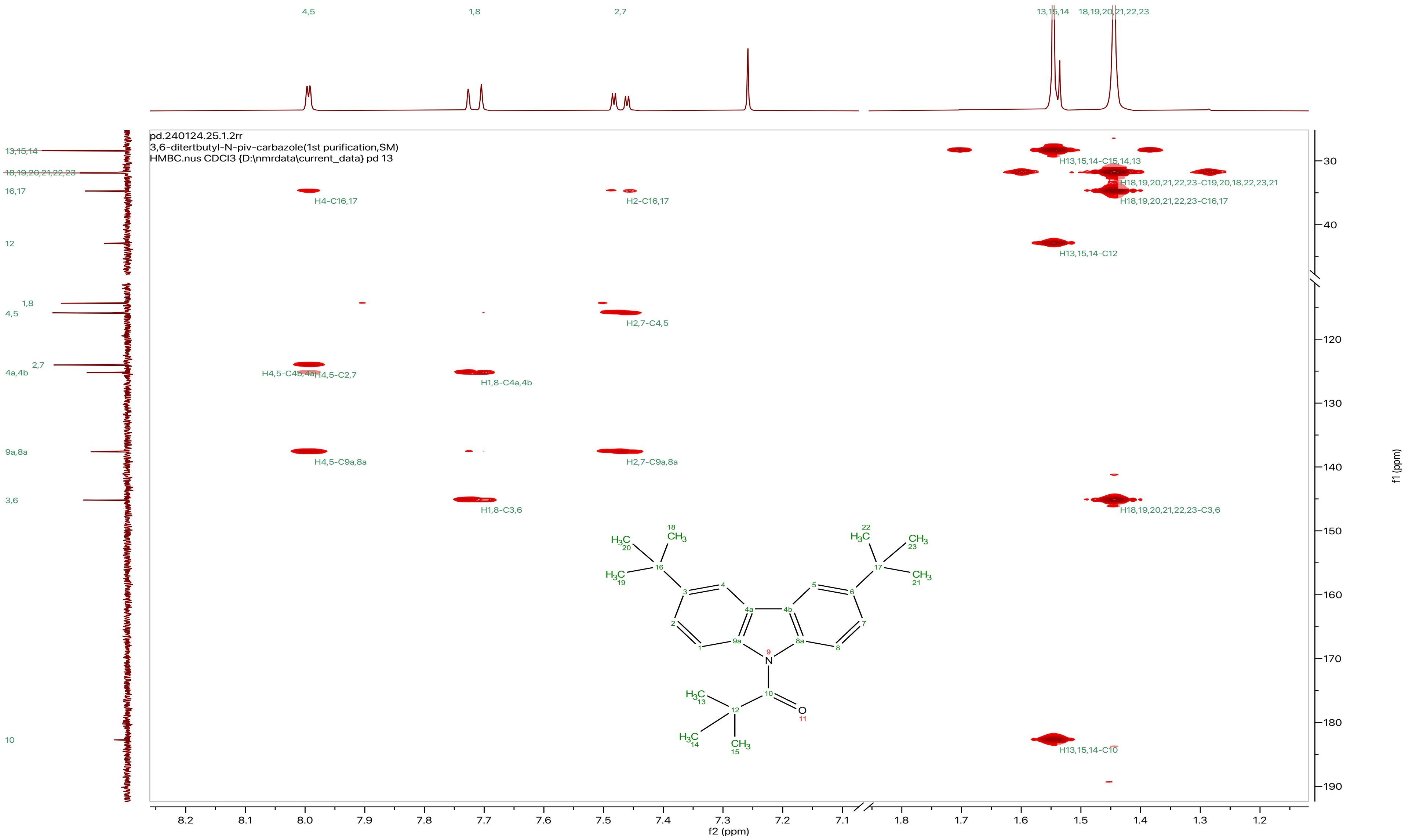

^{1}H-^{13}C{^{1}H} HMBC NMR (400/101 MHz, $CDCl_3$) of 3d

4ad

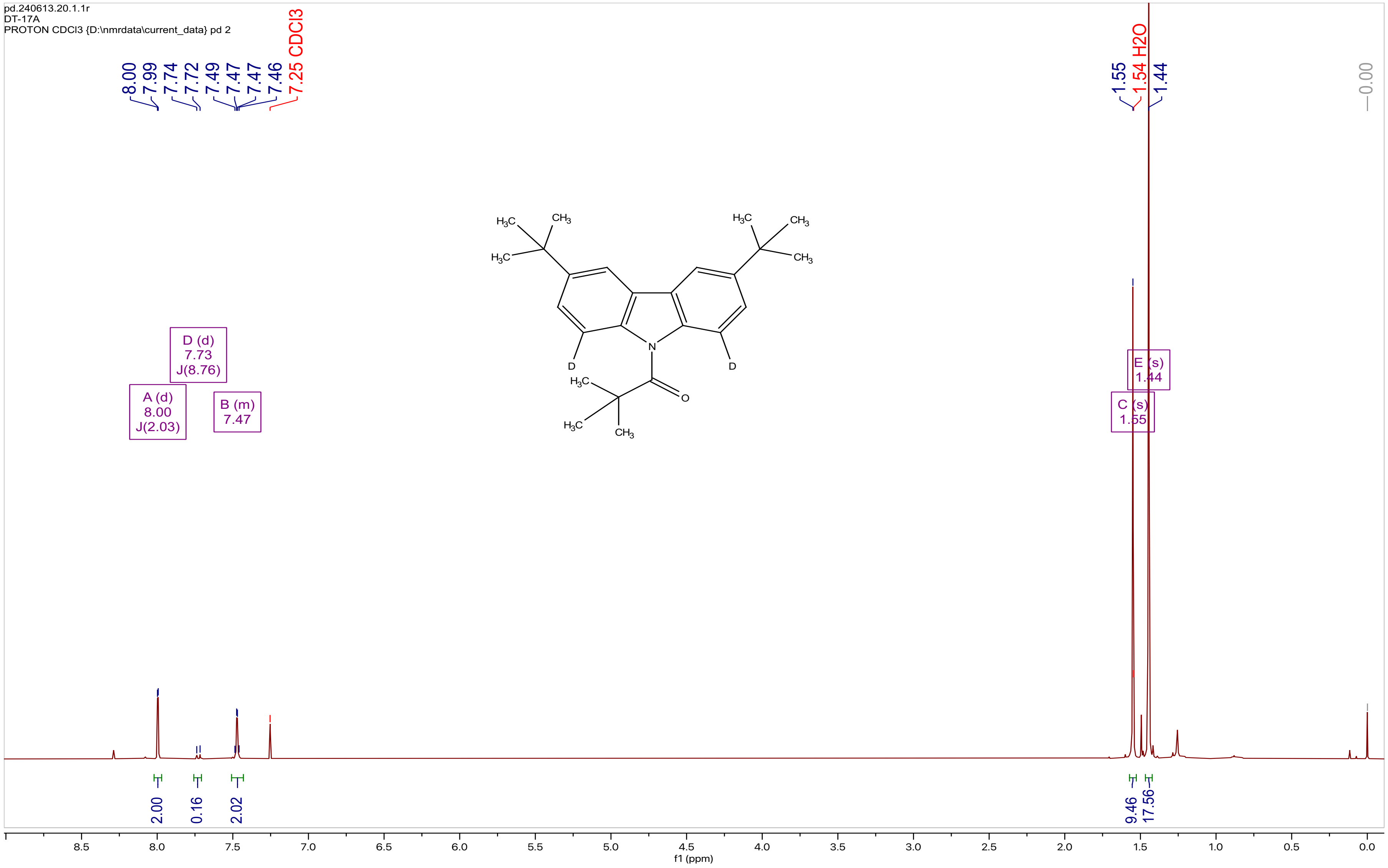

^{1}H NMR (400 MHz, $CDCl_3$) of 4ad

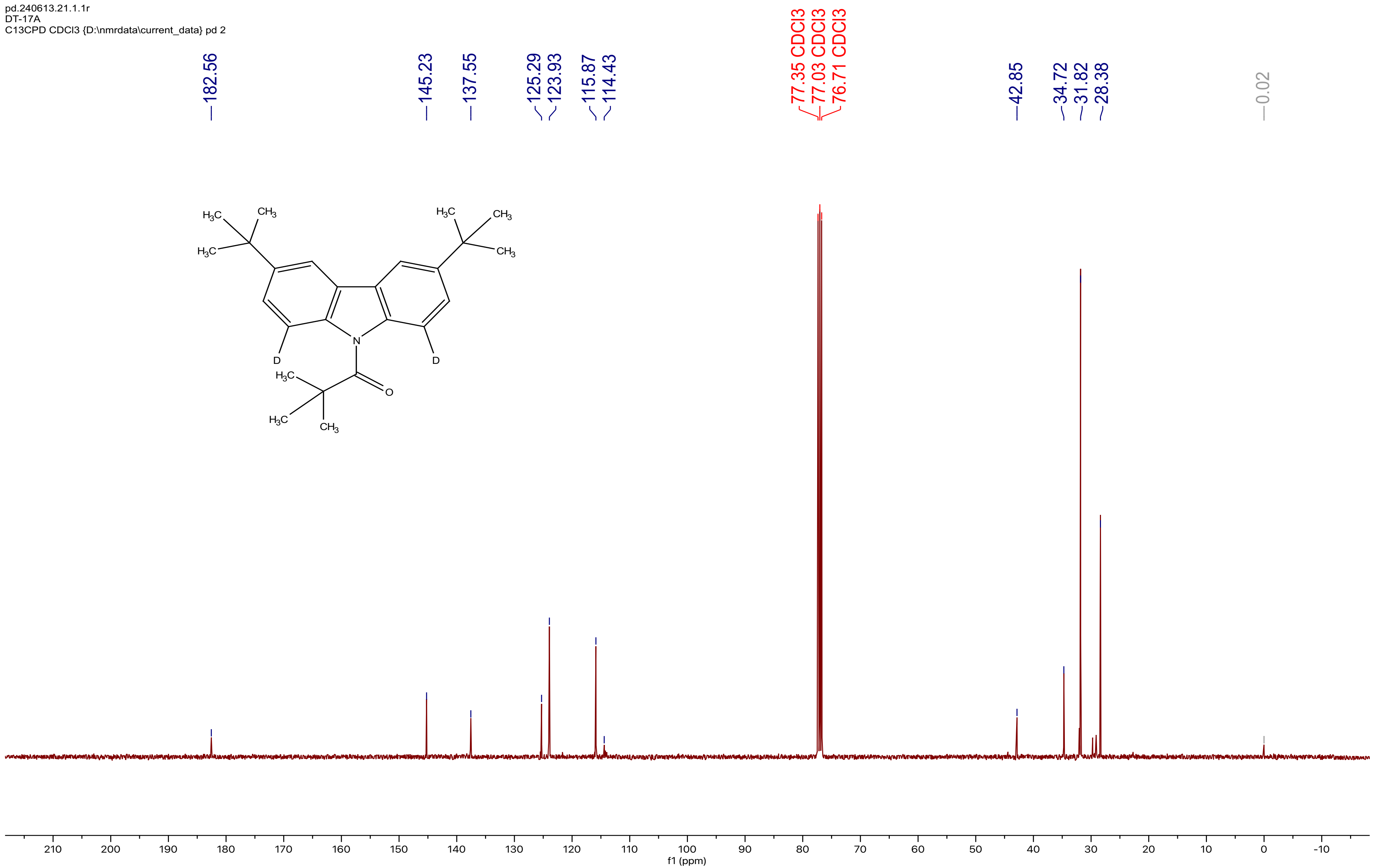

$^{13}C\{^1H\}$ NMR (101 MHz, $CDCl_3$) of 4ad

3d'

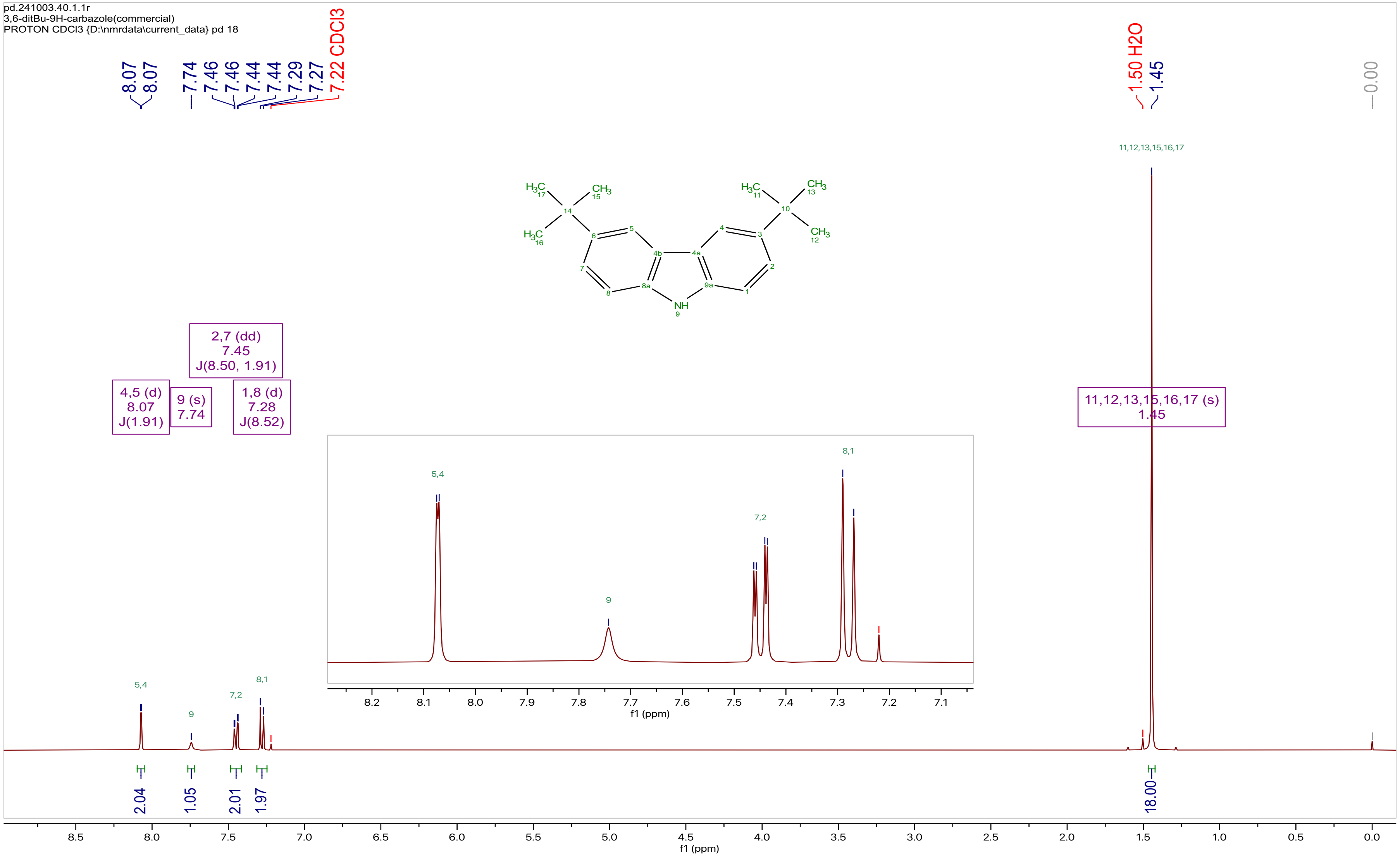

^1H NMR (400 MHz, $CDCl_3$) of 3d'

pd.241003.41.1.1r
3,6-ditBu-9H-carbazole(commercial)
C13CPD CDCl3 {D:\nmrdata\current_data} pd 18

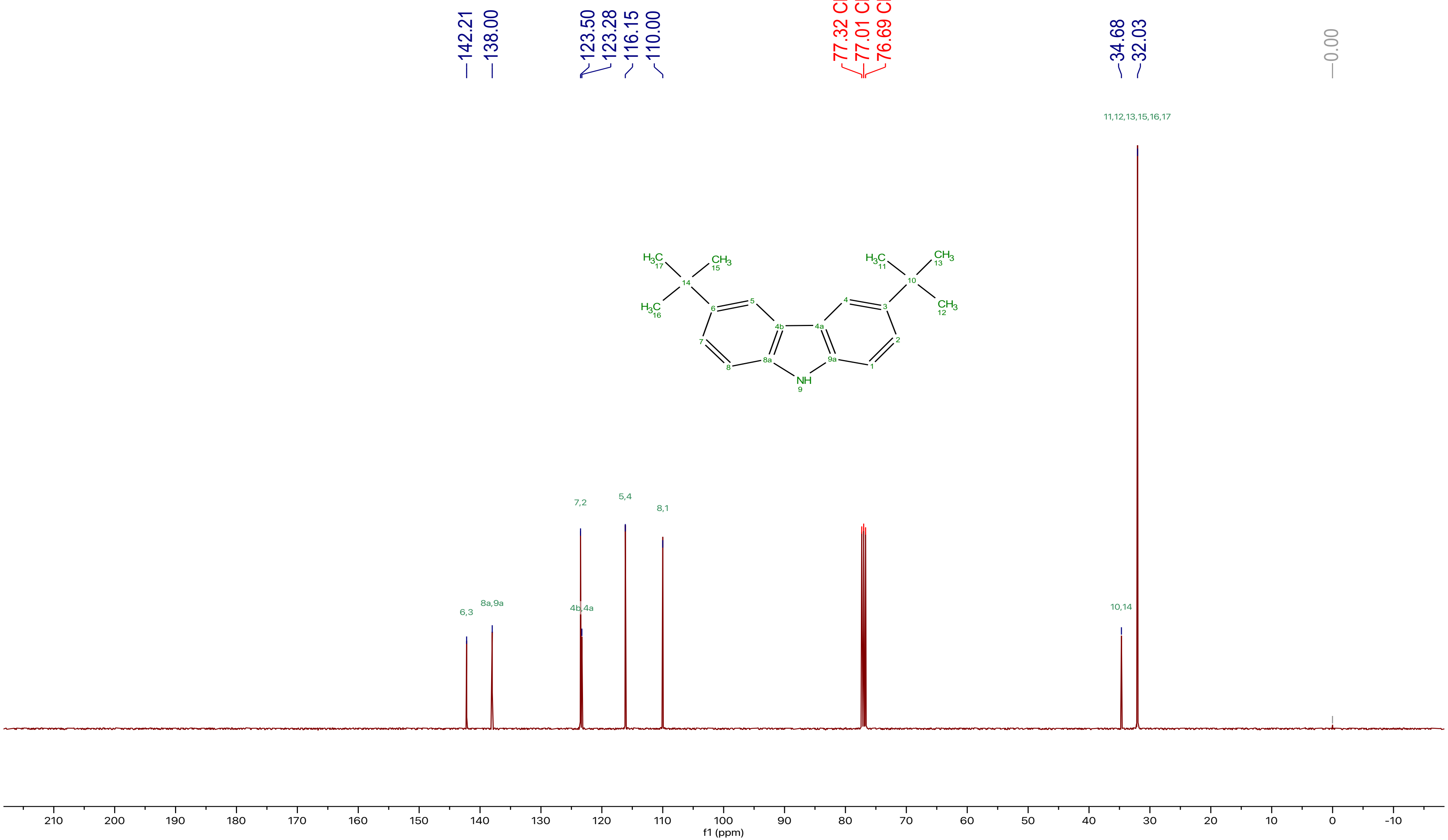

$^{13}C\{^1H\}$ NMR (101 MHz, $CDCl_3$) of 3d'

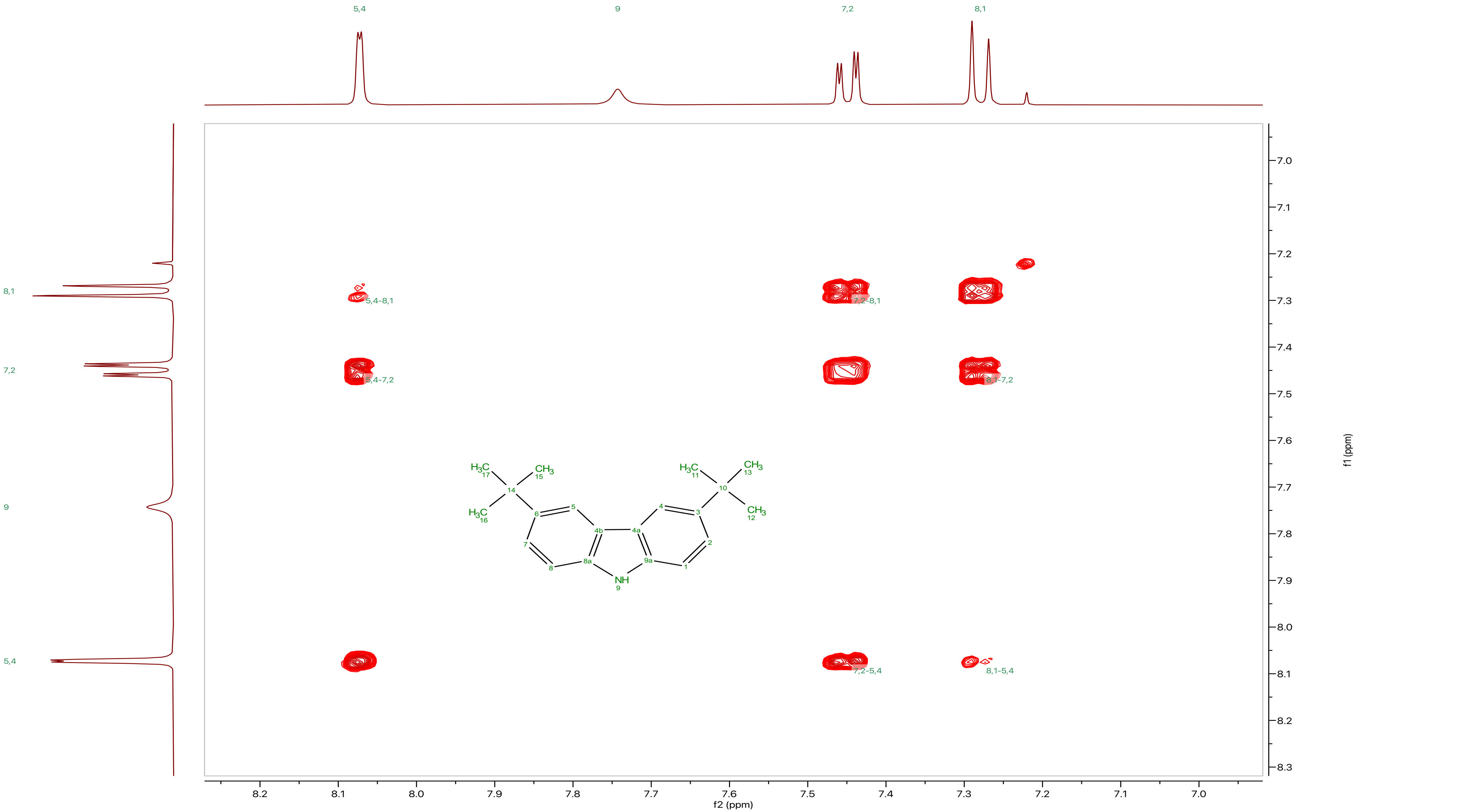

^1H-^1H COSY (400 MHz, $CDCl_3$) of 3d'

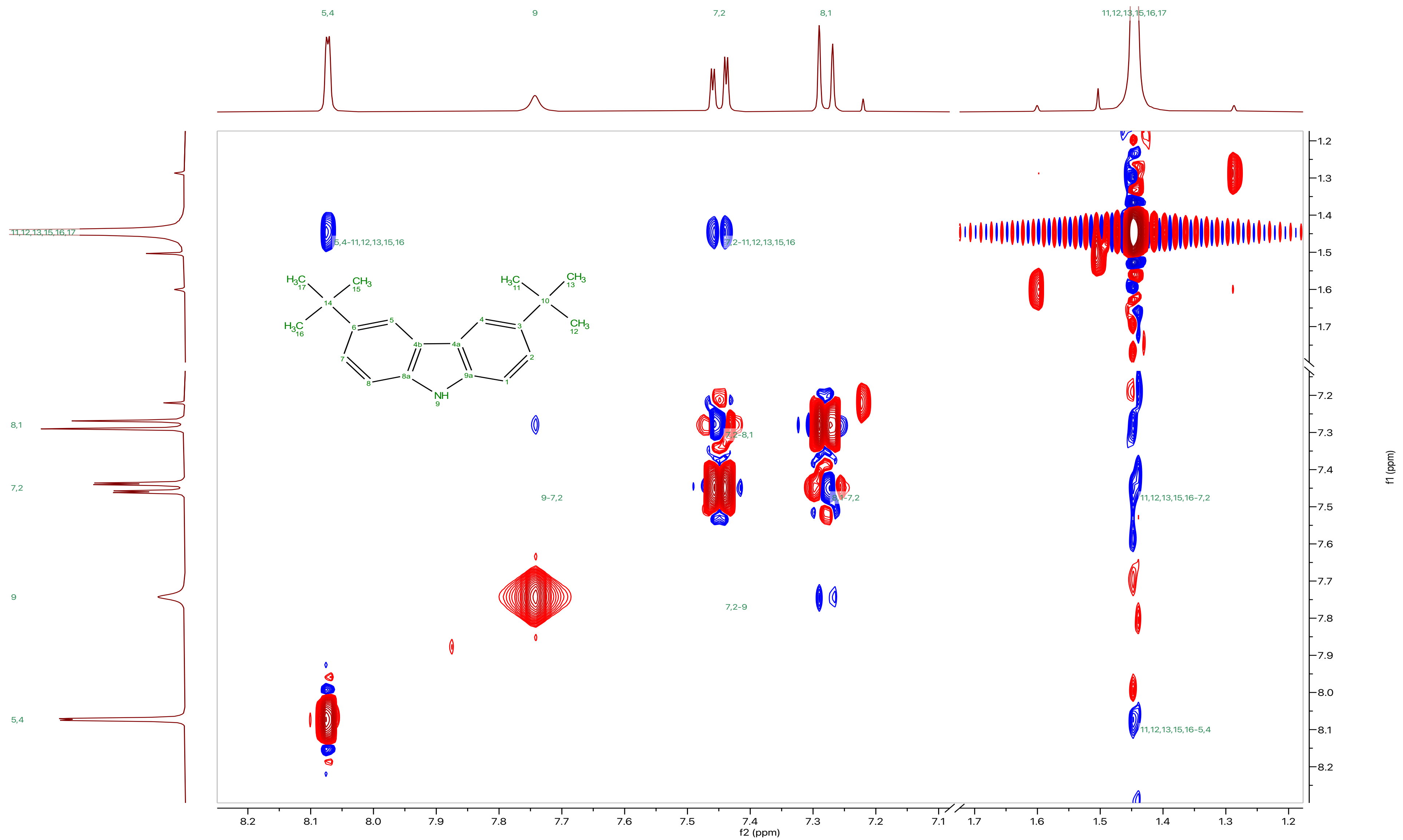

^1H-^1H NOESY (400 MHz, $CDCl_3$) of 3d'

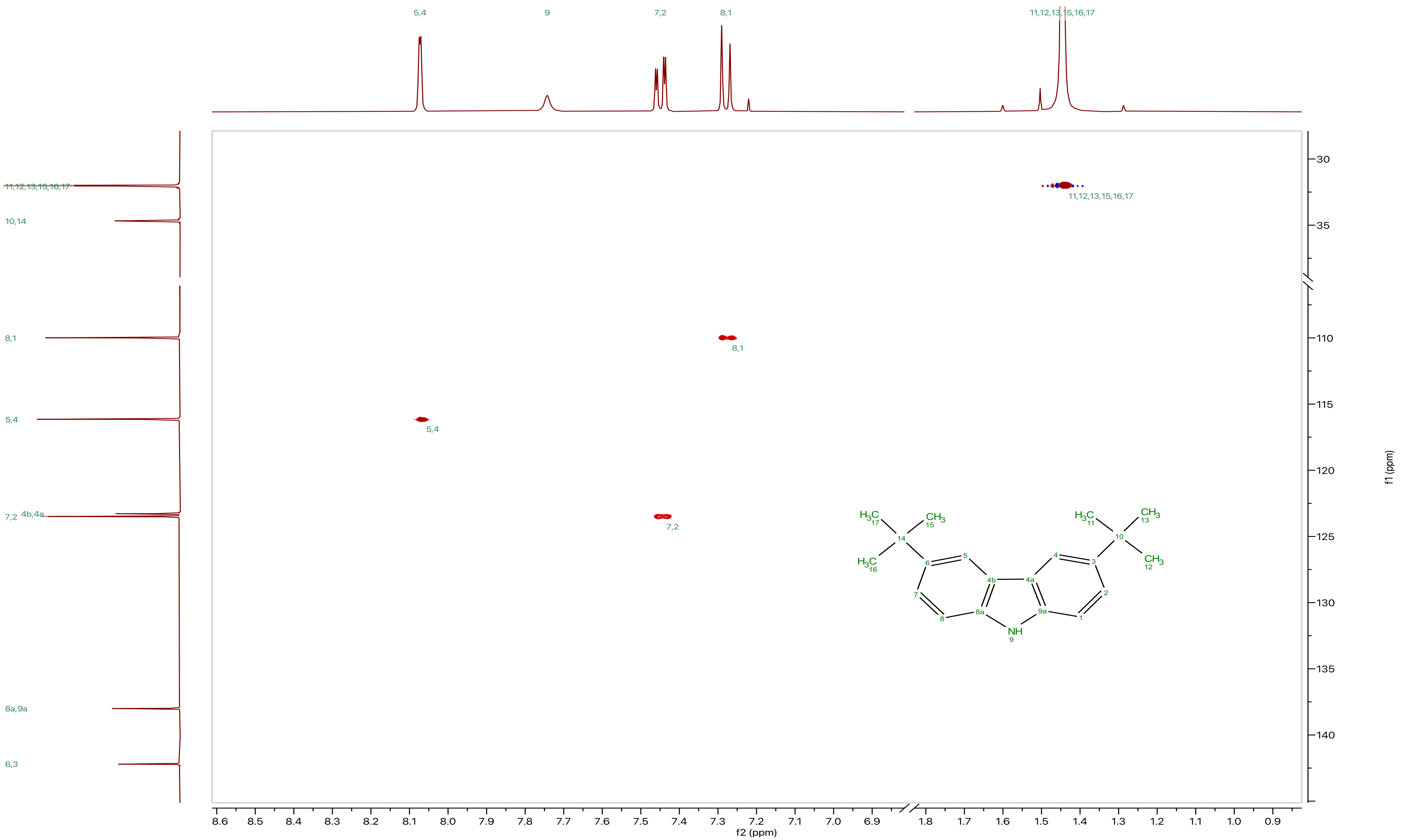

^{1}H-^{13}C{^{1}H} HSQC NMR (400/101 MHz, $CDCl_3$) of 3d’

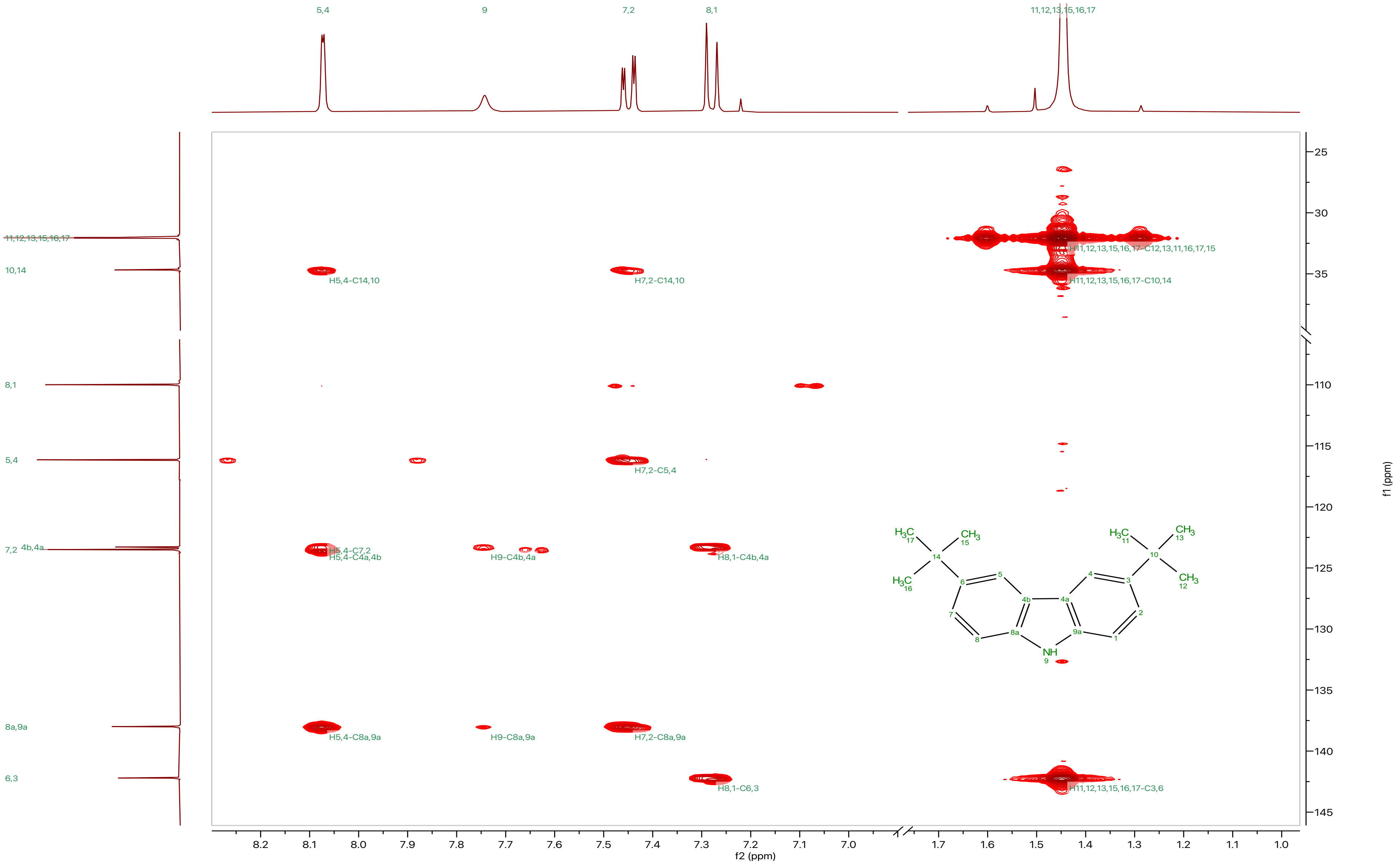

^{1}H-^{13}C{^{1}H} HMBC NMR (400/101 MHz, CDCl$_3$) of 3d’

4bd

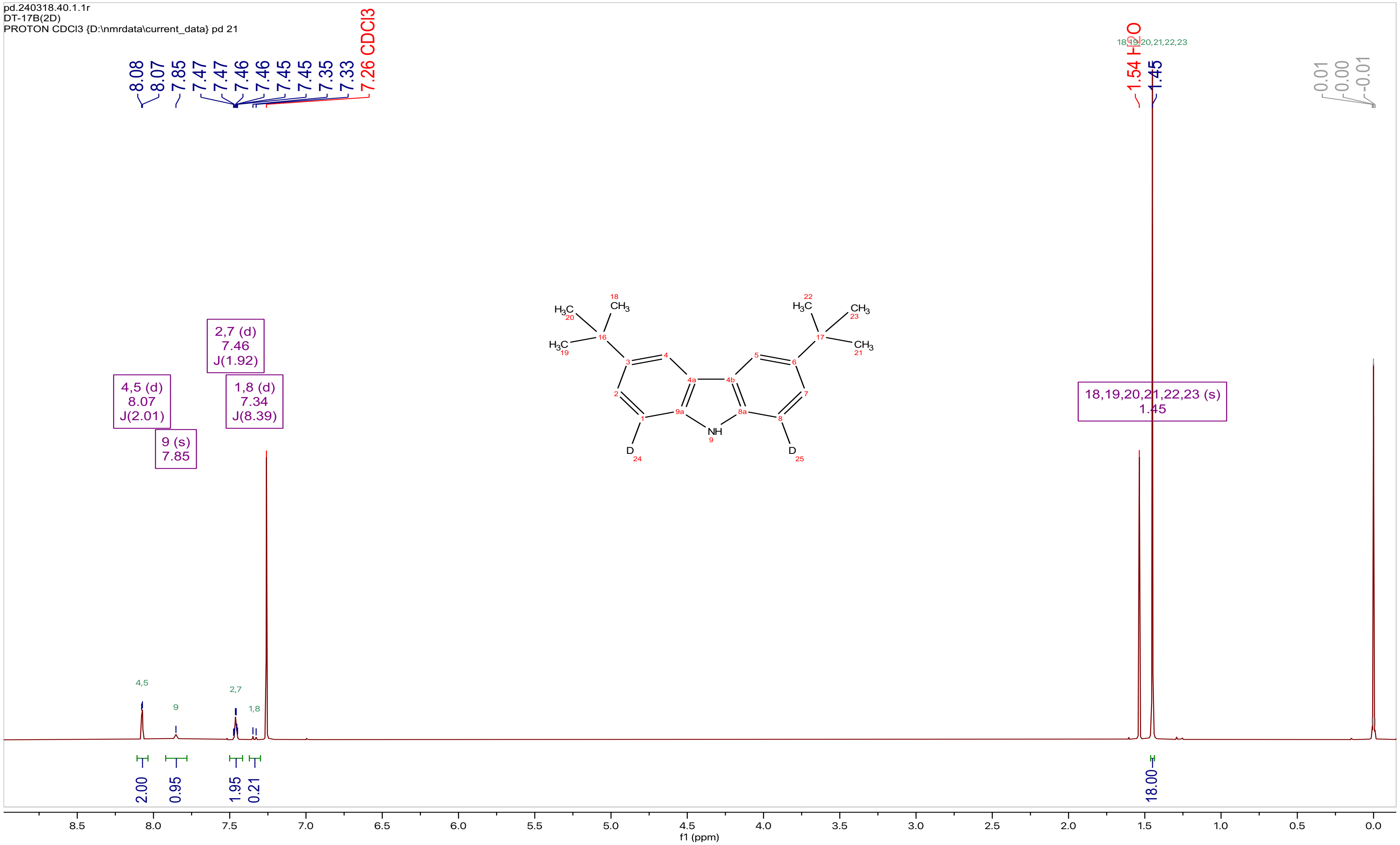

^{1}H NMR (400 MHz, $CDCl_3$) of 4bd

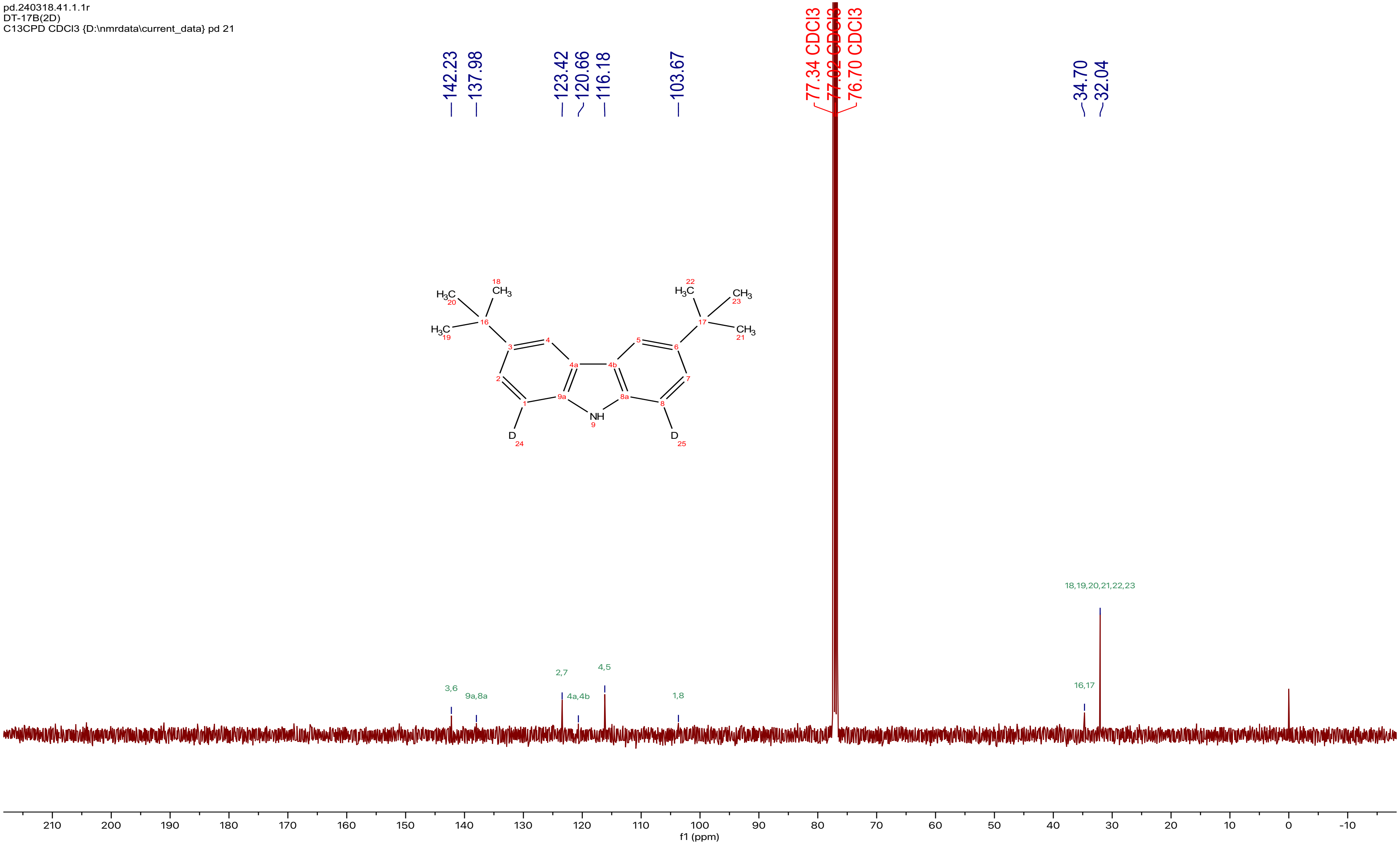

$^{13}C\{^{1}H\}$ NMR (101 MHz, $CDCl_3$) of 4bd

3e

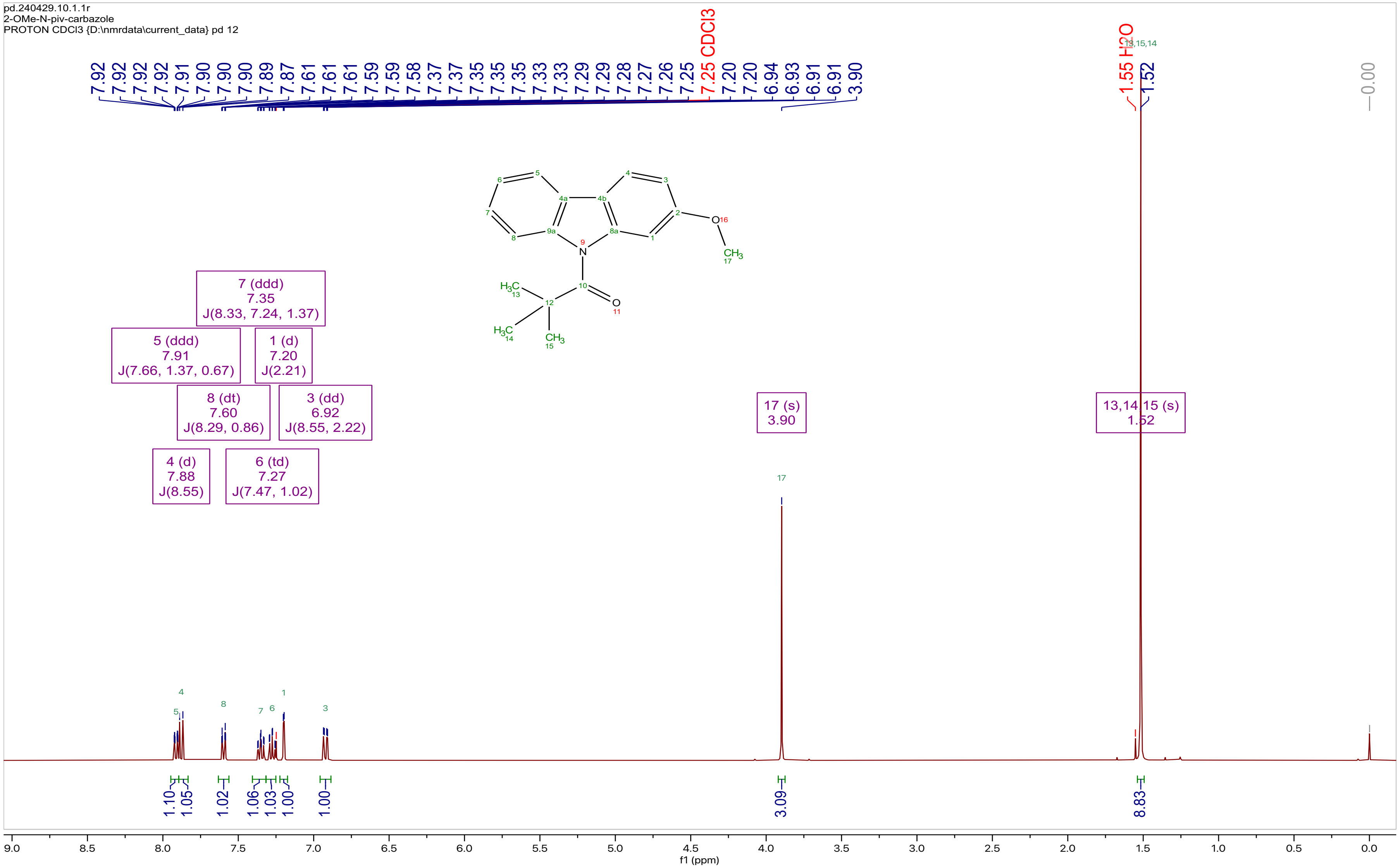

^{1}H NMR (400 MHz, $CDCl_3$) of 3e

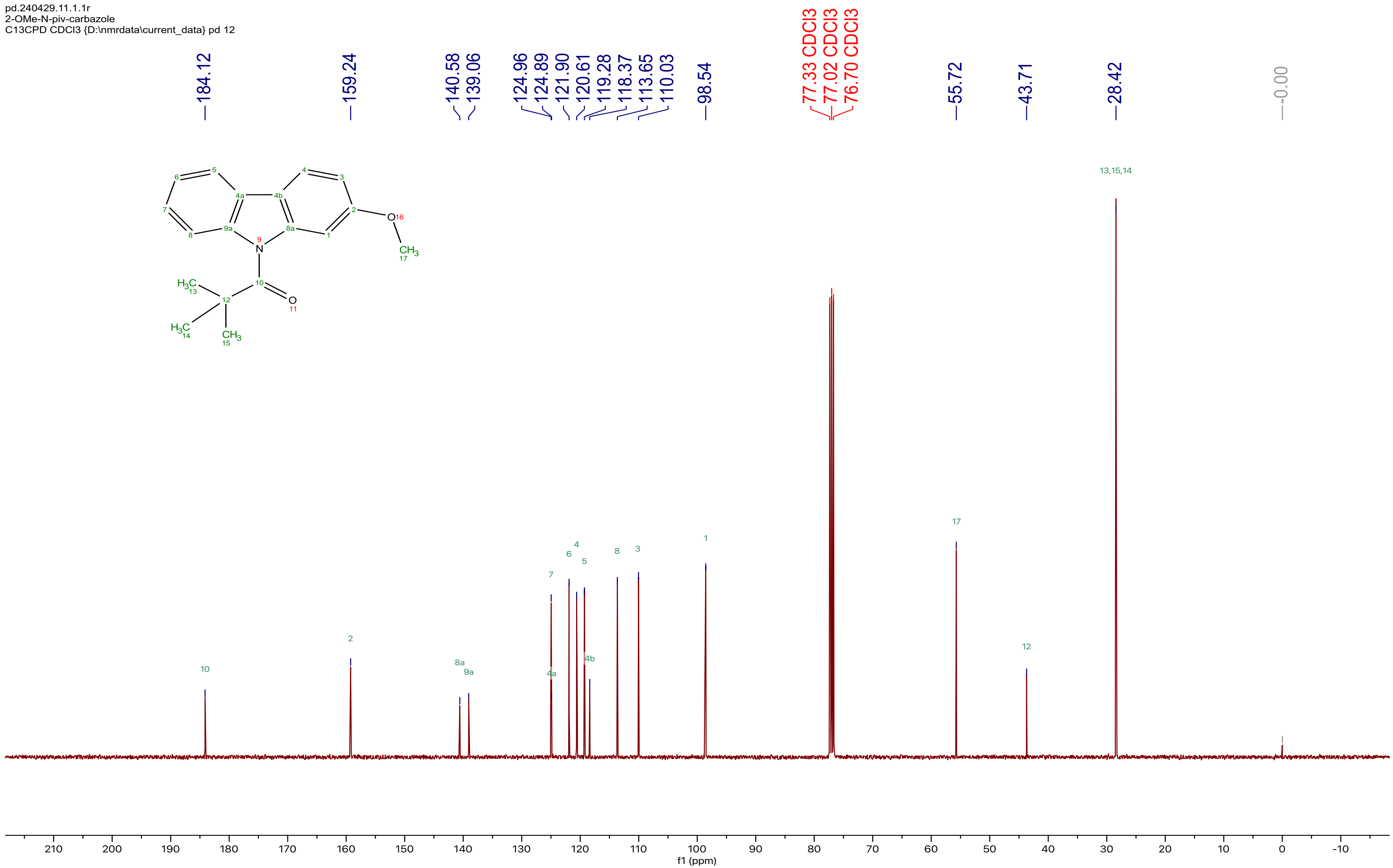

$^{13}C\{^{1}H\}$ NMR (101 MHz, $CDCl_3$) of 3e

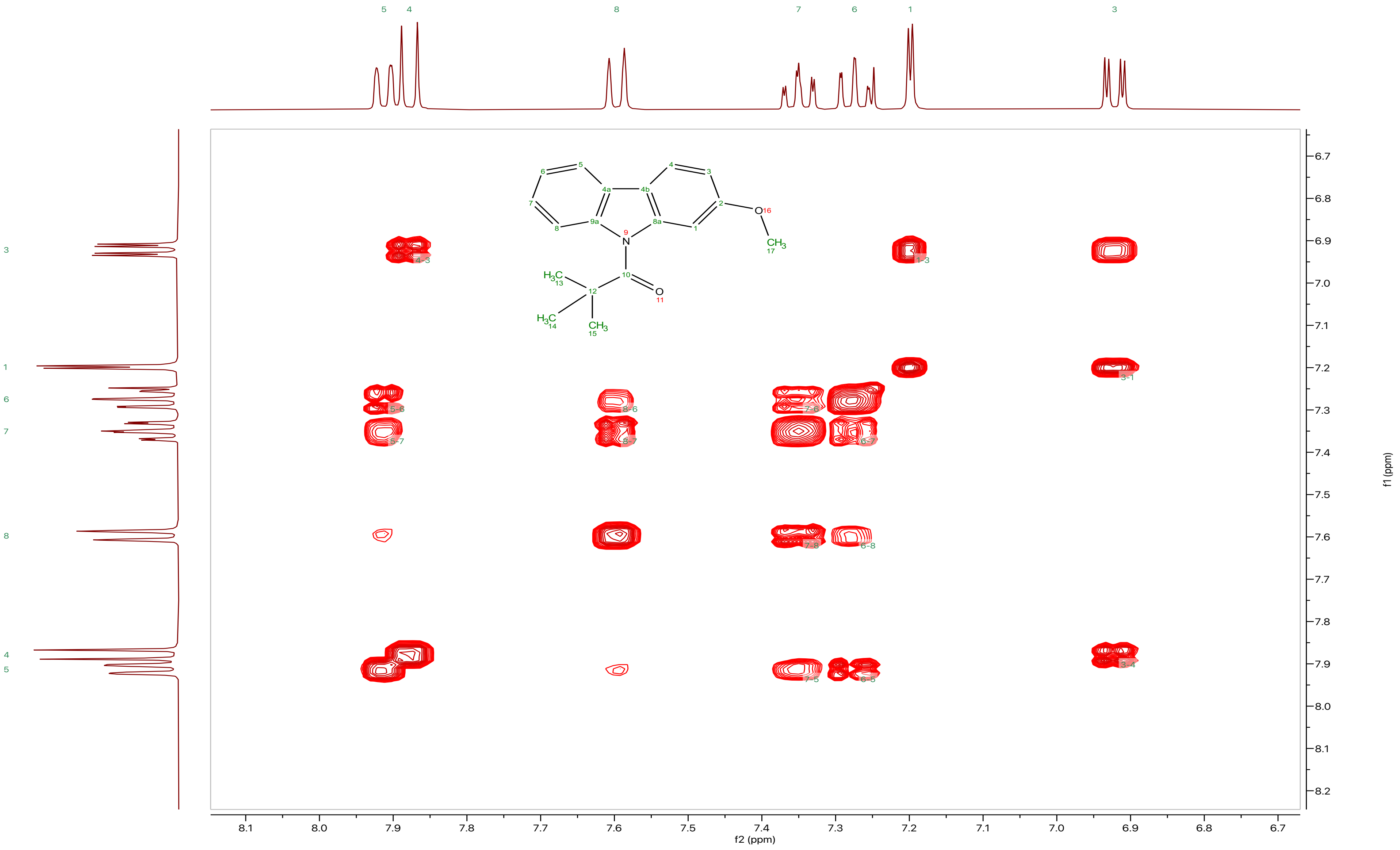

^{1}H-^{1}H COSY (400 MHz, $CDCl_3$) of 3e

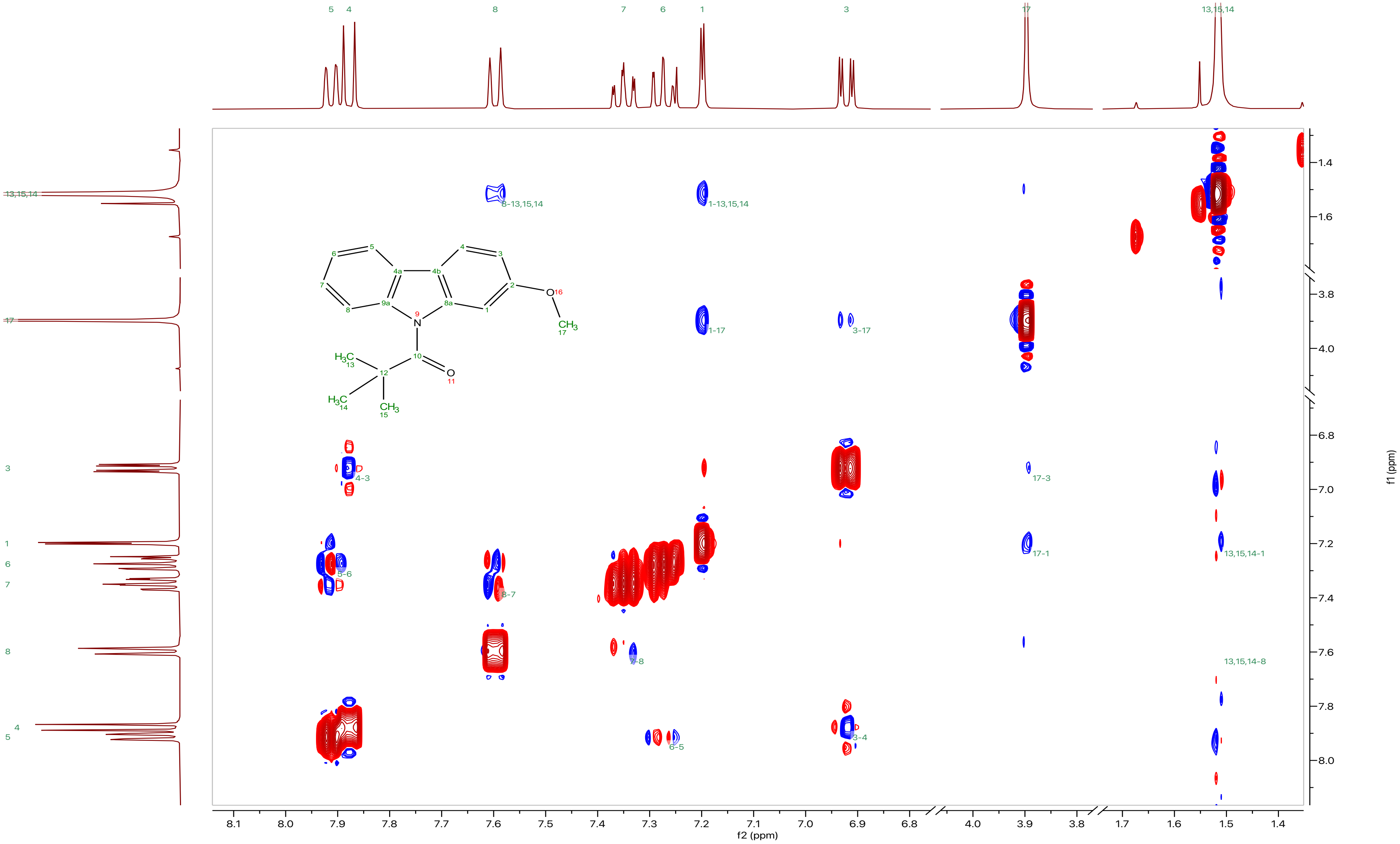

^1H-^1H NOESY (400 MHz, $CDCl_3$) of 3e

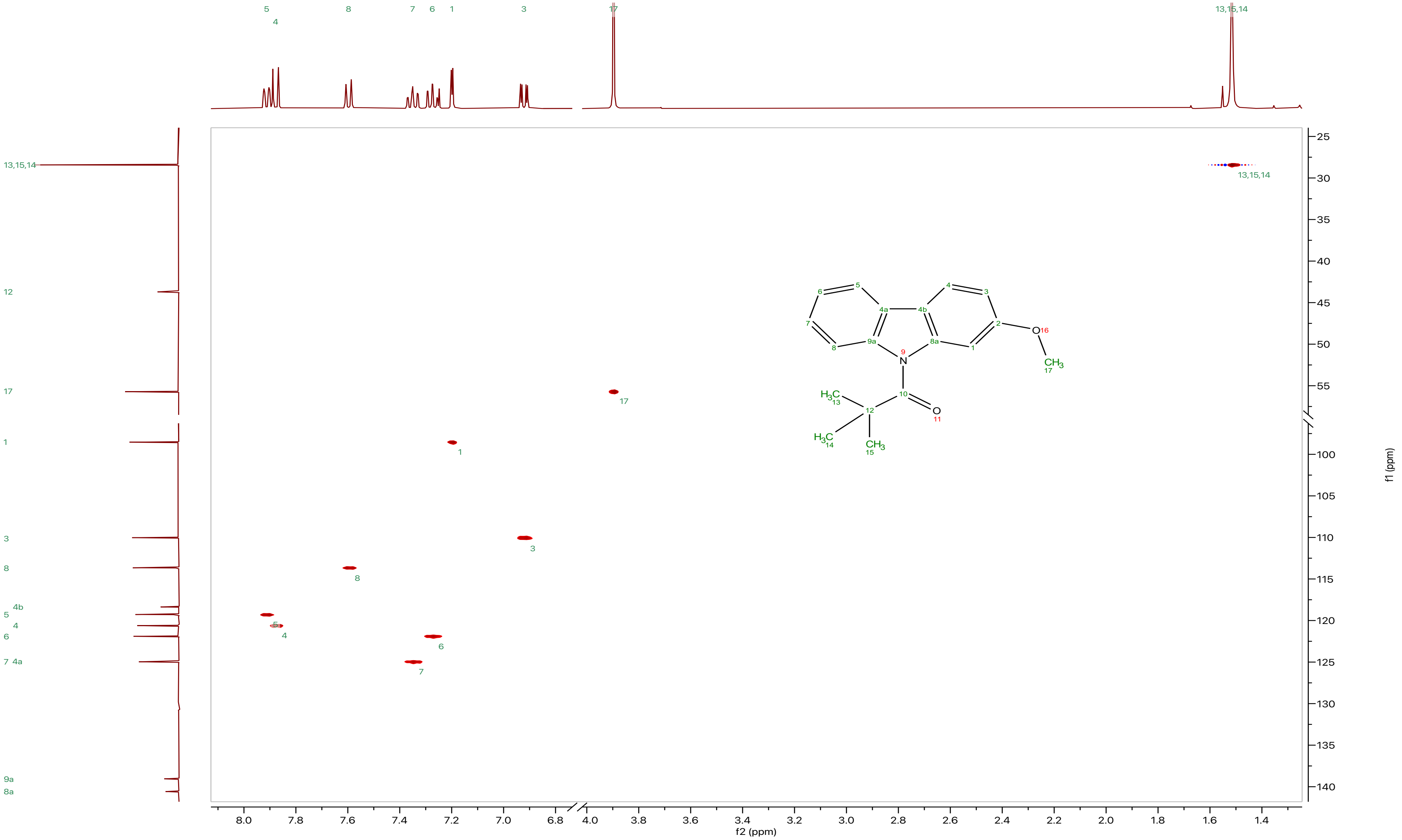

^{1}H-^{13}C{^{1}H} HSQC NMR (400/101 MHz, $CDCl_3$) of 3e

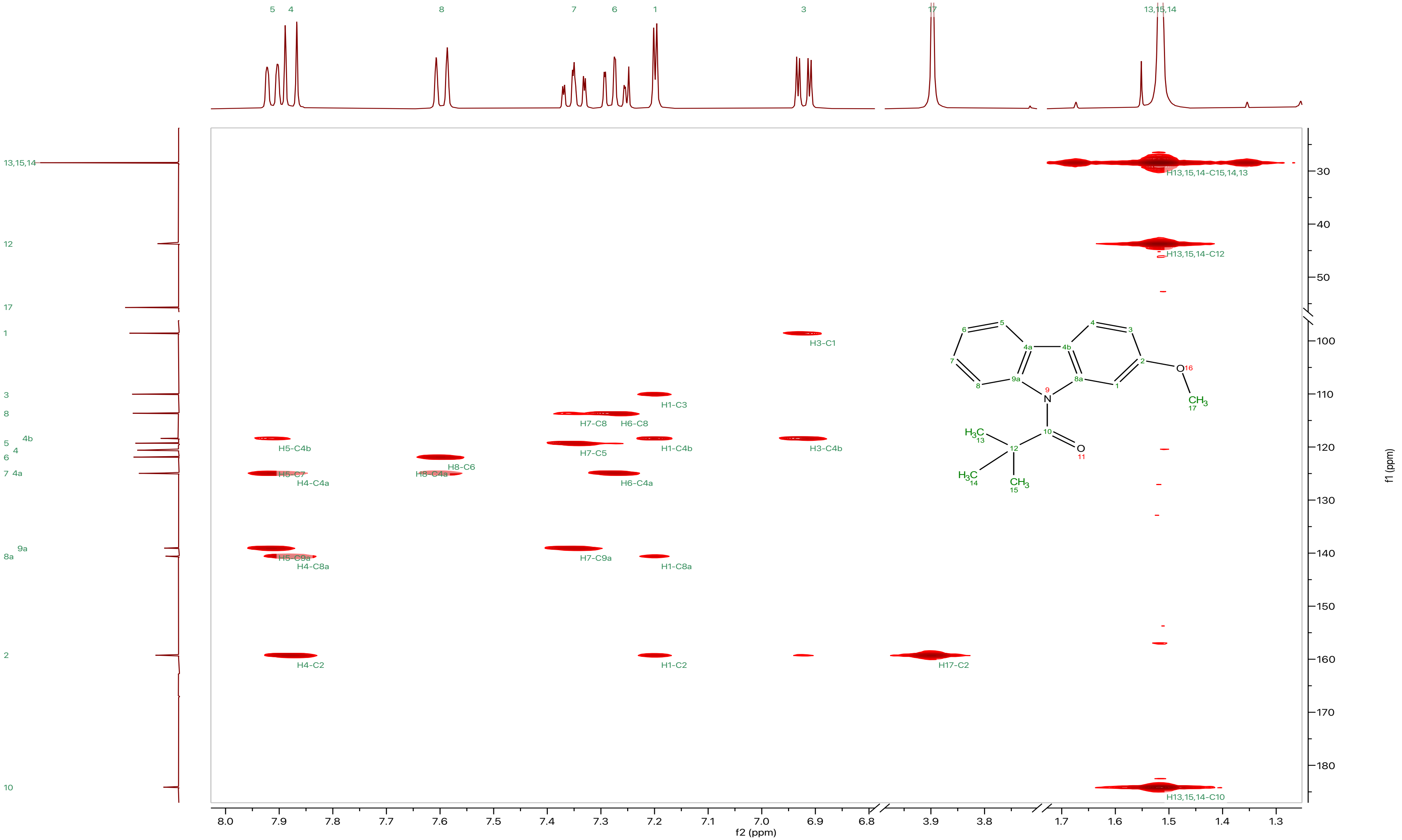

^{1}H-$^{13}C\{^{1}H\}$ HMBC NMR (400/101 MHz, $CDCl_3$) of 3e

4ae

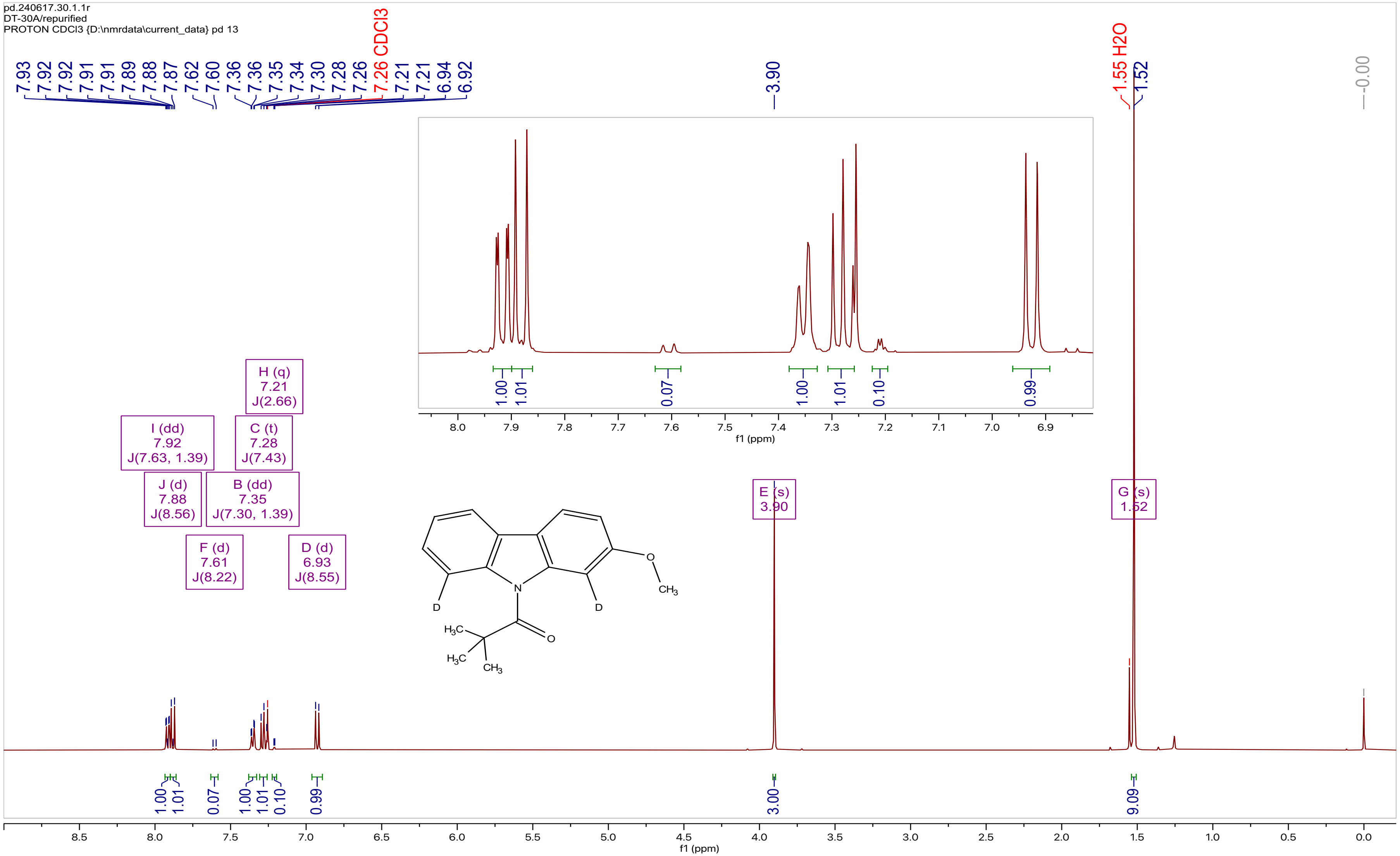

^{1}H NMR (400 MHz, $CDCl_3$) of 4ae

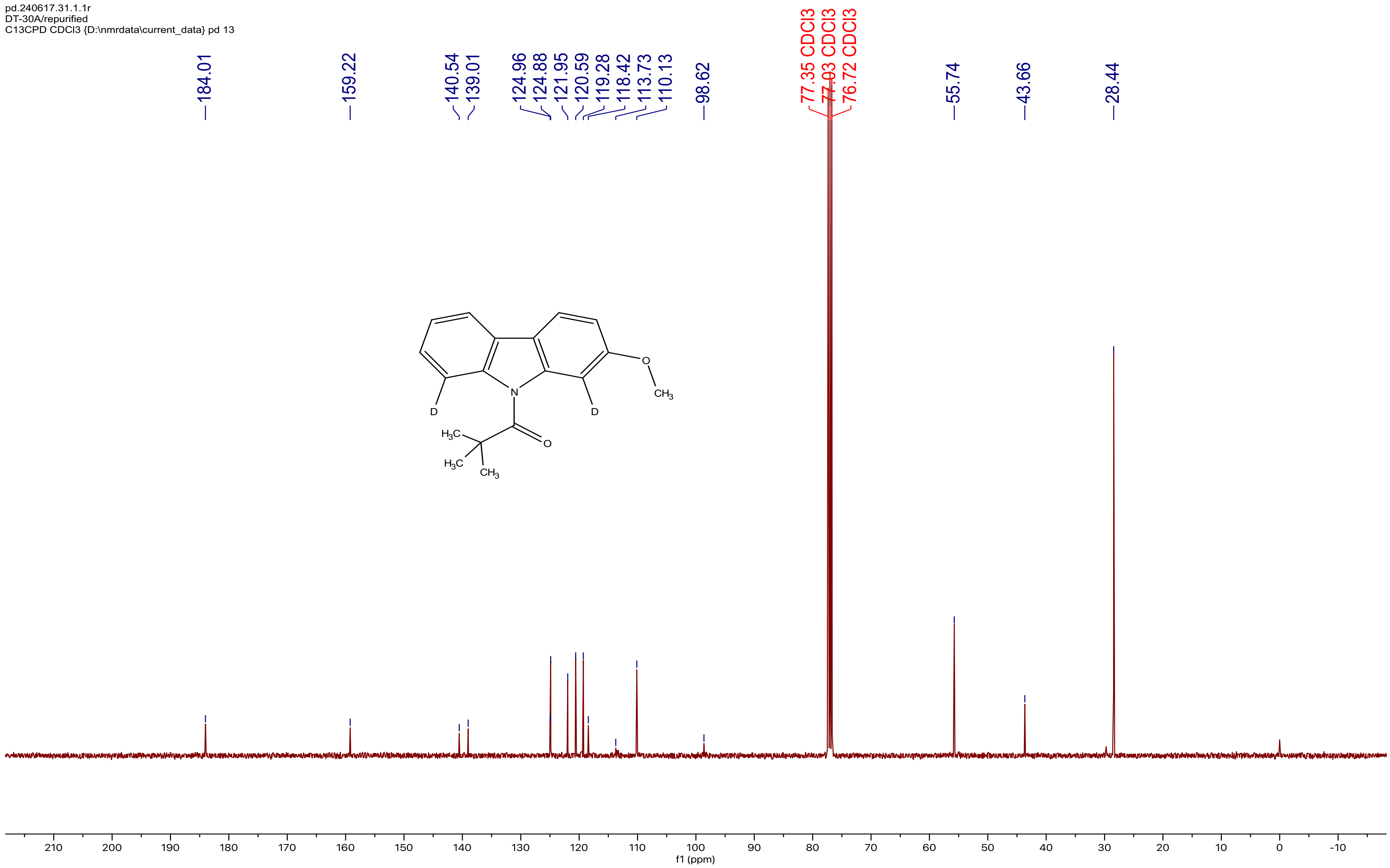

$^{13}C\{^1H\}$ NMR (101 MHz, $CDCl_3$) of 4ae

3e’

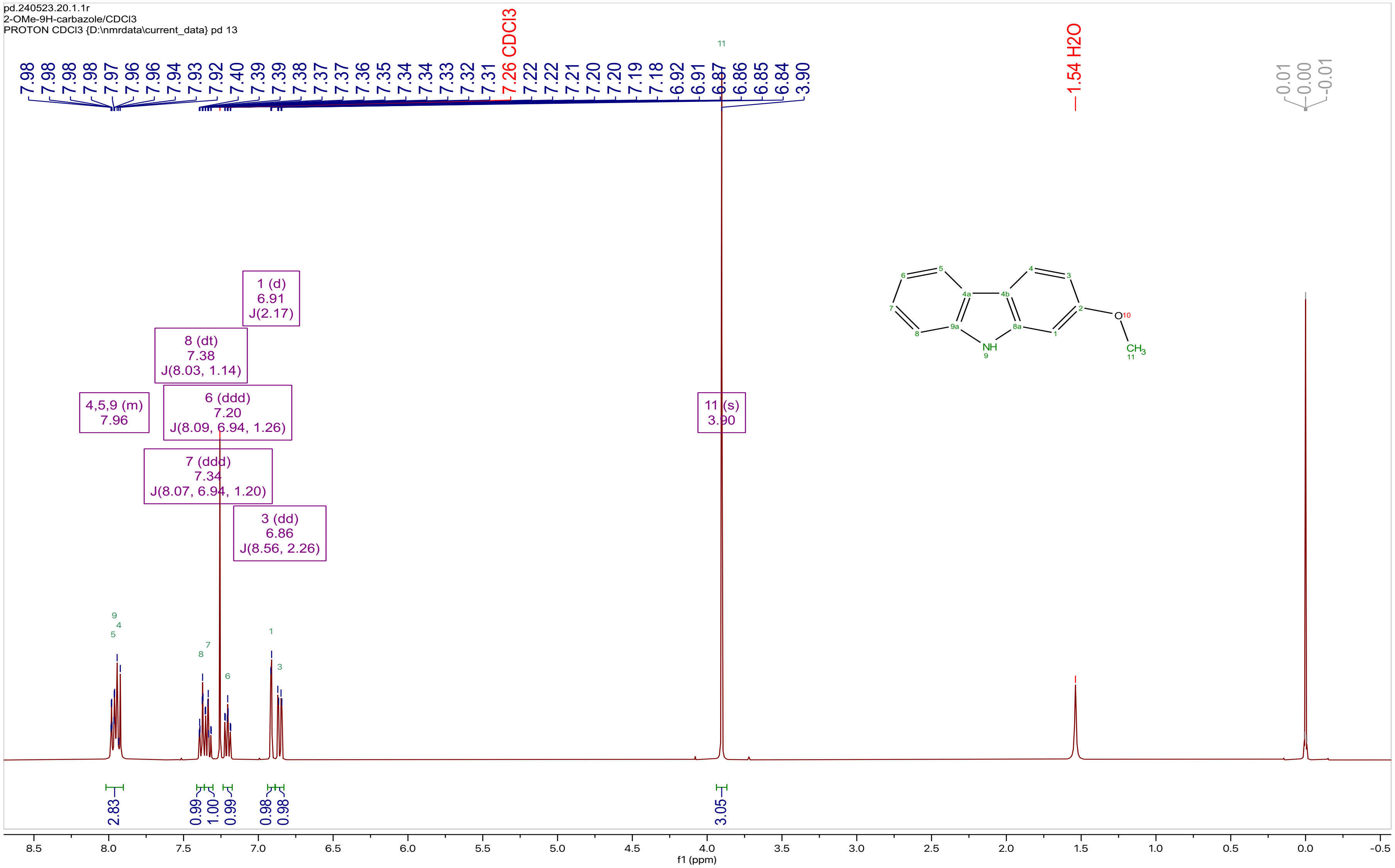

¹H NMR (400 MHz, $CDCl_3$) of 3e’

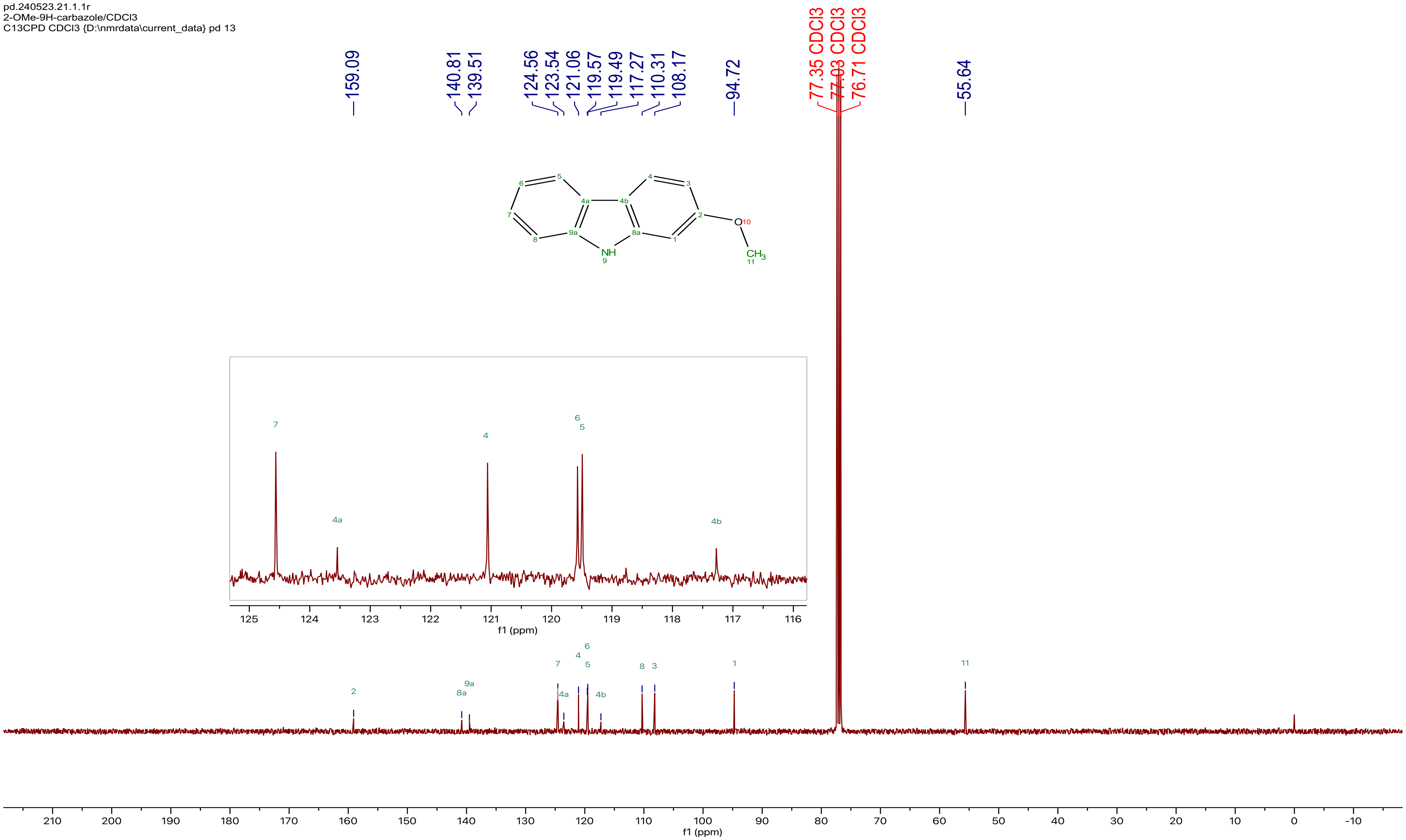

$^{13}C\{^1H\}$ NMR (101 MHz, $CDCl_3$) of 3e’

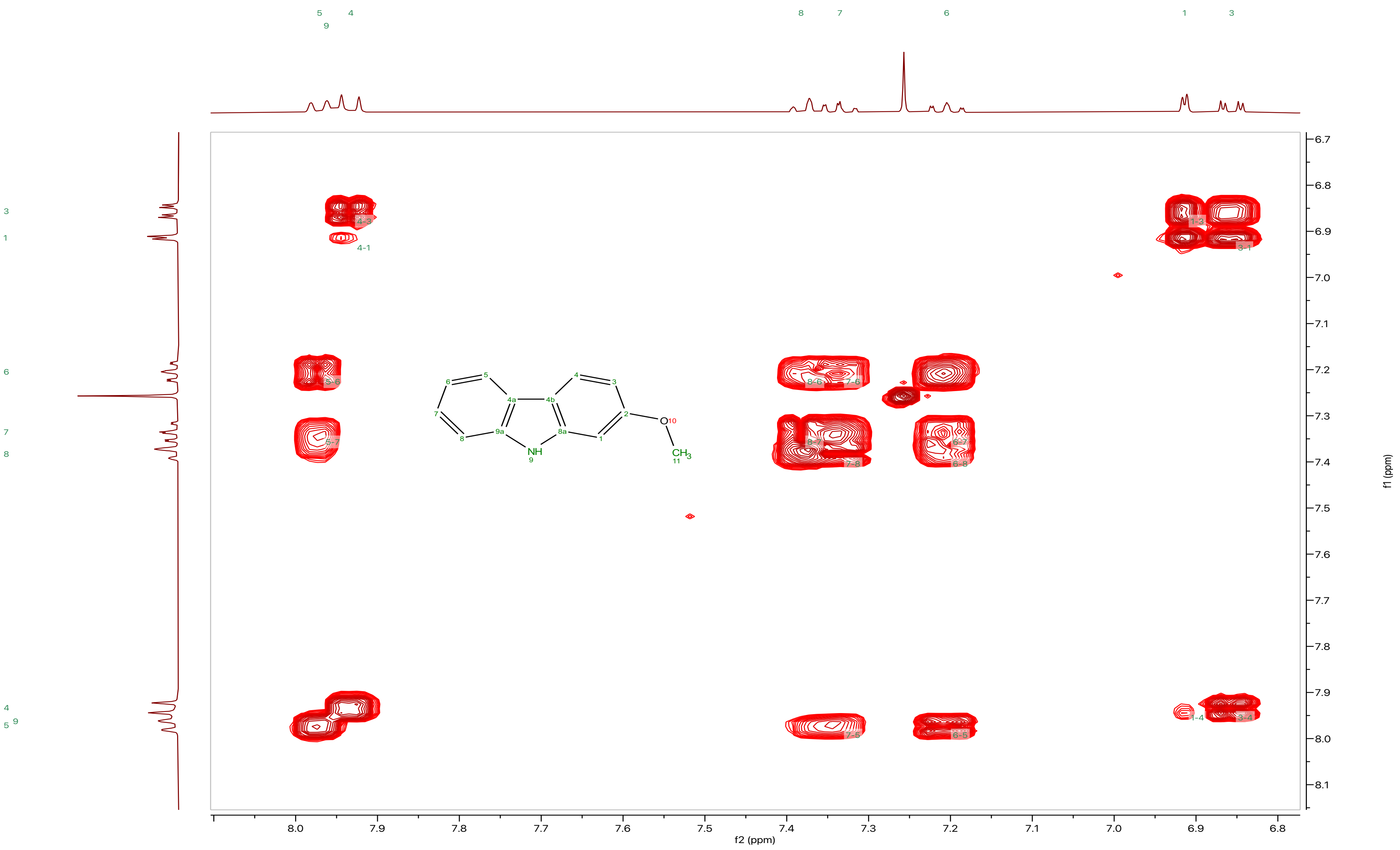

^{1}H-^{1}H COSY (400 MHz, $CDCl_3$) of 3e’

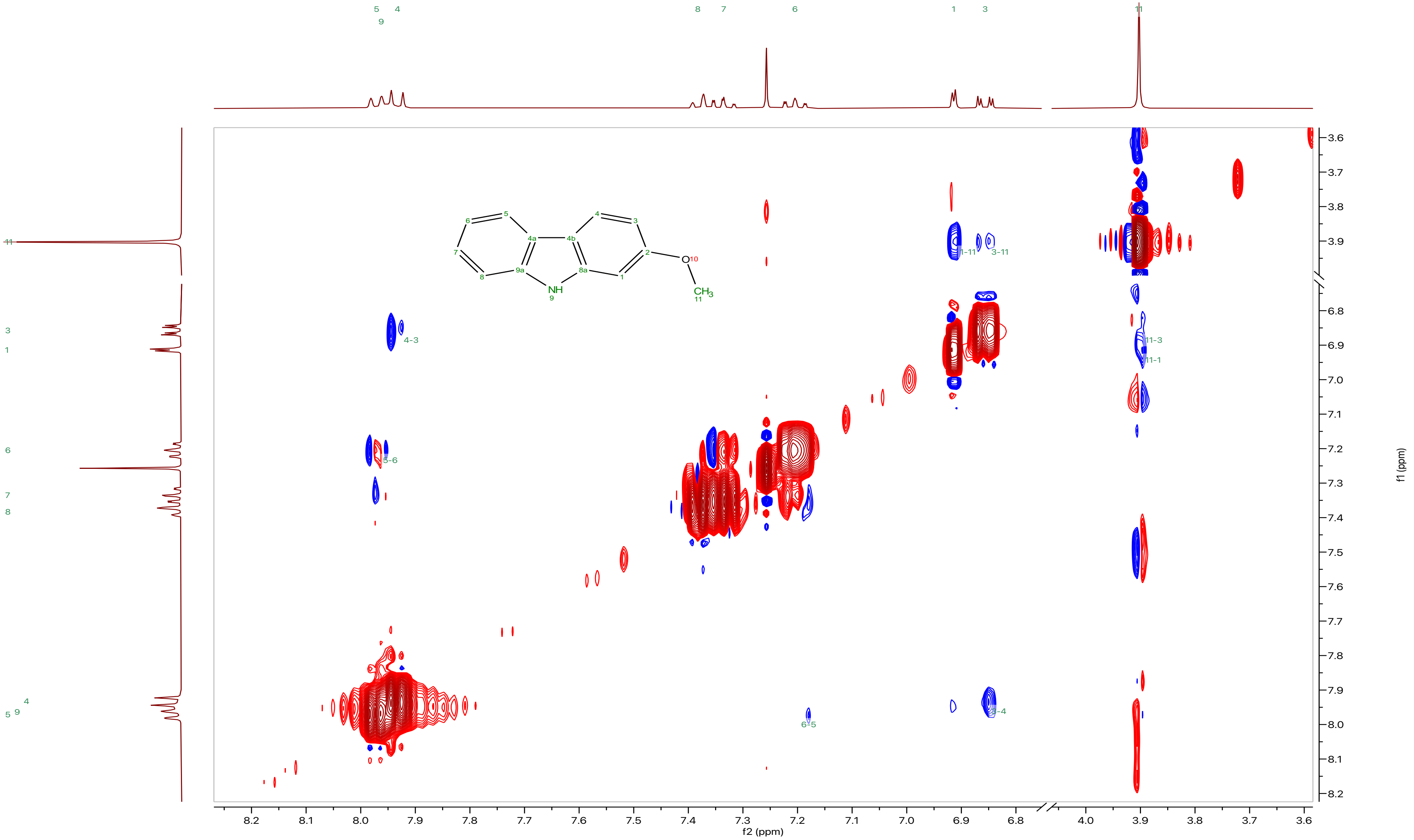

^1H-^1H NOESY (400 MHz, $CDCl_3$) of 3e'

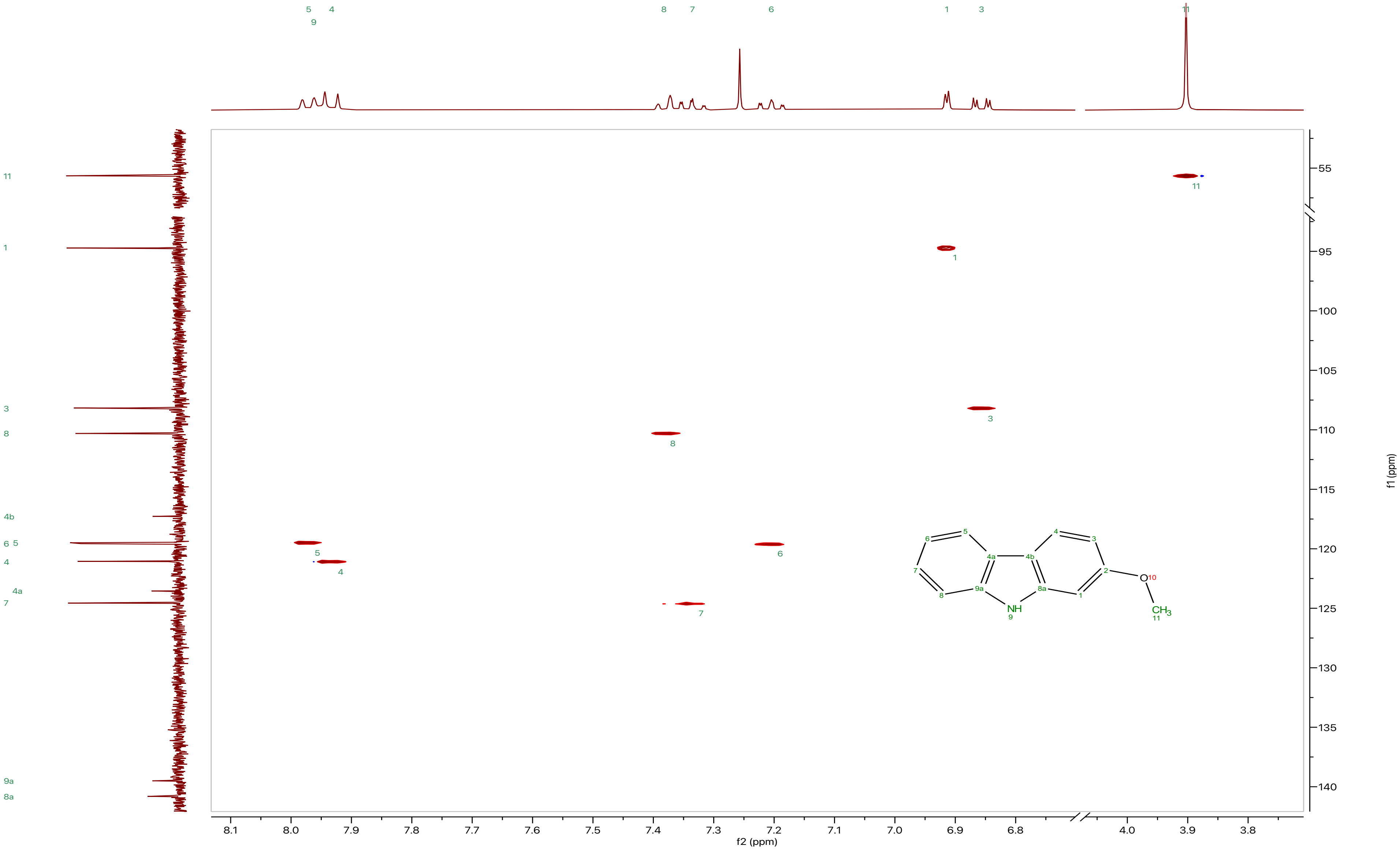

^{1}H-^{13}C{^{1}H} HSQC NMR (400/101 MHz, $CDCl_3$) of 3e'

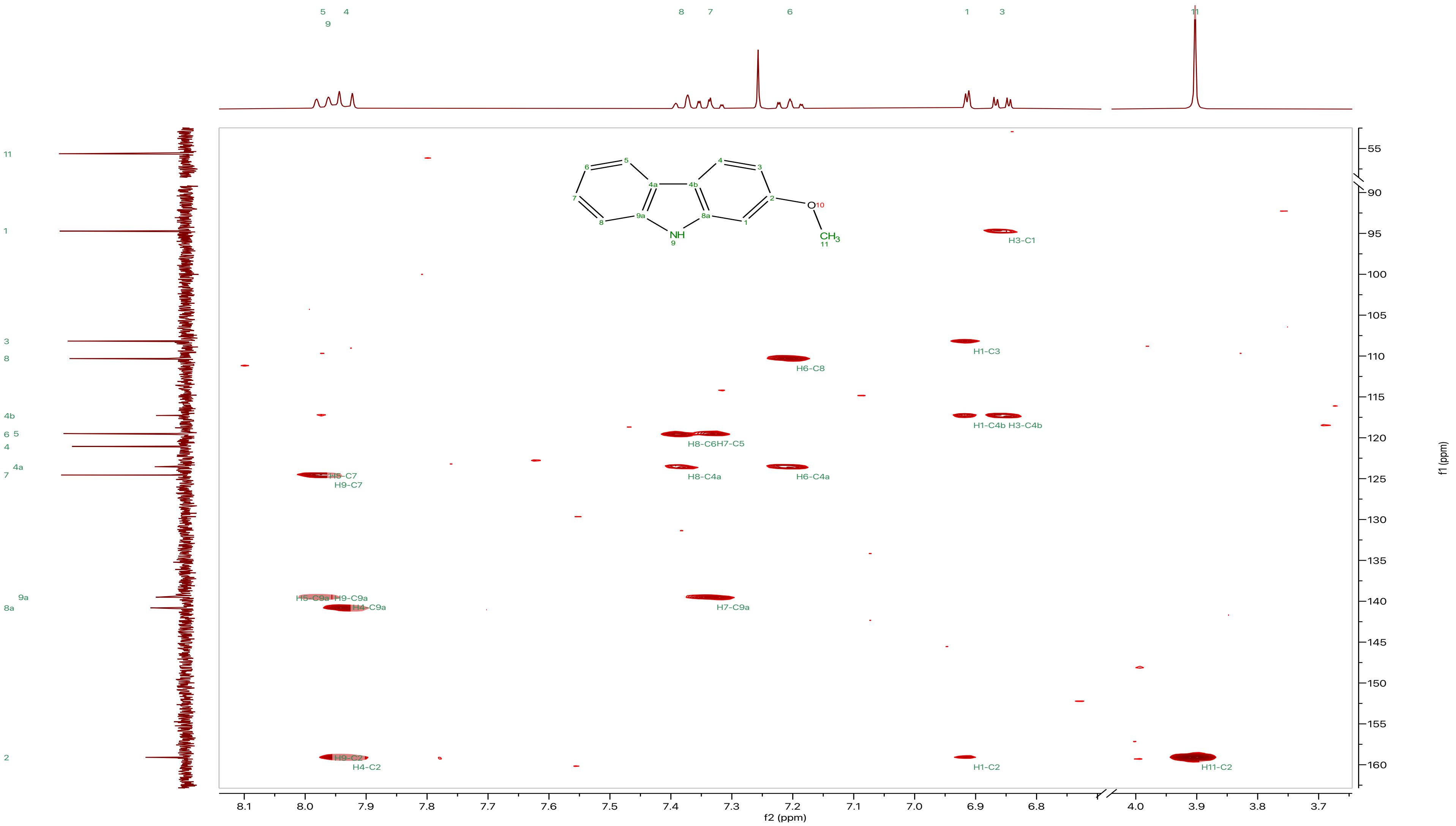

^{1}H-^{13}C{^{1}H} HMBC NMR (400/101 MHz, $CDCl_3$) of 3e'

4be

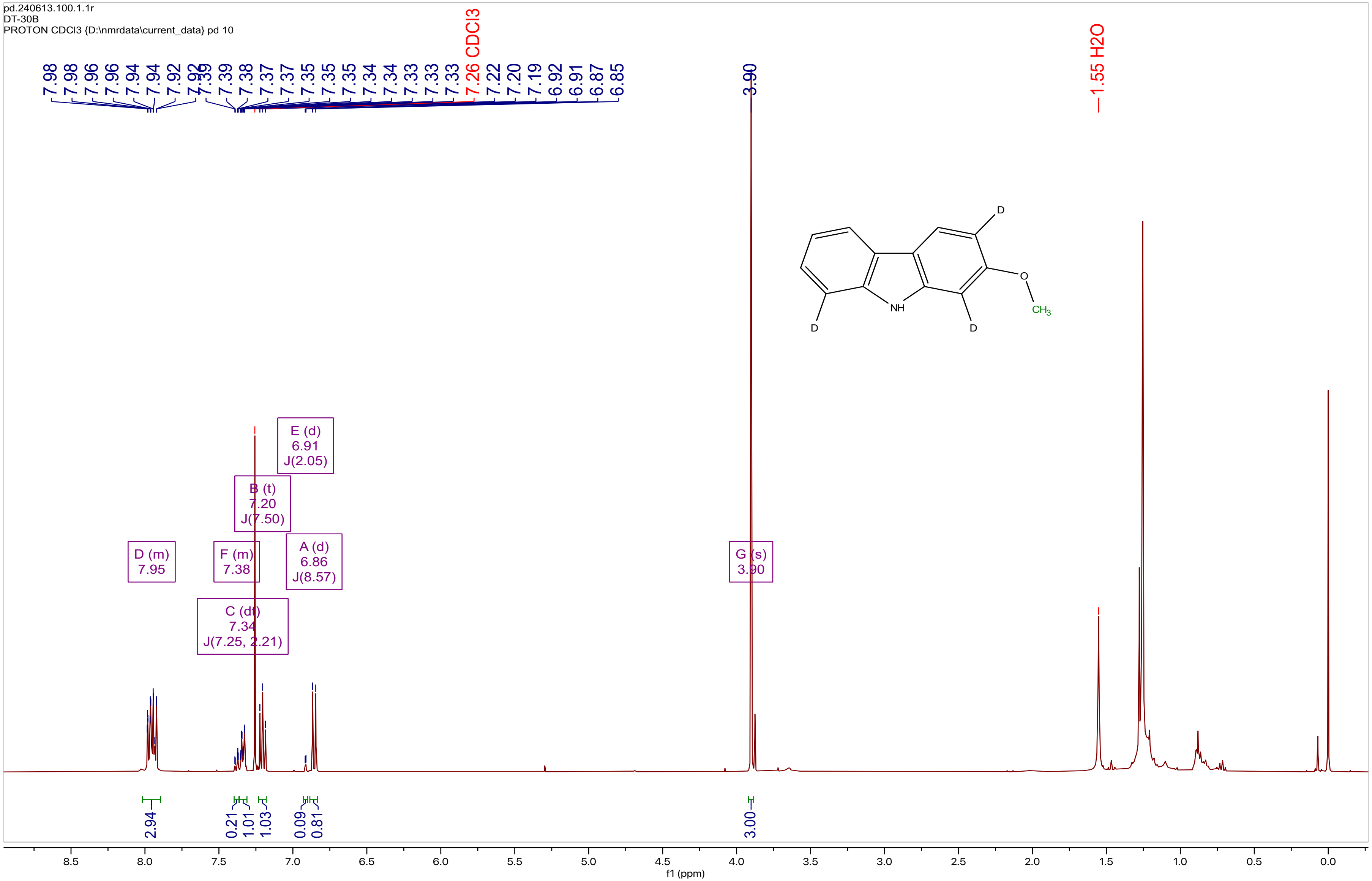

^{1}H NMR (400 MHz, $CDCl_3$) of 4be

pd.240613.101.1.1r
DT-30B
C13CPD CDCl3 {D:\nmrdata\current_data} pd 10

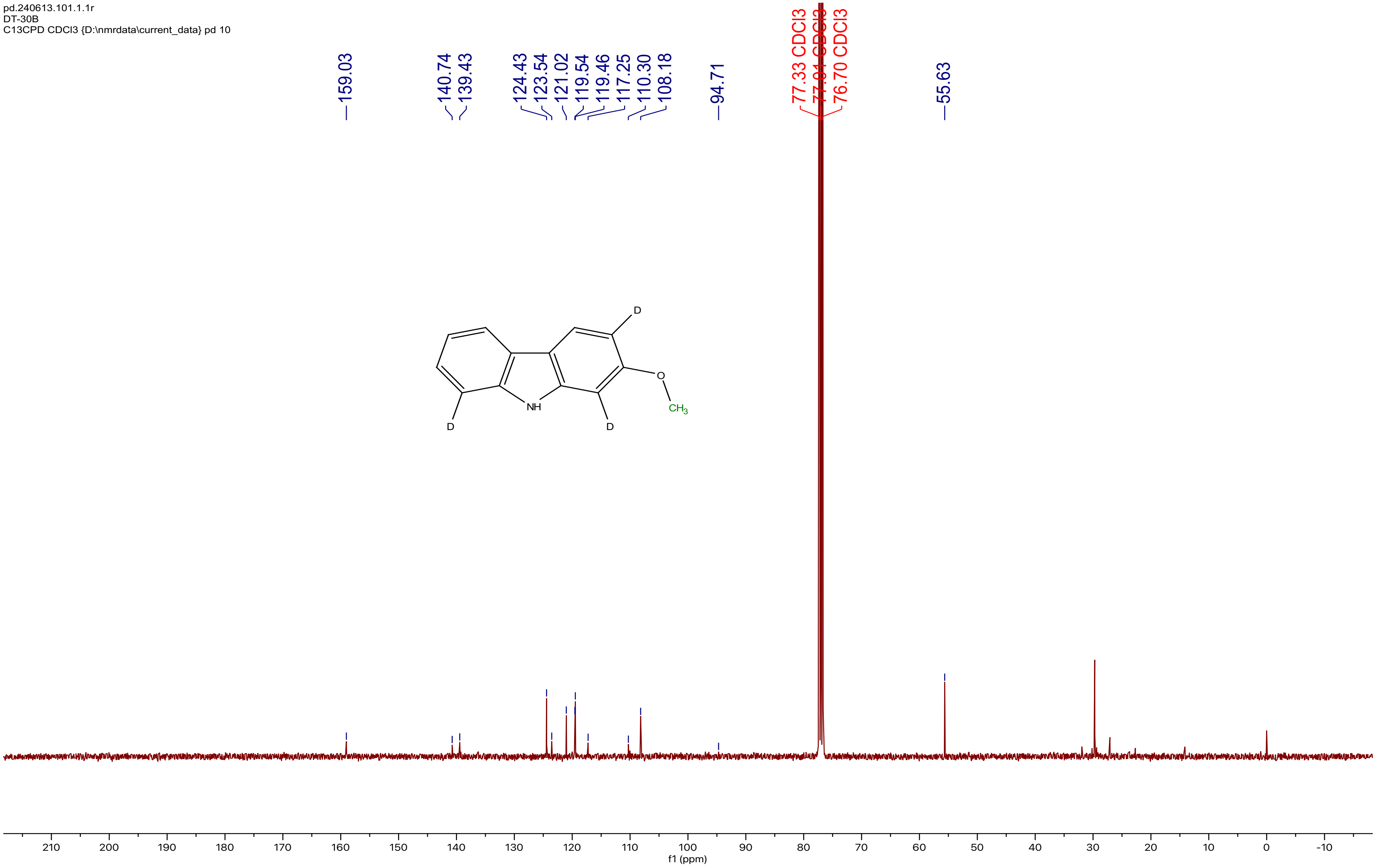

$^{13}C\{^{1}H\}$ NMR (101 MHz, $CDCl_3$) of 4be

3e’

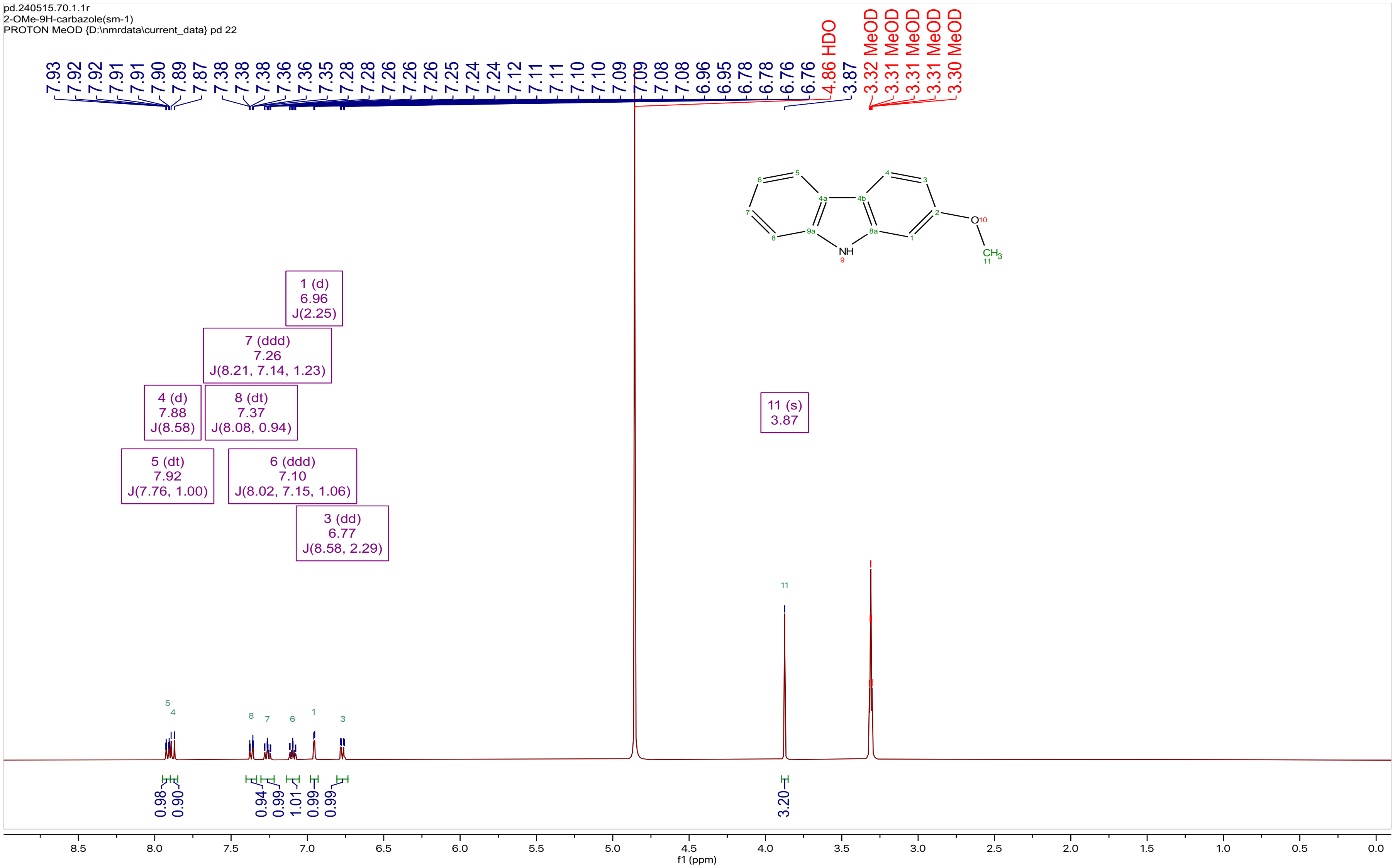

^{1}H NMR (400 MHz, MeOD) of 3e’

pd.240515.71.1.1r
2-OMe-9H-carbazole(sm-1)
C13CPD MeOD {D:\nmrdata\current_data} pd 22

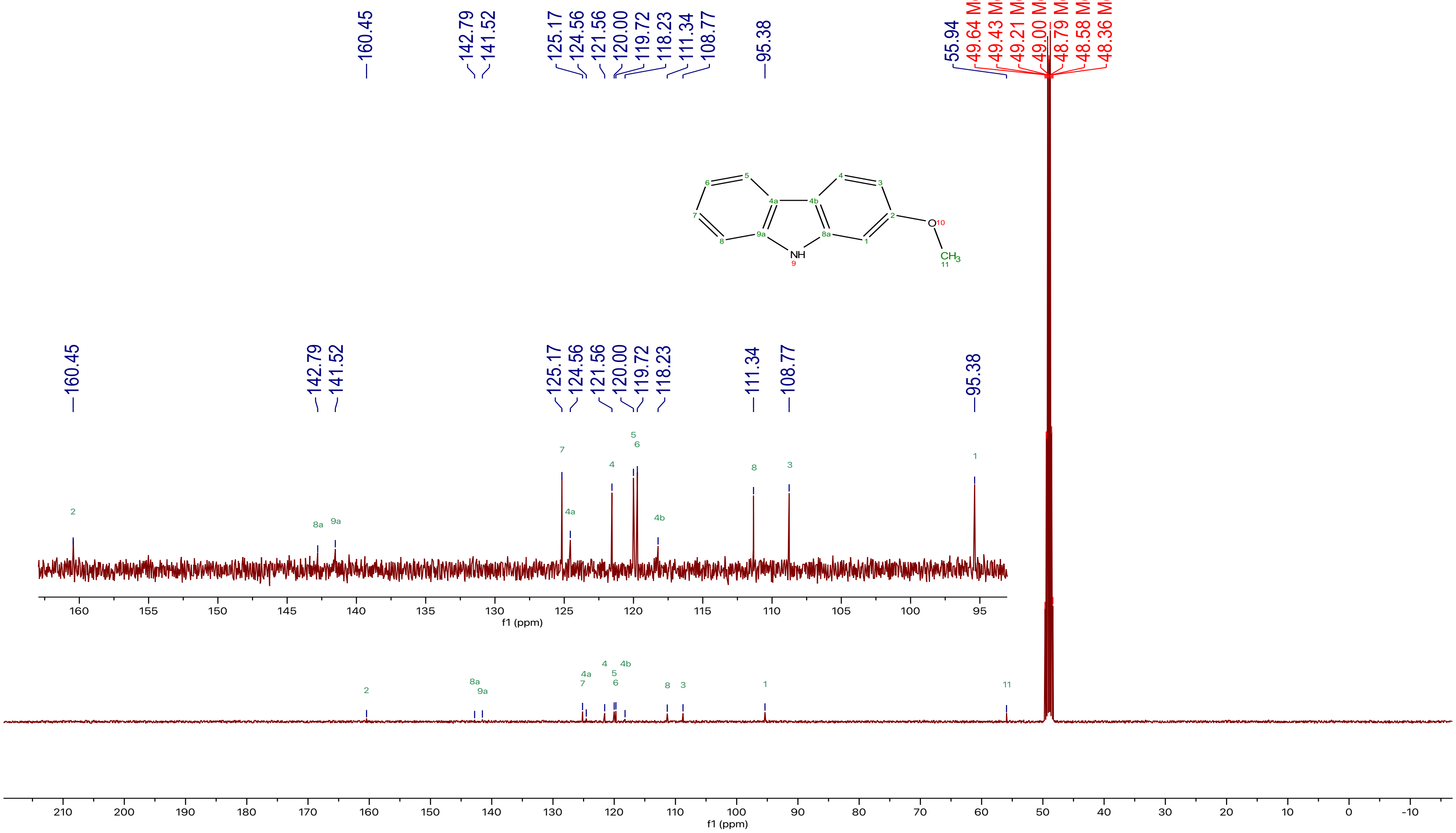

$^{13}C\{^{1}H\}$ NMR (101 MHz, MeOD) of 3e'

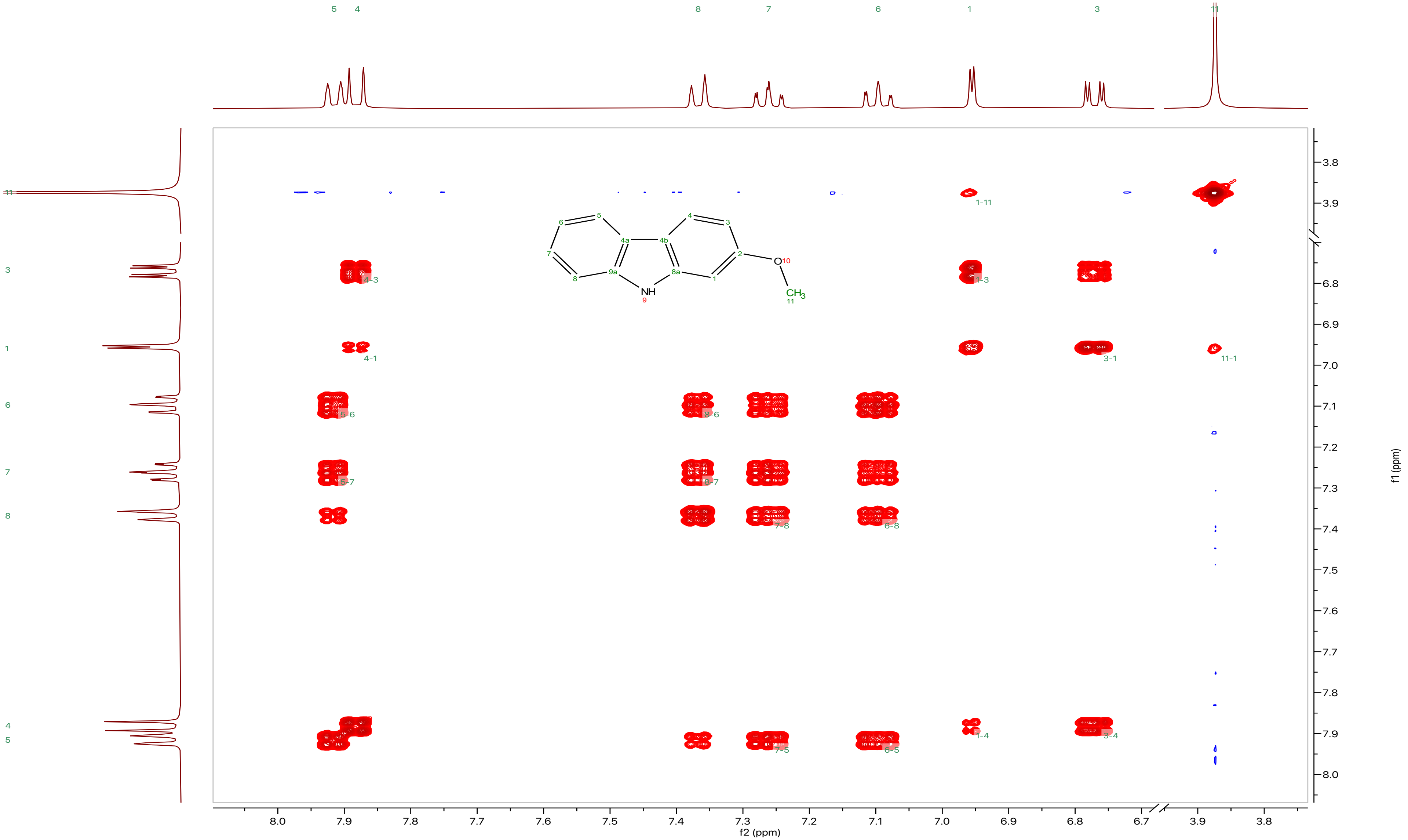

^{1}H-^{1}H COSY (400 MHz, MeOD) of 3e'

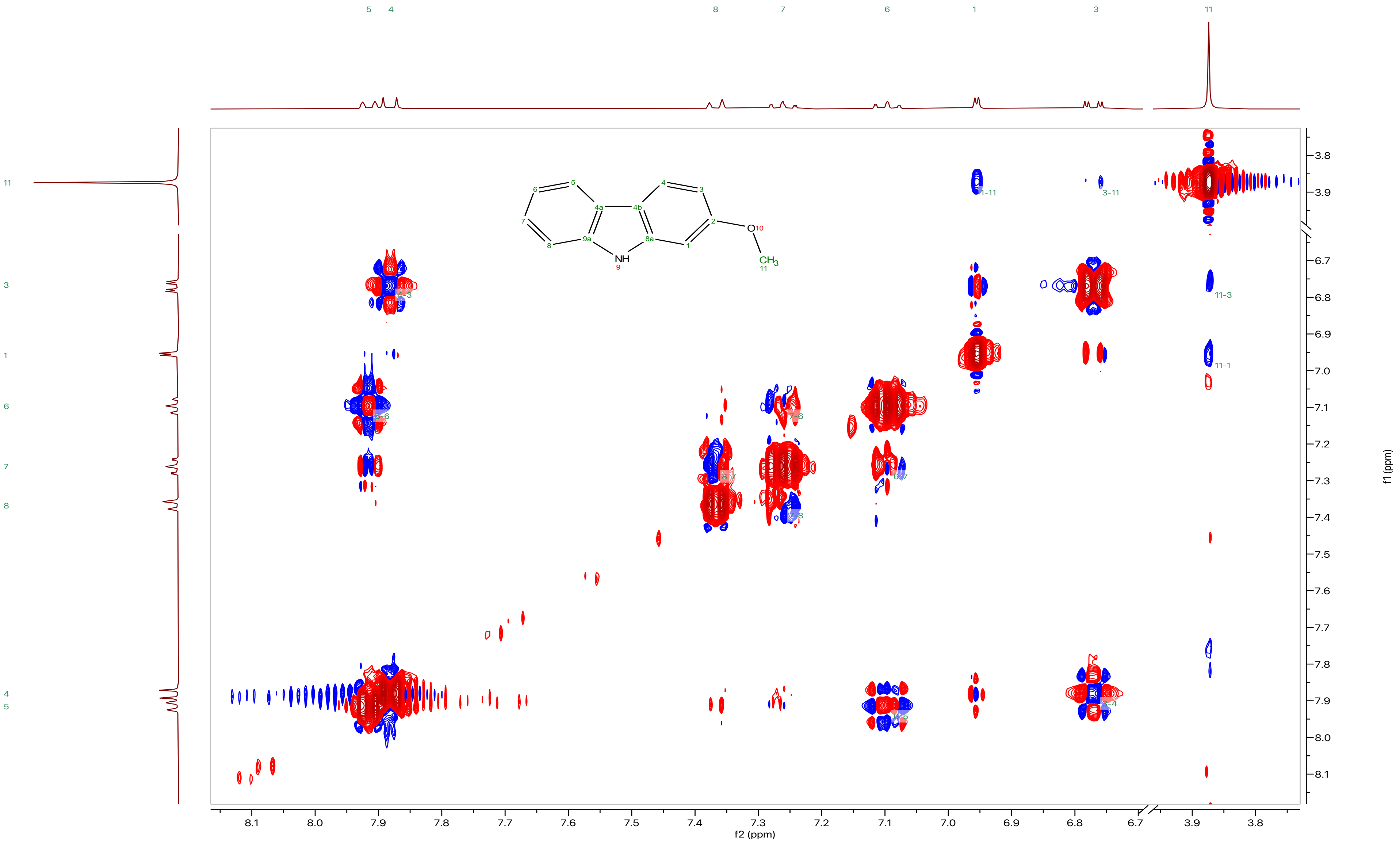

^1H-^1H NOESY (400 MHz, MeOD) of 3e'

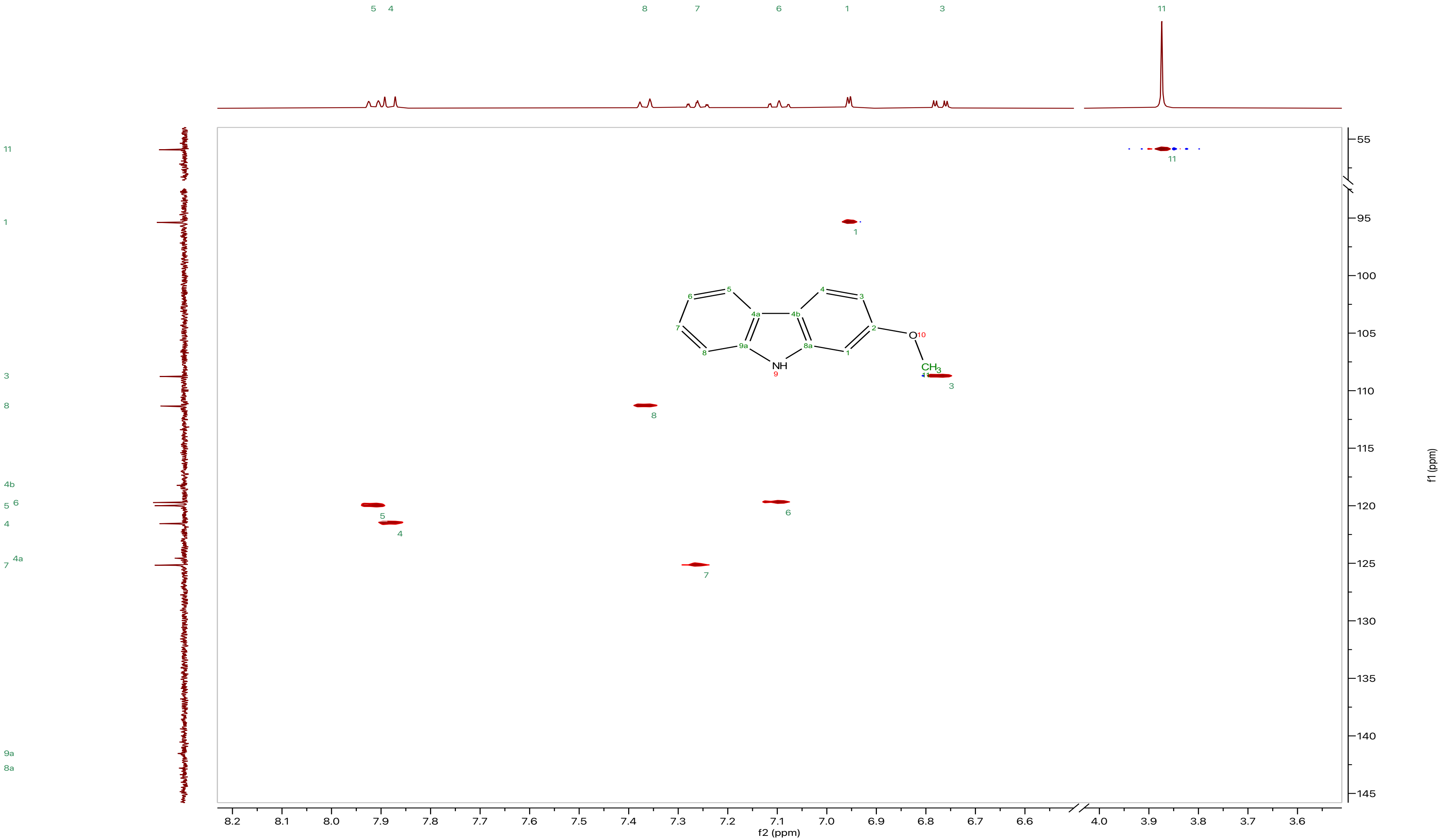

^{1}H-^{13}C{^{1}H} HSQC NMR (400/101 MHz, MeOD) of 3e’

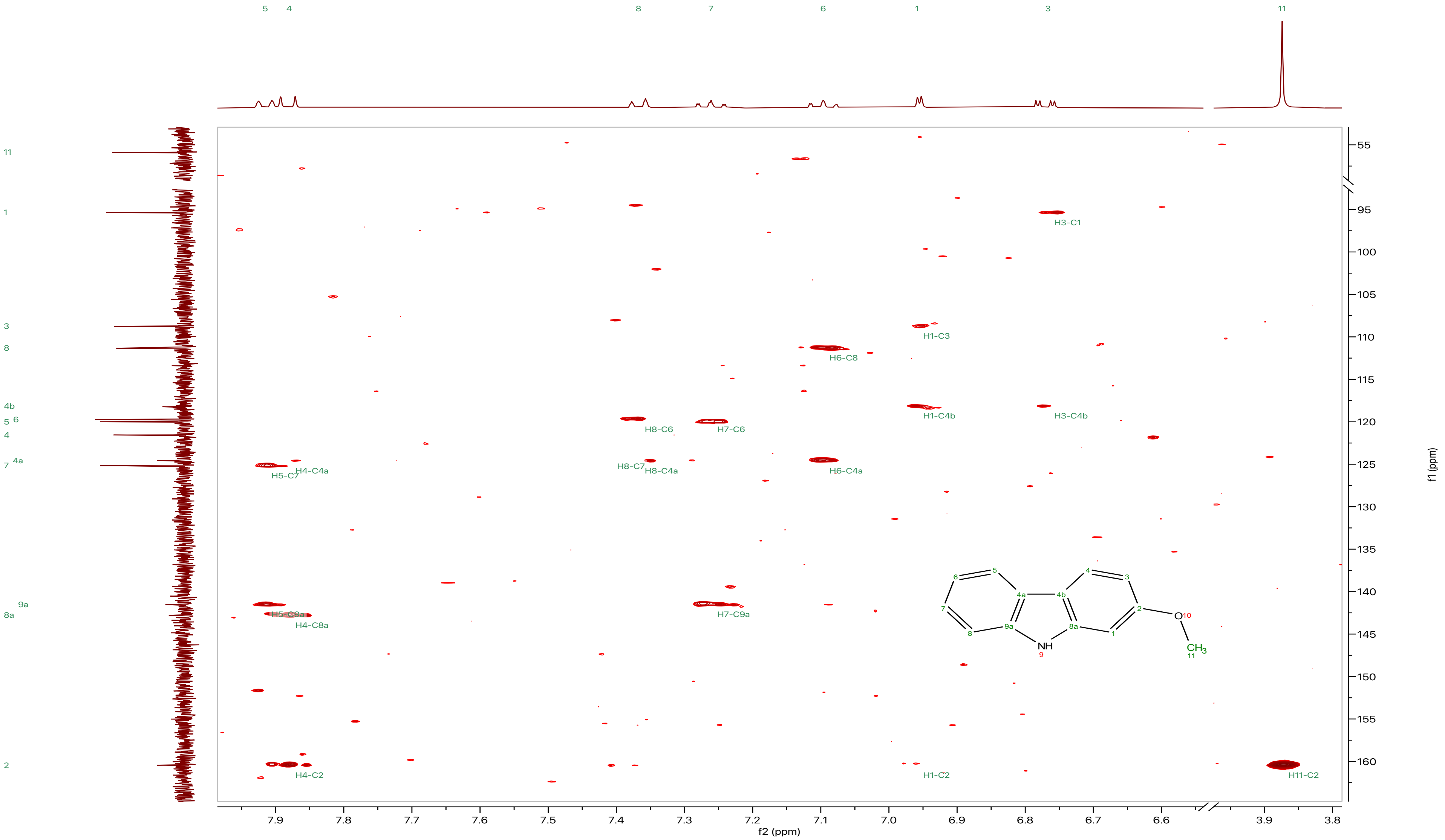

^{1}H-$^{13}C\{^{1}H\}$ HMBC NMR (400/101 MHz, MeOD) of 3e'

4be’

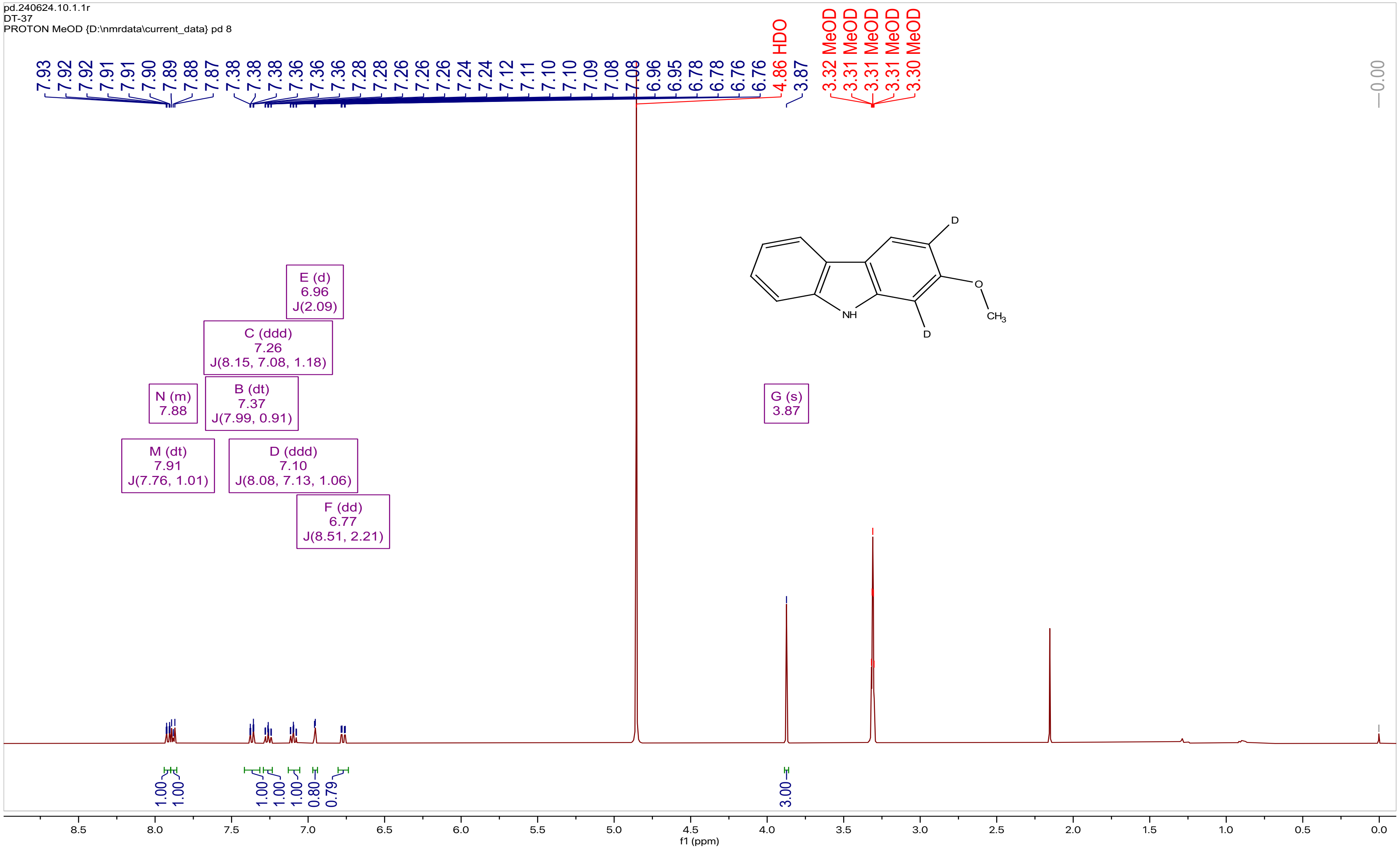

^{1}H NMR (400 MHz, MeOD) of 4be’

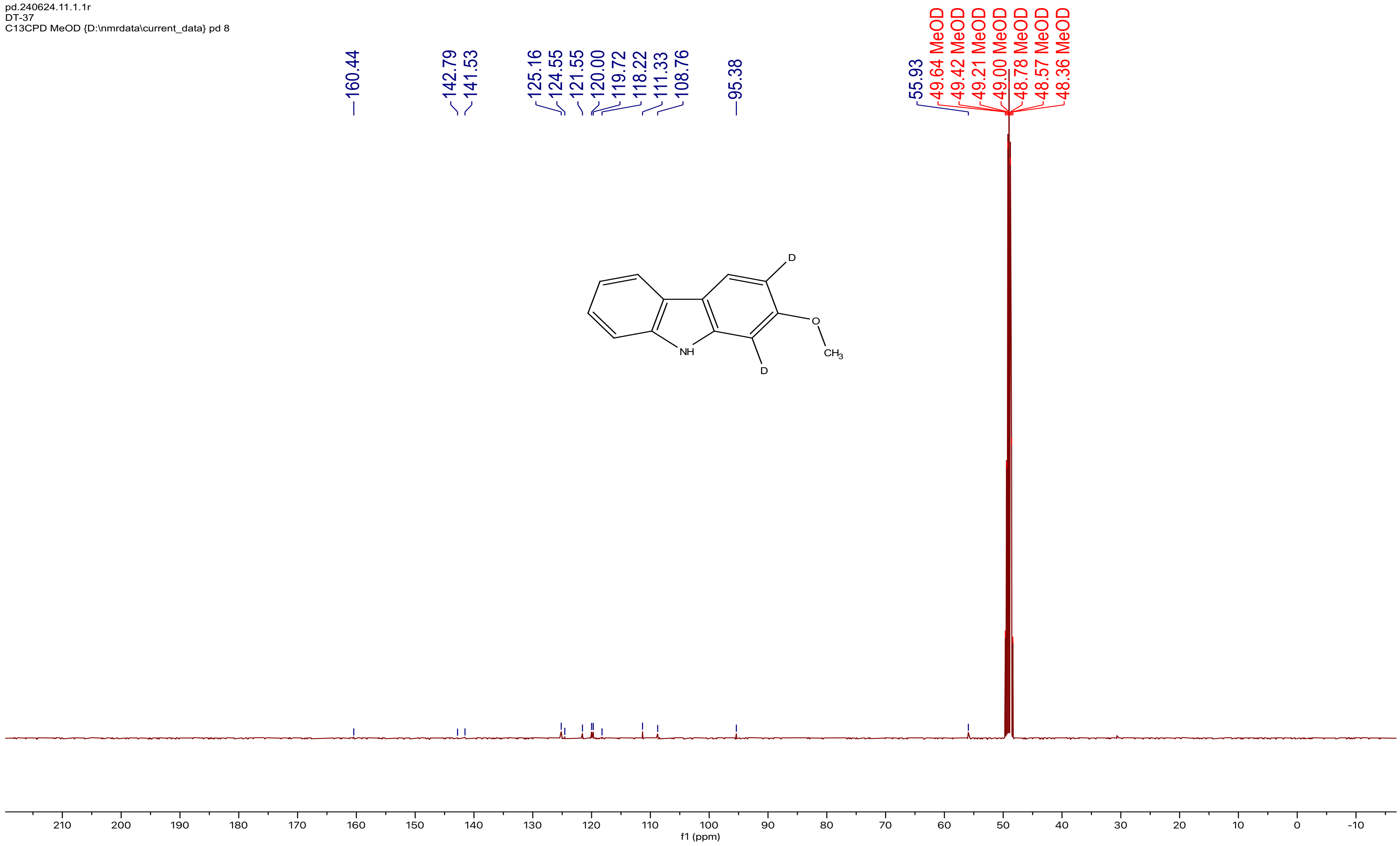

$^{13}C\{^1H\}$ NMR (101 MHz, MeOD) of 4be'

3f

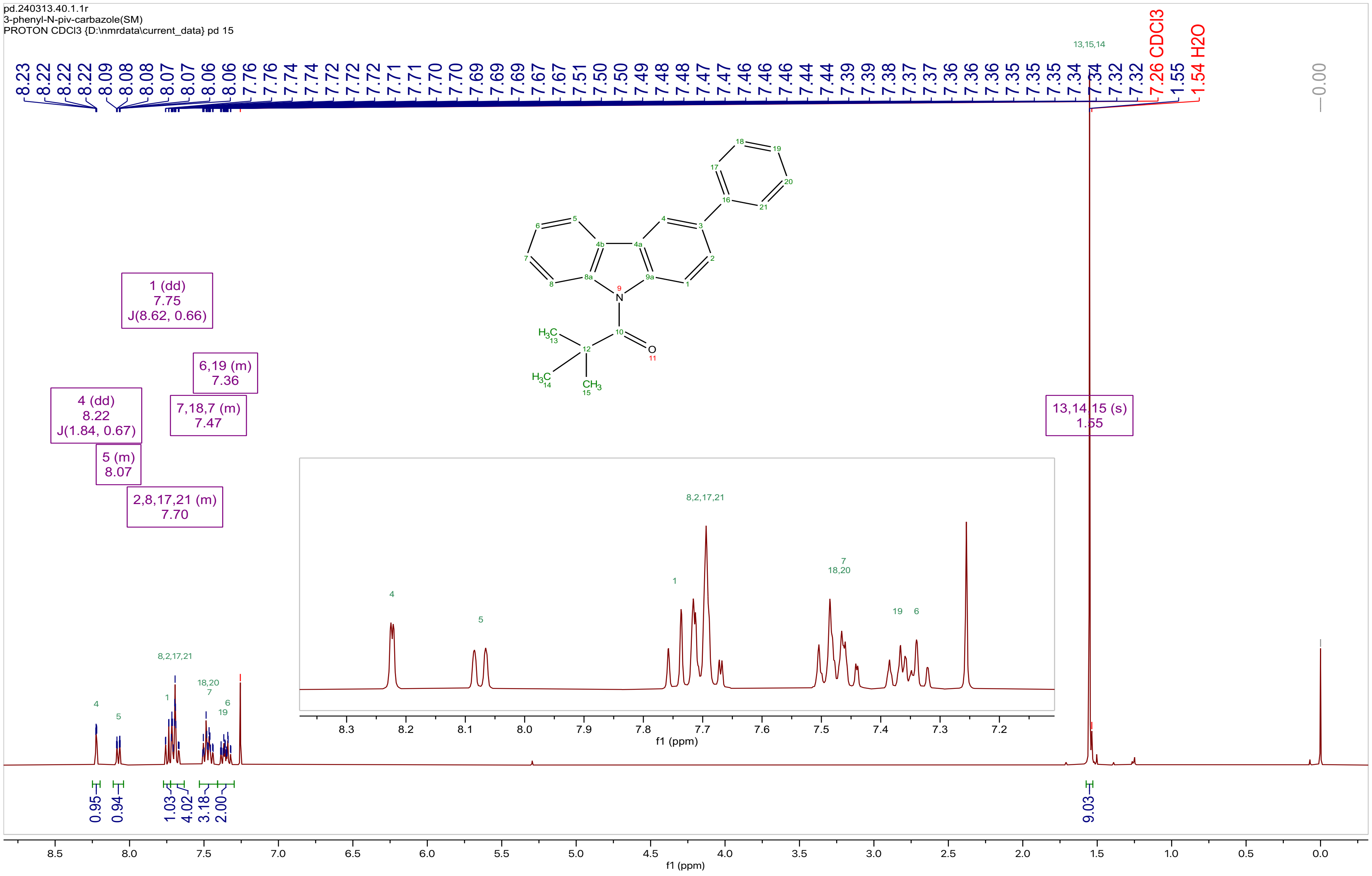

^{1}H NMR (400 MHz, $CDCl_3$) of 3f

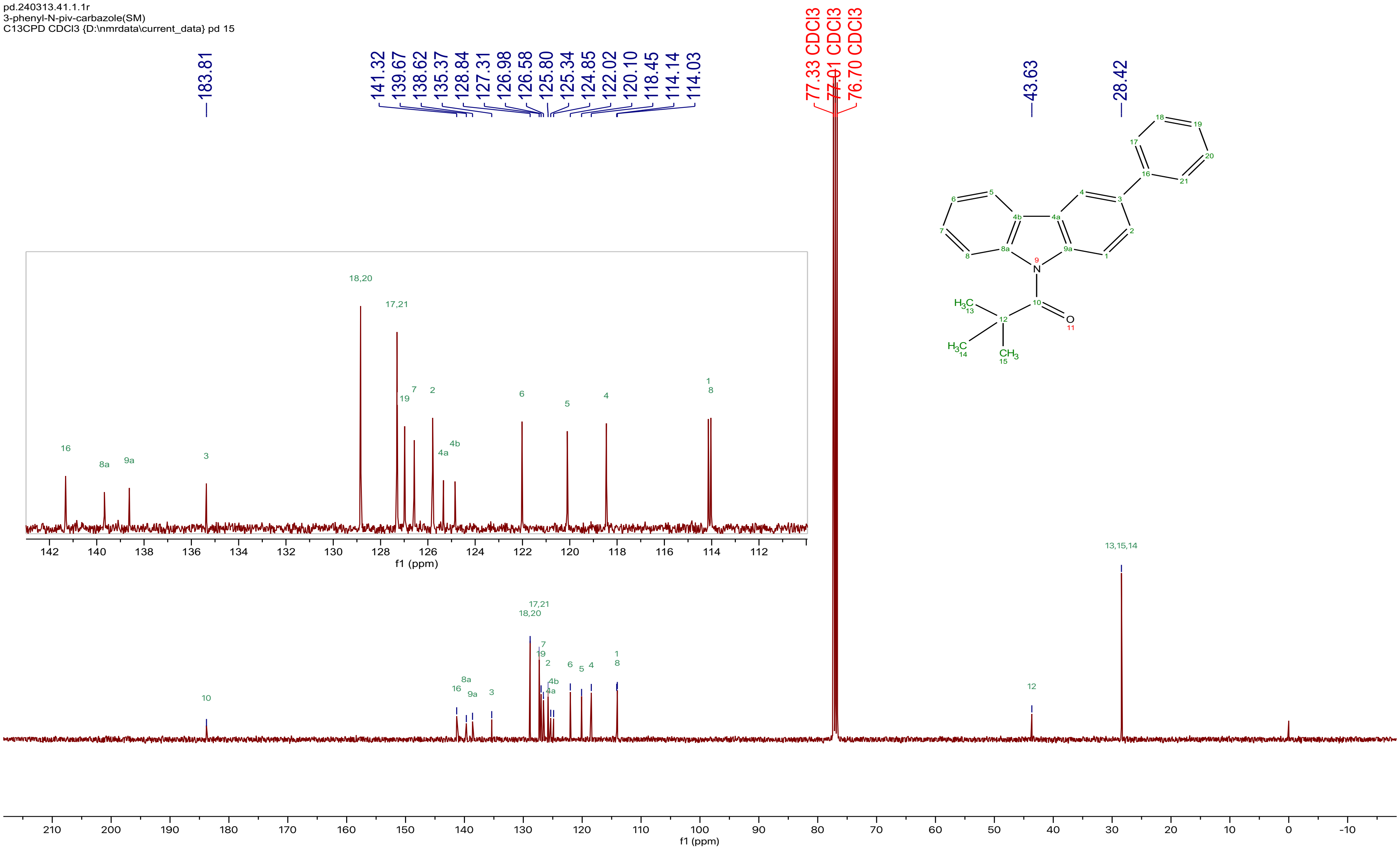

$^{13}C\{^{1}H\}$ NMR (101 MHz, $CDCl_3$) of 3f

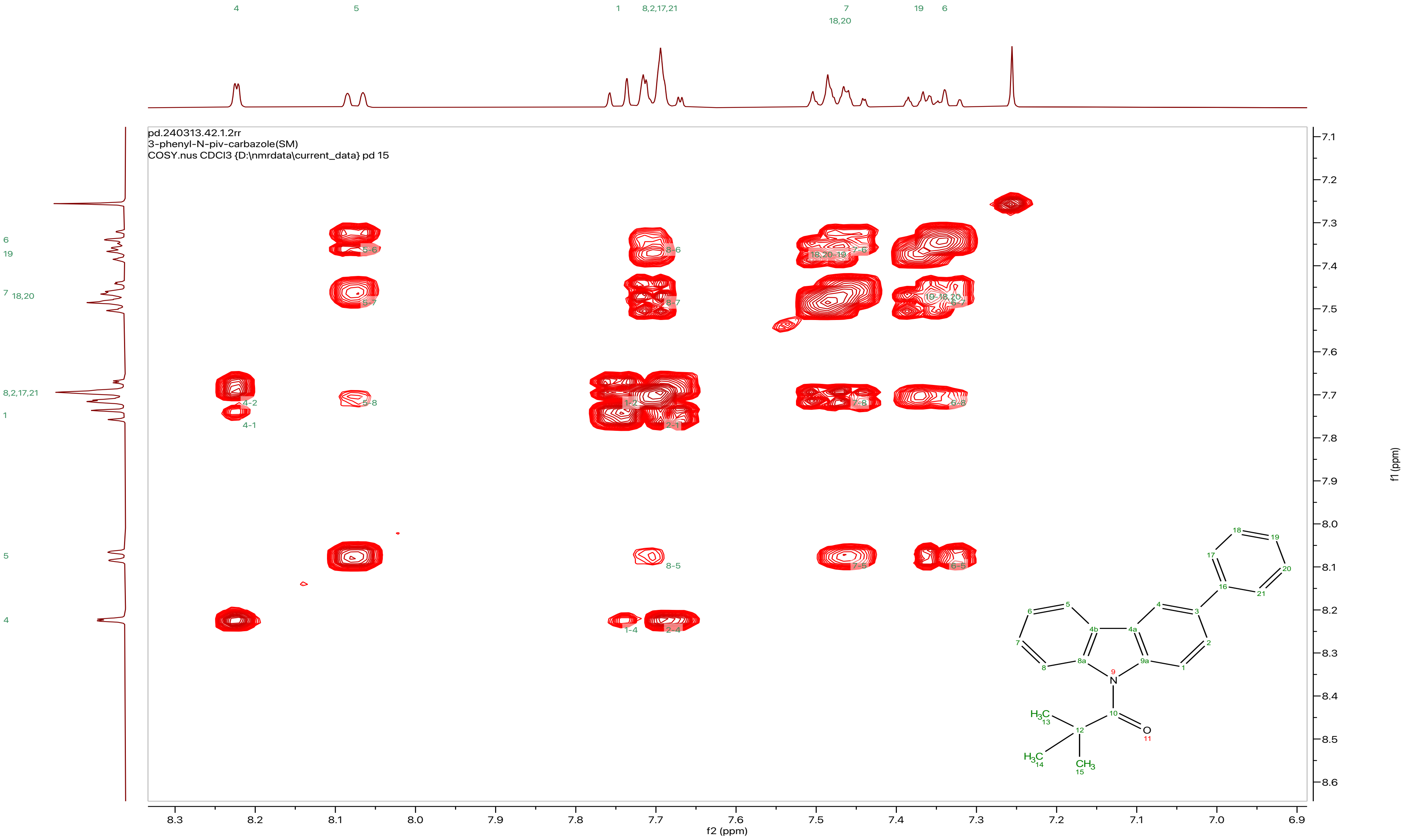

^{1}H-^{1}H COSY (400 MHz, $CDCl_3$) of 3f

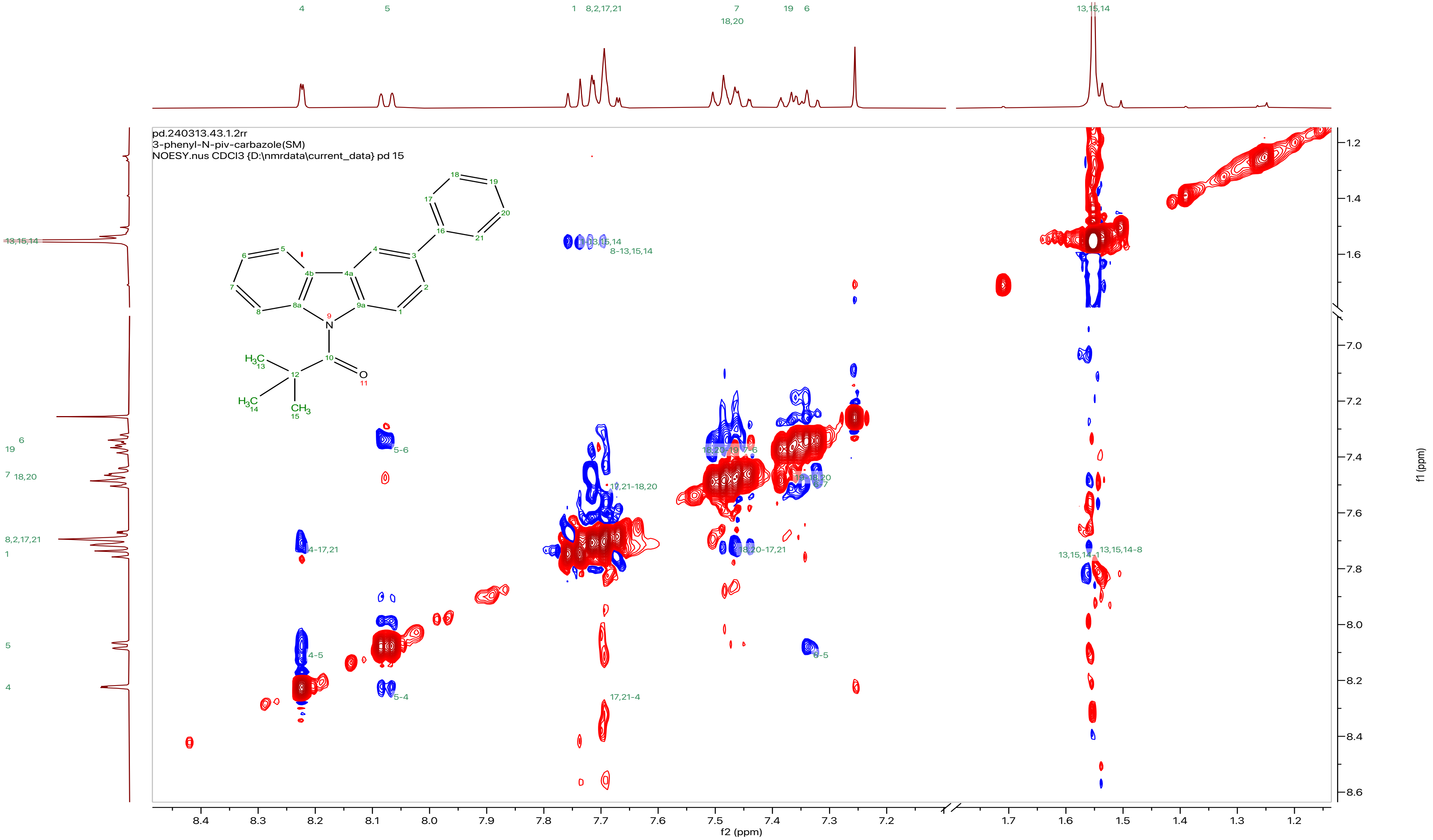

^{1}H-^{1}H NOESY (400 MHz, $CDCl_3$) of 3f

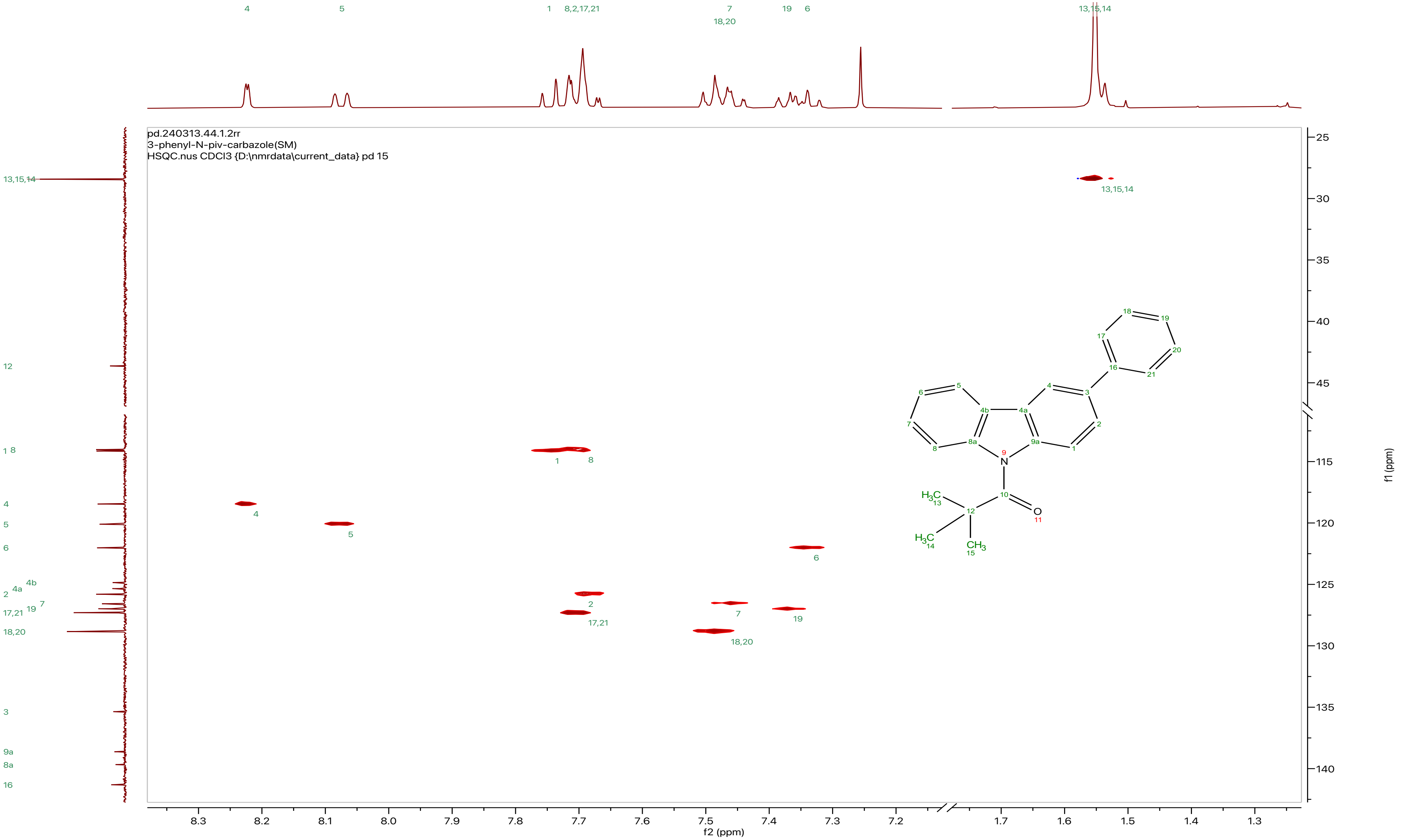

^{1}H-$^{13}C\{^{1}H\}$ HSQC NMR (400/101 MHz, $CDCl_3$) of 3f

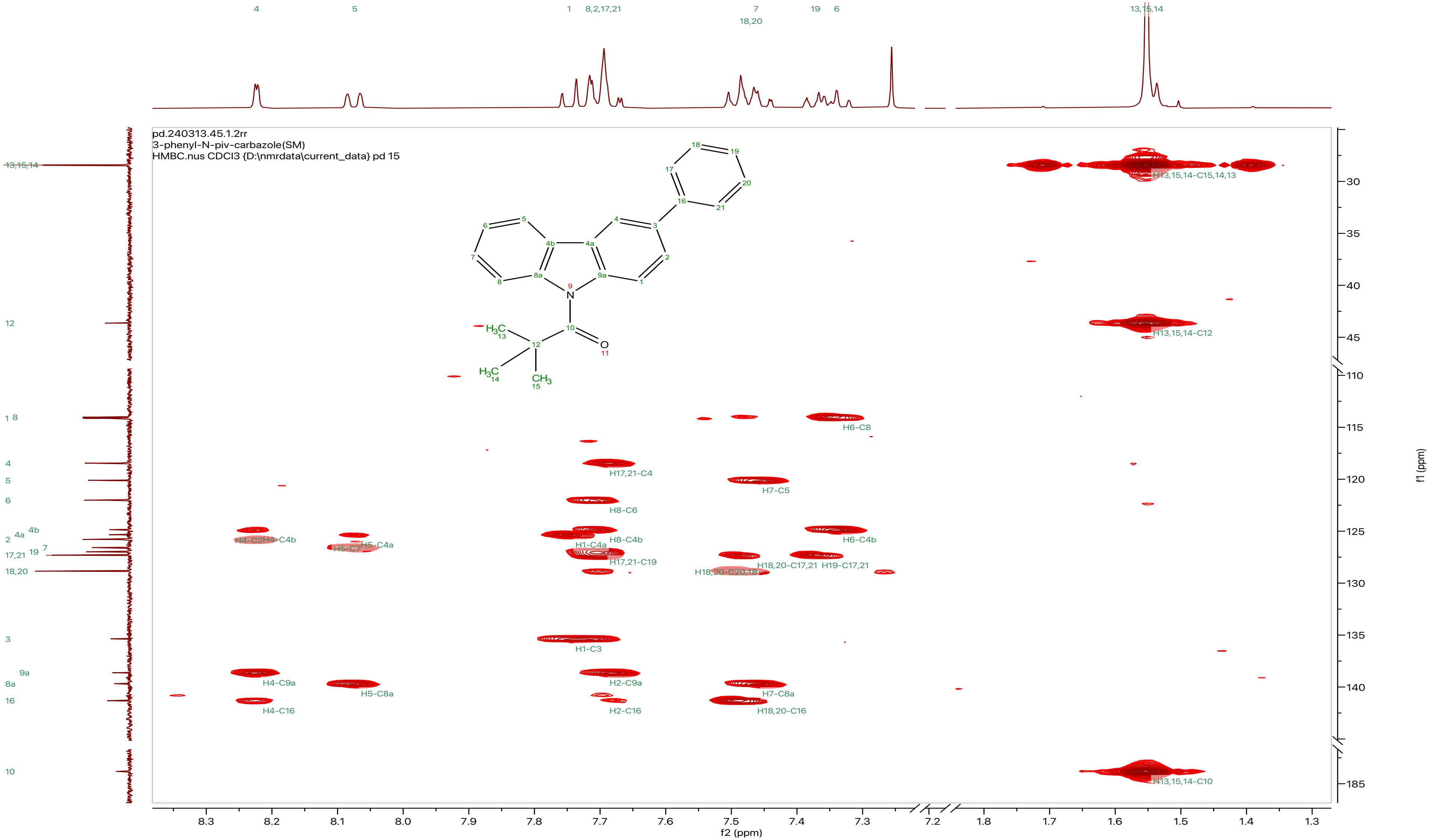

^{1}H-$^{13}C\{^{1}H\}$ HMBC NMR (400/101 MHz, $CDCl_3$) of 3f

4af

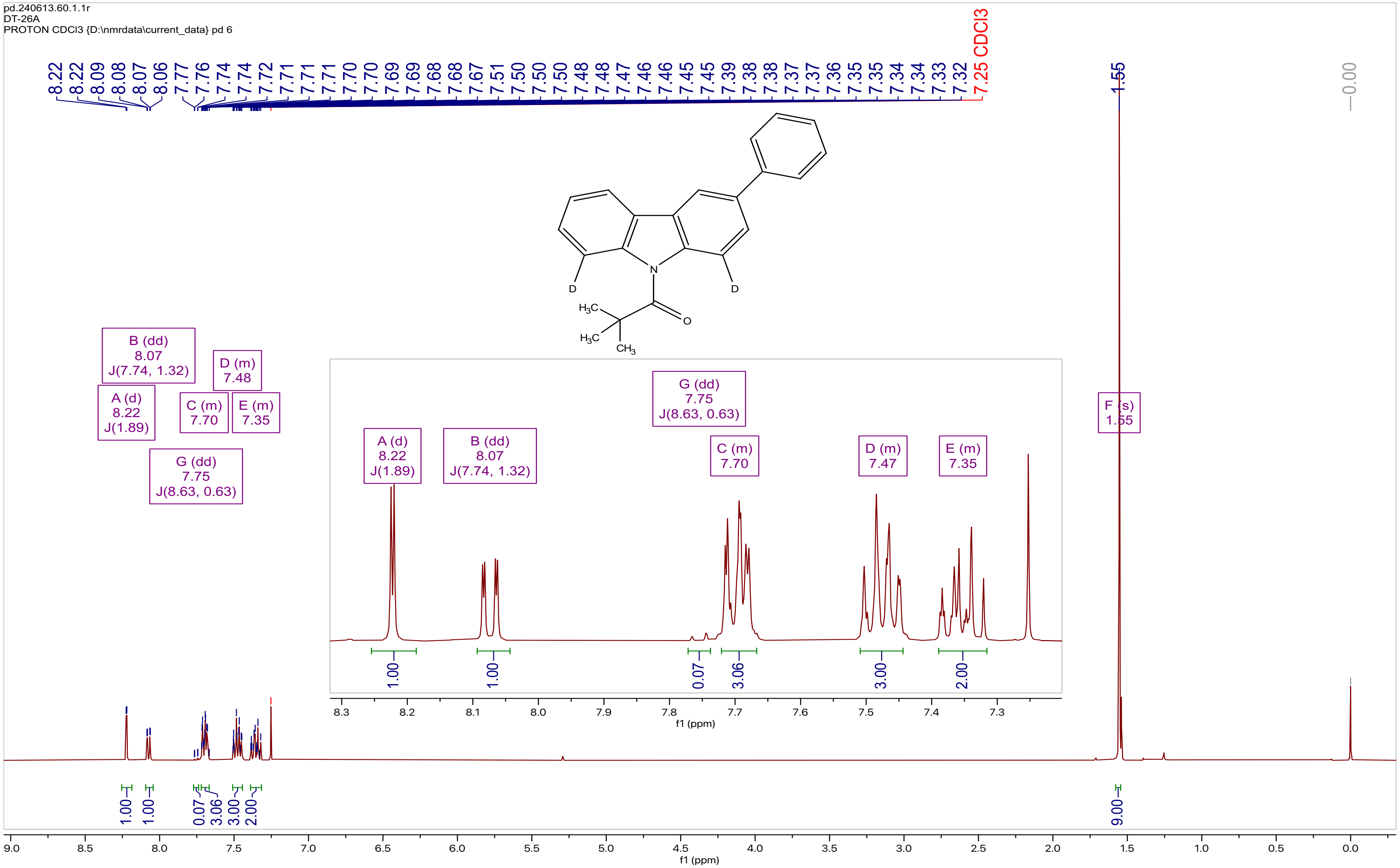

^{1}H NMR (400 MHz, $CDCl_3$) of 4af

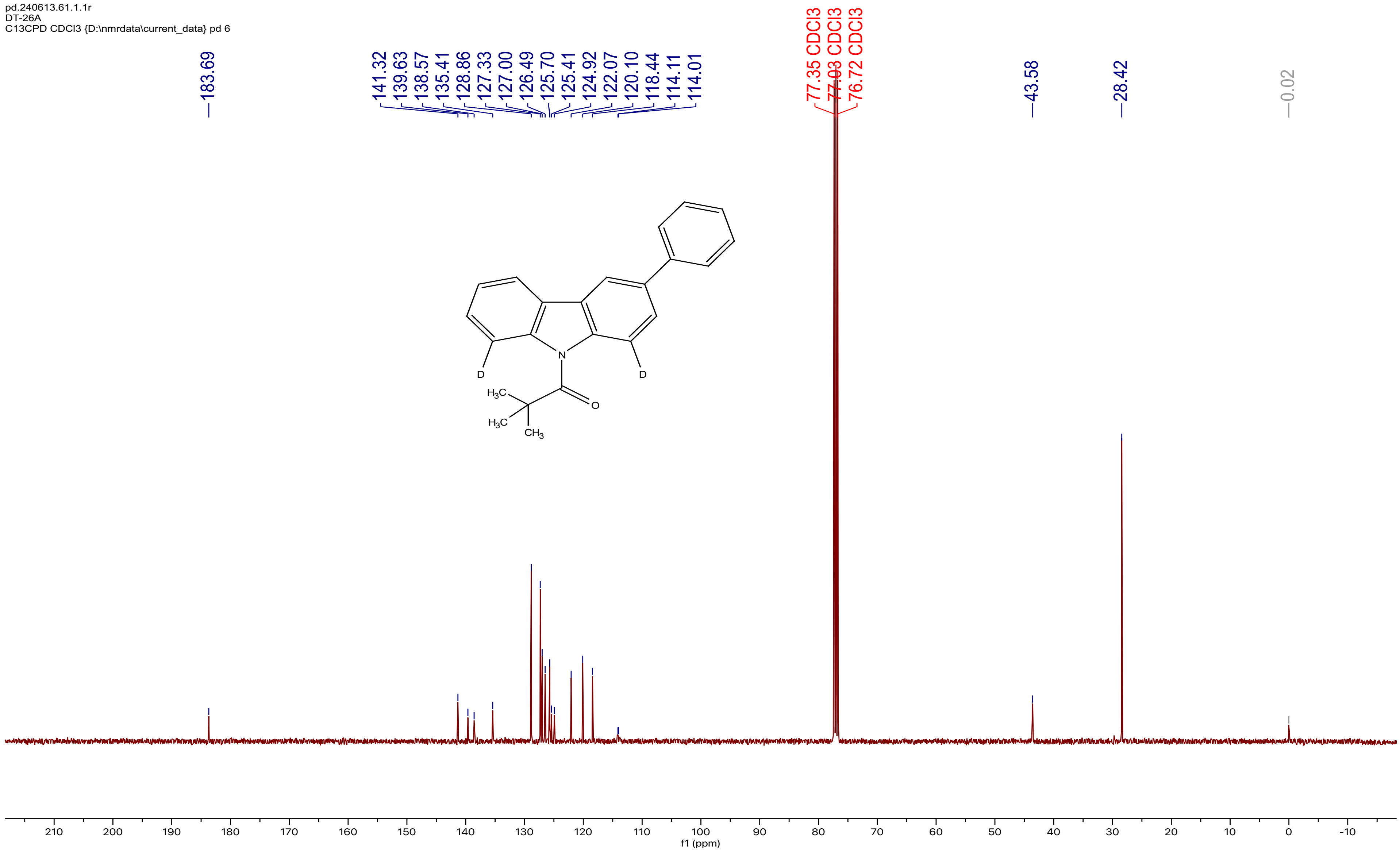

$^{13}C\{^1H\}$ NMR (101 MHz, $CDCl_3$) of 4af

3f'

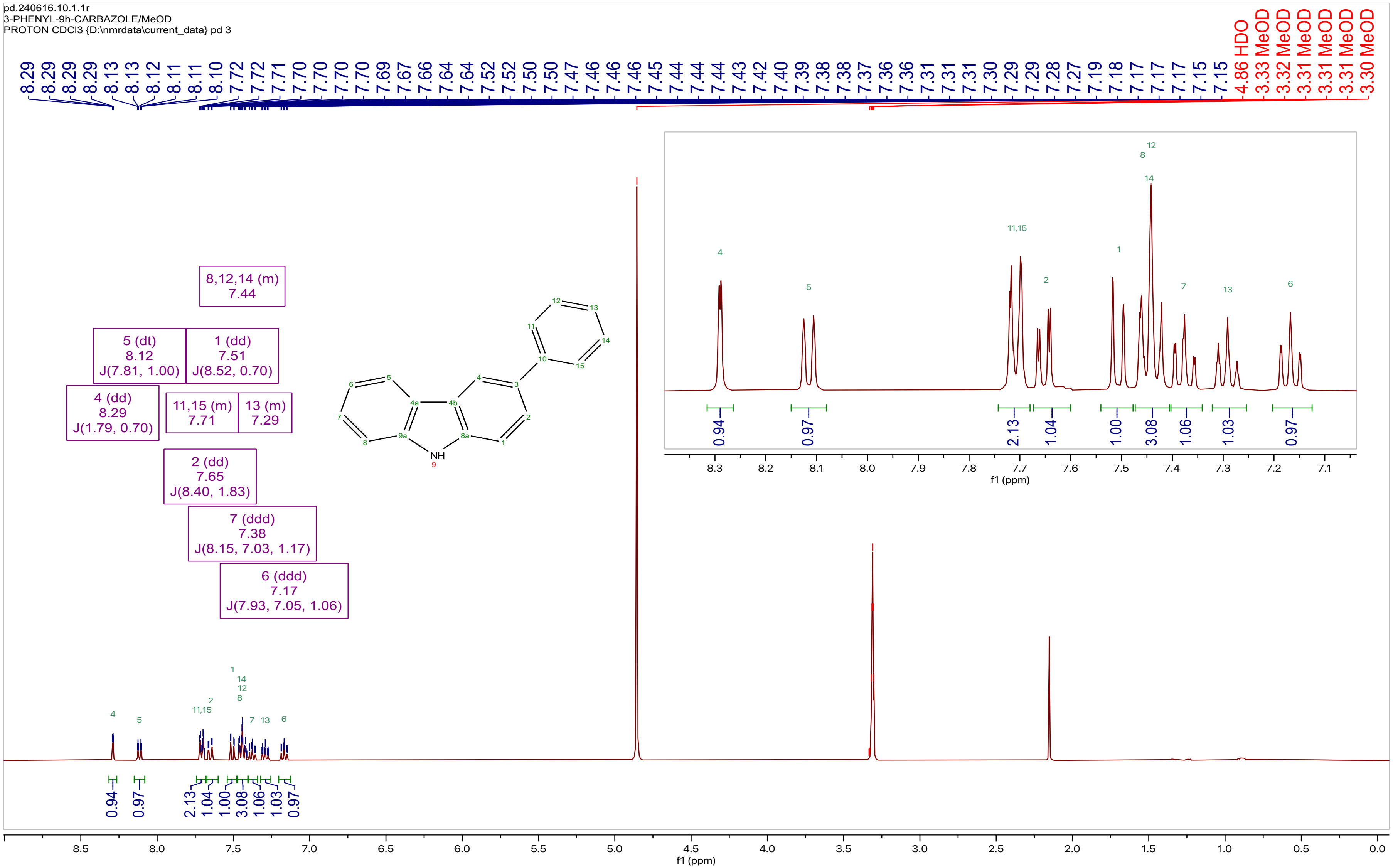

^{1}H NMR (400 MHz, $CDCl_3$) of 3f'

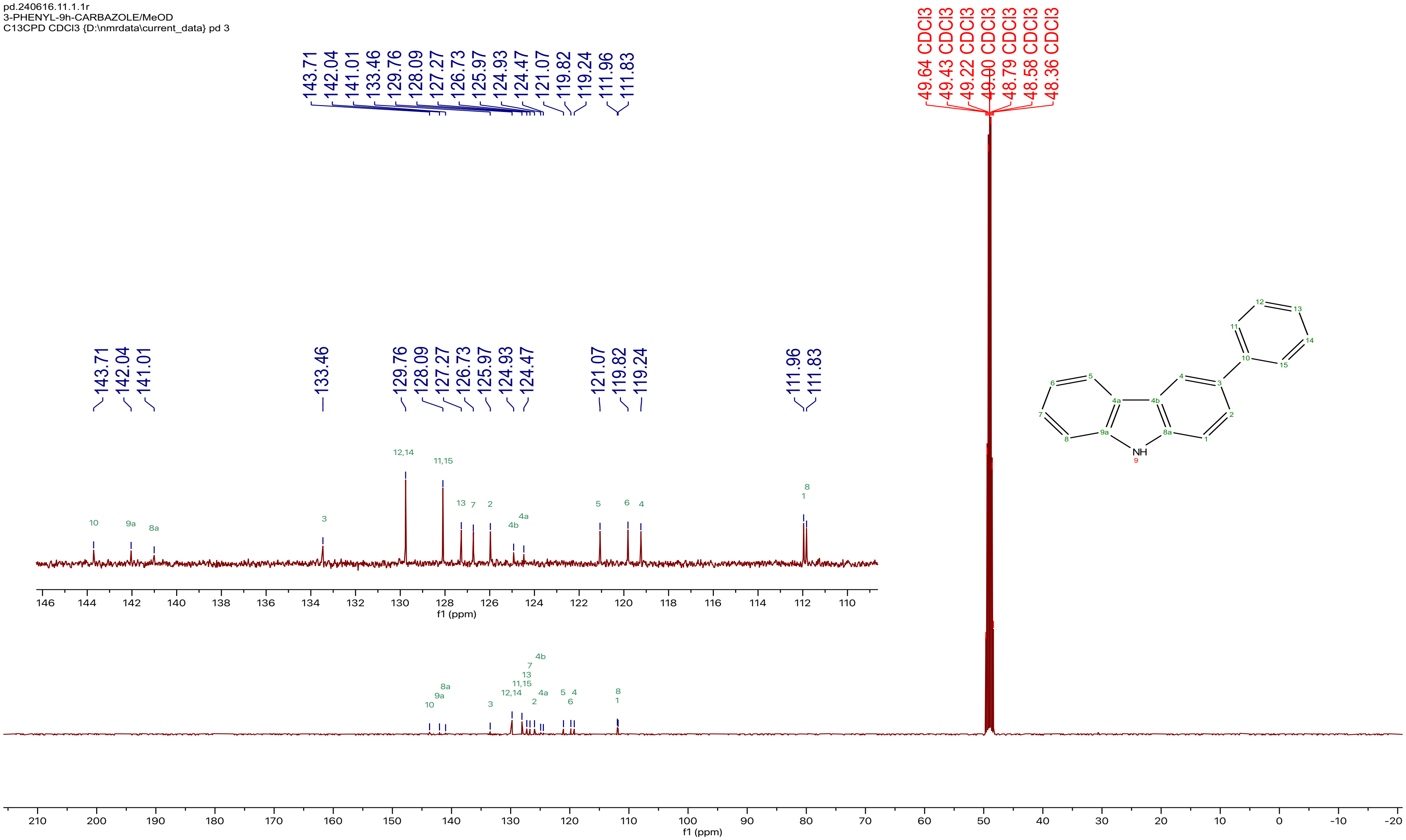

$^{13}C\{^{1}H\}$ NMR (101 MHz, $CDCl_3$) of 3f'

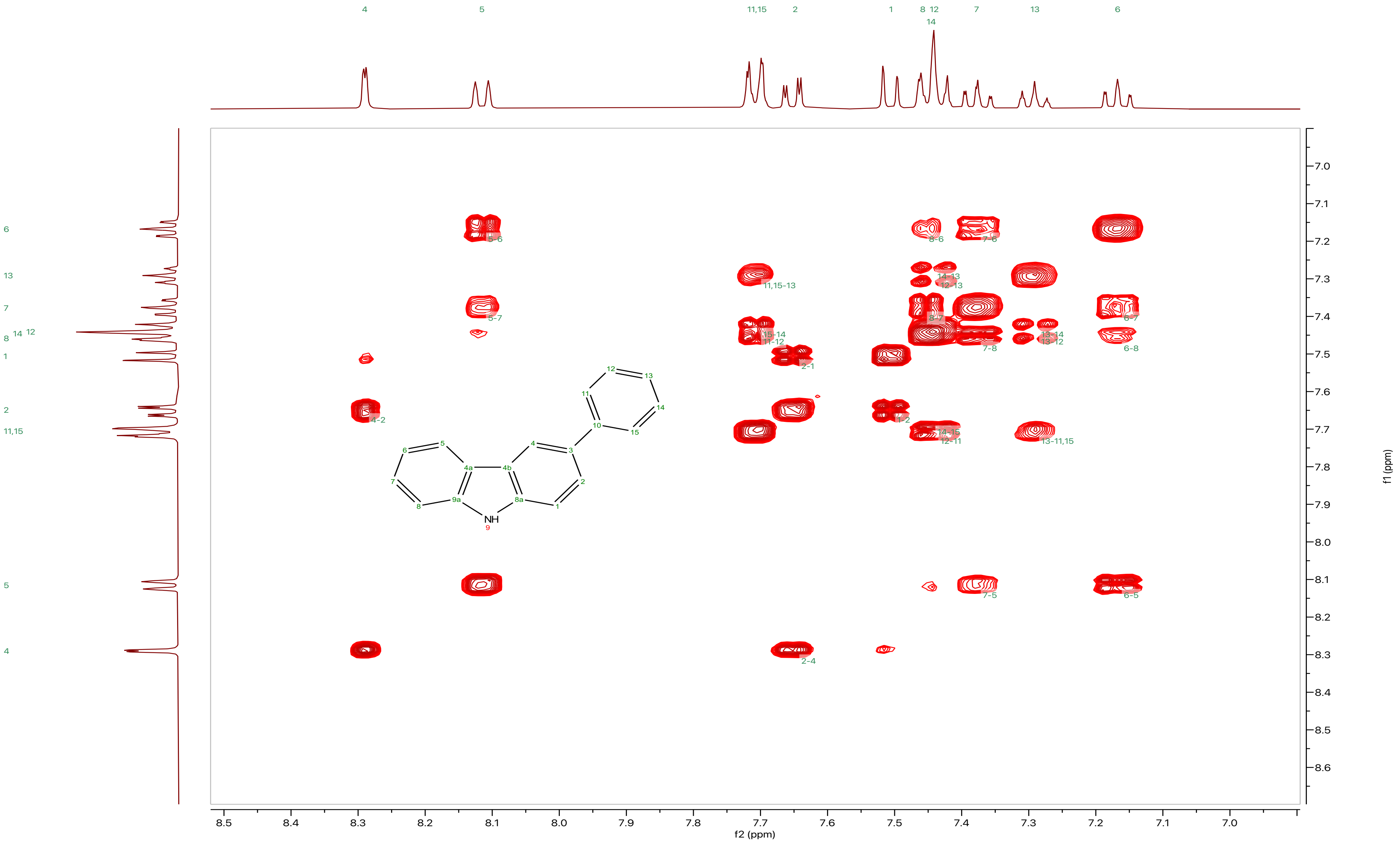

^1H-^1H COSY (400 MHz, $CDCl_3$) of 3f'

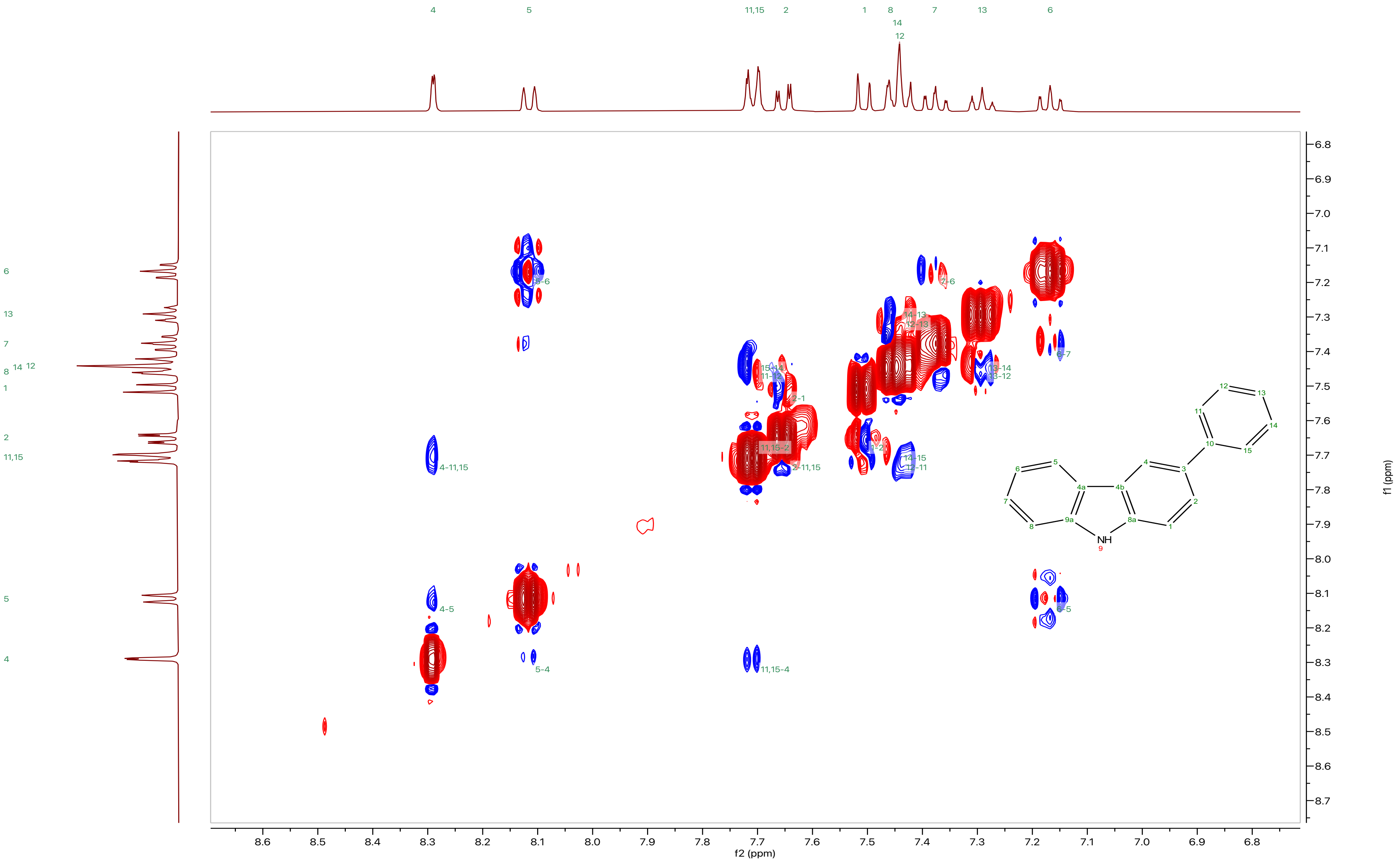

^1H-^1H NOESY (400 MHz, $CDCl_3$) of 3f'

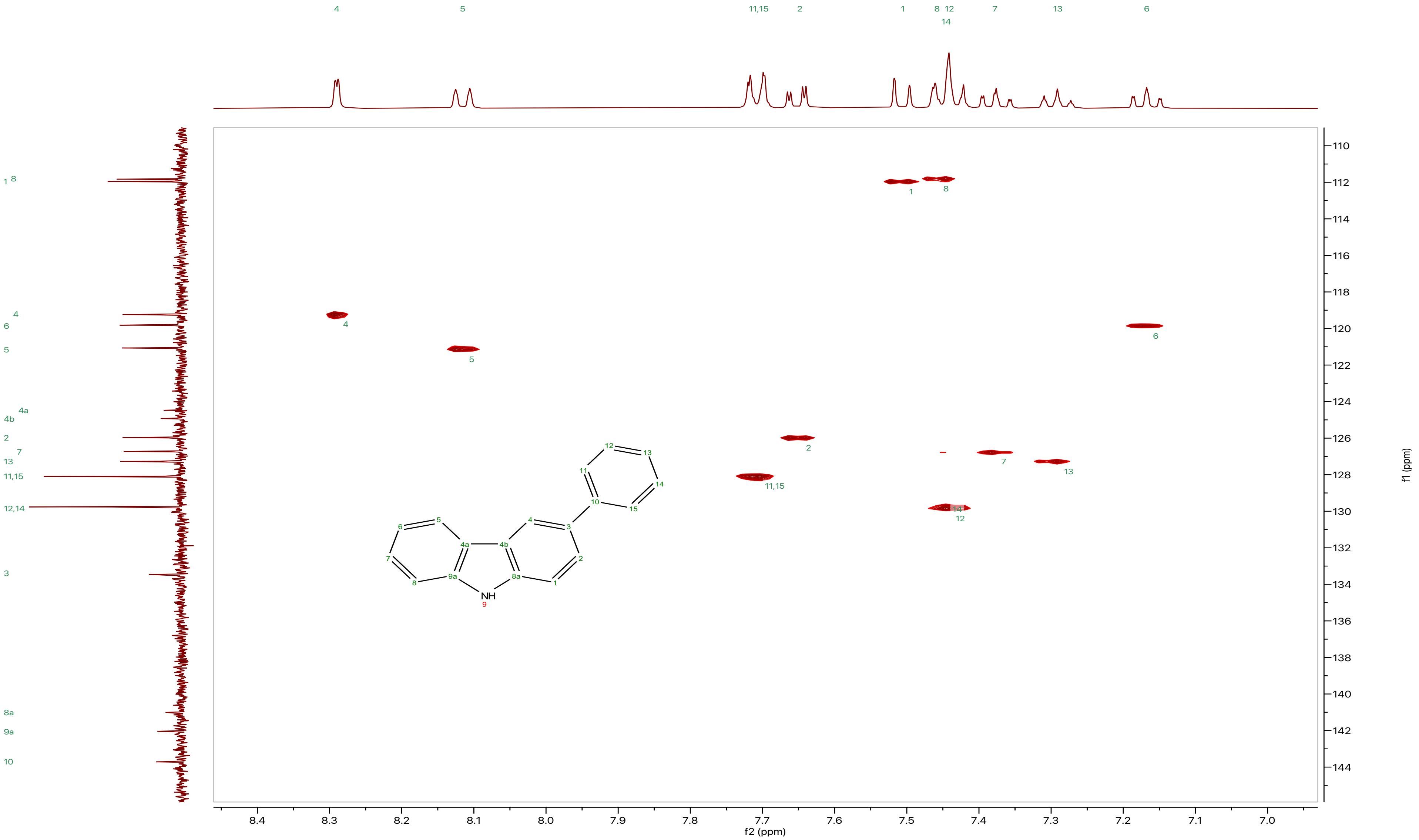

^{1}H-$^{13}C\{^{1}H\}$ HSQC NMR (400/101 MHz, $CDCl_3$) of 3f'

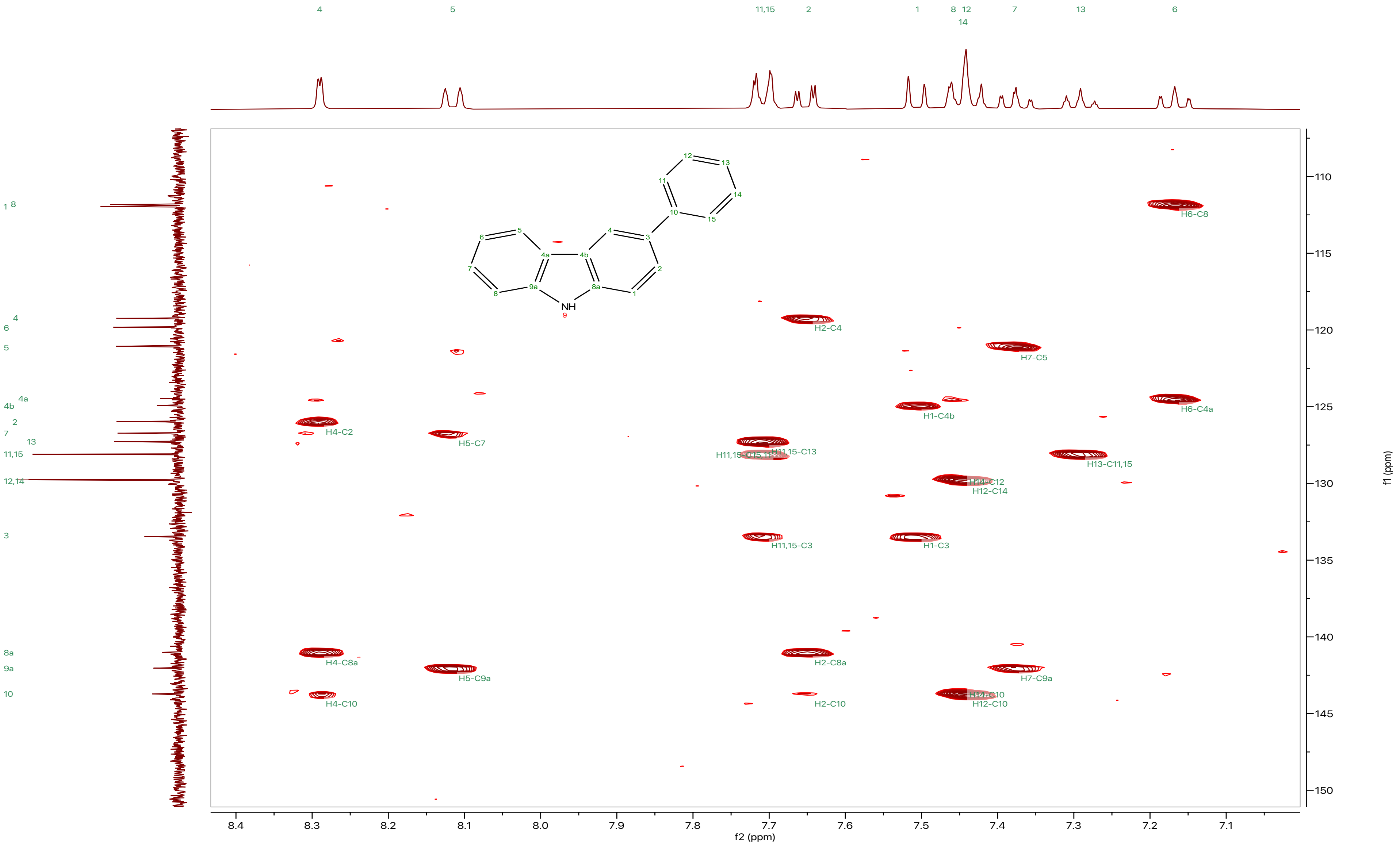

^{1}H-$^{13}C\{^{1}H\}$ HMBC NMR (400/101 MHz, $CDCl_3$) of 3f'

4bf

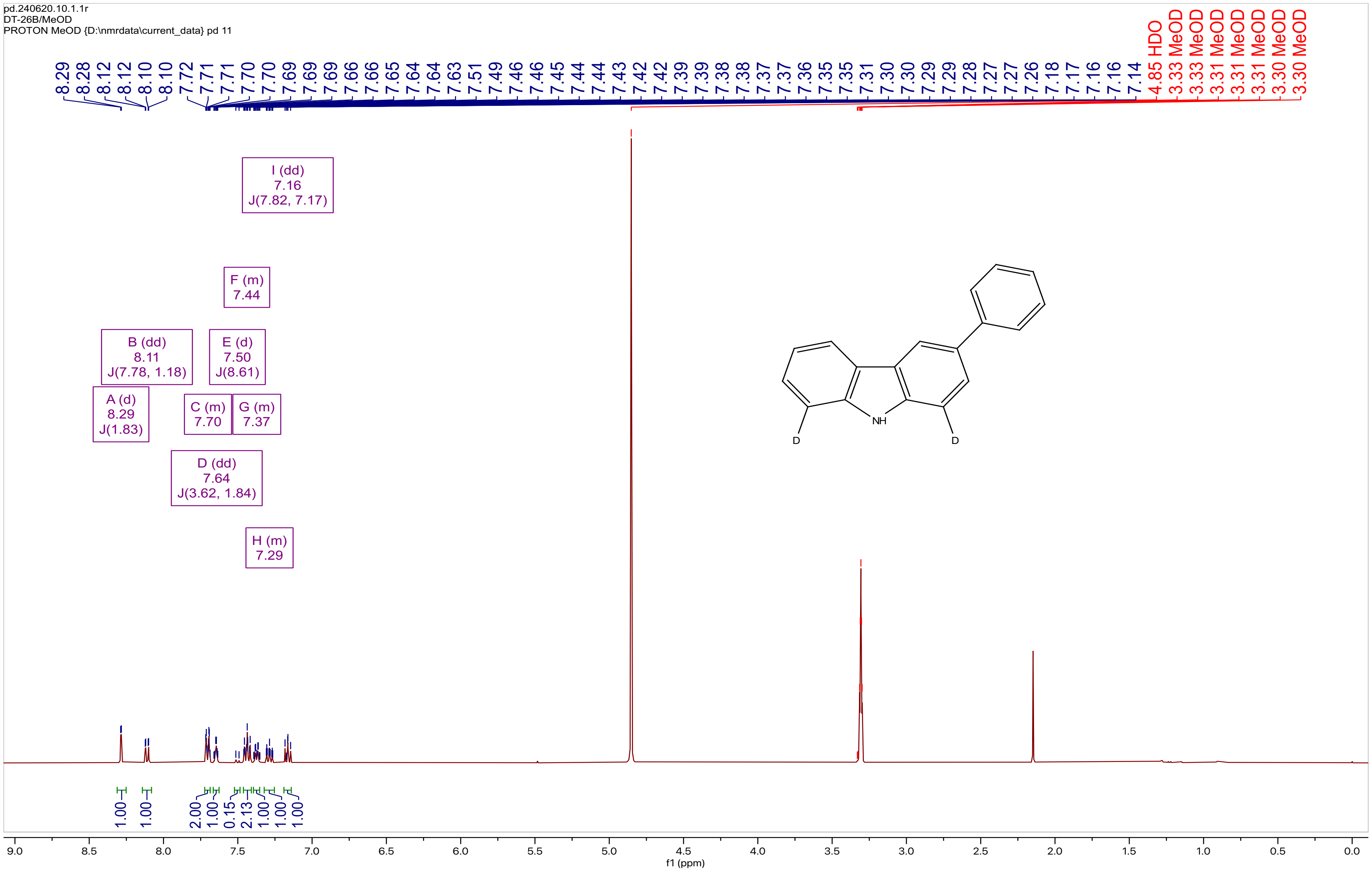

^{1}H NMR (400 MHz, MeOD) of 4bf

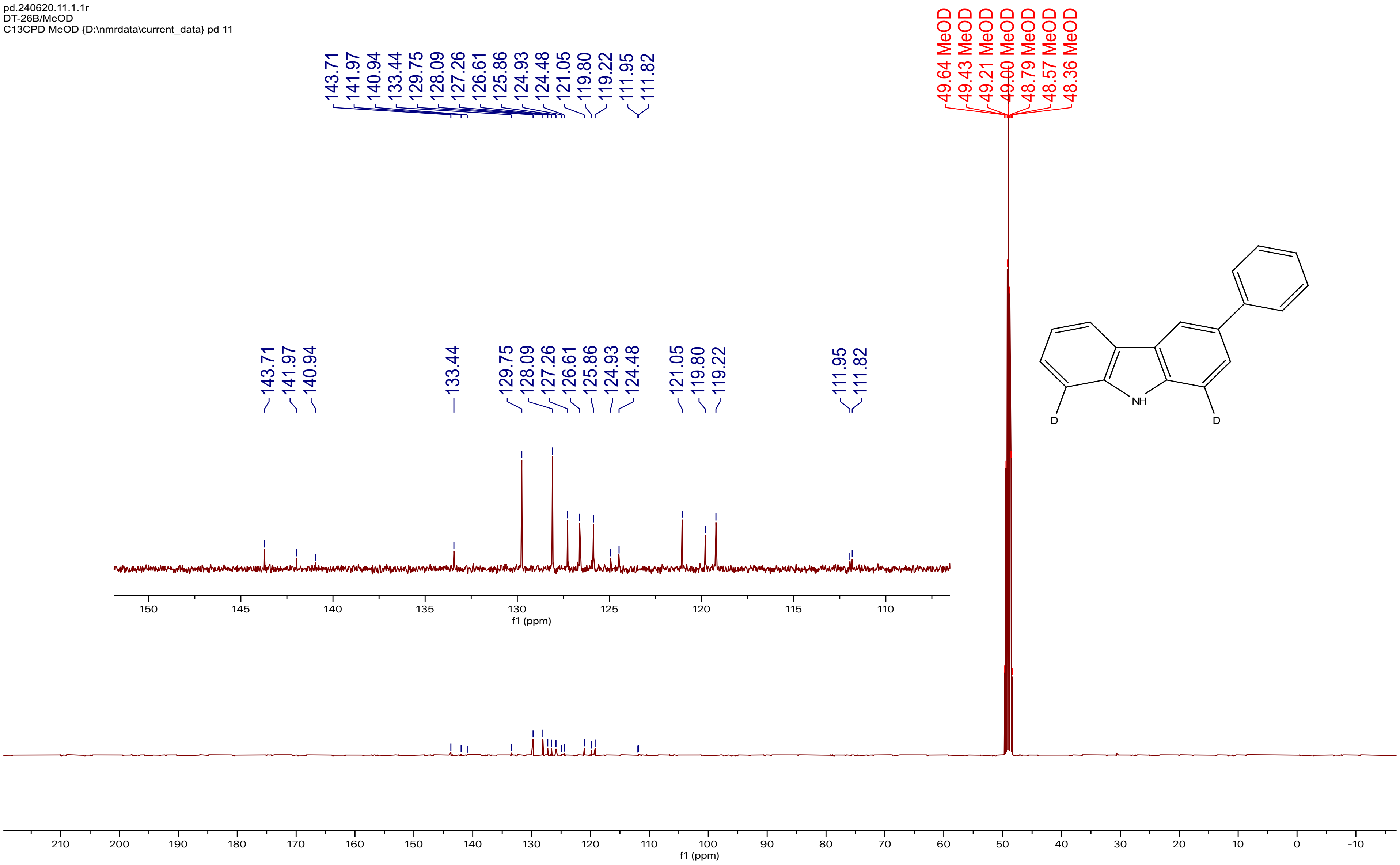

$^{13}C\{^{1}H\}$ NMR (101 MHz, MeOD) of 4bf

3g

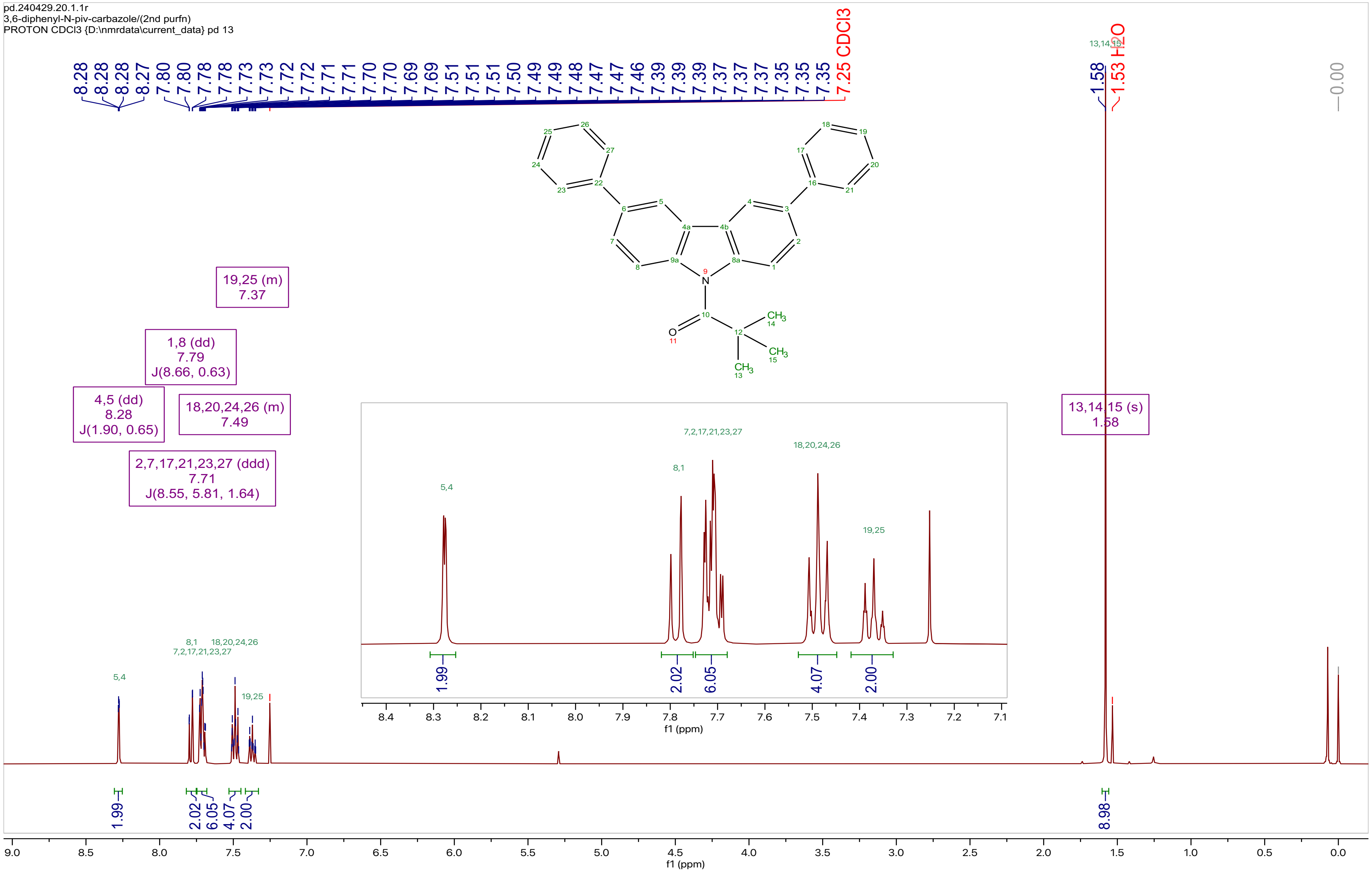

^{1}H NMR (400 MHz, $CDCl_3$) of 3g

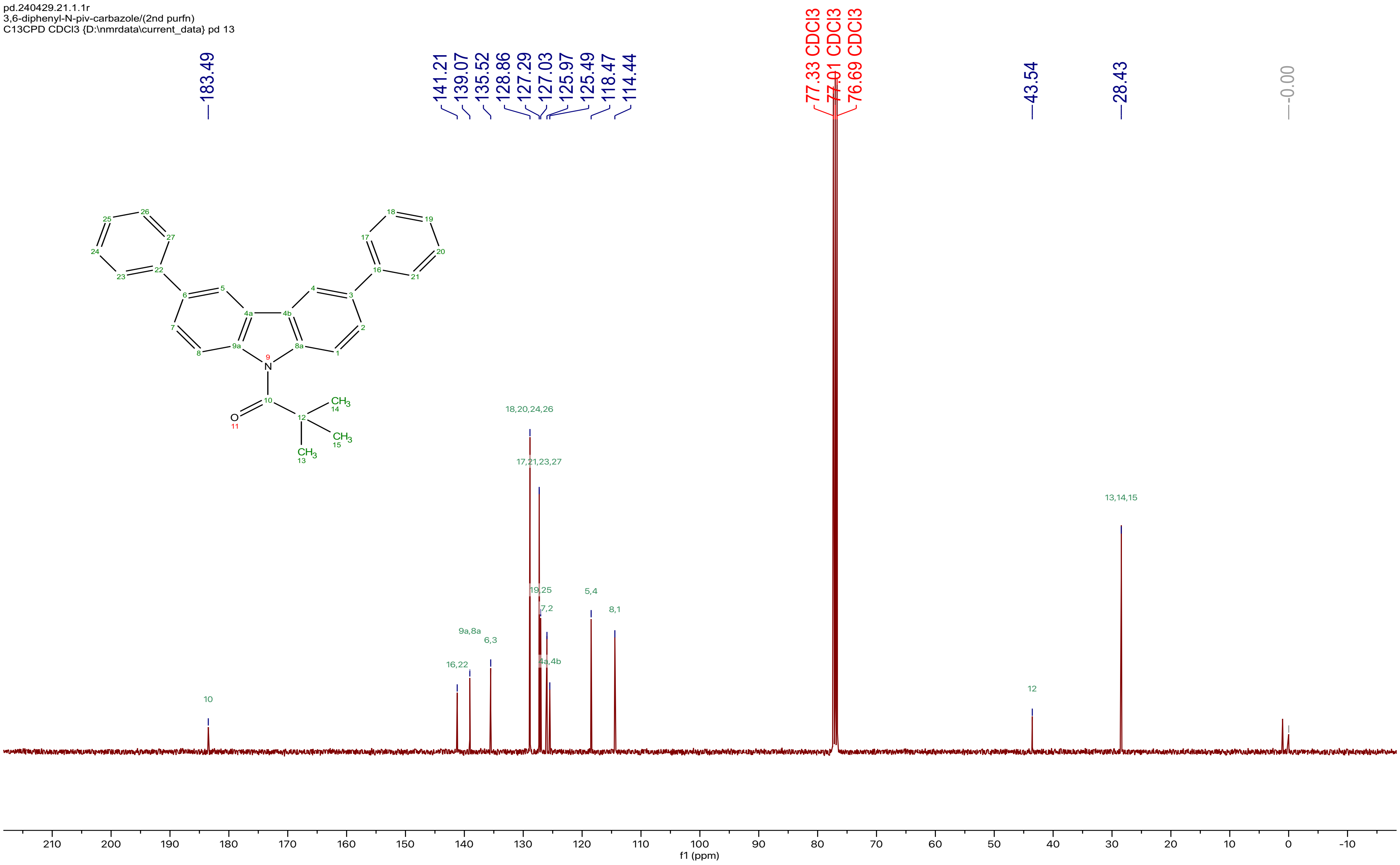

$^{13}C\{^{1}H\}$ NMR (101 MHz, $CDCl_3$) of 3g

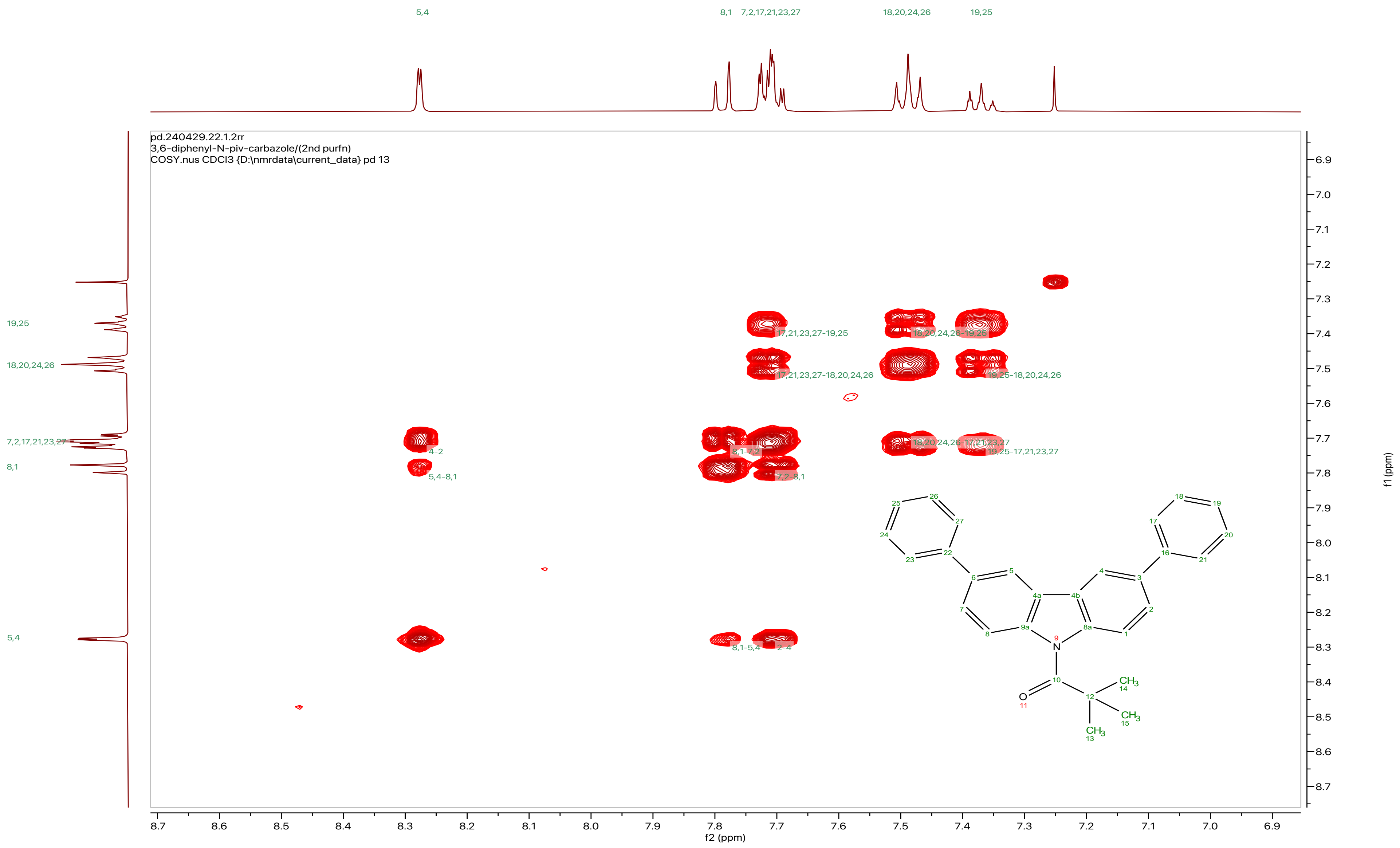

^{1}H-^{1}H COSY (400 MHz, $CDCl_3$) of 3g

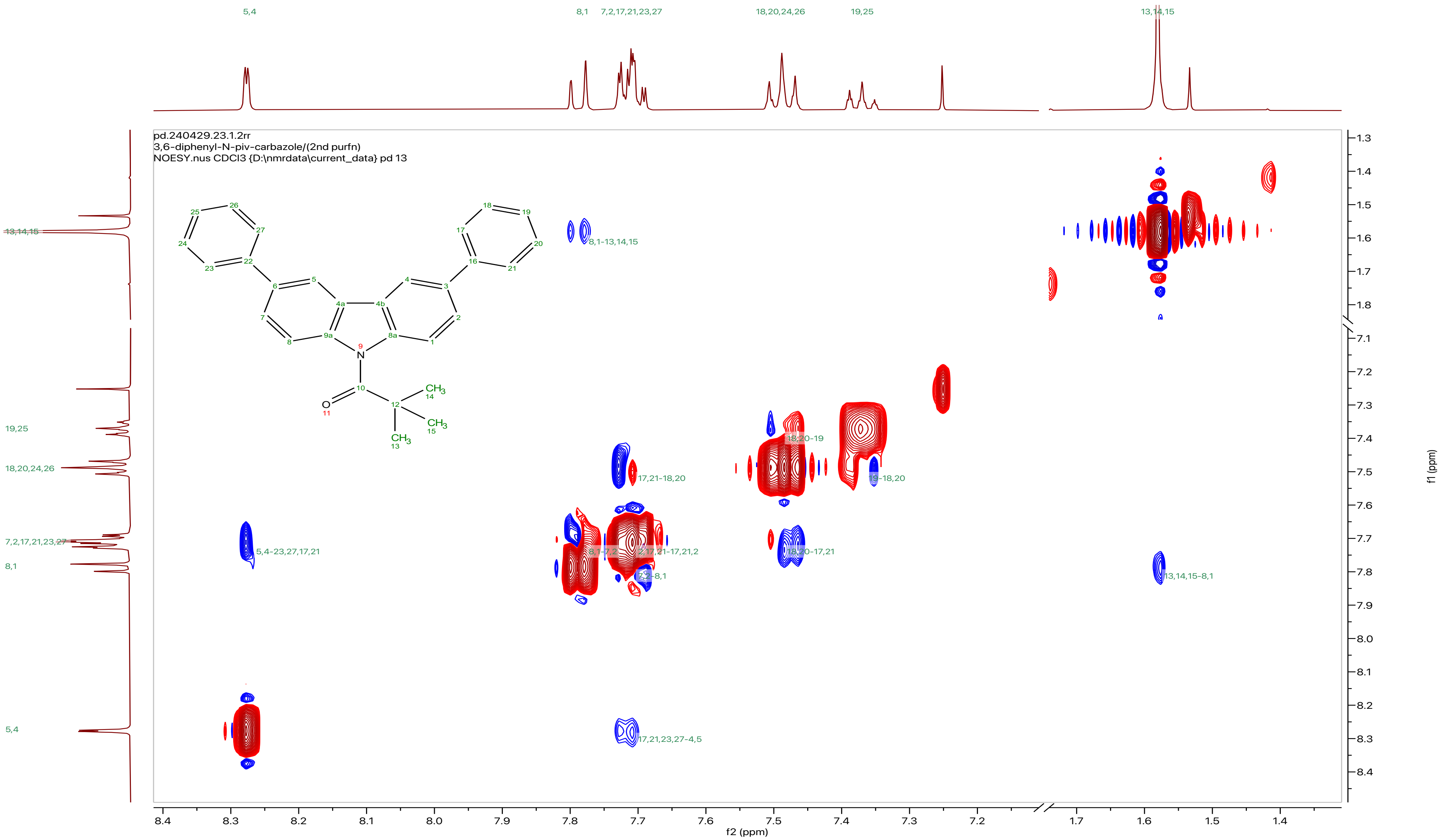

^{1}H-^{1}H NOESY (400 MHz, $CDCl_3$) of 3g

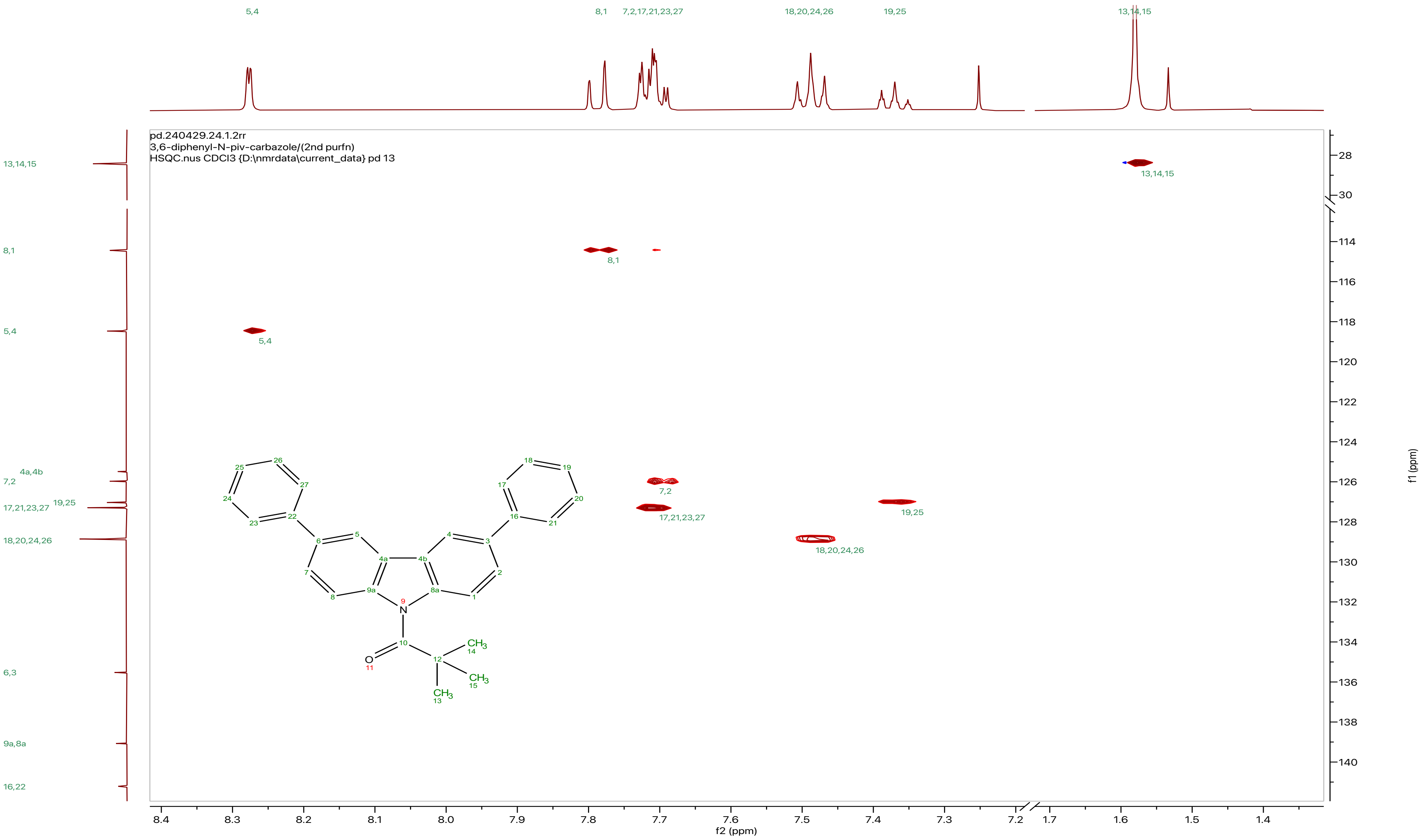

^{1}H-$^{13}C\{^{1}H\}$ HSQC NMR (400/101 MHz, $CDCl_3$) of 3g

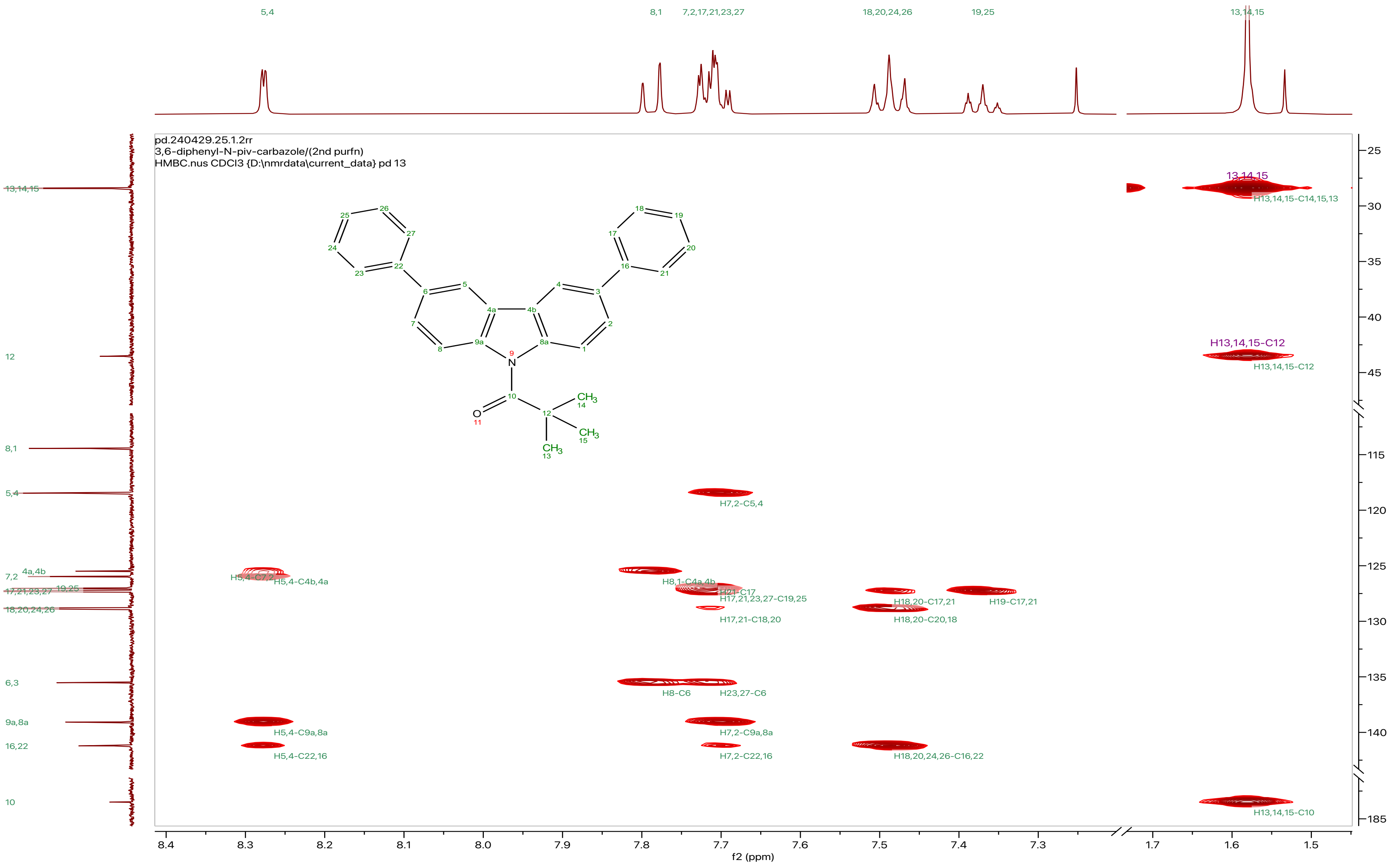

^1H-$^{13}C\{^1H\}$ HMBC NMR (400/101 MHz, $CDCl_3$) of 3g

4ag

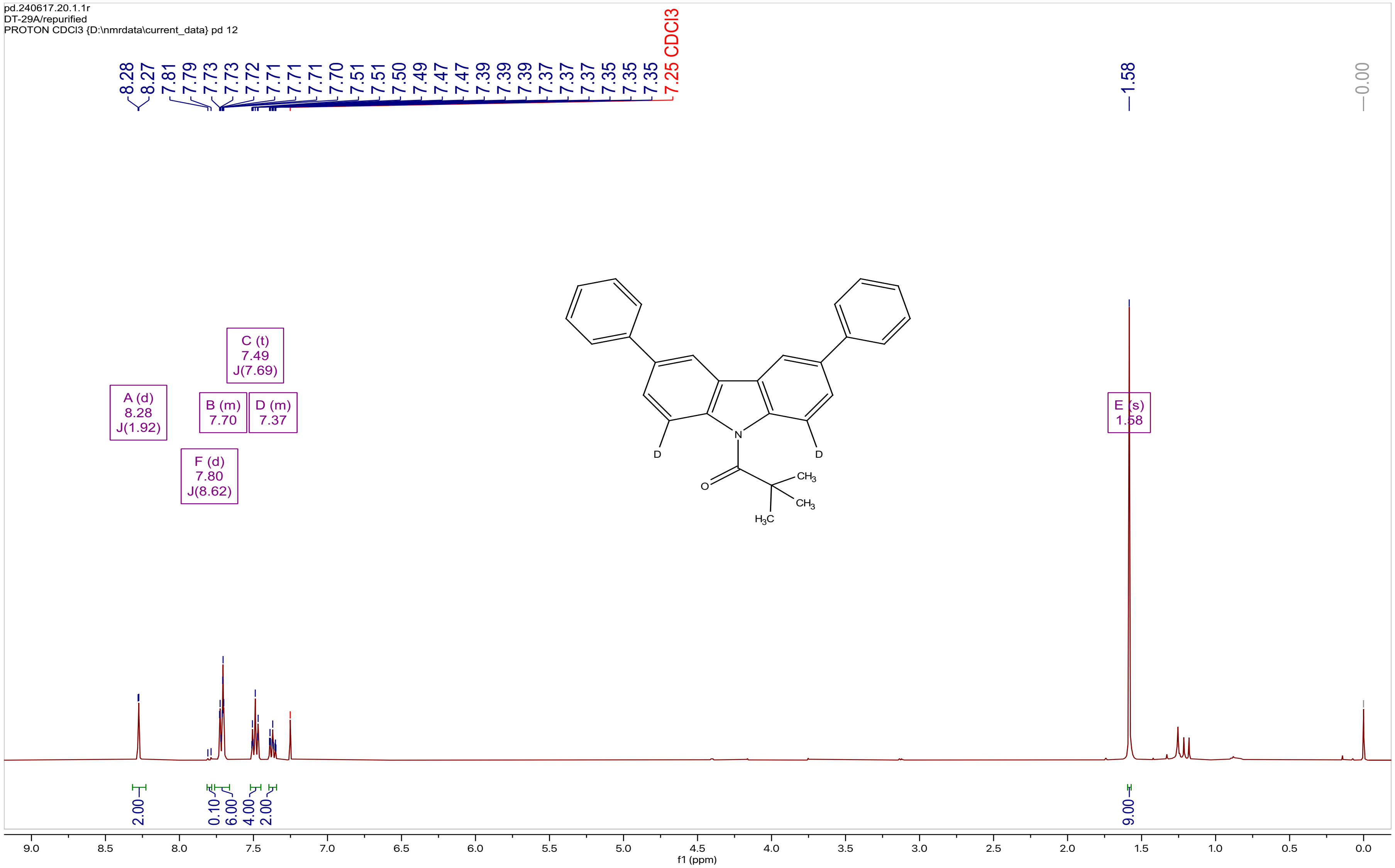

^{1}H NMR (400 MHz, $CDCl_3$) of 4ag

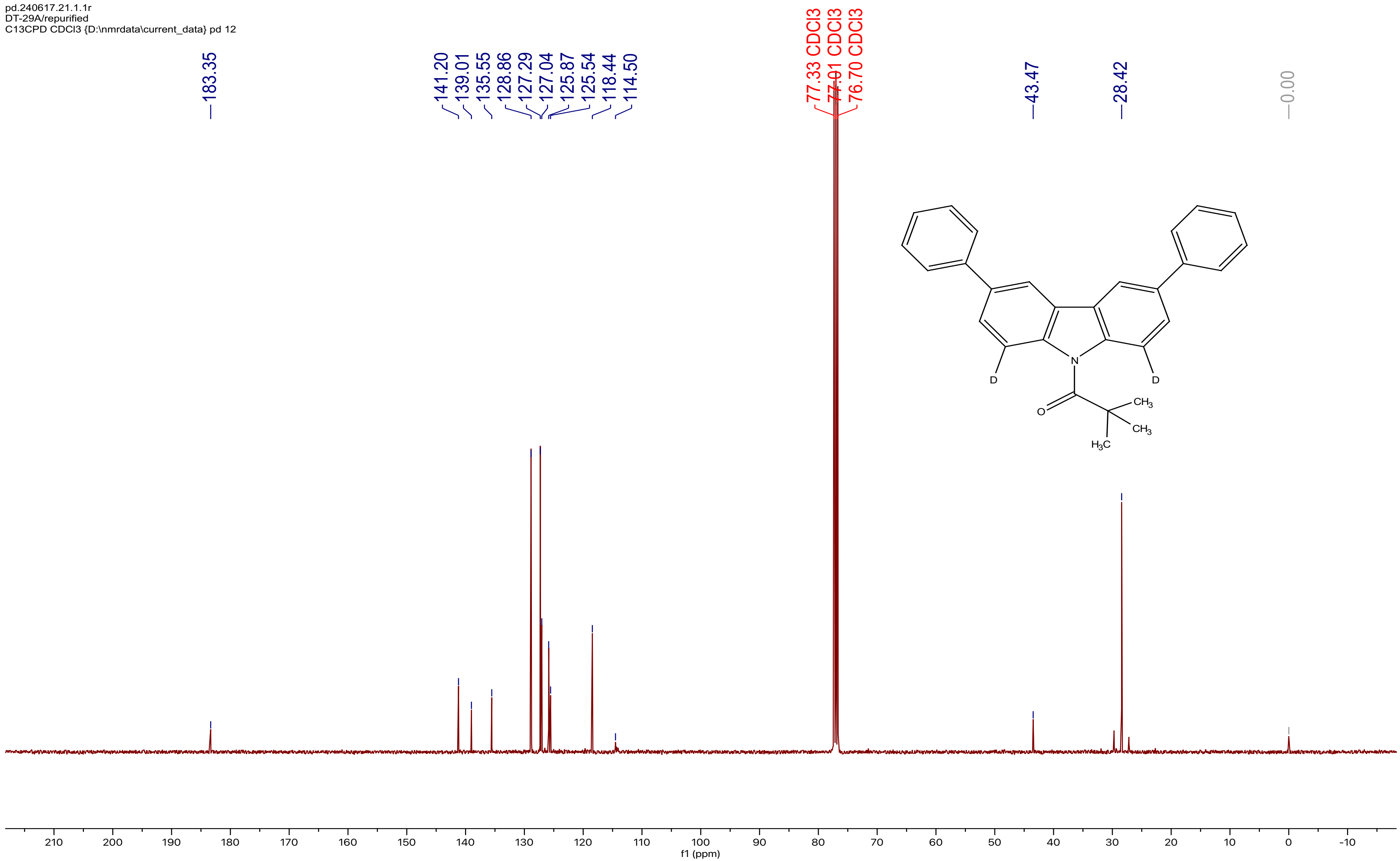

$^{13}C\{^{1}H\}$ NMR (101 MHz, $CDCl_3$) of 4ag

3g’

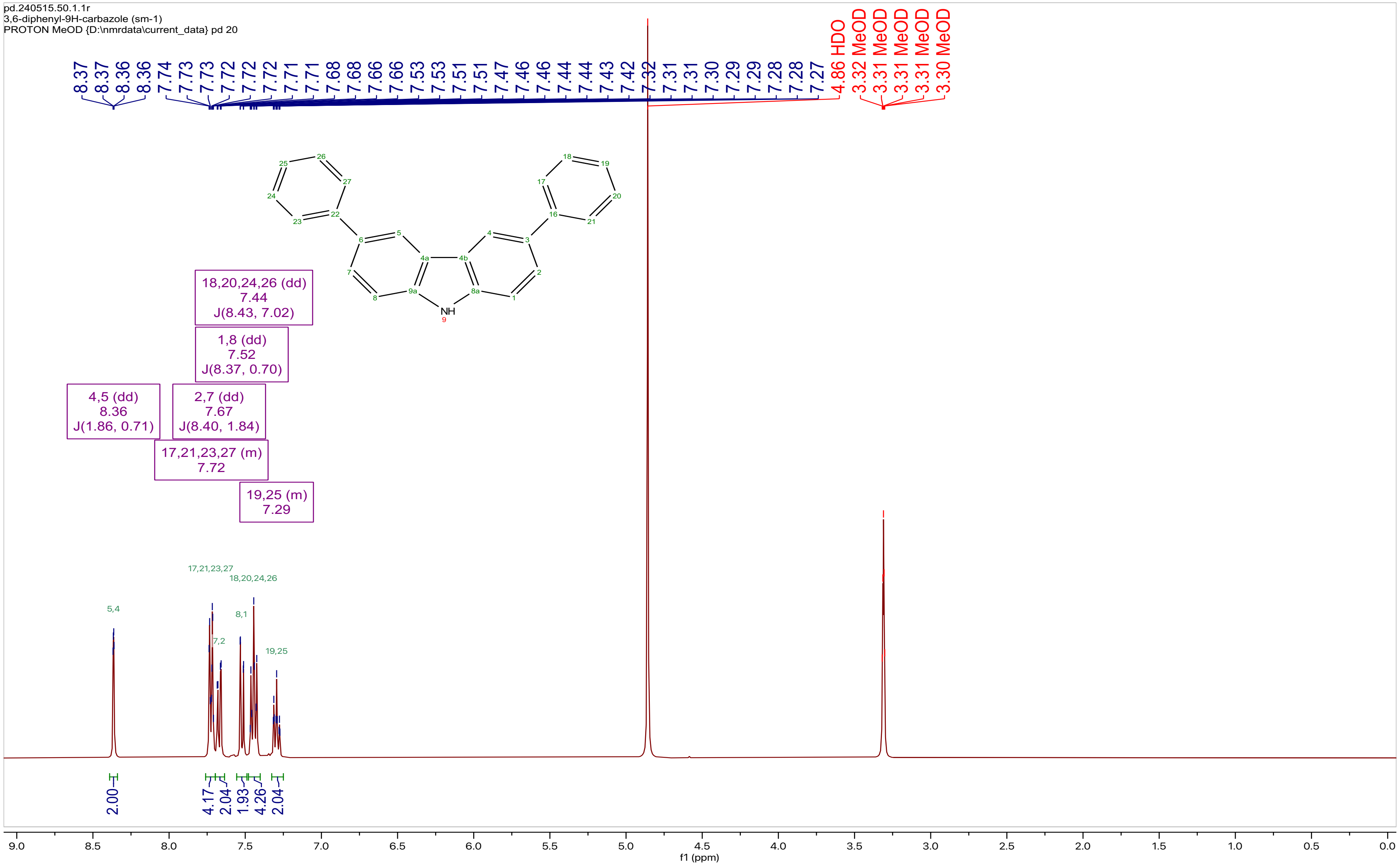

¹H NMR (400 MHz, MeOD) of 3g’

pd.240515.51.1.1r
3,6-diphenyl-9H-carbazole (sm-1)
C13CPD MeOD {D:\nmrdata\current_data} pd 20

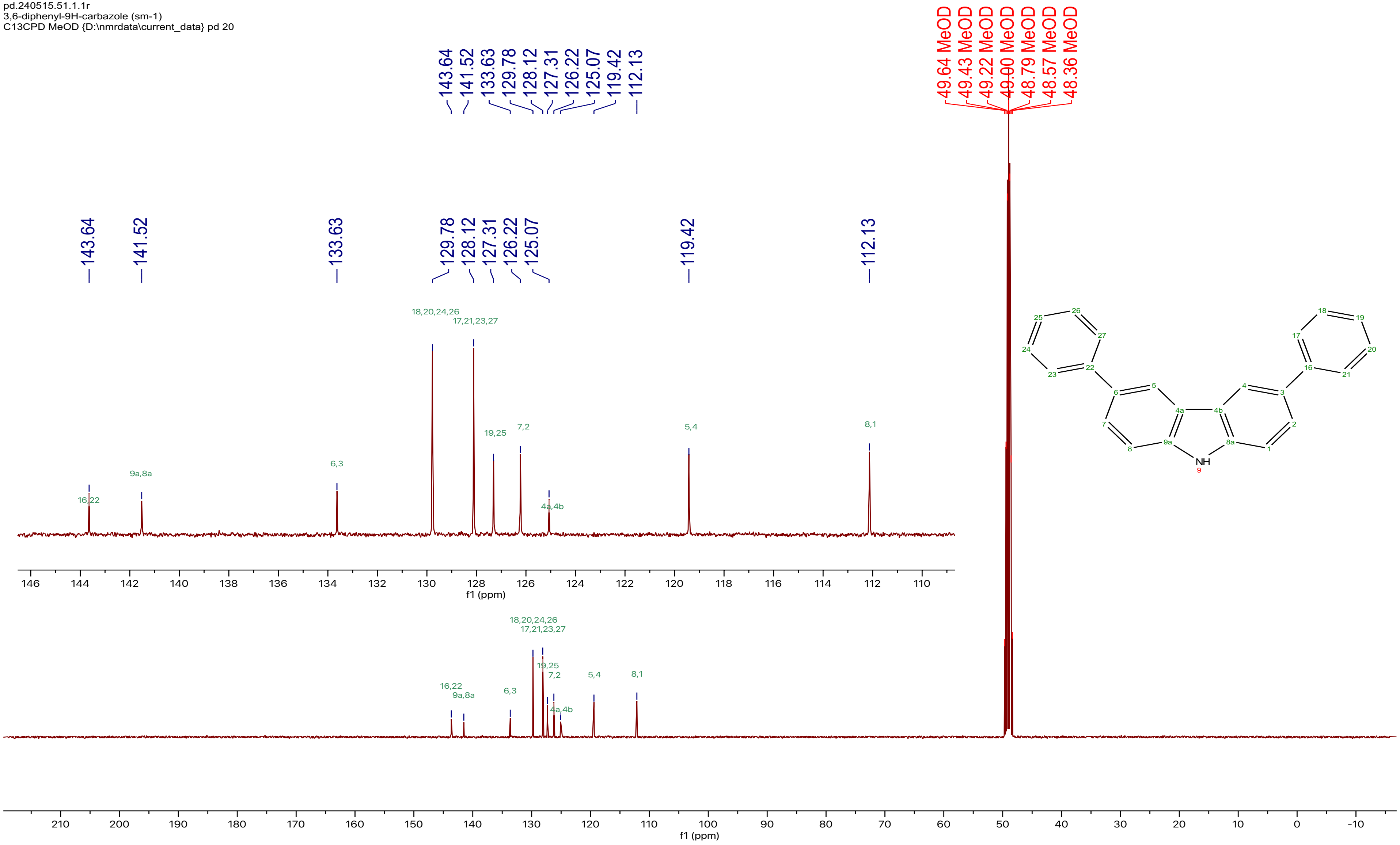

$^{13}C\{^1H\}$ NMR (101 MHz, MeOD) of 3g’

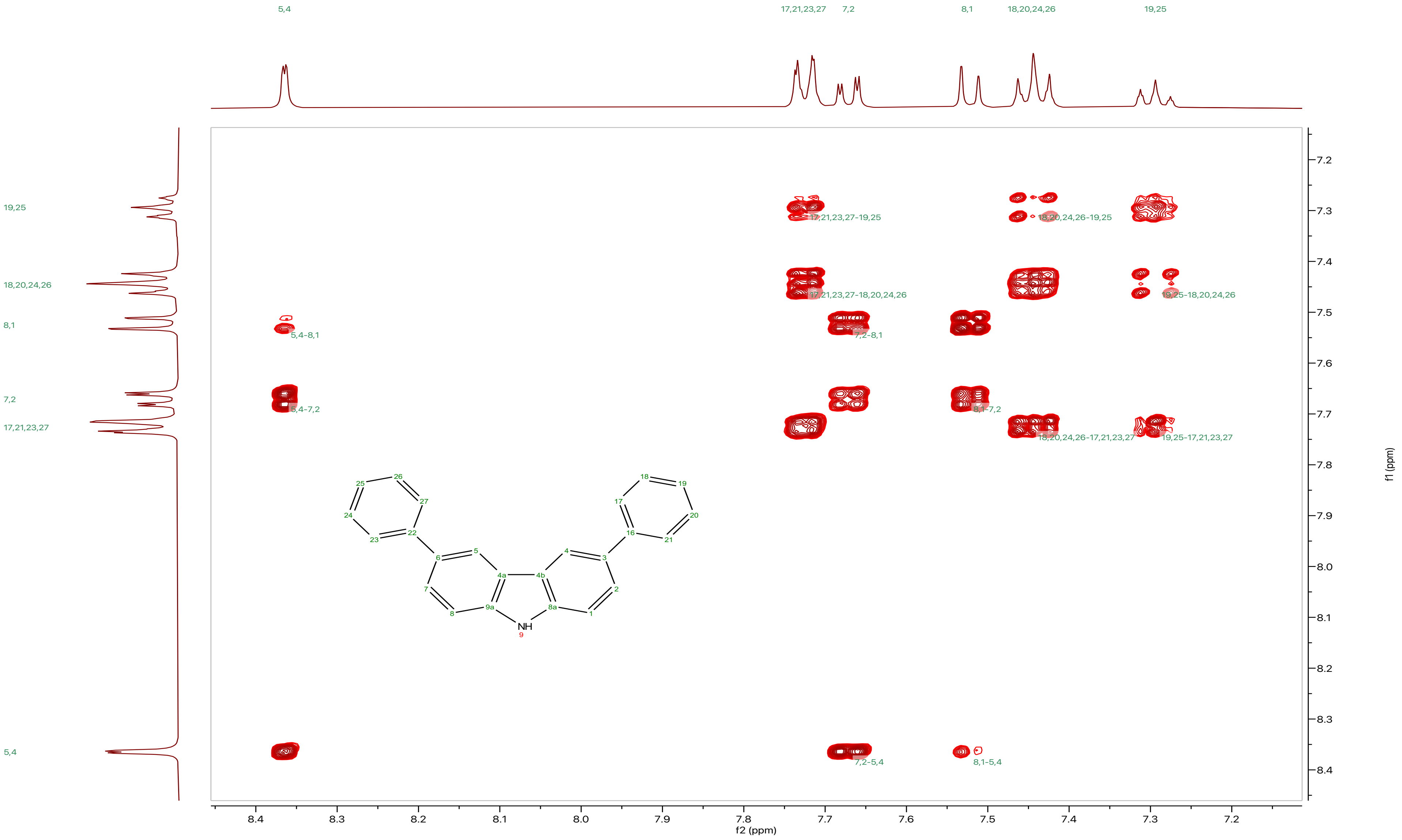

^{1}H-^{1}H COSY (400 MHz, MeOD) of 3g'

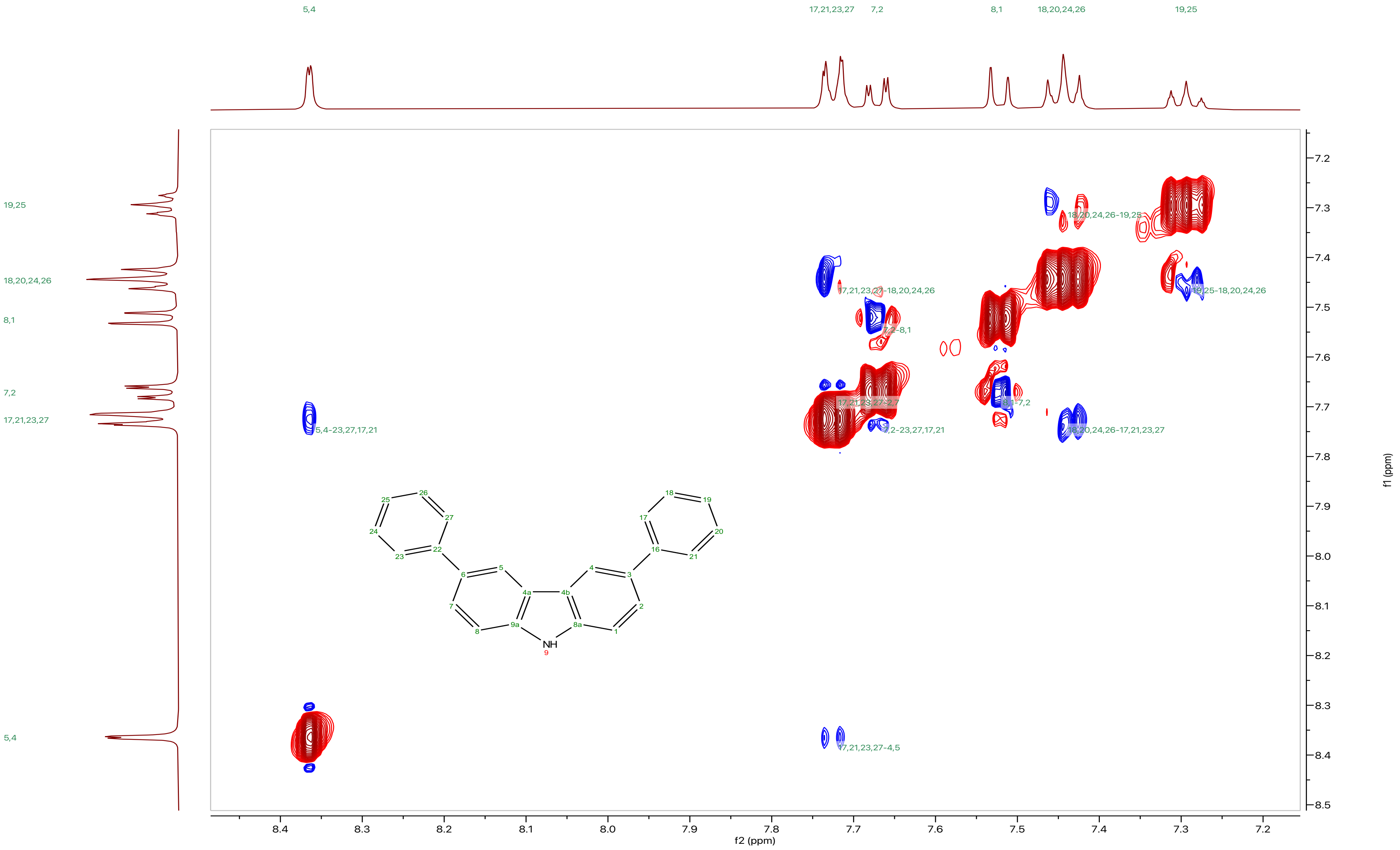

^{1}H-^{1}H NOESY (400 MHz, MeOD) of 3g'

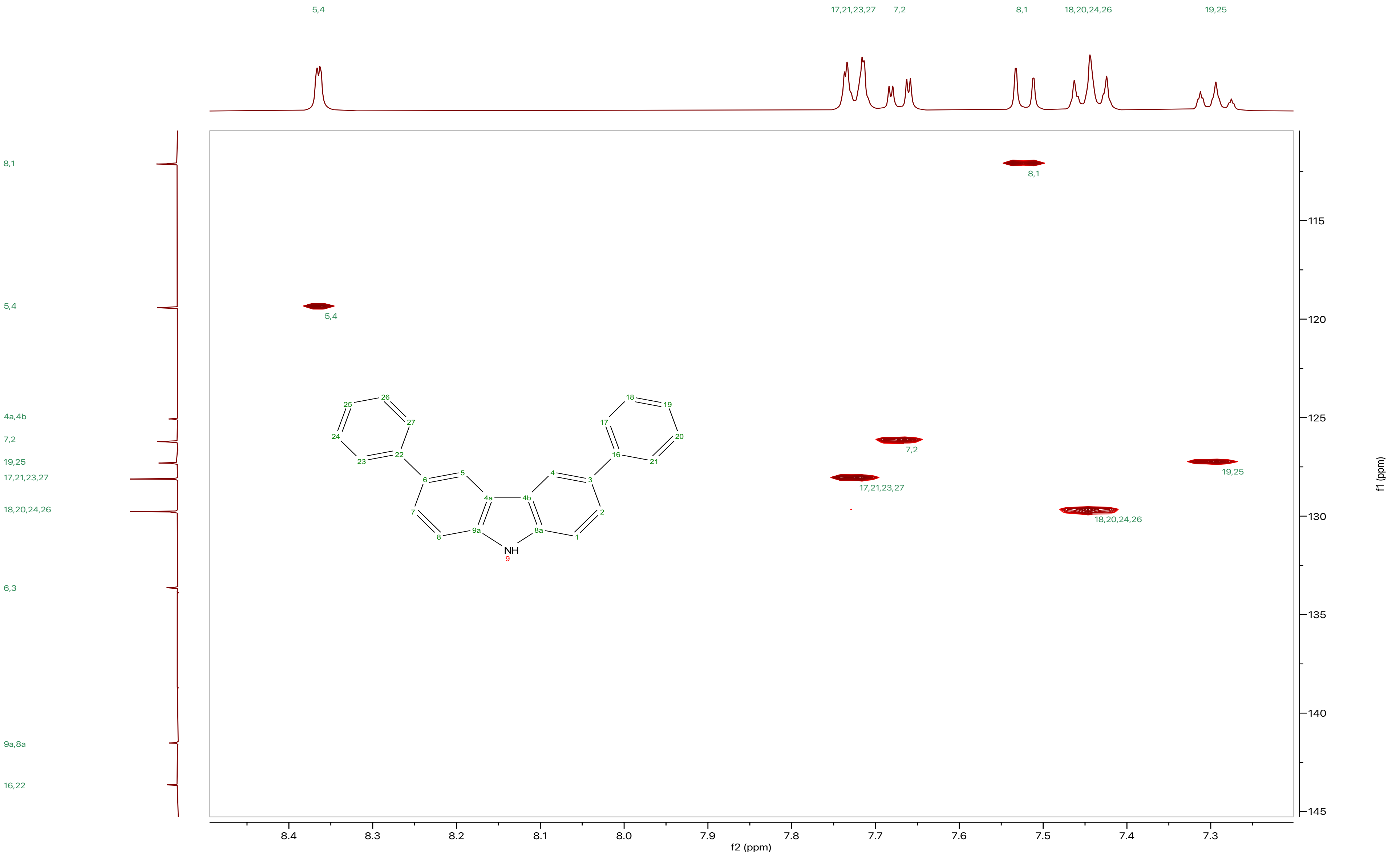

^{1}H-^{13}C{^{1}H} HSQC NMR (400/101 MHz, MeOD) of 3g'

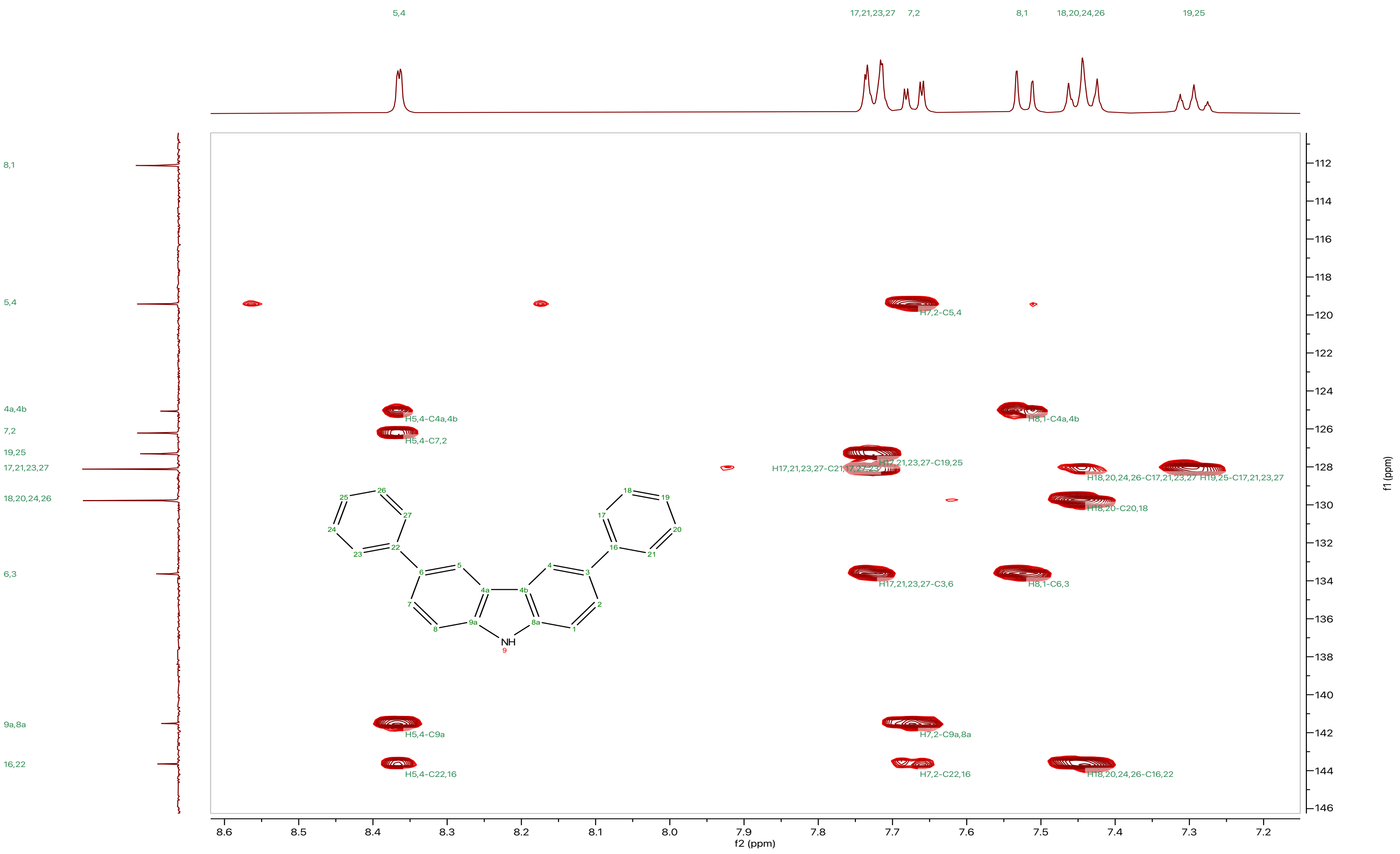

^1H-$^{13}C\{^1H\}$ HMBC NMR (400/101 MHz, MeOD) of 3g'

4bg

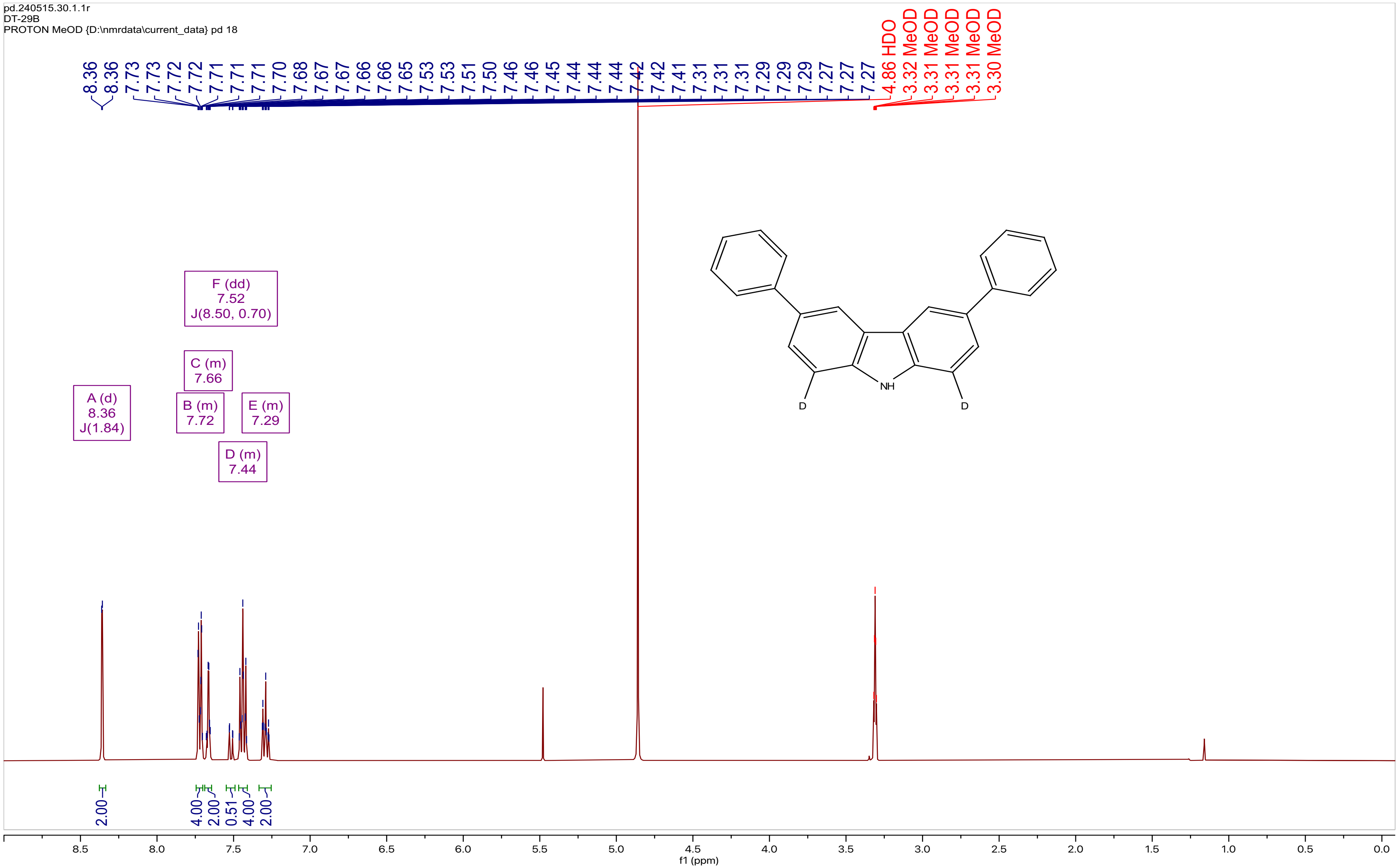

^{1}H NMR (400 MHz, MeOD) of 4bg

pd.240515.31.1.1r
DT-29B
C13CPD MeOD {D:\nmrdata\current_data} pd 18

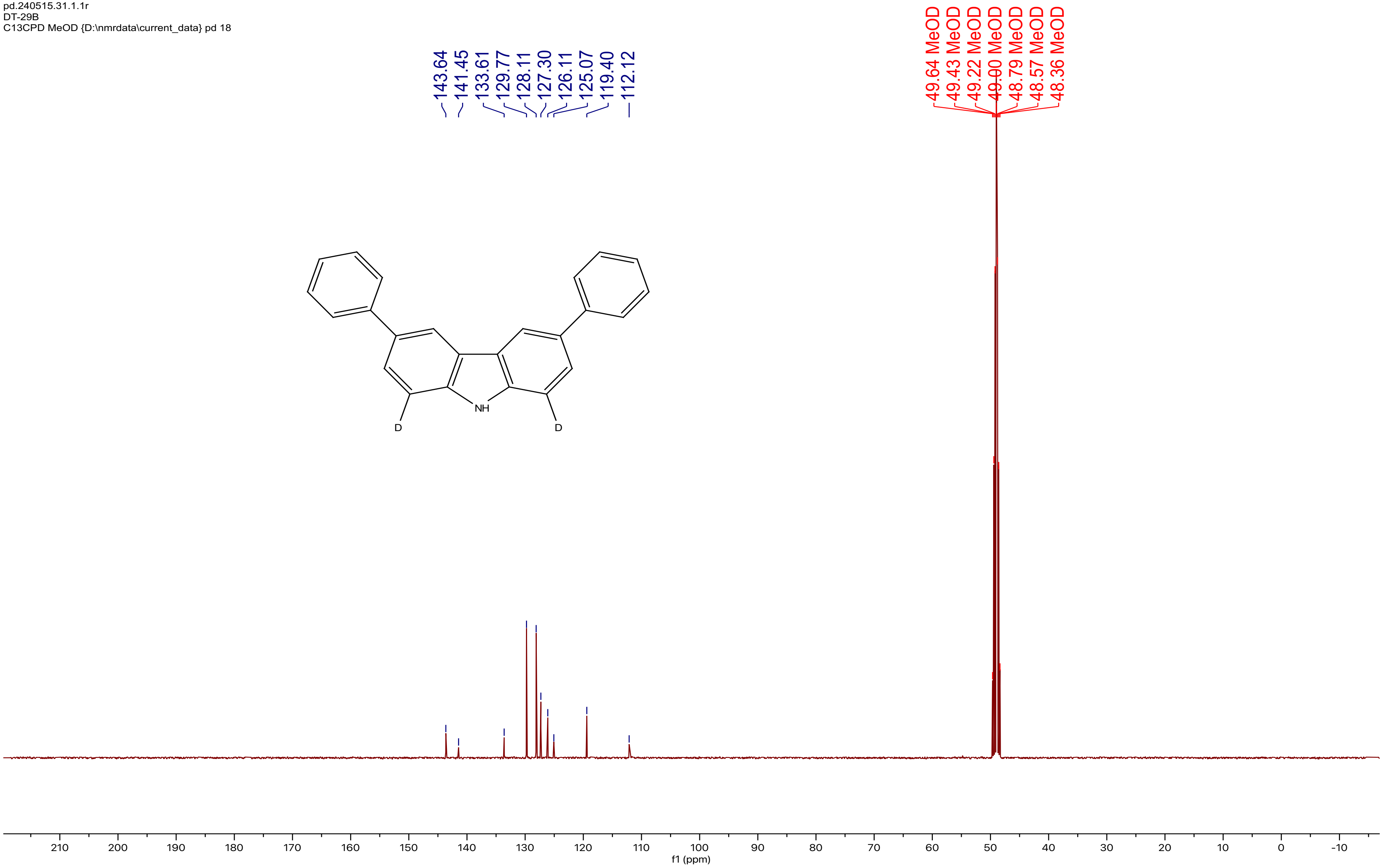

$^{13}C\{^1H\}$ NMR (101 MHz, MeOD) of 4bg

3h

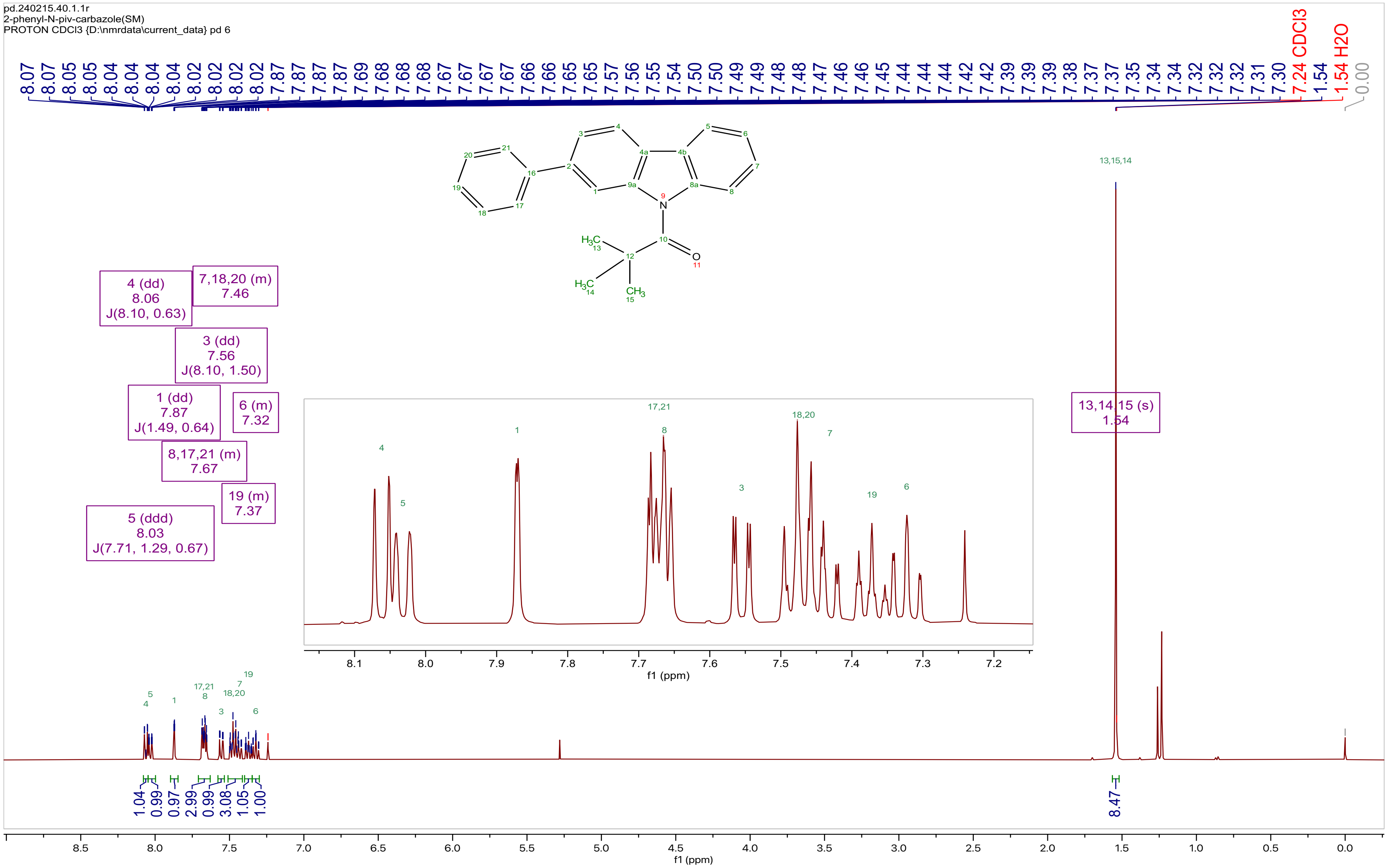

¹H NMR (400 MHz, $CDCl_3$) of 3h

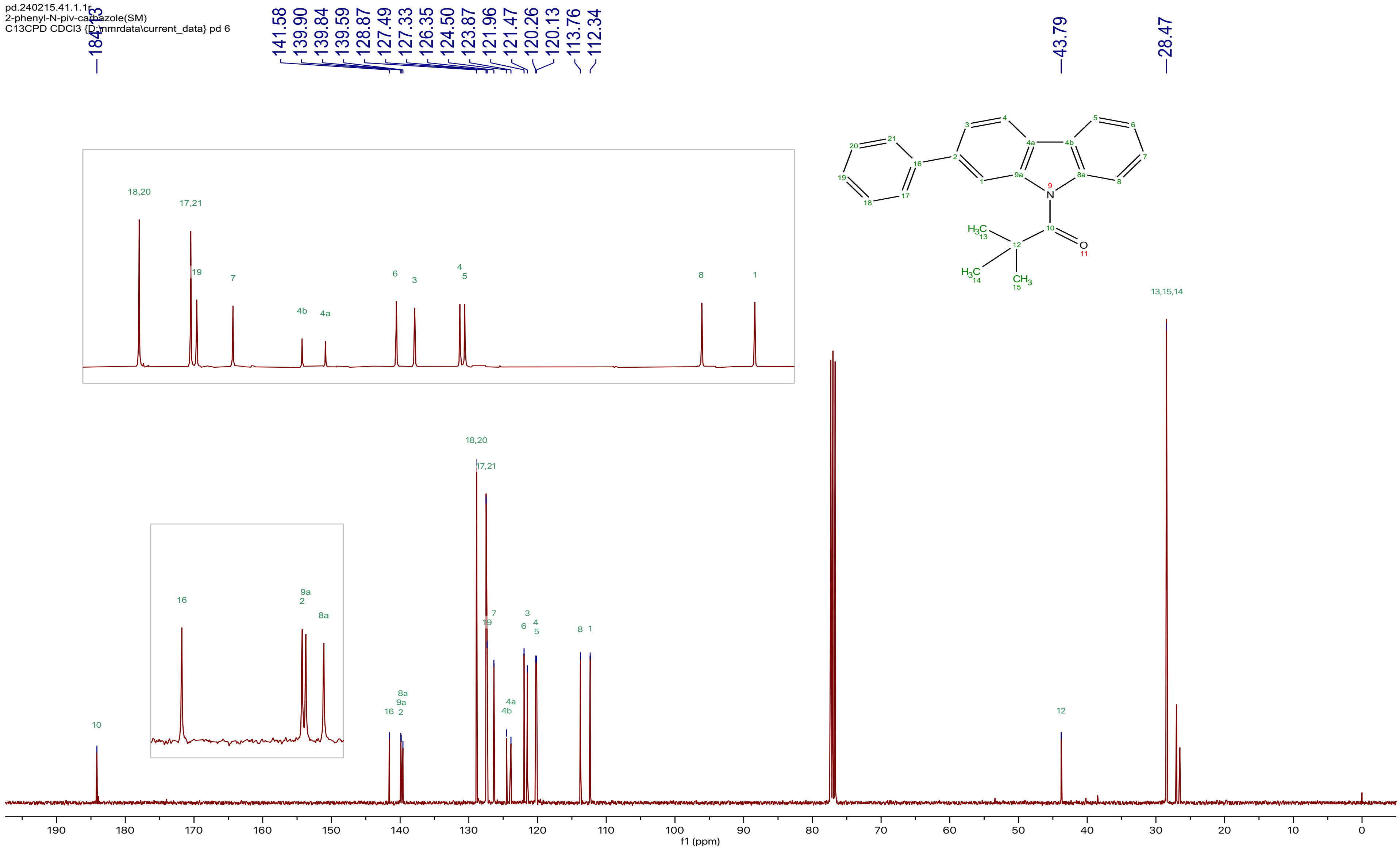

$^{13}C\{^1H\}$ NMR (101 MHz, $CDCl_3$) of 3h

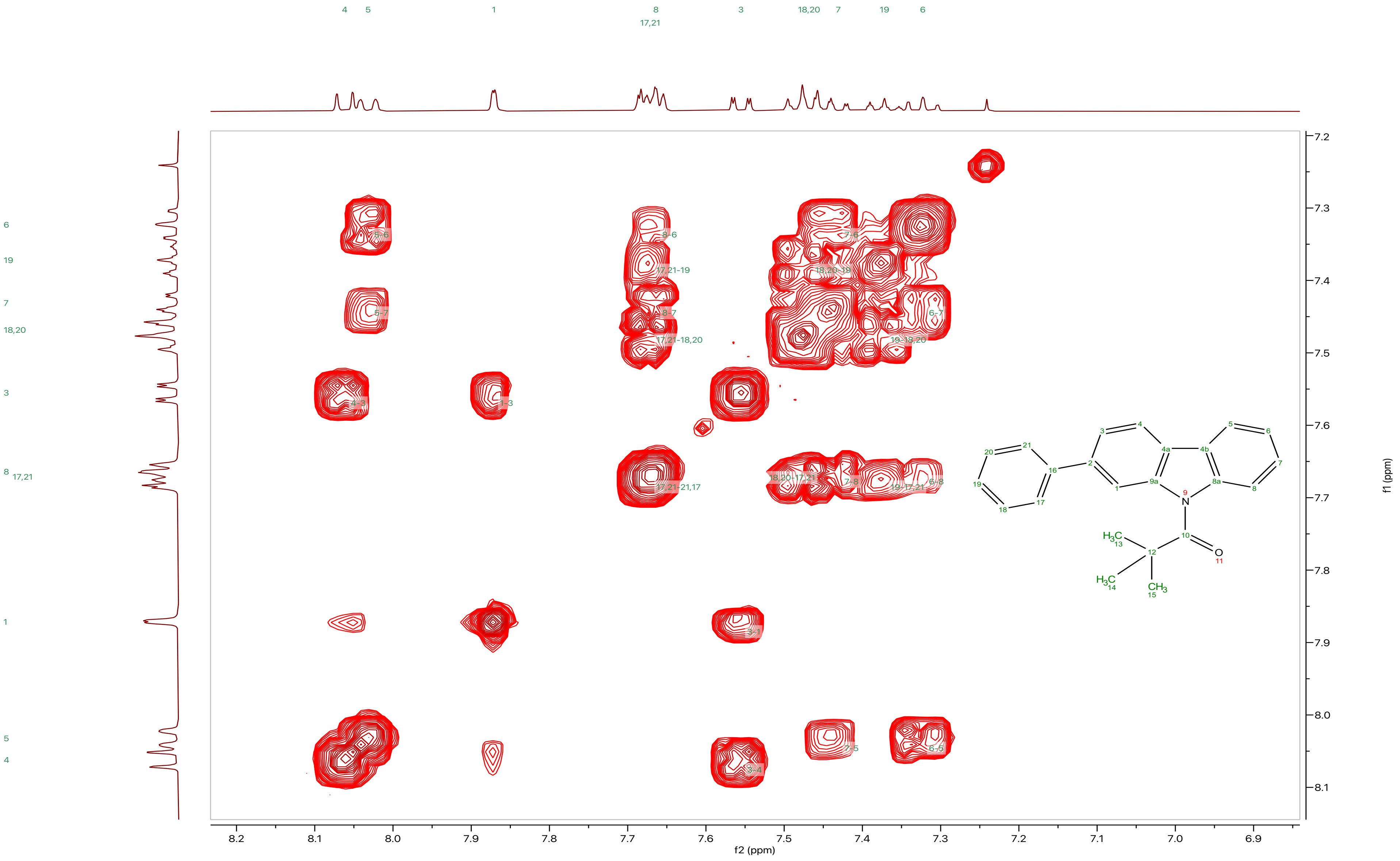

^1H-^1H COSY (400 MHz, $CDCl_3$) of 3h

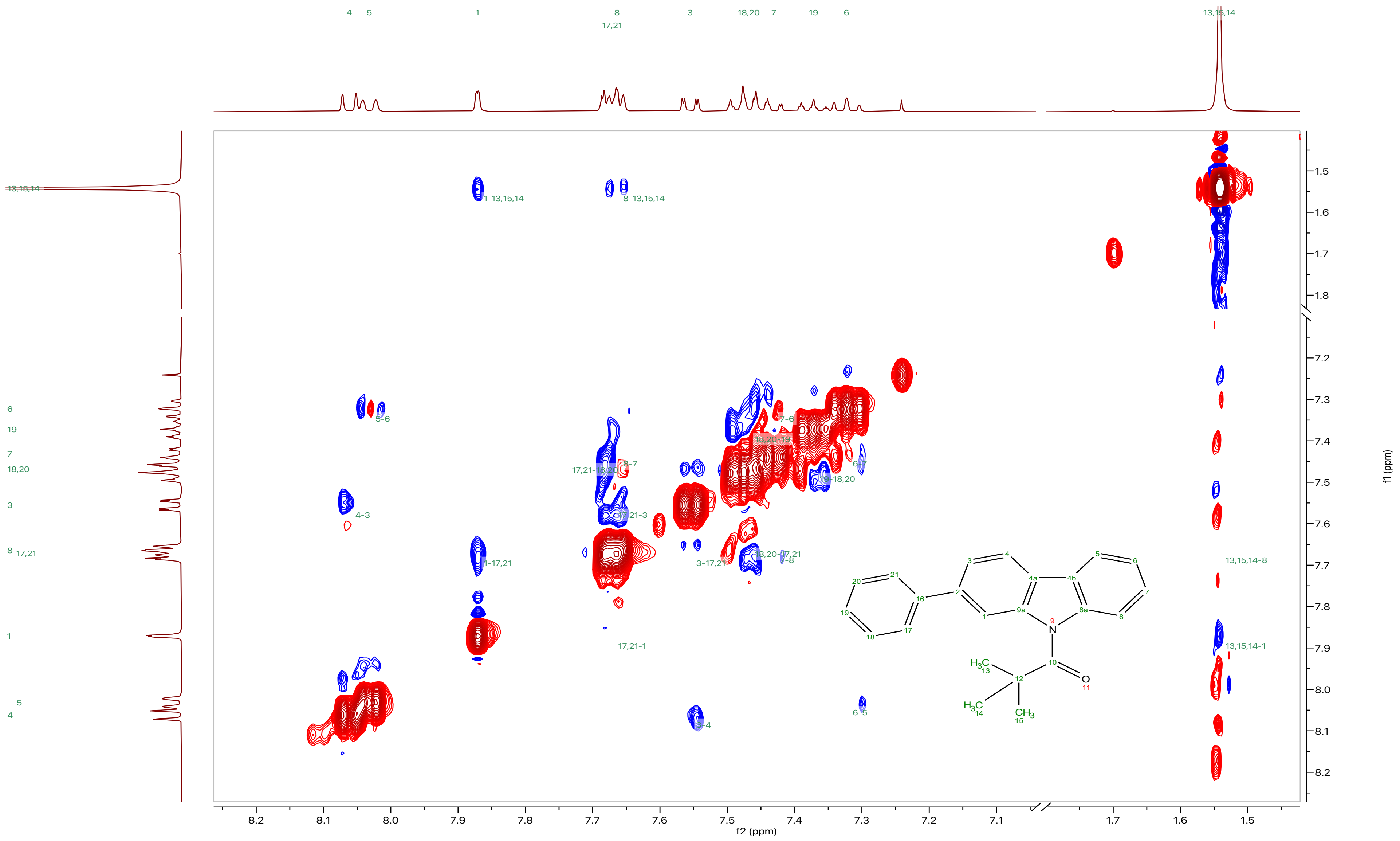

^1H-^1H NOESY (400 MHz, $CDCl_3$) of 3h

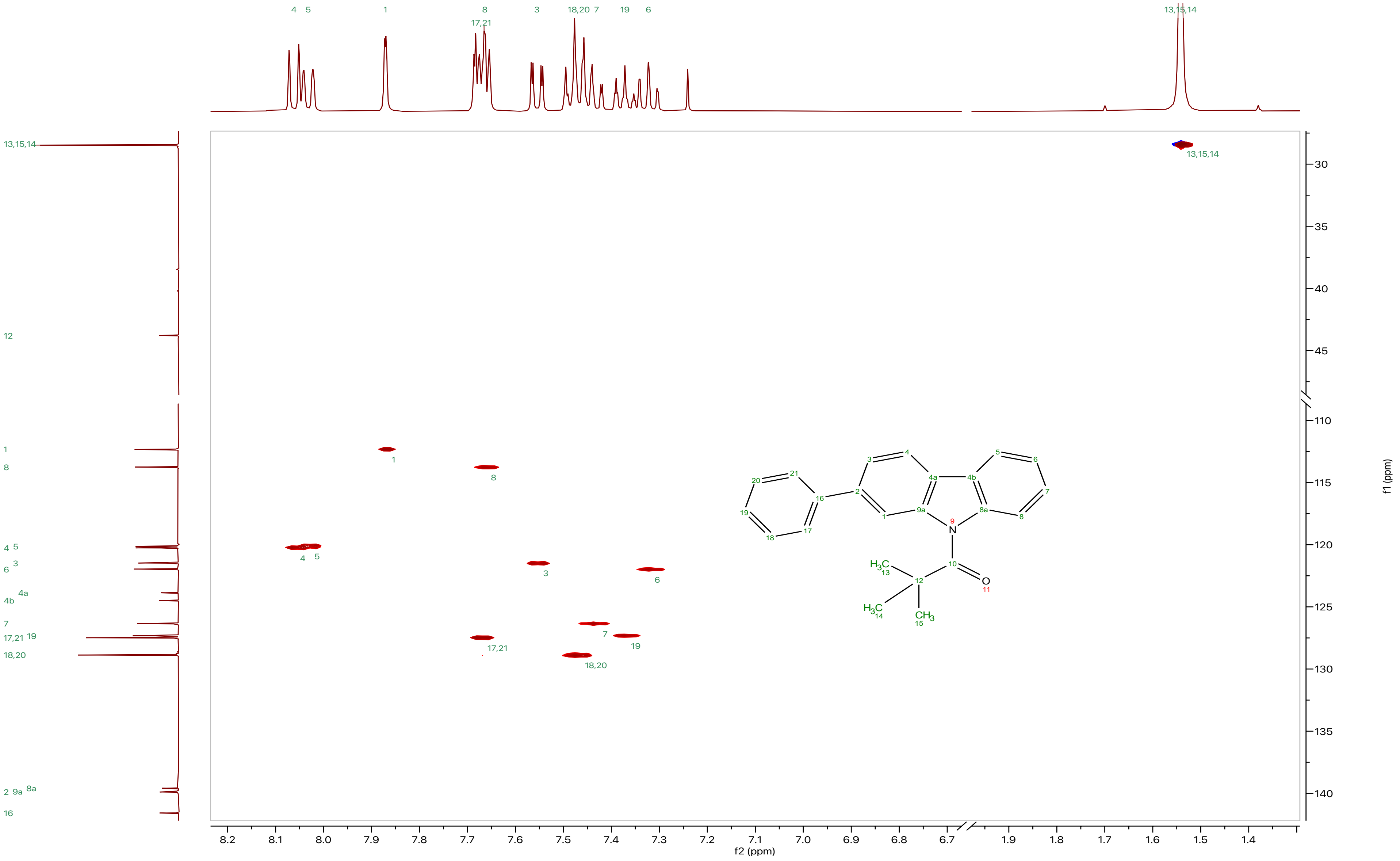

^{1}H-$^{13}C\{^{1}H\}$ HSQC NMR (400/101 MHz, $CDCl_3$) of 3h

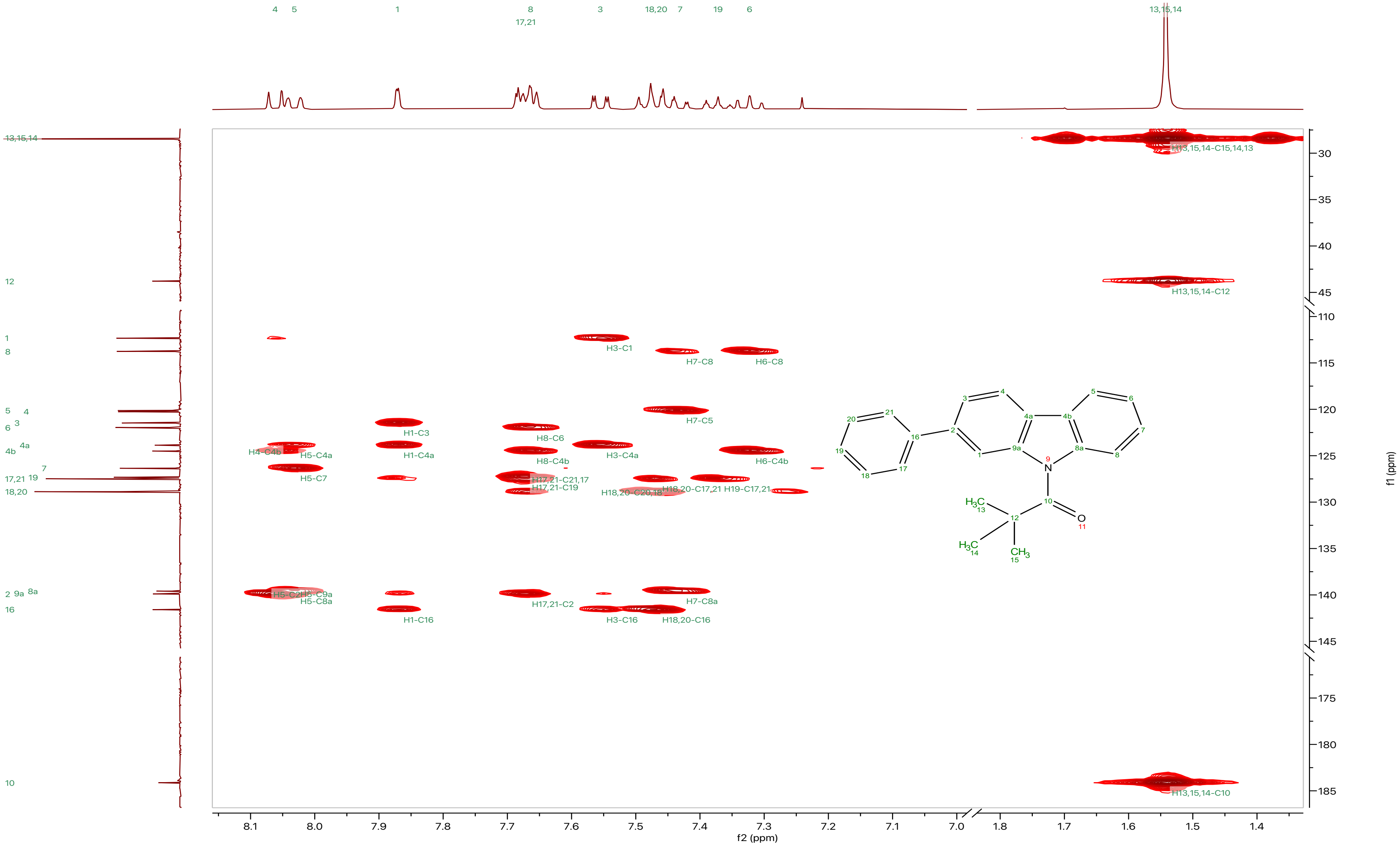

^{1}H-$^{13}C\{^{1}H\}$ HMBC NMR (400/101 MHz, $CDCl_3$) of 3h

4ah

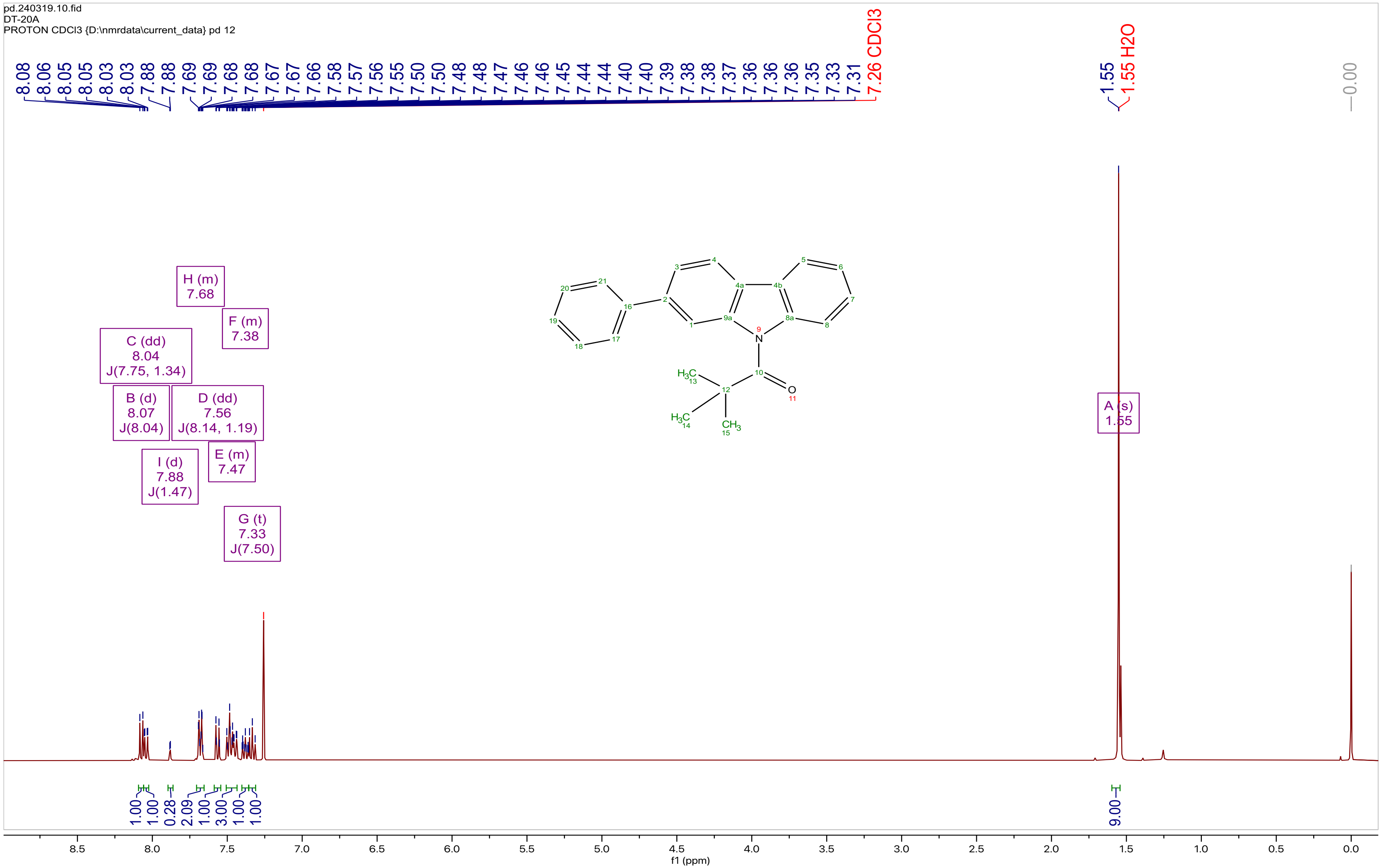

^{1}H NMR (400 MHz, $CDCl_3$) of 4ah

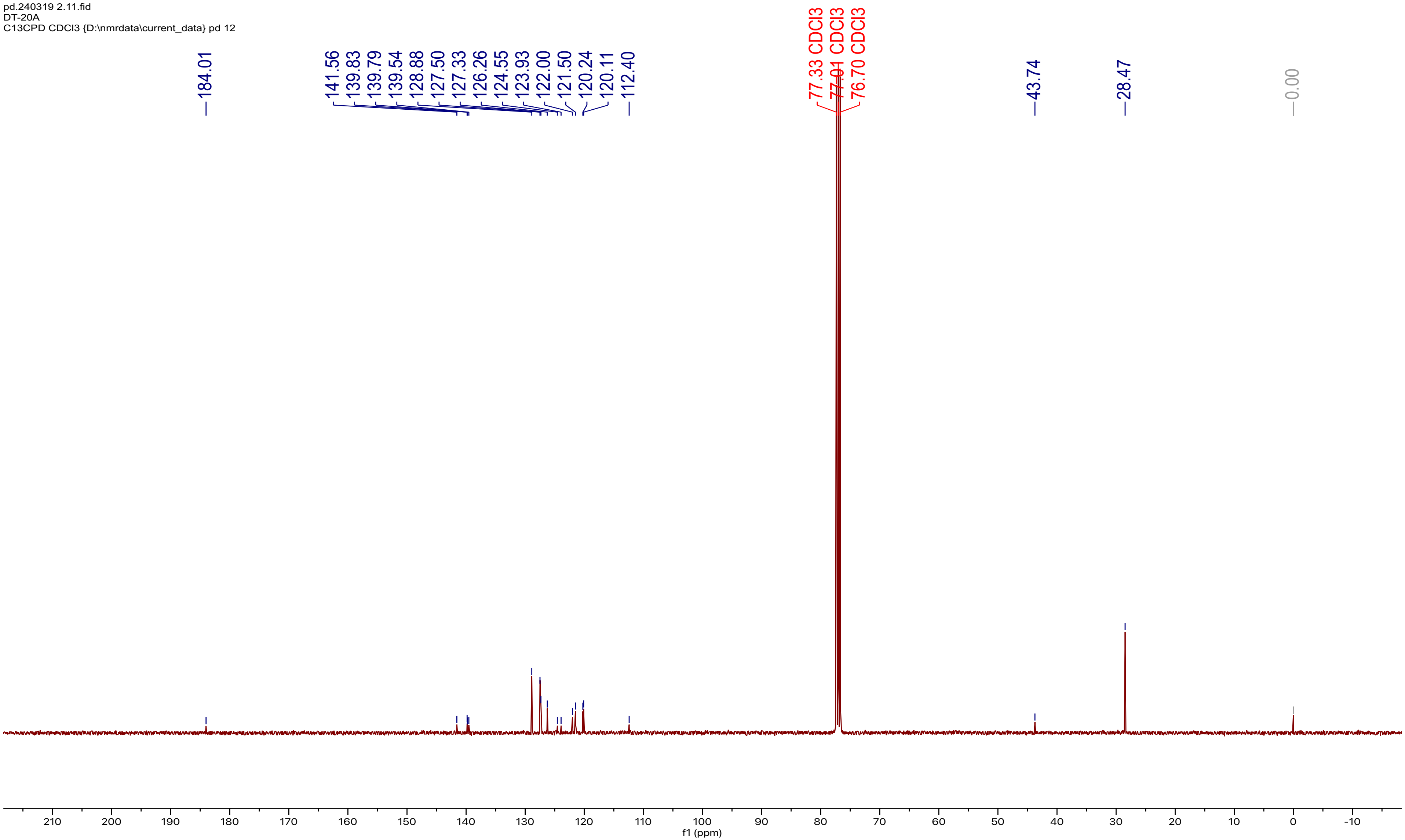

$^{13}C\{^{1}H\}$ NMR (101 MHz, $CDCl_3$) of 4ah

3h’

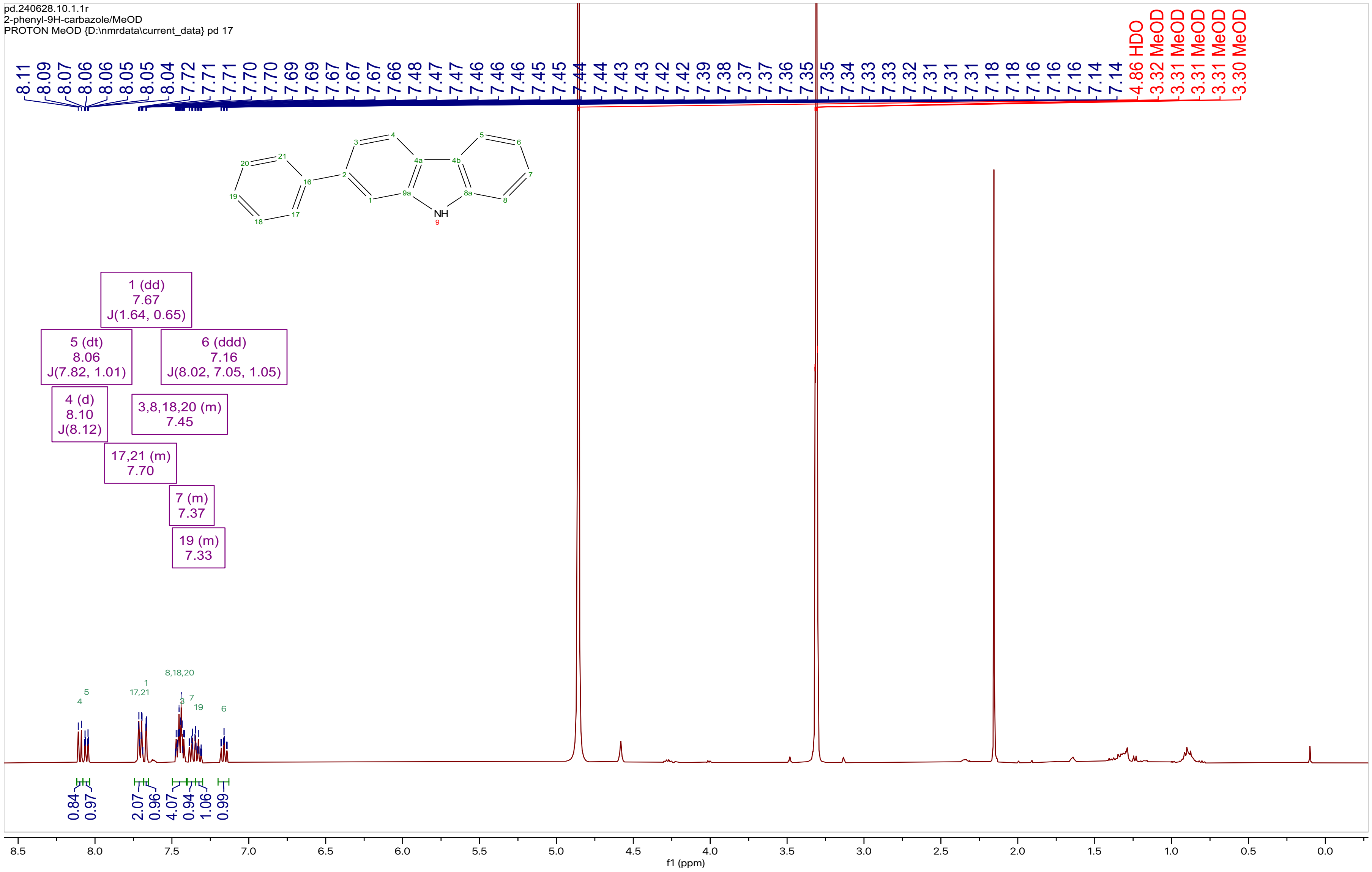

¹H NMR (400 MHz, MeOD) of 3h’

pd.240628.15.1.1r
2-phenyl-9-H-carbazole/MeOD
C13CPD MeOD {D:\nmrdata\current_data} pd 17

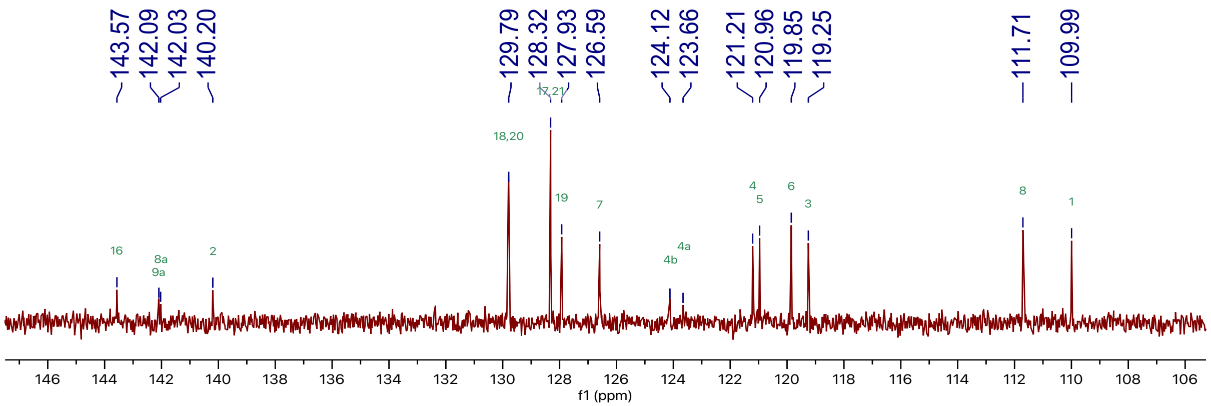

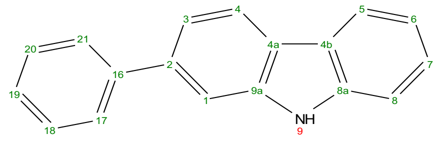

^{13}C NMR (101 MHz, MeOD) δ 143.6, 142.1, 142.0, 140.2, 129.8, 128.3, 127.9, 126.6, 124.1, 123.7, 121.2, 121.0, 119.9, 119.3, 111.7, 110.0.

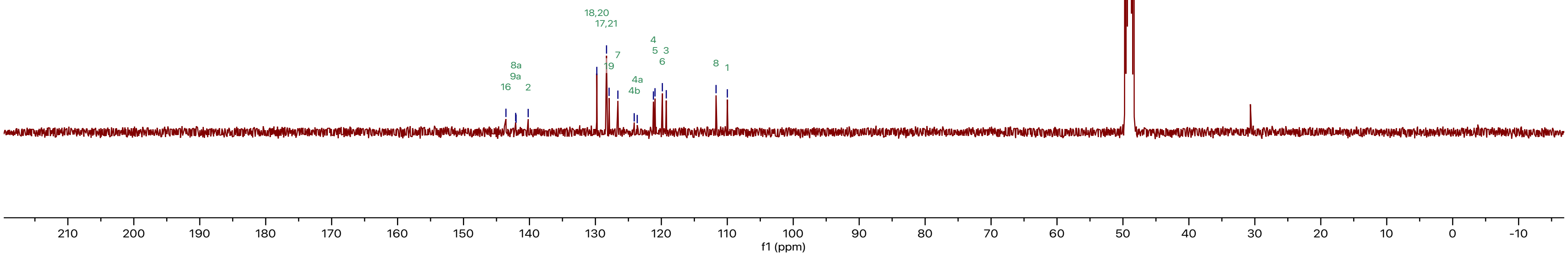

^{13}C{^{1}H} NMR (101 MHz, MeOD) of 3h'

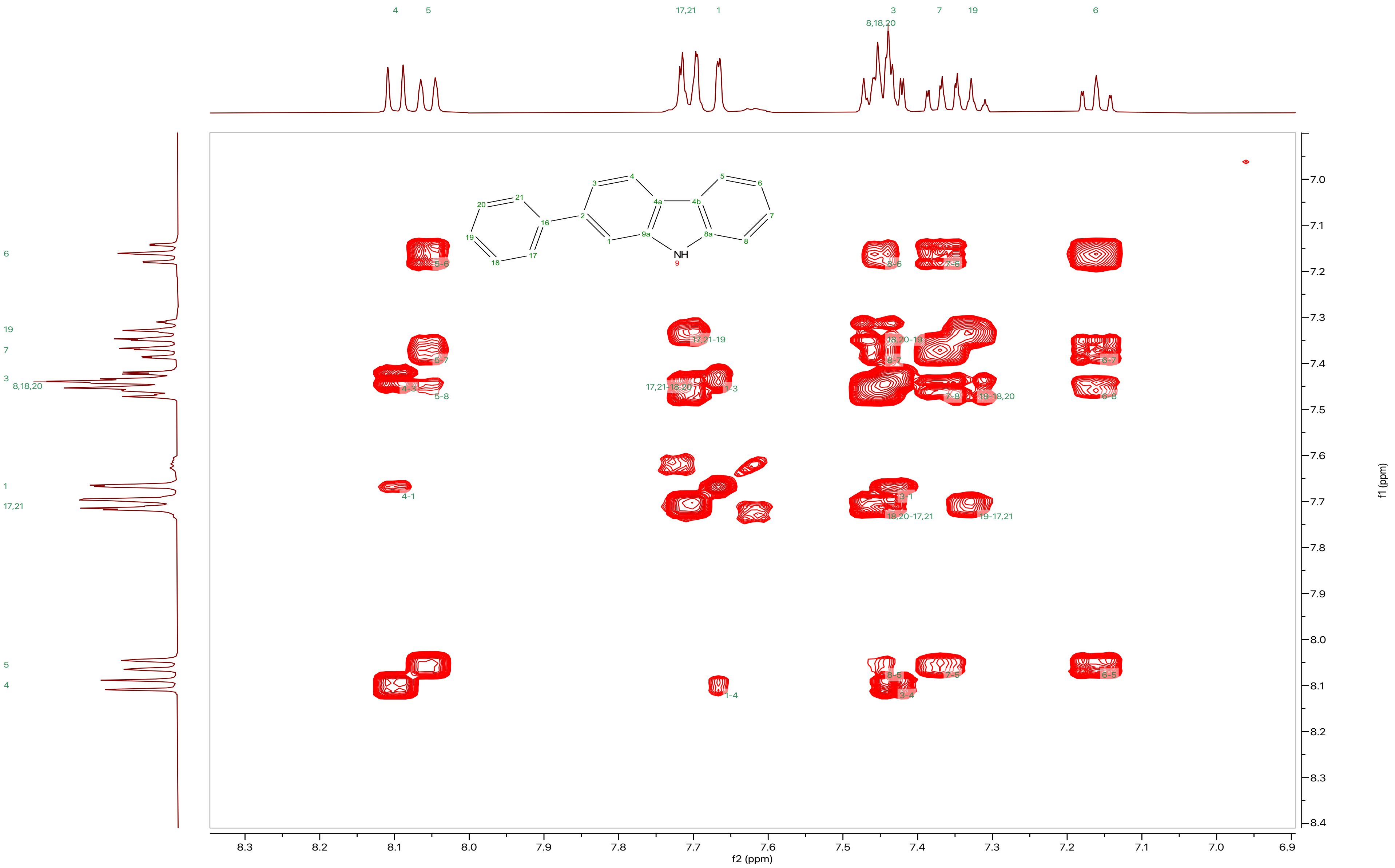

^{1}H-^{1}H COSY (400 MHz, MeOD) of 3h'

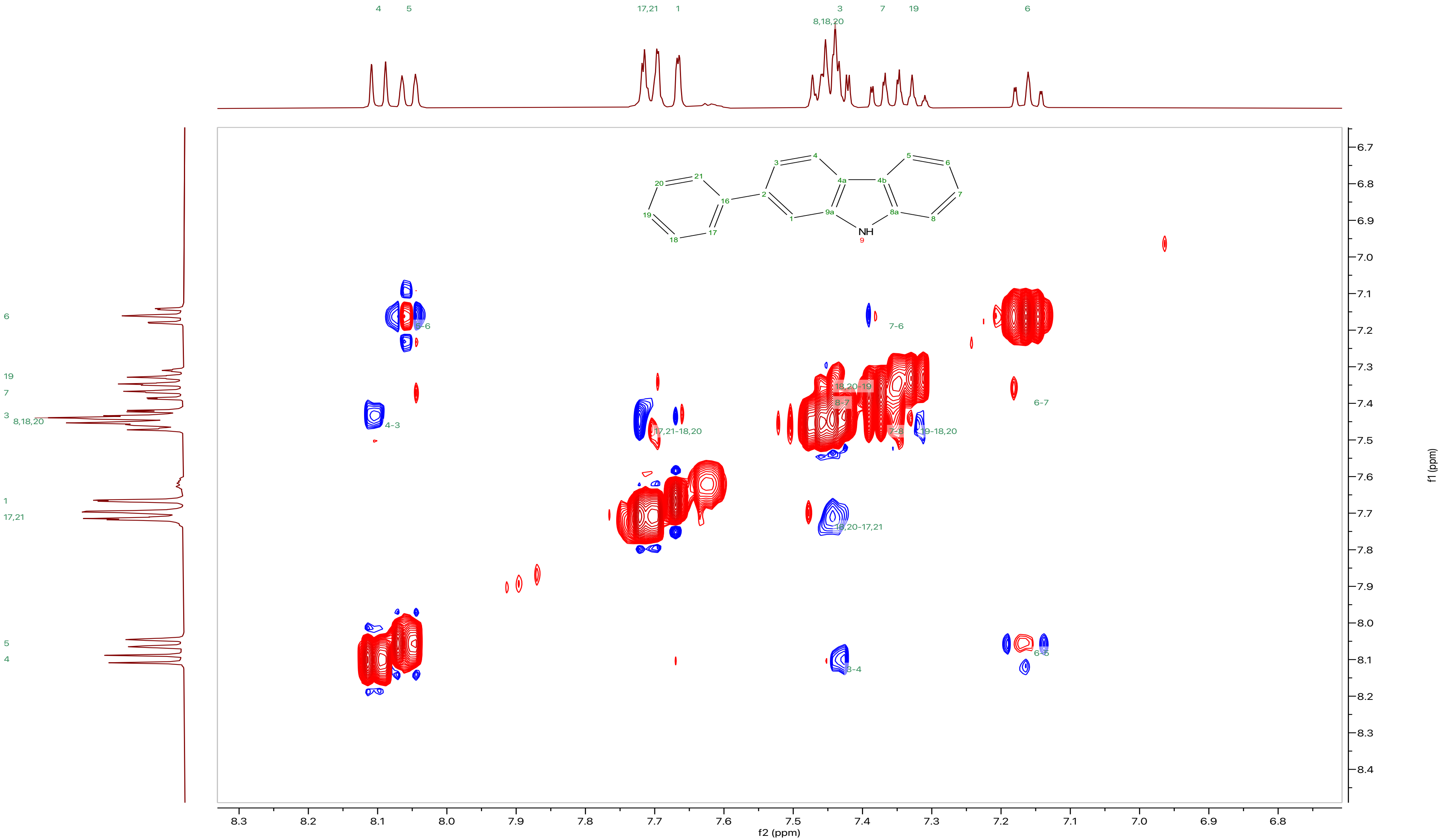

^{1}H-^{1}H NOESY (400 MHz, MeOD) of 3h'

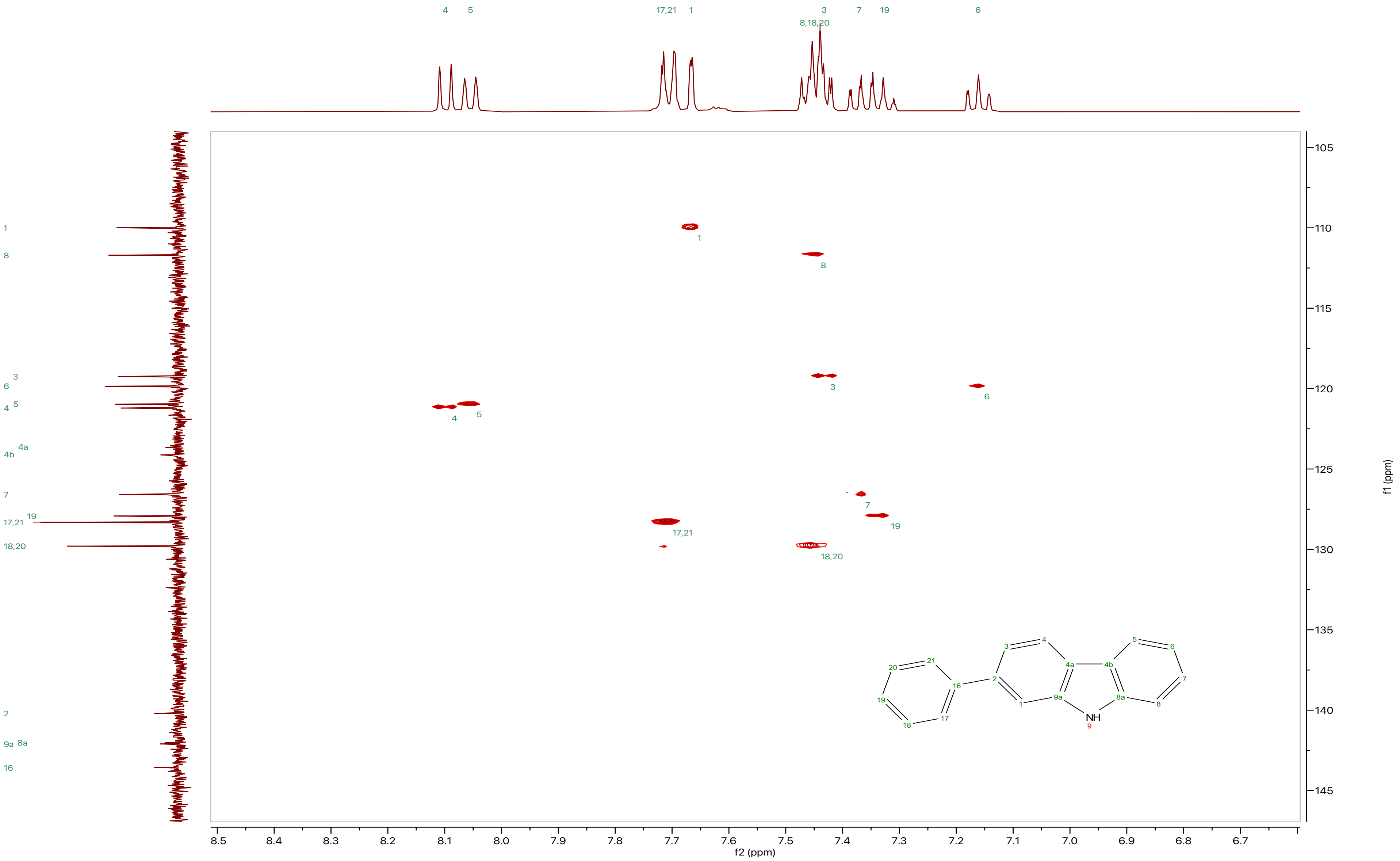

^{1}H-$^{13}C\{^{1}H\}$ HSQC NMR (400/101 MHz, MeOD) of 3h’

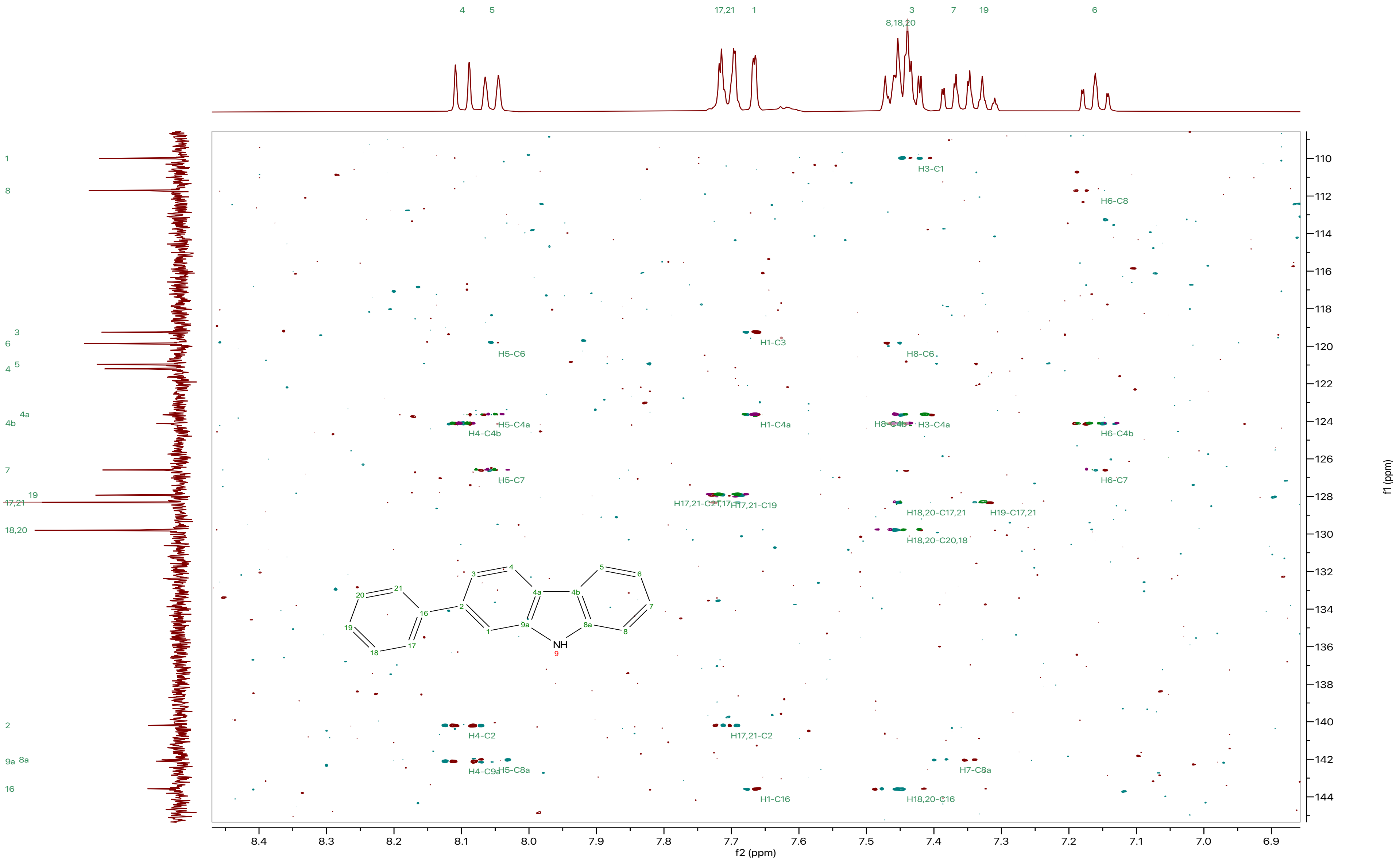

^{1}H-^{13}C{^{1}H} HMBC NMR (400/101 MHz, MeOD) of 3h'

4bh

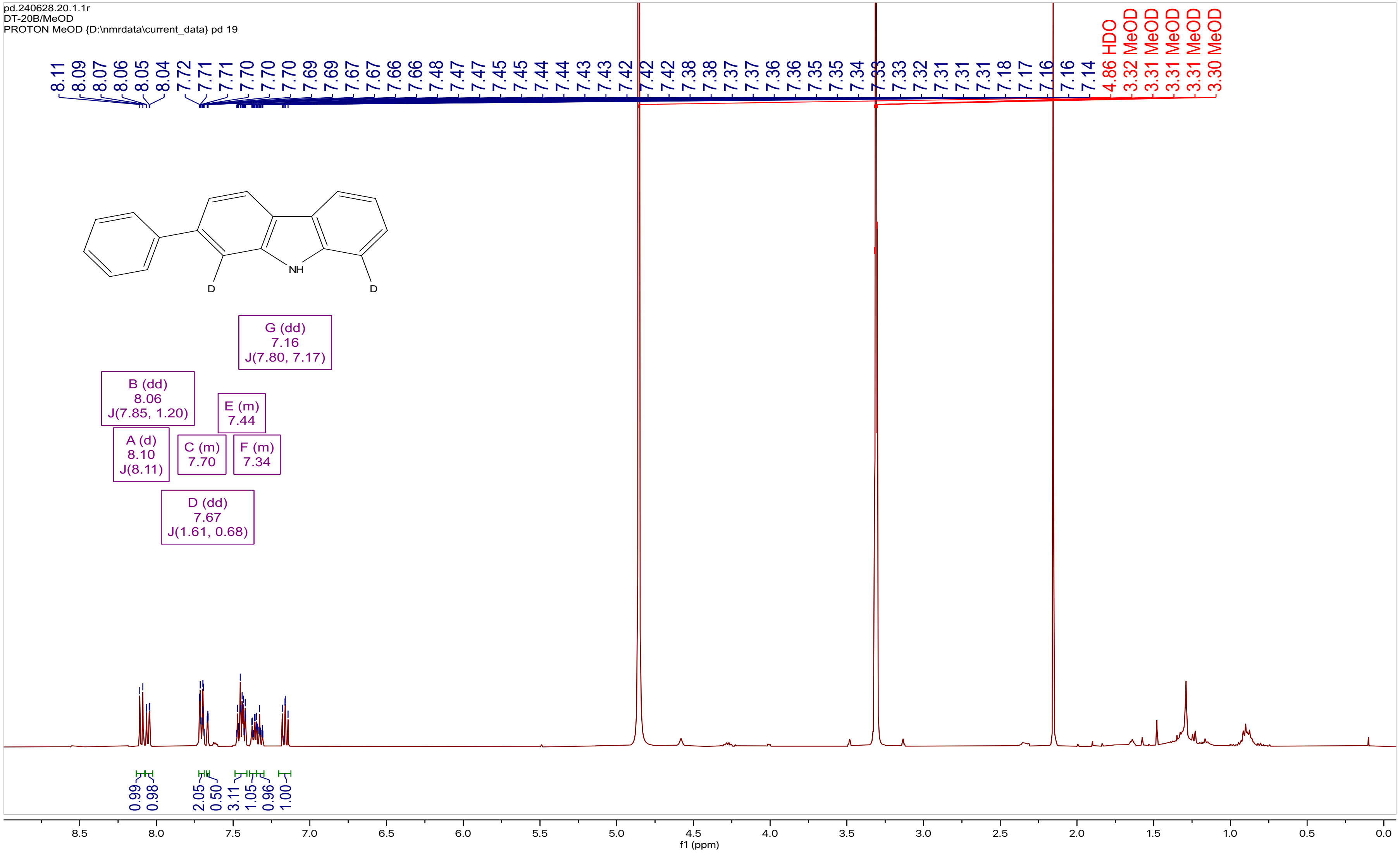

^{1}H NMR (400 MHz, MeOD) of 4bh

pd.240628.22.1.1r
DT-20B/MeOD
C13CPD MeOD {D:\nmrdata\current_data} pd 19

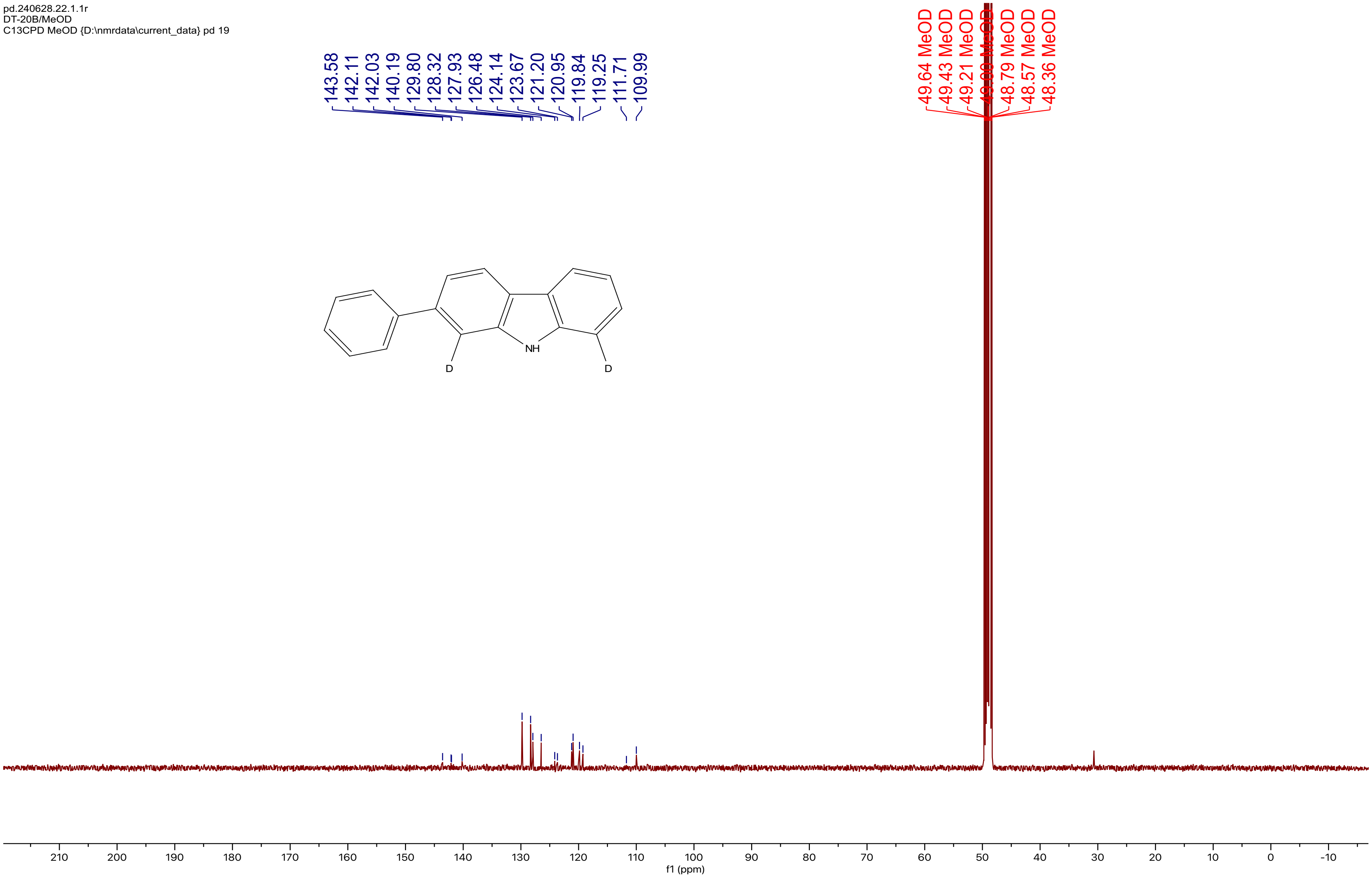

$^{13}C\{^{1}H\}$ NMR (101 MHz, MeOD) of 4bh

3i

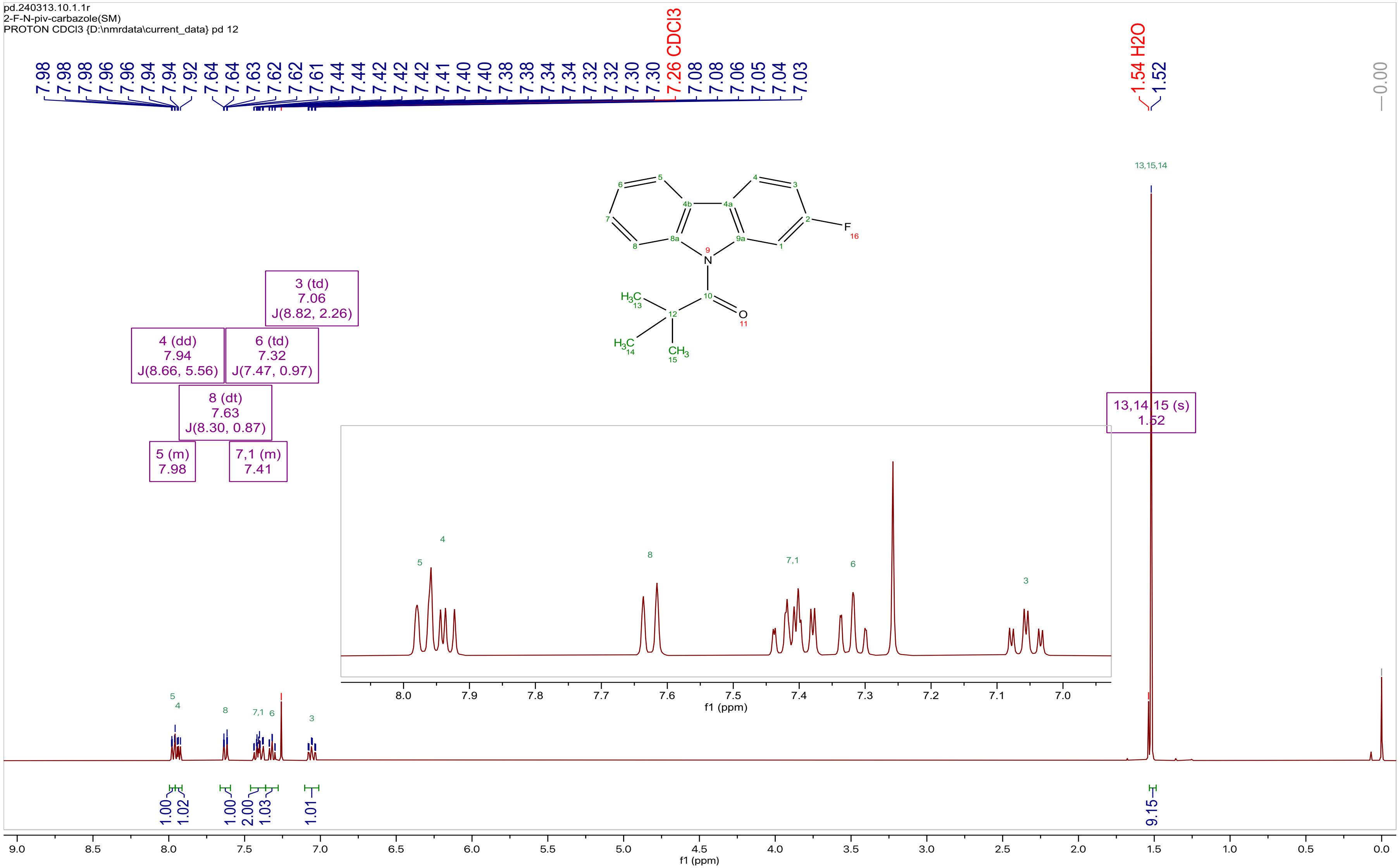

^1H NMR (400 MHz, $CDCl_3$) of 3i

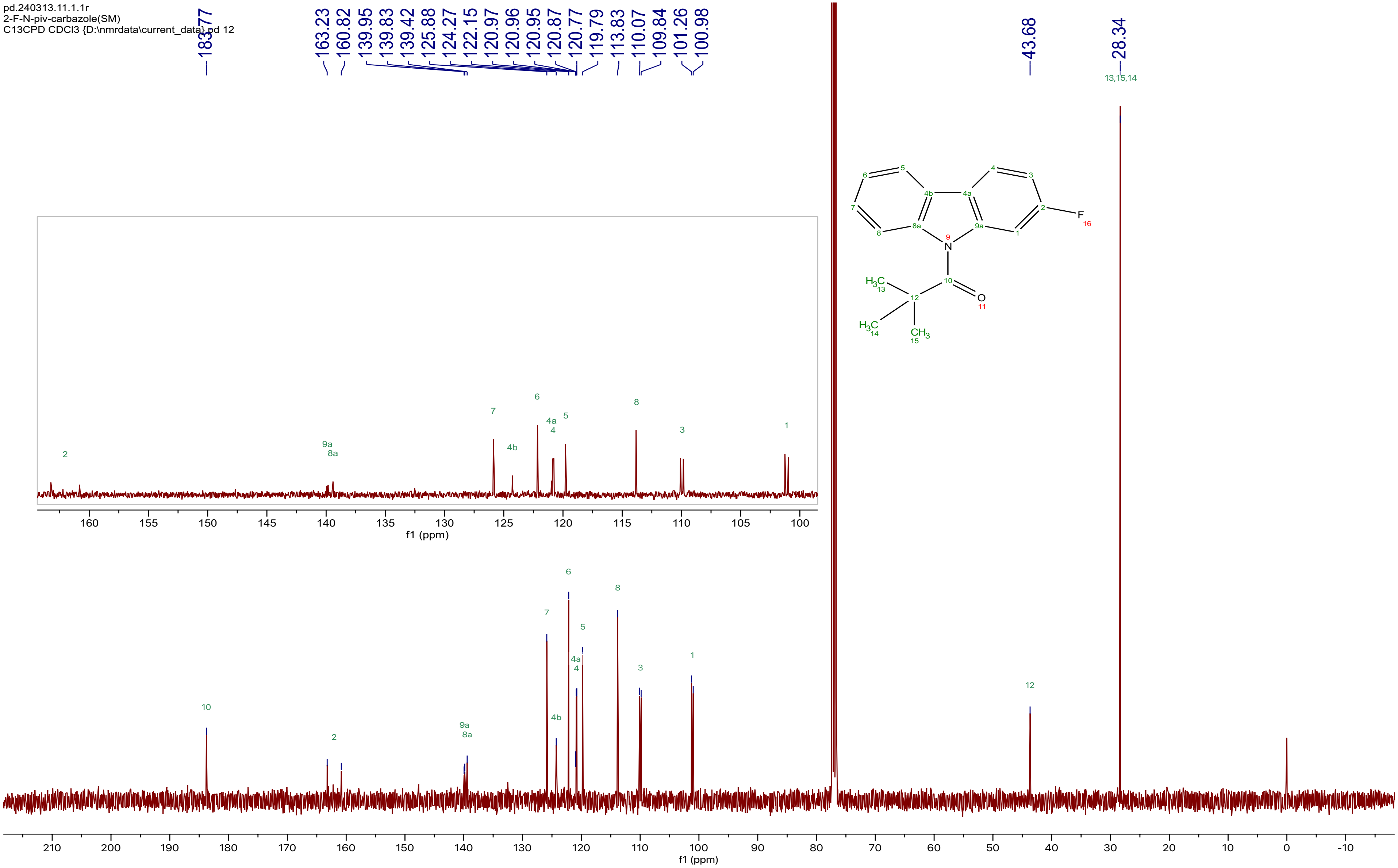

$^{13}C\{^{1}H\}$ NMR (101 MHz, $CDCl_3$) of 3i

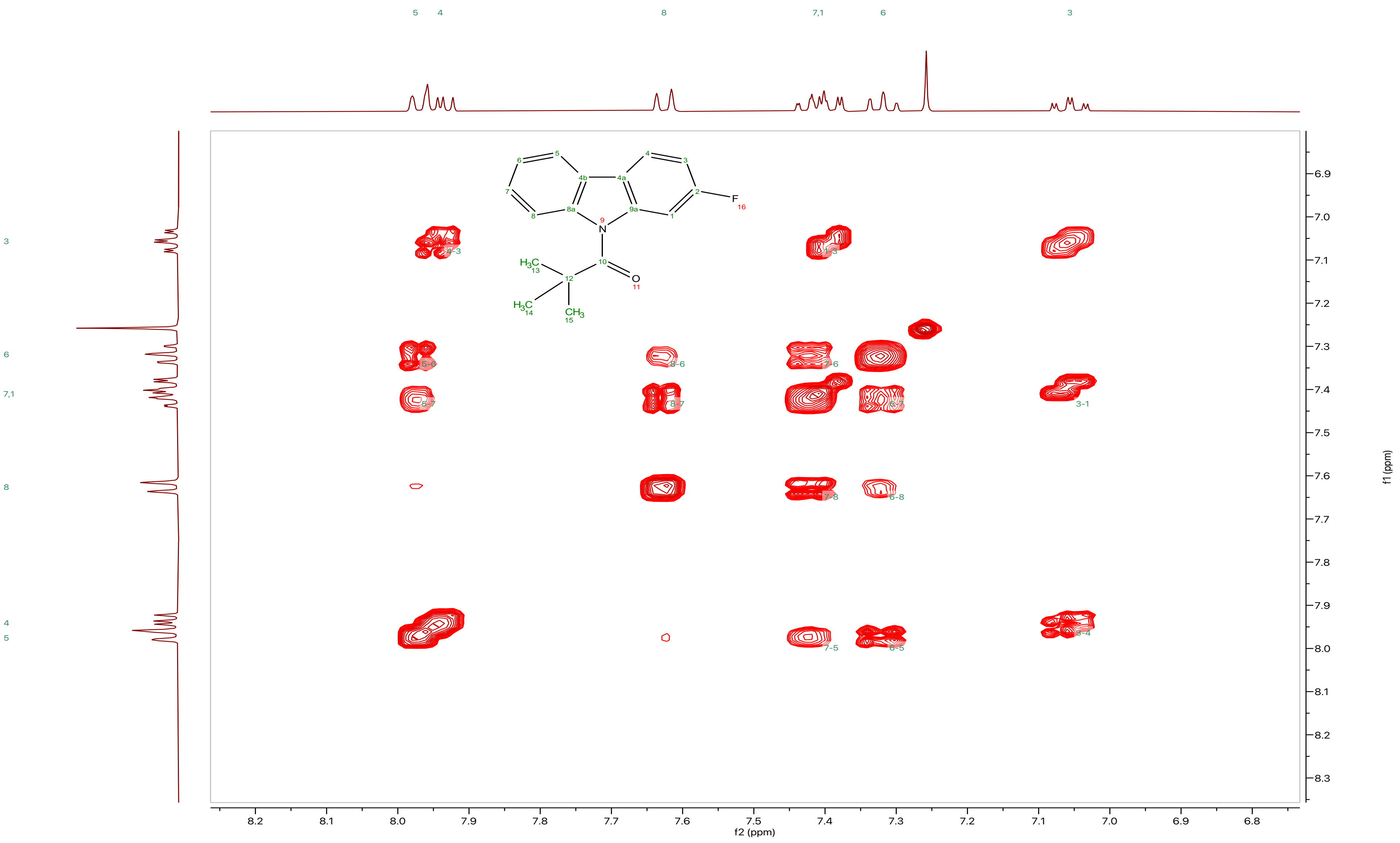

^{1}H-^{1}H COSY (400 MHz, $CDCl_3$) of 3i

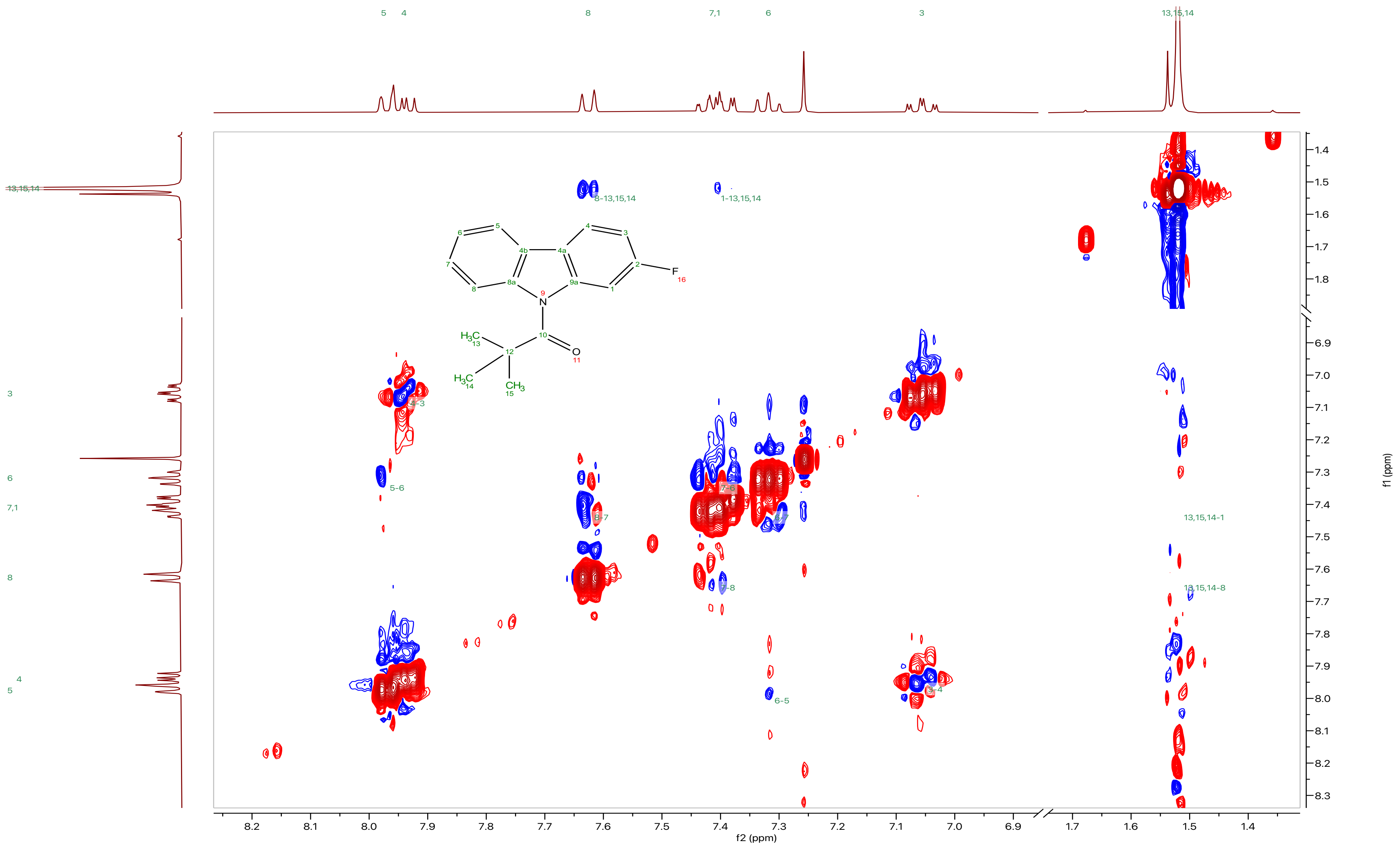

^{1}H-^{1}H NOESY (400 MHz, $CDCl_3$) of 3i

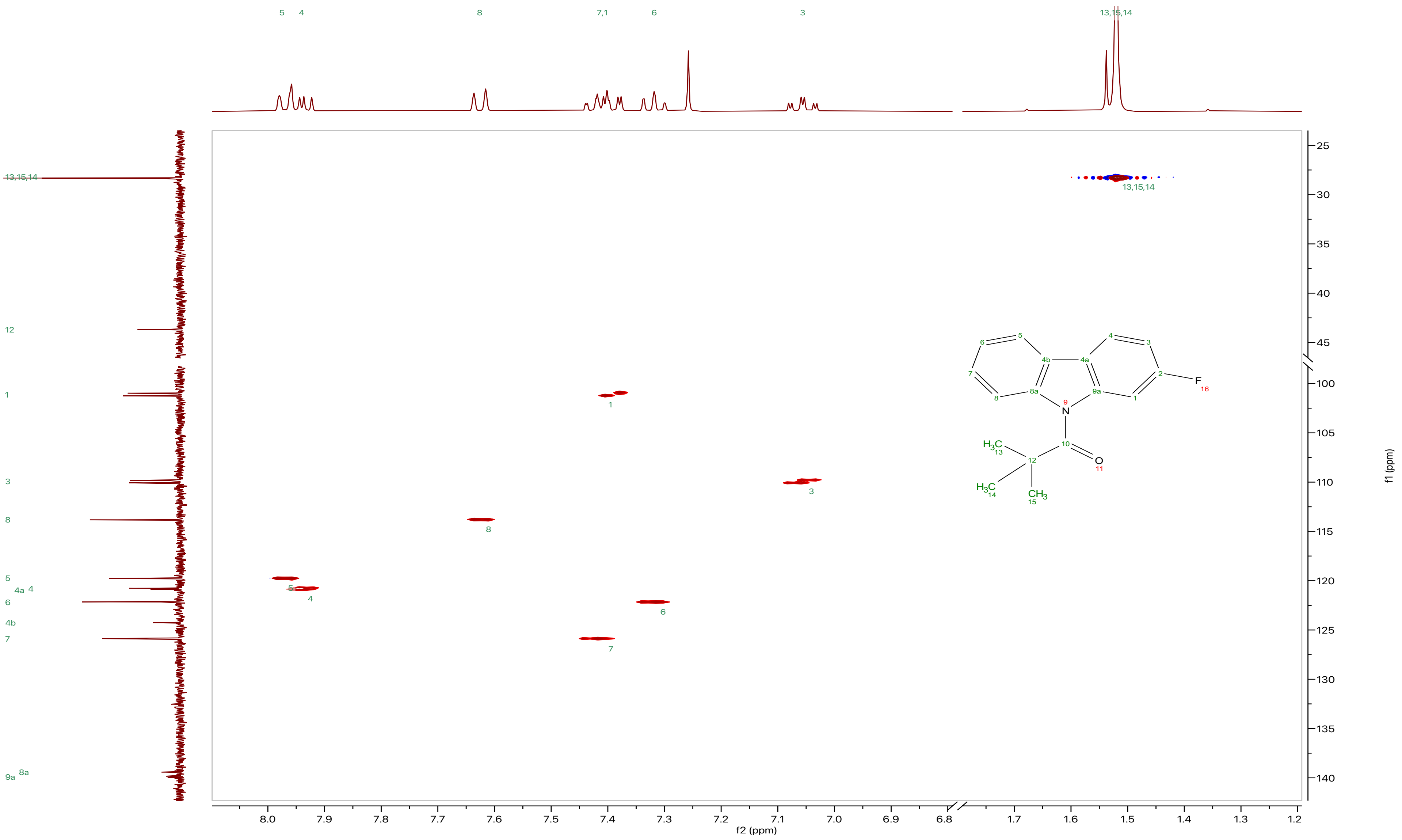

^{1}H-^{13}C{^{1}H} HSQC NMR (400/101 MHz, $CDCl_3$) of 3i

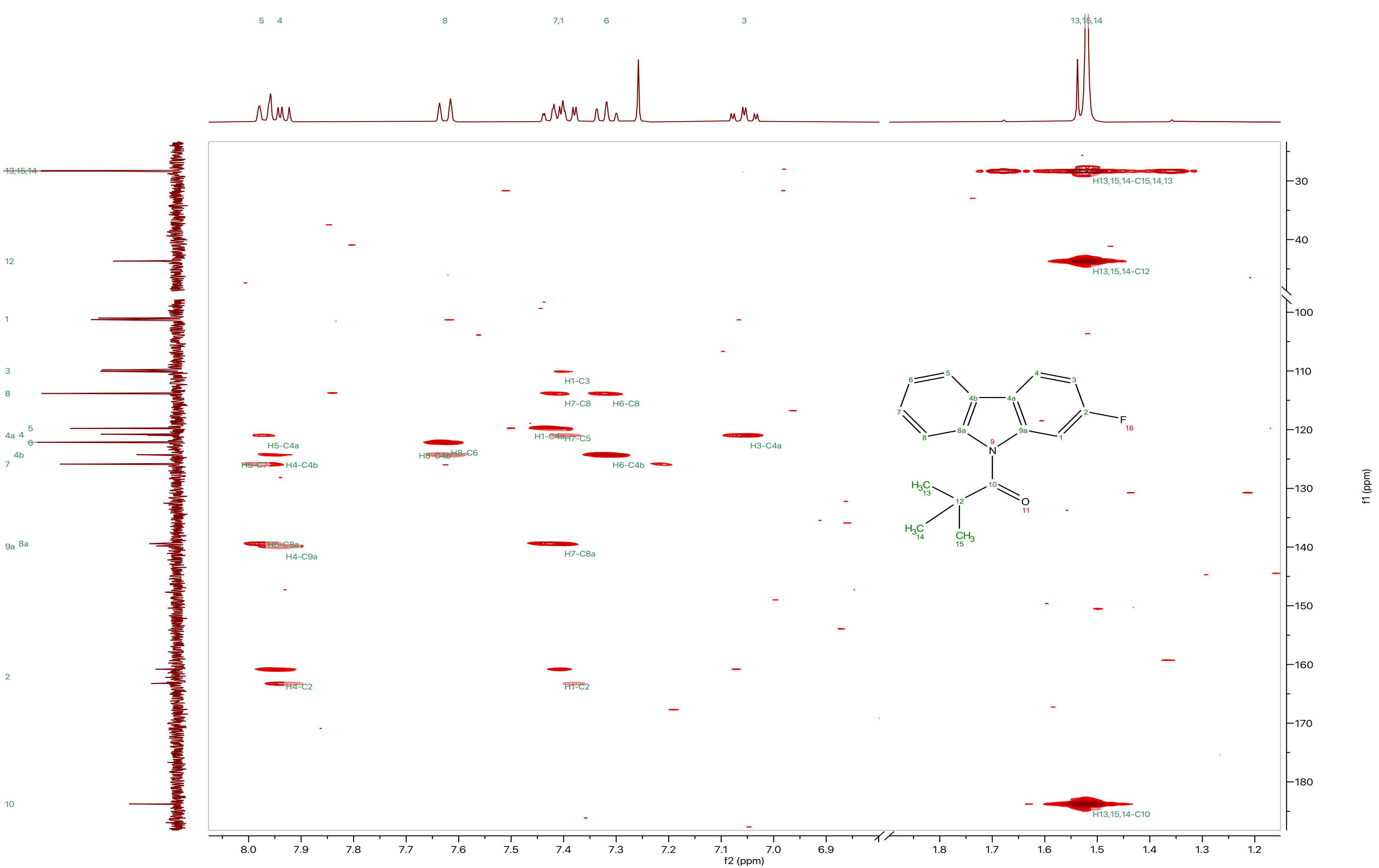

^{1}H-$^{13}C\{^{1}H\}$ HMBC NMR (400/101 MHz, $CDCl_3$) of 3i

3i'

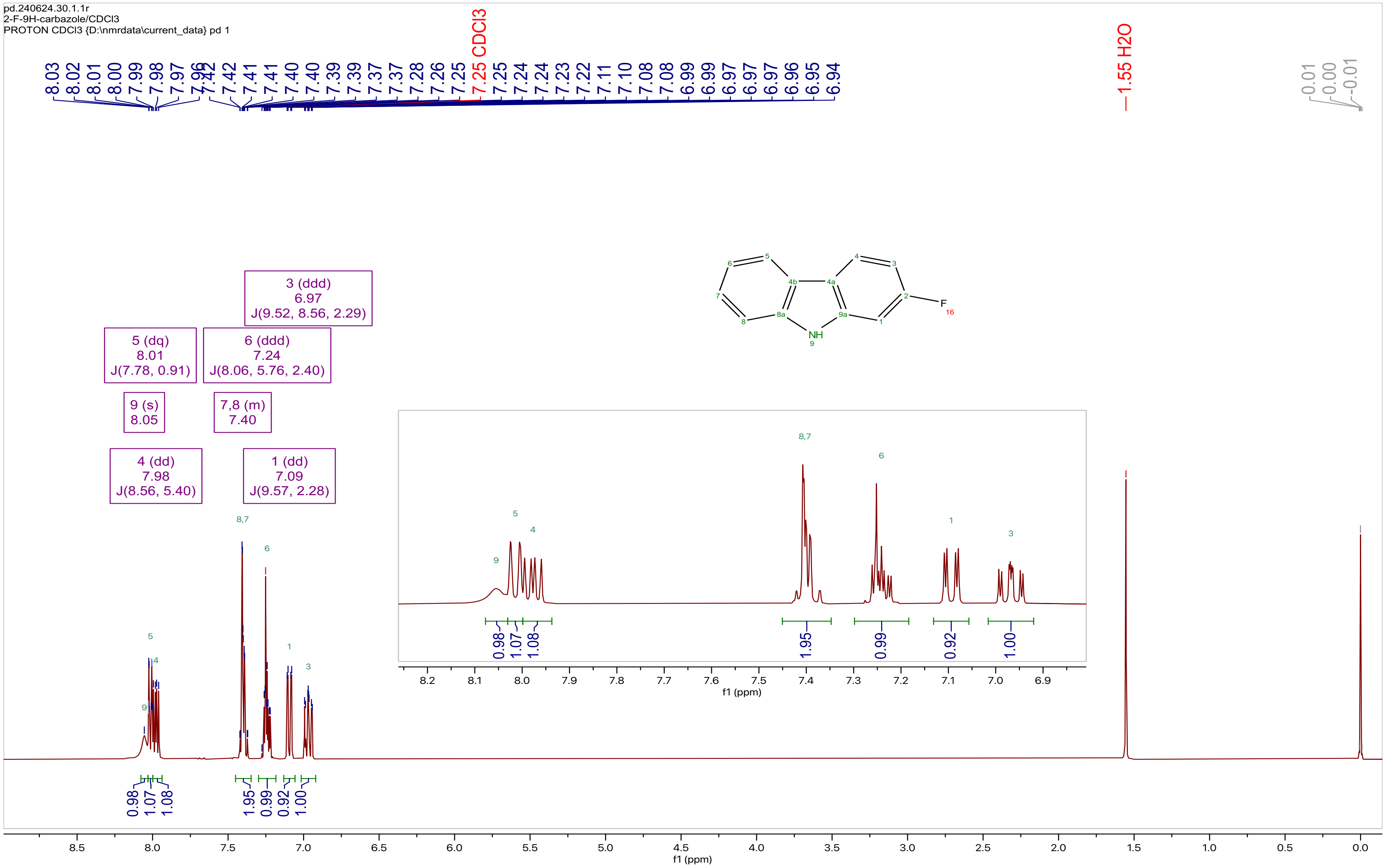

^1H NMR (400 MHz, $CDCl_3$) of 3i'

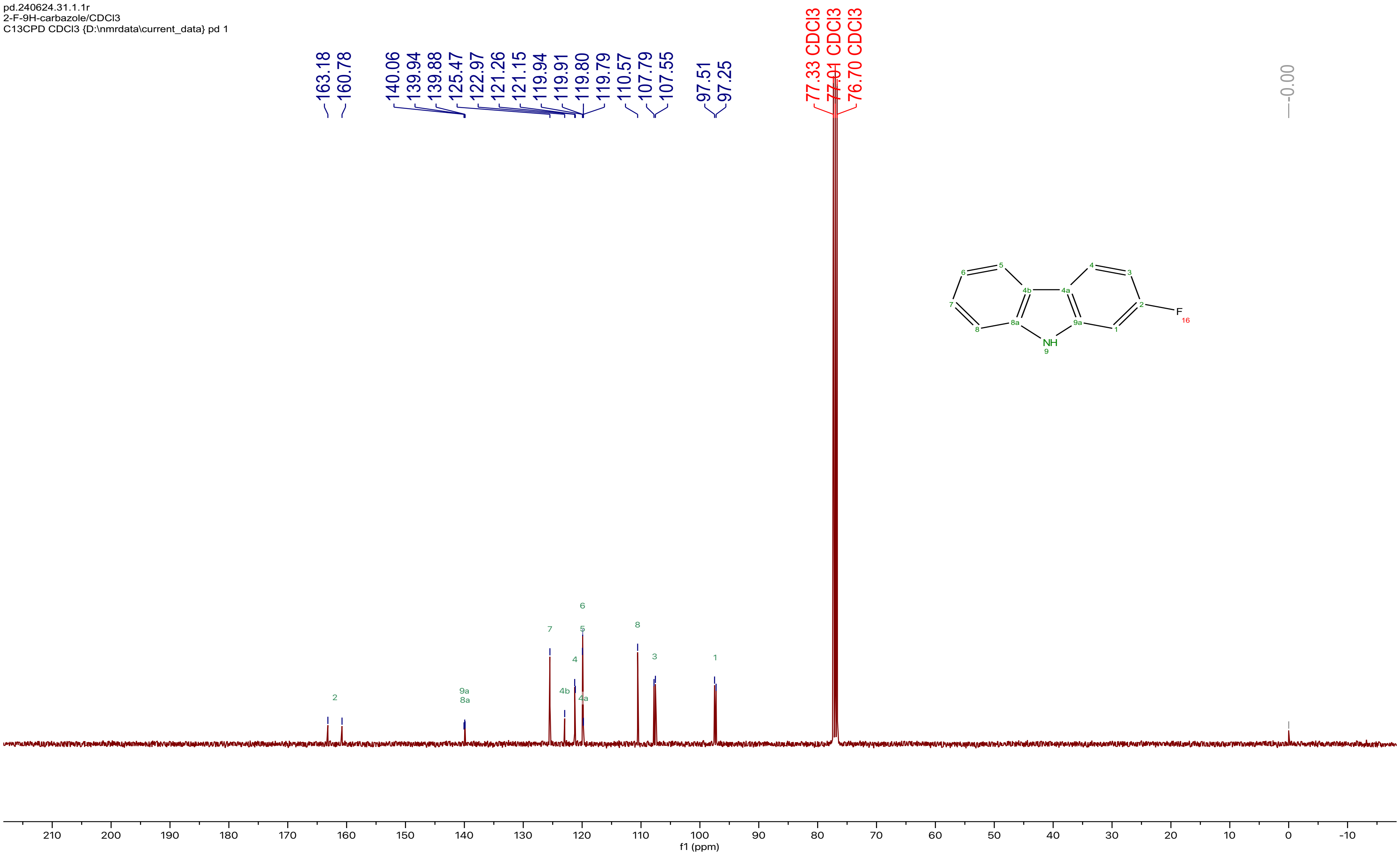

$^{13}C\{^{1}H\}$ NMR (101 MHz, $CDCl_3$) of 3i'

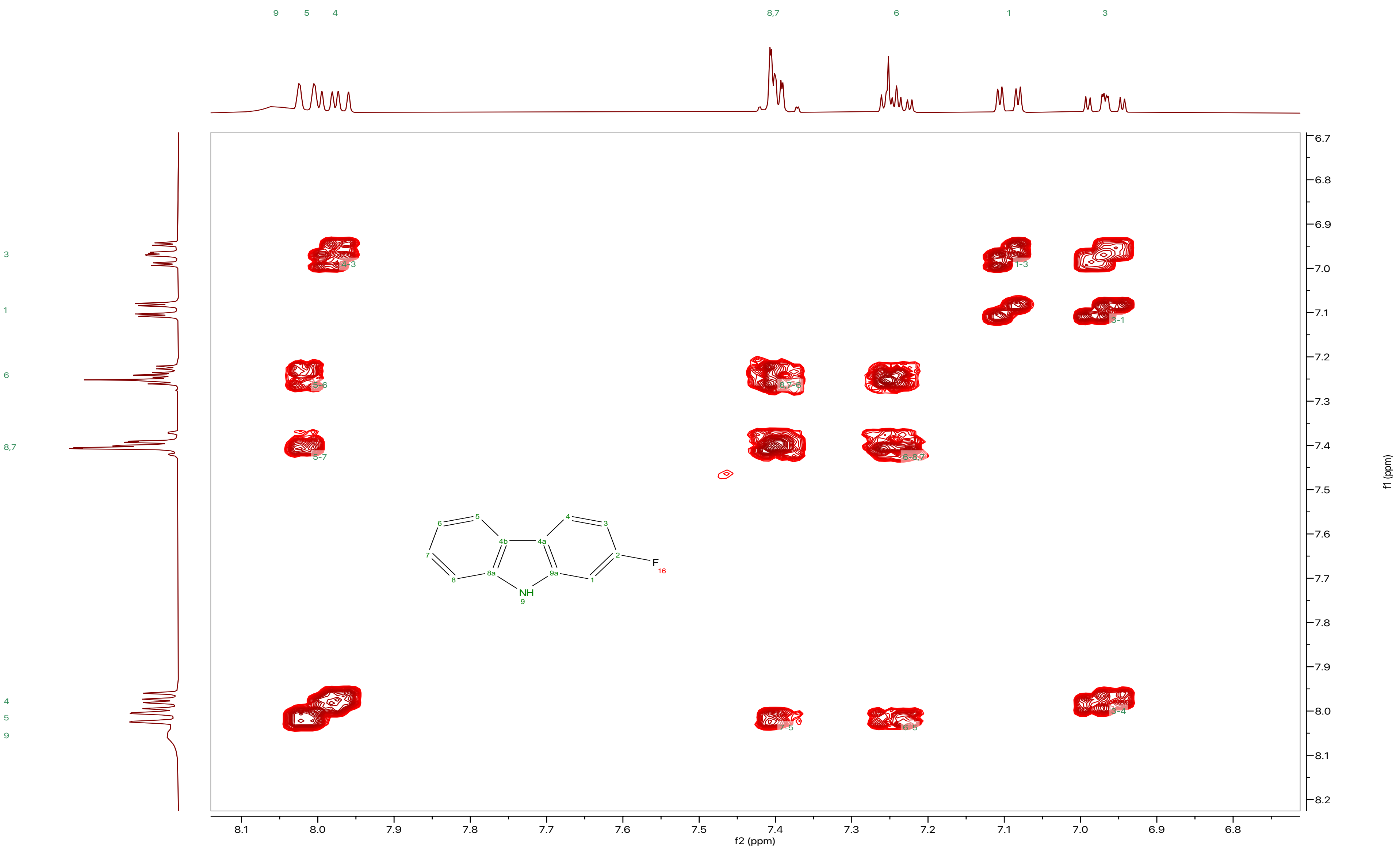

^{1}H-^{1}H COSY (400 MHz, $CDCl_3$) of 3i'

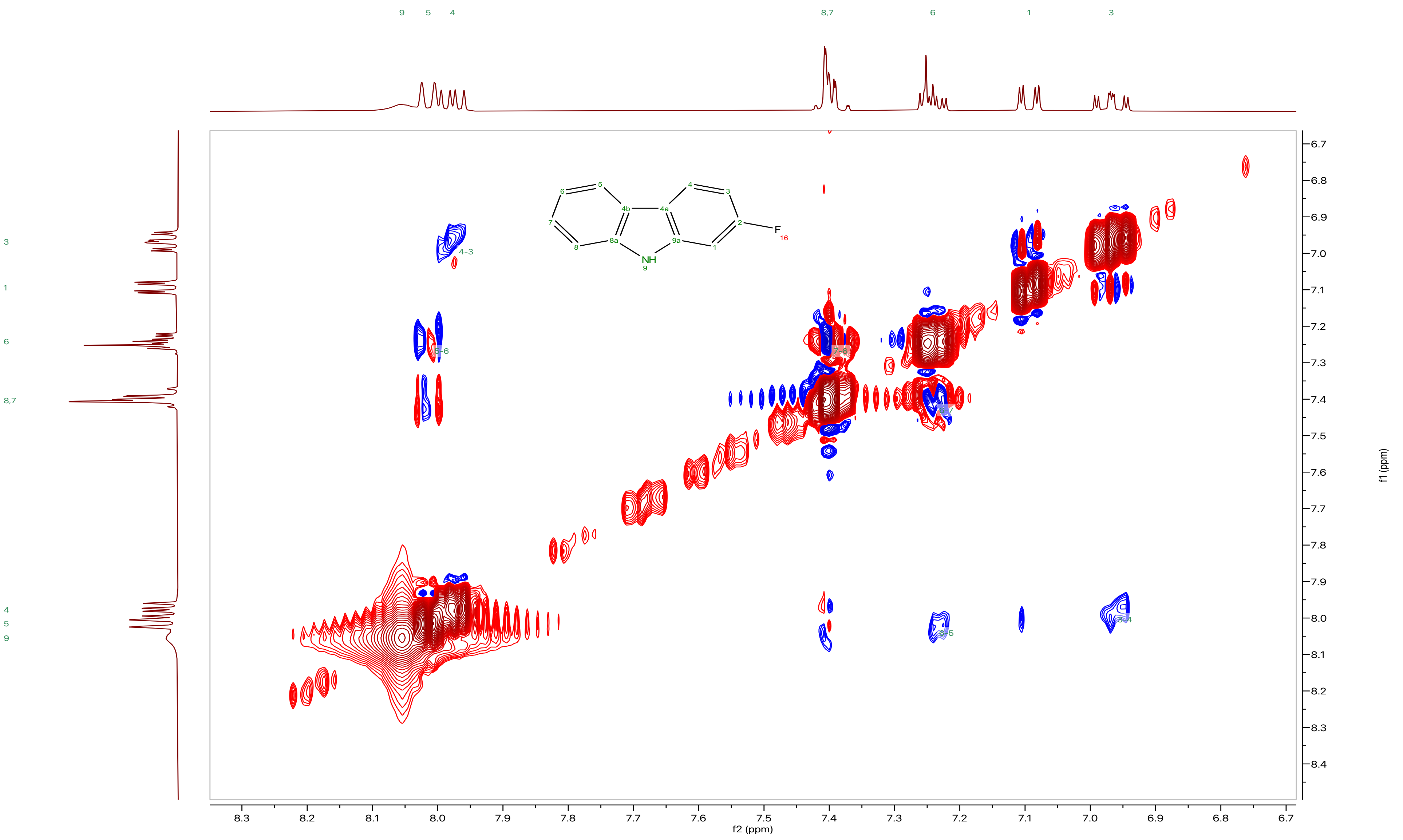

¹H-¹H NOESY (400 MHz, CDCl₃) of 3i’

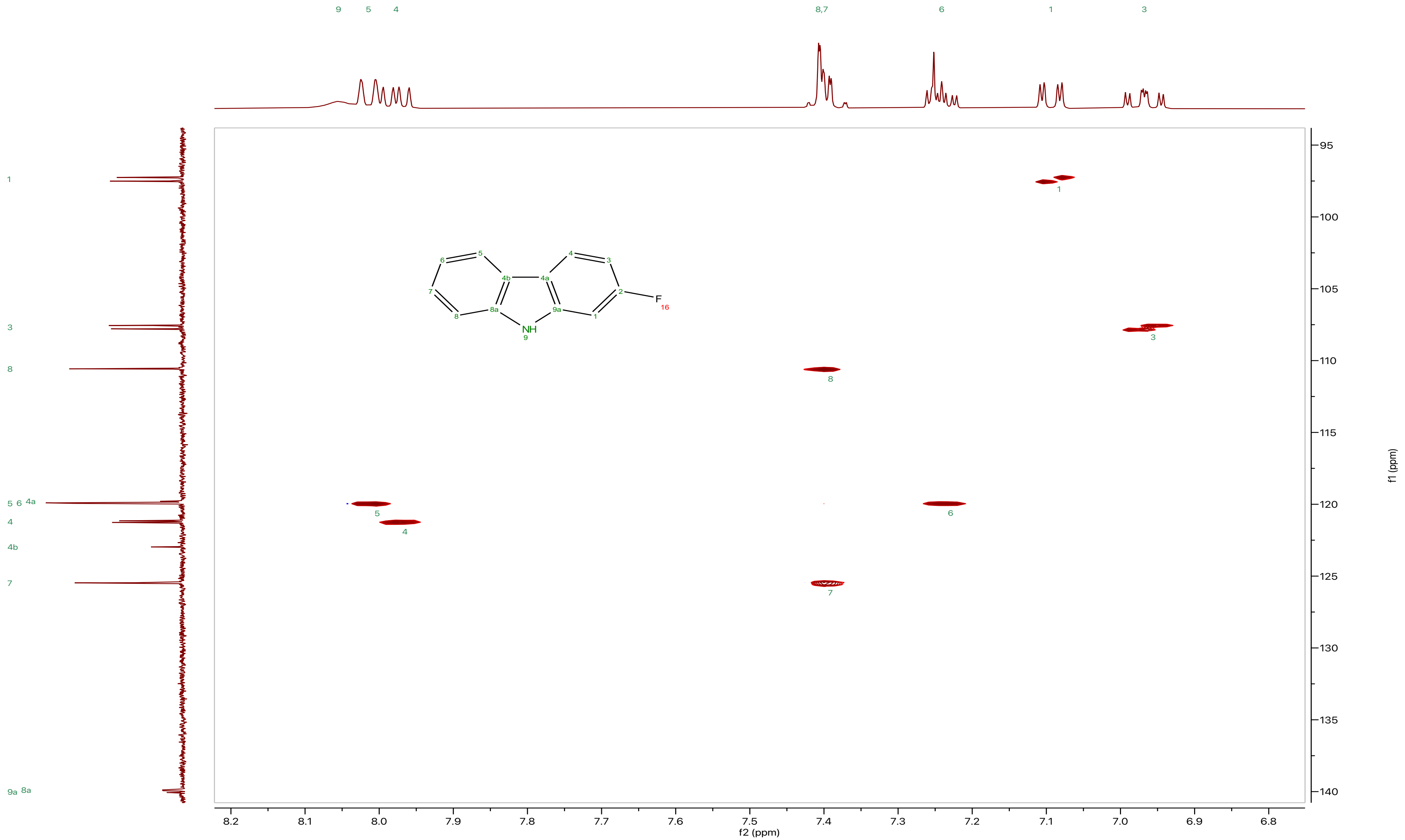

^{1}H-^{13}C{^{1}H} HSQC NMR (400/101 MHz, $CDCl_3$) of 3i'

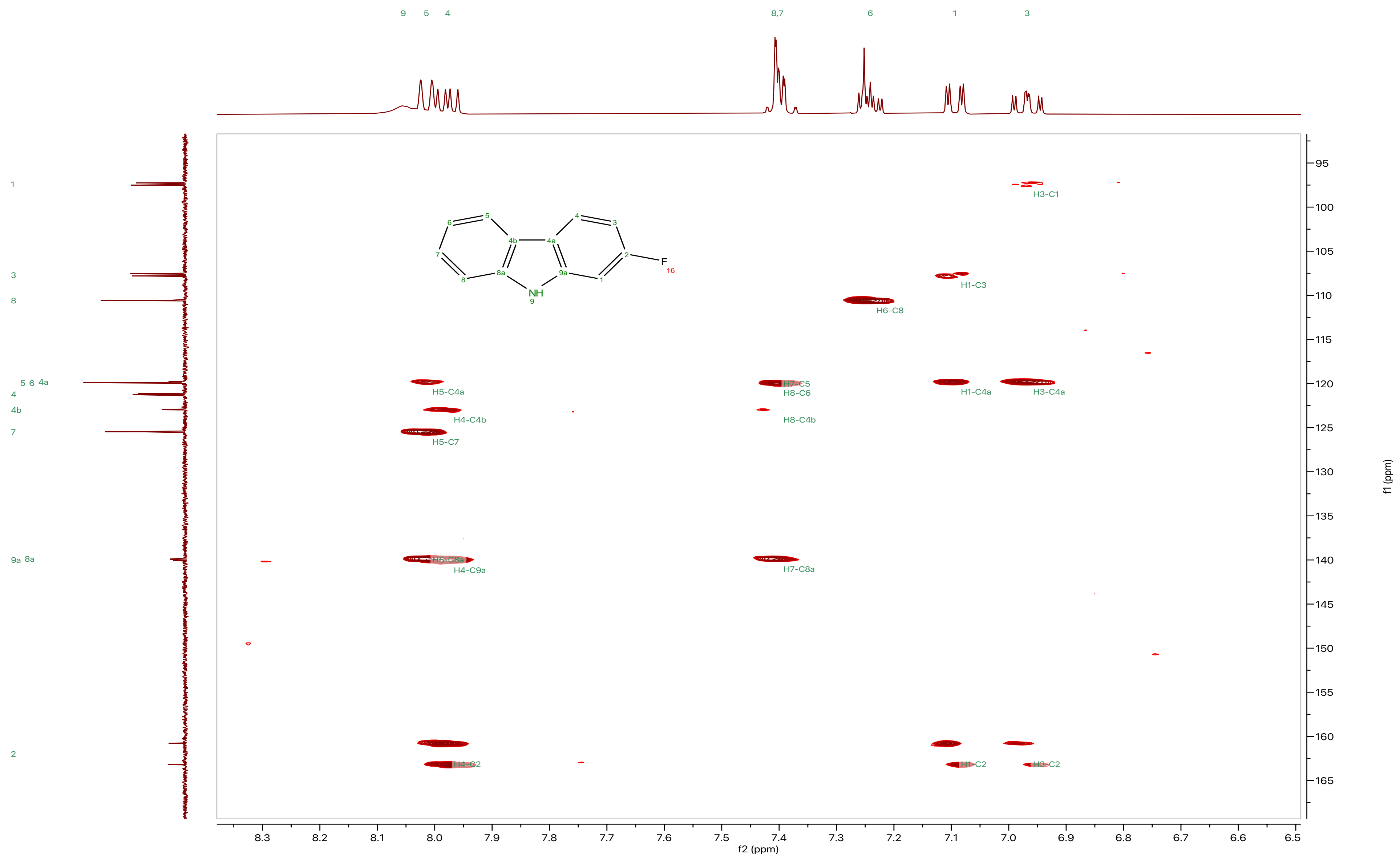

^{1}H-^{13}C{^{1}H} HMBC NMR (400/101 MHz, $CDCl_3$) of 3i’

4bi

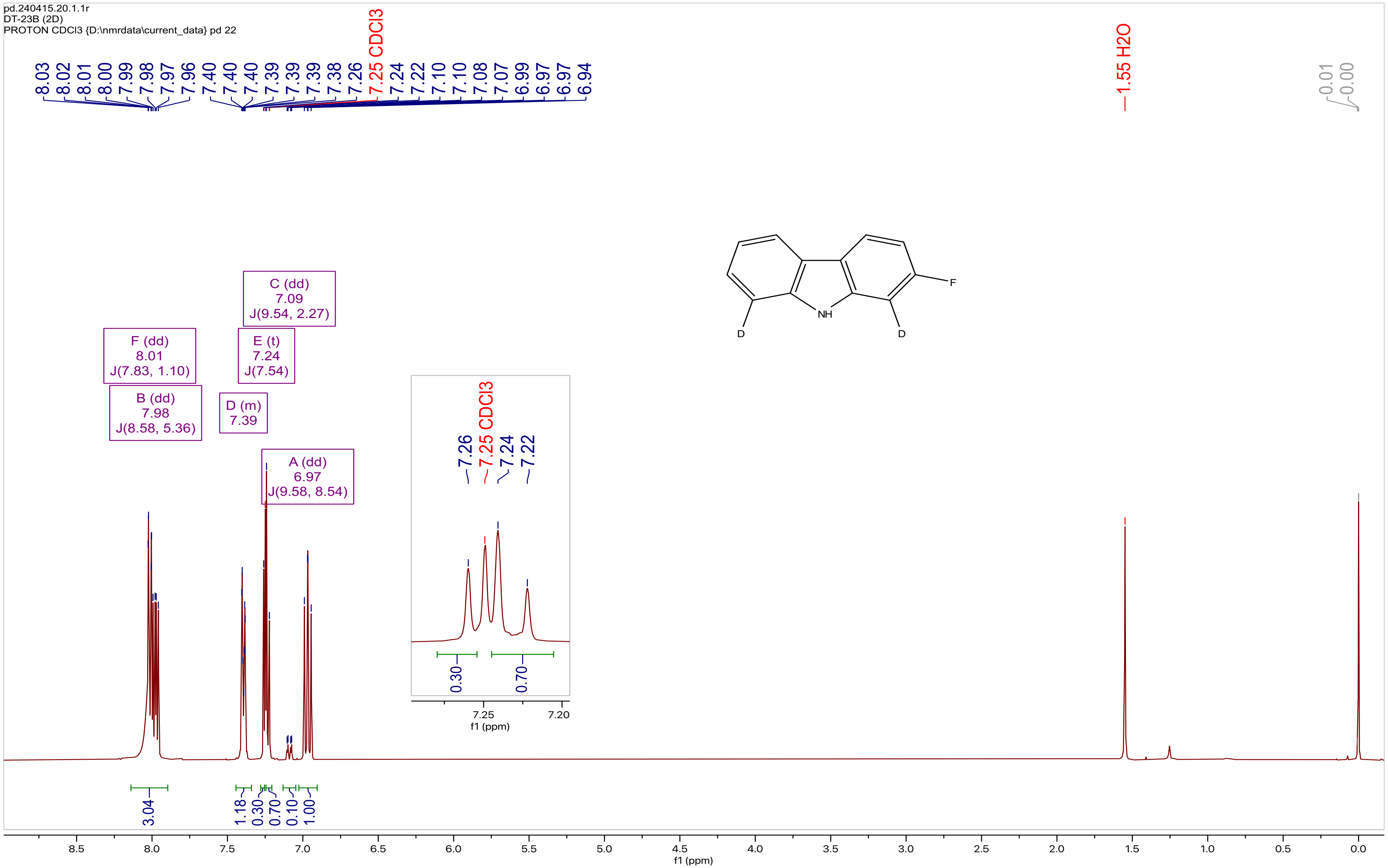

^{1}H NMR (400 MHz, $CDCl_3$) of 4bi

pd.240415.21.1.1r
DT-23B (2D)
C13CPD CDCl3 {D:\nmrdata\current_data} pd 22

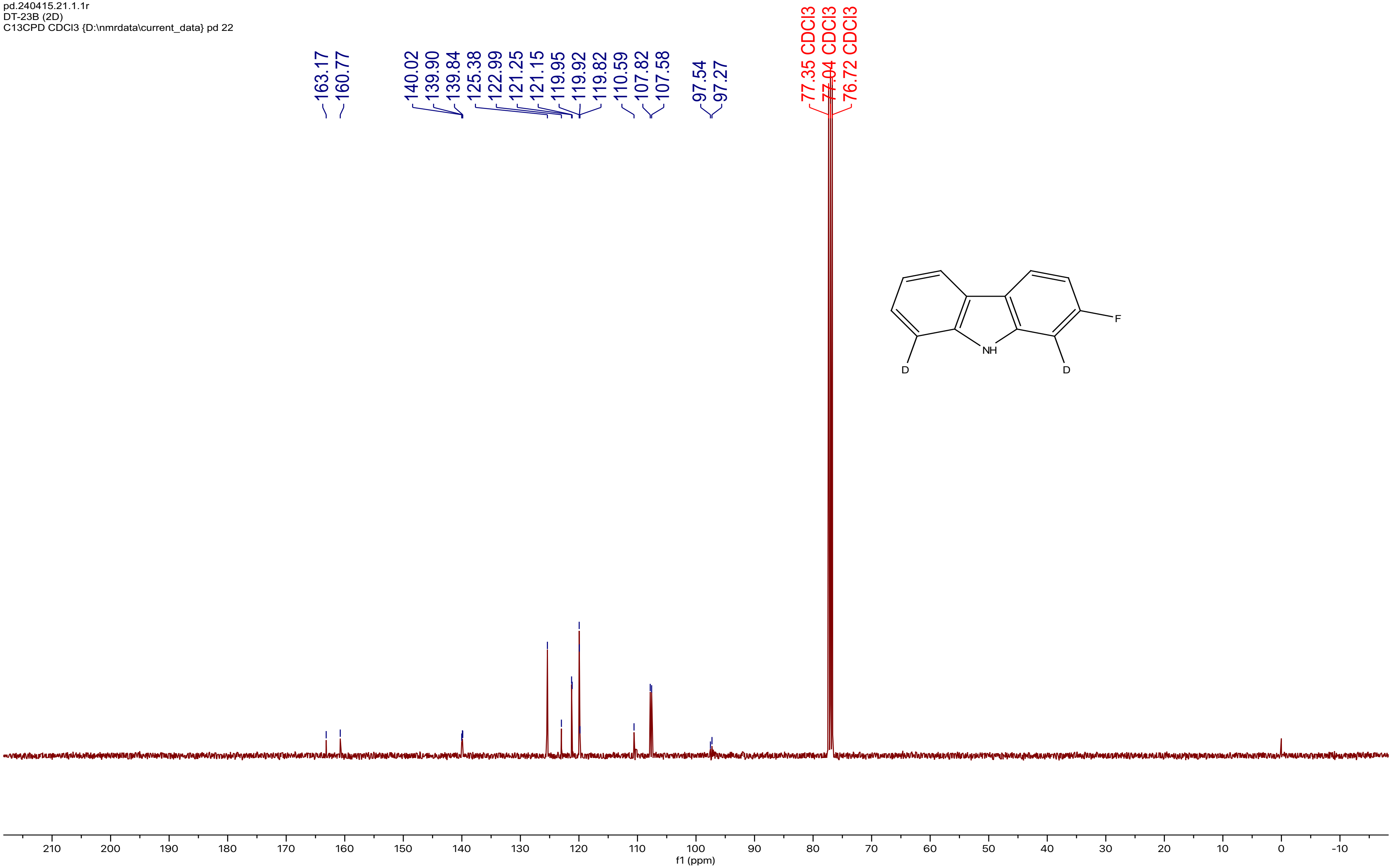

$^{13}C\{^1H\}$ NMR (101 MHz, $CDCl_3$) of 4bi

3j

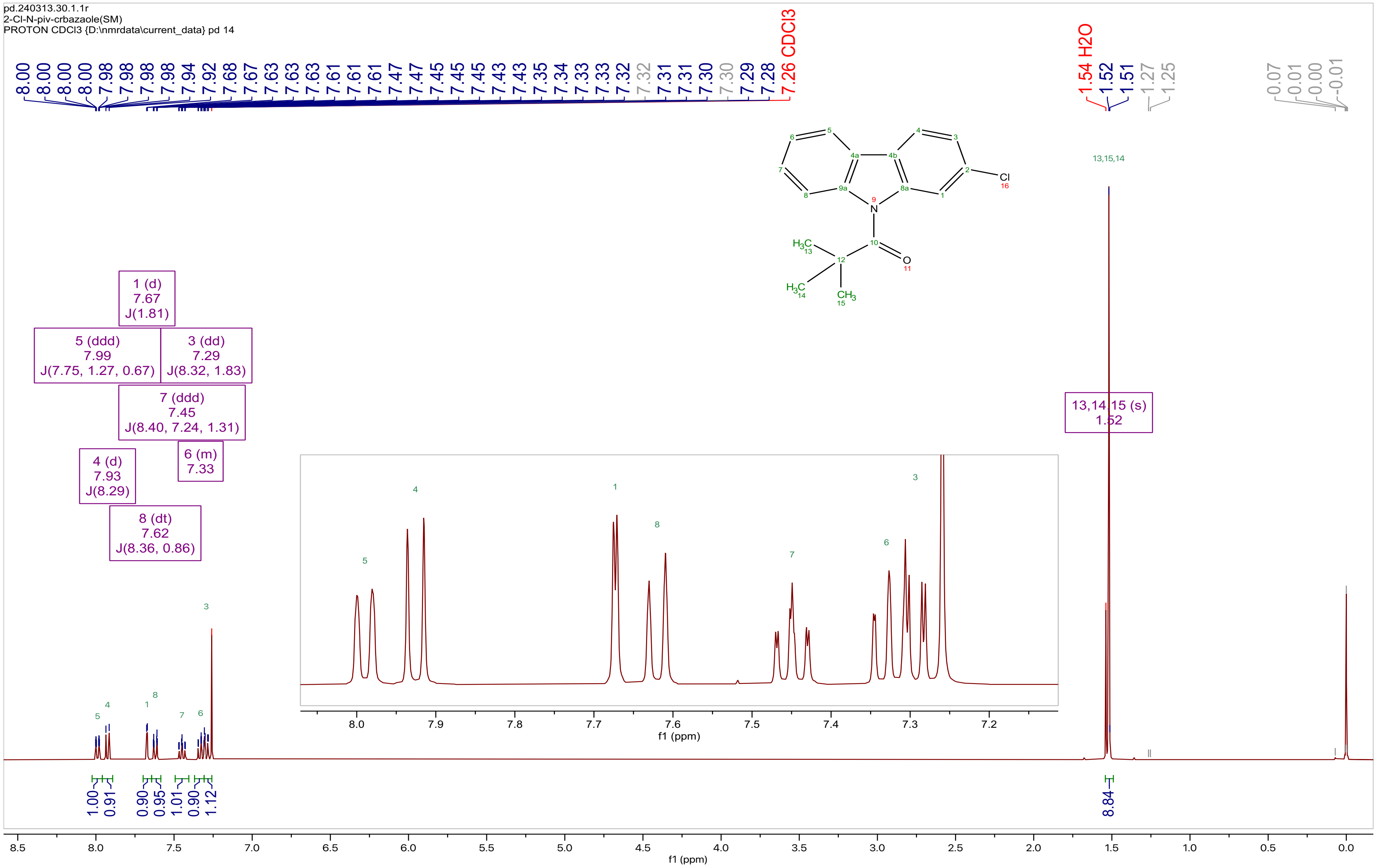

^{1}H NMR (400 MHz, $CDCl_3$) of 3j

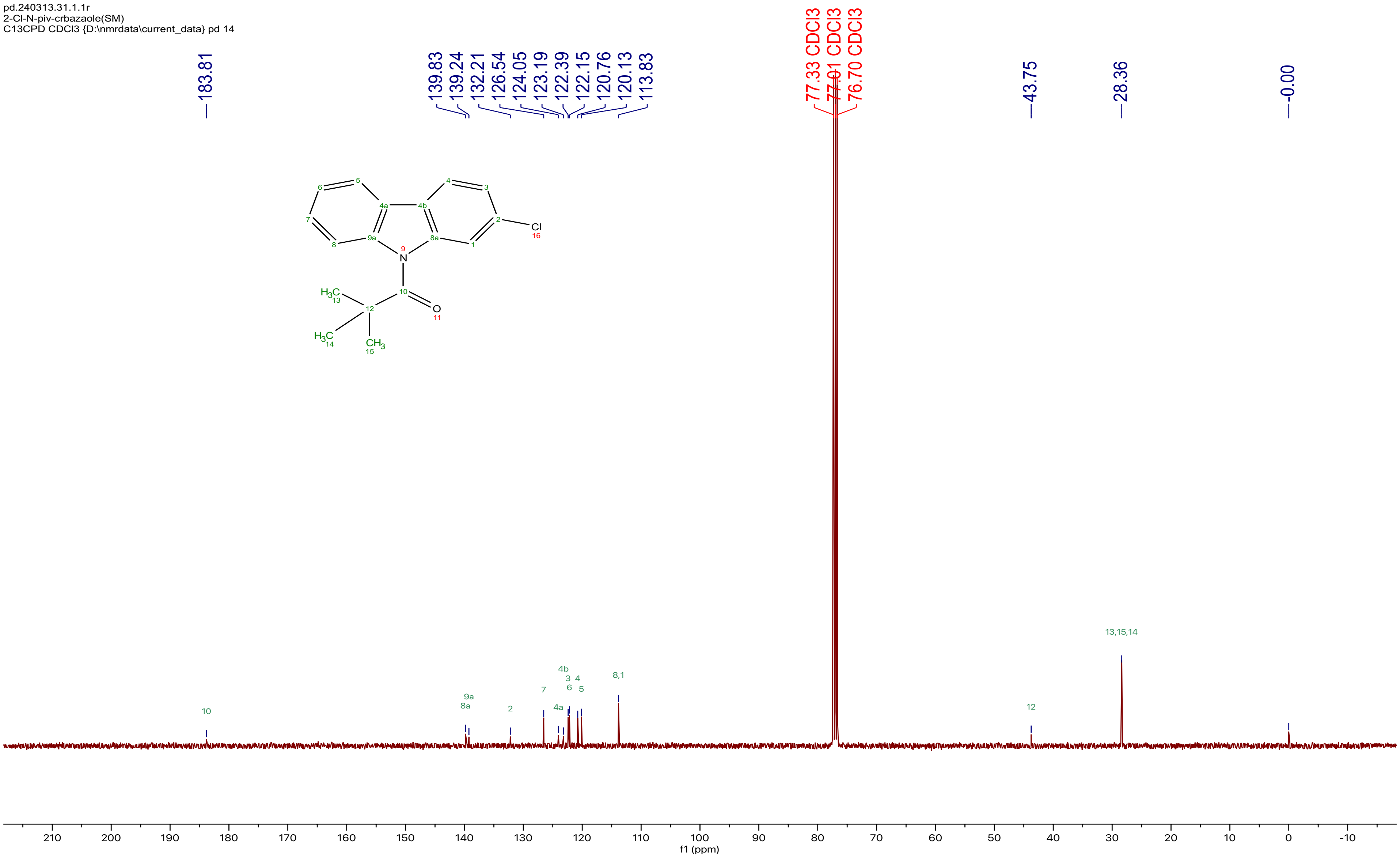

$^{13}C\{^{1}H\}$ NMR (101 MHz, $CDCl_3$) of 3j

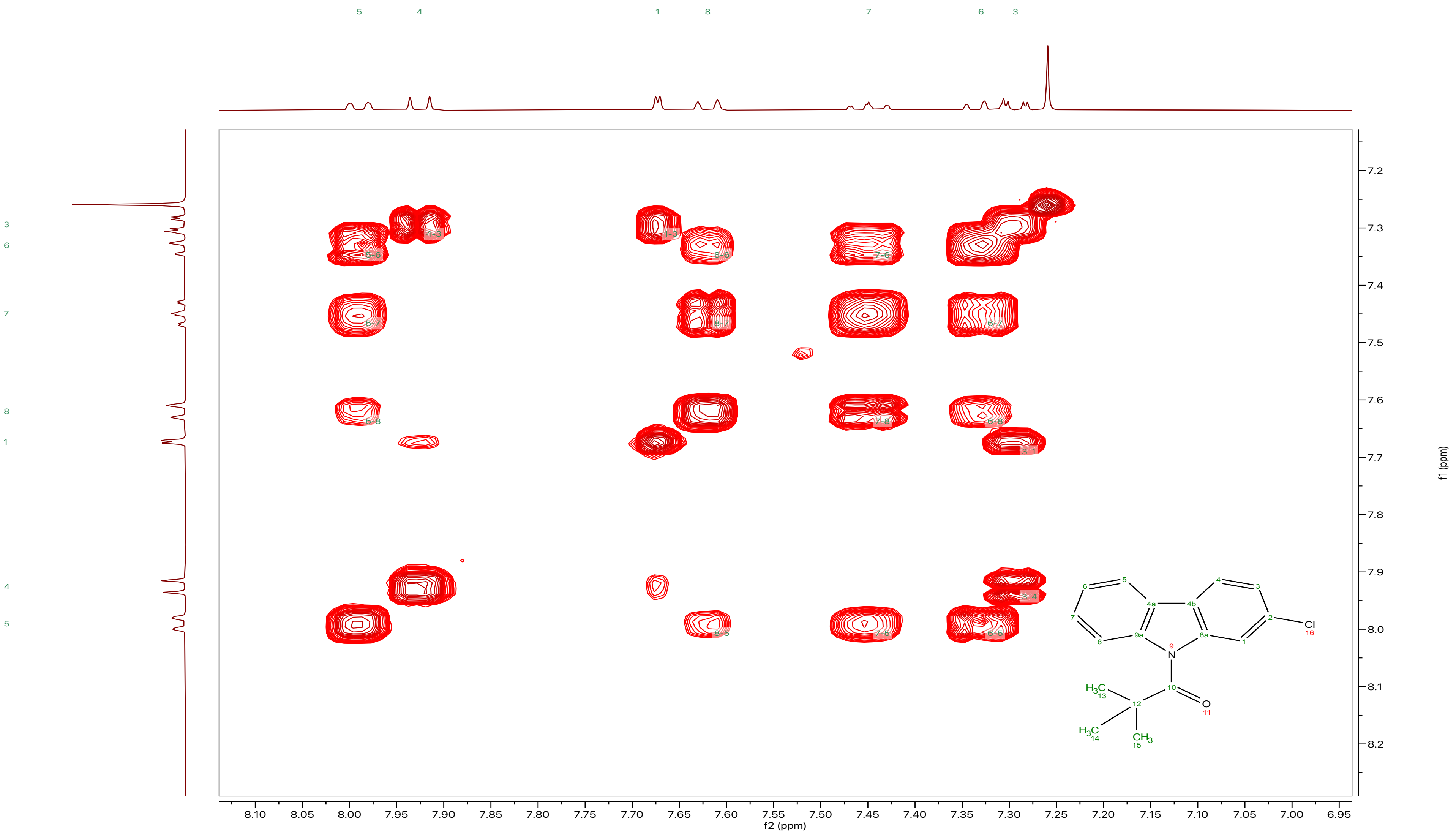

^{1}H-^{1}H COSY (400 MHz, $CDCl_3$) of 3j

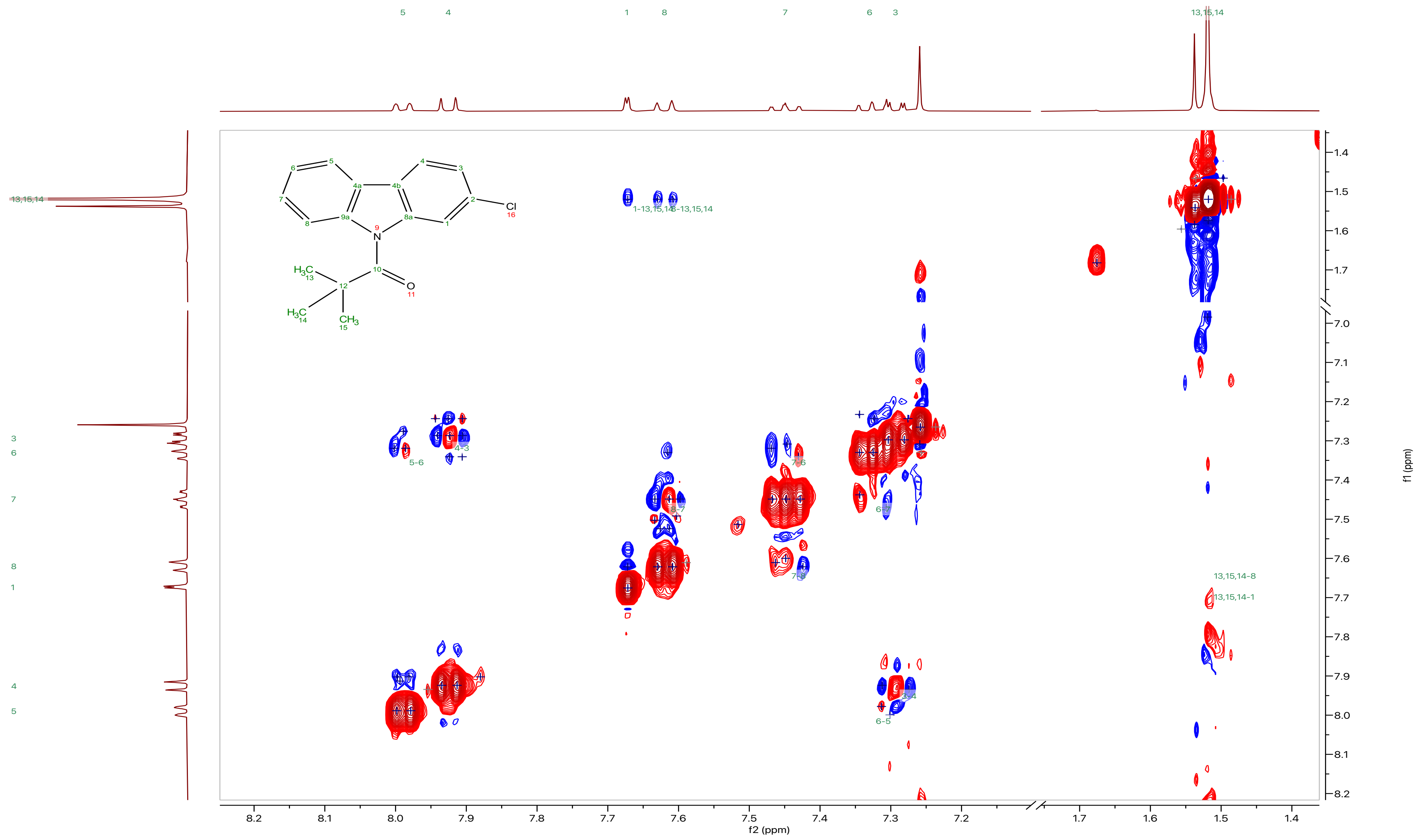

^{1}H-^{1}H NOESY (400 MHz, $CDCl_3$) of 3j

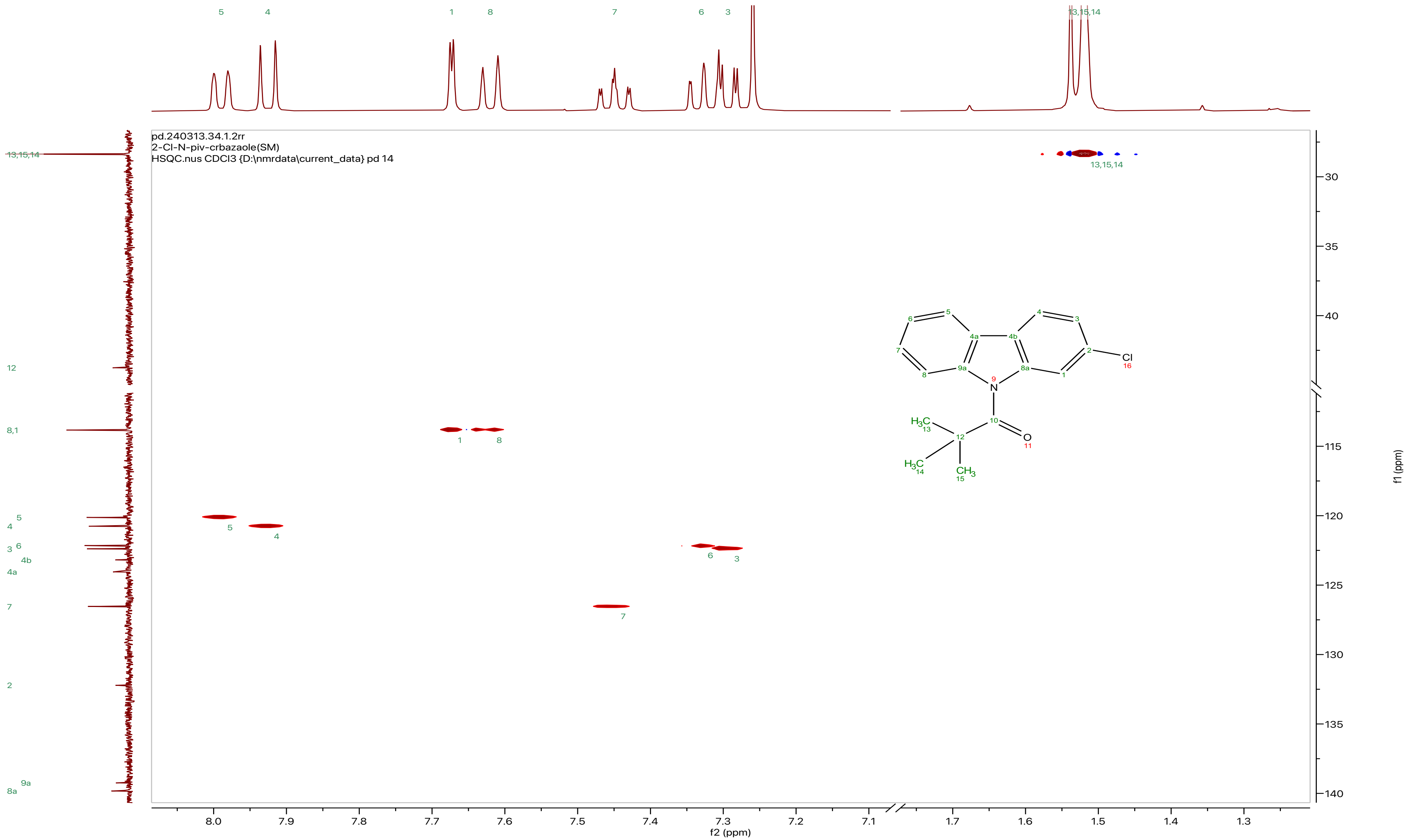

^{1}H-^{13}C{^{1}H} HSQC NMR (400/101 MHz, $CDCl_3$) of 3j

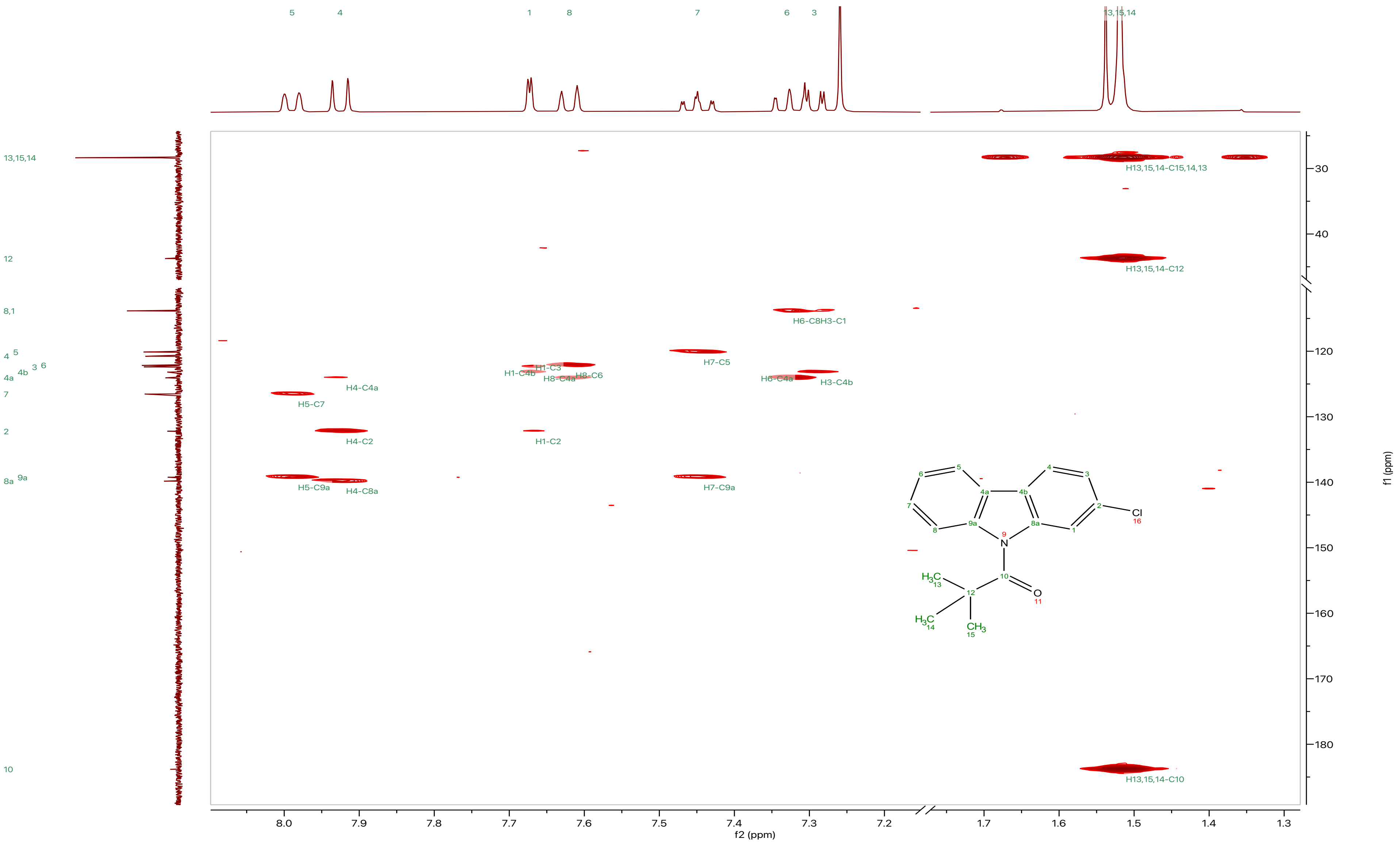

^{1}H-$^{13}C\{^{1}H\}$ HMBC NMR (400/101 MHz, $CDCl_3$) of 3j

4aj

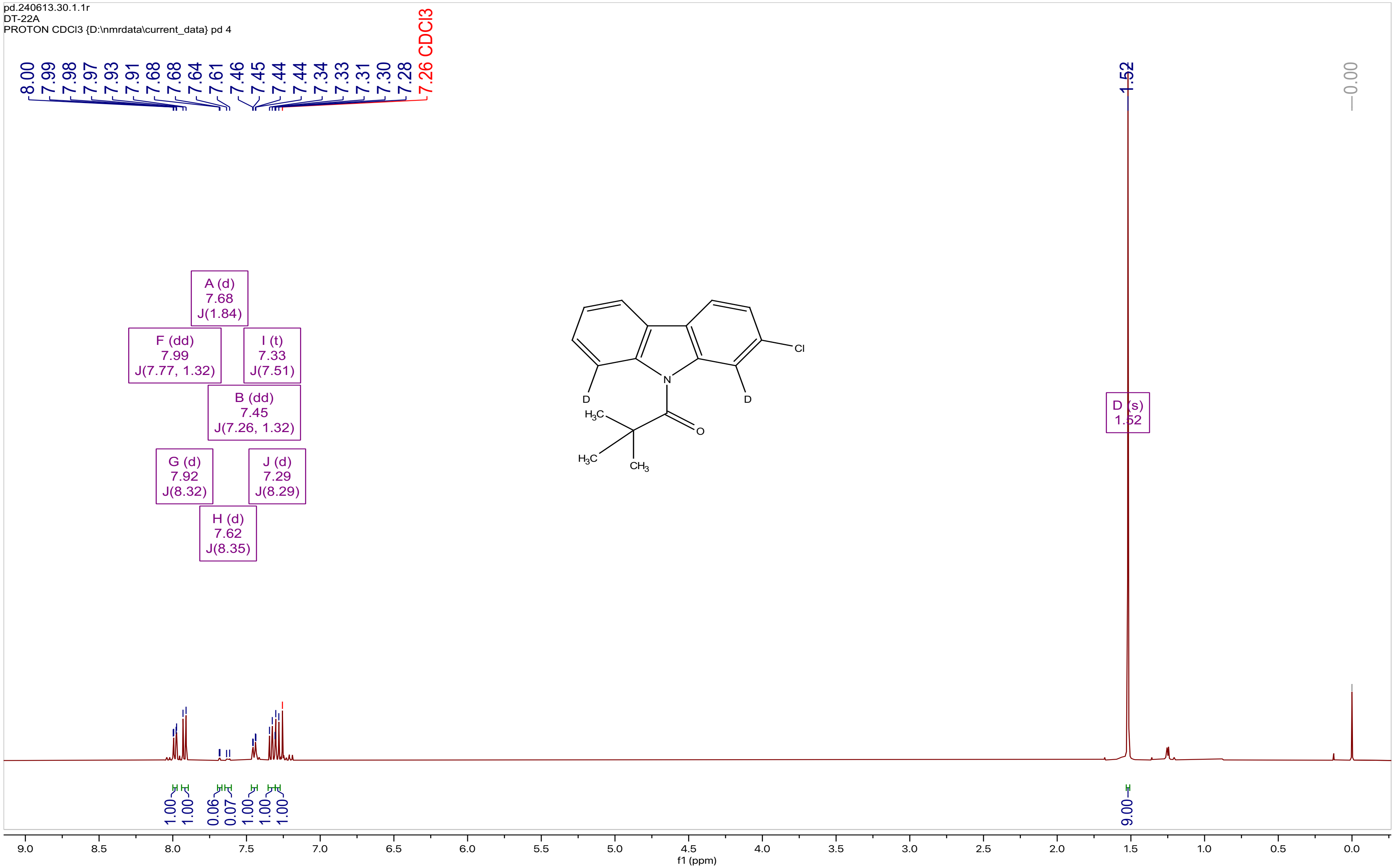

^{1}H NMR (400 MHz, $CDCl_3$) of 4aj

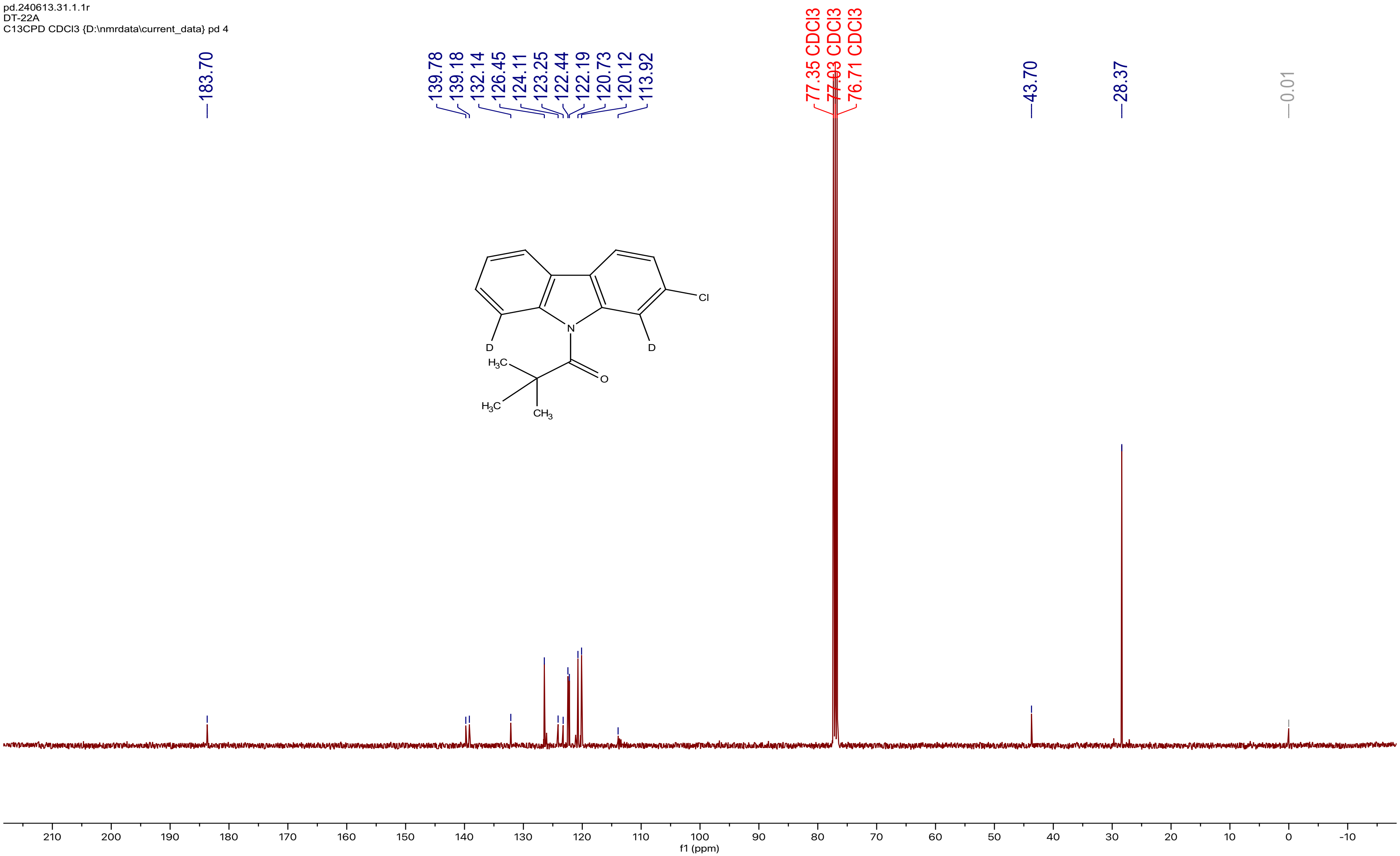

$^{13}C\{^1H\}$ NMR (101 MHz, $CDCl_3$) of 4aj

3j'

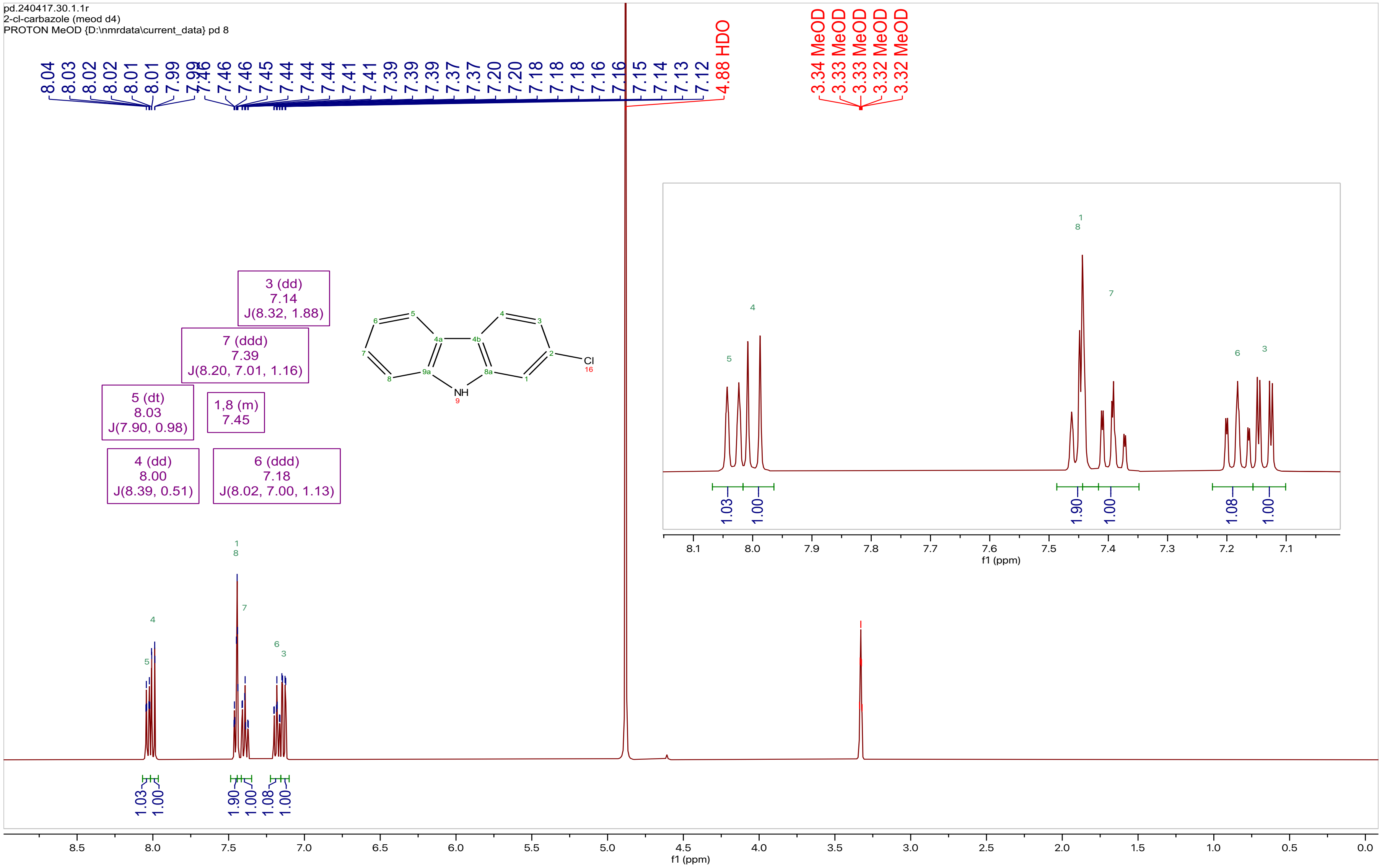

^{1}H NMR (400 MHz, MeOD) of 3j'

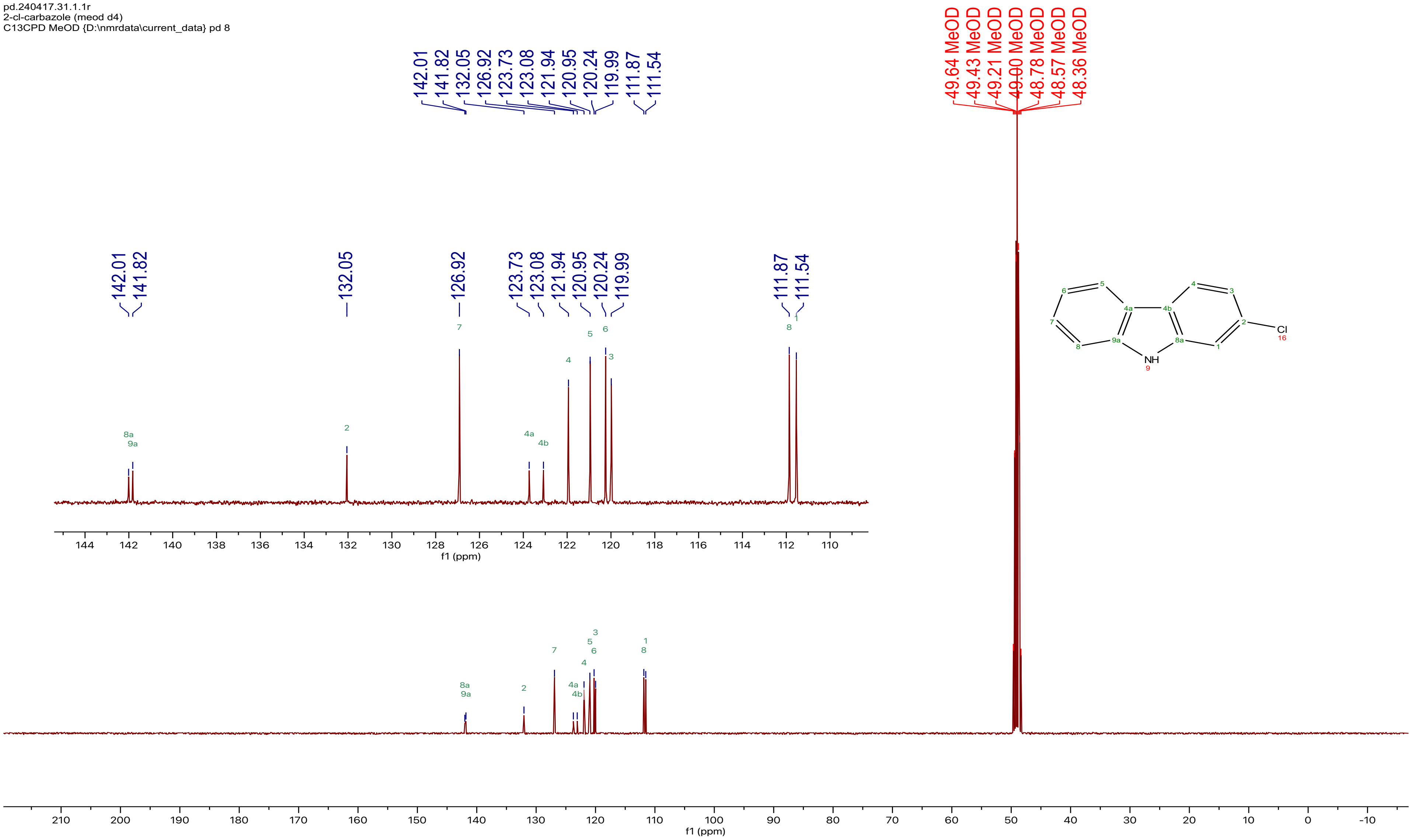

$^{13}C\{^{1}H\}$ NMR (101 MHz, MeOD) of 3j'

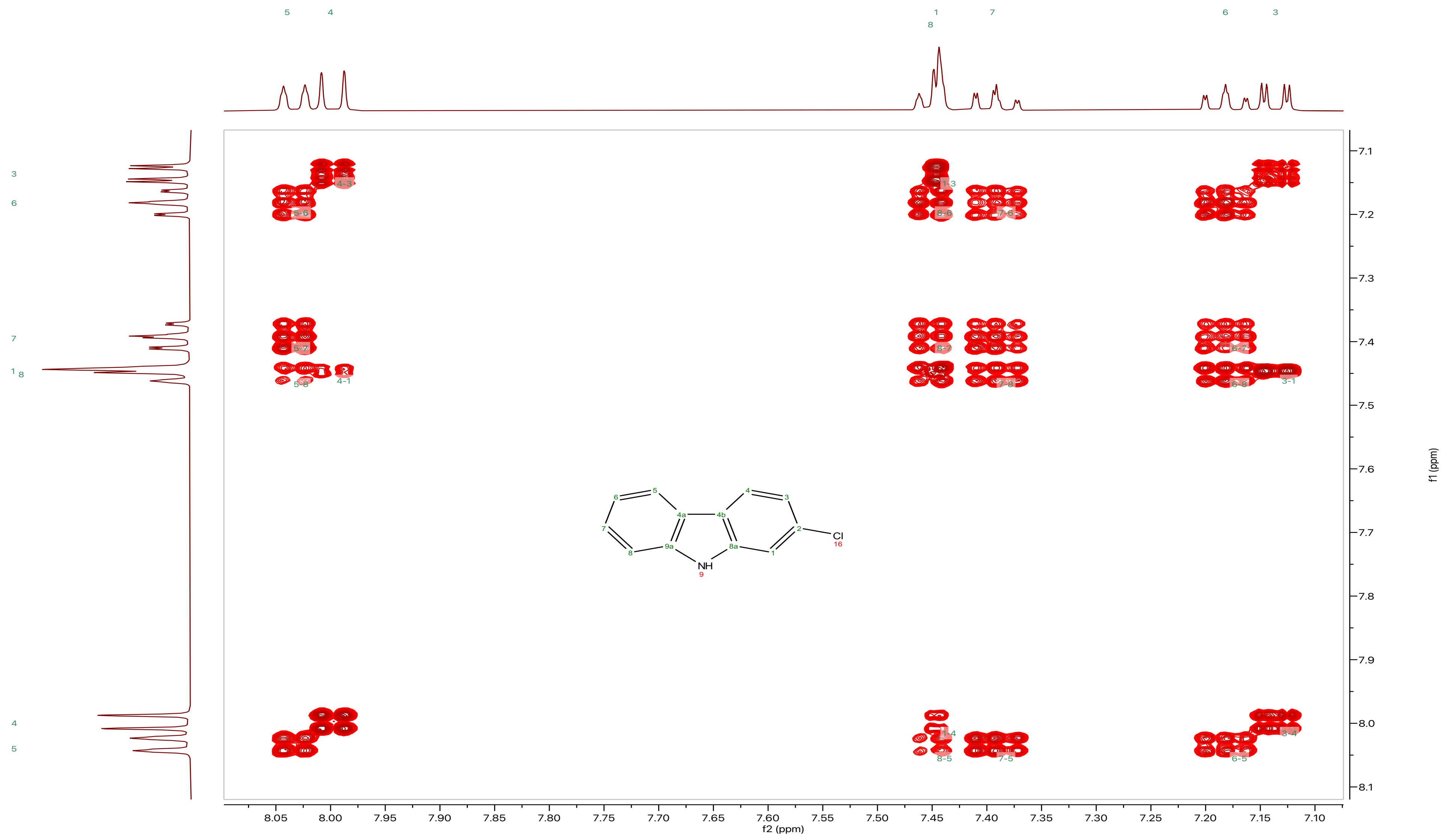

^{1}H-^{1}H COSY (400 MHz, MeOD) of 3j'

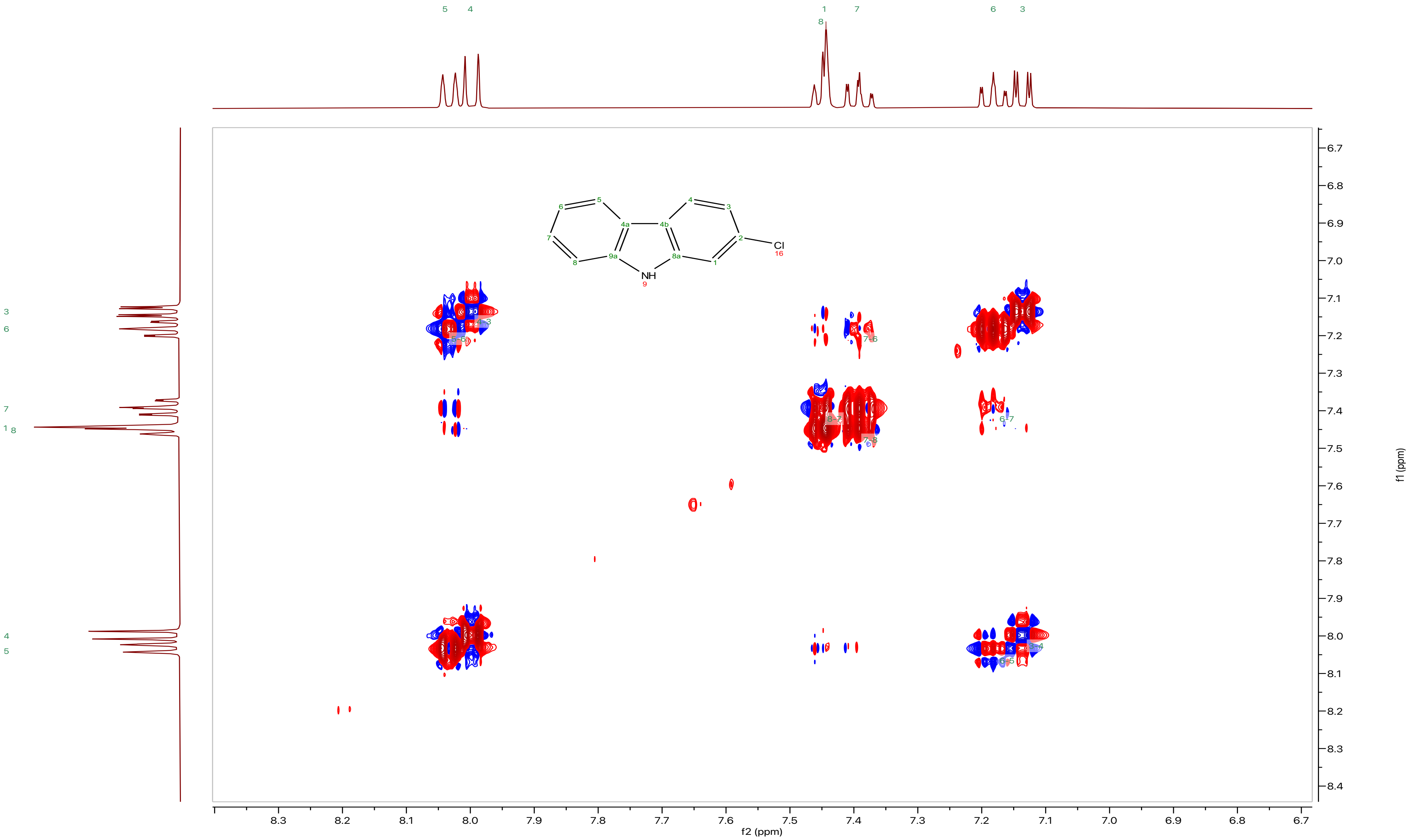

^{1}H-^{1}H NOESY (400 MHz, MeOD) of 3j'

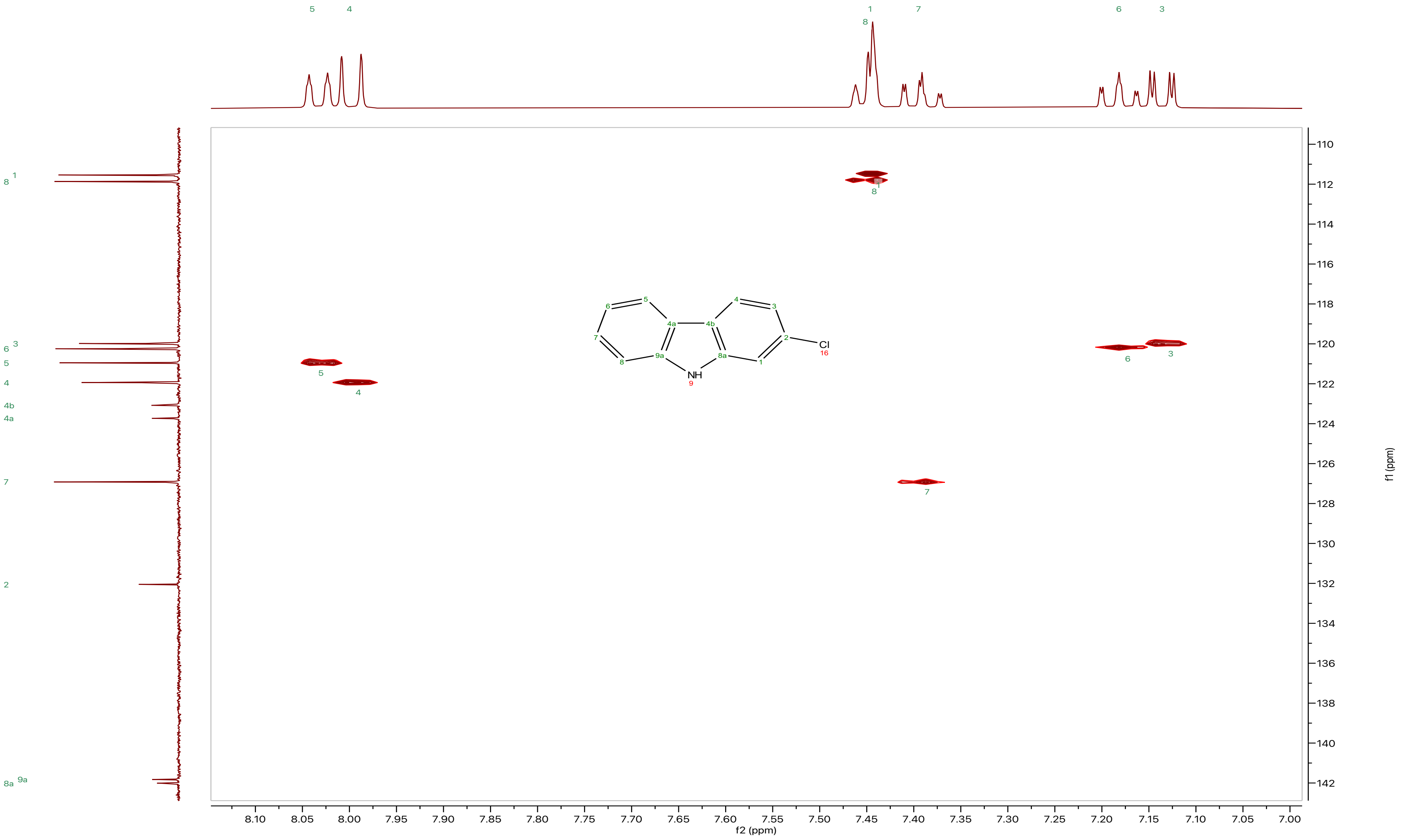

^{1}H-^{13}C{^{1}H} HSQC NMR (400/101 MHz, MeOD) of 3j'

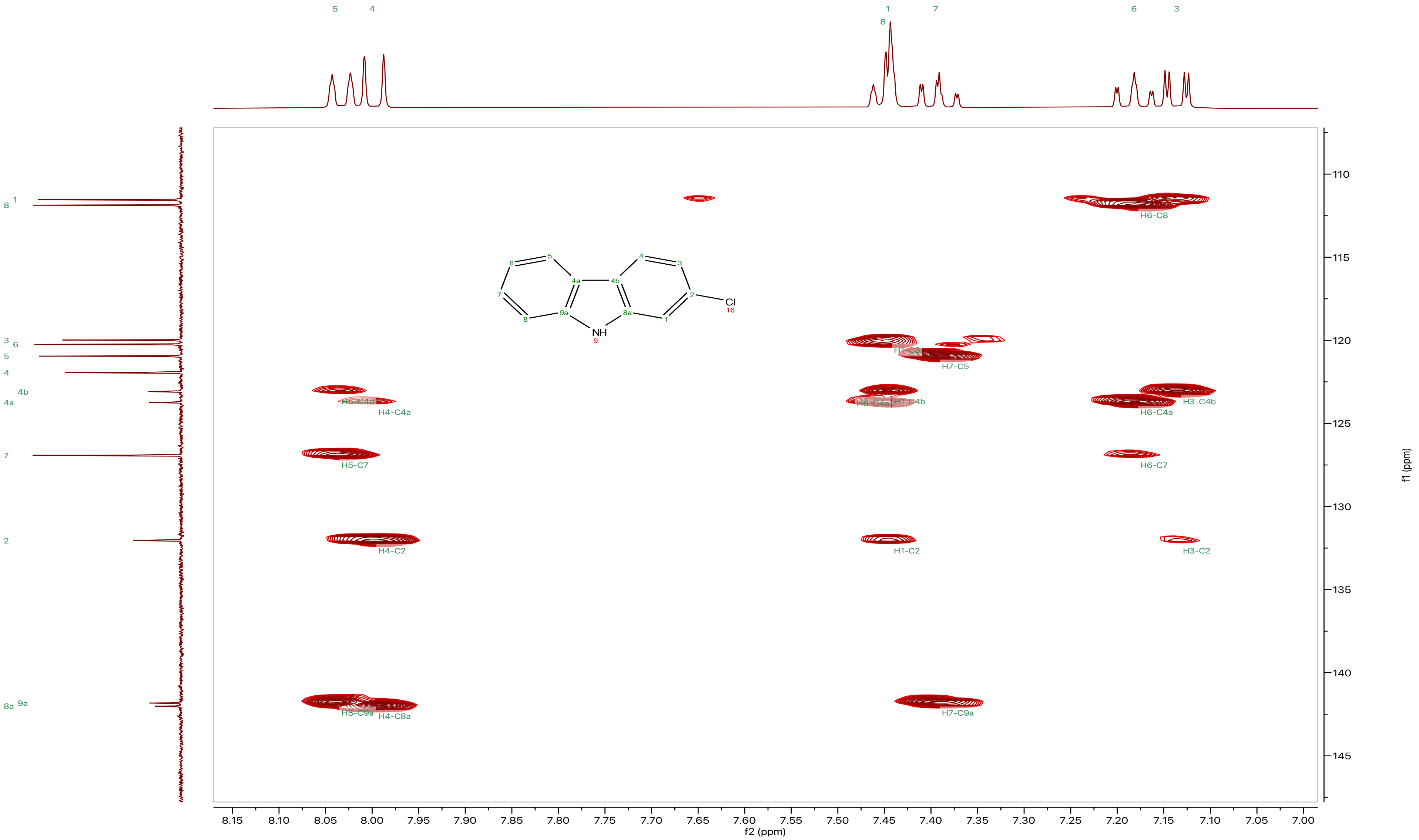

^{1}H-$^{13}C\{^{1}H\}$ HMBC NMR (400/101 MHz, MeOD) of 3j'

4bj

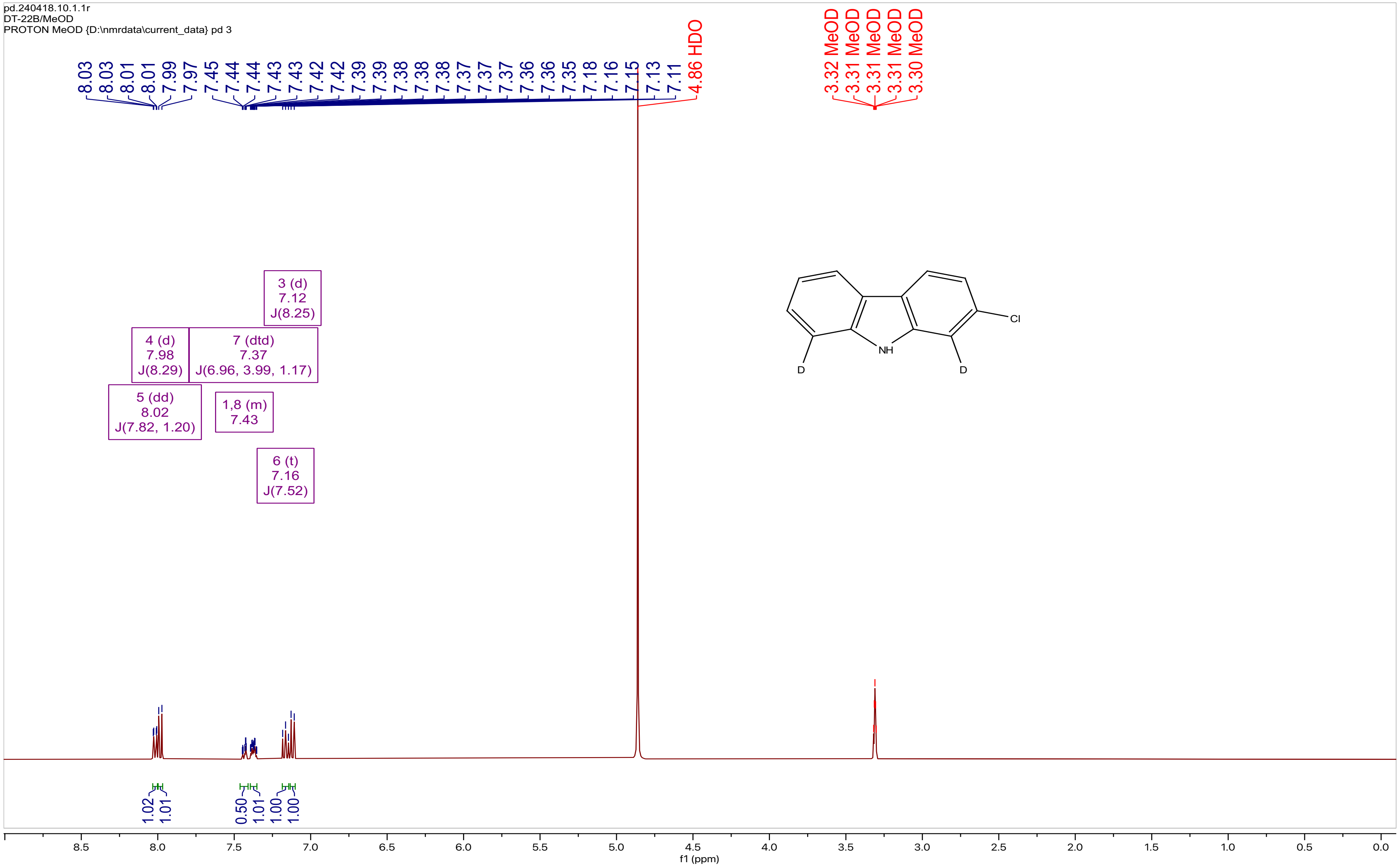

^{1}H NMR (400 MHz, MeOD) of 4bj

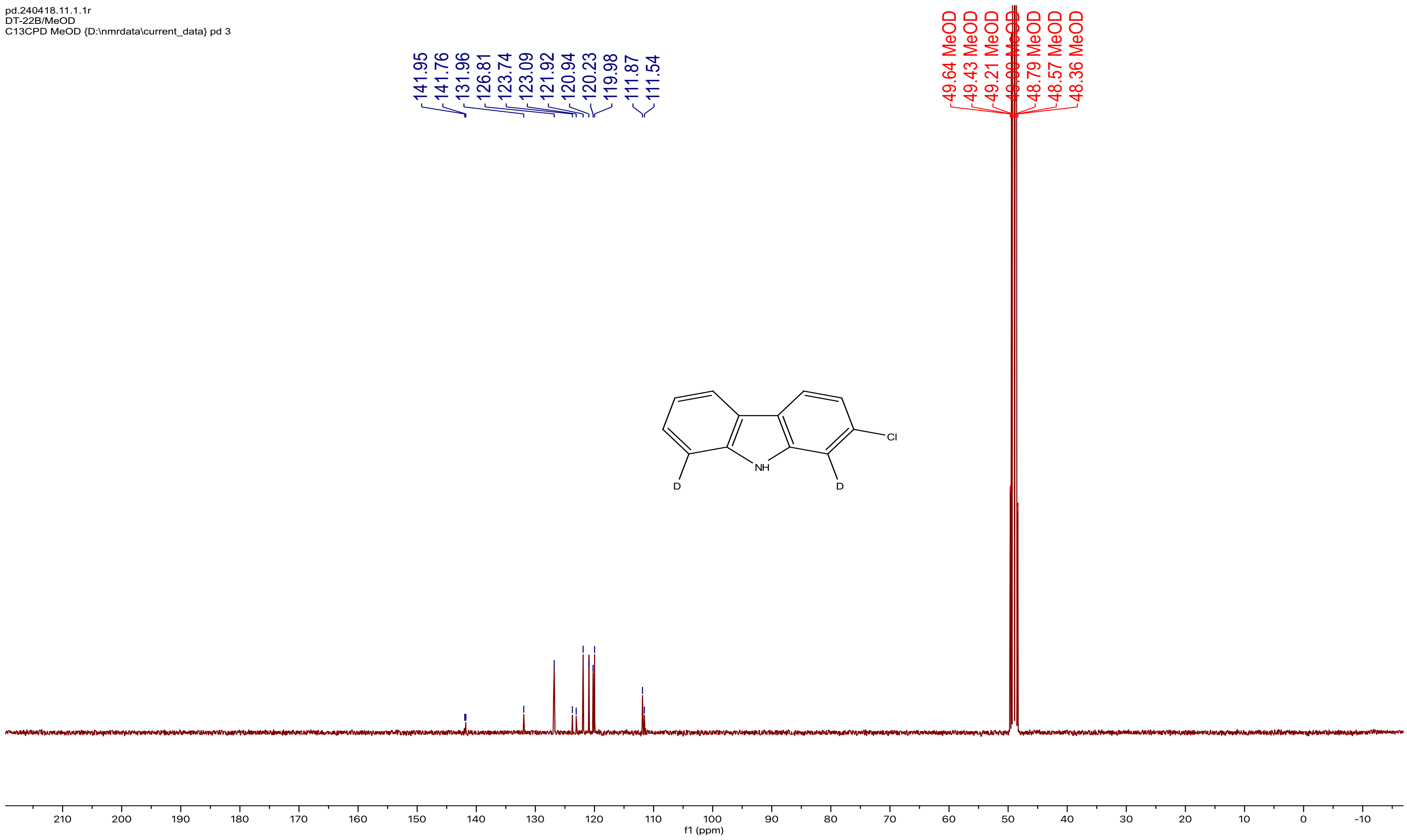

$^{13}C\{^{1}H\}$ NMR (101 MHz, MeOD) of 4bj

3k

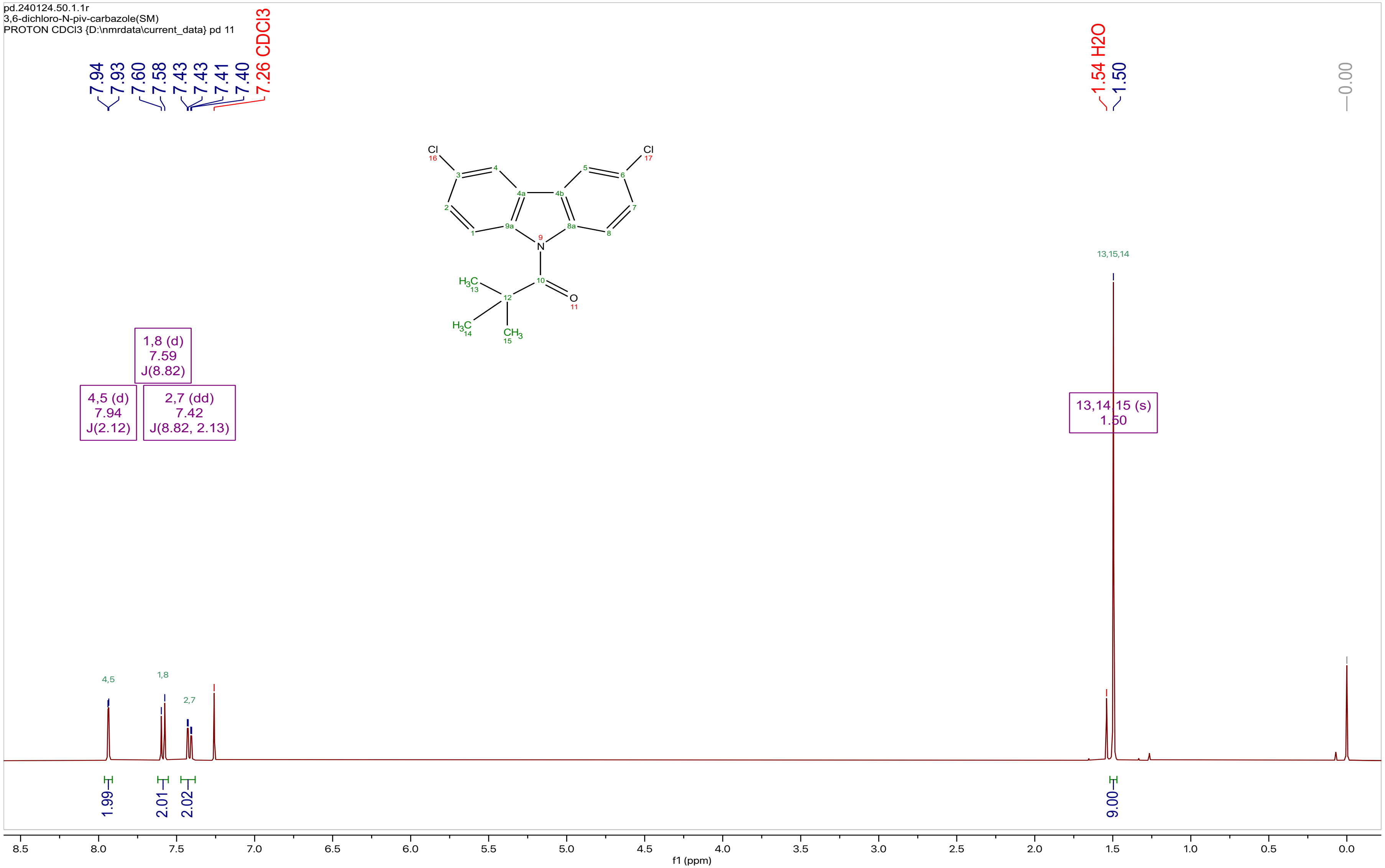

^{1}H NMR (400 MHz, $CDCl_3$) of 3k

pd.240124.51.1.1r
3,6-dichloro-N-piv-carbazole(SM)
C13CPD CDCl3 {D:\nmrdata\current_data} pd 11

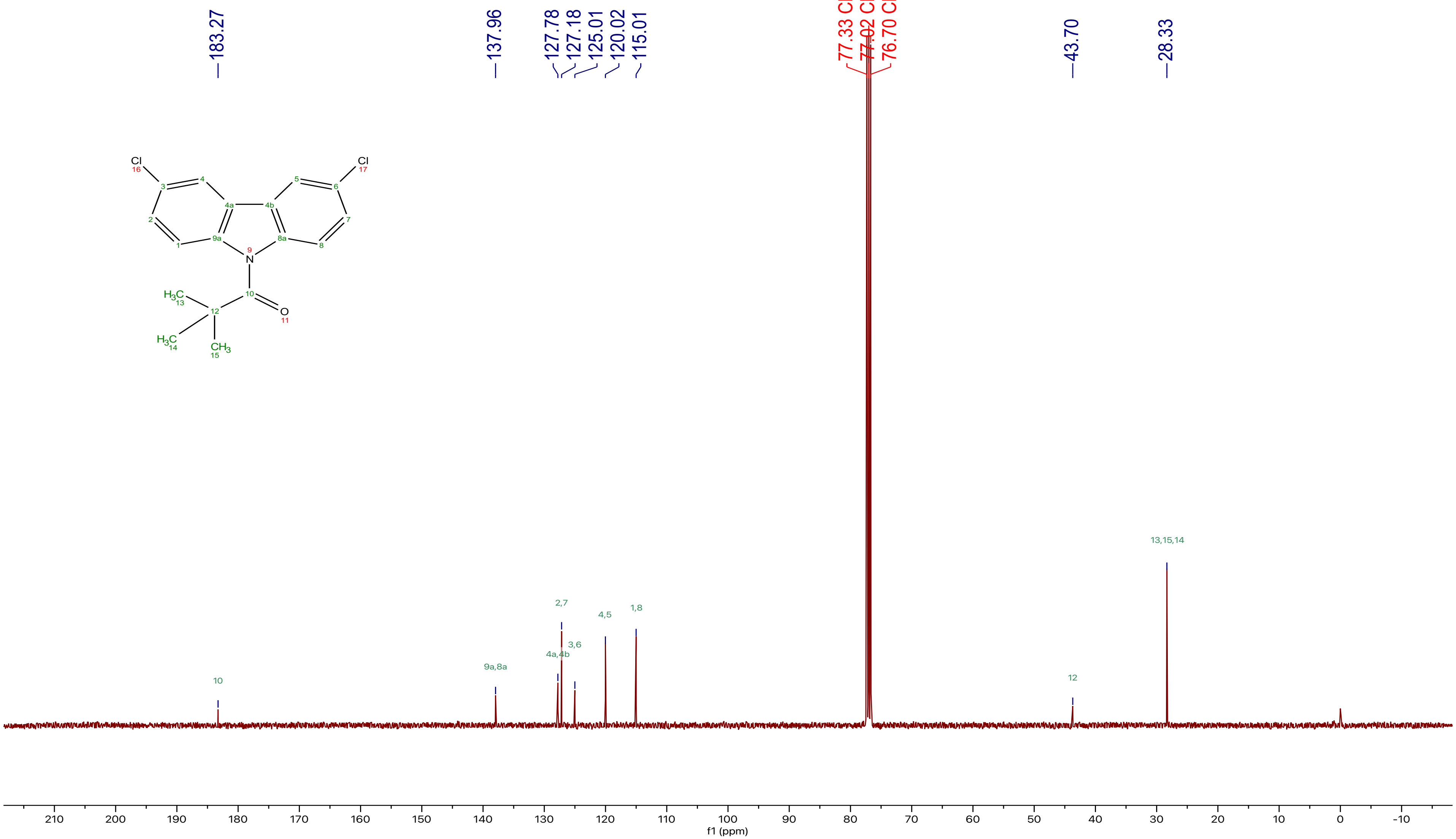

$^{13}C\{^1H\}$ NMR (101 MHz, $CDCl_3$) of 3k

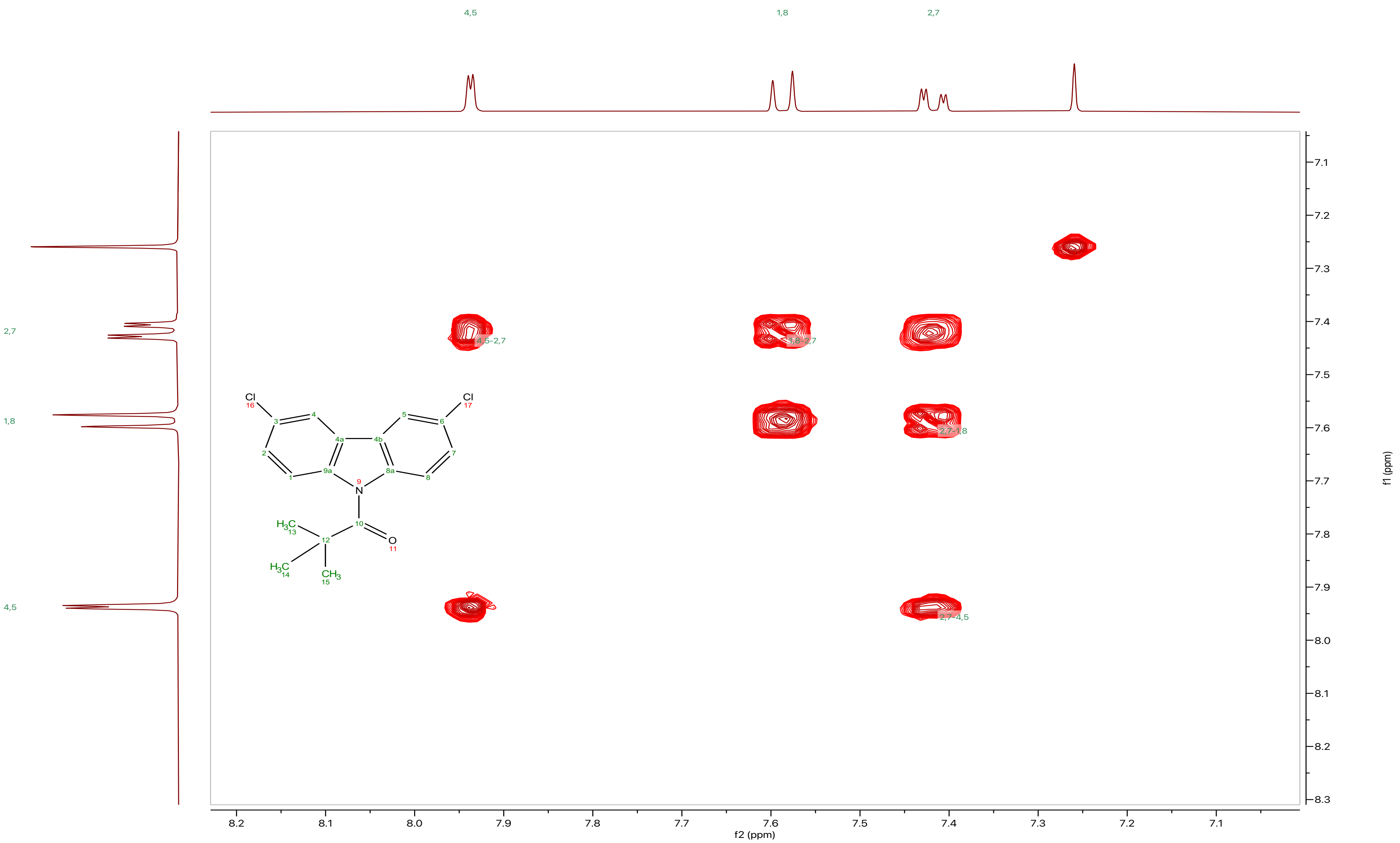

^1H-^1H COSY (400 MHz, $CDCl_3$) of 3k

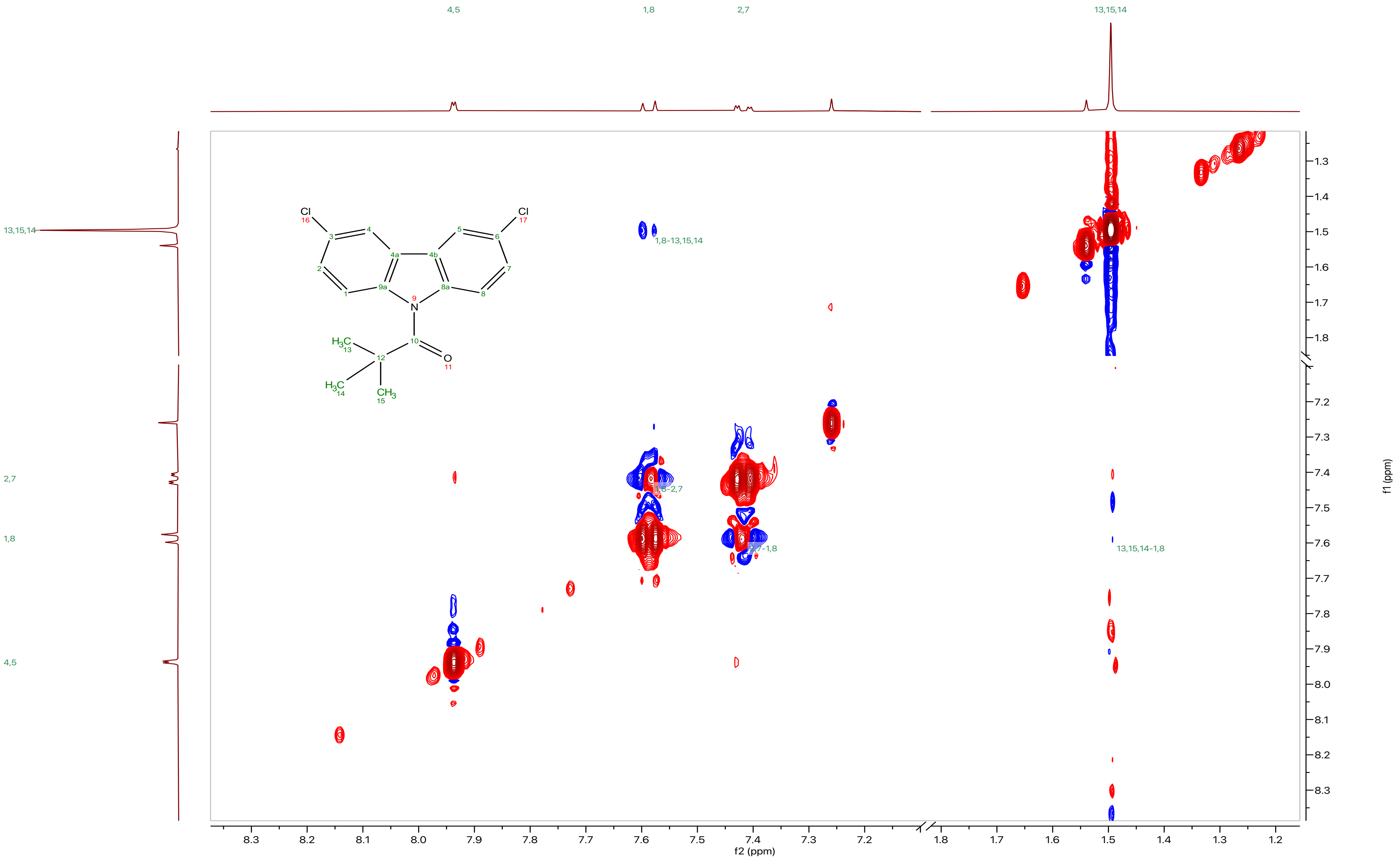

^{1}H-^{1}H NOESY (400 MHz, $CDCl_3$) of 3k

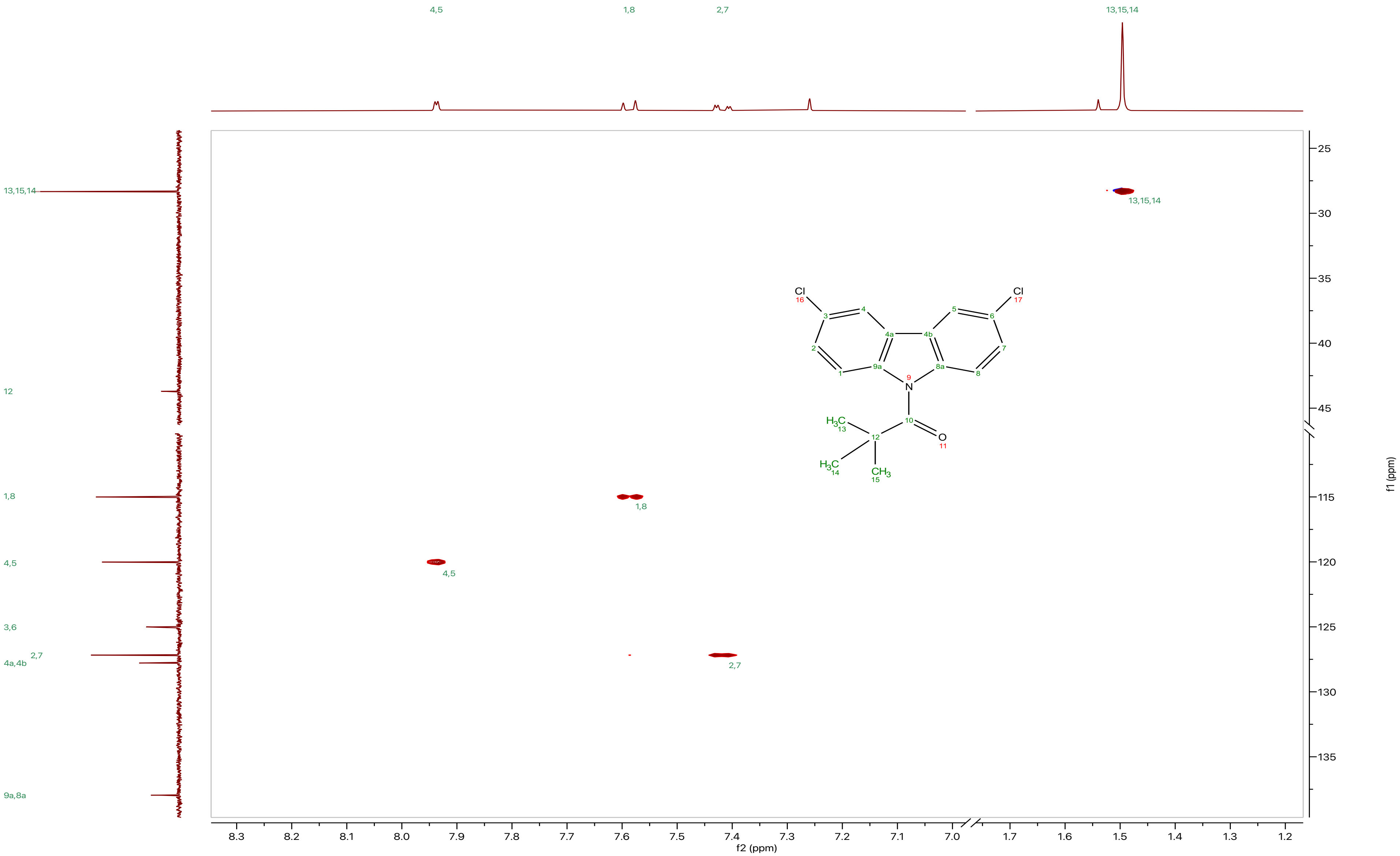

^{1}H-^{13}C{^{1}H} HSQC NMR (400/101 MHz, $CDCl_3$) of 3k

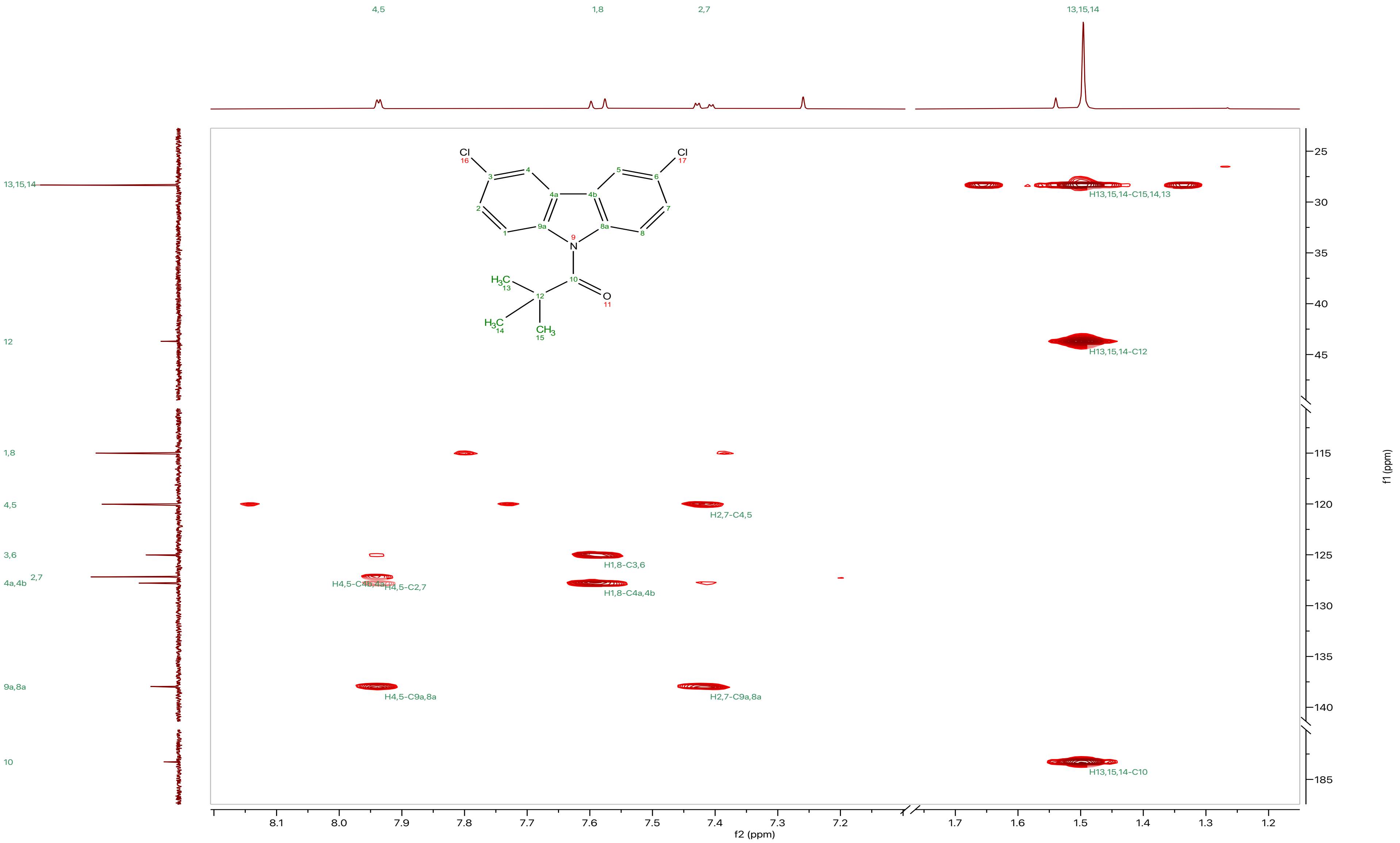

^{1}H-^{13}C{^{1}H} HMBC NMR (400/101 MHz, $CDCl_3$) of 3k

3k'

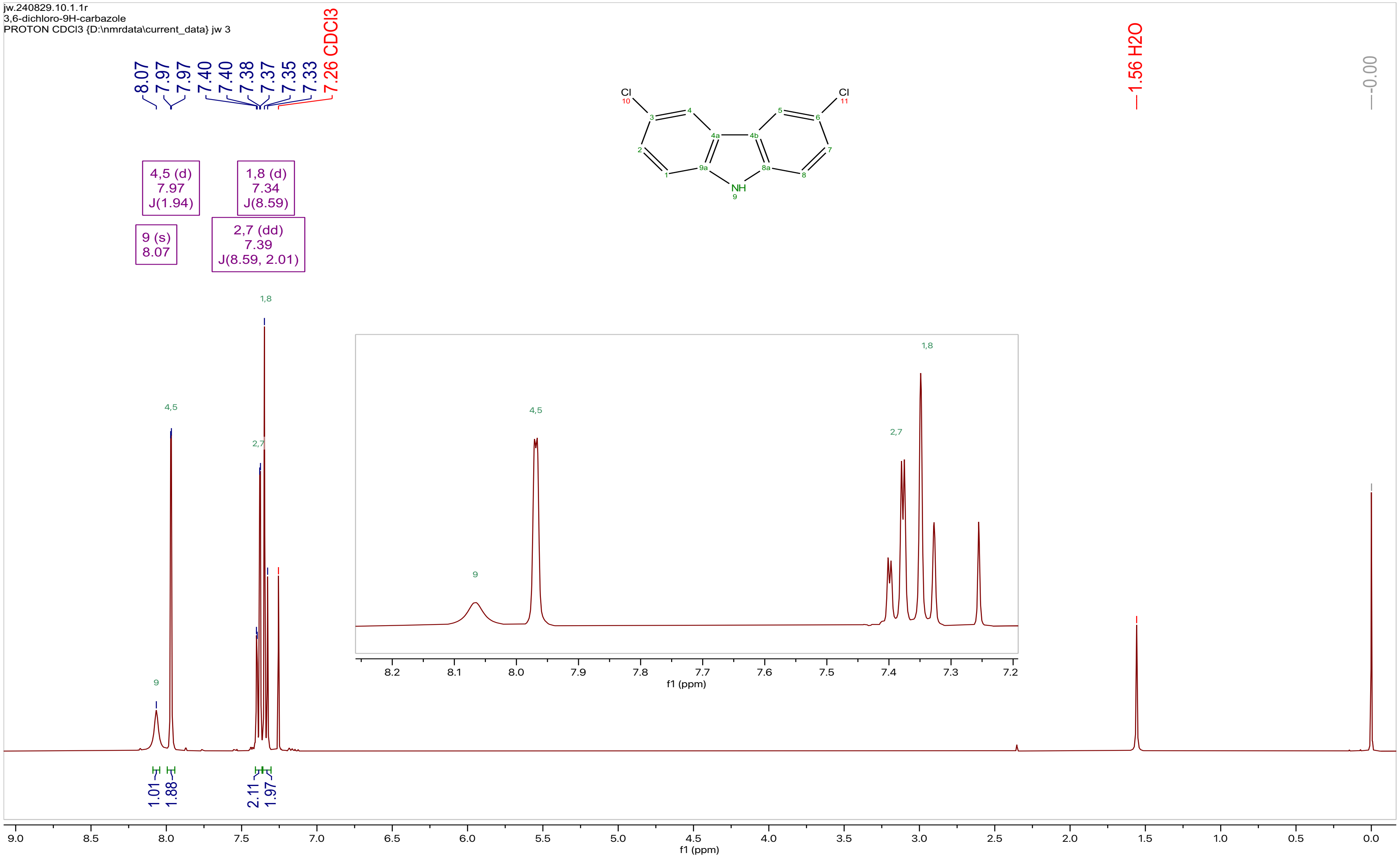

¹H NMR (400 MHz, $CDCl_3$) of 3k'

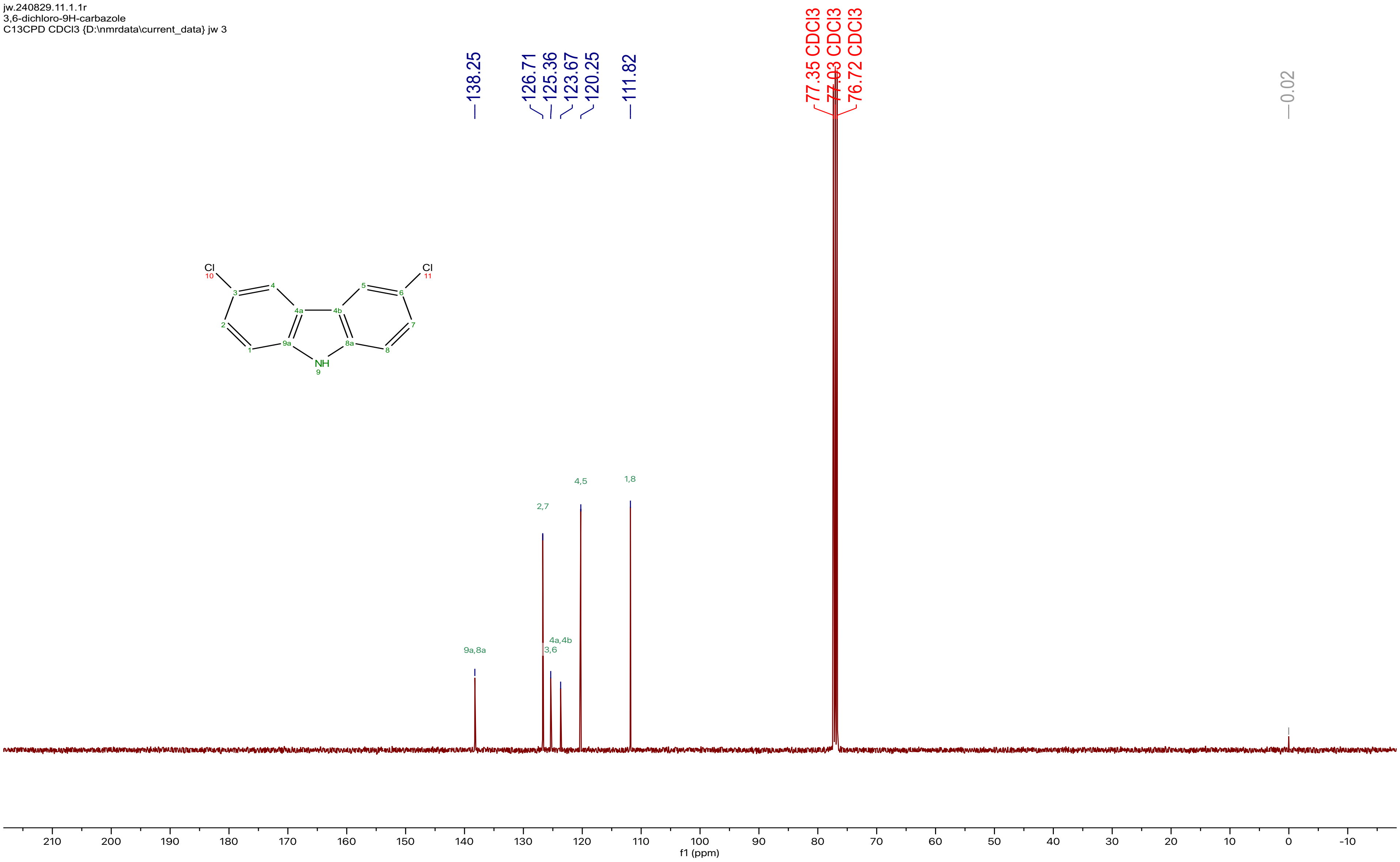

$^{13}C\{^1H\}$ NMR (101 MHz, $CDCl_3$) of 3k'

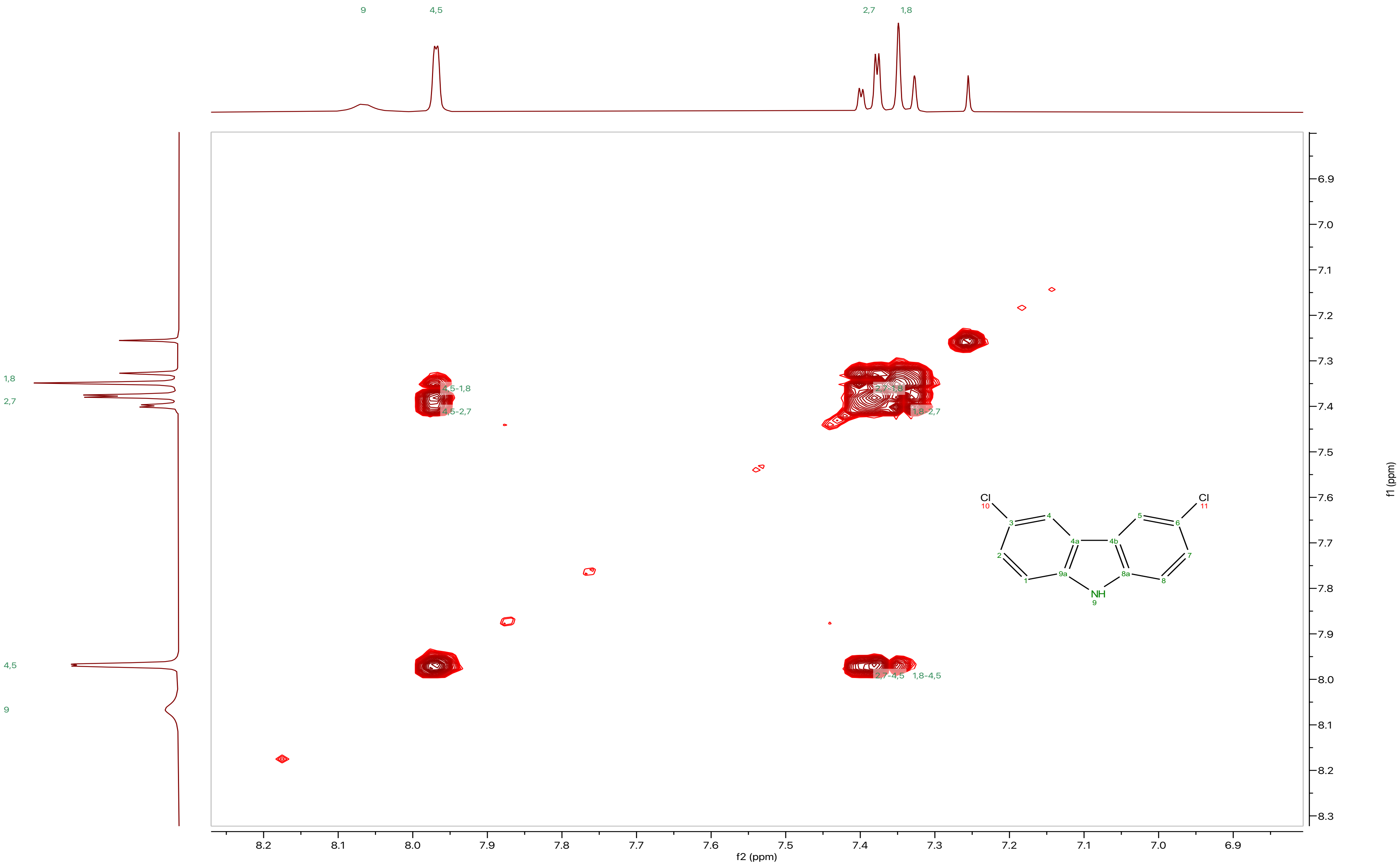

^1H-^1H COSY (400 MHz, $CDCl_3$) of 3k'

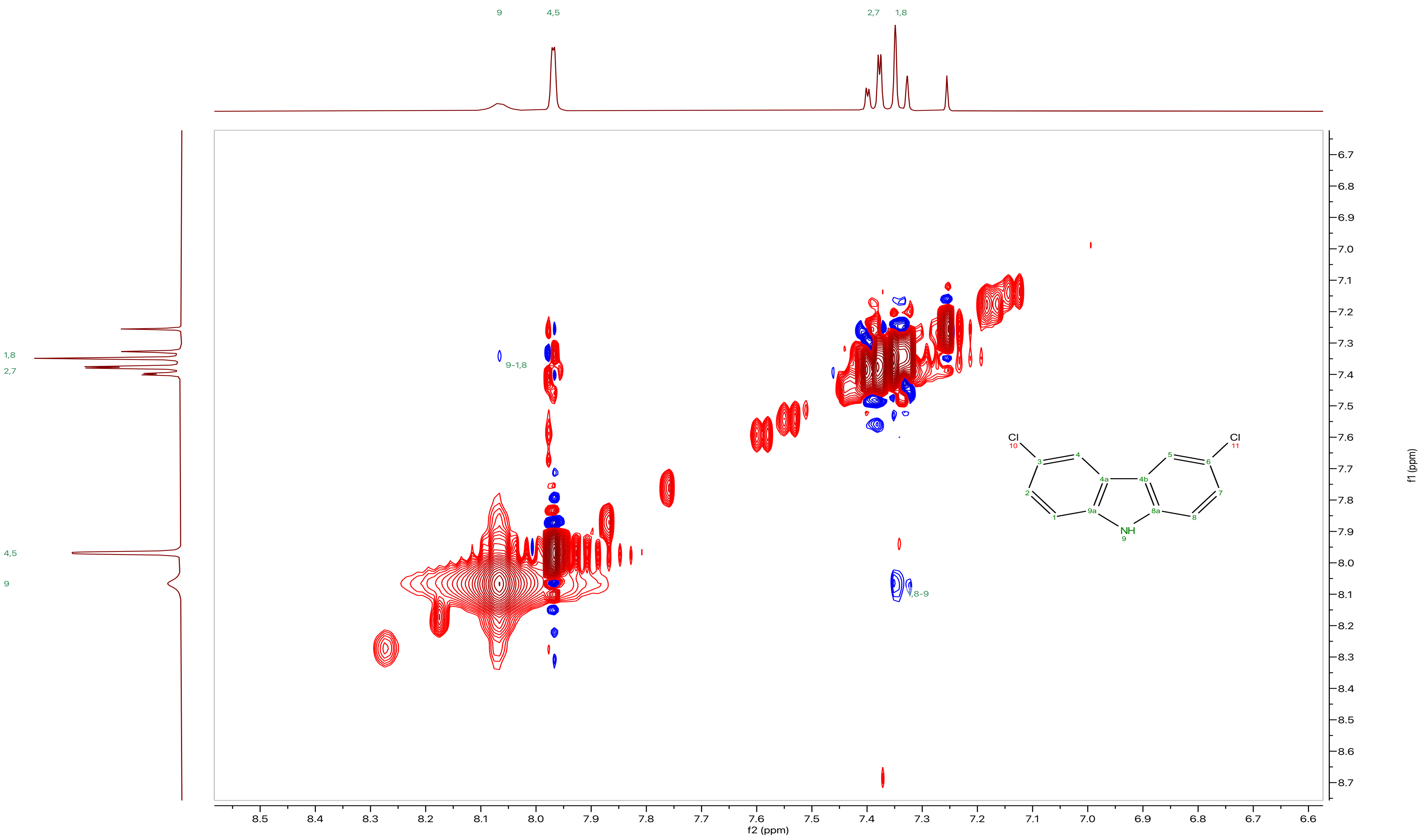

^{1}H-^{1}H NOESY (400 MHz, $CDCl_3$) of 3k'

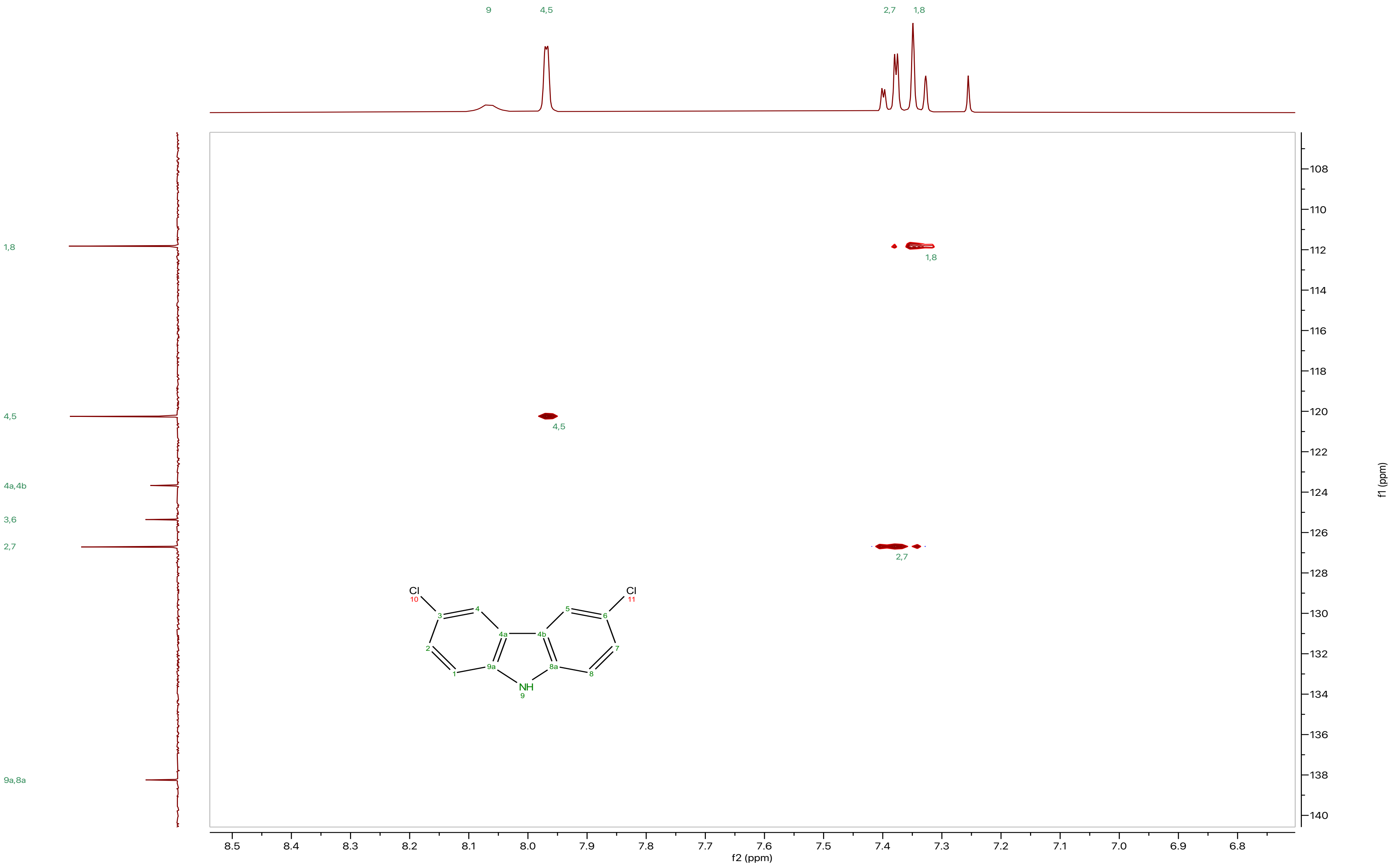

^{1}H-^{13}C{^{1}H} HSQC NMR (400/101 MHz, $CDCl_3$) of 3k'

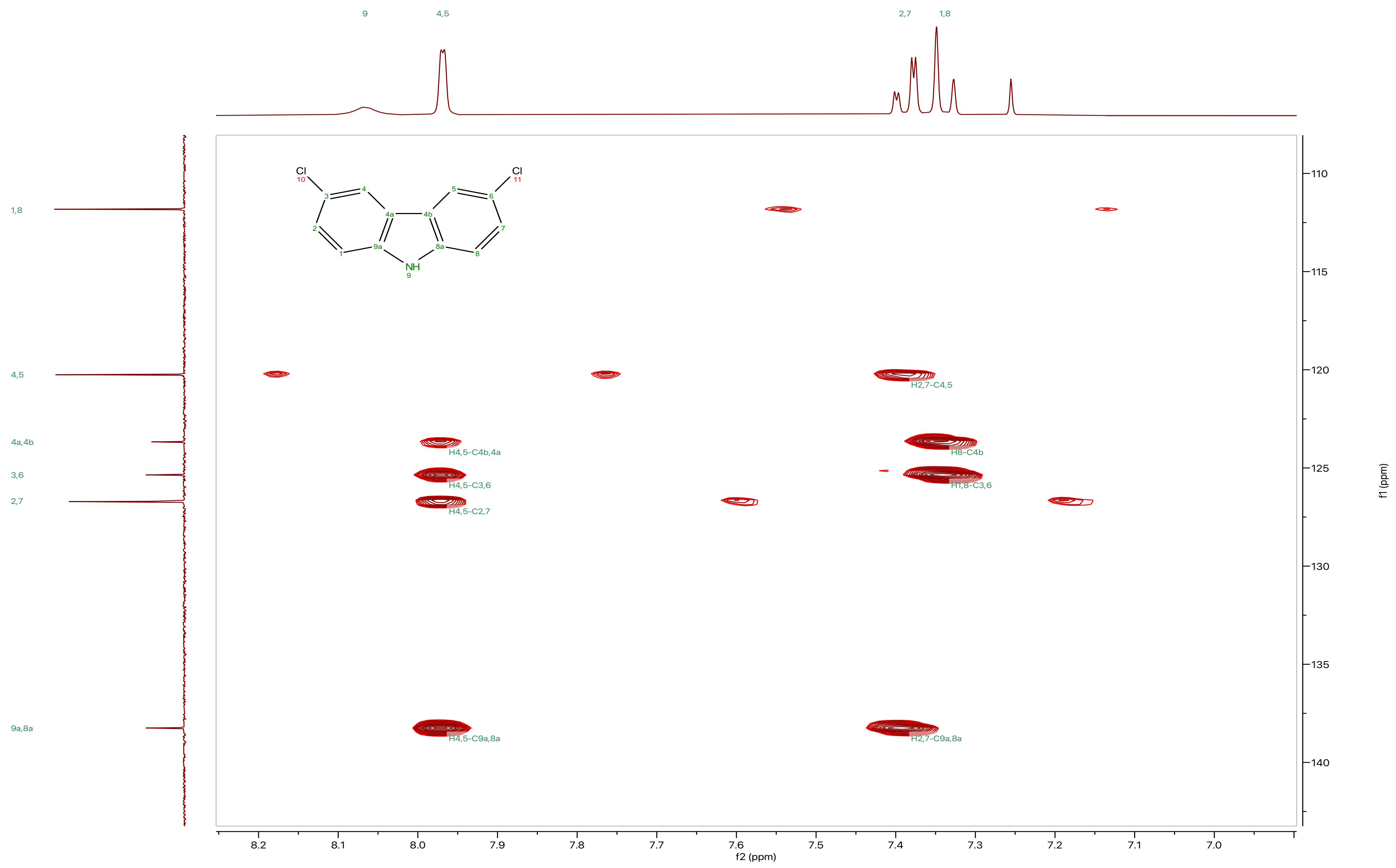

^{1}H-$^{13}C\{^{1}H\}$ HMBC NMR (400/101 MHz, $CDCl_3$) of 3k'

4bk

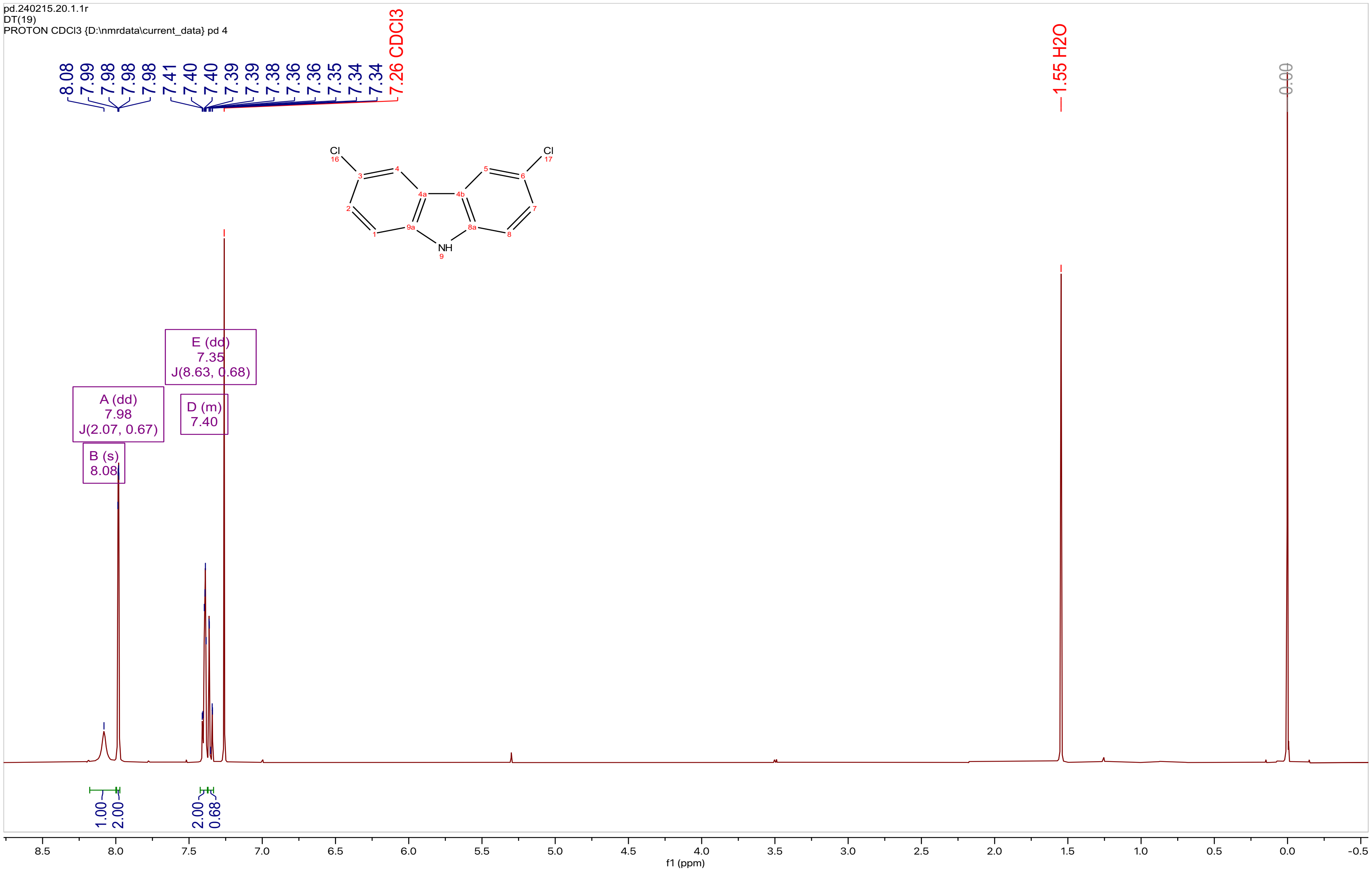

^{1}H NMR (400 MHz, $CDCl_3$) of 4bk

pd.240215.21.1.1r
DT(19)
C13CPD CDCl3 {D:\nmrdata\current_data} pd 4

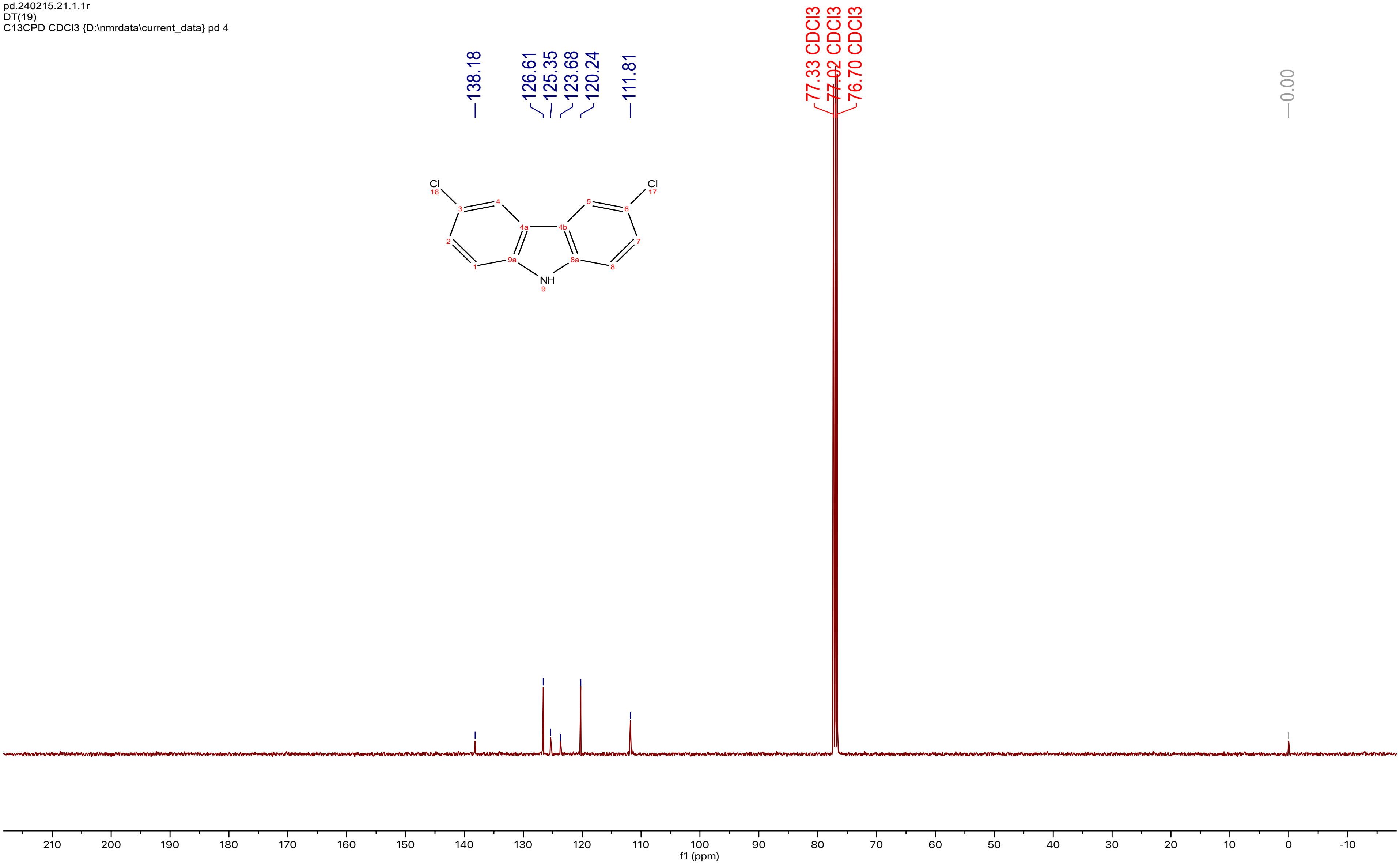

$^{13}C\{^{1}H\}$ NMR (101 MHz, $CDCl_3$) of 4bk

3l

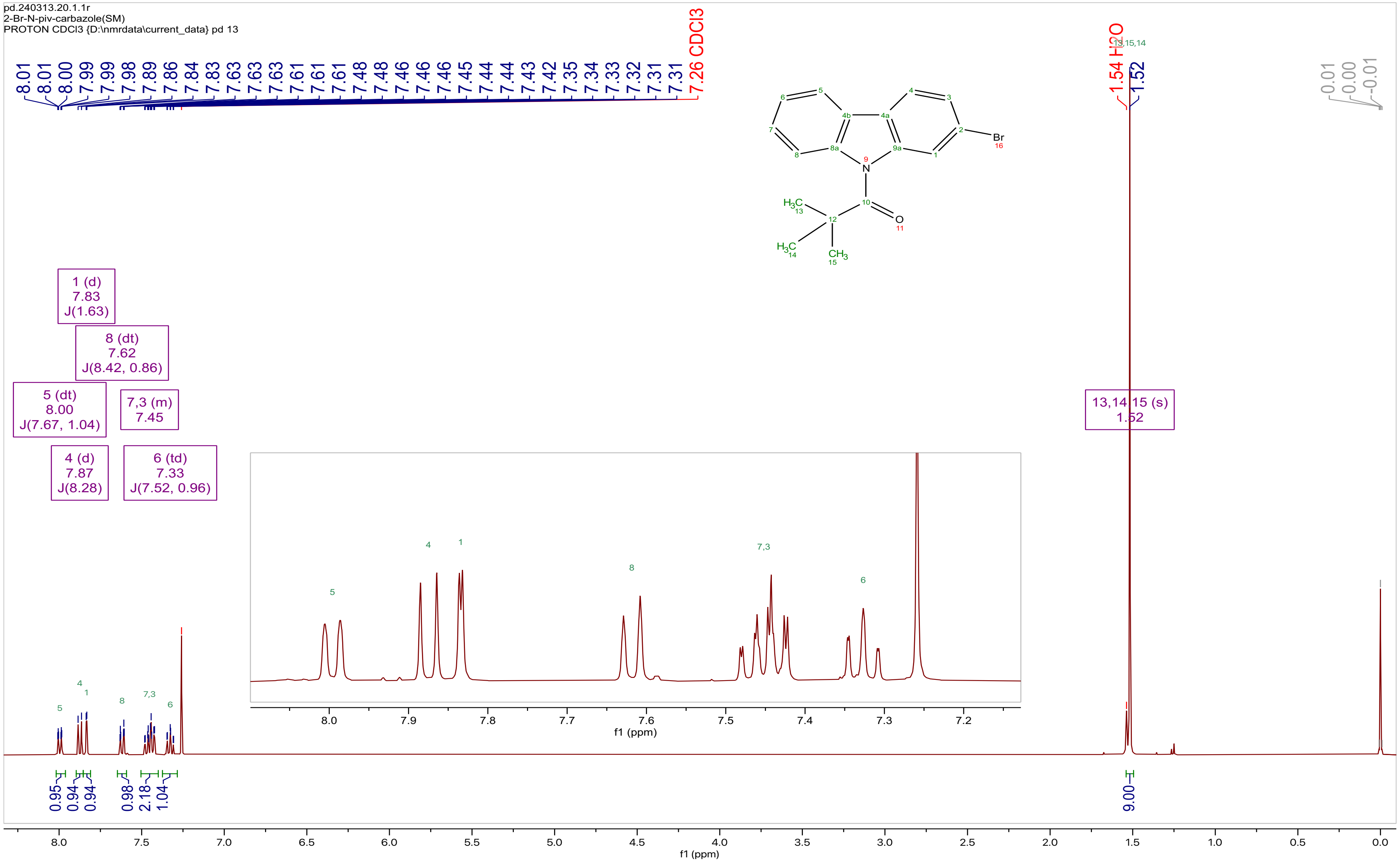

^{1}H NMR (400 MHz, $CDCl_3$) of 3l

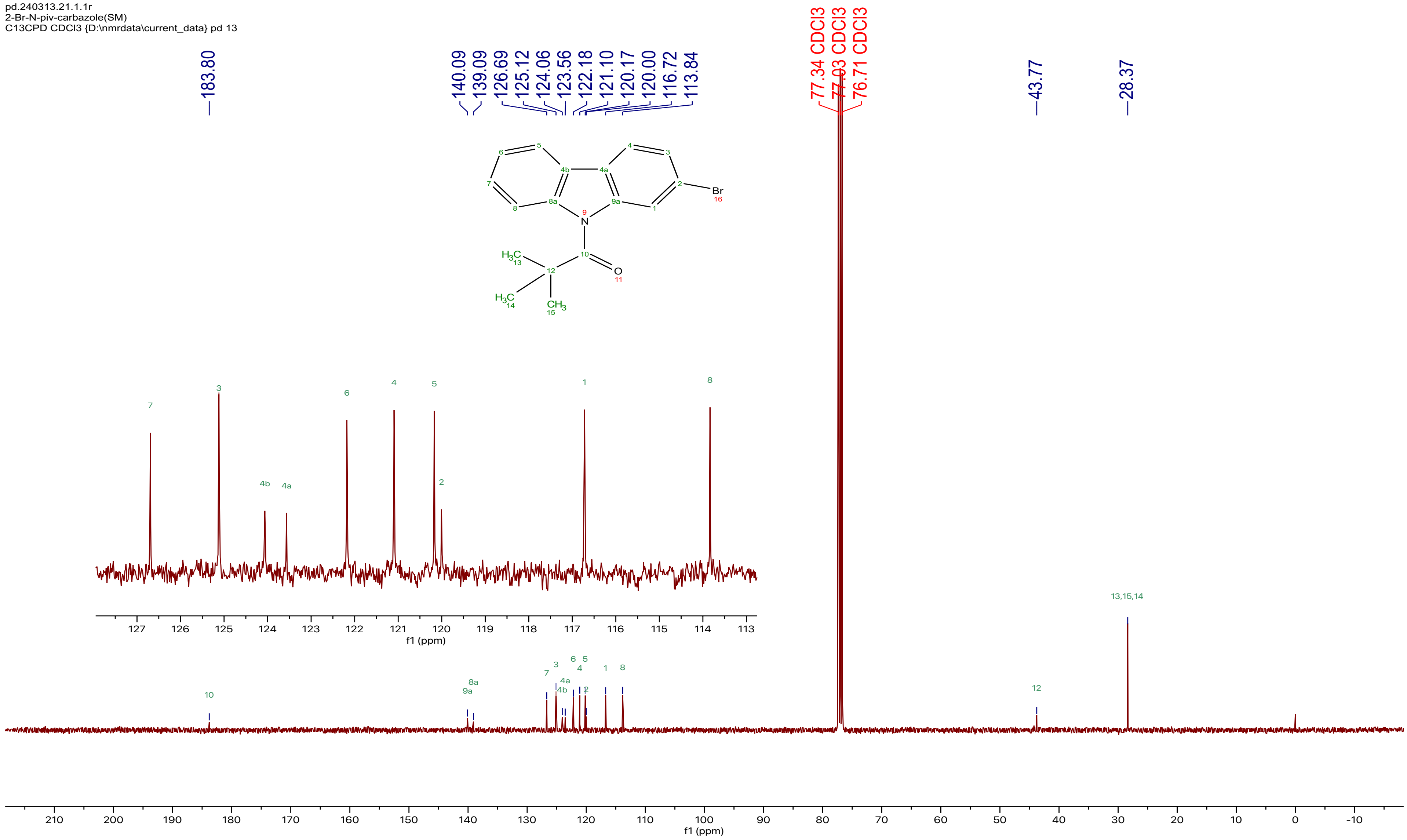

$^{13}C\{^{1}H\}$ NMR (101 MHz, $CDCl_3$) of 3l

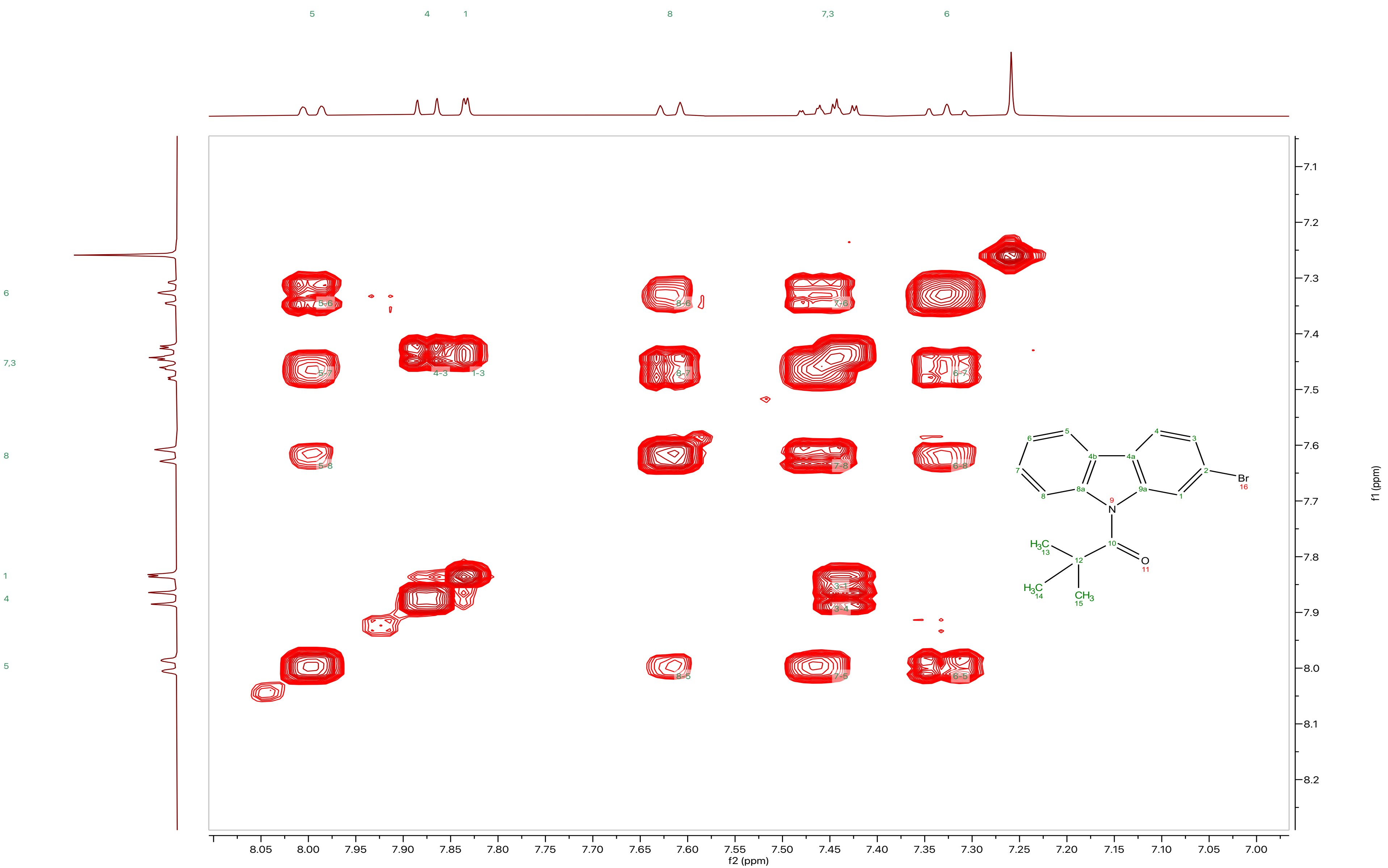

^{1}H-^{1}H COSY (400 MHz, $CDCl_3$) of 3l

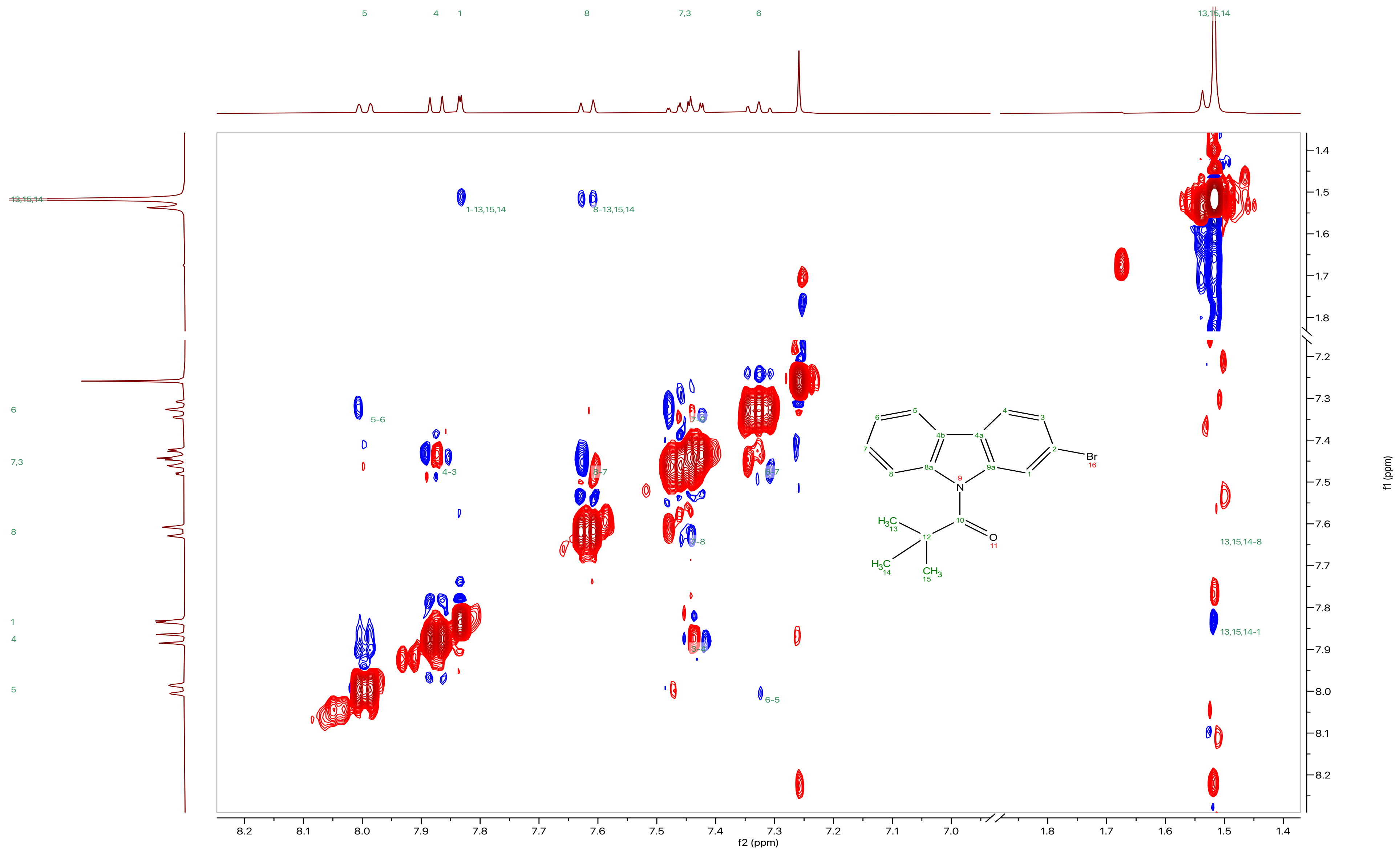

^{1}H-^{1}H NOESY (400 MHz, $CDCl_3$) of 3l

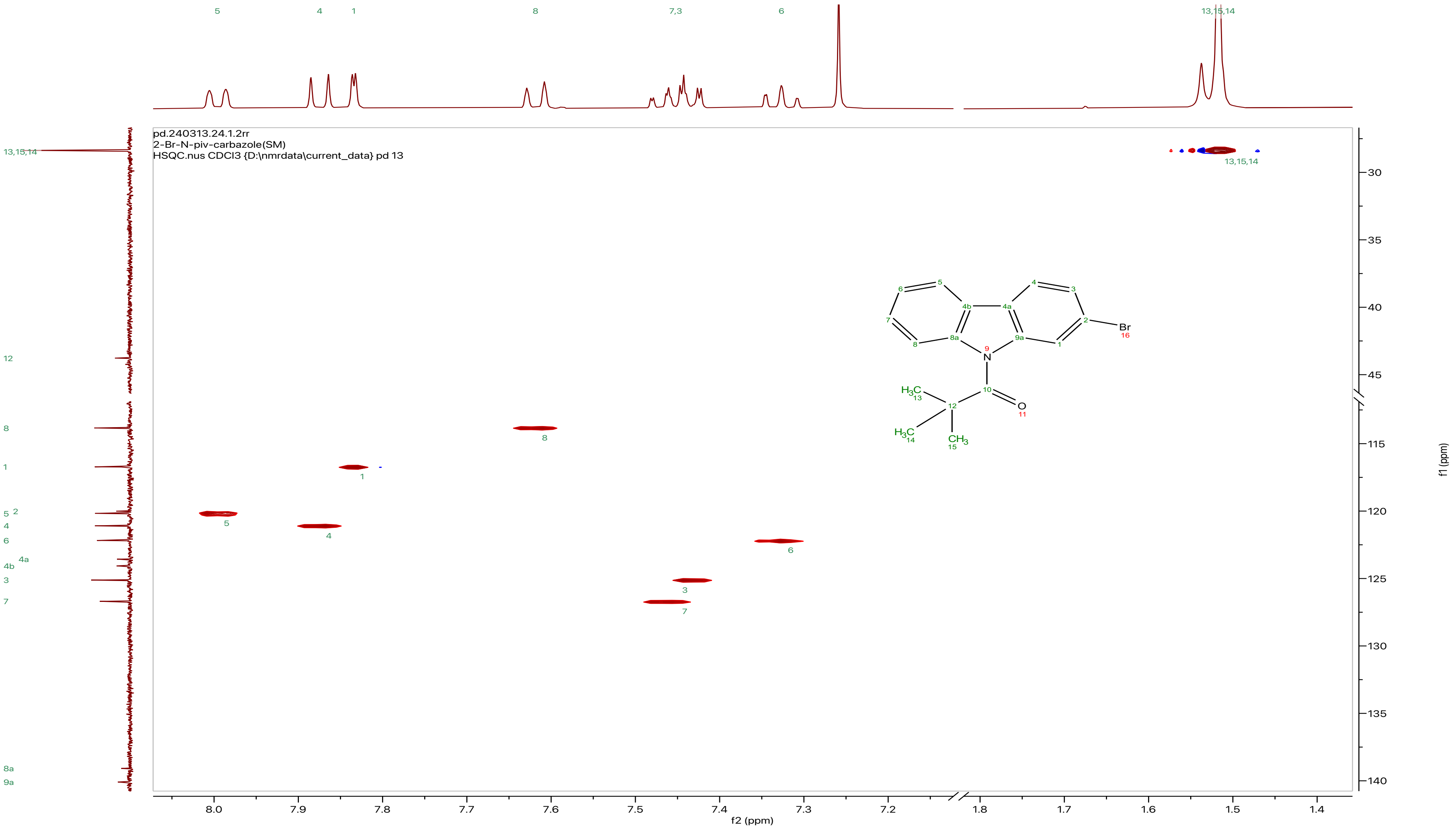

^{1}H-$^{13}C\{^{1}H\}$ HSQC NMR (400/101 MHz, $CDCl_3$) of 3l

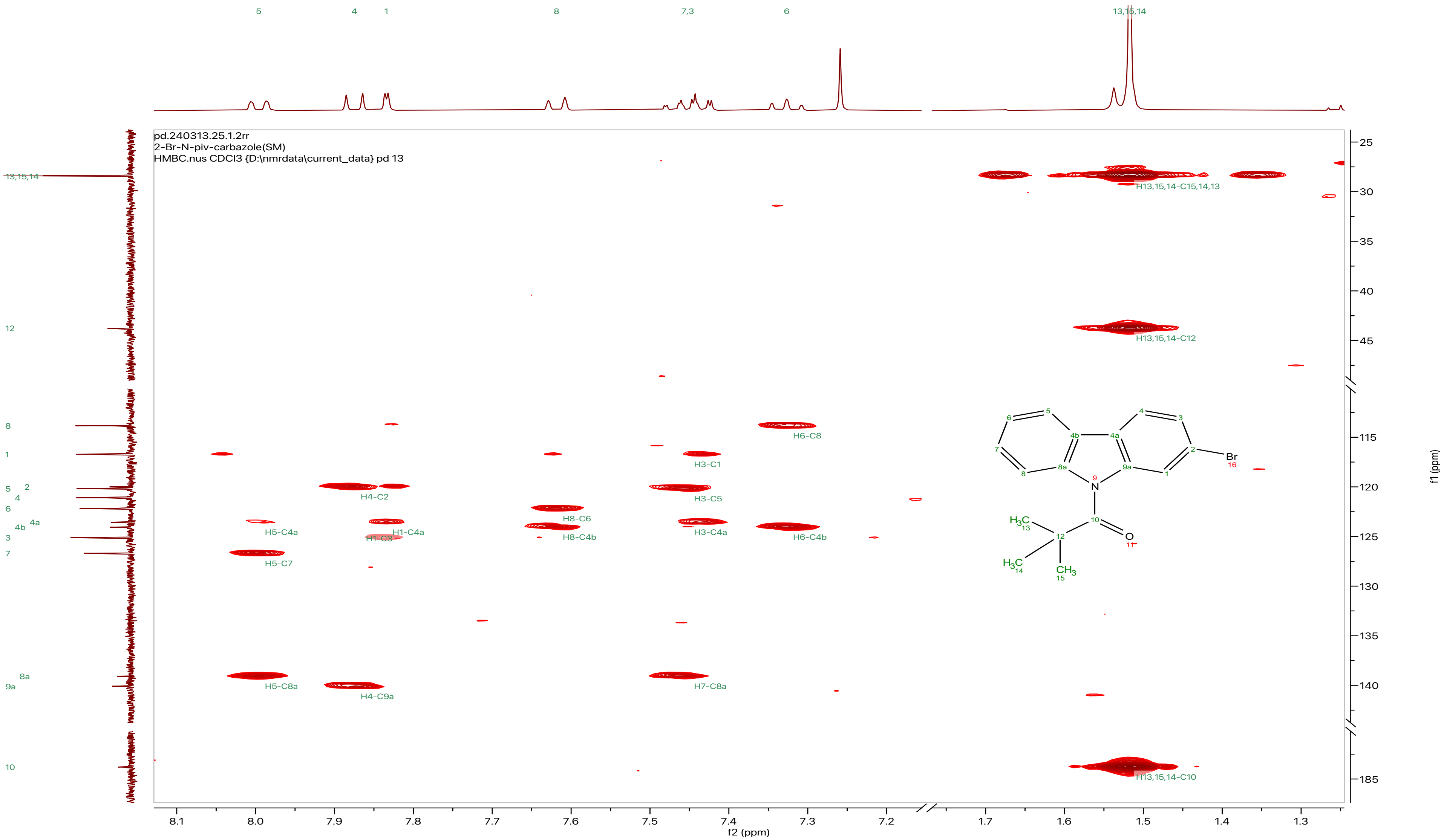

^{1}H-^{13}C{^{1}H} HMBC NMR (400/101 MHz, CDCl$_3$) of 3l

3l’

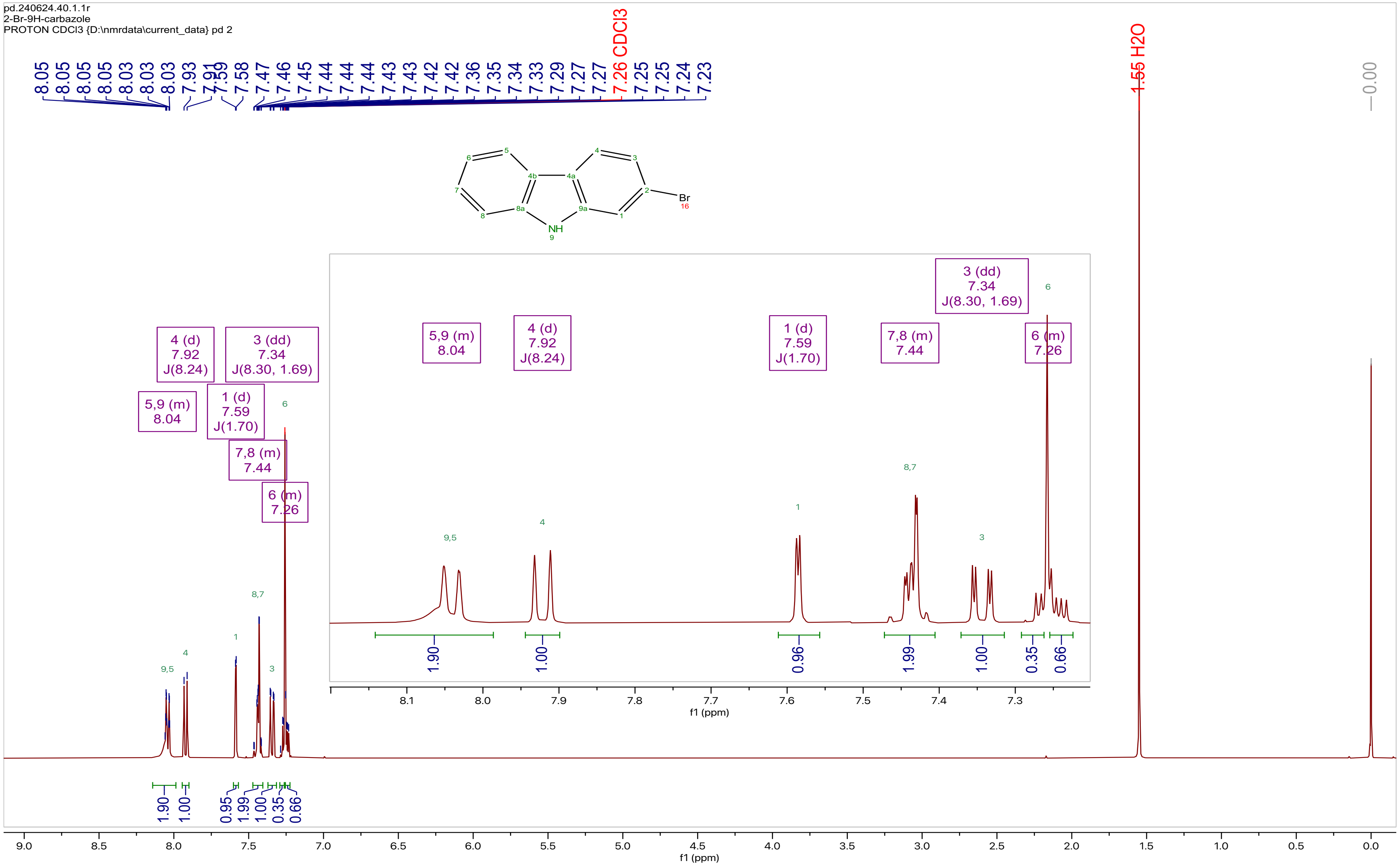

^{1}H NMR (400 MHz, $CDCl_3$) of 3l’

pd.240624.41.1.1r
2-Br-9H-carbazole
C13CPD CDCl3 {D:\nmrdata\current_data} pd 2

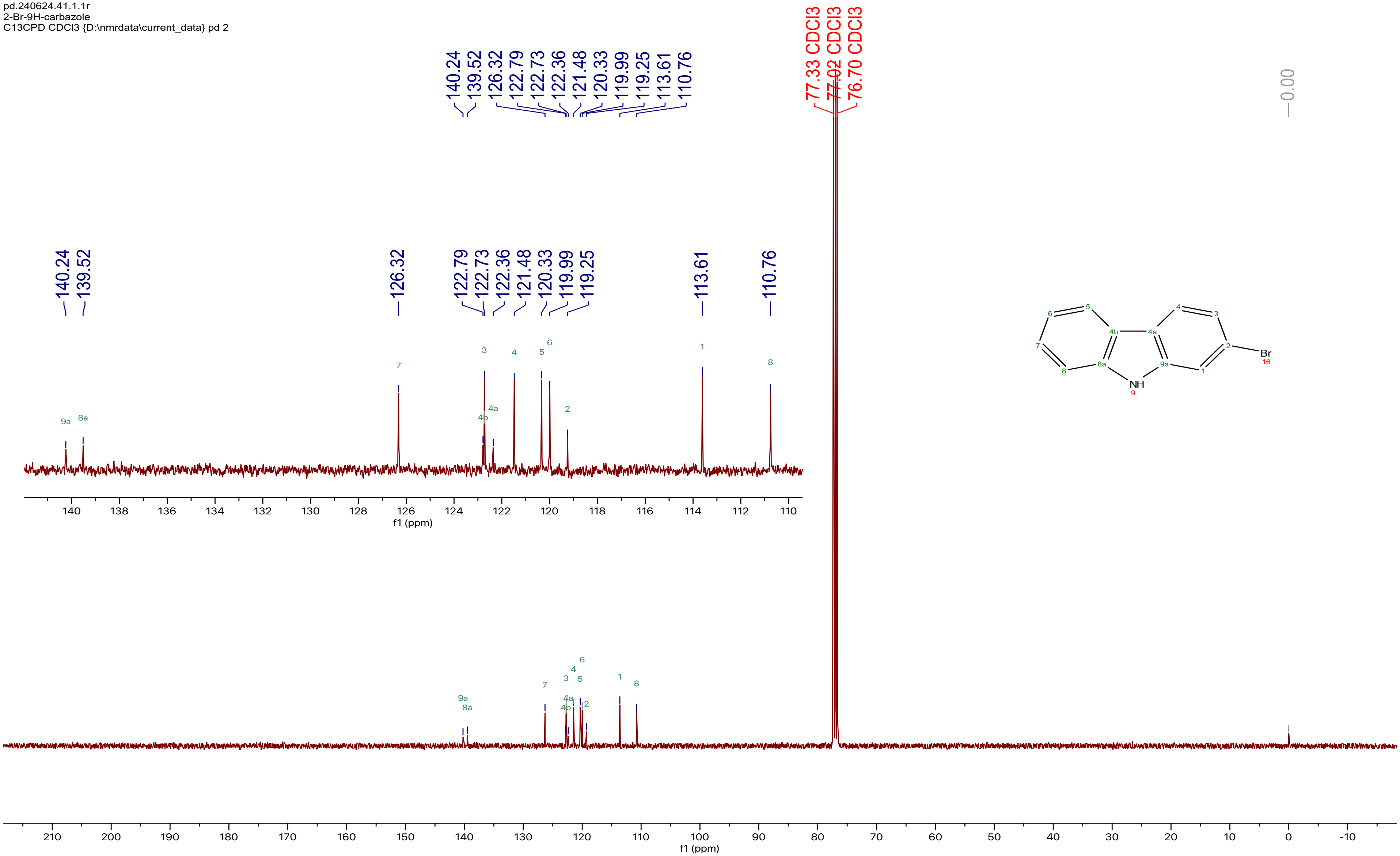

$^{13}C\{^1H\}$ NMR (101 MHz, $CDCl_3$) of 3l'

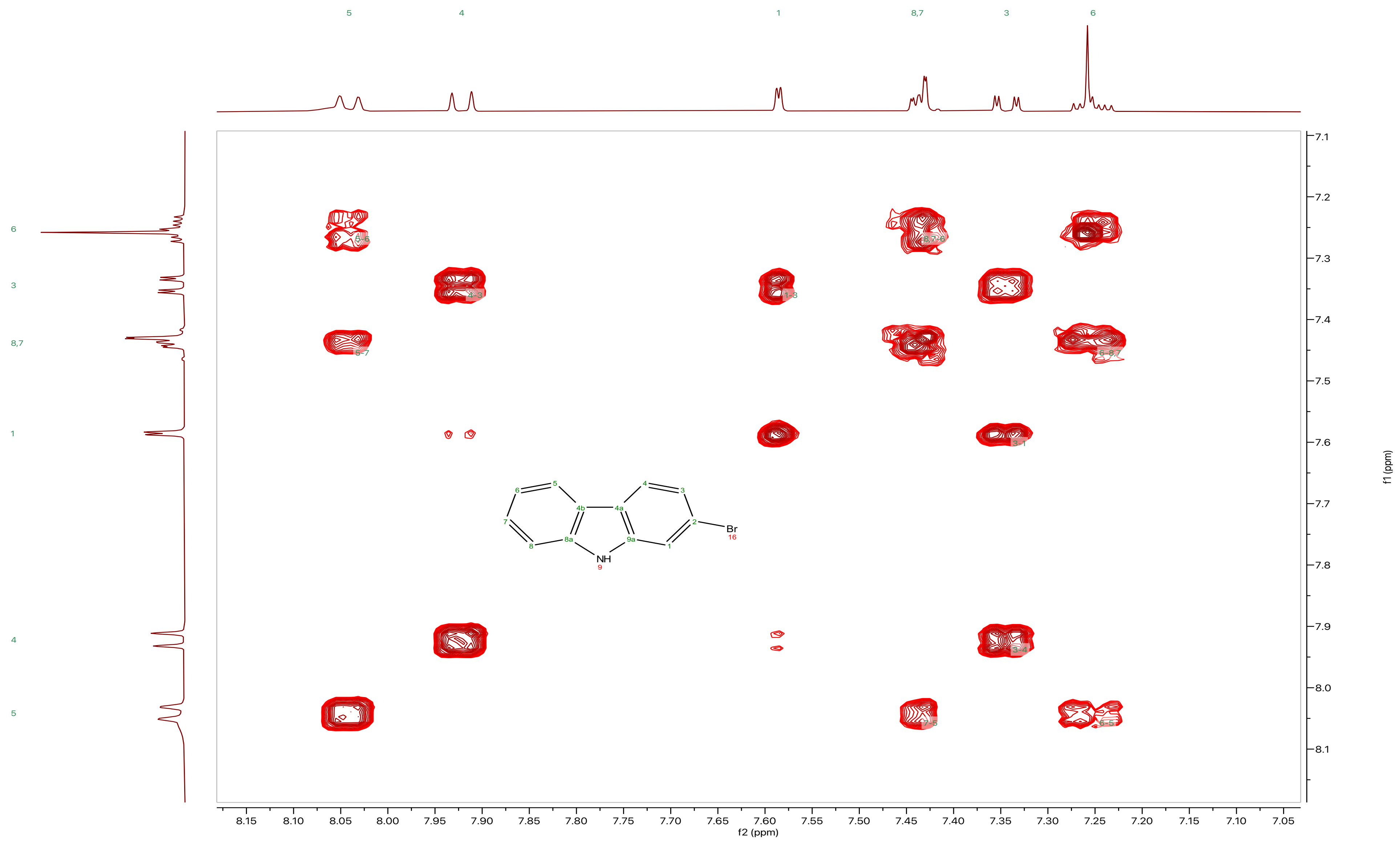

^1H-^1H COSY (400 MHz, $CDCl_3$) of 3l’

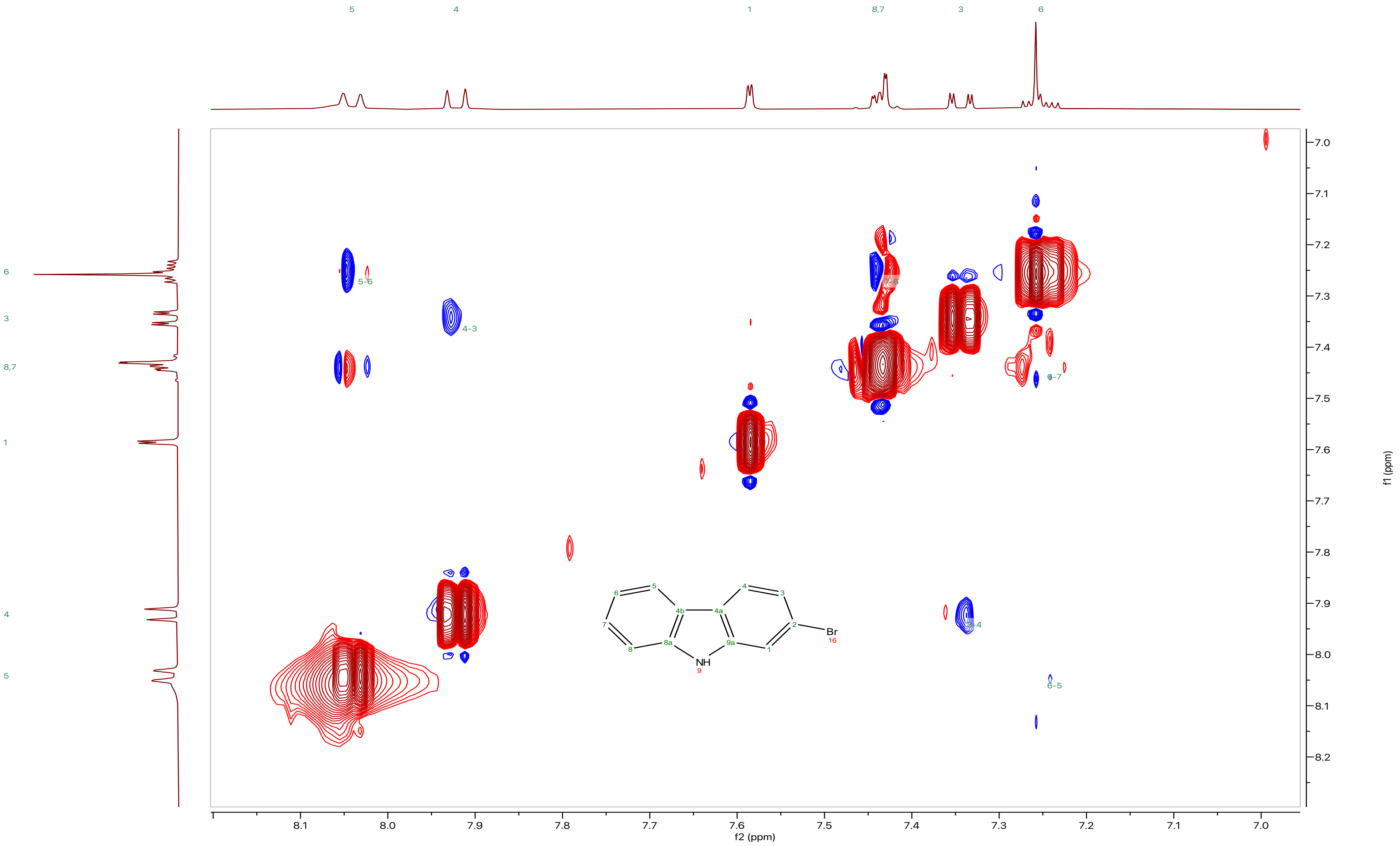

^1H-^1H NOESY (400 MHz, $CDCl_3$) of 3l'

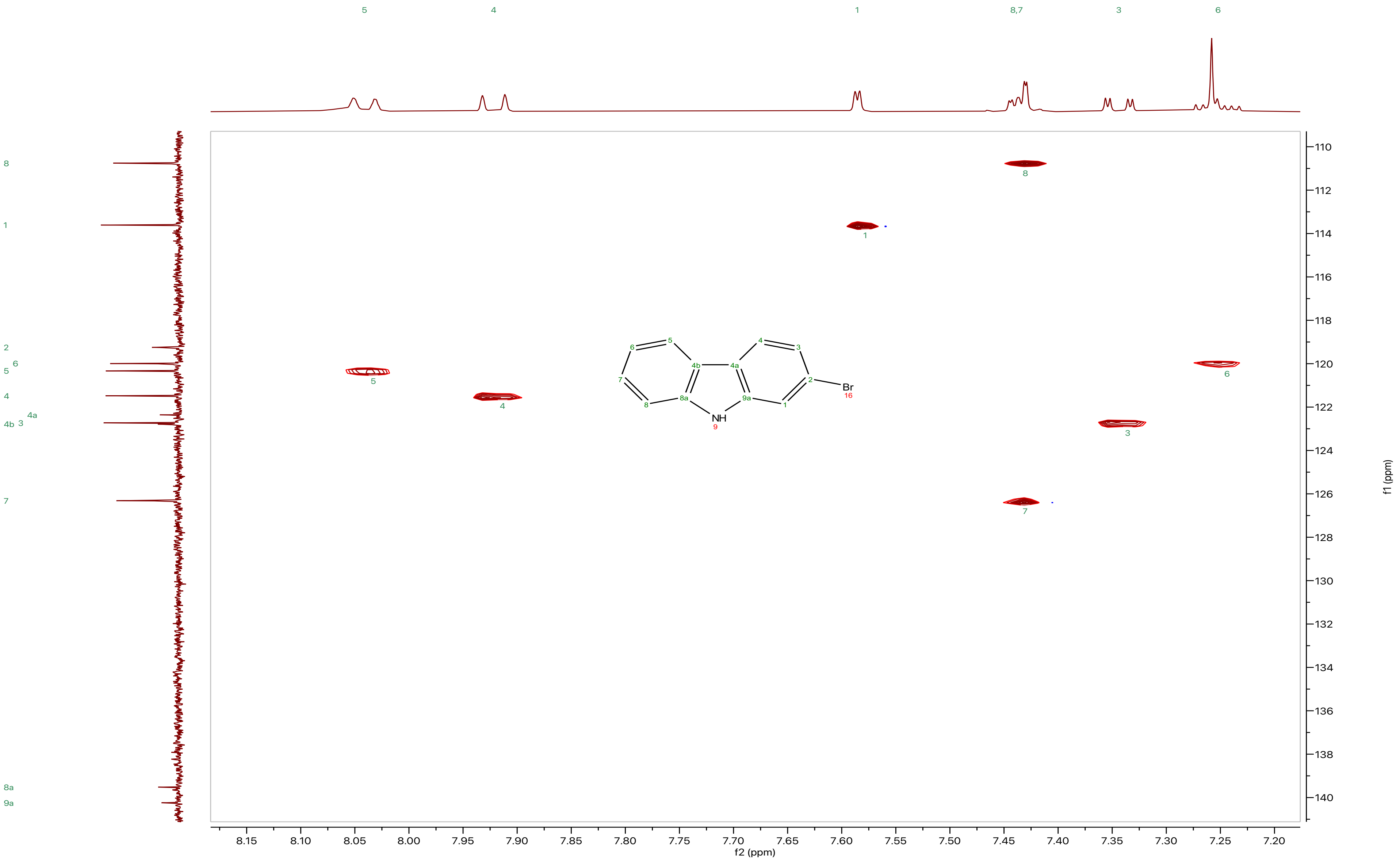

^{1}H-^{13}C{^{1}H} HSQC NMR (400/101 MHz, CDCl$_3$) of 3l'

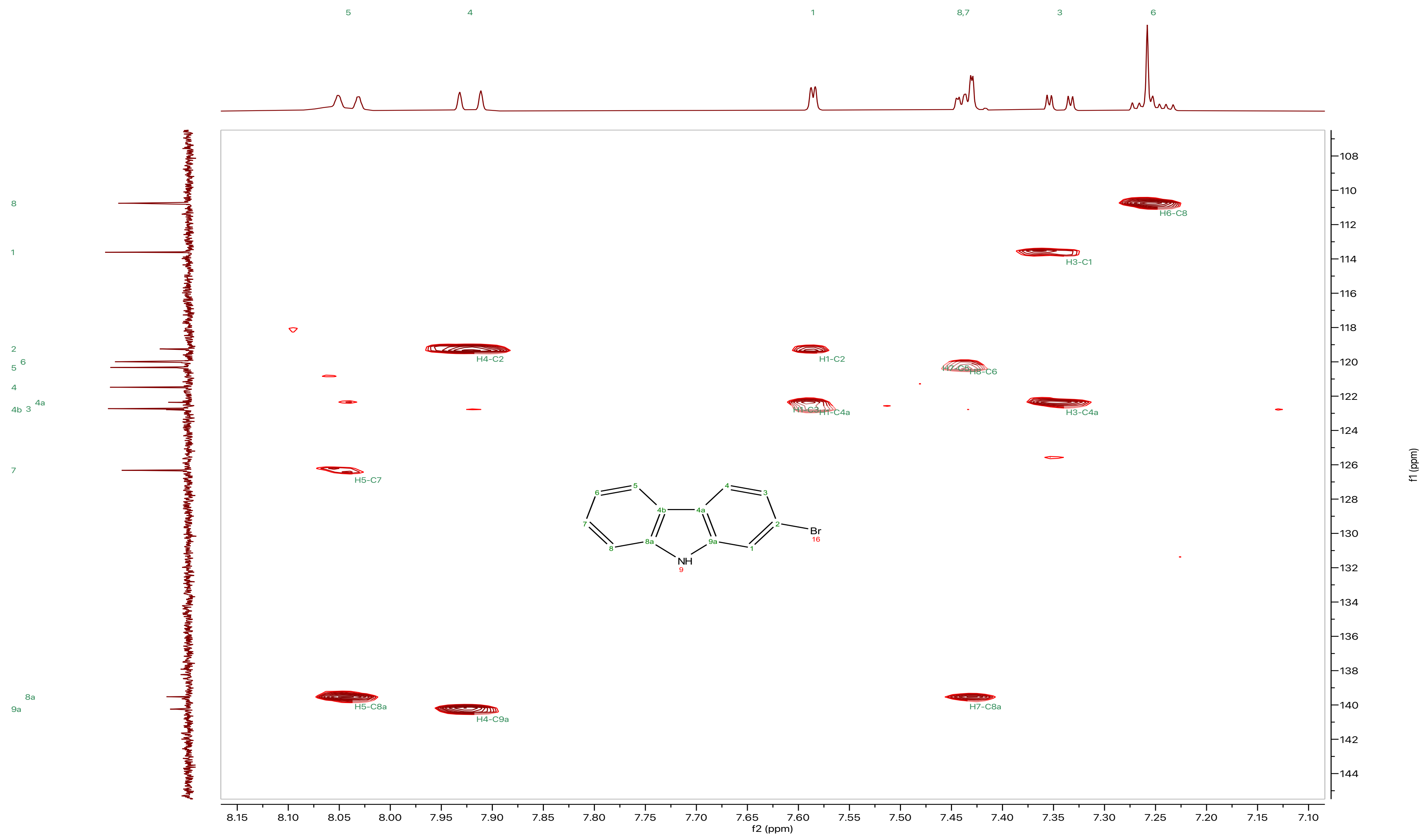

^{1}H-$^{13}C\{^{1}H\}$ HMBC NMR (400/101 MHz, $CDCl_3$) of 3l'

4bl

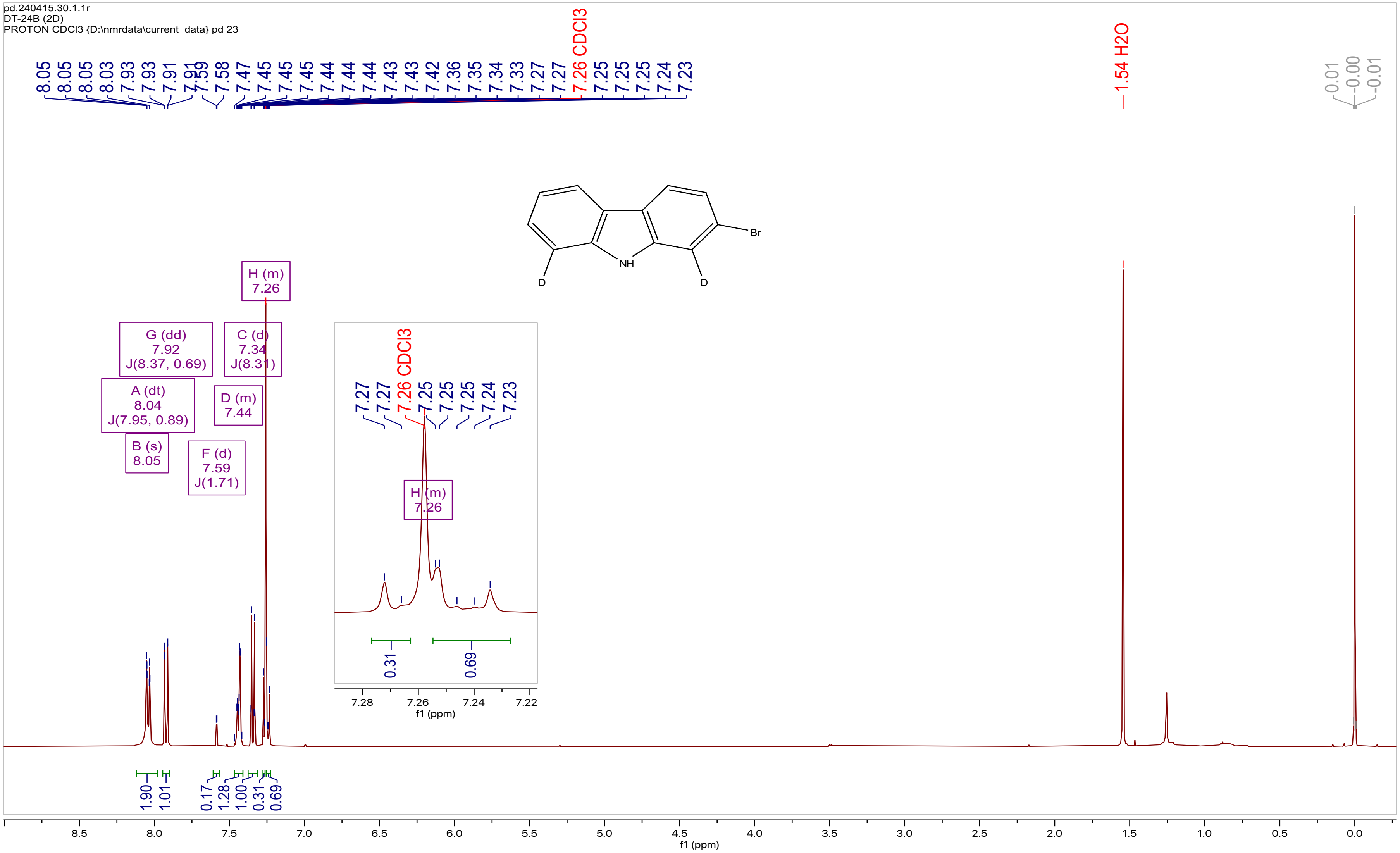

^{1}H NMR (400 MHz, $CDCl_3$) of 4bl

pd.240415.31.1.1r
DT-24B (2D)
C13CPD CDCl3 {D:\nmrdata\current_data} pd 23

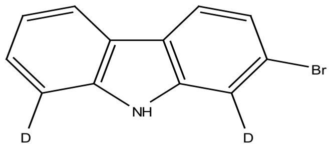

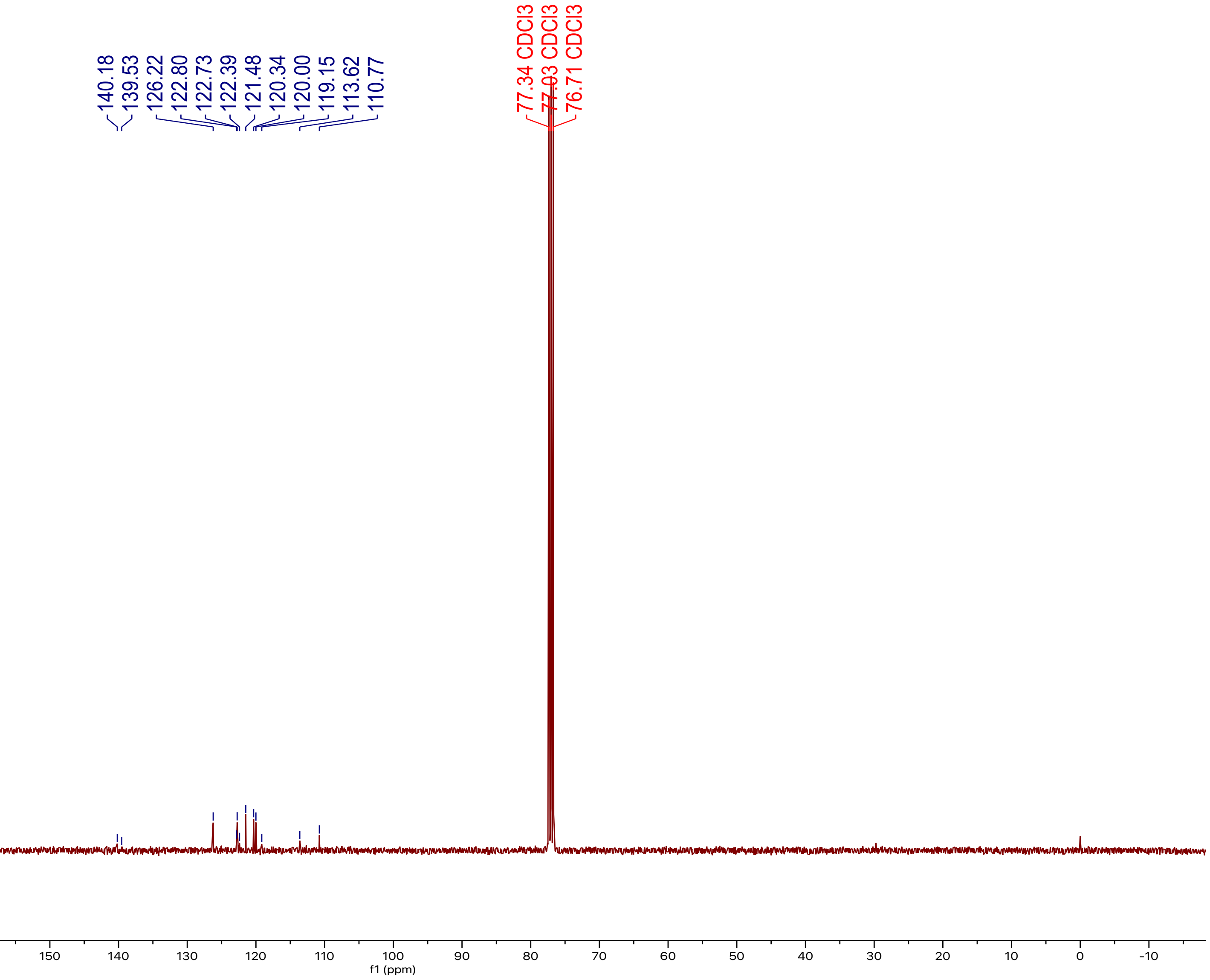

$^{13}C\{^{1}H\}$ NMR (101 MHz, $CDCl_3$) of 4bl

3m

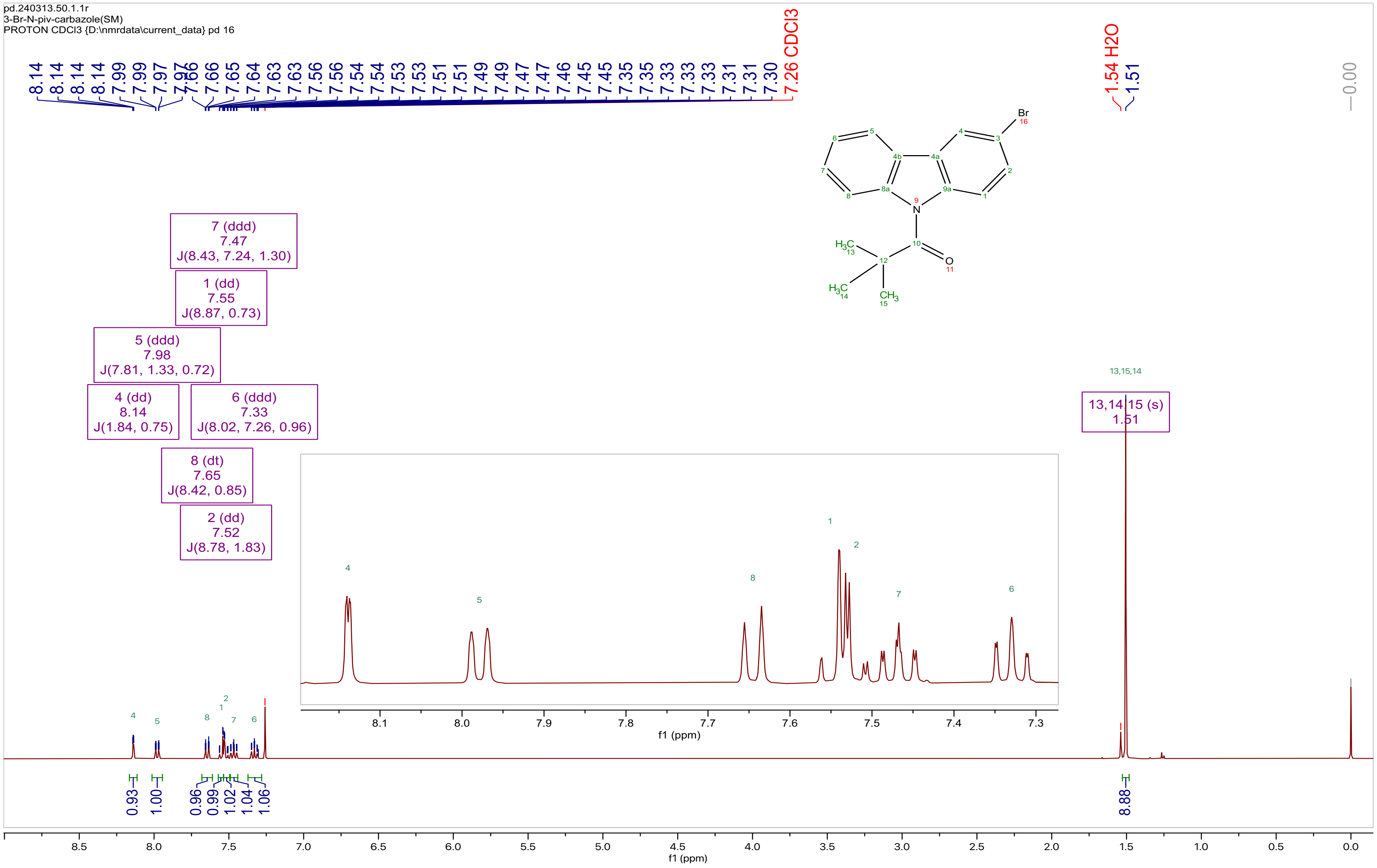

^{1}H NMR (400 MHz, $CDCl_3$) of 3m

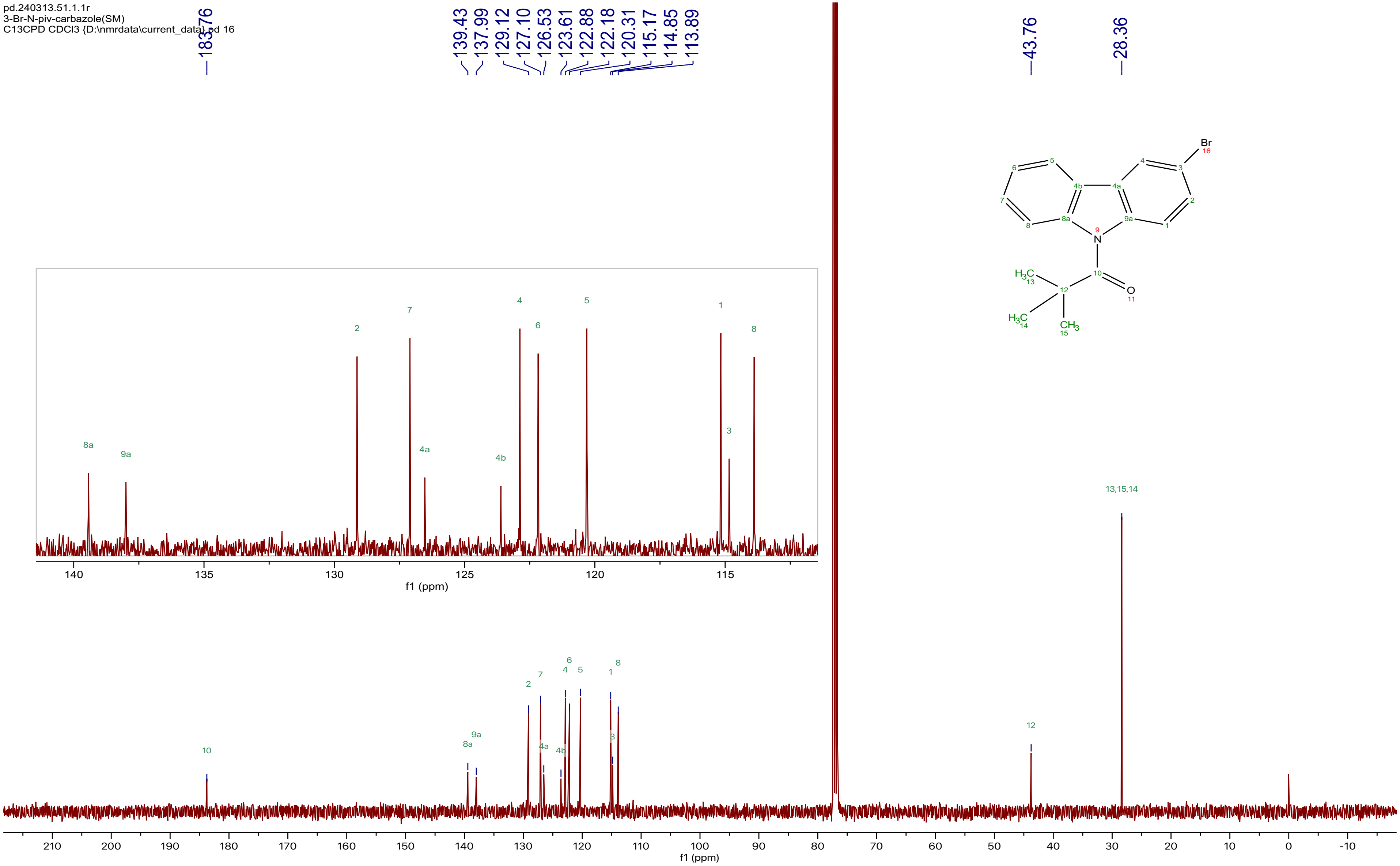

$^{13}C\{^1H\}$ NMR (101 MHz, $CDCl_3$) of 3m

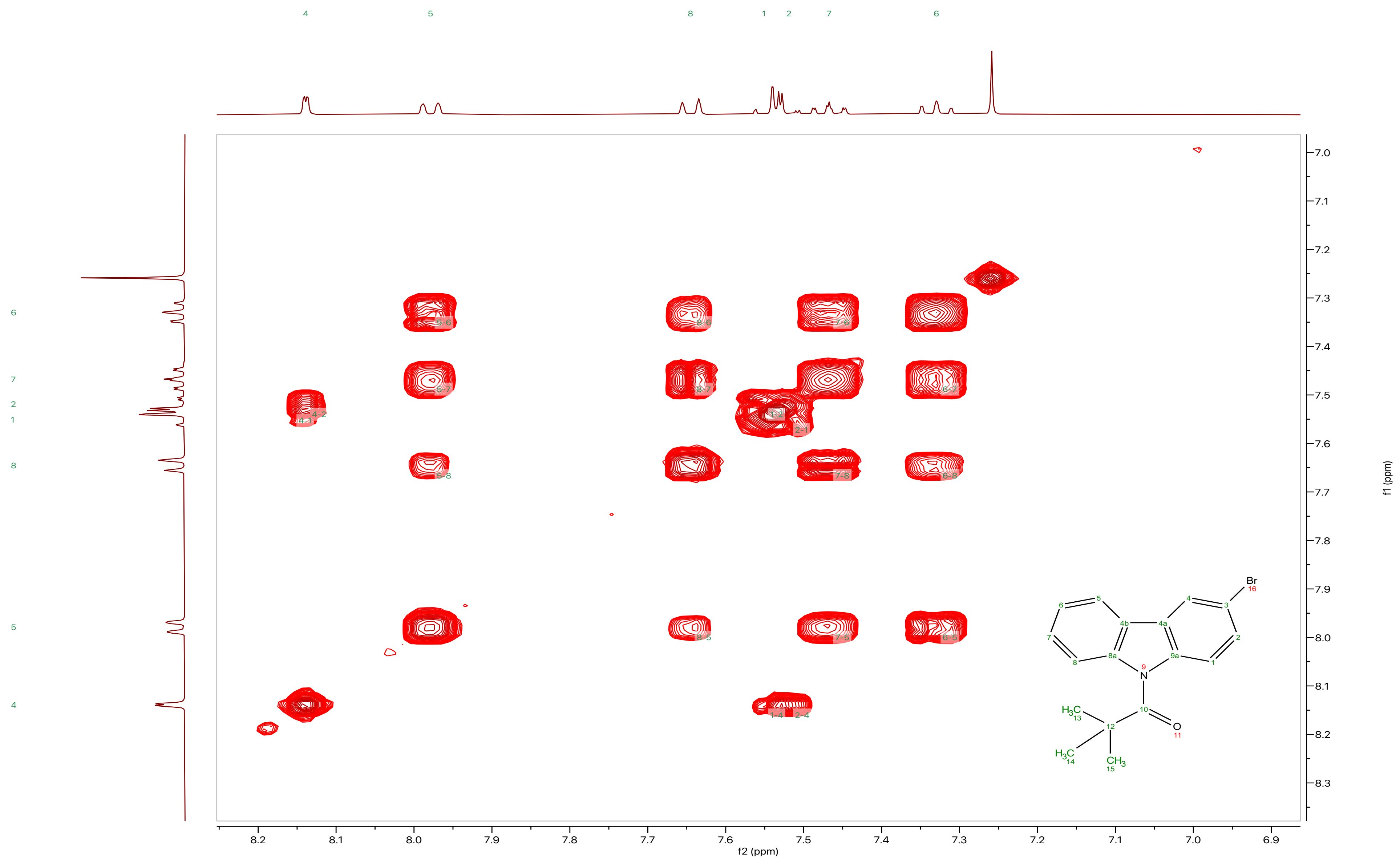

^{1}H-^{1}H COSY (400 MHz, $CDCl_3$) of 3m

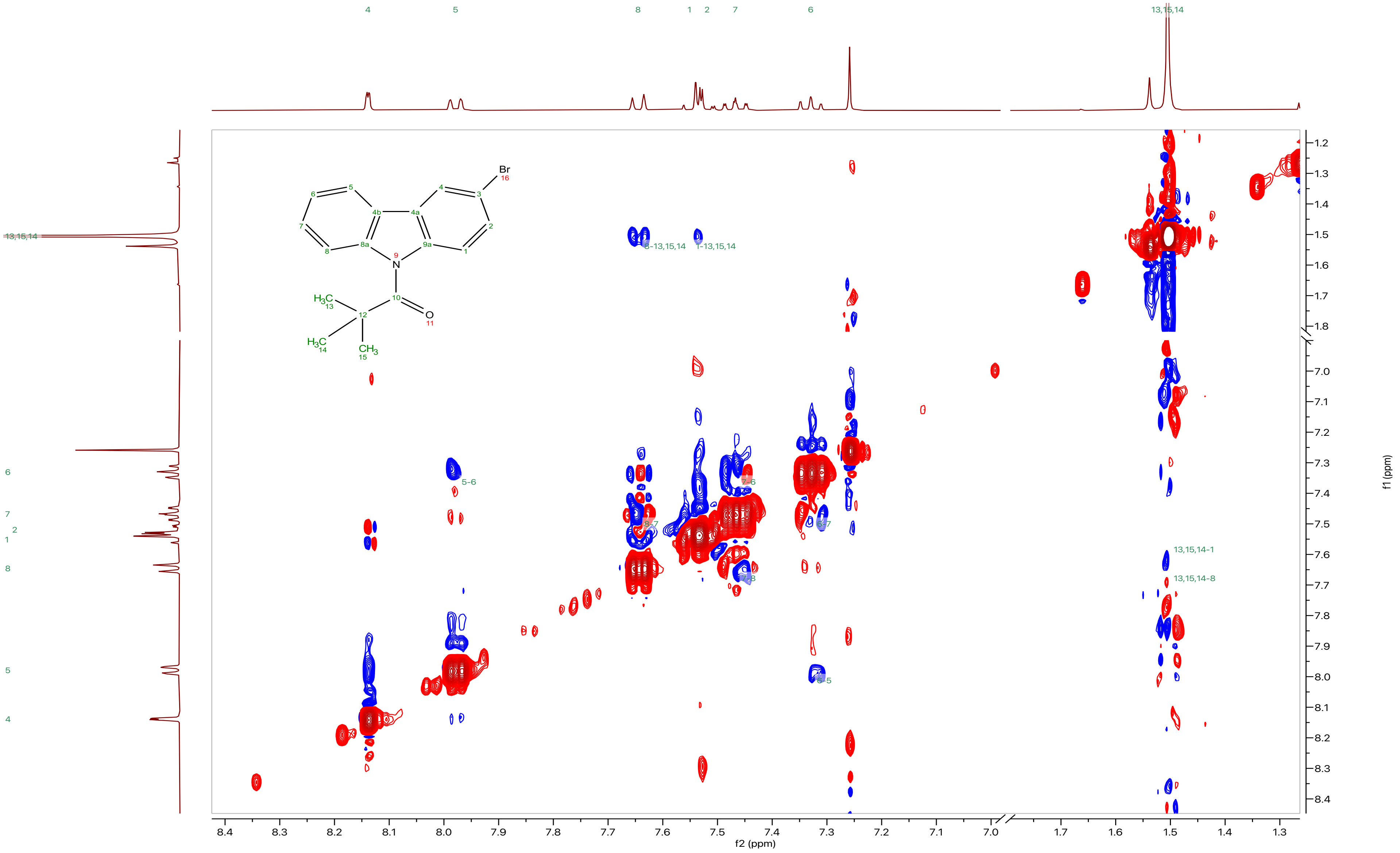

^1H-^1H NOESY (400 MHz, $CDCl_3$) of 3m

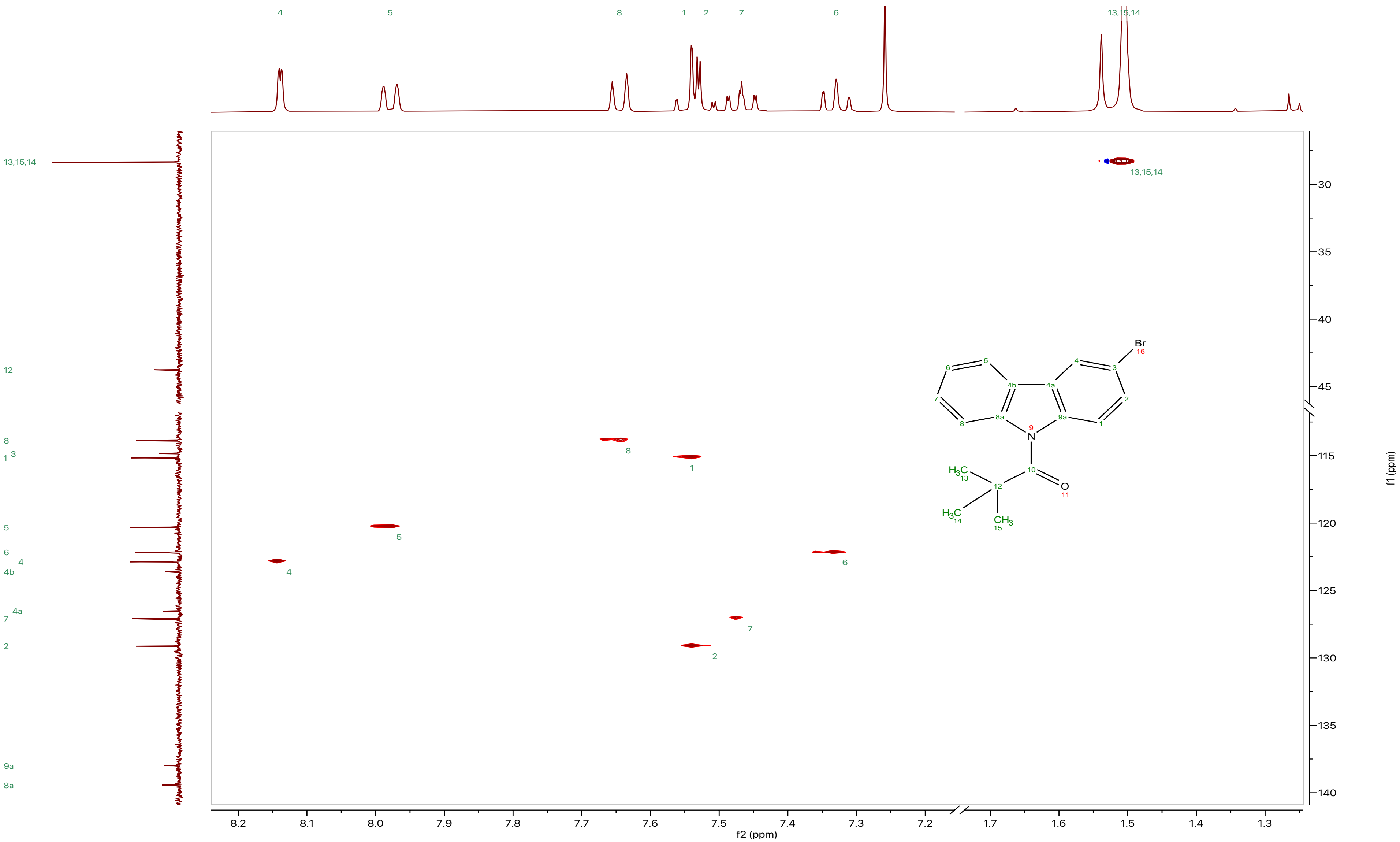

^{1}H-^{13}C{^{1}H} HSQC NMR (400/101 MHz, $CDCl_3$) of 3m

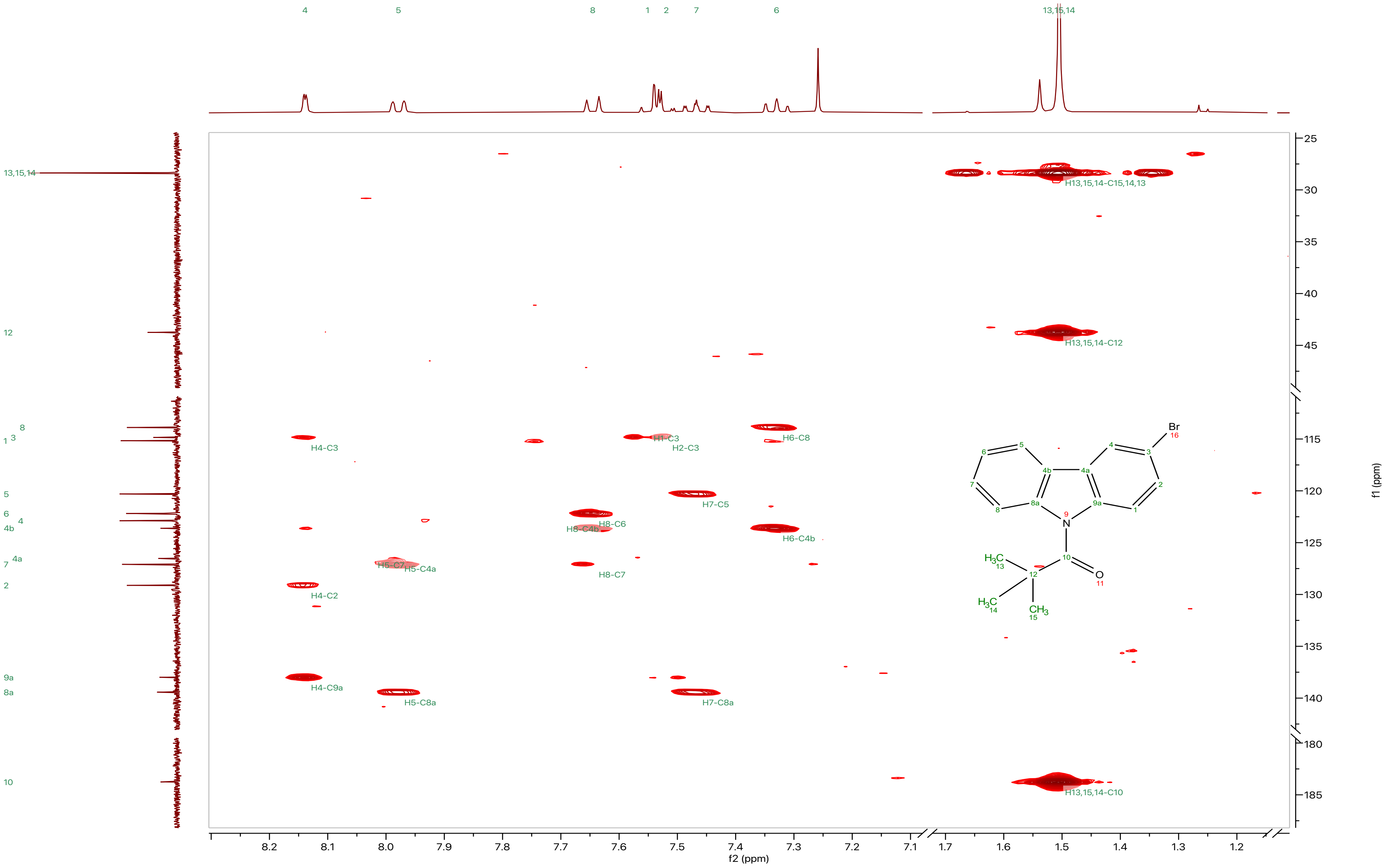

^1H-$^{13}C\{^1H\}$ HMBC NMR (400/101 MHz, $CDCl_3$) of 3m

3m'

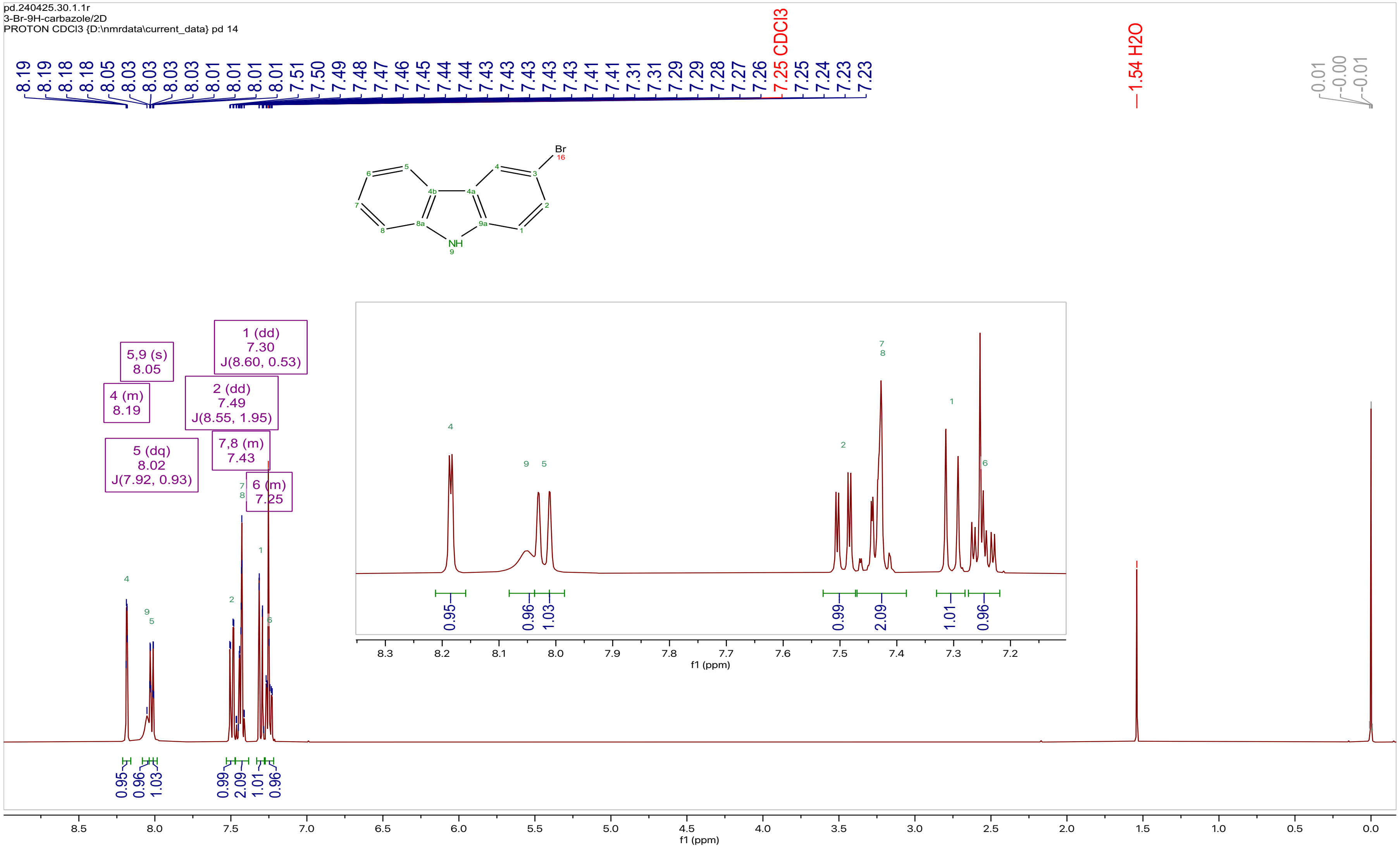

^{1}H NMR (400 MHz, $CDCl_3$) of 3m'

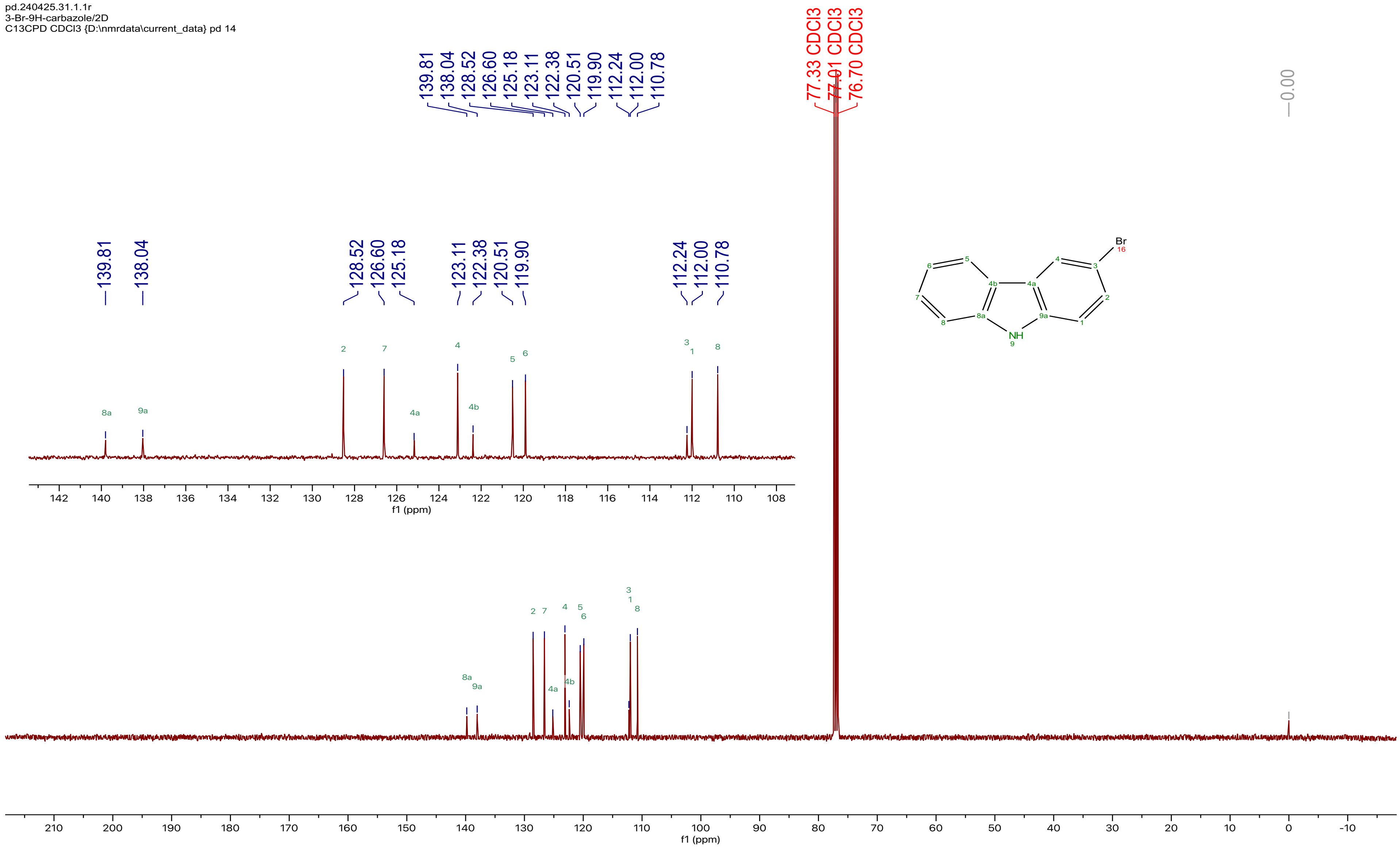

$^{13}C\{^{1}H\}$ NMR (101 MHz, $CDCl_3$) of 3m’

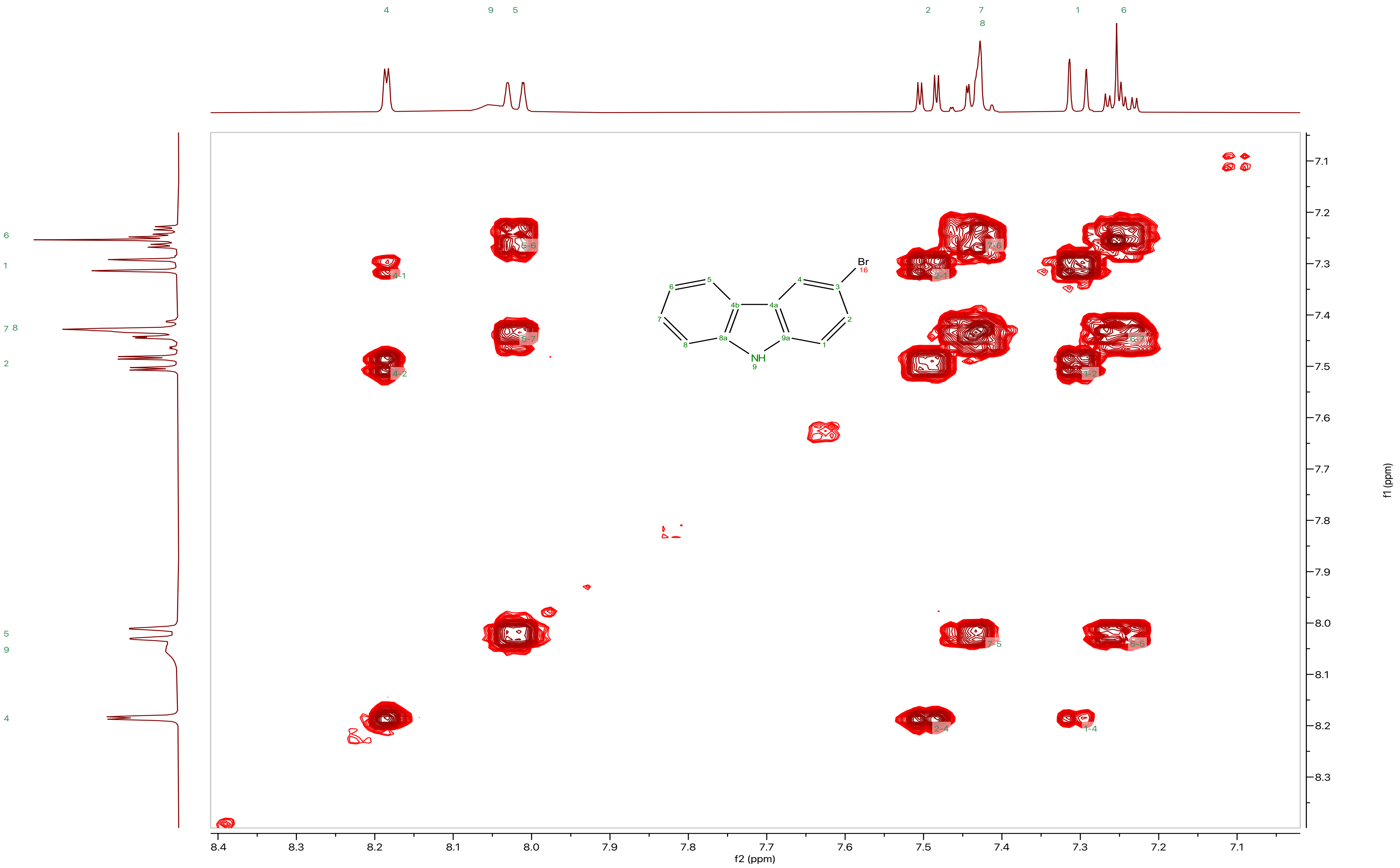

^1H-^1H COSY (400 MHz, $CDCl_3$) of 3m'

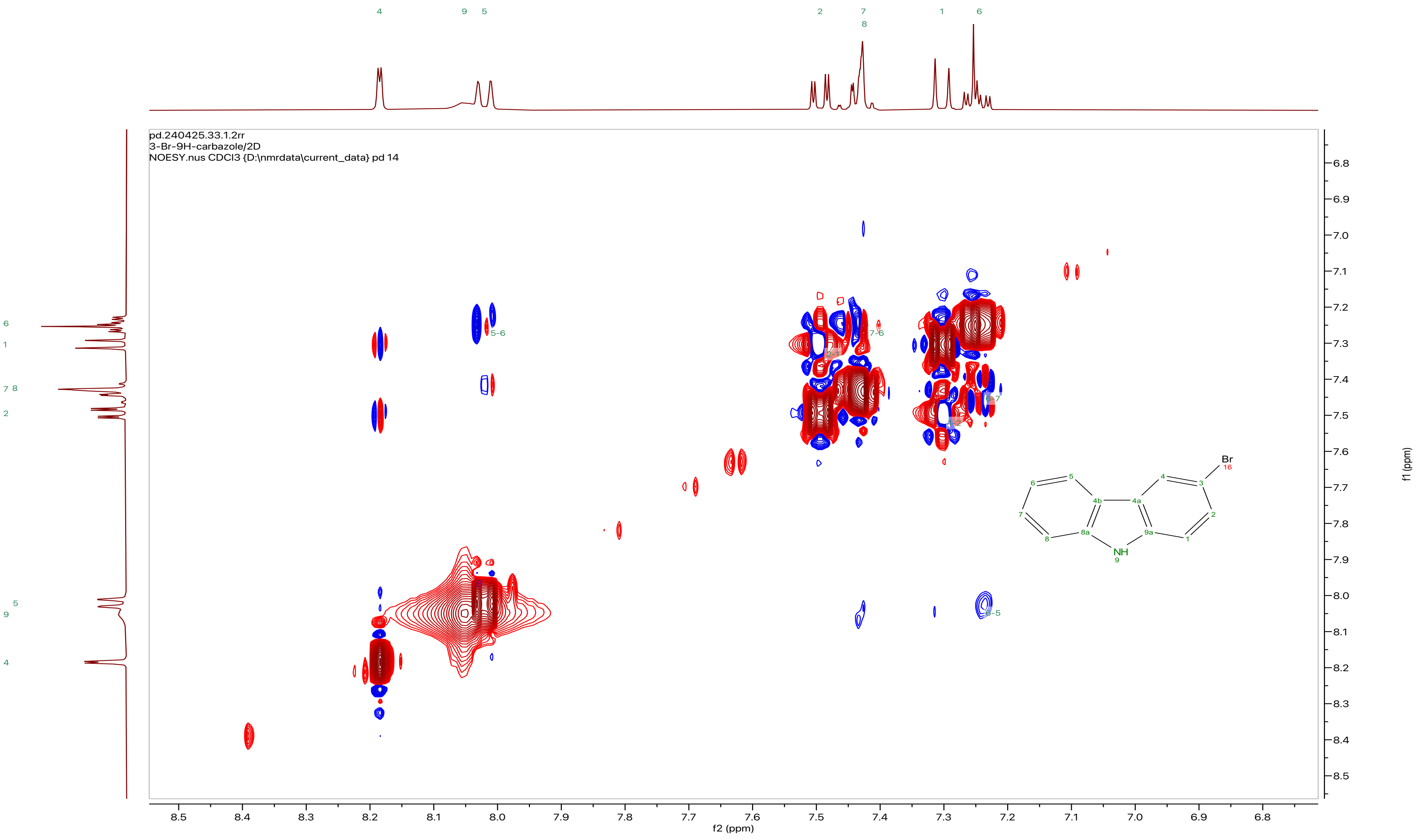

^1H-^1H NOESY (400 MHz, $CDCl_3$) of 3m'

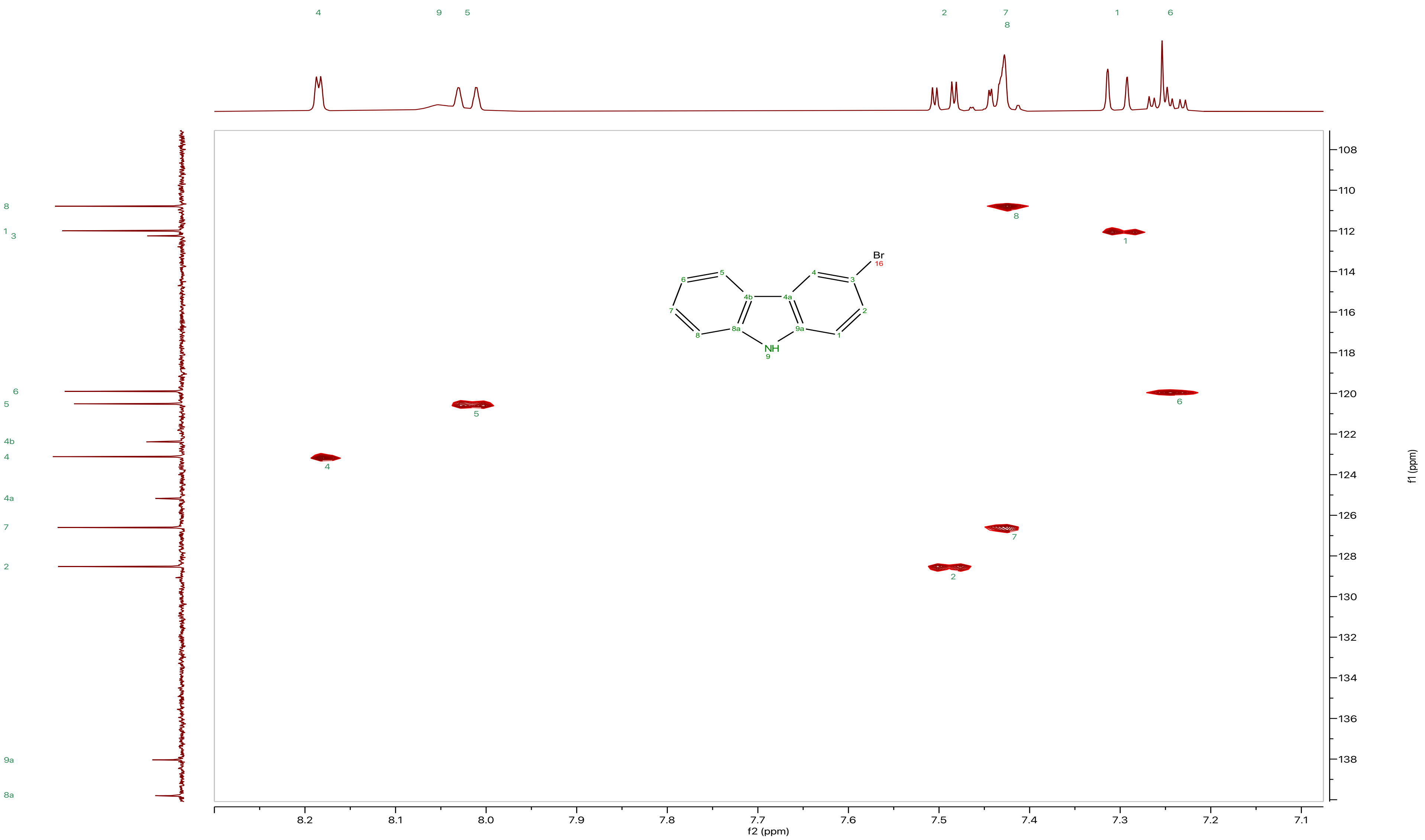

^{1}H-$^{13}C\{^{1}H\}$ HSQC NMR (400/101 MHz, $CDCl_3$) of 3m'

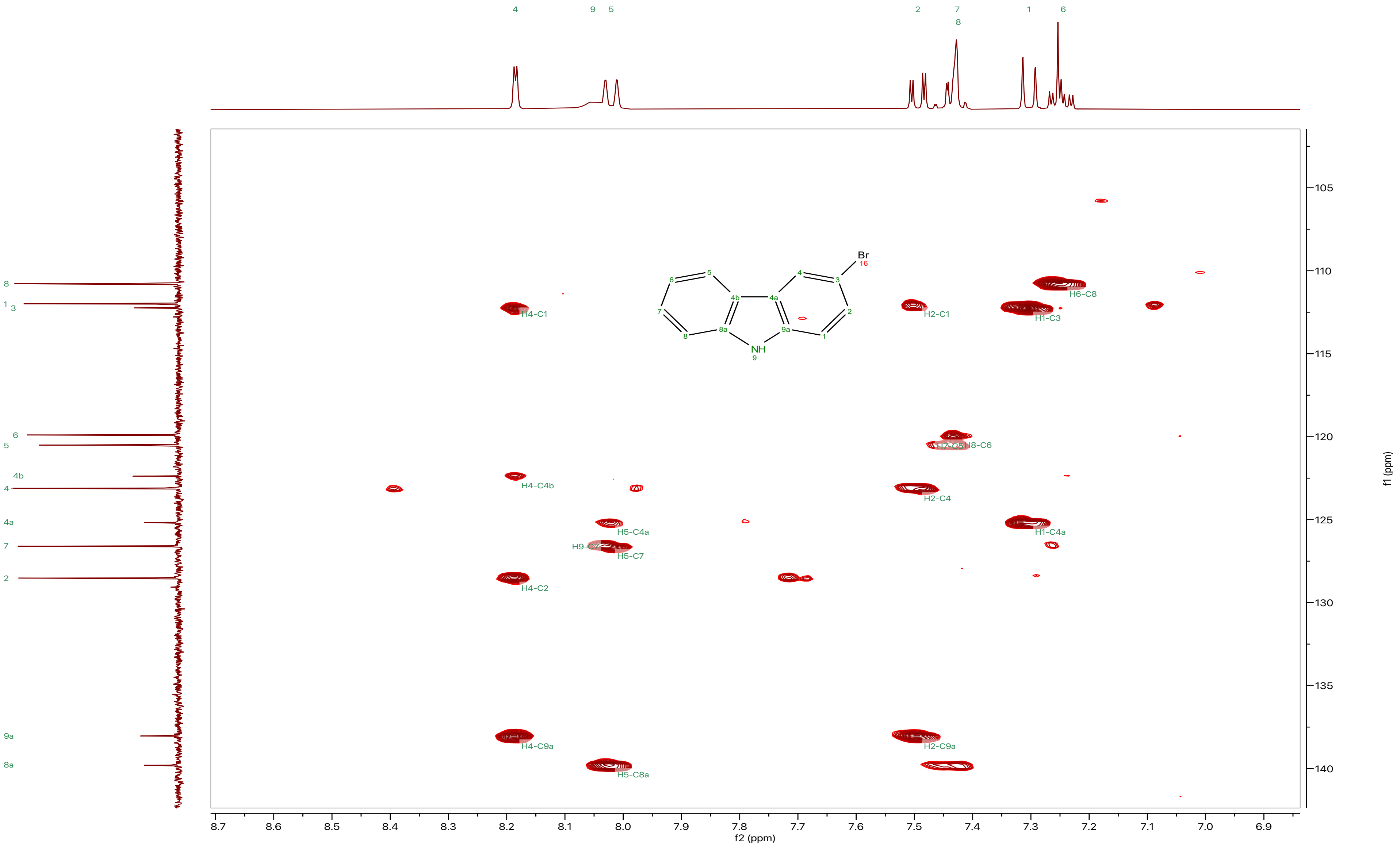

^{1}H-^{13}C{^{1}H} HMBC NMR (400/101 MHz, CDCl$_3$) of 3m'

4bm

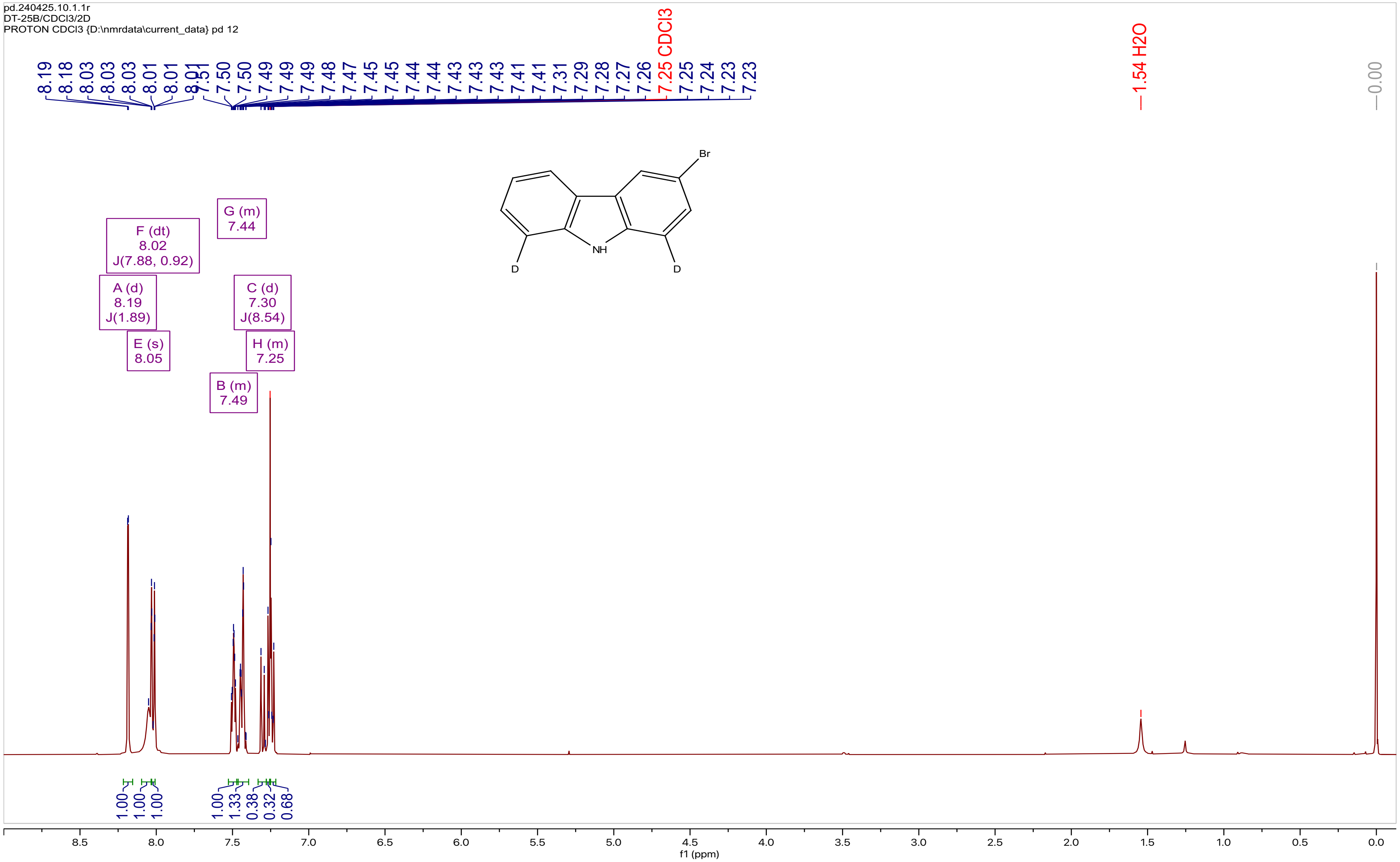

^{1}H NMR (400 MHz, $CDCl_3$) of 4bm

pd.240425.11.1.1r
DT-25B/CDCl3/2D
C13CPD CDCl3 {D:\nmrdata\current_data} pd 12

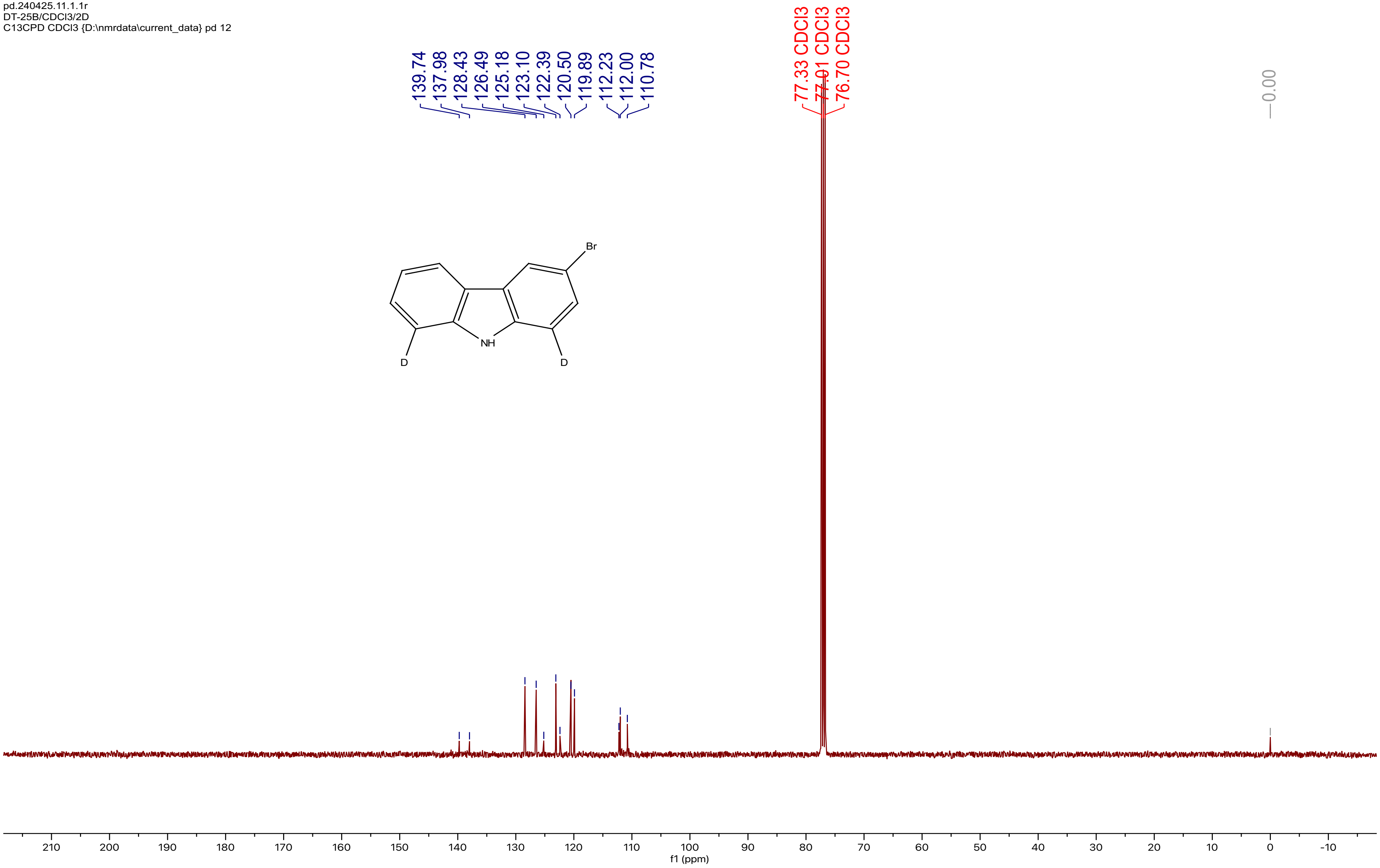

$^{13}C\{^1H\}$ NMR (101 MHz, $CDCl_3$) of 4bm

3n

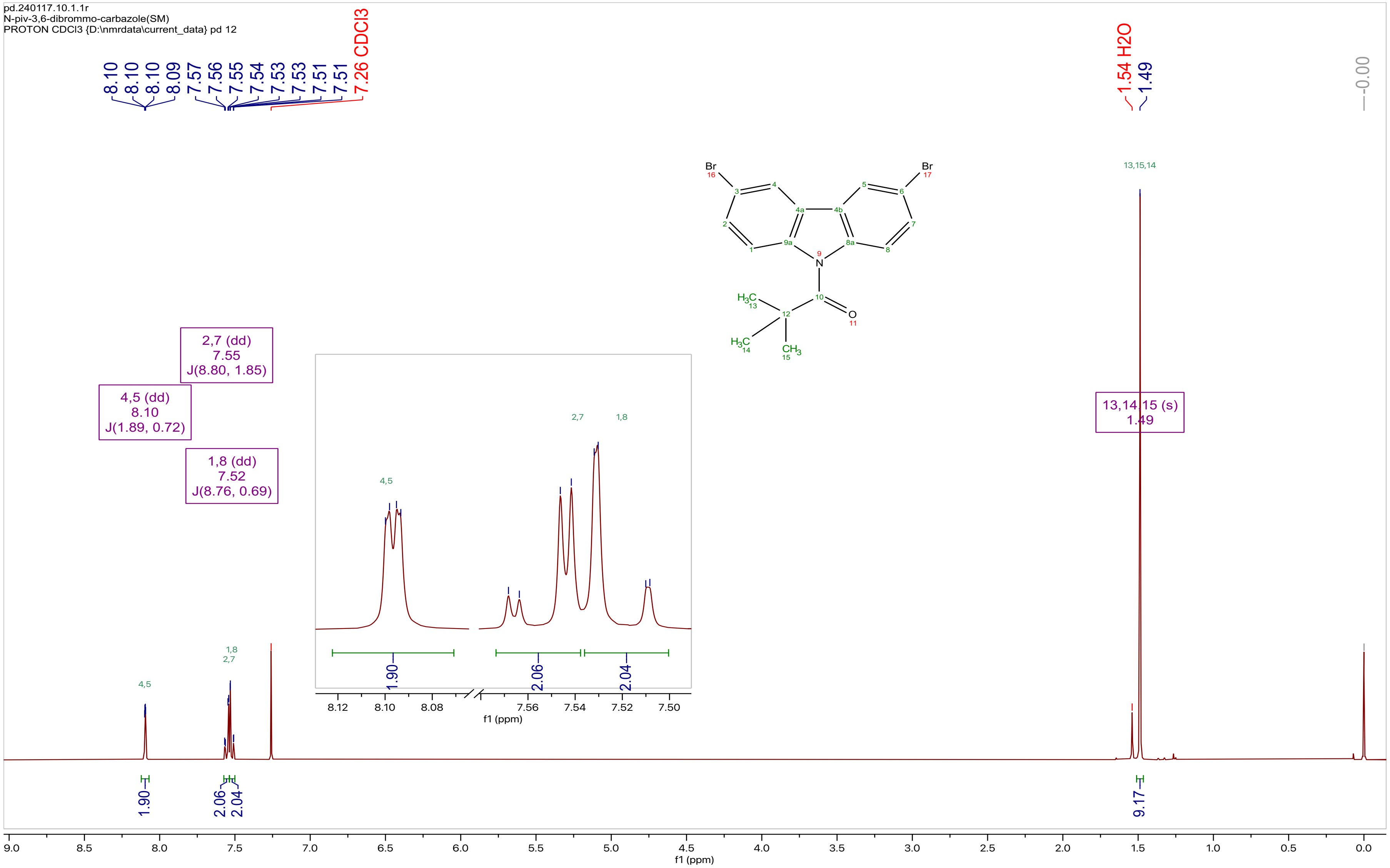

¹H NMR (400 MHz, $CDCl_3$) of 3n

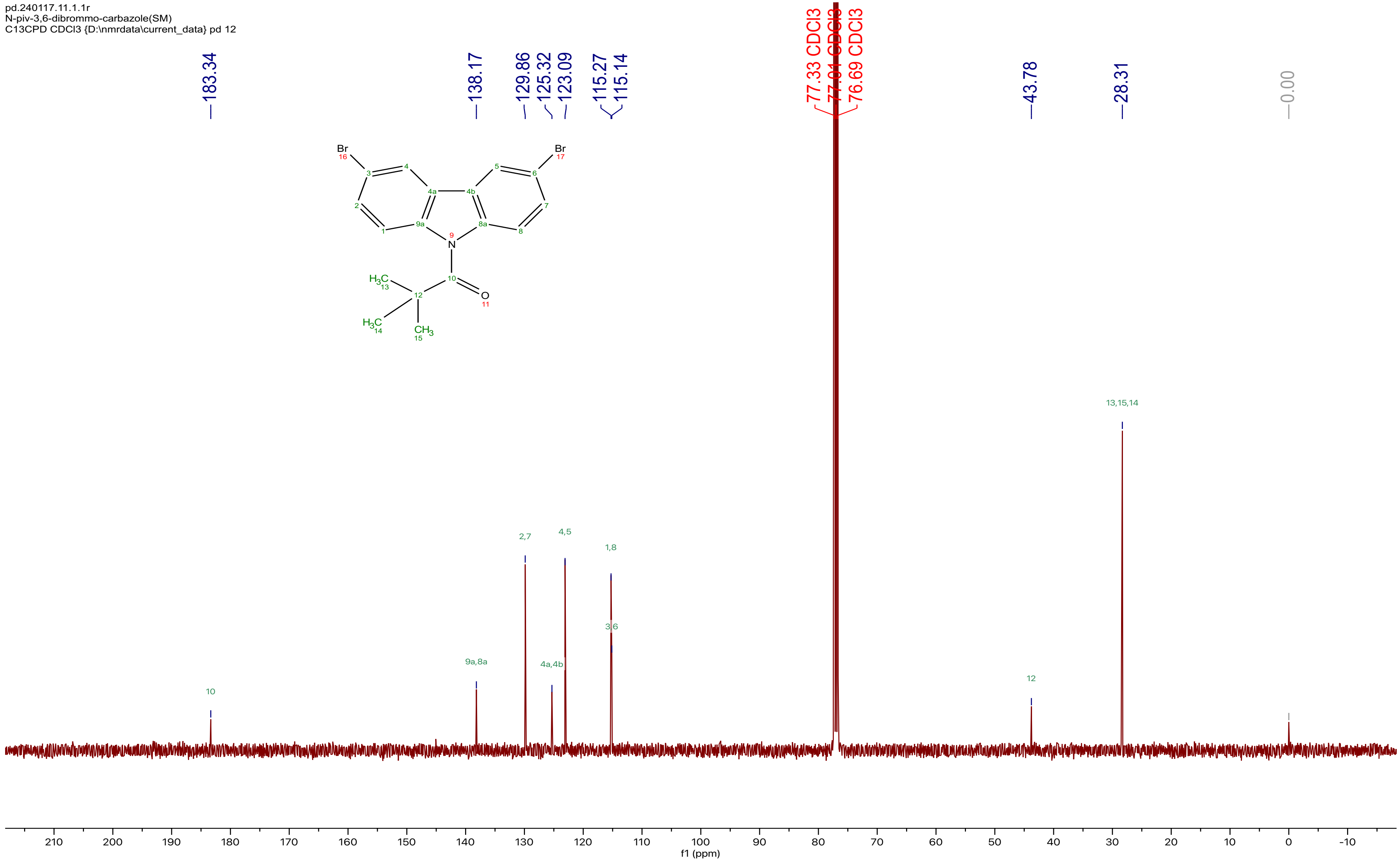

$^{13}C\{^{1}H\}$ NMR (101 MHz, $CDCl_3$) of 3n

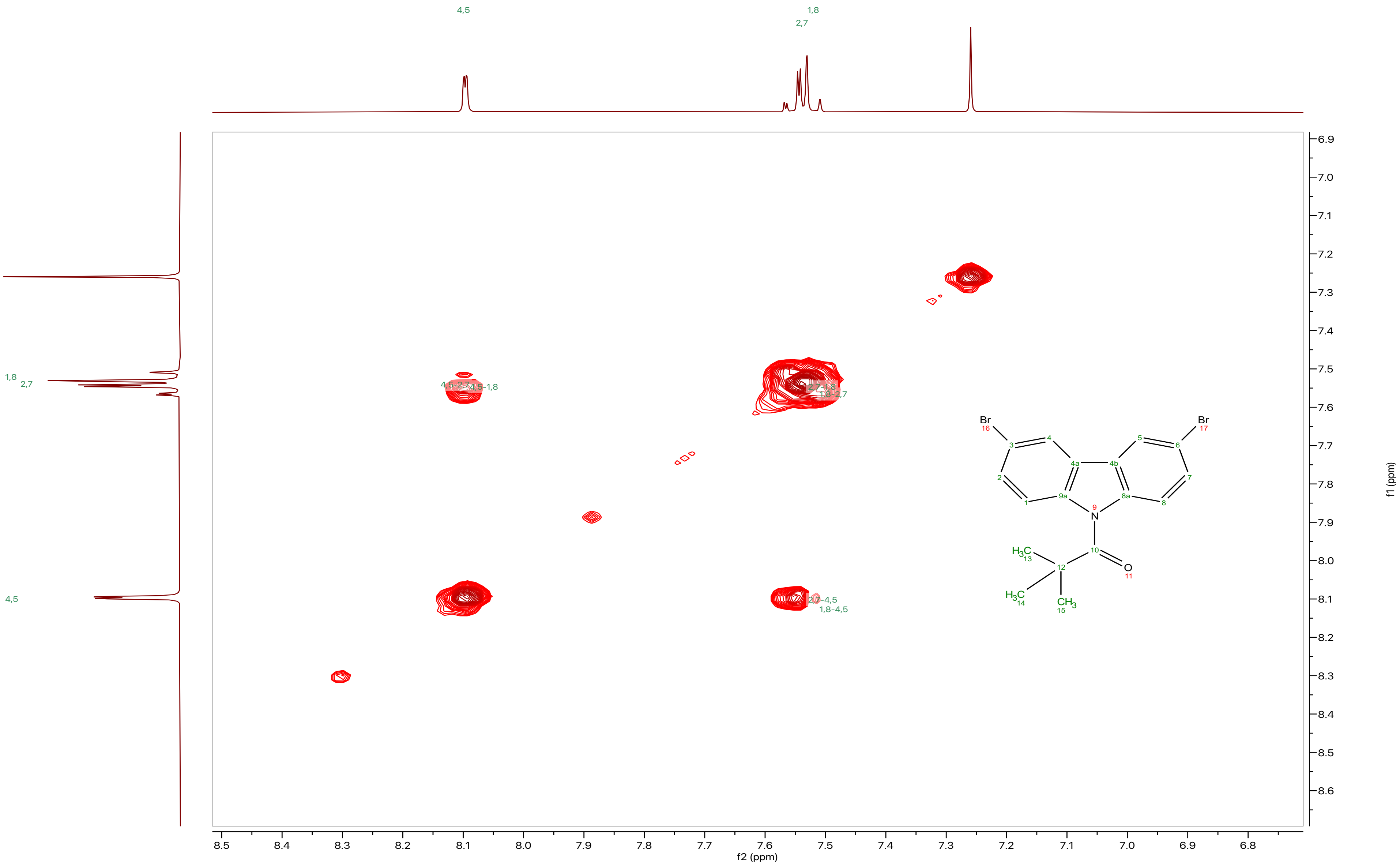

^1H-^1H COSY (400 MHz, $CDCl_3$) of 3n

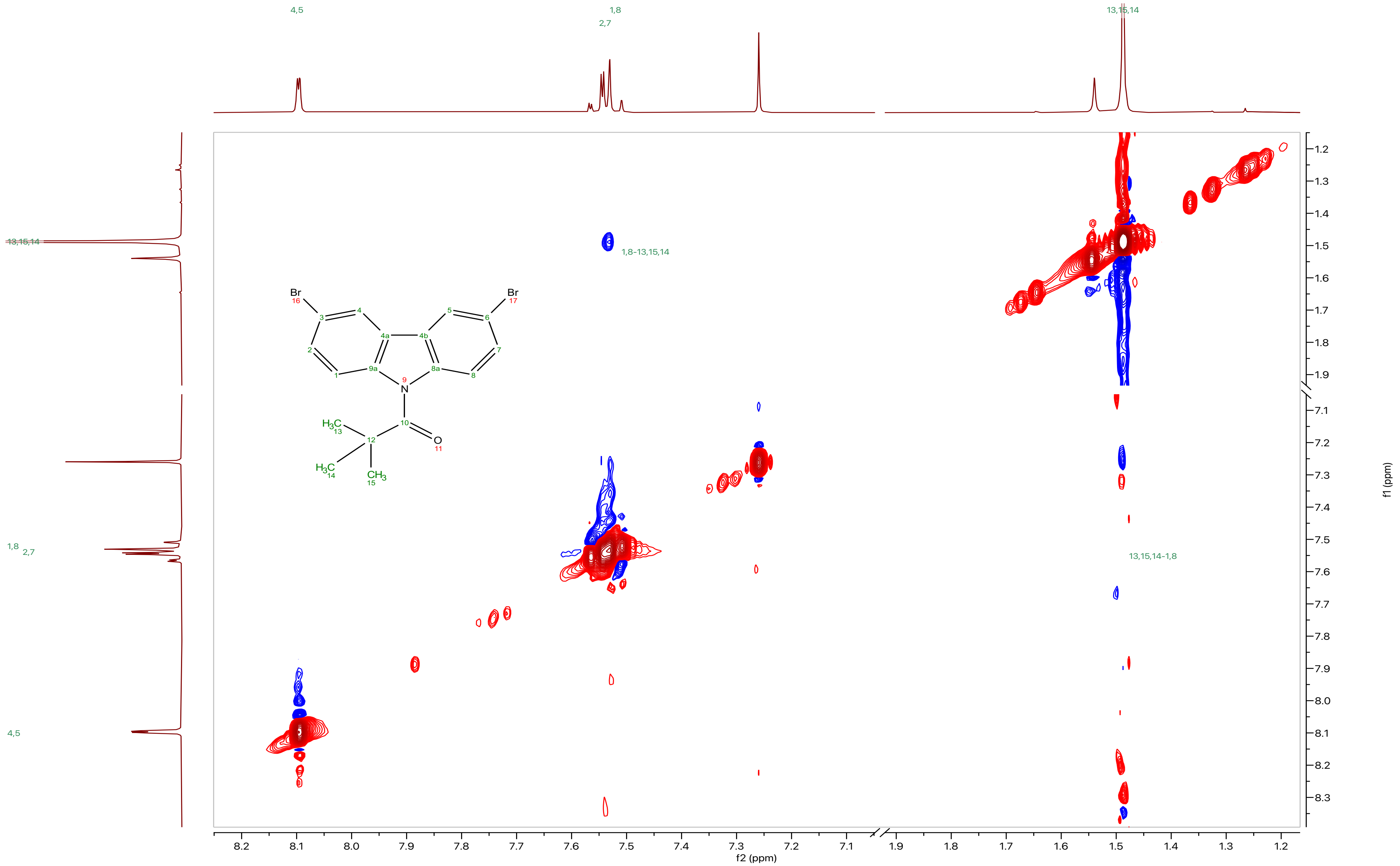

^1H-^1H NOESY (400 MHz, $CDCl_3$) of 3n

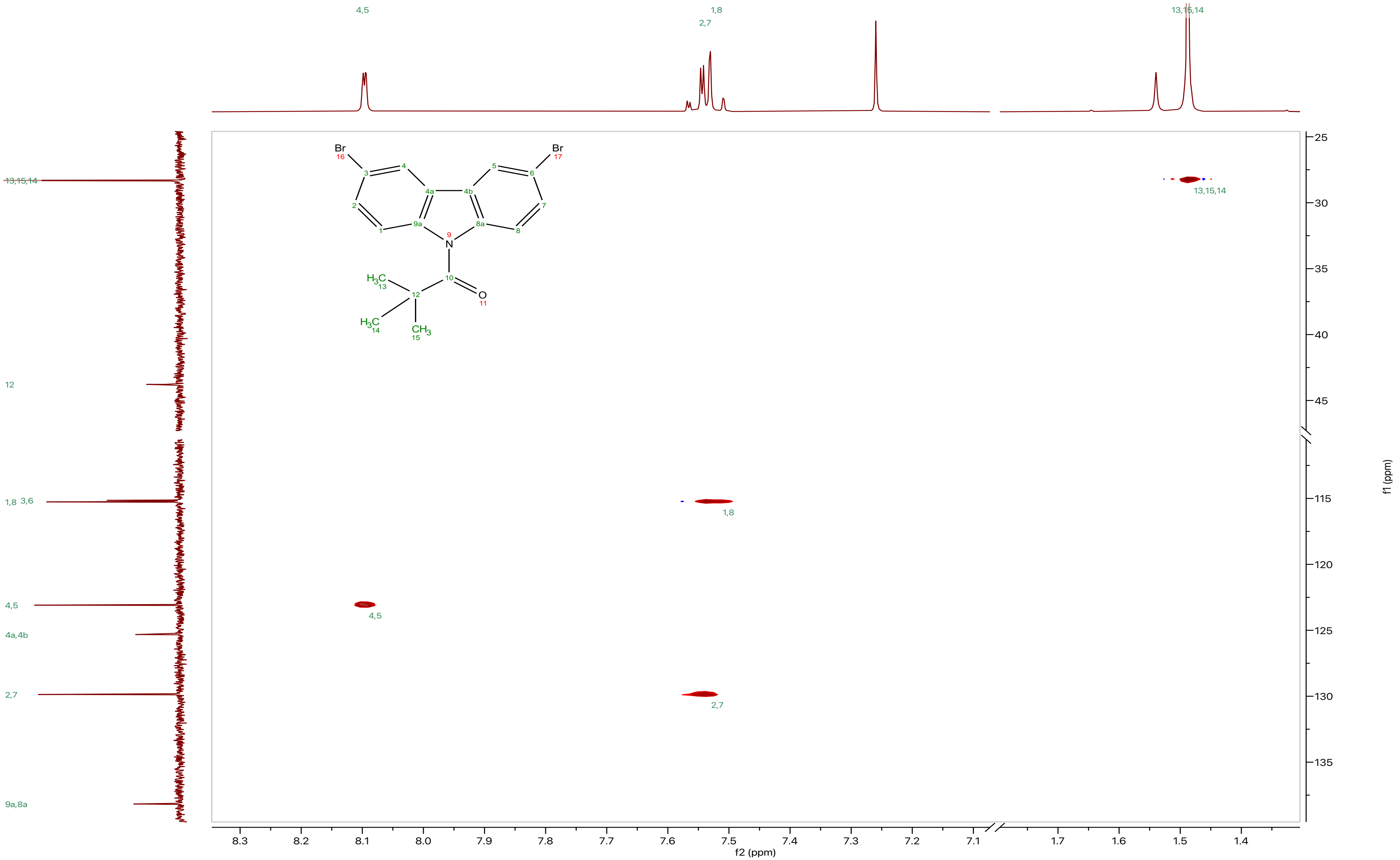

^{1}H-$^{13}C\{^{1}H\}$ HSQC NMR (400/101 MHz, $CDCl_3$) of 3n

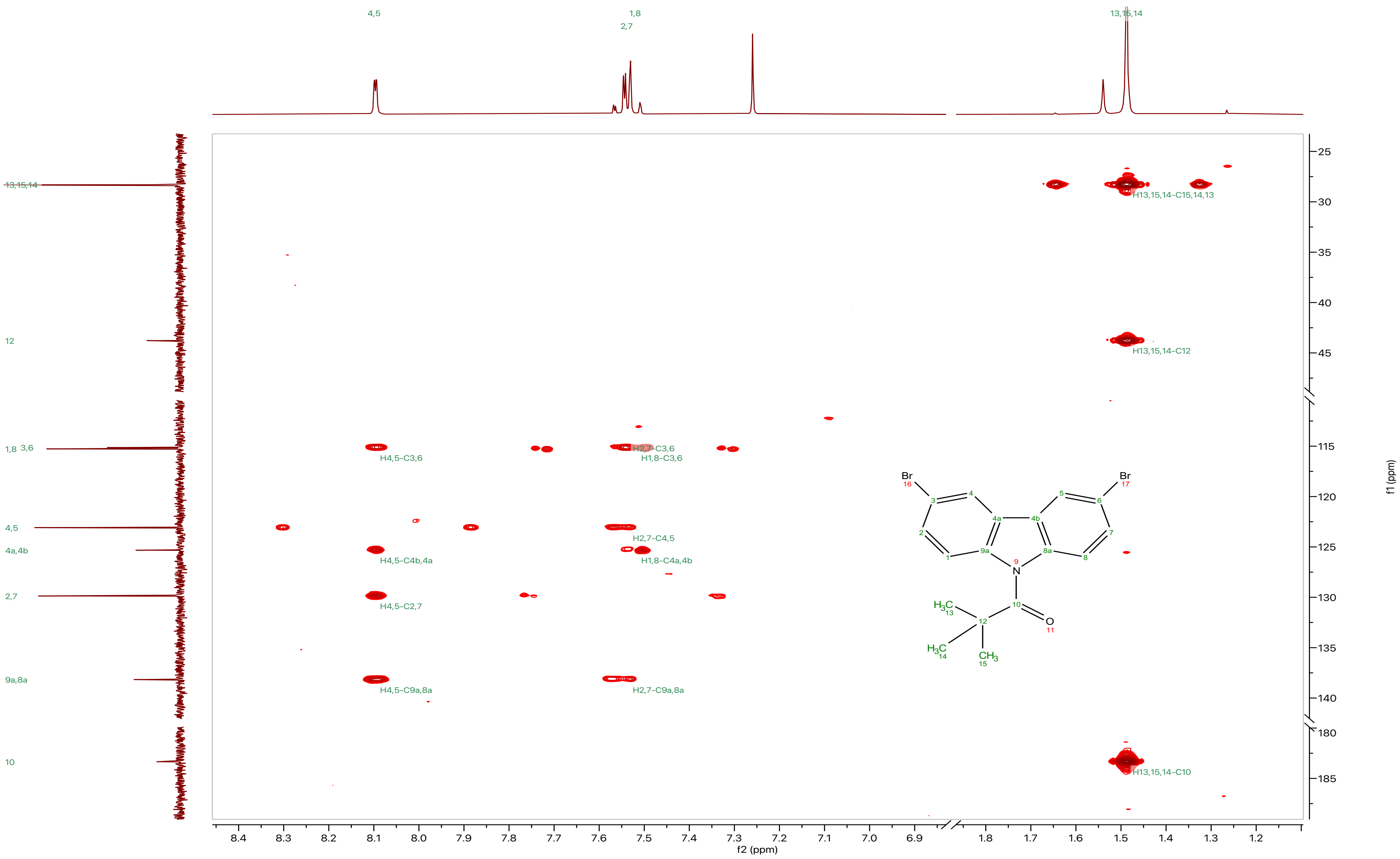

^{1}H-^{13}C{^{1}H} HMBC NMR (400/101 MHz, $CDCl_3$) of 3n

3n'

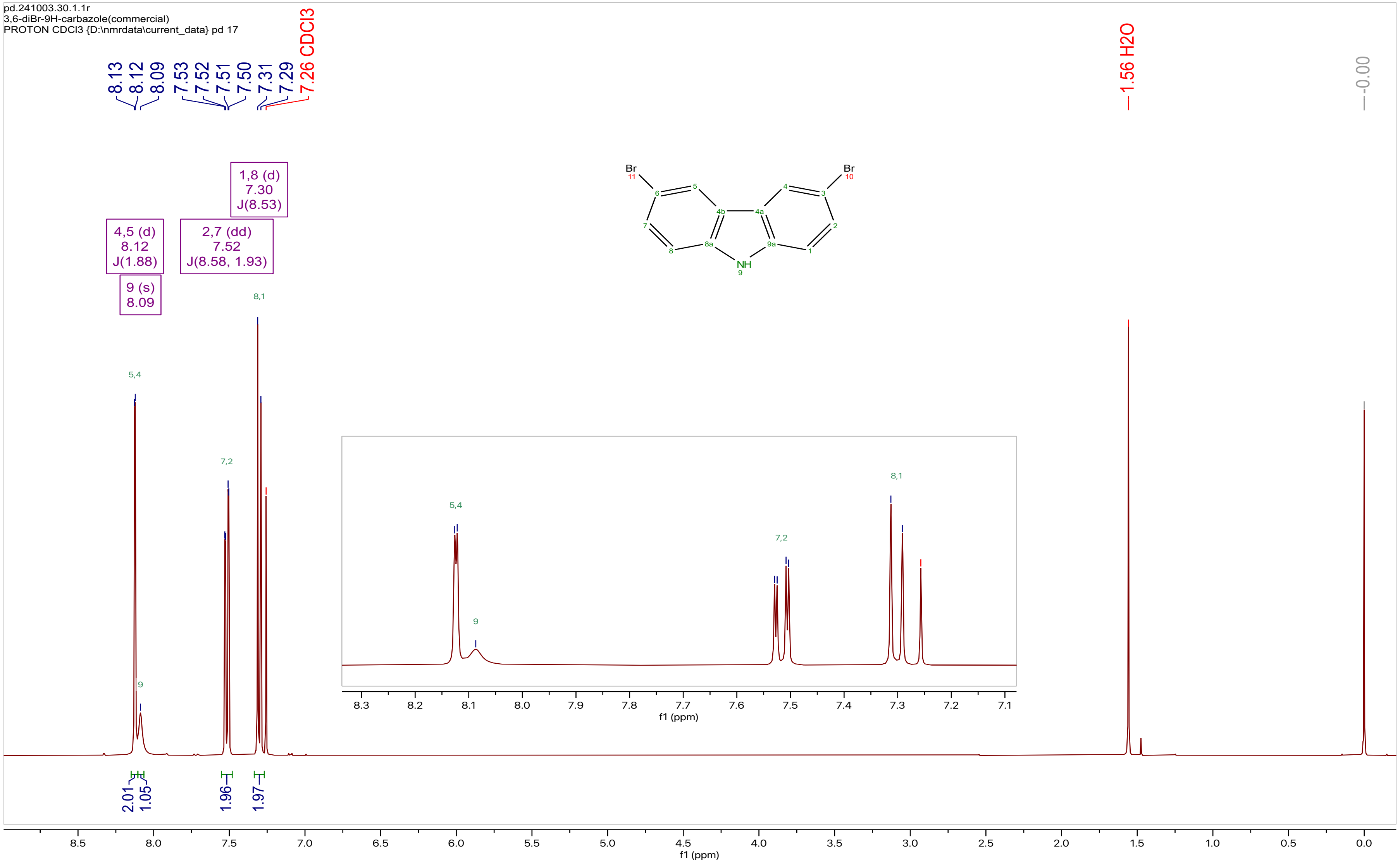

^{1}H NMR (400 MHz, $CDCl_3$) of 3n'

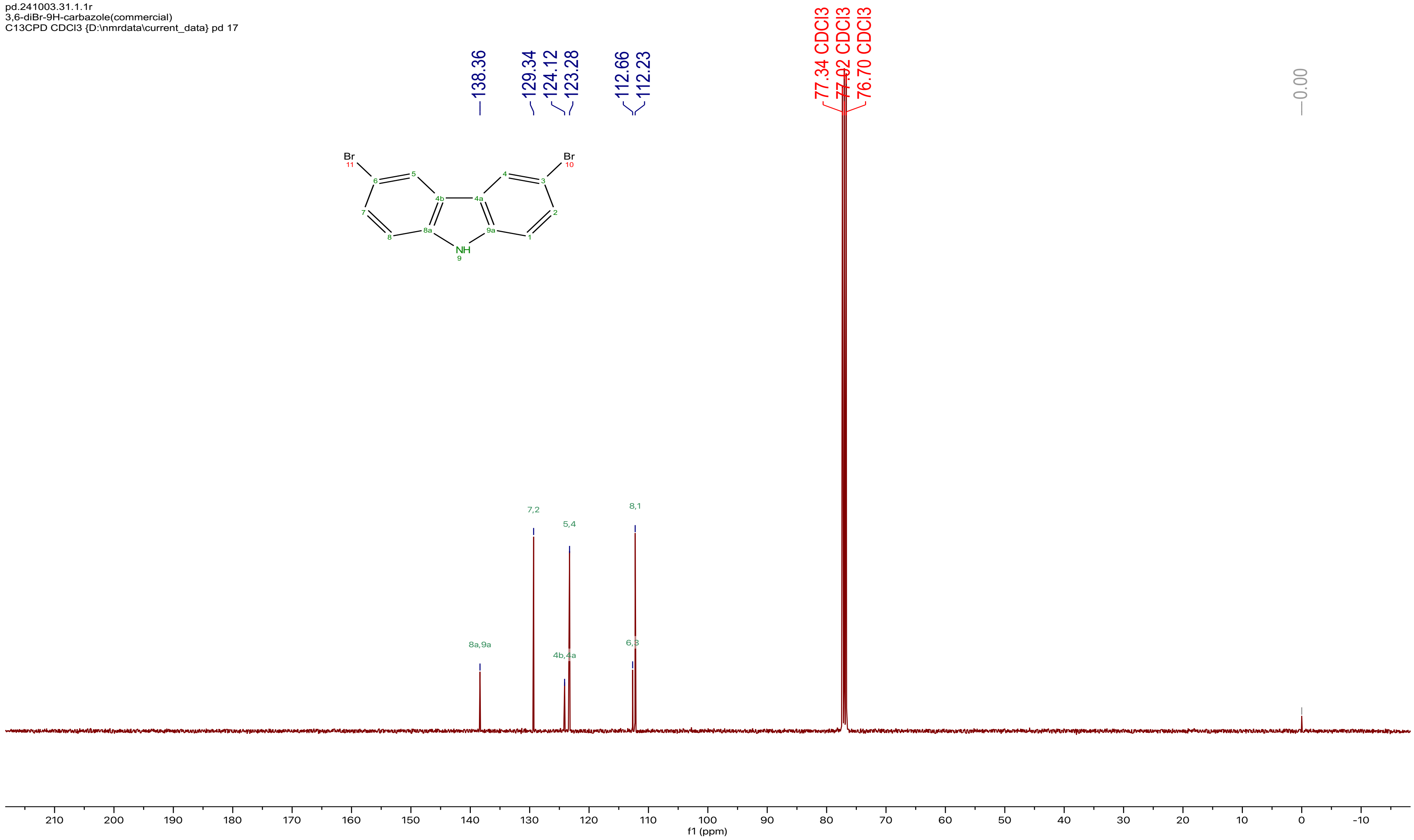

$^{13}C\{^{1}H\}$ NMR (101 MHz, $CDCl_3$) of 3n'

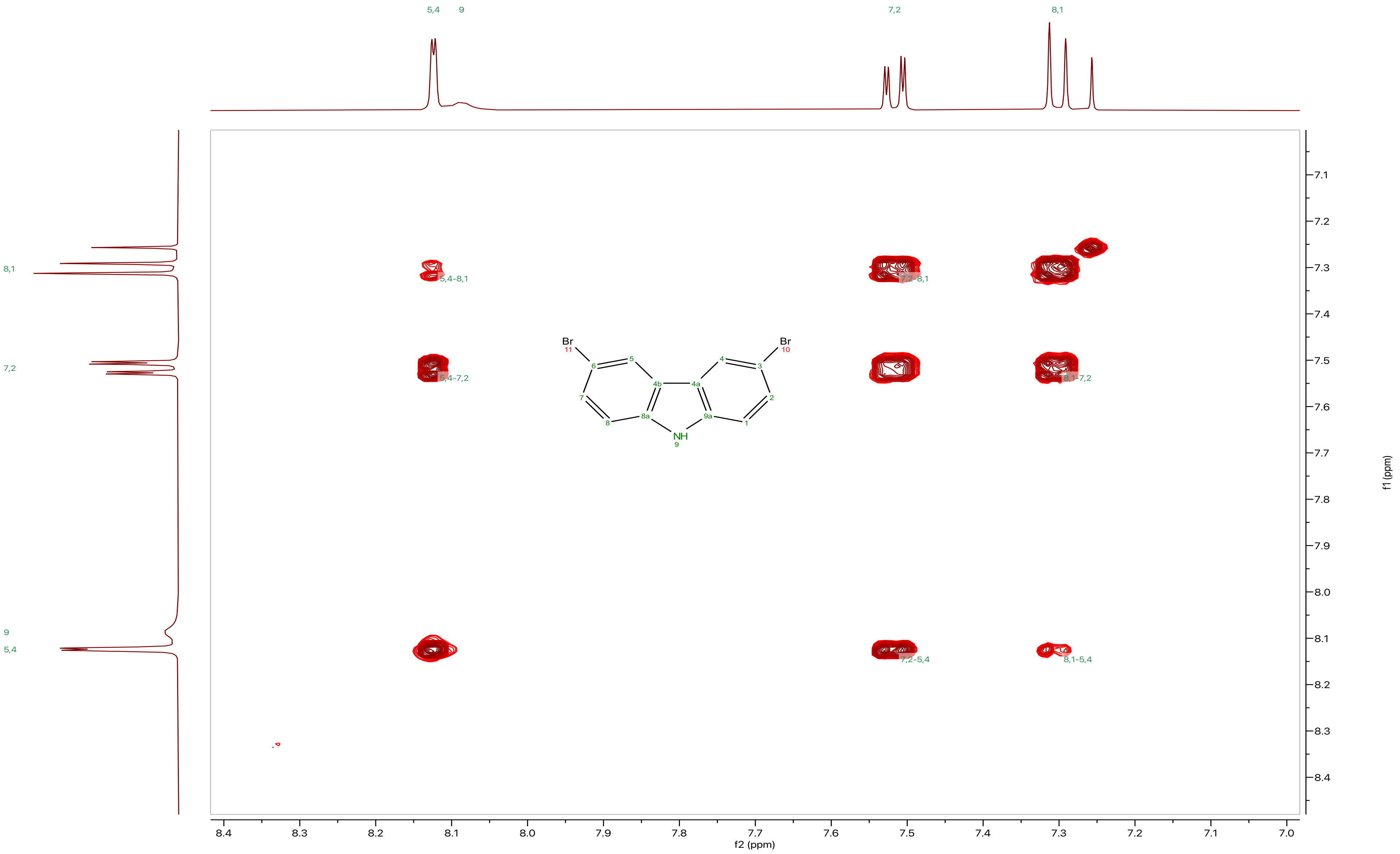

^1H-^1H COSY (400 MHz, $CDCl_3$) of 3n'

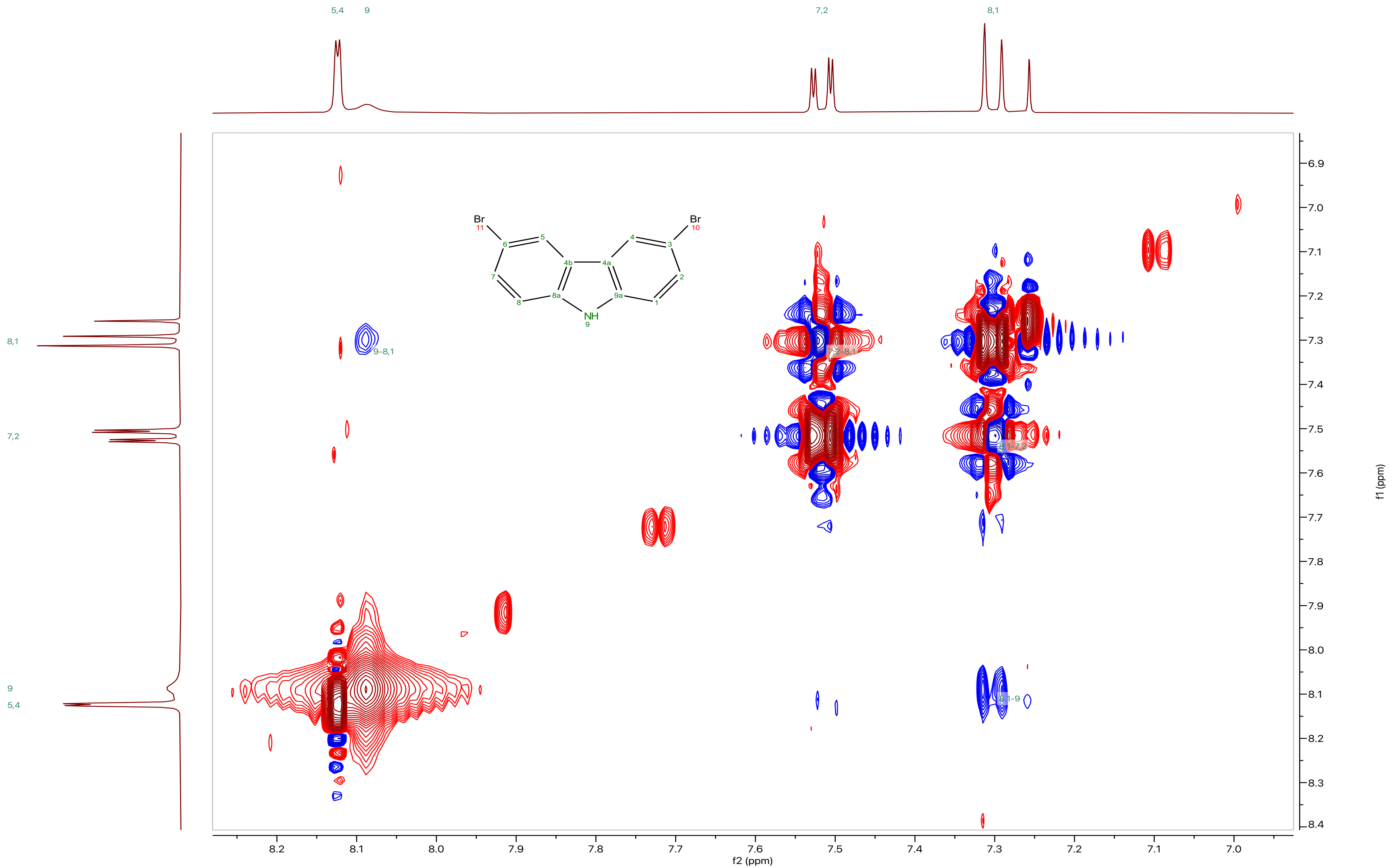

^1H-^1H NOESY (400 MHz, $CDCl_3$) of 3n'

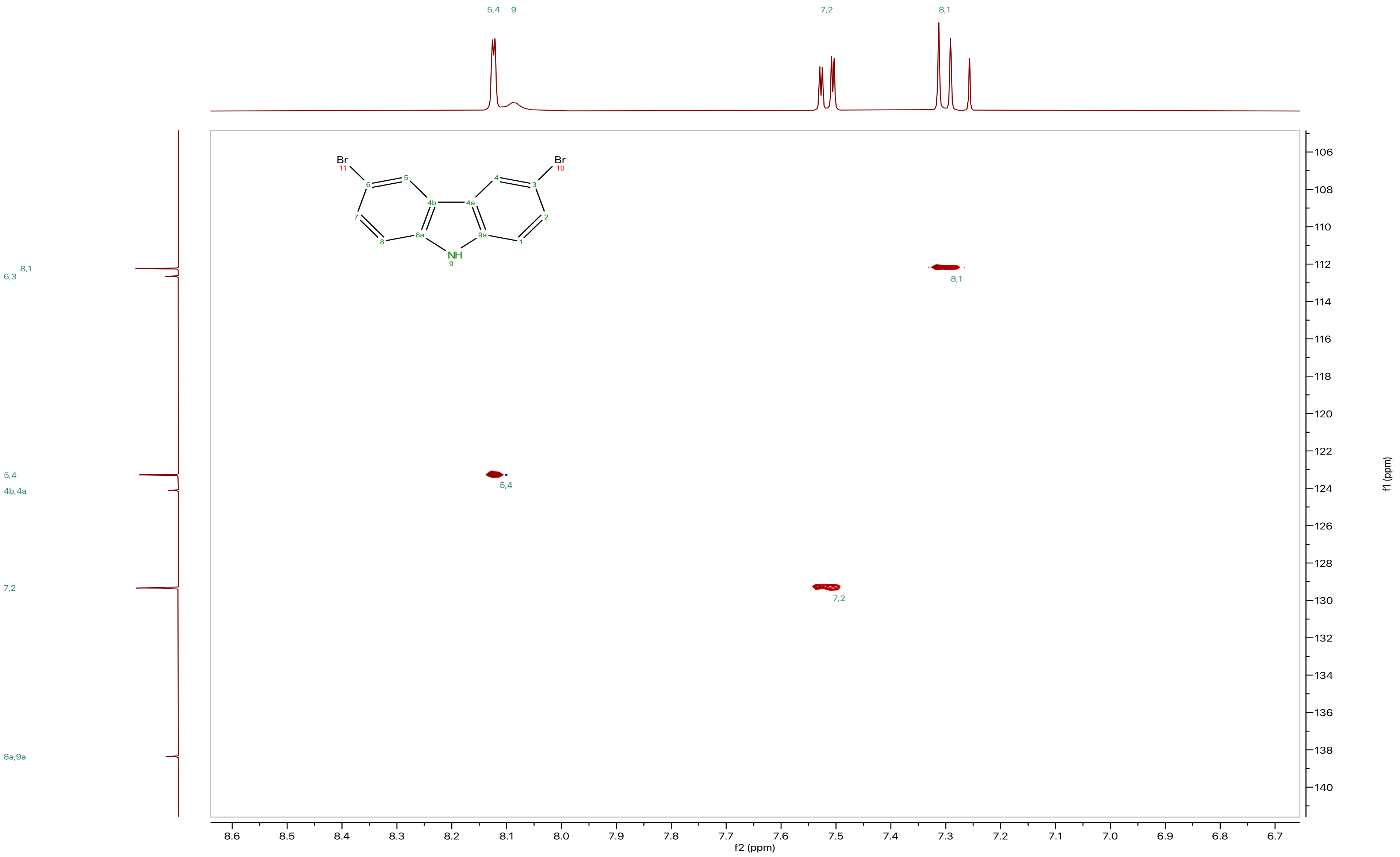

^{1}H-^{13}C{^{1}H} HSQC NMR (400/101 MHz, $CDCl_3$) of 3n'

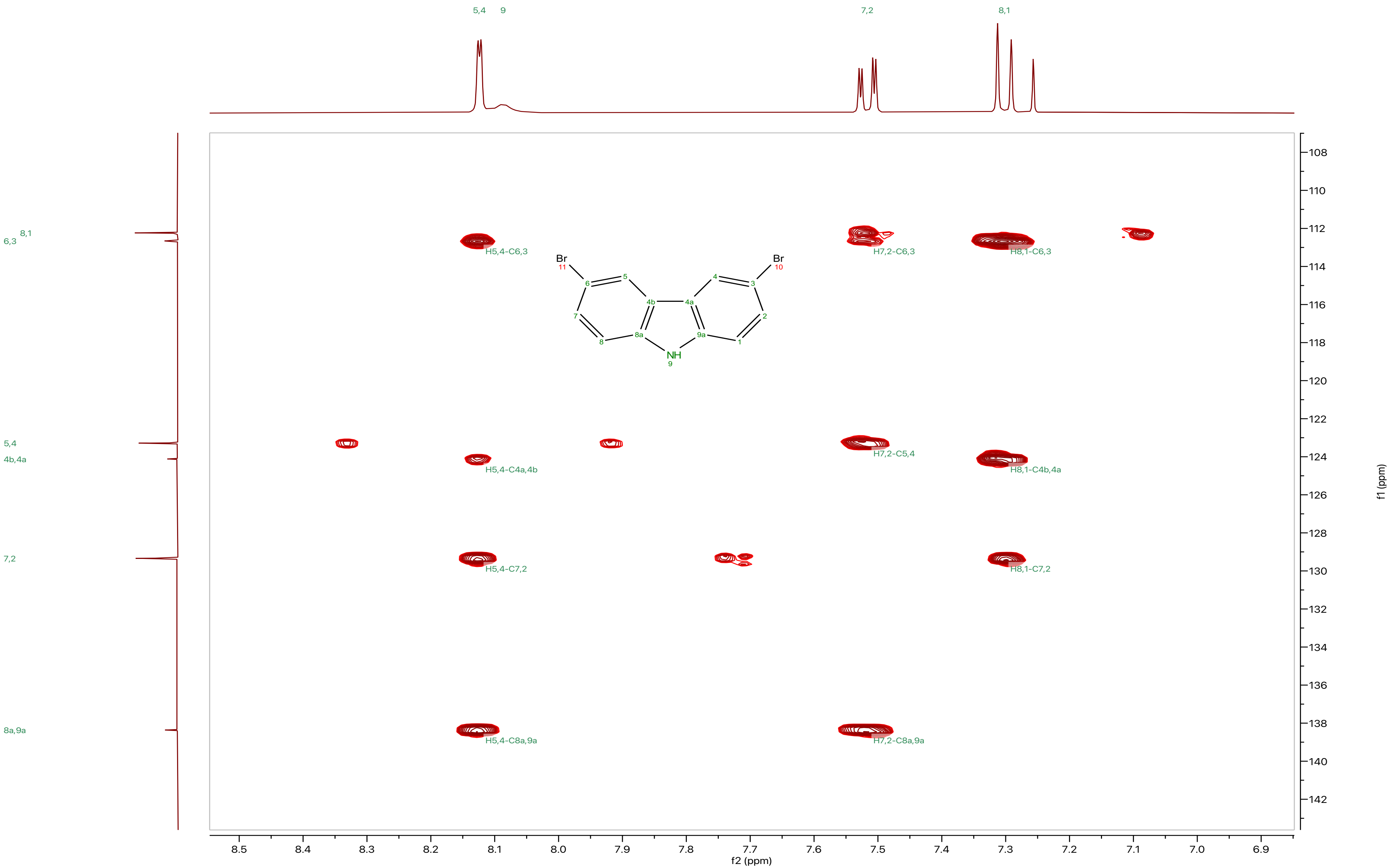

^{1}H-^{13}C{^{1}H} HMBC NMR (400/101 MHz, $CDCl_3$) of 3n'

4bn

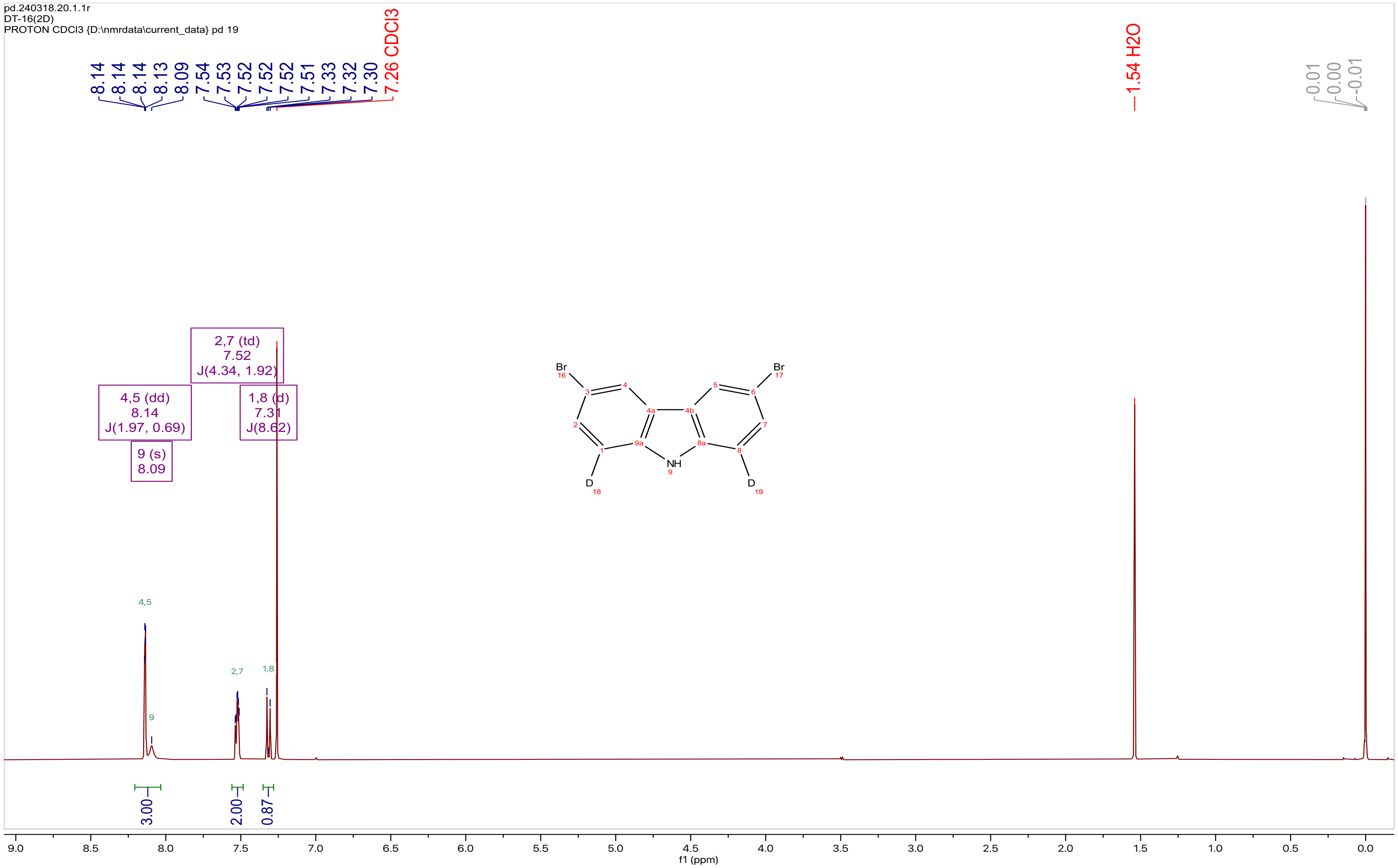

^{1}H NMR (400 MHz, CDCl$_3$) of 4bn

pd.240318.21.1.1r
DT-16(2D)
C13CPD CDCl3 {D:\nmrdata\current_data} pd 19

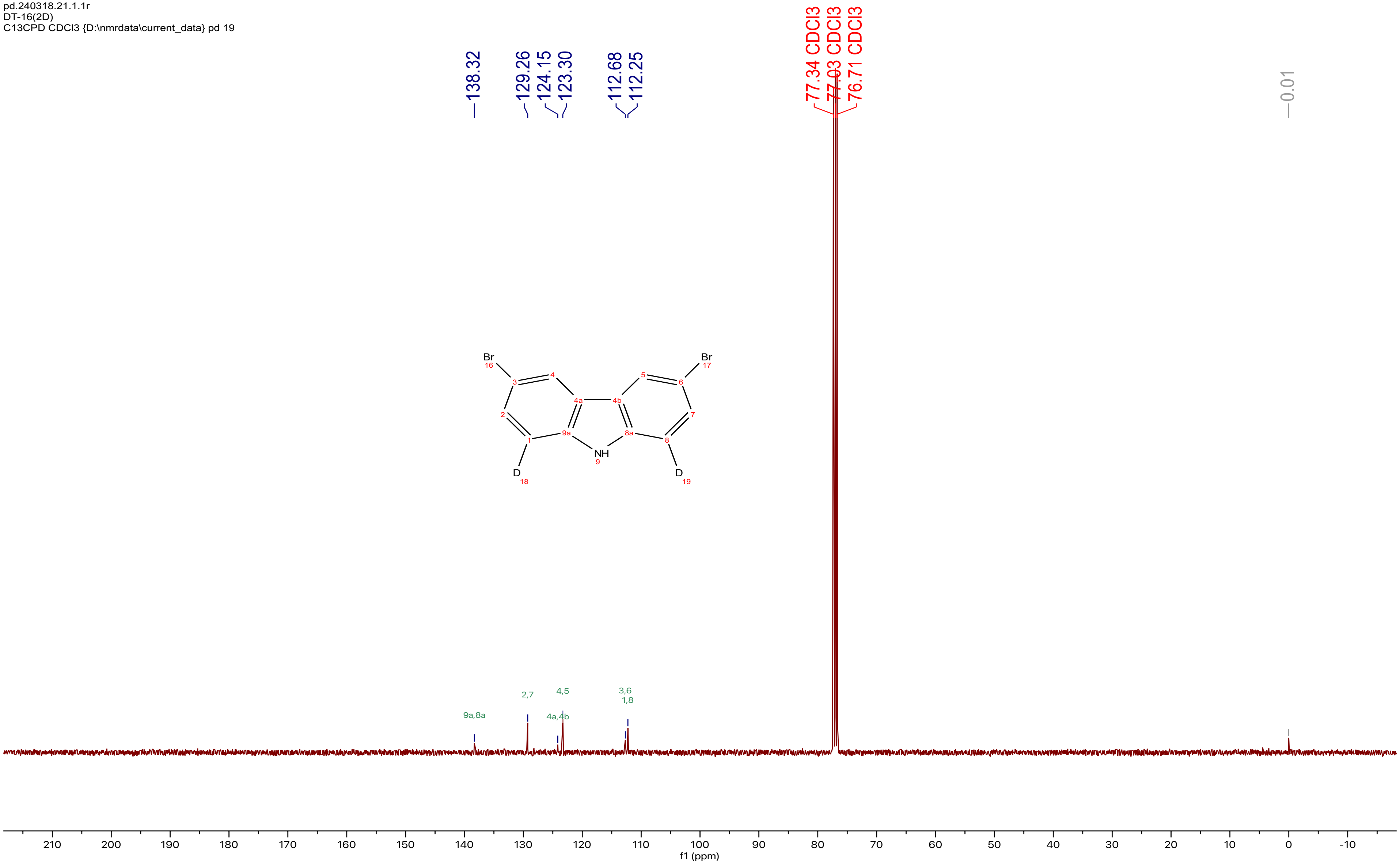

$^{13}C\{^1H\}$ NMR (101 MHz, $CDCl_3$) of 4bn

3o'

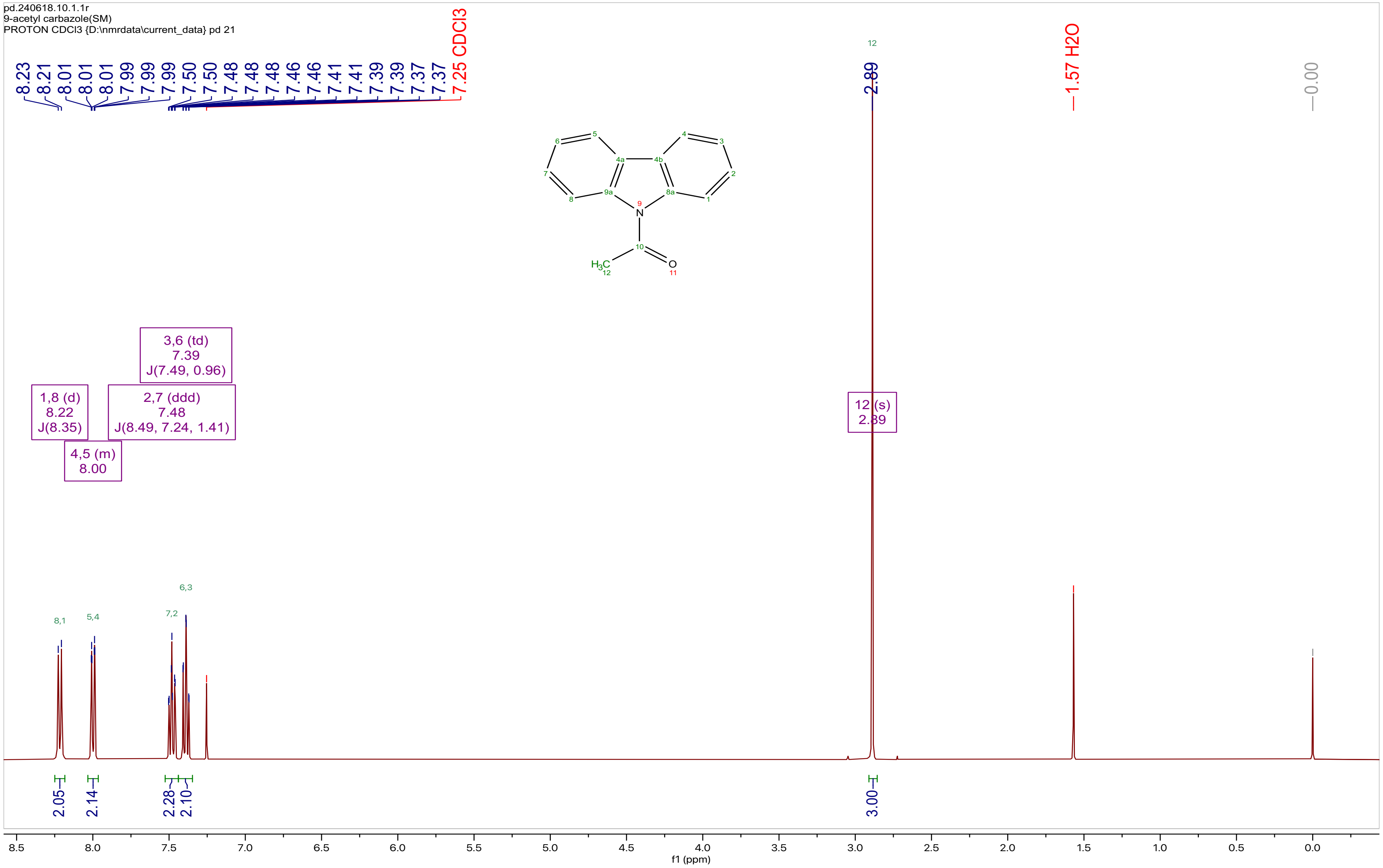

^{1}H NMR (400 MHz, $CDCl_3$) of 3o'

pd.240618.11.1.1r
9-acetyl carbazole(SM)
C13CPD CDCl3 {D:\nmrdata\current_data} pd 21

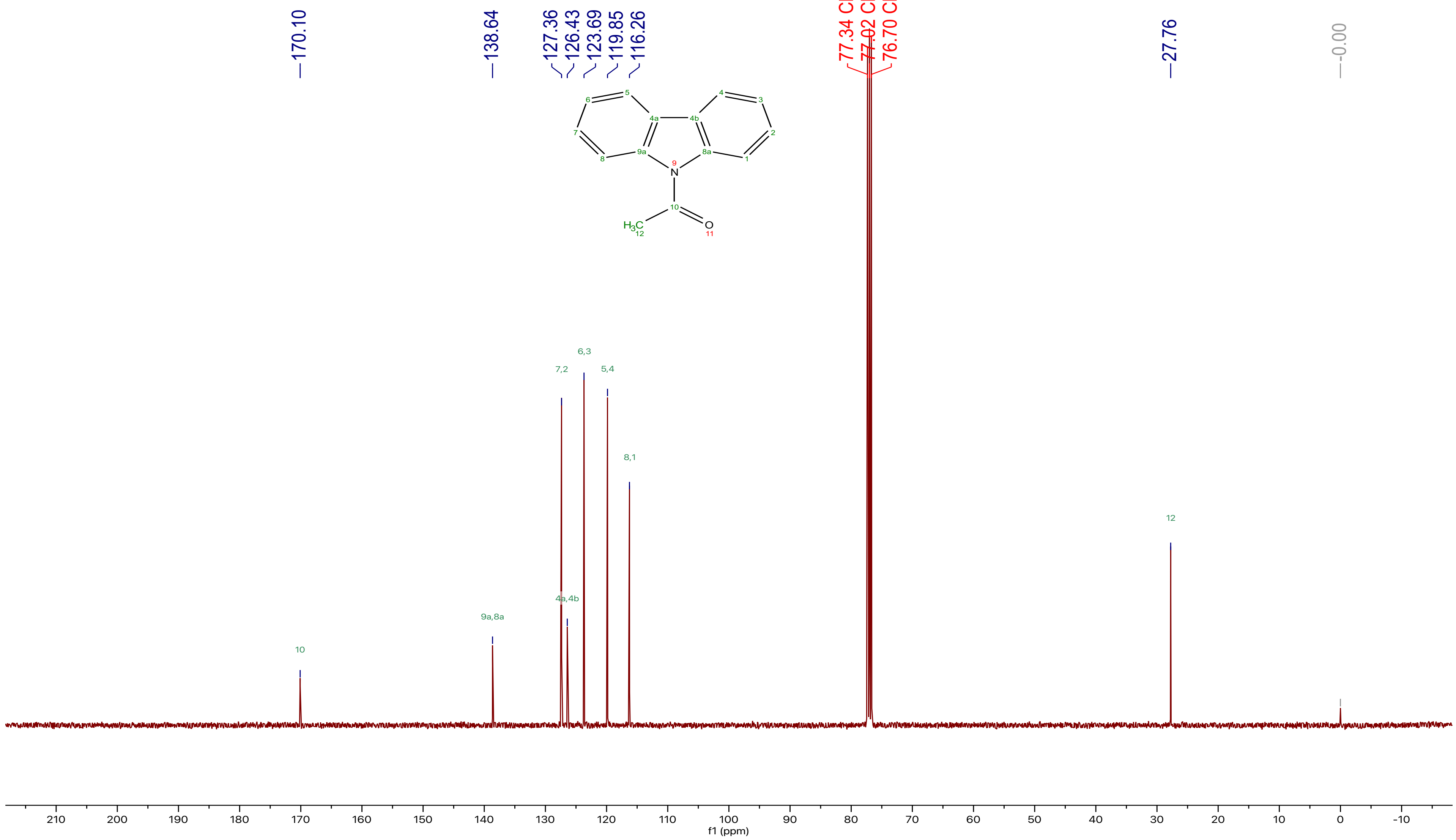

$^{13}C\{^1H\}$ NMR (101 MHz, $CDCl_3$) of 3o'

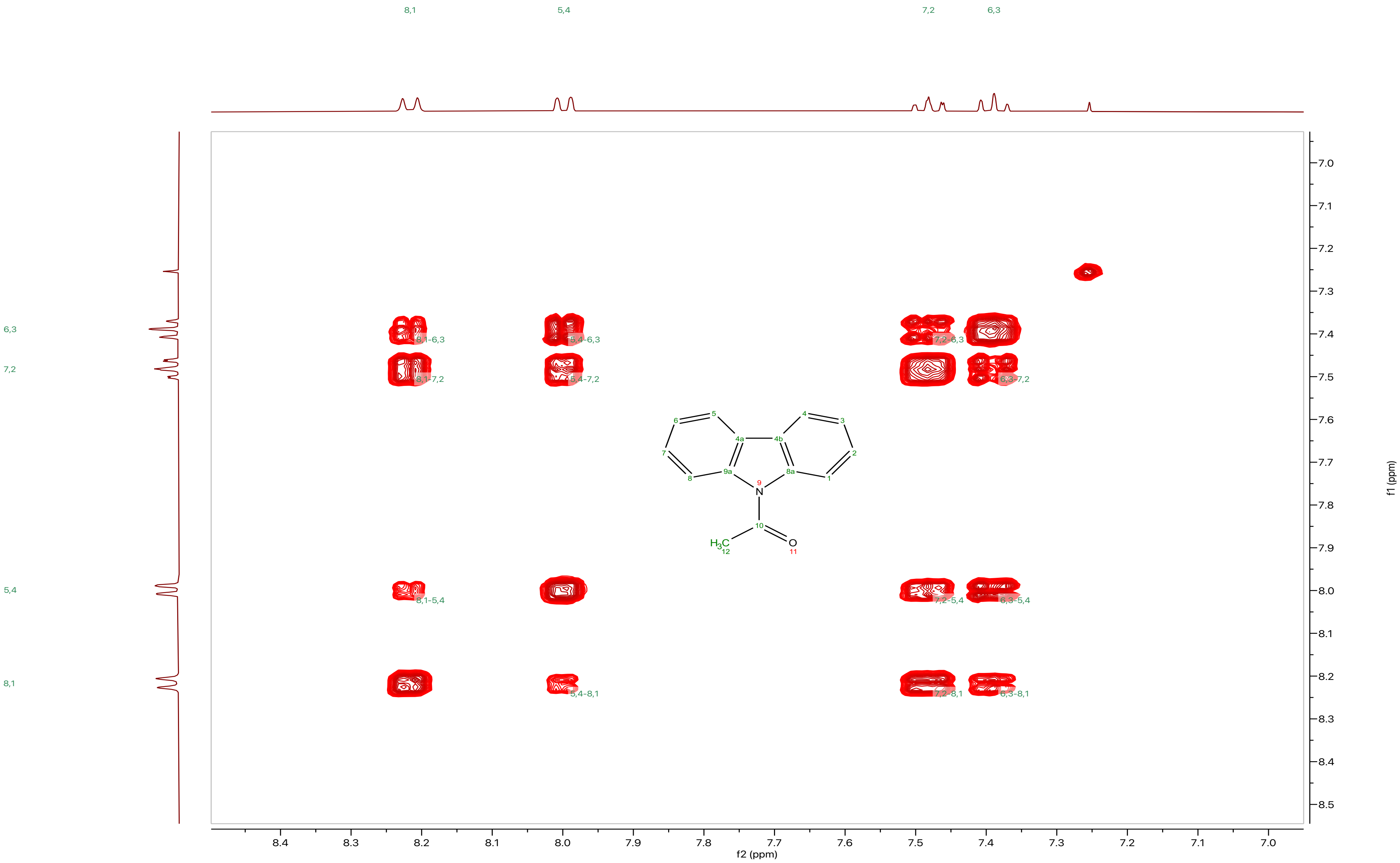

^1H-^1H COSY (400 MHz, $CDCl_3$) of 3o'

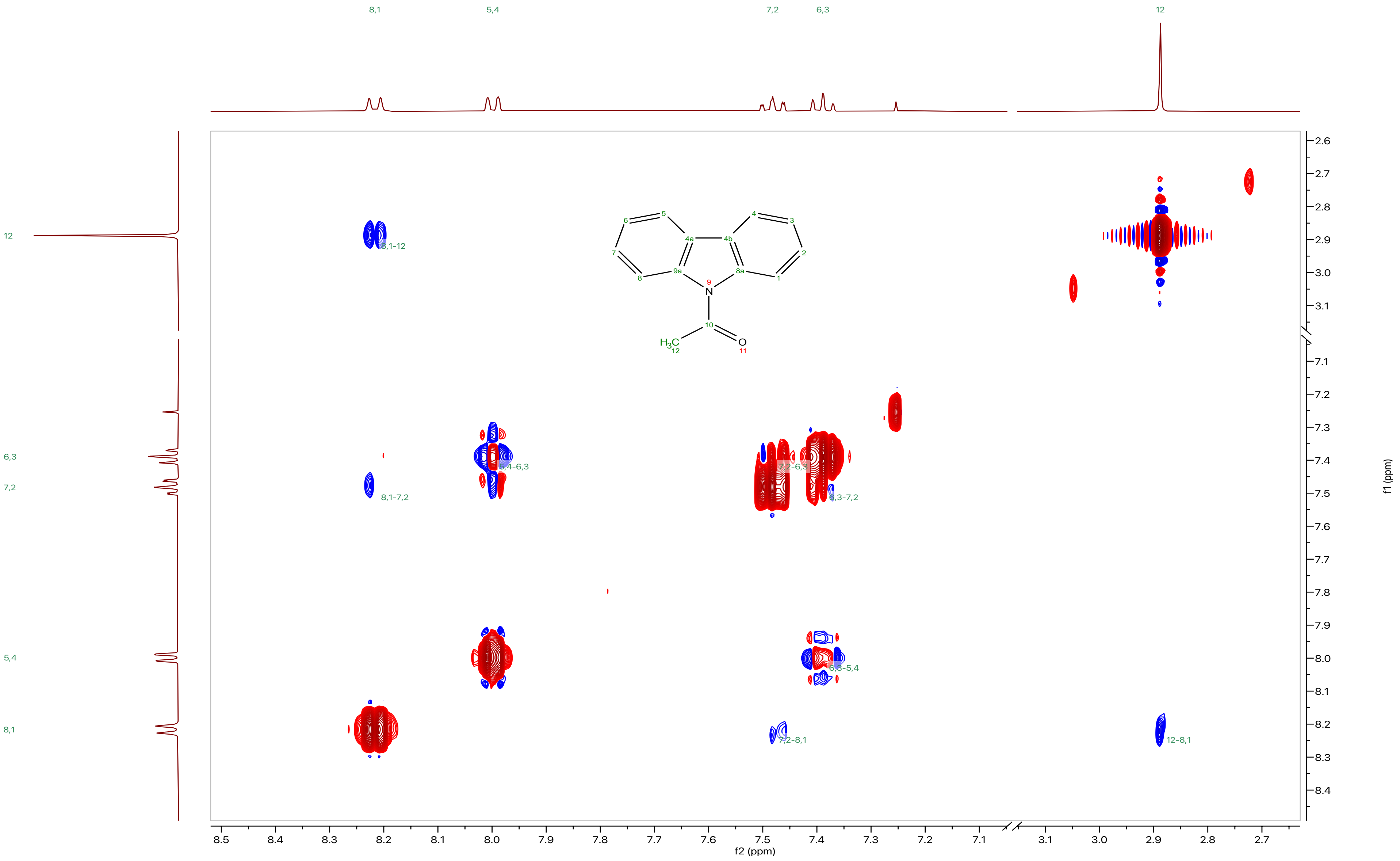

^{1}H-^{1}H NOESY (400 MHz, $CDCl_3$) of 3o'

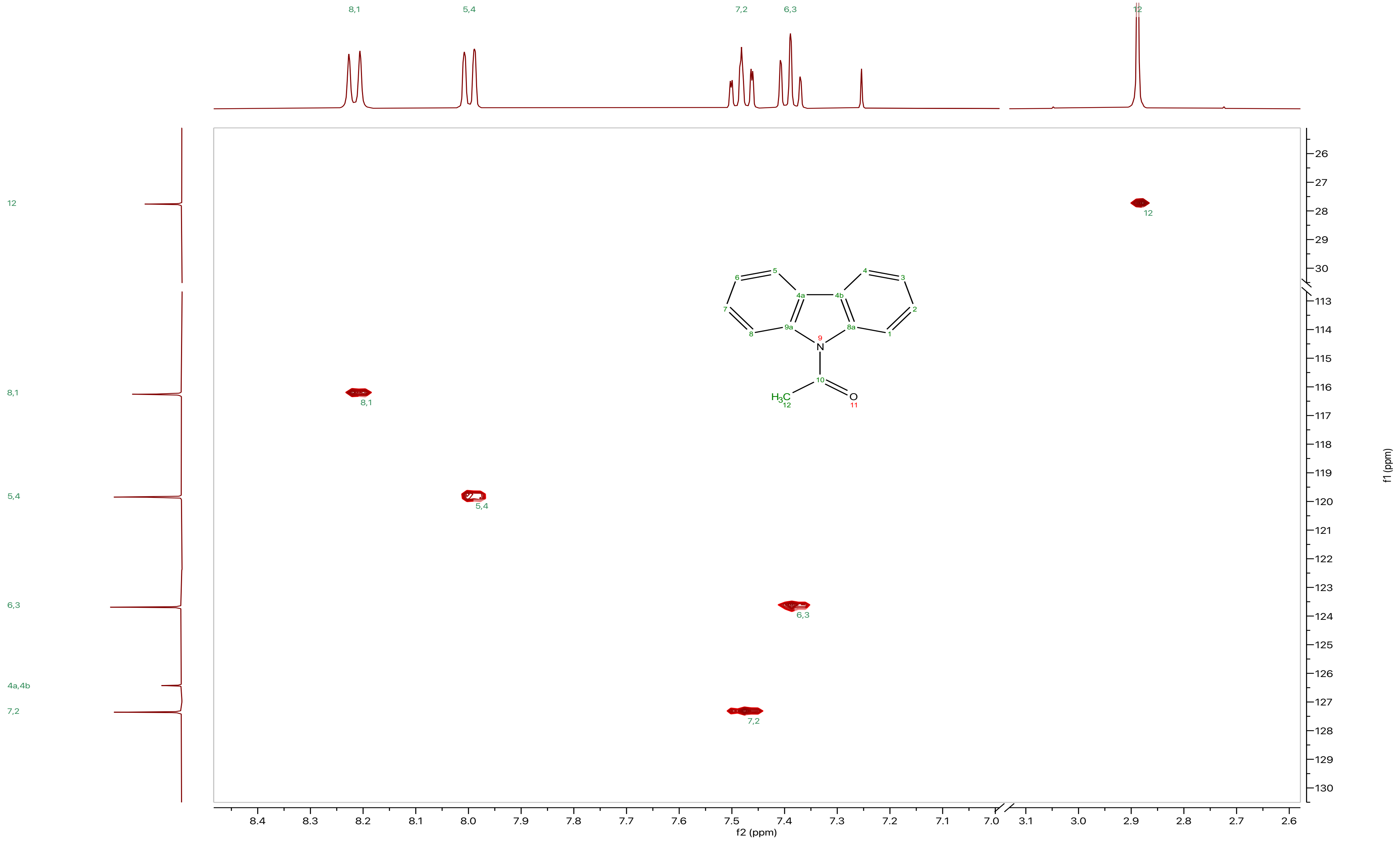

^{1}H-$^{13}C\{^{1}H\}$ HSQC NMR (400/101 MHz, $CDCl_3$) of 3o'

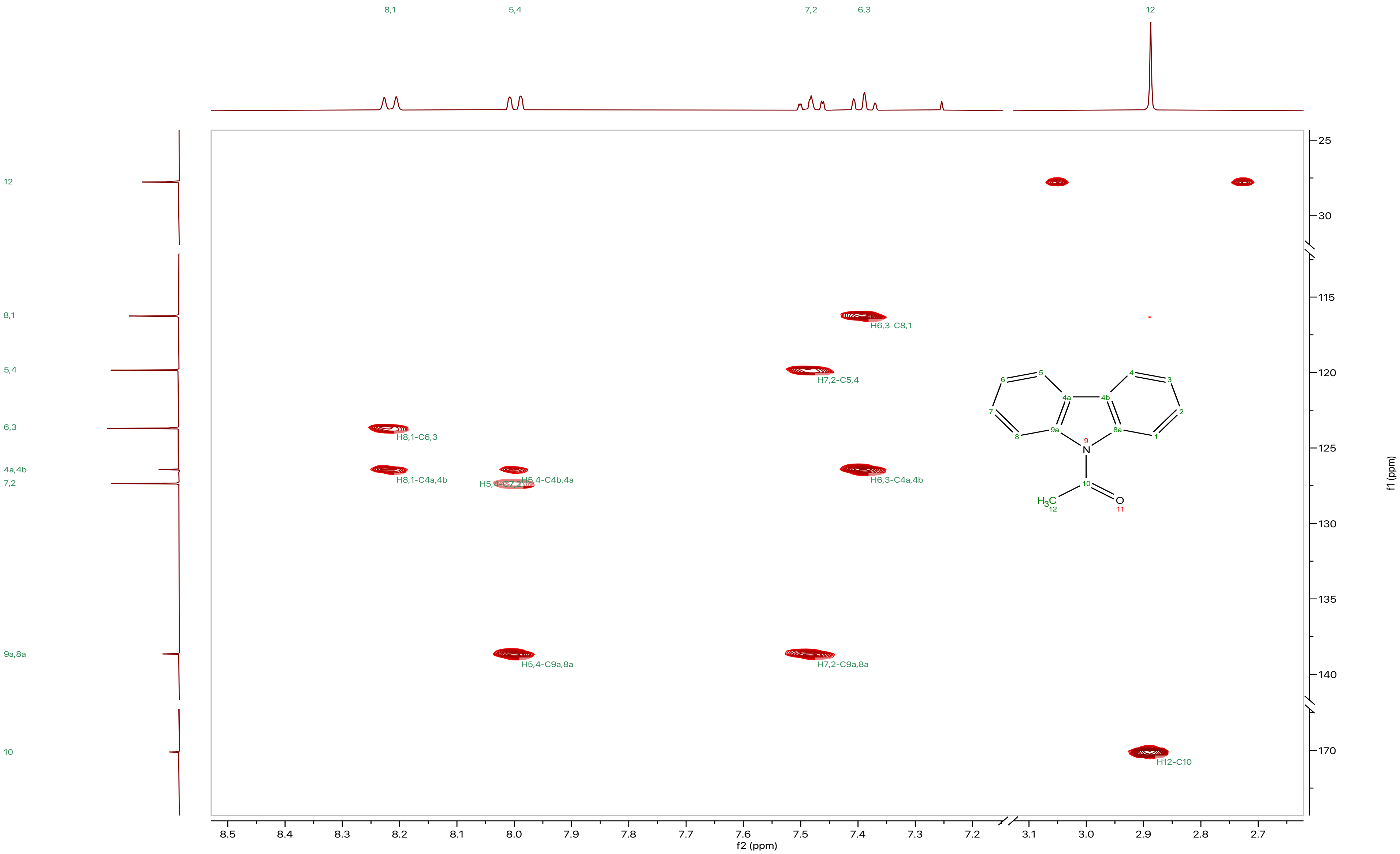

^{1}H-$^{13}C\{^{1}H\}$ HMBC NMR (400/101 MHz, $CDCl_3$) of 3o'

4ao'

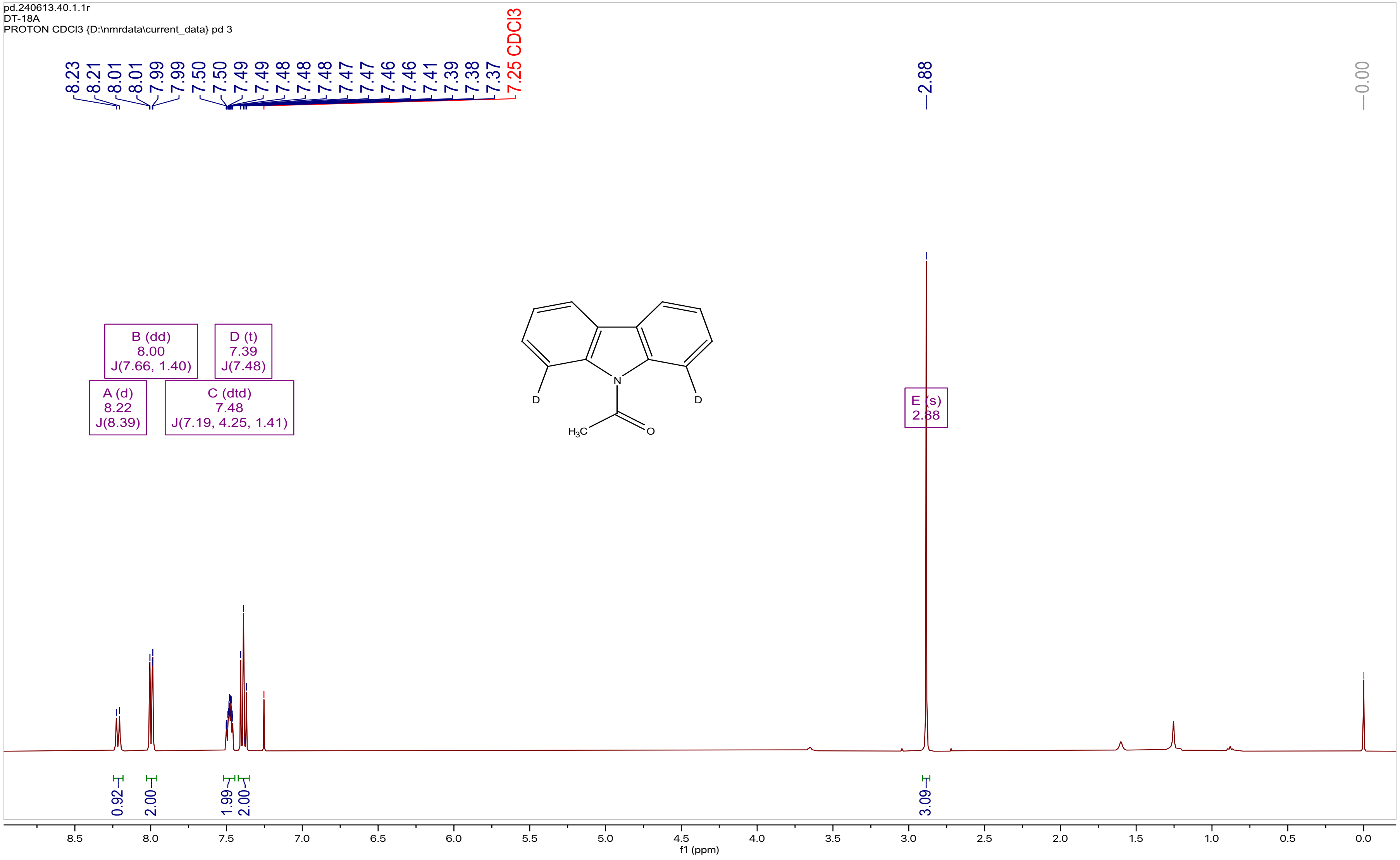

^{1}H NMR (400 MHz, $CDCl_3$) of 4ao'

pd.240613.41.1.1r
DT-18A
C13CPD CDCl3 {D:\nmrdata\current_data} pd 3

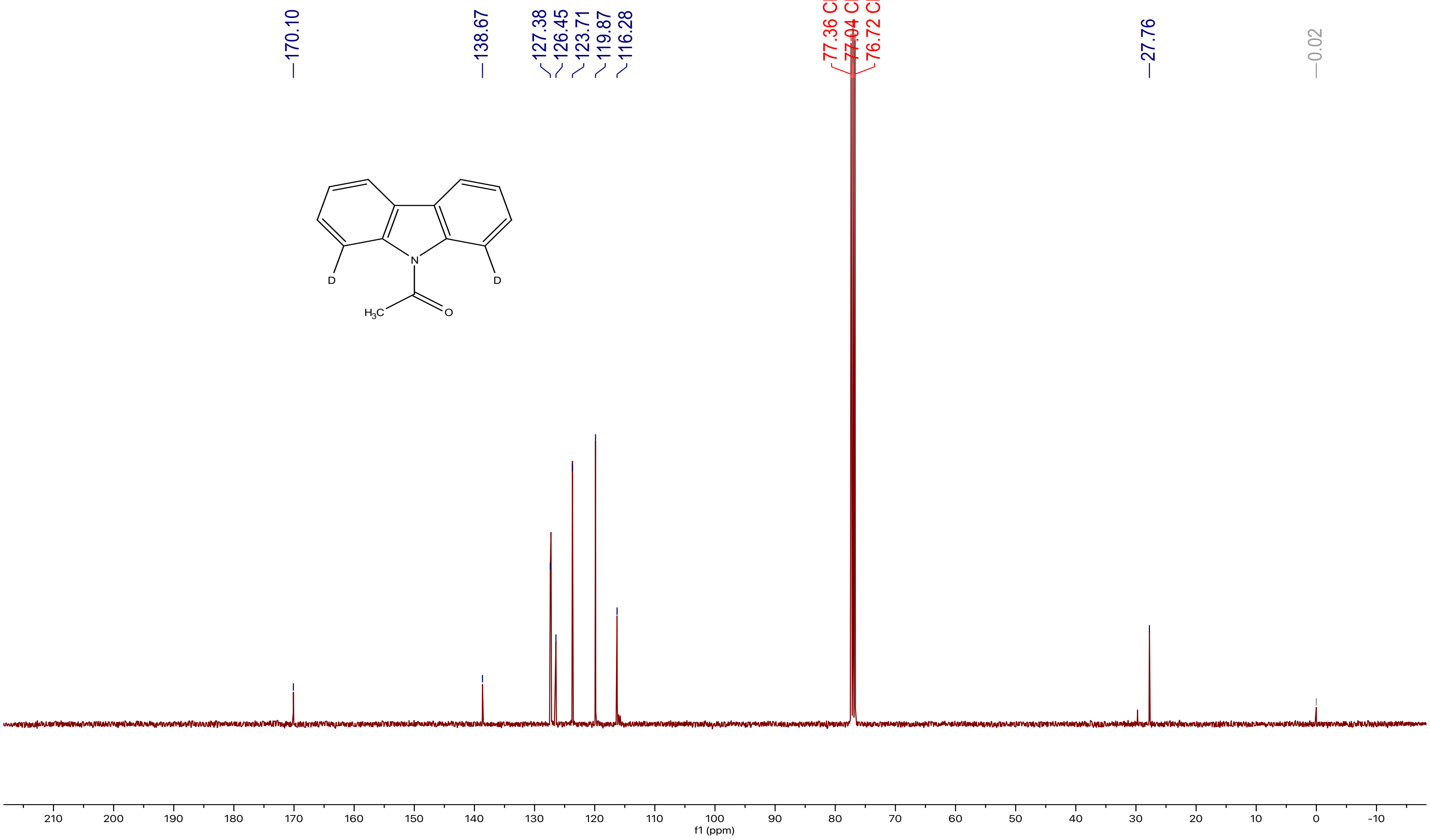

$^{13}C\{^{1}H\}$ NMR (101 MHz, $CDCl_3$) of 4ao'

Carbazole = **3a'**

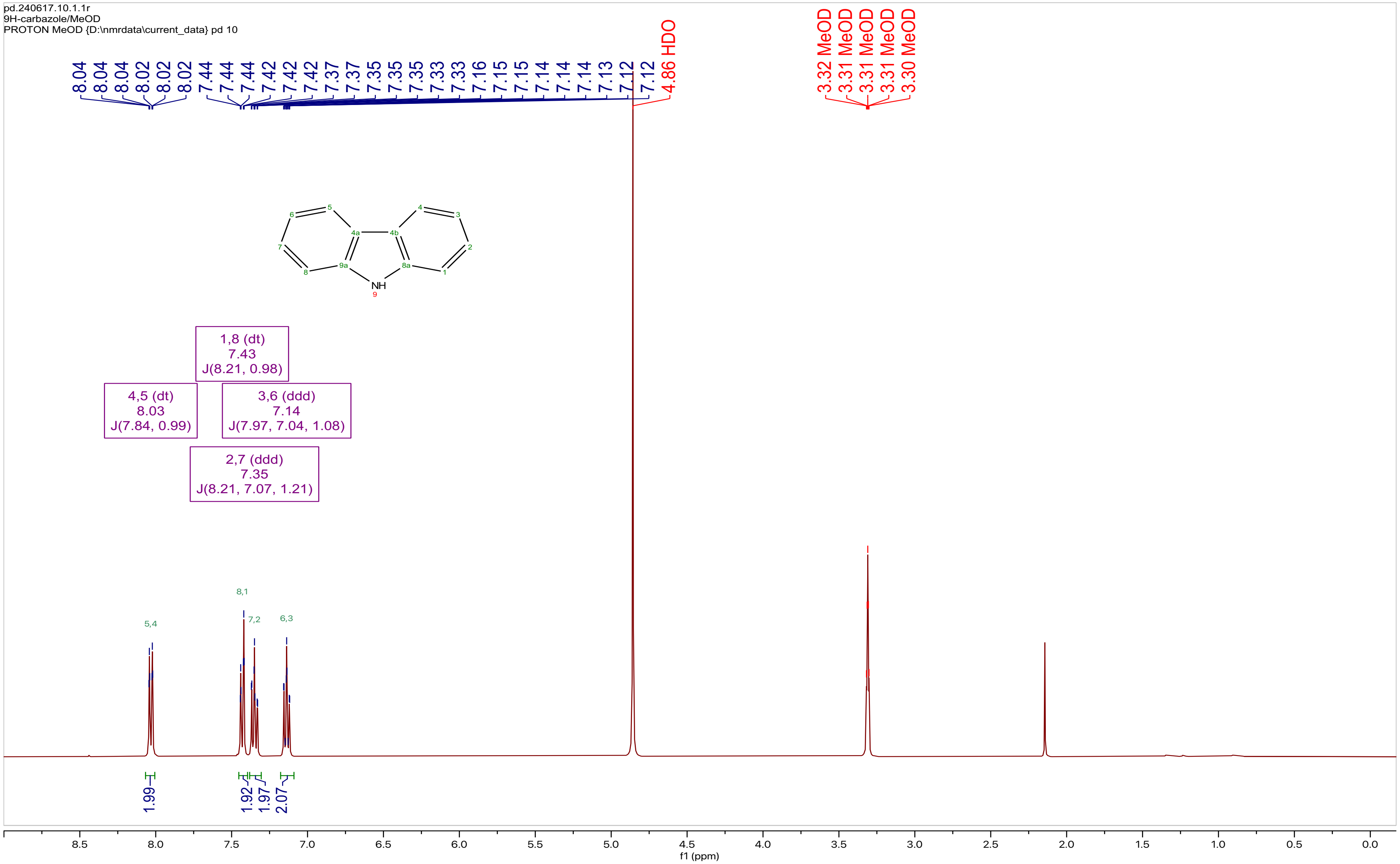

^{1}H NMR (400 MHz, MeOD) of 3a'

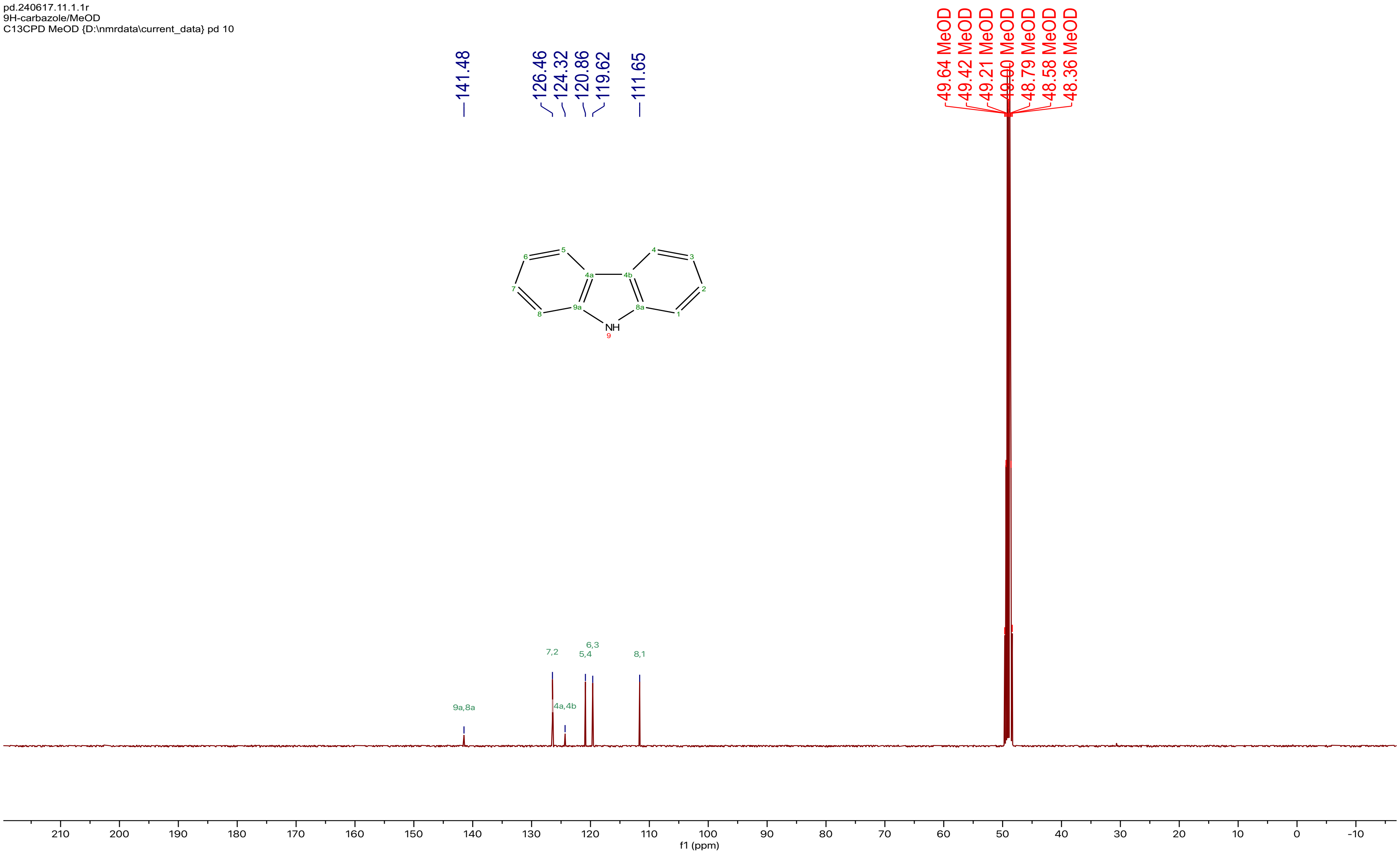

$^{13}C\{^1H\}$ NMR (101 MHz, MeOD) of 3a'

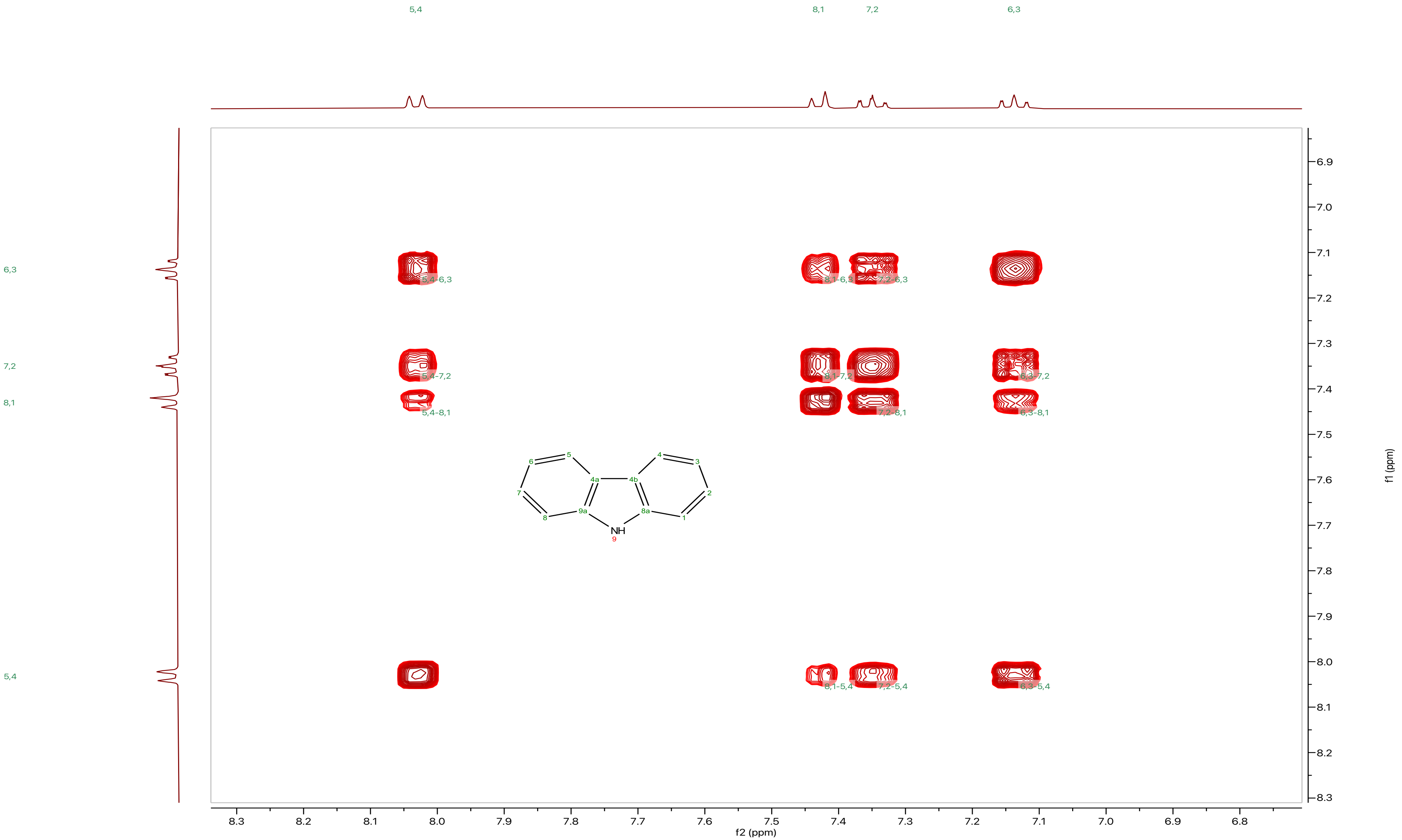

^{1}H-^{1}H COSY (400 MHz, MeOD) of 3a’

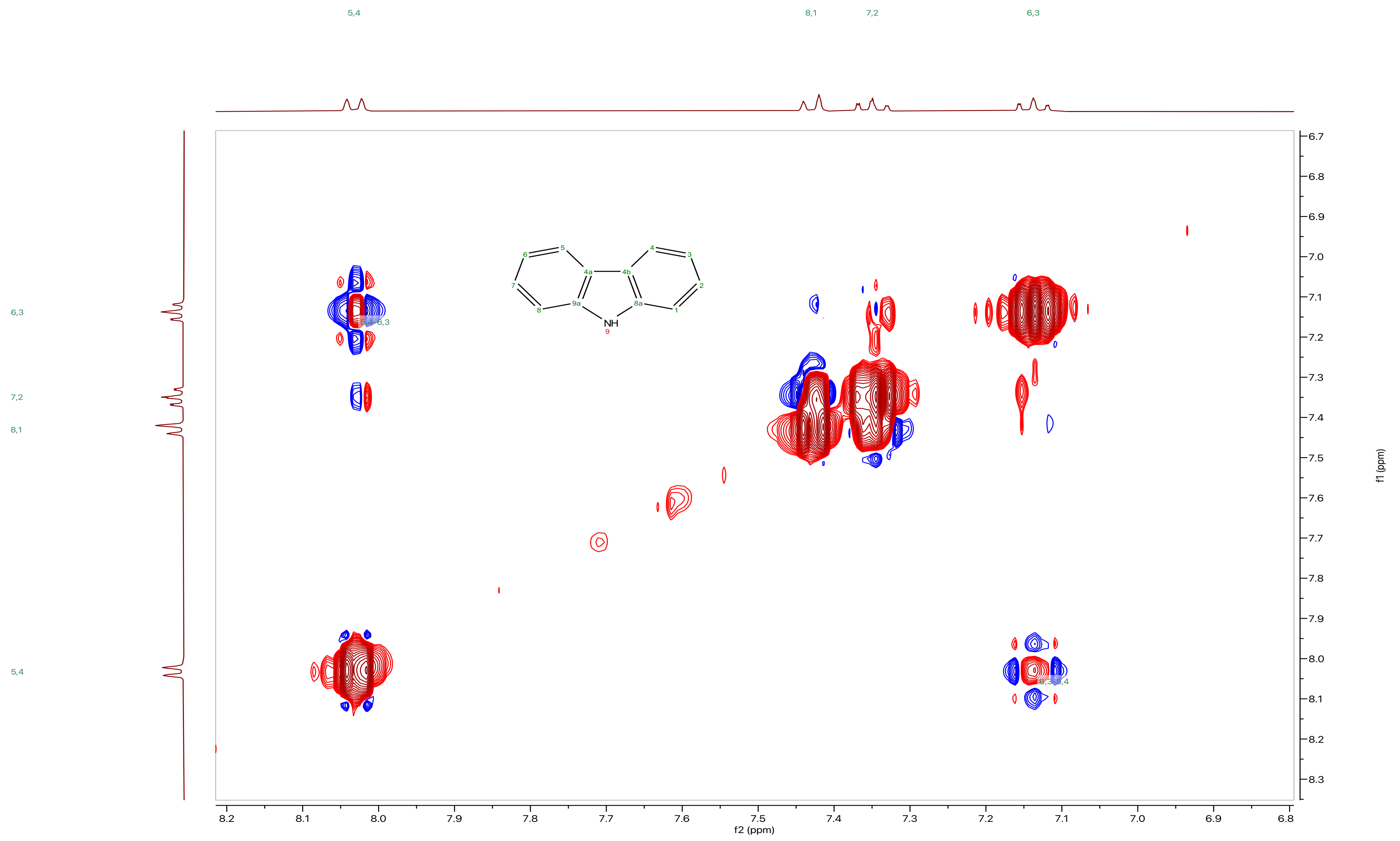

^{1}H-^{1}H NOESY (400 MHz, MeOD) of 3a'

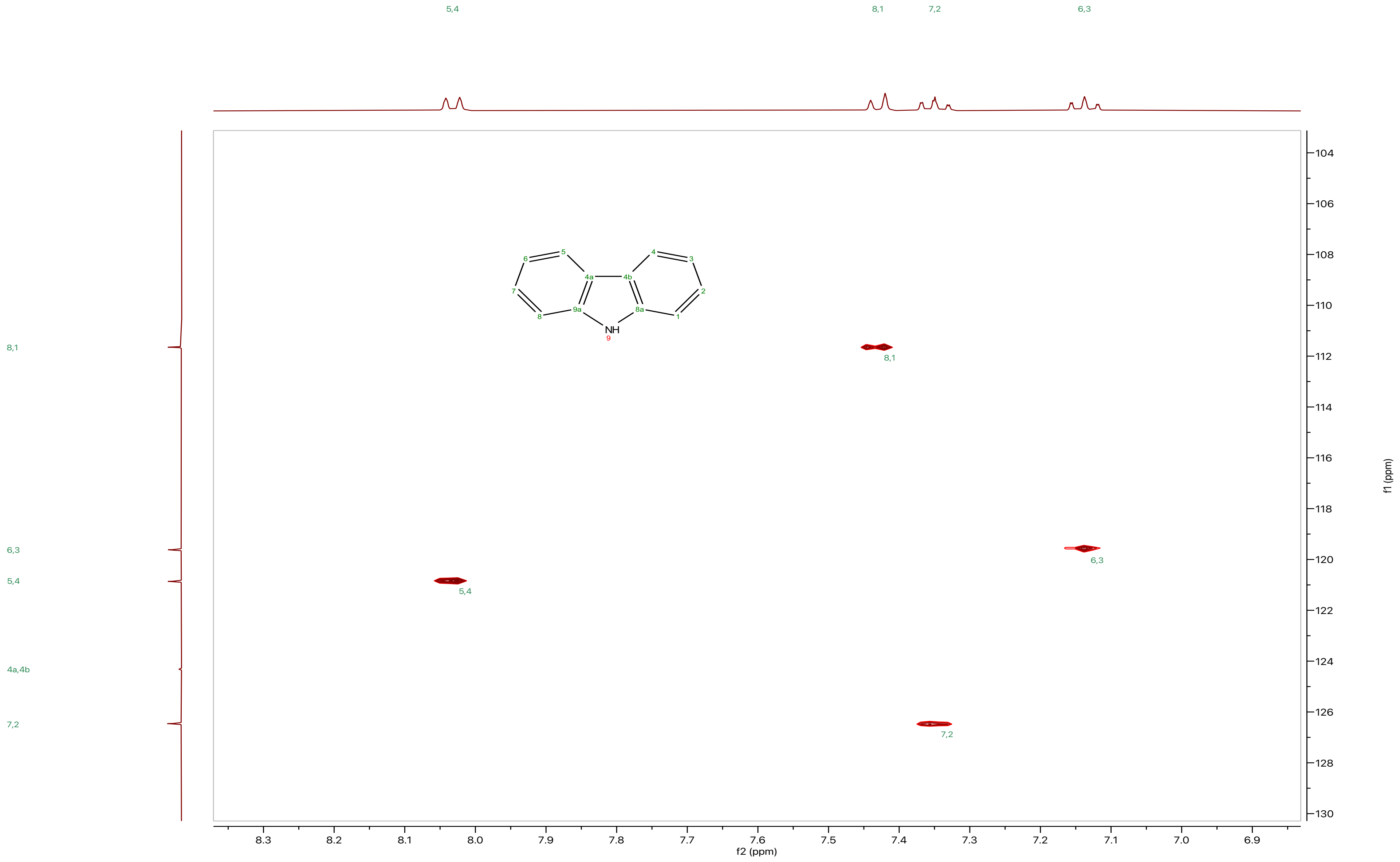

^{1}H-$^{13}C\{^{1}H\}$ HSQC NMR (400/101 MHz, MeOD) of 3a'

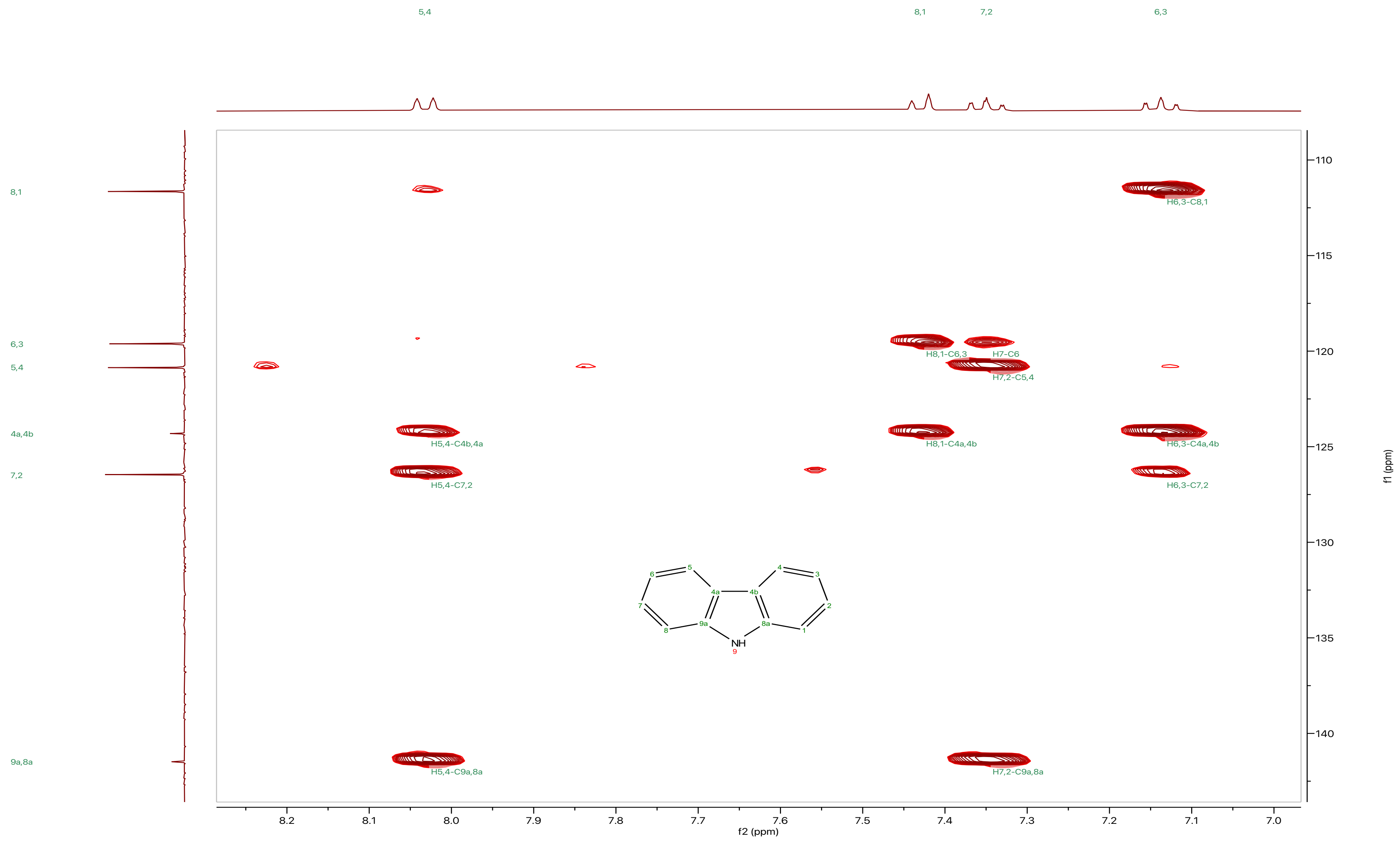

^{1}H-^{13}C{^{1}H} HMBC NMR (400/101 MHz, MeOD) of 3a’

4bo'

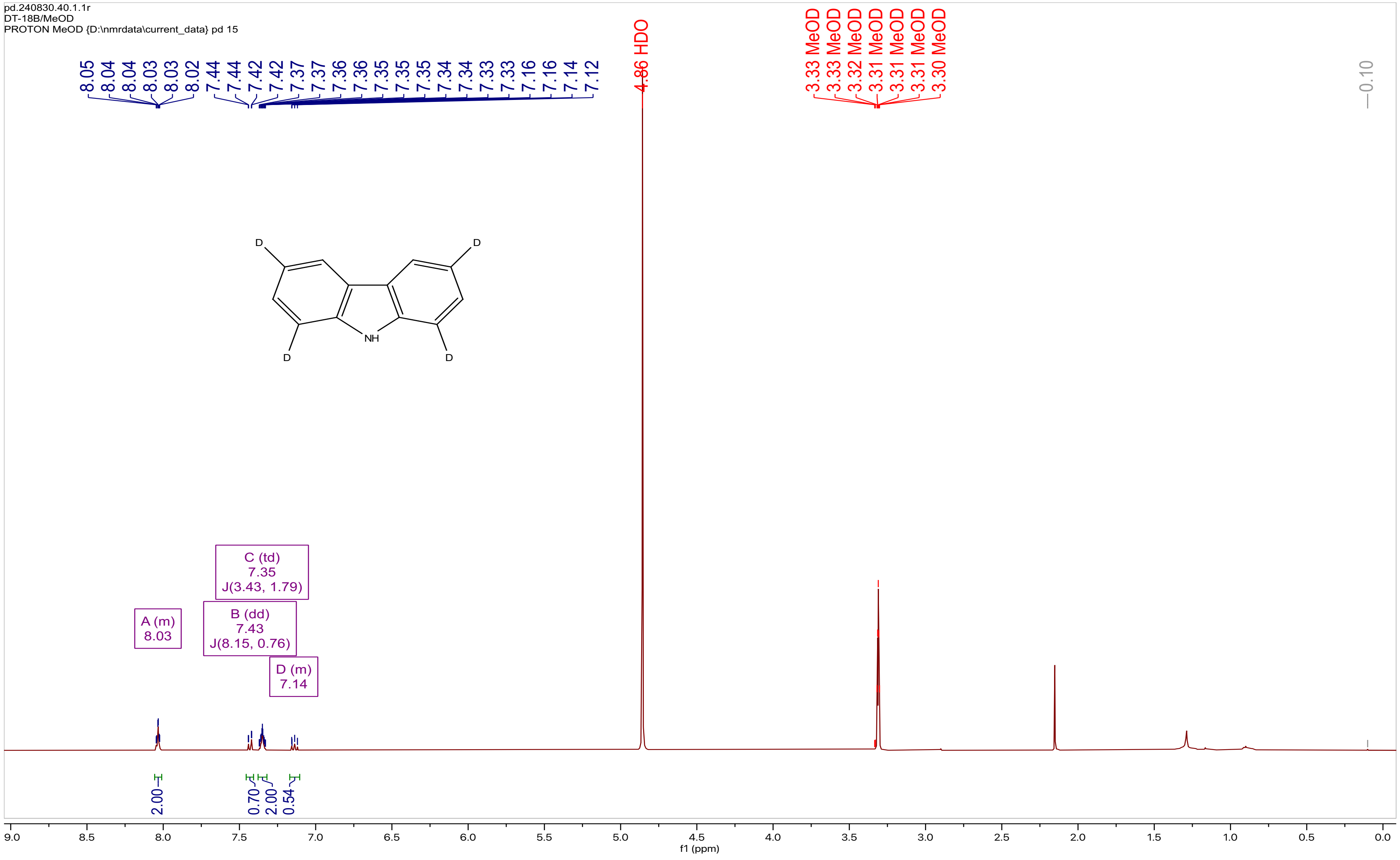

^{1}H NMR (400 MHz, MeOD) of 4bo'

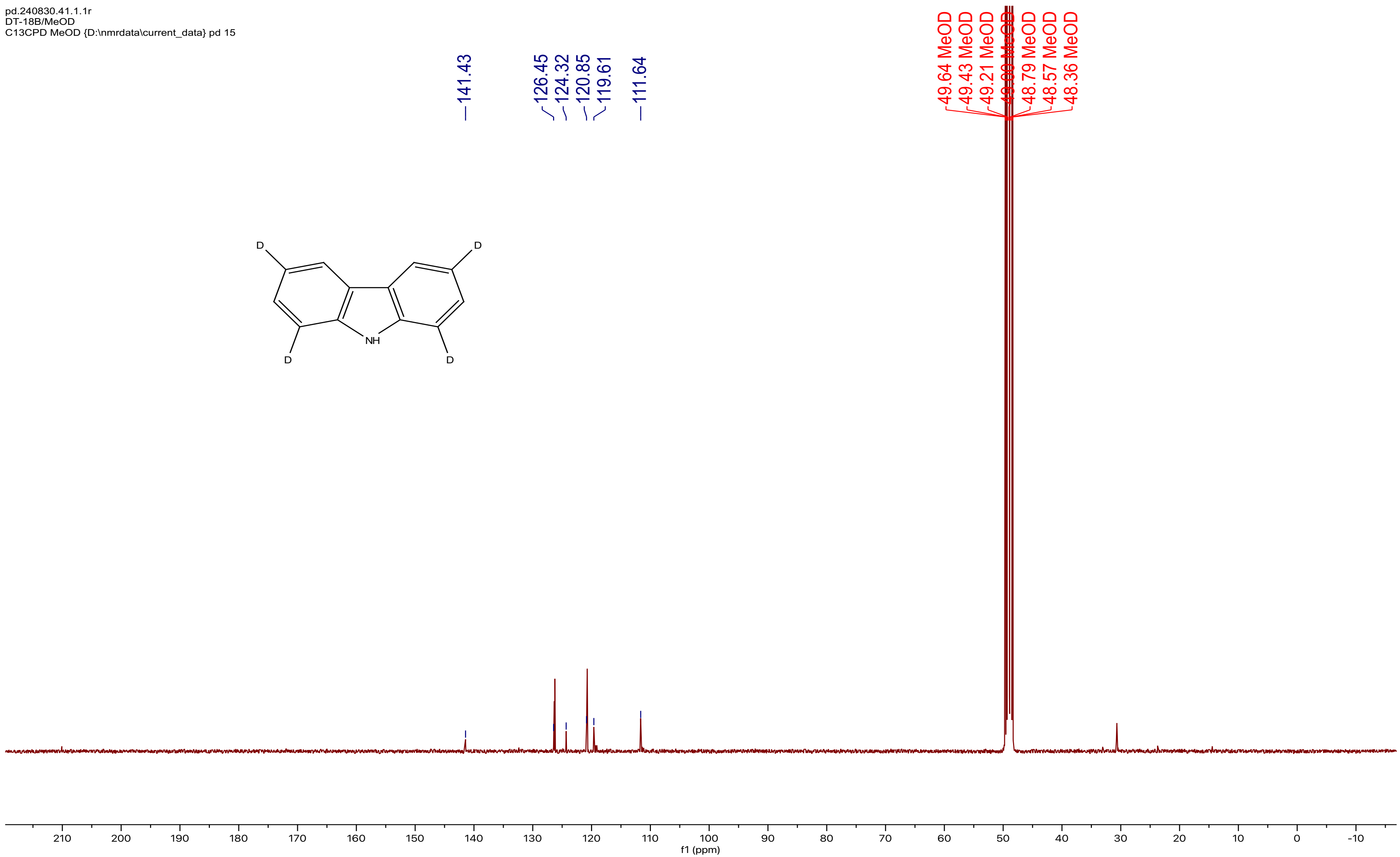

$^{13}C\{^1H\}$ NMR (101 MHz, MeOD) of 4bo'

3p

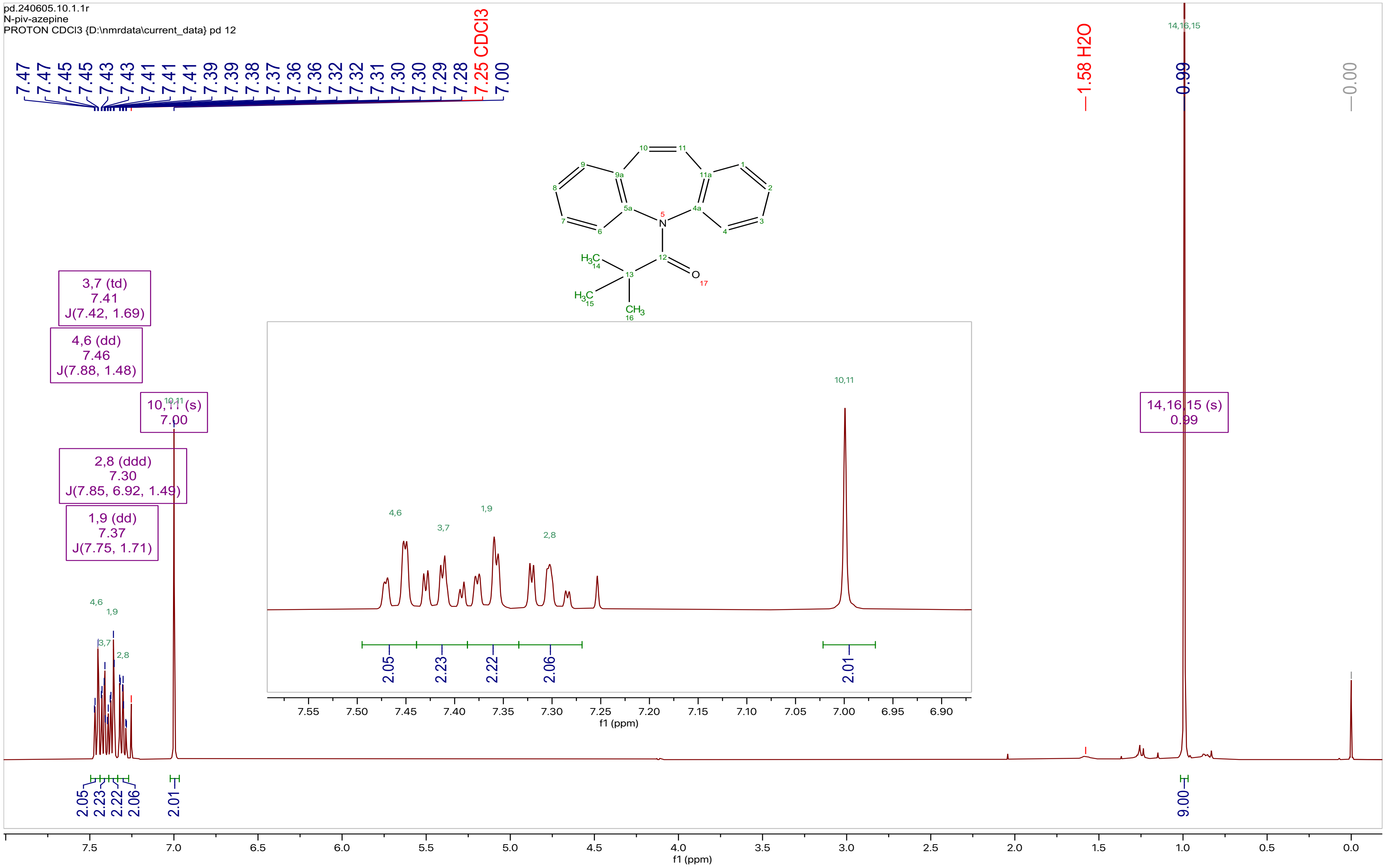

^1H NMR (400 MHz, $CDCl_3$) of 3p

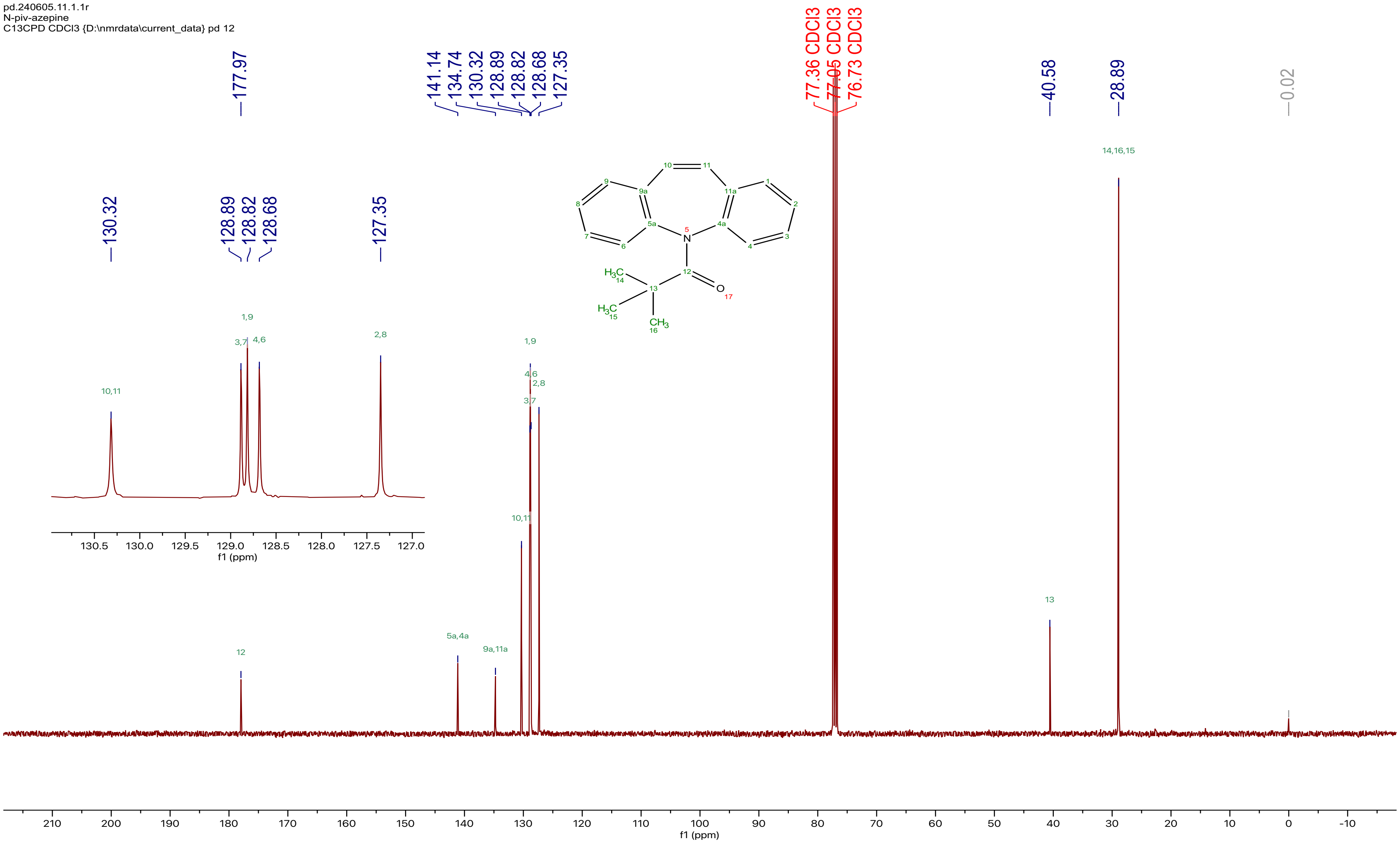

$^{13}C\{^1H\}$ NMR (101 MHz, $CDCl_3$) of 3p

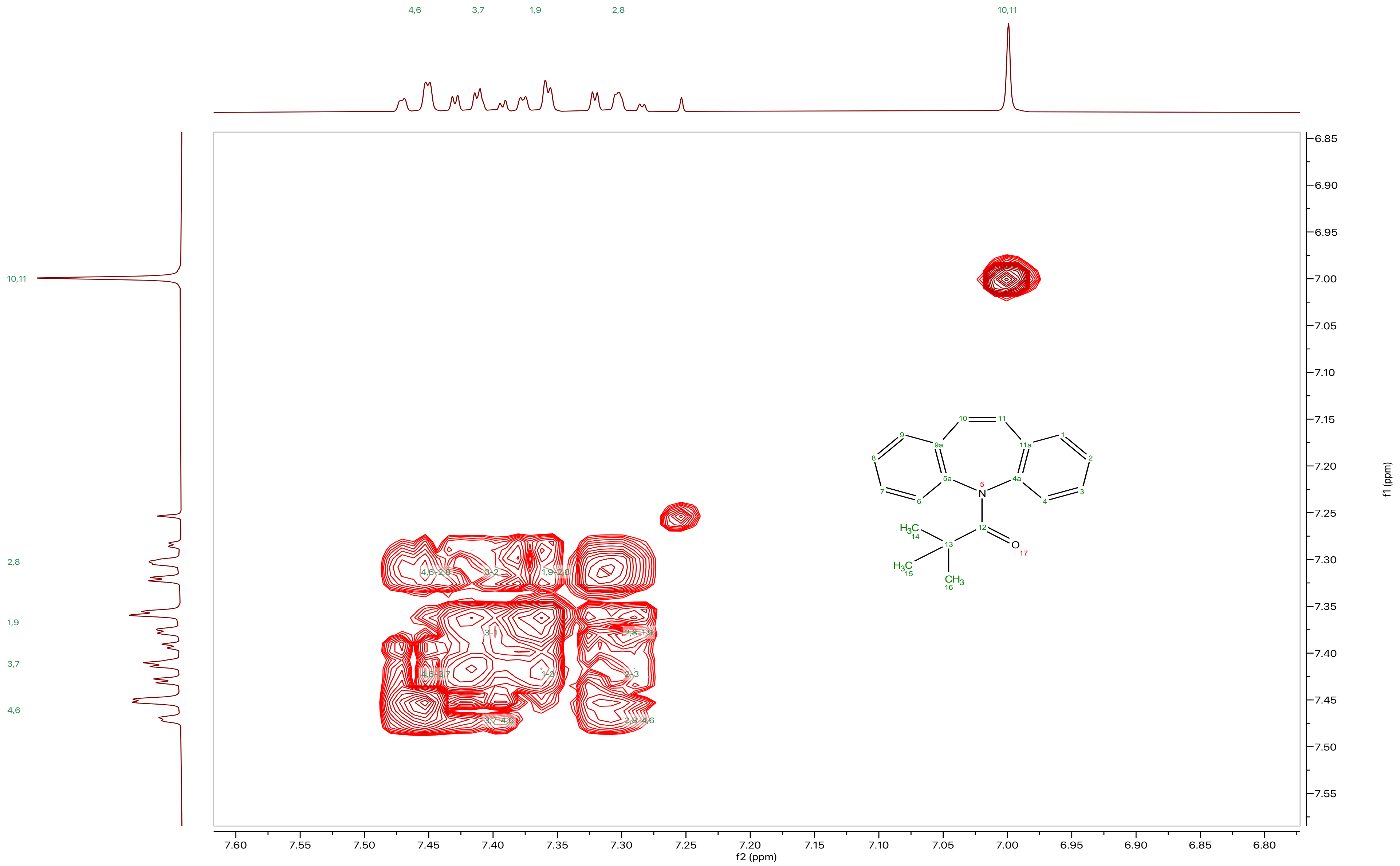

^1H-^1H COSY (400 MHz, $CDCl_3$) of 3p

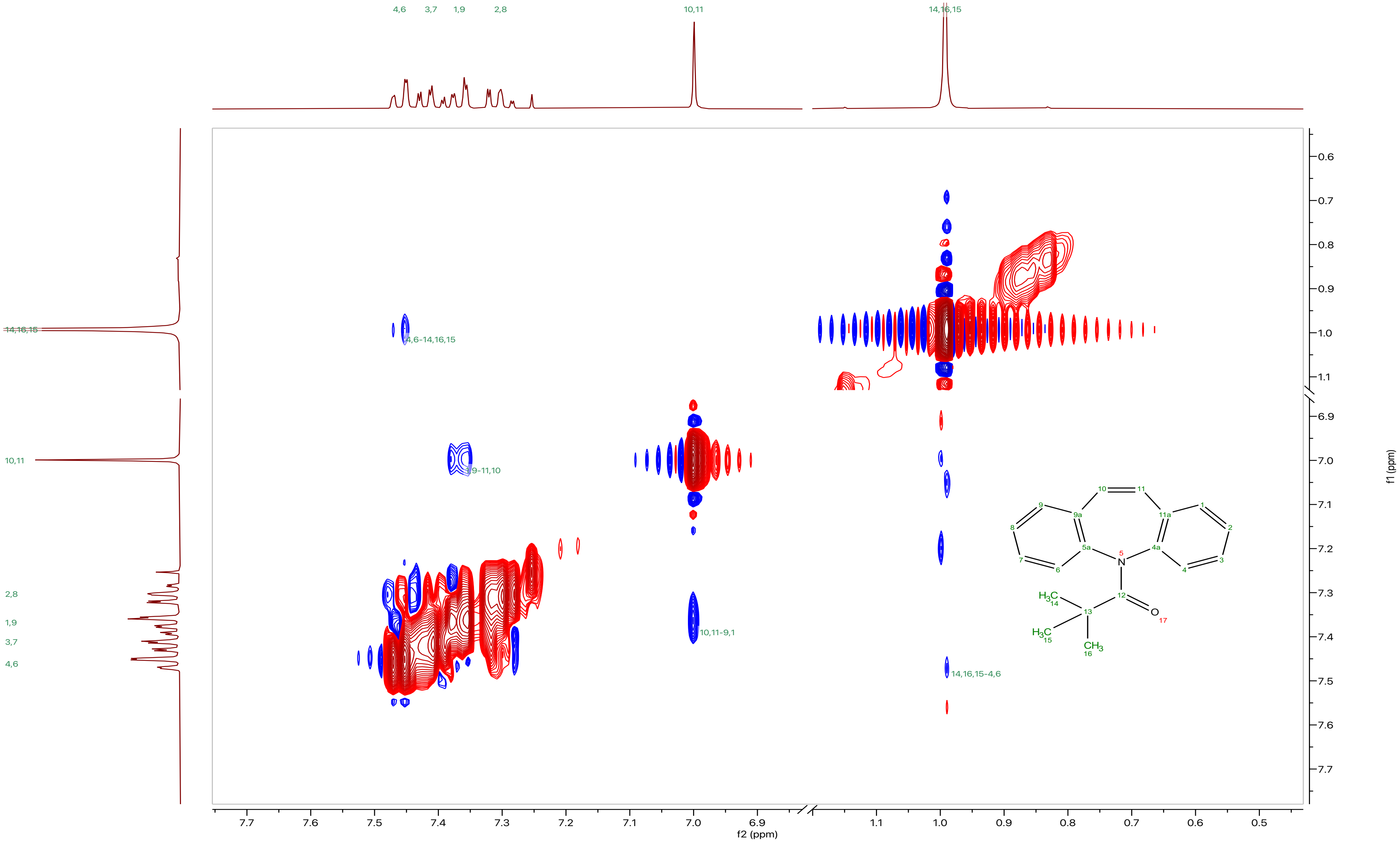

^{1}H-^{1}H NOESY (400 MHz, $CDCl_3$) of 3p

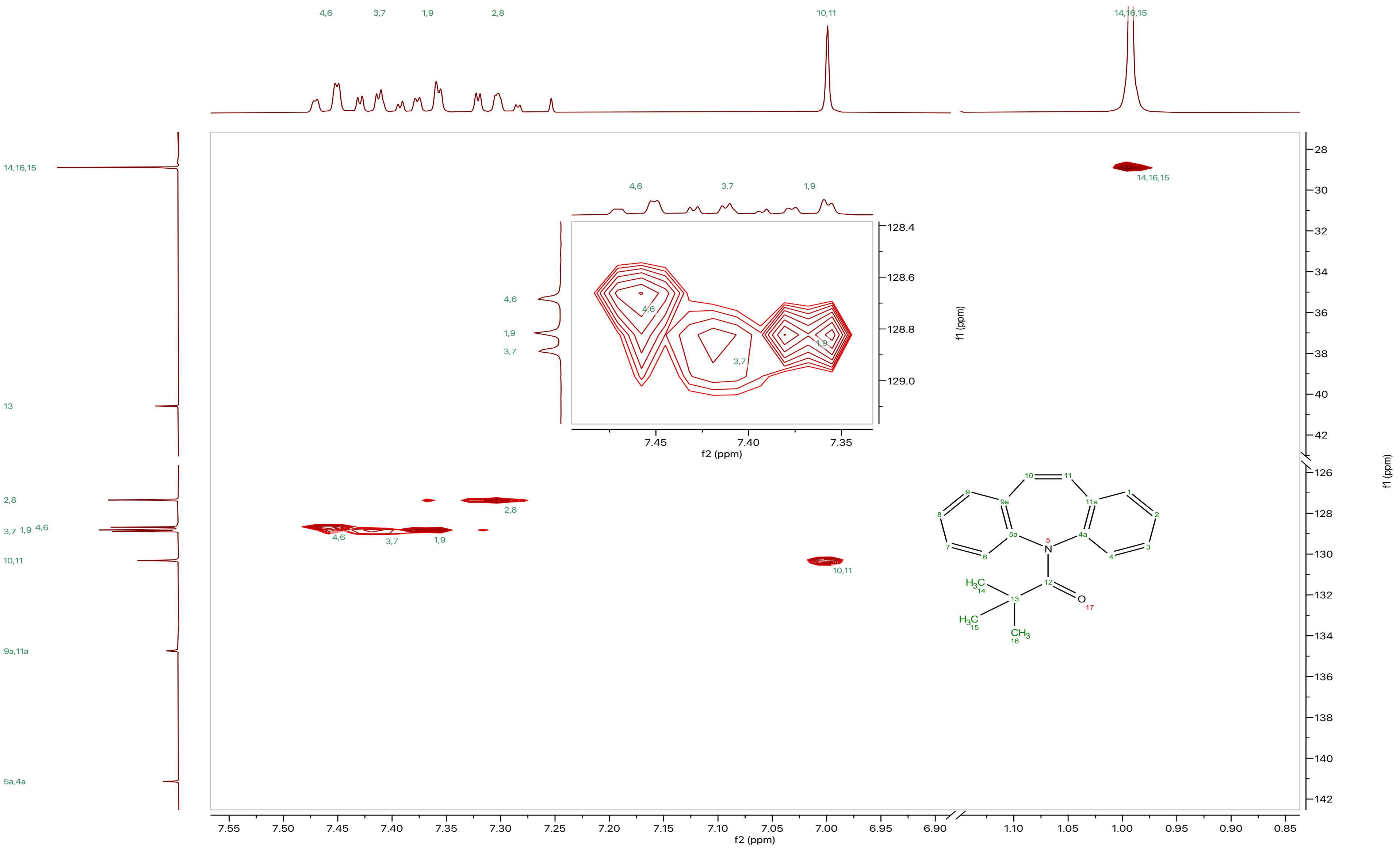

^{1}H-$^{13}C\{^{1}H\}$ HSQC NMR (400/101 MHz, $CDCl_3$) of 3p

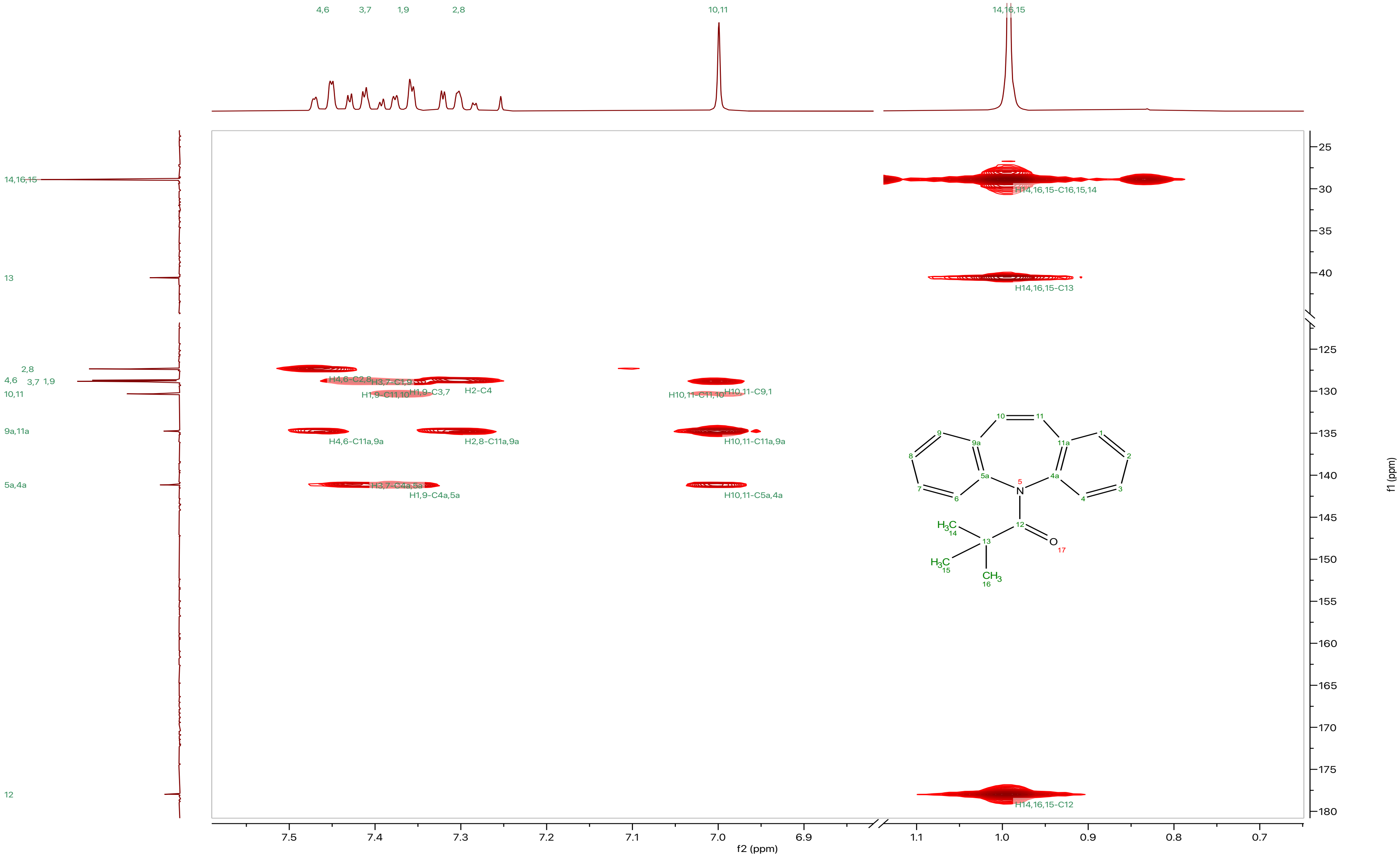

^{1}H-^{13}C{^{1}H} HMBC NMR (400/101 MHz, CDCl$_3$) of 3p

4ap

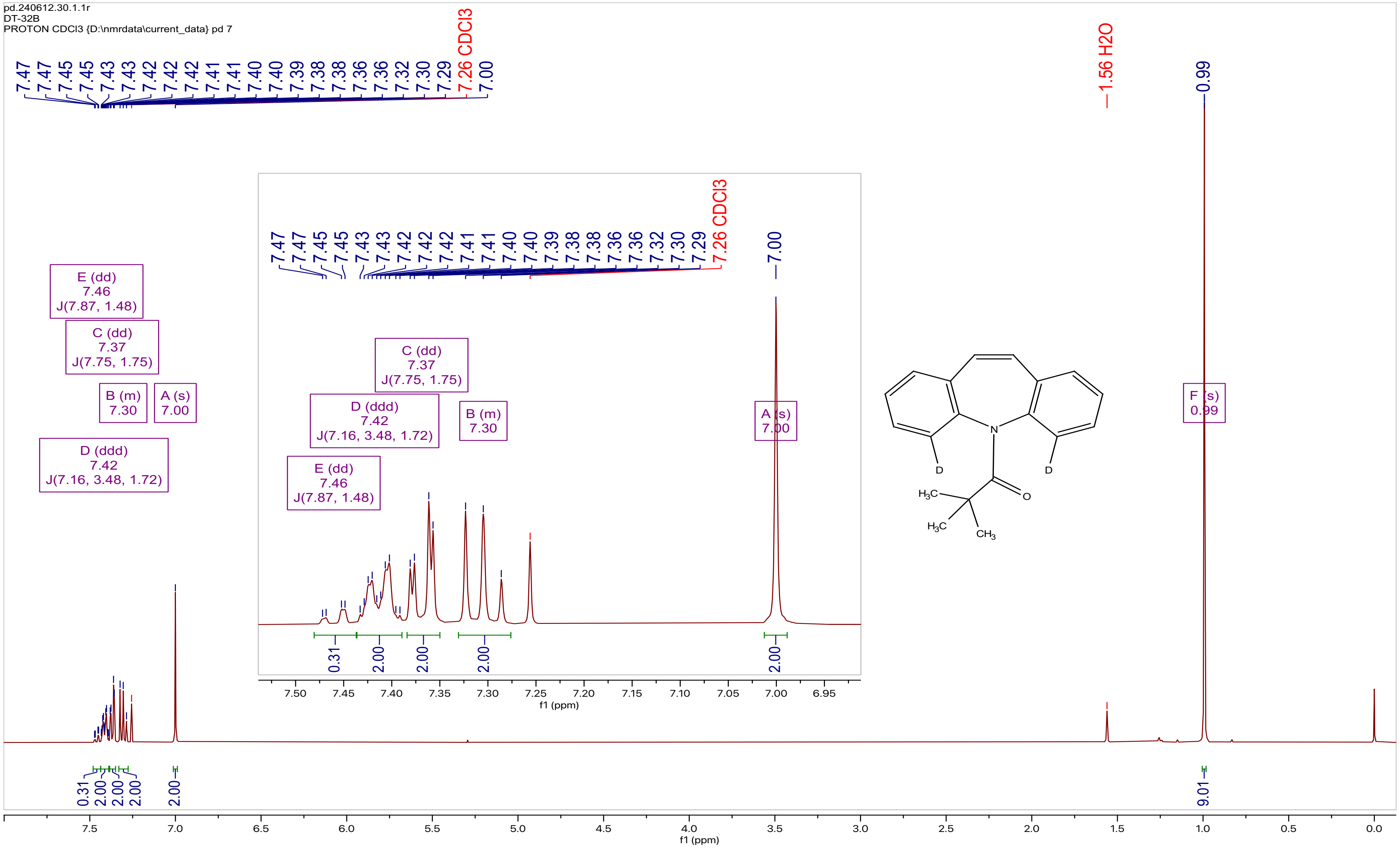

^{1}H NMR (400 MHz, $CDCl_3$) of 4ap

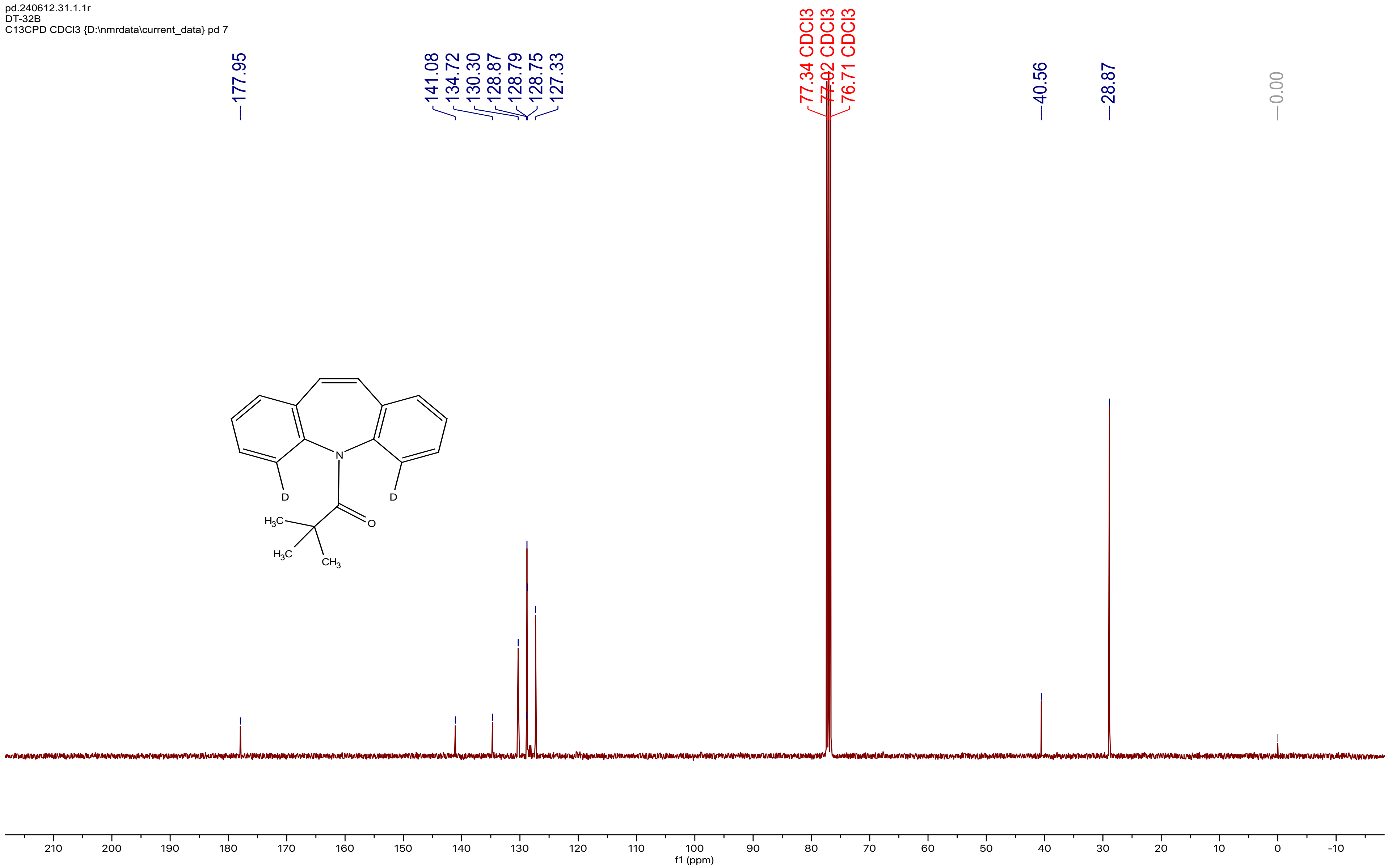

$^{13}C\{^{1}H\}$ NMR (101 MHz, $CDCl_3$) of 4ap

3p'

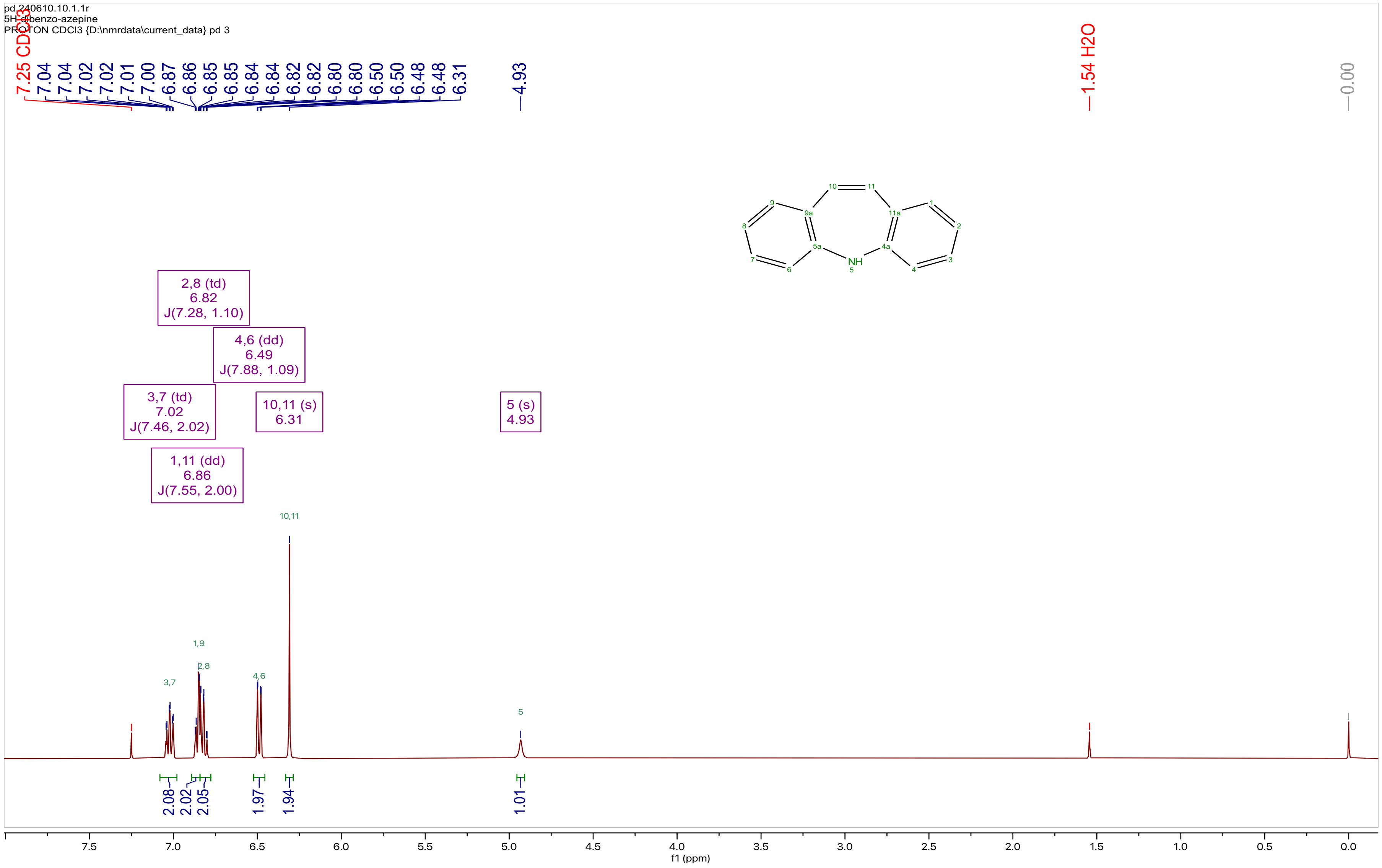

^{1}H NMR (400 MHz, $CDCl_3$) of 3p'

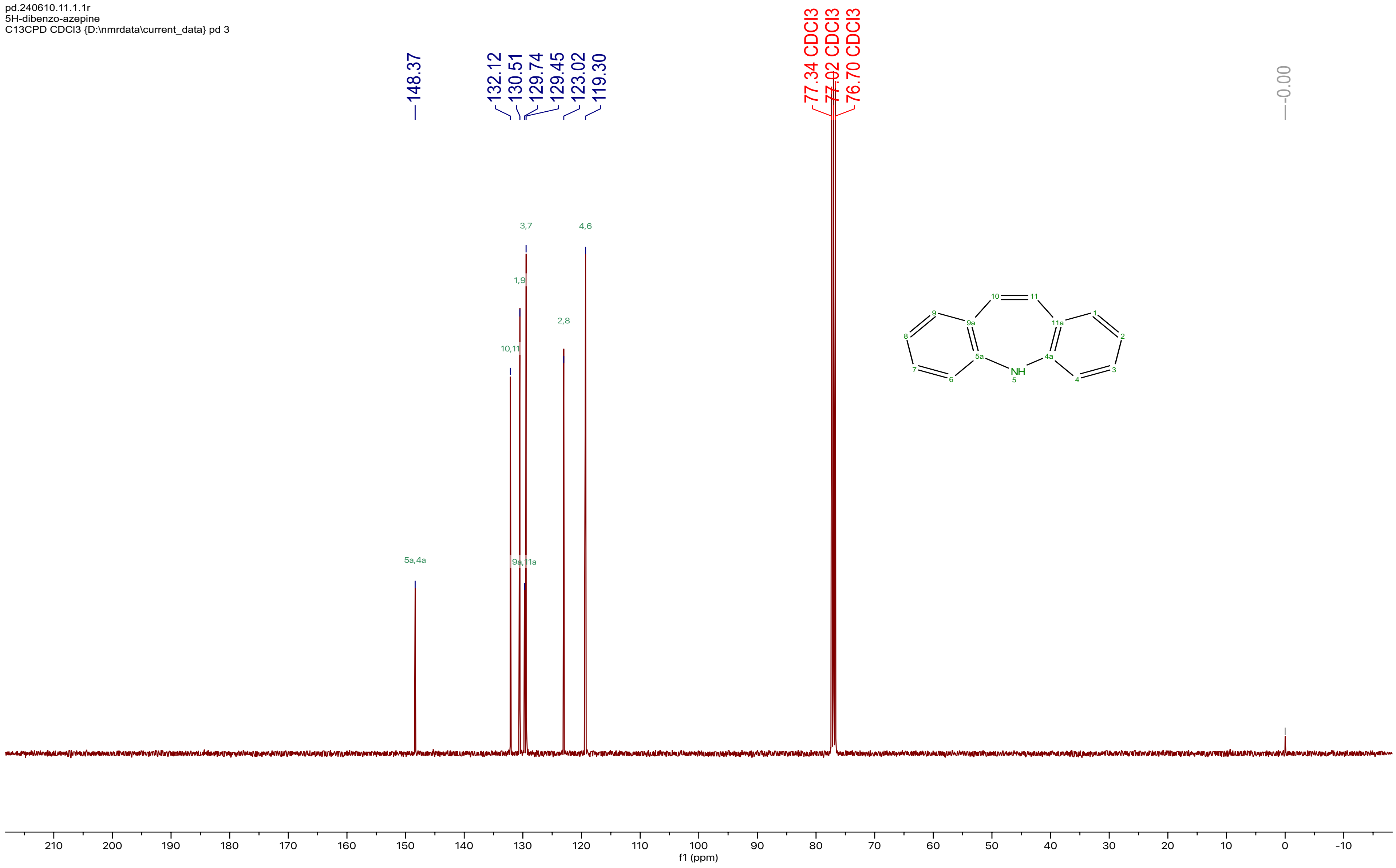

$^{13}C\{^{1}H\}$ NMR (101 MHz, $CDCl_3$) of 3p'

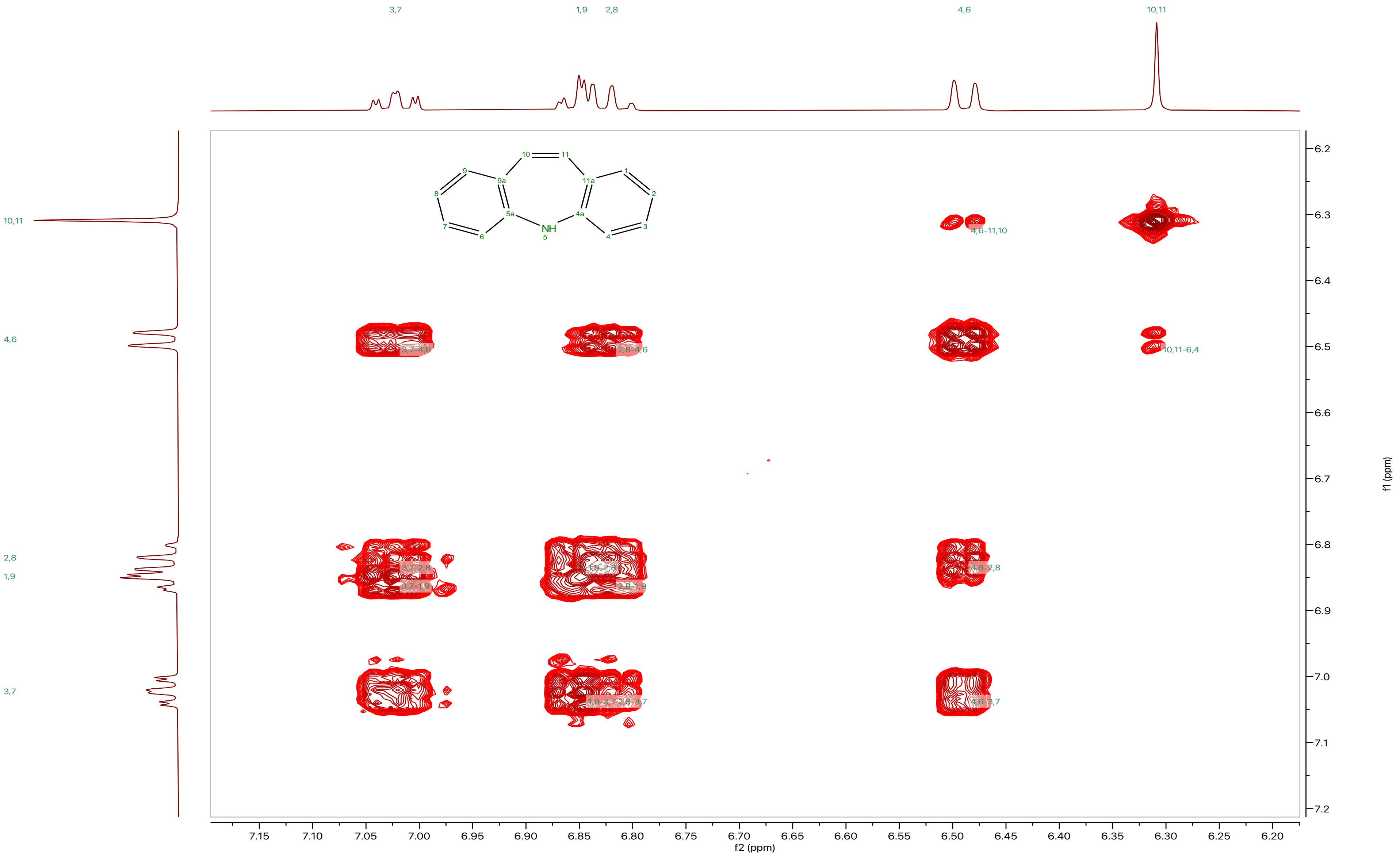

^{1}H-^{1}H COSY (400 MHz, $CDCl_3$) of 3p'

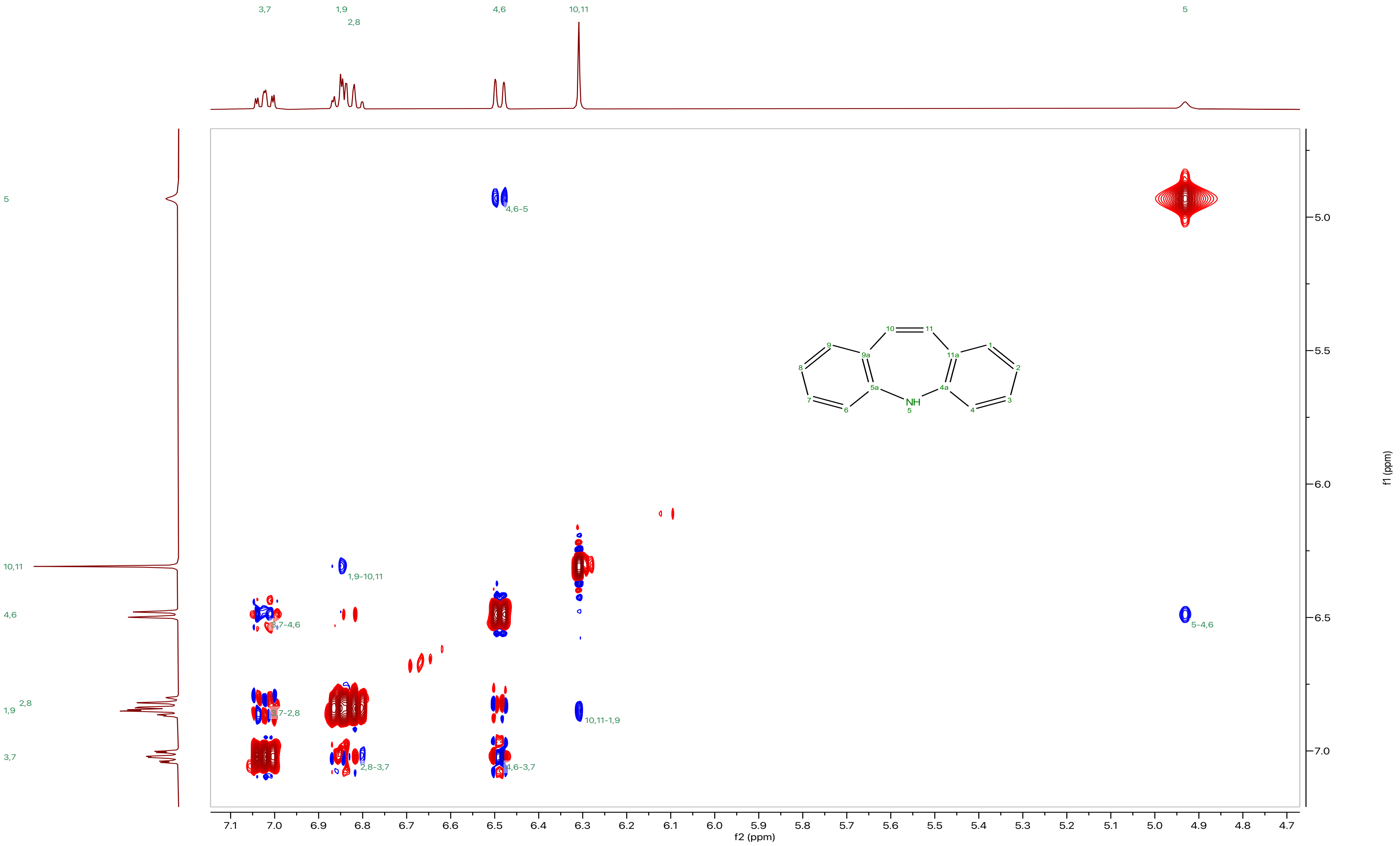

^1H-^1H NOESY (400 MHz, $CDCl_3$) of 3p'

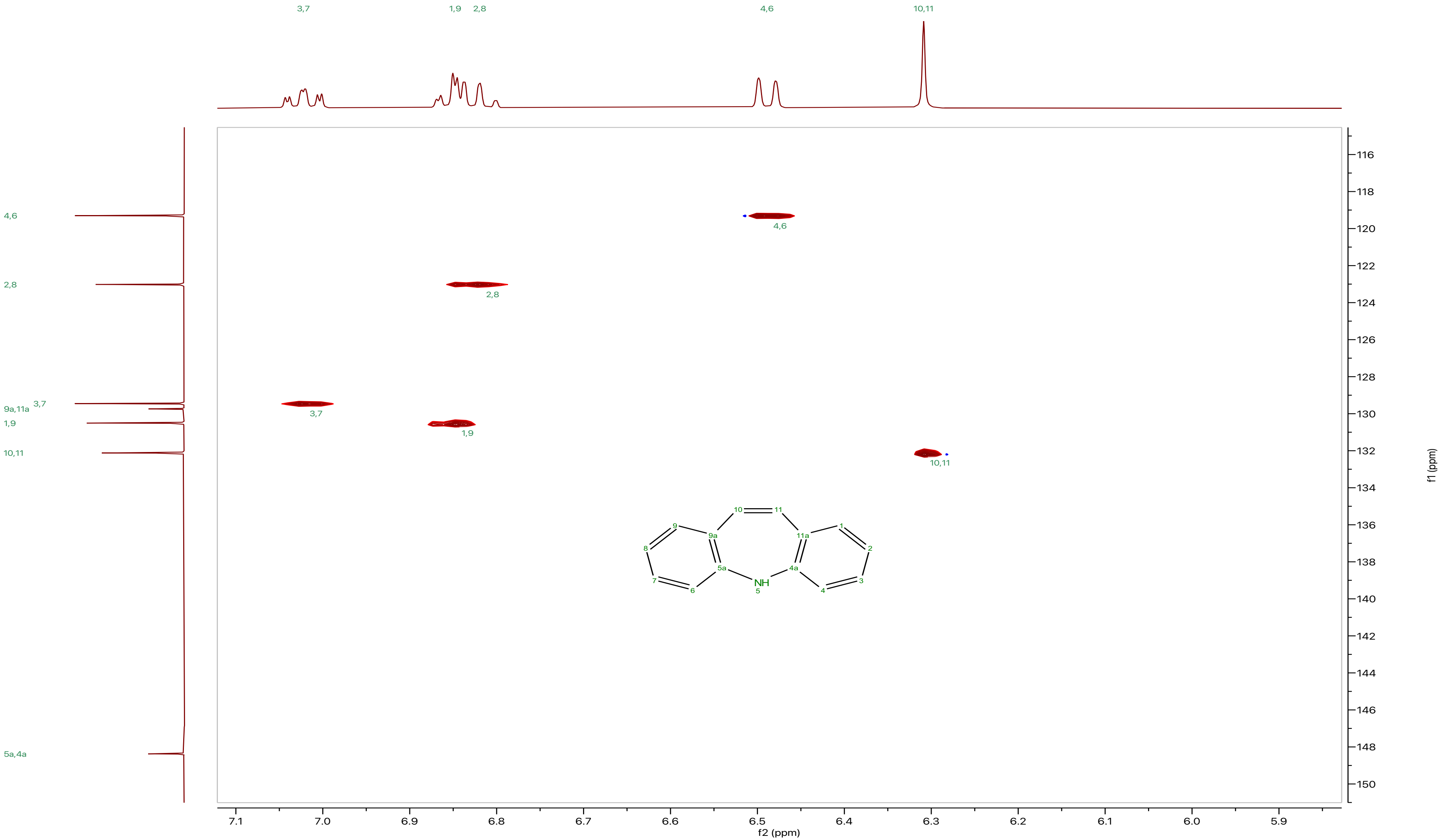

^{1}H-$^{13}C\{^{1}H\}$ HSQC NMR (400/101 MHz, $CDCl_3$) of 3p'

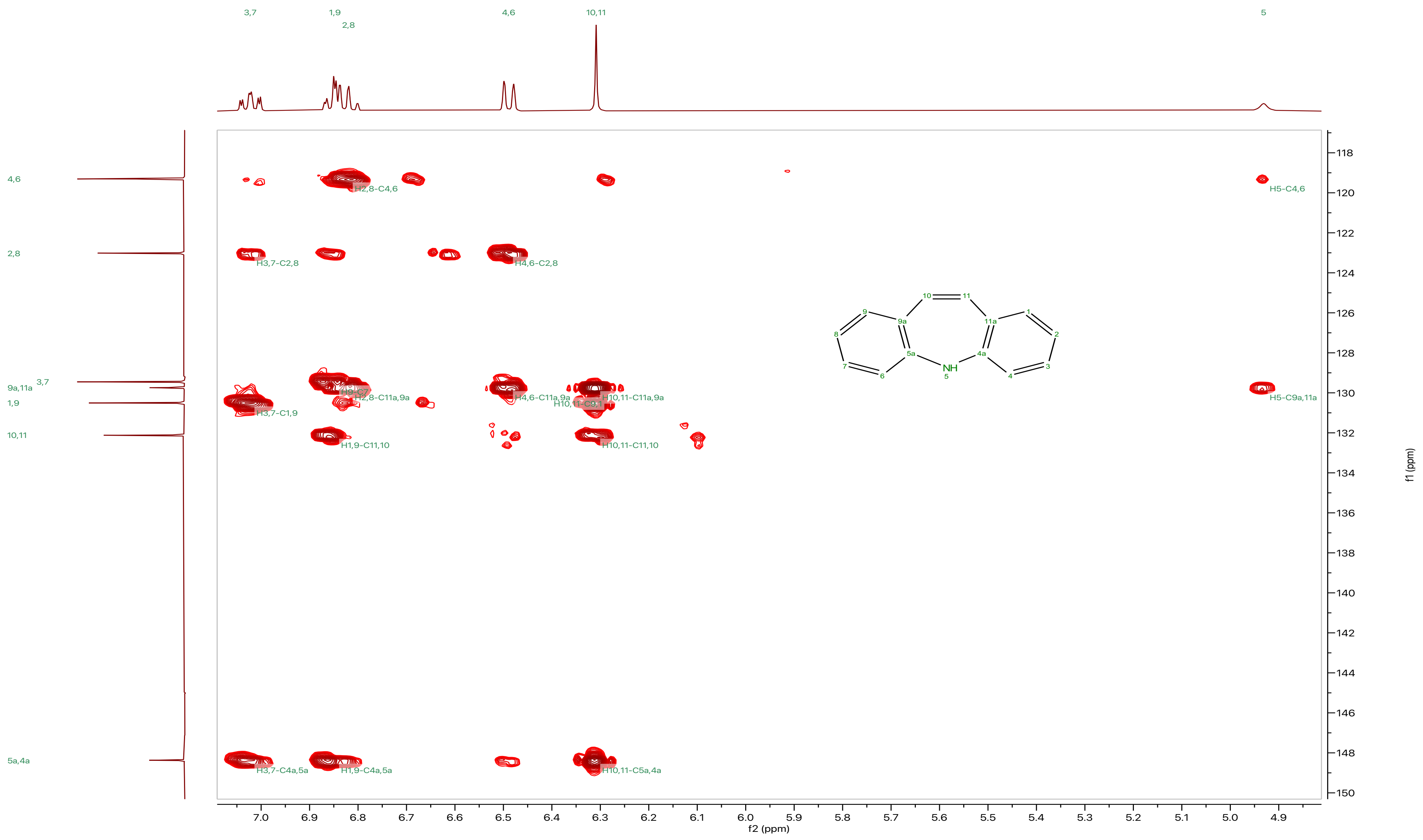

^{1}H-$^{13}C\{^{1}H\}$ HMBC NMR (400/101 MHz, $CDCl_3$) of 3p’

4bp

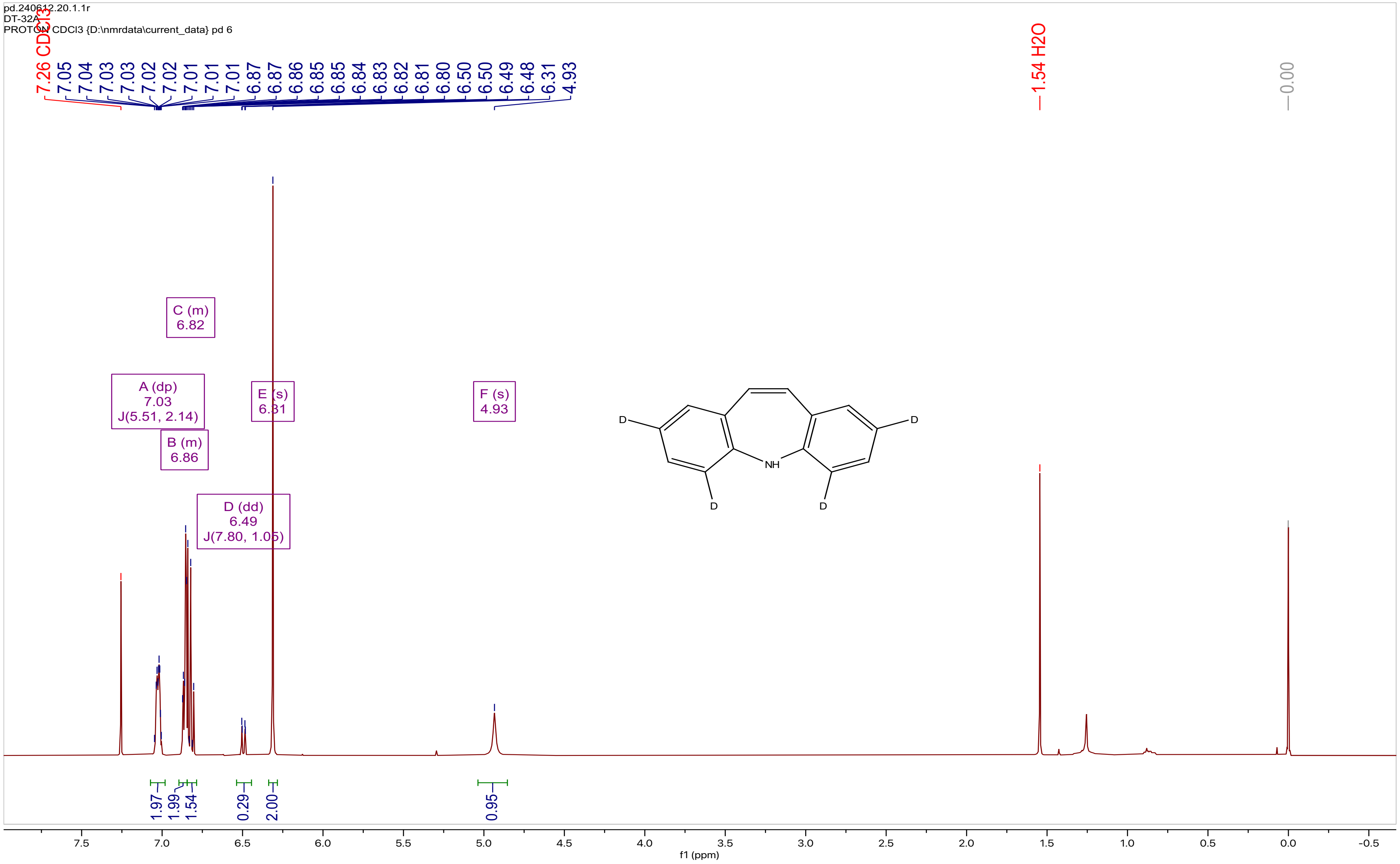

^1H NMR (400 MHz, $CDCl_3$) of 4bp

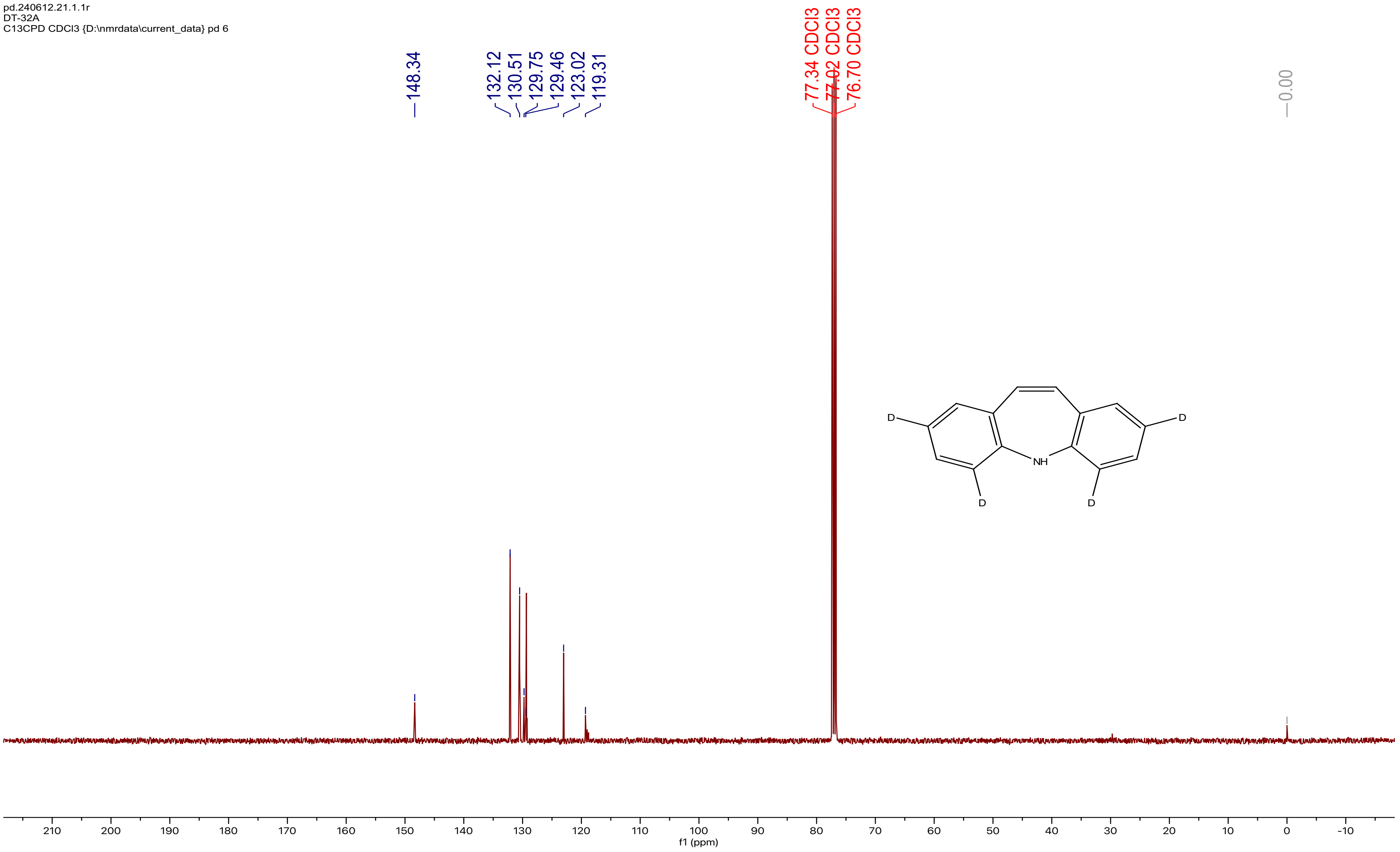

$^{13}C\{^{1}H\}$ NMR (101 MHz, $CDCl_3$) of 4bp

3q'

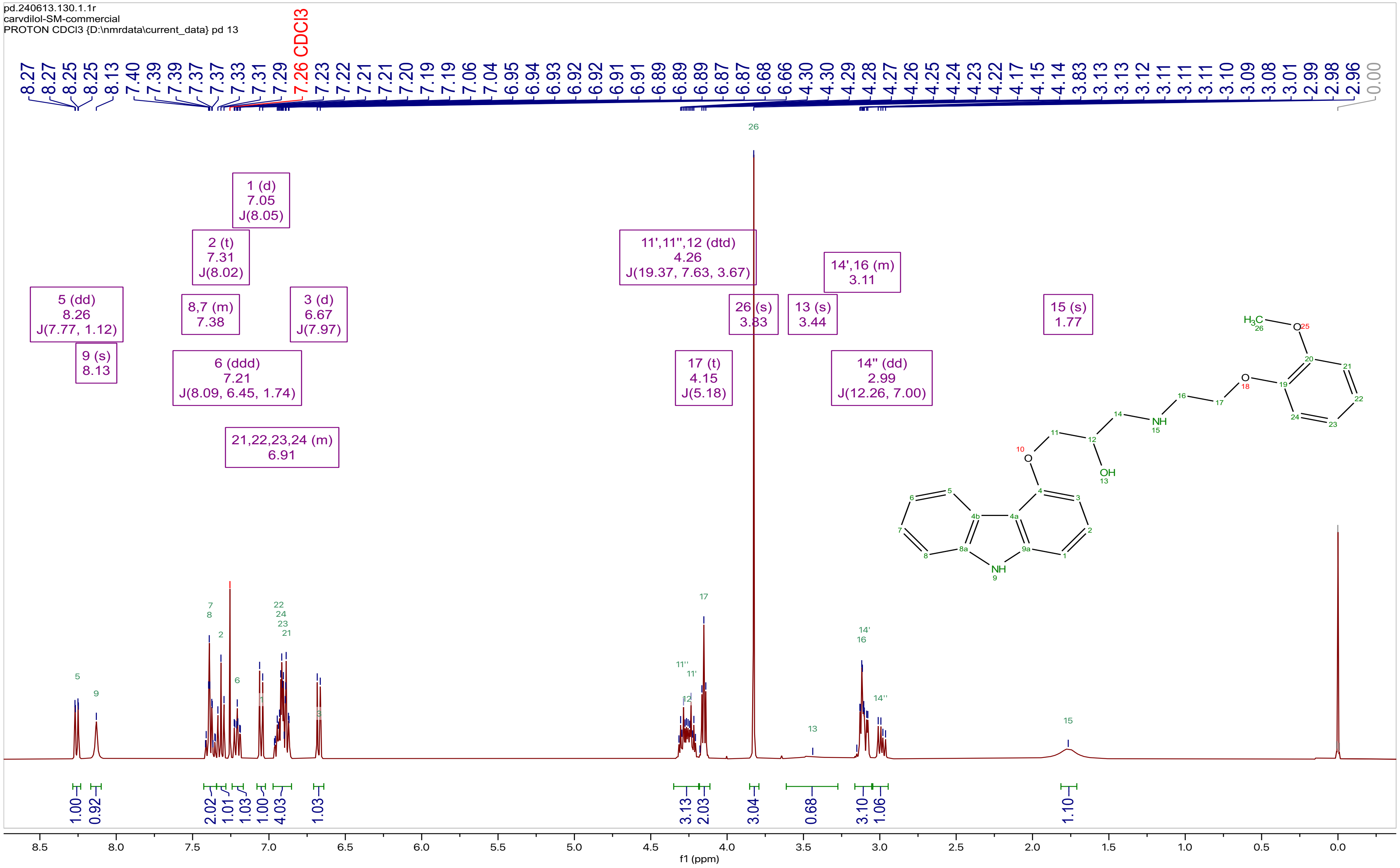

^{1}H NMR (400 MHz, $CDCl_3$) of 3q'

pd.240613.131.1.1r
carvdilol-SM-commercial
C13CPD CDCl3 {D:\nmrdata\current_data} pd 13

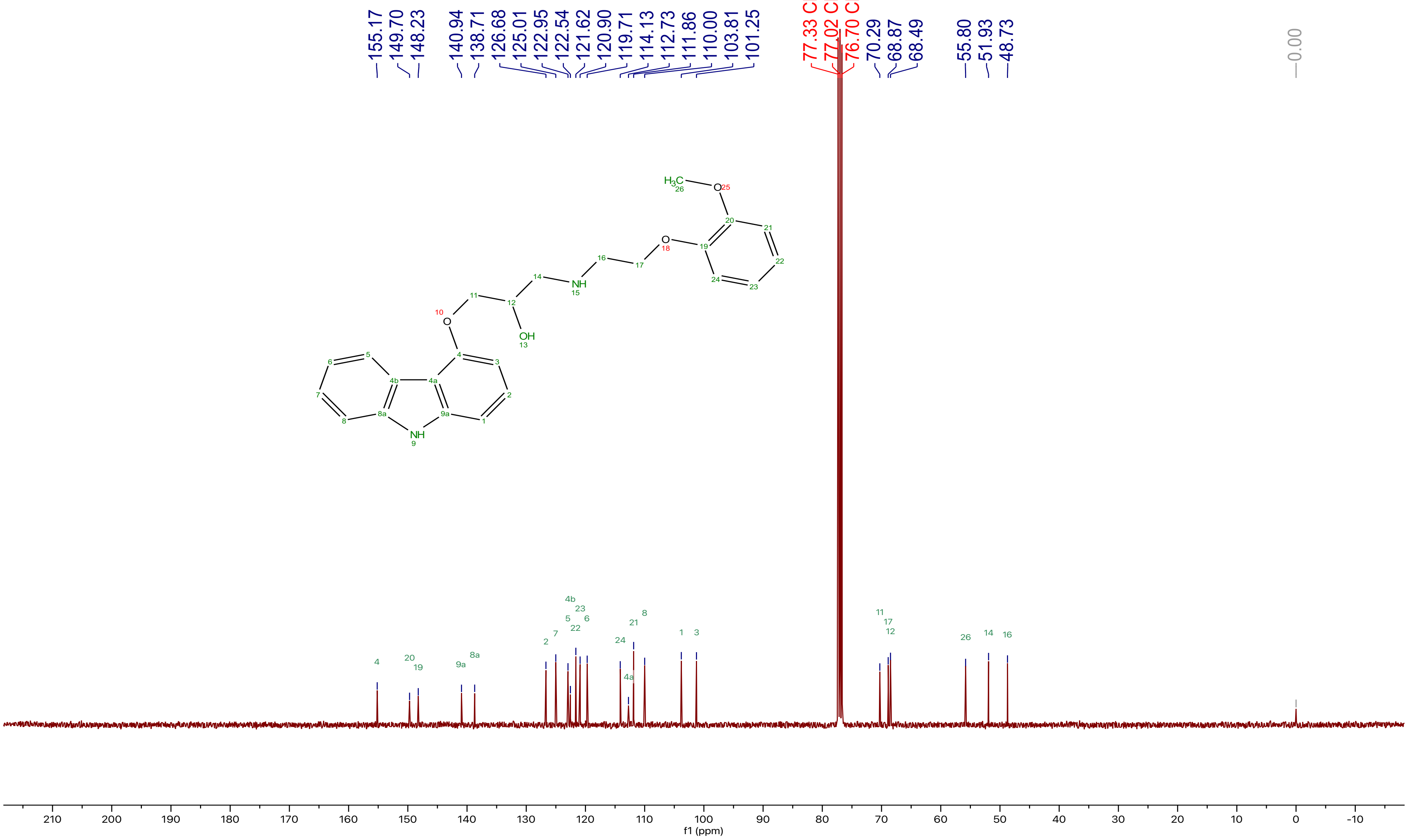

$^{13}C\{^{1}H\}$ NMR (101 MHz, $CDCl_3$) of 3q'

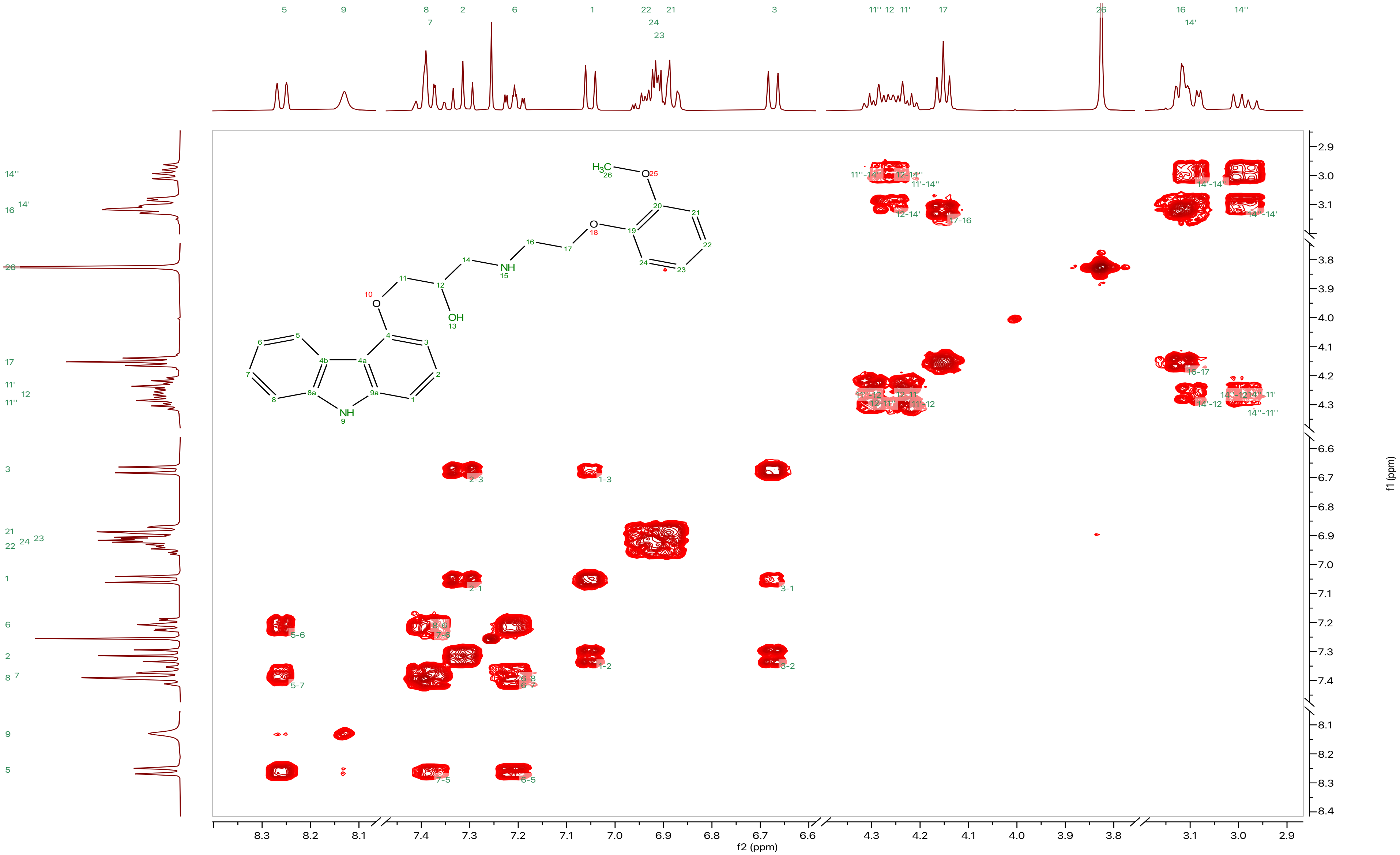

¹H-¹H COSY (400 MHz, CDCl₃) of 3q'

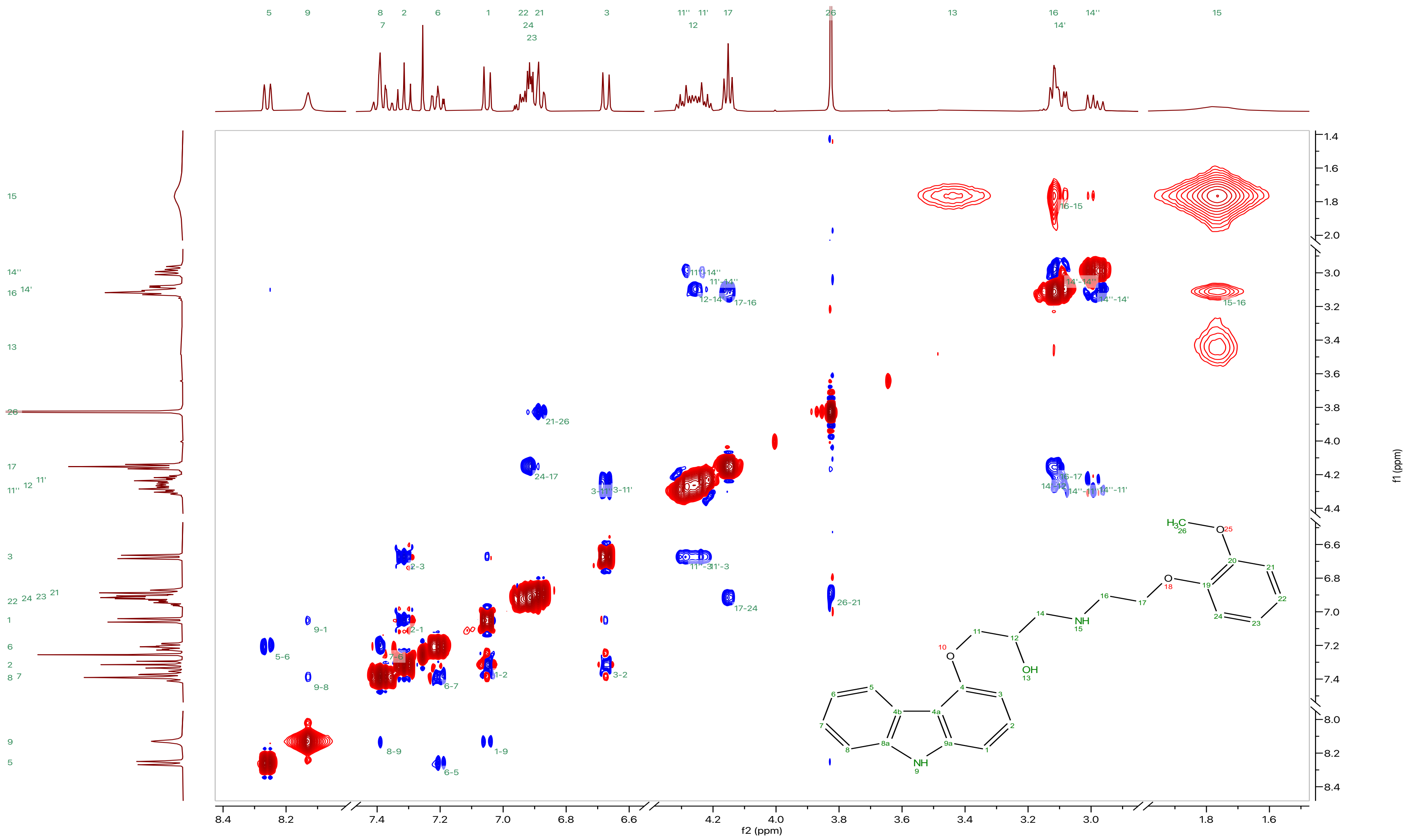

^{1}H-^{1}H NOESY (400 MHz, $CDCl_3$) of 3q'

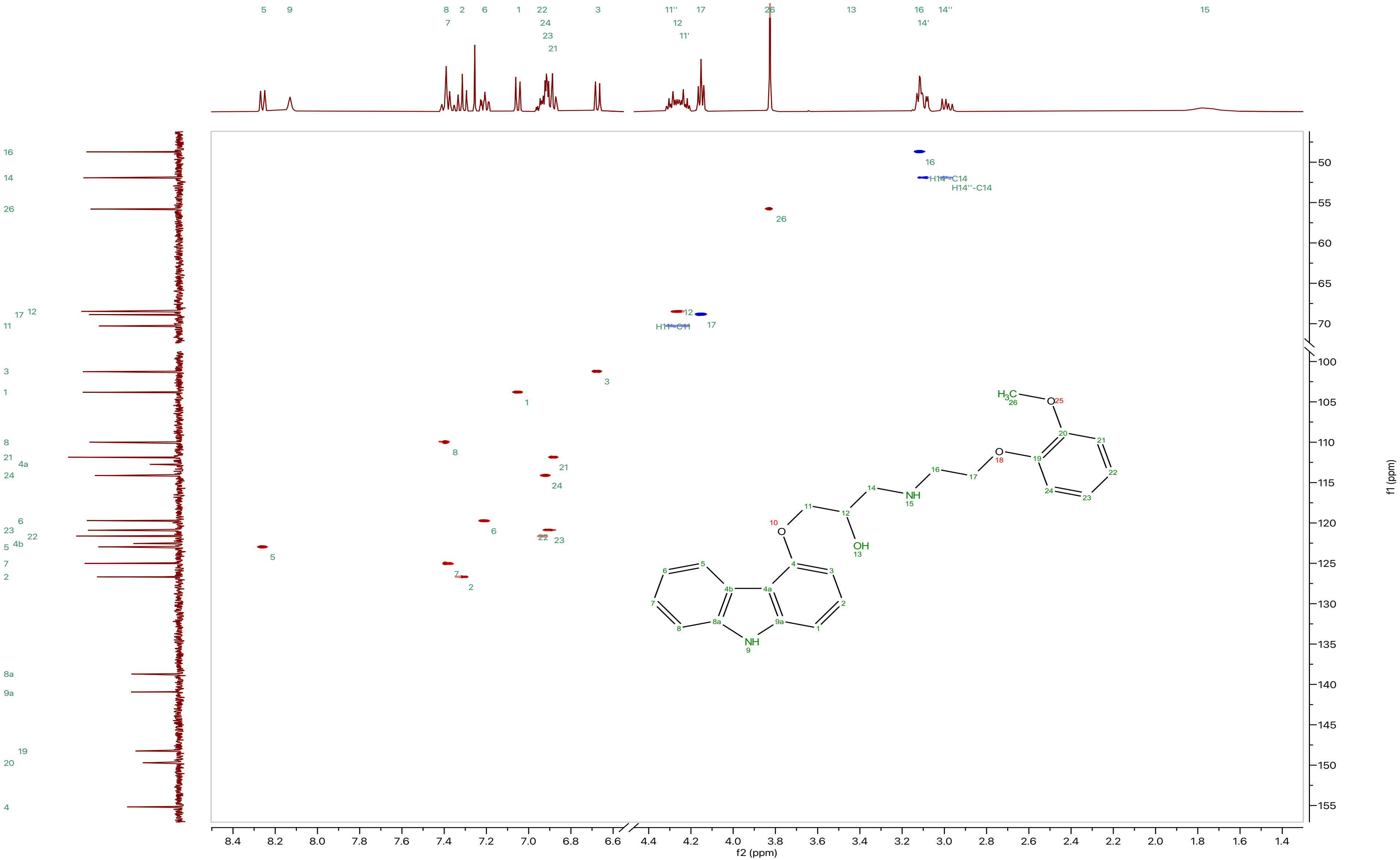

^{1}H-$^{13}C\{^{1}H\}$ HSQC NMR (400/101 MHz, $CDCl_3$) of 3q’

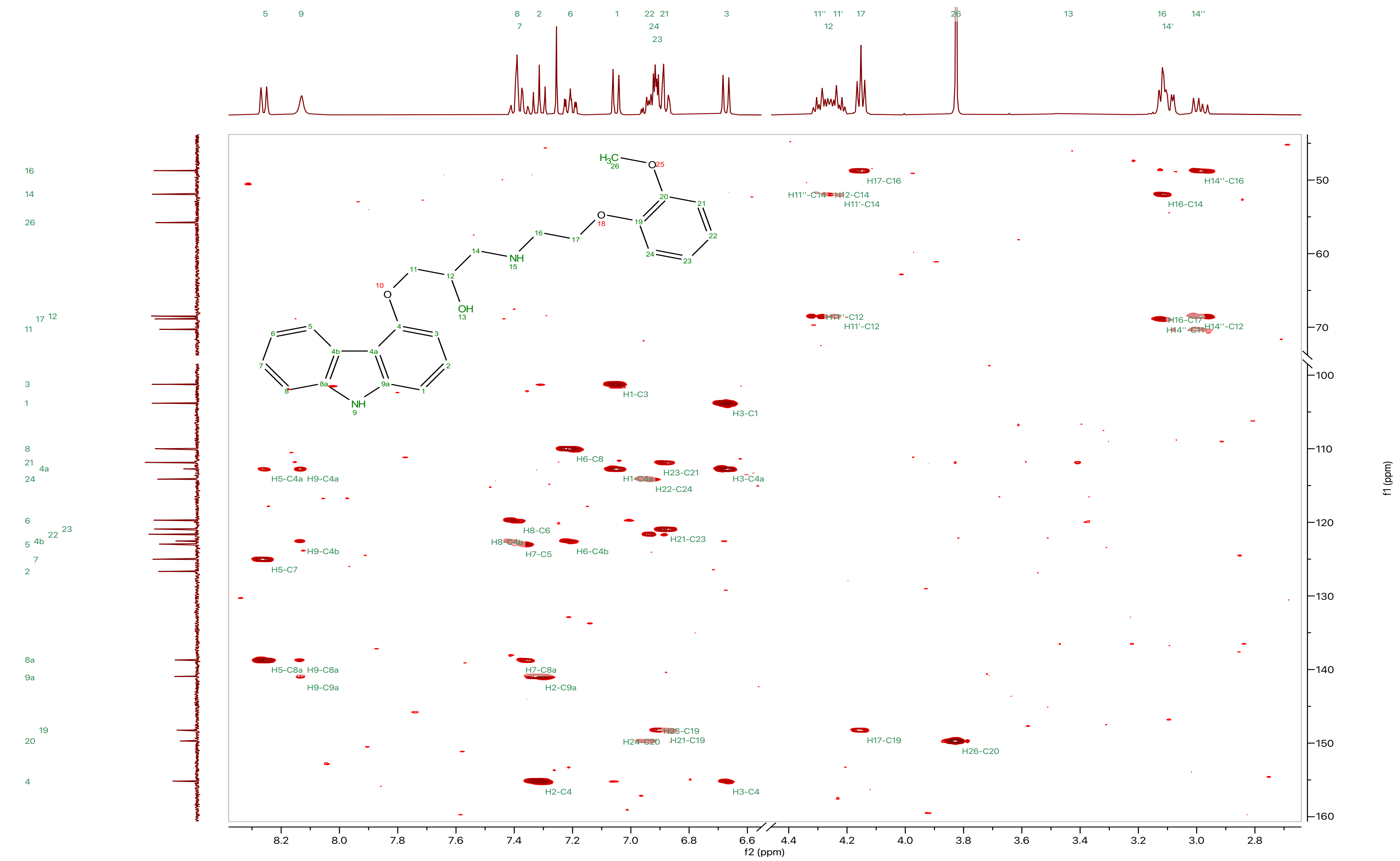

¹H-¹³C{¹H} HMBC NMR (400/101 MHz, CDCl₃) of 3q'

4bq'

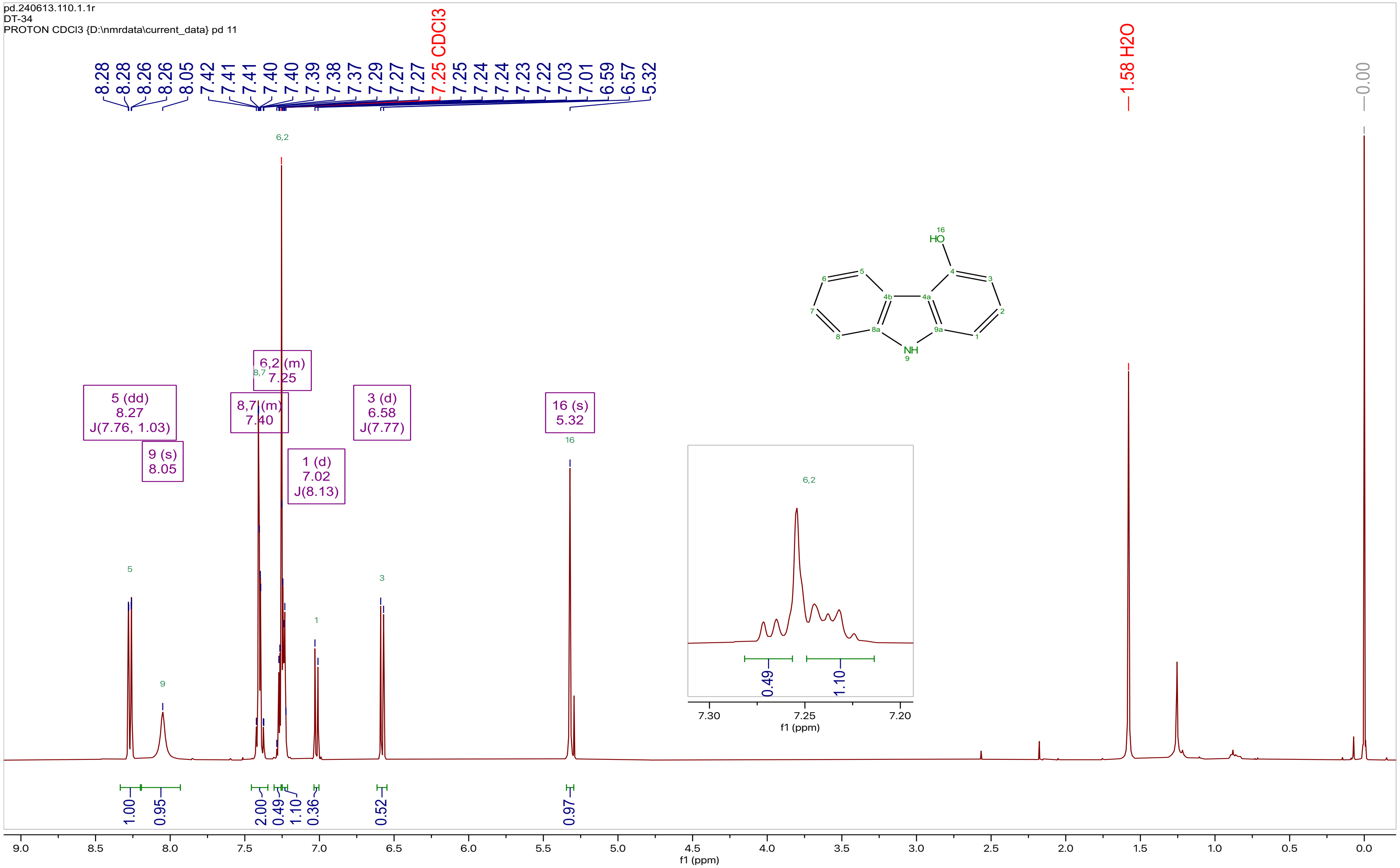

^{1}H NMR (400 MHz, $CDCl_3$) of 4bq'

pd.240613.111.1.1r
DT-34
C13CPD CDCl3 {D:\nmrdata\current_data} pd 11

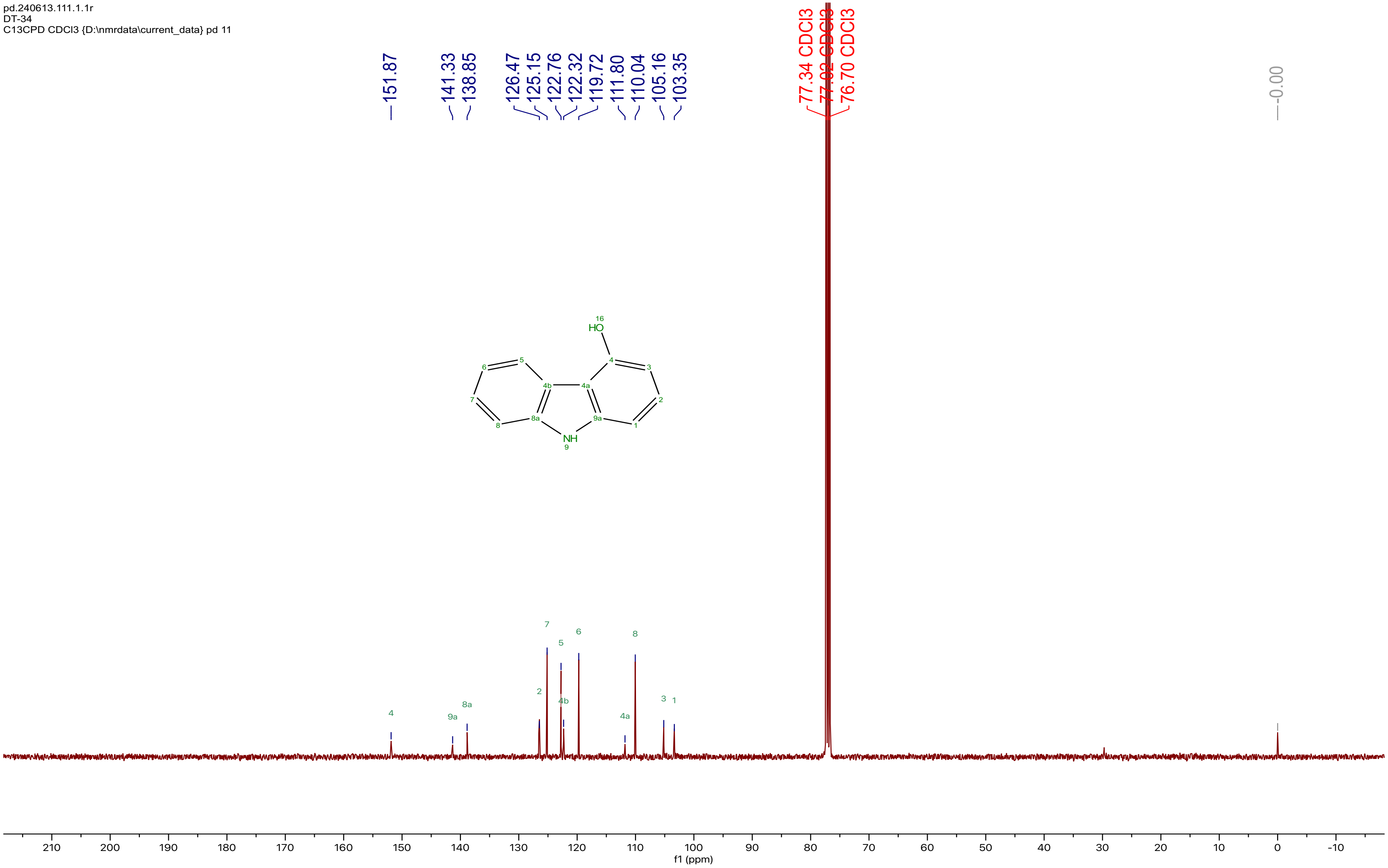

$^{13}C\{^{1}H\}$ NMR (101 MHz, $CDCl_3$) of 4bq'

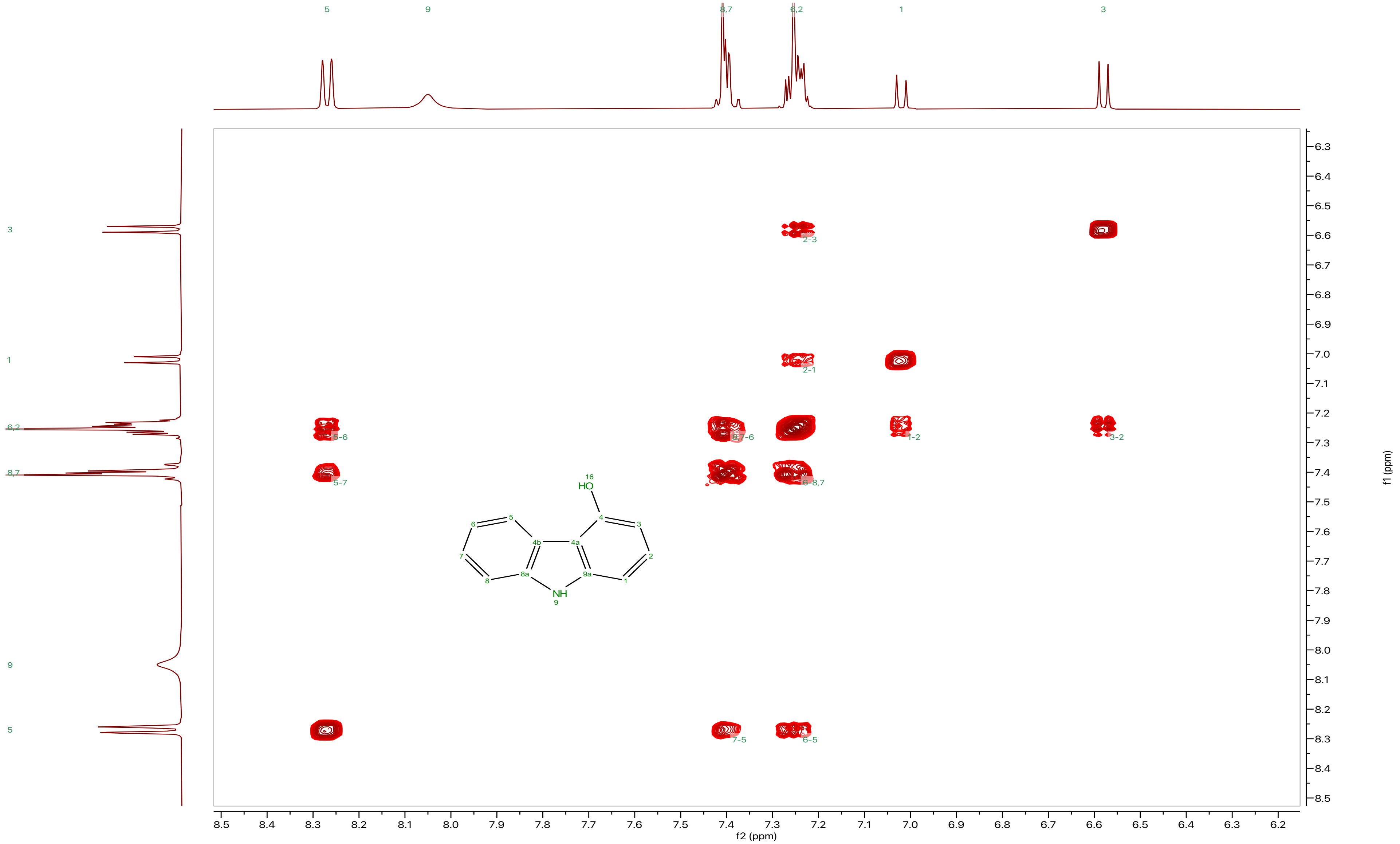

^{1}H-^{1}H COSY (400 MHz, $CDCl_3$) of 4bq’

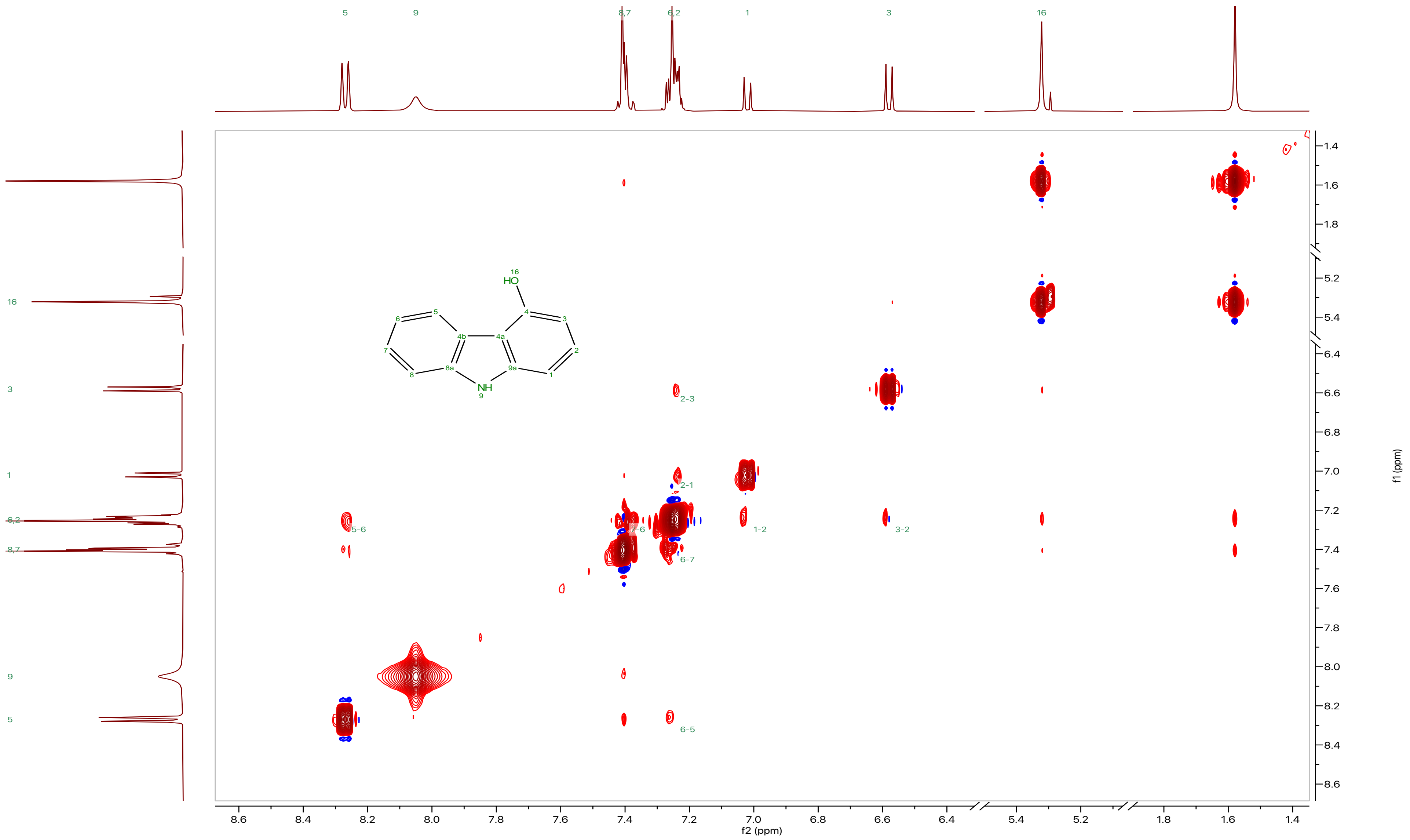

^{1}H-^{1}H NOESY (400 MHz, $CDCl_3$) of 4bq'

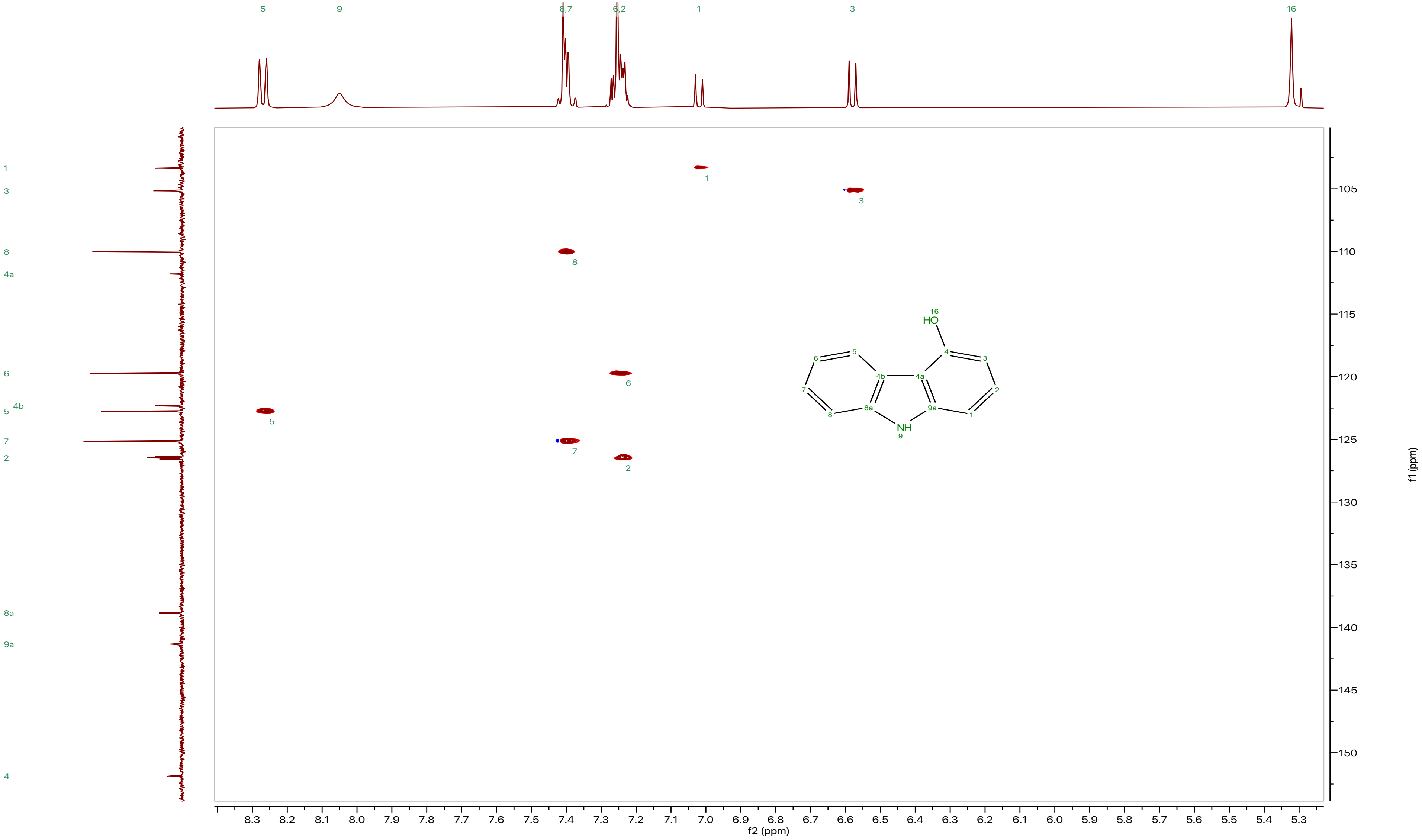

^{1}H-$^{13}C\{^{1}H\}$ HSQC NMR (400/101 MHz, $CDCl_3$) of 4bq'

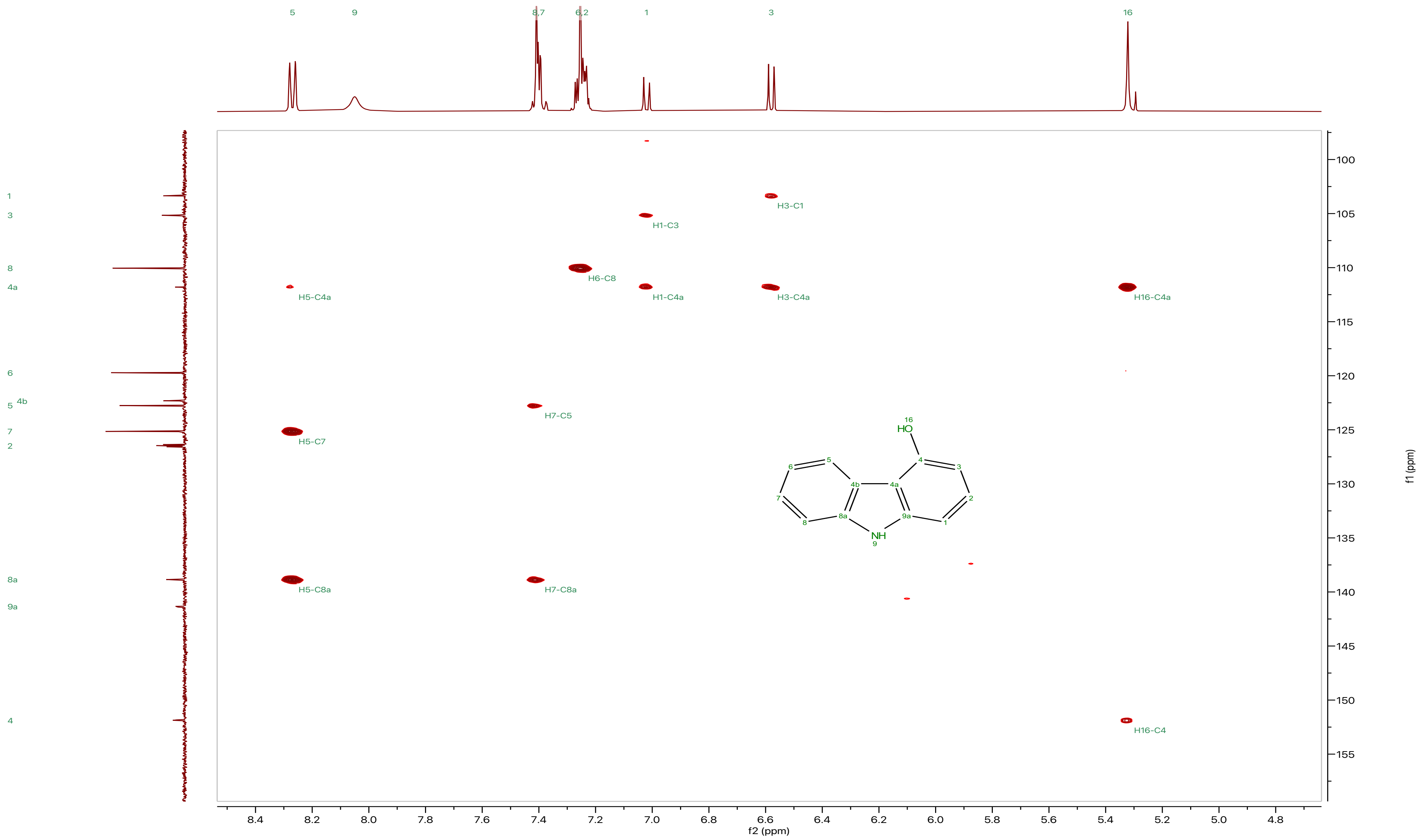

^{1}H-$^{13}C\{^{1}H\}$ HMBC NMR (400/101 MHz, $CDCl_3$) of 4bq'

3r

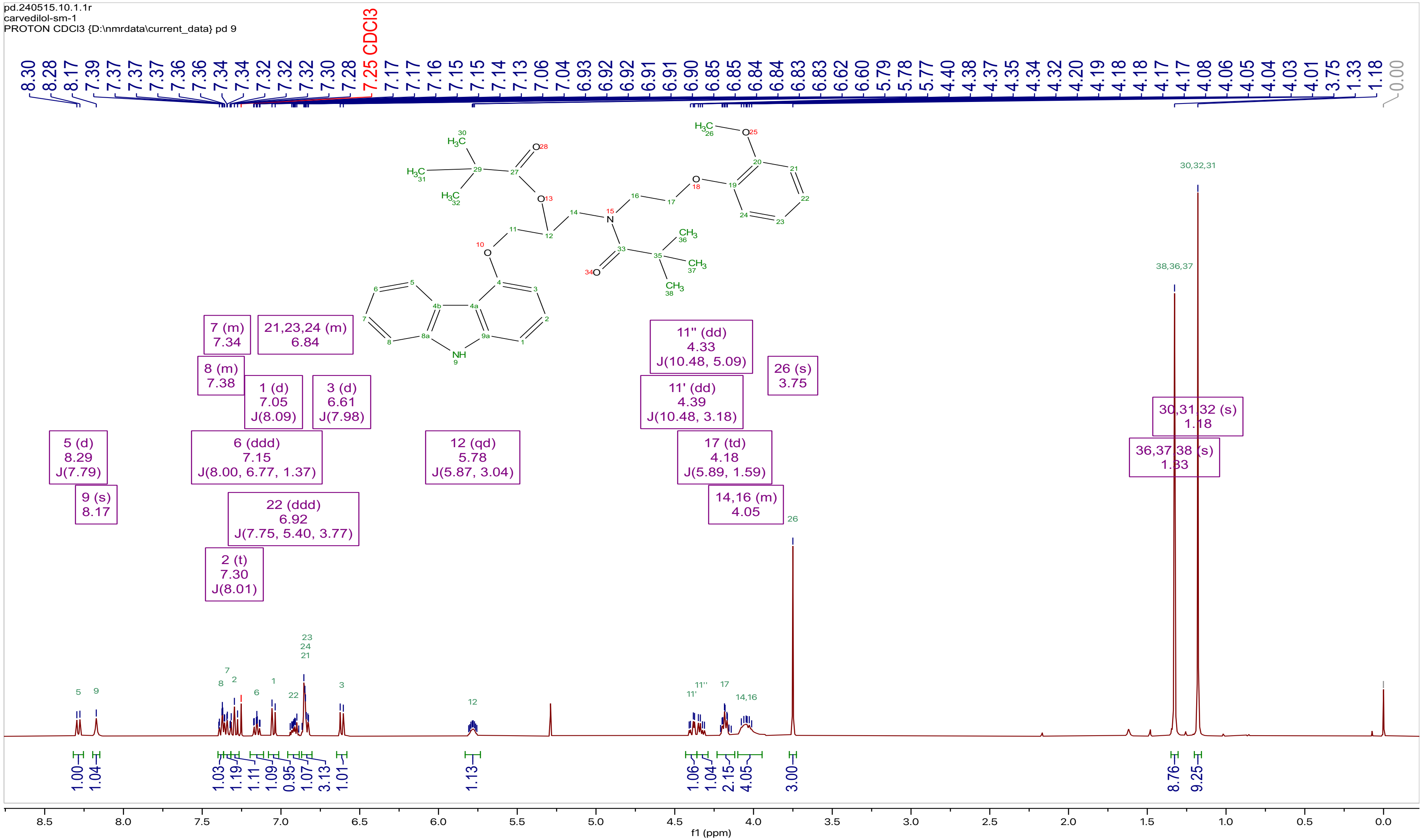

^1H NMR (400 MHz, $CDCl_3$) of 3r

pd.240515.11.1.1r
carvedilol-sm-1
C13CPD CDCl3 {D:\nmrdata\current_data} pd 9

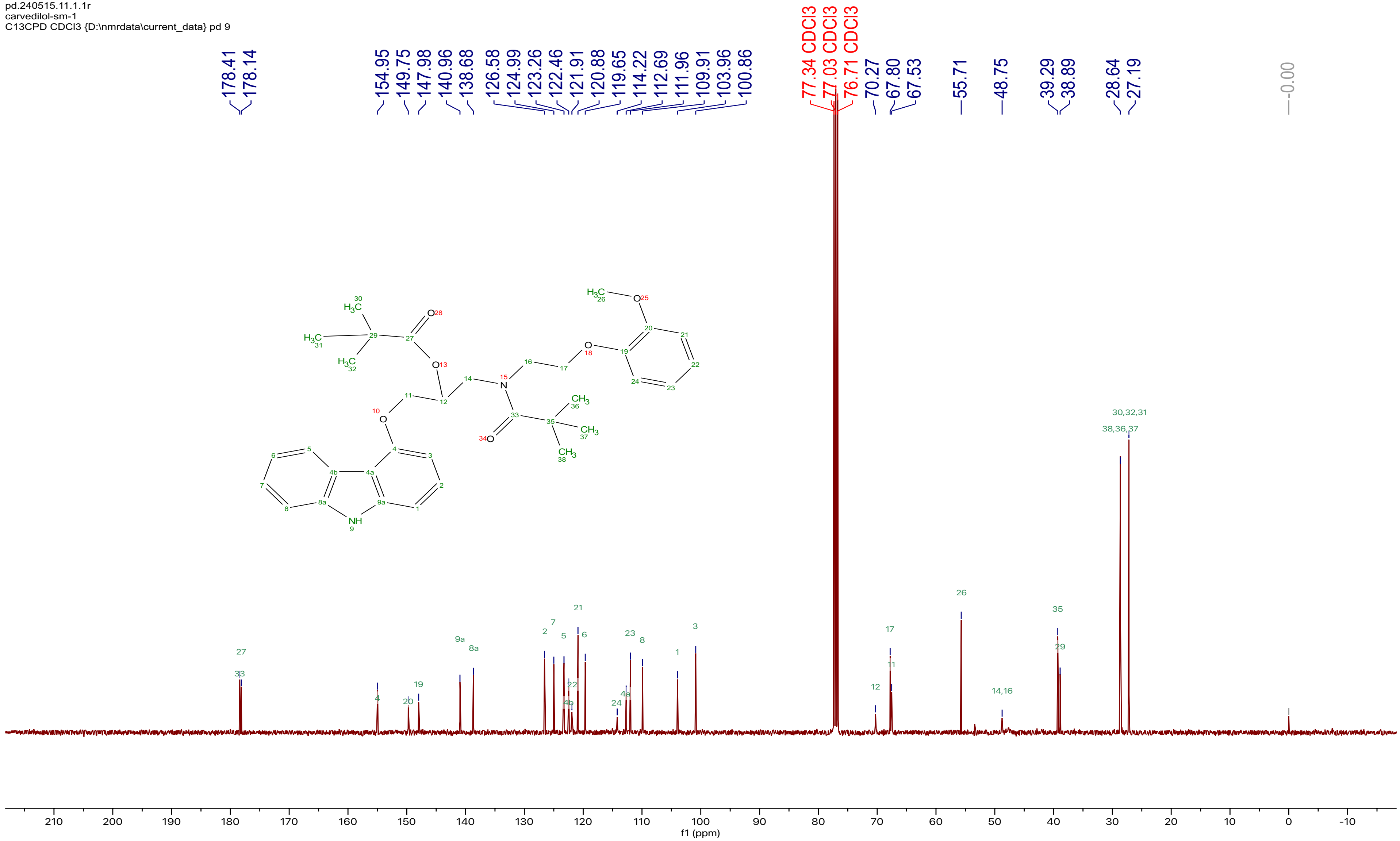

$^{13}C\{^1H\}$ NMR (101 MHz, $CDCl_3$) of 3r

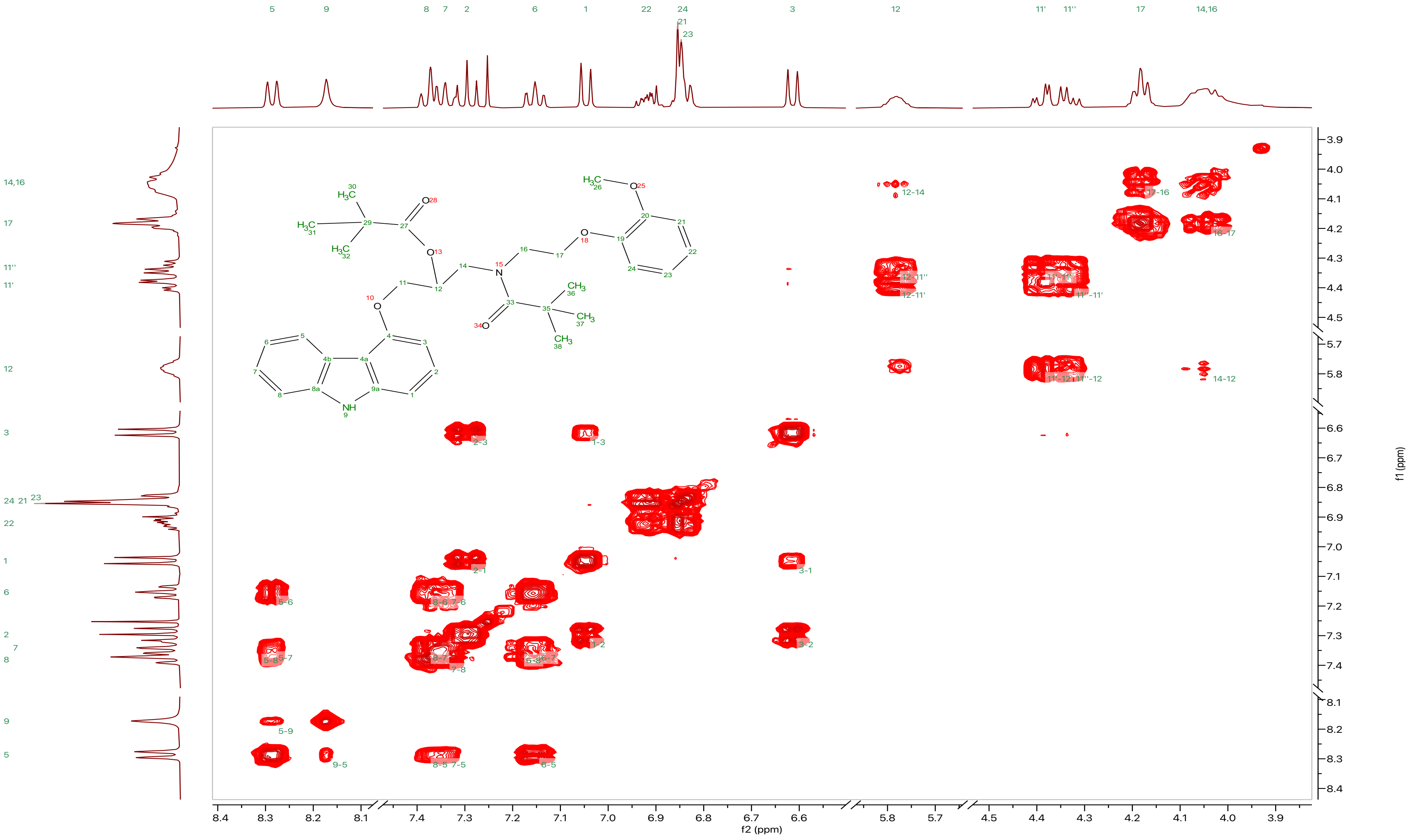

^{1}H-^{1}H COSY (400 MHz, $CDCl_3$) of 3r

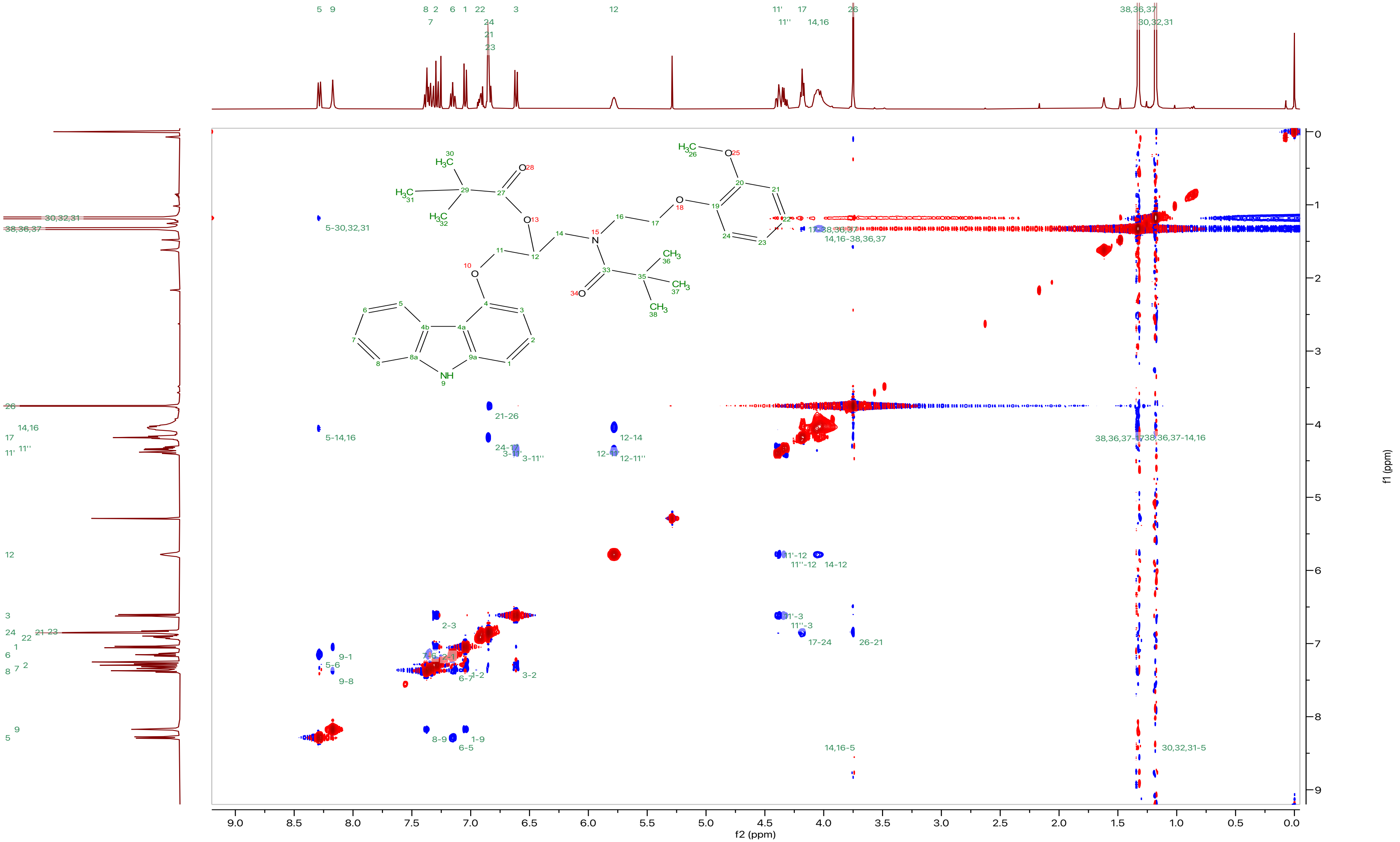

^{1}H-^{1}H NOESY (400 MHz, $CDCl_3$) of 3r

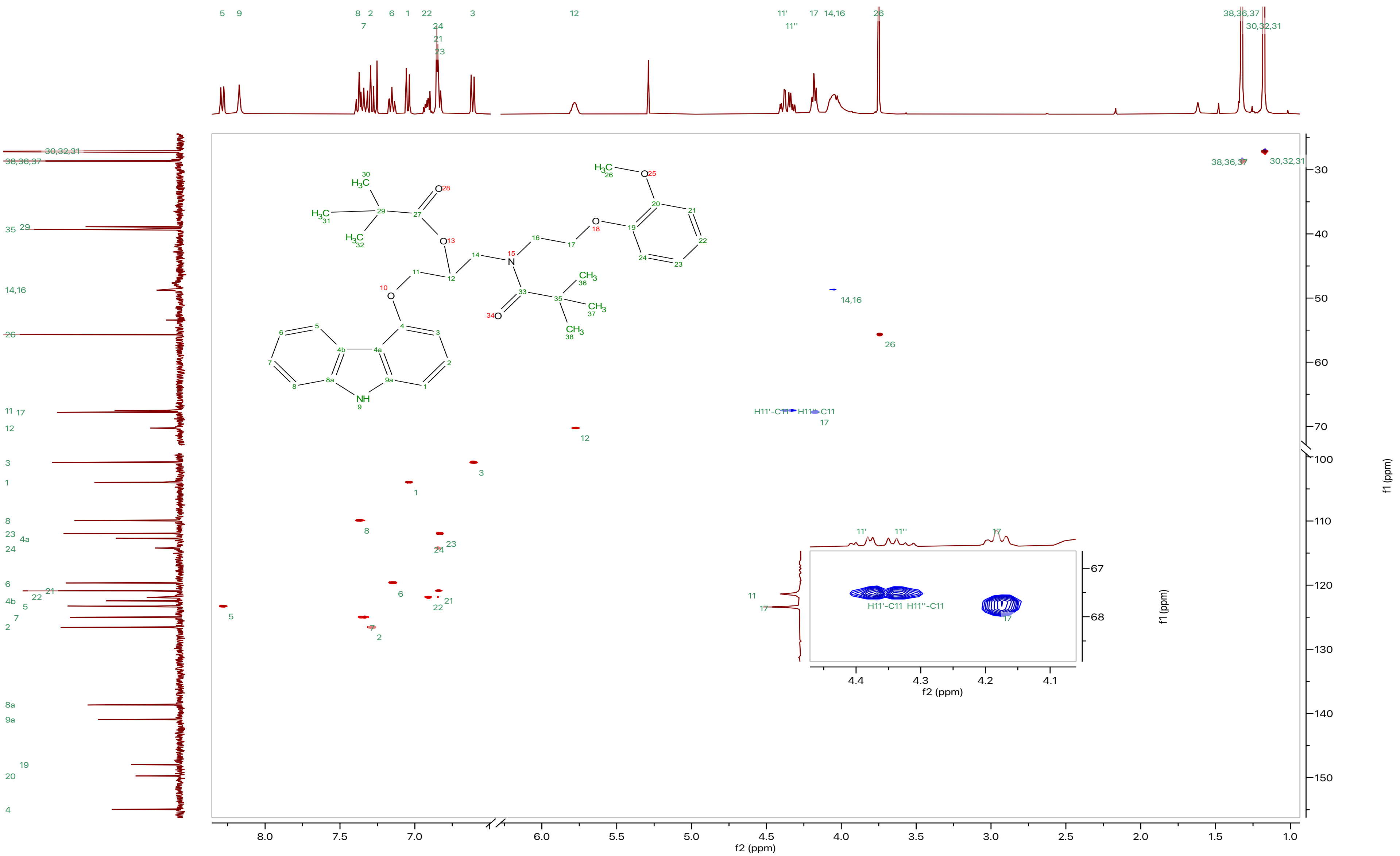

^{1}H-$^{13}C\{^{1}H\}$ HSQC NMR (400/101 MHz, $CDCl_3$) of 3r

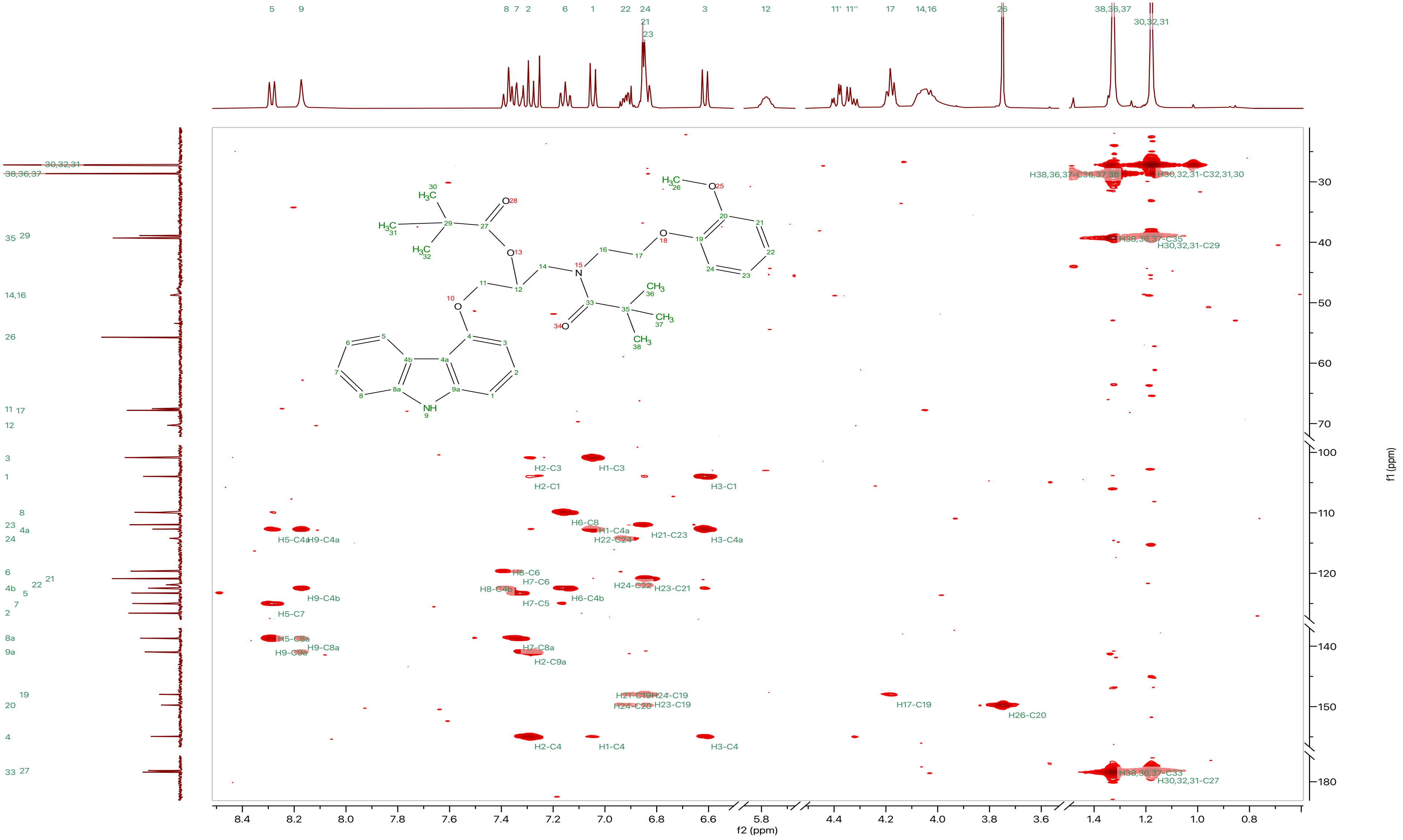

^{1}H-$^{13}C\{^{1}H\}$ HMBC NMR (400/101 MHz, $CDCl_3$) of 3r

4ar

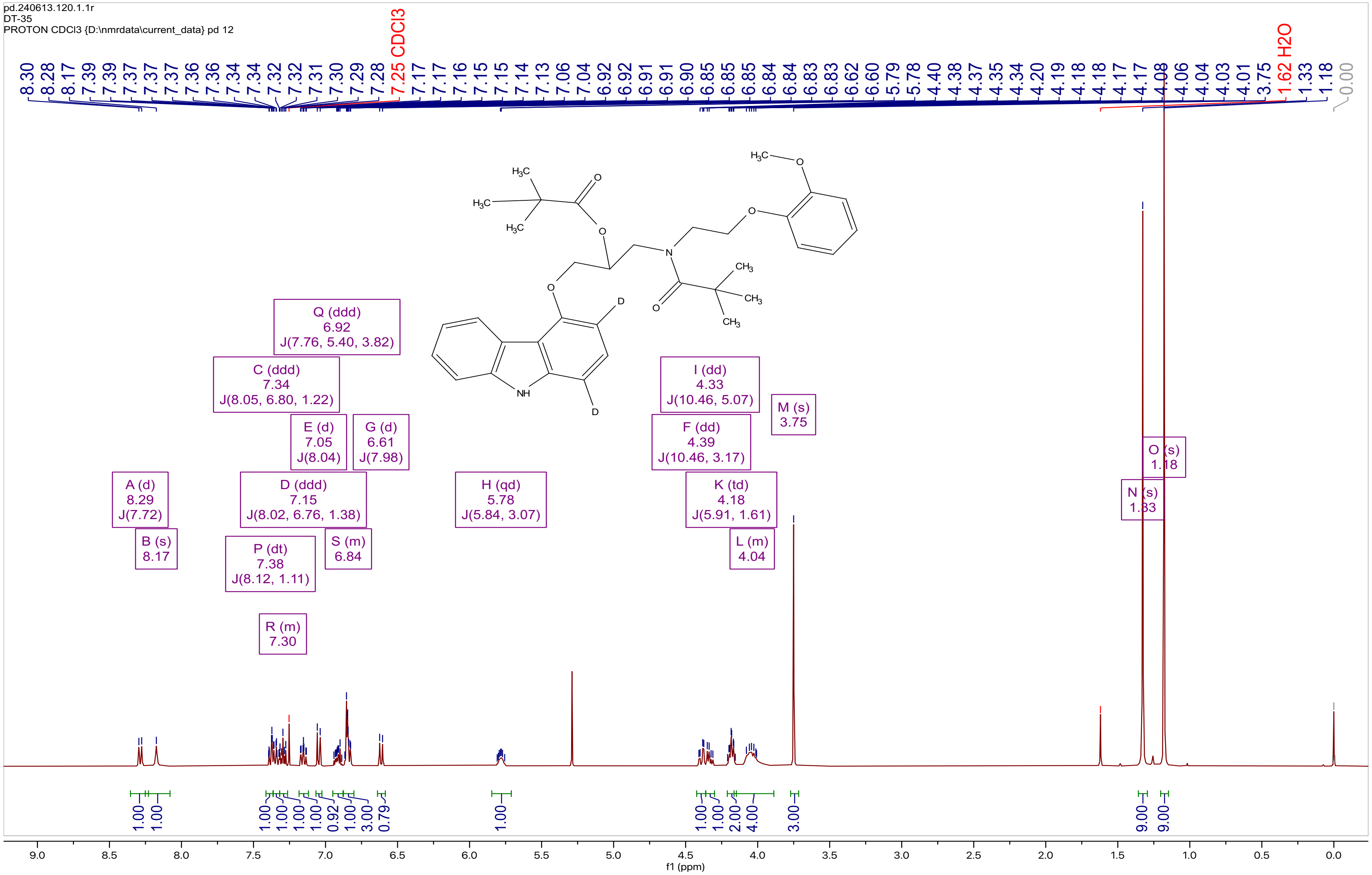

^{1}H NMR (400 MHz, $CDCl_3$) of 4ar

pd.240613.121.1.1r
DT-35
C13CPD CDCl3 {D:\nmrdata\current_data} pd 12

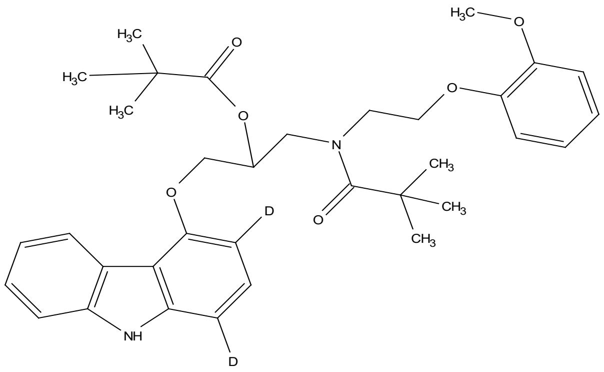

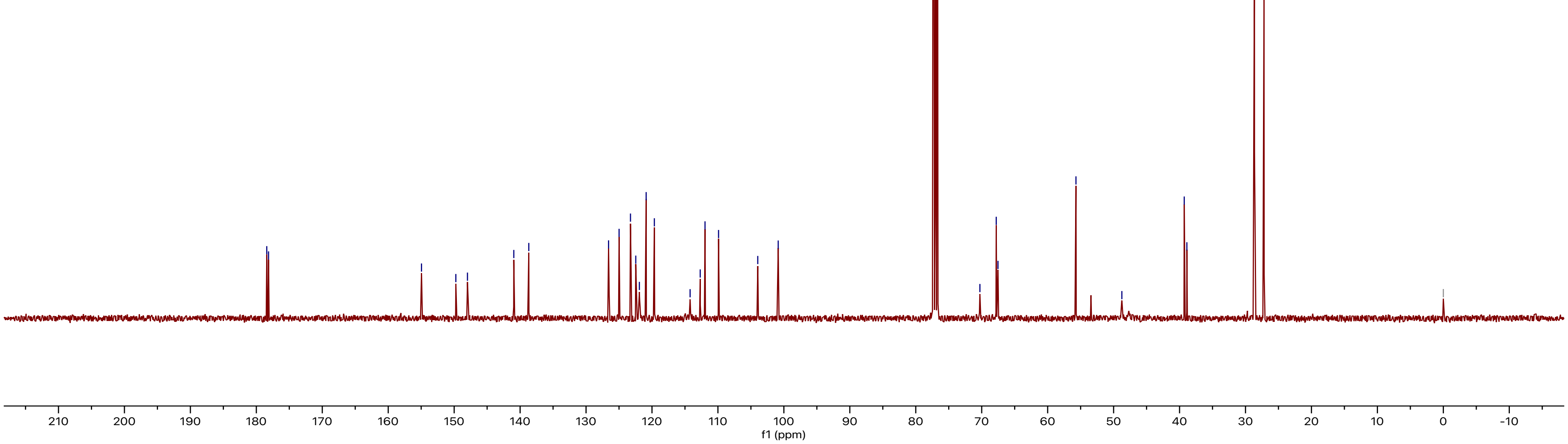

$^{13}C\{^{1}H\}$ NMR (101 MHz, $CDCl_3$) of 4ar

3s

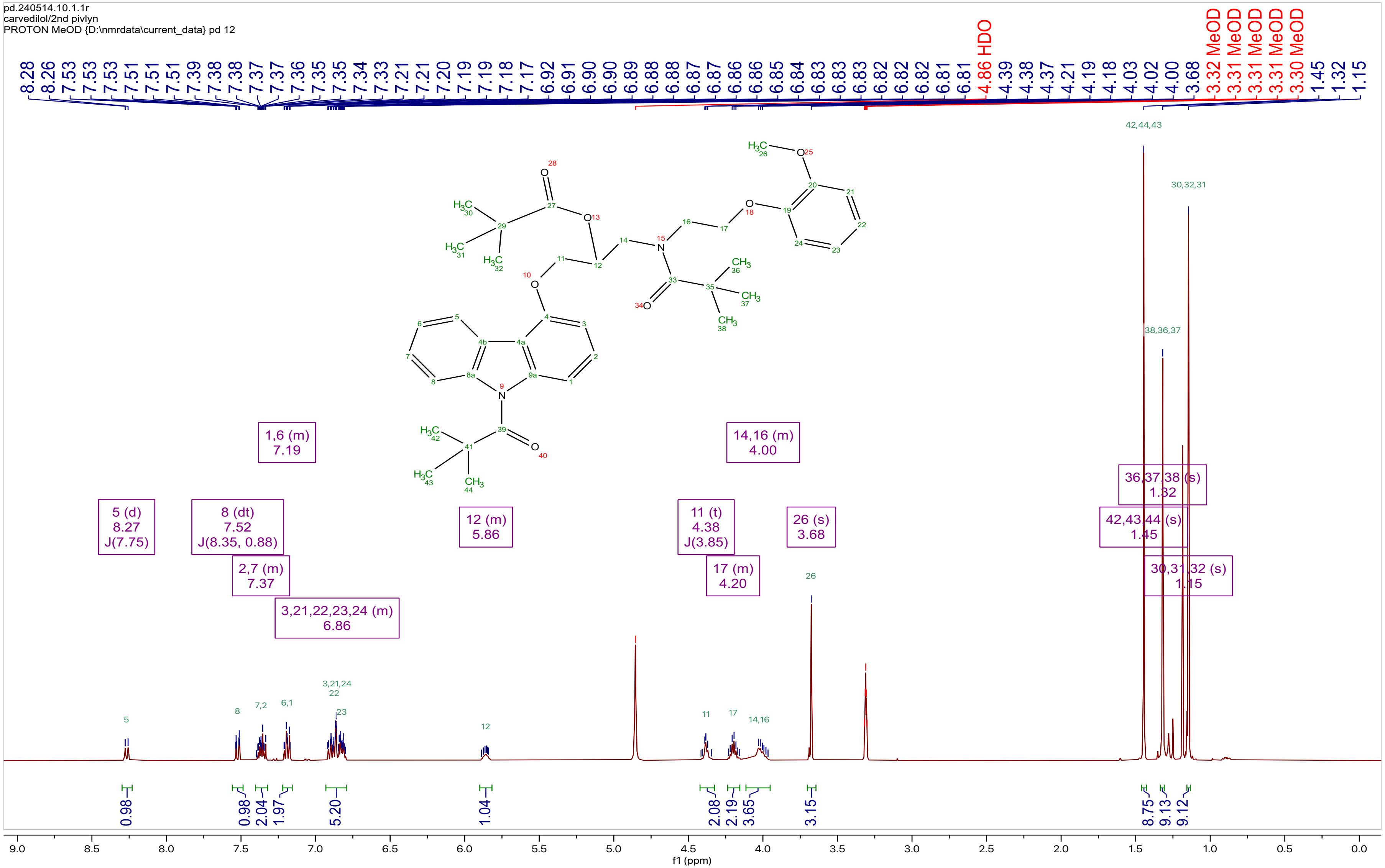

^{1}H NMR (400 MHz, MeOD) of 3s

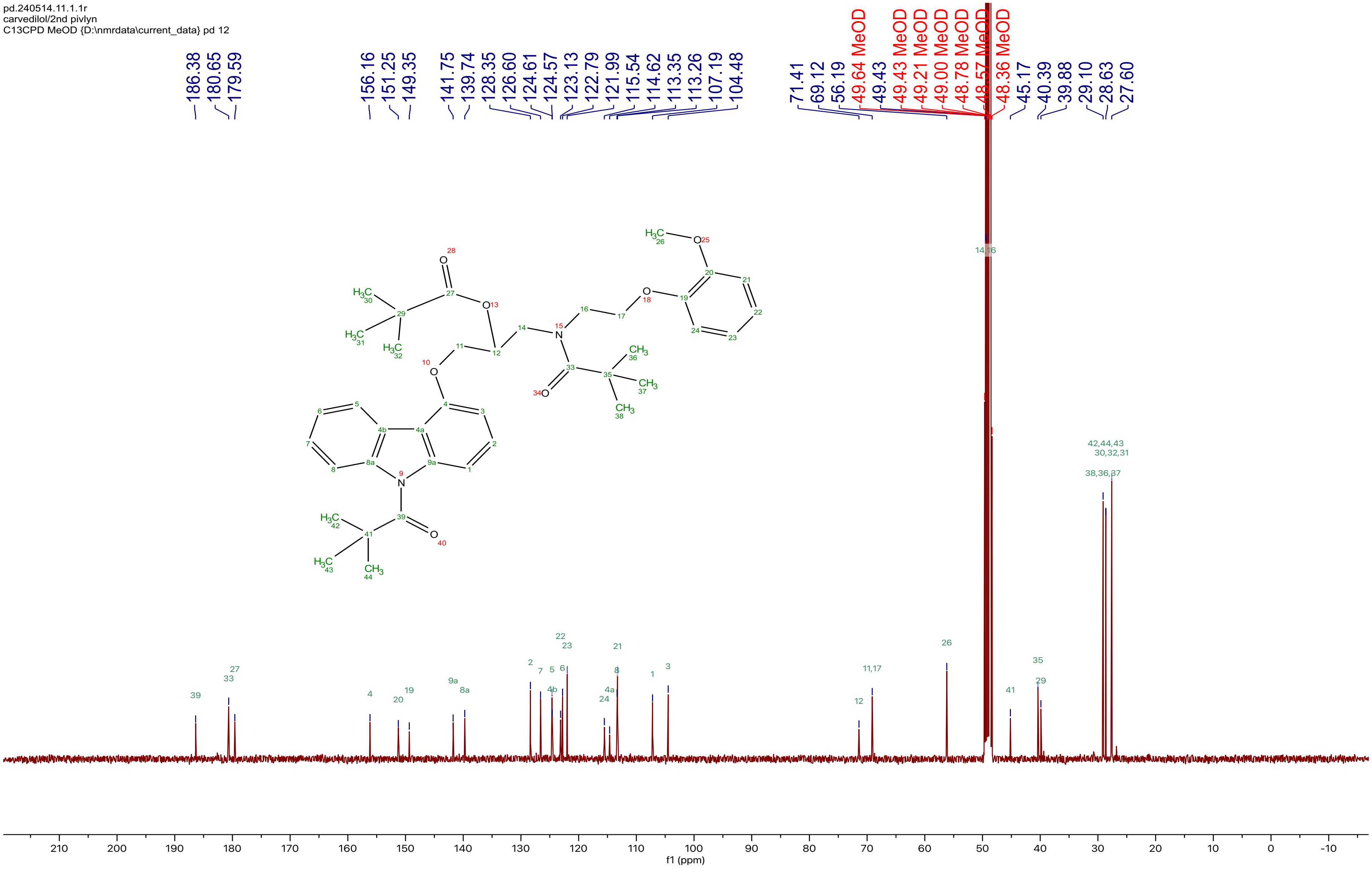

$^{13}C\{^{1}H\}$ NMR (101 MHz, MeOD) of 3s

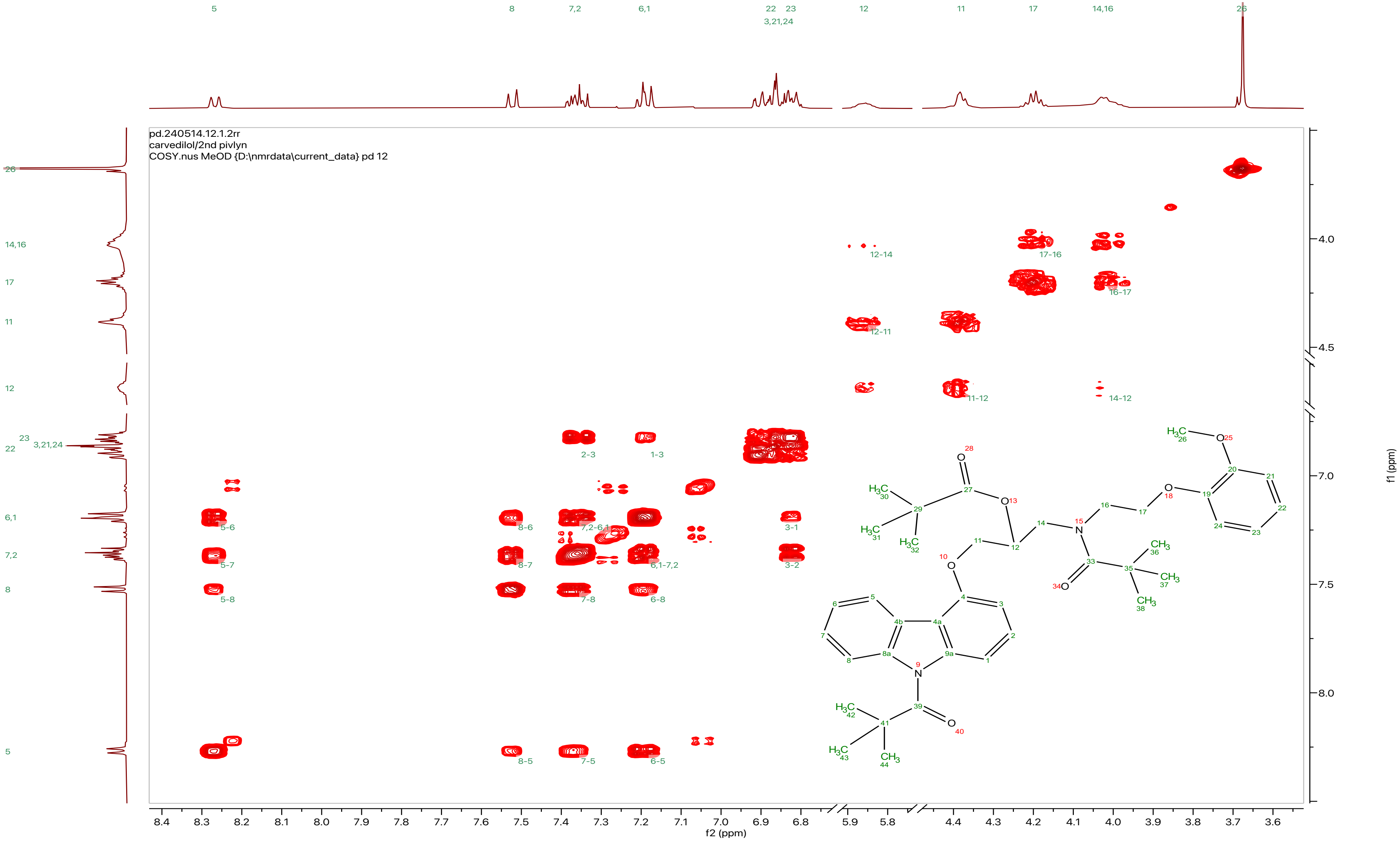

^{1}H-^{1}H COSY (400 MHz, MeOD) of 3s

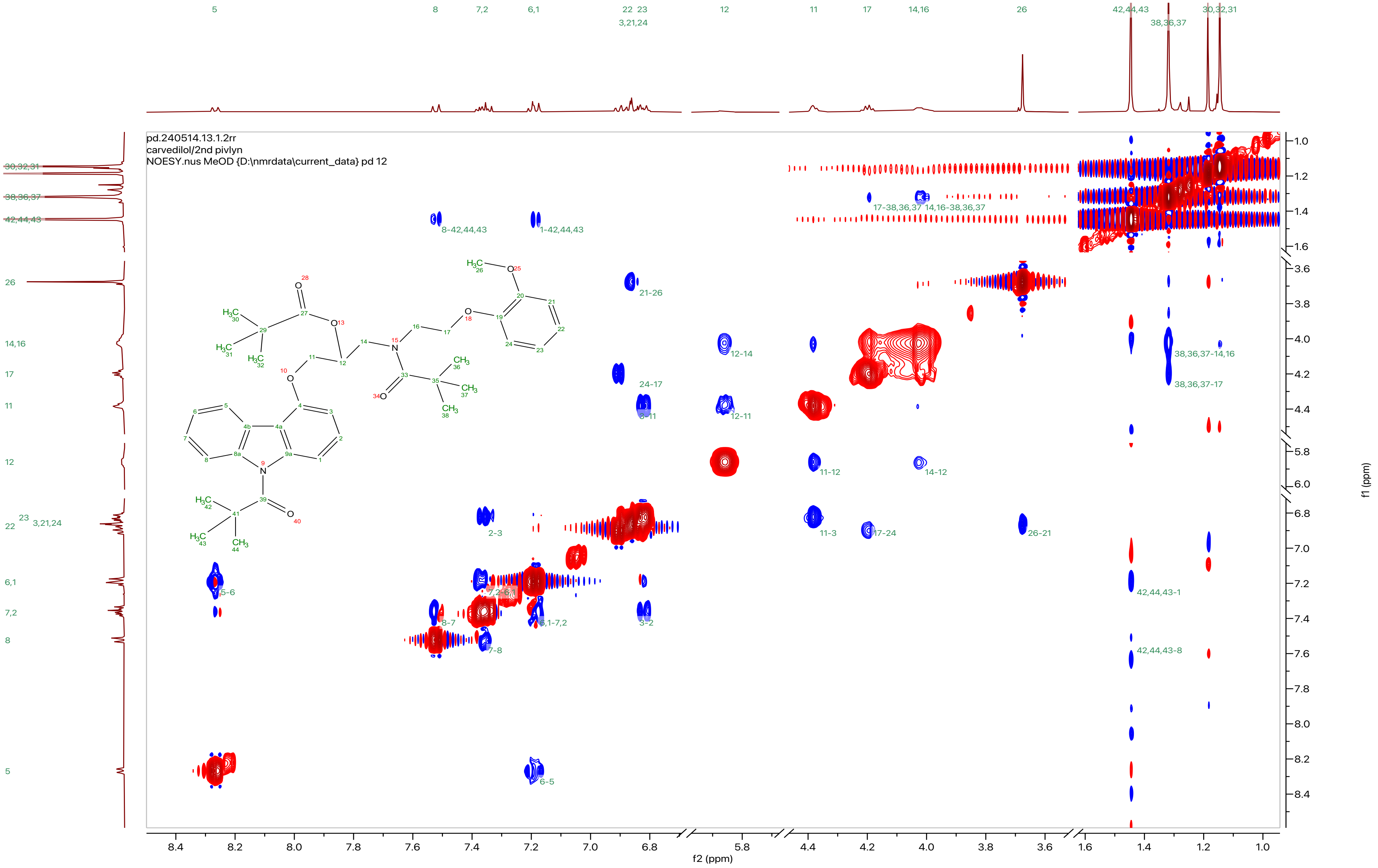

^1H-^1H NOESY (400 MHz, MeOD) of 3s

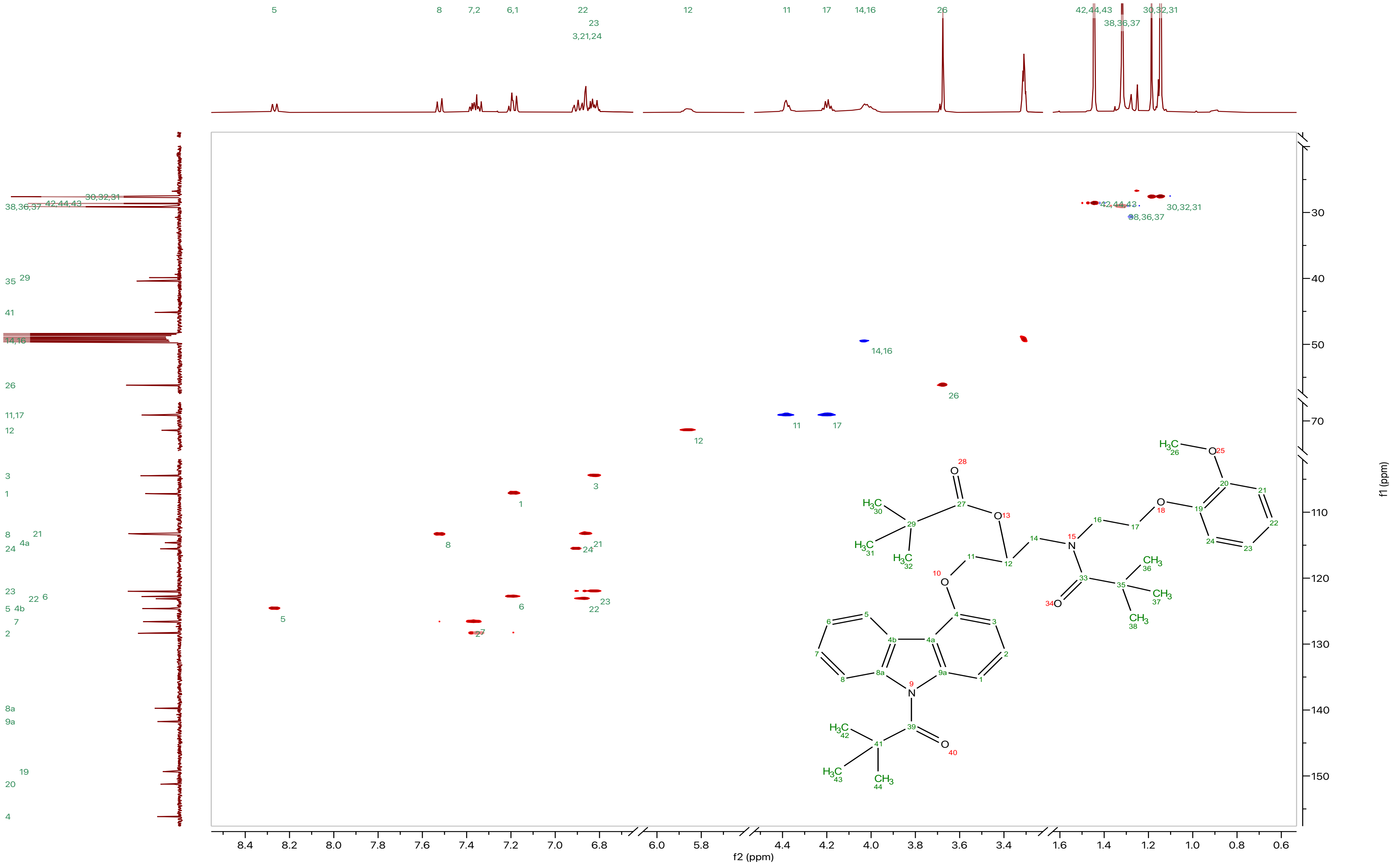

^{1}H-^{13}C{^{1}H} HSQC NMR (400/101 MHz, MeOD) of 3s

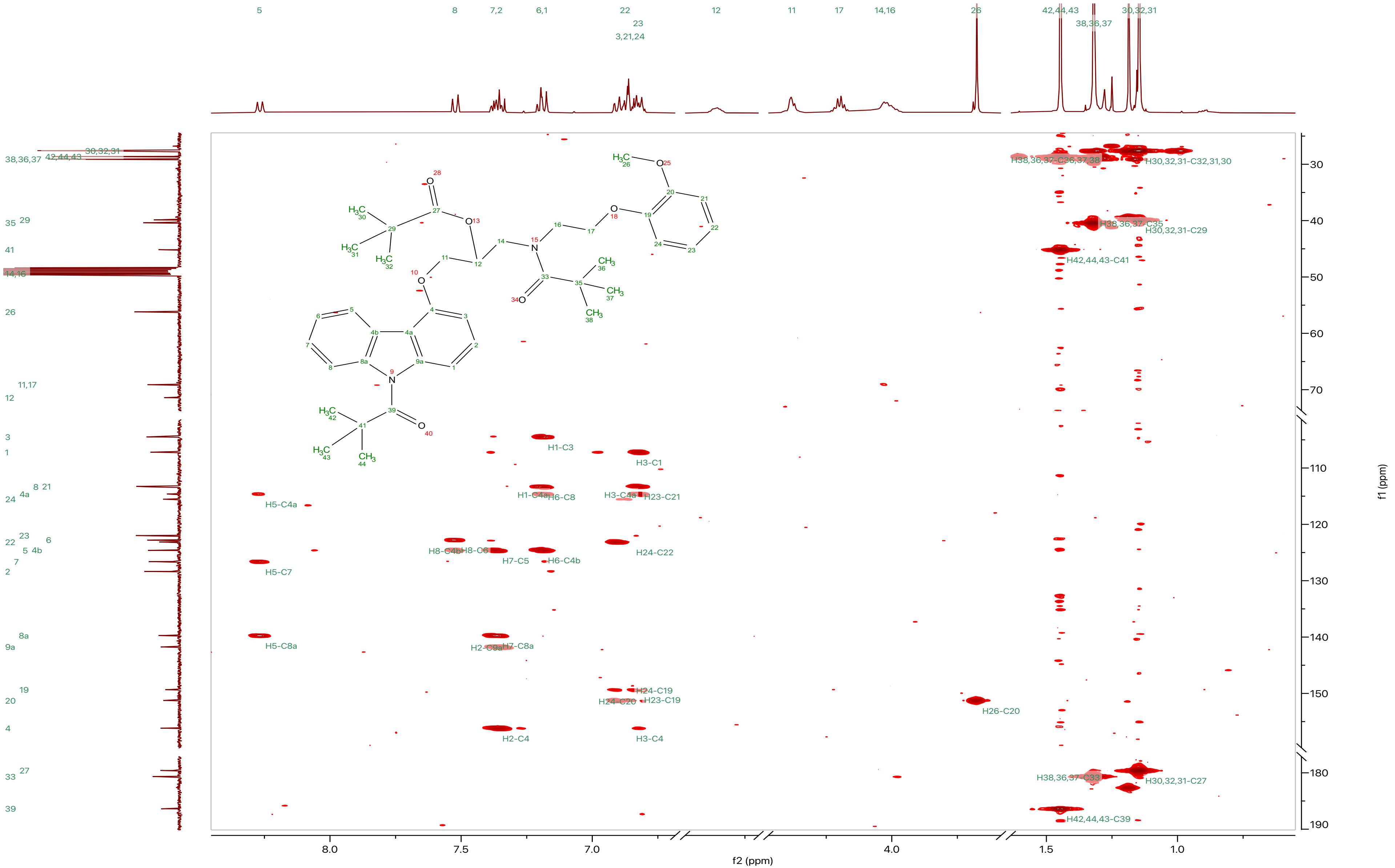

^{1}H-^{13}C{^{1}H} HMBC NMR (400/101 MHz, MeOD) of 3s

4as

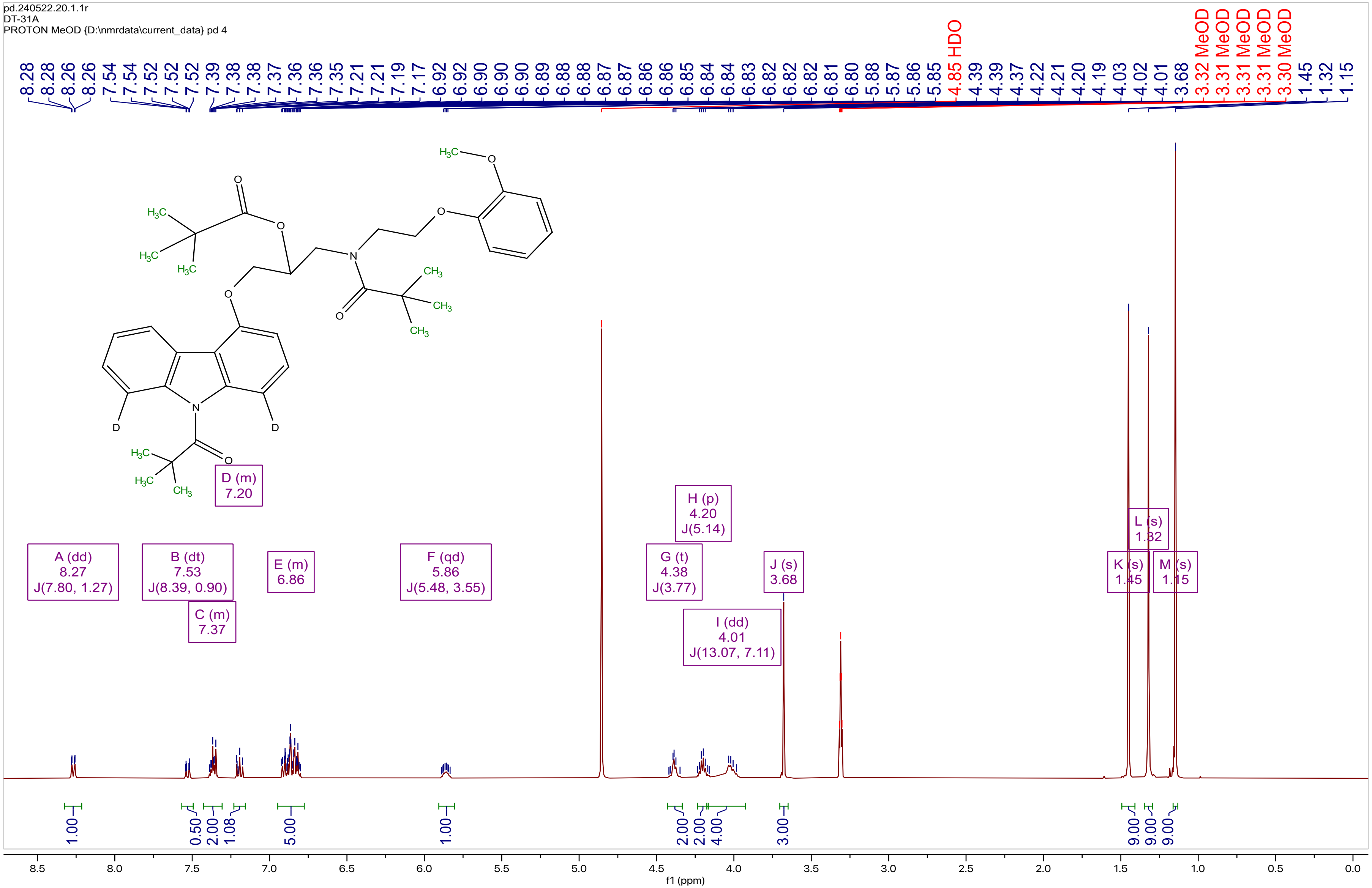

^{1}H NMR (400 MHz, MeOD) of 4as

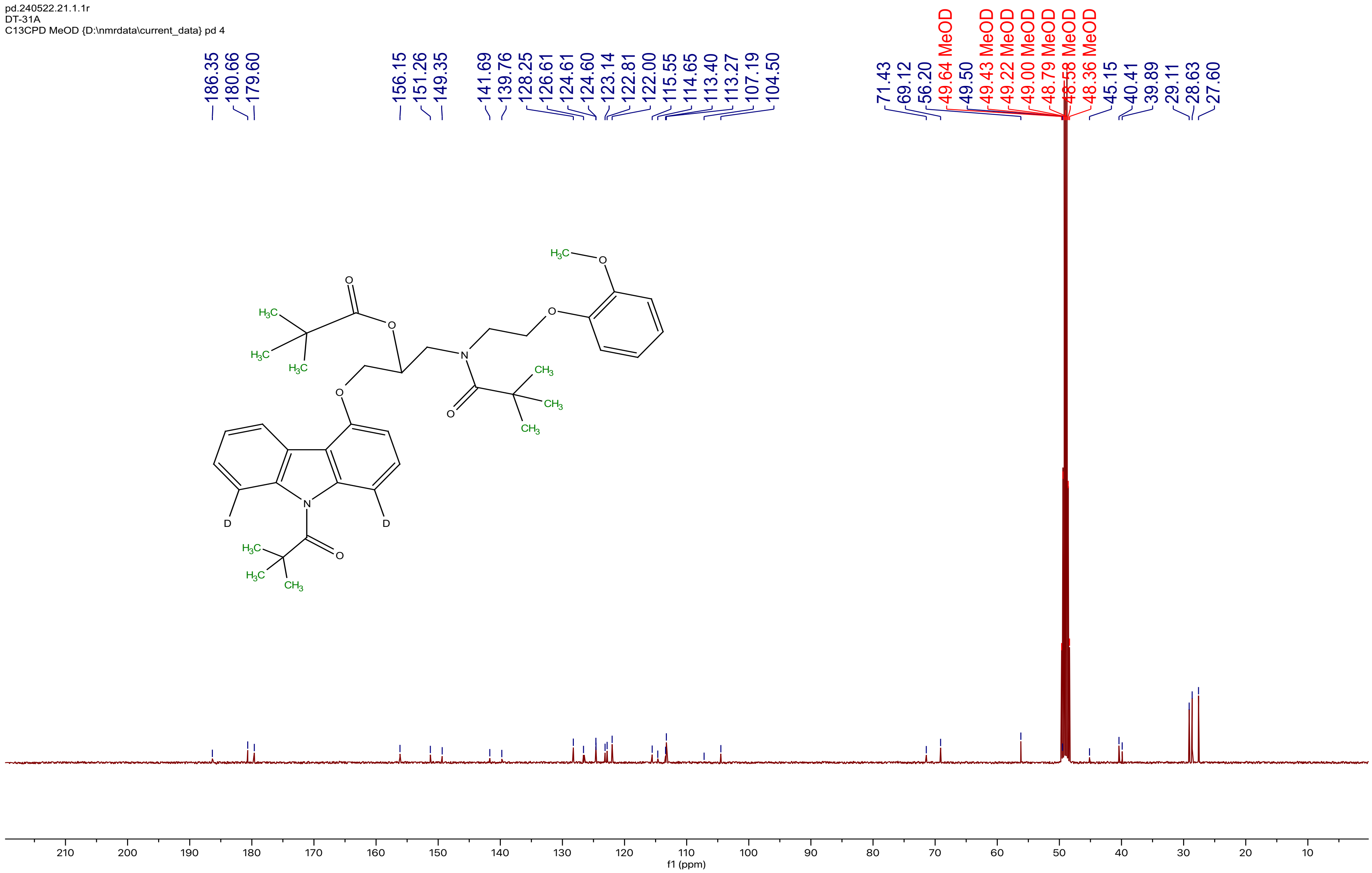

$^{13}C\{^{1}H\}$ NMR (101 MHz, MeOD) of 4as

4bs

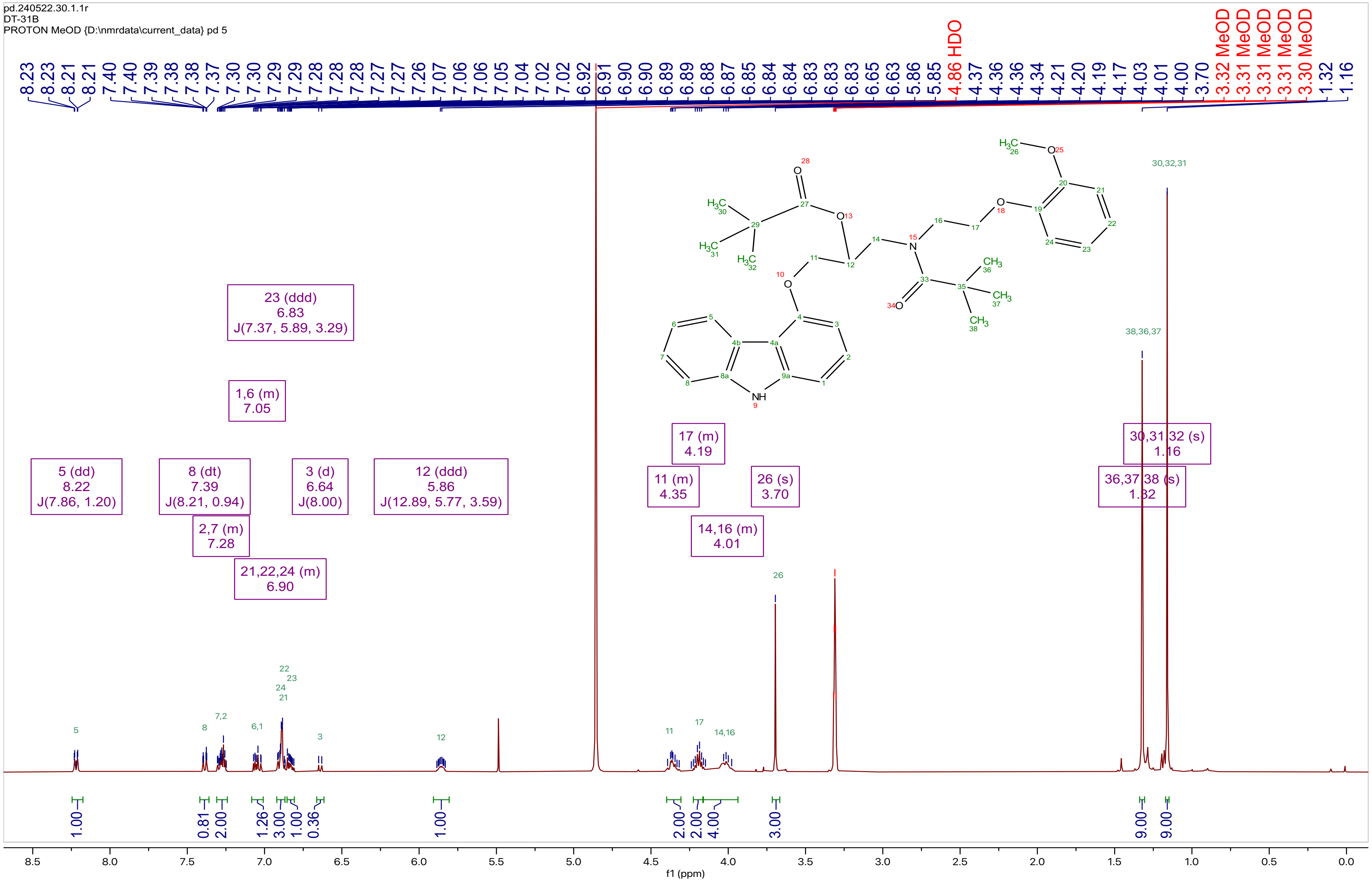

^{1}H NMR (400 MHz, MeOD) of 4bs

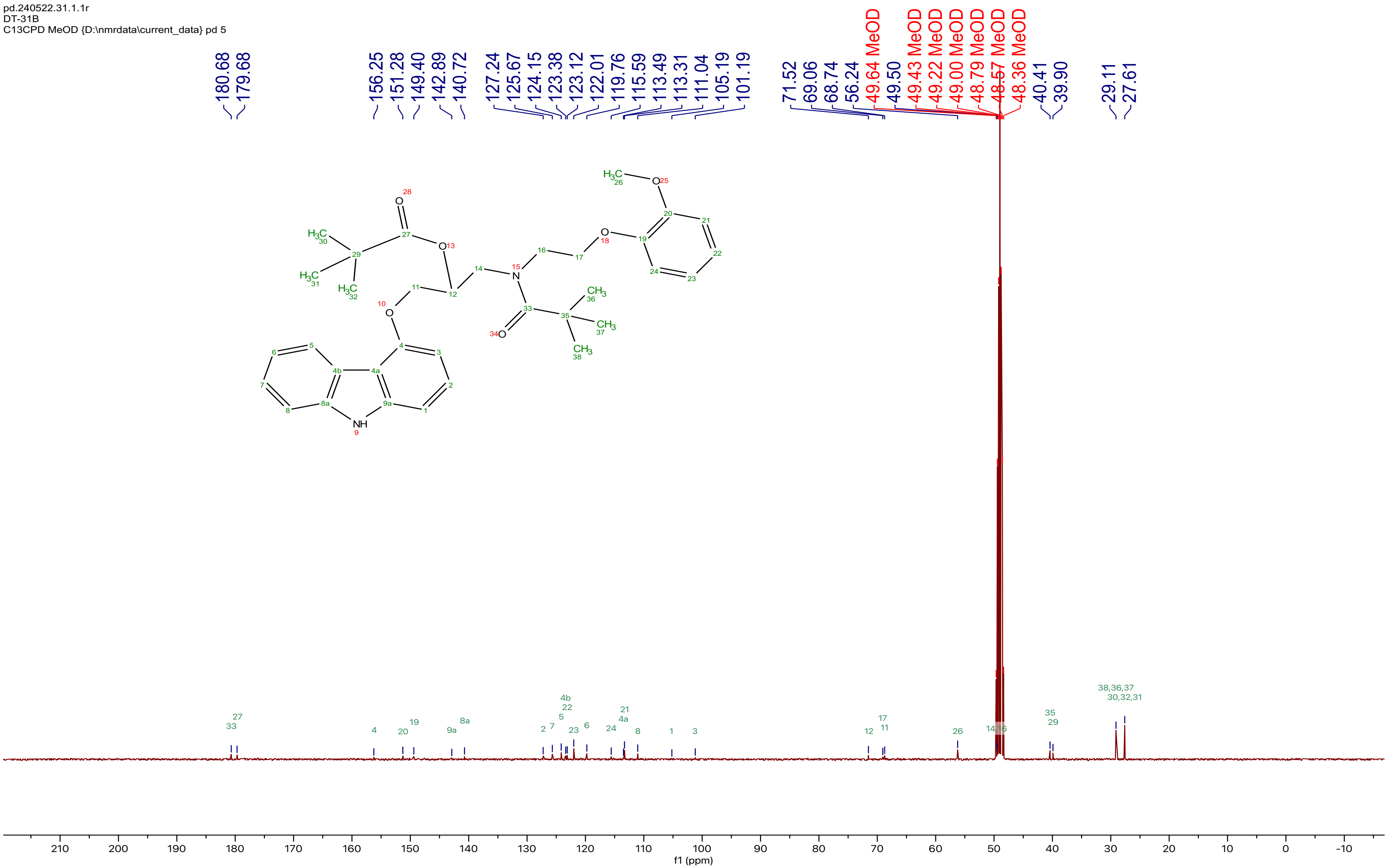

$^{13}C\{^{1}H\}$ NMR (101 MHz, MeOD) of 4bs

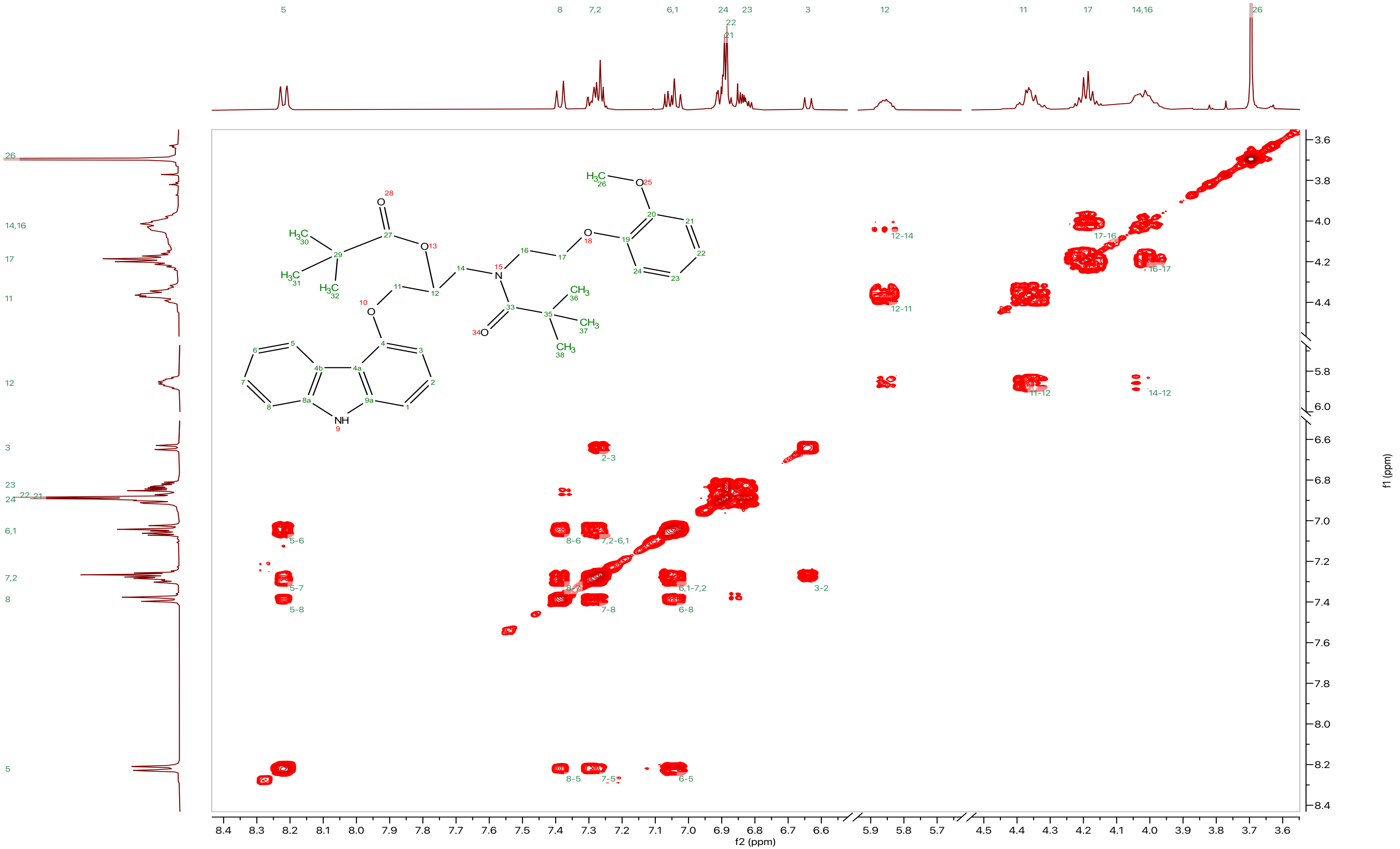

^{1}H-^{1}H COSY (400 MHz, MeOD) of 4bs

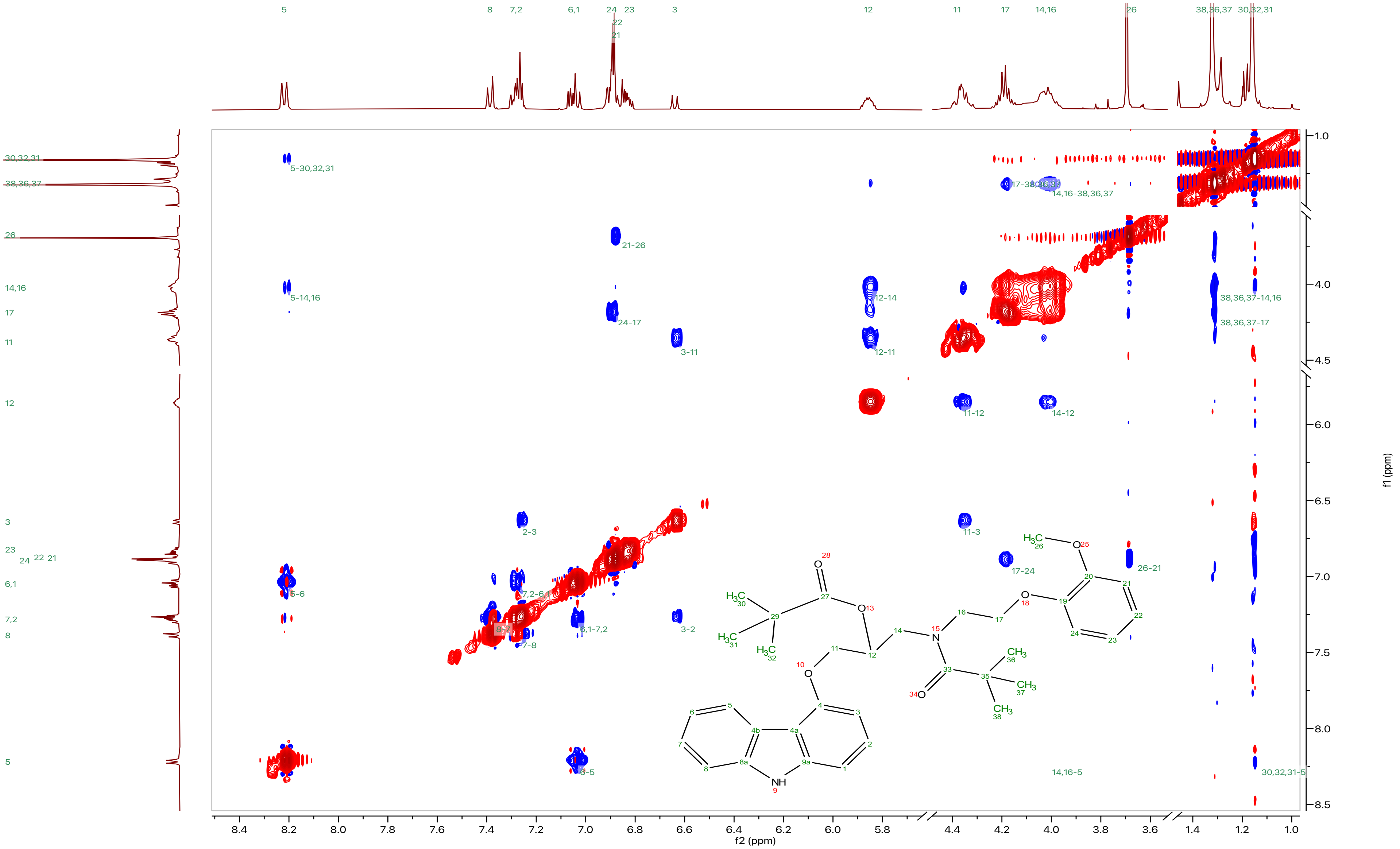

^1H-^1H NOESY (400 MHz, MeOD) of 4bs

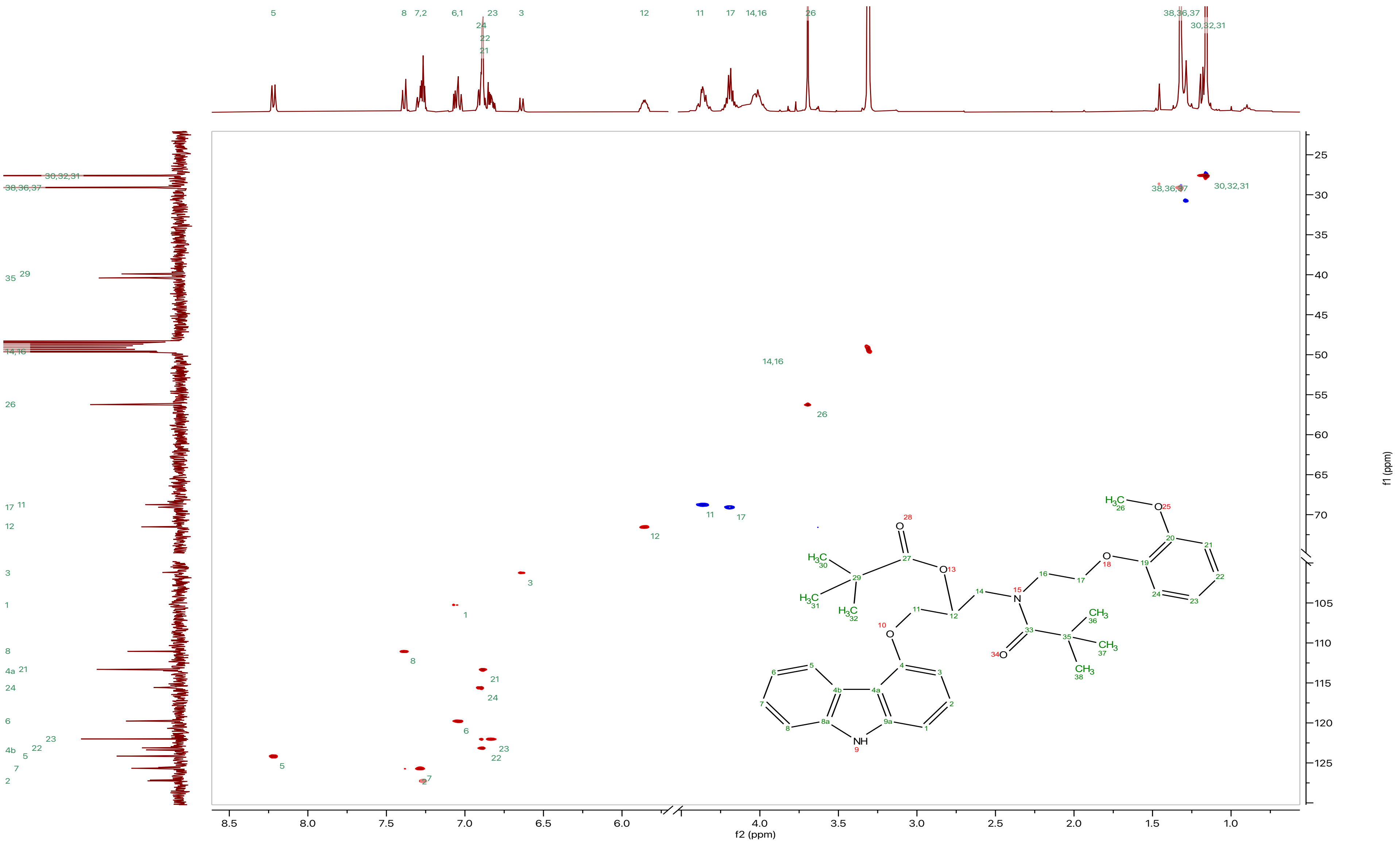

^{1}H-$^{13}C\{^{1}H\}$ HSQC NMR (400/101 MHz, MeOD) of 4bs

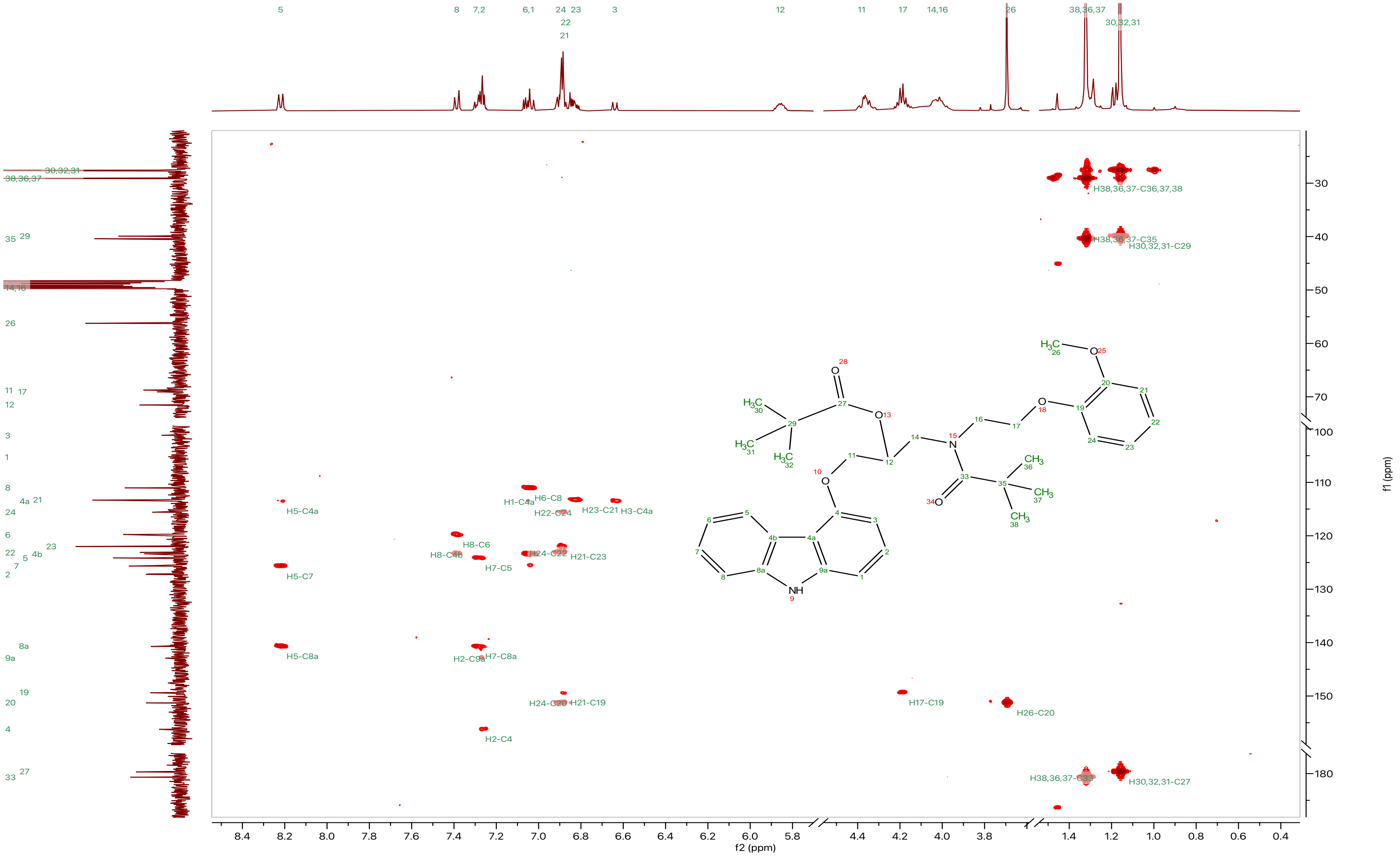

^{1}H-$^{13}C\{^{1}H\}$ HMBC NMR (400/101 MHz, MeOD) of 4b
